# Antibiotic prophylaxis in the context of VCUG/VUS in children: results of a multinational survey

**DOI:** 10.1186/s12887-026-06767-w

**Published:** 2026-03-28

**Authors:** Valentin Schaeben, Mark Born, Lutz T. Weber, Christian Dohna-Schwake, Maximilian Hohenadel, Julian A. Luetkens, Martha Dohna

**Affiliations:** 1 Clinic for Diagnostic and Interventional Radiology, University Hospital Bonn, University Bonn, Venusberg-Campus 1, Bonn, 53127 Germany; 2 Children‘s and Adolescents’ Hospital, University Hospital of Cologne, Gleueler Straße 115, Cologne, 50937 Germany; 3 Children’s Hospital Essen, University Duisburg-Essen, Hufelandstraße 55, Essen, 45147 Germany; 4 Department of Paediatric Nephrology, Children’s Hospital, University Hospital Bonn, University Bonn, Venusberg-Campus 1, Bonn, 53127 Germany

**Keywords:** Antibiotic prophylaxis, Voiding cystourethrography (VCUG), Voiding urosonography (VUS), Postprocedural urinary tract infection (ppUTI), Antimicrobial stewardship

## Abstract

**Purpose:**

Voiding cystourethrography (VCUG) and voiding urosonography (VUS) are standard procedures in children for diagnosing vesicoureteral reflux (VUR) but may cause postprocedural urinary tract infections (ppUTIs). The necessity of periprocedural antibiotic prophylaxis remains controversial, and no standardized guidelines exist. This survey investigates current antibiotic prophylaxis practices among paediatric specialists performing VCUG/VUS.

**Methods:**

An online questionnaire was distributed to paediatricians, paediatric radiologists, paediatric nephrologists, paediatric surgeons, and paediatric urologists to evaluate current practices regarding antibiotic prophylaxis in the context of VCUG and VUS.

**Results:**

A total of 126 responses from 20 countries were analyzed, mostly from Germany (63%, 79/126). Antibiotic prophylaxis during VCUG/VUS was routinely used by 58% (73/126) of respondents, while 13% (16/126) reported never using it and 29% (37/126) refrained from antibiotic prophylaxis in specific circumstances. Trimethoprim, cefaclor, and nitrofurantoin were the most common agents, with 48% (53/110) of respondents adapting their choice of antibiotic according to the patient’s age. Dosage and timing varied widely, including prophylactic, therapeutic, and double prophylactic doses. Age-inappropriate drug use was reported in 15% (16/110) of respondents. Significant differences between specialties were not found, but marked international variation in prophylaxis practices was observed.

**Conclusion:**

This survey demonstrates substantial variability in periinterventional antibiotic prophylaxis for VCUG and VUS in children. Differences in indication, agent choice, dosing, and time schedule reflect the absence of unified evidence-based standards. A standardized, multidisciplinary approach integrating current evidence and individual risk assessment is essential to optimize patient safety and antimicrobial stewardship.

**Supplementary Information:**

The online version contains supplementary material available at 10.1186/s12887-026-06767-w.

## Introduction

Voiding cystourethrography (VCUG) and voiding urosonography (VUS) are established radiological procedures for the detection of vesicoureteral reflux (VUR) and intrarenal reflux (IRR) [1–3]. Both procedures require either transurethral or suprapubic catheterization for contrast agent application. Although catheterization under aseptic conditions minimizes risk, postprocedural urinary tract infections (ppUTI) or, in case of reflux, ascending pyelonephritis may still occur [4–6]. Reports on ppUTI rates after VCUG range from 0 to 46%, leading to controversy over the need for antibiotic prophylaxis (Table 1) [3]. In addition to the short-term risks such as gastrointestinal side effects, anaphylactic reactions, and the development of antimicrobial resistance [7–9], numerous studies demonstrate that antibiotic use in early childhood may be associated with a higher risk of autoimmune diseases and later metabolic diseases [10–12]. Although some countries have a recommendation for antibiotic prophylaxis in VCUG/VUS [13, 14], a standardized protocol is not included in the current German guideline for urinary tract infection in children nor in the consensus paper by the European Society of Paediatric Radiology (ESPR) [15, 16].

In this survey we aim to bridge this data gap by assessing current practices of antibiotic use in the periprocedural period when performing VCUG/VUS among paediatricians, paediatric radiologists, paediatric nephrologists, paediatric surgeons, and paediatric urologists.

**Table 1:** Studies investigating the incidence of postprocedural urinary tract infection after voiding cystourethrography

Study	Year	Antibiotic prophylaxis status	Incidence of ppUTI
Glynn et al.	1970	Not reported	*n/a* (6%)
Maskell et al. ^abd^	1978	Partial cohort	18/60 (30%)
Guignard et al. ^abd^	1979	Partial cohort	5/142 (3.5%)
Kang et al. ^c^	2003	Partial cohort	18/932 (1.9%)
Rachmiel et al.	2005	Full cohort	7/421 (1.7%)
Ryu et al. ^c^	2008	Partial cohort	7/284 (2.5%)
Moorani et al. ^a^	2010	Not reported	12/100 (12%)
Moorthy et al. ^ac^	2010	Partial cohort	1/107 (0.9%)
Malhotra et al.	2016	Full cohort	1/47 (2.4%)
Johnson et al.	2017	Partial cohort	12/1.203 (1%)
Sinha et el. ^d^	2018	Partial cohort	9/120 (7.5%)
Martins et al. ^c^	2020	Partial cohort	23/531 (4.3%)
Doval et al. ^c^	2024	Partial cohort	12/318 (3.8%)

*ppUTI* postprocedural urinary tract infection

^a^ presence of bacteriuria (≥ 10⁵ CFU/mL) as definition for ppUTI

^b^ collection of urine samples done by parents

^c^ no significant difference in ppUTI incidence in patients with and without antibiotic prophylaxis

^d^ significant lower ppUTI incidence in patients with antibiotic prophylaxis

## Materials and methods

### Survey design

The survey was distributed by e-mail personally or via newsletter to all members of the German-speaking Society for Paediatric Radiology (GPR), the German Society for Paediatric Nephrology (GPN), and the German Society for Paediatric and Adolescent Surgery (DGKJCH). In addition, the survey was sent to members of the European Society of Paediatric Radiology (ESPR) via newsletter to include protocols from other countries. It was additionally shared via the SPR member open forum digest.

The survey was designed using the survey portal of the University Hospital Bonn (umfrage.ukbonn.de) and comprised 47 multiple-choice and free-text questions on institutional characteristics, antibiotic protocols, and factors influencing prophylaxis decisions in VCUG/VUS. It was conducted anonymously and did not collect any identifiable personal or patient-related data.

### Data collection and cleaning

The questionnaire was open from October 1st 2024 to April 30th 2025 and was formally closed on April 30, 2025. Following closure, the dataset was exported and reviewed for completeness and consistency. Data cleaning and verification were performed in May 2025. No specific eligibility criteria were applied, as the survey aimed to reach physicians potentially involved in VCUG/VUS procedures. Participation was voluntary, and respondents were expected to self-identify as relevant based on their clinical practice. A total of 126 surveys (50%) provided institutional information but did not include any information on whether antibiotic prophylaxis was used or on the specific regimen, and were therefore excluded from the analysis. Incomplete surveys were partially included in the analysis if the participant stated that antibiotic prophylaxis was not performed (*n* = 16). The participants’ individual comments in the free text sections were evaluated individually. Due to the anonymous design and data protection regulations, the total number of recipients and the proportion of respondents originating from each distribution channel could not be determined; therefore, a precise response rate cannot be calculated.

### Study outcomes

The primary outcome of this survey was to describe the variability in antibiotic prophylaxis practices among physicians performing VCUG/VUS. No secondary outcomes were defined, as the study was designed as a descriptive survey to capture current practices rather than to evaluate the effect of any intervention.

### Statistical analysis

Statistical analysis was conducted using IBM SPSS Statistics for Windows, Version 29.0 (IBM Corp., Armonk, NY, USA). Analysis included descriptive statistics for institutional characteristics. Significance tests of independence (χ^2^-test of independence) for categorical data were conducted applying Pearson’s χ^2^ and Fisher’s exact χ^2^, respectively. Dependence was considered statistically significant if *p* < 0.05. Due to the data collected and the exploratory nature of the survey, assessment of data distribution or multivariable modeling was not performed. Analogously, correction for multiple testing was not applied. All p-values reported should be interpreted as descriptive and exploratory only, and do not imply confirmatory or hypothesis-driven significance.

## Results

### Institutional information

**Fig. 1:**
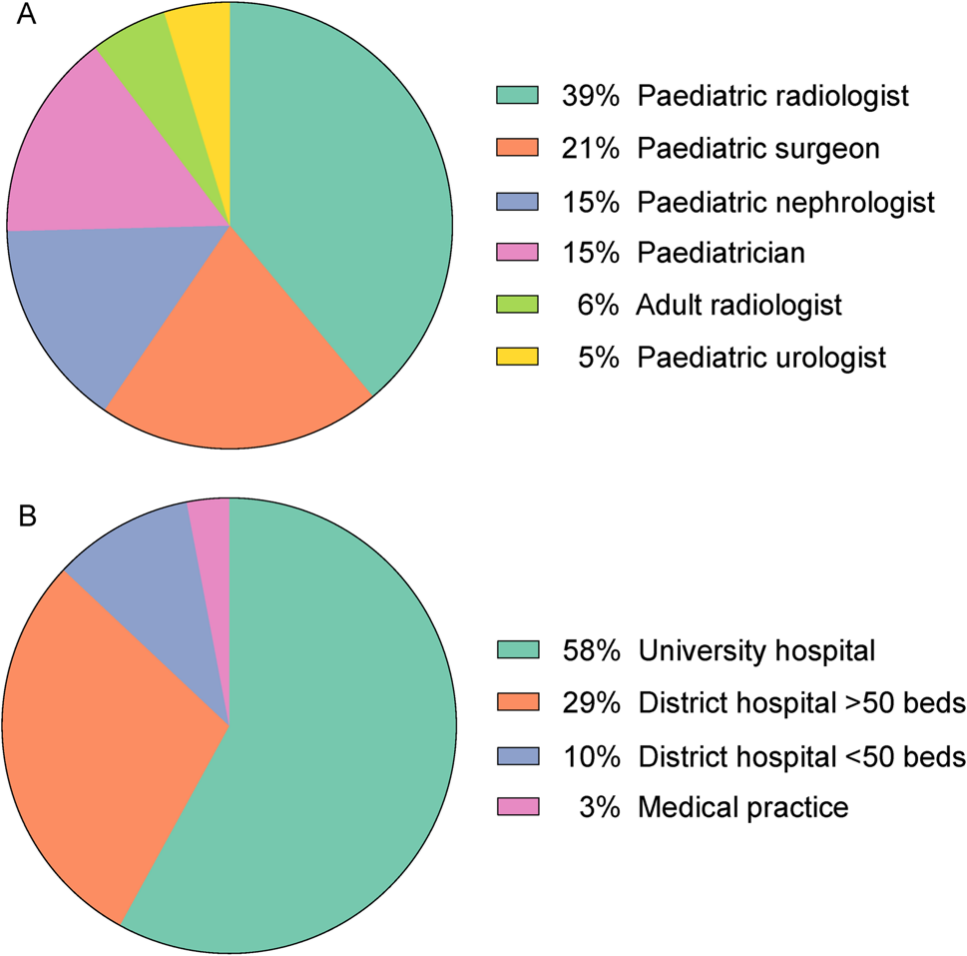
Institutional information of survey participants. (**A**) Survey respondents by medical specialties (*n* = 126). (**B**) Survey respondents by hospital size (*n* = 126)

We received a total of 252 responses from 20 different countries. After exclusion of incomplete questionnaires (126/252, 50%), 126 responses were included in the final analysis. Responding physicians came from six different subspecialties: paediatric radiology, adult radiology, paediatric surgery, paediatric nephrology, paediatrics, and paediatric urology. Hospital size included university hospitals, district hospitals, and medical practices. Institutional characteristics of survey participants is shown in Fig. 1.

We received a total of 79 answers (79/126, 62.7%) from German participants. The most represented other countries were Greece (6/126, 4.8%), Austria (5/126, 4%), United Kingdom (5/126, 4%), Netherlands (4/126, 3.2%), and Norway (4/126, 3.2%).

Ninety-two departments (92/110, 83%) had an internal departmental standard for antibiotic prophylaxis in the context of VCUGS/VUS, which did not necessarily exist in written form. In 61 of these departments (61/92, 66.3%), a formal written Standard Operation Procedure (SOP) was available. The presence of a written SOP did not seem to depend on the respondent’s specialty or size of hospital (*p* = 0.215, *p* = 0.641).

**Fig. 2:**
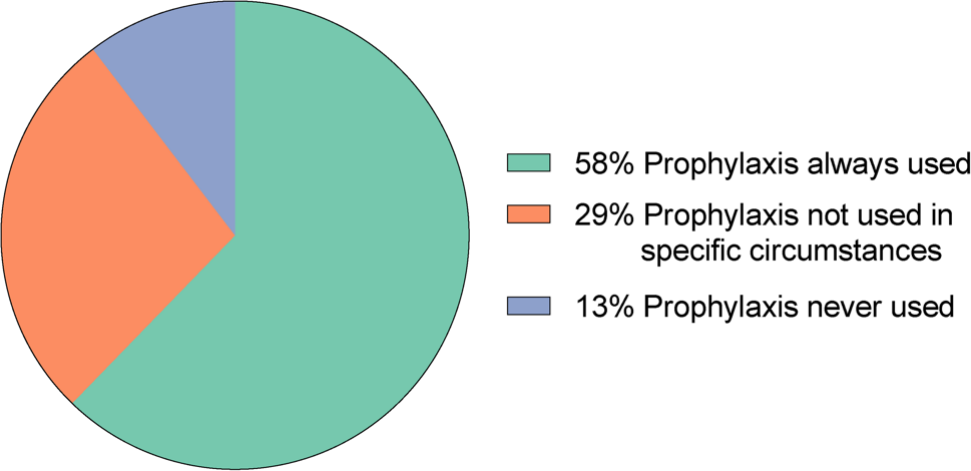
Use of antibiotic prophylaxis in VCUG/VUS (*n* = 126). VCUG: voiding cystourethrography, VUS: voiding urosonography

Participant’s use of antibiotic prophylaxis in the context of VCUG/VUS is shown in Fig. 2. When refraining from prophylaxis only in specific circumstances (37/126, 29.4%), the most frequently described setting were patients with low grade (< 2nd degree) urinary tract dilation and no UTI in medical history (14/37, 37.8%). None of the respondents reported using different antibiotic prophylaxis regimens for voiding urosonography (VUS) versus voiding cystourethrography (VCUG).

Survey participants from Germany reported significantly more frequent use of antibiotic prophylaxis compared to non-German respondents (76/79 [96.2%] vs. 32/47 [72.3%]; *p* < 0.001). All nine responses from North America indicated that antibiotic prophylaxis was not used in VCUG/VUS.

### Antibiotic agents

**Fig. 3:**
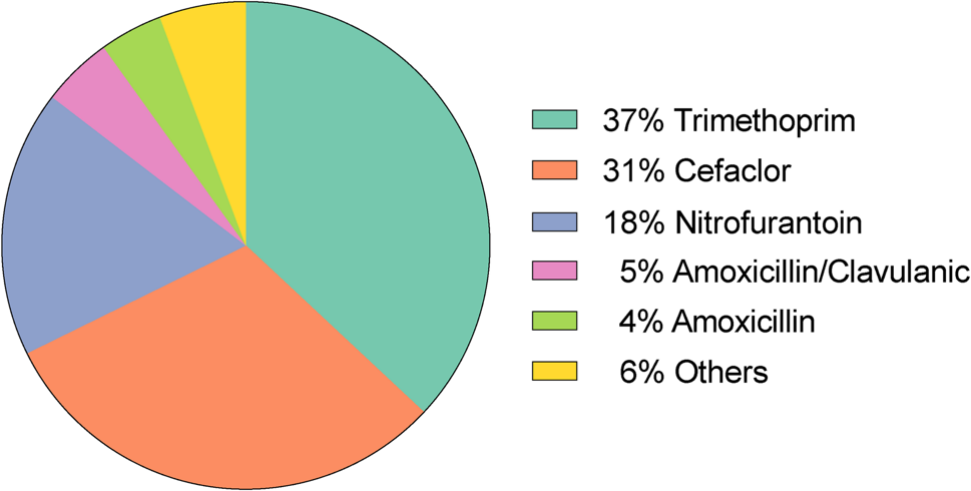
Choice of antibiotic agent for prophylaxis in VCUG/VUS (*n* = 192). VCUG: voiding cystourethrography, VUS: voiding urosonography

Seven different antibiotics were reported for periinterventional antibiotic prophylaxis in the context of VCUG/VUS. Of the 126 survey participants, 110 reported using antibiotic prophylaxis. Among these 110 respondents, 57 (57/110, 51.8%) reported exclusive use of a single antibiotic agent across all age groups. In contrast, 53 respondents (53/110, 48.2%) adapted their prophylaxis regimen according to patient age, selecting different antibiotic agents for different age groups. Among these, 22 participants (22/110, 20%) indicated the use of three different antibiotics depending on age. Because participants could report multiple antibiotics for different age groups, each age-specific antibiotic selection counted as a separate prophylactic regimen. This resulted in 192 age-specific antibiotic regimens, exceeding the number of individual survey participants (Fig. 3). The most frequently used antibiotic agents were trimethoprim (71/192, 37%), cefaclor (59/192, 30.7%), and nitrofurantoin (34/192, 17.7%) representing 164 of 192 total data sets (85.4%). Other substances used were amoxicillin-clavulanic acid (9/192, 4.6%), amoxicillin (8/192, 4.1%), cotrimoxazole (3/192, 1.6%), and cefpodoxime (2/192, 1%). Three respondents based their choice of antibiotics on the antimicrobial susceptibility profile of the preceding infection. Another participant reported administering an antibiotic concomitantly with the contrast agent via urethral catheter during the examination, without specifying the medication nor dosage.

When selecting an antibiotic, respondents could specify the age group for which they prescribed the medication. For trimethoprim, the most commonly reported minimum age was 6 weeks (23/71, 32.4%). Additional responses indicated prescription at various higher age thresholds (30/71, 42.2%). In contrast, 14 respondents (19.7%) reported using trimethoprim across all age groups. For cefaclor, the most commonly stated age category was “all ages” (18/59, 30.5%), followed by “until the age of six weeks” (12/59, 20.3%). Nitrofurantoin was the third most frequently mentioned antibiotic. The most commonly reported minimum age for its use was three months (11/34, 32.4%), while additional respondents indicated higher age thresholds (20/34, 58.8%). In three cases (3/34, 8.8%), nitrofurantoin was reported to be used across all age groups.

Ninety-seven participants (97/110, 88.2%) reported to use either one of trimethoprim, cefaclor, or nitrofurantoin. The choice of antibiotics seemed to be independent of the specialty of the respondent (trimethoprim: *p* = 0.534, cefaclor: *p* = 0.734, nitrofurantoin: *p* = 0.313).

### Dosage of antibiotics in the context of VCUG/VUS

Antibiotic dosing regimens were categorized based on the participants’ responses and standard prescribing definitions. A “prophylactic dose” was defined as the low-dose regimen recommended for continuous antibiotic prophylaxis according to the manufacturer’s prescribing information. A “therapeutic dose” refers to the standard dose recommended for the treatment of acute urinary tract infections. The term “double prophylactic dose” was used when participants explicitly reported administering twice the prophylactic dose. These definitions were applied uniformly across all reported antibiotics to allow standardized comparison.

The survey revealed moderate variation in the dosages used, without a clearly favoured dosage (Table 2). When one of the three most commonly used drugs trimethoprim, cefaclor, and nitrofurantoin was administered (164/192, 85.4%), a prophylactic dose as recommended by the manufacturer was used in 75 cases (75/164, 45.7%), a therapeutic dose in 45 cases (45/164, 27.4%), and a double prophylactic dose in 38 cases (38/164, 23.1%). In six responses, unspecified dosages were reported that deviated from the established therapeutic and prophylactic regimens, including a “fourfold prophylactic dosage (8 mg/kg body weight/day)” for trimethoprim. To further investigate, we contacted four participants by email, inquiring about the evidence for double prophylactic dosage. All four replied that it was the in-house standard for antibiotic prophylaxis and did not provide further information.

**Table 2:** Dosages for trimethoprim, cefaclor, and nitrofurantoin for antibiotic prophylaxis in voiding cystourethrography and voiding urosonography

Dosage	Medication			Total *n*, (%)
	Trimethoprim *n*, (%)	Cefaclor *n*, (%)	Nitrofurantoin *n*, (%)	
Prophylactic	29 (40.8%)	29 (49.2%)	17 (50%)	75 (45.7%)
Double prophylactic	20 (28.2%)	12 (20.3%)	6 (17.6%)	38 (23.1%)
Therapeutic	19 (26.8%)	16 (27.1%)	10 (29.4%)	45 (27.4%)
Other	3 (4.2%)	2 (3.4%)	1 (3%)	6 (3.7%)
Total	71 (100%)	59 (100%)	34 (100%)	164

### Time schedule for antibiotic prophylaxis

**Fig. 4:**
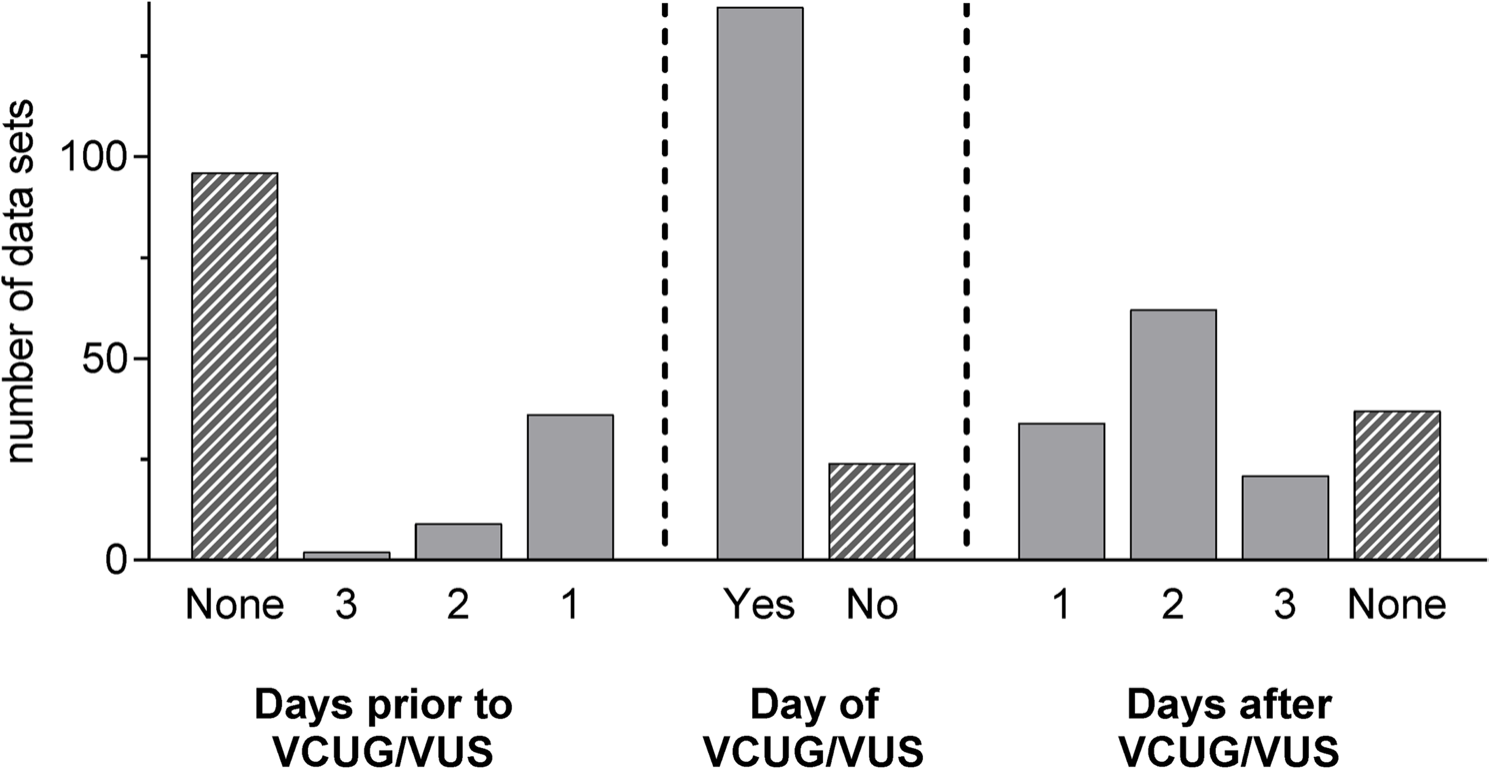
Time schedule for trimethoprim, cefaclor, and nitrofurantoin in VCUG/VUS (Number of data sets = 164). VCUG: Voiding cystourethrography; VUS: Voiding urosonography

The applied antibiotic timing schemes were highly variable (Fig. 4). Fourteen respondents (14/110, 12.7%) indicated to start antibiotic prophylaxis the moment the suspicion of VUR was raised and indication for VCUG/VUS was given. Fifteen participants (15/110, 13.6%) reported that the postinterventional antibiotic regimen depended on the examination findings. One comment stated that further prophylaxis following the examination was not necessary in clinically unremarkable patients when VUR was ruled out, whereas another participant indicated continuation of antibiotic prophylaxis for two days post-examination in the same setting. Another respondent reported administering therapeutic dosages for three days following diagnosis of vesicoureteral reflux grade III or higher.

Among survey participants who routinely used two or more antibiotics for prophylaxis depending on patient’s age, five respondents (5/53, 9.4%) reported using different time schedules for the respective antibiotics.

The number of days for antibiotic prophylaxis with the three most commonly used antibiotics (trimethoprim, cefaclor, and nitrofurantoin) seemed to be independent of the respondent’s department (trimethoprim: *p* = 0.42, cefaclor: *p* = 0.918, nitrofurantoin: *p* = 0.587).

## Discussion

The survey reveals substantial variability in the practice of periinterventional antibiotic prophylaxis for VCUG and VUS in children. This variability concerns the choice of substance, dosage, duration of administration, and factors influencing the antibiotic regimen.

### Evidence of ppUTI in the context of VCUG/VUS and necessity for antibiotic prophylaxis

The necessity of antibiotic prophylaxis in the context of transurethral catheterization for VCUG/VUS should be the subject of informed discussion. The stated goal of antibiotic prophylaxis is to prevent ppUTIs. To our knowledge only few studies have investigated the incidence of ppUTIs in the context of VCUG/VUS [3–5, 17–26]. Studies from the 1970s reported high ppUTI rates, fever, and even fatal outcomes after VCUG [6]. However, several of these studies used outdated definitions of UTI (bacteriuria ≥ 10⁵ CFU/mL) and relied on parents collecting urine samples [4–6], which likely increased the risk of contamination and possibly resulted in more frequent false-positive findings [27]. Based on these findings, antibiotic prophylaxis was deemed mandatory for VCUG by Maskell et al. [5]. Despite their methodological limitations, these studies have influenced the German guideline for urinary tract infection, which recommends antibacterial prophylaxis until VUR-evaluation only in patients after pyelonephritis. However, it does not provide any specifications regarding periinterventional prophylaxis [15]. Rachmiel et al. reported low ppUTI incidence under prophylaxis, forming the basis for the National Institute of Health and Care Excellence (NICE) guideline for United Kingdom recommending a 3-day regimen [14, 17]. Moorthy et al. questioned these findings due to the study’s retrospective design and lack of control group, sparking debate on the necessity of prophylaxis [20]. In 2017, Johnson et al. conducted the most comprehensive study to date on the occurrence of ppUTI and concluded that the risk of ppUTI after VCUG was very low (1%), and that children should not routinely be started on antibiotic prophylaxis for the sole purpose of ppUTI prevention [22]. In the first randomized clinical trial investigating antibiotic prophylaxis in the context of VCUG, reported a significantly lower incidence of ppUTIs in the prophylaxis group compared with controls (1/71 [1%] vs. 8/47 [17%], *p* = 0.002) [25]. In a more recent, larger study, Martins et al. did not find a significant difference in the incidence of ppUTIs between patients with and without antibiotic prophylaxis [23].

Interestingly, in most of the aforementioned studies, the occurrence of ppUTI was associated with either preexisting urological conditions or VUR detected during the examination [3, 19, 21, 22]. In their multivariate logistic regression analysis, Rachmiel et al. identified the presence of VUR and its severity as risk factors for the development of UTI following VCUG [17]. These findings suggest that effective antibiotic prophylaxis should be tailored to individual risk profiles as well as to the results of VCUG/VUS.

The uncertainty of whether or not to use antibiotic prophylaxis is reflected in our survey: 11% of respondents reported never using any periinterventional prophylaxis, whereas 54% used it at all times regardless of other factors. Indications were often based on prior febrile UTI, prenatal or postnatal urinary tract obstruction, or existing reflux, but varied widely in detail. Gauthier et al. reported a similar distribution of responses in their survey of paediatric nephrologists on the use of antibiotic prophylaxis in VCUG (46% reported not using prophylaxis, while 56% prescribed it routinely) [28]. A notable finding was the significant difference in use of antibiotic prophylaxis between participants from Germany (76/79, 96.2%) and those from other countries (34/47, 72.3%). All respondents from North America (9/126, 7.1%) reported not using prophylaxis, which aligns with the recommendations of the American College of Radiology and SPR of no routine antibiotic prophylaxis.

### Choice of antibiotic agent, dosage and time schedule

The survey showed a predominant and consistent use of a few antibiotics for prophylaxis, mainly trimethoprim (37.2%), cefaclor (30.7%), and nitrofurantoin (17.7%), aligning with German guideline recommendations for continuous antibiotic prophylaxis in VUR. However, substantial variation in dosage and duration was observed, reflecting the absence of clear guidance. Several responses reported unspecified or atypical dosages deviating from established regimens, including the frequently mentioned “double prophylactic” dose, for which no supporting evidence could be found in the literature, nor could it be provided by the respondents themselves. This variability, including the use of non-standard dosing regimens, raises concerns regarding patient safety and antimicrobial stewardship, underscoring the urgent need for evidence-based and standardized recommendations.

Significant associations were not identified between medical specialty and the choice, dosage, or duration of procedural prophylaxis, suggesting that prescribing practices rely more on personal or institution-specific habits than on evidence-based recommendations.

Of particular concern, 16 respondents (16/110, 14.5%) reported using trimethoprim or nitrofurantoin in age groups for which these drugs are not approved - trimethoprim in infants under 6 weeks and nitrofurantoin in those under 3 months. This underscores uncertainty across specialties regarding appropriate antibiotic use and highlights the need for targeted education. Developing age-adapted guidelines and SOPs, informed by infectious disease and pharmacology expertise, could help standardize and improve the safety of antibiotic prophylaxis in paediatric patients.

### Multidisciplinary practice variation and need for standardization

The heterogeneity of antibiotic prophylaxis practices in VCUG/VUS may also stem from the involvement of multiple specialties, each with their own internal protocols. In daily practice, the indication for reflux diagnostics may be made by one, while the procedure is performed by another. Johnson et al. also noted that VCUG results are often sent to referring physicians but may not be acted upon at the time of highest ppUTI risk [22]. This further emphasizes the importance of establishing cross-departmental standards for periinterventional prophylaxis, especially including a paediatric infectious disease specialist.

### Strengths and limitations

A substantial proportion of questionnaires had to be excluded due to missing information on antibiotic prophylaxis. This may introduce selection bias, as participants who completed the survey might differ systematically from those who did not complete the relevant sections. A limitation of this study is the voluntary participation of physicians involved in VCUG/VUS, which may have resulted in selection bias toward certain centers. Additionally, several physicians from the same institutions may have participated, potentially reporting similar protocols, and the anonymous nature of the survey prevents us from fully excluding the possibility of multiple responses from the same institution. Furthermore, we did not collect detailed information on participants’ clinical experience or procedural volume, and participants may have provided their personal opinions rather than strictly institutional standards. The majority of responses (62.7%) originated from Germany, which may limit the generalizability of the findings to other countries. Accordingly, the findings should be interpreted primarily as reflecting practice patterns in German-speaking centers, with some additional international input. Furthermore, as with all survey-based studies, the results may be subject to recall bias and social desirability bias. Another limitation is that dosing regimen in patients receiving prophylaxis with pre-existing VUR was not assessed. Finally, the descriptive design of this survey does not allow assessment of clinical effectiveness or safety of specific antibiotic regimens, which should be addressed in future prospective studies.

Nevertheless, the wide range of responses across different departments, including both small and large university hospitals and multiple countries, provides a representative overview of current practice and highlights the considerable heterogeneity in approaches.

## Conclusion

This survey reveals substantial heterogeneity in periinterventional antibiotic prophylaxis for VCUG/VUS in children, encompassing differences in indication, choice of agents, dosing regimens, and duration. Many practices appear to be driven by local institutional standards or individual clinical experience due to the lack of uniform, evidence-based recommendations. Key clinical questions remain regarding the indications for antibiotic prophylaxis, including whether prophylaxis should be initiated in cases of upper tract dilatation in general or restricted to ureteral dilatation. Furthermore, the optimal timing of prophylaxis initiation following UTI and prior to imaging remains unclear, particularly in light of the variability among existing guidelines from different professional societies. Standardized, cross-specialty protocols including antimicrobial stewardship teams - accounting for preexisting individual risk factors and the results of VCUG/VUS, while minimizing unnecessary antibiotic exposure - are essential to ensure patient safety.

## Supplementary Information

Supplementary Material 1.

Supplementary Material 2.

Supplementary Material 3.

## Data Availability

The datasets generated and/or analysed during the current study are available from the corresponding author on reasonable request.

## Abbreviations

VCUG: Voiding cystourethrography
VUS: Voiding urosonography
VUR: Vesicoureteral reflux
IRR: Intrarenal reflux
ppUTI: Postprocedural urinary tract infection
ESPR: European Society of Paediatric Radiology
GPR: Society for German-speaking Paediatric Radiologists
GPN: German Society for Paediatric Nephrology
DGKJCH: German Society for Paediatric Surgery
SPR: Society for Pediatric Radiology
SOP: Standard operation procedure
UTI: Urinary tract infection
