# Supplementary material for: Antibiotic prophylaxis in the context of VCUG/VUS in children: results of a multinational survey
Source: BMC Pediatr. 2026 Mar 28;26:372. doi: 10.1186/s12887-026-06767-w (PMC13107907; doi:10.1186/s12887-026-06767-w)
Supplement: Supplementary file 1 — Supplementary Material 1. [file 12887_2026_6767_MOESM1_ESM.docx]

**Online supplement to:**

**Antibiotic prophylaxis in the context of VCUG/VUS**

**in children: Results of a multinational survey**

**Valentin Schaeben^1^ (https://orcid.org/0009-0000-7962-602X), Mark Born^1^ (https://orcid.org/0000-0002-1740-0006), Lutz T. Weber^2^ (https://orcid.org/0000-0003-4116-598X), Christian Dohna-Schwake^3^ (https://orcid.org/0000-0001-8973-5406), Maximilian Hohenadel^4^, Martha Dohna^1^ (https://orcid.org/0000-0002-2471-8530)**

^1^ Clinic for Diagnostic and Interventional Radiology, Department of Paediatric Radiology, University Hospital Bonn, Venusberg-Campus 1, 53127 Bonn, Germany

^2^ Children‘s and Adolescents’ Hospital, University Hospital of Cologne, Gleueler Straße 115, 50937 Cologne, Faculty of Medicine, University of Cologne, Germany

^3^ Children’s Hospital Essen, University Duisburg-Essen, Hufelandstraße 55, 45147 Essen, Germany

^4^ Department of Paediatric Nephrology, Children’s Hospital, University Hospital Bonn, Venusberg-Campus 1, 53127 Bonn, Germany

Corresponding author: Valentin Schaeben, Clinic for Diagnostic and Interventional Radiology, University Hospital Bonn, Venusberg-Campus 1, 53127 Bonn, Germany, E-mail: [valentin.schaeben@ukbonn.de](mailto:valentin.schaeben@ukbonn.de), Phone: +49 228 287-15871, Fax: +49 228 287-16093

**Supplementary material and results**

**Indication for antibiotic prophylaxis**

The extent to which patient history or pre-existing findings influenced antibiotic prophylaxis was assessed. Sixty-eight participants (68/110, 78.2 %) reported administering antibiotic prophylaxis to all patients undergoing VCUG/VUS. In 26 cases, it was indicated that patients received antibiotic prophylaxis following febrile urinary tract infection, pyelonephritis, or urosepsis independent of gender. The time interval between VCUG/VUS and the prior infection varied between 1 and 30 days. In 16 cases, participants stated to prescribe antibiotic prophylaxis if sonographic evidence of urinary tract dilation was present: two cases for all grades of dilation, six cases for grade II and higher, seven cases for grade III and higher, and one case for grade IV. A prenatally diagnosed urinary tract dilation was an indication for antibiotic prophylaxis in nine cases: one case for all grade of dilation, four cases for grade II or higher, two cases for grade III or higher, and two cases for grade IV. A distinct pattern did not emerge for individually specified indications to establish peri-interventional antibiotic prophylaxis.

**Deviation from standard**

Furthermore, 86 survey participants (86/110, 78.2 %) reported to deviate from their standard prophylaxis regimen in certain situations or clinical findings. Seventy-four examiners (74/86, 86.0 %) reported adjusting their antibiotic prophylaxis in cases of resistant pathogens detected in a previous urine culture. Twenty-eight participants (28/86, 32.6 %) stated to modify prophylaxis in cases of recurrent urinary tract infections or breakthrough infections during ongoing continuous prophylaxis, often adapting to the susceptibility profile (7/28, 25 %) or increasing the dosage during VCUG/VUS (4/28, 14,3 %). In one case, intravenous prophylaxis for VCUG/VUS was considered for recurrent urinary tract infections. A previously confirmed vesicoureteral reflux (VUR) and established continuous infection prophylaxis were reasons for deviation from the standard protocol for 24 respondents (24/86, 27.9 %), although specific details of these deviations were not provided. One examiner reported administering intravenous gentamicin prophylaxis in newborns under three months of age. Overall, five respondents (5/86, 5.8 %) reported that additional peri-interventional prophylaxis was not prescribed during ongoing continuous antibiotic prophylaxis, and in one case (ID 181), prophylaxis was escalated to a therapeutic dose during reflux evaluation. One participant (ID 9) stated that prophylaxis was omitted in patients with an indwelling transurethral catheter, citing the procedure catheterization as the greatest risk factor for post-procedural urinary tract infections (ppUTIs). Difficult catheterization was a reason for deviation from standard practice in two respondents, with one (ID 116) considering prophylaxis only in this specific setting and the other (ID 191) administering increased dosage.

**Difference between VUS and VCUG, intravenous prophylaxis**

None of the respondents reported a difference in antibiotic prophylaxis between voiding urosonography (VUS) and voiding cystourethrography (VCUG). Overall, 21 participants (21/110, 19.1 %) indicated situational use of intravenous prophylaxis: In six cases when the examination was performed during ongoing therapy, in six cases upon detection of multidrug-resistant pathogens in urine culture, in two cases due to non-compliance, in two cases with newborns, in two cases with an indwelling peripheral venous catheter, and in one case with abnormal urine dipstick under ongoing infection prophylaxis. Two participants did not specify the circumstances under which intravenous prophylaxis was administered.

**Evidence on ppUTI and associated pathogens after VCUG/VUS**

The evidence regarding the incidence of ppUTI following VCUG/VUS and their causative pathogens is limited due to the lack of current studies. Moorani et al. reported 12 cases of ppUTI (12/100, 12%), with ppUTI defined as the presence of asymptomatic bacteriuria (>10⁵ colony forming units (CFU)): *E. coli* was the most frequently detected organism (43%), followed by *Pseudomonas* (33%) and *Klebsiella* (25%)[1]. Doval et al. included both clinical (fever) and laboratory (CRP, PCT) parameters in their definition of ppUTI [2]. They found a similar distribution of pathogens in their larger and more recent study that counted 12 cases of ppUTI after VCUG (12/318, 3.8%): *E. coli* (58.3%), *Klebsiella pneumoniae* (25.0%), *Enterobacter cloacae* (16.7%), and *S. aureus* (8.3%). Johnson et al. conducted the most comprehensive study to date on the occurrence of post-procedural urinary tract infections (ppUTI) following VCUG and documented only 12 cases of ppUTI among 1,203 examinations (1.0%) [3]. The pathogens were similarly distributed: *E. coli* (25.0%), *Enterococcus faecalis* (25.0%), *S. epidermidis* (16.7%), *Klebsiella pneumoniae* (8.3%), *Pseudomonas aeruginosa* (8.3%), and *Candida albicans* (n=1, 8.3%). In the only case of ppUTI (1/47, 2.1%) following retrograde urethrography (RUG), Malhotra et al. identified *E. coli* as the causative agent [4]. Similar to urinary tract infections in children [5], *E. coli* appears to be the most common pathogen in this sample of ppUTI cases (16/37, 43.2%).

References

[1] Moorani K, Parkash Jai, Kumar Lohana Makesh. Urinary tract infection in children undergoing diagnostic voiding cystourethrography. Journal of Surgery Pakistan (International) 2010.

[2] Doval L, Paye-Jaouen A, Pierucci UM, Lachkar A-A, Duneton C, Lopez P et al. Incidence of febrile post-procedural urinary tract infection following voiding cystourethrography in children without prior urine culture. World J Urol 2024;42(1):499. https://doi.org/10.1007/s00345-024-05217-5.

[3] Johnson EK, Malhotra NR, Shannon R, Jacobson DL, Green J, Rigsby CK et al. Urinary tract infection after voiding cystourethrogram. J Pediatr Urol 2017;13(4):384.e1-384.e7. https://doi.org/10.1016/j.jpurol.2017.04.018.

[4] Malhotra NR, Green JR, Rigsby CK, Holl JL, Cheng EY, Johnson EK. Urinary tract infection after retrograde urethrogram in children: A multicenter study. J Pediatr Urol 2017;13(6):623.e1-623.e5. https://doi.org/10.1016/j.jpurol.2017.04.026.

[5] Gesellschaft für Pädiatrische Nephrologie und Arbeitskreis Kinder- und Jugendurologie der Deutschen Gesellschaft für Urologie. Interdisziplinäre S2k-Leitlinie: Harnwegsinfektionen im Kindesalter: Diagnostik, Therapie und Prophylaxe. Version 1, 23.08.2021.
