# Supplementary material for: Antibiotic prophylaxis in the context of VCUG/VUS in children: results of a multinational survey
Source: BMC Pediatr. 2026 Mar 28;26:372. doi: 10.1186/s12887-026-06767-w (PMC13107907; doi:10.1186/s12887-026-06767-w)
Supplement: Supplementary file 2 — Supplementary Material 2. [file 12887_2026_6767_MOESM2_ESM.pdf]

**Current data regarding the necessity of antibacterial prophylaxis in vesicoureteral reflux (VUR) diagnostics is unclear. While there are guidelines on how to treat urinary tract infections and on long-term prophylaxis in existing VUR, there are no clear recommendations for antibiotic prophylaxis in the context of voiding urosonography (VUS) and voiding cystourethrography (VCUG). Whereas examination protocols for VUS/VCUG are largely standardized, protocols for infection prophylaxis in the context of VUS/VCUG highly vary between centers.**

**With this survey, we aim to assess indication for antibiotic prophylaxis and the different protocols of antibiotic infection prophylaxis when performing VUR diagnostics with VUS/VCUG.**

**Note: if you are not responsible for the implementation of antibiotic prophylaxis in your clinic/department, thank you for forwarding this survey to your colleagues in charge!**

**Time for completing the survey: 5-10 minutes.**

## Section A: Standard Operation Procedure

**A1. Who fills out this survey?**

- Pediatric Radiologist ☐
- Radiologist ☐
- Pediatric Surgeon ☐
- Pediatrician ☐
- Pediatric nephrologist ☐
- Pediatric urologist ☐
- Other ☐

Other

[ ]

LimeSurvey

**A2.** **In which type of hospital are you employed?**

- University Hospital ☐
- Community hospital (>50 beds for pediatric patients) ☐
- Community hospital (<50 beds for pediatric patients) ☐
- Other ☐

Other

[ ]

**A3.** **In which country do you work?**

[ ]

**A4.** **Does your department have a defined protocol for infection prophylaxis in the context of VUS/VCUG?**

- Yes ☐
- No ☐

**A5.** **Has this protocol been transformed into a written standard operating procedure (SOP)?**

- Yes ☐
- No ☐

**A6.** **Which department is the author of the SOP for infection prophylaxis in VUS/VCUG?**

- Pediatrics ☐
- Pediatric surgery ☐
- Pediatric nephrology ☐
- Pediatric radiology ☐
- Other ☐

Other

[ ]

# Section B: Antibiotic Prophylaxis

**B1.** **Are there situations/constellations in which you do not use antibiotic prophylaxis for VUS/VCUG?**

*Please note: this is not about long-term prophylaxis but about prophylaxis in the context of VUS/VCUG!*

- Yes ☐
- No ☐

**B2.** **Please specify these situations/constellations:**

- girl with first uncomplicated UTI without urinary tract dilation in ultrasound ☐
- boy with first uncomplicated UTI without urinary tract dilation in ultrasound ☐
- in patients with low grade urinary tract dilation (up to 2nd grade) and no UTI in medical history ☐
- Other ☐

Other

**B3.** **If you use antibiotic prophylaxis: which mediaction do you use? (multiple answers and subdivision into age groups possible)**

- Trimethoprim ☐
- Nitrofurantoin ☐
- Amoxicillin ☐
- Cefaclor ☐
- Amoxicillin/clavulanic acid ☐
- Other ☐

**B4.** **Trimethoprim: in what dosage?**

- prophylactic (2 mg/kg bodyweight/d) ☐
- double prophylakctic (4 mg/kg bodyweight/d) ☐
- therapeutic (6 mg/kg bodyweight/d) ☐
- Other ☐

Other

**B5.** **Trimethoprim: for which age group do you use trimethoprim?**

**B6.** **Trimethoprim: which time schedule do you use?**

Day(s) before VUS/VCUG, how many?

Comment

Day of VUS/VCUG

Comment

Day(s) after VUS/VCUG, how many?

Comment

**B7.** **Nitrofurantoin: in what dosage?**

prophylactic (1-2 mg/kg bodyweight/d)

double prophylactic (2-4 mg/kg bodyweight/d)

therapeutic (5 mg/kg bodyweight/d)

Other

Other

**B8.** **Nitrofurantoin: for which age group do you use nitrofurantoin?**

**B9.** **Nitrofurantoin: which time schedule do you use?**

Day(s) before VUS/VCUG, how many?

Comment

Day of VUS/VCUG

Comment

Day(s) after VUS/VCUG, how many?

Comment

**B10. Amoxicillin: in what dosage?**

prophylactic (10-20 mg/kg bodyweight/d)

double prophylactic (20-40 mg/kg bodyweight/d)

therapeutic (50-100 mg/kg bodyweight/d)

Other

Other

**B11. Amoxicillin: for which age group do you use amoxicillin?**

**B12. Amoxicillin: which time schedule do you use?**

Day(s) before VUS/VCUG, how many?

Comment

Day of VUS/VCUG

Comment

Day(s) after VUS/VCUG, how many?

Comment

LimeSurvey

**B13. Cefaclor: in what dosage?**

- prophylactic (10 mg/kg bodyweight/d) ☐
- double prophylactic (20 mg/kg bodyweight/d) ☐
- therapeutic (40 mg/kg bodyweight/d) ☐
- Other ☐

Other

**B14. Cefaclor: for which age group do you use cefaclor?**

**B15. Cefaclor: which time schedule do you use?**

Days(s) before VUS/VCUG, how many? ☐

Comment

Day of VUS/VCUG ☐

Comment

Day(s) after VUS/VCUG, how many? ☐

Comment

**B16. Amoxicillin/clavulanic acid: in what dosage? (Amoxicillin portion)**

- prophylaktic (10-20 mg/Kg bodyweight/d) ☐
- double prophylactic (20-40 mg/Kg bodyweight/d) ☐
- therapeutic (80-100 mg/Kg bodyweight/d) ☐

Other

Other

**B17.** **Amoxicillin/clavulanic acid: for which age group do you use amoxicillin/clavulanic acid?**

**B18.** **Amoxicillin/clavulanic acid: which time schedule do you use?**

Day(s) before VUS/VCUG

Comment

Day of VUS/VCUG

Comment

Day(s) after VUS/VCUG

Comment

**B19.** **Other: which medication and in what dosage?**

**B20.** **Other: for which age group do you use this medication?**

**B21.** **Other: which time schedule do you use?**

Day(s) before VUS/VCUG, how many?

Comment

Day of VUS/VCUG

Comment

Day(s) after VUS/VCUG, how many?

Comment

## Section C: Indication for prophylaxis

**C1. Which patients receive periinterventional antibiotic prophylaxis? (multiple answers possible)**

- all
- patients post urinary tract infection/pyelonephritis/urosepsis
- patients with urinary tract dilation in ultrasound
- patients with prenatal urinary tract dilation
- patients post VUR surgery
- none
- Other

Other

**C2. patients status post urinary tract infection/pyelonephritis/urosepsis: what is the minimum time between infection and VUS/VCUG? (in days)**

**C3.**

**In patients with urinary tract dilation in ultrasound: from what degree on?**

**Image from Beetz R, Bokenkamp A, Brandis M et al (2001) Diagnosis of congenital dilatation of the urinary tract. Consensus Group of the Pediatric Nephrology Working Society in cooperation with the Pediatric Urology Working Group of the German Society of Urology and with the Pediatric Urology Working Society in the Germany Society of Pediatric Surgery. Urologe A 40:495–507 (quiz 8–9)**

- grade 1 ☐
- grade 2 ☐
- grade 3 ☐
- grade 4 ☐

LimeSurvey

**C4.**

**In patients with prenatal urinary tract dilation: from what degree on?**

**Image from Beetz R, Bokenkamp A, Brandis M et al (2001) Diagnosis of congenital dilatation of the urinary tract. Consensus Group of the Pediatric Nephrology Working Society in cooperation with the Pediatric Urology Working Group of the German Society of Urology and with the Pediatric Urology Working Society in the Germany Society of Pediatric Surgery. Urologe A 40:495–507 (quiz 8–9)**

- grade 1 ☐
- grade 2 ☐
- grade 3 ☐
- grade 4 ☐

LimeSurvey

**C5. Are there situations or clinical findings in the patient where you deviate from your prophylaxis standard (medication, dose, duration, age of the patient)? (multiple answers possible, please specify in answer box)**

in case of already known vesicoureteral reflux (VUR) ☐

Comment

in patients with recurrent urinary tract infections (UTI) ☐

Comment

if multidrug-resistant organisms (MDRO) are detected in urine cultures ☐

Comment

in newborns ☐

Comment

Other ☐

Other

**C6. Are there situations in which you use intravenous prophylaxis?**

Yes ☐

No ☐

**C7. In which situations?**

# Section D: Voiding analysis

**D1.** **Are there differences in antibiotic prophylaxis between VUS and VCUG in your department?**

Yes ☐

No ☐

**D2.** **To what extent?**

**D3.** **Is a non-pathological urine strip test required in preparation for VUS/VCUG in your department?**

Yes ☐

No ☐

**D4.**

**What is the maximum amount of days accepted for urine strip test to be performed prior to the exam?**

**D5.** **Is a non-pathological urine culture required in preparation for VUS/VCUG in your department?**

Yes ☐

No ☐

**D6.** **What is the maximum amount of days accepted for urine culture to be performed prior to the exam?**

**D7.** **Do patients only receive post-procedure prophylaxis if vesicoureteral reflux (VUR) is detected in VUS/VCUG?**

*Please note: this is not about long-term prophylaxis but about prophylaxis in the context of VUS/VCUG!*

Yes ☐

No ☐

LimeSurvey

**D8.** **Please explain your approach:**

**D9.** **Do other MUS/MCU findings (e.g. urethral valves) have an influence on post-procedure prophylaxis?**

*Please note: this is not about long-term prophylaxis but about prophylaxis in the context of VUS/VCUG!*

Yes ☐

No ☐

**D10.** **Please specify:**

# Section E: End of survey

Thank you for taking the time to answer this survey!

**E1.** **May we contact you personally if we have further questions?**

Yes ☐

No ☐

**E2.** **E-mail address:**

**Thank you for taking the time to answer this questionnaire!**
