# Supplementary material for: Antibiotic prophylaxis in the context of VCUG/VUS in children: results of a multinational survey
Source: BMC Pediatr. 2026 Mar 28;26:372. doi: 10.1186/s12887-026-06767-w (PMC13107907; doi:10.1186/s12887-026-06767-w)
Supplement: Supplementary file 3 — Supplementary Material 3. [file 12887_2026_6767_MOESM3_ESM.pdf]

State of the Art: Infektionsprophylaxe im Rahmen von MUS/MCU

Survey response 1

Response ID
2

Last page
0

Start language
de

Seed
231346065

Date started
2024-11-13 10:11:59

Date last action
2024-11-13 10:11:59

Standard Operation Procedure

Does your department have a defined protocol for infection prophylaxis in the context of VUS/VCUG?
N/A

Has this protocol been transformed into a written standard operating procedure (SOP)?
N/A

Antibiotic Prophylaxis

Are there situations/constellations in which you do not use antibiotic prophylaxis for VUS/VCUG?
N/A

Please specify these situations/constellations: [girl with first uncomplicated UTI without urinary tract dilation in ultrasound]
N/A

Please specify these situations/constellations: [boy with first uncomplicated UTI without urinary tract dilation in ultrasound]
N/A

Please specify these situations/constellations: [in patients with low grade urinary tract dilation (up to 2nd grade) and no UTI in medical history]
N/A

If you use antibiotic prophylaxis: which mediaction do you use? (multiple answers and subdivision into age groups possible) [Trimethoprim]
N/A

If you use antibiotic prophylaxis: which mediaction do you use? (multiple answers and subdivision into age groups possible) [Nitrofurantoin]
N/A

If you use antibiotic prophylaxis: which mediaction do you use? (multiple answers and subdivision into age groups possible) [Amoxicillin]
N/A

If you use antibiotic prophylaxis: which mediaction do you use? (multiple answers and subdivision into age groups possible) [Cefaclor]
N/A

If you use antibiotic prophylaxis: which mediaction do you use? (multiple answers and subdivision into age groups possible) [Amoxicillin/clavulanic acid]
N/A

If you use antibiotic prophylaxis: which mediaction do you use? (multiple answers and subdivision into age groups possible) [Other]
N/A

Trimethoprim: which time schedule do you use? [Day(s) before VUS/VCUG, how many?]
N/A

Trimethoprim: which time schedule do you use? [Day of VUS/VCUG]
N/A

Trimethoprim: which time schedule do you use? [Day(s) after VUS/VCUG, how many?]
N/A

Nitrofurantoin: which time schedule do you use? [Day(s) before VUS/VCUG, how many?]
N/A

Nitrofurantoin: which time schedule do you use? [Day of VUS/VCUG]
N/A

Nitrofurantoin: which time schedule do you use? [Day(s) after VUS/VCUG, how many?]
N/A

Amoxicillin: which time schedule do you use? [Day(s) before VUS/VCUG, how many?]
N/A

Amoxicillin: which time schedule do you use? [Day of VUS/VCUG]
N/A

Amoxicillin: which time schedule do you use? [Day(s) after VUS/VCUG, how many?]
N/A

Cefaclor: which time schedule do you use? [Days(s) before VUS/VCUG, how many?]
N/A

Cefaclor: which time schedule do you use? [Day of VUS/VCUG]
N/A

Cefaclor: which time schedule do you use? [Day(s) after VUS/VCUG, how many?]
N/A

Amoxicillin/clavulanic acid: in what dosage? (Amoxicillin portion) [prophylaktic (10-20 mg/Kg bodyweight/d)]
N/A

Amoxicillin/clavulanic acid: in what dosage? (Amoxicillin portion) [double prophylactic (20-40 mg/Kg bodyweight/d)]
N/A

Amoxicillin/clavulanic acid: in what dosage? (Amoxicillin portion) [therapeutic (80-100 mg/Kg bodyweight/d)]
N/A

Amoxicillin/clavulanic acid: which time schedule do you use? [Day(s) before VUS/VCUG]
N/A

Amoxicillin/clavulanic acid: which time schedule do you use? [Day of VUS/VCUG]
N/A

Amoxicillin/clavulanic acid: which time schedule do you use? [Day(s) after VUS/VCUG]
N/A

Other: which time schedule do you use? [Day(s) before VUS/VCUG, how many?]
N/A

Other: which time schedule do you use? [Day of VUS/VCUG]
N/A

Other: which time schedule do you use? [Day(s) after VUS/VCUG, how many?]
N/A

Indication for prophylaxis

Which patients receive periinterventional antibiotic prophylaxis? (multiple answers possible) [all]
N/A

Which patients receive periinterventional antibiotic prophylaxis? (multiple answers possible) [patients post urinary tract infection/pyelonephritis/urosepsis]
N/A

Which patients receive periinterventional antibiotic prophylaxis? (multiple answers possible) [patients with urinary tract dilation in ultrasound]
N/A

Which patients receive periinterventional antibiotic prophylaxis? (multiple answers possible) [patients with prenatal urinary tract dilation]
N/A

Which patients receive periinterventional antibiotic prophylaxis? (multiple answers possible) [patients post VUR surgery]
N/A

Which patients receive periinterventional antibiotic prophylaxis? (multiple answers possible) [none]
N/A

Are there situations or clinical findings in the patient where you deviate from your prophylaxis standard (medication, dose, duration, age of the patient)? (multiple answers possible, please specify in answer box) [in case of already known vesicoureteral reflux (VUR)]
N/A

Are there situations or clinical findings in the patient where you deviate from your prophylaxis standard (medication, dose, duration, age of the patient)? (multiple answers possible, please specify in answer box) [in patients with recurrent urinary tract infections (UTI)]
N/A

Are there situations or clinical findings in the patient where you deviate from your prophylaxis standard (medication, dose, duration, age of the patient)? (multiple answers possible, please specify in answer box) [if multidrug-resistant organisms (MDRO) are detected in urine cultures]
N/A

Are there situations or clinical findings in the patient where you deviate from your prophylaxis standard (medication, dose, duration, age of the patient)? (multiple answers possible, please specify in answer box) [in newborns]
N/A

Are there situations in which you use intravenous prophylaxis?
N/A

Voiding analysis

Are there differences in antibiotic prophylaxis between VUS and VCUG in your department?
N/A

Is a non-pathological urine strip test required in preparation for VUS/VCUG in your department?
N/A

Is a non-pathological urine culture required in preparation for VUS/VCUG in your department?
N/A

Do patients only receive post-procedure prophylaxis if vesicoureteral reflux (VUR) is detected in VUS/VCUG?
N/A

Do other MUS/MCU findings (e.g. urethral valves) have an influence on post-procedure prophylaxis?
N/A

End of survey

May we contact you personally if we have further questions?
N/A

Survey response 2

Response ID
3

Date submitted
2024-11-13 11:17:41

Last page
5

Start language
de

Seed
1558386364

Date started
2024-11-13 10:59:29

Date last action
2024-11-13 11:17:41

Standard Operation Procedure

Who fills out this survey?
Pediatric Radiologist

Who fills out this survey? [Other]

In which type of hospital are you employed?
University Hospital

In which type of hospital are you employed? [Other]

In which country do you work?
Deutschland

Does your department have a defined protocol for infection prophylaxis in the context of VUS/VCUG?
Yes

Has this protocol been transformed into a written standard operating procedure (SOP)?
Yes

Which department is the author of the SOP for infection prophylaxis in VUS/VCUG?
Pediatric radiology

Which department is the author of the SOP for infection prophylaxis in VUS/VCUG? [Other]

Antibiotic Prophylaxis

Are there situations/constellations in which you do not use antibiotic prophylaxis for VUS/VCUG?
Yes

Please specify these situations/constellations: [girl with first uncomplicated UTI without urinary tract dilation in ultrasound]
No

Please specify these situations/constellations: [boy with first uncomplicated UTI without urinary tract dilation in ultrasound]
No

Please specify these situations/constellations: [in patients with low grade urinary tract dilation (up to 2nd grade) and no UTI in medical history]
No

Please specify these situations/constellations: [Other]
bekannte kompl. HWI nach Rücksprache mit päd.Nephrologen z.b. REesistenzen oder bekannte Keime oder spezialfall Transplantatniere

If you use antibiotic prophylaxis: which mediaction do you use? (multiple answers and subdivision into age groups possible) [Trimethoprim]
No

If you use antibiotic prophylaxis: which mediaction do you use? (multiple answers and subdivision into age groups possible) [Nitrofurantoin]
No

If you use antibiotic prophylaxis: which mediaction do you use? (multiple answers and subdivision into age groups possible) [Amoxicillin]
No

If you use antibiotic prophylaxis: which mediaction do you use? (multiple answers and subdivision into age groups possible) [Cefaclor]
Yes

If you use antibiotic prophylaxis: which mediaction do you use? (multiple answers and subdivision into age groups possible) [Amoxicillin/clavulanic acid]
No

If you use antibiotic prophylaxis: which mediaction do you use? (multiple answers and subdivision into age groups possible) [Other]
No

Trimethoprim: which time schedule do you use? [Day(s) before VUS/VCUG, how many?]
N/A

Trimethoprim: which time schedule do you use? [Day of VUS/VCUG]
N/A

Trimethoprim: which time schedule do you use? [Day(s) after VUS/VCUG, how many?]
N/A

Nitrofurantoin: which time schedule do you use? [Day(s) before VUS/VCUG, how many?]
N/A

Nitrofurantoin: which time schedule do you use? [Day of VUS/VCUG]
N/A

Nitrofurantoin: which time schedule do you use? [Day(s) after VUS/VCUG, how many?]
N/A

Amoxicillin: which time schedule do you use? [Day(s) before VUS/VCUG, how many?]
N/A

Amoxicillin: which time schedule do you use? [Day of VUS/VCUG]
N/A

Amoxicillin: which time schedule do you use? [Day(s) after VUS/VCUG, how many?]
N/A

Cefaclor: in what dosage?
prophylactic (10 mg/kg bodyweight/d)

Cefaclor: in what dosage? [Other]

Cefaclor: for which age group do you use cefaclor?

Cefaclor: which time schedule do you use? [Days(s) before VUS/VCUG, how many?]
No

Cefaclor: which time schedule do you use? [Days(s) before VUS/VCUG, how many?][Comment]

Cefaclor: which time schedule do you use? [Day of VUS/VCUG]
Yes

Cefaclor: which time schedule do you use? [Day of VUS/VCUG][Comment]

Cefaclor: which time schedule do you use? [Day(s) after VUS/VCUG, how many?]
No

Cefaclor: which time schedule do you use? [Day(s) after VUS/VCUG, how many?][Comment]

Amoxicillin/clavulanic acid: in what dosage? (Amoxicillin portion) [prophylaktic (10-20 mg/Kg bodyweight/d)]
N/A

Amoxicillin/clavulanic acid: in what dosage? (Amoxicillin portion) [double prophylactic (20-40 mg/Kg bodyweight/d)]
N/A

Amoxicillin/clavulanic acid: in what dosage? (Amoxicillin portion) [therapeutic (80-100 mg/Kg bodyweight/d)]
N/A

Amoxicillin/clavulanic acid: which time schedule do you use? [Day(s) before VUS/VCUG]
N/A

Amoxicillin/clavulanic acid: which time schedule do you use? [Day of VUS/VCUG]
N/A

Amoxicillin/clavulanic acid: which time schedule do you use? [Day(s) after VUS/VCUG]
N/A

Other: which time schedule do you use? [Day(s) before VUS/VCUG, how many?]
N/A

Other: which time schedule do you use? [Day of VUS/VCUG]
N/A

Other: which time schedule do you use? [Day(s) after VUS/VCUG, how many?]
N/A

Indication for prophylaxis

Which patients receive periinterventional antibiotic prophylaxis? (multiple answers possible) [all]
No

Which patients receive periinterventional antibiotic prophylaxis? (multiple answers possible) [patients post urinary tract infection/pyelonephritis/urosepsis]
No

Which patients receive periinterventional antibiotic prophylaxis? (multiple answers possible) [patients with urinary tract dilation in ultrasound]
No

Which patients receive periinterventional antibiotic prophylaxis? (multiple answers possible) [patients with prenatal urinary tract dilation]
No

Which patients receive periinterventional antibiotic prophylaxis? (multiple answers possible) [patients post VUR surgery]
No

Which patients receive periinterventional antibiotic prophylaxis? (multiple answers possible) [none]
No

Which patients receive periinterventional antibiotic prophylaxis? (multiple answers possible) [Other]
bei nachgewiesenem VUR wird nach der MUS/MCU mit Katheter einmalig Prophylaxe verabreicht.

Are there situations or clinical findings in the patient where you deviate from your prophylaxis standard (medication, dose, duration, age of the patient)? (multiple answers possible, please specify in answer box) [in case of already known vesicoureteral reflux (VUR)]
No

Are there situations or clinical findings in the patient where you deviate from your prophylaxis standard (medication, dose, duration, age of the patient)? (multiple answers possible, please specify in answer box) [in case of already known vesicoureteral reflux (VUR)][Comment]

Are there situations or clinical findings in the patient where you deviate from your prophylaxis standard (medication, dose, duration, age of the patient)? (multiple answers possible, please specify in answer box) [in patients with recurrent urinary tract infections (UTI)]
No

Are there situations or clinical findings in the patient where you deviate from your prophylaxis standard (medication, dose, duration, age of the patient)? (multiple answers possible, please specify in answer box) [in patients with recurrent urinary tract infections (UTI)][Comment]

Are there situations or clinical findings in the patient where you deviate from your prophylaxis standard (medication, dose, duration, age of the patient)? (multiple answers possible, please specify in answer box) [if multidrug-resistant organisms (MDRO) are detected in urine cultures]
Yes

Are there situations or clinical findings in the patient where you deviate from your prophylaxis standard (medication, dose, duration, age of the patient)? (multiple answers possible, please specify in answer box) [if multidrug-resistant organisms (MDRO) are detected in urine cultures][Comment]

Are there situations or clinical findings in the patient where you deviate from your prophylaxis standard (medication, dose, duration, age of the patient)? (multiple answers possible, please specify in answer box) [in newborns]
Yes

Are there situations or clinical findings in the patient where you deviate from your prophylaxis standard (medication, dose, duration, age of the patient)? (multiple answers possible, please specify in answer box) [in newborns][Comment]

Are there situations or clinical findings in the patient where you deviate from your prophylaxis standard (medication, dose, duration, age of the patient)? (multiple answers possible, please specify in answer box) [Other]
Transplantatnieren

Are there situations or clinical findings in the patient where you deviate from your prophylaxis standard (medication, dose, duration, age of the patient)? (multiple answers possible, please specify in answer box) [Other comment]
Therapie auch Prophylaxe legt Nephrologe fest

Are there situations in which you use intravenous prophylaxis?
No

Voiding analysis

Are there differences in antibiotic prophylaxis between VUS and VCUG in your department?
No

Is a non-pathological urine strip test required in preparation for VUS/VCUG in your department?
No

Is a non-pathological urine culture required in preparation for VUS/VCUG in your department?
No

Do patients only receive post-procedure prophylaxis if vesicoureteral reflux (VUR) is detected in VUS/VCUG?
Yes

Please explain your approach:
in routine bei klinisch unauffäligem Patienten , d.h. keine klinischen Infektzeichen, bei Blasenkatheter und MUS/MCU wenn VUR, dann Cephaclor einmal oral Prophylaxe, ohne VUR keine Prophylaxe<br>Punktions-MCU oder bei langfristigem Dauerkatheter oder bei Cystofix in Blase dann Prophylaxe immer nach Rücksprache mit Nephrologen/Neonatologen.

Do other MUS/MCU findings (e.g. urethral valves) have an influence on post-procedure prophylaxis?
No

End of survey

Thank you for taking the time to answer this survey!

May we contact you personally if we have further questions?
Yes

E-mail address:
jens-peter.schenk@med.uni-heidelberg.de

Survey response 3

Response ID
4

Last page
0

Start language
de

Seed
257042641

Date started
2024-11-13 12:28:38

Date last action
2024-11-13 12:29:01

Standard Operation Procedure

Who fills out this survey?

Who fills out this survey? [Other]

In which type of hospital are you employed?

In which type of hospital are you employed? [Other]

In which country do you work?

Does your department have a defined protocol for infection prophylaxis in the context of VUS/VCUG?
N/A

Has this protocol been transformed into a written standard operating procedure (SOP)?
N/A

Antibiotic Prophylaxis

Are there situations/constellations in which you do not use antibiotic prophylaxis for VUS/VCUG?
N/A

Please specify these situations/constellations: [girl with first uncomplicated UTI without urinary tract dilation in ultrasound]
N/A

Please specify these situations/constellations: [boy with first uncomplicated UTI without urinary tract dilation in ultrasound]
N/A

Please specify these situations/constellations: [in patients with low grade urinary tract dilation (up to 2nd grade) and no UTI in medical history]
N/A

If you use antibiotic prophylaxis: which mediaction do you use? (multiple answers and subdivision into age groups possible) [Trimethoprim]
N/A

If you use antibiotic prophylaxis: which mediaction do you use? (multiple answers and subdivision into age groups possible) [Nitrofurantoin]
N/A

If you use antibiotic prophylaxis: which mediaction do you use? (multiple answers and subdivision into age groups possible) [Amoxicillin]
N/A

If you use antibiotic prophylaxis: which mediaction do you use? (multiple answers and subdivision into age groups possible) [Cefaclor]
N/A

If you use antibiotic prophylaxis: which mediaction do you use? (multiple answers and subdivision into age groups possible) [Amoxicillin/clavulanic acid]
N/A

If you use antibiotic prophylaxis: which mediaction do you use? (multiple answers and subdivision into age groups possible) [Other]
N/A

Trimethoprim: which time schedule do you use? [Day(s) before VUS/VCUG, how many?]
N/A

Trimethoprim: which time schedule do you use? [Day of VUS/VCUG]
N/A

Trimethoprim: which time schedule do you use? [Day(s) after VUS/VCUG, how many?]
N/A

Nitrofurantoin: which time schedule do you use? [Day(s) before VUS/VCUG, how many?]
N/A

Nitrofurantoin: which time schedule do you use? [Day of VUS/VCUG]
N/A

Nitrofurantoin: which time schedule do you use? [Day(s) after VUS/VCUG, how many?]
N/A

Amoxicillin: which time schedule do you use? [Day(s) before VUS/VCUG, how many?]
N/A

Amoxicillin: which time schedule do you use? [Day of VUS/VCUG]
N/A

Amoxicillin: which time schedule do you use? [Day(s) after VUS/VCUG, how many?]
N/A

Cefaclor: which time schedule do you use? [Days(s) before VUS/VCUG, how many?]
N/A

Cefaclor: which time schedule do you use? [Day of VUS/VCUG]
N/A

Cefaclor: which time schedule do you use? [Day(s) after VUS/VCUG, how many?]
N/A

Amoxicillin/clavulanic acid: in what dosage? (Amoxicillin portion) [prophylaktic (10-20 mg/Kg bodyweight/d)]
N/A

Amoxicillin/clavulanic acid: in what dosage? (Amoxicillin portion) [double prophylactic (20-40 mg/Kg bodyweight/d)]
N/A

Amoxicillin/clavulanic acid: in what dosage? (Amoxicillin portion) [therapeutic (80-100 mg/Kg bodyweight/d)]
N/A

Amoxicillin/clavulanic acid: which time schedule do you use? [Day(s) before VUS/VCUG]
N/A

Amoxicillin/clavulanic acid: which time schedule do you use? [Day of VUS/VCUG]
N/A

Amoxicillin/clavulanic acid: which time schedule do you use? [Day(s) after VUS/VCUG]
N/A

Other: which time schedule do you use? [Day(s) before VUS/VCUG, how many?]
N/A

Other: which time schedule do you use? [Day of VUS/VCUG]
N/A

Other: which time schedule do you use? [Day(s) after VUS/VCUG, how many?]
N/A

Indication for prophylaxis

Which patients receive periinterventional antibiotic prophylaxis? (multiple answers possible) [all]
N/A

Which patients receive periinterventional antibiotic prophylaxis? (multiple answers possible) [patients post urinary tract infection/pyelonephritis/urosepsis]
N/A

Which patients receive periinterventional antibiotic prophylaxis? (multiple answers possible) [patients with urinary tract dilation in ultrasound]
N/A

Which patients receive periinterventional antibiotic prophylaxis? (multiple answers possible) [patients with prenatal urinary tract dilation]
N/A

Which patients receive periinterventional antibiotic prophylaxis? (multiple answers possible) [patients post VUR surgery]
N/A

Which patients receive periinterventional antibiotic prophylaxis? (multiple answers possible) [none]
N/A

Are there situations or clinical findings in the patient where you deviate from your prophylaxis standard (medication, dose, duration, age of the patient)? (multiple answers possible, please specify in answer box) [in case of already known vesicoureteral reflux (VUR)]
N/A

Are there situations or clinical findings in the patient where you deviate from your prophylaxis standard (medication, dose, duration, age of the patient)? (multiple answers possible, please specify in answer box) [in patients with recurrent urinary tract infections (UTI)]
N/A

Are there situations or clinical findings in the patient where you deviate from your prophylaxis standard (medication, dose, duration, age of the patient)? (multiple answers possible, please specify in answer box) [if multidrug-resistant organisms (MDRO) are detected in urine cultures]
N/A

Are there situations or clinical findings in the patient where you deviate from your prophylaxis standard (medication, dose, duration, age of the patient)? (multiple answers possible, please specify in answer box) [in newborns]
N/A

Are there situations in which you use intravenous prophylaxis?
N/A

Voiding analysis

Are there differences in antibiotic prophylaxis between VUS and VCUG in your department?
N/A

Is a non-pathological urine strip test required in preparation for VUS/VCUG in your department?
N/A

Is a non-pathological urine culture required in preparation for VUS/VCUG in your department?
N/A

Do patients only receive post-procedure prophylaxis if vesicoureteral reflux (VUR) is detected in VUS/VCUG?
N/A

Do other MUS/MCU findings (e.g. urethral valves) have an influence on post-procedure prophylaxis?
N/A

End of survey

May we contact you personally if we have further questions?
N/A

Survey response 4

Response ID
5

Last page
1

Start language
de

Seed
697678275

Date started
2024-11-13 13:00:06

Date last action
2024-11-13 13:03:12

Standard Operation Procedure

Who fills out this survey?
Pediatric Radiologist

Who fills out this survey? [Other]

In which type of hospital are you employed?
Community hospital (

In which type of hospital are you employed? [Other]

In which country do you work?
Baden- Württemberg

Does your department have a defined protocol for infection prophylaxis in the context of VUS/VCUG?
Yes

Has this protocol been transformed into a written standard operating procedure (SOP)?
No

Antibiotic Prophylaxis

Are there situations/constellations in which you do not use antibiotic prophylaxis for VUS/VCUG?
Yes

Please specify these situations/constellations: [girl with first uncomplicated UTI without urinary tract dilation in ultrasound]
No

Please specify these situations/constellations: [boy with first uncomplicated UTI without urinary tract dilation in ultrasound]
No

Please specify these situations/constellations: [in patients with low grade urinary tract dilation (up to 2nd grade) and no UTI in medical history]
No

Please specify these situations/constellations: [Other]

If you use antibiotic prophylaxis: which mediaction do you use? (multiple answers and subdivision into age groups possible) [Trimethoprim]
No

If you use antibiotic prophylaxis: which mediaction do you use? (multiple answers and subdivision into age groups possible) [Nitrofurantoin]
No

If you use antibiotic prophylaxis: which mediaction do you use? (multiple answers and subdivision into age groups possible) [Amoxicillin]
No

If you use antibiotic prophylaxis: which mediaction do you use? (multiple answers and subdivision into age groups possible) [Cefaclor]
Yes

If you use antibiotic prophylaxis: which mediaction do you use? (multiple answers and subdivision into age groups possible) [Amoxicillin/clavulanic acid]
No

If you use antibiotic prophylaxis: which mediaction do you use? (multiple answers and subdivision into age groups possible) [Other]
No

Trimethoprim: which time schedule do you use? [Day(s) before VUS/VCUG, how many?]
N/A

Trimethoprim: which time schedule do you use? [Day of VUS/VCUG]
N/A

Trimethoprim: which time schedule do you use? [Day(s) after VUS/VCUG, how many?]
N/A

Nitrofurantoin: which time schedule do you use? [Day(s) before VUS/VCUG, how many?]
N/A

Nitrofurantoin: which time schedule do you use? [Day of VUS/VCUG]
N/A

Nitrofurantoin: which time schedule do you use? [Day(s) after VUS/VCUG, how many?]
N/A

Amoxicillin: which time schedule do you use? [Day(s) before VUS/VCUG, how many?]
N/A

Amoxicillin: which time schedule do you use? [Day of VUS/VCUG]
N/A

Amoxicillin: which time schedule do you use? [Day(s) after VUS/VCUG, how many?]
N/A

Cefaclor: in what dosage?

Cefaclor: in what dosage? [Other]

Cefaclor: for which age group do you use cefaclor?

Cefaclor: which time schedule do you use? [Days(s) before VUS/VCUG, how many?]
No

Cefaclor: which time schedule do you use? [Days(s) before VUS/VCUG, how many?][Comment]

Cefaclor: which time schedule do you use? [Day of VUS/VCUG]
No

Cefaclor: which time schedule do you use? [Day of VUS/VCUG][Comment]

Cefaclor: which time schedule do you use? [Day(s) after VUS/VCUG, how many?]
No

Cefaclor: which time schedule do you use? [Day(s) after VUS/VCUG, how many?][Comment]

Amoxicillin/clavulanic acid: in what dosage? (Amoxicillin portion) [prophylaktic (10-20 mg/Kg bodyweight/d)]
N/A

Amoxicillin/clavulanic acid: in what dosage? (Amoxicillin portion) [double prophylactic (20-40 mg/Kg bodyweight/d)]
N/A

Amoxicillin/clavulanic acid: in what dosage? (Amoxicillin portion) [therapeutic (80-100 mg/Kg bodyweight/d)]
N/A

Amoxicillin/clavulanic acid: which time schedule do you use? [Day(s) before VUS/VCUG]
N/A

Amoxicillin/clavulanic acid: which time schedule do you use? [Day of VUS/VCUG]
N/A

Amoxicillin/clavulanic acid: which time schedule do you use? [Day(s) after VUS/VCUG]
N/A

Other: which time schedule do you use? [Day(s) before VUS/VCUG, how many?]
N/A

Other: which time schedule do you use? [Day of VUS/VCUG]
N/A

Other: which time schedule do you use? [Day(s) after VUS/VCUG, how many?]
N/A

Indication for prophylaxis

Which patients receive periinterventional antibiotic prophylaxis? (multiple answers possible) [all]
N/A

Which patients receive periinterventional antibiotic prophylaxis? (multiple answers possible) [patients post urinary tract infection/pyelonephritis/urosepsis]
N/A

Which patients receive periinterventional antibiotic prophylaxis? (multiple answers possible) [patients with urinary tract dilation in ultrasound]
N/A

Which patients receive periinterventional antibiotic prophylaxis? (multiple answers possible) [patients with prenatal urinary tract dilation]
N/A

Which patients receive periinterventional antibiotic prophylaxis? (multiple answers possible) [patients post VUR surgery]
N/A

Which patients receive periinterventional antibiotic prophylaxis? (multiple answers possible) [none]
N/A

Are there situations or clinical findings in the patient where you deviate from your prophylaxis standard (medication, dose, duration, age of the patient)? (multiple answers possible, please specify in answer box) [in case of already known vesicoureteral reflux (VUR)]
N/A

Are there situations or clinical findings in the patient where you deviate from your prophylaxis standard (medication, dose, duration, age of the patient)? (multiple answers possible, please specify in answer box) [in patients with recurrent urinary tract infections (UTI)]
N/A

Are there situations or clinical findings in the patient where you deviate from your prophylaxis standard (medication, dose, duration, age of the patient)? (multiple answers possible, please specify in answer box) [if multidrug-resistant organisms (MDRO) are detected in urine cultures]
N/A

Are there situations or clinical findings in the patient where you deviate from your prophylaxis standard (medication, dose, duration, age of the patient)? (multiple answers possible, please specify in answer box) [in newborns]
N/A

Are there situations in which you use intravenous prophylaxis?
N/A

Voiding analysis

Are there differences in antibiotic prophylaxis between VUS and VCUG in your department?
N/A

Is a non-pathological urine strip test required in preparation for VUS/VCUG in your department?
N/A

Is a non-pathological urine culture required in preparation for VUS/VCUG in your department?
N/A

Do patients only receive post-procedure prophylaxis if vesicoureteral reflux (VUR) is detected in VUS/VCUG?
N/A

Do other MUS/MCU findings (e.g. urethral valves) have an influence on post-procedure prophylaxis?
N/A

End of survey

May we contact you personally if we have further questions?
N/A

Survey response 5

Response ID
6

Date submitted
2024-11-13 13:27:43

Last page
5

Start language
de

Seed
2055405298

Date started
2024-11-13 13:16:26

Date last action
2024-11-13 13:27:43

Standard Operation Procedure

Who fills out this survey?
Pediatric Radiologist

Who fills out this survey? [Other]

In which type of hospital are you employed?
University Hospital

In which type of hospital are you employed? [Other]

In which country do you work?
Österreich

Does your department have a defined protocol for infection prophylaxis in the context of VUS/VCUG?
Yes

Has this protocol been transformed into a written standard operating procedure (SOP)?
Yes

Which department is the author of the SOP for infection prophylaxis in VUS/VCUG?
Pediatric radiology

Which department is the author of the SOP for infection prophylaxis in VUS/VCUG? [Other]

Antibiotic Prophylaxis

Are there situations/constellations in which you do not use antibiotic prophylaxis for VUS/VCUG?
No

Please specify these situations/constellations: [girl with first uncomplicated UTI without urinary tract dilation in ultrasound]
N/A

Please specify these situations/constellations: [boy with first uncomplicated UTI without urinary tract dilation in ultrasound]
N/A

Please specify these situations/constellations: [in patients with low grade urinary tract dilation (up to 2nd grade) and no UTI in medical history]
N/A

If you use antibiotic prophylaxis: which mediaction do you use? (multiple answers and subdivision into age groups possible) [Trimethoprim]
No

If you use antibiotic prophylaxis: which mediaction do you use? (multiple answers and subdivision into age groups possible) [Nitrofurantoin]
No

If you use antibiotic prophylaxis: which mediaction do you use? (multiple answers and subdivision into age groups possible) [Amoxicillin]
No

If you use antibiotic prophylaxis: which mediaction do you use? (multiple answers and subdivision into age groups possible) [Cefaclor]
Yes

If you use antibiotic prophylaxis: which mediaction do you use? (multiple answers and subdivision into age groups possible) [Amoxicillin/clavulanic acid]
No

If you use antibiotic prophylaxis: which mediaction do you use? (multiple answers and subdivision into age groups possible) [Other]
No

Trimethoprim: which time schedule do you use? [Day(s) before VUS/VCUG, how many?]
N/A

Trimethoprim: which time schedule do you use? [Day of VUS/VCUG]
N/A

Trimethoprim: which time schedule do you use? [Day(s) after VUS/VCUG, how many?]
N/A

Nitrofurantoin: which time schedule do you use? [Day(s) before VUS/VCUG, how many?]
N/A

Nitrofurantoin: which time schedule do you use? [Day of VUS/VCUG]
N/A

Nitrofurantoin: which time schedule do you use? [Day(s) after VUS/VCUG, how many?]
N/A

Amoxicillin: which time schedule do you use? [Day(s) before VUS/VCUG, how many?]
N/A

Amoxicillin: which time schedule do you use? [Day of VUS/VCUG]
N/A

Amoxicillin: which time schedule do you use? [Day(s) after VUS/VCUG, how many?]
N/A

Cefaclor: in what dosage?
prophylactic (10 mg/kg bodyweight/d)

Cefaclor: in what dosage? [Other]

Cefaclor: for which age group do you use cefaclor?
alle

Cefaclor: which time schedule do you use? [Days(s) before VUS/VCUG, how many?]
No

Cefaclor: which time schedule do you use? [Days(s) before VUS/VCUG, how many?][Comment]

Cefaclor: which time schedule do you use? [Day of VUS/VCUG]
Yes

Cefaclor: which time schedule do you use? [Day of VUS/VCUG][Comment]

Cefaclor: which time schedule do you use? [Day(s) after VUS/VCUG, how many?]
No

Cefaclor: which time schedule do you use? [Day(s) after VUS/VCUG, how many?][Comment]

Amoxicillin/clavulanic acid: in what dosage? (Amoxicillin portion) [prophylaktic (10-20 mg/Kg bodyweight/d)]
N/A

Amoxicillin/clavulanic acid: in what dosage? (Amoxicillin portion) [double prophylactic (20-40 mg/Kg bodyweight/d)]
N/A

Amoxicillin/clavulanic acid: in what dosage? (Amoxicillin portion) [therapeutic (80-100 mg/Kg bodyweight/d)]
N/A

Amoxicillin/clavulanic acid: which time schedule do you use? [Day(s) before VUS/VCUG]
N/A

Amoxicillin/clavulanic acid: which time schedule do you use? [Day of VUS/VCUG]
N/A

Amoxicillin/clavulanic acid: which time schedule do you use? [Day(s) after VUS/VCUG]
N/A

Other: which time schedule do you use? [Day(s) before VUS/VCUG, how many?]
N/A

Other: which time schedule do you use? [Day of VUS/VCUG]
N/A

Other: which time schedule do you use? [Day(s) after VUS/VCUG, how many?]
N/A

Indication for prophylaxis

Which patients receive periinterventional antibiotic prophylaxis? (multiple answers possible) [all]
Yes

Which patients receive periinterventional antibiotic prophylaxis? (multiple answers possible) [patients post urinary tract infection/pyelonephritis/urosepsis]
N/A

Which patients receive periinterventional antibiotic prophylaxis? (multiple answers possible) [patients with urinary tract dilation in ultrasound]
N/A

Which patients receive periinterventional antibiotic prophylaxis? (multiple answers possible) [patients with prenatal urinary tract dilation]
N/A

Which patients receive periinterventional antibiotic prophylaxis? (multiple answers possible) [patients post VUR surgery]
N/A

Which patients receive periinterventional antibiotic prophylaxis? (multiple answers possible) [none]
N/A

Are there situations or clinical findings in the patient where you deviate from your prophylaxis standard (medication, dose, duration, age of the patient)? (multiple answers possible, please specify in answer box) [in case of already known vesicoureteral reflux (VUR)]
Yes

Are there situations or clinical findings in the patient where you deviate from your prophylaxis standard (medication, dose, duration, age of the patient)? (multiple answers possible, please specify in answer box) [in case of already known vesicoureteral reflux (VUR)][Comment]
Nur bei bereits bestehender AB-Dauerprophylaxe wird kein Cefaclor gegeben.

Are there situations or clinical findings in the patient where you deviate from your prophylaxis standard (medication, dose, duration, age of the patient)? (multiple answers possible, please specify in answer box) [in patients with recurrent urinary tract infections (UTI)]
Yes

Are there situations or clinical findings in the patient where you deviate from your prophylaxis standard (medication, dose, duration, age of the patient)? (multiple answers possible, please specify in answer box) [in patients with recurrent urinary tract infections (UTI)][Comment]
Nur bei bereits bestehender AB-Dauerprophylaxe wird kein Cefaclor gegeben.

Are there situations or clinical findings in the patient where you deviate from your prophylaxis standard (medication, dose, duration, age of the patient)? (multiple answers possible, please specify in answer box) [if multidrug-resistant organisms (MDRO) are detected in urine cultures]
No

Are there situations or clinical findings in the patient where you deviate from your prophylaxis standard (medication, dose, duration, age of the patient)? (multiple answers possible, please specify in answer box) [if multidrug-resistant organisms (MDRO) are detected in urine cultures][Comment]

Are there situations or clinical findings in the patient where you deviate from your prophylaxis standard (medication, dose, duration, age of the patient)? (multiple answers possible, please specify in answer box) [in newborns]
No

Are there situations or clinical findings in the patient where you deviate from your prophylaxis standard (medication, dose, duration, age of the patient)? (multiple answers possible, please specify in answer box) [in newborns][Comment]

Are there situations or clinical findings in the patient where you deviate from your prophylaxis standard (medication, dose, duration, age of the patient)? (multiple answers possible, please specify in answer box) [Other]
bei Dauerprophylaxe

Are there situations or clinical findings in the patient where you deviate from your prophylaxis standard (medication, dose, duration, age of the patient)? (multiple answers possible, please specify in answer box) [Other comment]
Nur bei bereits bestehender AB-Dauerprophylaxe wird kein Cefaclor gegeben.

Are there situations in which you use intravenous prophylaxis?
No

Voiding analysis

Are there differences in antibiotic prophylaxis between VUS and VCUG in your department?
No

Is a non-pathological urine strip test required in preparation for VUS/VCUG in your department?
No

Is a non-pathological urine culture required in preparation for VUS/VCUG in your department?
No

Do patients only receive post-procedure prophylaxis if vesicoureteral reflux (VUR) is detected in VUS/VCUG?
No

Do other MUS/MCU findings (e.g. urethral valves) have an influence on post-procedure prophylaxis?
No

End of survey

Thank you for taking the time to answer this survey!

May we contact you personally if we have further questions?
Yes

E-mail address:
azadeh.hojreh@meduniwien.ac.at

Survey response 6

Response ID
7

Last page
0

Start language
de

Seed
518773894

Date started
2024-11-13 14:49:04

Date last action
2024-11-13 14:49:04

Standard Operation Procedure

Does your department have a defined protocol for infection prophylaxis in the context of VUS/VCUG?
N/A

Has this protocol been transformed into a written standard operating procedure (SOP)?
N/A

Antibiotic Prophylaxis

Are there situations/constellations in which you do not use antibiotic prophylaxis for VUS/VCUG?
N/A

Please specify these situations/constellations: [girl with first uncomplicated UTI without urinary tract dilation in ultrasound]
N/A

Please specify these situations/constellations: [boy with first uncomplicated UTI without urinary tract dilation in ultrasound]
N/A

Please specify these situations/constellations: [in patients with low grade urinary tract dilation (up to 2nd grade) and no UTI in medical history]
N/A

If you use antibiotic prophylaxis: which mediaction do you use? (multiple answers and subdivision into age groups possible) [Trimethoprim]
N/A

If you use antibiotic prophylaxis: which mediaction do you use? (multiple answers and subdivision into age groups possible) [Nitrofurantoin]
N/A

If you use antibiotic prophylaxis: which mediaction do you use? (multiple answers and subdivision into age groups possible) [Amoxicillin]
N/A

If you use antibiotic prophylaxis: which mediaction do you use? (multiple answers and subdivision into age groups possible) [Cefaclor]
N/A

If you use antibiotic prophylaxis: which mediaction do you use? (multiple answers and subdivision into age groups possible) [Amoxicillin/clavulanic acid]
N/A

If you use antibiotic prophylaxis: which mediaction do you use? (multiple answers and subdivision into age groups possible) [Other]
N/A

Trimethoprim: which time schedule do you use? [Day(s) before VUS/VCUG, how many?]
N/A

Trimethoprim: which time schedule do you use? [Day of VUS/VCUG]
N/A

Trimethoprim: which time schedule do you use? [Day(s) after VUS/VCUG, how many?]
N/A

Nitrofurantoin: which time schedule do you use? [Day(s) before VUS/VCUG, how many?]
N/A

Nitrofurantoin: which time schedule do you use? [Day of VUS/VCUG]
N/A

Nitrofurantoin: which time schedule do you use? [Day(s) after VUS/VCUG, how many?]
N/A

Amoxicillin: which time schedule do you use? [Day(s) before VUS/VCUG, how many?]
N/A

Amoxicillin: which time schedule do you use? [Day of VUS/VCUG]
N/A

Amoxicillin: which time schedule do you use? [Day(s) after VUS/VCUG, how many?]
N/A

Cefaclor: which time schedule do you use? [Days(s) before VUS/VCUG, how many?]
N/A

Cefaclor: which time schedule do you use? [Day of VUS/VCUG]
N/A

Cefaclor: which time schedule do you use? [Day(s) after VUS/VCUG, how many?]
N/A

Amoxicillin/clavulanic acid: in what dosage? (Amoxicillin portion) [prophylaktic (10-20 mg/Kg bodyweight/d)]
N/A

Amoxicillin/clavulanic acid: in what dosage? (Amoxicillin portion) [double prophylactic (20-40 mg/Kg bodyweight/d)]
N/A

Amoxicillin/clavulanic acid: in what dosage? (Amoxicillin portion) [therapeutic (80-100 mg/Kg bodyweight/d)]
N/A

Amoxicillin/clavulanic acid: which time schedule do you use? [Day(s) before VUS/VCUG]
N/A

Amoxicillin/clavulanic acid: which time schedule do you use? [Day of VUS/VCUG]
N/A

Amoxicillin/clavulanic acid: which time schedule do you use? [Day(s) after VUS/VCUG]
N/A

Other: which time schedule do you use? [Day(s) before VUS/VCUG, how many?]
N/A

Other: which time schedule do you use? [Day of VUS/VCUG]
N/A

Other: which time schedule do you use? [Day(s) after VUS/VCUG, how many?]
N/A

Indication for prophylaxis

Which patients receive periinterventional antibiotic prophylaxis? (multiple answers possible) [all]
N/A

Which patients receive periinterventional antibiotic prophylaxis? (multiple answers possible) [patients post urinary tract infection/pyelonephritis/urosepsis]
N/A

Which patients receive periinterventional antibiotic prophylaxis? (multiple answers possible) [patients with urinary tract dilation in ultrasound]
N/A

Which patients receive periinterventional antibiotic prophylaxis? (multiple answers possible) [patients with prenatal urinary tract dilation]
N/A

Which patients receive periinterventional antibiotic prophylaxis? (multiple answers possible) [patients post VUR surgery]
N/A

Which patients receive periinterventional antibiotic prophylaxis? (multiple answers possible) [none]
N/A

Are there situations or clinical findings in the patient where you deviate from your prophylaxis standard (medication, dose, duration, age of the patient)? (multiple answers possible, please specify in answer box) [in case of already known vesicoureteral reflux (VUR)]
N/A

Are there situations or clinical findings in the patient where you deviate from your prophylaxis standard (medication, dose, duration, age of the patient)? (multiple answers possible, please specify in answer box) [in patients with recurrent urinary tract infections (UTI)]
N/A

Are there situations or clinical findings in the patient where you deviate from your prophylaxis standard (medication, dose, duration, age of the patient)? (multiple answers possible, please specify in answer box) [if multidrug-resistant organisms (MDRO) are detected in urine cultures]
N/A

Are there situations or clinical findings in the patient where you deviate from your prophylaxis standard (medication, dose, duration, age of the patient)? (multiple answers possible, please specify in answer box) [in newborns]
N/A

Are there situations in which you use intravenous prophylaxis?
N/A

Voiding analysis

Are there differences in antibiotic prophylaxis between VUS and VCUG in your department?
N/A

Is a non-pathological urine strip test required in preparation for VUS/VCUG in your department?
N/A

Is a non-pathological urine culture required in preparation for VUS/VCUG in your department?
N/A

Do patients only receive post-procedure prophylaxis if vesicoureteral reflux (VUR) is detected in VUS/VCUG?
N/A

Do other MUS/MCU findings (e.g. urethral valves) have an influence on post-procedure prophylaxis?
N/A

End of survey

May we contact you personally if we have further questions?
N/A

Survey response 7

Response ID
8

Last page
0

Start language
de

Seed
1879978949

Date started
2024-11-13 15:33:14

Date last action
2024-11-13 15:33:14

Standard Operation Procedure

Does your department have a defined protocol for infection prophylaxis in the context of VUS/VCUG?
N/A

Has this protocol been transformed into a written standard operating procedure (SOP)?
N/A

Antibiotic Prophylaxis

Are there situations/constellations in which you do not use antibiotic prophylaxis for VUS/VCUG?
N/A

Please specify these situations/constellations: [girl with first uncomplicated UTI without urinary tract dilation in ultrasound]
N/A

Please specify these situations/constellations: [boy with first uncomplicated UTI without urinary tract dilation in ultrasound]
N/A

Please specify these situations/constellations: [in patients with low grade urinary tract dilation (up to 2nd grade) and no UTI in medical history]
N/A

If you use antibiotic prophylaxis: which mediaction do you use? (multiple answers and subdivision into age groups possible) [Trimethoprim]
N/A

If you use antibiotic prophylaxis: which mediaction do you use? (multiple answers and subdivision into age groups possible) [Nitrofurantoin]
N/A

If you use antibiotic prophylaxis: which mediaction do you use? (multiple answers and subdivision into age groups possible) [Amoxicillin]
N/A

If you use antibiotic prophylaxis: which mediaction do you use? (multiple answers and subdivision into age groups possible) [Cefaclor]
N/A

If you use antibiotic prophylaxis: which mediaction do you use? (multiple answers and subdivision into age groups possible) [Amoxicillin/clavulanic acid]
N/A

If you use antibiotic prophylaxis: which mediaction do you use? (multiple answers and subdivision into age groups possible) [Other]
N/A

Trimethoprim: which time schedule do you use? [Day(s) before VUS/VCUG, how many?]
N/A

Trimethoprim: which time schedule do you use? [Day of VUS/VCUG]
N/A

Trimethoprim: which time schedule do you use? [Day(s) after VUS/VCUG, how many?]
N/A

Nitrofurantoin: which time schedule do you use? [Day(s) before VUS/VCUG, how many?]
N/A

Nitrofurantoin: which time schedule do you use? [Day of VUS/VCUG]
N/A

Nitrofurantoin: which time schedule do you use? [Day(s) after VUS/VCUG, how many?]
N/A

Amoxicillin: which time schedule do you use? [Day(s) before VUS/VCUG, how many?]
N/A

Amoxicillin: which time schedule do you use? [Day of VUS/VCUG]
N/A

Amoxicillin: which time schedule do you use? [Day(s) after VUS/VCUG, how many?]
N/A

Cefaclor: which time schedule do you use? [Days(s) before VUS/VCUG, how many?]
N/A

Cefaclor: which time schedule do you use? [Day of VUS/VCUG]
N/A

Cefaclor: which time schedule do you use? [Day(s) after VUS/VCUG, how many?]
N/A

Amoxicillin/clavulanic acid: in what dosage? (Amoxicillin portion) [prophylaktic (10-20 mg/Kg bodyweight/d)]
N/A

Amoxicillin/clavulanic acid: in what dosage? (Amoxicillin portion) [double prophylactic (20-40 mg/Kg bodyweight/d)]
N/A

Amoxicillin/clavulanic acid: in what dosage? (Amoxicillin portion) [therapeutic (80-100 mg/Kg bodyweight/d)]
N/A

Amoxicillin/clavulanic acid: which time schedule do you use? [Day(s) before VUS/VCUG]
N/A

Amoxicillin/clavulanic acid: which time schedule do you use? [Day of VUS/VCUG]
N/A

Amoxicillin/clavulanic acid: which time schedule do you use? [Day(s) after VUS/VCUG]
N/A

Other: which time schedule do you use? [Day(s) before VUS/VCUG, how many?]
N/A

Other: which time schedule do you use? [Day of VUS/VCUG]
N/A

Other: which time schedule do you use? [Day(s) after VUS/VCUG, how many?]
N/A

Indication for prophylaxis

Which patients receive periinterventional antibiotic prophylaxis? (multiple answers possible) [all]
N/A

Which patients receive periinterventional antibiotic prophylaxis? (multiple answers possible) [patients post urinary tract infection/pyelonephritis/urosepsis]
N/A

Which patients receive periinterventional antibiotic prophylaxis? (multiple answers possible) [patients with urinary tract dilation in ultrasound]
N/A

Which patients receive periinterventional antibiotic prophylaxis? (multiple answers possible) [patients with prenatal urinary tract dilation]
N/A

Which patients receive periinterventional antibiotic prophylaxis? (multiple answers possible) [patients post VUR surgery]
N/A

Which patients receive periinterventional antibiotic prophylaxis? (multiple answers possible) [none]
N/A

Are there situations or clinical findings in the patient where you deviate from your prophylaxis standard (medication, dose, duration, age of the patient)? (multiple answers possible, please specify in answer box) [in case of already known vesicoureteral reflux (VUR)]
N/A

Are there situations or clinical findings in the patient where you deviate from your prophylaxis standard (medication, dose, duration, age of the patient)? (multiple answers possible, please specify in answer box) [in patients with recurrent urinary tract infections (UTI)]
N/A

Are there situations or clinical findings in the patient where you deviate from your prophylaxis standard (medication, dose, duration, age of the patient)? (multiple answers possible, please specify in answer box) [if multidrug-resistant organisms (MDRO) are detected in urine cultures]
N/A

Are there situations or clinical findings in the patient where you deviate from your prophylaxis standard (medication, dose, duration, age of the patient)? (multiple answers possible, please specify in answer box) [in newborns]
N/A

Are there situations in which you use intravenous prophylaxis?
N/A

Voiding analysis

Are there differences in antibiotic prophylaxis between VUS and VCUG in your department?
N/A

Is a non-pathological urine strip test required in preparation for VUS/VCUG in your department?
N/A

Is a non-pathological urine culture required in preparation for VUS/VCUG in your department?
N/A

Do patients only receive post-procedure prophylaxis if vesicoureteral reflux (VUR) is detected in VUS/VCUG?
N/A

Do other MUS/MCU findings (e.g. urethral valves) have an influence on post-procedure prophylaxis?
N/A

End of survey

May we contact you personally if we have further questions?
N/A

Survey response 8

Response ID
9

Date submitted
2024-11-13 15:57:48

Last page
5

Start language
de

Seed
589522374

Date started
2024-11-13 15:45:54

Date last action
2024-11-13 15:57:48

Standard Operation Procedure

Who fills out this survey?
Pediatric Radiologist

Who fills out this survey? [Other]

In which type of hospital are you employed?
University Hospital

In which type of hospital are you employed? [Other]

In which country do you work?
Deutschland

Does your department have a defined protocol for infection prophylaxis in the context of VUS/VCUG?
Yes

Has this protocol been transformed into a written standard operating procedure (SOP)?
Yes

Which department is the author of the SOP for infection prophylaxis in VUS/VCUG?
Pediatric nephrology

Which department is the author of the SOP for infection prophylaxis in VUS/VCUG? [Other]

Antibiotic Prophylaxis

Are there situations/constellations in which you do not use antibiotic prophylaxis for VUS/VCUG?
Yes

Please specify these situations/constellations: [girl with first uncomplicated UTI without urinary tract dilation in ultrasound]
No

Please specify these situations/constellations: [boy with first uncomplicated UTI without urinary tract dilation in ultrasound]
No

Please specify these situations/constellations: [in patients with low grade urinary tract dilation (up to 2nd grade) and no UTI in medical history]
No

Please specify these situations/constellations: [Other]
bei liegendem Katheter; bei antibiotischer Therapie

If you use antibiotic prophylaxis: which mediaction do you use? (multiple answers and subdivision into age groups possible) [Trimethoprim]
Yes

If you use antibiotic prophylaxis: which mediaction do you use? (multiple answers and subdivision into age groups possible) [Nitrofurantoin]
No

If you use antibiotic prophylaxis: which mediaction do you use? (multiple answers and subdivision into age groups possible) [Amoxicillin]
Yes

If you use antibiotic prophylaxis: which mediaction do you use? (multiple answers and subdivision into age groups possible) [Cefaclor]
Yes

If you use antibiotic prophylaxis: which mediaction do you use? (multiple answers and subdivision into age groups possible) [Amoxicillin/clavulanic acid]
No

If you use antibiotic prophylaxis: which mediaction do you use? (multiple answers and subdivision into age groups possible) [Other]
No

Trimethoprim: in what dosage?
prophylactic (2 mg/kg bodyweight/d)

Trimethoprim: in what dosage? [Other]

Trimethoprim: for which age group do you use trimethoprim?
ältere Kinder

Trimethoprim: which time schedule do you use? [Day(s) before VUS/VCUG, how many?]
No

Trimethoprim: which time schedule do you use? [Day(s) before VUS/VCUG, how many?][Comment]

Trimethoprim: which time schedule do you use? [Day of VUS/VCUG]
Yes

Trimethoprim: which time schedule do you use? [Day of VUS/VCUG][Comment]

Trimethoprim: which time schedule do you use? [Day(s) after VUS/VCUG, how many?]
Yes

Trimethoprim: which time schedule do you use? [Day(s) after VUS/VCUG, how many?][Comment]
3

Nitrofurantoin: which time schedule do you use? [Day(s) before VUS/VCUG, how many?]
N/A

Nitrofurantoin: which time schedule do you use? [Day of VUS/VCUG]
N/A

Nitrofurantoin: which time schedule do you use? [Day(s) after VUS/VCUG, how many?]
N/A

Amoxicillin: in what dosage?
therapeutic (50-100 mg/kg bodyweight/d)

Amoxicillin: in what dosage? [Other]

Amoxicillin: for which age group do you use amoxicillin?
< 2 Jahre

Amoxicillin: which time schedule do you use? [Day(s) before VUS/VCUG, how many?]
No

Amoxicillin: which time schedule do you use? [Day(s) before VUS/VCUG, how many?][Comment]

Amoxicillin: which time schedule do you use? [Day of VUS/VCUG]
Yes

Amoxicillin: which time schedule do you use? [Day of VUS/VCUG][Comment]

Amoxicillin: which time schedule do you use? [Day(s) after VUS/VCUG, how many?]
Yes

Amoxicillin: which time schedule do you use? [Day(s) after VUS/VCUG, how many?][Comment]
3

Cefaclor: in what dosage?
double prophylactic (20 mg/kg bodyweight/d)

Cefaclor: in what dosage? [Other]

Cefaclor: for which age group do you use cefaclor?
Neugeborene und Säuglinge bis 3 Lebensmonate

Cefaclor: which time schedule do you use? [Days(s) before VUS/VCUG, how many?]
No

Cefaclor: which time schedule do you use? [Days(s) before VUS/VCUG, how many?][Comment]

Cefaclor: which time schedule do you use? [Day of VUS/VCUG]
Yes

Cefaclor: which time schedule do you use? [Day of VUS/VCUG][Comment]

Cefaclor: which time schedule do you use? [Day(s) after VUS/VCUG, how many?]
Yes

Cefaclor: which time schedule do you use? [Day(s) after VUS/VCUG, how many?][Comment]
3

Amoxicillin/clavulanic acid: in what dosage? (Amoxicillin portion) [prophylaktic (10-20 mg/Kg bodyweight/d)]
N/A

Amoxicillin/clavulanic acid: in what dosage? (Amoxicillin portion) [double prophylactic (20-40 mg/Kg bodyweight/d)]
N/A

Amoxicillin/clavulanic acid: in what dosage? (Amoxicillin portion) [therapeutic (80-100 mg/Kg bodyweight/d)]
N/A

Amoxicillin/clavulanic acid: which time schedule do you use? [Day(s) before VUS/VCUG]
N/A

Amoxicillin/clavulanic acid: which time schedule do you use? [Day of VUS/VCUG]
N/A

Amoxicillin/clavulanic acid: which time schedule do you use? [Day(s) after VUS/VCUG]
N/A

Other: which time schedule do you use? [Day(s) before VUS/VCUG, how many?]
N/A

Other: which time schedule do you use? [Day of VUS/VCUG]
N/A

Other: which time schedule do you use? [Day(s) after VUS/VCUG, how many?]
N/A

Indication for prophylaxis

Which patients receive periinterventional antibiotic prophylaxis? (multiple answers possible) [all]
Yes

Which patients receive periinterventional antibiotic prophylaxis? (multiple answers possible) [patients post urinary tract infection/pyelonephritis/urosepsis]
N/A

Which patients receive periinterventional antibiotic prophylaxis? (multiple answers possible) [patients with urinary tract dilation in ultrasound]
N/A

Which patients receive periinterventional antibiotic prophylaxis? (multiple answers possible) [patients with prenatal urinary tract dilation]
N/A

Which patients receive periinterventional antibiotic prophylaxis? (multiple answers possible) [patients post VUR surgery]
N/A

Which patients receive periinterventional antibiotic prophylaxis? (multiple answers possible) [none]
N/A

Are there situations or clinical findings in the patient where you deviate from your prophylaxis standard (medication, dose, duration, age of the patient)? (multiple answers possible, please specify in answer box) [in case of already known vesicoureteral reflux (VUR)]
No

Are there situations or clinical findings in the patient where you deviate from your prophylaxis standard (medication, dose, duration, age of the patient)? (multiple answers possible, please specify in answer box) [in case of already known vesicoureteral reflux (VUR)][Comment]

Are there situations or clinical findings in the patient where you deviate from your prophylaxis standard (medication, dose, duration, age of the patient)? (multiple answers possible, please specify in answer box) [in patients with recurrent urinary tract infections (UTI)]
No

Are there situations or clinical findings in the patient where you deviate from your prophylaxis standard (medication, dose, duration, age of the patient)? (multiple answers possible, please specify in answer box) [in patients with recurrent urinary tract infections (UTI)][Comment]

Are there situations or clinical findings in the patient where you deviate from your prophylaxis standard (medication, dose, duration, age of the patient)? (multiple answers possible, please specify in answer box) [if multidrug-resistant organisms (MDRO) are detected in urine cultures]
No

Are there situations or clinical findings in the patient where you deviate from your prophylaxis standard (medication, dose, duration, age of the patient)? (multiple answers possible, please specify in answer box) [if multidrug-resistant organisms (MDRO) are detected in urine cultures][Comment]

Are there situations or clinical findings in the patient where you deviate from your prophylaxis standard (medication, dose, duration, age of the patient)? (multiple answers possible, please specify in answer box) [in newborns]
No

Are there situations or clinical findings in the patient where you deviate from your prophylaxis standard (medication, dose, duration, age of the patient)? (multiple answers possible, please specify in answer box) [in newborns][Comment]

Are there situations or clinical findings in the patient where you deviate from your prophylaxis standard (medication, dose, duration, age of the patient)? (multiple answers possible, please specify in answer box) [Other]
wenn Katheter liegt oder Infektionsbehandlung läuft

Are there situations or clinical findings in the patient where you deviate from your prophylaxis standard (medication, dose, duration, age of the patient)? (multiple answers possible, please specify in answer box) [Other comment]

Are there situations in which you use intravenous prophylaxis?
No

Voiding analysis

Are there differences in antibiotic prophylaxis between VUS and VCUG in your department?
No

Is a non-pathological urine strip test required in preparation for VUS/VCUG in your department?
No

Is a non-pathological urine culture required in preparation for VUS/VCUG in your department?
No

Do patients only receive post-procedure prophylaxis if vesicoureteral reflux (VUR) is detected in VUS/VCUG?
No

Do other MUS/MCU findings (e.g. urethral valves) have an influence on post-procedure prophylaxis?
No

End of survey

Thank you for taking the time to answer this survey!

May we contact you personally if we have further questions?
Yes

E-mail address:
hans-joachim.mentzel@med.uni-jena.de

Survey response 9

Response ID
10

Last page
0

Start language
de

Seed
669122082

Date started
2024-11-13 16:51:51

Date last action
2024-11-13 16:51:51

Standard Operation Procedure

Does your department have a defined protocol for infection prophylaxis in the context of VUS/VCUG?
N/A

Has this protocol been transformed into a written standard operating procedure (SOP)?
N/A

Antibiotic Prophylaxis

Are there situations/constellations in which you do not use antibiotic prophylaxis for VUS/VCUG?
N/A

Please specify these situations/constellations: [girl with first uncomplicated UTI without urinary tract dilation in ultrasound]
N/A

Please specify these situations/constellations: [boy with first uncomplicated UTI without urinary tract dilation in ultrasound]
N/A

Please specify these situations/constellations: [in patients with low grade urinary tract dilation (up to 2nd grade) and no UTI in medical history]
N/A

If you use antibiotic prophylaxis: which mediaction do you use? (multiple answers and subdivision into age groups possible) [Trimethoprim]
N/A

If you use antibiotic prophylaxis: which mediaction do you use? (multiple answers and subdivision into age groups possible) [Nitrofurantoin]
N/A

If you use antibiotic prophylaxis: which mediaction do you use? (multiple answers and subdivision into age groups possible) [Amoxicillin]
N/A

If you use antibiotic prophylaxis: which mediaction do you use? (multiple answers and subdivision into age groups possible) [Cefaclor]
N/A

If you use antibiotic prophylaxis: which mediaction do you use? (multiple answers and subdivision into age groups possible) [Amoxicillin/clavulanic acid]
N/A

If you use antibiotic prophylaxis: which mediaction do you use? (multiple answers and subdivision into age groups possible) [Other]
N/A

Trimethoprim: which time schedule do you use? [Day(s) before VUS/VCUG, how many?]
N/A

Trimethoprim: which time schedule do you use? [Day of VUS/VCUG]
N/A

Trimethoprim: which time schedule do you use? [Day(s) after VUS/VCUG, how many?]
N/A

Nitrofurantoin: which time schedule do you use? [Day(s) before VUS/VCUG, how many?]
N/A

Nitrofurantoin: which time schedule do you use? [Day of VUS/VCUG]
N/A

Nitrofurantoin: which time schedule do you use? [Day(s) after VUS/VCUG, how many?]
N/A

Amoxicillin: which time schedule do you use? [Day(s) before VUS/VCUG, how many?]
N/A

Amoxicillin: which time schedule do you use? [Day of VUS/VCUG]
N/A

Amoxicillin: which time schedule do you use? [Day(s) after VUS/VCUG, how many?]
N/A

Cefaclor: which time schedule do you use? [Days(s) before VUS/VCUG, how many?]
N/A

Cefaclor: which time schedule do you use? [Day of VUS/VCUG]
N/A

Cefaclor: which time schedule do you use? [Day(s) after VUS/VCUG, how many?]
N/A

Amoxicillin/clavulanic acid: in what dosage? (Amoxicillin portion) [prophylaktic (10-20 mg/Kg bodyweight/d)]
N/A

Amoxicillin/clavulanic acid: in what dosage? (Amoxicillin portion) [double prophylactic (20-40 mg/Kg bodyweight/d)]
N/A

Amoxicillin/clavulanic acid: in what dosage? (Amoxicillin portion) [therapeutic (80-100 mg/Kg bodyweight/d)]
N/A

Amoxicillin/clavulanic acid: which time schedule do you use? [Day(s) before VUS/VCUG]
N/A

Amoxicillin/clavulanic acid: which time schedule do you use? [Day of VUS/VCUG]
N/A

Amoxicillin/clavulanic acid: which time schedule do you use? [Day(s) after VUS/VCUG]
N/A

Other: which time schedule do you use? [Day(s) before VUS/VCUG, how many?]
N/A

Other: which time schedule do you use? [Day of VUS/VCUG]
N/A

Other: which time schedule do you use? [Day(s) after VUS/VCUG, how many?]
N/A

Indication for prophylaxis

Which patients receive periinterventional antibiotic prophylaxis? (multiple answers possible) [all]
N/A

Which patients receive periinterventional antibiotic prophylaxis? (multiple answers possible) [patients post urinary tract infection/pyelonephritis/urosepsis]
N/A

Which patients receive periinterventional antibiotic prophylaxis? (multiple answers possible) [patients with urinary tract dilation in ultrasound]
N/A

Which patients receive periinterventional antibiotic prophylaxis? (multiple answers possible) [patients with prenatal urinary tract dilation]
N/A

Which patients receive periinterventional antibiotic prophylaxis? (multiple answers possible) [patients post VUR surgery]
N/A

Which patients receive periinterventional antibiotic prophylaxis? (multiple answers possible) [none]
N/A

Are there situations or clinical findings in the patient where you deviate from your prophylaxis standard (medication, dose, duration, age of the patient)? (multiple answers possible, please specify in answer box) [in case of already known vesicoureteral reflux (VUR)]
N/A

Are there situations or clinical findings in the patient where you deviate from your prophylaxis standard (medication, dose, duration, age of the patient)? (multiple answers possible, please specify in answer box) [in patients with recurrent urinary tract infections (UTI)]
N/A

Are there situations or clinical findings in the patient where you deviate from your prophylaxis standard (medication, dose, duration, age of the patient)? (multiple answers possible, please specify in answer box) [if multidrug-resistant organisms (MDRO) are detected in urine cultures]
N/A

Are there situations or clinical findings in the patient where you deviate from your prophylaxis standard (medication, dose, duration, age of the patient)? (multiple answers possible, please specify in answer box) [in newborns]
N/A

Are there situations in which you use intravenous prophylaxis?
N/A

Voiding analysis

Are there differences in antibiotic prophylaxis between VUS and VCUG in your department?
N/A

Is a non-pathological urine strip test required in preparation for VUS/VCUG in your department?
N/A

Is a non-pathological urine culture required in preparation for VUS/VCUG in your department?
N/A

Do patients only receive post-procedure prophylaxis if vesicoureteral reflux (VUR) is detected in VUS/VCUG?
N/A

Do other MUS/MCU findings (e.g. urethral valves) have an influence on post-procedure prophylaxis?
N/A

End of survey

May we contact you personally if we have further questions?
N/A

Survey response 10

Response ID
11

Last page
0

Start language
de

Seed
1689485199

Date started
2024-11-13 17:49:56

Date last action
2024-11-13 17:50:01

Standard Operation Procedure

Who fills out this survey?

Who fills out this survey? [Other]

In which type of hospital are you employed?

In which type of hospital are you employed? [Other]

In which country do you work?

Does your department have a defined protocol for infection prophylaxis in the context of VUS/VCUG?
N/A

Has this protocol been transformed into a written standard operating procedure (SOP)?
N/A

Antibiotic Prophylaxis

Are there situations/constellations in which you do not use antibiotic prophylaxis for VUS/VCUG?
N/A

Please specify these situations/constellations: [girl with first uncomplicated UTI without urinary tract dilation in ultrasound]
N/A

Please specify these situations/constellations: [boy with first uncomplicated UTI without urinary tract dilation in ultrasound]
N/A

Please specify these situations/constellations: [in patients with low grade urinary tract dilation (up to 2nd grade) and no UTI in medical history]
N/A

If you use antibiotic prophylaxis: which mediaction do you use? (multiple answers and subdivision into age groups possible) [Trimethoprim]
N/A

If you use antibiotic prophylaxis: which mediaction do you use? (multiple answers and subdivision into age groups possible) [Nitrofurantoin]
N/A

If you use antibiotic prophylaxis: which mediaction do you use? (multiple answers and subdivision into age groups possible) [Amoxicillin]
N/A

If you use antibiotic prophylaxis: which mediaction do you use? (multiple answers and subdivision into age groups possible) [Cefaclor]
N/A

If you use antibiotic prophylaxis: which mediaction do you use? (multiple answers and subdivision into age groups possible) [Amoxicillin/clavulanic acid]
N/A

If you use antibiotic prophylaxis: which mediaction do you use? (multiple answers and subdivision into age groups possible) [Other]
N/A

Trimethoprim: which time schedule do you use? [Day(s) before VUS/VCUG, how many?]
N/A

Trimethoprim: which time schedule do you use? [Day of VUS/VCUG]
N/A

Trimethoprim: which time schedule do you use? [Day(s) after VUS/VCUG, how many?]
N/A

Nitrofurantoin: which time schedule do you use? [Day(s) before VUS/VCUG, how many?]
N/A

Nitrofurantoin: which time schedule do you use? [Day of VUS/VCUG]
N/A

Nitrofurantoin: which time schedule do you use? [Day(s) after VUS/VCUG, how many?]
N/A

Amoxicillin: which time schedule do you use? [Day(s) before VUS/VCUG, how many?]
N/A

Amoxicillin: which time schedule do you use? [Day of VUS/VCUG]
N/A

Amoxicillin: which time schedule do you use? [Day(s) after VUS/VCUG, how many?]
N/A

Cefaclor: which time schedule do you use? [Days(s) before VUS/VCUG, how many?]
N/A

Cefaclor: which time schedule do you use? [Day of VUS/VCUG]
N/A

Cefaclor: which time schedule do you use? [Day(s) after VUS/VCUG, how many?]
N/A

Amoxicillin/clavulanic acid: in what dosage? (Amoxicillin portion) [prophylaktic (10-20 mg/Kg bodyweight/d)]
N/A

Amoxicillin/clavulanic acid: in what dosage? (Amoxicillin portion) [double prophylactic (20-40 mg/Kg bodyweight/d)]
N/A

Amoxicillin/clavulanic acid: in what dosage? (Amoxicillin portion) [therapeutic (80-100 mg/Kg bodyweight/d)]
N/A

Amoxicillin/clavulanic acid: which time schedule do you use? [Day(s) before VUS/VCUG]
N/A

Amoxicillin/clavulanic acid: which time schedule do you use? [Day of VUS/VCUG]
N/A

Amoxicillin/clavulanic acid: which time schedule do you use? [Day(s) after VUS/VCUG]
N/A

Other: which time schedule do you use? [Day(s) before VUS/VCUG, how many?]
N/A

Other: which time schedule do you use? [Day of VUS/VCUG]
N/A

Other: which time schedule do you use? [Day(s) after VUS/VCUG, how many?]
N/A

Indication for prophylaxis

Which patients receive periinterventional antibiotic prophylaxis? (multiple answers possible) [all]
N/A

Which patients receive periinterventional antibiotic prophylaxis? (multiple answers possible) [patients post urinary tract infection/pyelonephritis/urosepsis]
N/A

Which patients receive periinterventional antibiotic prophylaxis? (multiple answers possible) [patients with urinary tract dilation in ultrasound]
N/A

Which patients receive periinterventional antibiotic prophylaxis? (multiple answers possible) [patients with prenatal urinary tract dilation]
N/A

Which patients receive periinterventional antibiotic prophylaxis? (multiple answers possible) [patients post VUR surgery]
N/A

Which patients receive periinterventional antibiotic prophylaxis? (multiple answers possible) [none]
N/A

Are there situations or clinical findings in the patient where you deviate from your prophylaxis standard (medication, dose, duration, age of the patient)? (multiple answers possible, please specify in answer box) [in case of already known vesicoureteral reflux (VUR)]
N/A

Are there situations or clinical findings in the patient where you deviate from your prophylaxis standard (medication, dose, duration, age of the patient)? (multiple answers possible, please specify in answer box) [in patients with recurrent urinary tract infections (UTI)]
N/A

Are there situations or clinical findings in the patient where you deviate from your prophylaxis standard (medication, dose, duration, age of the patient)? (multiple answers possible, please specify in answer box) [if multidrug-resistant organisms (MDRO) are detected in urine cultures]
N/A

Are there situations or clinical findings in the patient where you deviate from your prophylaxis standard (medication, dose, duration, age of the patient)? (multiple answers possible, please specify in answer box) [in newborns]
N/A

Are there situations in which you use intravenous prophylaxis?
N/A

Voiding analysis

Are there differences in antibiotic prophylaxis between VUS and VCUG in your department?
N/A

Is a non-pathological urine strip test required in preparation for VUS/VCUG in your department?
N/A

Is a non-pathological urine culture required in preparation for VUS/VCUG in your department?
N/A

Do patients only receive post-procedure prophylaxis if vesicoureteral reflux (VUR) is detected in VUS/VCUG?
N/A

Do other MUS/MCU findings (e.g. urethral valves) have an influence on post-procedure prophylaxis?
N/A

End of survey

May we contact you personally if we have further questions?
N/A

Survey response 11

Response ID
12

Last page
0

Start language
de

Seed
1282987497

Date started
2024-11-14 06:57:41

Date last action
2024-11-14 06:57:41

Standard Operation Procedure

Does your department have a defined protocol for infection prophylaxis in the context of VUS/VCUG?
N/A

Has this protocol been transformed into a written standard operating procedure (SOP)?
N/A

Antibiotic Prophylaxis

Are there situations/constellations in which you do not use antibiotic prophylaxis for VUS/VCUG?
N/A

Please specify these situations/constellations: [girl with first uncomplicated UTI without urinary tract dilation in ultrasound]
N/A

Please specify these situations/constellations: [boy with first uncomplicated UTI without urinary tract dilation in ultrasound]
N/A

Please specify these situations/constellations: [in patients with low grade urinary tract dilation (up to 2nd grade) and no UTI in medical history]
N/A

If you use antibiotic prophylaxis: which mediaction do you use? (multiple answers and subdivision into age groups possible) [Trimethoprim]
N/A

If you use antibiotic prophylaxis: which mediaction do you use? (multiple answers and subdivision into age groups possible) [Nitrofurantoin]
N/A

If you use antibiotic prophylaxis: which mediaction do you use? (multiple answers and subdivision into age groups possible) [Amoxicillin]
N/A

If you use antibiotic prophylaxis: which mediaction do you use? (multiple answers and subdivision into age groups possible) [Cefaclor]
N/A

If you use antibiotic prophylaxis: which mediaction do you use? (multiple answers and subdivision into age groups possible) [Amoxicillin/clavulanic acid]
N/A

If you use antibiotic prophylaxis: which mediaction do you use? (multiple answers and subdivision into age groups possible) [Other]
N/A

Trimethoprim: which time schedule do you use? [Day(s) before VUS/VCUG, how many?]
N/A

Trimethoprim: which time schedule do you use? [Day of VUS/VCUG]
N/A

Trimethoprim: which time schedule do you use? [Day(s) after VUS/VCUG, how many?]
N/A

Nitrofurantoin: which time schedule do you use? [Day(s) before VUS/VCUG, how many?]
N/A

Nitrofurantoin: which time schedule do you use? [Day of VUS/VCUG]
N/A

Nitrofurantoin: which time schedule do you use? [Day(s) after VUS/VCUG, how many?]
N/A

Amoxicillin: which time schedule do you use? [Day(s) before VUS/VCUG, how many?]
N/A

Amoxicillin: which time schedule do you use? [Day of VUS/VCUG]
N/A

Amoxicillin: which time schedule do you use? [Day(s) after VUS/VCUG, how many?]
N/A

Cefaclor: which time schedule do you use? [Days(s) before VUS/VCUG, how many?]
N/A

Cefaclor: which time schedule do you use? [Day of VUS/VCUG]
N/A

Cefaclor: which time schedule do you use? [Day(s) after VUS/VCUG, how many?]
N/A

Amoxicillin/clavulanic acid: in what dosage? (Amoxicillin portion) [prophylaktic (10-20 mg/Kg bodyweight/d)]
N/A

Amoxicillin/clavulanic acid: in what dosage? (Amoxicillin portion) [double prophylactic (20-40 mg/Kg bodyweight/d)]
N/A

Amoxicillin/clavulanic acid: in what dosage? (Amoxicillin portion) [therapeutic (80-100 mg/Kg bodyweight/d)]
N/A

Amoxicillin/clavulanic acid: which time schedule do you use? [Day(s) before VUS/VCUG]
N/A

Amoxicillin/clavulanic acid: which time schedule do you use? [Day of VUS/VCUG]
N/A

Amoxicillin/clavulanic acid: which time schedule do you use? [Day(s) after VUS/VCUG]
N/A

Other: which time schedule do you use? [Day(s) before VUS/VCUG, how many?]
N/A

Other: which time schedule do you use? [Day of VUS/VCUG]
N/A

Other: which time schedule do you use? [Day(s) after VUS/VCUG, how many?]
N/A

Indication for prophylaxis

Which patients receive periinterventional antibiotic prophylaxis? (multiple answers possible) [all]
N/A

Which patients receive periinterventional antibiotic prophylaxis? (multiple answers possible) [patients post urinary tract infection/pyelonephritis/urosepsis]
N/A

Which patients receive periinterventional antibiotic prophylaxis? (multiple answers possible) [patients with urinary tract dilation in ultrasound]
N/A

Which patients receive periinterventional antibiotic prophylaxis? (multiple answers possible) [patients with prenatal urinary tract dilation]
N/A

Which patients receive periinterventional antibiotic prophylaxis? (multiple answers possible) [patients post VUR surgery]
N/A

Which patients receive periinterventional antibiotic prophylaxis? (multiple answers possible) [none]
N/A

Are there situations or clinical findings in the patient where you deviate from your prophylaxis standard (medication, dose, duration, age of the patient)? (multiple answers possible, please specify in answer box) [in case of already known vesicoureteral reflux (VUR)]
N/A

Are there situations or clinical findings in the patient where you deviate from your prophylaxis standard (medication, dose, duration, age of the patient)? (multiple answers possible, please specify in answer box) [in patients with recurrent urinary tract infections (UTI)]
N/A

Are there situations or clinical findings in the patient where you deviate from your prophylaxis standard (medication, dose, duration, age of the patient)? (multiple answers possible, please specify in answer box) [if multidrug-resistant organisms (MDRO) are detected in urine cultures]
N/A

Are there situations or clinical findings in the patient where you deviate from your prophylaxis standard (medication, dose, duration, age of the patient)? (multiple answers possible, please specify in answer box) [in newborns]
N/A

Are there situations in which you use intravenous prophylaxis?
N/A

Voiding analysis

Are there differences in antibiotic prophylaxis between VUS and VCUG in your department?
N/A

Is a non-pathological urine strip test required in preparation for VUS/VCUG in your department?
N/A

Is a non-pathological urine culture required in preparation for VUS/VCUG in your department?
N/A

Do patients only receive post-procedure prophylaxis if vesicoureteral reflux (VUR) is detected in VUS/VCUG?
N/A

Do other MUS/MCU findings (e.g. urethral valves) have an influence on post-procedure prophylaxis?
N/A

End of survey

May we contact you personally if we have further questions?
N/A

Survey response 12

Response ID
13

Last page
0

Start language
de

Seed
347511898

Date started
2024-11-14 07:44:43

Date last action
2024-11-14 07:44:43

Standard Operation Procedure

Does your department have a defined protocol for infection prophylaxis in the context of VUS/VCUG?
N/A

Has this protocol been transformed into a written standard operating procedure (SOP)?
N/A

Antibiotic Prophylaxis

Are there situations/constellations in which you do not use antibiotic prophylaxis for VUS/VCUG?
N/A

Please specify these situations/constellations: [girl with first uncomplicated UTI without urinary tract dilation in ultrasound]
N/A

Please specify these situations/constellations: [boy with first uncomplicated UTI without urinary tract dilation in ultrasound]
N/A

Please specify these situations/constellations: [in patients with low grade urinary tract dilation (up to 2nd grade) and no UTI in medical history]
N/A

If you use antibiotic prophylaxis: which mediaction do you use? (multiple answers and subdivision into age groups possible) [Trimethoprim]
N/A

If you use antibiotic prophylaxis: which mediaction do you use? (multiple answers and subdivision into age groups possible) [Nitrofurantoin]
N/A

If you use antibiotic prophylaxis: which mediaction do you use? (multiple answers and subdivision into age groups possible) [Amoxicillin]
N/A

If you use antibiotic prophylaxis: which mediaction do you use? (multiple answers and subdivision into age groups possible) [Cefaclor]
N/A

If you use antibiotic prophylaxis: which mediaction do you use? (multiple answers and subdivision into age groups possible) [Amoxicillin/clavulanic acid]
N/A

If you use antibiotic prophylaxis: which mediaction do you use? (multiple answers and subdivision into age groups possible) [Other]
N/A

Trimethoprim: which time schedule do you use? [Day(s) before VUS/VCUG, how many?]
N/A

Trimethoprim: which time schedule do you use? [Day of VUS/VCUG]
N/A

Trimethoprim: which time schedule do you use? [Day(s) after VUS/VCUG, how many?]
N/A

Nitrofurantoin: which time schedule do you use? [Day(s) before VUS/VCUG, how many?]
N/A

Nitrofurantoin: which time schedule do you use? [Day of VUS/VCUG]
N/A

Nitrofurantoin: which time schedule do you use? [Day(s) after VUS/VCUG, how many?]
N/A

Amoxicillin: which time schedule do you use? [Day(s) before VUS/VCUG, how many?]
N/A

Amoxicillin: which time schedule do you use? [Day of VUS/VCUG]
N/A

Amoxicillin: which time schedule do you use? [Day(s) after VUS/VCUG, how many?]
N/A

Cefaclor: which time schedule do you use? [Days(s) before VUS/VCUG, how many?]
N/A

Cefaclor: which time schedule do you use? [Day of VUS/VCUG]
N/A

Cefaclor: which time schedule do you use? [Day(s) after VUS/VCUG, how many?]
N/A

Amoxicillin/clavulanic acid: in what dosage? (Amoxicillin portion) [prophylaktic (10-20 mg/Kg bodyweight/d)]
N/A

Amoxicillin/clavulanic acid: in what dosage? (Amoxicillin portion) [double prophylactic (20-40 mg/Kg bodyweight/d)]
N/A

Amoxicillin/clavulanic acid: in what dosage? (Amoxicillin portion) [therapeutic (80-100 mg/Kg bodyweight/d)]
N/A

Amoxicillin/clavulanic acid: which time schedule do you use? [Day(s) before VUS/VCUG]
N/A

Amoxicillin/clavulanic acid: which time schedule do you use? [Day of VUS/VCUG]
N/A

Amoxicillin/clavulanic acid: which time schedule do you use? [Day(s) after VUS/VCUG]
N/A

Other: which time schedule do you use? [Day(s) before VUS/VCUG, how many?]
N/A

Other: which time schedule do you use? [Day of VUS/VCUG]
N/A

Other: which time schedule do you use? [Day(s) after VUS/VCUG, how many?]
N/A

Indication for prophylaxis

Which patients receive periinterventional antibiotic prophylaxis? (multiple answers possible) [all]
N/A

Which patients receive periinterventional antibiotic prophylaxis? (multiple answers possible) [patients post urinary tract infection/pyelonephritis/urosepsis]
N/A

Which patients receive periinterventional antibiotic prophylaxis? (multiple answers possible) [patients with urinary tract dilation in ultrasound]
N/A

Which patients receive periinterventional antibiotic prophylaxis? (multiple answers possible) [patients with prenatal urinary tract dilation]
N/A

Which patients receive periinterventional antibiotic prophylaxis? (multiple answers possible) [patients post VUR surgery]
N/A

Which patients receive periinterventional antibiotic prophylaxis? (multiple answers possible) [none]
N/A

Are there situations or clinical findings in the patient where you deviate from your prophylaxis standard (medication, dose, duration, age of the patient)? (multiple answers possible, please specify in answer box) [in case of already known vesicoureteral reflux (VUR)]
N/A

Are there situations or clinical findings in the patient where you deviate from your prophylaxis standard (medication, dose, duration, age of the patient)? (multiple answers possible, please specify in answer box) [in patients with recurrent urinary tract infections (UTI)]
N/A

Are there situations or clinical findings in the patient where you deviate from your prophylaxis standard (medication, dose, duration, age of the patient)? (multiple answers possible, please specify in answer box) [if multidrug-resistant organisms (MDRO) are detected in urine cultures]
N/A

Are there situations or clinical findings in the patient where you deviate from your prophylaxis standard (medication, dose, duration, age of the patient)? (multiple answers possible, please specify in answer box) [in newborns]
N/A

Are there situations in which you use intravenous prophylaxis?
N/A

Voiding analysis

Are there differences in antibiotic prophylaxis between VUS and VCUG in your department?
N/A

Is a non-pathological urine strip test required in preparation for VUS/VCUG in your department?
N/A

Is a non-pathological urine culture required in preparation for VUS/VCUG in your department?
N/A

Do patients only receive post-procedure prophylaxis if vesicoureteral reflux (VUR) is detected in VUS/VCUG?
N/A

Do other MUS/MCU findings (e.g. urethral valves) have an influence on post-procedure prophylaxis?
N/A

End of survey

May we contact you personally if we have further questions?
N/A

Survey response 13

Response ID
14

Last page
1

Start language
de

Seed
1573570557

Date started
2024-11-14 08:04:56

Date last action
2024-11-14 08:05:29

Standard Operation Procedure

Who fills out this survey?
Pediatric Radiologist

Who fills out this survey? [Other]

In which type of hospital are you employed?
University Hospital

In which type of hospital are you employed? [Other]

In which country do you work?
Mecklenburg-Vorpommern

Does your department have a defined protocol for infection prophylaxis in the context of VUS/VCUG?
Yes

Has this protocol been transformed into a written standard operating procedure (SOP)?
Yes

Which department is the author of the SOP for infection prophylaxis in VUS/VCUG?
Pediatric nephrology

Which department is the author of the SOP for infection prophylaxis in VUS/VCUG? [Other]

Antibiotic Prophylaxis

Are there situations/constellations in which you do not use antibiotic prophylaxis for VUS/VCUG?
N/A

Please specify these situations/constellations: [girl with first uncomplicated UTI without urinary tract dilation in ultrasound]
N/A

Please specify these situations/constellations: [boy with first uncomplicated UTI without urinary tract dilation in ultrasound]
N/A

Please specify these situations/constellations: [in patients with low grade urinary tract dilation (up to 2nd grade) and no UTI in medical history]
N/A

If you use antibiotic prophylaxis: which mediaction do you use? (multiple answers and subdivision into age groups possible) [Trimethoprim]
N/A

If you use antibiotic prophylaxis: which mediaction do you use? (multiple answers and subdivision into age groups possible) [Nitrofurantoin]
N/A

If you use antibiotic prophylaxis: which mediaction do you use? (multiple answers and subdivision into age groups possible) [Amoxicillin]
N/A

If you use antibiotic prophylaxis: which mediaction do you use? (multiple answers and subdivision into age groups possible) [Cefaclor]
N/A

If you use antibiotic prophylaxis: which mediaction do you use? (multiple answers and subdivision into age groups possible) [Amoxicillin/clavulanic acid]
N/A

If you use antibiotic prophylaxis: which mediaction do you use? (multiple answers and subdivision into age groups possible) [Other]
N/A

Trimethoprim: which time schedule do you use? [Day(s) before VUS/VCUG, how many?]
N/A

Trimethoprim: which time schedule do you use? [Day of VUS/VCUG]
N/A

Trimethoprim: which time schedule do you use? [Day(s) after VUS/VCUG, how many?]
N/A

Nitrofurantoin: which time schedule do you use? [Day(s) before VUS/VCUG, how many?]
N/A

Nitrofurantoin: which time schedule do you use? [Day of VUS/VCUG]
N/A

Nitrofurantoin: which time schedule do you use? [Day(s) after VUS/VCUG, how many?]
N/A

Amoxicillin: which time schedule do you use? [Day(s) before VUS/VCUG, how many?]
N/A

Amoxicillin: which time schedule do you use? [Day of VUS/VCUG]
N/A

Amoxicillin: which time schedule do you use? [Day(s) after VUS/VCUG, how many?]
N/A

Cefaclor: which time schedule do you use? [Days(s) before VUS/VCUG, how many?]
N/A

Cefaclor: which time schedule do you use? [Day of VUS/VCUG]
N/A

Cefaclor: which time schedule do you use? [Day(s) after VUS/VCUG, how many?]
N/A

Amoxicillin/clavulanic acid: in what dosage? (Amoxicillin portion) [prophylaktic (10-20 mg/Kg bodyweight/d)]
N/A

Amoxicillin/clavulanic acid: in what dosage? (Amoxicillin portion) [double prophylactic (20-40 mg/Kg bodyweight/d)]
N/A

Amoxicillin/clavulanic acid: in what dosage? (Amoxicillin portion) [therapeutic (80-100 mg/Kg bodyweight/d)]
N/A

Amoxicillin/clavulanic acid: which time schedule do you use? [Day(s) before VUS/VCUG]
N/A

Amoxicillin/clavulanic acid: which time schedule do you use? [Day of VUS/VCUG]
N/A

Amoxicillin/clavulanic acid: which time schedule do you use? [Day(s) after VUS/VCUG]
N/A

Other: which time schedule do you use? [Day(s) before VUS/VCUG, how many?]
N/A

Other: which time schedule do you use? [Day of VUS/VCUG]
N/A

Other: which time schedule do you use? [Day(s) after VUS/VCUG, how many?]
N/A

Indication for prophylaxis

Which patients receive periinterventional antibiotic prophylaxis? (multiple answers possible) [all]
N/A

Which patients receive periinterventional antibiotic prophylaxis? (multiple answers possible) [patients post urinary tract infection/pyelonephritis/urosepsis]
N/A

Which patients receive periinterventional antibiotic prophylaxis? (multiple answers possible) [patients with urinary tract dilation in ultrasound]
N/A

Which patients receive periinterventional antibiotic prophylaxis? (multiple answers possible) [patients with prenatal urinary tract dilation]
N/A

Which patients receive periinterventional antibiotic prophylaxis? (multiple answers possible) [patients post VUR surgery]
N/A

Which patients receive periinterventional antibiotic prophylaxis? (multiple answers possible) [none]
N/A

Are there situations or clinical findings in the patient where you deviate from your prophylaxis standard (medication, dose, duration, age of the patient)? (multiple answers possible, please specify in answer box) [in case of already known vesicoureteral reflux (VUR)]
N/A

Are there situations or clinical findings in the patient where you deviate from your prophylaxis standard (medication, dose, duration, age of the patient)? (multiple answers possible, please specify in answer box) [in patients with recurrent urinary tract infections (UTI)]
N/A

Are there situations or clinical findings in the patient where you deviate from your prophylaxis standard (medication, dose, duration, age of the patient)? (multiple answers possible, please specify in answer box) [if multidrug-resistant organisms (MDRO) are detected in urine cultures]
N/A

Are there situations or clinical findings in the patient where you deviate from your prophylaxis standard (medication, dose, duration, age of the patient)? (multiple answers possible, please specify in answer box) [in newborns]
N/A

Are there situations in which you use intravenous prophylaxis?
N/A

Voiding analysis

Are there differences in antibiotic prophylaxis between VUS and VCUG in your department?
N/A

Is a non-pathological urine strip test required in preparation for VUS/VCUG in your department?
N/A

Is a non-pathological urine culture required in preparation for VUS/VCUG in your department?
N/A

Do patients only receive post-procedure prophylaxis if vesicoureteral reflux (VUR) is detected in VUS/VCUG?
N/A

Do other MUS/MCU findings (e.g. urethral valves) have an influence on post-procedure prophylaxis?
N/A

End of survey

May we contact you personally if we have further questions?
N/A

Survey response 14

Response ID
15

Last page
1

Start language
de

Seed
1748047093

Date started
2024-11-14 08:13:49

Date last action
2024-11-14 08:14:24

Standard Operation Procedure

Who fills out this survey?
Pediatric Radiologist

Who fills out this survey? [Other]

In which type of hospital are you employed?
University Hospital

In which type of hospital are you employed? [Other]

In which country do you work?
Deutschland

Does your department have a defined protocol for infection prophylaxis in the context of VUS/VCUG?
Yes

Has this protocol been transformed into a written standard operating procedure (SOP)?
Yes

Which department is the author of the SOP for infection prophylaxis in VUS/VCUG?
Other

Which department is the author of the SOP for infection prophylaxis in VUS/VCUG? [Other]
Kinderurologie

Antibiotic Prophylaxis

Are there situations/constellations in which you do not use antibiotic prophylaxis for VUS/VCUG?
N/A

Please specify these situations/constellations: [girl with first uncomplicated UTI without urinary tract dilation in ultrasound]
N/A

Please specify these situations/constellations: [boy with first uncomplicated UTI without urinary tract dilation in ultrasound]
N/A

Please specify these situations/constellations: [in patients with low grade urinary tract dilation (up to 2nd grade) and no UTI in medical history]
N/A

If you use antibiotic prophylaxis: which mediaction do you use? (multiple answers and subdivision into age groups possible) [Trimethoprim]
N/A

If you use antibiotic prophylaxis: which mediaction do you use? (multiple answers and subdivision into age groups possible) [Nitrofurantoin]
N/A

If you use antibiotic prophylaxis: which mediaction do you use? (multiple answers and subdivision into age groups possible) [Amoxicillin]
N/A

If you use antibiotic prophylaxis: which mediaction do you use? (multiple answers and subdivision into age groups possible) [Cefaclor]
N/A

If you use antibiotic prophylaxis: which mediaction do you use? (multiple answers and subdivision into age groups possible) [Amoxicillin/clavulanic acid]
N/A

If you use antibiotic prophylaxis: which mediaction do you use? (multiple answers and subdivision into age groups possible) [Other]
N/A

Trimethoprim: which time schedule do you use? [Day(s) before VUS/VCUG, how many?]
N/A

Trimethoprim: which time schedule do you use? [Day of VUS/VCUG]
N/A

Trimethoprim: which time schedule do you use? [Day(s) after VUS/VCUG, how many?]
N/A

Nitrofurantoin: which time schedule do you use? [Day(s) before VUS/VCUG, how many?]
N/A

Nitrofurantoin: which time schedule do you use? [Day of VUS/VCUG]
N/A

Nitrofurantoin: which time schedule do you use? [Day(s) after VUS/VCUG, how many?]
N/A

Amoxicillin: which time schedule do you use? [Day(s) before VUS/VCUG, how many?]
N/A

Amoxicillin: which time schedule do you use? [Day of VUS/VCUG]
N/A

Amoxicillin: which time schedule do you use? [Day(s) after VUS/VCUG, how many?]
N/A

Cefaclor: which time schedule do you use? [Days(s) before VUS/VCUG, how many?]
N/A

Cefaclor: which time schedule do you use? [Day of VUS/VCUG]
N/A

Cefaclor: which time schedule do you use? [Day(s) after VUS/VCUG, how many?]
N/A

Amoxicillin/clavulanic acid: in what dosage? (Amoxicillin portion) [prophylaktic (10-20 mg/Kg bodyweight/d)]
N/A

Amoxicillin/clavulanic acid: in what dosage? (Amoxicillin portion) [double prophylactic (20-40 mg/Kg bodyweight/d)]
N/A

Amoxicillin/clavulanic acid: in what dosage? (Amoxicillin portion) [therapeutic (80-100 mg/Kg bodyweight/d)]
N/A

Amoxicillin/clavulanic acid: which time schedule do you use? [Day(s) before VUS/VCUG]
N/A

Amoxicillin/clavulanic acid: which time schedule do you use? [Day of VUS/VCUG]
N/A

Amoxicillin/clavulanic acid: which time schedule do you use? [Day(s) after VUS/VCUG]
N/A

Other: which time schedule do you use? [Day(s) before VUS/VCUG, how many?]
N/A

Other: which time schedule do you use? [Day of VUS/VCUG]
N/A

Other: which time schedule do you use? [Day(s) after VUS/VCUG, how many?]
N/A

Indication for prophylaxis

Which patients receive periinterventional antibiotic prophylaxis? (multiple answers possible) [all]
N/A

Which patients receive periinterventional antibiotic prophylaxis? (multiple answers possible) [patients post urinary tract infection/pyelonephritis/urosepsis]
N/A

Which patients receive periinterventional antibiotic prophylaxis? (multiple answers possible) [patients with urinary tract dilation in ultrasound]
N/A

Which patients receive periinterventional antibiotic prophylaxis? (multiple answers possible) [patients with prenatal urinary tract dilation]
N/A

Which patients receive periinterventional antibiotic prophylaxis? (multiple answers possible) [patients post VUR surgery]
N/A

Which patients receive periinterventional antibiotic prophylaxis? (multiple answers possible) [none]
N/A

Are there situations or clinical findings in the patient where you deviate from your prophylaxis standard (medication, dose, duration, age of the patient)? (multiple answers possible, please specify in answer box) [in case of already known vesicoureteral reflux (VUR)]
N/A

Are there situations or clinical findings in the patient where you deviate from your prophylaxis standard (medication, dose, duration, age of the patient)? (multiple answers possible, please specify in answer box) [in patients with recurrent urinary tract infections (UTI)]
N/A

Are there situations or clinical findings in the patient where you deviate from your prophylaxis standard (medication, dose, duration, age of the patient)? (multiple answers possible, please specify in answer box) [if multidrug-resistant organisms (MDRO) are detected in urine cultures]
N/A

Are there situations or clinical findings in the patient where you deviate from your prophylaxis standard (medication, dose, duration, age of the patient)? (multiple answers possible, please specify in answer box) [in newborns]
N/A

Are there situations in which you use intravenous prophylaxis?
N/A

Voiding analysis

Are there differences in antibiotic prophylaxis between VUS and VCUG in your department?
N/A

Is a non-pathological urine strip test required in preparation for VUS/VCUG in your department?
N/A

Is a non-pathological urine culture required in preparation for VUS/VCUG in your department?
N/A

Do patients only receive post-procedure prophylaxis if vesicoureteral reflux (VUR) is detected in VUS/VCUG?
N/A

Do other MUS/MCU findings (e.g. urethral valves) have an influence on post-procedure prophylaxis?
N/A

End of survey

May we contact you personally if we have further questions?
N/A

Survey response 15

Response ID
16

Last page
0

Start language
de

Seed
1682503148

Date started
2024-11-14 08:14:15

Date last action
2024-11-14 08:14:15

Standard Operation Procedure

Does your department have a defined protocol for infection prophylaxis in the context of VUS/VCUG?
N/A

Has this protocol been transformed into a written standard operating procedure (SOP)?
N/A

Antibiotic Prophylaxis

Are there situations/constellations in which you do not use antibiotic prophylaxis for VUS/VCUG?
N/A

Please specify these situations/constellations: [girl with first uncomplicated UTI without urinary tract dilation in ultrasound]
N/A

Please specify these situations/constellations: [boy with first uncomplicated UTI without urinary tract dilation in ultrasound]
N/A

Please specify these situations/constellations: [in patients with low grade urinary tract dilation (up to 2nd grade) and no UTI in medical history]
N/A

If you use antibiotic prophylaxis: which mediaction do you use? (multiple answers and subdivision into age groups possible) [Trimethoprim]
N/A

If you use antibiotic prophylaxis: which mediaction do you use? (multiple answers and subdivision into age groups possible) [Nitrofurantoin]
N/A

If you use antibiotic prophylaxis: which mediaction do you use? (multiple answers and subdivision into age groups possible) [Amoxicillin]
N/A

If you use antibiotic prophylaxis: which mediaction do you use? (multiple answers and subdivision into age groups possible) [Cefaclor]
N/A

If you use antibiotic prophylaxis: which mediaction do you use? (multiple answers and subdivision into age groups possible) [Amoxicillin/clavulanic acid]
N/A

If you use antibiotic prophylaxis: which mediaction do you use? (multiple answers and subdivision into age groups possible) [Other]
N/A

Trimethoprim: which time schedule do you use? [Day(s) before VUS/VCUG, how many?]
N/A

Trimethoprim: which time schedule do you use? [Day of VUS/VCUG]
N/A

Trimethoprim: which time schedule do you use? [Day(s) after VUS/VCUG, how many?]
N/A

Nitrofurantoin: which time schedule do you use? [Day(s) before VUS/VCUG, how many?]
N/A

Nitrofurantoin: which time schedule do you use? [Day of VUS/VCUG]
N/A

Nitrofurantoin: which time schedule do you use? [Day(s) after VUS/VCUG, how many?]
N/A

Amoxicillin: which time schedule do you use? [Day(s) before VUS/VCUG, how many?]
N/A

Amoxicillin: which time schedule do you use? [Day of VUS/VCUG]
N/A

Amoxicillin: which time schedule do you use? [Day(s) after VUS/VCUG, how many?]
N/A

Cefaclor: which time schedule do you use? [Days(s) before VUS/VCUG, how many?]
N/A

Cefaclor: which time schedule do you use? [Day of VUS/VCUG]
N/A

Cefaclor: which time schedule do you use? [Day(s) after VUS/VCUG, how many?]
N/A

Amoxicillin/clavulanic acid: in what dosage? (Amoxicillin portion) [prophylaktic (10-20 mg/Kg bodyweight/d)]
N/A

Amoxicillin/clavulanic acid: in what dosage? (Amoxicillin portion) [double prophylactic (20-40 mg/Kg bodyweight/d)]
N/A

Amoxicillin/clavulanic acid: in what dosage? (Amoxicillin portion) [therapeutic (80-100 mg/Kg bodyweight/d)]
N/A

Amoxicillin/clavulanic acid: which time schedule do you use? [Day(s) before VUS/VCUG]
N/A

Amoxicillin/clavulanic acid: which time schedule do you use? [Day of VUS/VCUG]
N/A

Amoxicillin/clavulanic acid: which time schedule do you use? [Day(s) after VUS/VCUG]
N/A

Other: which time schedule do you use? [Day(s) before VUS/VCUG, how many?]
N/A

Other: which time schedule do you use? [Day of VUS/VCUG]
N/A

Other: which time schedule do you use? [Day(s) after VUS/VCUG, how many?]
N/A

Indication for prophylaxis

Which patients receive periinterventional antibiotic prophylaxis? (multiple answers possible) [all]
N/A

Which patients receive periinterventional antibiotic prophylaxis? (multiple answers possible) [patients post urinary tract infection/pyelonephritis/urosepsis]
N/A

Which patients receive periinterventional antibiotic prophylaxis? (multiple answers possible) [patients with urinary tract dilation in ultrasound]
N/A

Which patients receive periinterventional antibiotic prophylaxis? (multiple answers possible) [patients with prenatal urinary tract dilation]
N/A

Which patients receive periinterventional antibiotic prophylaxis? (multiple answers possible) [patients post VUR surgery]
N/A

Which patients receive periinterventional antibiotic prophylaxis? (multiple answers possible) [none]
N/A

Are there situations or clinical findings in the patient where you deviate from your prophylaxis standard (medication, dose, duration, age of the patient)? (multiple answers possible, please specify in answer box) [in case of already known vesicoureteral reflux (VUR)]
N/A

Are there situations or clinical findings in the patient where you deviate from your prophylaxis standard (medication, dose, duration, age of the patient)? (multiple answers possible, please specify in answer box) [in patients with recurrent urinary tract infections (UTI)]
N/A

Are there situations or clinical findings in the patient where you deviate from your prophylaxis standard (medication, dose, duration, age of the patient)? (multiple answers possible, please specify in answer box) [if multidrug-resistant organisms (MDRO) are detected in urine cultures]
N/A

Are there situations or clinical findings in the patient where you deviate from your prophylaxis standard (medication, dose, duration, age of the patient)? (multiple answers possible, please specify in answer box) [in newborns]
N/A

Are there situations in which you use intravenous prophylaxis?
N/A

Voiding analysis

Are there differences in antibiotic prophylaxis between VUS and VCUG in your department?
N/A

Is a non-pathological urine strip test required in preparation for VUS/VCUG in your department?
N/A

Is a non-pathological urine culture required in preparation for VUS/VCUG in your department?
N/A

Do patients only receive post-procedure prophylaxis if vesicoureteral reflux (VUR) is detected in VUS/VCUG?
N/A

Do other MUS/MCU findings (e.g. urethral valves) have an influence on post-procedure prophylaxis?
N/A

End of survey

May we contact you personally if we have further questions?
N/A

Survey response 16

Response ID
17

Last page
1

Start language
de

Seed
376159638

Date started
2024-11-14 08:46:44

Date last action
2024-11-14 08:47:45

Standard Operation Procedure

Who fills out this survey?
Pediatric Radiologist

Who fills out this survey? [Other]

In which type of hospital are you employed?
Community hospital (>50 beds for pediatric patients)

In which type of hospital are you employed? [Other]

In which country do you work?
d

Does your department have a defined protocol for infection prophylaxis in the context of VUS/VCUG?
No

Has this protocol been transformed into a written standard operating procedure (SOP)?
N/A

Antibiotic Prophylaxis

Are there situations/constellations in which you do not use antibiotic prophylaxis for VUS/VCUG?
N/A

Please specify these situations/constellations: [girl with first uncomplicated UTI without urinary tract dilation in ultrasound]
N/A

Please specify these situations/constellations: [boy with first uncomplicated UTI without urinary tract dilation in ultrasound]
N/A

Please specify these situations/constellations: [in patients with low grade urinary tract dilation (up to 2nd grade) and no UTI in medical history]
N/A

If you use antibiotic prophylaxis: which mediaction do you use? (multiple answers and subdivision into age groups possible) [Trimethoprim]
N/A

If you use antibiotic prophylaxis: which mediaction do you use? (multiple answers and subdivision into age groups possible) [Nitrofurantoin]
N/A

If you use antibiotic prophylaxis: which mediaction do you use? (multiple answers and subdivision into age groups possible) [Amoxicillin]
N/A

If you use antibiotic prophylaxis: which mediaction do you use? (multiple answers and subdivision into age groups possible) [Cefaclor]
N/A

If you use antibiotic prophylaxis: which mediaction do you use? (multiple answers and subdivision into age groups possible) [Amoxicillin/clavulanic acid]
N/A

If you use antibiotic prophylaxis: which mediaction do you use? (multiple answers and subdivision into age groups possible) [Other]
N/A

Trimethoprim: which time schedule do you use? [Day(s) before VUS/VCUG, how many?]
N/A

Trimethoprim: which time schedule do you use? [Day of VUS/VCUG]
N/A

Trimethoprim: which time schedule do you use? [Day(s) after VUS/VCUG, how many?]
N/A

Nitrofurantoin: which time schedule do you use? [Day(s) before VUS/VCUG, how many?]
N/A

Nitrofurantoin: which time schedule do you use? [Day of VUS/VCUG]
N/A

Nitrofurantoin: which time schedule do you use? [Day(s) after VUS/VCUG, how many?]
N/A

Amoxicillin: which time schedule do you use? [Day(s) before VUS/VCUG, how many?]
N/A

Amoxicillin: which time schedule do you use? [Day of VUS/VCUG]
N/A

Amoxicillin: which time schedule do you use? [Day(s) after VUS/VCUG, how many?]
N/A

Cefaclor: which time schedule do you use? [Days(s) before VUS/VCUG, how many?]
N/A

Cefaclor: which time schedule do you use? [Day of VUS/VCUG]
N/A

Cefaclor: which time schedule do you use? [Day(s) after VUS/VCUG, how many?]
N/A

Amoxicillin/clavulanic acid: in what dosage? (Amoxicillin portion) [prophylaktic (10-20 mg/Kg bodyweight/d)]
N/A

Amoxicillin/clavulanic acid: in what dosage? (Amoxicillin portion) [double prophylactic (20-40 mg/Kg bodyweight/d)]
N/A

Amoxicillin/clavulanic acid: in what dosage? (Amoxicillin portion) [therapeutic (80-100 mg/Kg bodyweight/d)]
N/A

Amoxicillin/clavulanic acid: which time schedule do you use? [Day(s) before VUS/VCUG]
N/A

Amoxicillin/clavulanic acid: which time schedule do you use? [Day of VUS/VCUG]
N/A

Amoxicillin/clavulanic acid: which time schedule do you use? [Day(s) after VUS/VCUG]
N/A

Other: which time schedule do you use? [Day(s) before VUS/VCUG, how many?]
N/A

Other: which time schedule do you use? [Day of VUS/VCUG]
N/A

Other: which time schedule do you use? [Day(s) after VUS/VCUG, how many?]
N/A

Indication for prophylaxis

Which patients receive periinterventional antibiotic prophylaxis? (multiple answers possible) [all]
N/A

Which patients receive periinterventional antibiotic prophylaxis? (multiple answers possible) [patients post urinary tract infection/pyelonephritis/urosepsis]
N/A

Which patients receive periinterventional antibiotic prophylaxis? (multiple answers possible) [patients with urinary tract dilation in ultrasound]
N/A

Which patients receive periinterventional antibiotic prophylaxis? (multiple answers possible) [patients with prenatal urinary tract dilation]
N/A

Which patients receive periinterventional antibiotic prophylaxis? (multiple answers possible) [patients post VUR surgery]
N/A

Which patients receive periinterventional antibiotic prophylaxis? (multiple answers possible) [none]
N/A

Are there situations or clinical findings in the patient where you deviate from your prophylaxis standard (medication, dose, duration, age of the patient)? (multiple answers possible, please specify in answer box) [in case of already known vesicoureteral reflux (VUR)]
N/A

Are there situations or clinical findings in the patient where you deviate from your prophylaxis standard (medication, dose, duration, age of the patient)? (multiple answers possible, please specify in answer box) [in patients with recurrent urinary tract infections (UTI)]
N/A

Are there situations or clinical findings in the patient where you deviate from your prophylaxis standard (medication, dose, duration, age of the patient)? (multiple answers possible, please specify in answer box) [if multidrug-resistant organisms (MDRO) are detected in urine cultures]
N/A

Are there situations or clinical findings in the patient where you deviate from your prophylaxis standard (medication, dose, duration, age of the patient)? (multiple answers possible, please specify in answer box) [in newborns]
N/A

Are there situations in which you use intravenous prophylaxis?
N/A

Voiding analysis

Are there differences in antibiotic prophylaxis between VUS and VCUG in your department?
N/A

Is a non-pathological urine strip test required in preparation for VUS/VCUG in your department?
N/A

Is a non-pathological urine culture required in preparation for VUS/VCUG in your department?
N/A

Do patients only receive post-procedure prophylaxis if vesicoureteral reflux (VUR) is detected in VUS/VCUG?
N/A

Do other MUS/MCU findings (e.g. urethral valves) have an influence on post-procedure prophylaxis?
N/A

End of survey

May we contact you personally if we have further questions?
N/A

Survey response 17

Response ID
18

Last page
1

Start language
de

Seed
1199321310

Date started
2024-11-14 09:07:44

Date last action
2024-11-14 09:08:16

Standard Operation Procedure

Who fills out this survey?
Pediatric Radiologist

Who fills out this survey? [Other]

In which type of hospital are you employed?
University Hospital

In which type of hospital are you employed? [Other]

In which country do you work?
Schweiz

Does your department have a defined protocol for infection prophylaxis in the context of VUS/VCUG?
Yes

Has this protocol been transformed into a written standard operating procedure (SOP)?
Yes

Which department is the author of the SOP for infection prophylaxis in VUS/VCUG?
Pediatric nephrology

Which department is the author of the SOP for infection prophylaxis in VUS/VCUG? [Other]

Antibiotic Prophylaxis

Are there situations/constellations in which you do not use antibiotic prophylaxis for VUS/VCUG?
N/A

Please specify these situations/constellations: [girl with first uncomplicated UTI without urinary tract dilation in ultrasound]
N/A

Please specify these situations/constellations: [boy with first uncomplicated UTI without urinary tract dilation in ultrasound]
N/A

Please specify these situations/constellations: [in patients with low grade urinary tract dilation (up to 2nd grade) and no UTI in medical history]
N/A

If you use antibiotic prophylaxis: which mediaction do you use? (multiple answers and subdivision into age groups possible) [Trimethoprim]
N/A

If you use antibiotic prophylaxis: which mediaction do you use? (multiple answers and subdivision into age groups possible) [Nitrofurantoin]
N/A

If you use antibiotic prophylaxis: which mediaction do you use? (multiple answers and subdivision into age groups possible) [Amoxicillin]
N/A

If you use antibiotic prophylaxis: which mediaction do you use? (multiple answers and subdivision into age groups possible) [Cefaclor]
N/A

If you use antibiotic prophylaxis: which mediaction do you use? (multiple answers and subdivision into age groups possible) [Amoxicillin/clavulanic acid]
N/A

If you use antibiotic prophylaxis: which mediaction do you use? (multiple answers and subdivision into age groups possible) [Other]
N/A

Trimethoprim: which time schedule do you use? [Day(s) before VUS/VCUG, how many?]
N/A

Trimethoprim: which time schedule do you use? [Day of VUS/VCUG]
N/A

Trimethoprim: which time schedule do you use? [Day(s) after VUS/VCUG, how many?]
N/A

Nitrofurantoin: which time schedule do you use? [Day(s) before VUS/VCUG, how many?]
N/A

Nitrofurantoin: which time schedule do you use? [Day of VUS/VCUG]
N/A

Nitrofurantoin: which time schedule do you use? [Day(s) after VUS/VCUG, how many?]
N/A

Amoxicillin: which time schedule do you use? [Day(s) before VUS/VCUG, how many?]
N/A

Amoxicillin: which time schedule do you use? [Day of VUS/VCUG]
N/A

Amoxicillin: which time schedule do you use? [Day(s) after VUS/VCUG, how many?]
N/A

Cefaclor: which time schedule do you use? [Days(s) before VUS/VCUG, how many?]
N/A

Cefaclor: which time schedule do you use? [Day of VUS/VCUG]
N/A

Cefaclor: which time schedule do you use? [Day(s) after VUS/VCUG, how many?]
N/A

Amoxicillin/clavulanic acid: in what dosage? (Amoxicillin portion) [prophylaktic (10-20 mg/Kg bodyweight/d)]
N/A

Amoxicillin/clavulanic acid: in what dosage? (Amoxicillin portion) [double prophylactic (20-40 mg/Kg bodyweight/d)]
N/A

Amoxicillin/clavulanic acid: in what dosage? (Amoxicillin portion) [therapeutic (80-100 mg/Kg bodyweight/d)]
N/A

Amoxicillin/clavulanic acid: which time schedule do you use? [Day(s) before VUS/VCUG]
N/A

Amoxicillin/clavulanic acid: which time schedule do you use? [Day of VUS/VCUG]
N/A

Amoxicillin/clavulanic acid: which time schedule do you use? [Day(s) after VUS/VCUG]
N/A

Other: which time schedule do you use? [Day(s) before VUS/VCUG, how many?]
N/A

Other: which time schedule do you use? [Day of VUS/VCUG]
N/A

Other: which time schedule do you use? [Day(s) after VUS/VCUG, how many?]
N/A

Indication for prophylaxis

Which patients receive periinterventional antibiotic prophylaxis? (multiple answers possible) [all]
N/A

Which patients receive periinterventional antibiotic prophylaxis? (multiple answers possible) [patients post urinary tract infection/pyelonephritis/urosepsis]
N/A

Which patients receive periinterventional antibiotic prophylaxis? (multiple answers possible) [patients with urinary tract dilation in ultrasound]
N/A

Which patients receive periinterventional antibiotic prophylaxis? (multiple answers possible) [patients with prenatal urinary tract dilation]
N/A

Which patients receive periinterventional antibiotic prophylaxis? (multiple answers possible) [patients post VUR surgery]
N/A

Which patients receive periinterventional antibiotic prophylaxis? (multiple answers possible) [none]
N/A

Are there situations or clinical findings in the patient where you deviate from your prophylaxis standard (medication, dose, duration, age of the patient)? (multiple answers possible, please specify in answer box) [in case of already known vesicoureteral reflux (VUR)]
N/A

Are there situations or clinical findings in the patient where you deviate from your prophylaxis standard (medication, dose, duration, age of the patient)? (multiple answers possible, please specify in answer box) [in patients with recurrent urinary tract infections (UTI)]
N/A

Are there situations or clinical findings in the patient where you deviate from your prophylaxis standard (medication, dose, duration, age of the patient)? (multiple answers possible, please specify in answer box) [if multidrug-resistant organisms (MDRO) are detected in urine cultures]
N/A

Are there situations or clinical findings in the patient where you deviate from your prophylaxis standard (medication, dose, duration, age of the patient)? (multiple answers possible, please specify in answer box) [in newborns]
N/A

Are there situations in which you use intravenous prophylaxis?
N/A

Voiding analysis

Are there differences in antibiotic prophylaxis between VUS and VCUG in your department?
N/A

Is a non-pathological urine strip test required in preparation for VUS/VCUG in your department?
N/A

Is a non-pathological urine culture required in preparation for VUS/VCUG in your department?
N/A

Do patients only receive post-procedure prophylaxis if vesicoureteral reflux (VUR) is detected in VUS/VCUG?
N/A

Do other MUS/MCU findings (e.g. urethral valves) have an influence on post-procedure prophylaxis?
N/A

End of survey

May we contact you personally if we have further questions?
N/A

Survey response 18

Response ID
19

Last page
0

Start language
de

Seed
2103521748

Date started
2024-11-14 09:37:11

Date last action
2024-11-14 09:39:45

Standard Operation Procedure

Who fills out this survey?
Pediatric Radiologist

Who fills out this survey? [Other]

In which type of hospital are you employed?
Community hospital (>50 beds for pediatric patients)

In which type of hospital are you employed? [Other]

In which country do you work?
Leiter Kinderradiologie

Does your department have a defined protocol for infection prophylaxis in the context of VUS/VCUG?
N/A

Has this protocol been transformed into a written standard operating procedure (SOP)?
N/A

Antibiotic Prophylaxis

Are there situations/constellations in which you do not use antibiotic prophylaxis for VUS/VCUG?
N/A

Please specify these situations/constellations: [girl with first uncomplicated UTI without urinary tract dilation in ultrasound]
N/A

Please specify these situations/constellations: [boy with first uncomplicated UTI without urinary tract dilation in ultrasound]
N/A

Please specify these situations/constellations: [in patients with low grade urinary tract dilation (up to 2nd grade) and no UTI in medical history]
N/A

If you use antibiotic prophylaxis: which mediaction do you use? (multiple answers and subdivision into age groups possible) [Trimethoprim]
N/A

If you use antibiotic prophylaxis: which mediaction do you use? (multiple answers and subdivision into age groups possible) [Nitrofurantoin]
N/A

If you use antibiotic prophylaxis: which mediaction do you use? (multiple answers and subdivision into age groups possible) [Amoxicillin]
N/A

If you use antibiotic prophylaxis: which mediaction do you use? (multiple answers and subdivision into age groups possible) [Cefaclor]
N/A

If you use antibiotic prophylaxis: which mediaction do you use? (multiple answers and subdivision into age groups possible) [Amoxicillin/clavulanic acid]
N/A

If you use antibiotic prophylaxis: which mediaction do you use? (multiple answers and subdivision into age groups possible) [Other]
N/A

Trimethoprim: which time schedule do you use? [Day(s) before VUS/VCUG, how many?]
N/A

Trimethoprim: which time schedule do you use? [Day of VUS/VCUG]
N/A

Trimethoprim: which time schedule do you use? [Day(s) after VUS/VCUG, how many?]
N/A

Nitrofurantoin: which time schedule do you use? [Day(s) before VUS/VCUG, how many?]
N/A

Nitrofurantoin: which time schedule do you use? [Day of VUS/VCUG]
N/A

Nitrofurantoin: which time schedule do you use? [Day(s) after VUS/VCUG, how many?]
N/A

Amoxicillin: which time schedule do you use? [Day(s) before VUS/VCUG, how many?]
N/A

Amoxicillin: which time schedule do you use? [Day of VUS/VCUG]
N/A

Amoxicillin: which time schedule do you use? [Day(s) after VUS/VCUG, how many?]
N/A

Cefaclor: which time schedule do you use? [Days(s) before VUS/VCUG, how many?]
N/A

Cefaclor: which time schedule do you use? [Day of VUS/VCUG]
N/A

Cefaclor: which time schedule do you use? [Day(s) after VUS/VCUG, how many?]
N/A

Amoxicillin/clavulanic acid: in what dosage? (Amoxicillin portion) [prophylaktic (10-20 mg/Kg bodyweight/d)]
N/A

Amoxicillin/clavulanic acid: in what dosage? (Amoxicillin portion) [double prophylactic (20-40 mg/Kg bodyweight/d)]
N/A

Amoxicillin/clavulanic acid: in what dosage? (Amoxicillin portion) [therapeutic (80-100 mg/Kg bodyweight/d)]
N/A

Amoxicillin/clavulanic acid: which time schedule do you use? [Day(s) before VUS/VCUG]
N/A

Amoxicillin/clavulanic acid: which time schedule do you use? [Day of VUS/VCUG]
N/A

Amoxicillin/clavulanic acid: which time schedule do you use? [Day(s) after VUS/VCUG]
N/A

Other: which time schedule do you use? [Day(s) before VUS/VCUG, how many?]
N/A

Other: which time schedule do you use? [Day of VUS/VCUG]
N/A

Other: which time schedule do you use? [Day(s) after VUS/VCUG, how many?]
N/A

Indication for prophylaxis

Which patients receive periinterventional antibiotic prophylaxis? (multiple answers possible) [all]
N/A

Which patients receive periinterventional antibiotic prophylaxis? (multiple answers possible) [patients post urinary tract infection/pyelonephritis/urosepsis]
N/A

Which patients receive periinterventional antibiotic prophylaxis? (multiple answers possible) [patients with urinary tract dilation in ultrasound]
N/A

Which patients receive periinterventional antibiotic prophylaxis? (multiple answers possible) [patients with prenatal urinary tract dilation]
N/A

Which patients receive periinterventional antibiotic prophylaxis? (multiple answers possible) [patients post VUR surgery]
N/A

Which patients receive periinterventional antibiotic prophylaxis? (multiple answers possible) [none]
N/A

Are there situations or clinical findings in the patient where you deviate from your prophylaxis standard (medication, dose, duration, age of the patient)? (multiple answers possible, please specify in answer box) [in case of already known vesicoureteral reflux (VUR)]
N/A

Are there situations or clinical findings in the patient where you deviate from your prophylaxis standard (medication, dose, duration, age of the patient)? (multiple answers possible, please specify in answer box) [in patients with recurrent urinary tract infections (UTI)]
N/A

Are there situations or clinical findings in the patient where you deviate from your prophylaxis standard (medication, dose, duration, age of the patient)? (multiple answers possible, please specify in answer box) [if multidrug-resistant organisms (MDRO) are detected in urine cultures]
N/A

Are there situations or clinical findings in the patient where you deviate from your prophylaxis standard (medication, dose, duration, age of the patient)? (multiple answers possible, please specify in answer box) [in newborns]
N/A

Are there situations in which you use intravenous prophylaxis?
N/A

Voiding analysis

Are there differences in antibiotic prophylaxis between VUS and VCUG in your department?
N/A

Is a non-pathological urine strip test required in preparation for VUS/VCUG in your department?
N/A

Is a non-pathological urine culture required in preparation for VUS/VCUG in your department?
N/A

Do patients only receive post-procedure prophylaxis if vesicoureteral reflux (VUR) is detected in VUS/VCUG?
N/A

Do other MUS/MCU findings (e.g. urethral valves) have an influence on post-procedure prophylaxis?
N/A

End of survey

May we contact you personally if we have further questions?
N/A

Survey response 19

Response ID
20

Last page
1

Start language
de

Seed
1144592552

Date started
2024-11-14 09:48:58

Date last action
2024-11-14 09:49:59

Standard Operation Procedure

Who fills out this survey?
Pediatric Radiologist

Who fills out this survey? [Other]

In which type of hospital are you employed?
Community hospital (>50 beds for pediatric patients)

In which type of hospital are you employed? [Other]

In which country do you work?
Deutschland

Does your department have a defined protocol for infection prophylaxis in the context of VUS/VCUG?
No

Has this protocol been transformed into a written standard operating procedure (SOP)?
N/A

Antibiotic Prophylaxis

Are there situations/constellations in which you do not use antibiotic prophylaxis for VUS/VCUG?
N/A

Please specify these situations/constellations: [girl with first uncomplicated UTI without urinary tract dilation in ultrasound]
N/A

Please specify these situations/constellations: [boy with first uncomplicated UTI without urinary tract dilation in ultrasound]
N/A

Please specify these situations/constellations: [in patients with low grade urinary tract dilation (up to 2nd grade) and no UTI in medical history]
N/A

If you use antibiotic prophylaxis: which mediaction do you use? (multiple answers and subdivision into age groups possible) [Trimethoprim]
N/A

If you use antibiotic prophylaxis: which mediaction do you use? (multiple answers and subdivision into age groups possible) [Nitrofurantoin]
N/A

If you use antibiotic prophylaxis: which mediaction do you use? (multiple answers and subdivision into age groups possible) [Amoxicillin]
N/A

If you use antibiotic prophylaxis: which mediaction do you use? (multiple answers and subdivision into age groups possible) [Cefaclor]
N/A

If you use antibiotic prophylaxis: which mediaction do you use? (multiple answers and subdivision into age groups possible) [Amoxicillin/clavulanic acid]
N/A

If you use antibiotic prophylaxis: which mediaction do you use? (multiple answers and subdivision into age groups possible) [Other]
N/A

Trimethoprim: which time schedule do you use? [Day(s) before VUS/VCUG, how many?]
N/A

Trimethoprim: which time schedule do you use? [Day of VUS/VCUG]
N/A

Trimethoprim: which time schedule do you use? [Day(s) after VUS/VCUG, how many?]
N/A

Nitrofurantoin: which time schedule do you use? [Day(s) before VUS/VCUG, how many?]
N/A

Nitrofurantoin: which time schedule do you use? [Day of VUS/VCUG]
N/A

Nitrofurantoin: which time schedule do you use? [Day(s) after VUS/VCUG, how many?]
N/A

Amoxicillin: which time schedule do you use? [Day(s) before VUS/VCUG, how many?]
N/A

Amoxicillin: which time schedule do you use? [Day of VUS/VCUG]
N/A

Amoxicillin: which time schedule do you use? [Day(s) after VUS/VCUG, how many?]
N/A

Cefaclor: which time schedule do you use? [Days(s) before VUS/VCUG, how many?]
N/A

Cefaclor: which time schedule do you use? [Day of VUS/VCUG]
N/A

Cefaclor: which time schedule do you use? [Day(s) after VUS/VCUG, how many?]
N/A

Amoxicillin/clavulanic acid: in what dosage? (Amoxicillin portion) [prophylaktic (10-20 mg/Kg bodyweight/d)]
N/A

Amoxicillin/clavulanic acid: in what dosage? (Amoxicillin portion) [double prophylactic (20-40 mg/Kg bodyweight/d)]
N/A

Amoxicillin/clavulanic acid: in what dosage? (Amoxicillin portion) [therapeutic (80-100 mg/Kg bodyweight/d)]
N/A

Amoxicillin/clavulanic acid: which time schedule do you use? [Day(s) before VUS/VCUG]
N/A

Amoxicillin/clavulanic acid: which time schedule do you use? [Day of VUS/VCUG]
N/A

Amoxicillin/clavulanic acid: which time schedule do you use? [Day(s) after VUS/VCUG]
N/A

Other: which time schedule do you use? [Day(s) before VUS/VCUG, how many?]
N/A

Other: which time schedule do you use? [Day of VUS/VCUG]
N/A

Other: which time schedule do you use? [Day(s) after VUS/VCUG, how many?]
N/A

Indication for prophylaxis

Which patients receive periinterventional antibiotic prophylaxis? (multiple answers possible) [all]
N/A

Which patients receive periinterventional antibiotic prophylaxis? (multiple answers possible) [patients post urinary tract infection/pyelonephritis/urosepsis]
N/A

Which patients receive periinterventional antibiotic prophylaxis? (multiple answers possible) [patients with urinary tract dilation in ultrasound]
N/A

Which patients receive periinterventional antibiotic prophylaxis? (multiple answers possible) [patients with prenatal urinary tract dilation]
N/A

Which patients receive periinterventional antibiotic prophylaxis? (multiple answers possible) [patients post VUR surgery]
N/A

Which patients receive periinterventional antibiotic prophylaxis? (multiple answers possible) [none]
N/A

Are there situations or clinical findings in the patient where you deviate from your prophylaxis standard (medication, dose, duration, age of the patient)? (multiple answers possible, please specify in answer box) [in case of already known vesicoureteral reflux (VUR)]
N/A

Are there situations or clinical findings in the patient where you deviate from your prophylaxis standard (medication, dose, duration, age of the patient)? (multiple answers possible, please specify in answer box) [in patients with recurrent urinary tract infections (UTI)]
N/A

Are there situations or clinical findings in the patient where you deviate from your prophylaxis standard (medication, dose, duration, age of the patient)? (multiple answers possible, please specify in answer box) [if multidrug-resistant organisms (MDRO) are detected in urine cultures]
N/A

Are there situations or clinical findings in the patient where you deviate from your prophylaxis standard (medication, dose, duration, age of the patient)? (multiple answers possible, please specify in answer box) [in newborns]
N/A

Are there situations in which you use intravenous prophylaxis?
N/A

Voiding analysis

Are there differences in antibiotic prophylaxis between VUS and VCUG in your department?
N/A

Is a non-pathological urine strip test required in preparation for VUS/VCUG in your department?
N/A

Is a non-pathological urine culture required in preparation for VUS/VCUG in your department?
N/A

Do patients only receive post-procedure prophylaxis if vesicoureteral reflux (VUR) is detected in VUS/VCUG?
N/A

Do other MUS/MCU findings (e.g. urethral valves) have an influence on post-procedure prophylaxis?
N/A

End of survey

May we contact you personally if we have further questions?
N/A

Survey response 20

Response ID
21

Last page
1

Start language
de

Seed
2114075722

Date started
2024-11-14 09:50:37

Date last action
2024-11-14 09:51:00

Standard Operation Procedure

Who fills out this survey?
Pediatric Radiologist

Who fills out this survey? [Other]

In which type of hospital are you employed?
Community hospital (

In which type of hospital are you employed? [Other]

In which country do you work?
Deutschland

Does your department have a defined protocol for infection prophylaxis in the context of VUS/VCUG?
No

Has this protocol been transformed into a written standard operating procedure (SOP)?
N/A

Antibiotic Prophylaxis

Are there situations/constellations in which you do not use antibiotic prophylaxis for VUS/VCUG?
N/A

Please specify these situations/constellations: [girl with first uncomplicated UTI without urinary tract dilation in ultrasound]
N/A

Please specify these situations/constellations: [boy with first uncomplicated UTI without urinary tract dilation in ultrasound]
N/A

Please specify these situations/constellations: [in patients with low grade urinary tract dilation (up to 2nd grade) and no UTI in medical history]
N/A

If you use antibiotic prophylaxis: which mediaction do you use? (multiple answers and subdivision into age groups possible) [Trimethoprim]
N/A

If you use antibiotic prophylaxis: which mediaction do you use? (multiple answers and subdivision into age groups possible) [Nitrofurantoin]
N/A

If you use antibiotic prophylaxis: which mediaction do you use? (multiple answers and subdivision into age groups possible) [Amoxicillin]
N/A

If you use antibiotic prophylaxis: which mediaction do you use? (multiple answers and subdivision into age groups possible) [Cefaclor]
N/A

If you use antibiotic prophylaxis: which mediaction do you use? (multiple answers and subdivision into age groups possible) [Amoxicillin/clavulanic acid]
N/A

If you use antibiotic prophylaxis: which mediaction do you use? (multiple answers and subdivision into age groups possible) [Other]
N/A

Trimethoprim: which time schedule do you use? [Day(s) before VUS/VCUG, how many?]
N/A

Trimethoprim: which time schedule do you use? [Day of VUS/VCUG]
N/A

Trimethoprim: which time schedule do you use? [Day(s) after VUS/VCUG, how many?]
N/A

Nitrofurantoin: which time schedule do you use? [Day(s) before VUS/VCUG, how many?]
N/A

Nitrofurantoin: which time schedule do you use? [Day of VUS/VCUG]
N/A

Nitrofurantoin: which time schedule do you use? [Day(s) after VUS/VCUG, how many?]
N/A

Amoxicillin: which time schedule do you use? [Day(s) before VUS/VCUG, how many?]
N/A

Amoxicillin: which time schedule do you use? [Day of VUS/VCUG]
N/A

Amoxicillin: which time schedule do you use? [Day(s) after VUS/VCUG, how many?]
N/A

Cefaclor: which time schedule do you use? [Days(s) before VUS/VCUG, how many?]
N/A

Cefaclor: which time schedule do you use? [Day of VUS/VCUG]
N/A

Cefaclor: which time schedule do you use? [Day(s) after VUS/VCUG, how many?]
N/A

Amoxicillin/clavulanic acid: in what dosage? (Amoxicillin portion) [prophylaktic (10-20 mg/Kg bodyweight/d)]
N/A

Amoxicillin/clavulanic acid: in what dosage? (Amoxicillin portion) [double prophylactic (20-40 mg/Kg bodyweight/d)]
N/A

Amoxicillin/clavulanic acid: in what dosage? (Amoxicillin portion) [therapeutic (80-100 mg/Kg bodyweight/d)]
N/A

Amoxicillin/clavulanic acid: which time schedule do you use? [Day(s) before VUS/VCUG]
N/A

Amoxicillin/clavulanic acid: which time schedule do you use? [Day of VUS/VCUG]
N/A

Amoxicillin/clavulanic acid: which time schedule do you use? [Day(s) after VUS/VCUG]
N/A

Other: which time schedule do you use? [Day(s) before VUS/VCUG, how many?]
N/A

Other: which time schedule do you use? [Day of VUS/VCUG]
N/A

Other: which time schedule do you use? [Day(s) after VUS/VCUG, how many?]
N/A

Indication for prophylaxis

Which patients receive periinterventional antibiotic prophylaxis? (multiple answers possible) [all]
N/A

Which patients receive periinterventional antibiotic prophylaxis? (multiple answers possible) [patients post urinary tract infection/pyelonephritis/urosepsis]
N/A

Which patients receive periinterventional antibiotic prophylaxis? (multiple answers possible) [patients with urinary tract dilation in ultrasound]
N/A

Which patients receive periinterventional antibiotic prophylaxis? (multiple answers possible) [patients with prenatal urinary tract dilation]
N/A

Which patients receive periinterventional antibiotic prophylaxis? (multiple answers possible) [patients post VUR surgery]
N/A

Which patients receive periinterventional antibiotic prophylaxis? (multiple answers possible) [none]
N/A

Are there situations or clinical findings in the patient where you deviate from your prophylaxis standard (medication, dose, duration, age of the patient)? (multiple answers possible, please specify in answer box) [in case of already known vesicoureteral reflux (VUR)]
N/A

Are there situations or clinical findings in the patient where you deviate from your prophylaxis standard (medication, dose, duration, age of the patient)? (multiple answers possible, please specify in answer box) [in patients with recurrent urinary tract infections (UTI)]
N/A

Are there situations or clinical findings in the patient where you deviate from your prophylaxis standard (medication, dose, duration, age of the patient)? (multiple answers possible, please specify in answer box) [if multidrug-resistant organisms (MDRO) are detected in urine cultures]
N/A

Are there situations or clinical findings in the patient where you deviate from your prophylaxis standard (medication, dose, duration, age of the patient)? (multiple answers possible, please specify in answer box) [in newborns]
N/A

Are there situations in which you use intravenous prophylaxis?
N/A

Voiding analysis

Are there differences in antibiotic prophylaxis between VUS and VCUG in your department?
N/A

Is a non-pathological urine strip test required in preparation for VUS/VCUG in your department?
N/A

Is a non-pathological urine culture required in preparation for VUS/VCUG in your department?
N/A

Do patients only receive post-procedure prophylaxis if vesicoureteral reflux (VUR) is detected in VUS/VCUG?
N/A

Do other MUS/MCU findings (e.g. urethral valves) have an influence on post-procedure prophylaxis?
N/A

End of survey

May we contact you personally if we have further questions?
N/A

Survey response 21

Response ID
22

Last page
1

Start language
de

Seed
1176739842

Date started
2024-11-14 09:52:54

Date last action
2024-11-14 09:54:13

Standard Operation Procedure

Who fills out this survey?
Pediatric Radiologist

Who fills out this survey? [Other]

In which type of hospital are you employed?
Community hospital (>50 beds for pediatric patients)

In which type of hospital are you employed? [Other]

In which country do you work?
Deutschland

Does your department have a defined protocol for infection prophylaxis in the context of VUS/VCUG?
No

Has this protocol been transformed into a written standard operating procedure (SOP)?
N/A

Antibiotic Prophylaxis

Are there situations/constellations in which you do not use antibiotic prophylaxis for VUS/VCUG?
Yes

Please specify these situations/constellations: [girl with first uncomplicated UTI without urinary tract dilation in ultrasound]
Yes

Please specify these situations/constellations: [boy with first uncomplicated UTI without urinary tract dilation in ultrasound]
Yes

Please specify these situations/constellations: [in patients with low grade urinary tract dilation (up to 2nd grade) and no UTI in medical history]
No

Please specify these situations/constellations: [Other]

If you use antibiotic prophylaxis: which mediaction do you use? (multiple answers and subdivision into age groups possible) [Trimethoprim]
Yes

If you use antibiotic prophylaxis: which mediaction do you use? (multiple answers and subdivision into age groups possible) [Nitrofurantoin]
No

If you use antibiotic prophylaxis: which mediaction do you use? (multiple answers and subdivision into age groups possible) [Amoxicillin]
No

If you use antibiotic prophylaxis: which mediaction do you use? (multiple answers and subdivision into age groups possible) [Cefaclor]
No

If you use antibiotic prophylaxis: which mediaction do you use? (multiple answers and subdivision into age groups possible) [Amoxicillin/clavulanic acid]
No

If you use antibiotic prophylaxis: which mediaction do you use? (multiple answers and subdivision into age groups possible) [Other]
No

Trimethoprim: in what dosage?
prophylactic (2 mg/kg bodyweight/d)

Trimethoprim: in what dosage? [Other]

Trimethoprim: for which age group do you use trimethoprim?

Trimethoprim: which time schedule do you use? [Day(s) before VUS/VCUG, how many?]
No

Trimethoprim: which time schedule do you use? [Day(s) before VUS/VCUG, how many?][Comment]

Trimethoprim: which time schedule do you use? [Day of VUS/VCUG]
No

Trimethoprim: which time schedule do you use? [Day of VUS/VCUG][Comment]

Trimethoprim: which time schedule do you use? [Day(s) after VUS/VCUG, how many?]
No

Trimethoprim: which time schedule do you use? [Day(s) after VUS/VCUG, how many?][Comment]

Nitrofurantoin: which time schedule do you use? [Day(s) before VUS/VCUG, how many?]
N/A

Nitrofurantoin: which time schedule do you use? [Day of VUS/VCUG]
N/A

Nitrofurantoin: which time schedule do you use? [Day(s) after VUS/VCUG, how many?]
N/A

Amoxicillin: which time schedule do you use? [Day(s) before VUS/VCUG, how many?]
N/A

Amoxicillin: which time schedule do you use? [Day of VUS/VCUG]
N/A

Amoxicillin: which time schedule do you use? [Day(s) after VUS/VCUG, how many?]
N/A

Cefaclor: which time schedule do you use? [Days(s) before VUS/VCUG, how many?]
N/A

Cefaclor: which time schedule do you use? [Day of VUS/VCUG]
N/A

Cefaclor: which time schedule do you use? [Day(s) after VUS/VCUG, how many?]
N/A

Amoxicillin/clavulanic acid: in what dosage? (Amoxicillin portion) [prophylaktic (10-20 mg/Kg bodyweight/d)]
N/A

Amoxicillin/clavulanic acid: in what dosage? (Amoxicillin portion) [double prophylactic (20-40 mg/Kg bodyweight/d)]
N/A

Amoxicillin/clavulanic acid: in what dosage? (Amoxicillin portion) [therapeutic (80-100 mg/Kg bodyweight/d)]
N/A

Amoxicillin/clavulanic acid: which time schedule do you use? [Day(s) before VUS/VCUG]
N/A

Amoxicillin/clavulanic acid: which time schedule do you use? [Day of VUS/VCUG]
N/A

Amoxicillin/clavulanic acid: which time schedule do you use? [Day(s) after VUS/VCUG]
N/A

Other: which time schedule do you use? [Day(s) before VUS/VCUG, how many?]
N/A

Other: which time schedule do you use? [Day of VUS/VCUG]
N/A

Other: which time schedule do you use? [Day(s) after VUS/VCUG, how many?]
N/A

Indication for prophylaxis

Which patients receive periinterventional antibiotic prophylaxis? (multiple answers possible) [all]
N/A

Which patients receive periinterventional antibiotic prophylaxis? (multiple answers possible) [patients post urinary tract infection/pyelonephritis/urosepsis]
N/A

Which patients receive periinterventional antibiotic prophylaxis? (multiple answers possible) [patients with urinary tract dilation in ultrasound]
N/A

Which patients receive periinterventional antibiotic prophylaxis? (multiple answers possible) [patients with prenatal urinary tract dilation]
N/A

Which patients receive periinterventional antibiotic prophylaxis? (multiple answers possible) [patients post VUR surgery]
N/A

Which patients receive periinterventional antibiotic prophylaxis? (multiple answers possible) [none]
N/A

Are there situations or clinical findings in the patient where you deviate from your prophylaxis standard (medication, dose, duration, age of the patient)? (multiple answers possible, please specify in answer box) [in case of already known vesicoureteral reflux (VUR)]
N/A

Are there situations or clinical findings in the patient where you deviate from your prophylaxis standard (medication, dose, duration, age of the patient)? (multiple answers possible, please specify in answer box) [in patients with recurrent urinary tract infections (UTI)]
N/A

Are there situations or clinical findings in the patient where you deviate from your prophylaxis standard (medication, dose, duration, age of the patient)? (multiple answers possible, please specify in answer box) [if multidrug-resistant organisms (MDRO) are detected in urine cultures]
N/A

Are there situations or clinical findings in the patient where you deviate from your prophylaxis standard (medication, dose, duration, age of the patient)? (multiple answers possible, please specify in answer box) [in newborns]
N/A

Are there situations in which you use intravenous prophylaxis?
N/A

Voiding analysis

Are there differences in antibiotic prophylaxis between VUS and VCUG in your department?
N/A

Is a non-pathological urine strip test required in preparation for VUS/VCUG in your department?
N/A

Is a non-pathological urine culture required in preparation for VUS/VCUG in your department?
N/A

Do patients only receive post-procedure prophylaxis if vesicoureteral reflux (VUR) is detected in VUS/VCUG?
N/A

Do other MUS/MCU findings (e.g. urethral valves) have an influence on post-procedure prophylaxis?
N/A

End of survey

May we contact you personally if we have further questions?
N/A

Survey response 22

Response ID
23

Last page
1

Start language
de

Seed
462790082

Date started
2024-11-14 10:14:31

Date last action
2024-11-14 10:15:44

Standard Operation Procedure

Who fills out this survey?
Pediatric Radiologist

Who fills out this survey? [Other]

In which type of hospital are you employed?
Community hospital (>50 beds for pediatric patients)

In which type of hospital are you employed? [Other]

In which country do you work?
Deutschland

Does your department have a defined protocol for infection prophylaxis in the context of VUS/VCUG?
No

Has this protocol been transformed into a written standard operating procedure (SOP)?
N/A

Antibiotic Prophylaxis

Are there situations/constellations in which you do not use antibiotic prophylaxis for VUS/VCUG?
N/A

Please specify these situations/constellations: [girl with first uncomplicated UTI without urinary tract dilation in ultrasound]
N/A

Please specify these situations/constellations: [boy with first uncomplicated UTI without urinary tract dilation in ultrasound]
N/A

Please specify these situations/constellations: [in patients with low grade urinary tract dilation (up to 2nd grade) and no UTI in medical history]
N/A

If you use antibiotic prophylaxis: which mediaction do you use? (multiple answers and subdivision into age groups possible) [Trimethoprim]
No

If you use antibiotic prophylaxis: which mediaction do you use? (multiple answers and subdivision into age groups possible) [Nitrofurantoin]
No

If you use antibiotic prophylaxis: which mediaction do you use? (multiple answers and subdivision into age groups possible) [Amoxicillin]
No

If you use antibiotic prophylaxis: which mediaction do you use? (multiple answers and subdivision into age groups possible) [Cefaclor]
No

If you use antibiotic prophylaxis: which mediaction do you use? (multiple answers and subdivision into age groups possible) [Amoxicillin/clavulanic acid]
No

If you use antibiotic prophylaxis: which mediaction do you use? (multiple answers and subdivision into age groups possible) [Other]
No

Trimethoprim: which time schedule do you use? [Day(s) before VUS/VCUG, how many?]
N/A

Trimethoprim: which time schedule do you use? [Day of VUS/VCUG]
N/A

Trimethoprim: which time schedule do you use? [Day(s) after VUS/VCUG, how many?]
N/A

Nitrofurantoin: which time schedule do you use? [Day(s) before VUS/VCUG, how many?]
N/A

Nitrofurantoin: which time schedule do you use? [Day of VUS/VCUG]
N/A

Nitrofurantoin: which time schedule do you use? [Day(s) after VUS/VCUG, how many?]
N/A

Amoxicillin: which time schedule do you use? [Day(s) before VUS/VCUG, how many?]
N/A

Amoxicillin: which time schedule do you use? [Day of VUS/VCUG]
N/A

Amoxicillin: which time schedule do you use? [Day(s) after VUS/VCUG, how many?]
N/A

Cefaclor: which time schedule do you use? [Days(s) before VUS/VCUG, how many?]
N/A

Cefaclor: which time schedule do you use? [Day of VUS/VCUG]
N/A

Cefaclor: which time schedule do you use? [Day(s) after VUS/VCUG, how many?]
N/A

Amoxicillin/clavulanic acid: in what dosage? (Amoxicillin portion) [prophylaktic (10-20 mg/Kg bodyweight/d)]
N/A

Amoxicillin/clavulanic acid: in what dosage? (Amoxicillin portion) [double prophylactic (20-40 mg/Kg bodyweight/d)]
N/A

Amoxicillin/clavulanic acid: in what dosage? (Amoxicillin portion) [therapeutic (80-100 mg/Kg bodyweight/d)]
N/A

Amoxicillin/clavulanic acid: which time schedule do you use? [Day(s) before VUS/VCUG]
N/A

Amoxicillin/clavulanic acid: which time schedule do you use? [Day of VUS/VCUG]
N/A

Amoxicillin/clavulanic acid: which time schedule do you use? [Day(s) after VUS/VCUG]
N/A

Other: which time schedule do you use? [Day(s) before VUS/VCUG, how many?]
N/A

Other: which time schedule do you use? [Day of VUS/VCUG]
N/A

Other: which time schedule do you use? [Day(s) after VUS/VCUG, how many?]
N/A

Indication for prophylaxis

Which patients receive periinterventional antibiotic prophylaxis? (multiple answers possible) [all]
N/A

Which patients receive periinterventional antibiotic prophylaxis? (multiple answers possible) [patients post urinary tract infection/pyelonephritis/urosepsis]
N/A

Which patients receive periinterventional antibiotic prophylaxis? (multiple answers possible) [patients with urinary tract dilation in ultrasound]
N/A

Which patients receive periinterventional antibiotic prophylaxis? (multiple answers possible) [patients with prenatal urinary tract dilation]
N/A

Which patients receive periinterventional antibiotic prophylaxis? (multiple answers possible) [patients post VUR surgery]
N/A

Which patients receive periinterventional antibiotic prophylaxis? (multiple answers possible) [none]
N/A

Are there situations or clinical findings in the patient where you deviate from your prophylaxis standard (medication, dose, duration, age of the patient)? (multiple answers possible, please specify in answer box) [in case of already known vesicoureteral reflux (VUR)]
N/A

Are there situations or clinical findings in the patient where you deviate from your prophylaxis standard (medication, dose, duration, age of the patient)? (multiple answers possible, please specify in answer box) [in patients with recurrent urinary tract infections (UTI)]
N/A

Are there situations or clinical findings in the patient where you deviate from your prophylaxis standard (medication, dose, duration, age of the patient)? (multiple answers possible, please specify in answer box) [if multidrug-resistant organisms (MDRO) are detected in urine cultures]
N/A

Are there situations or clinical findings in the patient where you deviate from your prophylaxis standard (medication, dose, duration, age of the patient)? (multiple answers possible, please specify in answer box) [in newborns]
N/A

Are there situations in which you use intravenous prophylaxis?
N/A

Voiding analysis

Are there differences in antibiotic prophylaxis between VUS and VCUG in your department?
N/A

Is a non-pathological urine strip test required in preparation for VUS/VCUG in your department?
N/A

Is a non-pathological urine culture required in preparation for VUS/VCUG in your department?
N/A

Do patients only receive post-procedure prophylaxis if vesicoureteral reflux (VUR) is detected in VUS/VCUG?
N/A

Do other MUS/MCU findings (e.g. urethral valves) have an influence on post-procedure prophylaxis?
N/A

End of survey

May we contact you personally if we have further questions?
N/A

Survey response 23

Response ID
24

Last page
1

Start language
de

Seed
1764369492

Date started
2024-11-14 10:19:08

Date last action
2024-11-14 10:19:44

Standard Operation Procedure

Who fills out this survey?
Pediatric Radiologist

Who fills out this survey? [Other]

In which type of hospital are you employed?
Community hospital (>50 beds for pediatric patients)

In which type of hospital are you employed? [Other]

In which country do you work?
Deutschlnad

Does your department have a defined protocol for infection prophylaxis in the context of VUS/VCUG?
No

Has this protocol been transformed into a written standard operating procedure (SOP)?
N/A

Antibiotic Prophylaxis

Are there situations/constellations in which you do not use antibiotic prophylaxis for VUS/VCUG?
N/A

Please specify these situations/constellations: [girl with first uncomplicated UTI without urinary tract dilation in ultrasound]
N/A

Please specify these situations/constellations: [boy with first uncomplicated UTI without urinary tract dilation in ultrasound]
N/A

Please specify these situations/constellations: [in patients with low grade urinary tract dilation (up to 2nd grade) and no UTI in medical history]
N/A

If you use antibiotic prophylaxis: which mediaction do you use? (multiple answers and subdivision into age groups possible) [Trimethoprim]
N/A

If you use antibiotic prophylaxis: which mediaction do you use? (multiple answers and subdivision into age groups possible) [Nitrofurantoin]
N/A

If you use antibiotic prophylaxis: which mediaction do you use? (multiple answers and subdivision into age groups possible) [Amoxicillin]
N/A

If you use antibiotic prophylaxis: which mediaction do you use? (multiple answers and subdivision into age groups possible) [Cefaclor]
N/A

If you use antibiotic prophylaxis: which mediaction do you use? (multiple answers and subdivision into age groups possible) [Amoxicillin/clavulanic acid]
N/A

If you use antibiotic prophylaxis: which mediaction do you use? (multiple answers and subdivision into age groups possible) [Other]
N/A

Trimethoprim: which time schedule do you use? [Day(s) before VUS/VCUG, how many?]
N/A

Trimethoprim: which time schedule do you use? [Day of VUS/VCUG]
N/A

Trimethoprim: which time schedule do you use? [Day(s) after VUS/VCUG, how many?]
N/A

Nitrofurantoin: which time schedule do you use? [Day(s) before VUS/VCUG, how many?]
N/A

Nitrofurantoin: which time schedule do you use? [Day of VUS/VCUG]
N/A

Nitrofurantoin: which time schedule do you use? [Day(s) after VUS/VCUG, how many?]
N/A

Amoxicillin: which time schedule do you use? [Day(s) before VUS/VCUG, how many?]
N/A

Amoxicillin: which time schedule do you use? [Day of VUS/VCUG]
N/A

Amoxicillin: which time schedule do you use? [Day(s) after VUS/VCUG, how many?]
N/A

Cefaclor: which time schedule do you use? [Days(s) before VUS/VCUG, how many?]
N/A

Cefaclor: which time schedule do you use? [Day of VUS/VCUG]
N/A

Cefaclor: which time schedule do you use? [Day(s) after VUS/VCUG, how many?]
N/A

Amoxicillin/clavulanic acid: in what dosage? (Amoxicillin portion) [prophylaktic (10-20 mg/Kg bodyweight/d)]
N/A

Amoxicillin/clavulanic acid: in what dosage? (Amoxicillin portion) [double prophylactic (20-40 mg/Kg bodyweight/d)]
N/A

Amoxicillin/clavulanic acid: in what dosage? (Amoxicillin portion) [therapeutic (80-100 mg/Kg bodyweight/d)]
N/A

Amoxicillin/clavulanic acid: which time schedule do you use? [Day(s) before VUS/VCUG]
N/A

Amoxicillin/clavulanic acid: which time schedule do you use? [Day of VUS/VCUG]
N/A

Amoxicillin/clavulanic acid: which time schedule do you use? [Day(s) after VUS/VCUG]
N/A

Other: which time schedule do you use? [Day(s) before VUS/VCUG, how many?]
N/A

Other: which time schedule do you use? [Day of VUS/VCUG]
N/A

Other: which time schedule do you use? [Day(s) after VUS/VCUG, how many?]
N/A

Indication for prophylaxis

Which patients receive periinterventional antibiotic prophylaxis? (multiple answers possible) [all]
N/A

Which patients receive periinterventional antibiotic prophylaxis? (multiple answers possible) [patients post urinary tract infection/pyelonephritis/urosepsis]
N/A

Which patients receive periinterventional antibiotic prophylaxis? (multiple answers possible) [patients with urinary tract dilation in ultrasound]
N/A

Which patients receive periinterventional antibiotic prophylaxis? (multiple answers possible) [patients with prenatal urinary tract dilation]
N/A

Which patients receive periinterventional antibiotic prophylaxis? (multiple answers possible) [patients post VUR surgery]
N/A

Which patients receive periinterventional antibiotic prophylaxis? (multiple answers possible) [none]
N/A

Are there situations or clinical findings in the patient where you deviate from your prophylaxis standard (medication, dose, duration, age of the patient)? (multiple answers possible, please specify in answer box) [in case of already known vesicoureteral reflux (VUR)]
N/A

Are there situations or clinical findings in the patient where you deviate from your prophylaxis standard (medication, dose, duration, age of the patient)? (multiple answers possible, please specify in answer box) [in patients with recurrent urinary tract infections (UTI)]
N/A

Are there situations or clinical findings in the patient where you deviate from your prophylaxis standard (medication, dose, duration, age of the patient)? (multiple answers possible, please specify in answer box) [if multidrug-resistant organisms (MDRO) are detected in urine cultures]
N/A

Are there situations or clinical findings in the patient where you deviate from your prophylaxis standard (medication, dose, duration, age of the patient)? (multiple answers possible, please specify in answer box) [in newborns]
N/A

Are there situations in which you use intravenous prophylaxis?
N/A

Voiding analysis

Are there differences in antibiotic prophylaxis between VUS and VCUG in your department?
N/A

Is a non-pathological urine strip test required in preparation for VUS/VCUG in your department?
N/A

Is a non-pathological urine culture required in preparation for VUS/VCUG in your department?
N/A

Do patients only receive post-procedure prophylaxis if vesicoureteral reflux (VUR) is detected in VUS/VCUG?
N/A

Do other MUS/MCU findings (e.g. urethral valves) have an influence on post-procedure prophylaxis?
N/A

End of survey

May we contact you personally if we have further questions?
N/A

Survey response 24

Response ID
25

Last page
1

Start language
de

Seed
1644144530

Date started
2024-11-14 14:33:46

Date last action
2024-11-14 14:34:08

Standard Operation Procedure

Who fills out this survey?
Radiologist

Who fills out this survey? [Other]

In which type of hospital are you employed?
University Hospital

In which type of hospital are you employed? [Other]

In which country do you work?
Schweiz

Does your department have a defined protocol for infection prophylaxis in the context of VUS/VCUG?
No

Has this protocol been transformed into a written standard operating procedure (SOP)?
N/A

Antibiotic Prophylaxis

Are there situations/constellations in which you do not use antibiotic prophylaxis for VUS/VCUG?
N/A

Please specify these situations/constellations: [girl with first uncomplicated UTI without urinary tract dilation in ultrasound]
N/A

Please specify these situations/constellations: [boy with first uncomplicated UTI without urinary tract dilation in ultrasound]
N/A

Please specify these situations/constellations: [in patients with low grade urinary tract dilation (up to 2nd grade) and no UTI in medical history]
N/A

If you use antibiotic prophylaxis: which mediaction do you use? (multiple answers and subdivision into age groups possible) [Trimethoprim]
N/A

If you use antibiotic prophylaxis: which mediaction do you use? (multiple answers and subdivision into age groups possible) [Nitrofurantoin]
N/A

If you use antibiotic prophylaxis: which mediaction do you use? (multiple answers and subdivision into age groups possible) [Amoxicillin]
N/A

If you use antibiotic prophylaxis: which mediaction do you use? (multiple answers and subdivision into age groups possible) [Cefaclor]
N/A

If you use antibiotic prophylaxis: which mediaction do you use? (multiple answers and subdivision into age groups possible) [Amoxicillin/clavulanic acid]
N/A

If you use antibiotic prophylaxis: which mediaction do you use? (multiple answers and subdivision into age groups possible) [Other]
N/A

Trimethoprim: which time schedule do you use? [Day(s) before VUS/VCUG, how many?]
N/A

Trimethoprim: which time schedule do you use? [Day of VUS/VCUG]
N/A

Trimethoprim: which time schedule do you use? [Day(s) after VUS/VCUG, how many?]
N/A

Nitrofurantoin: which time schedule do you use? [Day(s) before VUS/VCUG, how many?]
N/A

Nitrofurantoin: which time schedule do you use? [Day of VUS/VCUG]
N/A

Nitrofurantoin: which time schedule do you use? [Day(s) after VUS/VCUG, how many?]
N/A

Amoxicillin: which time schedule do you use? [Day(s) before VUS/VCUG, how many?]
N/A

Amoxicillin: which time schedule do you use? [Day of VUS/VCUG]
N/A

Amoxicillin: which time schedule do you use? [Day(s) after VUS/VCUG, how many?]
N/A

Cefaclor: which time schedule do you use? [Days(s) before VUS/VCUG, how many?]
N/A

Cefaclor: which time schedule do you use? [Day of VUS/VCUG]
N/A

Cefaclor: which time schedule do you use? [Day(s) after VUS/VCUG, how many?]
N/A

Amoxicillin/clavulanic acid: in what dosage? (Amoxicillin portion) [prophylaktic (10-20 mg/Kg bodyweight/d)]
N/A

Amoxicillin/clavulanic acid: in what dosage? (Amoxicillin portion) [double prophylactic (20-40 mg/Kg bodyweight/d)]
N/A

Amoxicillin/clavulanic acid: in what dosage? (Amoxicillin portion) [therapeutic (80-100 mg/Kg bodyweight/d)]
N/A

Amoxicillin/clavulanic acid: which time schedule do you use? [Day(s) before VUS/VCUG]
N/A

Amoxicillin/clavulanic acid: which time schedule do you use? [Day of VUS/VCUG]
N/A

Amoxicillin/clavulanic acid: which time schedule do you use? [Day(s) after VUS/VCUG]
N/A

Other: which time schedule do you use? [Day(s) before VUS/VCUG, how many?]
N/A

Other: which time schedule do you use? [Day of VUS/VCUG]
N/A

Other: which time schedule do you use? [Day(s) after VUS/VCUG, how many?]
N/A

Indication for prophylaxis

Which patients receive periinterventional antibiotic prophylaxis? (multiple answers possible) [all]
N/A

Which patients receive periinterventional antibiotic prophylaxis? (multiple answers possible) [patients post urinary tract infection/pyelonephritis/urosepsis]
N/A

Which patients receive periinterventional antibiotic prophylaxis? (multiple answers possible) [patients with urinary tract dilation in ultrasound]
N/A

Which patients receive periinterventional antibiotic prophylaxis? (multiple answers possible) [patients with prenatal urinary tract dilation]
N/A

Which patients receive periinterventional antibiotic prophylaxis? (multiple answers possible) [patients post VUR surgery]
N/A

Which patients receive periinterventional antibiotic prophylaxis? (multiple answers possible) [none]
N/A

Are there situations or clinical findings in the patient where you deviate from your prophylaxis standard (medication, dose, duration, age of the patient)? (multiple answers possible, please specify in answer box) [in case of already known vesicoureteral reflux (VUR)]
N/A

Are there situations or clinical findings in the patient where you deviate from your prophylaxis standard (medication, dose, duration, age of the patient)? (multiple answers possible, please specify in answer box) [in patients with recurrent urinary tract infections (UTI)]
N/A

Are there situations or clinical findings in the patient where you deviate from your prophylaxis standard (medication, dose, duration, age of the patient)? (multiple answers possible, please specify in answer box) [if multidrug-resistant organisms (MDRO) are detected in urine cultures]
N/A

Are there situations or clinical findings in the patient where you deviate from your prophylaxis standard (medication, dose, duration, age of the patient)? (multiple answers possible, please specify in answer box) [in newborns]
N/A

Are there situations in which you use intravenous prophylaxis?
N/A

Voiding analysis

Are there differences in antibiotic prophylaxis between VUS and VCUG in your department?
N/A

Is a non-pathological urine strip test required in preparation for VUS/VCUG in your department?
N/A

Is a non-pathological urine culture required in preparation for VUS/VCUG in your department?
N/A

Do patients only receive post-procedure prophylaxis if vesicoureteral reflux (VUR) is detected in VUS/VCUG?
N/A

Do other MUS/MCU findings (e.g. urethral valves) have an influence on post-procedure prophylaxis?
N/A

End of survey

May we contact you personally if we have further questions?
N/A

Survey response 25

Response ID
26

Date submitted
2024-11-14 15:20:14

Last page
5

Start language
de

Seed
2001302170

Date started
2024-11-14 15:13:24

Date last action
2024-11-14 15:20:14

Standard Operation Procedure

Who fills out this survey?
Pediatric Radiologist

Who fills out this survey? [Other]

In which type of hospital are you employed?
University Hospital

In which type of hospital are you employed? [Other]

In which country do you work?
MV

Does your department have a defined protocol for infection prophylaxis in the context of VUS/VCUG?
Yes

Has this protocol been transformed into a written standard operating procedure (SOP)?
Yes

Which department is the author of the SOP for infection prophylaxis in VUS/VCUG?
Pediatric nephrology

Which department is the author of the SOP for infection prophylaxis in VUS/VCUG? [Other]

Antibiotic Prophylaxis

Are there situations/constellations in which you do not use antibiotic prophylaxis for VUS/VCUG?
No

Please specify these situations/constellations: [girl with first uncomplicated UTI without urinary tract dilation in ultrasound]
N/A

Please specify these situations/constellations: [boy with first uncomplicated UTI without urinary tract dilation in ultrasound]
N/A

Please specify these situations/constellations: [in patients with low grade urinary tract dilation (up to 2nd grade) and no UTI in medical history]
N/A

If you use antibiotic prophylaxis: which mediaction do you use? (multiple answers and subdivision into age groups possible) [Trimethoprim]
No

If you use antibiotic prophylaxis: which mediaction do you use? (multiple answers and subdivision into age groups possible) [Nitrofurantoin]
No

If you use antibiotic prophylaxis: which mediaction do you use? (multiple answers and subdivision into age groups possible) [Amoxicillin]
Yes

If you use antibiotic prophylaxis: which mediaction do you use? (multiple answers and subdivision into age groups possible) [Cefaclor]
No

If you use antibiotic prophylaxis: which mediaction do you use? (multiple answers and subdivision into age groups possible) [Amoxicillin/clavulanic acid]
No

If you use antibiotic prophylaxis: which mediaction do you use? (multiple answers and subdivision into age groups possible) [Other]
No

Trimethoprim: which time schedule do you use? [Day(s) before VUS/VCUG, how many?]
N/A

Trimethoprim: which time schedule do you use? [Day of VUS/VCUG]
N/A

Trimethoprim: which time schedule do you use? [Day(s) after VUS/VCUG, how many?]
N/A

Nitrofurantoin: which time schedule do you use? [Day(s) before VUS/VCUG, how many?]
N/A

Nitrofurantoin: which time schedule do you use? [Day of VUS/VCUG]
N/A

Nitrofurantoin: which time schedule do you use? [Day(s) after VUS/VCUG, how many?]
N/A

Amoxicillin: in what dosage?
prophylactic (10-20 mg/kg bodyweight/d)

Amoxicillin: in what dosage? [Other]

Amoxicillin: for which age group do you use amoxicillin?
0-5

Amoxicillin: which time schedule do you use? [Day(s) before VUS/VCUG, how many?]
No

Amoxicillin: which time schedule do you use? [Day(s) before VUS/VCUG, how many?][Comment]

Amoxicillin: which time schedule do you use? [Day of VUS/VCUG]
Yes

Amoxicillin: which time schedule do you use? [Day of VUS/VCUG][Comment]

Amoxicillin: which time schedule do you use? [Day(s) after VUS/VCUG, how many?]
Yes

Amoxicillin: which time schedule do you use? [Day(s) after VUS/VCUG, how many?][Comment]
3

Cefaclor: which time schedule do you use? [Days(s) before VUS/VCUG, how many?]
N/A

Cefaclor: which time schedule do you use? [Day of VUS/VCUG]
N/A

Cefaclor: which time schedule do you use? [Day(s) after VUS/VCUG, how many?]
N/A

Amoxicillin/clavulanic acid: in what dosage? (Amoxicillin portion) [prophylaktic (10-20 mg/Kg bodyweight/d)]
N/A

Amoxicillin/clavulanic acid: in what dosage? (Amoxicillin portion) [double prophylactic (20-40 mg/Kg bodyweight/d)]
N/A

Amoxicillin/clavulanic acid: in what dosage? (Amoxicillin portion) [therapeutic (80-100 mg/Kg bodyweight/d)]
N/A

Amoxicillin/clavulanic acid: which time schedule do you use? [Day(s) before VUS/VCUG]
N/A

Amoxicillin/clavulanic acid: which time schedule do you use? [Day of VUS/VCUG]
N/A

Amoxicillin/clavulanic acid: which time schedule do you use? [Day(s) after VUS/VCUG]
N/A

Other: which time schedule do you use? [Day(s) before VUS/VCUG, how many?]
N/A

Other: which time schedule do you use? [Day of VUS/VCUG]
N/A

Other: which time schedule do you use? [Day(s) after VUS/VCUG, how many?]
N/A

Indication for prophylaxis

Which patients receive periinterventional antibiotic prophylaxis? (multiple answers possible) [all]
No

Which patients receive periinterventional antibiotic prophylaxis? (multiple answers possible) [patients post urinary tract infection/pyelonephritis/urosepsis]
Yes

Which patients receive periinterventional antibiotic prophylaxis? (multiple answers possible) [patients with urinary tract dilation in ultrasound]
No

Which patients receive periinterventional antibiotic prophylaxis? (multiple answers possible) [patients with prenatal urinary tract dilation]
No

Which patients receive periinterventional antibiotic prophylaxis? (multiple answers possible) [patients post VUR surgery]
No

Which patients receive periinterventional antibiotic prophylaxis? (multiple answers possible) [none]
No

Which patients receive periinterventional antibiotic prophylaxis? (multiple answers possible) [Other]

patients status post urinary tract infection/pyelonephritis/urosepsis: what is the minimum time between infection and VUS/VCUG? (in days)
14

Are there situations or clinical findings in the patient where you deviate from your prophylaxis standard (medication, dose, duration, age of the patient)? (multiple answers possible, please specify in answer box) [in case of already known vesicoureteral reflux (VUR)]
No

Are there situations or clinical findings in the patient where you deviate from your prophylaxis standard (medication, dose, duration, age of the patient)? (multiple answers possible, please specify in answer box) [in case of already known vesicoureteral reflux (VUR)][Comment]

Are there situations or clinical findings in the patient where you deviate from your prophylaxis standard (medication, dose, duration, age of the patient)? (multiple answers possible, please specify in answer box) [in patients with recurrent urinary tract infections (UTI)]
No

Are there situations or clinical findings in the patient where you deviate from your prophylaxis standard (medication, dose, duration, age of the patient)? (multiple answers possible, please specify in answer box) [in patients with recurrent urinary tract infections (UTI)][Comment]

Are there situations or clinical findings in the patient where you deviate from your prophylaxis standard (medication, dose, duration, age of the patient)? (multiple answers possible, please specify in answer box) [if multidrug-resistant organisms (MDRO) are detected in urine cultures]
Yes

Are there situations or clinical findings in the patient where you deviate from your prophylaxis standard (medication, dose, duration, age of the patient)? (multiple answers possible, please specify in answer box) [if multidrug-resistant organisms (MDRO) are detected in urine cultures][Comment]
Entsprechend Resitogramm

Are there situations or clinical findings in the patient where you deviate from your prophylaxis standard (medication, dose, duration, age of the patient)? (multiple answers possible, please specify in answer box) [in newborns]
No

Are there situations or clinical findings in the patient where you deviate from your prophylaxis standard (medication, dose, duration, age of the patient)? (multiple answers possible, please specify in answer box) [in newborns][Comment]

Are there situations or clinical findings in the patient where you deviate from your prophylaxis standard (medication, dose, duration, age of the patient)? (multiple answers possible, please specify in answer box) [Other]

Are there situations or clinical findings in the patient where you deviate from your prophylaxis standard (medication, dose, duration, age of the patient)? (multiple answers possible, please specify in answer box) [Other comment]

Are there situations in which you use intravenous prophylaxis?
No

Voiding analysis

Are there differences in antibiotic prophylaxis between VUS and VCUG in your department?
No

Is a non-pathological urine strip test required in preparation for VUS/VCUG in your department?
Yes

What is the maximum amount of days accepted for urine strip test to be performed prior to the exam?
0

Is a non-pathological urine culture required in preparation for VUS/VCUG in your department?
No

Do patients only receive post-procedure prophylaxis if vesicoureteral reflux (VUR) is detected in VUS/VCUG?
No

Do other MUS/MCU findings (e.g. urethral valves) have an influence on post-procedure prophylaxis?
No

End of survey

Thank you for taking the time to answer this survey!

May we contact you personally if we have further questions?
Yes

E-mail address:
Christina.Hauenstein@med.uni-Rostock.de

Survey response 26

Response ID
27

Date submitted
2024-11-14 17:47:41

Last page
5

Start language
de

Seed
1688229646

Date started
2024-11-14 17:37:22

Date last action
2024-11-14 17:47:41

Standard Operation Procedure

Who fills out this survey?
Pediatric Radiologist

Who fills out this survey? [Other]

In which type of hospital are you employed?
University Hospital

In which type of hospital are you employed? [Other]

In which country do you work?
Deutschland

Does your department have a defined protocol for infection prophylaxis in the context of VUS/VCUG?
Yes

Has this protocol been transformed into a written standard operating procedure (SOP)?
Yes

Which department is the author of the SOP for infection prophylaxis in VUS/VCUG?
Other

Which department is the author of the SOP for infection prophylaxis in VUS/VCUG? [Other]
Kinderurologie

Antibiotic Prophylaxis

Are there situations/constellations in which you do not use antibiotic prophylaxis for VUS/VCUG?
Yes

Please specify these situations/constellations: [girl with first uncomplicated UTI without urinary tract dilation in ultrasound]
Yes

Please specify these situations/constellations: [boy with first uncomplicated UTI without urinary tract dilation in ultrasound]
Yes

Please specify these situations/constellations: [in patients with low grade urinary tract dilation (up to 2nd grade) and no UTI in medical history]
Yes

Please specify these situations/constellations: [Other]

If you use antibiotic prophylaxis: which mediaction do you use? (multiple answers and subdivision into age groups possible) [Trimethoprim]
Yes

If you use antibiotic prophylaxis: which mediaction do you use? (multiple answers and subdivision into age groups possible) [Nitrofurantoin]
No

If you use antibiotic prophylaxis: which mediaction do you use? (multiple answers and subdivision into age groups possible) [Amoxicillin]
No

If you use antibiotic prophylaxis: which mediaction do you use? (multiple answers and subdivision into age groups possible) [Cefaclor]
No

If you use antibiotic prophylaxis: which mediaction do you use? (multiple answers and subdivision into age groups possible) [Amoxicillin/clavulanic acid]
No

If you use antibiotic prophylaxis: which mediaction do you use? (multiple answers and subdivision into age groups possible) [Other]
No

Trimethoprim: in what dosage?
prophylactic (2 mg/kg bodyweight/d)

Trimethoprim: in what dosage? [Other]

Trimethoprim: for which age group do you use trimethoprim?
Ab 6 Wochen

Trimethoprim: which time schedule do you use? [Day(s) before VUS/VCUG, how many?]
No

Trimethoprim: which time schedule do you use? [Day(s) before VUS/VCUG, how many?][Comment]

Trimethoprim: which time schedule do you use? [Day of VUS/VCUG]
Yes

Trimethoprim: which time schedule do you use? [Day of VUS/VCUG][Comment]

Trimethoprim: which time schedule do you use? [Day(s) after VUS/VCUG, how many?]
Yes

Trimethoprim: which time schedule do you use? [Day(s) after VUS/VCUG, how many?][Comment]
2 bei V.a. iatrogene Verschmutzung

Nitrofurantoin: which time schedule do you use? [Day(s) before VUS/VCUG, how many?]
N/A

Nitrofurantoin: which time schedule do you use? [Day of VUS/VCUG]
N/A

Nitrofurantoin: which time schedule do you use? [Day(s) after VUS/VCUG, how many?]
N/A

Amoxicillin: which time schedule do you use? [Day(s) before VUS/VCUG, how many?]
N/A

Amoxicillin: which time schedule do you use? [Day of VUS/VCUG]
N/A

Amoxicillin: which time schedule do you use? [Day(s) after VUS/VCUG, how many?]
N/A

Cefaclor: which time schedule do you use? [Days(s) before VUS/VCUG, how many?]
N/A

Cefaclor: which time schedule do you use? [Day of VUS/VCUG]
N/A

Cefaclor: which time schedule do you use? [Day(s) after VUS/VCUG, how many?]
N/A

Amoxicillin/clavulanic acid: in what dosage? (Amoxicillin portion) [prophylaktic (10-20 mg/Kg bodyweight/d)]
N/A

Amoxicillin/clavulanic acid: in what dosage? (Amoxicillin portion) [double prophylactic (20-40 mg/Kg bodyweight/d)]
N/A

Amoxicillin/clavulanic acid: in what dosage? (Amoxicillin portion) [therapeutic (80-100 mg/Kg bodyweight/d)]
N/A

Amoxicillin/clavulanic acid: which time schedule do you use? [Day(s) before VUS/VCUG]
N/A

Amoxicillin/clavulanic acid: which time schedule do you use? [Day of VUS/VCUG]
N/A

Amoxicillin/clavulanic acid: which time schedule do you use? [Day(s) after VUS/VCUG]
N/A

Other: which time schedule do you use? [Day(s) before VUS/VCUG, how many?]
N/A

Other: which time schedule do you use? [Day of VUS/VCUG]
N/A

Other: which time schedule do you use? [Day(s) after VUS/VCUG, how many?]
N/A

Indication for prophylaxis

Which patients receive periinterventional antibiotic prophylaxis? (multiple answers possible) [all]
No

Which patients receive periinterventional antibiotic prophylaxis? (multiple answers possible) [patients post urinary tract infection/pyelonephritis/urosepsis]
No

Which patients receive periinterventional antibiotic prophylaxis? (multiple answers possible) [patients with urinary tract dilation in ultrasound]
No

Which patients receive periinterventional antibiotic prophylaxis? (multiple answers possible) [patients with prenatal urinary tract dilation]
No

Which patients receive periinterventional antibiotic prophylaxis? (multiple answers possible) [patients post VUR surgery]
No

Which patients receive periinterventional antibiotic prophylaxis? (multiple answers possible) [none]
No

Which patients receive periinterventional antibiotic prophylaxis? (multiple answers possible) [Other]
Z.n. HWI fieberhaft mit Harntraktdilatation

Are there situations or clinical findings in the patient where you deviate from your prophylaxis standard (medication, dose, duration, age of the patient)? (multiple answers possible, please specify in answer box) [in case of already known vesicoureteral reflux (VUR)]
No

Are there situations or clinical findings in the patient where you deviate from your prophylaxis standard (medication, dose, duration, age of the patient)? (multiple answers possible, please specify in answer box) [in case of already known vesicoureteral reflux (VUR)][Comment]

Are there situations or clinical findings in the patient where you deviate from your prophylaxis standard (medication, dose, duration, age of the patient)? (multiple answers possible, please specify in answer box) [in patients with recurrent urinary tract infections (UTI)]
No

Are there situations or clinical findings in the patient where you deviate from your prophylaxis standard (medication, dose, duration, age of the patient)? (multiple answers possible, please specify in answer box) [in patients with recurrent urinary tract infections (UTI)][Comment]

Are there situations or clinical findings in the patient where you deviate from your prophylaxis standard (medication, dose, duration, age of the patient)? (multiple answers possible, please specify in answer box) [if multidrug-resistant organisms (MDRO) are detected in urine cultures]
Yes

Are there situations or clinical findings in the patient where you deviate from your prophylaxis standard (medication, dose, duration, age of the patient)? (multiple answers possible, please specify in answer box) [if multidrug-resistant organisms (MDRO) are detected in urine cultures][Comment]
Nach Antibiogramm

Are there situations or clinical findings in the patient where you deviate from your prophylaxis standard (medication, dose, duration, age of the patient)? (multiple answers possible, please specify in answer box) [in newborns]
No

Are there situations or clinical findings in the patient where you deviate from your prophylaxis standard (medication, dose, duration, age of the patient)? (multiple answers possible, please specify in answer box) [in newborns][Comment]

Are there situations or clinical findings in the patient where you deviate from your prophylaxis standard (medication, dose, duration, age of the patient)? (multiple answers possible, please specify in answer box) [Other]

Are there situations or clinical findings in the patient where you deviate from your prophylaxis standard (medication, dose, duration, age of the patient)? (multiple answers possible, please specify in answer box) [Other comment]

Are there situations in which you use intravenous prophylaxis?
Yes

In which situations?
Keine eigentliche Prophylaxe, sondern bei stationärem Aufenthalt zur i.v. Therapie bei HWI, im Rahmen dessen die Diagnostik durchgeführt wird

Voiding analysis

Are there differences in antibiotic prophylaxis between VUS and VCUG in your department?
No

Is a non-pathological urine strip test required in preparation for VUS/VCUG in your department?
No

Is a non-pathological urine culture required in preparation for VUS/VCUG in your department?
No

Do patients only receive post-procedure prophylaxis if vesicoureteral reflux (VUR) is detected in VUS/VCUG?
No

Do other MUS/MCU findings (e.g. urethral valves) have an influence on post-procedure prophylaxis?
No

End of survey

Thank you for taking the time to answer this survey!

May we contact you personally if we have further questions?
Yes

E-mail address:
Barbara.greiner@ukr.de

Survey response 27

Response ID
28

Last page
1

Start language
de

Seed
316073018

Date started
2024-11-14 20:50:19

Date last action
2024-11-14 20:52:58

Standard Operation Procedure

Who fills out this survey?
Pediatric Radiologist

Who fills out this survey? [Other]

In which type of hospital are you employed?
University Hospital

In which type of hospital are you employed? [Other]

In which country do you work?
Deutschland

Does your department have a defined protocol for infection prophylaxis in the context of VUS/VCUG?
No

Has this protocol been transformed into a written standard operating procedure (SOP)?
N/A

Antibiotic Prophylaxis

Are there situations/constellations in which you do not use antibiotic prophylaxis for VUS/VCUG?
Yes

Please specify these situations/constellations: [girl with first uncomplicated UTI without urinary tract dilation in ultrasound]
No

Please specify these situations/constellations: [boy with first uncomplicated UTI without urinary tract dilation in ultrasound]
No

Please specify these situations/constellations: [in patients with low grade urinary tract dilation (up to 2nd grade) and no UTI in medical history]
No

Please specify these situations/constellations: [Other]
suprapubische Punktion, welche daher immer angestrebt wird

If you use antibiotic prophylaxis: which mediaction do you use? (multiple answers and subdivision into age groups possible) [Trimethoprim]
No

If you use antibiotic prophylaxis: which mediaction do you use? (multiple answers and subdivision into age groups possible) [Nitrofurantoin]
No

If you use antibiotic prophylaxis: which mediaction do you use? (multiple answers and subdivision into age groups possible) [Amoxicillin]
No

If you use antibiotic prophylaxis: which mediaction do you use? (multiple answers and subdivision into age groups possible) [Cefaclor]
No

If you use antibiotic prophylaxis: which mediaction do you use? (multiple answers and subdivision into age groups possible) [Amoxicillin/clavulanic acid]
No

If you use antibiotic prophylaxis: which mediaction do you use? (multiple answers and subdivision into age groups possible) [Other]
No

Trimethoprim: which time schedule do you use? [Day(s) before VUS/VCUG, how many?]
N/A

Trimethoprim: which time schedule do you use? [Day of VUS/VCUG]
N/A

Trimethoprim: which time schedule do you use? [Day(s) after VUS/VCUG, how many?]
N/A

Nitrofurantoin: which time schedule do you use? [Day(s) before VUS/VCUG, how many?]
N/A

Nitrofurantoin: which time schedule do you use? [Day of VUS/VCUG]
N/A

Nitrofurantoin: which time schedule do you use? [Day(s) after VUS/VCUG, how many?]
N/A

Amoxicillin: which time schedule do you use? [Day(s) before VUS/VCUG, how many?]
N/A

Amoxicillin: which time schedule do you use? [Day of VUS/VCUG]
N/A

Amoxicillin: which time schedule do you use? [Day(s) after VUS/VCUG, how many?]
N/A

Cefaclor: which time schedule do you use? [Days(s) before VUS/VCUG, how many?]
N/A

Cefaclor: which time schedule do you use? [Day of VUS/VCUG]
N/A

Cefaclor: which time schedule do you use? [Day(s) after VUS/VCUG, how many?]
N/A

Amoxicillin/clavulanic acid: in what dosage? (Amoxicillin portion) [prophylaktic (10-20 mg/Kg bodyweight/d)]
N/A

Amoxicillin/clavulanic acid: in what dosage? (Amoxicillin portion) [double prophylactic (20-40 mg/Kg bodyweight/d)]
N/A

Amoxicillin/clavulanic acid: in what dosage? (Amoxicillin portion) [therapeutic (80-100 mg/Kg bodyweight/d)]
N/A

Amoxicillin/clavulanic acid: which time schedule do you use? [Day(s) before VUS/VCUG]
N/A

Amoxicillin/clavulanic acid: which time schedule do you use? [Day of VUS/VCUG]
N/A

Amoxicillin/clavulanic acid: which time schedule do you use? [Day(s) after VUS/VCUG]
N/A

Other: which time schedule do you use? [Day(s) before VUS/VCUG, how many?]
N/A

Other: which time schedule do you use? [Day of VUS/VCUG]
N/A

Other: which time schedule do you use? [Day(s) after VUS/VCUG, how many?]
N/A

Indication for prophylaxis

Which patients receive periinterventional antibiotic prophylaxis? (multiple answers possible) [all]
N/A

Which patients receive periinterventional antibiotic prophylaxis? (multiple answers possible) [patients post urinary tract infection/pyelonephritis/urosepsis]
N/A

Which patients receive periinterventional antibiotic prophylaxis? (multiple answers possible) [patients with urinary tract dilation in ultrasound]
N/A

Which patients receive periinterventional antibiotic prophylaxis? (multiple answers possible) [patients with prenatal urinary tract dilation]
N/A

Which patients receive periinterventional antibiotic prophylaxis? (multiple answers possible) [patients post VUR surgery]
N/A

Which patients receive periinterventional antibiotic prophylaxis? (multiple answers possible) [none]
N/A

Are there situations or clinical findings in the patient where you deviate from your prophylaxis standard (medication, dose, duration, age of the patient)? (multiple answers possible, please specify in answer box) [in case of already known vesicoureteral reflux (VUR)]
N/A

Are there situations or clinical findings in the patient where you deviate from your prophylaxis standard (medication, dose, duration, age of the patient)? (multiple answers possible, please specify in answer box) [in patients with recurrent urinary tract infections (UTI)]
N/A

Are there situations or clinical findings in the patient where you deviate from your prophylaxis standard (medication, dose, duration, age of the patient)? (multiple answers possible, please specify in answer box) [if multidrug-resistant organisms (MDRO) are detected in urine cultures]
N/A

Are there situations or clinical findings in the patient where you deviate from your prophylaxis standard (medication, dose, duration, age of the patient)? (multiple answers possible, please specify in answer box) [in newborns]
N/A

Are there situations in which you use intravenous prophylaxis?
N/A

Voiding analysis

Are there differences in antibiotic prophylaxis between VUS and VCUG in your department?
N/A

Is a non-pathological urine strip test required in preparation for VUS/VCUG in your department?
N/A

Is a non-pathological urine culture required in preparation for VUS/VCUG in your department?
N/A

Do patients only receive post-procedure prophylaxis if vesicoureteral reflux (VUR) is detected in VUS/VCUG?
N/A

Do other MUS/MCU findings (e.g. urethral valves) have an influence on post-procedure prophylaxis?
N/A

End of survey

May we contact you personally if we have further questions?
N/A

Survey response 28

Response ID
29

Last page
0

Start language
de

Seed
860383566

Date started
2024-11-15 07:24:36

Date last action
2024-11-15 07:24:36

Standard Operation Procedure

Does your department have a defined protocol for infection prophylaxis in the context of VUS/VCUG?
N/A

Has this protocol been transformed into a written standard operating procedure (SOP)?
N/A

Antibiotic Prophylaxis

Are there situations/constellations in which you do not use antibiotic prophylaxis for VUS/VCUG?
N/A

Please specify these situations/constellations: [girl with first uncomplicated UTI without urinary tract dilation in ultrasound]
N/A

Please specify these situations/constellations: [boy with first uncomplicated UTI without urinary tract dilation in ultrasound]
N/A

Please specify these situations/constellations: [in patients with low grade urinary tract dilation (up to 2nd grade) and no UTI in medical history]
N/A

If you use antibiotic prophylaxis: which mediaction do you use? (multiple answers and subdivision into age groups possible) [Trimethoprim]
N/A

If you use antibiotic prophylaxis: which mediaction do you use? (multiple answers and subdivision into age groups possible) [Nitrofurantoin]
N/A

If you use antibiotic prophylaxis: which mediaction do you use? (multiple answers and subdivision into age groups possible) [Amoxicillin]
N/A

If you use antibiotic prophylaxis: which mediaction do you use? (multiple answers and subdivision into age groups possible) [Cefaclor]
N/A

If you use antibiotic prophylaxis: which mediaction do you use? (multiple answers and subdivision into age groups possible) [Amoxicillin/clavulanic acid]
N/A

If you use antibiotic prophylaxis: which mediaction do you use? (multiple answers and subdivision into age groups possible) [Other]
N/A

Trimethoprim: which time schedule do you use? [Day(s) before VUS/VCUG, how many?]
N/A

Trimethoprim: which time schedule do you use? [Day of VUS/VCUG]
N/A

Trimethoprim: which time schedule do you use? [Day(s) after VUS/VCUG, how many?]
N/A

Nitrofurantoin: which time schedule do you use? [Day(s) before VUS/VCUG, how many?]
N/A

Nitrofurantoin: which time schedule do you use? [Day of VUS/VCUG]
N/A

Nitrofurantoin: which time schedule do you use? [Day(s) after VUS/VCUG, how many?]
N/A

Amoxicillin: which time schedule do you use? [Day(s) before VUS/VCUG, how many?]
N/A

Amoxicillin: which time schedule do you use? [Day of VUS/VCUG]
N/A

Amoxicillin: which time schedule do you use? [Day(s) after VUS/VCUG, how many?]
N/A

Cefaclor: which time schedule do you use? [Days(s) before VUS/VCUG, how many?]
N/A

Cefaclor: which time schedule do you use? [Day of VUS/VCUG]
N/A

Cefaclor: which time schedule do you use? [Day(s) after VUS/VCUG, how many?]
N/A

Amoxicillin/clavulanic acid: in what dosage? (Amoxicillin portion) [prophylaktic (10-20 mg/Kg bodyweight/d)]
N/A

Amoxicillin/clavulanic acid: in what dosage? (Amoxicillin portion) [double prophylactic (20-40 mg/Kg bodyweight/d)]
N/A

Amoxicillin/clavulanic acid: in what dosage? (Amoxicillin portion) [therapeutic (80-100 mg/Kg bodyweight/d)]
N/A

Amoxicillin/clavulanic acid: which time schedule do you use? [Day(s) before VUS/VCUG]
N/A

Amoxicillin/clavulanic acid: which time schedule do you use? [Day of VUS/VCUG]
N/A

Amoxicillin/clavulanic acid: which time schedule do you use? [Day(s) after VUS/VCUG]
N/A

Other: which time schedule do you use? [Day(s) before VUS/VCUG, how many?]
N/A

Other: which time schedule do you use? [Day of VUS/VCUG]
N/A

Other: which time schedule do you use? [Day(s) after VUS/VCUG, how many?]
N/A

Indication for prophylaxis

Which patients receive periinterventional antibiotic prophylaxis? (multiple answers possible) [all]
N/A

Which patients receive periinterventional antibiotic prophylaxis? (multiple answers possible) [patients post urinary tract infection/pyelonephritis/urosepsis]
N/A

Which patients receive periinterventional antibiotic prophylaxis? (multiple answers possible) [patients with urinary tract dilation in ultrasound]
N/A

Which patients receive periinterventional antibiotic prophylaxis? (multiple answers possible) [patients with prenatal urinary tract dilation]
N/A

Which patients receive periinterventional antibiotic prophylaxis? (multiple answers possible) [patients post VUR surgery]
N/A

Which patients receive periinterventional antibiotic prophylaxis? (multiple answers possible) [none]
N/A

Are there situations or clinical findings in the patient where you deviate from your prophylaxis standard (medication, dose, duration, age of the patient)? (multiple answers possible, please specify in answer box) [in case of already known vesicoureteral reflux (VUR)]
N/A

Are there situations or clinical findings in the patient where you deviate from your prophylaxis standard (medication, dose, duration, age of the patient)? (multiple answers possible, please specify in answer box) [in patients with recurrent urinary tract infections (UTI)]
N/A

Are there situations or clinical findings in the patient where you deviate from your prophylaxis standard (medication, dose, duration, age of the patient)? (multiple answers possible, please specify in answer box) [if multidrug-resistant organisms (MDRO) are detected in urine cultures]
N/A

Are there situations or clinical findings in the patient where you deviate from your prophylaxis standard (medication, dose, duration, age of the patient)? (multiple answers possible, please specify in answer box) [in newborns]
N/A

Are there situations in which you use intravenous prophylaxis?
N/A

Voiding analysis

Are there differences in antibiotic prophylaxis between VUS and VCUG in your department?
N/A

Is a non-pathological urine strip test required in preparation for VUS/VCUG in your department?
N/A

Is a non-pathological urine culture required in preparation for VUS/VCUG in your department?
N/A

Do patients only receive post-procedure prophylaxis if vesicoureteral reflux (VUR) is detected in VUS/VCUG?
N/A

Do other MUS/MCU findings (e.g. urethral valves) have an influence on post-procedure prophylaxis?
N/A

End of survey

May we contact you personally if we have further questions?
N/A

Survey response 29

Response ID
30

Last page
1

Start language
de

Seed
1072788738

Date started
2024-11-15 08:53:20

Date last action
2024-11-15 08:53:38

Standard Operation Procedure

Who fills out this survey?
Pediatric Radiologist

Who fills out this survey? [Other]

In which type of hospital are you employed?
University Hospital

In which type of hospital are you employed? [Other]

In which country do you work?
Deutschland

Does your department have a defined protocol for infection prophylaxis in the context of VUS/VCUG?
No

Has this protocol been transformed into a written standard operating procedure (SOP)?
N/A

Antibiotic Prophylaxis

Are there situations/constellations in which you do not use antibiotic prophylaxis for VUS/VCUG?
N/A

Please specify these situations/constellations: [girl with first uncomplicated UTI without urinary tract dilation in ultrasound]
N/A

Please specify these situations/constellations: [boy with first uncomplicated UTI without urinary tract dilation in ultrasound]
N/A

Please specify these situations/constellations: [in patients with low grade urinary tract dilation (up to 2nd grade) and no UTI in medical history]
N/A

If you use antibiotic prophylaxis: which mediaction do you use? (multiple answers and subdivision into age groups possible) [Trimethoprim]
N/A

If you use antibiotic prophylaxis: which mediaction do you use? (multiple answers and subdivision into age groups possible) [Nitrofurantoin]
N/A

If you use antibiotic prophylaxis: which mediaction do you use? (multiple answers and subdivision into age groups possible) [Amoxicillin]
N/A

If you use antibiotic prophylaxis: which mediaction do you use? (multiple answers and subdivision into age groups possible) [Cefaclor]
N/A

If you use antibiotic prophylaxis: which mediaction do you use? (multiple answers and subdivision into age groups possible) [Amoxicillin/clavulanic acid]
N/A

If you use antibiotic prophylaxis: which mediaction do you use? (multiple answers and subdivision into age groups possible) [Other]
N/A

Trimethoprim: which time schedule do you use? [Day(s) before VUS/VCUG, how many?]
N/A

Trimethoprim: which time schedule do you use? [Day of VUS/VCUG]
N/A

Trimethoprim: which time schedule do you use? [Day(s) after VUS/VCUG, how many?]
N/A

Nitrofurantoin: which time schedule do you use? [Day(s) before VUS/VCUG, how many?]
N/A

Nitrofurantoin: which time schedule do you use? [Day of VUS/VCUG]
N/A

Nitrofurantoin: which time schedule do you use? [Day(s) after VUS/VCUG, how many?]
N/A

Amoxicillin: which time schedule do you use? [Day(s) before VUS/VCUG, how many?]
N/A

Amoxicillin: which time schedule do you use? [Day of VUS/VCUG]
N/A

Amoxicillin: which time schedule do you use? [Day(s) after VUS/VCUG, how many?]
N/A

Cefaclor: which time schedule do you use? [Days(s) before VUS/VCUG, how many?]
N/A

Cefaclor: which time schedule do you use? [Day of VUS/VCUG]
N/A

Cefaclor: which time schedule do you use? [Day(s) after VUS/VCUG, how many?]
N/A

Amoxicillin/clavulanic acid: in what dosage? (Amoxicillin portion) [prophylaktic (10-20 mg/Kg bodyweight/d)]
N/A

Amoxicillin/clavulanic acid: in what dosage? (Amoxicillin portion) [double prophylactic (20-40 mg/Kg bodyweight/d)]
N/A

Amoxicillin/clavulanic acid: in what dosage? (Amoxicillin portion) [therapeutic (80-100 mg/Kg bodyweight/d)]
N/A

Amoxicillin/clavulanic acid: which time schedule do you use? [Day(s) before VUS/VCUG]
N/A

Amoxicillin/clavulanic acid: which time schedule do you use? [Day of VUS/VCUG]
N/A

Amoxicillin/clavulanic acid: which time schedule do you use? [Day(s) after VUS/VCUG]
N/A

Other: which time schedule do you use? [Day(s) before VUS/VCUG, how many?]
N/A

Other: which time schedule do you use? [Day of VUS/VCUG]
N/A

Other: which time schedule do you use? [Day(s) after VUS/VCUG, how many?]
N/A

Indication for prophylaxis

Which patients receive periinterventional antibiotic prophylaxis? (multiple answers possible) [all]
N/A

Which patients receive periinterventional antibiotic prophylaxis? (multiple answers possible) [patients post urinary tract infection/pyelonephritis/urosepsis]
N/A

Which patients receive periinterventional antibiotic prophylaxis? (multiple answers possible) [patients with urinary tract dilation in ultrasound]
N/A

Which patients receive periinterventional antibiotic prophylaxis? (multiple answers possible) [patients with prenatal urinary tract dilation]
N/A

Which patients receive periinterventional antibiotic prophylaxis? (multiple answers possible) [patients post VUR surgery]
N/A

Which patients receive periinterventional antibiotic prophylaxis? (multiple answers possible) [none]
N/A

Are there situations or clinical findings in the patient where you deviate from your prophylaxis standard (medication, dose, duration, age of the patient)? (multiple answers possible, please specify in answer box) [in case of already known vesicoureteral reflux (VUR)]
N/A

Are there situations or clinical findings in the patient where you deviate from your prophylaxis standard (medication, dose, duration, age of the patient)? (multiple answers possible, please specify in answer box) [in patients with recurrent urinary tract infections (UTI)]
N/A

Are there situations or clinical findings in the patient where you deviate from your prophylaxis standard (medication, dose, duration, age of the patient)? (multiple answers possible, please specify in answer box) [if multidrug-resistant organisms (MDRO) are detected in urine cultures]
N/A

Are there situations or clinical findings in the patient where you deviate from your prophylaxis standard (medication, dose, duration, age of the patient)? (multiple answers possible, please specify in answer box) [in newborns]
N/A

Are there situations in which you use intravenous prophylaxis?
N/A

Voiding analysis

Are there differences in antibiotic prophylaxis between VUS and VCUG in your department?
N/A

Is a non-pathological urine strip test required in preparation for VUS/VCUG in your department?
N/A

Is a non-pathological urine culture required in preparation for VUS/VCUG in your department?
N/A

Do patients only receive post-procedure prophylaxis if vesicoureteral reflux (VUR) is detected in VUS/VCUG?
N/A

Do other MUS/MCU findings (e.g. urethral valves) have an influence on post-procedure prophylaxis?
N/A

End of survey

May we contact you personally if we have further questions?
N/A

Survey response 30

Response ID
31

Last page
1

Start language
de

Seed
676483592

Date started
2024-11-15 09:13:46

Date last action
2024-11-15 09:13:56

Standard Operation Procedure

Who fills out this survey?
Pediatric Radiologist

Who fills out this survey? [Other]

In which type of hospital are you employed?
University Hospital

In which type of hospital are you employed? [Other]

In which country do you work?
Deutschland

Does your department have a defined protocol for infection prophylaxis in the context of VUS/VCUG?
No

Has this protocol been transformed into a written standard operating procedure (SOP)?
N/A

Antibiotic Prophylaxis

Are there situations/constellations in which you do not use antibiotic prophylaxis for VUS/VCUG?
N/A

Please specify these situations/constellations: [girl with first uncomplicated UTI without urinary tract dilation in ultrasound]
N/A

Please specify these situations/constellations: [boy with first uncomplicated UTI without urinary tract dilation in ultrasound]
N/A

Please specify these situations/constellations: [in patients with low grade urinary tract dilation (up to 2nd grade) and no UTI in medical history]
N/A

If you use antibiotic prophylaxis: which mediaction do you use? (multiple answers and subdivision into age groups possible) [Trimethoprim]
N/A

If you use antibiotic prophylaxis: which mediaction do you use? (multiple answers and subdivision into age groups possible) [Nitrofurantoin]
N/A

If you use antibiotic prophylaxis: which mediaction do you use? (multiple answers and subdivision into age groups possible) [Amoxicillin]
N/A

If you use antibiotic prophylaxis: which mediaction do you use? (multiple answers and subdivision into age groups possible) [Cefaclor]
N/A

If you use antibiotic prophylaxis: which mediaction do you use? (multiple answers and subdivision into age groups possible) [Amoxicillin/clavulanic acid]
N/A

If you use antibiotic prophylaxis: which mediaction do you use? (multiple answers and subdivision into age groups possible) [Other]
N/A

Trimethoprim: which time schedule do you use? [Day(s) before VUS/VCUG, how many?]
N/A

Trimethoprim: which time schedule do you use? [Day of VUS/VCUG]
N/A

Trimethoprim: which time schedule do you use? [Day(s) after VUS/VCUG, how many?]
N/A

Nitrofurantoin: which time schedule do you use? [Day(s) before VUS/VCUG, how many?]
N/A

Nitrofurantoin: which time schedule do you use? [Day of VUS/VCUG]
N/A

Nitrofurantoin: which time schedule do you use? [Day(s) after VUS/VCUG, how many?]
N/A

Amoxicillin: which time schedule do you use? [Day(s) before VUS/VCUG, how many?]
N/A

Amoxicillin: which time schedule do you use? [Day of VUS/VCUG]
N/A

Amoxicillin: which time schedule do you use? [Day(s) after VUS/VCUG, how many?]
N/A

Cefaclor: which time schedule do you use? [Days(s) before VUS/VCUG, how many?]
N/A

Cefaclor: which time schedule do you use? [Day of VUS/VCUG]
N/A

Cefaclor: which time schedule do you use? [Day(s) after VUS/VCUG, how many?]
N/A

Amoxicillin/clavulanic acid: in what dosage? (Amoxicillin portion) [prophylaktic (10-20 mg/Kg bodyweight/d)]
N/A

Amoxicillin/clavulanic acid: in what dosage? (Amoxicillin portion) [double prophylactic (20-40 mg/Kg bodyweight/d)]
N/A

Amoxicillin/clavulanic acid: in what dosage? (Amoxicillin portion) [therapeutic (80-100 mg/Kg bodyweight/d)]
N/A

Amoxicillin/clavulanic acid: which time schedule do you use? [Day(s) before VUS/VCUG]
N/A

Amoxicillin/clavulanic acid: which time schedule do you use? [Day of VUS/VCUG]
N/A

Amoxicillin/clavulanic acid: which time schedule do you use? [Day(s) after VUS/VCUG]
N/A

Other: which time schedule do you use? [Day(s) before VUS/VCUG, how many?]
N/A

Other: which time schedule do you use? [Day of VUS/VCUG]
N/A

Other: which time schedule do you use? [Day(s) after VUS/VCUG, how many?]
N/A

Indication for prophylaxis

Which patients receive periinterventional antibiotic prophylaxis? (multiple answers possible) [all]
N/A

Which patients receive periinterventional antibiotic prophylaxis? (multiple answers possible) [patients post urinary tract infection/pyelonephritis/urosepsis]
N/A

Which patients receive periinterventional antibiotic prophylaxis? (multiple answers possible) [patients with urinary tract dilation in ultrasound]
N/A

Which patients receive periinterventional antibiotic prophylaxis? (multiple answers possible) [patients with prenatal urinary tract dilation]
N/A

Which patients receive periinterventional antibiotic prophylaxis? (multiple answers possible) [patients post VUR surgery]
N/A

Which patients receive periinterventional antibiotic prophylaxis? (multiple answers possible) [none]
N/A

Are there situations or clinical findings in the patient where you deviate from your prophylaxis standard (medication, dose, duration, age of the patient)? (multiple answers possible, please specify in answer box) [in case of already known vesicoureteral reflux (VUR)]
N/A

Are there situations or clinical findings in the patient where you deviate from your prophylaxis standard (medication, dose, duration, age of the patient)? (multiple answers possible, please specify in answer box) [in patients with recurrent urinary tract infections (UTI)]
N/A

Are there situations or clinical findings in the patient where you deviate from your prophylaxis standard (medication, dose, duration, age of the patient)? (multiple answers possible, please specify in answer box) [if multidrug-resistant organisms (MDRO) are detected in urine cultures]
N/A

Are there situations or clinical findings in the patient where you deviate from your prophylaxis standard (medication, dose, duration, age of the patient)? (multiple answers possible, please specify in answer box) [in newborns]
N/A

Are there situations in which you use intravenous prophylaxis?
N/A

Voiding analysis

Are there differences in antibiotic prophylaxis between VUS and VCUG in your department?
N/A

Is a non-pathological urine strip test required in preparation for VUS/VCUG in your department?
N/A

Is a non-pathological urine culture required in preparation for VUS/VCUG in your department?
N/A

Do patients only receive post-procedure prophylaxis if vesicoureteral reflux (VUR) is detected in VUS/VCUG?
N/A

Do other MUS/MCU findings (e.g. urethral valves) have an influence on post-procedure prophylaxis?
N/A

End of survey

May we contact you personally if we have further questions?
N/A

Survey response 31

Response ID
32

Last page
1

Start language
de

Seed
1594888819

Date started
2024-11-15 10:04:13

Date last action
2024-11-15 10:04:38

Standard Operation Procedure

Who fills out this survey?
Pediatric Radiologist

Who fills out this survey? [Other]

In which type of hospital are you employed?
University Hospital

In which type of hospital are you employed? [Other]

In which country do you work?
Deutschland

Does your department have a defined protocol for infection prophylaxis in the context of VUS/VCUG?
No

Has this protocol been transformed into a written standard operating procedure (SOP)?
N/A

Antibiotic Prophylaxis

Are there situations/constellations in which you do not use antibiotic prophylaxis for VUS/VCUG?
N/A

Please specify these situations/constellations: [girl with first uncomplicated UTI without urinary tract dilation in ultrasound]
N/A

Please specify these situations/constellations: [boy with first uncomplicated UTI without urinary tract dilation in ultrasound]
N/A

Please specify these situations/constellations: [in patients with low grade urinary tract dilation (up to 2nd grade) and no UTI in medical history]
N/A

If you use antibiotic prophylaxis: which mediaction do you use? (multiple answers and subdivision into age groups possible) [Trimethoprim]
N/A

If you use antibiotic prophylaxis: which mediaction do you use? (multiple answers and subdivision into age groups possible) [Nitrofurantoin]
N/A

If you use antibiotic prophylaxis: which mediaction do you use? (multiple answers and subdivision into age groups possible) [Amoxicillin]
N/A

If you use antibiotic prophylaxis: which mediaction do you use? (multiple answers and subdivision into age groups possible) [Cefaclor]
N/A

If you use antibiotic prophylaxis: which mediaction do you use? (multiple answers and subdivision into age groups possible) [Amoxicillin/clavulanic acid]
N/A

If you use antibiotic prophylaxis: which mediaction do you use? (multiple answers and subdivision into age groups possible) [Other]
N/A

Trimethoprim: which time schedule do you use? [Day(s) before VUS/VCUG, how many?]
N/A

Trimethoprim: which time schedule do you use? [Day of VUS/VCUG]
N/A

Trimethoprim: which time schedule do you use? [Day(s) after VUS/VCUG, how many?]
N/A

Nitrofurantoin: which time schedule do you use? [Day(s) before VUS/VCUG, how many?]
N/A

Nitrofurantoin: which time schedule do you use? [Day of VUS/VCUG]
N/A

Nitrofurantoin: which time schedule do you use? [Day(s) after VUS/VCUG, how many?]
N/A

Amoxicillin: which time schedule do you use? [Day(s) before VUS/VCUG, how many?]
N/A

Amoxicillin: which time schedule do you use? [Day of VUS/VCUG]
N/A

Amoxicillin: which time schedule do you use? [Day(s) after VUS/VCUG, how many?]
N/A

Cefaclor: which time schedule do you use? [Days(s) before VUS/VCUG, how many?]
N/A

Cefaclor: which time schedule do you use? [Day of VUS/VCUG]
N/A

Cefaclor: which time schedule do you use? [Day(s) after VUS/VCUG, how many?]
N/A

Amoxicillin/clavulanic acid: in what dosage? (Amoxicillin portion) [prophylaktic (10-20 mg/Kg bodyweight/d)]
N/A

Amoxicillin/clavulanic acid: in what dosage? (Amoxicillin portion) [double prophylactic (20-40 mg/Kg bodyweight/d)]
N/A

Amoxicillin/clavulanic acid: in what dosage? (Amoxicillin portion) [therapeutic (80-100 mg/Kg bodyweight/d)]
N/A

Amoxicillin/clavulanic acid: which time schedule do you use? [Day(s) before VUS/VCUG]
N/A

Amoxicillin/clavulanic acid: which time schedule do you use? [Day of VUS/VCUG]
N/A

Amoxicillin/clavulanic acid: which time schedule do you use? [Day(s) after VUS/VCUG]
N/A

Other: which time schedule do you use? [Day(s) before VUS/VCUG, how many?]
N/A

Other: which time schedule do you use? [Day of VUS/VCUG]
N/A

Other: which time schedule do you use? [Day(s) after VUS/VCUG, how many?]
N/A

Indication for prophylaxis

Which patients receive periinterventional antibiotic prophylaxis? (multiple answers possible) [all]
N/A

Which patients receive periinterventional antibiotic prophylaxis? (multiple answers possible) [patients post urinary tract infection/pyelonephritis/urosepsis]
N/A

Which patients receive periinterventional antibiotic prophylaxis? (multiple answers possible) [patients with urinary tract dilation in ultrasound]
N/A

Which patients receive periinterventional antibiotic prophylaxis? (multiple answers possible) [patients with prenatal urinary tract dilation]
N/A

Which patients receive periinterventional antibiotic prophylaxis? (multiple answers possible) [patients post VUR surgery]
N/A

Which patients receive periinterventional antibiotic prophylaxis? (multiple answers possible) [none]
N/A

Are there situations or clinical findings in the patient where you deviate from your prophylaxis standard (medication, dose, duration, age of the patient)? (multiple answers possible, please specify in answer box) [in case of already known vesicoureteral reflux (VUR)]
N/A

Are there situations or clinical findings in the patient where you deviate from your prophylaxis standard (medication, dose, duration, age of the patient)? (multiple answers possible, please specify in answer box) [in patients with recurrent urinary tract infections (UTI)]
N/A

Are there situations or clinical findings in the patient where you deviate from your prophylaxis standard (medication, dose, duration, age of the patient)? (multiple answers possible, please specify in answer box) [if multidrug-resistant organisms (MDRO) are detected in urine cultures]
N/A

Are there situations or clinical findings in the patient where you deviate from your prophylaxis standard (medication, dose, duration, age of the patient)? (multiple answers possible, please specify in answer box) [in newborns]
N/A

Are there situations in which you use intravenous prophylaxis?
N/A

Voiding analysis

Are there differences in antibiotic prophylaxis between VUS and VCUG in your department?
N/A

Is a non-pathological urine strip test required in preparation for VUS/VCUG in your department?
N/A

Is a non-pathological urine culture required in preparation for VUS/VCUG in your department?
N/A

Do patients only receive post-procedure prophylaxis if vesicoureteral reflux (VUR) is detected in VUS/VCUG?
N/A

Do other MUS/MCU findings (e.g. urethral valves) have an influence on post-procedure prophylaxis?
N/A

End of survey

May we contact you personally if we have further questions?
N/A

Survey response 32

Response ID
33

Date submitted
2024-11-15 14:56:24

Last page
5

Start language
de

Seed
989013237

Date started
2024-11-15 14:53:45

Date last action
2024-11-15 14:56:24

Standard Operation Procedure

Who fills out this survey?
Pediatric Radiologist

Who fills out this survey? [Other]

In which type of hospital are you employed?
University Hospital

In which type of hospital are you employed? [Other]

In which country do you work?
Deutschland

Does your department have a defined protocol for infection prophylaxis in the context of VUS/VCUG?
Yes

Has this protocol been transformed into a written standard operating procedure (SOP)?
Yes

Which department is the author of the SOP for infection prophylaxis in VUS/VCUG?
Pediatrics

Which department is the author of the SOP for infection prophylaxis in VUS/VCUG? [Other]

Antibiotic Prophylaxis

Are there situations/constellations in which you do not use antibiotic prophylaxis for VUS/VCUG?
Yes

Please specify these situations/constellations: [girl with first uncomplicated UTI without urinary tract dilation in ultrasound]
No

Please specify these situations/constellations: [boy with first uncomplicated UTI without urinary tract dilation in ultrasound]
No

Please specify these situations/constellations: [in patients with low grade urinary tract dilation (up to 2nd grade) and no UTI in medical history]
Yes

Please specify these situations/constellations: [Other]

If you use antibiotic prophylaxis: which mediaction do you use? (multiple answers and subdivision into age groups possible) [Trimethoprim]
Yes

If you use antibiotic prophylaxis: which mediaction do you use? (multiple answers and subdivision into age groups possible) [Nitrofurantoin]
No

If you use antibiotic prophylaxis: which mediaction do you use? (multiple answers and subdivision into age groups possible) [Amoxicillin]
No

If you use antibiotic prophylaxis: which mediaction do you use? (multiple answers and subdivision into age groups possible) [Cefaclor]
No

If you use antibiotic prophylaxis: which mediaction do you use? (multiple answers and subdivision into age groups possible) [Amoxicillin/clavulanic acid]
No

If you use antibiotic prophylaxis: which mediaction do you use? (multiple answers and subdivision into age groups possible) [Other]
No

Trimethoprim: in what dosage?
prophylactic (2 mg/kg bodyweight/d)

Trimethoprim: in what dosage? [Other]

Trimethoprim: for which age group do you use trimethoprim?
NG SS KK

Trimethoprim: which time schedule do you use? [Day(s) before VUS/VCUG, how many?]
No

Trimethoprim: which time schedule do you use? [Day(s) before VUS/VCUG, how many?][Comment]

Trimethoprim: which time schedule do you use? [Day of VUS/VCUG]
Yes

Trimethoprim: which time schedule do you use? [Day of VUS/VCUG][Comment]

Trimethoprim: which time schedule do you use? [Day(s) after VUS/VCUG, how many?]
No

Trimethoprim: which time schedule do you use? [Day(s) after VUS/VCUG, how many?][Comment]

Nitrofurantoin: which time schedule do you use? [Day(s) before VUS/VCUG, how many?]
N/A

Nitrofurantoin: which time schedule do you use? [Day of VUS/VCUG]
N/A

Nitrofurantoin: which time schedule do you use? [Day(s) after VUS/VCUG, how many?]
N/A

Amoxicillin: which time schedule do you use? [Day(s) before VUS/VCUG, how many?]
N/A

Amoxicillin: which time schedule do you use? [Day of VUS/VCUG]
N/A

Amoxicillin: which time schedule do you use? [Day(s) after VUS/VCUG, how many?]
N/A

Cefaclor: which time schedule do you use? [Days(s) before VUS/VCUG, how many?]
N/A

Cefaclor: which time schedule do you use? [Day of VUS/VCUG]
N/A

Cefaclor: which time schedule do you use? [Day(s) after VUS/VCUG, how many?]
N/A

Amoxicillin/clavulanic acid: in what dosage? (Amoxicillin portion) [prophylaktic (10-20 mg/Kg bodyweight/d)]
N/A

Amoxicillin/clavulanic acid: in what dosage? (Amoxicillin portion) [double prophylactic (20-40 mg/Kg bodyweight/d)]
N/A

Amoxicillin/clavulanic acid: in what dosage? (Amoxicillin portion) [therapeutic (80-100 mg/Kg bodyweight/d)]
N/A

Amoxicillin/clavulanic acid: which time schedule do you use? [Day(s) before VUS/VCUG]
N/A

Amoxicillin/clavulanic acid: which time schedule do you use? [Day of VUS/VCUG]
N/A

Amoxicillin/clavulanic acid: which time schedule do you use? [Day(s) after VUS/VCUG]
N/A

Other: which time schedule do you use? [Day(s) before VUS/VCUG, how many?]
N/A

Other: which time schedule do you use? [Day of VUS/VCUG]
N/A

Other: which time schedule do you use? [Day(s) after VUS/VCUG, how many?]
N/A

Indication for prophylaxis

Which patients receive periinterventional antibiotic prophylaxis? (multiple answers possible) [all]
No

Which patients receive periinterventional antibiotic prophylaxis? (multiple answers possible) [patients post urinary tract infection/pyelonephritis/urosepsis]
Yes

Which patients receive periinterventional antibiotic prophylaxis? (multiple answers possible) [patients with urinary tract dilation in ultrasound]
No

Which patients receive periinterventional antibiotic prophylaxis? (multiple answers possible) [patients with prenatal urinary tract dilation]
Yes

Which patients receive periinterventional antibiotic prophylaxis? (multiple answers possible) [patients post VUR surgery]
No

Which patients receive periinterventional antibiotic prophylaxis? (multiple answers possible) [none]
No

Which patients receive periinterventional antibiotic prophylaxis? (multiple answers possible) [Other]

patients status post urinary tract infection/pyelonephritis/urosepsis: what is the minimum time between infection and VUS/VCUG? (in days)
1

In patients with prenatal urinary tract dilation: from what degree on? Image from Beetz R, Bokenkamp A, Brandis M et al (2001) Diagnosis of congenital dilatation of the urinary tract. Consensus Group of the Pediatric Nephrology Working Society in cooperation with the Pediatric Urology Working Group of the German Society of Urology and with the Pediatric Urology Working Society in the Germany Society of Pediatric Surgery. Urologe A 40:495–507 (quiz 8–9)
grade 3

Are there situations or clinical findings in the patient where you deviate from your prophylaxis standard (medication, dose, duration, age of the patient)? (multiple answers possible, please specify in answer box) [in case of already known vesicoureteral reflux (VUR)]
Yes

Are there situations or clinical findings in the patient where you deviate from your prophylaxis standard (medication, dose, duration, age of the patient)? (multiple answers possible, please specify in answer box) [in case of already known vesicoureteral reflux (VUR)][Comment]

Are there situations or clinical findings in the patient where you deviate from your prophylaxis standard (medication, dose, duration, age of the patient)? (multiple answers possible, please specify in answer box) [in patients with recurrent urinary tract infections (UTI)]
Yes

Are there situations or clinical findings in the patient where you deviate from your prophylaxis standard (medication, dose, duration, age of the patient)? (multiple answers possible, please specify in answer box) [in patients with recurrent urinary tract infections (UTI)][Comment]

Are there situations or clinical findings in the patient where you deviate from your prophylaxis standard (medication, dose, duration, age of the patient)? (multiple answers possible, please specify in answer box) [if multidrug-resistant organisms (MDRO) are detected in urine cultures]
Yes

Are there situations or clinical findings in the patient where you deviate from your prophylaxis standard (medication, dose, duration, age of the patient)? (multiple answers possible, please specify in answer box) [if multidrug-resistant organisms (MDRO) are detected in urine cultures][Comment]

Are there situations or clinical findings in the patient where you deviate from your prophylaxis standard (medication, dose, duration, age of the patient)? (multiple answers possible, please specify in answer box) [in newborns]
No

Are there situations or clinical findings in the patient where you deviate from your prophylaxis standard (medication, dose, duration, age of the patient)? (multiple answers possible, please specify in answer box) [in newborns][Comment]

Are there situations or clinical findings in the patient where you deviate from your prophylaxis standard (medication, dose, duration, age of the patient)? (multiple answers possible, please specify in answer box) [Other]

Are there situations or clinical findings in the patient where you deviate from your prophylaxis standard (medication, dose, duration, age of the patient)? (multiple answers possible, please specify in answer box) [Other comment]

Are there situations in which you use intravenous prophylaxis?
No

Voiding analysis

Are there differences in antibiotic prophylaxis between VUS and VCUG in your department?
No

Is a non-pathological urine strip test required in preparation for VUS/VCUG in your department?
Yes

What is the maximum amount of days accepted for urine strip test to be performed prior to the exam?
0

Is a non-pathological urine culture required in preparation for VUS/VCUG in your department?
No

Do patients only receive post-procedure prophylaxis if vesicoureteral reflux (VUR) is detected in VUS/VCUG?
No

Do other MUS/MCU findings (e.g. urethral valves) have an influence on post-procedure prophylaxis?
No

End of survey

Thank you for taking the time to answer this survey!

May we contact you personally if we have further questions?
No

Survey response 33

Response ID
34

Date submitted
2024-11-15 15:00:18

Last page
5

Start language
de

Seed
1596762644

Date started
2024-11-15 14:58:02

Date last action
2024-11-15 15:00:18

Standard Operation Procedure

Who fills out this survey?
Pediatric Radiologist

Who fills out this survey? [Other]

In which type of hospital are you employed?
Community hospital (

In which type of hospital are you employed? [Other]

In which country do you work?
Deutschland

Does your department have a defined protocol for infection prophylaxis in the context of VUS/VCUG?
No

Has this protocol been transformed into a written standard operating procedure (SOP)?
N/A

Antibiotic Prophylaxis

Are there situations/constellations in which you do not use antibiotic prophylaxis for VUS/VCUG?
No

Please specify these situations/constellations: [girl with first uncomplicated UTI without urinary tract dilation in ultrasound]
N/A

Please specify these situations/constellations: [boy with first uncomplicated UTI without urinary tract dilation in ultrasound]
N/A

Please specify these situations/constellations: [in patients with low grade urinary tract dilation (up to 2nd grade) and no UTI in medical history]
N/A

If you use antibiotic prophylaxis: which mediaction do you use? (multiple answers and subdivision into age groups possible) [Trimethoprim]
No

If you use antibiotic prophylaxis: which mediaction do you use? (multiple answers and subdivision into age groups possible) [Nitrofurantoin]
No

If you use antibiotic prophylaxis: which mediaction do you use? (multiple answers and subdivision into age groups possible) [Amoxicillin]
No

If you use antibiotic prophylaxis: which mediaction do you use? (multiple answers and subdivision into age groups possible) [Cefaclor]
No

If you use antibiotic prophylaxis: which mediaction do you use? (multiple answers and subdivision into age groups possible) [Amoxicillin/clavulanic acid]
No

If you use antibiotic prophylaxis: which mediaction do you use? (multiple answers and subdivision into age groups possible) [Other]
No

Trimethoprim: which time schedule do you use? [Day(s) before VUS/VCUG, how many?]
N/A

Trimethoprim: which time schedule do you use? [Day of VUS/VCUG]
N/A

Trimethoprim: which time schedule do you use? [Day(s) after VUS/VCUG, how many?]
N/A

Nitrofurantoin: which time schedule do you use? [Day(s) before VUS/VCUG, how many?]
N/A

Nitrofurantoin: which time schedule do you use? [Day of VUS/VCUG]
N/A

Nitrofurantoin: which time schedule do you use? [Day(s) after VUS/VCUG, how many?]
N/A

Amoxicillin: which time schedule do you use? [Day(s) before VUS/VCUG, how many?]
N/A

Amoxicillin: which time schedule do you use? [Day of VUS/VCUG]
N/A

Amoxicillin: which time schedule do you use? [Day(s) after VUS/VCUG, how many?]
N/A

Cefaclor: which time schedule do you use? [Days(s) before VUS/VCUG, how many?]
N/A

Cefaclor: which time schedule do you use? [Day of VUS/VCUG]
N/A

Cefaclor: which time schedule do you use? [Day(s) after VUS/VCUG, how many?]
N/A

Amoxicillin/clavulanic acid: in what dosage? (Amoxicillin portion) [prophylaktic (10-20 mg/Kg bodyweight/d)]
N/A

Amoxicillin/clavulanic acid: in what dosage? (Amoxicillin portion) [double prophylactic (20-40 mg/Kg bodyweight/d)]
N/A

Amoxicillin/clavulanic acid: in what dosage? (Amoxicillin portion) [therapeutic (80-100 mg/Kg bodyweight/d)]
N/A

Amoxicillin/clavulanic acid: which time schedule do you use? [Day(s) before VUS/VCUG]
N/A

Amoxicillin/clavulanic acid: which time schedule do you use? [Day of VUS/VCUG]
N/A

Amoxicillin/clavulanic acid: which time schedule do you use? [Day(s) after VUS/VCUG]
N/A

Other: which time schedule do you use? [Day(s) before VUS/VCUG, how many?]
N/A

Other: which time schedule do you use? [Day of VUS/VCUG]
N/A

Other: which time schedule do you use? [Day(s) after VUS/VCUG, how many?]
N/A

Indication for prophylaxis

Which patients receive periinterventional antibiotic prophylaxis? (multiple answers possible) [all]
No

Which patients receive periinterventional antibiotic prophylaxis? (multiple answers possible) [patients post urinary tract infection/pyelonephritis/urosepsis]
No

Which patients receive periinterventional antibiotic prophylaxis? (multiple answers possible) [patients with urinary tract dilation in ultrasound]
No

Which patients receive periinterventional antibiotic prophylaxis? (multiple answers possible) [patients with prenatal urinary tract dilation]
No

Which patients receive periinterventional antibiotic prophylaxis? (multiple answers possible) [patients post VUR surgery]
No

Which patients receive periinterventional antibiotic prophylaxis? (multiple answers possible) [none]
No

Which patients receive periinterventional antibiotic prophylaxis? (multiple answers possible) [Other]
läuft über Padiatrie

Are there situations or clinical findings in the patient where you deviate from your prophylaxis standard (medication, dose, duration, age of the patient)? (multiple answers possible, please specify in answer box) [in case of already known vesicoureteral reflux (VUR)]
No

Are there situations or clinical findings in the patient where you deviate from your prophylaxis standard (medication, dose, duration, age of the patient)? (multiple answers possible, please specify in answer box) [in case of already known vesicoureteral reflux (VUR)][Comment]

Are there situations or clinical findings in the patient where you deviate from your prophylaxis standard (medication, dose, duration, age of the patient)? (multiple answers possible, please specify in answer box) [in patients with recurrent urinary tract infections (UTI)]
No

Are there situations or clinical findings in the patient where you deviate from your prophylaxis standard (medication, dose, duration, age of the patient)? (multiple answers possible, please specify in answer box) [in patients with recurrent urinary tract infections (UTI)][Comment]

Are there situations or clinical findings in the patient where you deviate from your prophylaxis standard (medication, dose, duration, age of the patient)? (multiple answers possible, please specify in answer box) [if multidrug-resistant organisms (MDRO) are detected in urine cultures]
No

Are there situations or clinical findings in the patient where you deviate from your prophylaxis standard (medication, dose, duration, age of the patient)? (multiple answers possible, please specify in answer box) [if multidrug-resistant organisms (MDRO) are detected in urine cultures][Comment]

Are there situations or clinical findings in the patient where you deviate from your prophylaxis standard (medication, dose, duration, age of the patient)? (multiple answers possible, please specify in answer box) [in newborns]
No

Are there situations or clinical findings in the patient where you deviate from your prophylaxis standard (medication, dose, duration, age of the patient)? (multiple answers possible, please specify in answer box) [in newborns][Comment]

Are there situations or clinical findings in the patient where you deviate from your prophylaxis standard (medication, dose, duration, age of the patient)? (multiple answers possible, please specify in answer box) [Other]
so

Are there situations or clinical findings in the patient where you deviate from your prophylaxis standard (medication, dose, duration, age of the patient)? (multiple answers possible, please specify in answer box) [Other comment]

Are there situations in which you use intravenous prophylaxis?
No

Voiding analysis

Are there differences in antibiotic prophylaxis between VUS and VCUG in your department?
No

Is a non-pathological urine strip test required in preparation for VUS/VCUG in your department?
No

Is a non-pathological urine culture required in preparation for VUS/VCUG in your department?
No

Do patients only receive post-procedure prophylaxis if vesicoureteral reflux (VUR) is detected in VUS/VCUG?
No

Do other MUS/MCU findings (e.g. urethral valves) have an influence on post-procedure prophylaxis?
No

End of survey

Thank you for taking the time to answer this survey!

May we contact you personally if we have further questions?
No

Survey response 34

Response ID
35

Last page
1

Start language
de

Seed
362342137

Date started
2024-11-15 15:16:54

Date last action
2024-11-15 15:23:06

Standard Operation Procedure

Who fills out this survey?
Pediatric Radiologist

Who fills out this survey? [Other]

In which type of hospital are you employed?
University Hospital

In which type of hospital are you employed? [Other]

In which country do you work?
Deutschland

Does your department have a defined protocol for infection prophylaxis in the context of VUS/VCUG?
No

Has this protocol been transformed into a written standard operating procedure (SOP)?
N/A

Antibiotic Prophylaxis

Are there situations/constellations in which you do not use antibiotic prophylaxis for VUS/VCUG?
N/A

Please specify these situations/constellations: [girl with first uncomplicated UTI without urinary tract dilation in ultrasound]
N/A

Please specify these situations/constellations: [boy with first uncomplicated UTI without urinary tract dilation in ultrasound]
N/A

Please specify these situations/constellations: [in patients with low grade urinary tract dilation (up to 2nd grade) and no UTI in medical history]
N/A

If you use antibiotic prophylaxis: which mediaction do you use? (multiple answers and subdivision into age groups possible) [Trimethoprim]
N/A

If you use antibiotic prophylaxis: which mediaction do you use? (multiple answers and subdivision into age groups possible) [Nitrofurantoin]
N/A

If you use antibiotic prophylaxis: which mediaction do you use? (multiple answers and subdivision into age groups possible) [Amoxicillin]
N/A

If you use antibiotic prophylaxis: which mediaction do you use? (multiple answers and subdivision into age groups possible) [Cefaclor]
N/A

If you use antibiotic prophylaxis: which mediaction do you use? (multiple answers and subdivision into age groups possible) [Amoxicillin/clavulanic acid]
N/A

If you use antibiotic prophylaxis: which mediaction do you use? (multiple answers and subdivision into age groups possible) [Other]
N/A

Trimethoprim: which time schedule do you use? [Day(s) before VUS/VCUG, how many?]
N/A

Trimethoprim: which time schedule do you use? [Day of VUS/VCUG]
N/A

Trimethoprim: which time schedule do you use? [Day(s) after VUS/VCUG, how many?]
N/A

Nitrofurantoin: which time schedule do you use? [Day(s) before VUS/VCUG, how many?]
N/A

Nitrofurantoin: which time schedule do you use? [Day of VUS/VCUG]
N/A

Nitrofurantoin: which time schedule do you use? [Day(s) after VUS/VCUG, how many?]
N/A

Amoxicillin: which time schedule do you use? [Day(s) before VUS/VCUG, how many?]
N/A

Amoxicillin: which time schedule do you use? [Day of VUS/VCUG]
N/A

Amoxicillin: which time schedule do you use? [Day(s) after VUS/VCUG, how many?]
N/A

Cefaclor: which time schedule do you use? [Days(s) before VUS/VCUG, how many?]
N/A

Cefaclor: which time schedule do you use? [Day of VUS/VCUG]
N/A

Cefaclor: which time schedule do you use? [Day(s) after VUS/VCUG, how many?]
N/A

Amoxicillin/clavulanic acid: in what dosage? (Amoxicillin portion) [prophylaktic (10-20 mg/Kg bodyweight/d)]
N/A

Amoxicillin/clavulanic acid: in what dosage? (Amoxicillin portion) [double prophylactic (20-40 mg/Kg bodyweight/d)]
N/A

Amoxicillin/clavulanic acid: in what dosage? (Amoxicillin portion) [therapeutic (80-100 mg/Kg bodyweight/d)]
N/A

Amoxicillin/clavulanic acid: which time schedule do you use? [Day(s) before VUS/VCUG]
N/A

Amoxicillin/clavulanic acid: which time schedule do you use? [Day of VUS/VCUG]
N/A

Amoxicillin/clavulanic acid: which time schedule do you use? [Day(s) after VUS/VCUG]
N/A

Other: which time schedule do you use? [Day(s) before VUS/VCUG, how many?]
N/A

Other: which time schedule do you use? [Day of VUS/VCUG]
N/A

Other: which time schedule do you use? [Day(s) after VUS/VCUG, how many?]
N/A

Indication for prophylaxis

Which patients receive periinterventional antibiotic prophylaxis? (multiple answers possible) [all]
N/A

Which patients receive periinterventional antibiotic prophylaxis? (multiple answers possible) [patients post urinary tract infection/pyelonephritis/urosepsis]
N/A

Which patients receive periinterventional antibiotic prophylaxis? (multiple answers possible) [patients with urinary tract dilation in ultrasound]
N/A

Which patients receive periinterventional antibiotic prophylaxis? (multiple answers possible) [patients with prenatal urinary tract dilation]
N/A

Which patients receive periinterventional antibiotic prophylaxis? (multiple answers possible) [patients post VUR surgery]
N/A

Which patients receive periinterventional antibiotic prophylaxis? (multiple answers possible) [none]
N/A

Are there situations or clinical findings in the patient where you deviate from your prophylaxis standard (medication, dose, duration, age of the patient)? (multiple answers possible, please specify in answer box) [in case of already known vesicoureteral reflux (VUR)]
N/A

Are there situations or clinical findings in the patient where you deviate from your prophylaxis standard (medication, dose, duration, age of the patient)? (multiple answers possible, please specify in answer box) [in patients with recurrent urinary tract infections (UTI)]
N/A

Are there situations or clinical findings in the patient where you deviate from your prophylaxis standard (medication, dose, duration, age of the patient)? (multiple answers possible, please specify in answer box) [if multidrug-resistant organisms (MDRO) are detected in urine cultures]
N/A

Are there situations or clinical findings in the patient where you deviate from your prophylaxis standard (medication, dose, duration, age of the patient)? (multiple answers possible, please specify in answer box) [in newborns]
N/A

Are there situations in which you use intravenous prophylaxis?
N/A

Voiding analysis

Are there differences in antibiotic prophylaxis between VUS and VCUG in your department?
N/A

Is a non-pathological urine strip test required in preparation for VUS/VCUG in your department?
N/A

Is a non-pathological urine culture required in preparation for VUS/VCUG in your department?
N/A

Do patients only receive post-procedure prophylaxis if vesicoureteral reflux (VUR) is detected in VUS/VCUG?
N/A

Do other MUS/MCU findings (e.g. urethral valves) have an influence on post-procedure prophylaxis?
N/A

End of survey

May we contact you personally if we have further questions?
N/A

Survey response 35

Response ID
36

Last page
1

Start language
de

Seed
191006306

Date started
2024-11-15 15:23:51

Date last action
2024-11-15 15:24:06

Standard Operation Procedure

Who fills out this survey?
Pediatric Radiologist

Who fills out this survey? [Other]

In which type of hospital are you employed?
University Hospital

In which type of hospital are you employed? [Other]

In which country do you work?
Deutschland

Does your department have a defined protocol for infection prophylaxis in the context of VUS/VCUG?
Yes

Has this protocol been transformed into a written standard operating procedure (SOP)?
No

Antibiotic Prophylaxis

Are there situations/constellations in which you do not use antibiotic prophylaxis for VUS/VCUG?
N/A

Please specify these situations/constellations: [girl with first uncomplicated UTI without urinary tract dilation in ultrasound]
N/A

Please specify these situations/constellations: [boy with first uncomplicated UTI without urinary tract dilation in ultrasound]
N/A

Please specify these situations/constellations: [in patients with low grade urinary tract dilation (up to 2nd grade) and no UTI in medical history]
N/A

If you use antibiotic prophylaxis: which mediaction do you use? (multiple answers and subdivision into age groups possible) [Trimethoprim]
N/A

If you use antibiotic prophylaxis: which mediaction do you use? (multiple answers and subdivision into age groups possible) [Nitrofurantoin]
N/A

If you use antibiotic prophylaxis: which mediaction do you use? (multiple answers and subdivision into age groups possible) [Amoxicillin]
N/A

If you use antibiotic prophylaxis: which mediaction do you use? (multiple answers and subdivision into age groups possible) [Cefaclor]
N/A

If you use antibiotic prophylaxis: which mediaction do you use? (multiple answers and subdivision into age groups possible) [Amoxicillin/clavulanic acid]
N/A

If you use antibiotic prophylaxis: which mediaction do you use? (multiple answers and subdivision into age groups possible) [Other]
N/A

Trimethoprim: which time schedule do you use? [Day(s) before VUS/VCUG, how many?]
N/A

Trimethoprim: which time schedule do you use? [Day of VUS/VCUG]
N/A

Trimethoprim: which time schedule do you use? [Day(s) after VUS/VCUG, how many?]
N/A

Nitrofurantoin: which time schedule do you use? [Day(s) before VUS/VCUG, how many?]
N/A

Nitrofurantoin: which time schedule do you use? [Day of VUS/VCUG]
N/A

Nitrofurantoin: which time schedule do you use? [Day(s) after VUS/VCUG, how many?]
N/A

Amoxicillin: which time schedule do you use? [Day(s) before VUS/VCUG, how many?]
N/A

Amoxicillin: which time schedule do you use? [Day of VUS/VCUG]
N/A

Amoxicillin: which time schedule do you use? [Day(s) after VUS/VCUG, how many?]
N/A

Cefaclor: which time schedule do you use? [Days(s) before VUS/VCUG, how many?]
N/A

Cefaclor: which time schedule do you use? [Day of VUS/VCUG]
N/A

Cefaclor: which time schedule do you use? [Day(s) after VUS/VCUG, how many?]
N/A

Amoxicillin/clavulanic acid: in what dosage? (Amoxicillin portion) [prophylaktic (10-20 mg/Kg bodyweight/d)]
N/A

Amoxicillin/clavulanic acid: in what dosage? (Amoxicillin portion) [double prophylactic (20-40 mg/Kg bodyweight/d)]
N/A

Amoxicillin/clavulanic acid: in what dosage? (Amoxicillin portion) [therapeutic (80-100 mg/Kg bodyweight/d)]
N/A

Amoxicillin/clavulanic acid: which time schedule do you use? [Day(s) before VUS/VCUG]
N/A

Amoxicillin/clavulanic acid: which time schedule do you use? [Day of VUS/VCUG]
N/A

Amoxicillin/clavulanic acid: which time schedule do you use? [Day(s) after VUS/VCUG]
N/A

Other: which time schedule do you use? [Day(s) before VUS/VCUG, how many?]
N/A

Other: which time schedule do you use? [Day of VUS/VCUG]
N/A

Other: which time schedule do you use? [Day(s) after VUS/VCUG, how many?]
N/A

Indication for prophylaxis

Which patients receive periinterventional antibiotic prophylaxis? (multiple answers possible) [all]
N/A

Which patients receive periinterventional antibiotic prophylaxis? (multiple answers possible) [patients post urinary tract infection/pyelonephritis/urosepsis]
N/A

Which patients receive periinterventional antibiotic prophylaxis? (multiple answers possible) [patients with urinary tract dilation in ultrasound]
N/A

Which patients receive periinterventional antibiotic prophylaxis? (multiple answers possible) [patients with prenatal urinary tract dilation]
N/A

Which patients receive periinterventional antibiotic prophylaxis? (multiple answers possible) [patients post VUR surgery]
N/A

Which patients receive periinterventional antibiotic prophylaxis? (multiple answers possible) [none]
N/A

Are there situations or clinical findings in the patient where you deviate from your prophylaxis standard (medication, dose, duration, age of the patient)? (multiple answers possible, please specify in answer box) [in case of already known vesicoureteral reflux (VUR)]
N/A

Are there situations or clinical findings in the patient where you deviate from your prophylaxis standard (medication, dose, duration, age of the patient)? (multiple answers possible, please specify in answer box) [in patients with recurrent urinary tract infections (UTI)]
N/A

Are there situations or clinical findings in the patient where you deviate from your prophylaxis standard (medication, dose, duration, age of the patient)? (multiple answers possible, please specify in answer box) [if multidrug-resistant organisms (MDRO) are detected in urine cultures]
N/A

Are there situations or clinical findings in the patient where you deviate from your prophylaxis standard (medication, dose, duration, age of the patient)? (multiple answers possible, please specify in answer box) [in newborns]
N/A

Are there situations in which you use intravenous prophylaxis?
N/A

Voiding analysis

Are there differences in antibiotic prophylaxis between VUS and VCUG in your department?
N/A

Is a non-pathological urine strip test required in preparation for VUS/VCUG in your department?
N/A

Is a non-pathological urine culture required in preparation for VUS/VCUG in your department?
N/A

Do patients only receive post-procedure prophylaxis if vesicoureteral reflux (VUR) is detected in VUS/VCUG?
N/A

Do other MUS/MCU findings (e.g. urethral valves) have an influence on post-procedure prophylaxis?
N/A

End of survey

May we contact you personally if we have further questions?
N/A

Survey response 36

Response ID
37

Last page
4

Start language
de

Seed
2109640074

Date started
2024-11-15 15:28:23

Date last action
2024-11-15 15:34:49

Standard Operation Procedure

Who fills out this survey?
Pediatric Radiologist

Who fills out this survey? [Other]

In which type of hospital are you employed?
University Hospital

In which type of hospital are you employed? [Other]

In which country do you work?
Deutschland

Does your department have a defined protocol for infection prophylaxis in the context of VUS/VCUG?
Yes

Has this protocol been transformed into a written standard operating procedure (SOP)?
Yes

Which department is the author of the SOP for infection prophylaxis in VUS/VCUG?
Pediatrics

Which department is the author of the SOP for infection prophylaxis in VUS/VCUG? [Other]

Antibiotic Prophylaxis

Are there situations/constellations in which you do not use antibiotic prophylaxis for VUS/VCUG?
Yes

Please specify these situations/constellations: [girl with first uncomplicated UTI without urinary tract dilation in ultrasound]
No

Please specify these situations/constellations: [boy with first uncomplicated UTI without urinary tract dilation in ultrasound]
No

Please specify these situations/constellations: [in patients with low grade urinary tract dilation (up to 2nd grade) and no UTI in medical history]
Yes

Please specify these situations/constellations: [Other]

If you use antibiotic prophylaxis: which mediaction do you use? (multiple answers and subdivision into age groups possible) [Trimethoprim]
Yes

If you use antibiotic prophylaxis: which mediaction do you use? (multiple answers and subdivision into age groups possible) [Nitrofurantoin]
No

If you use antibiotic prophylaxis: which mediaction do you use? (multiple answers and subdivision into age groups possible) [Amoxicillin]
No

If you use antibiotic prophylaxis: which mediaction do you use? (multiple answers and subdivision into age groups possible) [Cefaclor]
Yes

If you use antibiotic prophylaxis: which mediaction do you use? (multiple answers and subdivision into age groups possible) [Amoxicillin/clavulanic acid]
No

If you use antibiotic prophylaxis: which mediaction do you use? (multiple answers and subdivision into age groups possible) [Other]
No

Trimethoprim: in what dosage?
prophylactic (2 mg/kg bodyweight/d)

Trimethoprim: in what dosage? [Other]

Trimethoprim: for which age group do you use trimethoprim?
> 6. LWo

Trimethoprim: which time schedule do you use? [Day(s) before VUS/VCUG, how many?]
Yes

Trimethoprim: which time schedule do you use? [Day(s) before VUS/VCUG, how many?][Comment]
bis zur MCU

Trimethoprim: which time schedule do you use? [Day of VUS/VCUG]
Yes

Trimethoprim: which time schedule do you use? [Day of VUS/VCUG][Comment]

Trimethoprim: which time schedule do you use? [Day(s) after VUS/VCUG, how many?]
Yes

Trimethoprim: which time schedule do you use? [Day(s) after VUS/VCUG, how many?][Comment]
1

Nitrofurantoin: which time schedule do you use? [Day(s) before VUS/VCUG, how many?]
N/A

Nitrofurantoin: which time schedule do you use? [Day of VUS/VCUG]
N/A

Nitrofurantoin: which time schedule do you use? [Day(s) after VUS/VCUG, how many?]
N/A

Amoxicillin: which time schedule do you use? [Day(s) before VUS/VCUG, how many?]
N/A

Amoxicillin: which time schedule do you use? [Day of VUS/VCUG]
N/A

Amoxicillin: which time schedule do you use? [Day(s) after VUS/VCUG, how many?]
N/A

Cefaclor: in what dosage?
prophylactic (10 mg/kg bodyweight/d)

Cefaclor: in what dosage? [Other]

Cefaclor: for which age group do you use cefaclor?
< 6. LWo

Cefaclor: which time schedule do you use? [Days(s) before VUS/VCUG, how many?]
Yes

Cefaclor: which time schedule do you use? [Days(s) before VUS/VCUG, how many?][Comment]
bis zur MCU

Cefaclor: which time schedule do you use? [Day of VUS/VCUG]
Yes

Cefaclor: which time schedule do you use? [Day of VUS/VCUG][Comment]

Cefaclor: which time schedule do you use? [Day(s) after VUS/VCUG, how many?]
Yes

Cefaclor: which time schedule do you use? [Day(s) after VUS/VCUG, how many?][Comment]
1

Amoxicillin/clavulanic acid: in what dosage? (Amoxicillin portion) [prophylaktic (10-20 mg/Kg bodyweight/d)]
N/A

Amoxicillin/clavulanic acid: in what dosage? (Amoxicillin portion) [double prophylactic (20-40 mg/Kg bodyweight/d)]
N/A

Amoxicillin/clavulanic acid: in what dosage? (Amoxicillin portion) [therapeutic (80-100 mg/Kg bodyweight/d)]
N/A

Amoxicillin/clavulanic acid: which time schedule do you use? [Day(s) before VUS/VCUG]
N/A

Amoxicillin/clavulanic acid: which time schedule do you use? [Day of VUS/VCUG]
N/A

Amoxicillin/clavulanic acid: which time schedule do you use? [Day(s) after VUS/VCUG]
N/A

Other: which time schedule do you use? [Day(s) before VUS/VCUG, how many?]
N/A

Other: which time schedule do you use? [Day of VUS/VCUG]
N/A

Other: which time schedule do you use? [Day(s) after VUS/VCUG, how many?]
N/A

Indication for prophylaxis

Which patients receive periinterventional antibiotic prophylaxis? (multiple answers possible) [all]
No

Which patients receive periinterventional antibiotic prophylaxis? (multiple answers possible) [patients post urinary tract infection/pyelonephritis/urosepsis]
Yes

Which patients receive periinterventional antibiotic prophylaxis? (multiple answers possible) [patients with urinary tract dilation in ultrasound]
No

Which patients receive periinterventional antibiotic prophylaxis? (multiple answers possible) [patients with prenatal urinary tract dilation]
No

Which patients receive periinterventional antibiotic prophylaxis? (multiple answers possible) [patients post VUR surgery]
No

Which patients receive periinterventional antibiotic prophylaxis? (multiple answers possible) [none]
No

Which patients receive periinterventional antibiotic prophylaxis? (multiple answers possible) [Other]

patients status post urinary tract infection/pyelonephritis/urosepsis: what is the minimum time between infection and VUS/VCUG? (in days)
7

Are there situations or clinical findings in the patient where you deviate from your prophylaxis standard (medication, dose, duration, age of the patient)? (multiple answers possible, please specify in answer box) [in case of already known vesicoureteral reflux (VUR)]
No

Are there situations or clinical findings in the patient where you deviate from your prophylaxis standard (medication, dose, duration, age of the patient)? (multiple answers possible, please specify in answer box) [in case of already known vesicoureteral reflux (VUR)][Comment]

Are there situations or clinical findings in the patient where you deviate from your prophylaxis standard (medication, dose, duration, age of the patient)? (multiple answers possible, please specify in answer box) [in patients with recurrent urinary tract infections (UTI)]
No

Are there situations or clinical findings in the patient where you deviate from your prophylaxis standard (medication, dose, duration, age of the patient)? (multiple answers possible, please specify in answer box) [in patients with recurrent urinary tract infections (UTI)][Comment]

Are there situations or clinical findings in the patient where you deviate from your prophylaxis standard (medication, dose, duration, age of the patient)? (multiple answers possible, please specify in answer box) [if multidrug-resistant organisms (MDRO) are detected in urine cultures]
No

Are there situations or clinical findings in the patient where you deviate from your prophylaxis standard (medication, dose, duration, age of the patient)? (multiple answers possible, please specify in answer box) [if multidrug-resistant organisms (MDRO) are detected in urine cultures][Comment]

Are there situations or clinical findings in the patient where you deviate from your prophylaxis standard (medication, dose, duration, age of the patient)? (multiple answers possible, please specify in answer box) [in newborns]
No

Are there situations or clinical findings in the patient where you deviate from your prophylaxis standard (medication, dose, duration, age of the patient)? (multiple answers possible, please specify in answer box) [in newborns][Comment]

Are there situations or clinical findings in the patient where you deviate from your prophylaxis standard (medication, dose, duration, age of the patient)? (multiple answers possible, please specify in answer box) [Other]
x

Are there situations or clinical findings in the patient where you deviate from your prophylaxis standard (medication, dose, duration, age of the patient)? (multiple answers possible, please specify in answer box) [Other comment]

Are there situations in which you use intravenous prophylaxis?
No

Voiding analysis

Are there differences in antibiotic prophylaxis between VUS and VCUG in your department?
No

Is a non-pathological urine strip test required in preparation for VUS/VCUG in your department?
No

Is a non-pathological urine culture required in preparation for VUS/VCUG in your department?
No

Do patients only receive post-procedure prophylaxis if vesicoureteral reflux (VUR) is detected in VUS/VCUG?
Yes

Please explain your approach:
Wenn Reflux nachgewiesen, Prophylaxe weiter bis Unterspritzung. Wenn kein Reflux nachgewiesen

Do other MUS/MCU findings (e.g. urethral valves) have an influence on post-procedure prophylaxis?
No

End of survey

May we contact you personally if we have further questions?
N/A

Survey response 37

Response ID
38

Last page
1

Start language
de

Seed
1962669578

Date started
2024-11-18 09:11:24

Date last action
2024-11-18 09:12:11

Standard Operation Procedure

Who fills out this survey?
Pediatric Radiologist

Who fills out this survey? [Other]

In which type of hospital are you employed?
Community hospital (>50 beds for pediatric patients)

In which type of hospital are you employed? [Other]

In which country do you work?
Deutschland

Does your department have a defined protocol for infection prophylaxis in the context of VUS/VCUG?
Yes

Has this protocol been transformed into a written standard operating procedure (SOP)?
Yes

Which department is the author of the SOP for infection prophylaxis in VUS/VCUG?
Pediatric radiology

Which department is the author of the SOP for infection prophylaxis in VUS/VCUG? [Other]

Antibiotic Prophylaxis

Are there situations/constellations in which you do not use antibiotic prophylaxis for VUS/VCUG?
N/A

Please specify these situations/constellations: [girl with first uncomplicated UTI without urinary tract dilation in ultrasound]
N/A

Please specify these situations/constellations: [boy with first uncomplicated UTI without urinary tract dilation in ultrasound]
N/A

Please specify these situations/constellations: [in patients with low grade urinary tract dilation (up to 2nd grade) and no UTI in medical history]
N/A

If you use antibiotic prophylaxis: which mediaction do you use? (multiple answers and subdivision into age groups possible) [Trimethoprim]
N/A

If you use antibiotic prophylaxis: which mediaction do you use? (multiple answers and subdivision into age groups possible) [Nitrofurantoin]
N/A

If you use antibiotic prophylaxis: which mediaction do you use? (multiple answers and subdivision into age groups possible) [Amoxicillin]
N/A

If you use antibiotic prophylaxis: which mediaction do you use? (multiple answers and subdivision into age groups possible) [Cefaclor]
N/A

If you use antibiotic prophylaxis: which mediaction do you use? (multiple answers and subdivision into age groups possible) [Amoxicillin/clavulanic acid]
N/A

If you use antibiotic prophylaxis: which mediaction do you use? (multiple answers and subdivision into age groups possible) [Other]
N/A

Trimethoprim: which time schedule do you use? [Day(s) before VUS/VCUG, how many?]
N/A

Trimethoprim: which time schedule do you use? [Day of VUS/VCUG]
N/A

Trimethoprim: which time schedule do you use? [Day(s) after VUS/VCUG, how many?]
N/A

Nitrofurantoin: which time schedule do you use? [Day(s) before VUS/VCUG, how many?]
N/A

Nitrofurantoin: which time schedule do you use? [Day of VUS/VCUG]
N/A

Nitrofurantoin: which time schedule do you use? [Day(s) after VUS/VCUG, how many?]
N/A

Amoxicillin: which time schedule do you use? [Day(s) before VUS/VCUG, how many?]
N/A

Amoxicillin: which time schedule do you use? [Day of VUS/VCUG]
N/A

Amoxicillin: which time schedule do you use? [Day(s) after VUS/VCUG, how many?]
N/A

Cefaclor: which time schedule do you use? [Days(s) before VUS/VCUG, how many?]
N/A

Cefaclor: which time schedule do you use? [Day of VUS/VCUG]
N/A

Cefaclor: which time schedule do you use? [Day(s) after VUS/VCUG, how many?]
N/A

Amoxicillin/clavulanic acid: in what dosage? (Amoxicillin portion) [prophylaktic (10-20 mg/Kg bodyweight/d)]
N/A

Amoxicillin/clavulanic acid: in what dosage? (Amoxicillin portion) [double prophylactic (20-40 mg/Kg bodyweight/d)]
N/A

Amoxicillin/clavulanic acid: in what dosage? (Amoxicillin portion) [therapeutic (80-100 mg/Kg bodyweight/d)]
N/A

Amoxicillin/clavulanic acid: which time schedule do you use? [Day(s) before VUS/VCUG]
N/A

Amoxicillin/clavulanic acid: which time schedule do you use? [Day of VUS/VCUG]
N/A

Amoxicillin/clavulanic acid: which time schedule do you use? [Day(s) after VUS/VCUG]
N/A

Other: which time schedule do you use? [Day(s) before VUS/VCUG, how many?]
N/A

Other: which time schedule do you use? [Day of VUS/VCUG]
N/A

Other: which time schedule do you use? [Day(s) after VUS/VCUG, how many?]
N/A

Indication for prophylaxis

Which patients receive periinterventional antibiotic prophylaxis? (multiple answers possible) [all]
N/A

Which patients receive periinterventional antibiotic prophylaxis? (multiple answers possible) [patients post urinary tract infection/pyelonephritis/urosepsis]
N/A

Which patients receive periinterventional antibiotic prophylaxis? (multiple answers possible) [patients with urinary tract dilation in ultrasound]
N/A

Which patients receive periinterventional antibiotic prophylaxis? (multiple answers possible) [patients with prenatal urinary tract dilation]
N/A

Which patients receive periinterventional antibiotic prophylaxis? (multiple answers possible) [patients post VUR surgery]
N/A

Which patients receive periinterventional antibiotic prophylaxis? (multiple answers possible) [none]
N/A

Are there situations or clinical findings in the patient where you deviate from your prophylaxis standard (medication, dose, duration, age of the patient)? (multiple answers possible, please specify in answer box) [in case of already known vesicoureteral reflux (VUR)]
N/A

Are there situations or clinical findings in the patient where you deviate from your prophylaxis standard (medication, dose, duration, age of the patient)? (multiple answers possible, please specify in answer box) [in patients with recurrent urinary tract infections (UTI)]
N/A

Are there situations or clinical findings in the patient where you deviate from your prophylaxis standard (medication, dose, duration, age of the patient)? (multiple answers possible, please specify in answer box) [if multidrug-resistant organisms (MDRO) are detected in urine cultures]
N/A

Are there situations or clinical findings in the patient where you deviate from your prophylaxis standard (medication, dose, duration, age of the patient)? (multiple answers possible, please specify in answer box) [in newborns]
N/A

Are there situations in which you use intravenous prophylaxis?
N/A

Voiding analysis

Are there differences in antibiotic prophylaxis between VUS and VCUG in your department?
N/A

Is a non-pathological urine strip test required in preparation for VUS/VCUG in your department?
N/A

Is a non-pathological urine culture required in preparation for VUS/VCUG in your department?
N/A

Do patients only receive post-procedure prophylaxis if vesicoureteral reflux (VUR) is detected in VUS/VCUG?
N/A

Do other MUS/MCU findings (e.g. urethral valves) have an influence on post-procedure prophylaxis?
N/A

End of survey

May we contact you personally if we have further questions?
N/A

Survey response 38

Response ID
39

Last page
0

Start language
de

Seed
1491901939

Date started
2024-11-18 09:59:16

Date last action
2024-11-18 09:59:16

Standard Operation Procedure

Does your department have a defined protocol for infection prophylaxis in the context of VUS/VCUG?
N/A

Has this protocol been transformed into a written standard operating procedure (SOP)?
N/A

Antibiotic Prophylaxis

Are there situations/constellations in which you do not use antibiotic prophylaxis for VUS/VCUG?
N/A

Please specify these situations/constellations: [girl with first uncomplicated UTI without urinary tract dilation in ultrasound]
N/A

Please specify these situations/constellations: [boy with first uncomplicated UTI without urinary tract dilation in ultrasound]
N/A

Please specify these situations/constellations: [in patients with low grade urinary tract dilation (up to 2nd grade) and no UTI in medical history]
N/A

If you use antibiotic prophylaxis: which mediaction do you use? (multiple answers and subdivision into age groups possible) [Trimethoprim]
N/A

If you use antibiotic prophylaxis: which mediaction do you use? (multiple answers and subdivision into age groups possible) [Nitrofurantoin]
N/A

If you use antibiotic prophylaxis: which mediaction do you use? (multiple answers and subdivision into age groups possible) [Amoxicillin]
N/A

If you use antibiotic prophylaxis: which mediaction do you use? (multiple answers and subdivision into age groups possible) [Cefaclor]
N/A

If you use antibiotic prophylaxis: which mediaction do you use? (multiple answers and subdivision into age groups possible) [Amoxicillin/clavulanic acid]
N/A

If you use antibiotic prophylaxis: which mediaction do you use? (multiple answers and subdivision into age groups possible) [Other]
N/A

Trimethoprim: which time schedule do you use? [Day(s) before VUS/VCUG, how many?]
N/A

Trimethoprim: which time schedule do you use? [Day of VUS/VCUG]
N/A

Trimethoprim: which time schedule do you use? [Day(s) after VUS/VCUG, how many?]
N/A

Nitrofurantoin: which time schedule do you use? [Day(s) before VUS/VCUG, how many?]
N/A

Nitrofurantoin: which time schedule do you use? [Day of VUS/VCUG]
N/A

Nitrofurantoin: which time schedule do you use? [Day(s) after VUS/VCUG, how many?]
N/A

Amoxicillin: which time schedule do you use? [Day(s) before VUS/VCUG, how many?]
N/A

Amoxicillin: which time schedule do you use? [Day of VUS/VCUG]
N/A

Amoxicillin: which time schedule do you use? [Day(s) after VUS/VCUG, how many?]
N/A

Cefaclor: which time schedule do you use? [Days(s) before VUS/VCUG, how many?]
N/A

Cefaclor: which time schedule do you use? [Day of VUS/VCUG]
N/A

Cefaclor: which time schedule do you use? [Day(s) after VUS/VCUG, how many?]
N/A

Amoxicillin/clavulanic acid: in what dosage? (Amoxicillin portion) [prophylaktic (10-20 mg/Kg bodyweight/d)]
N/A

Amoxicillin/clavulanic acid: in what dosage? (Amoxicillin portion) [double prophylactic (20-40 mg/Kg bodyweight/d)]
N/A

Amoxicillin/clavulanic acid: in what dosage? (Amoxicillin portion) [therapeutic (80-100 mg/Kg bodyweight/d)]
N/A

Amoxicillin/clavulanic acid: which time schedule do you use? [Day(s) before VUS/VCUG]
N/A

Amoxicillin/clavulanic acid: which time schedule do you use? [Day of VUS/VCUG]
N/A

Amoxicillin/clavulanic acid: which time schedule do you use? [Day(s) after VUS/VCUG]
N/A

Other: which time schedule do you use? [Day(s) before VUS/VCUG, how many?]
N/A

Other: which time schedule do you use? [Day of VUS/VCUG]
N/A

Other: which time schedule do you use? [Day(s) after VUS/VCUG, how many?]
N/A

Indication for prophylaxis

Which patients receive periinterventional antibiotic prophylaxis? (multiple answers possible) [all]
N/A

Which patients receive periinterventional antibiotic prophylaxis? (multiple answers possible) [patients post urinary tract infection/pyelonephritis/urosepsis]
N/A

Which patients receive periinterventional antibiotic prophylaxis? (multiple answers possible) [patients with urinary tract dilation in ultrasound]
N/A

Which patients receive periinterventional antibiotic prophylaxis? (multiple answers possible) [patients with prenatal urinary tract dilation]
N/A

Which patients receive periinterventional antibiotic prophylaxis? (multiple answers possible) [patients post VUR surgery]
N/A

Which patients receive periinterventional antibiotic prophylaxis? (multiple answers possible) [none]
N/A

Are there situations or clinical findings in the patient where you deviate from your prophylaxis standard (medication, dose, duration, age of the patient)? (multiple answers possible, please specify in answer box) [in case of already known vesicoureteral reflux (VUR)]
N/A

Are there situations or clinical findings in the patient where you deviate from your prophylaxis standard (medication, dose, duration, age of the patient)? (multiple answers possible, please specify in answer box) [in patients with recurrent urinary tract infections (UTI)]
N/A

Are there situations or clinical findings in the patient where you deviate from your prophylaxis standard (medication, dose, duration, age of the patient)? (multiple answers possible, please specify in answer box) [if multidrug-resistant organisms (MDRO) are detected in urine cultures]
N/A

Are there situations or clinical findings in the patient where you deviate from your prophylaxis standard (medication, dose, duration, age of the patient)? (multiple answers possible, please specify in answer box) [in newborns]
N/A

Are there situations in which you use intravenous prophylaxis?
N/A

Voiding analysis

Are there differences in antibiotic prophylaxis between VUS and VCUG in your department?
N/A

Is a non-pathological urine strip test required in preparation for VUS/VCUG in your department?
N/A

Is a non-pathological urine culture required in preparation for VUS/VCUG in your department?
N/A

Do patients only receive post-procedure prophylaxis if vesicoureteral reflux (VUR) is detected in VUS/VCUG?
N/A

Do other MUS/MCU findings (e.g. urethral valves) have an influence on post-procedure prophylaxis?
N/A

End of survey

May we contact you personally if we have further questions?
N/A

Survey response 39

Response ID
40

Last page
4

Start language
de

Seed
33158185

Date started
2024-11-18 09:59:30

Date last action
2024-11-18 10:09:32

Standard Operation Procedure

Who fills out this survey?
Pediatric Radiologist

Who fills out this survey? [Other]

In which type of hospital are you employed?
Community hospital (>50 beds for pediatric patients)

In which type of hospital are you employed? [Other]

In which country do you work?
Deutschland

Does your department have a defined protocol for infection prophylaxis in the context of VUS/VCUG?
Yes

Has this protocol been transformed into a written standard operating procedure (SOP)?
Yes

Which department is the author of the SOP for infection prophylaxis in VUS/VCUG?
Pediatric radiology

Which department is the author of the SOP for infection prophylaxis in VUS/VCUG? [Other]

Antibiotic Prophylaxis

Are there situations/constellations in which you do not use antibiotic prophylaxis for VUS/VCUG?
No

Please specify these situations/constellations: [girl with first uncomplicated UTI without urinary tract dilation in ultrasound]
N/A

Please specify these situations/constellations: [boy with first uncomplicated UTI without urinary tract dilation in ultrasound]
N/A

Please specify these situations/constellations: [in patients with low grade urinary tract dilation (up to 2nd grade) and no UTI in medical history]
N/A

If you use antibiotic prophylaxis: which mediaction do you use? (multiple answers and subdivision into age groups possible) [Trimethoprim]
Yes

If you use antibiotic prophylaxis: which mediaction do you use? (multiple answers and subdivision into age groups possible) [Nitrofurantoin]
No

If you use antibiotic prophylaxis: which mediaction do you use? (multiple answers and subdivision into age groups possible) [Amoxicillin]
No

If you use antibiotic prophylaxis: which mediaction do you use? (multiple answers and subdivision into age groups possible) [Cefaclor]
Yes

If you use antibiotic prophylaxis: which mediaction do you use? (multiple answers and subdivision into age groups possible) [Amoxicillin/clavulanic acid]
No

If you use antibiotic prophylaxis: which mediaction do you use? (multiple answers and subdivision into age groups possible) [Other]
No

Trimethoprim: in what dosage?
double prophylakctic (4 mg/kg bodyweight/d)

Trimethoprim: in what dosage? [Other]

Trimethoprim: for which age group do you use trimethoprim?
3 Monate bis 3. LJ

Trimethoprim: which time schedule do you use? [Day(s) before VUS/VCUG, how many?]
No

Trimethoprim: which time schedule do you use? [Day(s) before VUS/VCUG, how many?][Comment]

Trimethoprim: which time schedule do you use? [Day of VUS/VCUG]
Yes

Trimethoprim: which time schedule do you use? [Day of VUS/VCUG][Comment]

Trimethoprim: which time schedule do you use? [Day(s) after VUS/VCUG, how many?]
No

Trimethoprim: which time schedule do you use? [Day(s) after VUS/VCUG, how many?][Comment]

Nitrofurantoin: which time schedule do you use? [Day(s) before VUS/VCUG, how many?]
N/A

Nitrofurantoin: which time schedule do you use? [Day of VUS/VCUG]
N/A

Nitrofurantoin: which time schedule do you use? [Day(s) after VUS/VCUG, how many?]
N/A

Amoxicillin: which time schedule do you use? [Day(s) before VUS/VCUG, how many?]
N/A

Amoxicillin: which time schedule do you use? [Day of VUS/VCUG]
N/A

Amoxicillin: which time schedule do you use? [Day(s) after VUS/VCUG, how many?]
N/A

Cefaclor: in what dosage?
therapeutic (40 mg/kg bodyweight/d)

Cefaclor: in what dosage? [Other]

Cefaclor: for which age group do you use cefaclor?
bis 3 Monate

Cefaclor: which time schedule do you use? [Days(s) before VUS/VCUG, how many?]
No

Cefaclor: which time schedule do you use? [Days(s) before VUS/VCUG, how many?][Comment]

Cefaclor: which time schedule do you use? [Day of VUS/VCUG]
Yes

Cefaclor: which time schedule do you use? [Day of VUS/VCUG][Comment]

Cefaclor: which time schedule do you use? [Day(s) after VUS/VCUG, how many?]
No

Cefaclor: which time schedule do you use? [Day(s) after VUS/VCUG, how many?][Comment]

Amoxicillin/clavulanic acid: in what dosage? (Amoxicillin portion) [prophylaktic (10-20 mg/Kg bodyweight/d)]
N/A

Amoxicillin/clavulanic acid: in what dosage? (Amoxicillin portion) [double prophylactic (20-40 mg/Kg bodyweight/d)]
N/A

Amoxicillin/clavulanic acid: in what dosage? (Amoxicillin portion) [therapeutic (80-100 mg/Kg bodyweight/d)]
N/A

Amoxicillin/clavulanic acid: which time schedule do you use? [Day(s) before VUS/VCUG]
N/A

Amoxicillin/clavulanic acid: which time schedule do you use? [Day of VUS/VCUG]
N/A

Amoxicillin/clavulanic acid: which time schedule do you use? [Day(s) after VUS/VCUG]
N/A

Other: which time schedule do you use? [Day(s) before VUS/VCUG, how many?]
N/A

Other: which time schedule do you use? [Day of VUS/VCUG]
N/A

Other: which time schedule do you use? [Day(s) after VUS/VCUG, how many?]
N/A

Indication for prophylaxis

Which patients receive periinterventional antibiotic prophylaxis? (multiple answers possible) [all]
Yes

Which patients receive periinterventional antibiotic prophylaxis? (multiple answers possible) [patients post urinary tract infection/pyelonephritis/urosepsis]
N/A

Which patients receive periinterventional antibiotic prophylaxis? (multiple answers possible) [patients with urinary tract dilation in ultrasound]
N/A

Which patients receive periinterventional antibiotic prophylaxis? (multiple answers possible) [patients with prenatal urinary tract dilation]
N/A

Which patients receive periinterventional antibiotic prophylaxis? (multiple answers possible) [patients post VUR surgery]
N/A

Which patients receive periinterventional antibiotic prophylaxis? (multiple answers possible) [none]
N/A

Are there situations or clinical findings in the patient where you deviate from your prophylaxis standard (medication, dose, duration, age of the patient)? (multiple answers possible, please specify in answer box) [in case of already known vesicoureteral reflux (VUR)]
Yes

Are there situations or clinical findings in the patient where you deviate from your prophylaxis standard (medication, dose, duration, age of the patient)? (multiple answers possible, please specify in answer box) [in case of already known vesicoureteral reflux (VUR)][Comment]
bei laufender Prophylaxe

Are there situations or clinical findings in the patient where you deviate from your prophylaxis standard (medication, dose, duration, age of the patient)? (multiple answers possible, please specify in answer box) [in patients with recurrent urinary tract infections (UTI)]
Yes

Are there situations or clinical findings in the patient where you deviate from your prophylaxis standard (medication, dose, duration, age of the patient)? (multiple answers possible, please specify in answer box) [in patients with recurrent urinary tract infections (UTI)][Comment]
bei laufender Prophylaxe oder Therapie

Are there situations or clinical findings in the patient where you deviate from your prophylaxis standard (medication, dose, duration, age of the patient)? (multiple answers possible, please specify in answer box) [if multidrug-resistant organisms (MDRO) are detected in urine cultures]
Yes

Are there situations or clinical findings in the patient where you deviate from your prophylaxis standard (medication, dose, duration, age of the patient)? (multiple answers possible, please specify in answer box) [if multidrug-resistant organisms (MDRO) are detected in urine cultures][Comment]

Are there situations or clinical findings in the patient where you deviate from your prophylaxis standard (medication, dose, duration, age of the patient)? (multiple answers possible, please specify in answer box) [in newborns]
No

Are there situations or clinical findings in the patient where you deviate from your prophylaxis standard (medication, dose, duration, age of the patient)? (multiple answers possible, please specify in answer box) [in newborns][Comment]

Are there situations or clinical findings in the patient where you deviate from your prophylaxis standard (medication, dose, duration, age of the patient)? (multiple answers possible, please specify in answer box) [Other]

Are there situations or clinical findings in the patient where you deviate from your prophylaxis standard (medication, dose, duration, age of the patient)? (multiple answers possible, please specify in answer box) [Other comment]

Are there situations in which you use intravenous prophylaxis?
No

Voiding analysis

Are there differences in antibiotic prophylaxis between VUS and VCUG in your department?
No

Is a non-pathological urine strip test required in preparation for VUS/VCUG in your department?
Yes

What is the maximum amount of days accepted for urine strip test to be performed prior to the exam?
1

Is a non-pathological urine culture required in preparation for VUS/VCUG in your department?
No

Do patients only receive post-procedure prophylaxis if vesicoureteral reflux (VUR) is detected in VUS/VCUG?
No

Do other MUS/MCU findings (e.g. urethral valves) have an influence on post-procedure prophylaxis?
No

End of survey

May we contact you personally if we have further questions?
N/A

Survey response 40

Response ID
41

Last page
0

Start language
de

Seed
1320011971

Date started
2024-11-18 11:55:25

Date last action
2024-11-18 11:55:25

Standard Operation Procedure

Does your department have a defined protocol for infection prophylaxis in the context of VUS/VCUG?
N/A

Has this protocol been transformed into a written standard operating procedure (SOP)?
N/A

Antibiotic Prophylaxis

Are there situations/constellations in which you do not use antibiotic prophylaxis for VUS/VCUG?
N/A

Please specify these situations/constellations: [girl with first uncomplicated UTI without urinary tract dilation in ultrasound]
N/A

Please specify these situations/constellations: [boy with first uncomplicated UTI without urinary tract dilation in ultrasound]
N/A

Please specify these situations/constellations: [in patients with low grade urinary tract dilation (up to 2nd grade) and no UTI in medical history]
N/A

If you use antibiotic prophylaxis: which mediaction do you use? (multiple answers and subdivision into age groups possible) [Trimethoprim]
N/A

If you use antibiotic prophylaxis: which mediaction do you use? (multiple answers and subdivision into age groups possible) [Nitrofurantoin]
N/A

If you use antibiotic prophylaxis: which mediaction do you use? (multiple answers and subdivision into age groups possible) [Amoxicillin]
N/A

If you use antibiotic prophylaxis: which mediaction do you use? (multiple answers and subdivision into age groups possible) [Cefaclor]
N/A

If you use antibiotic prophylaxis: which mediaction do you use? (multiple answers and subdivision into age groups possible) [Amoxicillin/clavulanic acid]
N/A

If you use antibiotic prophylaxis: which mediaction do you use? (multiple answers and subdivision into age groups possible) [Other]
N/A

Trimethoprim: which time schedule do you use? [Day(s) before VUS/VCUG, how many?]
N/A

Trimethoprim: which time schedule do you use? [Day of VUS/VCUG]
N/A

Trimethoprim: which time schedule do you use? [Day(s) after VUS/VCUG, how many?]
N/A

Nitrofurantoin: which time schedule do you use? [Day(s) before VUS/VCUG, how many?]
N/A

Nitrofurantoin: which time schedule do you use? [Day of VUS/VCUG]
N/A

Nitrofurantoin: which time schedule do you use? [Day(s) after VUS/VCUG, how many?]
N/A

Amoxicillin: which time schedule do you use? [Day(s) before VUS/VCUG, how many?]
N/A

Amoxicillin: which time schedule do you use? [Day of VUS/VCUG]
N/A

Amoxicillin: which time schedule do you use? [Day(s) after VUS/VCUG, how many?]
N/A

Cefaclor: which time schedule do you use? [Days(s) before VUS/VCUG, how many?]
N/A

Cefaclor: which time schedule do you use? [Day of VUS/VCUG]
N/A

Cefaclor: which time schedule do you use? [Day(s) after VUS/VCUG, how many?]
N/A

Amoxicillin/clavulanic acid: in what dosage? (Amoxicillin portion) [prophylaktic (10-20 mg/Kg bodyweight/d)]
N/A

Amoxicillin/clavulanic acid: in what dosage? (Amoxicillin portion) [double prophylactic (20-40 mg/Kg bodyweight/d)]
N/A

Amoxicillin/clavulanic acid: in what dosage? (Amoxicillin portion) [therapeutic (80-100 mg/Kg bodyweight/d)]
N/A

Amoxicillin/clavulanic acid: which time schedule do you use? [Day(s) before VUS/VCUG]
N/A

Amoxicillin/clavulanic acid: which time schedule do you use? [Day of VUS/VCUG]
N/A

Amoxicillin/clavulanic acid: which time schedule do you use? [Day(s) after VUS/VCUG]
N/A

Other: which time schedule do you use? [Day(s) before VUS/VCUG, how many?]
N/A

Other: which time schedule do you use? [Day of VUS/VCUG]
N/A

Other: which time schedule do you use? [Day(s) after VUS/VCUG, how many?]
N/A

Indication for prophylaxis

Which patients receive periinterventional antibiotic prophylaxis? (multiple answers possible) [all]
N/A

Which patients receive periinterventional antibiotic prophylaxis? (multiple answers possible) [patients post urinary tract infection/pyelonephritis/urosepsis]
N/A

Which patients receive periinterventional antibiotic prophylaxis? (multiple answers possible) [patients with urinary tract dilation in ultrasound]
N/A

Which patients receive periinterventional antibiotic prophylaxis? (multiple answers possible) [patients with prenatal urinary tract dilation]
N/A

Which patients receive periinterventional antibiotic prophylaxis? (multiple answers possible) [patients post VUR surgery]
N/A

Which patients receive periinterventional antibiotic prophylaxis? (multiple answers possible) [none]
N/A

Are there situations or clinical findings in the patient where you deviate from your prophylaxis standard (medication, dose, duration, age of the patient)? (multiple answers possible, please specify in answer box) [in case of already known vesicoureteral reflux (VUR)]
N/A

Are there situations or clinical findings in the patient where you deviate from your prophylaxis standard (medication, dose, duration, age of the patient)? (multiple answers possible, please specify in answer box) [in patients with recurrent urinary tract infections (UTI)]
N/A

Are there situations or clinical findings in the patient where you deviate from your prophylaxis standard (medication, dose, duration, age of the patient)? (multiple answers possible, please specify in answer box) [if multidrug-resistant organisms (MDRO) are detected in urine cultures]
N/A

Are there situations or clinical findings in the patient where you deviate from your prophylaxis standard (medication, dose, duration, age of the patient)? (multiple answers possible, please specify in answer box) [in newborns]
N/A

Are there situations in which you use intravenous prophylaxis?
N/A

Voiding analysis

Are there differences in antibiotic prophylaxis between VUS and VCUG in your department?
N/A

Is a non-pathological urine strip test required in preparation for VUS/VCUG in your department?
N/A

Is a non-pathological urine culture required in preparation for VUS/VCUG in your department?
N/A

Do patients only receive post-procedure prophylaxis if vesicoureteral reflux (VUR) is detected in VUS/VCUG?
N/A

Do other MUS/MCU findings (e.g. urethral valves) have an influence on post-procedure prophylaxis?
N/A

End of survey

May we contact you personally if we have further questions?
N/A

Survey response 41

Response ID
42

Last page
0

Start language
de

Seed
1672384632

Date started
2024-11-19 08:00:27

Date last action
2024-11-19 08:00:27

Standard Operation Procedure

Does your department have a defined protocol for infection prophylaxis in the context of VUS/VCUG?
N/A

Has this protocol been transformed into a written standard operating procedure (SOP)?
N/A

Antibiotic Prophylaxis

Are there situations/constellations in which you do not use antibiotic prophylaxis for VUS/VCUG?
N/A

Please specify these situations/constellations: [girl with first uncomplicated UTI without urinary tract dilation in ultrasound]
N/A

Please specify these situations/constellations: [boy with first uncomplicated UTI without urinary tract dilation in ultrasound]
N/A

Please specify these situations/constellations: [in patients with low grade urinary tract dilation (up to 2nd grade) and no UTI in medical history]
N/A

If you use antibiotic prophylaxis: which mediaction do you use? (multiple answers and subdivision into age groups possible) [Trimethoprim]
N/A

If you use antibiotic prophylaxis: which mediaction do you use? (multiple answers and subdivision into age groups possible) [Nitrofurantoin]
N/A

If you use antibiotic prophylaxis: which mediaction do you use? (multiple answers and subdivision into age groups possible) [Amoxicillin]
N/A

If you use antibiotic prophylaxis: which mediaction do you use? (multiple answers and subdivision into age groups possible) [Cefaclor]
N/A

If you use antibiotic prophylaxis: which mediaction do you use? (multiple answers and subdivision into age groups possible) [Amoxicillin/clavulanic acid]
N/A

If you use antibiotic prophylaxis: which mediaction do you use? (multiple answers and subdivision into age groups possible) [Other]
N/A

Trimethoprim: which time schedule do you use? [Day(s) before VUS/VCUG, how many?]
N/A

Trimethoprim: which time schedule do you use? [Day of VUS/VCUG]
N/A

Trimethoprim: which time schedule do you use? [Day(s) after VUS/VCUG, how many?]
N/A

Nitrofurantoin: which time schedule do you use? [Day(s) before VUS/VCUG, how many?]
N/A

Nitrofurantoin: which time schedule do you use? [Day of VUS/VCUG]
N/A

Nitrofurantoin: which time schedule do you use? [Day(s) after VUS/VCUG, how many?]
N/A

Amoxicillin: which time schedule do you use? [Day(s) before VUS/VCUG, how many?]
N/A

Amoxicillin: which time schedule do you use? [Day of VUS/VCUG]
N/A

Amoxicillin: which time schedule do you use? [Day(s) after VUS/VCUG, how many?]
N/A

Cefaclor: which time schedule do you use? [Days(s) before VUS/VCUG, how many?]
N/A

Cefaclor: which time schedule do you use? [Day of VUS/VCUG]
N/A

Cefaclor: which time schedule do you use? [Day(s) after VUS/VCUG, how many?]
N/A

Amoxicillin/clavulanic acid: in what dosage? (Amoxicillin portion) [prophylaktic (10-20 mg/Kg bodyweight/d)]
N/A

Amoxicillin/clavulanic acid: in what dosage? (Amoxicillin portion) [double prophylactic (20-40 mg/Kg bodyweight/d)]
N/A

Amoxicillin/clavulanic acid: in what dosage? (Amoxicillin portion) [therapeutic (80-100 mg/Kg bodyweight/d)]
N/A

Amoxicillin/clavulanic acid: which time schedule do you use? [Day(s) before VUS/VCUG]
N/A

Amoxicillin/clavulanic acid: which time schedule do you use? [Day of VUS/VCUG]
N/A

Amoxicillin/clavulanic acid: which time schedule do you use? [Day(s) after VUS/VCUG]
N/A

Other: which time schedule do you use? [Day(s) before VUS/VCUG, how many?]
N/A

Other: which time schedule do you use? [Day of VUS/VCUG]
N/A

Other: which time schedule do you use? [Day(s) after VUS/VCUG, how many?]
N/A

Indication for prophylaxis

Which patients receive periinterventional antibiotic prophylaxis? (multiple answers possible) [all]
N/A

Which patients receive periinterventional antibiotic prophylaxis? (multiple answers possible) [patients post urinary tract infection/pyelonephritis/urosepsis]
N/A

Which patients receive periinterventional antibiotic prophylaxis? (multiple answers possible) [patients with urinary tract dilation in ultrasound]
N/A

Which patients receive periinterventional antibiotic prophylaxis? (multiple answers possible) [patients with prenatal urinary tract dilation]
N/A

Which patients receive periinterventional antibiotic prophylaxis? (multiple answers possible) [patients post VUR surgery]
N/A

Which patients receive periinterventional antibiotic prophylaxis? (multiple answers possible) [none]
N/A

Are there situations or clinical findings in the patient where you deviate from your prophylaxis standard (medication, dose, duration, age of the patient)? (multiple answers possible, please specify in answer box) [in case of already known vesicoureteral reflux (VUR)]
N/A

Are there situations or clinical findings in the patient where you deviate from your prophylaxis standard (medication, dose, duration, age of the patient)? (multiple answers possible, please specify in answer box) [in patients with recurrent urinary tract infections (UTI)]
N/A

Are there situations or clinical findings in the patient where you deviate from your prophylaxis standard (medication, dose, duration, age of the patient)? (multiple answers possible, please specify in answer box) [if multidrug-resistant organisms (MDRO) are detected in urine cultures]
N/A

Are there situations or clinical findings in the patient where you deviate from your prophylaxis standard (medication, dose, duration, age of the patient)? (multiple answers possible, please specify in answer box) [in newborns]
N/A

Are there situations in which you use intravenous prophylaxis?
N/A

Voiding analysis

Are there differences in antibiotic prophylaxis between VUS and VCUG in your department?
N/A

Is a non-pathological urine strip test required in preparation for VUS/VCUG in your department?
N/A

Is a non-pathological urine culture required in preparation for VUS/VCUG in your department?
N/A

Do patients only receive post-procedure prophylaxis if vesicoureteral reflux (VUR) is detected in VUS/VCUG?
N/A

Do other MUS/MCU findings (e.g. urethral valves) have an influence on post-procedure prophylaxis?
N/A

End of survey

May we contact you personally if we have further questions?
N/A

Survey response 42

Response ID
43

Date submitted
2024-11-19 09:43:32

Last page
5

Start language
de

Seed
642485837

Date started
2024-11-19 09:29:12

Date last action
2024-11-19 09:43:32

Standard Operation Procedure

Who fills out this survey?
Pediatric Radiologist

Who fills out this survey? [Other]

In which type of hospital are you employed?
University Hospital

In which type of hospital are you employed? [Other]

In which country do you work?
D

Does your department have a defined protocol for infection prophylaxis in the context of VUS/VCUG?
Yes

Has this protocol been transformed into a written standard operating procedure (SOP)?
No

Antibiotic Prophylaxis

Are there situations/constellations in which you do not use antibiotic prophylaxis for VUS/VCUG?
No

Please specify these situations/constellations: [girl with first uncomplicated UTI without urinary tract dilation in ultrasound]
N/A

Please specify these situations/constellations: [boy with first uncomplicated UTI without urinary tract dilation in ultrasound]
N/A

Please specify these situations/constellations: [in patients with low grade urinary tract dilation (up to 2nd grade) and no UTI in medical history]
N/A

If you use antibiotic prophylaxis: which mediaction do you use? (multiple answers and subdivision into age groups possible) [Trimethoprim]
No

If you use antibiotic prophylaxis: which mediaction do you use? (multiple answers and subdivision into age groups possible) [Nitrofurantoin]
No

If you use antibiotic prophylaxis: which mediaction do you use? (multiple answers and subdivision into age groups possible) [Amoxicillin]
No

If you use antibiotic prophylaxis: which mediaction do you use? (multiple answers and subdivision into age groups possible) [Cefaclor]
Yes

If you use antibiotic prophylaxis: which mediaction do you use? (multiple answers and subdivision into age groups possible) [Amoxicillin/clavulanic acid]
No

If you use antibiotic prophylaxis: which mediaction do you use? (multiple answers and subdivision into age groups possible) [Other]
No

Trimethoprim: which time schedule do you use? [Day(s) before VUS/VCUG, how many?]
N/A

Trimethoprim: which time schedule do you use? [Day of VUS/VCUG]
N/A

Trimethoprim: which time schedule do you use? [Day(s) after VUS/VCUG, how many?]
N/A

Nitrofurantoin: which time schedule do you use? [Day(s) before VUS/VCUG, how many?]
N/A

Nitrofurantoin: which time schedule do you use? [Day of VUS/VCUG]
N/A

Nitrofurantoin: which time schedule do you use? [Day(s) after VUS/VCUG, how many?]
N/A

Amoxicillin: which time schedule do you use? [Day(s) before VUS/VCUG, how many?]
N/A

Amoxicillin: which time schedule do you use? [Day of VUS/VCUG]
N/A

Amoxicillin: which time schedule do you use? [Day(s) after VUS/VCUG, how many?]
N/A

Cefaclor: in what dosage?
prophylactic (10 mg/kg bodyweight/d)

Cefaclor: in what dosage? [Other]

Cefaclor: for which age group do you use cefaclor?
Säglinge und Kleinkinder

Cefaclor: which time schedule do you use? [Days(s) before VUS/VCUG, how many?]
No

Cefaclor: which time schedule do you use? [Days(s) before VUS/VCUG, how many?][Comment]

Cefaclor: which time schedule do you use? [Day of VUS/VCUG]
Yes

Cefaclor: which time schedule do you use? [Day of VUS/VCUG][Comment]

Cefaclor: which time schedule do you use? [Day(s) after VUS/VCUG, how many?]
Yes

Cefaclor: which time schedule do you use? [Day(s) after VUS/VCUG, how many?][Comment]
3

Amoxicillin/clavulanic acid: in what dosage? (Amoxicillin portion) [prophylaktic (10-20 mg/Kg bodyweight/d)]
N/A

Amoxicillin/clavulanic acid: in what dosage? (Amoxicillin portion) [double prophylactic (20-40 mg/Kg bodyweight/d)]
N/A

Amoxicillin/clavulanic acid: in what dosage? (Amoxicillin portion) [therapeutic (80-100 mg/Kg bodyweight/d)]
N/A

Amoxicillin/clavulanic acid: which time schedule do you use? [Day(s) before VUS/VCUG]
N/A

Amoxicillin/clavulanic acid: which time schedule do you use? [Day of VUS/VCUG]
N/A

Amoxicillin/clavulanic acid: which time schedule do you use? [Day(s) after VUS/VCUG]
N/A

Other: which time schedule do you use? [Day(s) before VUS/VCUG, how many?]
N/A

Other: which time schedule do you use? [Day of VUS/VCUG]
N/A

Other: which time schedule do you use? [Day(s) after VUS/VCUG, how many?]
N/A

Indication for prophylaxis

Which patients receive periinterventional antibiotic prophylaxis? (multiple answers possible) [all]
Yes

Which patients receive periinterventional antibiotic prophylaxis? (multiple answers possible) [patients post urinary tract infection/pyelonephritis/urosepsis]
N/A

Which patients receive periinterventional antibiotic prophylaxis? (multiple answers possible) [patients with urinary tract dilation in ultrasound]
N/A

Which patients receive periinterventional antibiotic prophylaxis? (multiple answers possible) [patients with prenatal urinary tract dilation]
N/A

Which patients receive periinterventional antibiotic prophylaxis? (multiple answers possible) [patients post VUR surgery]
N/A

Which patients receive periinterventional antibiotic prophylaxis? (multiple answers possible) [none]
N/A

Are there situations or clinical findings in the patient where you deviate from your prophylaxis standard (medication, dose, duration, age of the patient)? (multiple answers possible, please specify in answer box) [in case of already known vesicoureteral reflux (VUR)]
No

Are there situations or clinical findings in the patient where you deviate from your prophylaxis standard (medication, dose, duration, age of the patient)? (multiple answers possible, please specify in answer box) [in case of already known vesicoureteral reflux (VUR)][Comment]

Are there situations or clinical findings in the patient where you deviate from your prophylaxis standard (medication, dose, duration, age of the patient)? (multiple answers possible, please specify in answer box) [in patients with recurrent urinary tract infections (UTI)]
Yes

Are there situations or clinical findings in the patient where you deviate from your prophylaxis standard (medication, dose, duration, age of the patient)? (multiple answers possible, please specify in answer box) [in patients with recurrent urinary tract infections (UTI)][Comment]

Are there situations or clinical findings in the patient where you deviate from your prophylaxis standard (medication, dose, duration, age of the patient)? (multiple answers possible, please specify in answer box) [if multidrug-resistant organisms (MDRO) are detected in urine cultures]
No

Are there situations or clinical findings in the patient where you deviate from your prophylaxis standard (medication, dose, duration, age of the patient)? (multiple answers possible, please specify in answer box) [if multidrug-resistant organisms (MDRO) are detected in urine cultures][Comment]

Are there situations or clinical findings in the patient where you deviate from your prophylaxis standard (medication, dose, duration, age of the patient)? (multiple answers possible, please specify in answer box) [in newborns]
No

Are there situations or clinical findings in the patient where you deviate from your prophylaxis standard (medication, dose, duration, age of the patient)? (multiple answers possible, please specify in answer box) [in newborns][Comment]

Are there situations or clinical findings in the patient where you deviate from your prophylaxis standard (medication, dose, duration, age of the patient)? (multiple answers possible, please specify in answer box) [Other]

Are there situations or clinical findings in the patient where you deviate from your prophylaxis standard (medication, dose, duration, age of the patient)? (multiple answers possible, please specify in answer box) [Other comment]

Are there situations in which you use intravenous prophylaxis?
No

Voiding analysis

Are there differences in antibiotic prophylaxis between VUS and VCUG in your department?
No

Is a non-pathological urine strip test required in preparation for VUS/VCUG in your department?
Yes

What is the maximum amount of days accepted for urine strip test to be performed prior to the exam?
1

Is a non-pathological urine culture required in preparation for VUS/VCUG in your department?
No

Do patients only receive post-procedure prophylaxis if vesicoureteral reflux (VUR) is detected in VUS/VCUG?
No

Do other MUS/MCU findings (e.g. urethral valves) have an influence on post-procedure prophylaxis?
No

End of survey

Thank you for taking the time to answer this survey!

May we contact you personally if we have further questions?
No

Survey response 43

Response ID
44

Last page
0

Start language
de

Seed
983284352

Date started
2024-11-20 05:56:33

Date last action
2024-11-20 05:56:33

Standard Operation Procedure

Does your department have a defined protocol for infection prophylaxis in the context of VUS/VCUG?
N/A

Has this protocol been transformed into a written standard operating procedure (SOP)?
N/A

Antibiotic Prophylaxis

Are there situations/constellations in which you do not use antibiotic prophylaxis for VUS/VCUG?
N/A

Please specify these situations/constellations: [girl with first uncomplicated UTI without urinary tract dilation in ultrasound]
N/A

Please specify these situations/constellations: [boy with first uncomplicated UTI without urinary tract dilation in ultrasound]
N/A

Please specify these situations/constellations: [in patients with low grade urinary tract dilation (up to 2nd grade) and no UTI in medical history]
N/A

If you use antibiotic prophylaxis: which mediaction do you use? (multiple answers and subdivision into age groups possible) [Trimethoprim]
N/A

If you use antibiotic prophylaxis: which mediaction do you use? (multiple answers and subdivision into age groups possible) [Nitrofurantoin]
N/A

If you use antibiotic prophylaxis: which mediaction do you use? (multiple answers and subdivision into age groups possible) [Amoxicillin]
N/A

If you use antibiotic prophylaxis: which mediaction do you use? (multiple answers and subdivision into age groups possible) [Cefaclor]
N/A

If you use antibiotic prophylaxis: which mediaction do you use? (multiple answers and subdivision into age groups possible) [Amoxicillin/clavulanic acid]
N/A

If you use antibiotic prophylaxis: which mediaction do you use? (multiple answers and subdivision into age groups possible) [Other]
N/A

Trimethoprim: which time schedule do you use? [Day(s) before VUS/VCUG, how many?]
N/A

Trimethoprim: which time schedule do you use? [Day of VUS/VCUG]
N/A

Trimethoprim: which time schedule do you use? [Day(s) after VUS/VCUG, how many?]
N/A

Nitrofurantoin: which time schedule do you use? [Day(s) before VUS/VCUG, how many?]
N/A

Nitrofurantoin: which time schedule do you use? [Day of VUS/VCUG]
N/A

Nitrofurantoin: which time schedule do you use? [Day(s) after VUS/VCUG, how many?]
N/A

Amoxicillin: which time schedule do you use? [Day(s) before VUS/VCUG, how many?]
N/A

Amoxicillin: which time schedule do you use? [Day of VUS/VCUG]
N/A

Amoxicillin: which time schedule do you use? [Day(s) after VUS/VCUG, how many?]
N/A

Cefaclor: which time schedule do you use? [Days(s) before VUS/VCUG, how many?]
N/A

Cefaclor: which time schedule do you use? [Day of VUS/VCUG]
N/A

Cefaclor: which time schedule do you use? [Day(s) after VUS/VCUG, how many?]
N/A

Amoxicillin/clavulanic acid: in what dosage? (Amoxicillin portion) [prophylaktic (10-20 mg/Kg bodyweight/d)]
N/A

Amoxicillin/clavulanic acid: in what dosage? (Amoxicillin portion) [double prophylactic (20-40 mg/Kg bodyweight/d)]
N/A

Amoxicillin/clavulanic acid: in what dosage? (Amoxicillin portion) [therapeutic (80-100 mg/Kg bodyweight/d)]
N/A

Amoxicillin/clavulanic acid: which time schedule do you use? [Day(s) before VUS/VCUG]
N/A

Amoxicillin/clavulanic acid: which time schedule do you use? [Day of VUS/VCUG]
N/A

Amoxicillin/clavulanic acid: which time schedule do you use? [Day(s) after VUS/VCUG]
N/A

Other: which time schedule do you use? [Day(s) before VUS/VCUG, how many?]
N/A

Other: which time schedule do you use? [Day of VUS/VCUG]
N/A

Other: which time schedule do you use? [Day(s) after VUS/VCUG, how many?]
N/A

Indication for prophylaxis

Which patients receive periinterventional antibiotic prophylaxis? (multiple answers possible) [all]
N/A

Which patients receive periinterventional antibiotic prophylaxis? (multiple answers possible) [patients post urinary tract infection/pyelonephritis/urosepsis]
N/A

Which patients receive periinterventional antibiotic prophylaxis? (multiple answers possible) [patients with urinary tract dilation in ultrasound]
N/A

Which patients receive periinterventional antibiotic prophylaxis? (multiple answers possible) [patients with prenatal urinary tract dilation]
N/A

Which patients receive periinterventional antibiotic prophylaxis? (multiple answers possible) [patients post VUR surgery]
N/A

Which patients receive periinterventional antibiotic prophylaxis? (multiple answers possible) [none]
N/A

Are there situations or clinical findings in the patient where you deviate from your prophylaxis standard (medication, dose, duration, age of the patient)? (multiple answers possible, please specify in answer box) [in case of already known vesicoureteral reflux (VUR)]
N/A

Are there situations or clinical findings in the patient where you deviate from your prophylaxis standard (medication, dose, duration, age of the patient)? (multiple answers possible, please specify in answer box) [in patients with recurrent urinary tract infections (UTI)]
N/A

Are there situations or clinical findings in the patient where you deviate from your prophylaxis standard (medication, dose, duration, age of the patient)? (multiple answers possible, please specify in answer box) [if multidrug-resistant organisms (MDRO) are detected in urine cultures]
N/A

Are there situations or clinical findings in the patient where you deviate from your prophylaxis standard (medication, dose, duration, age of the patient)? (multiple answers possible, please specify in answer box) [in newborns]
N/A

Are there situations in which you use intravenous prophylaxis?
N/A

Voiding analysis

Are there differences in antibiotic prophylaxis between VUS and VCUG in your department?
N/A

Is a non-pathological urine strip test required in preparation for VUS/VCUG in your department?
N/A

Is a non-pathological urine culture required in preparation for VUS/VCUG in your department?
N/A

Do patients only receive post-procedure prophylaxis if vesicoureteral reflux (VUR) is detected in VUS/VCUG?
N/A

Do other MUS/MCU findings (e.g. urethral valves) have an influence on post-procedure prophylaxis?
N/A

End of survey

May we contact you personally if we have further questions?
N/A

Survey response 44

Response ID
45

Date submitted
2024-11-20 11:01:58

Last page
5

Start language
de

Seed
1175241164

Date started
2024-11-20 10:54:27

Date last action
2024-11-20 11:01:58

Standard Operation Procedure

Who fills out this survey?
Pediatric Surgeon

Who fills out this survey? [Other]

In which type of hospital are you employed?
University Hospital

In which type of hospital are you employed? [Other]

In which country do you work?
Deutschland

Does your department have a defined protocol for infection prophylaxis in the context of VUS/VCUG?
Yes

Has this protocol been transformed into a written standard operating procedure (SOP)?
Yes

Which department is the author of the SOP for infection prophylaxis in VUS/VCUG?
Pediatric nephrology

Which department is the author of the SOP for infection prophylaxis in VUS/VCUG? [Other]

Antibiotic Prophylaxis

Are there situations/constellations in which you do not use antibiotic prophylaxis for VUS/VCUG?
No

Please specify these situations/constellations: [girl with first uncomplicated UTI without urinary tract dilation in ultrasound]
N/A

Please specify these situations/constellations: [boy with first uncomplicated UTI without urinary tract dilation in ultrasound]
N/A

Please specify these situations/constellations: [in patients with low grade urinary tract dilation (up to 2nd grade) and no UTI in medical history]
N/A

If you use antibiotic prophylaxis: which mediaction do you use? (multiple answers and subdivision into age groups possible) [Trimethoprim]
Yes

If you use antibiotic prophylaxis: which mediaction do you use? (multiple answers and subdivision into age groups possible) [Nitrofurantoin]
No

If you use antibiotic prophylaxis: which mediaction do you use? (multiple answers and subdivision into age groups possible) [Amoxicillin]
No

If you use antibiotic prophylaxis: which mediaction do you use? (multiple answers and subdivision into age groups possible) [Cefaclor]
Yes

If you use antibiotic prophylaxis: which mediaction do you use? (multiple answers and subdivision into age groups possible) [Amoxicillin/clavulanic acid]
No

If you use antibiotic prophylaxis: which mediaction do you use? (multiple answers and subdivision into age groups possible) [Other]
No

Trimethoprim: in what dosage?
prophylactic (2 mg/kg bodyweight/d)

Trimethoprim: in what dosage? [Other]

Trimethoprim: for which age group do you use trimethoprim?
Ab 3 LJ

Trimethoprim: which time schedule do you use? [Day(s) before VUS/VCUG, how many?]
Yes

Trimethoprim: which time schedule do you use? [Day(s) before VUS/VCUG, how many?][Comment]
Ab Infekt

Trimethoprim: which time schedule do you use? [Day of VUS/VCUG]
Yes

Trimethoprim: which time schedule do you use? [Day of VUS/VCUG][Comment]

Trimethoprim: which time schedule do you use? [Day(s) after VUS/VCUG, how many?]
Yes

Trimethoprim: which time schedule do you use? [Day(s) after VUS/VCUG, how many?][Comment]
Bis operative Therapie

Nitrofurantoin: which time schedule do you use? [Day(s) before VUS/VCUG, how many?]
N/A

Nitrofurantoin: which time schedule do you use? [Day of VUS/VCUG]
N/A

Nitrofurantoin: which time schedule do you use? [Day(s) after VUS/VCUG, how many?]
N/A

Amoxicillin: which time schedule do you use? [Day(s) before VUS/VCUG, how many?]
N/A

Amoxicillin: which time schedule do you use? [Day of VUS/VCUG]
N/A

Amoxicillin: which time schedule do you use? [Day(s) after VUS/VCUG, how many?]
N/A

Cefaclor: in what dosage?
prophylactic (10 mg/kg bodyweight/d)

Cefaclor: in what dosage? [Other]

Cefaclor: for which age group do you use cefaclor?
< 3 LJ

Cefaclor: which time schedule do you use? [Days(s) before VUS/VCUG, how many?]
Yes

Cefaclor: which time schedule do you use? [Days(s) before VUS/VCUG, how many?][Comment]
Ab Infekt

Cefaclor: which time schedule do you use? [Day of VUS/VCUG]
Yes

Cefaclor: which time schedule do you use? [Day of VUS/VCUG][Comment]

Cefaclor: which time schedule do you use? [Day(s) after VUS/VCUG, how many?]
Yes

Cefaclor: which time schedule do you use? [Day(s) after VUS/VCUG, how many?][Comment]
Bis operative Therapie

Amoxicillin/clavulanic acid: in what dosage? (Amoxicillin portion) [prophylaktic (10-20 mg/Kg bodyweight/d)]
N/A

Amoxicillin/clavulanic acid: in what dosage? (Amoxicillin portion) [double prophylactic (20-40 mg/Kg bodyweight/d)]
N/A

Amoxicillin/clavulanic acid: in what dosage? (Amoxicillin portion) [therapeutic (80-100 mg/Kg bodyweight/d)]
N/A

Amoxicillin/clavulanic acid: which time schedule do you use? [Day(s) before VUS/VCUG]
N/A

Amoxicillin/clavulanic acid: which time schedule do you use? [Day of VUS/VCUG]
N/A

Amoxicillin/clavulanic acid: which time schedule do you use? [Day(s) after VUS/VCUG]
N/A

Other: which time schedule do you use? [Day(s) before VUS/VCUG, how many?]
N/A

Other: which time schedule do you use? [Day of VUS/VCUG]
N/A

Other: which time schedule do you use? [Day(s) after VUS/VCUG, how many?]
N/A

Indication for prophylaxis

Which patients receive periinterventional antibiotic prophylaxis? (multiple answers possible) [all]
Yes

Which patients receive periinterventional antibiotic prophylaxis? (multiple answers possible) [patients post urinary tract infection/pyelonephritis/urosepsis]
N/A

Which patients receive periinterventional antibiotic prophylaxis? (multiple answers possible) [patients with urinary tract dilation in ultrasound]
N/A

Which patients receive periinterventional antibiotic prophylaxis? (multiple answers possible) [patients with prenatal urinary tract dilation]
N/A

Which patients receive periinterventional antibiotic prophylaxis? (multiple answers possible) [patients post VUR surgery]
N/A

Which patients receive periinterventional antibiotic prophylaxis? (multiple answers possible) [none]
N/A

Are there situations or clinical findings in the patient where you deviate from your prophylaxis standard (medication, dose, duration, age of the patient)? (multiple answers possible, please specify in answer box) [in case of already known vesicoureteral reflux (VUR)]
No

Are there situations or clinical findings in the patient where you deviate from your prophylaxis standard (medication, dose, duration, age of the patient)? (multiple answers possible, please specify in answer box) [in case of already known vesicoureteral reflux (VUR)][Comment]

Are there situations or clinical findings in the patient where you deviate from your prophylaxis standard (medication, dose, duration, age of the patient)? (multiple answers possible, please specify in answer box) [in patients with recurrent urinary tract infections (UTI)]
No

Are there situations or clinical findings in the patient where you deviate from your prophylaxis standard (medication, dose, duration, age of the patient)? (multiple answers possible, please specify in answer box) [in patients with recurrent urinary tract infections (UTI)][Comment]

Are there situations or clinical findings in the patient where you deviate from your prophylaxis standard (medication, dose, duration, age of the patient)? (multiple answers possible, please specify in answer box) [if multidrug-resistant organisms (MDRO) are detected in urine cultures]
No

Are there situations or clinical findings in the patient where you deviate from your prophylaxis standard (medication, dose, duration, age of the patient)? (multiple answers possible, please specify in answer box) [if multidrug-resistant organisms (MDRO) are detected in urine cultures][Comment]

Are there situations or clinical findings in the patient where you deviate from your prophylaxis standard (medication, dose, duration, age of the patient)? (multiple answers possible, please specify in answer box) [in newborns]
No

Are there situations or clinical findings in the patient where you deviate from your prophylaxis standard (medication, dose, duration, age of the patient)? (multiple answers possible, please specify in answer box) [in newborns][Comment]

Are there situations or clinical findings in the patient where you deviate from your prophylaxis standard (medication, dose, duration, age of the patient)? (multiple answers possible, please specify in answer box) [Other]
Nein

Are there situations or clinical findings in the patient where you deviate from your prophylaxis standard (medication, dose, duration, age of the patient)? (multiple answers possible, please specify in answer box) [Other comment]

Are there situations in which you use intravenous prophylaxis?
No

Voiding analysis

Are there differences in antibiotic prophylaxis between VUS and VCUG in your department?
No

Is a non-pathological urine strip test required in preparation for VUS/VCUG in your department?
Yes

What is the maximum amount of days accepted for urine strip test to be performed prior to the exam?
bei Untersuchung

Is a non-pathological urine culture required in preparation for VUS/VCUG in your department?
No

Do patients only receive post-procedure prophylaxis if vesicoureteral reflux (VUR) is detected in VUS/VCUG?
No

Do other MUS/MCU findings (e.g. urethral valves) have an influence on post-procedure prophylaxis?
No

End of survey

Thank you for taking the time to answer this survey!

May we contact you personally if we have further questions?
No

Survey response 45

Response ID
46

Date submitted
2024-11-20 18:55:06

Last page
5

Start language
de

Seed
1253169131

Date started
2024-11-20 18:45:39

Date last action
2024-11-20 18:55:06

Standard Operation Procedure

Who fills out this survey?
Pediatric Radiologist

Who fills out this survey? [Other]

In which type of hospital are you employed?
University Hospital

In which type of hospital are you employed? [Other]

In which country do you work?
Deutschland

Does your department have a defined protocol for infection prophylaxis in the context of VUS/VCUG?
Yes

Has this protocol been transformed into a written standard operating procedure (SOP)?
Yes

Which department is the author of the SOP for infection prophylaxis in VUS/VCUG?
Pediatric surgery

Which department is the author of the SOP for infection prophylaxis in VUS/VCUG? [Other]

Antibiotic Prophylaxis

Are there situations/constellations in which you do not use antibiotic prophylaxis for VUS/VCUG?
No

Please specify these situations/constellations: [girl with first uncomplicated UTI without urinary tract dilation in ultrasound]
N/A

Please specify these situations/constellations: [boy with first uncomplicated UTI without urinary tract dilation in ultrasound]
N/A

Please specify these situations/constellations: [in patients with low grade urinary tract dilation (up to 2nd grade) and no UTI in medical history]
N/A

If you use antibiotic prophylaxis: which mediaction do you use? (multiple answers and subdivision into age groups possible) [Trimethoprim]
Yes

If you use antibiotic prophylaxis: which mediaction do you use? (multiple answers and subdivision into age groups possible) [Nitrofurantoin]
Yes

If you use antibiotic prophylaxis: which mediaction do you use? (multiple answers and subdivision into age groups possible) [Amoxicillin]
No

If you use antibiotic prophylaxis: which mediaction do you use? (multiple answers and subdivision into age groups possible) [Cefaclor]
Yes

If you use antibiotic prophylaxis: which mediaction do you use? (multiple answers and subdivision into age groups possible) [Amoxicillin/clavulanic acid]
No

If you use antibiotic prophylaxis: which mediaction do you use? (multiple answers and subdivision into age groups possible) [Other]
No

Trimethoprim: in what dosage?
prophylactic (2 mg/kg bodyweight/d)

Trimethoprim: in what dosage? [Other]

Trimethoprim: for which age group do you use trimethoprim?
Alle Altersklassen

Trimethoprim: which time schedule do you use? [Day(s) before VUS/VCUG, how many?]
No

Trimethoprim: which time schedule do you use? [Day(s) before VUS/VCUG, how many?][Comment]

Trimethoprim: which time schedule do you use? [Day of VUS/VCUG]
Yes

Trimethoprim: which time schedule do you use? [Day of VUS/VCUG][Comment]

Trimethoprim: which time schedule do you use? [Day(s) after VUS/VCUG, how many?]
Yes

Trimethoprim: which time schedule do you use? [Day(s) after VUS/VCUG, how many?][Comment]
3

Nitrofurantoin: in what dosage?
prophylactic (1-2 mg/kg bodyweight/d)

Nitrofurantoin: in what dosage? [Other]

Nitrofurantoin: for which age group do you use nitrofurantoin?
Alle Altersklassen

Nitrofurantoin: which time schedule do you use? [Day(s) before VUS/VCUG, how many?]
No

Nitrofurantoin: which time schedule do you use? [Day(s) before VUS/VCUG, how many?][Comment]

Nitrofurantoin: which time schedule do you use? [Day of VUS/VCUG]
Yes

Nitrofurantoin: which time schedule do you use? [Day of VUS/VCUG][Comment]

Nitrofurantoin: which time schedule do you use? [Day(s) after VUS/VCUG, how many?]
Yes

Nitrofurantoin: which time schedule do you use? [Day(s) after VUS/VCUG, how many?][Comment]
3

Amoxicillin: which time schedule do you use? [Day(s) before VUS/VCUG, how many?]
N/A

Amoxicillin: which time schedule do you use? [Day of VUS/VCUG]
N/A

Amoxicillin: which time schedule do you use? [Day(s) after VUS/VCUG, how many?]
N/A

Cefaclor: in what dosage?
prophylactic (10 mg/kg bodyweight/d)

Cefaclor: in what dosage? [Other]

Cefaclor: for which age group do you use cefaclor?
Alle Altersklassen

Cefaclor: which time schedule do you use? [Days(s) before VUS/VCUG, how many?]
No

Cefaclor: which time schedule do you use? [Days(s) before VUS/VCUG, how many?][Comment]

Cefaclor: which time schedule do you use? [Day of VUS/VCUG]
Yes

Cefaclor: which time schedule do you use? [Day of VUS/VCUG][Comment]

Cefaclor: which time schedule do you use? [Day(s) after VUS/VCUG, how many?]
Yes

Cefaclor: which time schedule do you use? [Day(s) after VUS/VCUG, how many?][Comment]
3

Amoxicillin/clavulanic acid: in what dosage? (Amoxicillin portion) [prophylaktic (10-20 mg/Kg bodyweight/d)]
N/A

Amoxicillin/clavulanic acid: in what dosage? (Amoxicillin portion) [double prophylactic (20-40 mg/Kg bodyweight/d)]
N/A

Amoxicillin/clavulanic acid: in what dosage? (Amoxicillin portion) [therapeutic (80-100 mg/Kg bodyweight/d)]
N/A

Amoxicillin/clavulanic acid: which time schedule do you use? [Day(s) before VUS/VCUG]
N/A

Amoxicillin/clavulanic acid: which time schedule do you use? [Day of VUS/VCUG]
N/A

Amoxicillin/clavulanic acid: which time schedule do you use? [Day(s) after VUS/VCUG]
N/A

Other: which time schedule do you use? [Day(s) before VUS/VCUG, how many?]
N/A

Other: which time schedule do you use? [Day of VUS/VCUG]
N/A

Other: which time schedule do you use? [Day(s) after VUS/VCUG, how many?]
N/A

Indication for prophylaxis

Which patients receive periinterventional antibiotic prophylaxis? (multiple answers possible) [all]
Yes

Which patients receive periinterventional antibiotic prophylaxis? (multiple answers possible) [patients post urinary tract infection/pyelonephritis/urosepsis]
N/A

Which patients receive periinterventional antibiotic prophylaxis? (multiple answers possible) [patients with urinary tract dilation in ultrasound]
N/A

Which patients receive periinterventional antibiotic prophylaxis? (multiple answers possible) [patients with prenatal urinary tract dilation]
N/A

Which patients receive periinterventional antibiotic prophylaxis? (multiple answers possible) [patients post VUR surgery]
N/A

Which patients receive periinterventional antibiotic prophylaxis? (multiple answers possible) [none]
N/A

Are there situations or clinical findings in the patient where you deviate from your prophylaxis standard (medication, dose, duration, age of the patient)? (multiple answers possible, please specify in answer box) [in case of already known vesicoureteral reflux (VUR)]
Yes

Are there situations or clinical findings in the patient where you deviate from your prophylaxis standard (medication, dose, duration, age of the patient)? (multiple answers possible, please specify in answer box) [in case of already known vesicoureteral reflux (VUR)][Comment]
Keine Änderung

Are there situations or clinical findings in the patient where you deviate from your prophylaxis standard (medication, dose, duration, age of the patient)? (multiple answers possible, please specify in answer box) [in patients with recurrent urinary tract infections (UTI)]
Yes

Are there situations or clinical findings in the patient where you deviate from your prophylaxis standard (medication, dose, duration, age of the patient)? (multiple answers possible, please specify in answer box) [in patients with recurrent urinary tract infections (UTI)][Comment]
Keine Änderung

Are there situations or clinical findings in the patient where you deviate from your prophylaxis standard (medication, dose, duration, age of the patient)? (multiple answers possible, please specify in answer box) [if multidrug-resistant organisms (MDRO) are detected in urine cultures]
Yes

Are there situations or clinical findings in the patient where you deviate from your prophylaxis standard (medication, dose, duration, age of the patient)? (multiple answers possible, please specify in answer box) [if multidrug-resistant organisms (MDRO) are detected in urine cultures][Comment]
Keine Änderung

Are there situations or clinical findings in the patient where you deviate from your prophylaxis standard (medication, dose, duration, age of the patient)? (multiple answers possible, please specify in answer box) [in newborns]
Yes

Are there situations or clinical findings in the patient where you deviate from your prophylaxis standard (medication, dose, duration, age of the patient)? (multiple answers possible, please specify in answer box) [in newborns][Comment]
Keine Änderung

Are there situations or clinical findings in the patient where you deviate from your prophylaxis standard (medication, dose, duration, age of the patient)? (multiple answers possible, please specify in answer box) [Other]

Are there situations or clinical findings in the patient where you deviate from your prophylaxis standard (medication, dose, duration, age of the patient)? (multiple answers possible, please specify in answer box) [Other comment]

Are there situations in which you use intravenous prophylaxis?
No

Voiding analysis

Are there differences in antibiotic prophylaxis between VUS and VCUG in your department?
No

Is a non-pathological urine strip test required in preparation for VUS/VCUG in your department?
Yes

What is the maximum amount of days accepted for urine strip test to be performed prior to the exam?
3

Is a non-pathological urine culture required in preparation for VUS/VCUG in your department?
No

Do patients only receive post-procedure prophylaxis if vesicoureteral reflux (VUR) is detected in VUS/VCUG?
No

Do other MUS/MCU findings (e.g. urethral valves) have an influence on post-procedure prophylaxis?
No

End of survey

Thank you for taking the time to answer this survey!

May we contact you personally if we have further questions?
Yes

E-mail address:
renz.diane@mh-hannover.de

Survey response 46

Response ID
47

Last page
1

Start language
en

Seed
411118577

Date started
2024-11-21 07:07:50

Date last action
2024-11-21 07:10:26

Standard Operation Procedure

Who fills out this survey?
Pediatric Radiologist

Who fills out this survey? [Other]

In which type of hospital are you employed?
University Hospital

In which type of hospital are you employed? [Other]

In which country do you work?
Usa

Does your department have a defined protocol for infection prophylaxis in the context of VUS/VCUG?
No

Has this protocol been transformed into a written standard operating procedure (SOP)?
N/A

Antibiotic Prophylaxis

Are there situations/constellations in which you do not use antibiotic prophylaxis for VUS/VCUG?
Yes

Please specify these situations/constellations: [girl with first uncomplicated UTI without urinary tract dilation in ultrasound]
No

Please specify these situations/constellations: [boy with first uncomplicated UTI without urinary tract dilation in ultrasound]
No

Please specify these situations/constellations: [in patients with low grade urinary tract dilation (up to 2nd grade) and no UTI in medical history]
No

Please specify these situations/constellations: [Other]

If you use antibiotic prophylaxis: which mediaction do you use? (multiple answers and subdivision into age groups possible) [Trimethoprim]
No

If you use antibiotic prophylaxis: which mediaction do you use? (multiple answers and subdivision into age groups possible) [Nitrofurantoin]
No

If you use antibiotic prophylaxis: which mediaction do you use? (multiple answers and subdivision into age groups possible) [Amoxicillin]
No

If you use antibiotic prophylaxis: which mediaction do you use? (multiple answers and subdivision into age groups possible) [Cefaclor]
No

If you use antibiotic prophylaxis: which mediaction do you use? (multiple answers and subdivision into age groups possible) [Amoxicillin/clavulanic acid]
No

If you use antibiotic prophylaxis: which mediaction do you use? (multiple answers and subdivision into age groups possible) [Other]
Yes

Trimethoprim: which time schedule do you use? [Day(s) before VUS/VCUG, how many?]
N/A

Trimethoprim: which time schedule do you use? [Day of VUS/VCUG]
N/A

Trimethoprim: which time schedule do you use? [Day(s) after VUS/VCUG, how many?]
N/A

Nitrofurantoin: which time schedule do you use? [Day(s) before VUS/VCUG, how many?]
N/A

Nitrofurantoin: which time schedule do you use? [Day of VUS/VCUG]
N/A

Nitrofurantoin: which time schedule do you use? [Day(s) after VUS/VCUG, how many?]
N/A

Amoxicillin: which time schedule do you use? [Day(s) before VUS/VCUG, how many?]
N/A

Amoxicillin: which time schedule do you use? [Day of VUS/VCUG]
N/A

Amoxicillin: which time schedule do you use? [Day(s) after VUS/VCUG, how many?]
N/A

Cefaclor: which time schedule do you use? [Days(s) before VUS/VCUG, how many?]
N/A

Cefaclor: which time schedule do you use? [Day of VUS/VCUG]
N/A

Cefaclor: which time schedule do you use? [Day(s) after VUS/VCUG, how many?]
N/A

Amoxicillin/clavulanic acid: in what dosage? (Amoxicillin portion) [prophylaktic (10-20 mg/Kg bodyweight/d)]
N/A

Amoxicillin/clavulanic acid: in what dosage? (Amoxicillin portion) [double prophylactic (20-40 mg/Kg bodyweight/d)]
N/A

Amoxicillin/clavulanic acid: in what dosage? (Amoxicillin portion) [therapeutic (80-100 mg/Kg bodyweight/d)]
N/A

Amoxicillin/clavulanic acid: which time schedule do you use? [Day(s) before VUS/VCUG]
N/A

Amoxicillin/clavulanic acid: which time schedule do you use? [Day of VUS/VCUG]
N/A

Amoxicillin/clavulanic acid: which time schedule do you use? [Day(s) after VUS/VCUG]
N/A

Other: which medication and in what dosage?
We never give antibiotic prophylaxis

Other: for which age group do you use this medication?
None

Other: which time schedule do you use? [Day(s) before VUS/VCUG, how many?]
Yes

Other: which time schedule do you use? [Day(s) before VUS/VCUG, how many?][Comment]
We do not give prophylaxis and I have never heeds of anyone else doing it

Other: which time schedule do you use? [Day of VUS/VCUG]
No

Other: which time schedule do you use? [Day of VUS/VCUG][Comment]

Other: which time schedule do you use? [Day(s) after VUS/VCUG, how many?]
No

Other: which time schedule do you use? [Day(s) after VUS/VCUG, how many?][Comment]

Indication for prophylaxis

Which patients receive periinterventional antibiotic prophylaxis? (multiple answers possible) [all]
N/A

Which patients receive periinterventional antibiotic prophylaxis? (multiple answers possible) [patients post urinary tract infection/pyelonephritis/urosepsis]
N/A

Which patients receive periinterventional antibiotic prophylaxis? (multiple answers possible) [patients with urinary tract dilation in ultrasound]
N/A

Which patients receive periinterventional antibiotic prophylaxis? (multiple answers possible) [patients with prenatal urinary tract dilation]
N/A

Which patients receive periinterventional antibiotic prophylaxis? (multiple answers possible) [patients post VUR surgery]
N/A

Which patients receive periinterventional antibiotic prophylaxis? (multiple answers possible) [none]
N/A

Are there situations or clinical findings in the patient where you deviate from your prophylaxis standard (medication, dose, duration, age of the patient)? (multiple answers possible, please specify in answer box) [in case of already known vesicoureteral reflux (VUR)]
N/A

Are there situations or clinical findings in the patient where you deviate from your prophylaxis standard (medication, dose, duration, age of the patient)? (multiple answers possible, please specify in answer box) [in patients with recurrent urinary tract infections (UTI)]
N/A

Are there situations or clinical findings in the patient where you deviate from your prophylaxis standard (medication, dose, duration, age of the patient)? (multiple answers possible, please specify in answer box) [if multidrug-resistant organisms (MDRO) are detected in urine cultures]
N/A

Are there situations or clinical findings in the patient where you deviate from your prophylaxis standard (medication, dose, duration, age of the patient)? (multiple answers possible, please specify in answer box) [in newborns]
N/A

Are there situations in which you use intravenous prophylaxis?
N/A

Voiding analysis

Are there differences in antibiotic prophylaxis between VUS and VCUG in your department?
N/A

Is a non-pathological urine strip test required in preparation for VUS/VCUG in your department?
N/A

Is a non-pathological urine culture required in preparation for VUS/VCUG in your department?
N/A

Do patients only receive post-procedure prophylaxis if vesicoureteral reflux (VUR) is detected in VUS/VCUG?
N/A

Do other MUS/MCU findings (e.g. urethral valves) have an influence on post-procedure prophylaxis?
N/A

End of survey

May we contact you personally if we have further questions?
N/A

Survey response 47

Response ID
48

Last page
0

Start language
de

Seed
693779131

Date started
2024-11-21 08:15:30

Date last action
2024-11-21 08:15:30

Standard Operation Procedure

Does your department have a defined protocol for infection prophylaxis in the context of VUS/VCUG?
N/A

Has this protocol been transformed into a written standard operating procedure (SOP)?
N/A

Antibiotic Prophylaxis

Are there situations/constellations in which you do not use antibiotic prophylaxis for VUS/VCUG?
N/A

Please specify these situations/constellations: [girl with first uncomplicated UTI without urinary tract dilation in ultrasound]
N/A

Please specify these situations/constellations: [boy with first uncomplicated UTI without urinary tract dilation in ultrasound]
N/A

Please specify these situations/constellations: [in patients with low grade urinary tract dilation (up to 2nd grade) and no UTI in medical history]
N/A

If you use antibiotic prophylaxis: which mediaction do you use? (multiple answers and subdivision into age groups possible) [Trimethoprim]
N/A

If you use antibiotic prophylaxis: which mediaction do you use? (multiple answers and subdivision into age groups possible) [Nitrofurantoin]
N/A

If you use antibiotic prophylaxis: which mediaction do you use? (multiple answers and subdivision into age groups possible) [Amoxicillin]
N/A

If you use antibiotic prophylaxis: which mediaction do you use? (multiple answers and subdivision into age groups possible) [Cefaclor]
N/A

If you use antibiotic prophylaxis: which mediaction do you use? (multiple answers and subdivision into age groups possible) [Amoxicillin/clavulanic acid]
N/A

If you use antibiotic prophylaxis: which mediaction do you use? (multiple answers and subdivision into age groups possible) [Other]
N/A

Trimethoprim: which time schedule do you use? [Day(s) before VUS/VCUG, how many?]
N/A

Trimethoprim: which time schedule do you use? [Day of VUS/VCUG]
N/A

Trimethoprim: which time schedule do you use? [Day(s) after VUS/VCUG, how many?]
N/A

Nitrofurantoin: which time schedule do you use? [Day(s) before VUS/VCUG, how many?]
N/A

Nitrofurantoin: which time schedule do you use? [Day of VUS/VCUG]
N/A

Nitrofurantoin: which time schedule do you use? [Day(s) after VUS/VCUG, how many?]
N/A

Amoxicillin: which time schedule do you use? [Day(s) before VUS/VCUG, how many?]
N/A

Amoxicillin: which time schedule do you use? [Day of VUS/VCUG]
N/A

Amoxicillin: which time schedule do you use? [Day(s) after VUS/VCUG, how many?]
N/A

Cefaclor: which time schedule do you use? [Days(s) before VUS/VCUG, how many?]
N/A

Cefaclor: which time schedule do you use? [Day of VUS/VCUG]
N/A

Cefaclor: which time schedule do you use? [Day(s) after VUS/VCUG, how many?]
N/A

Amoxicillin/clavulanic acid: in what dosage? (Amoxicillin portion) [prophylaktic (10-20 mg/Kg bodyweight/d)]
N/A

Amoxicillin/clavulanic acid: in what dosage? (Amoxicillin portion) [double prophylactic (20-40 mg/Kg bodyweight/d)]
N/A

Amoxicillin/clavulanic acid: in what dosage? (Amoxicillin portion) [therapeutic (80-100 mg/Kg bodyweight/d)]
N/A

Amoxicillin/clavulanic acid: which time schedule do you use? [Day(s) before VUS/VCUG]
N/A

Amoxicillin/clavulanic acid: which time schedule do you use? [Day of VUS/VCUG]
N/A

Amoxicillin/clavulanic acid: which time schedule do you use? [Day(s) after VUS/VCUG]
N/A

Other: which time schedule do you use? [Day(s) before VUS/VCUG, how many?]
N/A

Other: which time schedule do you use? [Day of VUS/VCUG]
N/A

Other: which time schedule do you use? [Day(s) after VUS/VCUG, how many?]
N/A

Indication for prophylaxis

Which patients receive periinterventional antibiotic prophylaxis? (multiple answers possible) [all]
N/A

Which patients receive periinterventional antibiotic prophylaxis? (multiple answers possible) [patients post urinary tract infection/pyelonephritis/urosepsis]
N/A

Which patients receive periinterventional antibiotic prophylaxis? (multiple answers possible) [patients with urinary tract dilation in ultrasound]
N/A

Which patients receive periinterventional antibiotic prophylaxis? (multiple answers possible) [patients with prenatal urinary tract dilation]
N/A

Which patients receive periinterventional antibiotic prophylaxis? (multiple answers possible) [patients post VUR surgery]
N/A

Which patients receive periinterventional antibiotic prophylaxis? (multiple answers possible) [none]
N/A

Are there situations or clinical findings in the patient where you deviate from your prophylaxis standard (medication, dose, duration, age of the patient)? (multiple answers possible, please specify in answer box) [in case of already known vesicoureteral reflux (VUR)]
N/A

Are there situations or clinical findings in the patient where you deviate from your prophylaxis standard (medication, dose, duration, age of the patient)? (multiple answers possible, please specify in answer box) [in patients with recurrent urinary tract infections (UTI)]
N/A

Are there situations or clinical findings in the patient where you deviate from your prophylaxis standard (medication, dose, duration, age of the patient)? (multiple answers possible, please specify in answer box) [if multidrug-resistant organisms (MDRO) are detected in urine cultures]
N/A

Are there situations or clinical findings in the patient where you deviate from your prophylaxis standard (medication, dose, duration, age of the patient)? (multiple answers possible, please specify in answer box) [in newborns]
N/A

Are there situations in which you use intravenous prophylaxis?
N/A

Voiding analysis

Are there differences in antibiotic prophylaxis between VUS and VCUG in your department?
N/A

Is a non-pathological urine strip test required in preparation for VUS/VCUG in your department?
N/A

Is a non-pathological urine culture required in preparation for VUS/VCUG in your department?
N/A

Do patients only receive post-procedure prophylaxis if vesicoureteral reflux (VUR) is detected in VUS/VCUG?
N/A

Do other MUS/MCU findings (e.g. urethral valves) have an influence on post-procedure prophylaxis?
N/A

End of survey

May we contact you personally if we have further questions?
N/A

Survey response 48

Response ID
49

Last page
2

Start language
en

Seed
694238888

Date started
2024-11-21 12:00:44

Date last action
2024-11-21 12:02:47

Standard Operation Procedure

Who fills out this survey?
Pediatric Radiologist

Who fills out this survey? [Other]

In which type of hospital are you employed?
University Hospital

In which type of hospital are you employed? [Other]

In which country do you work?
Canada

Does your department have a defined protocol for infection prophylaxis in the context of VUS/VCUG?
No

Has this protocol been transformed into a written standard operating procedure (SOP)?
N/A

Antibiotic Prophylaxis

Are there situations/constellations in which you do not use antibiotic prophylaxis for VUS/VCUG?
Yes

Please specify these situations/constellations: [girl with first uncomplicated UTI without urinary tract dilation in ultrasound]
Yes

Please specify these situations/constellations: [boy with first uncomplicated UTI without urinary tract dilation in ultrasound]
Yes

Please specify these situations/constellations: [in patients with low grade urinary tract dilation (up to 2nd grade) and no UTI in medical history]
Yes

Please specify these situations/constellations: [Other]
No routine Abx prophylaxis

If you use antibiotic prophylaxis: which mediaction do you use? (multiple answers and subdivision into age groups possible) [Trimethoprim]
No

If you use antibiotic prophylaxis: which mediaction do you use? (multiple answers and subdivision into age groups possible) [Nitrofurantoin]
No

If you use antibiotic prophylaxis: which mediaction do you use? (multiple answers and subdivision into age groups possible) [Amoxicillin]
No

If you use antibiotic prophylaxis: which mediaction do you use? (multiple answers and subdivision into age groups possible) [Cefaclor]
No

If you use antibiotic prophylaxis: which mediaction do you use? (multiple answers and subdivision into age groups possible) [Amoxicillin/clavulanic acid]
No

If you use antibiotic prophylaxis: which mediaction do you use? (multiple answers and subdivision into age groups possible) [Other]
No

Trimethoprim: which time schedule do you use? [Day(s) before VUS/VCUG, how many?]
N/A

Trimethoprim: which time schedule do you use? [Day of VUS/VCUG]
N/A

Trimethoprim: which time schedule do you use? [Day(s) after VUS/VCUG, how many?]
N/A

Nitrofurantoin: which time schedule do you use? [Day(s) before VUS/VCUG, how many?]
N/A

Nitrofurantoin: which time schedule do you use? [Day of VUS/VCUG]
N/A

Nitrofurantoin: which time schedule do you use? [Day(s) after VUS/VCUG, how many?]
N/A

Amoxicillin: which time schedule do you use? [Day(s) before VUS/VCUG, how many?]
N/A

Amoxicillin: which time schedule do you use? [Day of VUS/VCUG]
N/A

Amoxicillin: which time schedule do you use? [Day(s) after VUS/VCUG, how many?]
N/A

Cefaclor: which time schedule do you use? [Days(s) before VUS/VCUG, how many?]
N/A

Cefaclor: which time schedule do you use? [Day of VUS/VCUG]
N/A

Cefaclor: which time schedule do you use? [Day(s) after VUS/VCUG, how many?]
N/A

Amoxicillin/clavulanic acid: in what dosage? (Amoxicillin portion) [prophylaktic (10-20 mg/Kg bodyweight/d)]
N/A

Amoxicillin/clavulanic acid: in what dosage? (Amoxicillin portion) [double prophylactic (20-40 mg/Kg bodyweight/d)]
N/A

Amoxicillin/clavulanic acid: in what dosage? (Amoxicillin portion) [therapeutic (80-100 mg/Kg bodyweight/d)]
N/A

Amoxicillin/clavulanic acid: which time schedule do you use? [Day(s) before VUS/VCUG]
N/A

Amoxicillin/clavulanic acid: which time schedule do you use? [Day of VUS/VCUG]
N/A

Amoxicillin/clavulanic acid: which time schedule do you use? [Day(s) after VUS/VCUG]
N/A

Other: which time schedule do you use? [Day(s) before VUS/VCUG, how many?]
N/A

Other: which time schedule do you use? [Day of VUS/VCUG]
N/A

Other: which time schedule do you use? [Day(s) after VUS/VCUG, how many?]
N/A

Indication for prophylaxis

Which patients receive periinterventional antibiotic prophylaxis? (multiple answers possible) [all]
No

Which patients receive periinterventional antibiotic prophylaxis? (multiple answers possible) [patients post urinary tract infection/pyelonephritis/urosepsis]
No

Which patients receive periinterventional antibiotic prophylaxis? (multiple answers possible) [patients with urinary tract dilation in ultrasound]
No

Which patients receive periinterventional antibiotic prophylaxis? (multiple answers possible) [patients with prenatal urinary tract dilation]
No

Which patients receive periinterventional antibiotic prophylaxis? (multiple answers possible) [patients post VUR surgery]
No

Which patients receive periinterventional antibiotic prophylaxis? (multiple answers possible) [none]
Yes

Which patients receive periinterventional antibiotic prophylaxis? (multiple answers possible) [Other]

Are there situations or clinical findings in the patient where you deviate from your prophylaxis standard (medication, dose, duration, age of the patient)? (multiple answers possible, please specify in answer box) [in case of already known vesicoureteral reflux (VUR)]
No

Are there situations or clinical findings in the patient where you deviate from your prophylaxis standard (medication, dose, duration, age of the patient)? (multiple answers possible, please specify in answer box) [in case of already known vesicoureteral reflux (VUR)][Comment]

Are there situations or clinical findings in the patient where you deviate from your prophylaxis standard (medication, dose, duration, age of the patient)? (multiple answers possible, please specify in answer box) [in patients with recurrent urinary tract infections (UTI)]
No

Are there situations or clinical findings in the patient where you deviate from your prophylaxis standard (medication, dose, duration, age of the patient)? (multiple answers possible, please specify in answer box) [in patients with recurrent urinary tract infections (UTI)][Comment]

Are there situations or clinical findings in the patient where you deviate from your prophylaxis standard (medication, dose, duration, age of the patient)? (multiple answers possible, please specify in answer box) [if multidrug-resistant organisms (MDRO) are detected in urine cultures]
No

Are there situations or clinical findings in the patient where you deviate from your prophylaxis standard (medication, dose, duration, age of the patient)? (multiple answers possible, please specify in answer box) [if multidrug-resistant organisms (MDRO) are detected in urine cultures][Comment]

Are there situations or clinical findings in the patient where you deviate from your prophylaxis standard (medication, dose, duration, age of the patient)? (multiple answers possible, please specify in answer box) [in newborns]
No

Are there situations or clinical findings in the patient where you deviate from your prophylaxis standard (medication, dose, duration, age of the patient)? (multiple answers possible, please specify in answer box) [in newborns][Comment]

Are there situations or clinical findings in the patient where you deviate from your prophylaxis standard (medication, dose, duration, age of the patient)? (multiple answers possible, please specify in answer box) [Other]

Are there situations or clinical findings in the patient where you deviate from your prophylaxis standard (medication, dose, duration, age of the patient)? (multiple answers possible, please specify in answer box) [Other comment]

Are there situations in which you use intravenous prophylaxis?
No

Voiding analysis

Are there differences in antibiotic prophylaxis between VUS and VCUG in your department?
N/A

Is a non-pathological urine strip test required in preparation for VUS/VCUG in your department?
N/A

Is a non-pathological urine culture required in preparation for VUS/VCUG in your department?
N/A

Do patients only receive post-procedure prophylaxis if vesicoureteral reflux (VUR) is detected in VUS/VCUG?
N/A

Do other MUS/MCU findings (e.g. urethral valves) have an influence on post-procedure prophylaxis?
N/A

End of survey

May we contact you personally if we have further questions?
N/A

Survey response 49

Response ID
50

Last page
0

Start language
en

Seed
633228888

Date started
2024-11-21 23:18:41

Date last action
2024-11-21 23:18:41

Standard Operation Procedure

Does your department have a defined protocol for infection prophylaxis in the context of VUS/VCUG?
N/A

Has this protocol been transformed into a written standard operating procedure (SOP)?
N/A

Antibiotic Prophylaxis

Are there situations/constellations in which you do not use antibiotic prophylaxis for VUS/VCUG?
N/A

Please specify these situations/constellations: [girl with first uncomplicated UTI without urinary tract dilation in ultrasound]
N/A

Please specify these situations/constellations: [boy with first uncomplicated UTI without urinary tract dilation in ultrasound]
N/A

Please specify these situations/constellations: [in patients with low grade urinary tract dilation (up to 2nd grade) and no UTI in medical history]
N/A

If you use antibiotic prophylaxis: which mediaction do you use? (multiple answers and subdivision into age groups possible) [Trimethoprim]
N/A

If you use antibiotic prophylaxis: which mediaction do you use? (multiple answers and subdivision into age groups possible) [Nitrofurantoin]
N/A

If you use antibiotic prophylaxis: which mediaction do you use? (multiple answers and subdivision into age groups possible) [Amoxicillin]
N/A

If you use antibiotic prophylaxis: which mediaction do you use? (multiple answers and subdivision into age groups possible) [Cefaclor]
N/A

If you use antibiotic prophylaxis: which mediaction do you use? (multiple answers and subdivision into age groups possible) [Amoxicillin/clavulanic acid]
N/A

If you use antibiotic prophylaxis: which mediaction do you use? (multiple answers and subdivision into age groups possible) [Other]
N/A

Trimethoprim: which time schedule do you use? [Day(s) before VUS/VCUG, how many?]
N/A

Trimethoprim: which time schedule do you use? [Day of VUS/VCUG]
N/A

Trimethoprim: which time schedule do you use? [Day(s) after VUS/VCUG, how many?]
N/A

Nitrofurantoin: which time schedule do you use? [Day(s) before VUS/VCUG, how many?]
N/A

Nitrofurantoin: which time schedule do you use? [Day of VUS/VCUG]
N/A

Nitrofurantoin: which time schedule do you use? [Day(s) after VUS/VCUG, how many?]
N/A

Amoxicillin: which time schedule do you use? [Day(s) before VUS/VCUG, how many?]
N/A

Amoxicillin: which time schedule do you use? [Day of VUS/VCUG]
N/A

Amoxicillin: which time schedule do you use? [Day(s) after VUS/VCUG, how many?]
N/A

Cefaclor: which time schedule do you use? [Days(s) before VUS/VCUG, how many?]
N/A

Cefaclor: which time schedule do you use? [Day of VUS/VCUG]
N/A

Cefaclor: which time schedule do you use? [Day(s) after VUS/VCUG, how many?]
N/A

Amoxicillin/clavulanic acid: in what dosage? (Amoxicillin portion) [prophylaktic (10-20 mg/Kg bodyweight/d)]
N/A

Amoxicillin/clavulanic acid: in what dosage? (Amoxicillin portion) [double prophylactic (20-40 mg/Kg bodyweight/d)]
N/A

Amoxicillin/clavulanic acid: in what dosage? (Amoxicillin portion) [therapeutic (80-100 mg/Kg bodyweight/d)]
N/A

Amoxicillin/clavulanic acid: which time schedule do you use? [Day(s) before VUS/VCUG]
N/A

Amoxicillin/clavulanic acid: which time schedule do you use? [Day of VUS/VCUG]
N/A

Amoxicillin/clavulanic acid: which time schedule do you use? [Day(s) after VUS/VCUG]
N/A

Other: which time schedule do you use? [Day(s) before VUS/VCUG, how many?]
N/A

Other: which time schedule do you use? [Day of VUS/VCUG]
N/A

Other: which time schedule do you use? [Day(s) after VUS/VCUG, how many?]
N/A

Indication for prophylaxis

Which patients receive periinterventional antibiotic prophylaxis? (multiple answers possible) [all]
N/A

Which patients receive periinterventional antibiotic prophylaxis? (multiple answers possible) [patients post urinary tract infection/pyelonephritis/urosepsis]
N/A

Which patients receive periinterventional antibiotic prophylaxis? (multiple answers possible) [patients with urinary tract dilation in ultrasound]
N/A

Which patients receive periinterventional antibiotic prophylaxis? (multiple answers possible) [patients with prenatal urinary tract dilation]
N/A

Which patients receive periinterventional antibiotic prophylaxis? (multiple answers possible) [patients post VUR surgery]
N/A

Which patients receive periinterventional antibiotic prophylaxis? (multiple answers possible) [none]
N/A

Are there situations or clinical findings in the patient where you deviate from your prophylaxis standard (medication, dose, duration, age of the patient)? (multiple answers possible, please specify in answer box) [in case of already known vesicoureteral reflux (VUR)]
N/A

Are there situations or clinical findings in the patient where you deviate from your prophylaxis standard (medication, dose, duration, age of the patient)? (multiple answers possible, please specify in answer box) [in patients with recurrent urinary tract infections (UTI)]
N/A

Are there situations or clinical findings in the patient where you deviate from your prophylaxis standard (medication, dose, duration, age of the patient)? (multiple answers possible, please specify in answer box) [if multidrug-resistant organisms (MDRO) are detected in urine cultures]
N/A

Are there situations or clinical findings in the patient where you deviate from your prophylaxis standard (medication, dose, duration, age of the patient)? (multiple answers possible, please specify in answer box) [in newborns]
N/A

Are there situations in which you use intravenous prophylaxis?
N/A

Voiding analysis

Are there differences in antibiotic prophylaxis between VUS and VCUG in your department?
N/A

Is a non-pathological urine strip test required in preparation for VUS/VCUG in your department?
N/A

Is a non-pathological urine culture required in preparation for VUS/VCUG in your department?
N/A

Do patients only receive post-procedure prophylaxis if vesicoureteral reflux (VUR) is detected in VUS/VCUG?
N/A

Do other MUS/MCU findings (e.g. urethral valves) have an influence on post-procedure prophylaxis?
N/A

End of survey

May we contact you personally if we have further questions?
N/A

Survey response 50

Response ID
51

Last page
1

Start language
de

Seed
594317100

Date started
2024-11-22 07:49:31

Date last action
2024-11-22 07:50:44

Standard Operation Procedure

Who fills out this survey?
Pediatric Radiologist

Who fills out this survey? [Other]

In which type of hospital are you employed?
University Hospital

In which type of hospital are you employed? [Other]

In which country do you work?
Deutschland

Does your department have a defined protocol for infection prophylaxis in the context of VUS/VCUG?
Yes

Has this protocol been transformed into a written standard operating procedure (SOP)?
Yes

Which department is the author of the SOP for infection prophylaxis in VUS/VCUG?
Pediatric surgery

Which department is the author of the SOP for infection prophylaxis in VUS/VCUG? [Other]

Antibiotic Prophylaxis

Are there situations/constellations in which you do not use antibiotic prophylaxis for VUS/VCUG?
N/A

Please specify these situations/constellations: [girl with first uncomplicated UTI without urinary tract dilation in ultrasound]
N/A

Please specify these situations/constellations: [boy with first uncomplicated UTI without urinary tract dilation in ultrasound]
N/A

Please specify these situations/constellations: [in patients with low grade urinary tract dilation (up to 2nd grade) and no UTI in medical history]
N/A

If you use antibiotic prophylaxis: which mediaction do you use? (multiple answers and subdivision into age groups possible) [Trimethoprim]
N/A

If you use antibiotic prophylaxis: which mediaction do you use? (multiple answers and subdivision into age groups possible) [Nitrofurantoin]
N/A

If you use antibiotic prophylaxis: which mediaction do you use? (multiple answers and subdivision into age groups possible) [Amoxicillin]
N/A

If you use antibiotic prophylaxis: which mediaction do you use? (multiple answers and subdivision into age groups possible) [Cefaclor]
N/A

If you use antibiotic prophylaxis: which mediaction do you use? (multiple answers and subdivision into age groups possible) [Amoxicillin/clavulanic acid]
N/A

If you use antibiotic prophylaxis: which mediaction do you use? (multiple answers and subdivision into age groups possible) [Other]
N/A

Trimethoprim: which time schedule do you use? [Day(s) before VUS/VCUG, how many?]
N/A

Trimethoprim: which time schedule do you use? [Day of VUS/VCUG]
N/A

Trimethoprim: which time schedule do you use? [Day(s) after VUS/VCUG, how many?]
N/A

Nitrofurantoin: which time schedule do you use? [Day(s) before VUS/VCUG, how many?]
N/A

Nitrofurantoin: which time schedule do you use? [Day of VUS/VCUG]
N/A

Nitrofurantoin: which time schedule do you use? [Day(s) after VUS/VCUG, how many?]
N/A

Amoxicillin: which time schedule do you use? [Day(s) before VUS/VCUG, how many?]
N/A

Amoxicillin: which time schedule do you use? [Day of VUS/VCUG]
N/A

Amoxicillin: which time schedule do you use? [Day(s) after VUS/VCUG, how many?]
N/A

Cefaclor: which time schedule do you use? [Days(s) before VUS/VCUG, how many?]
N/A

Cefaclor: which time schedule do you use? [Day of VUS/VCUG]
N/A

Cefaclor: which time schedule do you use? [Day(s) after VUS/VCUG, how many?]
N/A

Amoxicillin/clavulanic acid: in what dosage? (Amoxicillin portion) [prophylaktic (10-20 mg/Kg bodyweight/d)]
N/A

Amoxicillin/clavulanic acid: in what dosage? (Amoxicillin portion) [double prophylactic (20-40 mg/Kg bodyweight/d)]
N/A

Amoxicillin/clavulanic acid: in what dosage? (Amoxicillin portion) [therapeutic (80-100 mg/Kg bodyweight/d)]
N/A

Amoxicillin/clavulanic acid: which time schedule do you use? [Day(s) before VUS/VCUG]
N/A

Amoxicillin/clavulanic acid: which time schedule do you use? [Day of VUS/VCUG]
N/A

Amoxicillin/clavulanic acid: which time schedule do you use? [Day(s) after VUS/VCUG]
N/A

Other: which time schedule do you use? [Day(s) before VUS/VCUG, how many?]
N/A

Other: which time schedule do you use? [Day of VUS/VCUG]
N/A

Other: which time schedule do you use? [Day(s) after VUS/VCUG, how many?]
N/A

Indication for prophylaxis

Which patients receive periinterventional antibiotic prophylaxis? (multiple answers possible) [all]
N/A

Which patients receive periinterventional antibiotic prophylaxis? (multiple answers possible) [patients post urinary tract infection/pyelonephritis/urosepsis]
N/A

Which patients receive periinterventional antibiotic prophylaxis? (multiple answers possible) [patients with urinary tract dilation in ultrasound]
N/A

Which patients receive periinterventional antibiotic prophylaxis? (multiple answers possible) [patients with prenatal urinary tract dilation]
N/A

Which patients receive periinterventional antibiotic prophylaxis? (multiple answers possible) [patients post VUR surgery]
N/A

Which patients receive periinterventional antibiotic prophylaxis? (multiple answers possible) [none]
N/A

Are there situations or clinical findings in the patient where you deviate from your prophylaxis standard (medication, dose, duration, age of the patient)? (multiple answers possible, please specify in answer box) [in case of already known vesicoureteral reflux (VUR)]
N/A

Are there situations or clinical findings in the patient where you deviate from your prophylaxis standard (medication, dose, duration, age of the patient)? (multiple answers possible, please specify in answer box) [in patients with recurrent urinary tract infections (UTI)]
N/A

Are there situations or clinical findings in the patient where you deviate from your prophylaxis standard (medication, dose, duration, age of the patient)? (multiple answers possible, please specify in answer box) [if multidrug-resistant organisms (MDRO) are detected in urine cultures]
N/A

Are there situations or clinical findings in the patient where you deviate from your prophylaxis standard (medication, dose, duration, age of the patient)? (multiple answers possible, please specify in answer box) [in newborns]
N/A

Are there situations in which you use intravenous prophylaxis?
N/A

Voiding analysis

Are there differences in antibiotic prophylaxis between VUS and VCUG in your department?
N/A

Is a non-pathological urine strip test required in preparation for VUS/VCUG in your department?
N/A

Is a non-pathological urine culture required in preparation for VUS/VCUG in your department?
N/A

Do patients only receive post-procedure prophylaxis if vesicoureteral reflux (VUR) is detected in VUS/VCUG?
N/A

Do other MUS/MCU findings (e.g. urethral valves) have an influence on post-procedure prophylaxis?
N/A

End of survey

May we contact you personally if we have further questions?
N/A

Survey response 51

Response ID
52

Last page
0

Start language
de

Seed
570808804

Date started
2024-11-23 21:00:15

Date last action
2024-11-23 21:00:15

Standard Operation Procedure

Does your department have a defined protocol for infection prophylaxis in the context of VUS/VCUG?
N/A

Has this protocol been transformed into a written standard operating procedure (SOP)?
N/A

Antibiotic Prophylaxis

Are there situations/constellations in which you do not use antibiotic prophylaxis for VUS/VCUG?
N/A

Please specify these situations/constellations: [girl with first uncomplicated UTI without urinary tract dilation in ultrasound]
N/A

Please specify these situations/constellations: [boy with first uncomplicated UTI without urinary tract dilation in ultrasound]
N/A

Please specify these situations/constellations: [in patients with low grade urinary tract dilation (up to 2nd grade) and no UTI in medical history]
N/A

If you use antibiotic prophylaxis: which mediaction do you use? (multiple answers and subdivision into age groups possible) [Trimethoprim]
N/A

If you use antibiotic prophylaxis: which mediaction do you use? (multiple answers and subdivision into age groups possible) [Nitrofurantoin]
N/A

If you use antibiotic prophylaxis: which mediaction do you use? (multiple answers and subdivision into age groups possible) [Amoxicillin]
N/A

If you use antibiotic prophylaxis: which mediaction do you use? (multiple answers and subdivision into age groups possible) [Cefaclor]
N/A

If you use antibiotic prophylaxis: which mediaction do you use? (multiple answers and subdivision into age groups possible) [Amoxicillin/clavulanic acid]
N/A

If you use antibiotic prophylaxis: which mediaction do you use? (multiple answers and subdivision into age groups possible) [Other]
N/A

Trimethoprim: which time schedule do you use? [Day(s) before VUS/VCUG, how many?]
N/A

Trimethoprim: which time schedule do you use? [Day of VUS/VCUG]
N/A

Trimethoprim: which time schedule do you use? [Day(s) after VUS/VCUG, how many?]
N/A

Nitrofurantoin: which time schedule do you use? [Day(s) before VUS/VCUG, how many?]
N/A

Nitrofurantoin: which time schedule do you use? [Day of VUS/VCUG]
N/A

Nitrofurantoin: which time schedule do you use? [Day(s) after VUS/VCUG, how many?]
N/A

Amoxicillin: which time schedule do you use? [Day(s) before VUS/VCUG, how many?]
N/A

Amoxicillin: which time schedule do you use? [Day of VUS/VCUG]
N/A

Amoxicillin: which time schedule do you use? [Day(s) after VUS/VCUG, how many?]
N/A

Cefaclor: which time schedule do you use? [Days(s) before VUS/VCUG, how many?]
N/A

Cefaclor: which time schedule do you use? [Day of VUS/VCUG]
N/A

Cefaclor: which time schedule do you use? [Day(s) after VUS/VCUG, how many?]
N/A

Amoxicillin/clavulanic acid: in what dosage? (Amoxicillin portion) [prophylaktic (10-20 mg/Kg bodyweight/d)]
N/A

Amoxicillin/clavulanic acid: in what dosage? (Amoxicillin portion) [double prophylactic (20-40 mg/Kg bodyweight/d)]
N/A

Amoxicillin/clavulanic acid: in what dosage? (Amoxicillin portion) [therapeutic (80-100 mg/Kg bodyweight/d)]
N/A

Amoxicillin/clavulanic acid: which time schedule do you use? [Day(s) before VUS/VCUG]
N/A

Amoxicillin/clavulanic acid: which time schedule do you use? [Day of VUS/VCUG]
N/A

Amoxicillin/clavulanic acid: which time schedule do you use? [Day(s) after VUS/VCUG]
N/A

Other: which time schedule do you use? [Day(s) before VUS/VCUG, how many?]
N/A

Other: which time schedule do you use? [Day of VUS/VCUG]
N/A

Other: which time schedule do you use? [Day(s) after VUS/VCUG, how many?]
N/A

Indication for prophylaxis

Which patients receive periinterventional antibiotic prophylaxis? (multiple answers possible) [all]
N/A

Which patients receive periinterventional antibiotic prophylaxis? (multiple answers possible) [patients post urinary tract infection/pyelonephritis/urosepsis]
N/A

Which patients receive periinterventional antibiotic prophylaxis? (multiple answers possible) [patients with urinary tract dilation in ultrasound]
N/A

Which patients receive periinterventional antibiotic prophylaxis? (multiple answers possible) [patients with prenatal urinary tract dilation]
N/A

Which patients receive periinterventional antibiotic prophylaxis? (multiple answers possible) [patients post VUR surgery]
N/A

Which patients receive periinterventional antibiotic prophylaxis? (multiple answers possible) [none]
N/A

Are there situations or clinical findings in the patient where you deviate from your prophylaxis standard (medication, dose, duration, age of the patient)? (multiple answers possible, please specify in answer box) [in case of already known vesicoureteral reflux (VUR)]
N/A

Are there situations or clinical findings in the patient where you deviate from your prophylaxis standard (medication, dose, duration, age of the patient)? (multiple answers possible, please specify in answer box) [in patients with recurrent urinary tract infections (UTI)]
N/A

Are there situations or clinical findings in the patient where you deviate from your prophylaxis standard (medication, dose, duration, age of the patient)? (multiple answers possible, please specify in answer box) [if multidrug-resistant organisms (MDRO) are detected in urine cultures]
N/A

Are there situations or clinical findings in the patient where you deviate from your prophylaxis standard (medication, dose, duration, age of the patient)? (multiple answers possible, please specify in answer box) [in newborns]
N/A

Are there situations in which you use intravenous prophylaxis?
N/A

Voiding analysis

Are there differences in antibiotic prophylaxis between VUS and VCUG in your department?
N/A

Is a non-pathological urine strip test required in preparation for VUS/VCUG in your department?
N/A

Is a non-pathological urine culture required in preparation for VUS/VCUG in your department?
N/A

Do patients only receive post-procedure prophylaxis if vesicoureteral reflux (VUR) is detected in VUS/VCUG?
N/A

Do other MUS/MCU findings (e.g. urethral valves) have an influence on post-procedure prophylaxis?
N/A

End of survey

May we contact you personally if we have further questions?
N/A

Survey response 52

Response ID
53

Date submitted
2024-11-24 05:56:45

Last page
5

Start language
en

Seed
193997933

Date started
2024-11-24 05:51:27

Date last action
2024-11-24 05:56:45

Standard Operation Procedure

Who fills out this survey?
Pediatric Radiologist

Who fills out this survey? [Other]

In which type of hospital are you employed?
University Hospital

In which type of hospital are you employed? [Other]

In which country do you work?
Romania

Does your department have a defined protocol for infection prophylaxis in the context of VUS/VCUG?
Yes

Has this protocol been transformed into a written standard operating procedure (SOP)?
Yes

Which department is the author of the SOP for infection prophylaxis in VUS/VCUG?
Pediatric nephrology

Which department is the author of the SOP for infection prophylaxis in VUS/VCUG? [Other]

Antibiotic Prophylaxis

Are there situations/constellations in which you do not use antibiotic prophylaxis for VUS/VCUG?
Yes

Please specify these situations/constellations: [girl with first uncomplicated UTI without urinary tract dilation in ultrasound]
Yes

Please specify these situations/constellations: [boy with first uncomplicated UTI without urinary tract dilation in ultrasound]
Yes

Please specify these situations/constellations: [in patients with low grade urinary tract dilation (up to 2nd grade) and no UTI in medical history]
No

Please specify these situations/constellations: [Other]

If you use antibiotic prophylaxis: which mediaction do you use? (multiple answers and subdivision into age groups possible) [Trimethoprim]
No

If you use antibiotic prophylaxis: which mediaction do you use? (multiple answers and subdivision into age groups possible) [Nitrofurantoin]
Yes

If you use antibiotic prophylaxis: which mediaction do you use? (multiple answers and subdivision into age groups possible) [Amoxicillin]
No

If you use antibiotic prophylaxis: which mediaction do you use? (multiple answers and subdivision into age groups possible) [Cefaclor]
No

If you use antibiotic prophylaxis: which mediaction do you use? (multiple answers and subdivision into age groups possible) [Amoxicillin/clavulanic acid]
No

If you use antibiotic prophylaxis: which mediaction do you use? (multiple answers and subdivision into age groups possible) [Other]
No

Trimethoprim: which time schedule do you use? [Day(s) before VUS/VCUG, how many?]
N/A

Trimethoprim: which time schedule do you use? [Day of VUS/VCUG]
N/A

Trimethoprim: which time schedule do you use? [Day(s) after VUS/VCUG, how many?]
N/A

Nitrofurantoin: in what dosage?
prophylactic (1-2 mg/kg bodyweight/d)

Nitrofurantoin: in what dosage? [Other]

Nitrofurantoin: for which age group do you use nitrofurantoin?
0-5

Nitrofurantoin: which time schedule do you use? [Day(s) before VUS/VCUG, how many?]
No

Nitrofurantoin: which time schedule do you use? [Day(s) before VUS/VCUG, how many?][Comment]

Nitrofurantoin: which time schedule do you use? [Day of VUS/VCUG]
Yes

Nitrofurantoin: which time schedule do you use? [Day of VUS/VCUG][Comment]

Nitrofurantoin: which time schedule do you use? [Day(s) after VUS/VCUG, how many?]
No

Nitrofurantoin: which time schedule do you use? [Day(s) after VUS/VCUG, how many?][Comment]

Amoxicillin: which time schedule do you use? [Day(s) before VUS/VCUG, how many?]
N/A

Amoxicillin: which time schedule do you use? [Day of VUS/VCUG]
N/A

Amoxicillin: which time schedule do you use? [Day(s) after VUS/VCUG, how many?]
N/A

Cefaclor: which time schedule do you use? [Days(s) before VUS/VCUG, how many?]
N/A

Cefaclor: which time schedule do you use? [Day of VUS/VCUG]
N/A

Cefaclor: which time schedule do you use? [Day(s) after VUS/VCUG, how many?]
N/A

Amoxicillin/clavulanic acid: in what dosage? (Amoxicillin portion) [prophylaktic (10-20 mg/Kg bodyweight/d)]
N/A

Amoxicillin/clavulanic acid: in what dosage? (Amoxicillin portion) [double prophylactic (20-40 mg/Kg bodyweight/d)]
N/A

Amoxicillin/clavulanic acid: in what dosage? (Amoxicillin portion) [therapeutic (80-100 mg/Kg bodyweight/d)]
N/A

Amoxicillin/clavulanic acid: which time schedule do you use? [Day(s) before VUS/VCUG]
N/A

Amoxicillin/clavulanic acid: which time schedule do you use? [Day of VUS/VCUG]
N/A

Amoxicillin/clavulanic acid: which time schedule do you use? [Day(s) after VUS/VCUG]
N/A

Other: which time schedule do you use? [Day(s) before VUS/VCUG, how many?]
N/A

Other: which time schedule do you use? [Day of VUS/VCUG]
N/A

Other: which time schedule do you use? [Day(s) after VUS/VCUG, how many?]
N/A

Indication for prophylaxis

Which patients receive periinterventional antibiotic prophylaxis? (multiple answers possible) [all]
No

Which patients receive periinterventional antibiotic prophylaxis? (multiple answers possible) [patients post urinary tract infection/pyelonephritis/urosepsis]
No

Which patients receive periinterventional antibiotic prophylaxis? (multiple answers possible) [patients with urinary tract dilation in ultrasound]
Yes

Which patients receive periinterventional antibiotic prophylaxis? (multiple answers possible) [patients with prenatal urinary tract dilation]
No

Which patients receive periinterventional antibiotic prophylaxis? (multiple answers possible) [patients post VUR surgery]
No

Which patients receive periinterventional antibiotic prophylaxis? (multiple answers possible) [none]
No

Which patients receive periinterventional antibiotic prophylaxis? (multiple answers possible) [Other]

In patients with urinary tract dilation in ultrasound: from what degree on? Image from Beetz R, Bokenkamp A, Brandis M et al (2001) Diagnosis of congenital dilatation of the urinary tract. Consensus Group of the Pediatric Nephrology Working Society in cooperation with the Pediatric Urology Working Group of the German Society of Urology and with the Pediatric Urology Working Society in the Germany Society of Pediatric Surgery. Urologe A 40:495–507 (quiz 8–9)
grade 3

Are there situations or clinical findings in the patient where you deviate from your prophylaxis standard (medication, dose, duration, age of the patient)? (multiple answers possible, please specify in answer box) [in case of already known vesicoureteral reflux (VUR)]
No

Are there situations or clinical findings in the patient where you deviate from your prophylaxis standard (medication, dose, duration, age of the patient)? (multiple answers possible, please specify in answer box) [in case of already known vesicoureteral reflux (VUR)][Comment]

Are there situations or clinical findings in the patient where you deviate from your prophylaxis standard (medication, dose, duration, age of the patient)? (multiple answers possible, please specify in answer box) [in patients with recurrent urinary tract infections (UTI)]
Yes

Are there situations or clinical findings in the patient where you deviate from your prophylaxis standard (medication, dose, duration, age of the patient)? (multiple answers possible, please specify in answer box) [in patients with recurrent urinary tract infections (UTI)][Comment]

Are there situations or clinical findings in the patient where you deviate from your prophylaxis standard (medication, dose, duration, age of the patient)? (multiple answers possible, please specify in answer box) [if multidrug-resistant organisms (MDRO) are detected in urine cultures]
No

Are there situations or clinical findings in the patient where you deviate from your prophylaxis standard (medication, dose, duration, age of the patient)? (multiple answers possible, please specify in answer box) [if multidrug-resistant organisms (MDRO) are detected in urine cultures][Comment]

Are there situations or clinical findings in the patient where you deviate from your prophylaxis standard (medication, dose, duration, age of the patient)? (multiple answers possible, please specify in answer box) [in newborns]
No

Are there situations or clinical findings in the patient where you deviate from your prophylaxis standard (medication, dose, duration, age of the patient)? (multiple answers possible, please specify in answer box) [in newborns][Comment]

Are there situations or clinical findings in the patient where you deviate from your prophylaxis standard (medication, dose, duration, age of the patient)? (multiple answers possible, please specify in answer box) [Other]

Are there situations or clinical findings in the patient where you deviate from your prophylaxis standard (medication, dose, duration, age of the patient)? (multiple answers possible, please specify in answer box) [Other comment]

Are there situations in which you use intravenous prophylaxis?
No

Voiding analysis

Are there differences in antibiotic prophylaxis between VUS and VCUG in your department?
No

Is a non-pathological urine strip test required in preparation for VUS/VCUG in your department?
No

Is a non-pathological urine culture required in preparation for VUS/VCUG in your department?
No

Do patients only receive post-procedure prophylaxis if vesicoureteral reflux (VUR) is detected in VUS/VCUG?
No

Do other MUS/MCU findings (e.g. urethral valves) have an influence on post-procedure prophylaxis?
No

End of survey

Thank you for taking the time to answer this survey!

May we contact you personally if we have further questions?
No

Survey response 53

Response ID
54

Last page
4

Start language
de

Seed
1468053422

Date started
2024-11-25 13:16:53

Date last action
2024-11-25 13:21:13

Standard Operation Procedure

Who fills out this survey?
Other

Who fills out this survey? [Other]
Kindernephrologie

In which type of hospital are you employed?
University Hospital

In which type of hospital are you employed? [Other]

In which country do you work?
Deutschland

Does your department have a defined protocol for infection prophylaxis in the context of VUS/VCUG?
Yes

Has this protocol been transformed into a written standard operating procedure (SOP)?
Yes

Which department is the author of the SOP for infection prophylaxis in VUS/VCUG?
Pediatric nephrology

Which department is the author of the SOP for infection prophylaxis in VUS/VCUG? [Other]

Antibiotic Prophylaxis

Are there situations/constellations in which you do not use antibiotic prophylaxis for VUS/VCUG?
No

Please specify these situations/constellations: [girl with first uncomplicated UTI without urinary tract dilation in ultrasound]
N/A

Please specify these situations/constellations: [boy with first uncomplicated UTI without urinary tract dilation in ultrasound]
N/A

Please specify these situations/constellations: [in patients with low grade urinary tract dilation (up to 2nd grade) and no UTI in medical history]
N/A

If you use antibiotic prophylaxis: which mediaction do you use? (multiple answers and subdivision into age groups possible) [Trimethoprim]
Yes

If you use antibiotic prophylaxis: which mediaction do you use? (multiple answers and subdivision into age groups possible) [Nitrofurantoin]
Yes

If you use antibiotic prophylaxis: which mediaction do you use? (multiple answers and subdivision into age groups possible) [Amoxicillin]
No

If you use antibiotic prophylaxis: which mediaction do you use? (multiple answers and subdivision into age groups possible) [Cefaclor]
Yes

If you use antibiotic prophylaxis: which mediaction do you use? (multiple answers and subdivision into age groups possible) [Amoxicillin/clavulanic acid]
No

If you use antibiotic prophylaxis: which mediaction do you use? (multiple answers and subdivision into age groups possible) [Other]
No

Trimethoprim: in what dosage?
prophylactic (2 mg/kg bodyweight/d)

Trimethoprim: in what dosage? [Other]

Trimethoprim: for which age group do you use trimethoprim?
alle

Trimethoprim: which time schedule do you use? [Day(s) before VUS/VCUG, how many?]
Yes

Trimethoprim: which time schedule do you use? [Day(s) before VUS/VCUG, how many?][Comment]
2

Trimethoprim: which time schedule do you use? [Day of VUS/VCUG]
No

Trimethoprim: which time schedule do you use? [Day of VUS/VCUG][Comment]

Trimethoprim: which time schedule do you use? [Day(s) after VUS/VCUG, how many?]
Yes

Trimethoprim: which time schedule do you use? [Day(s) after VUS/VCUG, how many?][Comment]
1

Nitrofurantoin: in what dosage?
prophylactic (1-2 mg/kg bodyweight/d)

Nitrofurantoin: in what dosage? [Other]

Nitrofurantoin: for which age group do you use nitrofurantoin?
> 1 jahr

Nitrofurantoin: which time schedule do you use? [Day(s) before VUS/VCUG, how many?]
Yes

Nitrofurantoin: which time schedule do you use? [Day(s) before VUS/VCUG, how many?][Comment]
2

Nitrofurantoin: which time schedule do you use? [Day of VUS/VCUG]
No

Nitrofurantoin: which time schedule do you use? [Day of VUS/VCUG][Comment]

Nitrofurantoin: which time schedule do you use? [Day(s) after VUS/VCUG, how many?]
Yes

Nitrofurantoin: which time schedule do you use? [Day(s) after VUS/VCUG, how many?][Comment]
1

Amoxicillin: which time schedule do you use? [Day(s) before VUS/VCUG, how many?]
N/A

Amoxicillin: which time schedule do you use? [Day of VUS/VCUG]
N/A

Amoxicillin: which time schedule do you use? [Day(s) after VUS/VCUG, how many?]
N/A

Cefaclor: in what dosage?
prophylactic (10 mg/kg bodyweight/d)

Cefaclor: in what dosage? [Other]

Cefaclor: for which age group do you use cefaclor?
alle

Cefaclor: which time schedule do you use? [Days(s) before VUS/VCUG, how many?]
Yes

Cefaclor: which time schedule do you use? [Days(s) before VUS/VCUG, how many?][Comment]
2

Cefaclor: which time schedule do you use? [Day of VUS/VCUG]
No

Cefaclor: which time schedule do you use? [Day of VUS/VCUG][Comment]

Cefaclor: which time schedule do you use? [Day(s) after VUS/VCUG, how many?]
Yes

Cefaclor: which time schedule do you use? [Day(s) after VUS/VCUG, how many?][Comment]
1

Amoxicillin/clavulanic acid: in what dosage? (Amoxicillin portion) [prophylaktic (10-20 mg/Kg bodyweight/d)]
N/A

Amoxicillin/clavulanic acid: in what dosage? (Amoxicillin portion) [double prophylactic (20-40 mg/Kg bodyweight/d)]
N/A

Amoxicillin/clavulanic acid: in what dosage? (Amoxicillin portion) [therapeutic (80-100 mg/Kg bodyweight/d)]
N/A

Amoxicillin/clavulanic acid: which time schedule do you use? [Day(s) before VUS/VCUG]
N/A

Amoxicillin/clavulanic acid: which time schedule do you use? [Day of VUS/VCUG]
N/A

Amoxicillin/clavulanic acid: which time schedule do you use? [Day(s) after VUS/VCUG]
N/A

Other: which time schedule do you use? [Day(s) before VUS/VCUG, how many?]
N/A

Other: which time schedule do you use? [Day of VUS/VCUG]
N/A

Other: which time schedule do you use? [Day(s) after VUS/VCUG, how many?]
N/A

Indication for prophylaxis

Which patients receive periinterventional antibiotic prophylaxis? (multiple answers possible) [all]
Yes

Which patients receive periinterventional antibiotic prophylaxis? (multiple answers possible) [patients post urinary tract infection/pyelonephritis/urosepsis]
N/A

Which patients receive periinterventional antibiotic prophylaxis? (multiple answers possible) [patients with urinary tract dilation in ultrasound]
N/A

Which patients receive periinterventional antibiotic prophylaxis? (multiple answers possible) [patients with prenatal urinary tract dilation]
N/A

Which patients receive periinterventional antibiotic prophylaxis? (multiple answers possible) [patients post VUR surgery]
N/A

Which patients receive periinterventional antibiotic prophylaxis? (multiple answers possible) [none]
N/A

Are there situations or clinical findings in the patient where you deviate from your prophylaxis standard (medication, dose, duration, age of the patient)? (multiple answers possible, please specify in answer box) [in case of already known vesicoureteral reflux (VUR)]
No

Are there situations or clinical findings in the patient where you deviate from your prophylaxis standard (medication, dose, duration, age of the patient)? (multiple answers possible, please specify in answer box) [in case of already known vesicoureteral reflux (VUR)][Comment]

Are there situations or clinical findings in the patient where you deviate from your prophylaxis standard (medication, dose, duration, age of the patient)? (multiple answers possible, please specify in answer box) [in patients with recurrent urinary tract infections (UTI)]
No

Are there situations or clinical findings in the patient where you deviate from your prophylaxis standard (medication, dose, duration, age of the patient)? (multiple answers possible, please specify in answer box) [in patients with recurrent urinary tract infections (UTI)][Comment]

Are there situations or clinical findings in the patient where you deviate from your prophylaxis standard (medication, dose, duration, age of the patient)? (multiple answers possible, please specify in answer box) [if multidrug-resistant organisms (MDRO) are detected in urine cultures]
Yes

Are there situations or clinical findings in the patient where you deviate from your prophylaxis standard (medication, dose, duration, age of the patient)? (multiple answers possible, please specify in answer box) [if multidrug-resistant organisms (MDRO) are detected in urine cultures][Comment]

Are there situations or clinical findings in the patient where you deviate from your prophylaxis standard (medication, dose, duration, age of the patient)? (multiple answers possible, please specify in answer box) [in newborns]
No

Are there situations or clinical findings in the patient where you deviate from your prophylaxis standard (medication, dose, duration, age of the patient)? (multiple answers possible, please specify in answer box) [in newborns][Comment]

Are there situations or clinical findings in the patient where you deviate from your prophylaxis standard (medication, dose, duration, age of the patient)? (multiple answers possible, please specify in answer box) [Other]

Are there situations or clinical findings in the patient where you deviate from your prophylaxis standard (medication, dose, duration, age of the patient)? (multiple answers possible, please specify in answer box) [Other comment]

Are there situations in which you use intravenous prophylaxis?
No

Voiding analysis

Are there differences in antibiotic prophylaxis between VUS and VCUG in your department?
No

Is a non-pathological urine strip test required in preparation for VUS/VCUG in your department?
Yes

What is the maximum amount of days accepted for urine strip test to be performed prior to the exam?
1

Is a non-pathological urine culture required in preparation for VUS/VCUG in your department?
No

Do patients only receive post-procedure prophylaxis if vesicoureteral reflux (VUR) is detected in VUS/VCUG?
No

Do other MUS/MCU findings (e.g. urethral valves) have an influence on post-procedure prophylaxis?
No

End of survey

May we contact you personally if we have further questions?
N/A

Survey response 54

Response ID
56

Last page
2

Start language
de

Seed
648830613

Date started
2024-11-25 17:08:56

Date last action
2024-11-25 17:09:17

Standard Operation Procedure

Who fills out this survey?
Other

Who fills out this survey? [Other]

In which type of hospital are you employed?
Other

In which type of hospital are you employed? [Other]

In which country do you work?
adasd

Does your department have a defined protocol for infection prophylaxis in the context of VUS/VCUG?
No

Has this protocol been transformed into a written standard operating procedure (SOP)?
N/A

Antibiotic Prophylaxis

Are there situations/constellations in which you do not use antibiotic prophylaxis for VUS/VCUG?
No

Please specify these situations/constellations: [girl with first uncomplicated UTI without urinary tract dilation in ultrasound]
N/A

Please specify these situations/constellations: [boy with first uncomplicated UTI without urinary tract dilation in ultrasound]
N/A

Please specify these situations/constellations: [in patients with low grade urinary tract dilation (up to 2nd grade) and no UTI in medical history]
N/A

If you use antibiotic prophylaxis: which mediaction do you use? (multiple answers and subdivision into age groups possible) [Trimethoprim]
No

If you use antibiotic prophylaxis: which mediaction do you use? (multiple answers and subdivision into age groups possible) [Nitrofurantoin]
No

If you use antibiotic prophylaxis: which mediaction do you use? (multiple answers and subdivision into age groups possible) [Amoxicillin]
Yes

If you use antibiotic prophylaxis: which mediaction do you use? (multiple answers and subdivision into age groups possible) [Cefaclor]
No

If you use antibiotic prophylaxis: which mediaction do you use? (multiple answers and subdivision into age groups possible) [Amoxicillin/clavulanic acid]
No

If you use antibiotic prophylaxis: which mediaction do you use? (multiple answers and subdivision into age groups possible) [Other]
No

Trimethoprim: which time schedule do you use? [Day(s) before VUS/VCUG, how many?]
N/A

Trimethoprim: which time schedule do you use? [Day of VUS/VCUG]
N/A

Trimethoprim: which time schedule do you use? [Day(s) after VUS/VCUG, how many?]
N/A

Nitrofurantoin: which time schedule do you use? [Day(s) before VUS/VCUG, how many?]
N/A

Nitrofurantoin: which time schedule do you use? [Day of VUS/VCUG]
N/A

Nitrofurantoin: which time schedule do you use? [Day(s) after VUS/VCUG, how many?]
N/A

Amoxicillin: in what dosage?
prophylactic (10-20 mg/kg bodyweight/d)

Amoxicillin: in what dosage? [Other]

Amoxicillin: for which age group do you use amoxicillin?
adad

Amoxicillin: which time schedule do you use? [Day(s) before VUS/VCUG, how many?]
Yes

Amoxicillin: which time schedule do you use? [Day(s) before VUS/VCUG, how many?][Comment]

Amoxicillin: which time schedule do you use? [Day of VUS/VCUG]
Yes

Amoxicillin: which time schedule do you use? [Day of VUS/VCUG][Comment]

Amoxicillin: which time schedule do you use? [Day(s) after VUS/VCUG, how many?]
No

Amoxicillin: which time schedule do you use? [Day(s) after VUS/VCUG, how many?][Comment]

Cefaclor: which time schedule do you use? [Days(s) before VUS/VCUG, how many?]
N/A

Cefaclor: which time schedule do you use? [Day of VUS/VCUG]
N/A

Cefaclor: which time schedule do you use? [Day(s) after VUS/VCUG, how many?]
N/A

Amoxicillin/clavulanic acid: in what dosage? (Amoxicillin portion) [prophylaktic (10-20 mg/Kg bodyweight/d)]
N/A

Amoxicillin/clavulanic acid: in what dosage? (Amoxicillin portion) [double prophylactic (20-40 mg/Kg bodyweight/d)]
N/A

Amoxicillin/clavulanic acid: in what dosage? (Amoxicillin portion) [therapeutic (80-100 mg/Kg bodyweight/d)]
N/A

Amoxicillin/clavulanic acid: which time schedule do you use? [Day(s) before VUS/VCUG]
N/A

Amoxicillin/clavulanic acid: which time schedule do you use? [Day of VUS/VCUG]
N/A

Amoxicillin/clavulanic acid: which time schedule do you use? [Day(s) after VUS/VCUG]
N/A

Other: which time schedule do you use? [Day(s) before VUS/VCUG, how many?]
N/A

Other: which time schedule do you use? [Day of VUS/VCUG]
N/A

Other: which time schedule do you use? [Day(s) after VUS/VCUG, how many?]
N/A

Indication for prophylaxis

Which patients receive periinterventional antibiotic prophylaxis? (multiple answers possible) [all]
N/A

Which patients receive periinterventional antibiotic prophylaxis? (multiple answers possible) [patients post urinary tract infection/pyelonephritis/urosepsis]
N/A

Which patients receive periinterventional antibiotic prophylaxis? (multiple answers possible) [patients with urinary tract dilation in ultrasound]
N/A

Which patients receive periinterventional antibiotic prophylaxis? (multiple answers possible) [patients with prenatal urinary tract dilation]
N/A

Which patients receive periinterventional antibiotic prophylaxis? (multiple answers possible) [patients post VUR surgery]
N/A

Which patients receive periinterventional antibiotic prophylaxis? (multiple answers possible) [none]
N/A

Are there situations or clinical findings in the patient where you deviate from your prophylaxis standard (medication, dose, duration, age of the patient)? (multiple answers possible, please specify in answer box) [in case of already known vesicoureteral reflux (VUR)]
N/A

Are there situations or clinical findings in the patient where you deviate from your prophylaxis standard (medication, dose, duration, age of the patient)? (multiple answers possible, please specify in answer box) [in patients with recurrent urinary tract infections (UTI)]
N/A

Are there situations or clinical findings in the patient where you deviate from your prophylaxis standard (medication, dose, duration, age of the patient)? (multiple answers possible, please specify in answer box) [if multidrug-resistant organisms (MDRO) are detected in urine cultures]
N/A

Are there situations or clinical findings in the patient where you deviate from your prophylaxis standard (medication, dose, duration, age of the patient)? (multiple answers possible, please specify in answer box) [in newborns]
N/A

Are there situations in which you use intravenous prophylaxis?
N/A

Voiding analysis

Are there differences in antibiotic prophylaxis between VUS and VCUG in your department?
N/A

Is a non-pathological urine strip test required in preparation for VUS/VCUG in your department?
N/A

Is a non-pathological urine culture required in preparation for VUS/VCUG in your department?
N/A

Do patients only receive post-procedure prophylaxis if vesicoureteral reflux (VUR) is detected in VUS/VCUG?
N/A

Do other MUS/MCU findings (e.g. urethral valves) have an influence on post-procedure prophylaxis?
N/A

End of survey

May we contact you personally if we have further questions?
N/A

Survey response 55

Response ID
57

Last page
0

Start language
de

Seed
1083228478

Date started
2024-11-30 10:18:29

Date last action
2024-11-30 10:18:29

Standard Operation Procedure

Does your department have a defined protocol for infection prophylaxis in the context of VUS/VCUG?
N/A

Has this protocol been transformed into a written standard operating procedure (SOP)?
N/A

Antibiotic Prophylaxis

Are there situations/constellations in which you do not use antibiotic prophylaxis for VUS/VCUG?
N/A

Please specify these situations/constellations: [girl with first uncomplicated UTI without urinary tract dilation in ultrasound]
N/A

Please specify these situations/constellations: [boy with first uncomplicated UTI without urinary tract dilation in ultrasound]
N/A

Please specify these situations/constellations: [in patients with low grade urinary tract dilation (up to 2nd grade) and no UTI in medical history]
N/A

If you use antibiotic prophylaxis: which mediaction do you use? (multiple answers and subdivision into age groups possible) [Trimethoprim]
N/A

If you use antibiotic prophylaxis: which mediaction do you use? (multiple answers and subdivision into age groups possible) [Nitrofurantoin]
N/A

If you use antibiotic prophylaxis: which mediaction do you use? (multiple answers and subdivision into age groups possible) [Amoxicillin]
N/A

If you use antibiotic prophylaxis: which mediaction do you use? (multiple answers and subdivision into age groups possible) [Cefaclor]
N/A

If you use antibiotic prophylaxis: which mediaction do you use? (multiple answers and subdivision into age groups possible) [Amoxicillin/clavulanic acid]
N/A

If you use antibiotic prophylaxis: which mediaction do you use? (multiple answers and subdivision into age groups possible) [Other]
N/A

Trimethoprim: which time schedule do you use? [Day(s) before VUS/VCUG, how many?]
N/A

Trimethoprim: which time schedule do you use? [Day of VUS/VCUG]
N/A

Trimethoprim: which time schedule do you use? [Day(s) after VUS/VCUG, how many?]
N/A

Nitrofurantoin: which time schedule do you use? [Day(s) before VUS/VCUG, how many?]
N/A

Nitrofurantoin: which time schedule do you use? [Day of VUS/VCUG]
N/A

Nitrofurantoin: which time schedule do you use? [Day(s) after VUS/VCUG, how many?]
N/A

Amoxicillin: which time schedule do you use? [Day(s) before VUS/VCUG, how many?]
N/A

Amoxicillin: which time schedule do you use? [Day of VUS/VCUG]
N/A

Amoxicillin: which time schedule do you use? [Day(s) after VUS/VCUG, how many?]
N/A

Cefaclor: which time schedule do you use? [Days(s) before VUS/VCUG, how many?]
N/A

Cefaclor: which time schedule do you use? [Day of VUS/VCUG]
N/A

Cefaclor: which time schedule do you use? [Day(s) after VUS/VCUG, how many?]
N/A

Amoxicillin/clavulanic acid: in what dosage? (Amoxicillin portion) [prophylaktic (10-20 mg/Kg bodyweight/d)]
N/A

Amoxicillin/clavulanic acid: in what dosage? (Amoxicillin portion) [double prophylactic (20-40 mg/Kg bodyweight/d)]
N/A

Amoxicillin/clavulanic acid: in what dosage? (Amoxicillin portion) [therapeutic (80-100 mg/Kg bodyweight/d)]
N/A

Amoxicillin/clavulanic acid: which time schedule do you use? [Day(s) before VUS/VCUG]
N/A

Amoxicillin/clavulanic acid: which time schedule do you use? [Day of VUS/VCUG]
N/A

Amoxicillin/clavulanic acid: which time schedule do you use? [Day(s) after VUS/VCUG]
N/A

Other: which time schedule do you use? [Day(s) before VUS/VCUG, how many?]
N/A

Other: which time schedule do you use? [Day of VUS/VCUG]
N/A

Other: which time schedule do you use? [Day(s) after VUS/VCUG, how many?]
N/A

Indication for prophylaxis

Which patients receive periinterventional antibiotic prophylaxis? (multiple answers possible) [all]
N/A

Which patients receive periinterventional antibiotic prophylaxis? (multiple answers possible) [patients post urinary tract infection/pyelonephritis/urosepsis]
N/A

Which patients receive periinterventional antibiotic prophylaxis? (multiple answers possible) [patients with urinary tract dilation in ultrasound]
N/A

Which patients receive periinterventional antibiotic prophylaxis? (multiple answers possible) [patients with prenatal urinary tract dilation]
N/A

Which patients receive periinterventional antibiotic prophylaxis? (multiple answers possible) [patients post VUR surgery]
N/A

Which patients receive periinterventional antibiotic prophylaxis? (multiple answers possible) [none]
N/A

Are there situations or clinical findings in the patient where you deviate from your prophylaxis standard (medication, dose, duration, age of the patient)? (multiple answers possible, please specify in answer box) [in case of already known vesicoureteral reflux (VUR)]
N/A

Are there situations or clinical findings in the patient where you deviate from your prophylaxis standard (medication, dose, duration, age of the patient)? (multiple answers possible, please specify in answer box) [in patients with recurrent urinary tract infections (UTI)]
N/A

Are there situations or clinical findings in the patient where you deviate from your prophylaxis standard (medication, dose, duration, age of the patient)? (multiple answers possible, please specify in answer box) [if multidrug-resistant organisms (MDRO) are detected in urine cultures]
N/A

Are there situations or clinical findings in the patient where you deviate from your prophylaxis standard (medication, dose, duration, age of the patient)? (multiple answers possible, please specify in answer box) [in newborns]
N/A

Are there situations in which you use intravenous prophylaxis?
N/A

Voiding analysis

Are there differences in antibiotic prophylaxis between VUS and VCUG in your department?
N/A

Is a non-pathological urine strip test required in preparation for VUS/VCUG in your department?
N/A

Is a non-pathological urine culture required in preparation for VUS/VCUG in your department?
N/A

Do patients only receive post-procedure prophylaxis if vesicoureteral reflux (VUR) is detected in VUS/VCUG?
N/A

Do other MUS/MCU findings (e.g. urethral valves) have an influence on post-procedure prophylaxis?
N/A

End of survey

May we contact you personally if we have further questions?
N/A

Survey response 56

Response ID
58

Last page
0

Start language
de

Seed
1078420584

Date started
2024-12-01 21:54:56

Date last action
2024-12-01 21:55:16

Standard Operation Procedure

Who fills out this survey?
Pediatric Radiologist

Who fills out this survey? [Other]

In which type of hospital are you employed?

In which type of hospital are you employed? [Other]

In which country do you work?

Does your department have a defined protocol for infection prophylaxis in the context of VUS/VCUG?
N/A

Has this protocol been transformed into a written standard operating procedure (SOP)?
N/A

Antibiotic Prophylaxis

Are there situations/constellations in which you do not use antibiotic prophylaxis for VUS/VCUG?
N/A

Please specify these situations/constellations: [girl with first uncomplicated UTI without urinary tract dilation in ultrasound]
N/A

Please specify these situations/constellations: [boy with first uncomplicated UTI without urinary tract dilation in ultrasound]
N/A

Please specify these situations/constellations: [in patients with low grade urinary tract dilation (up to 2nd grade) and no UTI in medical history]
N/A

If you use antibiotic prophylaxis: which mediaction do you use? (multiple answers and subdivision into age groups possible) [Trimethoprim]
N/A

If you use antibiotic prophylaxis: which mediaction do you use? (multiple answers and subdivision into age groups possible) [Nitrofurantoin]
N/A

If you use antibiotic prophylaxis: which mediaction do you use? (multiple answers and subdivision into age groups possible) [Amoxicillin]
N/A

If you use antibiotic prophylaxis: which mediaction do you use? (multiple answers and subdivision into age groups possible) [Cefaclor]
N/A

If you use antibiotic prophylaxis: which mediaction do you use? (multiple answers and subdivision into age groups possible) [Amoxicillin/clavulanic acid]
N/A

If you use antibiotic prophylaxis: which mediaction do you use? (multiple answers and subdivision into age groups possible) [Other]
N/A

Trimethoprim: which time schedule do you use? [Day(s) before VUS/VCUG, how many?]
N/A

Trimethoprim: which time schedule do you use? [Day of VUS/VCUG]
N/A

Trimethoprim: which time schedule do you use? [Day(s) after VUS/VCUG, how many?]
N/A

Nitrofurantoin: which time schedule do you use? [Day(s) before VUS/VCUG, how many?]
N/A

Nitrofurantoin: which time schedule do you use? [Day of VUS/VCUG]
N/A

Nitrofurantoin: which time schedule do you use? [Day(s) after VUS/VCUG, how many?]
N/A

Amoxicillin: which time schedule do you use? [Day(s) before VUS/VCUG, how many?]
N/A

Amoxicillin: which time schedule do you use? [Day of VUS/VCUG]
N/A

Amoxicillin: which time schedule do you use? [Day(s) after VUS/VCUG, how many?]
N/A

Cefaclor: which time schedule do you use? [Days(s) before VUS/VCUG, how many?]
N/A

Cefaclor: which time schedule do you use? [Day of VUS/VCUG]
N/A

Cefaclor: which time schedule do you use? [Day(s) after VUS/VCUG, how many?]
N/A

Amoxicillin/clavulanic acid: in what dosage? (Amoxicillin portion) [prophylaktic (10-20 mg/Kg bodyweight/d)]
N/A

Amoxicillin/clavulanic acid: in what dosage? (Amoxicillin portion) [double prophylactic (20-40 mg/Kg bodyweight/d)]
N/A

Amoxicillin/clavulanic acid: in what dosage? (Amoxicillin portion) [therapeutic (80-100 mg/Kg bodyweight/d)]
N/A

Amoxicillin/clavulanic acid: which time schedule do you use? [Day(s) before VUS/VCUG]
N/A

Amoxicillin/clavulanic acid: which time schedule do you use? [Day of VUS/VCUG]
N/A

Amoxicillin/clavulanic acid: which time schedule do you use? [Day(s) after VUS/VCUG]
N/A

Other: which time schedule do you use? [Day(s) before VUS/VCUG, how many?]
N/A

Other: which time schedule do you use? [Day of VUS/VCUG]
N/A

Other: which time schedule do you use? [Day(s) after VUS/VCUG, how many?]
N/A

Indication for prophylaxis

Which patients receive periinterventional antibiotic prophylaxis? (multiple answers possible) [all]
N/A

Which patients receive periinterventional antibiotic prophylaxis? (multiple answers possible) [patients post urinary tract infection/pyelonephritis/urosepsis]
N/A

Which patients receive periinterventional antibiotic prophylaxis? (multiple answers possible) [patients with urinary tract dilation in ultrasound]
N/A

Which patients receive periinterventional antibiotic prophylaxis? (multiple answers possible) [patients with prenatal urinary tract dilation]
N/A

Which patients receive periinterventional antibiotic prophylaxis? (multiple answers possible) [patients post VUR surgery]
N/A

Which patients receive periinterventional antibiotic prophylaxis? (multiple answers possible) [none]
N/A

Are there situations or clinical findings in the patient where you deviate from your prophylaxis standard (medication, dose, duration, age of the patient)? (multiple answers possible, please specify in answer box) [in case of already known vesicoureteral reflux (VUR)]
N/A

Are there situations or clinical findings in the patient where you deviate from your prophylaxis standard (medication, dose, duration, age of the patient)? (multiple answers possible, please specify in answer box) [in patients with recurrent urinary tract infections (UTI)]
N/A

Are there situations or clinical findings in the patient where you deviate from your prophylaxis standard (medication, dose, duration, age of the patient)? (multiple answers possible, please specify in answer box) [if multidrug-resistant organisms (MDRO) are detected in urine cultures]
N/A

Are there situations or clinical findings in the patient where you deviate from your prophylaxis standard (medication, dose, duration, age of the patient)? (multiple answers possible, please specify in answer box) [in newborns]
N/A

Are there situations in which you use intravenous prophylaxis?
N/A

Voiding analysis

Are there differences in antibiotic prophylaxis between VUS and VCUG in your department?
N/A

Is a non-pathological urine strip test required in preparation for VUS/VCUG in your department?
N/A

Is a non-pathological urine culture required in preparation for VUS/VCUG in your department?
N/A

Do patients only receive post-procedure prophylaxis if vesicoureteral reflux (VUR) is detected in VUS/VCUG?
N/A

Do other MUS/MCU findings (e.g. urethral valves) have an influence on post-procedure prophylaxis?
N/A

End of survey

May we contact you personally if we have further questions?
N/A

Survey response 57

Response ID
59

Last page
0

Start language
de

Seed
1558754523

Date started
2024-12-02 18:59:30

Date last action
2024-12-02 18:59:30

Standard Operation Procedure

Does your department have a defined protocol for infection prophylaxis in the context of VUS/VCUG?
N/A

Has this protocol been transformed into a written standard operating procedure (SOP)?
N/A

Antibiotic Prophylaxis

Are there situations/constellations in which you do not use antibiotic prophylaxis for VUS/VCUG?
N/A

Please specify these situations/constellations: [girl with first uncomplicated UTI without urinary tract dilation in ultrasound]
N/A

Please specify these situations/constellations: [boy with first uncomplicated UTI without urinary tract dilation in ultrasound]
N/A

Please specify these situations/constellations: [in patients with low grade urinary tract dilation (up to 2nd grade) and no UTI in medical history]
N/A

If you use antibiotic prophylaxis: which mediaction do you use? (multiple answers and subdivision into age groups possible) [Trimethoprim]
N/A

If you use antibiotic prophylaxis: which mediaction do you use? (multiple answers and subdivision into age groups possible) [Nitrofurantoin]
N/A

If you use antibiotic prophylaxis: which mediaction do you use? (multiple answers and subdivision into age groups possible) [Amoxicillin]
N/A

If you use antibiotic prophylaxis: which mediaction do you use? (multiple answers and subdivision into age groups possible) [Cefaclor]
N/A

If you use antibiotic prophylaxis: which mediaction do you use? (multiple answers and subdivision into age groups possible) [Amoxicillin/clavulanic acid]
N/A

If you use antibiotic prophylaxis: which mediaction do you use? (multiple answers and subdivision into age groups possible) [Other]
N/A

Trimethoprim: which time schedule do you use? [Day(s) before VUS/VCUG, how many?]
N/A

Trimethoprim: which time schedule do you use? [Day of VUS/VCUG]
N/A

Trimethoprim: which time schedule do you use? [Day(s) after VUS/VCUG, how many?]
N/A

Nitrofurantoin: which time schedule do you use? [Day(s) before VUS/VCUG, how many?]
N/A

Nitrofurantoin: which time schedule do you use? [Day of VUS/VCUG]
N/A

Nitrofurantoin: which time schedule do you use? [Day(s) after VUS/VCUG, how many?]
N/A

Amoxicillin: which time schedule do you use? [Day(s) before VUS/VCUG, how many?]
N/A

Amoxicillin: which time schedule do you use? [Day of VUS/VCUG]
N/A

Amoxicillin: which time schedule do you use? [Day(s) after VUS/VCUG, how many?]
N/A

Cefaclor: which time schedule do you use? [Days(s) before VUS/VCUG, how many?]
N/A

Cefaclor: which time schedule do you use? [Day of VUS/VCUG]
N/A

Cefaclor: which time schedule do you use? [Day(s) after VUS/VCUG, how many?]
N/A

Amoxicillin/clavulanic acid: in what dosage? (Amoxicillin portion) [prophylaktic (10-20 mg/Kg bodyweight/d)]
N/A

Amoxicillin/clavulanic acid: in what dosage? (Amoxicillin portion) [double prophylactic (20-40 mg/Kg bodyweight/d)]
N/A

Amoxicillin/clavulanic acid: in what dosage? (Amoxicillin portion) [therapeutic (80-100 mg/Kg bodyweight/d)]
N/A

Amoxicillin/clavulanic acid: which time schedule do you use? [Day(s) before VUS/VCUG]
N/A

Amoxicillin/clavulanic acid: which time schedule do you use? [Day of VUS/VCUG]
N/A

Amoxicillin/clavulanic acid: which time schedule do you use? [Day(s) after VUS/VCUG]
N/A

Other: which time schedule do you use? [Day(s) before VUS/VCUG, how many?]
N/A

Other: which time schedule do you use? [Day of VUS/VCUG]
N/A

Other: which time schedule do you use? [Day(s) after VUS/VCUG, how many?]
N/A

Indication for prophylaxis

Which patients receive periinterventional antibiotic prophylaxis? (multiple answers possible) [all]
N/A

Which patients receive periinterventional antibiotic prophylaxis? (multiple answers possible) [patients post urinary tract infection/pyelonephritis/urosepsis]
N/A

Which patients receive periinterventional antibiotic prophylaxis? (multiple answers possible) [patients with urinary tract dilation in ultrasound]
N/A

Which patients receive periinterventional antibiotic prophylaxis? (multiple answers possible) [patients with prenatal urinary tract dilation]
N/A

Which patients receive periinterventional antibiotic prophylaxis? (multiple answers possible) [patients post VUR surgery]
N/A

Which patients receive periinterventional antibiotic prophylaxis? (multiple answers possible) [none]
N/A

Are there situations or clinical findings in the patient where you deviate from your prophylaxis standard (medication, dose, duration, age of the patient)? (multiple answers possible, please specify in answer box) [in case of already known vesicoureteral reflux (VUR)]
N/A

Are there situations or clinical findings in the patient where you deviate from your prophylaxis standard (medication, dose, duration, age of the patient)? (multiple answers possible, please specify in answer box) [in patients with recurrent urinary tract infections (UTI)]
N/A

Are there situations or clinical findings in the patient where you deviate from your prophylaxis standard (medication, dose, duration, age of the patient)? (multiple answers possible, please specify in answer box) [if multidrug-resistant organisms (MDRO) are detected in urine cultures]
N/A

Are there situations or clinical findings in the patient where you deviate from your prophylaxis standard (medication, dose, duration, age of the patient)? (multiple answers possible, please specify in answer box) [in newborns]
N/A

Are there situations in which you use intravenous prophylaxis?
N/A

Voiding analysis

Are there differences in antibiotic prophylaxis between VUS and VCUG in your department?
N/A

Is a non-pathological urine strip test required in preparation for VUS/VCUG in your department?
N/A

Is a non-pathological urine culture required in preparation for VUS/VCUG in your department?
N/A

Do patients only receive post-procedure prophylaxis if vesicoureteral reflux (VUR) is detected in VUS/VCUG?
N/A

Do other MUS/MCU findings (e.g. urethral valves) have an influence on post-procedure prophylaxis?
N/A

End of survey

May we contact you personally if we have further questions?
N/A

Survey response 58

Response ID
60

Last page
1

Start language
de

Seed
146429098

Date started
2024-12-03 10:37:59

Date last action
2024-12-03 10:38:25

Standard Operation Procedure

Who fills out this survey?
Pediatric Radiologist

Who fills out this survey? [Other]

In which type of hospital are you employed?
University Hospital

In which type of hospital are you employed? [Other]

In which country do you work?
Österreich

Does your department have a defined protocol for infection prophylaxis in the context of VUS/VCUG?
No

Has this protocol been transformed into a written standard operating procedure (SOP)?
N/A

Antibiotic Prophylaxis

Are there situations/constellations in which you do not use antibiotic prophylaxis for VUS/VCUG?
N/A

Please specify these situations/constellations: [girl with first uncomplicated UTI without urinary tract dilation in ultrasound]
N/A

Please specify these situations/constellations: [boy with first uncomplicated UTI without urinary tract dilation in ultrasound]
N/A

Please specify these situations/constellations: [in patients with low grade urinary tract dilation (up to 2nd grade) and no UTI in medical history]
N/A

If you use antibiotic prophylaxis: which mediaction do you use? (multiple answers and subdivision into age groups possible) [Trimethoprim]
N/A

If you use antibiotic prophylaxis: which mediaction do you use? (multiple answers and subdivision into age groups possible) [Nitrofurantoin]
N/A

If you use antibiotic prophylaxis: which mediaction do you use? (multiple answers and subdivision into age groups possible) [Amoxicillin]
N/A

If you use antibiotic prophylaxis: which mediaction do you use? (multiple answers and subdivision into age groups possible) [Cefaclor]
N/A

If you use antibiotic prophylaxis: which mediaction do you use? (multiple answers and subdivision into age groups possible) [Amoxicillin/clavulanic acid]
N/A

If you use antibiotic prophylaxis: which mediaction do you use? (multiple answers and subdivision into age groups possible) [Other]
N/A

Trimethoprim: which time schedule do you use? [Day(s) before VUS/VCUG, how many?]
N/A

Trimethoprim: which time schedule do you use? [Day of VUS/VCUG]
N/A

Trimethoprim: which time schedule do you use? [Day(s) after VUS/VCUG, how many?]
N/A

Nitrofurantoin: which time schedule do you use? [Day(s) before VUS/VCUG, how many?]
N/A

Nitrofurantoin: which time schedule do you use? [Day of VUS/VCUG]
N/A

Nitrofurantoin: which time schedule do you use? [Day(s) after VUS/VCUG, how many?]
N/A

Amoxicillin: which time schedule do you use? [Day(s) before VUS/VCUG, how many?]
N/A

Amoxicillin: which time schedule do you use? [Day of VUS/VCUG]
N/A

Amoxicillin: which time schedule do you use? [Day(s) after VUS/VCUG, how many?]
N/A

Cefaclor: which time schedule do you use? [Days(s) before VUS/VCUG, how many?]
N/A

Cefaclor: which time schedule do you use? [Day of VUS/VCUG]
N/A

Cefaclor: which time schedule do you use? [Day(s) after VUS/VCUG, how many?]
N/A

Amoxicillin/clavulanic acid: in what dosage? (Amoxicillin portion) [prophylaktic (10-20 mg/Kg bodyweight/d)]
N/A

Amoxicillin/clavulanic acid: in what dosage? (Amoxicillin portion) [double prophylactic (20-40 mg/Kg bodyweight/d)]
N/A

Amoxicillin/clavulanic acid: in what dosage? (Amoxicillin portion) [therapeutic (80-100 mg/Kg bodyweight/d)]
N/A

Amoxicillin/clavulanic acid: which time schedule do you use? [Day(s) before VUS/VCUG]
N/A

Amoxicillin/clavulanic acid: which time schedule do you use? [Day of VUS/VCUG]
N/A

Amoxicillin/clavulanic acid: which time schedule do you use? [Day(s) after VUS/VCUG]
N/A

Other: which time schedule do you use? [Day(s) before VUS/VCUG, how many?]
N/A

Other: which time schedule do you use? [Day of VUS/VCUG]
N/A

Other: which time schedule do you use? [Day(s) after VUS/VCUG, how many?]
N/A

Indication for prophylaxis

Which patients receive periinterventional antibiotic prophylaxis? (multiple answers possible) [all]
N/A

Which patients receive periinterventional antibiotic prophylaxis? (multiple answers possible) [patients post urinary tract infection/pyelonephritis/urosepsis]
N/A

Which patients receive periinterventional antibiotic prophylaxis? (multiple answers possible) [patients with urinary tract dilation in ultrasound]
N/A

Which patients receive periinterventional antibiotic prophylaxis? (multiple answers possible) [patients with prenatal urinary tract dilation]
N/A

Which patients receive periinterventional antibiotic prophylaxis? (multiple answers possible) [patients post VUR surgery]
N/A

Which patients receive periinterventional antibiotic prophylaxis? (multiple answers possible) [none]
N/A

Are there situations or clinical findings in the patient where you deviate from your prophylaxis standard (medication, dose, duration, age of the patient)? (multiple answers possible, please specify in answer box) [in case of already known vesicoureteral reflux (VUR)]
N/A

Are there situations or clinical findings in the patient where you deviate from your prophylaxis standard (medication, dose, duration, age of the patient)? (multiple answers possible, please specify in answer box) [in patients with recurrent urinary tract infections (UTI)]
N/A

Are there situations or clinical findings in the patient where you deviate from your prophylaxis standard (medication, dose, duration, age of the patient)? (multiple answers possible, please specify in answer box) [if multidrug-resistant organisms (MDRO) are detected in urine cultures]
N/A

Are there situations or clinical findings in the patient where you deviate from your prophylaxis standard (medication, dose, duration, age of the patient)? (multiple answers possible, please specify in answer box) [in newborns]
N/A

Are there situations in which you use intravenous prophylaxis?
N/A

Voiding analysis

Are there differences in antibiotic prophylaxis between VUS and VCUG in your department?
N/A

Is a non-pathological urine strip test required in preparation for VUS/VCUG in your department?
N/A

Is a non-pathological urine culture required in preparation for VUS/VCUG in your department?
N/A

Do patients only receive post-procedure prophylaxis if vesicoureteral reflux (VUR) is detected in VUS/VCUG?
N/A

Do other MUS/MCU findings (e.g. urethral valves) have an influence on post-procedure prophylaxis?
N/A

End of survey

May we contact you personally if we have further questions?
N/A

Survey response 59

Response ID
61

Last page
0

Start language
de

Seed
1963357581

Date started
2024-12-03 16:17:08

Date last action
2024-12-03 16:17:08

Standard Operation Procedure

Does your department have a defined protocol for infection prophylaxis in the context of VUS/VCUG?
N/A

Has this protocol been transformed into a written standard operating procedure (SOP)?
N/A

Antibiotic Prophylaxis

Are there situations/constellations in which you do not use antibiotic prophylaxis for VUS/VCUG?
N/A

Please specify these situations/constellations: [girl with first uncomplicated UTI without urinary tract dilation in ultrasound]
N/A

Please specify these situations/constellations: [boy with first uncomplicated UTI without urinary tract dilation in ultrasound]
N/A

Please specify these situations/constellations: [in patients with low grade urinary tract dilation (up to 2nd grade) and no UTI in medical history]
N/A

If you use antibiotic prophylaxis: which mediaction do you use? (multiple answers and subdivision into age groups possible) [Trimethoprim]
N/A

If you use antibiotic prophylaxis: which mediaction do you use? (multiple answers and subdivision into age groups possible) [Nitrofurantoin]
N/A

If you use antibiotic prophylaxis: which mediaction do you use? (multiple answers and subdivision into age groups possible) [Amoxicillin]
N/A

If you use antibiotic prophylaxis: which mediaction do you use? (multiple answers and subdivision into age groups possible) [Cefaclor]
N/A

If you use antibiotic prophylaxis: which mediaction do you use? (multiple answers and subdivision into age groups possible) [Amoxicillin/clavulanic acid]
N/A

If you use antibiotic prophylaxis: which mediaction do you use? (multiple answers and subdivision into age groups possible) [Other]
N/A

Trimethoprim: which time schedule do you use? [Day(s) before VUS/VCUG, how many?]
N/A

Trimethoprim: which time schedule do you use? [Day of VUS/VCUG]
N/A

Trimethoprim: which time schedule do you use? [Day(s) after VUS/VCUG, how many?]
N/A

Nitrofurantoin: which time schedule do you use? [Day(s) before VUS/VCUG, how many?]
N/A

Nitrofurantoin: which time schedule do you use? [Day of VUS/VCUG]
N/A

Nitrofurantoin: which time schedule do you use? [Day(s) after VUS/VCUG, how many?]
N/A

Amoxicillin: which time schedule do you use? [Day(s) before VUS/VCUG, how many?]
N/A

Amoxicillin: which time schedule do you use? [Day of VUS/VCUG]
N/A

Amoxicillin: which time schedule do you use? [Day(s) after VUS/VCUG, how many?]
N/A

Cefaclor: which time schedule do you use? [Days(s) before VUS/VCUG, how many?]
N/A

Cefaclor: which time schedule do you use? [Day of VUS/VCUG]
N/A

Cefaclor: which time schedule do you use? [Day(s) after VUS/VCUG, how many?]
N/A

Amoxicillin/clavulanic acid: in what dosage? (Amoxicillin portion) [prophylaktic (10-20 mg/Kg bodyweight/d)]
N/A

Amoxicillin/clavulanic acid: in what dosage? (Amoxicillin portion) [double prophylactic (20-40 mg/Kg bodyweight/d)]
N/A

Amoxicillin/clavulanic acid: in what dosage? (Amoxicillin portion) [therapeutic (80-100 mg/Kg bodyweight/d)]
N/A

Amoxicillin/clavulanic acid: which time schedule do you use? [Day(s) before VUS/VCUG]
N/A

Amoxicillin/clavulanic acid: which time schedule do you use? [Day of VUS/VCUG]
N/A

Amoxicillin/clavulanic acid: which time schedule do you use? [Day(s) after VUS/VCUG]
N/A

Other: which time schedule do you use? [Day(s) before VUS/VCUG, how many?]
N/A

Other: which time schedule do you use? [Day of VUS/VCUG]
N/A

Other: which time schedule do you use? [Day(s) after VUS/VCUG, how many?]
N/A

Indication for prophylaxis

Which patients receive periinterventional antibiotic prophylaxis? (multiple answers possible) [all]
N/A

Which patients receive periinterventional antibiotic prophylaxis? (multiple answers possible) [patients post urinary tract infection/pyelonephritis/urosepsis]
N/A

Which patients receive periinterventional antibiotic prophylaxis? (multiple answers possible) [patients with urinary tract dilation in ultrasound]
N/A

Which patients receive periinterventional antibiotic prophylaxis? (multiple answers possible) [patients with prenatal urinary tract dilation]
N/A

Which patients receive periinterventional antibiotic prophylaxis? (multiple answers possible) [patients post VUR surgery]
N/A

Which patients receive periinterventional antibiotic prophylaxis? (multiple answers possible) [none]
N/A

Are there situations or clinical findings in the patient where you deviate from your prophylaxis standard (medication, dose, duration, age of the patient)? (multiple answers possible, please specify in answer box) [in case of already known vesicoureteral reflux (VUR)]
N/A

Are there situations or clinical findings in the patient where you deviate from your prophylaxis standard (medication, dose, duration, age of the patient)? (multiple answers possible, please specify in answer box) [in patients with recurrent urinary tract infections (UTI)]
N/A

Are there situations or clinical findings in the patient where you deviate from your prophylaxis standard (medication, dose, duration, age of the patient)? (multiple answers possible, please specify in answer box) [if multidrug-resistant organisms (MDRO) are detected in urine cultures]
N/A

Are there situations or clinical findings in the patient where you deviate from your prophylaxis standard (medication, dose, duration, age of the patient)? (multiple answers possible, please specify in answer box) [in newborns]
N/A

Are there situations in which you use intravenous prophylaxis?
N/A

Voiding analysis

Are there differences in antibiotic prophylaxis between VUS and VCUG in your department?
N/A

Is a non-pathological urine strip test required in preparation for VUS/VCUG in your department?
N/A

Is a non-pathological urine culture required in preparation for VUS/VCUG in your department?
N/A

Do patients only receive post-procedure prophylaxis if vesicoureteral reflux (VUR) is detected in VUS/VCUG?
N/A

Do other MUS/MCU findings (e.g. urethral valves) have an influence on post-procedure prophylaxis?
N/A

End of survey

May we contact you personally if we have further questions?
N/A

Survey response 60

Response ID
62

Last page
0

Start language
en

Seed
1960677879

Date started
2024-12-04 08:32:24

Date last action
2024-12-04 08:32:24

Standard Operation Procedure

Does your department have a defined protocol for infection prophylaxis in the context of VUS/VCUG?
N/A

Has this protocol been transformed into a written standard operating procedure (SOP)?
N/A

Antibiotic Prophylaxis

Are there situations/constellations in which you do not use antibiotic prophylaxis for VUS/VCUG?
N/A

Please specify these situations/constellations: [girl with first uncomplicated UTI without urinary tract dilation in ultrasound]
N/A

Please specify these situations/constellations: [boy with first uncomplicated UTI without urinary tract dilation in ultrasound]
N/A

Please specify these situations/constellations: [in patients with low grade urinary tract dilation (up to 2nd grade) and no UTI in medical history]
N/A

If you use antibiotic prophylaxis: which mediaction do you use? (multiple answers and subdivision into age groups possible) [Trimethoprim]
N/A

If you use antibiotic prophylaxis: which mediaction do you use? (multiple answers and subdivision into age groups possible) [Nitrofurantoin]
N/A

If you use antibiotic prophylaxis: which mediaction do you use? (multiple answers and subdivision into age groups possible) [Amoxicillin]
N/A

If you use antibiotic prophylaxis: which mediaction do you use? (multiple answers and subdivision into age groups possible) [Cefaclor]
N/A

If you use antibiotic prophylaxis: which mediaction do you use? (multiple answers and subdivision into age groups possible) [Amoxicillin/clavulanic acid]
N/A

If you use antibiotic prophylaxis: which mediaction do you use? (multiple answers and subdivision into age groups possible) [Other]
N/A

Trimethoprim: which time schedule do you use? [Day(s) before VUS/VCUG, how many?]
N/A

Trimethoprim: which time schedule do you use? [Day of VUS/VCUG]
N/A

Trimethoprim: which time schedule do you use? [Day(s) after VUS/VCUG, how many?]
N/A

Nitrofurantoin: which time schedule do you use? [Day(s) before VUS/VCUG, how many?]
N/A

Nitrofurantoin: which time schedule do you use? [Day of VUS/VCUG]
N/A

Nitrofurantoin: which time schedule do you use? [Day(s) after VUS/VCUG, how many?]
N/A

Amoxicillin: which time schedule do you use? [Day(s) before VUS/VCUG, how many?]
N/A

Amoxicillin: which time schedule do you use? [Day of VUS/VCUG]
N/A

Amoxicillin: which time schedule do you use? [Day(s) after VUS/VCUG, how many?]
N/A

Cefaclor: which time schedule do you use? [Days(s) before VUS/VCUG, how many?]
N/A

Cefaclor: which time schedule do you use? [Day of VUS/VCUG]
N/A

Cefaclor: which time schedule do you use? [Day(s) after VUS/VCUG, how many?]
N/A

Amoxicillin/clavulanic acid: in what dosage? (Amoxicillin portion) [prophylaktic (10-20 mg/Kg bodyweight/d)]
N/A

Amoxicillin/clavulanic acid: in what dosage? (Amoxicillin portion) [double prophylactic (20-40 mg/Kg bodyweight/d)]
N/A

Amoxicillin/clavulanic acid: in what dosage? (Amoxicillin portion) [therapeutic (80-100 mg/Kg bodyweight/d)]
N/A

Amoxicillin/clavulanic acid: which time schedule do you use? [Day(s) before VUS/VCUG]
N/A

Amoxicillin/clavulanic acid: which time schedule do you use? [Day of VUS/VCUG]
N/A

Amoxicillin/clavulanic acid: which time schedule do you use? [Day(s) after VUS/VCUG]
N/A

Other: which time schedule do you use? [Day(s) before VUS/VCUG, how many?]
N/A

Other: which time schedule do you use? [Day of VUS/VCUG]
N/A

Other: which time schedule do you use? [Day(s) after VUS/VCUG, how many?]
N/A

Indication for prophylaxis

Which patients receive periinterventional antibiotic prophylaxis? (multiple answers possible) [all]
N/A

Which patients receive periinterventional antibiotic prophylaxis? (multiple answers possible) [patients post urinary tract infection/pyelonephritis/urosepsis]
N/A

Which patients receive periinterventional antibiotic prophylaxis? (multiple answers possible) [patients with urinary tract dilation in ultrasound]
N/A

Which patients receive periinterventional antibiotic prophylaxis? (multiple answers possible) [patients with prenatal urinary tract dilation]
N/A

Which patients receive periinterventional antibiotic prophylaxis? (multiple answers possible) [patients post VUR surgery]
N/A

Which patients receive periinterventional antibiotic prophylaxis? (multiple answers possible) [none]
N/A

Are there situations or clinical findings in the patient where you deviate from your prophylaxis standard (medication, dose, duration, age of the patient)? (multiple answers possible, please specify in answer box) [in case of already known vesicoureteral reflux (VUR)]
N/A

Are there situations or clinical findings in the patient where you deviate from your prophylaxis standard (medication, dose, duration, age of the patient)? (multiple answers possible, please specify in answer box) [in patients with recurrent urinary tract infections (UTI)]
N/A

Are there situations or clinical findings in the patient where you deviate from your prophylaxis standard (medication, dose, duration, age of the patient)? (multiple answers possible, please specify in answer box) [if multidrug-resistant organisms (MDRO) are detected in urine cultures]
N/A

Are there situations or clinical findings in the patient where you deviate from your prophylaxis standard (medication, dose, duration, age of the patient)? (multiple answers possible, please specify in answer box) [in newborns]
N/A

Are there situations in which you use intravenous prophylaxis?
N/A

Voiding analysis

Are there differences in antibiotic prophylaxis between VUS and VCUG in your department?
N/A

Is a non-pathological urine strip test required in preparation for VUS/VCUG in your department?
N/A

Is a non-pathological urine culture required in preparation for VUS/VCUG in your department?
N/A

Do patients only receive post-procedure prophylaxis if vesicoureteral reflux (VUR) is detected in VUS/VCUG?
N/A

Do other MUS/MCU findings (e.g. urethral valves) have an influence on post-procedure prophylaxis?
N/A

End of survey

May we contact you personally if we have further questions?
N/A

Survey response 61

Response ID
63

Last page
1

Start language
en

Seed
1327906769

Date started
2024-12-04 18:25:59

Date last action
2024-12-04 18:26:19

Standard Operation Procedure

Who fills out this survey?
Pediatric Radiologist

Who fills out this survey? [Other]

In which type of hospital are you employed?
Community hospital (>50 beds for pediatric patients)

In which type of hospital are you employed? [Other]

In which country do you work?
United States

Does your department have a defined protocol for infection prophylaxis in the context of VUS/VCUG?
No

Has this protocol been transformed into a written standard operating procedure (SOP)?
N/A

Antibiotic Prophylaxis

Are there situations/constellations in which you do not use antibiotic prophylaxis for VUS/VCUG?
N/A

Please specify these situations/constellations: [girl with first uncomplicated UTI without urinary tract dilation in ultrasound]
N/A

Please specify these situations/constellations: [boy with first uncomplicated UTI without urinary tract dilation in ultrasound]
N/A

Please specify these situations/constellations: [in patients with low grade urinary tract dilation (up to 2nd grade) and no UTI in medical history]
N/A

If you use antibiotic prophylaxis: which mediaction do you use? (multiple answers and subdivision into age groups possible) [Trimethoprim]
N/A

If you use antibiotic prophylaxis: which mediaction do you use? (multiple answers and subdivision into age groups possible) [Nitrofurantoin]
N/A

If you use antibiotic prophylaxis: which mediaction do you use? (multiple answers and subdivision into age groups possible) [Amoxicillin]
N/A

If you use antibiotic prophylaxis: which mediaction do you use? (multiple answers and subdivision into age groups possible) [Cefaclor]
N/A

If you use antibiotic prophylaxis: which mediaction do you use? (multiple answers and subdivision into age groups possible) [Amoxicillin/clavulanic acid]
N/A

If you use antibiotic prophylaxis: which mediaction do you use? (multiple answers and subdivision into age groups possible) [Other]
N/A

Trimethoprim: which time schedule do you use? [Day(s) before VUS/VCUG, how many?]
N/A

Trimethoprim: which time schedule do you use? [Day of VUS/VCUG]
N/A

Trimethoprim: which time schedule do you use? [Day(s) after VUS/VCUG, how many?]
N/A

Nitrofurantoin: which time schedule do you use? [Day(s) before VUS/VCUG, how many?]
N/A

Nitrofurantoin: which time schedule do you use? [Day of VUS/VCUG]
N/A

Nitrofurantoin: which time schedule do you use? [Day(s) after VUS/VCUG, how many?]
N/A

Amoxicillin: which time schedule do you use? [Day(s) before VUS/VCUG, how many?]
N/A

Amoxicillin: which time schedule do you use? [Day of VUS/VCUG]
N/A

Amoxicillin: which time schedule do you use? [Day(s) after VUS/VCUG, how many?]
N/A

Cefaclor: which time schedule do you use? [Days(s) before VUS/VCUG, how many?]
N/A

Cefaclor: which time schedule do you use? [Day of VUS/VCUG]
N/A

Cefaclor: which time schedule do you use? [Day(s) after VUS/VCUG, how many?]
N/A

Amoxicillin/clavulanic acid: in what dosage? (Amoxicillin portion) [prophylaktic (10-20 mg/Kg bodyweight/d)]
N/A

Amoxicillin/clavulanic acid: in what dosage? (Amoxicillin portion) [double prophylactic (20-40 mg/Kg bodyweight/d)]
N/A

Amoxicillin/clavulanic acid: in what dosage? (Amoxicillin portion) [therapeutic (80-100 mg/Kg bodyweight/d)]
N/A

Amoxicillin/clavulanic acid: which time schedule do you use? [Day(s) before VUS/VCUG]
N/A

Amoxicillin/clavulanic acid: which time schedule do you use? [Day of VUS/VCUG]
N/A

Amoxicillin/clavulanic acid: which time schedule do you use? [Day(s) after VUS/VCUG]
N/A

Other: which time schedule do you use? [Day(s) before VUS/VCUG, how many?]
N/A

Other: which time schedule do you use? [Day of VUS/VCUG]
N/A

Other: which time schedule do you use? [Day(s) after VUS/VCUG, how many?]
N/A

Indication for prophylaxis

Which patients receive periinterventional antibiotic prophylaxis? (multiple answers possible) [all]
N/A

Which patients receive periinterventional antibiotic prophylaxis? (multiple answers possible) [patients post urinary tract infection/pyelonephritis/urosepsis]
N/A

Which patients receive periinterventional antibiotic prophylaxis? (multiple answers possible) [patients with urinary tract dilation in ultrasound]
N/A

Which patients receive periinterventional antibiotic prophylaxis? (multiple answers possible) [patients with prenatal urinary tract dilation]
N/A

Which patients receive periinterventional antibiotic prophylaxis? (multiple answers possible) [patients post VUR surgery]
N/A

Which patients receive periinterventional antibiotic prophylaxis? (multiple answers possible) [none]
N/A

Are there situations or clinical findings in the patient where you deviate from your prophylaxis standard (medication, dose, duration, age of the patient)? (multiple answers possible, please specify in answer box) [in case of already known vesicoureteral reflux (VUR)]
N/A

Are there situations or clinical findings in the patient where you deviate from your prophylaxis standard (medication, dose, duration, age of the patient)? (multiple answers possible, please specify in answer box) [in patients with recurrent urinary tract infections (UTI)]
N/A

Are there situations or clinical findings in the patient where you deviate from your prophylaxis standard (medication, dose, duration, age of the patient)? (multiple answers possible, please specify in answer box) [if multidrug-resistant organisms (MDRO) are detected in urine cultures]
N/A

Are there situations or clinical findings in the patient where you deviate from your prophylaxis standard (medication, dose, duration, age of the patient)? (multiple answers possible, please specify in answer box) [in newborns]
N/A

Are there situations in which you use intravenous prophylaxis?
N/A

Voiding analysis

Are there differences in antibiotic prophylaxis between VUS and VCUG in your department?
N/A

Is a non-pathological urine strip test required in preparation for VUS/VCUG in your department?
N/A

Is a non-pathological urine culture required in preparation for VUS/VCUG in your department?
N/A

Do patients only receive post-procedure prophylaxis if vesicoureteral reflux (VUR) is detected in VUS/VCUG?
N/A

Do other MUS/MCU findings (e.g. urethral valves) have an influence on post-procedure prophylaxis?
N/A

End of survey

May we contact you personally if we have further questions?
N/A

Survey response 62

Response ID
64

Last page
1

Start language
de

Seed
1482170554

Date started
2024-12-05 07:36:39

Date last action
2024-12-05 07:37:27

Standard Operation Procedure

Who fills out this survey?
Pediatric Radiologist

Who fills out this survey? [Other]

In which type of hospital are you employed?
Community hospital (>50 beds for pediatric patients)

In which type of hospital are you employed? [Other]

In which country do you work?
Deutschland

Does your department have a defined protocol for infection prophylaxis in the context of VUS/VCUG?
Yes

Has this protocol been transformed into a written standard operating procedure (SOP)?
Yes

Which department is the author of the SOP for infection prophylaxis in VUS/VCUG?
Pediatric radiology

Which department is the author of the SOP for infection prophylaxis in VUS/VCUG? [Other]

Antibiotic Prophylaxis

Are there situations/constellations in which you do not use antibiotic prophylaxis for VUS/VCUG?
N/A

Please specify these situations/constellations: [girl with first uncomplicated UTI without urinary tract dilation in ultrasound]
N/A

Please specify these situations/constellations: [boy with first uncomplicated UTI without urinary tract dilation in ultrasound]
N/A

Please specify these situations/constellations: [in patients with low grade urinary tract dilation (up to 2nd grade) and no UTI in medical history]
N/A

If you use antibiotic prophylaxis: which mediaction do you use? (multiple answers and subdivision into age groups possible) [Trimethoprim]
N/A

If you use antibiotic prophylaxis: which mediaction do you use? (multiple answers and subdivision into age groups possible) [Nitrofurantoin]
N/A

If you use antibiotic prophylaxis: which mediaction do you use? (multiple answers and subdivision into age groups possible) [Amoxicillin]
N/A

If you use antibiotic prophylaxis: which mediaction do you use? (multiple answers and subdivision into age groups possible) [Cefaclor]
N/A

If you use antibiotic prophylaxis: which mediaction do you use? (multiple answers and subdivision into age groups possible) [Amoxicillin/clavulanic acid]
N/A

If you use antibiotic prophylaxis: which mediaction do you use? (multiple answers and subdivision into age groups possible) [Other]
N/A

Trimethoprim: which time schedule do you use? [Day(s) before VUS/VCUG, how many?]
N/A

Trimethoprim: which time schedule do you use? [Day of VUS/VCUG]
N/A

Trimethoprim: which time schedule do you use? [Day(s) after VUS/VCUG, how many?]
N/A

Nitrofurantoin: which time schedule do you use? [Day(s) before VUS/VCUG, how many?]
N/A

Nitrofurantoin: which time schedule do you use? [Day of VUS/VCUG]
N/A

Nitrofurantoin: which time schedule do you use? [Day(s) after VUS/VCUG, how many?]
N/A

Amoxicillin: which time schedule do you use? [Day(s) before VUS/VCUG, how many?]
N/A

Amoxicillin: which time schedule do you use? [Day of VUS/VCUG]
N/A

Amoxicillin: which time schedule do you use? [Day(s) after VUS/VCUG, how many?]
N/A

Cefaclor: which time schedule do you use? [Days(s) before VUS/VCUG, how many?]
N/A

Cefaclor: which time schedule do you use? [Day of VUS/VCUG]
N/A

Cefaclor: which time schedule do you use? [Day(s) after VUS/VCUG, how many?]
N/A

Amoxicillin/clavulanic acid: in what dosage? (Amoxicillin portion) [prophylaktic (10-20 mg/Kg bodyweight/d)]
N/A

Amoxicillin/clavulanic acid: in what dosage? (Amoxicillin portion) [double prophylactic (20-40 mg/Kg bodyweight/d)]
N/A

Amoxicillin/clavulanic acid: in what dosage? (Amoxicillin portion) [therapeutic (80-100 mg/Kg bodyweight/d)]
N/A

Amoxicillin/clavulanic acid: which time schedule do you use? [Day(s) before VUS/VCUG]
N/A

Amoxicillin/clavulanic acid: which time schedule do you use? [Day of VUS/VCUG]
N/A

Amoxicillin/clavulanic acid: which time schedule do you use? [Day(s) after VUS/VCUG]
N/A

Other: which time schedule do you use? [Day(s) before VUS/VCUG, how many?]
N/A

Other: which time schedule do you use? [Day of VUS/VCUG]
N/A

Other: which time schedule do you use? [Day(s) after VUS/VCUG, how many?]
N/A

Indication for prophylaxis

Which patients receive periinterventional antibiotic prophylaxis? (multiple answers possible) [all]
N/A

Which patients receive periinterventional antibiotic prophylaxis? (multiple answers possible) [patients post urinary tract infection/pyelonephritis/urosepsis]
N/A

Which patients receive periinterventional antibiotic prophylaxis? (multiple answers possible) [patients with urinary tract dilation in ultrasound]
N/A

Which patients receive periinterventional antibiotic prophylaxis? (multiple answers possible) [patients with prenatal urinary tract dilation]
N/A

Which patients receive periinterventional antibiotic prophylaxis? (multiple answers possible) [patients post VUR surgery]
N/A

Which patients receive periinterventional antibiotic prophylaxis? (multiple answers possible) [none]
N/A

Are there situations or clinical findings in the patient where you deviate from your prophylaxis standard (medication, dose, duration, age of the patient)? (multiple answers possible, please specify in answer box) [in case of already known vesicoureteral reflux (VUR)]
N/A

Are there situations or clinical findings in the patient where you deviate from your prophylaxis standard (medication, dose, duration, age of the patient)? (multiple answers possible, please specify in answer box) [in patients with recurrent urinary tract infections (UTI)]
N/A

Are there situations or clinical findings in the patient where you deviate from your prophylaxis standard (medication, dose, duration, age of the patient)? (multiple answers possible, please specify in answer box) [if multidrug-resistant organisms (MDRO) are detected in urine cultures]
N/A

Are there situations or clinical findings in the patient where you deviate from your prophylaxis standard (medication, dose, duration, age of the patient)? (multiple answers possible, please specify in answer box) [in newborns]
N/A

Are there situations in which you use intravenous prophylaxis?
N/A

Voiding analysis

Are there differences in antibiotic prophylaxis between VUS and VCUG in your department?
N/A

Is a non-pathological urine strip test required in preparation for VUS/VCUG in your department?
N/A

Is a non-pathological urine culture required in preparation for VUS/VCUG in your department?
N/A

Do patients only receive post-procedure prophylaxis if vesicoureteral reflux (VUR) is detected in VUS/VCUG?
N/A

Do other MUS/MCU findings (e.g. urethral valves) have an influence on post-procedure prophylaxis?
N/A

End of survey

May we contact you personally if we have further questions?
N/A

Survey response 63

Response ID
65

Last page
0

Start language
de

Seed
1192466041

Date started
2024-12-05 14:01:26

Date last action
2024-12-05 14:01:26

Standard Operation Procedure

Does your department have a defined protocol for infection prophylaxis in the context of VUS/VCUG?
N/A

Has this protocol been transformed into a written standard operating procedure (SOP)?
N/A

Antibiotic Prophylaxis

Are there situations/constellations in which you do not use antibiotic prophylaxis for VUS/VCUG?
N/A

Please specify these situations/constellations: [girl with first uncomplicated UTI without urinary tract dilation in ultrasound]
N/A

Please specify these situations/constellations: [boy with first uncomplicated UTI without urinary tract dilation in ultrasound]
N/A

Please specify these situations/constellations: [in patients with low grade urinary tract dilation (up to 2nd grade) and no UTI in medical history]
N/A

If you use antibiotic prophylaxis: which mediaction do you use? (multiple answers and subdivision into age groups possible) [Trimethoprim]
N/A

If you use antibiotic prophylaxis: which mediaction do you use? (multiple answers and subdivision into age groups possible) [Nitrofurantoin]
N/A

If you use antibiotic prophylaxis: which mediaction do you use? (multiple answers and subdivision into age groups possible) [Amoxicillin]
N/A

If you use antibiotic prophylaxis: which mediaction do you use? (multiple answers and subdivision into age groups possible) [Cefaclor]
N/A

If you use antibiotic prophylaxis: which mediaction do you use? (multiple answers and subdivision into age groups possible) [Amoxicillin/clavulanic acid]
N/A

If you use antibiotic prophylaxis: which mediaction do you use? (multiple answers and subdivision into age groups possible) [Other]
N/A

Trimethoprim: which time schedule do you use? [Day(s) before VUS/VCUG, how many?]
N/A

Trimethoprim: which time schedule do you use? [Day of VUS/VCUG]
N/A

Trimethoprim: which time schedule do you use? [Day(s) after VUS/VCUG, how many?]
N/A

Nitrofurantoin: which time schedule do you use? [Day(s) before VUS/VCUG, how many?]
N/A

Nitrofurantoin: which time schedule do you use? [Day of VUS/VCUG]
N/A

Nitrofurantoin: which time schedule do you use? [Day(s) after VUS/VCUG, how many?]
N/A

Amoxicillin: which time schedule do you use? [Day(s) before VUS/VCUG, how many?]
N/A

Amoxicillin: which time schedule do you use? [Day of VUS/VCUG]
N/A

Amoxicillin: which time schedule do you use? [Day(s) after VUS/VCUG, how many?]
N/A

Cefaclor: which time schedule do you use? [Days(s) before VUS/VCUG, how many?]
N/A

Cefaclor: which time schedule do you use? [Day of VUS/VCUG]
N/A

Cefaclor: which time schedule do you use? [Day(s) after VUS/VCUG, how many?]
N/A

Amoxicillin/clavulanic acid: in what dosage? (Amoxicillin portion) [prophylaktic (10-20 mg/Kg bodyweight/d)]
N/A

Amoxicillin/clavulanic acid: in what dosage? (Amoxicillin portion) [double prophylactic (20-40 mg/Kg bodyweight/d)]
N/A

Amoxicillin/clavulanic acid: in what dosage? (Amoxicillin portion) [therapeutic (80-100 mg/Kg bodyweight/d)]
N/A

Amoxicillin/clavulanic acid: which time schedule do you use? [Day(s) before VUS/VCUG]
N/A

Amoxicillin/clavulanic acid: which time schedule do you use? [Day of VUS/VCUG]
N/A

Amoxicillin/clavulanic acid: which time schedule do you use? [Day(s) after VUS/VCUG]
N/A

Other: which time schedule do you use? [Day(s) before VUS/VCUG, how many?]
N/A

Other: which time schedule do you use? [Day of VUS/VCUG]
N/A

Other: which time schedule do you use? [Day(s) after VUS/VCUG, how many?]
N/A

Indication for prophylaxis

Which patients receive periinterventional antibiotic prophylaxis? (multiple answers possible) [all]
N/A

Which patients receive periinterventional antibiotic prophylaxis? (multiple answers possible) [patients post urinary tract infection/pyelonephritis/urosepsis]
N/A

Which patients receive periinterventional antibiotic prophylaxis? (multiple answers possible) [patients with urinary tract dilation in ultrasound]
N/A

Which patients receive periinterventional antibiotic prophylaxis? (multiple answers possible) [patients with prenatal urinary tract dilation]
N/A

Which patients receive periinterventional antibiotic prophylaxis? (multiple answers possible) [patients post VUR surgery]
N/A

Which patients receive periinterventional antibiotic prophylaxis? (multiple answers possible) [none]
N/A

Are there situations or clinical findings in the patient where you deviate from your prophylaxis standard (medication, dose, duration, age of the patient)? (multiple answers possible, please specify in answer box) [in case of already known vesicoureteral reflux (VUR)]
N/A

Are there situations or clinical findings in the patient where you deviate from your prophylaxis standard (medication, dose, duration, age of the patient)? (multiple answers possible, please specify in answer box) [in patients with recurrent urinary tract infections (UTI)]
N/A

Are there situations or clinical findings in the patient where you deviate from your prophylaxis standard (medication, dose, duration, age of the patient)? (multiple answers possible, please specify in answer box) [if multidrug-resistant organisms (MDRO) are detected in urine cultures]
N/A

Are there situations or clinical findings in the patient where you deviate from your prophylaxis standard (medication, dose, duration, age of the patient)? (multiple answers possible, please specify in answer box) [in newborns]
N/A

Are there situations in which you use intravenous prophylaxis?
N/A

Voiding analysis

Are there differences in antibiotic prophylaxis between VUS and VCUG in your department?
N/A

Is a non-pathological urine strip test required in preparation for VUS/VCUG in your department?
N/A

Is a non-pathological urine culture required in preparation for VUS/VCUG in your department?
N/A

Do patients only receive post-procedure prophylaxis if vesicoureteral reflux (VUR) is detected in VUS/VCUG?
N/A

Do other MUS/MCU findings (e.g. urethral valves) have an influence on post-procedure prophylaxis?
N/A

End of survey

May we contact you personally if we have further questions?
N/A

Survey response 64

Response ID
66

Last page
0

Start language
de

Seed
1371287730

Date started
2024-12-18 17:37:44

Date last action
2024-12-18 17:37:44

Standard Operation Procedure

Does your department have a defined protocol for infection prophylaxis in the context of VUS/VCUG?
N/A

Has this protocol been transformed into a written standard operating procedure (SOP)?
N/A

Antibiotic Prophylaxis

Are there situations/constellations in which you do not use antibiotic prophylaxis for VUS/VCUG?
N/A

Please specify these situations/constellations: [girl with first uncomplicated UTI without urinary tract dilation in ultrasound]
N/A

Please specify these situations/constellations: [boy with first uncomplicated UTI without urinary tract dilation in ultrasound]
N/A

Please specify these situations/constellations: [in patients with low grade urinary tract dilation (up to 2nd grade) and no UTI in medical history]
N/A

If you use antibiotic prophylaxis: which mediaction do you use? (multiple answers and subdivision into age groups possible) [Trimethoprim]
N/A

If you use antibiotic prophylaxis: which mediaction do you use? (multiple answers and subdivision into age groups possible) [Nitrofurantoin]
N/A

If you use antibiotic prophylaxis: which mediaction do you use? (multiple answers and subdivision into age groups possible) [Amoxicillin]
N/A

If you use antibiotic prophylaxis: which mediaction do you use? (multiple answers and subdivision into age groups possible) [Cefaclor]
N/A

If you use antibiotic prophylaxis: which mediaction do you use? (multiple answers and subdivision into age groups possible) [Amoxicillin/clavulanic acid]
N/A

If you use antibiotic prophylaxis: which mediaction do you use? (multiple answers and subdivision into age groups possible) [Other]
N/A

Trimethoprim: which time schedule do you use? [Day(s) before VUS/VCUG, how many?]
N/A

Trimethoprim: which time schedule do you use? [Day of VUS/VCUG]
N/A

Trimethoprim: which time schedule do you use? [Day(s) after VUS/VCUG, how many?]
N/A

Nitrofurantoin: which time schedule do you use? [Day(s) before VUS/VCUG, how many?]
N/A

Nitrofurantoin: which time schedule do you use? [Day of VUS/VCUG]
N/A

Nitrofurantoin: which time schedule do you use? [Day(s) after VUS/VCUG, how many?]
N/A

Amoxicillin: which time schedule do you use? [Day(s) before VUS/VCUG, how many?]
N/A

Amoxicillin: which time schedule do you use? [Day of VUS/VCUG]
N/A

Amoxicillin: which time schedule do you use? [Day(s) after VUS/VCUG, how many?]
N/A

Cefaclor: which time schedule do you use? [Days(s) before VUS/VCUG, how many?]
N/A

Cefaclor: which time schedule do you use? [Day of VUS/VCUG]
N/A

Cefaclor: which time schedule do you use? [Day(s) after VUS/VCUG, how many?]
N/A

Amoxicillin/clavulanic acid: in what dosage? (Amoxicillin portion) [prophylaktic (10-20 mg/Kg bodyweight/d)]
N/A

Amoxicillin/clavulanic acid: in what dosage? (Amoxicillin portion) [double prophylactic (20-40 mg/Kg bodyweight/d)]
N/A

Amoxicillin/clavulanic acid: in what dosage? (Amoxicillin portion) [therapeutic (80-100 mg/Kg bodyweight/d)]
N/A

Amoxicillin/clavulanic acid: which time schedule do you use? [Day(s) before VUS/VCUG]
N/A

Amoxicillin/clavulanic acid: which time schedule do you use? [Day of VUS/VCUG]
N/A

Amoxicillin/clavulanic acid: which time schedule do you use? [Day(s) after VUS/VCUG]
N/A

Other: which time schedule do you use? [Day(s) before VUS/VCUG, how many?]
N/A

Other: which time schedule do you use? [Day of VUS/VCUG]
N/A

Other: which time schedule do you use? [Day(s) after VUS/VCUG, how many?]
N/A

Indication for prophylaxis

Which patients receive periinterventional antibiotic prophylaxis? (multiple answers possible) [all]
N/A

Which patients receive periinterventional antibiotic prophylaxis? (multiple answers possible) [patients post urinary tract infection/pyelonephritis/urosepsis]
N/A

Which patients receive periinterventional antibiotic prophylaxis? (multiple answers possible) [patients with urinary tract dilation in ultrasound]
N/A

Which patients receive periinterventional antibiotic prophylaxis? (multiple answers possible) [patients with prenatal urinary tract dilation]
N/A

Which patients receive periinterventional antibiotic prophylaxis? (multiple answers possible) [patients post VUR surgery]
N/A

Which patients receive periinterventional antibiotic prophylaxis? (multiple answers possible) [none]
N/A

Are there situations or clinical findings in the patient where you deviate from your prophylaxis standard (medication, dose, duration, age of the patient)? (multiple answers possible, please specify in answer box) [in case of already known vesicoureteral reflux (VUR)]
N/A

Are there situations or clinical findings in the patient where you deviate from your prophylaxis standard (medication, dose, duration, age of the patient)? (multiple answers possible, please specify in answer box) [in patients with recurrent urinary tract infections (UTI)]
N/A

Are there situations or clinical findings in the patient where you deviate from your prophylaxis standard (medication, dose, duration, age of the patient)? (multiple answers possible, please specify in answer box) [if multidrug-resistant organisms (MDRO) are detected in urine cultures]
N/A

Are there situations or clinical findings in the patient where you deviate from your prophylaxis standard (medication, dose, duration, age of the patient)? (multiple answers possible, please specify in answer box) [in newborns]
N/A

Are there situations in which you use intravenous prophylaxis?
N/A

Voiding analysis

Are there differences in antibiotic prophylaxis between VUS and VCUG in your department?
N/A

Is a non-pathological urine strip test required in preparation for VUS/VCUG in your department?
N/A

Is a non-pathological urine culture required in preparation for VUS/VCUG in your department?
N/A

Do patients only receive post-procedure prophylaxis if vesicoureteral reflux (VUR) is detected in VUS/VCUG?
N/A

Do other MUS/MCU findings (e.g. urethral valves) have an influence on post-procedure prophylaxis?
N/A

End of survey

May we contact you personally if we have further questions?
N/A

Survey response 65

Response ID
67

Date submitted
2024-12-20 16:19:48

Last page
5

Start language
de

Seed
854130598

Date started
2024-12-20 16:13:34

Date last action
2024-12-20 16:19:48

Standard Operation Procedure

Who fills out this survey?
Pediatric Surgeon

Who fills out this survey? [Other]

In which type of hospital are you employed?
Community hospital (>50 beds for pediatric patients)

In which type of hospital are you employed? [Other]

In which country do you work?
Deutschland

Does your department have a defined protocol for infection prophylaxis in the context of VUS/VCUG?
Yes

Has this protocol been transformed into a written standard operating procedure (SOP)?
Yes

Which department is the author of the SOP for infection prophylaxis in VUS/VCUG?
Pediatric surgery

Which department is the author of the SOP for infection prophylaxis in VUS/VCUG? [Other]

Antibiotic Prophylaxis

Are there situations/constellations in which you do not use antibiotic prophylaxis for VUS/VCUG?
No

Please specify these situations/constellations: [girl with first uncomplicated UTI without urinary tract dilation in ultrasound]
N/A

Please specify these situations/constellations: [boy with first uncomplicated UTI without urinary tract dilation in ultrasound]
N/A

Please specify these situations/constellations: [in patients with low grade urinary tract dilation (up to 2nd grade) and no UTI in medical history]
N/A

If you use antibiotic prophylaxis: which mediaction do you use? (multiple answers and subdivision into age groups possible) [Trimethoprim]
Yes

If you use antibiotic prophylaxis: which mediaction do you use? (multiple answers and subdivision into age groups possible) [Nitrofurantoin]
No

If you use antibiotic prophylaxis: which mediaction do you use? (multiple answers and subdivision into age groups possible) [Amoxicillin]
No

If you use antibiotic prophylaxis: which mediaction do you use? (multiple answers and subdivision into age groups possible) [Cefaclor]
Yes

If you use antibiotic prophylaxis: which mediaction do you use? (multiple answers and subdivision into age groups possible) [Amoxicillin/clavulanic acid]
No

If you use antibiotic prophylaxis: which mediaction do you use? (multiple answers and subdivision into age groups possible) [Other]
No

Trimethoprim: in what dosage?
prophylactic (2 mg/kg bodyweight/d)

Trimethoprim: in what dosage? [Other]

Trimethoprim: for which age group do you use trimethoprim?
Älter 3 Monate

Trimethoprim: which time schedule do you use? [Day(s) before VUS/VCUG, how many?]
Yes

Trimethoprim: which time schedule do you use? [Day(s) before VUS/VCUG, how many?][Comment]
Vortag abends, Untersuchungstag morgens & abends

Trimethoprim: which time schedule do you use? [Day of VUS/VCUG]
Yes

Trimethoprim: which time schedule do you use? [Day of VUS/VCUG][Comment]

Trimethoprim: which time schedule do you use? [Day(s) after VUS/VCUG, how many?]
No

Trimethoprim: which time schedule do you use? [Day(s) after VUS/VCUG, how many?][Comment]

Nitrofurantoin: which time schedule do you use? [Day(s) before VUS/VCUG, how many?]
N/A

Nitrofurantoin: which time schedule do you use? [Day of VUS/VCUG]
N/A

Nitrofurantoin: which time schedule do you use? [Day(s) after VUS/VCUG, how many?]
N/A

Amoxicillin: which time schedule do you use? [Day(s) before VUS/VCUG, how many?]
N/A

Amoxicillin: which time schedule do you use? [Day of VUS/VCUG]
N/A

Amoxicillin: which time schedule do you use? [Day(s) after VUS/VCUG, how many?]
N/A

Cefaclor: in what dosage?
prophylactic (10 mg/kg bodyweight/d)

Cefaclor: in what dosage? [Other]

Cefaclor: for which age group do you use cefaclor?
< 3 Monate

Cefaclor: which time schedule do you use? [Days(s) before VUS/VCUG, how many?]
Yes

Cefaclor: which time schedule do you use? [Days(s) before VUS/VCUG, how many?][Comment]
Vortag abends, Untersuchungstag morgens & abends

Cefaclor: which time schedule do you use? [Day of VUS/VCUG]
Yes

Cefaclor: which time schedule do you use? [Day of VUS/VCUG][Comment]

Cefaclor: which time schedule do you use? [Day(s) after VUS/VCUG, how many?]
No

Cefaclor: which time schedule do you use? [Day(s) after VUS/VCUG, how many?][Comment]

Amoxicillin/clavulanic acid: in what dosage? (Amoxicillin portion) [prophylaktic (10-20 mg/Kg bodyweight/d)]
N/A

Amoxicillin/clavulanic acid: in what dosage? (Amoxicillin portion) [double prophylactic (20-40 mg/Kg bodyweight/d)]
N/A

Amoxicillin/clavulanic acid: in what dosage? (Amoxicillin portion) [therapeutic (80-100 mg/Kg bodyweight/d)]
N/A

Amoxicillin/clavulanic acid: which time schedule do you use? [Day(s) before VUS/VCUG]
N/A

Amoxicillin/clavulanic acid: which time schedule do you use? [Day of VUS/VCUG]
N/A

Amoxicillin/clavulanic acid: which time schedule do you use? [Day(s) after VUS/VCUG]
N/A

Other: which time schedule do you use? [Day(s) before VUS/VCUG, how many?]
N/A

Other: which time schedule do you use? [Day of VUS/VCUG]
N/A

Other: which time schedule do you use? [Day(s) after VUS/VCUG, how many?]
N/A

Indication for prophylaxis

Which patients receive periinterventional antibiotic prophylaxis? (multiple answers possible) [all]
Yes

Which patients receive periinterventional antibiotic prophylaxis? (multiple answers possible) [patients post urinary tract infection/pyelonephritis/urosepsis]
N/A

Which patients receive periinterventional antibiotic prophylaxis? (multiple answers possible) [patients with urinary tract dilation in ultrasound]
N/A

Which patients receive periinterventional antibiotic prophylaxis? (multiple answers possible) [patients with prenatal urinary tract dilation]
N/A

Which patients receive periinterventional antibiotic prophylaxis? (multiple answers possible) [patients post VUR surgery]
N/A

Which patients receive periinterventional antibiotic prophylaxis? (multiple answers possible) [none]
N/A

Are there situations or clinical findings in the patient where you deviate from your prophylaxis standard (medication, dose, duration, age of the patient)? (multiple answers possible, please specify in answer box) [in case of already known vesicoureteral reflux (VUR)]
Yes

Are there situations or clinical findings in the patient where you deviate from your prophylaxis standard (medication, dose, duration, age of the patient)? (multiple answers possible, please specify in answer box) [in case of already known vesicoureteral reflux (VUR)][Comment]
Wenn Dauerprophylaxe

Are there situations or clinical findings in the patient where you deviate from your prophylaxis standard (medication, dose, duration, age of the patient)? (multiple answers possible, please specify in answer box) [in patients with recurrent urinary tract infections (UTI)]
No

Are there situations or clinical findings in the patient where you deviate from your prophylaxis standard (medication, dose, duration, age of the patient)? (multiple answers possible, please specify in answer box) [in patients with recurrent urinary tract infections (UTI)][Comment]

Are there situations or clinical findings in the patient where you deviate from your prophylaxis standard (medication, dose, duration, age of the patient)? (multiple answers possible, please specify in answer box) [if multidrug-resistant organisms (MDRO) are detected in urine cultures]
No

Are there situations or clinical findings in the patient where you deviate from your prophylaxis standard (medication, dose, duration, age of the patient)? (multiple answers possible, please specify in answer box) [if multidrug-resistant organisms (MDRO) are detected in urine cultures][Comment]

Are there situations or clinical findings in the patient where you deviate from your prophylaxis standard (medication, dose, duration, age of the patient)? (multiple answers possible, please specify in answer box) [in newborns]
No

Are there situations or clinical findings in the patient where you deviate from your prophylaxis standard (medication, dose, duration, age of the patient)? (multiple answers possible, please specify in answer box) [in newborns][Comment]

Are there situations or clinical findings in the patient where you deviate from your prophylaxis standard (medication, dose, duration, age of the patient)? (multiple answers possible, please specify in answer box) [Other]

Are there situations or clinical findings in the patient where you deviate from your prophylaxis standard (medication, dose, duration, age of the patient)? (multiple answers possible, please specify in answer box) [Other comment]

Are there situations in which you use intravenous prophylaxis?
No

Voiding analysis

Are there differences in antibiotic prophylaxis between VUS and VCUG in your department?
No

Is a non-pathological urine strip test required in preparation for VUS/VCUG in your department?
No

Is a non-pathological urine culture required in preparation for VUS/VCUG in your department?
No

Do patients only receive post-procedure prophylaxis if vesicoureteral reflux (VUR) is detected in VUS/VCUG?
No

Do other MUS/MCU findings (e.g. urethral valves) have an influence on post-procedure prophylaxis?
No

End of survey

Thank you for taking the time to answer this survey!

May we contact you personally if we have further questions?
Yes

E-mail address:
benjamin.schwab-eckhardt@diakoneo.de

Survey response 66

Response ID
68

Last page
2

Start language
de

Seed
118932521

Date started
2024-12-20 16:20:31

Date last action
2024-12-20 16:25:18

Standard Operation Procedure

Who fills out this survey?
Pediatric Surgeon

Who fills out this survey? [Other]

In which type of hospital are you employed?
Community hospital (>50 beds for pediatric patients)

In which type of hospital are you employed? [Other]

In which country do you work?
Deutschland

Does your department have a defined protocol for infection prophylaxis in the context of VUS/VCUG?
Yes

Has this protocol been transformed into a written standard operating procedure (SOP)?
No

Antibiotic Prophylaxis

Are there situations/constellations in which you do not use antibiotic prophylaxis for VUS/VCUG?
Yes

Please specify these situations/constellations: [girl with first uncomplicated UTI without urinary tract dilation in ultrasound]
Yes

Please specify these situations/constellations: [boy with first uncomplicated UTI without urinary tract dilation in ultrasound]
No

Please specify these situations/constellations: [in patients with low grade urinary tract dilation (up to 2nd grade) and no UTI in medical history]
Yes

Please specify these situations/constellations: [Other]

If you use antibiotic prophylaxis: which mediaction do you use? (multiple answers and subdivision into age groups possible) [Trimethoprim]
Yes

If you use antibiotic prophylaxis: which mediaction do you use? (multiple answers and subdivision into age groups possible) [Nitrofurantoin]
No

If you use antibiotic prophylaxis: which mediaction do you use? (multiple answers and subdivision into age groups possible) [Amoxicillin]
No

If you use antibiotic prophylaxis: which mediaction do you use? (multiple answers and subdivision into age groups possible) [Cefaclor]
Yes

If you use antibiotic prophylaxis: which mediaction do you use? (multiple answers and subdivision into age groups possible) [Amoxicillin/clavulanic acid]
No

If you use antibiotic prophylaxis: which mediaction do you use? (multiple answers and subdivision into age groups possible) [Other]
No

Trimethoprim: in what dosage?
prophylactic (2 mg/kg bodyweight/d)

Trimethoprim: in what dosage? [Other]

Trimethoprim: for which age group do you use trimethoprim?
Ab 3 Monaten

Trimethoprim: which time schedule do you use? [Day(s) before VUS/VCUG, how many?]
Yes

Trimethoprim: which time schedule do you use? [Day(s) before VUS/VCUG, how many?][Comment]
HWI - bis 2 Tage danach

Trimethoprim: which time schedule do you use? [Day of VUS/VCUG]
No

Trimethoprim: which time schedule do you use? [Day of VUS/VCUG][Comment]

Trimethoprim: which time schedule do you use? [Day(s) after VUS/VCUG, how many?]
No

Trimethoprim: which time schedule do you use? [Day(s) after VUS/VCUG, how many?][Comment]

Nitrofurantoin: which time schedule do you use? [Day(s) before VUS/VCUG, how many?]
N/A

Nitrofurantoin: which time schedule do you use? [Day of VUS/VCUG]
N/A

Nitrofurantoin: which time schedule do you use? [Day(s) after VUS/VCUG, how many?]
N/A

Amoxicillin: which time schedule do you use? [Day(s) before VUS/VCUG, how many?]
N/A

Amoxicillin: which time schedule do you use? [Day of VUS/VCUG]
N/A

Amoxicillin: which time schedule do you use? [Day(s) after VUS/VCUG, how many?]
N/A

Cefaclor: in what dosage?
prophylactic (10 mg/kg bodyweight/d)

Cefaclor: in what dosage? [Other]

Cefaclor: for which age group do you use cefaclor?
Unter 3 Monaten

Cefaclor: which time schedule do you use? [Days(s) before VUS/VCUG, how many?]
Yes

Cefaclor: which time schedule do you use? [Days(s) before VUS/VCUG, how many?][Comment]
HWI - bis 2 Tage

Cefaclor: which time schedule do you use? [Day of VUS/VCUG]
No

Cefaclor: which time schedule do you use? [Day of VUS/VCUG][Comment]

Cefaclor: which time schedule do you use? [Day(s) after VUS/VCUG, how many?]
No

Cefaclor: which time schedule do you use? [Day(s) after VUS/VCUG, how many?][Comment]

Amoxicillin/clavulanic acid: in what dosage? (Amoxicillin portion) [prophylaktic (10-20 mg/Kg bodyweight/d)]
N/A

Amoxicillin/clavulanic acid: in what dosage? (Amoxicillin portion) [double prophylactic (20-40 mg/Kg bodyweight/d)]
N/A

Amoxicillin/clavulanic acid: in what dosage? (Amoxicillin portion) [therapeutic (80-100 mg/Kg bodyweight/d)]
N/A

Amoxicillin/clavulanic acid: which time schedule do you use? [Day(s) before VUS/VCUG]
N/A

Amoxicillin/clavulanic acid: which time schedule do you use? [Day of VUS/VCUG]
N/A

Amoxicillin/clavulanic acid: which time schedule do you use? [Day(s) after VUS/VCUG]
N/A

Other: which time schedule do you use? [Day(s) before VUS/VCUG, how many?]
N/A

Other: which time schedule do you use? [Day of VUS/VCUG]
N/A

Other: which time schedule do you use? [Day(s) after VUS/VCUG, how many?]
N/A

Indication for prophylaxis

Which patients receive periinterventional antibiotic prophylaxis? (multiple answers possible) [all]
N/A

Which patients receive periinterventional antibiotic prophylaxis? (multiple answers possible) [patients post urinary tract infection/pyelonephritis/urosepsis]
N/A

Which patients receive periinterventional antibiotic prophylaxis? (multiple answers possible) [patients with urinary tract dilation in ultrasound]
N/A

Which patients receive periinterventional antibiotic prophylaxis? (multiple answers possible) [patients with prenatal urinary tract dilation]
N/A

Which patients receive periinterventional antibiotic prophylaxis? (multiple answers possible) [patients post VUR surgery]
N/A

Which patients receive periinterventional antibiotic prophylaxis? (multiple answers possible) [none]
N/A

Are there situations or clinical findings in the patient where you deviate from your prophylaxis standard (medication, dose, duration, age of the patient)? (multiple answers possible, please specify in answer box) [in case of already known vesicoureteral reflux (VUR)]
N/A

Are there situations or clinical findings in the patient where you deviate from your prophylaxis standard (medication, dose, duration, age of the patient)? (multiple answers possible, please specify in answer box) [in patients with recurrent urinary tract infections (UTI)]
N/A

Are there situations or clinical findings in the patient where you deviate from your prophylaxis standard (medication, dose, duration, age of the patient)? (multiple answers possible, please specify in answer box) [if multidrug-resistant organisms (MDRO) are detected in urine cultures]
N/A

Are there situations or clinical findings in the patient where you deviate from your prophylaxis standard (medication, dose, duration, age of the patient)? (multiple answers possible, please specify in answer box) [in newborns]
N/A

Are there situations in which you use intravenous prophylaxis?
N/A

Voiding analysis

Are there differences in antibiotic prophylaxis between VUS and VCUG in your department?
N/A

Is a non-pathological urine strip test required in preparation for VUS/VCUG in your department?
N/A

Is a non-pathological urine culture required in preparation for VUS/VCUG in your department?
N/A

Do patients only receive post-procedure prophylaxis if vesicoureteral reflux (VUR) is detected in VUS/VCUG?
N/A

Do other MUS/MCU findings (e.g. urethral valves) have an influence on post-procedure prophylaxis?
N/A

End of survey

May we contact you personally if we have further questions?
N/A

Survey response 67

Response ID
69

Last page
1

Start language
de

Seed
2036821317

Date started
2024-12-20 16:27:18

Date last action
2024-12-20 16:27:56

Standard Operation Procedure

Who fills out this survey?
Pediatric Surgeon

Who fills out this survey? [Other]

In which type of hospital are you employed?
University Hospital

In which type of hospital are you employed? [Other]

In which country do you work?
Deutschland

Does your department have a defined protocol for infection prophylaxis in the context of VUS/VCUG?
No

Has this protocol been transformed into a written standard operating procedure (SOP)?
N/A

Antibiotic Prophylaxis

Are there situations/constellations in which you do not use antibiotic prophylaxis for VUS/VCUG?
N/A

Please specify these situations/constellations: [girl with first uncomplicated UTI without urinary tract dilation in ultrasound]
N/A

Please specify these situations/constellations: [boy with first uncomplicated UTI without urinary tract dilation in ultrasound]
N/A

Please specify these situations/constellations: [in patients with low grade urinary tract dilation (up to 2nd grade) and no UTI in medical history]
N/A

If you use antibiotic prophylaxis: which mediaction do you use? (multiple answers and subdivision into age groups possible) [Trimethoprim]
N/A

If you use antibiotic prophylaxis: which mediaction do you use? (multiple answers and subdivision into age groups possible) [Nitrofurantoin]
N/A

If you use antibiotic prophylaxis: which mediaction do you use? (multiple answers and subdivision into age groups possible) [Amoxicillin]
N/A

If you use antibiotic prophylaxis: which mediaction do you use? (multiple answers and subdivision into age groups possible) [Cefaclor]
N/A

If you use antibiotic prophylaxis: which mediaction do you use? (multiple answers and subdivision into age groups possible) [Amoxicillin/clavulanic acid]
N/A

If you use antibiotic prophylaxis: which mediaction do you use? (multiple answers and subdivision into age groups possible) [Other]
N/A

Trimethoprim: which time schedule do you use? [Day(s) before VUS/VCUG, how many?]
N/A

Trimethoprim: which time schedule do you use? [Day of VUS/VCUG]
N/A

Trimethoprim: which time schedule do you use? [Day(s) after VUS/VCUG, how many?]
N/A

Nitrofurantoin: which time schedule do you use? [Day(s) before VUS/VCUG, how many?]
N/A

Nitrofurantoin: which time schedule do you use? [Day of VUS/VCUG]
N/A

Nitrofurantoin: which time schedule do you use? [Day(s) after VUS/VCUG, how many?]
N/A

Amoxicillin: which time schedule do you use? [Day(s) before VUS/VCUG, how many?]
N/A

Amoxicillin: which time schedule do you use? [Day of VUS/VCUG]
N/A

Amoxicillin: which time schedule do you use? [Day(s) after VUS/VCUG, how many?]
N/A

Cefaclor: which time schedule do you use? [Days(s) before VUS/VCUG, how many?]
N/A

Cefaclor: which time schedule do you use? [Day of VUS/VCUG]
N/A

Cefaclor: which time schedule do you use? [Day(s) after VUS/VCUG, how many?]
N/A

Amoxicillin/clavulanic acid: in what dosage? (Amoxicillin portion) [prophylaktic (10-20 mg/Kg bodyweight/d)]
N/A

Amoxicillin/clavulanic acid: in what dosage? (Amoxicillin portion) [double prophylactic (20-40 mg/Kg bodyweight/d)]
N/A

Amoxicillin/clavulanic acid: in what dosage? (Amoxicillin portion) [therapeutic (80-100 mg/Kg bodyweight/d)]
N/A

Amoxicillin/clavulanic acid: which time schedule do you use? [Day(s) before VUS/VCUG]
N/A

Amoxicillin/clavulanic acid: which time schedule do you use? [Day of VUS/VCUG]
N/A

Amoxicillin/clavulanic acid: which time schedule do you use? [Day(s) after VUS/VCUG]
N/A

Other: which time schedule do you use? [Day(s) before VUS/VCUG, how many?]
N/A

Other: which time schedule do you use? [Day of VUS/VCUG]
N/A

Other: which time schedule do you use? [Day(s) after VUS/VCUG, how many?]
N/A

Indication for prophylaxis

Which patients receive periinterventional antibiotic prophylaxis? (multiple answers possible) [all]
N/A

Which patients receive periinterventional antibiotic prophylaxis? (multiple answers possible) [patients post urinary tract infection/pyelonephritis/urosepsis]
N/A

Which patients receive periinterventional antibiotic prophylaxis? (multiple answers possible) [patients with urinary tract dilation in ultrasound]
N/A

Which patients receive periinterventional antibiotic prophylaxis? (multiple answers possible) [patients with prenatal urinary tract dilation]
N/A

Which patients receive periinterventional antibiotic prophylaxis? (multiple answers possible) [patients post VUR surgery]
N/A

Which patients receive periinterventional antibiotic prophylaxis? (multiple answers possible) [none]
N/A

Are there situations or clinical findings in the patient where you deviate from your prophylaxis standard (medication, dose, duration, age of the patient)? (multiple answers possible, please specify in answer box) [in case of already known vesicoureteral reflux (VUR)]
N/A

Are there situations or clinical findings in the patient where you deviate from your prophylaxis standard (medication, dose, duration, age of the patient)? (multiple answers possible, please specify in answer box) [in patients with recurrent urinary tract infections (UTI)]
N/A

Are there situations or clinical findings in the patient where you deviate from your prophylaxis standard (medication, dose, duration, age of the patient)? (multiple answers possible, please specify in answer box) [if multidrug-resistant organisms (MDRO) are detected in urine cultures]
N/A

Are there situations or clinical findings in the patient where you deviate from your prophylaxis standard (medication, dose, duration, age of the patient)? (multiple answers possible, please specify in answer box) [in newborns]
N/A

Are there situations in which you use intravenous prophylaxis?
N/A

Voiding analysis

Are there differences in antibiotic prophylaxis between VUS and VCUG in your department?
N/A

Is a non-pathological urine strip test required in preparation for VUS/VCUG in your department?
N/A

Is a non-pathological urine culture required in preparation for VUS/VCUG in your department?
N/A

Do patients only receive post-procedure prophylaxis if vesicoureteral reflux (VUR) is detected in VUS/VCUG?
N/A

Do other MUS/MCU findings (e.g. urethral valves) have an influence on post-procedure prophylaxis?
N/A

End of survey

May we contact you personally if we have further questions?
N/A

Survey response 68

Response ID
70

Last page
1

Start language
de

Seed
1833035295

Date started
2024-12-20 16:48:04

Date last action
2024-12-20 16:48:45

Standard Operation Procedure

Who fills out this survey?
Pediatric Surgeon

Who fills out this survey? [Other]

In which type of hospital are you employed?
Community hospital (>50 beds for pediatric patients)

In which type of hospital are you employed? [Other]

In which country do you work?
Deutschland

Does your department have a defined protocol for infection prophylaxis in the context of VUS/VCUG?
No

Has this protocol been transformed into a written standard operating procedure (SOP)?
N/A

Antibiotic Prophylaxis

Are there situations/constellations in which you do not use antibiotic prophylaxis for VUS/VCUG?
N/A

Please specify these situations/constellations: [girl with first uncomplicated UTI without urinary tract dilation in ultrasound]
N/A

Please specify these situations/constellations: [boy with first uncomplicated UTI without urinary tract dilation in ultrasound]
N/A

Please specify these situations/constellations: [in patients with low grade urinary tract dilation (up to 2nd grade) and no UTI in medical history]
N/A

If you use antibiotic prophylaxis: which mediaction do you use? (multiple answers and subdivision into age groups possible) [Trimethoprim]
N/A

If you use antibiotic prophylaxis: which mediaction do you use? (multiple answers and subdivision into age groups possible) [Nitrofurantoin]
N/A

If you use antibiotic prophylaxis: which mediaction do you use? (multiple answers and subdivision into age groups possible) [Amoxicillin]
N/A

If you use antibiotic prophylaxis: which mediaction do you use? (multiple answers and subdivision into age groups possible) [Cefaclor]
N/A

If you use antibiotic prophylaxis: which mediaction do you use? (multiple answers and subdivision into age groups possible) [Amoxicillin/clavulanic acid]
N/A

If you use antibiotic prophylaxis: which mediaction do you use? (multiple answers and subdivision into age groups possible) [Other]
N/A

Trimethoprim: which time schedule do you use? [Day(s) before VUS/VCUG, how many?]
N/A

Trimethoprim: which time schedule do you use? [Day of VUS/VCUG]
N/A

Trimethoprim: which time schedule do you use? [Day(s) after VUS/VCUG, how many?]
N/A

Nitrofurantoin: which time schedule do you use? [Day(s) before VUS/VCUG, how many?]
N/A

Nitrofurantoin: which time schedule do you use? [Day of VUS/VCUG]
N/A

Nitrofurantoin: which time schedule do you use? [Day(s) after VUS/VCUG, how many?]
N/A

Amoxicillin: which time schedule do you use? [Day(s) before VUS/VCUG, how many?]
N/A

Amoxicillin: which time schedule do you use? [Day of VUS/VCUG]
N/A

Amoxicillin: which time schedule do you use? [Day(s) after VUS/VCUG, how many?]
N/A

Cefaclor: which time schedule do you use? [Days(s) before VUS/VCUG, how many?]
N/A

Cefaclor: which time schedule do you use? [Day of VUS/VCUG]
N/A

Cefaclor: which time schedule do you use? [Day(s) after VUS/VCUG, how many?]
N/A

Amoxicillin/clavulanic acid: in what dosage? (Amoxicillin portion) [prophylaktic (10-20 mg/Kg bodyweight/d)]
N/A

Amoxicillin/clavulanic acid: in what dosage? (Amoxicillin portion) [double prophylactic (20-40 mg/Kg bodyweight/d)]
N/A

Amoxicillin/clavulanic acid: in what dosage? (Amoxicillin portion) [therapeutic (80-100 mg/Kg bodyweight/d)]
N/A

Amoxicillin/clavulanic acid: which time schedule do you use? [Day(s) before VUS/VCUG]
N/A

Amoxicillin/clavulanic acid: which time schedule do you use? [Day of VUS/VCUG]
N/A

Amoxicillin/clavulanic acid: which time schedule do you use? [Day(s) after VUS/VCUG]
N/A

Other: which time schedule do you use? [Day(s) before VUS/VCUG, how many?]
N/A

Other: which time schedule do you use? [Day of VUS/VCUG]
N/A

Other: which time schedule do you use? [Day(s) after VUS/VCUG, how many?]
N/A

Indication for prophylaxis

Which patients receive periinterventional antibiotic prophylaxis? (multiple answers possible) [all]
N/A

Which patients receive periinterventional antibiotic prophylaxis? (multiple answers possible) [patients post urinary tract infection/pyelonephritis/urosepsis]
N/A

Which patients receive periinterventional antibiotic prophylaxis? (multiple answers possible) [patients with urinary tract dilation in ultrasound]
N/A

Which patients receive periinterventional antibiotic prophylaxis? (multiple answers possible) [patients with prenatal urinary tract dilation]
N/A

Which patients receive periinterventional antibiotic prophylaxis? (multiple answers possible) [patients post VUR surgery]
N/A

Which patients receive periinterventional antibiotic prophylaxis? (multiple answers possible) [none]
N/A

Are there situations or clinical findings in the patient where you deviate from your prophylaxis standard (medication, dose, duration, age of the patient)? (multiple answers possible, please specify in answer box) [in case of already known vesicoureteral reflux (VUR)]
N/A

Are there situations or clinical findings in the patient where you deviate from your prophylaxis standard (medication, dose, duration, age of the patient)? (multiple answers possible, please specify in answer box) [in patients with recurrent urinary tract infections (UTI)]
N/A

Are there situations or clinical findings in the patient where you deviate from your prophylaxis standard (medication, dose, duration, age of the patient)? (multiple answers possible, please specify in answer box) [if multidrug-resistant organisms (MDRO) are detected in urine cultures]
N/A

Are there situations or clinical findings in the patient where you deviate from your prophylaxis standard (medication, dose, duration, age of the patient)? (multiple answers possible, please specify in answer box) [in newborns]
N/A

Are there situations in which you use intravenous prophylaxis?
N/A

Voiding analysis

Are there differences in antibiotic prophylaxis between VUS and VCUG in your department?
N/A

Is a non-pathological urine strip test required in preparation for VUS/VCUG in your department?
N/A

Is a non-pathological urine culture required in preparation for VUS/VCUG in your department?
N/A

Do patients only receive post-procedure prophylaxis if vesicoureteral reflux (VUR) is detected in VUS/VCUG?
N/A

Do other MUS/MCU findings (e.g. urethral valves) have an influence on post-procedure prophylaxis?
N/A

End of survey

May we contact you personally if we have further questions?
N/A

Survey response 69

Response ID
71

Last page
1

Start language
de

Seed
1555048031

Date started
2024-12-20 17:27:20

Date last action
2024-12-20 17:28:03

Standard Operation Procedure

Who fills out this survey?
Pediatric Surgeon

Who fills out this survey? [Other]

In which type of hospital are you employed?
Community hospital (>50 beds for pediatric patients)

In which type of hospital are you employed? [Other]

In which country do you work?
Deutschland, Berlin

Does your department have a defined protocol for infection prophylaxis in the context of VUS/VCUG?
Yes

Has this protocol been transformed into a written standard operating procedure (SOP)?
No

Antibiotic Prophylaxis

Are there situations/constellations in which you do not use antibiotic prophylaxis for VUS/VCUG?
N/A

Please specify these situations/constellations: [girl with first uncomplicated UTI without urinary tract dilation in ultrasound]
N/A

Please specify these situations/constellations: [boy with first uncomplicated UTI without urinary tract dilation in ultrasound]
N/A

Please specify these situations/constellations: [in patients with low grade urinary tract dilation (up to 2nd grade) and no UTI in medical history]
N/A

If you use antibiotic prophylaxis: which mediaction do you use? (multiple answers and subdivision into age groups possible) [Trimethoprim]
N/A

If you use antibiotic prophylaxis: which mediaction do you use? (multiple answers and subdivision into age groups possible) [Nitrofurantoin]
N/A

If you use antibiotic prophylaxis: which mediaction do you use? (multiple answers and subdivision into age groups possible) [Amoxicillin]
N/A

If you use antibiotic prophylaxis: which mediaction do you use? (multiple answers and subdivision into age groups possible) [Cefaclor]
N/A

If you use antibiotic prophylaxis: which mediaction do you use? (multiple answers and subdivision into age groups possible) [Amoxicillin/clavulanic acid]
N/A

If you use antibiotic prophylaxis: which mediaction do you use? (multiple answers and subdivision into age groups possible) [Other]
N/A

Trimethoprim: which time schedule do you use? [Day(s) before VUS/VCUG, how many?]
N/A

Trimethoprim: which time schedule do you use? [Day of VUS/VCUG]
N/A

Trimethoprim: which time schedule do you use? [Day(s) after VUS/VCUG, how many?]
N/A

Nitrofurantoin: which time schedule do you use? [Day(s) before VUS/VCUG, how many?]
N/A

Nitrofurantoin: which time schedule do you use? [Day of VUS/VCUG]
N/A

Nitrofurantoin: which time schedule do you use? [Day(s) after VUS/VCUG, how many?]
N/A

Amoxicillin: which time schedule do you use? [Day(s) before VUS/VCUG, how many?]
N/A

Amoxicillin: which time schedule do you use? [Day of VUS/VCUG]
N/A

Amoxicillin: which time schedule do you use? [Day(s) after VUS/VCUG, how many?]
N/A

Cefaclor: which time schedule do you use? [Days(s) before VUS/VCUG, how many?]
N/A

Cefaclor: which time schedule do you use? [Day of VUS/VCUG]
N/A

Cefaclor: which time schedule do you use? [Day(s) after VUS/VCUG, how many?]
N/A

Amoxicillin/clavulanic acid: in what dosage? (Amoxicillin portion) [prophylaktic (10-20 mg/Kg bodyweight/d)]
N/A

Amoxicillin/clavulanic acid: in what dosage? (Amoxicillin portion) [double prophylactic (20-40 mg/Kg bodyweight/d)]
N/A

Amoxicillin/clavulanic acid: in what dosage? (Amoxicillin portion) [therapeutic (80-100 mg/Kg bodyweight/d)]
N/A

Amoxicillin/clavulanic acid: which time schedule do you use? [Day(s) before VUS/VCUG]
N/A

Amoxicillin/clavulanic acid: which time schedule do you use? [Day of VUS/VCUG]
N/A

Amoxicillin/clavulanic acid: which time schedule do you use? [Day(s) after VUS/VCUG]
N/A

Other: which time schedule do you use? [Day(s) before VUS/VCUG, how many?]
N/A

Other: which time schedule do you use? [Day of VUS/VCUG]
N/A

Other: which time schedule do you use? [Day(s) after VUS/VCUG, how many?]
N/A

Indication for prophylaxis

Which patients receive periinterventional antibiotic prophylaxis? (multiple answers possible) [all]
N/A

Which patients receive periinterventional antibiotic prophylaxis? (multiple answers possible) [patients post urinary tract infection/pyelonephritis/urosepsis]
N/A

Which patients receive periinterventional antibiotic prophylaxis? (multiple answers possible) [patients with urinary tract dilation in ultrasound]
N/A

Which patients receive periinterventional antibiotic prophylaxis? (multiple answers possible) [patients with prenatal urinary tract dilation]
N/A

Which patients receive periinterventional antibiotic prophylaxis? (multiple answers possible) [patients post VUR surgery]
N/A

Which patients receive periinterventional antibiotic prophylaxis? (multiple answers possible) [none]
N/A

Are there situations or clinical findings in the patient where you deviate from your prophylaxis standard (medication, dose, duration, age of the patient)? (multiple answers possible, please specify in answer box) [in case of already known vesicoureteral reflux (VUR)]
N/A

Are there situations or clinical findings in the patient where you deviate from your prophylaxis standard (medication, dose, duration, age of the patient)? (multiple answers possible, please specify in answer box) [in patients with recurrent urinary tract infections (UTI)]
N/A

Are there situations or clinical findings in the patient where you deviate from your prophylaxis standard (medication, dose, duration, age of the patient)? (multiple answers possible, please specify in answer box) [if multidrug-resistant organisms (MDRO) are detected in urine cultures]
N/A

Are there situations or clinical findings in the patient where you deviate from your prophylaxis standard (medication, dose, duration, age of the patient)? (multiple answers possible, please specify in answer box) [in newborns]
N/A

Are there situations in which you use intravenous prophylaxis?
N/A

Voiding analysis

Are there differences in antibiotic prophylaxis between VUS and VCUG in your department?
N/A

Is a non-pathological urine strip test required in preparation for VUS/VCUG in your department?
N/A

Is a non-pathological urine culture required in preparation for VUS/VCUG in your department?
N/A

Do patients only receive post-procedure prophylaxis if vesicoureteral reflux (VUR) is detected in VUS/VCUG?
N/A

Do other MUS/MCU findings (e.g. urethral valves) have an influence on post-procedure prophylaxis?
N/A

End of survey

May we contact you personally if we have further questions?
N/A

Survey response 70

Response ID
72

Last page
0

Start language
de

Seed
920334586

Date started
2024-12-20 17:36:22

Date last action
2024-12-20 17:36:36

Standard Operation Procedure

Who fills out this survey?
Pediatric Surgeon

Who fills out this survey? [Other]

In which type of hospital are you employed?

In which type of hospital are you employed? [Other]

In which country do you work?

Does your department have a defined protocol for infection prophylaxis in the context of VUS/VCUG?
N/A

Has this protocol been transformed into a written standard operating procedure (SOP)?
N/A

Antibiotic Prophylaxis

Are there situations/constellations in which you do not use antibiotic prophylaxis for VUS/VCUG?
N/A

Please specify these situations/constellations: [girl with first uncomplicated UTI without urinary tract dilation in ultrasound]
N/A

Please specify these situations/constellations: [boy with first uncomplicated UTI without urinary tract dilation in ultrasound]
N/A

Please specify these situations/constellations: [in patients with low grade urinary tract dilation (up to 2nd grade) and no UTI in medical history]
N/A

If you use antibiotic prophylaxis: which mediaction do you use? (multiple answers and subdivision into age groups possible) [Trimethoprim]
N/A

If you use antibiotic prophylaxis: which mediaction do you use? (multiple answers and subdivision into age groups possible) [Nitrofurantoin]
N/A

If you use antibiotic prophylaxis: which mediaction do you use? (multiple answers and subdivision into age groups possible) [Amoxicillin]
N/A

If you use antibiotic prophylaxis: which mediaction do you use? (multiple answers and subdivision into age groups possible) [Cefaclor]
N/A

If you use antibiotic prophylaxis: which mediaction do you use? (multiple answers and subdivision into age groups possible) [Amoxicillin/clavulanic acid]
N/A

If you use antibiotic prophylaxis: which mediaction do you use? (multiple answers and subdivision into age groups possible) [Other]
N/A

Trimethoprim: which time schedule do you use? [Day(s) before VUS/VCUG, how many?]
N/A

Trimethoprim: which time schedule do you use? [Day of VUS/VCUG]
N/A

Trimethoprim: which time schedule do you use? [Day(s) after VUS/VCUG, how many?]
N/A

Nitrofurantoin: which time schedule do you use? [Day(s) before VUS/VCUG, how many?]
N/A

Nitrofurantoin: which time schedule do you use? [Day of VUS/VCUG]
N/A

Nitrofurantoin: which time schedule do you use? [Day(s) after VUS/VCUG, how many?]
N/A

Amoxicillin: which time schedule do you use? [Day(s) before VUS/VCUG, how many?]
N/A

Amoxicillin: which time schedule do you use? [Day of VUS/VCUG]
N/A

Amoxicillin: which time schedule do you use? [Day(s) after VUS/VCUG, how many?]
N/A

Cefaclor: which time schedule do you use? [Days(s) before VUS/VCUG, how many?]
N/A

Cefaclor: which time schedule do you use? [Day of VUS/VCUG]
N/A

Cefaclor: which time schedule do you use? [Day(s) after VUS/VCUG, how many?]
N/A

Amoxicillin/clavulanic acid: in what dosage? (Amoxicillin portion) [prophylaktic (10-20 mg/Kg bodyweight/d)]
N/A

Amoxicillin/clavulanic acid: in what dosage? (Amoxicillin portion) [double prophylactic (20-40 mg/Kg bodyweight/d)]
N/A

Amoxicillin/clavulanic acid: in what dosage? (Amoxicillin portion) [therapeutic (80-100 mg/Kg bodyweight/d)]
N/A

Amoxicillin/clavulanic acid: which time schedule do you use? [Day(s) before VUS/VCUG]
N/A

Amoxicillin/clavulanic acid: which time schedule do you use? [Day of VUS/VCUG]
N/A

Amoxicillin/clavulanic acid: which time schedule do you use? [Day(s) after VUS/VCUG]
N/A

Other: which time schedule do you use? [Day(s) before VUS/VCUG, how many?]
N/A

Other: which time schedule do you use? [Day of VUS/VCUG]
N/A

Other: which time schedule do you use? [Day(s) after VUS/VCUG, how many?]
N/A

Indication for prophylaxis

Which patients receive periinterventional antibiotic prophylaxis? (multiple answers possible) [all]
N/A

Which patients receive periinterventional antibiotic prophylaxis? (multiple answers possible) [patients post urinary tract infection/pyelonephritis/urosepsis]
N/A

Which patients receive periinterventional antibiotic prophylaxis? (multiple answers possible) [patients with urinary tract dilation in ultrasound]
N/A

Which patients receive periinterventional antibiotic prophylaxis? (multiple answers possible) [patients with prenatal urinary tract dilation]
N/A

Which patients receive periinterventional antibiotic prophylaxis? (multiple answers possible) [patients post VUR surgery]
N/A

Which patients receive periinterventional antibiotic prophylaxis? (multiple answers possible) [none]
N/A

Are there situations or clinical findings in the patient where you deviate from your prophylaxis standard (medication, dose, duration, age of the patient)? (multiple answers possible, please specify in answer box) [in case of already known vesicoureteral reflux (VUR)]
N/A

Are there situations or clinical findings in the patient where you deviate from your prophylaxis standard (medication, dose, duration, age of the patient)? (multiple answers possible, please specify in answer box) [in patients with recurrent urinary tract infections (UTI)]
N/A

Are there situations or clinical findings in the patient where you deviate from your prophylaxis standard (medication, dose, duration, age of the patient)? (multiple answers possible, please specify in answer box) [if multidrug-resistant organisms (MDRO) are detected in urine cultures]
N/A

Are there situations or clinical findings in the patient where you deviate from your prophylaxis standard (medication, dose, duration, age of the patient)? (multiple answers possible, please specify in answer box) [in newborns]
N/A

Are there situations in which you use intravenous prophylaxis?
N/A

Voiding analysis

Are there differences in antibiotic prophylaxis between VUS and VCUG in your department?
N/A

Is a non-pathological urine strip test required in preparation for VUS/VCUG in your department?
N/A

Is a non-pathological urine culture required in preparation for VUS/VCUG in your department?
N/A

Do patients only receive post-procedure prophylaxis if vesicoureteral reflux (VUR) is detected in VUS/VCUG?
N/A

Do other MUS/MCU findings (e.g. urethral valves) have an influence on post-procedure prophylaxis?
N/A

End of survey

May we contact you personally if we have further questions?
N/A

Survey response 71

Response ID
73

Date submitted
2024-12-22 09:34:01

Last page
5

Start language
de

Seed
1454768332

Date started
2024-12-22 09:27:24

Date last action
2024-12-22 09:34:01

Standard Operation Procedure

Who fills out this survey?
Pediatric Surgeon

Who fills out this survey? [Other]

In which type of hospital are you employed?
Community hospital (>50 beds for pediatric patients)

In which type of hospital are you employed? [Other]

In which country do you work?
Brandenburg

Does your department have a defined protocol for infection prophylaxis in the context of VUS/VCUG?
No

Has this protocol been transformed into a written standard operating procedure (SOP)?
N/A

Antibiotic Prophylaxis

Are there situations/constellations in which you do not use antibiotic prophylaxis for VUS/VCUG?
No

Please specify these situations/constellations: [girl with first uncomplicated UTI without urinary tract dilation in ultrasound]
N/A

Please specify these situations/constellations: [boy with first uncomplicated UTI without urinary tract dilation in ultrasound]
N/A

Please specify these situations/constellations: [in patients with low grade urinary tract dilation (up to 2nd grade) and no UTI in medical history]
N/A

If you use antibiotic prophylaxis: which mediaction do you use? (multiple answers and subdivision into age groups possible) [Trimethoprim]
No

If you use antibiotic prophylaxis: which mediaction do you use? (multiple answers and subdivision into age groups possible) [Nitrofurantoin]
No

If you use antibiotic prophylaxis: which mediaction do you use? (multiple answers and subdivision into age groups possible) [Amoxicillin]
No

If you use antibiotic prophylaxis: which mediaction do you use? (multiple answers and subdivision into age groups possible) [Cefaclor]
No

If you use antibiotic prophylaxis: which mediaction do you use? (multiple answers and subdivision into age groups possible) [Amoxicillin/clavulanic acid]
No

If you use antibiotic prophylaxis: which mediaction do you use? (multiple answers and subdivision into age groups possible) [Other]
Yes

Trimethoprim: which time schedule do you use? [Day(s) before VUS/VCUG, how many?]
N/A

Trimethoprim: which time schedule do you use? [Day of VUS/VCUG]
N/A

Trimethoprim: which time schedule do you use? [Day(s) after VUS/VCUG, how many?]
N/A

Nitrofurantoin: which time schedule do you use? [Day(s) before VUS/VCUG, how many?]
N/A

Nitrofurantoin: which time schedule do you use? [Day of VUS/VCUG]
N/A

Nitrofurantoin: which time schedule do you use? [Day(s) after VUS/VCUG, how many?]
N/A

Amoxicillin: which time schedule do you use? [Day(s) before VUS/VCUG, how many?]
N/A

Amoxicillin: which time schedule do you use? [Day of VUS/VCUG]
N/A

Amoxicillin: which time schedule do you use? [Day(s) after VUS/VCUG, how many?]
N/A

Cefaclor: which time schedule do you use? [Days(s) before VUS/VCUG, how many?]
N/A

Cefaclor: which time schedule do you use? [Day of VUS/VCUG]
N/A

Cefaclor: which time schedule do you use? [Day(s) after VUS/VCUG, how many?]
N/A

Amoxicillin/clavulanic acid: in what dosage? (Amoxicillin portion) [prophylaktic (10-20 mg/Kg bodyweight/d)]
N/A

Amoxicillin/clavulanic acid: in what dosage? (Amoxicillin portion) [double prophylactic (20-40 mg/Kg bodyweight/d)]
N/A

Amoxicillin/clavulanic acid: in what dosage? (Amoxicillin portion) [therapeutic (80-100 mg/Kg bodyweight/d)]
N/A

Amoxicillin/clavulanic acid: which time schedule do you use? [Day(s) before VUS/VCUG]
N/A

Amoxicillin/clavulanic acid: which time schedule do you use? [Day of VUS/VCUG]
N/A

Amoxicillin/clavulanic acid: which time schedule do you use? [Day(s) after VUS/VCUG]
N/A

Other: which medication and in what dosage?
entsprechend des Resistogramms des letzten HWI

Other: for which age group do you use this medication?
alle

Other: which time schedule do you use? [Day(s) before VUS/VCUG, how many?]
Yes

Other: which time schedule do you use? [Day(s) before VUS/VCUG, how many?][Comment]
durchweg seit PN

Other: which time schedule do you use? [Day of VUS/VCUG]
No

Other: which time schedule do you use? [Day of VUS/VCUG][Comment]

Other: which time schedule do you use? [Day(s) after VUS/VCUG, how many?]
Yes

Other: which time schedule do you use? [Day(s) after VUS/VCUG, how many?][Comment]
wenn keine VUR nachgewiesen nach 2 Tagen ab, sonst weiter

Indication for prophylaxis

Which patients receive periinterventional antibiotic prophylaxis? (multiple answers possible) [all]
No

Which patients receive periinterventional antibiotic prophylaxis? (multiple answers possible) [patients post urinary tract infection/pyelonephritis/urosepsis]
Yes

Which patients receive periinterventional antibiotic prophylaxis? (multiple answers possible) [patients with urinary tract dilation in ultrasound]
Yes

Which patients receive periinterventional antibiotic prophylaxis? (multiple answers possible) [patients with prenatal urinary tract dilation]
No

Which patients receive periinterventional antibiotic prophylaxis? (multiple answers possible) [patients post VUR surgery]
No

Which patients receive periinterventional antibiotic prophylaxis? (multiple answers possible) [none]
No

Which patients receive periinterventional antibiotic prophylaxis? (multiple answers possible) [Other]

patients status post urinary tract infection/pyelonephritis/urosepsis: what is the minimum time between infection and VUS/VCUG? (in days)
28

In patients with urinary tract dilation in ultrasound: from what degree on? Image from Beetz R, Bokenkamp A, Brandis M et al (2001) Diagnosis of congenital dilatation of the urinary tract. Consensus Group of the Pediatric Nephrology Working Society in cooperation with the Pediatric Urology Working Group of the German Society of Urology and with the Pediatric Urology Working Society in the Germany Society of Pediatric Surgery. Urologe A 40:495–507 (quiz 8–9)
grade 3

Are there situations or clinical findings in the patient where you deviate from your prophylaxis standard (medication, dose, duration, age of the patient)? (multiple answers possible, please specify in answer box) [in case of already known vesicoureteral reflux (VUR)]
Yes

Are there situations or clinical findings in the patient where you deviate from your prophylaxis standard (medication, dose, duration, age of the patient)? (multiple answers possible, please specify in answer box) [in case of already known vesicoureteral reflux (VUR)][Comment]
Prophylaxe ja, allerdings vom Grad abhängig

Are there situations or clinical findings in the patient where you deviate from your prophylaxis standard (medication, dose, duration, age of the patient)? (multiple answers possible, please specify in answer box) [in patients with recurrent urinary tract infections (UTI)]
Yes

Are there situations or clinical findings in the patient where you deviate from your prophylaxis standard (medication, dose, duration, age of the patient)? (multiple answers possible, please specify in answer box) [in patients with recurrent urinary tract infections (UTI)][Comment]
Prophylaxe ja

Are there situations or clinical findings in the patient where you deviate from your prophylaxis standard (medication, dose, duration, age of the patient)? (multiple answers possible, please specify in answer box) [if multidrug-resistant organisms (MDRO) are detected in urine cultures]
No

Are there situations or clinical findings in the patient where you deviate from your prophylaxis standard (medication, dose, duration, age of the patient)? (multiple answers possible, please specify in answer box) [if multidrug-resistant organisms (MDRO) are detected in urine cultures][Comment]

Are there situations or clinical findings in the patient where you deviate from your prophylaxis standard (medication, dose, duration, age of the patient)? (multiple answers possible, please specify in answer box) [in newborns]
No

Are there situations or clinical findings in the patient where you deviate from your prophylaxis standard (medication, dose, duration, age of the patient)? (multiple answers possible, please specify in answer box) [in newborns][Comment]

Are there situations or clinical findings in the patient where you deviate from your prophylaxis standard (medication, dose, duration, age of the patient)? (multiple answers possible, please specify in answer box) [Other]

Are there situations or clinical findings in the patient where you deviate from your prophylaxis standard (medication, dose, duration, age of the patient)? (multiple answers possible, please specify in answer box) [Other comment]

Are there situations in which you use intravenous prophylaxis?
No

Voiding analysis

Are there differences in antibiotic prophylaxis between VUS and VCUG in your department?
No

Is a non-pathological urine strip test required in preparation for VUS/VCUG in your department?
Yes

What is the maximum amount of days accepted for urine strip test to be performed prior to the exam?
wird immer am Tag der Untersuchung gemacht !

Is a non-pathological urine culture required in preparation for VUS/VCUG in your department?
No

Do patients only receive post-procedure prophylaxis if vesicoureteral reflux (VUR) is detected in VUS/VCUG?
Yes

Please explain your approach:
wenn kein VUR antibiotische Prophylaxe nach 2 Tagen ab !

Do other MUS/MCU findings (e.g. urethral valves) have an influence on post-procedure prophylaxis?
No

End of survey

Thank you for taking the time to answer this survey!

May we contact you personally if we have further questions?
Yes

E-mail address:
petra.degenhardt@klinikumwb.de

Survey response 72

Response ID
74

Last page
0

Start language
de

Seed
2104542958

Date started
2024-12-23 09:07:16

Date last action
2024-12-23 09:07:16

Standard Operation Procedure

Does your department have a defined protocol for infection prophylaxis in the context of VUS/VCUG?
N/A

Has this protocol been transformed into a written standard operating procedure (SOP)?
N/A

Antibiotic Prophylaxis

Are there situations/constellations in which you do not use antibiotic prophylaxis for VUS/VCUG?
N/A

Please specify these situations/constellations: [girl with first uncomplicated UTI without urinary tract dilation in ultrasound]
N/A

Please specify these situations/constellations: [boy with first uncomplicated UTI without urinary tract dilation in ultrasound]
N/A

Please specify these situations/constellations: [in patients with low grade urinary tract dilation (up to 2nd grade) and no UTI in medical history]
N/A

If you use antibiotic prophylaxis: which mediaction do you use? (multiple answers and subdivision into age groups possible) [Trimethoprim]
N/A

If you use antibiotic prophylaxis: which mediaction do you use? (multiple answers and subdivision into age groups possible) [Nitrofurantoin]
N/A

If you use antibiotic prophylaxis: which mediaction do you use? (multiple answers and subdivision into age groups possible) [Amoxicillin]
N/A

If you use antibiotic prophylaxis: which mediaction do you use? (multiple answers and subdivision into age groups possible) [Cefaclor]
N/A

If you use antibiotic prophylaxis: which mediaction do you use? (multiple answers and subdivision into age groups possible) [Amoxicillin/clavulanic acid]
N/A

If you use antibiotic prophylaxis: which mediaction do you use? (multiple answers and subdivision into age groups possible) [Other]
N/A

Trimethoprim: which time schedule do you use? [Day(s) before VUS/VCUG, how many?]
N/A

Trimethoprim: which time schedule do you use? [Day of VUS/VCUG]
N/A

Trimethoprim: which time schedule do you use? [Day(s) after VUS/VCUG, how many?]
N/A

Nitrofurantoin: which time schedule do you use? [Day(s) before VUS/VCUG, how many?]
N/A

Nitrofurantoin: which time schedule do you use? [Day of VUS/VCUG]
N/A

Nitrofurantoin: which time schedule do you use? [Day(s) after VUS/VCUG, how many?]
N/A

Amoxicillin: which time schedule do you use? [Day(s) before VUS/VCUG, how many?]
N/A

Amoxicillin: which time schedule do you use? [Day of VUS/VCUG]
N/A

Amoxicillin: which time schedule do you use? [Day(s) after VUS/VCUG, how many?]
N/A

Cefaclor: which time schedule do you use? [Days(s) before VUS/VCUG, how many?]
N/A

Cefaclor: which time schedule do you use? [Day of VUS/VCUG]
N/A

Cefaclor: which time schedule do you use? [Day(s) after VUS/VCUG, how many?]
N/A

Amoxicillin/clavulanic acid: in what dosage? (Amoxicillin portion) [prophylaktic (10-20 mg/Kg bodyweight/d)]
N/A

Amoxicillin/clavulanic acid: in what dosage? (Amoxicillin portion) [double prophylactic (20-40 mg/Kg bodyweight/d)]
N/A

Amoxicillin/clavulanic acid: in what dosage? (Amoxicillin portion) [therapeutic (80-100 mg/Kg bodyweight/d)]
N/A

Amoxicillin/clavulanic acid: which time schedule do you use? [Day(s) before VUS/VCUG]
N/A

Amoxicillin/clavulanic acid: which time schedule do you use? [Day of VUS/VCUG]
N/A

Amoxicillin/clavulanic acid: which time schedule do you use? [Day(s) after VUS/VCUG]
N/A

Other: which time schedule do you use? [Day(s) before VUS/VCUG, how many?]
N/A

Other: which time schedule do you use? [Day of VUS/VCUG]
N/A

Other: which time schedule do you use? [Day(s) after VUS/VCUG, how many?]
N/A

Indication for prophylaxis

Which patients receive periinterventional antibiotic prophylaxis? (multiple answers possible) [all]
N/A

Which patients receive periinterventional antibiotic prophylaxis? (multiple answers possible) [patients post urinary tract infection/pyelonephritis/urosepsis]
N/A

Which patients receive periinterventional antibiotic prophylaxis? (multiple answers possible) [patients with urinary tract dilation in ultrasound]
N/A

Which patients receive periinterventional antibiotic prophylaxis? (multiple answers possible) [patients with prenatal urinary tract dilation]
N/A

Which patients receive periinterventional antibiotic prophylaxis? (multiple answers possible) [patients post VUR surgery]
N/A

Which patients receive periinterventional antibiotic prophylaxis? (multiple answers possible) [none]
N/A

Are there situations or clinical findings in the patient where you deviate from your prophylaxis standard (medication, dose, duration, age of the patient)? (multiple answers possible, please specify in answer box) [in case of already known vesicoureteral reflux (VUR)]
N/A

Are there situations or clinical findings in the patient where you deviate from your prophylaxis standard (medication, dose, duration, age of the patient)? (multiple answers possible, please specify in answer box) [in patients with recurrent urinary tract infections (UTI)]
N/A

Are there situations or clinical findings in the patient where you deviate from your prophylaxis standard (medication, dose, duration, age of the patient)? (multiple answers possible, please specify in answer box) [if multidrug-resistant organisms (MDRO) are detected in urine cultures]
N/A

Are there situations or clinical findings in the patient where you deviate from your prophylaxis standard (medication, dose, duration, age of the patient)? (multiple answers possible, please specify in answer box) [in newborns]
N/A

Are there situations in which you use intravenous prophylaxis?
N/A

Voiding analysis

Are there differences in antibiotic prophylaxis between VUS and VCUG in your department?
N/A

Is a non-pathological urine strip test required in preparation for VUS/VCUG in your department?
N/A

Is a non-pathological urine culture required in preparation for VUS/VCUG in your department?
N/A

Do patients only receive post-procedure prophylaxis if vesicoureteral reflux (VUR) is detected in VUS/VCUG?
N/A

Do other MUS/MCU findings (e.g. urethral valves) have an influence on post-procedure prophylaxis?
N/A

End of survey

May we contact you personally if we have further questions?
N/A

Survey response 73

Response ID
75

Last page
1

Start language
de

Seed
2046945430

Date started
2024-12-23 14:24:47

Date last action
2024-12-23 14:25:16

Standard Operation Procedure

Who fills out this survey?
Pediatric Surgeon

Who fills out this survey? [Other]

In which type of hospital are you employed?
Community hospital (>50 beds for pediatric patients)

In which type of hospital are you employed? [Other]

In which country do you work?
Deutschland

Does your department have a defined protocol for infection prophylaxis in the context of VUS/VCUG?
Yes

Has this protocol been transformed into a written standard operating procedure (SOP)?
Yes

Which department is the author of the SOP for infection prophylaxis in VUS/VCUG?
Pediatric surgery

Which department is the author of the SOP for infection prophylaxis in VUS/VCUG? [Other]

Antibiotic Prophylaxis

Are there situations/constellations in which you do not use antibiotic prophylaxis for VUS/VCUG?
N/A

Please specify these situations/constellations: [girl with first uncomplicated UTI without urinary tract dilation in ultrasound]
N/A

Please specify these situations/constellations: [boy with first uncomplicated UTI without urinary tract dilation in ultrasound]
N/A

Please specify these situations/constellations: [in patients with low grade urinary tract dilation (up to 2nd grade) and no UTI in medical history]
N/A

If you use antibiotic prophylaxis: which mediaction do you use? (multiple answers and subdivision into age groups possible) [Trimethoprim]
N/A

If you use antibiotic prophylaxis: which mediaction do you use? (multiple answers and subdivision into age groups possible) [Nitrofurantoin]
N/A

If you use antibiotic prophylaxis: which mediaction do you use? (multiple answers and subdivision into age groups possible) [Amoxicillin]
N/A

If you use antibiotic prophylaxis: which mediaction do you use? (multiple answers and subdivision into age groups possible) [Cefaclor]
N/A

If you use antibiotic prophylaxis: which mediaction do you use? (multiple answers and subdivision into age groups possible) [Amoxicillin/clavulanic acid]
N/A

If you use antibiotic prophylaxis: which mediaction do you use? (multiple answers and subdivision into age groups possible) [Other]
N/A

Trimethoprim: which time schedule do you use? [Day(s) before VUS/VCUG, how many?]
N/A

Trimethoprim: which time schedule do you use? [Day of VUS/VCUG]
N/A

Trimethoprim: which time schedule do you use? [Day(s) after VUS/VCUG, how many?]
N/A

Nitrofurantoin: which time schedule do you use? [Day(s) before VUS/VCUG, how many?]
N/A

Nitrofurantoin: which time schedule do you use? [Day of VUS/VCUG]
N/A

Nitrofurantoin: which time schedule do you use? [Day(s) after VUS/VCUG, how many?]
N/A

Amoxicillin: which time schedule do you use? [Day(s) before VUS/VCUG, how many?]
N/A

Amoxicillin: which time schedule do you use? [Day of VUS/VCUG]
N/A

Amoxicillin: which time schedule do you use? [Day(s) after VUS/VCUG, how many?]
N/A

Cefaclor: which time schedule do you use? [Days(s) before VUS/VCUG, how many?]
N/A

Cefaclor: which time schedule do you use? [Day of VUS/VCUG]
N/A

Cefaclor: which time schedule do you use? [Day(s) after VUS/VCUG, how many?]
N/A

Amoxicillin/clavulanic acid: in what dosage? (Amoxicillin portion) [prophylaktic (10-20 mg/Kg bodyweight/d)]
N/A

Amoxicillin/clavulanic acid: in what dosage? (Amoxicillin portion) [double prophylactic (20-40 mg/Kg bodyweight/d)]
N/A

Amoxicillin/clavulanic acid: in what dosage? (Amoxicillin portion) [therapeutic (80-100 mg/Kg bodyweight/d)]
N/A

Amoxicillin/clavulanic acid: which time schedule do you use? [Day(s) before VUS/VCUG]
N/A

Amoxicillin/clavulanic acid: which time schedule do you use? [Day of VUS/VCUG]
N/A

Amoxicillin/clavulanic acid: which time schedule do you use? [Day(s) after VUS/VCUG]
N/A

Other: which time schedule do you use? [Day(s) before VUS/VCUG, how many?]
N/A

Other: which time schedule do you use? [Day of VUS/VCUG]
N/A

Other: which time schedule do you use? [Day(s) after VUS/VCUG, how many?]
N/A

Indication for prophylaxis

Which patients receive periinterventional antibiotic prophylaxis? (multiple answers possible) [all]
N/A

Which patients receive periinterventional antibiotic prophylaxis? (multiple answers possible) [patients post urinary tract infection/pyelonephritis/urosepsis]
N/A

Which patients receive periinterventional antibiotic prophylaxis? (multiple answers possible) [patients with urinary tract dilation in ultrasound]
N/A

Which patients receive periinterventional antibiotic prophylaxis? (multiple answers possible) [patients with prenatal urinary tract dilation]
N/A

Which patients receive periinterventional antibiotic prophylaxis? (multiple answers possible) [patients post VUR surgery]
N/A

Which patients receive periinterventional antibiotic prophylaxis? (multiple answers possible) [none]
N/A

Are there situations or clinical findings in the patient where you deviate from your prophylaxis standard (medication, dose, duration, age of the patient)? (multiple answers possible, please specify in answer box) [in case of already known vesicoureteral reflux (VUR)]
N/A

Are there situations or clinical findings in the patient where you deviate from your prophylaxis standard (medication, dose, duration, age of the patient)? (multiple answers possible, please specify in answer box) [in patients with recurrent urinary tract infections (UTI)]
N/A

Are there situations or clinical findings in the patient where you deviate from your prophylaxis standard (medication, dose, duration, age of the patient)? (multiple answers possible, please specify in answer box) [if multidrug-resistant organisms (MDRO) are detected in urine cultures]
N/A

Are there situations or clinical findings in the patient where you deviate from your prophylaxis standard (medication, dose, duration, age of the patient)? (multiple answers possible, please specify in answer box) [in newborns]
N/A

Are there situations in which you use intravenous prophylaxis?
N/A

Voiding analysis

Are there differences in antibiotic prophylaxis between VUS and VCUG in your department?
N/A

Is a non-pathological urine strip test required in preparation for VUS/VCUG in your department?
N/A

Is a non-pathological urine culture required in preparation for VUS/VCUG in your department?
N/A

Do patients only receive post-procedure prophylaxis if vesicoureteral reflux (VUR) is detected in VUS/VCUG?
N/A

Do other MUS/MCU findings (e.g. urethral valves) have an influence on post-procedure prophylaxis?
N/A

End of survey

May we contact you personally if we have further questions?
N/A

Survey response 74

Response ID
76

Date submitted
2024-12-25 17:25:58

Last page
5

Start language
de

Seed
2036406561

Date started
2024-12-25 17:19:50

Date last action
2024-12-25 17:25:58

Standard Operation Procedure

Who fills out this survey?
Pediatric Surgeon

Who fills out this survey? [Other]

In which type of hospital are you employed?
Other

In which type of hospital are you employed? [Other]
Helios Klinik

In which country do you work?
Berlin, Deutschland

Does your department have a defined protocol for infection prophylaxis in the context of VUS/VCUG?
Yes

Has this protocol been transformed into a written standard operating procedure (SOP)?
No

Antibiotic Prophylaxis

Are there situations/constellations in which you do not use antibiotic prophylaxis for VUS/VCUG?
Yes

Please specify these situations/constellations: [girl with first uncomplicated UTI without urinary tract dilation in ultrasound]
No

Please specify these situations/constellations: [boy with first uncomplicated UTI without urinary tract dilation in ultrasound]
No

Please specify these situations/constellations: [in patients with low grade urinary tract dilation (up to 2nd grade) and no UTI in medical history]
No

Please specify these situations/constellations: [Other]
wenn ich im MUS keinen Reflux feststelle

If you use antibiotic prophylaxis: which mediaction do you use? (multiple answers and subdivision into age groups possible) [Trimethoprim]
Yes

If you use antibiotic prophylaxis: which mediaction do you use? (multiple answers and subdivision into age groups possible) [Nitrofurantoin]
No

If you use antibiotic prophylaxis: which mediaction do you use? (multiple answers and subdivision into age groups possible) [Amoxicillin]
No

If you use antibiotic prophylaxis: which mediaction do you use? (multiple answers and subdivision into age groups possible) [Cefaclor]
No

If you use antibiotic prophylaxis: which mediaction do you use? (multiple answers and subdivision into age groups possible) [Amoxicillin/clavulanic acid]
No

If you use antibiotic prophylaxis: which mediaction do you use? (multiple answers and subdivision into age groups possible) [Other]
No

Trimethoprim: in what dosage?
therapeutic (6 mg/kg bodyweight/d)

Trimethoprim: in what dosage? [Other]

Trimethoprim: for which age group do you use trimethoprim?
ab 6. LW

Trimethoprim: which time schedule do you use? [Day(s) before VUS/VCUG, how many?]
No

Trimethoprim: which time schedule do you use? [Day(s) before VUS/VCUG, how many?][Comment]

Trimethoprim: which time schedule do you use? [Day of VUS/VCUG]
Yes

Trimethoprim: which time schedule do you use? [Day of VUS/VCUG][Comment]

Trimethoprim: which time schedule do you use? [Day(s) after VUS/VCUG, how many?]
Yes

Trimethoprim: which time schedule do you use? [Day(s) after VUS/VCUG, how many?][Comment]
3 Tage

Nitrofurantoin: which time schedule do you use? [Day(s) before VUS/VCUG, how many?]
N/A

Nitrofurantoin: which time schedule do you use? [Day of VUS/VCUG]
N/A

Nitrofurantoin: which time schedule do you use? [Day(s) after VUS/VCUG, how many?]
N/A

Amoxicillin: which time schedule do you use? [Day(s) before VUS/VCUG, how many?]
N/A

Amoxicillin: which time schedule do you use? [Day of VUS/VCUG]
N/A

Amoxicillin: which time schedule do you use? [Day(s) after VUS/VCUG, how many?]
N/A

Cefaclor: which time schedule do you use? [Days(s) before VUS/VCUG, how many?]
N/A

Cefaclor: which time schedule do you use? [Day of VUS/VCUG]
N/A

Cefaclor: which time schedule do you use? [Day(s) after VUS/VCUG, how many?]
N/A

Amoxicillin/clavulanic acid: in what dosage? (Amoxicillin portion) [prophylaktic (10-20 mg/Kg bodyweight/d)]
N/A

Amoxicillin/clavulanic acid: in what dosage? (Amoxicillin portion) [double prophylactic (20-40 mg/Kg bodyweight/d)]
N/A

Amoxicillin/clavulanic acid: in what dosage? (Amoxicillin portion) [therapeutic (80-100 mg/Kg bodyweight/d)]
N/A

Amoxicillin/clavulanic acid: which time schedule do you use? [Day(s) before VUS/VCUG]
N/A

Amoxicillin/clavulanic acid: which time schedule do you use? [Day of VUS/VCUG]
N/A

Amoxicillin/clavulanic acid: which time schedule do you use? [Day(s) after VUS/VCUG]
N/A

Other: which time schedule do you use? [Day(s) before VUS/VCUG, how many?]
N/A

Other: which time schedule do you use? [Day of VUS/VCUG]
N/A

Other: which time schedule do you use? [Day(s) after VUS/VCUG, how many?]
N/A

Indication for prophylaxis

Which patients receive periinterventional antibiotic prophylaxis? (multiple answers possible) [all]
No

Which patients receive periinterventional antibiotic prophylaxis? (multiple answers possible) [patients post urinary tract infection/pyelonephritis/urosepsis]
No

Which patients receive periinterventional antibiotic prophylaxis? (multiple answers possible) [patients with urinary tract dilation in ultrasound]
No

Which patients receive periinterventional antibiotic prophylaxis? (multiple answers possible) [patients with prenatal urinary tract dilation]
No

Which patients receive periinterventional antibiotic prophylaxis? (multiple answers possible) [patients post VUR surgery]
No

Which patients receive periinterventional antibiotic prophylaxis? (multiple answers possible) [none]
No

Which patients receive periinterventional antibiotic prophylaxis? (multiple answers possible) [Other]
wenn ich einen Reflux nachweise

Are there situations or clinical findings in the patient where you deviate from your prophylaxis standard (medication, dose, duration, age of the patient)? (multiple answers possible, please specify in answer box) [in case of already known vesicoureteral reflux (VUR)]
Yes

Are there situations or clinical findings in the patient where you deviate from your prophylaxis standard (medication, dose, duration, age of the patient)? (multiple answers possible, please specify in answer box) [in case of already known vesicoureteral reflux (VUR)][Comment]

Are there situations or clinical findings in the patient where you deviate from your prophylaxis standard (medication, dose, duration, age of the patient)? (multiple answers possible, please specify in answer box) [in patients with recurrent urinary tract infections (UTI)]
Yes

Are there situations or clinical findings in the patient where you deviate from your prophylaxis standard (medication, dose, duration, age of the patient)? (multiple answers possible, please specify in answer box) [in patients with recurrent urinary tract infections (UTI)][Comment]

Are there situations or clinical findings in the patient where you deviate from your prophylaxis standard (medication, dose, duration, age of the patient)? (multiple answers possible, please specify in answer box) [if multidrug-resistant organisms (MDRO) are detected in urine cultures]
No

Are there situations or clinical findings in the patient where you deviate from your prophylaxis standard (medication, dose, duration, age of the patient)? (multiple answers possible, please specify in answer box) [if multidrug-resistant organisms (MDRO) are detected in urine cultures][Comment]

Are there situations or clinical findings in the patient where you deviate from your prophylaxis standard (medication, dose, duration, age of the patient)? (multiple answers possible, please specify in answer box) [in newborns]
Yes

Are there situations or clinical findings in the patient where you deviate from your prophylaxis standard (medication, dose, duration, age of the patient)? (multiple answers possible, please specify in answer box) [in newborns][Comment]

Are there situations or clinical findings in the patient where you deviate from your prophylaxis standard (medication, dose, duration, age of the patient)? (multiple answers possible, please specify in answer box) [Other]

Are there situations or clinical findings in the patient where you deviate from your prophylaxis standard (medication, dose, duration, age of the patient)? (multiple answers possible, please specify in answer box) [Other comment]

Are there situations in which you use intravenous prophylaxis?
No

Voiding analysis

Are there differences in antibiotic prophylaxis between VUS and VCUG in your department?
No

Is a non-pathological urine strip test required in preparation for VUS/VCUG in your department?
Yes

What is the maximum amount of days accepted for urine strip test to be performed prior to the exam?
direkt vor der Untersuchung bei Einlage des Katheters

Is a non-pathological urine culture required in preparation for VUS/VCUG in your department?
No

Do patients only receive post-procedure prophylaxis if vesicoureteral reflux (VUR) is detected in VUS/VCUG?
Yes

Please explain your approach:
nur dann besteht m.E. die Gefahr einer PN durch die Untersuchung

Do other MUS/MCU findings (e.g. urethral valves) have an influence on post-procedure prophylaxis?
No

End of survey

Thank you for taking the time to answer this survey!

May we contact you personally if we have further questions?
Yes

E-mail address:
gabriele.buelchmann@helios-gesundheit.de

Survey response 75

Response ID
77

Last page
0

Start language
de

Seed
944364677

Date started
2024-12-30 12:25:43

Date last action
2024-12-30 12:25:43

Standard Operation Procedure

Does your department have a defined protocol for infection prophylaxis in the context of VUS/VCUG?
N/A

Has this protocol been transformed into a written standard operating procedure (SOP)?
N/A

Antibiotic Prophylaxis

Are there situations/constellations in which you do not use antibiotic prophylaxis for VUS/VCUG?
N/A

Please specify these situations/constellations: [girl with first uncomplicated UTI without urinary tract dilation in ultrasound]
N/A

Please specify these situations/constellations: [boy with first uncomplicated UTI without urinary tract dilation in ultrasound]
N/A

Please specify these situations/constellations: [in patients with low grade urinary tract dilation (up to 2nd grade) and no UTI in medical history]
N/A

If you use antibiotic prophylaxis: which mediaction do you use? (multiple answers and subdivision into age groups possible) [Trimethoprim]
N/A

If you use antibiotic prophylaxis: which mediaction do you use? (multiple answers and subdivision into age groups possible) [Nitrofurantoin]
N/A

If you use antibiotic prophylaxis: which mediaction do you use? (multiple answers and subdivision into age groups possible) [Amoxicillin]
N/A

If you use antibiotic prophylaxis: which mediaction do you use? (multiple answers and subdivision into age groups possible) [Cefaclor]
N/A

If you use antibiotic prophylaxis: which mediaction do you use? (multiple answers and subdivision into age groups possible) [Amoxicillin/clavulanic acid]
N/A

If you use antibiotic prophylaxis: which mediaction do you use? (multiple answers and subdivision into age groups possible) [Other]
N/A

Trimethoprim: which time schedule do you use? [Day(s) before VUS/VCUG, how many?]
N/A

Trimethoprim: which time schedule do you use? [Day of VUS/VCUG]
N/A

Trimethoprim: which time schedule do you use? [Day(s) after VUS/VCUG, how many?]
N/A

Nitrofurantoin: which time schedule do you use? [Day(s) before VUS/VCUG, how many?]
N/A

Nitrofurantoin: which time schedule do you use? [Day of VUS/VCUG]
N/A

Nitrofurantoin: which time schedule do you use? [Day(s) after VUS/VCUG, how many?]
N/A

Amoxicillin: which time schedule do you use? [Day(s) before VUS/VCUG, how many?]
N/A

Amoxicillin: which time schedule do you use? [Day of VUS/VCUG]
N/A

Amoxicillin: which time schedule do you use? [Day(s) after VUS/VCUG, how many?]
N/A

Cefaclor: which time schedule do you use? [Days(s) before VUS/VCUG, how many?]
N/A

Cefaclor: which time schedule do you use? [Day of VUS/VCUG]
N/A

Cefaclor: which time schedule do you use? [Day(s) after VUS/VCUG, how many?]
N/A

Amoxicillin/clavulanic acid: in what dosage? (Amoxicillin portion) [prophylaktic (10-20 mg/Kg bodyweight/d)]
N/A

Amoxicillin/clavulanic acid: in what dosage? (Amoxicillin portion) [double prophylactic (20-40 mg/Kg bodyweight/d)]
N/A

Amoxicillin/clavulanic acid: in what dosage? (Amoxicillin portion) [therapeutic (80-100 mg/Kg bodyweight/d)]
N/A

Amoxicillin/clavulanic acid: which time schedule do you use? [Day(s) before VUS/VCUG]
N/A

Amoxicillin/clavulanic acid: which time schedule do you use? [Day of VUS/VCUG]
N/A

Amoxicillin/clavulanic acid: which time schedule do you use? [Day(s) after VUS/VCUG]
N/A

Other: which time schedule do you use? [Day(s) before VUS/VCUG, how many?]
N/A

Other: which time schedule do you use? [Day of VUS/VCUG]
N/A

Other: which time schedule do you use? [Day(s) after VUS/VCUG, how many?]
N/A

Indication for prophylaxis

Which patients receive periinterventional antibiotic prophylaxis? (multiple answers possible) [all]
N/A

Which patients receive periinterventional antibiotic prophylaxis? (multiple answers possible) [patients post urinary tract infection/pyelonephritis/urosepsis]
N/A

Which patients receive periinterventional antibiotic prophylaxis? (multiple answers possible) [patients with urinary tract dilation in ultrasound]
N/A

Which patients receive periinterventional antibiotic prophylaxis? (multiple answers possible) [patients with prenatal urinary tract dilation]
N/A

Which patients receive periinterventional antibiotic prophylaxis? (multiple answers possible) [patients post VUR surgery]
N/A

Which patients receive periinterventional antibiotic prophylaxis? (multiple answers possible) [none]
N/A

Are there situations or clinical findings in the patient where you deviate from your prophylaxis standard (medication, dose, duration, age of the patient)? (multiple answers possible, please specify in answer box) [in case of already known vesicoureteral reflux (VUR)]
N/A

Are there situations or clinical findings in the patient where you deviate from your prophylaxis standard (medication, dose, duration, age of the patient)? (multiple answers possible, please specify in answer box) [in patients with recurrent urinary tract infections (UTI)]
N/A

Are there situations or clinical findings in the patient where you deviate from your prophylaxis standard (medication, dose, duration, age of the patient)? (multiple answers possible, please specify in answer box) [if multidrug-resistant organisms (MDRO) are detected in urine cultures]
N/A

Are there situations or clinical findings in the patient where you deviate from your prophylaxis standard (medication, dose, duration, age of the patient)? (multiple answers possible, please specify in answer box) [in newborns]
N/A

Are there situations in which you use intravenous prophylaxis?
N/A

Voiding analysis

Are there differences in antibiotic prophylaxis between VUS and VCUG in your department?
N/A

Is a non-pathological urine strip test required in preparation for VUS/VCUG in your department?
N/A

Is a non-pathological urine culture required in preparation for VUS/VCUG in your department?
N/A

Do patients only receive post-procedure prophylaxis if vesicoureteral reflux (VUR) is detected in VUS/VCUG?
N/A

Do other MUS/MCU findings (e.g. urethral valves) have an influence on post-procedure prophylaxis?
N/A

End of survey

May we contact you personally if we have further questions?
N/A

Survey response 76

Response ID
78

Last page
1

Start language
de

Seed
1603832004

Date started
2024-12-30 16:09:01

Date last action
2024-12-30 16:12:08

Standard Operation Procedure

Who fills out this survey?
Pediatric Radiologist

Who fills out this survey? [Other]

In which type of hospital are you employed?
University Hospital

In which type of hospital are you employed? [Other]

In which country do you work?
Österreich

Does your department have a defined protocol for infection prophylaxis in the context of VUS/VCUG?
Yes

Has this protocol been transformed into a written standard operating procedure (SOP)?
Yes

Which department is the author of the SOP for infection prophylaxis in VUS/VCUG?
Other

Which department is the author of the SOP for infection prophylaxis in VUS/VCUG? [Other]
Vorhanden, aber Verfasser müsste ich nachschauen

Antibiotic Prophylaxis

Are there situations/constellations in which you do not use antibiotic prophylaxis for VUS/VCUG?
No

Please specify these situations/constellations: [girl with first uncomplicated UTI without urinary tract dilation in ultrasound]
N/A

Please specify these situations/constellations: [boy with first uncomplicated UTI without urinary tract dilation in ultrasound]
N/A

Please specify these situations/constellations: [in patients with low grade urinary tract dilation (up to 2nd grade) and no UTI in medical history]
N/A

If you use antibiotic prophylaxis: which mediaction do you use? (multiple answers and subdivision into age groups possible) [Trimethoprim]
No

If you use antibiotic prophylaxis: which mediaction do you use? (multiple answers and subdivision into age groups possible) [Nitrofurantoin]
No

If you use antibiotic prophylaxis: which mediaction do you use? (multiple answers and subdivision into age groups possible) [Amoxicillin]
No

If you use antibiotic prophylaxis: which mediaction do you use? (multiple answers and subdivision into age groups possible) [Cefaclor]
Yes

If you use antibiotic prophylaxis: which mediaction do you use? (multiple answers and subdivision into age groups possible) [Amoxicillin/clavulanic acid]
No

If you use antibiotic prophylaxis: which mediaction do you use? (multiple answers and subdivision into age groups possible) [Other]
Yes

Trimethoprim: which time schedule do you use? [Day(s) before VUS/VCUG, how many?]
N/A

Trimethoprim: which time schedule do you use? [Day of VUS/VCUG]
N/A

Trimethoprim: which time schedule do you use? [Day(s) after VUS/VCUG, how many?]
N/A

Nitrofurantoin: which time schedule do you use? [Day(s) before VUS/VCUG, how many?]
N/A

Nitrofurantoin: which time schedule do you use? [Day of VUS/VCUG]
N/A

Nitrofurantoin: which time schedule do you use? [Day(s) after VUS/VCUG, how many?]
N/A

Amoxicillin: which time schedule do you use? [Day(s) before VUS/VCUG, how many?]
N/A

Amoxicillin: which time schedule do you use? [Day of VUS/VCUG]
N/A

Amoxicillin: which time schedule do you use? [Day(s) after VUS/VCUG, how many?]
N/A

Cefaclor: in what dosage?

Cefaclor: in what dosage? [Other]

Cefaclor: for which age group do you use cefaclor?

Cefaclor: which time schedule do you use? [Days(s) before VUS/VCUG, how many?]
No

Cefaclor: which time schedule do you use? [Days(s) before VUS/VCUG, how many?][Comment]

Cefaclor: which time schedule do you use? [Day of VUS/VCUG]
No

Cefaclor: which time schedule do you use? [Day of VUS/VCUG][Comment]

Cefaclor: which time schedule do you use? [Day(s) after VUS/VCUG, how many?]
No

Cefaclor: which time schedule do you use? [Day(s) after VUS/VCUG, how many?][Comment]

Amoxicillin/clavulanic acid: in what dosage? (Amoxicillin portion) [prophylaktic (10-20 mg/Kg bodyweight/d)]
N/A

Amoxicillin/clavulanic acid: in what dosage? (Amoxicillin portion) [double prophylactic (20-40 mg/Kg bodyweight/d)]
N/A

Amoxicillin/clavulanic acid: in what dosage? (Amoxicillin portion) [therapeutic (80-100 mg/Kg bodyweight/d)]
N/A

Amoxicillin/clavulanic acid: which time schedule do you use? [Day(s) before VUS/VCUG]
N/A

Amoxicillin/clavulanic acid: which time schedule do you use? [Day of VUS/VCUG]
N/A

Amoxicillin/clavulanic acid: which time schedule do you use? [Day(s) after VUS/VCUG]
N/A

Other: which medication and in what dosage?

Other: for which age group do you use this medication?

Other: which time schedule do you use? [Day(s) before VUS/VCUG, how many?]
No

Other: which time schedule do you use? [Day(s) before VUS/VCUG, how many?][Comment]

Other: which time schedule do you use? [Day of VUS/VCUG]
No

Other: which time schedule do you use? [Day of VUS/VCUG][Comment]

Other: which time schedule do you use? [Day(s) after VUS/VCUG, how many?]
No

Other: which time schedule do you use? [Day(s) after VUS/VCUG, how many?][Comment]

Indication for prophylaxis

Which patients receive periinterventional antibiotic prophylaxis? (multiple answers possible) [all]
N/A

Which patients receive periinterventional antibiotic prophylaxis? (multiple answers possible) [patients post urinary tract infection/pyelonephritis/urosepsis]
N/A

Which patients receive periinterventional antibiotic prophylaxis? (multiple answers possible) [patients with urinary tract dilation in ultrasound]
N/A

Which patients receive periinterventional antibiotic prophylaxis? (multiple answers possible) [patients with prenatal urinary tract dilation]
N/A

Which patients receive periinterventional antibiotic prophylaxis? (multiple answers possible) [patients post VUR surgery]
N/A

Which patients receive periinterventional antibiotic prophylaxis? (multiple answers possible) [none]
N/A

Are there situations or clinical findings in the patient where you deviate from your prophylaxis standard (medication, dose, duration, age of the patient)? (multiple answers possible, please specify in answer box) [in case of already known vesicoureteral reflux (VUR)]
N/A

Are there situations or clinical findings in the patient where you deviate from your prophylaxis standard (medication, dose, duration, age of the patient)? (multiple answers possible, please specify in answer box) [in patients with recurrent urinary tract infections (UTI)]
N/A

Are there situations or clinical findings in the patient where you deviate from your prophylaxis standard (medication, dose, duration, age of the patient)? (multiple answers possible, please specify in answer box) [if multidrug-resistant organisms (MDRO) are detected in urine cultures]
N/A

Are there situations or clinical findings in the patient where you deviate from your prophylaxis standard (medication, dose, duration, age of the patient)? (multiple answers possible, please specify in answer box) [in newborns]
N/A

Are there situations in which you use intravenous prophylaxis?
N/A

Voiding analysis

Are there differences in antibiotic prophylaxis between VUS and VCUG in your department?
N/A

Is a non-pathological urine strip test required in preparation for VUS/VCUG in your department?
N/A

Is a non-pathological urine culture required in preparation for VUS/VCUG in your department?
N/A

Do patients only receive post-procedure prophylaxis if vesicoureteral reflux (VUR) is detected in VUS/VCUG?
N/A

Do other MUS/MCU findings (e.g. urethral valves) have an influence on post-procedure prophylaxis?
N/A

End of survey

May we contact you personally if we have further questions?
N/A

Survey response 77

Response ID
79

Date submitted
2025-01-04 13:10:43

Last page
5

Start language
de

Seed
1389527653

Date started
2025-01-04 13:05:42

Date last action
2025-01-04 13:10:43

Standard Operation Procedure

Who fills out this survey?
Pediatric Surgeon

Who fills out this survey? [Other]

In which type of hospital are you employed?
Community hospital (>50 beds for pediatric patients)

In which type of hospital are you employed? [Other]

In which country do you work?
Deutschland

Does your department have a defined protocol for infection prophylaxis in the context of VUS/VCUG?
Yes

Has this protocol been transformed into a written standard operating procedure (SOP)?
Yes

Which department is the author of the SOP for infection prophylaxis in VUS/VCUG?
Pediatric nephrology

Which department is the author of the SOP for infection prophylaxis in VUS/VCUG? [Other]

Antibiotic Prophylaxis

Are there situations/constellations in which you do not use antibiotic prophylaxis for VUS/VCUG?
No

Please specify these situations/constellations: [girl with first uncomplicated UTI without urinary tract dilation in ultrasound]
N/A

Please specify these situations/constellations: [boy with first uncomplicated UTI without urinary tract dilation in ultrasound]
N/A

Please specify these situations/constellations: [in patients with low grade urinary tract dilation (up to 2nd grade) and no UTI in medical history]
N/A

If you use antibiotic prophylaxis: which mediaction do you use? (multiple answers and subdivision into age groups possible) [Trimethoprim]
No

If you use antibiotic prophylaxis: which mediaction do you use? (multiple answers and subdivision into age groups possible) [Nitrofurantoin]
Yes

If you use antibiotic prophylaxis: which mediaction do you use? (multiple answers and subdivision into age groups possible) [Amoxicillin]
No

If you use antibiotic prophylaxis: which mediaction do you use? (multiple answers and subdivision into age groups possible) [Cefaclor]
No

If you use antibiotic prophylaxis: which mediaction do you use? (multiple answers and subdivision into age groups possible) [Amoxicillin/clavulanic acid]
No

If you use antibiotic prophylaxis: which mediaction do you use? (multiple answers and subdivision into age groups possible) [Other]
No

Trimethoprim: which time schedule do you use? [Day(s) before VUS/VCUG, how many?]
N/A

Trimethoprim: which time schedule do you use? [Day of VUS/VCUG]
N/A

Trimethoprim: which time schedule do you use? [Day(s) after VUS/VCUG, how many?]
N/A

Nitrofurantoin: in what dosage?
prophylactic (1-2 mg/kg bodyweight/d)

Nitrofurantoin: in what dosage? [Other]

Nitrofurantoin: for which age group do you use nitrofurantoin?
alle

Nitrofurantoin: which time schedule do you use? [Day(s) before VUS/VCUG, how many?]
Yes

Nitrofurantoin: which time schedule do you use? [Day(s) before VUS/VCUG, how many?][Comment]
3

Nitrofurantoin: which time schedule do you use? [Day of VUS/VCUG]
No

Nitrofurantoin: which time schedule do you use? [Day of VUS/VCUG][Comment]

Nitrofurantoin: which time schedule do you use? [Day(s) after VUS/VCUG, how many?]
No

Nitrofurantoin: which time schedule do you use? [Day(s) after VUS/VCUG, how many?][Comment]

Amoxicillin: which time schedule do you use? [Day(s) before VUS/VCUG, how many?]
N/A

Amoxicillin: which time schedule do you use? [Day of VUS/VCUG]
N/A

Amoxicillin: which time schedule do you use? [Day(s) after VUS/VCUG, how many?]
N/A

Cefaclor: which time schedule do you use? [Days(s) before VUS/VCUG, how many?]
N/A

Cefaclor: which time schedule do you use? [Day of VUS/VCUG]
N/A

Cefaclor: which time schedule do you use? [Day(s) after VUS/VCUG, how many?]
N/A

Amoxicillin/clavulanic acid: in what dosage? (Amoxicillin portion) [prophylaktic (10-20 mg/Kg bodyweight/d)]
N/A

Amoxicillin/clavulanic acid: in what dosage? (Amoxicillin portion) [double prophylactic (20-40 mg/Kg bodyweight/d)]
N/A

Amoxicillin/clavulanic acid: in what dosage? (Amoxicillin portion) [therapeutic (80-100 mg/Kg bodyweight/d)]
N/A

Amoxicillin/clavulanic acid: which time schedule do you use? [Day(s) before VUS/VCUG]
N/A

Amoxicillin/clavulanic acid: which time schedule do you use? [Day of VUS/VCUG]
N/A

Amoxicillin/clavulanic acid: which time schedule do you use? [Day(s) after VUS/VCUG]
N/A

Other: which time schedule do you use? [Day(s) before VUS/VCUG, how many?]
N/A

Other: which time schedule do you use? [Day of VUS/VCUG]
N/A

Other: which time schedule do you use? [Day(s) after VUS/VCUG, how many?]
N/A

Indication for prophylaxis

Which patients receive periinterventional antibiotic prophylaxis? (multiple answers possible) [all]
Yes

Which patients receive periinterventional antibiotic prophylaxis? (multiple answers possible) [patients post urinary tract infection/pyelonephritis/urosepsis]
N/A

Which patients receive periinterventional antibiotic prophylaxis? (multiple answers possible) [patients with urinary tract dilation in ultrasound]
N/A

Which patients receive periinterventional antibiotic prophylaxis? (multiple answers possible) [patients with prenatal urinary tract dilation]
N/A

Which patients receive periinterventional antibiotic prophylaxis? (multiple answers possible) [patients post VUR surgery]
N/A

Which patients receive periinterventional antibiotic prophylaxis? (multiple answers possible) [none]
N/A

Are there situations or clinical findings in the patient where you deviate from your prophylaxis standard (medication, dose, duration, age of the patient)? (multiple answers possible, please specify in answer box) [in case of already known vesicoureteral reflux (VUR)]
No

Are there situations or clinical findings in the patient where you deviate from your prophylaxis standard (medication, dose, duration, age of the patient)? (multiple answers possible, please specify in answer box) [in case of already known vesicoureteral reflux (VUR)][Comment]

Are there situations or clinical findings in the patient where you deviate from your prophylaxis standard (medication, dose, duration, age of the patient)? (multiple answers possible, please specify in answer box) [in patients with recurrent urinary tract infections (UTI)]
No

Are there situations or clinical findings in the patient where you deviate from your prophylaxis standard (medication, dose, duration, age of the patient)? (multiple answers possible, please specify in answer box) [in patients with recurrent urinary tract infections (UTI)][Comment]

Are there situations or clinical findings in the patient where you deviate from your prophylaxis standard (medication, dose, duration, age of the patient)? (multiple answers possible, please specify in answer box) [if multidrug-resistant organisms (MDRO) are detected in urine cultures]
Yes

Are there situations or clinical findings in the patient where you deviate from your prophylaxis standard (medication, dose, duration, age of the patient)? (multiple answers possible, please specify in answer box) [if multidrug-resistant organisms (MDRO) are detected in urine cultures][Comment]
nach Resistogramm

Are there situations or clinical findings in the patient where you deviate from your prophylaxis standard (medication, dose, duration, age of the patient)? (multiple answers possible, please specify in answer box) [in newborns]
No

Are there situations or clinical findings in the patient where you deviate from your prophylaxis standard (medication, dose, duration, age of the patient)? (multiple answers possible, please specify in answer box) [in newborns][Comment]

Are there situations or clinical findings in the patient where you deviate from your prophylaxis standard (medication, dose, duration, age of the patient)? (multiple answers possible, please specify in answer box) [Other]

Are there situations or clinical findings in the patient where you deviate from your prophylaxis standard (medication, dose, duration, age of the patient)? (multiple answers possible, please specify in answer box) [Other comment]

Are there situations in which you use intravenous prophylaxis?
No

Voiding analysis

Are there differences in antibiotic prophylaxis between VUS and VCUG in your department?
No

Is a non-pathological urine strip test required in preparation for VUS/VCUG in your department?
Yes

What is the maximum amount of days accepted for urine strip test to be performed prior to the exam?
3

Is a non-pathological urine culture required in preparation for VUS/VCUG in your department?
No

Do patients only receive post-procedure prophylaxis if vesicoureteral reflux (VUR) is detected in VUS/VCUG?
No

Do other MUS/MCU findings (e.g. urethral valves) have an influence on post-procedure prophylaxis?
Yes

Please specify:
3 Tage therapeutische Dosierung, dann prophylaktische Dosierung weiter.

End of survey

Thank you for taking the time to answer this survey!

May we contact you personally if we have further questions?
No

Survey response 78

Response ID
80

Date submitted
2025-01-07 13:21:17

Last page
5

Start language
de

Seed
1533652948

Date started
2025-01-07 13:15:29

Date last action
2025-01-07 13:21:17

Standard Operation Procedure

Who fills out this survey?
Pediatric Surgeon

Who fills out this survey? [Other]

In which type of hospital are you employed?
University Hospital

In which type of hospital are you employed? [Other]

In which country do you work?
Deutschland

Does your department have a defined protocol for infection prophylaxis in the context of VUS/VCUG?
No

Has this protocol been transformed into a written standard operating procedure (SOP)?
N/A

Antibiotic Prophylaxis

Are there situations/constellations in which you do not use antibiotic prophylaxis for VUS/VCUG?
No

Please specify these situations/constellations: [girl with first uncomplicated UTI without urinary tract dilation in ultrasound]
N/A

Please specify these situations/constellations: [boy with first uncomplicated UTI without urinary tract dilation in ultrasound]
N/A

Please specify these situations/constellations: [in patients with low grade urinary tract dilation (up to 2nd grade) and no UTI in medical history]
N/A

If you use antibiotic prophylaxis: which mediaction do you use? (multiple answers and subdivision into age groups possible) [Trimethoprim]
Yes

If you use antibiotic prophylaxis: which mediaction do you use? (multiple answers and subdivision into age groups possible) [Nitrofurantoin]
Yes

If you use antibiotic prophylaxis: which mediaction do you use? (multiple answers and subdivision into age groups possible) [Amoxicillin]
No

If you use antibiotic prophylaxis: which mediaction do you use? (multiple answers and subdivision into age groups possible) [Cefaclor]
Yes

If you use antibiotic prophylaxis: which mediaction do you use? (multiple answers and subdivision into age groups possible) [Amoxicillin/clavulanic acid]
No

If you use antibiotic prophylaxis: which mediaction do you use? (multiple answers and subdivision into age groups possible) [Other]
No

Trimethoprim: in what dosage?
double prophylakctic (4 mg/kg bodyweight/d)

Trimethoprim: in what dosage? [Other]

Trimethoprim: for which age group do you use trimethoprim?
ab 1. LJ

Trimethoprim: which time schedule do you use? [Day(s) before VUS/VCUG, how many?]
No

Trimethoprim: which time schedule do you use? [Day(s) before VUS/VCUG, how many?][Comment]

Trimethoprim: which time schedule do you use? [Day of VUS/VCUG]
Yes

Trimethoprim: which time schedule do you use? [Day of VUS/VCUG][Comment]

Trimethoprim: which time schedule do you use? [Day(s) after VUS/VCUG, how many?]
Yes

Trimethoprim: which time schedule do you use? [Day(s) after VUS/VCUG, how many?][Comment]
2 weitere Tage falls vorherige Infekte/VUR bekannt

Nitrofurantoin: in what dosage?
therapeutic (5 mg/kg bodyweight/d)

Nitrofurantoin: in what dosage? [Other]

Nitrofurantoin: for which age group do you use nitrofurantoin?
ab. 2. LJ

Nitrofurantoin: which time schedule do you use? [Day(s) before VUS/VCUG, how many?]
No

Nitrofurantoin: which time schedule do you use? [Day(s) before VUS/VCUG, how many?][Comment]

Nitrofurantoin: which time schedule do you use? [Day of VUS/VCUG]
Yes

Nitrofurantoin: which time schedule do you use? [Day of VUS/VCUG][Comment]

Nitrofurantoin: which time schedule do you use? [Day(s) after VUS/VCUG, how many?]
Yes

Nitrofurantoin: which time schedule do you use? [Day(s) after VUS/VCUG, how many?][Comment]
für 2 weitere Tage falls VUR bekannt/Infekte im Vorfeld bekannt bei kompl. Harnwegen

Amoxicillin: which time schedule do you use? [Day(s) before VUS/VCUG, how many?]
N/A

Amoxicillin: which time schedule do you use? [Day of VUS/VCUG]
N/A

Amoxicillin: which time schedule do you use? [Day(s) after VUS/VCUG, how many?]
N/A

Cefaclor: in what dosage?
therapeutic (40 mg/kg bodyweight/d)

Cefaclor: in what dosage? [Other]

Cefaclor: for which age group do you use cefaclor?
im 1. LJ

Cefaclor: which time schedule do you use? [Days(s) before VUS/VCUG, how many?]
No

Cefaclor: which time schedule do you use? [Days(s) before VUS/VCUG, how many?][Comment]

Cefaclor: which time schedule do you use? [Day of VUS/VCUG]
Yes

Cefaclor: which time schedule do you use? [Day of VUS/VCUG][Comment]

Cefaclor: which time schedule do you use? [Day(s) after VUS/VCUG, how many?]
Yes

Cefaclor: which time schedule do you use? [Day(s) after VUS/VCUG, how many?][Comment]
falls Bakteriurie im U-Status, VUR bekannt, 2-5 Tage,

Amoxicillin/clavulanic acid: in what dosage? (Amoxicillin portion) [prophylaktic (10-20 mg/Kg bodyweight/d)]
N/A

Amoxicillin/clavulanic acid: in what dosage? (Amoxicillin portion) [double prophylactic (20-40 mg/Kg bodyweight/d)]
N/A

Amoxicillin/clavulanic acid: in what dosage? (Amoxicillin portion) [therapeutic (80-100 mg/Kg bodyweight/d)]
N/A

Amoxicillin/clavulanic acid: which time schedule do you use? [Day(s) before VUS/VCUG]
N/A

Amoxicillin/clavulanic acid: which time schedule do you use? [Day of VUS/VCUG]
N/A

Amoxicillin/clavulanic acid: which time schedule do you use? [Day(s) after VUS/VCUG]
N/A

Other: which time schedule do you use? [Day(s) before VUS/VCUG, how many?]
N/A

Other: which time schedule do you use? [Day of VUS/VCUG]
N/A

Other: which time schedule do you use? [Day(s) after VUS/VCUG, how many?]
N/A

Indication for prophylaxis

Which patients receive periinterventional antibiotic prophylaxis? (multiple answers possible) [all]
No

Which patients receive periinterventional antibiotic prophylaxis? (multiple answers possible) [patients post urinary tract infection/pyelonephritis/urosepsis]
Yes

Which patients receive periinterventional antibiotic prophylaxis? (multiple answers possible) [patients with urinary tract dilation in ultrasound]
Yes

Which patients receive periinterventional antibiotic prophylaxis? (multiple answers possible) [patients with prenatal urinary tract dilation]
Yes

Which patients receive periinterventional antibiotic prophylaxis? (multiple answers possible) [patients post VUR surgery]
Yes

Which patients receive periinterventional antibiotic prophylaxis? (multiple answers possible) [none]
No

Which patients receive periinterventional antibiotic prophylaxis? (multiple answers possible) [Other]

patients status post urinary tract infection/pyelonephritis/urosepsis: what is the minimum time between infection and VUS/VCUG? (in days)
14

In patients with urinary tract dilation in ultrasound: from what degree on? Image from Beetz R, Bokenkamp A, Brandis M et al (2001) Diagnosis of congenital dilatation of the urinary tract. Consensus Group of the Pediatric Nephrology Working Society in cooperation with the Pediatric Urology Working Group of the German Society of Urology and with the Pediatric Urology Working Society in the Germany Society of Pediatric Surgery. Urologe A 40:495–507 (quiz 8–9)
grade 3

In patients with prenatal urinary tract dilation: from what degree on? Image from Beetz R, Bokenkamp A, Brandis M et al (2001) Diagnosis of congenital dilatation of the urinary tract. Consensus Group of the Pediatric Nephrology Working Society in cooperation with the Pediatric Urology Working Group of the German Society of Urology and with the Pediatric Urology Working Society in the Germany Society of Pediatric Surgery. Urologe A 40:495–507 (quiz 8–9)
grade 3

Are there situations or clinical findings in the patient where you deviate from your prophylaxis standard (medication, dose, duration, age of the patient)? (multiple answers possible, please specify in answer box) [in case of already known vesicoureteral reflux (VUR)]
No

Are there situations or clinical findings in the patient where you deviate from your prophylaxis standard (medication, dose, duration, age of the patient)? (multiple answers possible, please specify in answer box) [in case of already known vesicoureteral reflux (VUR)][Comment]

Are there situations or clinical findings in the patient where you deviate from your prophylaxis standard (medication, dose, duration, age of the patient)? (multiple answers possible, please specify in answer box) [in patients with recurrent urinary tract infections (UTI)]
Yes

Are there situations or clinical findings in the patient where you deviate from your prophylaxis standard (medication, dose, duration, age of the patient)? (multiple answers possible, please specify in answer box) [in patients with recurrent urinary tract infections (UTI)][Comment]
nach Resistogramm der Vorinfekte

Are there situations or clinical findings in the patient where you deviate from your prophylaxis standard (medication, dose, duration, age of the patient)? (multiple answers possible, please specify in answer box) [if multidrug-resistant organisms (MDRO) are detected in urine cultures]
Yes

Are there situations or clinical findings in the patient where you deviate from your prophylaxis standard (medication, dose, duration, age of the patient)? (multiple answers possible, please specify in answer box) [if multidrug-resistant organisms (MDRO) are detected in urine cultures][Comment]
nach Resistogramm

Are there situations or clinical findings in the patient where you deviate from your prophylaxis standard (medication, dose, duration, age of the patient)? (multiple answers possible, please specify in answer box) [in newborns]
Yes

Are there situations or clinical findings in the patient where you deviate from your prophylaxis standard (medication, dose, duration, age of the patient)? (multiple answers possible, please specify in answer box) [in newborns][Comment]
wenn VUR bekannt wird

Are there situations or clinical findings in the patient where you deviate from your prophylaxis standard (medication, dose, duration, age of the patient)? (multiple answers possible, please specify in answer box) [Other]

Are there situations or clinical findings in the patient where you deviate from your prophylaxis standard (medication, dose, duration, age of the patient)? (multiple answers possible, please specify in answer box) [Other comment]

Are there situations in which you use intravenous prophylaxis?
Yes

In which situations?
bei Untersuchungen "aus Infekt heraus" bzw. im Anschluss an eine Zystoskopie am Vortag (und MUS am Folgetag) bei suprapubischer Kathetereinlage

Voiding analysis

Are there differences in antibiotic prophylaxis between VUS and VCUG in your department?
No

Is a non-pathological urine strip test required in preparation for VUS/VCUG in your department?
No

Is a non-pathological urine culture required in preparation for VUS/VCUG in your department?
No

Do patients only receive post-procedure prophylaxis if vesicoureteral reflux (VUR) is detected in VUS/VCUG?
Yes

Please explain your approach:
wenn kein VUR/keine Infekte, kein Restharn/Blasendysfunktion und wenn Eltern auch keine Antibiose wünschen (manche wollen lieber Prophylaxe)

Do other MUS/MCU findings (e.g. urethral valves) have an influence on post-procedure prophylaxis?
Yes

Please specify:
bei Auffälligkeiten im MUS (Klappen, Restharn etc.) eher Entscheidung für Prophylaxe

End of survey

Thank you for taking the time to answer this survey!

May we contact you personally if we have further questions?
Yes

E-mail address:
friederike.heidtmann@med.uni-jena.de

Survey response 79

Response ID
81

Last page
2

Start language
de

Seed
587208056

Date started
2025-01-15 08:32:54

Date last action
2025-01-15 08:44:26

Standard Operation Procedure

Who fills out this survey?
Pediatric Radiologist

Who fills out this survey? [Other]

In which type of hospital are you employed?
Community hospital (>50 beds for pediatric patients)

In which type of hospital are you employed? [Other]

In which country do you work?
Deutschland

Does your department have a defined protocol for infection prophylaxis in the context of VUS/VCUG?
Yes

Has this protocol been transformed into a written standard operating procedure (SOP)?
Yes

Which department is the author of the SOP for infection prophylaxis in VUS/VCUG?
Pediatrics

Which department is the author of the SOP for infection prophylaxis in VUS/VCUG? [Other]

Antibiotic Prophylaxis

Are there situations/constellations in which you do not use antibiotic prophylaxis for VUS/VCUG?
No

Please specify these situations/constellations: [girl with first uncomplicated UTI without urinary tract dilation in ultrasound]
N/A

Please specify these situations/constellations: [boy with first uncomplicated UTI without urinary tract dilation in ultrasound]
N/A

Please specify these situations/constellations: [in patients with low grade urinary tract dilation (up to 2nd grade) and no UTI in medical history]
N/A

If you use antibiotic prophylaxis: which mediaction do you use? (multiple answers and subdivision into age groups possible) [Trimethoprim]
Yes

If you use antibiotic prophylaxis: which mediaction do you use? (multiple answers and subdivision into age groups possible) [Nitrofurantoin]
Yes

If you use antibiotic prophylaxis: which mediaction do you use? (multiple answers and subdivision into age groups possible) [Amoxicillin]
No

If you use antibiotic prophylaxis: which mediaction do you use? (multiple answers and subdivision into age groups possible) [Cefaclor]
Yes

If you use antibiotic prophylaxis: which mediaction do you use? (multiple answers and subdivision into age groups possible) [Amoxicillin/clavulanic acid]
No

If you use antibiotic prophylaxis: which mediaction do you use? (multiple answers and subdivision into age groups possible) [Other]
No

Trimethoprim: in what dosage?
prophylactic (2 mg/kg bodyweight/d)

Trimethoprim: in what dosage? [Other]

Trimethoprim: for which age group do you use trimethoprim?
ab 6. Lebenswoche

Trimethoprim: which time schedule do you use? [Day(s) before VUS/VCUG, how many?]
Yes

Trimethoprim: which time schedule do you use? [Day(s) before VUS/VCUG, how many?][Comment]
Im Anschluß an die therapeutische Dosis nach Pyelonephritis bis einschließlich zum 3. Tag nach MCU/MUS

Trimethoprim: which time schedule do you use? [Day of VUS/VCUG]
Yes

Trimethoprim: which time schedule do you use? [Day of VUS/VCUG][Comment]

Trimethoprim: which time schedule do you use? [Day(s) after VUS/VCUG, how many?]
Yes

Trimethoprim: which time schedule do you use? [Day(s) after VUS/VCUG, how many?][Comment]
Tag des MCU + Tag 1 und Tag 2 nach MCU

Nitrofurantoin: in what dosage?
prophylactic (1-2 mg/kg bodyweight/d)

Nitrofurantoin: in what dosage? [Other]

Nitrofurantoin: for which age group do you use nitrofurantoin?
> 3. Lebensmonat

Nitrofurantoin: which time schedule do you use? [Day(s) before VUS/VCUG, how many?]
Yes

Nitrofurantoin: which time schedule do you use? [Day(s) before VUS/VCUG, how many?][Comment]
Im Anschluß an die therapeutische Dosis nach Pyelonephritis bis einschließlich zum 3. Tag nach MCU/MUS

Nitrofurantoin: which time schedule do you use? [Day of VUS/VCUG]
Yes

Nitrofurantoin: which time schedule do you use? [Day of VUS/VCUG][Comment]

Nitrofurantoin: which time schedule do you use? [Day(s) after VUS/VCUG, how many?]
Yes

Nitrofurantoin: which time schedule do you use? [Day(s) after VUS/VCUG, how many?][Comment]
Tag des MCU + Tag 1 und Tag 2 nach MCU

Amoxicillin: which time schedule do you use? [Day(s) before VUS/VCUG, how many?]
N/A

Amoxicillin: which time schedule do you use? [Day of VUS/VCUG]
N/A

Amoxicillin: which time schedule do you use? [Day(s) after VUS/VCUG, how many?]
N/A

Cefaclor: in what dosage?
prophylactic (10 mg/kg bodyweight/d)

Cefaclor: in what dosage? [Other]

Cefaclor: for which age group do you use cefaclor?
< 6 Lebenswochen

Cefaclor: which time schedule do you use? [Days(s) before VUS/VCUG, how many?]
Yes

Cefaclor: which time schedule do you use? [Days(s) before VUS/VCUG, how many?][Comment]
Im Anschluß an die therapeutische Dosis nach Pyelonephritis bis einschließlich zum 3. Tag nach MCU/MUS

Cefaclor: which time schedule do you use? [Day of VUS/VCUG]
Yes

Cefaclor: which time schedule do you use? [Day of VUS/VCUG][Comment]

Cefaclor: which time schedule do you use? [Day(s) after VUS/VCUG, how many?]
Yes

Cefaclor: which time schedule do you use? [Day(s) after VUS/VCUG, how many?][Comment]
Tag des MCU + Tag 1 und Tag 2 nach MCU

Amoxicillin/clavulanic acid: in what dosage? (Amoxicillin portion) [prophylaktic (10-20 mg/Kg bodyweight/d)]
N/A

Amoxicillin/clavulanic acid: in what dosage? (Amoxicillin portion) [double prophylactic (20-40 mg/Kg bodyweight/d)]
N/A

Amoxicillin/clavulanic acid: in what dosage? (Amoxicillin portion) [therapeutic (80-100 mg/Kg bodyweight/d)]
N/A

Amoxicillin/clavulanic acid: which time schedule do you use? [Day(s) before VUS/VCUG]
N/A

Amoxicillin/clavulanic acid: which time schedule do you use? [Day of VUS/VCUG]
N/A

Amoxicillin/clavulanic acid: which time schedule do you use? [Day(s) after VUS/VCUG]
N/A

Other: which time schedule do you use? [Day(s) before VUS/VCUG, how many?]
N/A

Other: which time schedule do you use? [Day of VUS/VCUG]
N/A

Other: which time schedule do you use? [Day(s) after VUS/VCUG, how many?]
N/A

Indication for prophylaxis

Which patients receive periinterventional antibiotic prophylaxis? (multiple answers possible) [all]
N/A

Which patients receive periinterventional antibiotic prophylaxis? (multiple answers possible) [patients post urinary tract infection/pyelonephritis/urosepsis]
N/A

Which patients receive periinterventional antibiotic prophylaxis? (multiple answers possible) [patients with urinary tract dilation in ultrasound]
N/A

Which patients receive periinterventional antibiotic prophylaxis? (multiple answers possible) [patients with prenatal urinary tract dilation]
N/A

Which patients receive periinterventional antibiotic prophylaxis? (multiple answers possible) [patients post VUR surgery]
N/A

Which patients receive periinterventional antibiotic prophylaxis? (multiple answers possible) [none]
N/A

Are there situations or clinical findings in the patient where you deviate from your prophylaxis standard (medication, dose, duration, age of the patient)? (multiple answers possible, please specify in answer box) [in case of already known vesicoureteral reflux (VUR)]
N/A

Are there situations or clinical findings in the patient where you deviate from your prophylaxis standard (medication, dose, duration, age of the patient)? (multiple answers possible, please specify in answer box) [in patients with recurrent urinary tract infections (UTI)]
N/A

Are there situations or clinical findings in the patient where you deviate from your prophylaxis standard (medication, dose, duration, age of the patient)? (multiple answers possible, please specify in answer box) [if multidrug-resistant organisms (MDRO) are detected in urine cultures]
N/A

Are there situations or clinical findings in the patient where you deviate from your prophylaxis standard (medication, dose, duration, age of the patient)? (multiple answers possible, please specify in answer box) [in newborns]
N/A

Are there situations in which you use intravenous prophylaxis?
N/A

Voiding analysis

Are there differences in antibiotic prophylaxis between VUS and VCUG in your department?
N/A

Is a non-pathological urine strip test required in preparation for VUS/VCUG in your department?
N/A

Is a non-pathological urine culture required in preparation for VUS/VCUG in your department?
N/A

Do patients only receive post-procedure prophylaxis if vesicoureteral reflux (VUR) is detected in VUS/VCUG?
N/A

Do other MUS/MCU findings (e.g. urethral valves) have an influence on post-procedure prophylaxis?
N/A

End of survey

May we contact you personally if we have further questions?
N/A

Survey response 80

Response ID
82

Date submitted
2025-01-15 09:51:18

Last page
5

Start language
de

Seed
1390456618

Date started
2025-01-15 09:35:26

Date last action
2025-01-15 09:51:18

Standard Operation Procedure

Who fills out this survey?
Pediatric Radiologist

Who fills out this survey? [Other]

In which type of hospital are you employed?
Community hospital (>50 beds for pediatric patients)

In which type of hospital are you employed? [Other]

In which country do you work?
Deutschland

Does your department have a defined protocol for infection prophylaxis in the context of VUS/VCUG?
Yes

Has this protocol been transformed into a written standard operating procedure (SOP)?
Yes

Which department is the author of the SOP for infection prophylaxis in VUS/VCUG?
Pediatrics

Which department is the author of the SOP for infection prophylaxis in VUS/VCUG? [Other]

Antibiotic Prophylaxis

Are there situations/constellations in which you do not use antibiotic prophylaxis for VUS/VCUG?
No

Please specify these situations/constellations: [girl with first uncomplicated UTI without urinary tract dilation in ultrasound]
N/A

Please specify these situations/constellations: [boy with first uncomplicated UTI without urinary tract dilation in ultrasound]
N/A

Please specify these situations/constellations: [in patients with low grade urinary tract dilation (up to 2nd grade) and no UTI in medical history]
N/A

If you use antibiotic prophylaxis: which mediaction do you use? (multiple answers and subdivision into age groups possible) [Trimethoprim]
Yes

If you use antibiotic prophylaxis: which mediaction do you use? (multiple answers and subdivision into age groups possible) [Nitrofurantoin]
Yes

If you use antibiotic prophylaxis: which mediaction do you use? (multiple answers and subdivision into age groups possible) [Amoxicillin]
No

If you use antibiotic prophylaxis: which mediaction do you use? (multiple answers and subdivision into age groups possible) [Cefaclor]
Yes

If you use antibiotic prophylaxis: which mediaction do you use? (multiple answers and subdivision into age groups possible) [Amoxicillin/clavulanic acid]
No

If you use antibiotic prophylaxis: which mediaction do you use? (multiple answers and subdivision into age groups possible) [Other]
No

Trimethoprim: in what dosage?
double prophylakctic (4 mg/kg bodyweight/d)

Trimethoprim: in what dosage? [Other]

Trimethoprim: for which age group do you use trimethoprim?
ab 6. Lebenswoche

Trimethoprim: which time schedule do you use? [Day(s) before VUS/VCUG, how many?]
Yes

Trimethoprim: which time schedule do you use? [Day(s) before VUS/VCUG, how many?][Comment]
Einfach prophylaktisch ab Therapie-Ende bis zur Durchführung des MCU/MUS

Trimethoprim: which time schedule do you use? [Day of VUS/VCUG]
Yes

Trimethoprim: which time schedule do you use? [Day of VUS/VCUG][Comment]

Trimethoprim: which time schedule do you use? [Day(s) after VUS/VCUG, how many?]
Yes

Trimethoprim: which time schedule do you use? [Day(s) after VUS/VCUG, how many?][Comment]
Am Tag des MCU/MUS und den 2 Folgetagen wie oben angegeben doppelt prophylaktisch

Nitrofurantoin: in what dosage?
double prophylactic (2-4 mg/kg bodyweight/d)

Nitrofurantoin: in what dosage? [Other]

Nitrofurantoin: for which age group do you use nitrofurantoin?
> 3. Lebensmonat

Nitrofurantoin: which time schedule do you use? [Day(s) before VUS/VCUG, how many?]
Yes

Nitrofurantoin: which time schedule do you use? [Day(s) before VUS/VCUG, how many?][Comment]
Einfach prophylaktisch ab Therapie-Ende bis zur Durchführung des MCU/MUS

Nitrofurantoin: which time schedule do you use? [Day of VUS/VCUG]
Yes

Nitrofurantoin: which time schedule do you use? [Day of VUS/VCUG][Comment]

Nitrofurantoin: which time schedule do you use? [Day(s) after VUS/VCUG, how many?]
Yes

Nitrofurantoin: which time schedule do you use? [Day(s) after VUS/VCUG, how many?][Comment]
Am Tag des MCU/MUS und den 2 Folgetagen wie oben angegeben doppelt prophylaktisch

Amoxicillin: which time schedule do you use? [Day(s) before VUS/VCUG, how many?]
N/A

Amoxicillin: which time schedule do you use? [Day of VUS/VCUG]
N/A

Amoxicillin: which time schedule do you use? [Day(s) after VUS/VCUG, how many?]
N/A

Cefaclor: in what dosage?
double prophylactic (20 mg/kg bodyweight/d)

Cefaclor: in what dosage? [Other]

Cefaclor: for which age group do you use cefaclor?
< 6 Lebenswochen

Cefaclor: which time schedule do you use? [Days(s) before VUS/VCUG, how many?]
Yes

Cefaclor: which time schedule do you use? [Days(s) before VUS/VCUG, how many?][Comment]
Einfach prophylaktisch ab Therapie-Ende bis zur Durchführung des MCU/MUS

Cefaclor: which time schedule do you use? [Day of VUS/VCUG]
Yes

Cefaclor: which time schedule do you use? [Day of VUS/VCUG][Comment]

Cefaclor: which time schedule do you use? [Day(s) after VUS/VCUG, how many?]
Yes

Cefaclor: which time schedule do you use? [Day(s) after VUS/VCUG, how many?][Comment]
Am Tag des MCU/MUS und den 2 Folgetagen wie oben angegeben doppelt prophylaktisch

Amoxicillin/clavulanic acid: in what dosage? (Amoxicillin portion) [prophylaktic (10-20 mg/Kg bodyweight/d)]
N/A

Amoxicillin/clavulanic acid: in what dosage? (Amoxicillin portion) [double prophylactic (20-40 mg/Kg bodyweight/d)]
N/A

Amoxicillin/clavulanic acid: in what dosage? (Amoxicillin portion) [therapeutic (80-100 mg/Kg bodyweight/d)]
N/A

Amoxicillin/clavulanic acid: which time schedule do you use? [Day(s) before VUS/VCUG]
N/A

Amoxicillin/clavulanic acid: which time schedule do you use? [Day of VUS/VCUG]
N/A

Amoxicillin/clavulanic acid: which time schedule do you use? [Day(s) after VUS/VCUG]
N/A

Other: which time schedule do you use? [Day(s) before VUS/VCUG, how many?]
N/A

Other: which time schedule do you use? [Day of VUS/VCUG]
N/A

Other: which time schedule do you use? [Day(s) after VUS/VCUG, how many?]
N/A

Indication for prophylaxis

Which patients receive periinterventional antibiotic prophylaxis? (multiple answers possible) [all]
Yes

Which patients receive periinterventional antibiotic prophylaxis? (multiple answers possible) [patients post urinary tract infection/pyelonephritis/urosepsis]
N/A

Which patients receive periinterventional antibiotic prophylaxis? (multiple answers possible) [patients with urinary tract dilation in ultrasound]
N/A

Which patients receive periinterventional antibiotic prophylaxis? (multiple answers possible) [patients with prenatal urinary tract dilation]
N/A

Which patients receive periinterventional antibiotic prophylaxis? (multiple answers possible) [patients post VUR surgery]
N/A

Which patients receive periinterventional antibiotic prophylaxis? (multiple answers possible) [none]
N/A

Are there situations or clinical findings in the patient where you deviate from your prophylaxis standard (medication, dose, duration, age of the patient)? (multiple answers possible, please specify in answer box) [in case of already known vesicoureteral reflux (VUR)]
No

Are there situations or clinical findings in the patient where you deviate from your prophylaxis standard (medication, dose, duration, age of the patient)? (multiple answers possible, please specify in answer box) [in case of already known vesicoureteral reflux (VUR)][Comment]

Are there situations or clinical findings in the patient where you deviate from your prophylaxis standard (medication, dose, duration, age of the patient)? (multiple answers possible, please specify in answer box) [in patients with recurrent urinary tract infections (UTI)]
No

Are there situations or clinical findings in the patient where you deviate from your prophylaxis standard (medication, dose, duration, age of the patient)? (multiple answers possible, please specify in answer box) [in patients with recurrent urinary tract infections (UTI)][Comment]

Are there situations or clinical findings in the patient where you deviate from your prophylaxis standard (medication, dose, duration, age of the patient)? (multiple answers possible, please specify in answer box) [if multidrug-resistant organisms (MDRO) are detected in urine cultures]
Yes

Are there situations or clinical findings in the patient where you deviate from your prophylaxis standard (medication, dose, duration, age of the patient)? (multiple answers possible, please specify in answer box) [if multidrug-resistant organisms (MDRO) are detected in urine cultures][Comment]
Anpassung (erfolgt durch Pädiatrie)

Are there situations or clinical findings in the patient where you deviate from your prophylaxis standard (medication, dose, duration, age of the patient)? (multiple answers possible, please specify in answer box) [in newborns]
No

Are there situations or clinical findings in the patient where you deviate from your prophylaxis standard (medication, dose, duration, age of the patient)? (multiple answers possible, please specify in answer box) [in newborns][Comment]

Are there situations or clinical findings in the patient where you deviate from your prophylaxis standard (medication, dose, duration, age of the patient)? (multiple answers possible, please specify in answer box) [Other]

Are there situations or clinical findings in the patient where you deviate from your prophylaxis standard (medication, dose, duration, age of the patient)? (multiple answers possible, please specify in answer box) [Other comment]

Are there situations in which you use intravenous prophylaxis?
No

Voiding analysis

Are there differences in antibiotic prophylaxis between VUS and VCUG in your department?
No

Is a non-pathological urine strip test required in preparation for VUS/VCUG in your department?
No

Is a non-pathological urine culture required in preparation for VUS/VCUG in your department?
No

Do patients only receive post-procedure prophylaxis if vesicoureteral reflux (VUR) is detected in VUS/VCUG?
No

Do other MUS/MCU findings (e.g. urethral valves) have an influence on post-procedure prophylaxis?
No

End of survey

Thank you for taking the time to answer this survey!

May we contact you personally if we have further questions?
Yes

E-mail address:
georg.von.chamier@rems-murr-klniken.de

Survey response 81

Response ID
83

Last page
2

Start language
de

Seed
1136443535

Date started
2025-01-28 12:05:22

Date last action
2025-01-28 12:07:55

Standard Operation Procedure

Who fills out this survey?
Pediatrician

Who fills out this survey? [Other]

In which type of hospital are you employed?
University Hospital

In which type of hospital are you employed? [Other]

In which country do you work?
Deutschland

Does your department have a defined protocol for infection prophylaxis in the context of VUS/VCUG?
No

Has this protocol been transformed into a written standard operating procedure (SOP)?
N/A

Antibiotic Prophylaxis

Are there situations/constellations in which you do not use antibiotic prophylaxis for VUS/VCUG?
Yes

Please specify these situations/constellations: [girl with first uncomplicated UTI without urinary tract dilation in ultrasound]
Yes

Please specify these situations/constellations: [boy with first uncomplicated UTI without urinary tract dilation in ultrasound]
Yes

Please specify these situations/constellations: [in patients with low grade urinary tract dilation (up to 2nd grade) and no UTI in medical history]
Yes

Please specify these situations/constellations: [Other]

If you use antibiotic prophylaxis: which mediaction do you use? (multiple answers and subdivision into age groups possible) [Trimethoprim]
No

If you use antibiotic prophylaxis: which mediaction do you use? (multiple answers and subdivision into age groups possible) [Nitrofurantoin]
No

If you use antibiotic prophylaxis: which mediaction do you use? (multiple answers and subdivision into age groups possible) [Amoxicillin]
Yes

If you use antibiotic prophylaxis: which mediaction do you use? (multiple answers and subdivision into age groups possible) [Cefaclor]
No

If you use antibiotic prophylaxis: which mediaction do you use? (multiple answers and subdivision into age groups possible) [Amoxicillin/clavulanic acid]
No

If you use antibiotic prophylaxis: which mediaction do you use? (multiple answers and subdivision into age groups possible) [Other]
No

Trimethoprim: which time schedule do you use? [Day(s) before VUS/VCUG, how many?]
N/A

Trimethoprim: which time schedule do you use? [Day of VUS/VCUG]
N/A

Trimethoprim: which time schedule do you use? [Day(s) after VUS/VCUG, how many?]
N/A

Nitrofurantoin: which time schedule do you use? [Day(s) before VUS/VCUG, how many?]
N/A

Nitrofurantoin: which time schedule do you use? [Day of VUS/VCUG]
N/A

Nitrofurantoin: which time schedule do you use? [Day(s) after VUS/VCUG, how many?]
N/A

Amoxicillin: in what dosage?
prophylactic (10-20 mg/kg bodyweight/d)

Amoxicillin: in what dosage? [Other]

Amoxicillin: for which age group do you use amoxicillin?
jede

Amoxicillin: which time schedule do you use? [Day(s) before VUS/VCUG, how many?]
No

Amoxicillin: which time schedule do you use? [Day(s) before VUS/VCUG, how many?][Comment]

Amoxicillin: which time schedule do you use? [Day of VUS/VCUG]
Yes

Amoxicillin: which time schedule do you use? [Day of VUS/VCUG][Comment]

Amoxicillin: which time schedule do you use? [Day(s) after VUS/VCUG, how many?]
No

Amoxicillin: which time schedule do you use? [Day(s) after VUS/VCUG, how many?][Comment]

Cefaclor: which time schedule do you use? [Days(s) before VUS/VCUG, how many?]
N/A

Cefaclor: which time schedule do you use? [Day of VUS/VCUG]
N/A

Cefaclor: which time schedule do you use? [Day(s) after VUS/VCUG, how many?]
N/A

Amoxicillin/clavulanic acid: in what dosage? (Amoxicillin portion) [prophylaktic (10-20 mg/Kg bodyweight/d)]
N/A

Amoxicillin/clavulanic acid: in what dosage? (Amoxicillin portion) [double prophylactic (20-40 mg/Kg bodyweight/d)]
N/A

Amoxicillin/clavulanic acid: in what dosage? (Amoxicillin portion) [therapeutic (80-100 mg/Kg bodyweight/d)]
N/A

Amoxicillin/clavulanic acid: which time schedule do you use? [Day(s) before VUS/VCUG]
N/A

Amoxicillin/clavulanic acid: which time schedule do you use? [Day of VUS/VCUG]
N/A

Amoxicillin/clavulanic acid: which time schedule do you use? [Day(s) after VUS/VCUG]
N/A

Other: which time schedule do you use? [Day(s) before VUS/VCUG, how many?]
N/A

Other: which time schedule do you use? [Day of VUS/VCUG]
N/A

Other: which time schedule do you use? [Day(s) after VUS/VCUG, how many?]
N/A

Indication for prophylaxis

Which patients receive periinterventional antibiotic prophylaxis? (multiple answers possible) [all]
No

Which patients receive periinterventional antibiotic prophylaxis? (multiple answers possible) [patients post urinary tract infection/pyelonephritis/urosepsis]
No

Which patients receive periinterventional antibiotic prophylaxis? (multiple answers possible) [patients with urinary tract dilation in ultrasound]
No

Which patients receive periinterventional antibiotic prophylaxis? (multiple answers possible) [patients with prenatal urinary tract dilation]
No

Which patients receive periinterventional antibiotic prophylaxis? (multiple answers possible) [patients post VUR surgery]
No

Which patients receive periinterventional antibiotic prophylaxis? (multiple answers possible) [none]
Yes

Which patients receive periinterventional antibiotic prophylaxis? (multiple answers possible) [Other]

Are there situations or clinical findings in the patient where you deviate from your prophylaxis standard (medication, dose, duration, age of the patient)? (multiple answers possible, please specify in answer box) [in case of already known vesicoureteral reflux (VUR)]
No

Are there situations or clinical findings in the patient where you deviate from your prophylaxis standard (medication, dose, duration, age of the patient)? (multiple answers possible, please specify in answer box) [in case of already known vesicoureteral reflux (VUR)][Comment]

Are there situations or clinical findings in the patient where you deviate from your prophylaxis standard (medication, dose, duration, age of the patient)? (multiple answers possible, please specify in answer box) [in patients with recurrent urinary tract infections (UTI)]
No

Are there situations or clinical findings in the patient where you deviate from your prophylaxis standard (medication, dose, duration, age of the patient)? (multiple answers possible, please specify in answer box) [in patients with recurrent urinary tract infections (UTI)][Comment]

Are there situations or clinical findings in the patient where you deviate from your prophylaxis standard (medication, dose, duration, age of the patient)? (multiple answers possible, please specify in answer box) [if multidrug-resistant organisms (MDRO) are detected in urine cultures]
No

Are there situations or clinical findings in the patient where you deviate from your prophylaxis standard (medication, dose, duration, age of the patient)? (multiple answers possible, please specify in answer box) [if multidrug-resistant organisms (MDRO) are detected in urine cultures][Comment]

Are there situations or clinical findings in the patient where you deviate from your prophylaxis standard (medication, dose, duration, age of the patient)? (multiple answers possible, please specify in answer box) [in newborns]
No

Are there situations or clinical findings in the patient where you deviate from your prophylaxis standard (medication, dose, duration, age of the patient)? (multiple answers possible, please specify in answer box) [in newborns][Comment]

Are there situations or clinical findings in the patient where you deviate from your prophylaxis standard (medication, dose, duration, age of the patient)? (multiple answers possible, please specify in answer box) [Other]

Are there situations or clinical findings in the patient where you deviate from your prophylaxis standard (medication, dose, duration, age of the patient)? (multiple answers possible, please specify in answer box) [Other comment]

Are there situations in which you use intravenous prophylaxis?
No

Voiding analysis

Are there differences in antibiotic prophylaxis between VUS and VCUG in your department?
N/A

Is a non-pathological urine strip test required in preparation for VUS/VCUG in your department?
N/A

Is a non-pathological urine culture required in preparation for VUS/VCUG in your department?
N/A

Do patients only receive post-procedure prophylaxis if vesicoureteral reflux (VUR) is detected in VUS/VCUG?
N/A

Do other MUS/MCU findings (e.g. urethral valves) have an influence on post-procedure prophylaxis?
N/A

End of survey

May we contact you personally if we have further questions?
N/A

Survey response 82

Response ID
84

Date submitted
2025-01-28 12:08:23

Last page
5

Start language
de

Seed
996513007

Date started
2025-01-28 12:05:41

Date last action
2025-01-28 12:08:23

Standard Operation Procedure

Who fills out this survey?
Other

Who fills out this survey? [Other]
Kindernephrologie

In which type of hospital are you employed?
Other

In which type of hospital are you employed? [Other]
Praxis

In which country do you work?
Deutschland

Does your department have a defined protocol for infection prophylaxis in the context of VUS/VCUG?
Yes

Has this protocol been transformed into a written standard operating procedure (SOP)?
Yes

Which department is the author of the SOP for infection prophylaxis in VUS/VCUG?
Pediatric nephrology

Which department is the author of the SOP for infection prophylaxis in VUS/VCUG? [Other]

Antibiotic Prophylaxis

Are there situations/constellations in which you do not use antibiotic prophylaxis for VUS/VCUG?
No

Please specify these situations/constellations: [girl with first uncomplicated UTI without urinary tract dilation in ultrasound]
N/A

Please specify these situations/constellations: [boy with first uncomplicated UTI without urinary tract dilation in ultrasound]
N/A

Please specify these situations/constellations: [in patients with low grade urinary tract dilation (up to 2nd grade) and no UTI in medical history]
N/A

If you use antibiotic prophylaxis: which mediaction do you use? (multiple answers and subdivision into age groups possible) [Trimethoprim]
Yes

If you use antibiotic prophylaxis: which mediaction do you use? (multiple answers and subdivision into age groups possible) [Nitrofurantoin]
No

If you use antibiotic prophylaxis: which mediaction do you use? (multiple answers and subdivision into age groups possible) [Amoxicillin]
No

If you use antibiotic prophylaxis: which mediaction do you use? (multiple answers and subdivision into age groups possible) [Cefaclor]
No

If you use antibiotic prophylaxis: which mediaction do you use? (multiple answers and subdivision into age groups possible) [Amoxicillin/clavulanic acid]
No

If you use antibiotic prophylaxis: which mediaction do you use? (multiple answers and subdivision into age groups possible) [Other]
No

Trimethoprim: in what dosage?
therapeutic (6 mg/kg bodyweight/d)

Trimethoprim: in what dosage? [Other]

Trimethoprim: for which age group do you use trimethoprim?
>6 Lebenswochen

Trimethoprim: which time schedule do you use? [Day(s) before VUS/VCUG, how many?]
No

Trimethoprim: which time schedule do you use? [Day(s) before VUS/VCUG, how many?][Comment]

Trimethoprim: which time schedule do you use? [Day of VUS/VCUG]
Yes

Trimethoprim: which time schedule do you use? [Day of VUS/VCUG][Comment]

Trimethoprim: which time schedule do you use? [Day(s) after VUS/VCUG, how many?]
Yes

Trimethoprim: which time schedule do you use? [Day(s) after VUS/VCUG, how many?][Comment]
2

Nitrofurantoin: which time schedule do you use? [Day(s) before VUS/VCUG, how many?]
N/A

Nitrofurantoin: which time schedule do you use? [Day of VUS/VCUG]
N/A

Nitrofurantoin: which time schedule do you use? [Day(s) after VUS/VCUG, how many?]
N/A

Amoxicillin: which time schedule do you use? [Day(s) before VUS/VCUG, how many?]
N/A

Amoxicillin: which time schedule do you use? [Day of VUS/VCUG]
N/A

Amoxicillin: which time schedule do you use? [Day(s) after VUS/VCUG, how many?]
N/A

Cefaclor: which time schedule do you use? [Days(s) before VUS/VCUG, how many?]
N/A

Cefaclor: which time schedule do you use? [Day of VUS/VCUG]
N/A

Cefaclor: which time schedule do you use? [Day(s) after VUS/VCUG, how many?]
N/A

Amoxicillin/clavulanic acid: in what dosage? (Amoxicillin portion) [prophylaktic (10-20 mg/Kg bodyweight/d)]
N/A

Amoxicillin/clavulanic acid: in what dosage? (Amoxicillin portion) [double prophylactic (20-40 mg/Kg bodyweight/d)]
N/A

Amoxicillin/clavulanic acid: in what dosage? (Amoxicillin portion) [therapeutic (80-100 mg/Kg bodyweight/d)]
N/A

Amoxicillin/clavulanic acid: which time schedule do you use? [Day(s) before VUS/VCUG]
N/A

Amoxicillin/clavulanic acid: which time schedule do you use? [Day of VUS/VCUG]
N/A

Amoxicillin/clavulanic acid: which time schedule do you use? [Day(s) after VUS/VCUG]
N/A

Other: which time schedule do you use? [Day(s) before VUS/VCUG, how many?]
N/A

Other: which time schedule do you use? [Day of VUS/VCUG]
N/A

Other: which time schedule do you use? [Day(s) after VUS/VCUG, how many?]
N/A

Indication for prophylaxis

Which patients receive periinterventional antibiotic prophylaxis? (multiple answers possible) [all]
Yes

Which patients receive periinterventional antibiotic prophylaxis? (multiple answers possible) [patients post urinary tract infection/pyelonephritis/urosepsis]
N/A

Which patients receive periinterventional antibiotic prophylaxis? (multiple answers possible) [patients with urinary tract dilation in ultrasound]
N/A

Which patients receive periinterventional antibiotic prophylaxis? (multiple answers possible) [patients with prenatal urinary tract dilation]
N/A

Which patients receive periinterventional antibiotic prophylaxis? (multiple answers possible) [patients post VUR surgery]
N/A

Which patients receive periinterventional antibiotic prophylaxis? (multiple answers possible) [none]
N/A

Are there situations or clinical findings in the patient where you deviate from your prophylaxis standard (medication, dose, duration, age of the patient)? (multiple answers possible, please specify in answer box) [in case of already known vesicoureteral reflux (VUR)]
No

Are there situations or clinical findings in the patient where you deviate from your prophylaxis standard (medication, dose, duration, age of the patient)? (multiple answers possible, please specify in answer box) [in case of already known vesicoureteral reflux (VUR)][Comment]

Are there situations or clinical findings in the patient where you deviate from your prophylaxis standard (medication, dose, duration, age of the patient)? (multiple answers possible, please specify in answer box) [in patients with recurrent urinary tract infections (UTI)]
No

Are there situations or clinical findings in the patient where you deviate from your prophylaxis standard (medication, dose, duration, age of the patient)? (multiple answers possible, please specify in answer box) [in patients with recurrent urinary tract infections (UTI)][Comment]

Are there situations or clinical findings in the patient where you deviate from your prophylaxis standard (medication, dose, duration, age of the patient)? (multiple answers possible, please specify in answer box) [if multidrug-resistant organisms (MDRO) are detected in urine cultures]
Yes

Are there situations or clinical findings in the patient where you deviate from your prophylaxis standard (medication, dose, duration, age of the patient)? (multiple answers possible, please specify in answer box) [if multidrug-resistant organisms (MDRO) are detected in urine cultures][Comment]

Are there situations or clinical findings in the patient where you deviate from your prophylaxis standard (medication, dose, duration, age of the patient)? (multiple answers possible, please specify in answer box) [in newborns]
No

Are there situations or clinical findings in the patient where you deviate from your prophylaxis standard (medication, dose, duration, age of the patient)? (multiple answers possible, please specify in answer box) [in newborns][Comment]

Are there situations or clinical findings in the patient where you deviate from your prophylaxis standard (medication, dose, duration, age of the patient)? (multiple answers possible, please specify in answer box) [Other]

Are there situations or clinical findings in the patient where you deviate from your prophylaxis standard (medication, dose, duration, age of the patient)? (multiple answers possible, please specify in answer box) [Other comment]

Are there situations in which you use intravenous prophylaxis?
No

Voiding analysis

Are there differences in antibiotic prophylaxis between VUS and VCUG in your department?
No

Is a non-pathological urine strip test required in preparation for VUS/VCUG in your department?
Yes

What is the maximum amount of days accepted for urine strip test to be performed prior to the exam?
1

Is a non-pathological urine culture required in preparation for VUS/VCUG in your department?
No

Do patients only receive post-procedure prophylaxis if vesicoureteral reflux (VUR) is detected in VUS/VCUG?
No

Do other MUS/MCU findings (e.g. urethral valves) have an influence on post-procedure prophylaxis?
No

End of survey

Thank you for taking the time to answer this survey!

May we contact you personally if we have further questions?
No

Survey response 83

Response ID
85

Last page
0

Start language
de

Seed
1416122137

Date started
2025-01-28 12:06:11

Date last action
2025-01-28 12:06:11

Standard Operation Procedure

Does your department have a defined protocol for infection prophylaxis in the context of VUS/VCUG?
N/A

Has this protocol been transformed into a written standard operating procedure (SOP)?
N/A

Antibiotic Prophylaxis

Are there situations/constellations in which you do not use antibiotic prophylaxis for VUS/VCUG?
N/A

Please specify these situations/constellations: [girl with first uncomplicated UTI without urinary tract dilation in ultrasound]
N/A

Please specify these situations/constellations: [boy with first uncomplicated UTI without urinary tract dilation in ultrasound]
N/A

Please specify these situations/constellations: [in patients with low grade urinary tract dilation (up to 2nd grade) and no UTI in medical history]
N/A

If you use antibiotic prophylaxis: which mediaction do you use? (multiple answers and subdivision into age groups possible) [Trimethoprim]
N/A

If you use antibiotic prophylaxis: which mediaction do you use? (multiple answers and subdivision into age groups possible) [Nitrofurantoin]
N/A

If you use antibiotic prophylaxis: which mediaction do you use? (multiple answers and subdivision into age groups possible) [Amoxicillin]
N/A

If you use antibiotic prophylaxis: which mediaction do you use? (multiple answers and subdivision into age groups possible) [Cefaclor]
N/A

If you use antibiotic prophylaxis: which mediaction do you use? (multiple answers and subdivision into age groups possible) [Amoxicillin/clavulanic acid]
N/A

If you use antibiotic prophylaxis: which mediaction do you use? (multiple answers and subdivision into age groups possible) [Other]
N/A

Trimethoprim: which time schedule do you use? [Day(s) before VUS/VCUG, how many?]
N/A

Trimethoprim: which time schedule do you use? [Day of VUS/VCUG]
N/A

Trimethoprim: which time schedule do you use? [Day(s) after VUS/VCUG, how many?]
N/A

Nitrofurantoin: which time schedule do you use? [Day(s) before VUS/VCUG, how many?]
N/A

Nitrofurantoin: which time schedule do you use? [Day of VUS/VCUG]
N/A

Nitrofurantoin: which time schedule do you use? [Day(s) after VUS/VCUG, how many?]
N/A

Amoxicillin: which time schedule do you use? [Day(s) before VUS/VCUG, how many?]
N/A

Amoxicillin: which time schedule do you use? [Day of VUS/VCUG]
N/A

Amoxicillin: which time schedule do you use? [Day(s) after VUS/VCUG, how many?]
N/A

Cefaclor: which time schedule do you use? [Days(s) before VUS/VCUG, how many?]
N/A

Cefaclor: which time schedule do you use? [Day of VUS/VCUG]
N/A

Cefaclor: which time schedule do you use? [Day(s) after VUS/VCUG, how many?]
N/A

Amoxicillin/clavulanic acid: in what dosage? (Amoxicillin portion) [prophylaktic (10-20 mg/Kg bodyweight/d)]
N/A

Amoxicillin/clavulanic acid: in what dosage? (Amoxicillin portion) [double prophylactic (20-40 mg/Kg bodyweight/d)]
N/A

Amoxicillin/clavulanic acid: in what dosage? (Amoxicillin portion) [therapeutic (80-100 mg/Kg bodyweight/d)]
N/A

Amoxicillin/clavulanic acid: which time schedule do you use? [Day(s) before VUS/VCUG]
N/A

Amoxicillin/clavulanic acid: which time schedule do you use? [Day of VUS/VCUG]
N/A

Amoxicillin/clavulanic acid: which time schedule do you use? [Day(s) after VUS/VCUG]
N/A

Other: which time schedule do you use? [Day(s) before VUS/VCUG, how many?]
N/A

Other: which time schedule do you use? [Day of VUS/VCUG]
N/A

Other: which time schedule do you use? [Day(s) after VUS/VCUG, how many?]
N/A

Indication for prophylaxis

Which patients receive periinterventional antibiotic prophylaxis? (multiple answers possible) [all]
N/A

Which patients receive periinterventional antibiotic prophylaxis? (multiple answers possible) [patients post urinary tract infection/pyelonephritis/urosepsis]
N/A

Which patients receive periinterventional antibiotic prophylaxis? (multiple answers possible) [patients with urinary tract dilation in ultrasound]
N/A

Which patients receive periinterventional antibiotic prophylaxis? (multiple answers possible) [patients with prenatal urinary tract dilation]
N/A

Which patients receive periinterventional antibiotic prophylaxis? (multiple answers possible) [patients post VUR surgery]
N/A

Which patients receive periinterventional antibiotic prophylaxis? (multiple answers possible) [none]
N/A

Are there situations or clinical findings in the patient where you deviate from your prophylaxis standard (medication, dose, duration, age of the patient)? (multiple answers possible, please specify in answer box) [in case of already known vesicoureteral reflux (VUR)]
N/A

Are there situations or clinical findings in the patient where you deviate from your prophylaxis standard (medication, dose, duration, age of the patient)? (multiple answers possible, please specify in answer box) [in patients with recurrent urinary tract infections (UTI)]
N/A

Are there situations or clinical findings in the patient where you deviate from your prophylaxis standard (medication, dose, duration, age of the patient)? (multiple answers possible, please specify in answer box) [if multidrug-resistant organisms (MDRO) are detected in urine cultures]
N/A

Are there situations or clinical findings in the patient where you deviate from your prophylaxis standard (medication, dose, duration, age of the patient)? (multiple answers possible, please specify in answer box) [in newborns]
N/A

Are there situations in which you use intravenous prophylaxis?
N/A

Voiding analysis

Are there differences in antibiotic prophylaxis between VUS and VCUG in your department?
N/A

Is a non-pathological urine strip test required in preparation for VUS/VCUG in your department?
N/A

Is a non-pathological urine culture required in preparation for VUS/VCUG in your department?
N/A

Do patients only receive post-procedure prophylaxis if vesicoureteral reflux (VUR) is detected in VUS/VCUG?
N/A

Do other MUS/MCU findings (e.g. urethral valves) have an influence on post-procedure prophylaxis?
N/A

End of survey

May we contact you personally if we have further questions?
N/A

Survey response 84

Response ID
86

Date submitted
2025-01-28 12:14:44

Last page
5

Start language
de

Seed
2002060493

Date started
2025-01-28 12:11:07

Date last action
2025-01-28 12:14:44

Standard Operation Procedure

Who fills out this survey?
Pediatrician

Who fills out this survey? [Other]

In which type of hospital are you employed?
University Hospital

In which type of hospital are you employed? [Other]

In which country do you work?
Deutschland

Does your department have a defined protocol for infection prophylaxis in the context of VUS/VCUG?
Yes

Has this protocol been transformed into a written standard operating procedure (SOP)?
No

Antibiotic Prophylaxis

Are there situations/constellations in which you do not use antibiotic prophylaxis for VUS/VCUG?
No

Please specify these situations/constellations: [girl with first uncomplicated UTI without urinary tract dilation in ultrasound]
N/A

Please specify these situations/constellations: [boy with first uncomplicated UTI without urinary tract dilation in ultrasound]
N/A

Please specify these situations/constellations: [in patients with low grade urinary tract dilation (up to 2nd grade) and no UTI in medical history]
N/A

If you use antibiotic prophylaxis: which mediaction do you use? (multiple answers and subdivision into age groups possible) [Trimethoprim]
Yes

If you use antibiotic prophylaxis: which mediaction do you use? (multiple answers and subdivision into age groups possible) [Nitrofurantoin]
No

If you use antibiotic prophylaxis: which mediaction do you use? (multiple answers and subdivision into age groups possible) [Amoxicillin]
No

If you use antibiotic prophylaxis: which mediaction do you use? (multiple answers and subdivision into age groups possible) [Cefaclor]
Yes

If you use antibiotic prophylaxis: which mediaction do you use? (multiple answers and subdivision into age groups possible) [Amoxicillin/clavulanic acid]
No

If you use antibiotic prophylaxis: which mediaction do you use? (multiple answers and subdivision into age groups possible) [Other]
No

Trimethoprim: in what dosage?
prophylactic (2 mg/kg bodyweight/d)

Trimethoprim: in what dosage? [Other]

Trimethoprim: for which age group do you use trimethoprim?
ab 8 Wochen

Trimethoprim: which time schedule do you use? [Day(s) before VUS/VCUG, how many?]
No

Trimethoprim: which time schedule do you use? [Day(s) before VUS/VCUG, how many?][Comment]

Trimethoprim: which time schedule do you use? [Day of VUS/VCUG]
Yes

Trimethoprim: which time schedule do you use? [Day of VUS/VCUG][Comment]

Trimethoprim: which time schedule do you use? [Day(s) after VUS/VCUG, how many?]
No

Trimethoprim: which time schedule do you use? [Day(s) after VUS/VCUG, how many?][Comment]

Nitrofurantoin: which time schedule do you use? [Day(s) before VUS/VCUG, how many?]
N/A

Nitrofurantoin: which time schedule do you use? [Day of VUS/VCUG]
N/A

Nitrofurantoin: which time schedule do you use? [Day(s) after VUS/VCUG, how many?]
N/A

Amoxicillin: which time schedule do you use? [Day(s) before VUS/VCUG, how many?]
N/A

Amoxicillin: which time schedule do you use? [Day of VUS/VCUG]
N/A

Amoxicillin: which time schedule do you use? [Day(s) after VUS/VCUG, how many?]
N/A

Cefaclor: in what dosage?
prophylactic (10 mg/kg bodyweight/d)

Cefaclor: in what dosage? [Other]

Cefaclor: for which age group do you use cefaclor?
Geburt - 8 Wochen

Cefaclor: which time schedule do you use? [Days(s) before VUS/VCUG, how many?]
No

Cefaclor: which time schedule do you use? [Days(s) before VUS/VCUG, how many?][Comment]

Cefaclor: which time schedule do you use? [Day of VUS/VCUG]
Yes

Cefaclor: which time schedule do you use? [Day of VUS/VCUG][Comment]

Cefaclor: which time schedule do you use? [Day(s) after VUS/VCUG, how many?]
No

Cefaclor: which time schedule do you use? [Day(s) after VUS/VCUG, how many?][Comment]

Amoxicillin/clavulanic acid: in what dosage? (Amoxicillin portion) [prophylaktic (10-20 mg/Kg bodyweight/d)]
N/A

Amoxicillin/clavulanic acid: in what dosage? (Amoxicillin portion) [double prophylactic (20-40 mg/Kg bodyweight/d)]
N/A

Amoxicillin/clavulanic acid: in what dosage? (Amoxicillin portion) [therapeutic (80-100 mg/Kg bodyweight/d)]
N/A

Amoxicillin/clavulanic acid: which time schedule do you use? [Day(s) before VUS/VCUG]
N/A

Amoxicillin/clavulanic acid: which time schedule do you use? [Day of VUS/VCUG]
N/A

Amoxicillin/clavulanic acid: which time schedule do you use? [Day(s) after VUS/VCUG]
N/A

Other: which time schedule do you use? [Day(s) before VUS/VCUG, how many?]
N/A

Other: which time schedule do you use? [Day of VUS/VCUG]
N/A

Other: which time schedule do you use? [Day(s) after VUS/VCUG, how many?]
N/A

Indication for prophylaxis

Which patients receive periinterventional antibiotic prophylaxis? (multiple answers possible) [all]
Yes

Which patients receive periinterventional antibiotic prophylaxis? (multiple answers possible) [patients post urinary tract infection/pyelonephritis/urosepsis]
N/A

Which patients receive periinterventional antibiotic prophylaxis? (multiple answers possible) [patients with urinary tract dilation in ultrasound]
N/A

Which patients receive periinterventional antibiotic prophylaxis? (multiple answers possible) [patients with prenatal urinary tract dilation]
N/A

Which patients receive periinterventional antibiotic prophylaxis? (multiple answers possible) [patients post VUR surgery]
N/A

Which patients receive periinterventional antibiotic prophylaxis? (multiple answers possible) [none]
N/A

Are there situations or clinical findings in the patient where you deviate from your prophylaxis standard (medication, dose, duration, age of the patient)? (multiple answers possible, please specify in answer box) [in case of already known vesicoureteral reflux (VUR)]
No

Are there situations or clinical findings in the patient where you deviate from your prophylaxis standard (medication, dose, duration, age of the patient)? (multiple answers possible, please specify in answer box) [in case of already known vesicoureteral reflux (VUR)][Comment]

Are there situations or clinical findings in the patient where you deviate from your prophylaxis standard (medication, dose, duration, age of the patient)? (multiple answers possible, please specify in answer box) [in patients with recurrent urinary tract infections (UTI)]
No

Are there situations or clinical findings in the patient where you deviate from your prophylaxis standard (medication, dose, duration, age of the patient)? (multiple answers possible, please specify in answer box) [in patients with recurrent urinary tract infections (UTI)][Comment]

Are there situations or clinical findings in the patient where you deviate from your prophylaxis standard (medication, dose, duration, age of the patient)? (multiple answers possible, please specify in answer box) [if multidrug-resistant organisms (MDRO) are detected in urine cultures]
Yes

Are there situations or clinical findings in the patient where you deviate from your prophylaxis standard (medication, dose, duration, age of the patient)? (multiple answers possible, please specify in answer box) [if multidrug-resistant organisms (MDRO) are detected in urine cultures][Comment]
je nach Antibiogramm, ggf. Nitrofurantoin oder Amoxicillin/Clavulansäure

Are there situations or clinical findings in the patient where you deviate from your prophylaxis standard (medication, dose, duration, age of the patient)? (multiple answers possible, please specify in answer box) [in newborns]
No

Are there situations or clinical findings in the patient where you deviate from your prophylaxis standard (medication, dose, duration, age of the patient)? (multiple answers possible, please specify in answer box) [in newborns][Comment]

Are there situations or clinical findings in the patient where you deviate from your prophylaxis standard (medication, dose, duration, age of the patient)? (multiple answers possible, please specify in answer box) [Other]

Are there situations or clinical findings in the patient where you deviate from your prophylaxis standard (medication, dose, duration, age of the patient)? (multiple answers possible, please specify in answer box) [Other comment]

Are there situations in which you use intravenous prophylaxis?
Yes

In which situations?
bei multiresistenten Keimen wenn keine orale Alternative möglich ist

Voiding analysis

Are there differences in antibiotic prophylaxis between VUS and VCUG in your department?
No

Is a non-pathological urine strip test required in preparation for VUS/VCUG in your department?
Yes

What is the maximum amount of days accepted for urine strip test to be performed prior to the exam?
1

Is a non-pathological urine culture required in preparation for VUS/VCUG in your department?
No

Do patients only receive post-procedure prophylaxis if vesicoureteral reflux (VUR) is detected in VUS/VCUG?
No

Do other MUS/MCU findings (e.g. urethral valves) have an influence on post-procedure prophylaxis?
No

End of survey

Thank you for taking the time to answer this survey!

May we contact you personally if we have further questions?
No

Survey response 85

Response ID
87

Date submitted
2025-01-28 12:25:30

Last page
5

Start language
de

Seed
1873357362

Date started
2025-01-28 12:13:26

Date last action
2025-01-28 12:25:30

Standard Operation Procedure

Who fills out this survey?
Pediatrician

Who fills out this survey? [Other]

In which type of hospital are you employed?
Community hospital (>50 beds for pediatric patients)

In which type of hospital are you employed? [Other]

In which country do you work?
Deutschland

Does your department have a defined protocol for infection prophylaxis in the context of VUS/VCUG?
No

Has this protocol been transformed into a written standard operating procedure (SOP)?
N/A

Antibiotic Prophylaxis

Are there situations/constellations in which you do not use antibiotic prophylaxis for VUS/VCUG?
Yes

Please specify these situations/constellations: [girl with first uncomplicated UTI without urinary tract dilation in ultrasound]
Yes

Please specify these situations/constellations: [boy with first uncomplicated UTI without urinary tract dilation in ultrasound]
Yes

Please specify these situations/constellations: [in patients with low grade urinary tract dilation (up to 2nd grade) and no UTI in medical history]
Yes

Please specify these situations/constellations: [Other]

If you use antibiotic prophylaxis: which mediaction do you use? (multiple answers and subdivision into age groups possible) [Trimethoprim]
No

If you use antibiotic prophylaxis: which mediaction do you use? (multiple answers and subdivision into age groups possible) [Nitrofurantoin]
No

If you use antibiotic prophylaxis: which mediaction do you use? (multiple answers and subdivision into age groups possible) [Amoxicillin]
No

If you use antibiotic prophylaxis: which mediaction do you use? (multiple answers and subdivision into age groups possible) [Cefaclor]
No

If you use antibiotic prophylaxis: which mediaction do you use? (multiple answers and subdivision into age groups possible) [Amoxicillin/clavulanic acid]
No

If you use antibiotic prophylaxis: which mediaction do you use? (multiple answers and subdivision into age groups possible) [Other]
No

Trimethoprim: which time schedule do you use? [Day(s) before VUS/VCUG, how many?]
N/A

Trimethoprim: which time schedule do you use? [Day of VUS/VCUG]
N/A

Trimethoprim: which time schedule do you use? [Day(s) after VUS/VCUG, how many?]
N/A

Nitrofurantoin: which time schedule do you use? [Day(s) before VUS/VCUG, how many?]
N/A

Nitrofurantoin: which time schedule do you use? [Day of VUS/VCUG]
N/A

Nitrofurantoin: which time schedule do you use? [Day(s) after VUS/VCUG, how many?]
N/A

Amoxicillin: which time schedule do you use? [Day(s) before VUS/VCUG, how many?]
N/A

Amoxicillin: which time schedule do you use? [Day of VUS/VCUG]
N/A

Amoxicillin: which time schedule do you use? [Day(s) after VUS/VCUG, how many?]
N/A

Cefaclor: which time schedule do you use? [Days(s) before VUS/VCUG, how many?]
N/A

Cefaclor: which time schedule do you use? [Day of VUS/VCUG]
N/A

Cefaclor: which time schedule do you use? [Day(s) after VUS/VCUG, how many?]
N/A

Amoxicillin/clavulanic acid: in what dosage? (Amoxicillin portion) [prophylaktic (10-20 mg/Kg bodyweight/d)]
N/A

Amoxicillin/clavulanic acid: in what dosage? (Amoxicillin portion) [double prophylactic (20-40 mg/Kg bodyweight/d)]
N/A

Amoxicillin/clavulanic acid: in what dosage? (Amoxicillin portion) [therapeutic (80-100 mg/Kg bodyweight/d)]
N/A

Amoxicillin/clavulanic acid: which time schedule do you use? [Day(s) before VUS/VCUG]
N/A

Amoxicillin/clavulanic acid: which time schedule do you use? [Day of VUS/VCUG]
N/A

Amoxicillin/clavulanic acid: which time schedule do you use? [Day(s) after VUS/VCUG]
N/A

Other: which time schedule do you use? [Day(s) before VUS/VCUG, how many?]
N/A

Other: which time schedule do you use? [Day of VUS/VCUG]
N/A

Other: which time schedule do you use? [Day(s) after VUS/VCUG, how many?]
N/A

Indication for prophylaxis

Which patients receive periinterventional antibiotic prophylaxis? (multiple answers possible) [all]
No

Which patients receive periinterventional antibiotic prophylaxis? (multiple answers possible) [patients post urinary tract infection/pyelonephritis/urosepsis]
No

Which patients receive periinterventional antibiotic prophylaxis? (multiple answers possible) [patients with urinary tract dilation in ultrasound]
No

Which patients receive periinterventional antibiotic prophylaxis? (multiple answers possible) [patients with prenatal urinary tract dilation]
No

Which patients receive periinterventional antibiotic prophylaxis? (multiple answers possible) [patients post VUR surgery]
No

Which patients receive periinterventional antibiotic prophylaxis? (multiple answers possible) [none]
Yes

Which patients receive periinterventional antibiotic prophylaxis? (multiple answers possible) [Other]
Es es denn, einen dauerprohylaxe besteht bereits

Are there situations or clinical findings in the patient where you deviate from your prophylaxis standard (medication, dose, duration, age of the patient)? (multiple answers possible, please specify in answer box) [in case of already known vesicoureteral reflux (VUR)]
No

Are there situations or clinical findings in the patient where you deviate from your prophylaxis standard (medication, dose, duration, age of the patient)? (multiple answers possible, please specify in answer box) [in case of already known vesicoureteral reflux (VUR)][Comment]

Are there situations or clinical findings in the patient where you deviate from your prophylaxis standard (medication, dose, duration, age of the patient)? (multiple answers possible, please specify in answer box) [in patients with recurrent urinary tract infections (UTI)]
No

Are there situations or clinical findings in the patient where you deviate from your prophylaxis standard (medication, dose, duration, age of the patient)? (multiple answers possible, please specify in answer box) [in patients with recurrent urinary tract infections (UTI)][Comment]

Are there situations or clinical findings in the patient where you deviate from your prophylaxis standard (medication, dose, duration, age of the patient)? (multiple answers possible, please specify in answer box) [if multidrug-resistant organisms (MDRO) are detected in urine cultures]
Yes

Are there situations or clinical findings in the patient where you deviate from your prophylaxis standard (medication, dose, duration, age of the patient)? (multiple answers possible, please specify in answer box) [if multidrug-resistant organisms (MDRO) are detected in urine cultures][Comment]
Bezieht sich auf die dauerprophylaxe, da keine periinterventionelle Prophylaxe

Are there situations or clinical findings in the patient where you deviate from your prophylaxis standard (medication, dose, duration, age of the patient)? (multiple answers possible, please specify in answer box) [in newborns]
No

Are there situations or clinical findings in the patient where you deviate from your prophylaxis standard (medication, dose, duration, age of the patient)? (multiple answers possible, please specify in answer box) [in newborns][Comment]

Are there situations or clinical findings in the patient where you deviate from your prophylaxis standard (medication, dose, duration, age of the patient)? (multiple answers possible, please specify in answer box) [Other]

Are there situations or clinical findings in the patient where you deviate from your prophylaxis standard (medication, dose, duration, age of the patient)? (multiple answers possible, please specify in answer box) [Other comment]

Are there situations in which you use intravenous prophylaxis?
No

Voiding analysis

Are there differences in antibiotic prophylaxis between VUS and VCUG in your department?
No

Is a non-pathological urine strip test required in preparation for VUS/VCUG in your department?
Yes

What is the maximum amount of days accepted for urine strip test to be performed prior to the exam?
1

Is a non-pathological urine culture required in preparation for VUS/VCUG in your department?
No

Do patients only receive post-procedure prophylaxis if vesicoureteral reflux (VUR) is detected in VUS/VCUG?
Yes

Please explain your approach:
Prophylaxe TMP/ Nitrofurantoin nach Alter/ Zulassung postinterventionell geht in Dauerprophylaxe über

Do other MUS/MCU findings (e.g. urethral valves) have an influence on post-procedure prophylaxis?
No

End of survey

Thank you for taking the time to answer this survey!

May we contact you personally if we have further questions?
No

Survey response 86

Response ID
88

Date submitted
2025-01-28 12:51:21

Last page
5

Start language
de

Seed
780297580

Date started
2025-01-28 12:42:16

Date last action
2025-01-28 12:51:21

Standard Operation Procedure

Who fills out this survey?
Pediatrician

Who fills out this survey? [Other]

In which type of hospital are you employed?
University Hospital

In which type of hospital are you employed? [Other]

In which country do you work?
D

Does your department have a defined protocol for infection prophylaxis in the context of VUS/VCUG?
No

Has this protocol been transformed into a written standard operating procedure (SOP)?
N/A

Antibiotic Prophylaxis

Are there situations/constellations in which you do not use antibiotic prophylaxis for VUS/VCUG?
No

Please specify these situations/constellations: [girl with first uncomplicated UTI without urinary tract dilation in ultrasound]
N/A

Please specify these situations/constellations: [boy with first uncomplicated UTI without urinary tract dilation in ultrasound]
N/A

Please specify these situations/constellations: [in patients with low grade urinary tract dilation (up to 2nd grade) and no UTI in medical history]
N/A

If you use antibiotic prophylaxis: which mediaction do you use? (multiple answers and subdivision into age groups possible) [Trimethoprim]
Yes

If you use antibiotic prophylaxis: which mediaction do you use? (multiple answers and subdivision into age groups possible) [Nitrofurantoin]
Yes

If you use antibiotic prophylaxis: which mediaction do you use? (multiple answers and subdivision into age groups possible) [Amoxicillin]
No

If you use antibiotic prophylaxis: which mediaction do you use? (multiple answers and subdivision into age groups possible) [Cefaclor]
No

If you use antibiotic prophylaxis: which mediaction do you use? (multiple answers and subdivision into age groups possible) [Amoxicillin/clavulanic acid]
Yes

If you use antibiotic prophylaxis: which mediaction do you use? (multiple answers and subdivision into age groups possible) [Other]
No

Trimethoprim: in what dosage?
therapeutic (6 mg/kg bodyweight/d)

Trimethoprim: in what dosage? [Other]

Trimethoprim: for which age group do you use trimethoprim?
Ab dem 30. Lebenstag

Trimethoprim: which time schedule do you use? [Day(s) before VUS/VCUG, how many?]
Yes

Trimethoprim: which time schedule do you use? [Day(s) before VUS/VCUG, how many?][Comment]
1

Trimethoprim: which time schedule do you use? [Day of VUS/VCUG]
Yes

Trimethoprim: which time schedule do you use? [Day of VUS/VCUG][Comment]

Trimethoprim: which time schedule do you use? [Day(s) after VUS/VCUG, how many?]
Yes

Trimethoprim: which time schedule do you use? [Day(s) after VUS/VCUG, how many?][Comment]
1

Nitrofurantoin: in what dosage?
therapeutic (5 mg/kg bodyweight/d)

Nitrofurantoin: in what dosage? [Other]

Nitrofurantoin: for which age group do you use nitrofurantoin?
Ab dem 2-3. Lebensmonat

Nitrofurantoin: which time schedule do you use? [Day(s) before VUS/VCUG, how many?]
Yes

Nitrofurantoin: which time schedule do you use? [Day(s) before VUS/VCUG, how many?][Comment]
1

Nitrofurantoin: which time schedule do you use? [Day of VUS/VCUG]
Yes

Nitrofurantoin: which time schedule do you use? [Day of VUS/VCUG][Comment]

Nitrofurantoin: which time schedule do you use? [Day(s) after VUS/VCUG, how many?]
Yes

Nitrofurantoin: which time schedule do you use? [Day(s) after VUS/VCUG, how many?][Comment]
1

Amoxicillin: which time schedule do you use? [Day(s) before VUS/VCUG, how many?]
N/A

Amoxicillin: which time schedule do you use? [Day of VUS/VCUG]
N/A

Amoxicillin: which time schedule do you use? [Day(s) after VUS/VCUG, how many?]
N/A

Cefaclor: which time schedule do you use? [Days(s) before VUS/VCUG, how many?]
N/A

Cefaclor: which time schedule do you use? [Day of VUS/VCUG]
N/A

Cefaclor: which time schedule do you use? [Day(s) after VUS/VCUG, how many?]
N/A

Amoxicillin/clavulanic acid: in what dosage? (Amoxicillin portion) [prophylaktic (10-20 mg/Kg bodyweight/d)]
No

Amoxicillin/clavulanic acid: in what dosage? (Amoxicillin portion) [double prophylactic (20-40 mg/Kg bodyweight/d)]
Yes

Amoxicillin/clavulanic acid: in what dosage? (Amoxicillin portion) [therapeutic (80-100 mg/Kg bodyweight/d)]
No

Amoxicillin/clavulanic acid: in what dosage? (Amoxicillin portion) [Other]

Amoxicillin/clavulanic acid: for which age group do you use amoxicillin/clavulanic acid?
Ab dem 1. Lebenstag

Amoxicillin/clavulanic acid: which time schedule do you use? [Day(s) before VUS/VCUG]
Yes

Amoxicillin/clavulanic acid: which time schedule do you use? [Day(s) before VUS/VCUG][Comment]
1

Amoxicillin/clavulanic acid: which time schedule do you use? [Day of VUS/VCUG]
Yes

Amoxicillin/clavulanic acid: which time schedule do you use? [Day of VUS/VCUG][Comment]

Amoxicillin/clavulanic acid: which time schedule do you use? [Day(s) after VUS/VCUG]
Yes

Amoxicillin/clavulanic acid: which time schedule do you use? [Day(s) after VUS/VCUG][Comment]
1

Other: which time schedule do you use? [Day(s) before VUS/VCUG, how many?]
N/A

Other: which time schedule do you use? [Day of VUS/VCUG]
N/A

Other: which time schedule do you use? [Day(s) after VUS/VCUG, how many?]
N/A

Indication for prophylaxis

Which patients receive periinterventional antibiotic prophylaxis? (multiple answers possible) [all]
Yes

Which patients receive periinterventional antibiotic prophylaxis? (multiple answers possible) [patients post urinary tract infection/pyelonephritis/urosepsis]
N/A

Which patients receive periinterventional antibiotic prophylaxis? (multiple answers possible) [patients with urinary tract dilation in ultrasound]
N/A

Which patients receive periinterventional antibiotic prophylaxis? (multiple answers possible) [patients with prenatal urinary tract dilation]
N/A

Which patients receive periinterventional antibiotic prophylaxis? (multiple answers possible) [patients post VUR surgery]
N/A

Which patients receive periinterventional antibiotic prophylaxis? (multiple answers possible) [none]
N/A

Are there situations or clinical findings in the patient where you deviate from your prophylaxis standard (medication, dose, duration, age of the patient)? (multiple answers possible, please specify in answer box) [in case of already known vesicoureteral reflux (VUR)]
No

Are there situations or clinical findings in the patient where you deviate from your prophylaxis standard (medication, dose, duration, age of the patient)? (multiple answers possible, please specify in answer box) [in case of already known vesicoureteral reflux (VUR)][Comment]

Are there situations or clinical findings in the patient where you deviate from your prophylaxis standard (medication, dose, duration, age of the patient)? (multiple answers possible, please specify in answer box) [in patients with recurrent urinary tract infections (UTI)]
Yes

Are there situations or clinical findings in the patient where you deviate from your prophylaxis standard (medication, dose, duration, age of the patient)? (multiple answers possible, please specify in answer box) [in patients with recurrent urinary tract infections (UTI)][Comment]
Nach Durbruchsinfekten

Are there situations or clinical findings in the patient where you deviate from your prophylaxis standard (medication, dose, duration, age of the patient)? (multiple answers possible, please specify in answer box) [if multidrug-resistant organisms (MDRO) are detected in urine cultures]
Yes

Are there situations or clinical findings in the patient where you deviate from your prophylaxis standard (medication, dose, duration, age of the patient)? (multiple answers possible, please specify in answer box) [if multidrug-resistant organisms (MDRO) are detected in urine cultures][Comment]
Gelegentlich sogar Pip/Taz Meropenem I.v. bei Pseudomonas und Pyelonephritiden in der Vorgeschichte

Are there situations or clinical findings in the patient where you deviate from your prophylaxis standard (medication, dose, duration, age of the patient)? (multiple answers possible, please specify in answer box) [in newborns]
Yes

Are there situations or clinical findings in the patient where you deviate from your prophylaxis standard (medication, dose, duration, age of the patient)? (multiple answers possible, please specify in answer box) [in newborns][Comment]
Amoxi/Clav

Are there situations or clinical findings in the patient where you deviate from your prophylaxis standard (medication, dose, duration, age of the patient)? (multiple answers possible, please specify in answer box) [Other]

Are there situations or clinical findings in the patient where you deviate from your prophylaxis standard (medication, dose, duration, age of the patient)? (multiple answers possible, please specify in answer box) [Other comment]

Are there situations in which you use intravenous prophylaxis?
Yes

In which situations?
Vorgeschichte mit Problemkeimen Resitente Stämme E.coli/Klebsiellen oder Pseudomonas aeruginosa. Nach urologischen OPs oder Fremdplastik (SPK, Doppel J). Wird individuell im Team entschieden

Voiding analysis

Are there differences in antibiotic prophylaxis between VUS and VCUG in your department?
No

Is a non-pathological urine strip test required in preparation for VUS/VCUG in your department?
No

Is a non-pathological urine culture required in preparation for VUS/VCUG in your department?
No

Do patients only receive post-procedure prophylaxis if vesicoureteral reflux (VUR) is detected in VUS/VCUG?
No

Do other MUS/MCU findings (e.g. urethral valves) have an influence on post-procedure prophylaxis?
No

End of survey

Thank you for taking the time to answer this survey!

May we contact you personally if we have further questions?
Yes

E-mail address:
maximilian.hohenadel@ukbonn.de

Survey response 87

Response ID
89

Date submitted
2025-01-28 13:02:34

Last page
5

Start language
de

Seed
658479483

Date started
2025-01-28 12:55:17

Date last action
2025-01-28 13:02:34

Standard Operation Procedure

Who fills out this survey?
Pediatrician

Who fills out this survey? [Other]

In which type of hospital are you employed?
University Hospital

In which type of hospital are you employed? [Other]

In which country do you work?
Deutschland

Does your department have a defined protocol for infection prophylaxis in the context of VUS/VCUG?
No

Has this protocol been transformed into a written standard operating procedure (SOP)?
N/A

Antibiotic Prophylaxis

Are there situations/constellations in which you do not use antibiotic prophylaxis for VUS/VCUG?
Yes

Please specify these situations/constellations: [girl with first uncomplicated UTI without urinary tract dilation in ultrasound]
No

Please specify these situations/constellations: [boy with first uncomplicated UTI without urinary tract dilation in ultrasound]
No

Please specify these situations/constellations: [in patients with low grade urinary tract dilation (up to 2nd grade) and no UTI in medical history]
No

Please specify these situations/constellations: [Other]
wenn aktuell noch Antibiose aufgurnd der Pyelonephritis läuft

If you use antibiotic prophylaxis: which mediaction do you use? (multiple answers and subdivision into age groups possible) [Trimethoprim]
Yes

If you use antibiotic prophylaxis: which mediaction do you use? (multiple answers and subdivision into age groups possible) [Nitrofurantoin]
No

If you use antibiotic prophylaxis: which mediaction do you use? (multiple answers and subdivision into age groups possible) [Amoxicillin]
No

If you use antibiotic prophylaxis: which mediaction do you use? (multiple answers and subdivision into age groups possible) [Cefaclor]
Yes

If you use antibiotic prophylaxis: which mediaction do you use? (multiple answers and subdivision into age groups possible) [Amoxicillin/clavulanic acid]
No

If you use antibiotic prophylaxis: which mediaction do you use? (multiple answers and subdivision into age groups possible) [Other]
Yes

Trimethoprim: in what dosage?
prophylactic (2 mg/kg bodyweight/d)

Trimethoprim: in what dosage? [Other]

Trimethoprim: for which age group do you use trimethoprim?
ab 3 Monaten

Trimethoprim: which time schedule do you use? [Day(s) before VUS/VCUG, how many?]
No

Trimethoprim: which time schedule do you use? [Day(s) before VUS/VCUG, how many?][Comment]

Trimethoprim: which time schedule do you use? [Day of VUS/VCUG]
Yes

Trimethoprim: which time schedule do you use? [Day of VUS/VCUG][Comment]

Trimethoprim: which time schedule do you use? [Day(s) after VUS/VCUG, how many?]
Yes

Trimethoprim: which time schedule do you use? [Day(s) after VUS/VCUG, how many?][Comment]
1-2

Nitrofurantoin: which time schedule do you use? [Day(s) before VUS/VCUG, how many?]
N/A

Nitrofurantoin: which time schedule do you use? [Day of VUS/VCUG]
N/A

Nitrofurantoin: which time schedule do you use? [Day(s) after VUS/VCUG, how many?]
N/A

Amoxicillin: which time schedule do you use? [Day(s) before VUS/VCUG, how many?]
N/A

Amoxicillin: which time schedule do you use? [Day of VUS/VCUG]
N/A

Amoxicillin: which time schedule do you use? [Day(s) after VUS/VCUG, how many?]
N/A

Cefaclor: in what dosage?
prophylactic (10 mg/kg bodyweight/d)

Cefaclor: in what dosage? [Other]

Cefaclor: for which age group do you use cefaclor?
alle

Cefaclor: which time schedule do you use? [Days(s) before VUS/VCUG, how many?]
No

Cefaclor: which time schedule do you use? [Days(s) before VUS/VCUG, how many?][Comment]

Cefaclor: which time schedule do you use? [Day of VUS/VCUG]
Yes

Cefaclor: which time schedule do you use? [Day of VUS/VCUG][Comment]

Cefaclor: which time schedule do you use? [Day(s) after VUS/VCUG, how many?]
Yes

Cefaclor: which time schedule do you use? [Day(s) after VUS/VCUG, how many?][Comment]
1-2

Amoxicillin/clavulanic acid: in what dosage? (Amoxicillin portion) [prophylaktic (10-20 mg/Kg bodyweight/d)]
N/A

Amoxicillin/clavulanic acid: in what dosage? (Amoxicillin portion) [double prophylactic (20-40 mg/Kg bodyweight/d)]
N/A

Amoxicillin/clavulanic acid: in what dosage? (Amoxicillin portion) [therapeutic (80-100 mg/Kg bodyweight/d)]
N/A

Amoxicillin/clavulanic acid: which time schedule do you use? [Day(s) before VUS/VCUG]
N/A

Amoxicillin/clavulanic acid: which time schedule do you use? [Day of VUS/VCUG]
N/A

Amoxicillin/clavulanic acid: which time schedule do you use? [Day(s) after VUS/VCUG]
N/A

Other: which medication and in what dosage?
Resistogrammhgerecht nach dem Keim des letzten Infektes

Other: for which age group do you use this medication?
alle

Other: which time schedule do you use? [Day(s) before VUS/VCUG, how many?]
No

Other: which time schedule do you use? [Day(s) before VUS/VCUG, how many?][Comment]

Other: which time schedule do you use? [Day of VUS/VCUG]
Yes

Other: which time schedule do you use? [Day of VUS/VCUG][Comment]

Other: which time schedule do you use? [Day(s) after VUS/VCUG, how many?]
Yes

Other: which time schedule do you use? [Day(s) after VUS/VCUG, how many?][Comment]
1-2

Indication for prophylaxis

Which patients receive periinterventional antibiotic prophylaxis? (multiple answers possible) [all]
Yes

Which patients receive periinterventional antibiotic prophylaxis? (multiple answers possible) [patients post urinary tract infection/pyelonephritis/urosepsis]
N/A

Which patients receive periinterventional antibiotic prophylaxis? (multiple answers possible) [patients with urinary tract dilation in ultrasound]
N/A

Which patients receive periinterventional antibiotic prophylaxis? (multiple answers possible) [patients with prenatal urinary tract dilation]
N/A

Which patients receive periinterventional antibiotic prophylaxis? (multiple answers possible) [patients post VUR surgery]
N/A

Which patients receive periinterventional antibiotic prophylaxis? (multiple answers possible) [none]
N/A

Are there situations or clinical findings in the patient where you deviate from your prophylaxis standard (medication, dose, duration, age of the patient)? (multiple answers possible, please specify in answer box) [in case of already known vesicoureteral reflux (VUR)]
No

Are there situations or clinical findings in the patient where you deviate from your prophylaxis standard (medication, dose, duration, age of the patient)? (multiple answers possible, please specify in answer box) [in case of already known vesicoureteral reflux (VUR)][Comment]

Are there situations or clinical findings in the patient where you deviate from your prophylaxis standard (medication, dose, duration, age of the patient)? (multiple answers possible, please specify in answer box) [in patients with recurrent urinary tract infections (UTI)]
No

Are there situations or clinical findings in the patient where you deviate from your prophylaxis standard (medication, dose, duration, age of the patient)? (multiple answers possible, please specify in answer box) [in patients with recurrent urinary tract infections (UTI)][Comment]

Are there situations or clinical findings in the patient where you deviate from your prophylaxis standard (medication, dose, duration, age of the patient)? (multiple answers possible, please specify in answer box) [if multidrug-resistant organisms (MDRO) are detected in urine cultures]
Yes

Are there situations or clinical findings in the patient where you deviate from your prophylaxis standard (medication, dose, duration, age of the patient)? (multiple answers possible, please specify in answer box) [if multidrug-resistant organisms (MDRO) are detected in urine cultures][Comment]
resistogrammgerecht

Are there situations or clinical findings in the patient where you deviate from your prophylaxis standard (medication, dose, duration, age of the patient)? (multiple answers possible, please specify in answer box) [in newborns]
Yes

Are there situations or clinical findings in the patient where you deviate from your prophylaxis standard (medication, dose, duration, age of the patient)? (multiple answers possible, please specify in answer box) [in newborns][Comment]
bei Z.n. Infektion eher mal therapeutische Dosierung

Are there situations or clinical findings in the patient where you deviate from your prophylaxis standard (medication, dose, duration, age of the patient)? (multiple answers possible, please specify in answer box) [Other]
immundefizienz oder komplizi. Infekt

Are there situations or clinical findings in the patient where you deviate from your prophylaxis standard (medication, dose, duration, age of the patient)? (multiple answers possible, please specify in answer box) [Other comment]
eher therapeutische Dosis

Are there situations in which you use intravenous prophylaxis?
No

Voiding analysis

Are there differences in antibiotic prophylaxis between VUS and VCUG in your department?
No

Is a non-pathological urine strip test required in preparation for VUS/VCUG in your department?
No

Is a non-pathological urine culture required in preparation for VUS/VCUG in your department?
No

Do patients only receive post-procedure prophylaxis if vesicoureteral reflux (VUR) is detected in VUS/VCUG?
No

Do other MUS/MCU findings (e.g. urethral valves) have an influence on post-procedure prophylaxis?
Yes

Please specify:
eher therapeutische Dosis

End of survey

Thank you for taking the time to answer this survey!

May we contact you personally if we have further questions?
No

Survey response 88

Response ID
90

Date submitted
2025-01-28 13:17:02

Last page
5

Start language
de

Seed
213724876

Date started
2025-01-28 12:56:04

Date last action
2025-01-28 13:17:02

Standard Operation Procedure

Who fills out this survey?
Pediatrician

Who fills out this survey? [Other]

In which type of hospital are you employed?
University Hospital

In which type of hospital are you employed? [Other]

In which country do you work?
Deutschland

Does your department have a defined protocol for infection prophylaxis in the context of VUS/VCUG?
Yes

Has this protocol been transformed into a written standard operating procedure (SOP)?
Yes

Which department is the author of the SOP for infection prophylaxis in VUS/VCUG?
Pediatric nephrology

Which department is the author of the SOP for infection prophylaxis in VUS/VCUG? [Other]

Antibiotic Prophylaxis

Are there situations/constellations in which you do not use antibiotic prophylaxis for VUS/VCUG?
No

Please specify these situations/constellations: [girl with first uncomplicated UTI without urinary tract dilation in ultrasound]
N/A

Please specify these situations/constellations: [boy with first uncomplicated UTI without urinary tract dilation in ultrasound]
N/A

Please specify these situations/constellations: [in patients with low grade urinary tract dilation (up to 2nd grade) and no UTI in medical history]
N/A

If you use antibiotic prophylaxis: which mediaction do you use? (multiple answers and subdivision into age groups possible) [Trimethoprim]
Yes

If you use antibiotic prophylaxis: which mediaction do you use? (multiple answers and subdivision into age groups possible) [Nitrofurantoin]
Yes

If you use antibiotic prophylaxis: which mediaction do you use? (multiple answers and subdivision into age groups possible) [Amoxicillin]
No

If you use antibiotic prophylaxis: which mediaction do you use? (multiple answers and subdivision into age groups possible) [Cefaclor]
Yes

If you use antibiotic prophylaxis: which mediaction do you use? (multiple answers and subdivision into age groups possible) [Amoxicillin/clavulanic acid]
No

If you use antibiotic prophylaxis: which mediaction do you use? (multiple answers and subdivision into age groups possible) [Other]
Yes

Trimethoprim: in what dosage?
prophylactic (2 mg/kg bodyweight/d)

Trimethoprim: in what dosage? [Other]

Trimethoprim: for which age group do you use trimethoprim?
ab 6 Wochen

Trimethoprim: which time schedule do you use? [Day(s) before VUS/VCUG, how many?]
No

Trimethoprim: which time schedule do you use? [Day(s) before VUS/VCUG, how many?][Comment]

Trimethoprim: which time schedule do you use? [Day of VUS/VCUG]
Yes

Trimethoprim: which time schedule do you use? [Day of VUS/VCUG][Comment]

Trimethoprim: which time schedule do you use? [Day(s) after VUS/VCUG, how many?]
Yes

Trimethoprim: which time schedule do you use? [Day(s) after VUS/VCUG, how many?][Comment]
2

Nitrofurantoin: in what dosage?
prophylactic (1-2 mg/kg bodyweight/d)

Nitrofurantoin: in what dosage? [Other]

Nitrofurantoin: for which age group do you use nitrofurantoin?
ab 3 Monaten

Nitrofurantoin: which time schedule do you use? [Day(s) before VUS/VCUG, how many?]
No

Nitrofurantoin: which time schedule do you use? [Day(s) before VUS/VCUG, how many?][Comment]

Nitrofurantoin: which time schedule do you use? [Day of VUS/VCUG]
Yes

Nitrofurantoin: which time schedule do you use? [Day of VUS/VCUG][Comment]

Nitrofurantoin: which time schedule do you use? [Day(s) after VUS/VCUG, how many?]
Yes

Nitrofurantoin: which time schedule do you use? [Day(s) after VUS/VCUG, how many?][Comment]
2

Amoxicillin: which time schedule do you use? [Day(s) before VUS/VCUG, how many?]
N/A

Amoxicillin: which time schedule do you use? [Day of VUS/VCUG]
N/A

Amoxicillin: which time schedule do you use? [Day(s) after VUS/VCUG, how many?]
N/A

Cefaclor: in what dosage?
prophylactic (10 mg/kg bodyweight/d)

Cefaclor: in what dosage? [Other]

Cefaclor: for which age group do you use cefaclor?
0-6 Wochen

Cefaclor: which time schedule do you use? [Days(s) before VUS/VCUG, how many?]
No

Cefaclor: which time schedule do you use? [Days(s) before VUS/VCUG, how many?][Comment]

Cefaclor: which time schedule do you use? [Day of VUS/VCUG]
Yes

Cefaclor: which time schedule do you use? [Day of VUS/VCUG][Comment]

Cefaclor: which time schedule do you use? [Day(s) after VUS/VCUG, how many?]
Yes

Cefaclor: which time schedule do you use? [Day(s) after VUS/VCUG, how many?][Comment]
2

Amoxicillin/clavulanic acid: in what dosage? (Amoxicillin portion) [prophylaktic (10-20 mg/Kg bodyweight/d)]
N/A

Amoxicillin/clavulanic acid: in what dosage? (Amoxicillin portion) [double prophylactic (20-40 mg/Kg bodyweight/d)]
N/A

Amoxicillin/clavulanic acid: in what dosage? (Amoxicillin portion) [therapeutic (80-100 mg/Kg bodyweight/d)]
N/A

Amoxicillin/clavulanic acid: which time schedule do you use? [Day(s) before VUS/VCUG]
N/A

Amoxicillin/clavulanic acid: which time schedule do you use? [Day of VUS/VCUG]
N/A

Amoxicillin/clavulanic acid: which time schedule do you use? [Day(s) after VUS/VCUG]
N/A

Other: which medication and in what dosage?
Cefpodoxim, Dosis 1x2-3 mg/kg/d

Other: for which age group do you use this medication?
0-6 Wochen

Other: which time schedule do you use? [Day(s) before VUS/VCUG, how many?]
No

Other: which time schedule do you use? [Day(s) before VUS/VCUG, how many?][Comment]

Other: which time schedule do you use? [Day of VUS/VCUG]
Yes

Other: which time schedule do you use? [Day of VUS/VCUG][Comment]

Other: which time schedule do you use? [Day(s) after VUS/VCUG, how many?]
Yes

Other: which time schedule do you use? [Day(s) after VUS/VCUG, how many?][Comment]
2

Indication for prophylaxis

Which patients receive periinterventional antibiotic prophylaxis? (multiple answers possible) [all]
No

Which patients receive periinterventional antibiotic prophylaxis? (multiple answers possible) [patients post urinary tract infection/pyelonephritis/urosepsis]
Yes

Which patients receive periinterventional antibiotic prophylaxis? (multiple answers possible) [patients with urinary tract dilation in ultrasound]
No

Which patients receive periinterventional antibiotic prophylaxis? (multiple answers possible) [patients with prenatal urinary tract dilation]
No

Which patients receive periinterventional antibiotic prophylaxis? (multiple answers possible) [patients post VUR surgery]
No

Which patients receive periinterventional antibiotic prophylaxis? (multiple answers possible) [none]
No

Which patients receive periinterventional antibiotic prophylaxis? (multiple answers possible) [Other]

patients status post urinary tract infection/pyelonephritis/urosepsis: what is the minimum time between infection and VUS/VCUG? (in days)
medizinisch: nur wenige Tage bis zum Ansprechen auf die Antibiotika-Therapie; ökonomisch: 15 d nach Entlassung von Station zum Abrechnen der Rö-MCU

Are there situations or clinical findings in the patient where you deviate from your prophylaxis standard (medication, dose, duration, age of the patient)? (multiple answers possible, please specify in answer box) [in case of already known vesicoureteral reflux (VUR)]
No

Are there situations or clinical findings in the patient where you deviate from your prophylaxis standard (medication, dose, duration, age of the patient)? (multiple answers possible, please specify in answer box) [in case of already known vesicoureteral reflux (VUR)][Comment]

Are there situations or clinical findings in the patient where you deviate from your prophylaxis standard (medication, dose, duration, age of the patient)? (multiple answers possible, please specify in answer box) [in patients with recurrent urinary tract infections (UTI)]
No

Are there situations or clinical findings in the patient where you deviate from your prophylaxis standard (medication, dose, duration, age of the patient)? (multiple answers possible, please specify in answer box) [in patients with recurrent urinary tract infections (UTI)][Comment]

Are there situations or clinical findings in the patient where you deviate from your prophylaxis standard (medication, dose, duration, age of the patient)? (multiple answers possible, please specify in answer box) [if multidrug-resistant organisms (MDRO) are detected in urine cultures]
Yes

Are there situations or clinical findings in the patient where you deviate from your prophylaxis standard (medication, dose, duration, age of the patient)? (multiple answers possible, please specify in answer box) [if multidrug-resistant organisms (MDRO) are detected in urine cultures][Comment]

Are there situations or clinical findings in the patient where you deviate from your prophylaxis standard (medication, dose, duration, age of the patient)? (multiple answers possible, please specify in answer box) [in newborns]
No

Are there situations or clinical findings in the patient where you deviate from your prophylaxis standard (medication, dose, duration, age of the patient)? (multiple answers possible, please specify in answer box) [in newborns][Comment]

Are there situations or clinical findings in the patient where you deviate from your prophylaxis standard (medication, dose, duration, age of the patient)? (multiple answers possible, please specify in answer box) [Other]

Are there situations or clinical findings in the patient where you deviate from your prophylaxis standard (medication, dose, duration, age of the patient)? (multiple answers possible, please specify in answer box) [Other comment]

Are there situations in which you use intravenous prophylaxis?
No

Voiding analysis

Are there differences in antibiotic prophylaxis between VUS and VCUG in your department?
No

Is a non-pathological urine strip test required in preparation for VUS/VCUG in your department?
Yes

What is the maximum amount of days accepted for urine strip test to be performed prior to the exam?
ist am gleichen Tag

Is a non-pathological urine culture required in preparation for VUS/VCUG in your department?
No

Do patients only receive post-procedure prophylaxis if vesicoureteral reflux (VUR) is detected in VUS/VCUG?
No

Do other MUS/MCU findings (e.g. urethral valves) have an influence on post-procedure prophylaxis?
No

End of survey

Thank you for taking the time to answer this survey!

May we contact you personally if we have further questions?
No

Survey response 89

Response ID
91

Date submitted
2025-01-28 13:09:26

Last page
5

Start language
de

Seed
1979863977

Date started
2025-01-28 13:00:27

Date last action
2025-01-28 13:09:26

Standard Operation Procedure

Who fills out this survey?
Other

Who fills out this survey? [Other]
Kindernephrolog:in

In which type of hospital are you employed?
University Hospital

In which type of hospital are you employed? [Other]

In which country do you work?
Deutschland

Does your department have a defined protocol for infection prophylaxis in the context of VUS/VCUG?
Yes

Has this protocol been transformed into a written standard operating procedure (SOP)?
No

Antibiotic Prophylaxis

Are there situations/constellations in which you do not use antibiotic prophylaxis for VUS/VCUG?
No

Please specify these situations/constellations: [girl with first uncomplicated UTI without urinary tract dilation in ultrasound]
N/A

Please specify these situations/constellations: [boy with first uncomplicated UTI without urinary tract dilation in ultrasound]
N/A

Please specify these situations/constellations: [in patients with low grade urinary tract dilation (up to 2nd grade) and no UTI in medical history]
N/A

If you use antibiotic prophylaxis: which mediaction do you use? (multiple answers and subdivision into age groups possible) [Trimethoprim]
Yes

If you use antibiotic prophylaxis: which mediaction do you use? (multiple answers and subdivision into age groups possible) [Nitrofurantoin]
Yes

If you use antibiotic prophylaxis: which mediaction do you use? (multiple answers and subdivision into age groups possible) [Amoxicillin]
Yes

If you use antibiotic prophylaxis: which mediaction do you use? (multiple answers and subdivision into age groups possible) [Cefaclor]
Yes

If you use antibiotic prophylaxis: which mediaction do you use? (multiple answers and subdivision into age groups possible) [Amoxicillin/clavulanic acid]
Yes

If you use antibiotic prophylaxis: which mediaction do you use? (multiple answers and subdivision into age groups possible) [Other]
No

Trimethoprim: in what dosage?
therapeutic (6 mg/kg bodyweight/d)

Trimethoprim: in what dosage? [Other]

Trimethoprim: for which age group do you use trimethoprim?
Alle über 6 Wochen

Trimethoprim: which time schedule do you use? [Day(s) before VUS/VCUG, how many?]
Yes

Trimethoprim: which time schedule do you use? [Day(s) before VUS/VCUG, how many?][Comment]
1

Trimethoprim: which time schedule do you use? [Day of VUS/VCUG]
Yes

Trimethoprim: which time schedule do you use? [Day of VUS/VCUG][Comment]

Trimethoprim: which time schedule do you use? [Day(s) after VUS/VCUG, how many?]
Yes

Trimethoprim: which time schedule do you use? [Day(s) after VUS/VCUG, how many?][Comment]
1

Nitrofurantoin: in what dosage?
therapeutic (5 mg/kg bodyweight/d)

Nitrofurantoin: in what dosage? [Other]

Nitrofurantoin: for which age group do you use nitrofurantoin?
Alle über 1/2 Jahr

Nitrofurantoin: which time schedule do you use? [Day(s) before VUS/VCUG, how many?]
Yes

Nitrofurantoin: which time schedule do you use? [Day(s) before VUS/VCUG, how many?][Comment]
1

Nitrofurantoin: which time schedule do you use? [Day of VUS/VCUG]
Yes

Nitrofurantoin: which time schedule do you use? [Day of VUS/VCUG][Comment]

Nitrofurantoin: which time schedule do you use? [Day(s) after VUS/VCUG, how many?]
Yes

Nitrofurantoin: which time schedule do you use? [Day(s) after VUS/VCUG, how many?][Comment]
1

Amoxicillin: in what dosage?
therapeutic (50-100 mg/kg bodyweight/d)

Amoxicillin: in what dosage? [Other]

Amoxicillin: for which age group do you use amoxicillin?
Alle

Amoxicillin: which time schedule do you use? [Day(s) before VUS/VCUG, how many?]
Yes

Amoxicillin: which time schedule do you use? [Day(s) before VUS/VCUG, how many?][Comment]
1

Amoxicillin: which time schedule do you use? [Day of VUS/VCUG]
Yes

Amoxicillin: which time schedule do you use? [Day of VUS/VCUG][Comment]

Amoxicillin: which time schedule do you use? [Day(s) after VUS/VCUG, how many?]
Yes

Amoxicillin: which time schedule do you use? [Day(s) after VUS/VCUG, how many?][Comment]
1

Cefaclor: in what dosage?
therapeutic (40 mg/kg bodyweight/d)

Cefaclor: in what dosage? [Other]

Cefaclor: for which age group do you use cefaclor?
Alle

Cefaclor: which time schedule do you use? [Days(s) before VUS/VCUG, how many?]
Yes

Cefaclor: which time schedule do you use? [Days(s) before VUS/VCUG, how many?][Comment]
1

Cefaclor: which time schedule do you use? [Day of VUS/VCUG]
Yes

Cefaclor: which time schedule do you use? [Day of VUS/VCUG][Comment]

Cefaclor: which time schedule do you use? [Day(s) after VUS/VCUG, how many?]
Yes

Cefaclor: which time schedule do you use? [Day(s) after VUS/VCUG, how many?][Comment]
1

Amoxicillin/clavulanic acid: in what dosage? (Amoxicillin portion) [prophylaktic (10-20 mg/Kg bodyweight/d)]
No

Amoxicillin/clavulanic acid: in what dosage? (Amoxicillin portion) [double prophylactic (20-40 mg/Kg bodyweight/d)]
No

Amoxicillin/clavulanic acid: in what dosage? (Amoxicillin portion) [therapeutic (80-100 mg/Kg bodyweight/d)]
Yes

Amoxicillin/clavulanic acid: in what dosage? (Amoxicillin portion) [Other]

Amoxicillin/clavulanic acid: for which age group do you use amoxicillin/clavulanic acid?
Alle

Amoxicillin/clavulanic acid: which time schedule do you use? [Day(s) before VUS/VCUG]
Yes

Amoxicillin/clavulanic acid: which time schedule do you use? [Day(s) before VUS/VCUG][Comment]
1

Amoxicillin/clavulanic acid: which time schedule do you use? [Day of VUS/VCUG]
Yes

Amoxicillin/clavulanic acid: which time schedule do you use? [Day of VUS/VCUG][Comment]

Amoxicillin/clavulanic acid: which time schedule do you use? [Day(s) after VUS/VCUG]
Yes

Amoxicillin/clavulanic acid: which time schedule do you use? [Day(s) after VUS/VCUG][Comment]
1

Other: which time schedule do you use? [Day(s) before VUS/VCUG, how many?]
N/A

Other: which time schedule do you use? [Day of VUS/VCUG]
N/A

Other: which time schedule do you use? [Day(s) after VUS/VCUG, how many?]
N/A

Indication for prophylaxis

Which patients receive periinterventional antibiotic prophylaxis? (multiple answers possible) [all]
Yes

Which patients receive periinterventional antibiotic prophylaxis? (multiple answers possible) [patients post urinary tract infection/pyelonephritis/urosepsis]
N/A

Which patients receive periinterventional antibiotic prophylaxis? (multiple answers possible) [patients with urinary tract dilation in ultrasound]
N/A

Which patients receive periinterventional antibiotic prophylaxis? (multiple answers possible) [patients with prenatal urinary tract dilation]
N/A

Which patients receive periinterventional antibiotic prophylaxis? (multiple answers possible) [patients post VUR surgery]
N/A

Which patients receive periinterventional antibiotic prophylaxis? (multiple answers possible) [none]
N/A

Are there situations or clinical findings in the patient where you deviate from your prophylaxis standard (medication, dose, duration, age of the patient)? (multiple answers possible, please specify in answer box) [in case of already known vesicoureteral reflux (VUR)]
No

Are there situations or clinical findings in the patient where you deviate from your prophylaxis standard (medication, dose, duration, age of the patient)? (multiple answers possible, please specify in answer box) [in case of already known vesicoureteral reflux (VUR)][Comment]

Are there situations or clinical findings in the patient where you deviate from your prophylaxis standard (medication, dose, duration, age of the patient)? (multiple answers possible, please specify in answer box) [in patients with recurrent urinary tract infections (UTI)]
No

Are there situations or clinical findings in the patient where you deviate from your prophylaxis standard (medication, dose, duration, age of the patient)? (multiple answers possible, please specify in answer box) [in patients with recurrent urinary tract infections (UTI)][Comment]

Are there situations or clinical findings in the patient where you deviate from your prophylaxis standard (medication, dose, duration, age of the patient)? (multiple answers possible, please specify in answer box) [if multidrug-resistant organisms (MDRO) are detected in urine cultures]
No

Are there situations or clinical findings in the patient where you deviate from your prophylaxis standard (medication, dose, duration, age of the patient)? (multiple answers possible, please specify in answer box) [if multidrug-resistant organisms (MDRO) are detected in urine cultures][Comment]

Are there situations or clinical findings in the patient where you deviate from your prophylaxis standard (medication, dose, duration, age of the patient)? (multiple answers possible, please specify in answer box) [in newborns]
No

Are there situations or clinical findings in the patient where you deviate from your prophylaxis standard (medication, dose, duration, age of the patient)? (multiple answers possible, please specify in answer box) [in newborns][Comment]

Are there situations or clinical findings in the patient where you deviate from your prophylaxis standard (medication, dose, duration, age of the patient)? (multiple answers possible, please specify in answer box) [Other]
Prophylaxe

Are there situations or clinical findings in the patient where you deviate from your prophylaxis standard (medication, dose, duration, age of the patient)? (multiple answers possible, please specify in answer box) [Other comment]
Bei bereits bestehender antibakterieller Prophylaxe wird diese oft fortgeführt und für die Untersuchung nicht intensiviert.

Are there situations in which you use intravenous prophylaxis?
No

Voiding analysis

Are there differences in antibiotic prophylaxis between VUS and VCUG in your department?
No

Is a non-pathological urine strip test required in preparation for VUS/VCUG in your department?
Yes

What is the maximum amount of days accepted for urine strip test to be performed prior to the exam?
1 Tag

Is a non-pathological urine culture required in preparation for VUS/VCUG in your department?
No

Do patients only receive post-procedure prophylaxis if vesicoureteral reflux (VUR) is detected in VUS/VCUG?
No

Do other MUS/MCU findings (e.g. urethral valves) have an influence on post-procedure prophylaxis?
No

End of survey

Thank you for taking the time to answer this survey!

May we contact you personally if we have further questions?
No

Survey response 90

Response ID
92

Date submitted
2025-01-28 13:29:02

Last page
5

Start language
de

Seed
177101419

Date started
2025-01-28 13:07:40

Date last action
2025-01-28 13:29:02

Standard Operation Procedure

Who fills out this survey?
Pediatrician

Who fills out this survey? [Other]

In which type of hospital are you employed?
Community hospital (>50 beds for pediatric patients)

In which type of hospital are you employed? [Other]

In which country do you work?
Sachsen

Does your department have a defined protocol for infection prophylaxis in the context of VUS/VCUG?
Yes

Has this protocol been transformed into a written standard operating procedure (SOP)?
Yes

Which department is the author of the SOP for infection prophylaxis in VUS/VCUG?
Pediatric nephrology

Which department is the author of the SOP for infection prophylaxis in VUS/VCUG? [Other]

Antibiotic Prophylaxis

Are there situations/constellations in which you do not use antibiotic prophylaxis for VUS/VCUG?
Yes

Please specify these situations/constellations: [girl with first uncomplicated UTI without urinary tract dilation in ultrasound]
No

Please specify these situations/constellations: [boy with first uncomplicated UTI without urinary tract dilation in ultrasound]
No

Please specify these situations/constellations: [in patients with low grade urinary tract dilation (up to 2nd grade) and no UTI in medical history]
Yes

Please specify these situations/constellations: [Other]
wenn Patient ohne Prophylaxe zur MCU kommt wird vorher keine angesetzt (laufende werden fortgeführt), d.h. MCU ist keine eigenständige Indikation für neue Prophylaxe

If you use antibiotic prophylaxis: which mediaction do you use? (multiple answers and subdivision into age groups possible) [Trimethoprim]
Yes

If you use antibiotic prophylaxis: which mediaction do you use? (multiple answers and subdivision into age groups possible) [Nitrofurantoin]
Yes

If you use antibiotic prophylaxis: which mediaction do you use? (multiple answers and subdivision into age groups possible) [Amoxicillin]
No

If you use antibiotic prophylaxis: which mediaction do you use? (multiple answers and subdivision into age groups possible) [Cefaclor]
Yes

If you use antibiotic prophylaxis: which mediaction do you use? (multiple answers and subdivision into age groups possible) [Amoxicillin/clavulanic acid]
No

If you use antibiotic prophylaxis: which mediaction do you use? (multiple answers and subdivision into age groups possible) [Other]
No

Trimethoprim: in what dosage?
prophylactic (2 mg/kg bodyweight/d)

Trimethoprim: in what dosage? [Other]

Trimethoprim: for which age group do you use trimethoprim?
alle Säuglinge ab 6 Wochen + Kleinkinder

Trimethoprim: which time schedule do you use? [Day(s) before VUS/VCUG, how many?]
Yes

Trimethoprim: which time schedule do you use? [Day(s) before VUS/VCUG, how many?][Comment]
unveränderte Fortführung wenn Prophylaxe läuft; wenn keine läuft wird keine angesetzt

Trimethoprim: which time schedule do you use? [Day of VUS/VCUG]
No

Trimethoprim: which time schedule do you use? [Day of VUS/VCUG][Comment]

Trimethoprim: which time schedule do you use? [Day(s) after VUS/VCUG, how many?]
Yes

Trimethoprim: which time schedule do you use? [Day(s) after VUS/VCUG, how many?][Comment]
VUR >=3° therapeutisch Dosis für 3d (da einzige Risikokonstellation) ; sonst unveränderte Fortführung der vorbestehenden Therapie,

Nitrofurantoin: in what dosage?
prophylactic (1-2 mg/kg bodyweight/d)

Nitrofurantoin: in what dosage? [Other]

Nitrofurantoin: for which age group do you use nitrofurantoin?
eher Schulkinder, Kleinkinder wenn Durchbruchsinfektionen unter TMP

Nitrofurantoin: which time schedule do you use? [Day(s) before VUS/VCUG, how many?]
Yes

Nitrofurantoin: which time schedule do you use? [Day(s) before VUS/VCUG, how many?][Comment]
unveränderte Fortführung wenn Prophylaxe läuft; wenn keine läuft wird keine angesetzt

Nitrofurantoin: which time schedule do you use? [Day of VUS/VCUG]
No

Nitrofurantoin: which time schedule do you use? [Day of VUS/VCUG][Comment]

Nitrofurantoin: which time schedule do you use? [Day(s) after VUS/VCUG, how many?]
Yes

Nitrofurantoin: which time schedule do you use? [Day(s) after VUS/VCUG, how many?][Comment]
VUR >=3° therapeutisch Dosis für 3d (da einzige Risikokonstellation) ; sonst unveränderte Fortführung der vorbestehenden Therapie,

Amoxicillin: which time schedule do you use? [Day(s) before VUS/VCUG, how many?]
N/A

Amoxicillin: which time schedule do you use? [Day of VUS/VCUG]
N/A

Amoxicillin: which time schedule do you use? [Day(s) after VUS/VCUG, how many?]
N/A

Cefaclor: in what dosage?
prophylactic (10 mg/kg bodyweight/d)

Cefaclor: in what dosage? [Other]

Cefaclor: for which age group do you use cefaclor?
Säuglinge

Cefaclor: which time schedule do you use? [Days(s) before VUS/VCUG, how many?]
Yes

Cefaclor: which time schedule do you use? [Days(s) before VUS/VCUG, how many?][Comment]
unveränderte Fortführung wenn Prophylaxe läuft; wenn keine läuft wird keine angesetzt

Cefaclor: which time schedule do you use? [Day of VUS/VCUG]
No

Cefaclor: which time schedule do you use? [Day of VUS/VCUG][Comment]

Cefaclor: which time schedule do you use? [Day(s) after VUS/VCUG, how many?]
Yes

Cefaclor: which time schedule do you use? [Day(s) after VUS/VCUG, how many?][Comment]
VUR >=3° therapeutisch Dosis für 3d (da einzige Risikokonstellation) ; sonst unveränderte Fortführung der vorbestehenden Therapie,

Amoxicillin/clavulanic acid: in what dosage? (Amoxicillin portion) [prophylaktic (10-20 mg/Kg bodyweight/d)]
N/A

Amoxicillin/clavulanic acid: in what dosage? (Amoxicillin portion) [double prophylactic (20-40 mg/Kg bodyweight/d)]
N/A

Amoxicillin/clavulanic acid: in what dosage? (Amoxicillin portion) [therapeutic (80-100 mg/Kg bodyweight/d)]
N/A

Amoxicillin/clavulanic acid: which time schedule do you use? [Day(s) before VUS/VCUG]
N/A

Amoxicillin/clavulanic acid: which time schedule do you use? [Day of VUS/VCUG]
N/A

Amoxicillin/clavulanic acid: which time schedule do you use? [Day(s) after VUS/VCUG]
N/A

Other: which time schedule do you use? [Day(s) before VUS/VCUG, how many?]
N/A

Other: which time schedule do you use? [Day of VUS/VCUG]
N/A

Other: which time schedule do you use? [Day(s) after VUS/VCUG, how many?]
N/A

Indication for prophylaxis

Which patients receive periinterventional antibiotic prophylaxis? (multiple answers possible) [all]
No

Which patients receive periinterventional antibiotic prophylaxis? (multiple answers possible) [patients post urinary tract infection/pyelonephritis/urosepsis]
Yes

Which patients receive periinterventional antibiotic prophylaxis? (multiple answers possible) [patients with urinary tract dilation in ultrasound]
Yes

Which patients receive periinterventional antibiotic prophylaxis? (multiple answers possible) [patients with prenatal urinary tract dilation]
No

Which patients receive periinterventional antibiotic prophylaxis? (multiple answers possible) [patients post VUR surgery]
No

Which patients receive periinterventional antibiotic prophylaxis? (multiple answers possible) [none]
No

Which patients receive periinterventional antibiotic prophylaxis? (multiple answers possible) [Other]

patients status post urinary tract infection/pyelonephritis/urosepsis: what is the minimum time between infection and VUS/VCUG? (in days)
14

In patients with urinary tract dilation in ultrasound: from what degree on? Image from Beetz R, Bokenkamp A, Brandis M et al (2001) Diagnosis of congenital dilatation of the urinary tract. Consensus Group of the Pediatric Nephrology Working Society in cooperation with the Pediatric Urology Working Group of the German Society of Urology and with the Pediatric Urology Working Society in the Germany Society of Pediatric Surgery. Urologe A 40:495–507 (quiz 8–9)
grade 3

Are there situations or clinical findings in the patient where you deviate from your prophylaxis standard (medication, dose, duration, age of the patient)? (multiple answers possible, please specify in answer box) [in case of already known vesicoureteral reflux (VUR)]
No

Are there situations or clinical findings in the patient where you deviate from your prophylaxis standard (medication, dose, duration, age of the patient)? (multiple answers possible, please specify in answer box) [in case of already known vesicoureteral reflux (VUR)][Comment]

Are there situations or clinical findings in the patient where you deviate from your prophylaxis standard (medication, dose, duration, age of the patient)? (multiple answers possible, please specify in answer box) [in patients with recurrent urinary tract infections (UTI)]
No

Are there situations or clinical findings in the patient where you deviate from your prophylaxis standard (medication, dose, duration, age of the patient)? (multiple answers possible, please specify in answer box) [in patients with recurrent urinary tract infections (UTI)][Comment]

Are there situations or clinical findings in the patient where you deviate from your prophylaxis standard (medication, dose, duration, age of the patient)? (multiple answers possible, please specify in answer box) [if multidrug-resistant organisms (MDRO) are detected in urine cultures]
Yes

Are there situations or clinical findings in the patient where you deviate from your prophylaxis standard (medication, dose, duration, age of the patient)? (multiple answers possible, please specify in answer box) [if multidrug-resistant organisms (MDRO) are detected in urine cultures][Comment]
nur wenn aktuell im Katheterurin nachweisbar

Are there situations or clinical findings in the patient where you deviate from your prophylaxis standard (medication, dose, duration, age of the patient)? (multiple answers possible, please specify in answer box) [in newborns]
No

Are there situations or clinical findings in the patient where you deviate from your prophylaxis standard (medication, dose, duration, age of the patient)? (multiple answers possible, please specify in answer box) [in newborns][Comment]

Are there situations or clinical findings in the patient where you deviate from your prophylaxis standard (medication, dose, duration, age of the patient)? (multiple answers possible, please specify in answer box) [Other]

Are there situations or clinical findings in the patient where you deviate from your prophylaxis standard (medication, dose, duration, age of the patient)? (multiple answers possible, please specify in answer box) [Other comment]

Are there situations in which you use intravenous prophylaxis?
No

Voiding analysis

Are there differences in antibiotic prophylaxis between VUS and VCUG in your department?
No

Is a non-pathological urine strip test required in preparation for VUS/VCUG in your department?
Yes

What is the maximum amount of days accepted for urine strip test to be performed prior to the exam?
5

Is a non-pathological urine culture required in preparation for VUS/VCUG in your department?
No

Do patients only receive post-procedure prophylaxis if vesicoureteral reflux (VUR) is detected in VUS/VCUG?
Yes

Please explain your approach:
- Patient mit PN/Urosepsis (inbs. männlich) --> MCU emfohlen --> Prophylaxe bis zur MCU (da Reflux nicht ausgeschlossen) egal ob HTS oder nicht; (wenn Eltern keine MCU wollen dann keine Prophylaxe bzw. Individualentscheidung)<br>- Voruntersuchung für MCU: Urinstix --> wenn Leukos pos. (im Zweifel K-Urin) --> Mibi --> wenn pos. Therapie / wenn neg. MCU<br>- laufende Prophylaxe wird für MCU unverändert fortgesetzt (wenn Patient keine hatte z.B. Überweisung von Auswärts wird keine angesetzt) --> wenn VUR =3° 3d therapeutische Dosis, danach Prophylaxedosis

Do other MUS/MCU findings (e.g. urethral valves) have an influence on post-procedure prophylaxis?
No

End of survey

Thank you for taking the time to answer this survey!

May we contact you personally if we have further questions?
Yes

E-mail address:
tillmann.wallborn@kfh.de

Survey response 91

Response ID
93

Date submitted
2025-01-28 13:20:35

Last page
5

Start language
de

Seed
1696128466

Date started
2025-01-28 13:16:04

Date last action
2025-01-28 13:20:35

Standard Operation Procedure

Who fills out this survey?
Pediatrician

Who fills out this survey? [Other]

In which type of hospital are you employed?
University Hospital

In which type of hospital are you employed? [Other]

In which country do you work?
Deutschland

Does your department have a defined protocol for infection prophylaxis in the context of VUS/VCUG?
Yes

Has this protocol been transformed into a written standard operating procedure (SOP)?
No

Antibiotic Prophylaxis

Are there situations/constellations in which you do not use antibiotic prophylaxis for VUS/VCUG?
No

Please specify these situations/constellations: [girl with first uncomplicated UTI without urinary tract dilation in ultrasound]
N/A

Please specify these situations/constellations: [boy with first uncomplicated UTI without urinary tract dilation in ultrasound]
N/A

Please specify these situations/constellations: [in patients with low grade urinary tract dilation (up to 2nd grade) and no UTI in medical history]
N/A

If you use antibiotic prophylaxis: which mediaction do you use? (multiple answers and subdivision into age groups possible) [Trimethoprim]
No

If you use antibiotic prophylaxis: which mediaction do you use? (multiple answers and subdivision into age groups possible) [Nitrofurantoin]
No

If you use antibiotic prophylaxis: which mediaction do you use? (multiple answers and subdivision into age groups possible) [Amoxicillin]
No

If you use antibiotic prophylaxis: which mediaction do you use? (multiple answers and subdivision into age groups possible) [Cefaclor]
No

If you use antibiotic prophylaxis: which mediaction do you use? (multiple answers and subdivision into age groups possible) [Amoxicillin/clavulanic acid]
Yes

If you use antibiotic prophylaxis: which mediaction do you use? (multiple answers and subdivision into age groups possible) [Other]
Yes

Trimethoprim: which time schedule do you use? [Day(s) before VUS/VCUG, how many?]
N/A

Trimethoprim: which time schedule do you use? [Day of VUS/VCUG]
N/A

Trimethoprim: which time schedule do you use? [Day(s) after VUS/VCUG, how many?]
N/A

Nitrofurantoin: which time schedule do you use? [Day(s) before VUS/VCUG, how many?]
N/A

Nitrofurantoin: which time schedule do you use? [Day of VUS/VCUG]
N/A

Nitrofurantoin: which time schedule do you use? [Day(s) after VUS/VCUG, how many?]
N/A

Amoxicillin: which time schedule do you use? [Day(s) before VUS/VCUG, how many?]
N/A

Amoxicillin: which time schedule do you use? [Day of VUS/VCUG]
N/A

Amoxicillin: which time schedule do you use? [Day(s) after VUS/VCUG, how many?]
N/A

Cefaclor: which time schedule do you use? [Days(s) before VUS/VCUG, how many?]
N/A

Cefaclor: which time schedule do you use? [Day of VUS/VCUG]
N/A

Cefaclor: which time schedule do you use? [Day(s) after VUS/VCUG, how many?]
N/A

Amoxicillin/clavulanic acid: in what dosage? (Amoxicillin portion) [prophylaktic (10-20 mg/Kg bodyweight/d)]
No

Amoxicillin/clavulanic acid: in what dosage? (Amoxicillin portion) [double prophylactic (20-40 mg/Kg bodyweight/d)]
No

Amoxicillin/clavulanic acid: in what dosage? (Amoxicillin portion) [therapeutic (80-100 mg/Kg bodyweight/d)]
Yes

Amoxicillin/clavulanic acid: in what dosage? (Amoxicillin portion) [Other]

Amoxicillin/clavulanic acid: for which age group do you use amoxicillin/clavulanic acid?
ab 4 wochen

Amoxicillin/clavulanic acid: which time schedule do you use? [Day(s) before VUS/VCUG]
No

Amoxicillin/clavulanic acid: which time schedule do you use? [Day(s) before VUS/VCUG][Comment]

Amoxicillin/clavulanic acid: which time schedule do you use? [Day of VUS/VCUG]
Yes

Amoxicillin/clavulanic acid: which time schedule do you use? [Day of VUS/VCUG][Comment]

Amoxicillin/clavulanic acid: which time schedule do you use? [Day(s) after VUS/VCUG]
Yes

Amoxicillin/clavulanic acid: which time schedule do you use? [Day(s) after VUS/VCUG][Comment]
2

Other: which medication and in what dosage?
cotrim 3 tage therapeutisch tmp 7 mg/kg/d

Other: for which age group do you use this medication?
4 wochen

Other: which time schedule do you use? [Day(s) before VUS/VCUG, how many?]
Yes

Other: which time schedule do you use? [Day(s) before VUS/VCUG, how many?][Comment]
0

Other: which time schedule do you use? [Day of VUS/VCUG]
Yes

Other: which time schedule do you use? [Day of VUS/VCUG][Comment]

Other: which time schedule do you use? [Day(s) after VUS/VCUG, how many?]
Yes

Other: which time schedule do you use? [Day(s) after VUS/VCUG, how many?][Comment]
2

Indication for prophylaxis

Which patients receive periinterventional antibiotic prophylaxis? (multiple answers possible) [all]
Yes

Which patients receive periinterventional antibiotic prophylaxis? (multiple answers possible) [patients post urinary tract infection/pyelonephritis/urosepsis]
N/A

Which patients receive periinterventional antibiotic prophylaxis? (multiple answers possible) [patients with urinary tract dilation in ultrasound]
N/A

Which patients receive periinterventional antibiotic prophylaxis? (multiple answers possible) [patients with prenatal urinary tract dilation]
N/A

Which patients receive periinterventional antibiotic prophylaxis? (multiple answers possible) [patients post VUR surgery]
N/A

Which patients receive periinterventional antibiotic prophylaxis? (multiple answers possible) [none]
N/A

Are there situations or clinical findings in the patient where you deviate from your prophylaxis standard (medication, dose, duration, age of the patient)? (multiple answers possible, please specify in answer box) [in case of already known vesicoureteral reflux (VUR)]
No

Are there situations or clinical findings in the patient where you deviate from your prophylaxis standard (medication, dose, duration, age of the patient)? (multiple answers possible, please specify in answer box) [in case of already known vesicoureteral reflux (VUR)][Comment]

Are there situations or clinical findings in the patient where you deviate from your prophylaxis standard (medication, dose, duration, age of the patient)? (multiple answers possible, please specify in answer box) [in patients with recurrent urinary tract infections (UTI)]
No

Are there situations or clinical findings in the patient where you deviate from your prophylaxis standard (medication, dose, duration, age of the patient)? (multiple answers possible, please specify in answer box) [in patients with recurrent urinary tract infections (UTI)][Comment]

Are there situations or clinical findings in the patient where you deviate from your prophylaxis standard (medication, dose, duration, age of the patient)? (multiple answers possible, please specify in answer box) [if multidrug-resistant organisms (MDRO) are detected in urine cultures]
Yes

Are there situations or clinical findings in the patient where you deviate from your prophylaxis standard (medication, dose, duration, age of the patient)? (multiple answers possible, please specify in answer box) [if multidrug-resistant organisms (MDRO) are detected in urine cultures][Comment]
ja

Are there situations or clinical findings in the patient where you deviate from your prophylaxis standard (medication, dose, duration, age of the patient)? (multiple answers possible, please specify in answer box) [in newborns]
Yes

Are there situations or clinical findings in the patient where you deviate from your prophylaxis standard (medication, dose, duration, age of the patient)? (multiple answers possible, please specify in answer box) [in newborns][Comment]
cefclor

Are there situations or clinical findings in the patient where you deviate from your prophylaxis standard (medication, dose, duration, age of the patient)? (multiple answers possible, please specify in answer box) [Other]

Are there situations or clinical findings in the patient where you deviate from your prophylaxis standard (medication, dose, duration, age of the patient)? (multiple answers possible, please specify in answer box) [Other comment]

Are there situations in which you use intravenous prophylaxis?
No

Voiding analysis

Are there differences in antibiotic prophylaxis between VUS and VCUG in your department?
No

Is a non-pathological urine strip test required in preparation for VUS/VCUG in your department?
Yes

What is the maximum amount of days accepted for urine strip test to be performed prior to the exam?
1

Is a non-pathological urine culture required in preparation for VUS/VCUG in your department?
No

Do patients only receive post-procedure prophylaxis if vesicoureteral reflux (VUR) is detected in VUS/VCUG?
No

Do other MUS/MCU findings (e.g. urethral valves) have an influence on post-procedure prophylaxis?
No

End of survey

Thank you for taking the time to answer this survey!

May we contact you personally if we have further questions?
Yes

E-mail address:
Martin.Koemhoff@uk-gm.de

Survey response 92

Response ID
94

Date submitted
2025-01-28 13:28:13

Last page
5

Start language
de

Seed
1116097674

Date started
2025-01-28 13:20:40

Date last action
2025-01-28 13:28:13

Standard Operation Procedure

Who fills out this survey?
Pediatrician

Who fills out this survey? [Other]

In which type of hospital are you employed?
Community hospital (>50 beds for pediatric patients)

In which type of hospital are you employed? [Other]

In which country do you work?
Deutschland

Does your department have a defined protocol for infection prophylaxis in the context of VUS/VCUG?
No

Has this protocol been transformed into a written standard operating procedure (SOP)?
N/A

Antibiotic Prophylaxis

Are there situations/constellations in which you do not use antibiotic prophylaxis for VUS/VCUG?
No

Please specify these situations/constellations: [girl with first uncomplicated UTI without urinary tract dilation in ultrasound]
N/A

Please specify these situations/constellations: [boy with first uncomplicated UTI without urinary tract dilation in ultrasound]
N/A

Please specify these situations/constellations: [in patients with low grade urinary tract dilation (up to 2nd grade) and no UTI in medical history]
N/A

If you use antibiotic prophylaxis: which mediaction do you use? (multiple answers and subdivision into age groups possible) [Trimethoprim]
No

If you use antibiotic prophylaxis: which mediaction do you use? (multiple answers and subdivision into age groups possible) [Nitrofurantoin]
No

If you use antibiotic prophylaxis: which mediaction do you use? (multiple answers and subdivision into age groups possible) [Amoxicillin]
No

If you use antibiotic prophylaxis: which mediaction do you use? (multiple answers and subdivision into age groups possible) [Cefaclor]
Yes

If you use antibiotic prophylaxis: which mediaction do you use? (multiple answers and subdivision into age groups possible) [Amoxicillin/clavulanic acid]
No

If you use antibiotic prophylaxis: which mediaction do you use? (multiple answers and subdivision into age groups possible) [Other]
No

Trimethoprim: which time schedule do you use? [Day(s) before VUS/VCUG, how many?]
N/A

Trimethoprim: which time schedule do you use? [Day of VUS/VCUG]
N/A

Trimethoprim: which time schedule do you use? [Day(s) after VUS/VCUG, how many?]
N/A

Nitrofurantoin: which time schedule do you use? [Day(s) before VUS/VCUG, how many?]
N/A

Nitrofurantoin: which time schedule do you use? [Day of VUS/VCUG]
N/A

Nitrofurantoin: which time schedule do you use? [Day(s) after VUS/VCUG, how many?]
N/A

Amoxicillin: which time schedule do you use? [Day(s) before VUS/VCUG, how many?]
N/A

Amoxicillin: which time schedule do you use? [Day of VUS/VCUG]
N/A

Amoxicillin: which time schedule do you use? [Day(s) after VUS/VCUG, how many?]
N/A

Cefaclor: in what dosage?
prophylactic (10 mg/kg bodyweight/d)

Cefaclor: in what dosage? [Other]

Cefaclor: for which age group do you use cefaclor?
Alle

Cefaclor: which time schedule do you use? [Days(s) before VUS/VCUG, how many?]
No

Cefaclor: which time schedule do you use? [Days(s) before VUS/VCUG, how many?][Comment]

Cefaclor: which time schedule do you use? [Day of VUS/VCUG]
Yes

Cefaclor: which time schedule do you use? [Day of VUS/VCUG][Comment]

Cefaclor: which time schedule do you use? [Day(s) after VUS/VCUG, how many?]
No

Cefaclor: which time schedule do you use? [Day(s) after VUS/VCUG, how many?][Comment]

Amoxicillin/clavulanic acid: in what dosage? (Amoxicillin portion) [prophylaktic (10-20 mg/Kg bodyweight/d)]
N/A

Amoxicillin/clavulanic acid: in what dosage? (Amoxicillin portion) [double prophylactic (20-40 mg/Kg bodyweight/d)]
N/A

Amoxicillin/clavulanic acid: in what dosage? (Amoxicillin portion) [therapeutic (80-100 mg/Kg bodyweight/d)]
N/A

Amoxicillin/clavulanic acid: which time schedule do you use? [Day(s) before VUS/VCUG]
N/A

Amoxicillin/clavulanic acid: which time schedule do you use? [Day of VUS/VCUG]
N/A

Amoxicillin/clavulanic acid: which time schedule do you use? [Day(s) after VUS/VCUG]
N/A

Other: which time schedule do you use? [Day(s) before VUS/VCUG, how many?]
N/A

Other: which time schedule do you use? [Day of VUS/VCUG]
N/A

Other: which time schedule do you use? [Day(s) after VUS/VCUG, how many?]
N/A

Indication for prophylaxis

Which patients receive periinterventional antibiotic prophylaxis? (multiple answers possible) [all]
Yes

Which patients receive periinterventional antibiotic prophylaxis? (multiple answers possible) [patients post urinary tract infection/pyelonephritis/urosepsis]
N/A

Which patients receive periinterventional antibiotic prophylaxis? (multiple answers possible) [patients with urinary tract dilation in ultrasound]
N/A

Which patients receive periinterventional antibiotic prophylaxis? (multiple answers possible) [patients with prenatal urinary tract dilation]
N/A

Which patients receive periinterventional antibiotic prophylaxis? (multiple answers possible) [patients post VUR surgery]
N/A

Which patients receive periinterventional antibiotic prophylaxis? (multiple answers possible) [none]
N/A

Are there situations or clinical findings in the patient where you deviate from your prophylaxis standard (medication, dose, duration, age of the patient)? (multiple answers possible, please specify in answer box) [in case of already known vesicoureteral reflux (VUR)]
No

Are there situations or clinical findings in the patient where you deviate from your prophylaxis standard (medication, dose, duration, age of the patient)? (multiple answers possible, please specify in answer box) [in case of already known vesicoureteral reflux (VUR)][Comment]

Are there situations or clinical findings in the patient where you deviate from your prophylaxis standard (medication, dose, duration, age of the patient)? (multiple answers possible, please specify in answer box) [in patients with recurrent urinary tract infections (UTI)]
No

Are there situations or clinical findings in the patient where you deviate from your prophylaxis standard (medication, dose, duration, age of the patient)? (multiple answers possible, please specify in answer box) [in patients with recurrent urinary tract infections (UTI)][Comment]

Are there situations or clinical findings in the patient where you deviate from your prophylaxis standard (medication, dose, duration, age of the patient)? (multiple answers possible, please specify in answer box) [if multidrug-resistant organisms (MDRO) are detected in urine cultures]
Yes

Are there situations or clinical findings in the patient where you deviate from your prophylaxis standard (medication, dose, duration, age of the patient)? (multiple answers possible, please specify in answer box) [if multidrug-resistant organisms (MDRO) are detected in urine cultures][Comment]
Z. B. Bekannter 3mrgn

Are there situations or clinical findings in the patient where you deviate from your prophylaxis standard (medication, dose, duration, age of the patient)? (multiple answers possible, please specify in answer box) [in newborns]
No

Are there situations or clinical findings in the patient where you deviate from your prophylaxis standard (medication, dose, duration, age of the patient)? (multiple answers possible, please specify in answer box) [in newborns][Comment]

Are there situations or clinical findings in the patient where you deviate from your prophylaxis standard (medication, dose, duration, age of the patient)? (multiple answers possible, please specify in answer box) [Other]

Are there situations or clinical findings in the patient where you deviate from your prophylaxis standard (medication, dose, duration, age of the patient)? (multiple answers possible, please specify in answer box) [Other comment]

Are there situations in which you use intravenous prophylaxis?
No

Voiding analysis

Are there differences in antibiotic prophylaxis between VUS and VCUG in your department?
No

Is a non-pathological urine strip test required in preparation for VUS/VCUG in your department?
No

Is a non-pathological urine culture required in preparation for VUS/VCUG in your department?
No

Do patients only receive post-procedure prophylaxis if vesicoureteral reflux (VUR) is detected in VUS/VCUG?
No

Do other MUS/MCU findings (e.g. urethral valves) have an influence on post-procedure prophylaxis?
No

End of survey

Thank you for taking the time to answer this survey!

May we contact you personally if we have further questions?
Yes

E-mail address:
Matthias.Hansen@kfh.de

Survey response 93

Response ID
95

Date submitted
2025-01-28 13:24:45

Last page
5

Start language
de

Seed
2136880925

Date started
2025-01-28 13:22:10

Date last action
2025-01-28 13:24:45

Standard Operation Procedure

Who fills out this survey?
Pediatrician

Who fills out this survey? [Other]

In which type of hospital are you employed?
University Hospital

In which type of hospital are you employed? [Other]

In which country do you work?
Deutschland

Does your department have a defined protocol for infection prophylaxis in the context of VUS/VCUG?
No

Has this protocol been transformed into a written standard operating procedure (SOP)?
N/A

Antibiotic Prophylaxis

Are there situations/constellations in which you do not use antibiotic prophylaxis for VUS/VCUG?
No

Please specify these situations/constellations: [girl with first uncomplicated UTI without urinary tract dilation in ultrasound]
N/A

Please specify these situations/constellations: [boy with first uncomplicated UTI without urinary tract dilation in ultrasound]
N/A

Please specify these situations/constellations: [in patients with low grade urinary tract dilation (up to 2nd grade) and no UTI in medical history]
N/A

If you use antibiotic prophylaxis: which mediaction do you use? (multiple answers and subdivision into age groups possible) [Trimethoprim]
Yes

If you use antibiotic prophylaxis: which mediaction do you use? (multiple answers and subdivision into age groups possible) [Nitrofurantoin]
No

If you use antibiotic prophylaxis: which mediaction do you use? (multiple answers and subdivision into age groups possible) [Amoxicillin]
No

If you use antibiotic prophylaxis: which mediaction do you use? (multiple answers and subdivision into age groups possible) [Cefaclor]
No

If you use antibiotic prophylaxis: which mediaction do you use? (multiple answers and subdivision into age groups possible) [Amoxicillin/clavulanic acid]
No

If you use antibiotic prophylaxis: which mediaction do you use? (multiple answers and subdivision into age groups possible) [Other]
No

Trimethoprim: in what dosage?
prophylactic (2 mg/kg bodyweight/d)

Trimethoprim: in what dosage? [Other]

Trimethoprim: for which age group do you use trimethoprim?
> 6 Wochen

Trimethoprim: which time schedule do you use? [Day(s) before VUS/VCUG, how many?]
No

Trimethoprim: which time schedule do you use? [Day(s) before VUS/VCUG, how many?][Comment]

Trimethoprim: which time schedule do you use? [Day of VUS/VCUG]
Yes

Trimethoprim: which time schedule do you use? [Day of VUS/VCUG][Comment]

Trimethoprim: which time schedule do you use? [Day(s) after VUS/VCUG, how many?]
Yes

Trimethoprim: which time schedule do you use? [Day(s) after VUS/VCUG, how many?][Comment]
1

Nitrofurantoin: which time schedule do you use? [Day(s) before VUS/VCUG, how many?]
N/A

Nitrofurantoin: which time schedule do you use? [Day of VUS/VCUG]
N/A

Nitrofurantoin: which time schedule do you use? [Day(s) after VUS/VCUG, how many?]
N/A

Amoxicillin: which time schedule do you use? [Day(s) before VUS/VCUG, how many?]
N/A

Amoxicillin: which time schedule do you use? [Day of VUS/VCUG]
N/A

Amoxicillin: which time schedule do you use? [Day(s) after VUS/VCUG, how many?]
N/A

Cefaclor: which time schedule do you use? [Days(s) before VUS/VCUG, how many?]
N/A

Cefaclor: which time schedule do you use? [Day of VUS/VCUG]
N/A

Cefaclor: which time schedule do you use? [Day(s) after VUS/VCUG, how many?]
N/A

Amoxicillin/clavulanic acid: in what dosage? (Amoxicillin portion) [prophylaktic (10-20 mg/Kg bodyweight/d)]
N/A

Amoxicillin/clavulanic acid: in what dosage? (Amoxicillin portion) [double prophylactic (20-40 mg/Kg bodyweight/d)]
N/A

Amoxicillin/clavulanic acid: in what dosage? (Amoxicillin portion) [therapeutic (80-100 mg/Kg bodyweight/d)]
N/A

Amoxicillin/clavulanic acid: which time schedule do you use? [Day(s) before VUS/VCUG]
N/A

Amoxicillin/clavulanic acid: which time schedule do you use? [Day of VUS/VCUG]
N/A

Amoxicillin/clavulanic acid: which time schedule do you use? [Day(s) after VUS/VCUG]
N/A

Other: which time schedule do you use? [Day(s) before VUS/VCUG, how many?]
N/A

Other: which time schedule do you use? [Day of VUS/VCUG]
N/A

Other: which time schedule do you use? [Day(s) after VUS/VCUG, how many?]
N/A

Indication for prophylaxis

Which patients receive periinterventional antibiotic prophylaxis? (multiple answers possible) [all]
Yes

Which patients receive periinterventional antibiotic prophylaxis? (multiple answers possible) [patients post urinary tract infection/pyelonephritis/urosepsis]
N/A

Which patients receive periinterventional antibiotic prophylaxis? (multiple answers possible) [patients with urinary tract dilation in ultrasound]
N/A

Which patients receive periinterventional antibiotic prophylaxis? (multiple answers possible) [patients with prenatal urinary tract dilation]
N/A

Which patients receive periinterventional antibiotic prophylaxis? (multiple answers possible) [patients post VUR surgery]
N/A

Which patients receive periinterventional antibiotic prophylaxis? (multiple answers possible) [none]
N/A

Are there situations or clinical findings in the patient where you deviate from your prophylaxis standard (medication, dose, duration, age of the patient)? (multiple answers possible, please specify in answer box) [in case of already known vesicoureteral reflux (VUR)]
Yes

Are there situations or clinical findings in the patient where you deviate from your prophylaxis standard (medication, dose, duration, age of the patient)? (multiple answers possible, please specify in answer box) [in case of already known vesicoureteral reflux (VUR)][Comment]
Fortführung Prophylaxe

Are there situations or clinical findings in the patient where you deviate from your prophylaxis standard (medication, dose, duration, age of the patient)? (multiple answers possible, please specify in answer box) [in patients with recurrent urinary tract infections (UTI)]
No

Are there situations or clinical findings in the patient where you deviate from your prophylaxis standard (medication, dose, duration, age of the patient)? (multiple answers possible, please specify in answer box) [in patients with recurrent urinary tract infections (UTI)][Comment]

Are there situations or clinical findings in the patient where you deviate from your prophylaxis standard (medication, dose, duration, age of the patient)? (multiple answers possible, please specify in answer box) [if multidrug-resistant organisms (MDRO) are detected in urine cultures]
No

Are there situations or clinical findings in the patient where you deviate from your prophylaxis standard (medication, dose, duration, age of the patient)? (multiple answers possible, please specify in answer box) [if multidrug-resistant organisms (MDRO) are detected in urine cultures][Comment]

Are there situations or clinical findings in the patient where you deviate from your prophylaxis standard (medication, dose, duration, age of the patient)? (multiple answers possible, please specify in answer box) [in newborns]
No

Are there situations or clinical findings in the patient where you deviate from your prophylaxis standard (medication, dose, duration, age of the patient)? (multiple answers possible, please specify in answer box) [in newborns][Comment]

Are there situations or clinical findings in the patient where you deviate from your prophylaxis standard (medication, dose, duration, age of the patient)? (multiple answers possible, please specify in answer box) [Other]

Are there situations or clinical findings in the patient where you deviate from your prophylaxis standard (medication, dose, duration, age of the patient)? (multiple answers possible, please specify in answer box) [Other comment]

Are there situations in which you use intravenous prophylaxis?
No

Voiding analysis

Are there differences in antibiotic prophylaxis between VUS and VCUG in your department?
No

Is a non-pathological urine strip test required in preparation for VUS/VCUG in your department?
Yes

What is the maximum amount of days accepted for urine strip test to be performed prior to the exam?
3

Is a non-pathological urine culture required in preparation for VUS/VCUG in your department?
Yes

What is the maximum amount of days accepted for urine culture to be performed prior to the exam?
3

Do patients only receive post-procedure prophylaxis if vesicoureteral reflux (VUR) is detected in VUS/VCUG?
No

Do other MUS/MCU findings (e.g. urethral valves) have an influence on post-procedure prophylaxis?
No

End of survey

Thank you for taking the time to answer this survey!

May we contact you personally if we have further questions?
No

Survey response 94

Response ID
96

Date submitted
2025-01-28 13:31:27

Last page
5

Start language
de

Seed
74104002

Date started
2025-01-28 13:24:37

Date last action
2025-01-28 13:31:27

Standard Operation Procedure

Who fills out this survey?
Other

Who fills out this survey? [Other]
Kindernephrologe

In which type of hospital are you employed?
Community hospital (>50 beds for pediatric patients)

In which type of hospital are you employed? [Other]

In which country do you work?
Hessen

Does your department have a defined protocol for infection prophylaxis in the context of VUS/VCUG?
Yes

Has this protocol been transformed into a written standard operating procedure (SOP)?
No

Antibiotic Prophylaxis

Are there situations/constellations in which you do not use antibiotic prophylaxis for VUS/VCUG?
No

Please specify these situations/constellations: [girl with first uncomplicated UTI without urinary tract dilation in ultrasound]
N/A

Please specify these situations/constellations: [boy with first uncomplicated UTI without urinary tract dilation in ultrasound]
N/A

Please specify these situations/constellations: [in patients with low grade urinary tract dilation (up to 2nd grade) and no UTI in medical history]
N/A

If you use antibiotic prophylaxis: which mediaction do you use? (multiple answers and subdivision into age groups possible) [Trimethoprim]
Yes

If you use antibiotic prophylaxis: which mediaction do you use? (multiple answers and subdivision into age groups possible) [Nitrofurantoin]
Yes

If you use antibiotic prophylaxis: which mediaction do you use? (multiple answers and subdivision into age groups possible) [Amoxicillin]
No

If you use antibiotic prophylaxis: which mediaction do you use? (multiple answers and subdivision into age groups possible) [Cefaclor]
Yes

If you use antibiotic prophylaxis: which mediaction do you use? (multiple answers and subdivision into age groups possible) [Amoxicillin/clavulanic acid]
No

If you use antibiotic prophylaxis: which mediaction do you use? (multiple answers and subdivision into age groups possible) [Other]
No

Trimethoprim: in what dosage?
prophylactic (2 mg/kg bodyweight/d)

Trimethoprim: in what dosage? [Other]

Trimethoprim: for which age group do you use trimethoprim?
Ab 6 Wochen

Trimethoprim: which time schedule do you use? [Day(s) before VUS/VCUG, how many?]
No

Trimethoprim: which time schedule do you use? [Day(s) before VUS/VCUG, how many?][Comment]

Trimethoprim: which time schedule do you use? [Day of VUS/VCUG]
Yes

Trimethoprim: which time schedule do you use? [Day of VUS/VCUG][Comment]

Trimethoprim: which time schedule do you use? [Day(s) after VUS/VCUG, how many?]
No

Trimethoprim: which time schedule do you use? [Day(s) after VUS/VCUG, how many?][Comment]

Nitrofurantoin: in what dosage?
prophylactic (1-2 mg/kg bodyweight/d)

Nitrofurantoin: in what dosage? [Other]

Nitrofurantoin: for which age group do you use nitrofurantoin?
Ab 6 Monate

Nitrofurantoin: which time schedule do you use? [Day(s) before VUS/VCUG, how many?]
No

Nitrofurantoin: which time schedule do you use? [Day(s) before VUS/VCUG, how many?][Comment]

Nitrofurantoin: which time schedule do you use? [Day of VUS/VCUG]
Yes

Nitrofurantoin: which time schedule do you use? [Day of VUS/VCUG][Comment]

Nitrofurantoin: which time schedule do you use? [Day(s) after VUS/VCUG, how many?]
No

Nitrofurantoin: which time schedule do you use? [Day(s) after VUS/VCUG, how many?][Comment]

Amoxicillin: which time schedule do you use? [Day(s) before VUS/VCUG, how many?]
N/A

Amoxicillin: which time schedule do you use? [Day of VUS/VCUG]
N/A

Amoxicillin: which time schedule do you use? [Day(s) after VUS/VCUG, how many?]
N/A

Cefaclor: in what dosage?
Other

Cefaclor: in what dosage? [Other]
15/kg

Cefaclor: for which age group do you use cefaclor?
Neugeborene

Cefaclor: which time schedule do you use? [Days(s) before VUS/VCUG, how many?]
No

Cefaclor: which time schedule do you use? [Days(s) before VUS/VCUG, how many?][Comment]

Cefaclor: which time schedule do you use? [Day of VUS/VCUG]
Yes

Cefaclor: which time schedule do you use? [Day of VUS/VCUG][Comment]

Cefaclor: which time schedule do you use? [Day(s) after VUS/VCUG, how many?]
No

Cefaclor: which time schedule do you use? [Day(s) after VUS/VCUG, how many?][Comment]

Amoxicillin/clavulanic acid: in what dosage? (Amoxicillin portion) [prophylaktic (10-20 mg/Kg bodyweight/d)]
N/A

Amoxicillin/clavulanic acid: in what dosage? (Amoxicillin portion) [double prophylactic (20-40 mg/Kg bodyweight/d)]
N/A

Amoxicillin/clavulanic acid: in what dosage? (Amoxicillin portion) [therapeutic (80-100 mg/Kg bodyweight/d)]
N/A

Amoxicillin/clavulanic acid: which time schedule do you use? [Day(s) before VUS/VCUG]
N/A

Amoxicillin/clavulanic acid: which time schedule do you use? [Day of VUS/VCUG]
N/A

Amoxicillin/clavulanic acid: which time schedule do you use? [Day(s) after VUS/VCUG]
N/A

Other: which time schedule do you use? [Day(s) before VUS/VCUG, how many?]
N/A

Other: which time schedule do you use? [Day of VUS/VCUG]
N/A

Other: which time schedule do you use? [Day(s) after VUS/VCUG, how many?]
N/A

Indication for prophylaxis

Which patients receive periinterventional antibiotic prophylaxis? (multiple answers possible) [all]
Yes

Which patients receive periinterventional antibiotic prophylaxis? (multiple answers possible) [patients post urinary tract infection/pyelonephritis/urosepsis]
N/A

Which patients receive periinterventional antibiotic prophylaxis? (multiple answers possible) [patients with urinary tract dilation in ultrasound]
N/A

Which patients receive periinterventional antibiotic prophylaxis? (multiple answers possible) [patients with prenatal urinary tract dilation]
N/A

Which patients receive periinterventional antibiotic prophylaxis? (multiple answers possible) [patients post VUR surgery]
N/A

Which patients receive periinterventional antibiotic prophylaxis? (multiple answers possible) [none]
N/A

Are there situations or clinical findings in the patient where you deviate from your prophylaxis standard (medication, dose, duration, age of the patient)? (multiple answers possible, please specify in answer box) [in case of already known vesicoureteral reflux (VUR)]
Yes

Are there situations or clinical findings in the patient where you deviate from your prophylaxis standard (medication, dose, duration, age of the patient)? (multiple answers possible, please specify in answer box) [in case of already known vesicoureteral reflux (VUR)][Comment]
Nach antibiogramm

Are there situations or clinical findings in the patient where you deviate from your prophylaxis standard (medication, dose, duration, age of the patient)? (multiple answers possible, please specify in answer box) [in patients with recurrent urinary tract infections (UTI)]
Yes

Are there situations or clinical findings in the patient where you deviate from your prophylaxis standard (medication, dose, duration, age of the patient)? (multiple answers possible, please specify in answer box) [in patients with recurrent urinary tract infections (UTI)][Comment]
Antibiogramm

Are there situations or clinical findings in the patient where you deviate from your prophylaxis standard (medication, dose, duration, age of the patient)? (multiple answers possible, please specify in answer box) [if multidrug-resistant organisms (MDRO) are detected in urine cultures]
Yes

Are there situations or clinical findings in the patient where you deviate from your prophylaxis standard (medication, dose, duration, age of the patient)? (multiple answers possible, please specify in answer box) [if multidrug-resistant organisms (MDRO) are detected in urine cultures][Comment]
Antibiogramm

Are there situations or clinical findings in the patient where you deviate from your prophylaxis standard (medication, dose, duration, age of the patient)? (multiple answers possible, please specify in answer box) [in newborns]
Yes

Are there situations or clinical findings in the patient where you deviate from your prophylaxis standard (medication, dose, duration, age of the patient)? (multiple answers possible, please specify in answer box) [in newborns][Comment]
Nur Cefaclor

Are there situations or clinical findings in the patient where you deviate from your prophylaxis standard (medication, dose, duration, age of the patient)? (multiple answers possible, please specify in answer box) [Other]

Are there situations or clinical findings in the patient where you deviate from your prophylaxis standard (medication, dose, duration, age of the patient)? (multiple answers possible, please specify in answer box) [Other comment]

Are there situations in which you use intravenous prophylaxis?
No

Voiding analysis

Are there differences in antibiotic prophylaxis between VUS and VCUG in your department?
No

Is a non-pathological urine strip test required in preparation for VUS/VCUG in your department?
No

Is a non-pathological urine culture required in preparation for VUS/VCUG in your department?
No

Do patients only receive post-procedure prophylaxis if vesicoureteral reflux (VUR) is detected in VUS/VCUG?
No

Do other MUS/MCU findings (e.g. urethral valves) have an influence on post-procedure prophylaxis?
Yes

Please specify:
Dann bleibt Prophylaxe

End of survey

Thank you for taking the time to answer this survey!

May we contact you personally if we have further questions?
No

Survey response 95

Response ID
97

Date submitted
2025-01-28 13:44:13

Last page
5

Start language
de

Seed
426335023

Date started
2025-01-28 13:33:51

Date last action
2025-01-28 13:44:13

Standard Operation Procedure

Who fills out this survey?
Pediatrician

Who fills out this survey? [Other]

In which type of hospital are you employed?
Community hospital (>50 beds for pediatric patients)

In which type of hospital are you employed? [Other]

In which country do you work?
Deutschland

Does your department have a defined protocol for infection prophylaxis in the context of VUS/VCUG?
Yes

Has this protocol been transformed into a written standard operating procedure (SOP)?
No

Antibiotic Prophylaxis

Are there situations/constellations in which you do not use antibiotic prophylaxis for VUS/VCUG?
No

Please specify these situations/constellations: [girl with first uncomplicated UTI without urinary tract dilation in ultrasound]
N/A

Please specify these situations/constellations: [boy with first uncomplicated UTI without urinary tract dilation in ultrasound]
N/A

Please specify these situations/constellations: [in patients with low grade urinary tract dilation (up to 2nd grade) and no UTI in medical history]
N/A

If you use antibiotic prophylaxis: which mediaction do you use? (multiple answers and subdivision into age groups possible) [Trimethoprim]
Yes

If you use antibiotic prophylaxis: which mediaction do you use? (multiple answers and subdivision into age groups possible) [Nitrofurantoin]
Yes

If you use antibiotic prophylaxis: which mediaction do you use? (multiple answers and subdivision into age groups possible) [Amoxicillin]
No

If you use antibiotic prophylaxis: which mediaction do you use? (multiple answers and subdivision into age groups possible) [Cefaclor]
Yes

If you use antibiotic prophylaxis: which mediaction do you use? (multiple answers and subdivision into age groups possible) [Amoxicillin/clavulanic acid]
No

If you use antibiotic prophylaxis: which mediaction do you use? (multiple answers and subdivision into age groups possible) [Other]
No

Trimethoprim: in what dosage?
prophylactic (2 mg/kg bodyweight/d)

Trimethoprim: in what dosage? [Other]

Trimethoprim: for which age group do you use trimethoprim?
ab 6 Wochen

Trimethoprim: which time schedule do you use? [Day(s) before VUS/VCUG, how many?]
Yes

Trimethoprim: which time schedule do you use? [Day(s) before VUS/VCUG, how many?][Comment]
1, wenn keine Dauerprophylaxe

Trimethoprim: which time schedule do you use? [Day of VUS/VCUG]
Yes

Trimethoprim: which time schedule do you use? [Day of VUS/VCUG][Comment]

Trimethoprim: which time schedule do you use? [Day(s) after VUS/VCUG, how many?]
Yes

Trimethoprim: which time schedule do you use? [Day(s) after VUS/VCUG, how many?][Comment]
1, wenn keine Dauerprophylaxe

Nitrofurantoin: in what dosage?
prophylactic (1-2 mg/kg bodyweight/d)

Nitrofurantoin: in what dosage? [Other]

Nitrofurantoin: for which age group do you use nitrofurantoin?
> 3 Monate

Nitrofurantoin: which time schedule do you use? [Day(s) before VUS/VCUG, how many?]
Yes

Nitrofurantoin: which time schedule do you use? [Day(s) before VUS/VCUG, how many?][Comment]
1, wenn keine Dauerprophylaxe

Nitrofurantoin: which time schedule do you use? [Day of VUS/VCUG]
Yes

Nitrofurantoin: which time schedule do you use? [Day of VUS/VCUG][Comment]

Nitrofurantoin: which time schedule do you use? [Day(s) after VUS/VCUG, how many?]
Yes

Nitrofurantoin: which time schedule do you use? [Day(s) after VUS/VCUG, how many?][Comment]
1, wenn keine Dauerprophylaxe

Amoxicillin: which time schedule do you use? [Day(s) before VUS/VCUG, how many?]
N/A

Amoxicillin: which time schedule do you use? [Day of VUS/VCUG]
N/A

Amoxicillin: which time schedule do you use? [Day(s) after VUS/VCUG, how many?]
N/A

Cefaclor: in what dosage?
prophylactic (10 mg/kg bodyweight/d)

Cefaclor: in what dosage? [Other]

Cefaclor: for which age group do you use cefaclor?
ab 1 Monat

Cefaclor: which time schedule do you use? [Days(s) before VUS/VCUG, how many?]
Yes

Cefaclor: which time schedule do you use? [Days(s) before VUS/VCUG, how many?][Comment]
1, wenn keine Dauerprophylaxe

Cefaclor: which time schedule do you use? [Day of VUS/VCUG]
Yes

Cefaclor: which time schedule do you use? [Day of VUS/VCUG][Comment]

Cefaclor: which time schedule do you use? [Day(s) after VUS/VCUG, how many?]
Yes

Cefaclor: which time schedule do you use? [Day(s) after VUS/VCUG, how many?][Comment]
1, wenn keine Dauerprophylaxe

Amoxicillin/clavulanic acid: in what dosage? (Amoxicillin portion) [prophylaktic (10-20 mg/Kg bodyweight/d)]
N/A

Amoxicillin/clavulanic acid: in what dosage? (Amoxicillin portion) [double prophylactic (20-40 mg/Kg bodyweight/d)]
N/A

Amoxicillin/clavulanic acid: in what dosage? (Amoxicillin portion) [therapeutic (80-100 mg/Kg bodyweight/d)]
N/A

Amoxicillin/clavulanic acid: which time schedule do you use? [Day(s) before VUS/VCUG]
N/A

Amoxicillin/clavulanic acid: which time schedule do you use? [Day of VUS/VCUG]
N/A

Amoxicillin/clavulanic acid: which time schedule do you use? [Day(s) after VUS/VCUG]
N/A

Other: which time schedule do you use? [Day(s) before VUS/VCUG, how many?]
N/A

Other: which time schedule do you use? [Day of VUS/VCUG]
N/A

Other: which time schedule do you use? [Day(s) after VUS/VCUG, how many?]
N/A

Indication for prophylaxis

Which patients receive periinterventional antibiotic prophylaxis? (multiple answers possible) [all]
Yes

Which patients receive periinterventional antibiotic prophylaxis? (multiple answers possible) [patients post urinary tract infection/pyelonephritis/urosepsis]
N/A

Which patients receive periinterventional antibiotic prophylaxis? (multiple answers possible) [patients with urinary tract dilation in ultrasound]
N/A

Which patients receive periinterventional antibiotic prophylaxis? (multiple answers possible) [patients with prenatal urinary tract dilation]
N/A

Which patients receive periinterventional antibiotic prophylaxis? (multiple answers possible) [patients post VUR surgery]
N/A

Which patients receive periinterventional antibiotic prophylaxis? (multiple answers possible) [none]
N/A

Are there situations or clinical findings in the patient where you deviate from your prophylaxis standard (medication, dose, duration, age of the patient)? (multiple answers possible, please specify in answer box) [in case of already known vesicoureteral reflux (VUR)]
Yes

Are there situations or clinical findings in the patient where you deviate from your prophylaxis standard (medication, dose, duration, age of the patient)? (multiple answers possible, please specify in answer box) [in case of already known vesicoureteral reflux (VUR)][Comment]
Dauerprophylaxe täglich in prophylaktischer Dosierung bis mind. zum Erhalt des Befundes

Are there situations or clinical findings in the patient where you deviate from your prophylaxis standard (medication, dose, duration, age of the patient)? (multiple answers possible, please specify in answer box) [in patients with recurrent urinary tract infections (UTI)]
Yes

Are there situations or clinical findings in the patient where you deviate from your prophylaxis standard (medication, dose, duration, age of the patient)? (multiple answers possible, please specify in answer box) [in patients with recurrent urinary tract infections (UTI)][Comment]
ggf. Dauerprophylaxe täglich in prophylaktischer Dosierung bis mind. zum Erhalt des Befundes

Are there situations or clinical findings in the patient where you deviate from your prophylaxis standard (medication, dose, duration, age of the patient)? (multiple answers possible, please specify in answer box) [if multidrug-resistant organisms (MDRO) are detected in urine cultures]
Yes

Are there situations or clinical findings in the patient where you deviate from your prophylaxis standard (medication, dose, duration, age of the patient)? (multiple answers possible, please specify in answer box) [if multidrug-resistant organisms (MDRO) are detected in urine cultures][Comment]
Anpassung der Prophylaxe je nach Resistenzlage

Are there situations or clinical findings in the patient where you deviate from your prophylaxis standard (medication, dose, duration, age of the patient)? (multiple answers possible, please specify in answer box) [in newborns]
No

Are there situations or clinical findings in the patient where you deviate from your prophylaxis standard (medication, dose, duration, age of the patient)? (multiple answers possible, please specify in answer box) [in newborns][Comment]

Are there situations or clinical findings in the patient where you deviate from your prophylaxis standard (medication, dose, duration, age of the patient)? (multiple answers possible, please specify in answer box) [Other]
noch i.v. behandelter fieberh. HWI

Are there situations or clinical findings in the patient where you deviate from your prophylaxis standard (medication, dose, duration, age of the patient)? (multiple answers possible, please specify in answer box) [Other comment]
am Ende der i.v. Therapie unter therapeutischer Dosierung

Are there situations in which you use intravenous prophylaxis?
Yes

In which situations?
noch i.v. behandelter fieberh. HWI mit Zeichen einer Harntransportstörung oder ungewöhnlichem Keim:<br>wenn MCU noch stationär möglich in Radiologie Duchführung am Ende der i.v. Therapie unter therapeutischer Dosierung

Voiding analysis

Are there differences in antibiotic prophylaxis between VUS and VCUG in your department?
No

Is a non-pathological urine strip test required in preparation for VUS/VCUG in your department?
Yes

What is the maximum amount of days accepted for urine strip test to be performed prior to the exam?
1

Is a non-pathological urine culture required in preparation for VUS/VCUG in your department?
No

Do patients only receive post-procedure prophylaxis if vesicoureteral reflux (VUR) is detected in VUS/VCUG?
No

Do other MUS/MCU findings (e.g. urethral valves) have an influence on post-procedure prophylaxis?
No

End of survey

Thank you for taking the time to answer this survey!

May we contact you personally if we have further questions?
No

Survey response 96

Response ID
98

Date submitted
2025-01-28 14:05:31

Last page
5

Start language
de

Seed
1311725032

Date started
2025-01-28 13:51:32

Date last action
2025-01-28 14:05:31

Standard Operation Procedure

Who fills out this survey?
Other

Who fills out this survey? [Other]
Kindernephrologin

In which type of hospital are you employed?
University Hospital

In which type of hospital are you employed? [Other]

In which country do you work?
Deutschland

Does your department have a defined protocol for infection prophylaxis in the context of VUS/VCUG?
Yes

Has this protocol been transformed into a written standard operating procedure (SOP)?
Yes

Which department is the author of the SOP for infection prophylaxis in VUS/VCUG?
Pediatric nephrology

Which department is the author of the SOP for infection prophylaxis in VUS/VCUG? [Other]

Antibiotic Prophylaxis

Are there situations/constellations in which you do not use antibiotic prophylaxis for VUS/VCUG?
No

Please specify these situations/constellations: [girl with first uncomplicated UTI without urinary tract dilation in ultrasound]
N/A

Please specify these situations/constellations: [boy with first uncomplicated UTI without urinary tract dilation in ultrasound]
N/A

Please specify these situations/constellations: [in patients with low grade urinary tract dilation (up to 2nd grade) and no UTI in medical history]
N/A

If you use antibiotic prophylaxis: which mediaction do you use? (multiple answers and subdivision into age groups possible) [Trimethoprim]
No

If you use antibiotic prophylaxis: which mediaction do you use? (multiple answers and subdivision into age groups possible) [Nitrofurantoin]
Yes

If you use antibiotic prophylaxis: which mediaction do you use? (multiple answers and subdivision into age groups possible) [Amoxicillin]
No

If you use antibiotic prophylaxis: which mediaction do you use? (multiple answers and subdivision into age groups possible) [Cefaclor]
Yes

If you use antibiotic prophylaxis: which mediaction do you use? (multiple answers and subdivision into age groups possible) [Amoxicillin/clavulanic acid]
No

If you use antibiotic prophylaxis: which mediaction do you use? (multiple answers and subdivision into age groups possible) [Other]
No

Trimethoprim: which time schedule do you use? [Day(s) before VUS/VCUG, how many?]
N/A

Trimethoprim: which time schedule do you use? [Day of VUS/VCUG]
N/A

Trimethoprim: which time schedule do you use? [Day(s) after VUS/VCUG, how many?]
N/A

Nitrofurantoin: in what dosage?
double prophylactic (2-4 mg/kg bodyweight/d)

Nitrofurantoin: in what dosage? [Other]

Nitrofurantoin: for which age group do you use nitrofurantoin?
>3 Monate

Nitrofurantoin: which time schedule do you use? [Day(s) before VUS/VCUG, how many?]
No

Nitrofurantoin: which time schedule do you use? [Day(s) before VUS/VCUG, how many?][Comment]

Nitrofurantoin: which time schedule do you use? [Day of VUS/VCUG]
Yes

Nitrofurantoin: which time schedule do you use? [Day of VUS/VCUG][Comment]

Nitrofurantoin: which time schedule do you use? [Day(s) after VUS/VCUG, how many?]
Yes

Nitrofurantoin: which time schedule do you use? [Day(s) after VUS/VCUG, how many?][Comment]
2 Tage

Amoxicillin: which time schedule do you use? [Day(s) before VUS/VCUG, how many?]
N/A

Amoxicillin: which time schedule do you use? [Day of VUS/VCUG]
N/A

Amoxicillin: which time schedule do you use? [Day(s) after VUS/VCUG, how many?]
N/A

Cefaclor: in what dosage?
double prophylactic (20 mg/kg bodyweight/d)

Cefaclor: in what dosage? [Other]

Cefaclor: for which age group do you use cefaclor?
unter 3 Monate

Cefaclor: which time schedule do you use? [Days(s) before VUS/VCUG, how many?]
No

Cefaclor: which time schedule do you use? [Days(s) before VUS/VCUG, how many?][Comment]

Cefaclor: which time schedule do you use? [Day of VUS/VCUG]
Yes

Cefaclor: which time schedule do you use? [Day of VUS/VCUG][Comment]

Cefaclor: which time schedule do you use? [Day(s) after VUS/VCUG, how many?]
Yes

Cefaclor: which time schedule do you use? [Day(s) after VUS/VCUG, how many?][Comment]
2

Amoxicillin/clavulanic acid: in what dosage? (Amoxicillin portion) [prophylaktic (10-20 mg/Kg bodyweight/d)]
N/A

Amoxicillin/clavulanic acid: in what dosage? (Amoxicillin portion) [double prophylactic (20-40 mg/Kg bodyweight/d)]
N/A

Amoxicillin/clavulanic acid: in what dosage? (Amoxicillin portion) [therapeutic (80-100 mg/Kg bodyweight/d)]
N/A

Amoxicillin/clavulanic acid: which time schedule do you use? [Day(s) before VUS/VCUG]
N/A

Amoxicillin/clavulanic acid: which time schedule do you use? [Day of VUS/VCUG]
N/A

Amoxicillin/clavulanic acid: which time schedule do you use? [Day(s) after VUS/VCUG]
N/A

Other: which time schedule do you use? [Day(s) before VUS/VCUG, how many?]
N/A

Other: which time schedule do you use? [Day of VUS/VCUG]
N/A

Other: which time schedule do you use? [Day(s) after VUS/VCUG, how many?]
N/A

Indication for prophylaxis

Which patients receive periinterventional antibiotic prophylaxis? (multiple answers possible) [all]
Yes

Which patients receive periinterventional antibiotic prophylaxis? (multiple answers possible) [patients post urinary tract infection/pyelonephritis/urosepsis]
N/A

Which patients receive periinterventional antibiotic prophylaxis? (multiple answers possible) [patients with urinary tract dilation in ultrasound]
N/A

Which patients receive periinterventional antibiotic prophylaxis? (multiple answers possible) [patients with prenatal urinary tract dilation]
N/A

Which patients receive periinterventional antibiotic prophylaxis? (multiple answers possible) [patients post VUR surgery]
N/A

Which patients receive periinterventional antibiotic prophylaxis? (multiple answers possible) [none]
N/A

Are there situations or clinical findings in the patient where you deviate from your prophylaxis standard (medication, dose, duration, age of the patient)? (multiple answers possible, please specify in answer box) [in case of already known vesicoureteral reflux (VUR)]
Yes

Are there situations or clinical findings in the patient where you deviate from your prophylaxis standard (medication, dose, duration, age of the patient)? (multiple answers possible, please specify in answer box) [in case of already known vesicoureteral reflux (VUR)][Comment]
s.u.

Are there situations or clinical findings in the patient where you deviate from your prophylaxis standard (medication, dose, duration, age of the patient)? (multiple answers possible, please specify in answer box) [in patients with recurrent urinary tract infections (UTI)]
Yes

Are there situations or clinical findings in the patient where you deviate from your prophylaxis standard (medication, dose, duration, age of the patient)? (multiple answers possible, please specify in answer box) [in patients with recurrent urinary tract infections (UTI)][Comment]
bei Pat. mit schon bestehender Prophylaxe wird auf die Therapeutische Dosis gesteigert

Are there situations or clinical findings in the patient where you deviate from your prophylaxis standard (medication, dose, duration, age of the patient)? (multiple answers possible, please specify in answer box) [if multidrug-resistant organisms (MDRO) are detected in urine cultures]
No

Are there situations or clinical findings in the patient where you deviate from your prophylaxis standard (medication, dose, duration, age of the patient)? (multiple answers possible, please specify in answer box) [if multidrug-resistant organisms (MDRO) are detected in urine cultures][Comment]

Are there situations or clinical findings in the patient where you deviate from your prophylaxis standard (medication, dose, duration, age of the patient)? (multiple answers possible, please specify in answer box) [in newborns]
No

Are there situations or clinical findings in the patient where you deviate from your prophylaxis standard (medication, dose, duration, age of the patient)? (multiple answers possible, please specify in answer box) [in newborns][Comment]

Are there situations or clinical findings in the patient where you deviate from your prophylaxis standard (medication, dose, duration, age of the patient)? (multiple answers possible, please specify in answer box) [Other]

Are there situations or clinical findings in the patient where you deviate from your prophylaxis standard (medication, dose, duration, age of the patient)? (multiple answers possible, please specify in answer box) [Other comment]

Are there situations in which you use intravenous prophylaxis?
No

Voiding analysis

Are there differences in antibiotic prophylaxis between VUS and VCUG in your department?
No

Is a non-pathological urine strip test required in preparation for VUS/VCUG in your department?
No

Is a non-pathological urine culture required in preparation for VUS/VCUG in your department?
No

Do patients only receive post-procedure prophylaxis if vesicoureteral reflux (VUR) is detected in VUS/VCUG?
No

Do other MUS/MCU findings (e.g. urethral valves) have an influence on post-procedure prophylaxis?
No

End of survey

Thank you for taking the time to answer this survey!

May we contact you personally if we have further questions?
Yes

E-mail address:
melanie.rohmann@med.uni-jena.de

Survey response 97

Response ID
99

Last page
1

Start language
de

Seed
1841783737

Date started
2025-01-28 14:06:19

Date last action
2025-01-28 14:07:41

Standard Operation Procedure

Who fills out this survey?
Pediatrician

Who fills out this survey? [Other]

In which type of hospital are you employed?
Community hospital (>50 beds for pediatric patients)

In which type of hospital are you employed? [Other]

In which country do you work?
De

Does your department have a defined protocol for infection prophylaxis in the context of VUS/VCUG?
Yes

Has this protocol been transformed into a written standard operating procedure (SOP)?
Yes

Which department is the author of the SOP for infection prophylaxis in VUS/VCUG?
Pediatrics

Which department is the author of the SOP for infection prophylaxis in VUS/VCUG? [Other]

Antibiotic Prophylaxis

Are there situations/constellations in which you do not use antibiotic prophylaxis for VUS/VCUG?
Yes

Please specify these situations/constellations: [girl with first uncomplicated UTI without urinary tract dilation in ultrasound]
No

Please specify these situations/constellations: [boy with first uncomplicated UTI without urinary tract dilation in ultrasound]
No

Please specify these situations/constellations: [in patients with low grade urinary tract dilation (up to 2nd grade) and no UTI in medical history]
No

Please specify these situations/constellations: [Other]

If you use antibiotic prophylaxis: which mediaction do you use? (multiple answers and subdivision into age groups possible) [Trimethoprim]
Yes

If you use antibiotic prophylaxis: which mediaction do you use? (multiple answers and subdivision into age groups possible) [Nitrofurantoin]
Yes

If you use antibiotic prophylaxis: which mediaction do you use? (multiple answers and subdivision into age groups possible) [Amoxicillin]
No

If you use antibiotic prophylaxis: which mediaction do you use? (multiple answers and subdivision into age groups possible) [Cefaclor]
No

If you use antibiotic prophylaxis: which mediaction do you use? (multiple answers and subdivision into age groups possible) [Amoxicillin/clavulanic acid]
No

If you use antibiotic prophylaxis: which mediaction do you use? (multiple answers and subdivision into age groups possible) [Other]
No

Trimethoprim: in what dosage?

Trimethoprim: in what dosage? [Other]

Trimethoprim: for which age group do you use trimethoprim?

Trimethoprim: which time schedule do you use? [Day(s) before VUS/VCUG, how many?]
No

Trimethoprim: which time schedule do you use? [Day(s) before VUS/VCUG, how many?][Comment]

Trimethoprim: which time schedule do you use? [Day of VUS/VCUG]
No

Trimethoprim: which time schedule do you use? [Day of VUS/VCUG][Comment]

Trimethoprim: which time schedule do you use? [Day(s) after VUS/VCUG, how many?]
No

Trimethoprim: which time schedule do you use? [Day(s) after VUS/VCUG, how many?][Comment]

Nitrofurantoin: in what dosage?

Nitrofurantoin: in what dosage? [Other]

Nitrofurantoin: for which age group do you use nitrofurantoin?

Nitrofurantoin: which time schedule do you use? [Day(s) before VUS/VCUG, how many?]
No

Nitrofurantoin: which time schedule do you use? [Day(s) before VUS/VCUG, how many?][Comment]

Nitrofurantoin: which time schedule do you use? [Day of VUS/VCUG]
No

Nitrofurantoin: which time schedule do you use? [Day of VUS/VCUG][Comment]

Nitrofurantoin: which time schedule do you use? [Day(s) after VUS/VCUG, how many?]
No

Nitrofurantoin: which time schedule do you use? [Day(s) after VUS/VCUG, how many?][Comment]

Amoxicillin: which time schedule do you use? [Day(s) before VUS/VCUG, how many?]
N/A

Amoxicillin: which time schedule do you use? [Day of VUS/VCUG]
N/A

Amoxicillin: which time schedule do you use? [Day(s) after VUS/VCUG, how many?]
N/A

Cefaclor: which time schedule do you use? [Days(s) before VUS/VCUG, how many?]
N/A

Cefaclor: which time schedule do you use? [Day of VUS/VCUG]
N/A

Cefaclor: which time schedule do you use? [Day(s) after VUS/VCUG, how many?]
N/A

Amoxicillin/clavulanic acid: in what dosage? (Amoxicillin portion) [prophylaktic (10-20 mg/Kg bodyweight/d)]
N/A

Amoxicillin/clavulanic acid: in what dosage? (Amoxicillin portion) [double prophylactic (20-40 mg/Kg bodyweight/d)]
N/A

Amoxicillin/clavulanic acid: in what dosage? (Amoxicillin portion) [therapeutic (80-100 mg/Kg bodyweight/d)]
N/A

Amoxicillin/clavulanic acid: which time schedule do you use? [Day(s) before VUS/VCUG]
N/A

Amoxicillin/clavulanic acid: which time schedule do you use? [Day of VUS/VCUG]
N/A

Amoxicillin/clavulanic acid: which time schedule do you use? [Day(s) after VUS/VCUG]
N/A

Other: which time schedule do you use? [Day(s) before VUS/VCUG, how many?]
N/A

Other: which time schedule do you use? [Day of VUS/VCUG]
N/A

Other: which time schedule do you use? [Day(s) after VUS/VCUG, how many?]
N/A

Indication for prophylaxis

Which patients receive periinterventional antibiotic prophylaxis? (multiple answers possible) [all]
N/A

Which patients receive periinterventional antibiotic prophylaxis? (multiple answers possible) [patients post urinary tract infection/pyelonephritis/urosepsis]
N/A

Which patients receive periinterventional antibiotic prophylaxis? (multiple answers possible) [patients with urinary tract dilation in ultrasound]
N/A

Which patients receive periinterventional antibiotic prophylaxis? (multiple answers possible) [patients with prenatal urinary tract dilation]
N/A

Which patients receive periinterventional antibiotic prophylaxis? (multiple answers possible) [patients post VUR surgery]
N/A

Which patients receive periinterventional antibiotic prophylaxis? (multiple answers possible) [none]
N/A

Are there situations or clinical findings in the patient where you deviate from your prophylaxis standard (medication, dose, duration, age of the patient)? (multiple answers possible, please specify in answer box) [in case of already known vesicoureteral reflux (VUR)]
N/A

Are there situations or clinical findings in the patient where you deviate from your prophylaxis standard (medication, dose, duration, age of the patient)? (multiple answers possible, please specify in answer box) [in patients with recurrent urinary tract infections (UTI)]
N/A

Are there situations or clinical findings in the patient where you deviate from your prophylaxis standard (medication, dose, duration, age of the patient)? (multiple answers possible, please specify in answer box) [if multidrug-resistant organisms (MDRO) are detected in urine cultures]
N/A

Are there situations or clinical findings in the patient where you deviate from your prophylaxis standard (medication, dose, duration, age of the patient)? (multiple answers possible, please specify in answer box) [in newborns]
N/A

Are there situations in which you use intravenous prophylaxis?
N/A

Voiding analysis

Are there differences in antibiotic prophylaxis between VUS and VCUG in your department?
N/A

Is a non-pathological urine strip test required in preparation for VUS/VCUG in your department?
N/A

Is a non-pathological urine culture required in preparation for VUS/VCUG in your department?
N/A

Do patients only receive post-procedure prophylaxis if vesicoureteral reflux (VUR) is detected in VUS/VCUG?
N/A

Do other MUS/MCU findings (e.g. urethral valves) have an influence on post-procedure prophylaxis?
N/A

End of survey

May we contact you personally if we have further questions?
N/A

Survey response 98

Response ID
100

Date submitted
2025-01-28 14:51:10

Last page
5

Start language
de

Seed
2035297155

Date started
2025-01-28 14:06:44

Date last action
2025-01-28 14:51:10

Standard Operation Procedure

Who fills out this survey?
Pediatrician

Who fills out this survey? [Other]

In which type of hospital are you employed?
Community hospital (>50 beds for pediatric patients)

In which type of hospital are you employed? [Other]

In which country do you work?
Deutschland

Does your department have a defined protocol for infection prophylaxis in the context of VUS/VCUG?
Yes

Has this protocol been transformed into a written standard operating procedure (SOP)?
Yes

Which department is the author of the SOP for infection prophylaxis in VUS/VCUG?
Pediatric nephrology

Which department is the author of the SOP for infection prophylaxis in VUS/VCUG? [Other]

Antibiotic Prophylaxis

Are there situations/constellations in which you do not use antibiotic prophylaxis for VUS/VCUG?
Yes

Please specify these situations/constellations: [girl with first uncomplicated UTI without urinary tract dilation in ultrasound]
No

Please specify these situations/constellations: [boy with first uncomplicated UTI without urinary tract dilation in ultrasound]
No

Please specify these situations/constellations: [in patients with low grade urinary tract dilation (up to 2nd grade) and no UTI in medical history]
No

Please specify these situations/constellations: [Other]
unauffälliger Urin vor MCU

If you use antibiotic prophylaxis: which mediaction do you use? (multiple answers and subdivision into age groups possible) [Trimethoprim]
Yes

If you use antibiotic prophylaxis: which mediaction do you use? (multiple answers and subdivision into age groups possible) [Nitrofurantoin]
Yes

If you use antibiotic prophylaxis: which mediaction do you use? (multiple answers and subdivision into age groups possible) [Amoxicillin]
No

If you use antibiotic prophylaxis: which mediaction do you use? (multiple answers and subdivision into age groups possible) [Cefaclor]
Yes

If you use antibiotic prophylaxis: which mediaction do you use? (multiple answers and subdivision into age groups possible) [Amoxicillin/clavulanic acid]
Yes

If you use antibiotic prophylaxis: which mediaction do you use? (multiple answers and subdivision into age groups possible) [Other]
No

Trimethoprim: in what dosage?
therapeutic (6 mg/kg bodyweight/d)

Trimethoprim: in what dosage? [Other]

Trimethoprim: for which age group do you use trimethoprim?
ab 6 Monaten bis 6 Jahre

Trimethoprim: which time schedule do you use? [Day(s) before VUS/VCUG, how many?]
No

Trimethoprim: which time schedule do you use? [Day(s) before VUS/VCUG, how many?][Comment]

Trimethoprim: which time schedule do you use? [Day of VUS/VCUG]
Yes

Trimethoprim: which time schedule do you use? [Day of VUS/VCUG][Comment]

Trimethoprim: which time schedule do you use? [Day(s) after VUS/VCUG, how many?]
Yes

Trimethoprim: which time schedule do you use? [Day(s) after VUS/VCUG, how many?][Comment]
2

Nitrofurantoin: in what dosage?
therapeutic (5 mg/kg bodyweight/d)

Nitrofurantoin: in what dosage? [Other]

Nitrofurantoin: for which age group do you use nitrofurantoin?
ab 6 Monaten

Nitrofurantoin: which time schedule do you use? [Day(s) before VUS/VCUG, how many?]
No

Nitrofurantoin: which time schedule do you use? [Day(s) before VUS/VCUG, how many?][Comment]

Nitrofurantoin: which time schedule do you use? [Day of VUS/VCUG]
Yes

Nitrofurantoin: which time schedule do you use? [Day of VUS/VCUG][Comment]

Nitrofurantoin: which time schedule do you use? [Day(s) after VUS/VCUG, how many?]
Yes

Nitrofurantoin: which time schedule do you use? [Day(s) after VUS/VCUG, how many?][Comment]
2

Amoxicillin: which time schedule do you use? [Day(s) before VUS/VCUG, how many?]
N/A

Amoxicillin: which time schedule do you use? [Day of VUS/VCUG]
N/A

Amoxicillin: which time schedule do you use? [Day(s) after VUS/VCUG, how many?]
N/A

Cefaclor: in what dosage?
therapeutic (40 mg/kg bodyweight/d)

Cefaclor: in what dosage? [Other]

Cefaclor: for which age group do you use cefaclor?
jede

Cefaclor: which time schedule do you use? [Days(s) before VUS/VCUG, how many?]
No

Cefaclor: which time schedule do you use? [Days(s) before VUS/VCUG, how many?][Comment]

Cefaclor: which time schedule do you use? [Day of VUS/VCUG]
Yes

Cefaclor: which time schedule do you use? [Day of VUS/VCUG][Comment]

Cefaclor: which time schedule do you use? [Day(s) after VUS/VCUG, how many?]
Yes

Cefaclor: which time schedule do you use? [Day(s) after VUS/VCUG, how many?][Comment]
2

Amoxicillin/clavulanic acid: in what dosage? (Amoxicillin portion) [prophylaktic (10-20 mg/Kg bodyweight/d)]
No

Amoxicillin/clavulanic acid: in what dosage? (Amoxicillin portion) [double prophylactic (20-40 mg/Kg bodyweight/d)]
No

Amoxicillin/clavulanic acid: in what dosage? (Amoxicillin portion) [therapeutic (80-100 mg/Kg bodyweight/d)]
Yes

Amoxicillin/clavulanic acid: in what dosage? (Amoxicillin portion) [Other]

Amoxicillin/clavulanic acid: for which age group do you use amoxicillin/clavulanic acid?
jede

Amoxicillin/clavulanic acid: which time schedule do you use? [Day(s) before VUS/VCUG]
No

Amoxicillin/clavulanic acid: which time schedule do you use? [Day(s) before VUS/VCUG][Comment]

Amoxicillin/clavulanic acid: which time schedule do you use? [Day of VUS/VCUG]
Yes

Amoxicillin/clavulanic acid: which time schedule do you use? [Day of VUS/VCUG][Comment]

Amoxicillin/clavulanic acid: which time schedule do you use? [Day(s) after VUS/VCUG]
Yes

Amoxicillin/clavulanic acid: which time schedule do you use? [Day(s) after VUS/VCUG][Comment]
2

Other: which time schedule do you use? [Day(s) before VUS/VCUG, how many?]
N/A

Other: which time schedule do you use? [Day of VUS/VCUG]
N/A

Other: which time schedule do you use? [Day(s) after VUS/VCUG, how many?]
N/A

Indication for prophylaxis

Which patients receive periinterventional antibiotic prophylaxis? (multiple answers possible) [all]
No

Which patients receive periinterventional antibiotic prophylaxis? (multiple answers possible) [patients post urinary tract infection/pyelonephritis/urosepsis]
Yes

Which patients receive periinterventional antibiotic prophylaxis? (multiple answers possible) [patients with urinary tract dilation in ultrasound]
Yes

Which patients receive periinterventional antibiotic prophylaxis? (multiple answers possible) [patients with prenatal urinary tract dilation]
Yes

Which patients receive periinterventional antibiotic prophylaxis? (multiple answers possible) [patients post VUR surgery]
Yes

Which patients receive periinterventional antibiotic prophylaxis? (multiple answers possible) [none]
No

Which patients receive periinterventional antibiotic prophylaxis? (multiple answers possible) [Other]

patients status post urinary tract infection/pyelonephritis/urosepsis: what is the minimum time between infection and VUS/VCUG? (in days)
30

In patients with urinary tract dilation in ultrasound: from what degree on? Image from Beetz R, Bokenkamp A, Brandis M et al (2001) Diagnosis of congenital dilatation of the urinary tract. Consensus Group of the Pediatric Nephrology Working Society in cooperation with the Pediatric Urology Working Group of the German Society of Urology and with the Pediatric Urology Working Society in the Germany Society of Pediatric Surgery. Urologe A 40:495–507 (quiz 8–9)
grade 3

In patients with prenatal urinary tract dilation: from what degree on? Image from Beetz R, Bokenkamp A, Brandis M et al (2001) Diagnosis of congenital dilatation of the urinary tract. Consensus Group of the Pediatric Nephrology Working Society in cooperation with the Pediatric Urology Working Group of the German Society of Urology and with the Pediatric Urology Working Society in the Germany Society of Pediatric Surgery. Urologe A 40:495–507 (quiz 8–9)
grade 4

Are there situations or clinical findings in the patient where you deviate from your prophylaxis standard (medication, dose, duration, age of the patient)? (multiple answers possible, please specify in answer box) [in case of already known vesicoureteral reflux (VUR)]
Yes

Are there situations or clinical findings in the patient where you deviate from your prophylaxis standard (medication, dose, duration, age of the patient)? (multiple answers possible, please specify in answer box) [in case of already known vesicoureteral reflux (VUR)][Comment]
anschließend bei höhergradigen VUR und Zn PN weiter Prophylaxe

Are there situations or clinical findings in the patient where you deviate from your prophylaxis standard (medication, dose, duration, age of the patient)? (multiple answers possible, please specify in answer box) [in patients with recurrent urinary tract infections (UTI)]
No

Are there situations or clinical findings in the patient where you deviate from your prophylaxis standard (medication, dose, duration, age of the patient)? (multiple answers possible, please specify in answer box) [in patients with recurrent urinary tract infections (UTI)][Comment]

Are there situations or clinical findings in the patient where you deviate from your prophylaxis standard (medication, dose, duration, age of the patient)? (multiple answers possible, please specify in answer box) [if multidrug-resistant organisms (MDRO) are detected in urine cultures]
Yes

Are there situations or clinical findings in the patient where you deviate from your prophylaxis standard (medication, dose, duration, age of the patient)? (multiple answers possible, please specify in answer box) [if multidrug-resistant organisms (MDRO) are detected in urine cultures][Comment]
bei Zn PN und höhergradigem VUR

Are there situations or clinical findings in the patient where you deviate from your prophylaxis standard (medication, dose, duration, age of the patient)? (multiple answers possible, please specify in answer box) [in newborns]
No

Are there situations or clinical findings in the patient where you deviate from your prophylaxis standard (medication, dose, duration, age of the patient)? (multiple answers possible, please specify in answer box) [in newborns][Comment]

Are there situations or clinical findings in the patient where you deviate from your prophylaxis standard (medication, dose, duration, age of the patient)? (multiple answers possible, please specify in answer box) [Other]

Are there situations or clinical findings in the patient where you deviate from your prophylaxis standard (medication, dose, duration, age of the patient)? (multiple answers possible, please specify in answer box) [Other comment]

Are there situations in which you use intravenous prophylaxis?
No

Voiding analysis

Are there differences in antibiotic prophylaxis between VUS and VCUG in your department?
No

Is a non-pathological urine strip test required in preparation for VUS/VCUG in your department?
No

Is a non-pathological urine culture required in preparation for VUS/VCUG in your department?
No

Do patients only receive post-procedure prophylaxis if vesicoureteral reflux (VUR) is detected in VUS/VCUG?
No

Do other MUS/MCU findings (e.g. urethral valves) have an influence on post-procedure prophylaxis?
No

End of survey

Thank you for taking the time to answer this survey!

May we contact you personally if we have further questions?
Yes

E-mail address:
frauke.wilkening@helios-gesundheit.de

Survey response 99

Response ID
101

Last page
1

Start language
de

Seed
1033362951

Date started
2025-01-28 14:26:29

Date last action
2025-01-28 14:27:04

Standard Operation Procedure

Who fills out this survey?
Pediatrician

Who fills out this survey? [Other]

In which type of hospital are you employed?
University Hospital

In which type of hospital are you employed? [Other]

In which country do you work?
Deutschland

Does your department have a defined protocol for infection prophylaxis in the context of VUS/VCUG?
Yes

Has this protocol been transformed into a written standard operating procedure (SOP)?
Yes

Which department is the author of the SOP for infection prophylaxis in VUS/VCUG?
Pediatric nephrology

Which department is the author of the SOP for infection prophylaxis in VUS/VCUG? [Other]

Antibiotic Prophylaxis

Are there situations/constellations in which you do not use antibiotic prophylaxis for VUS/VCUG?
N/A

Please specify these situations/constellations: [girl with first uncomplicated UTI without urinary tract dilation in ultrasound]
N/A

Please specify these situations/constellations: [boy with first uncomplicated UTI without urinary tract dilation in ultrasound]
N/A

Please specify these situations/constellations: [in patients with low grade urinary tract dilation (up to 2nd grade) and no UTI in medical history]
N/A

If you use antibiotic prophylaxis: which mediaction do you use? (multiple answers and subdivision into age groups possible) [Trimethoprim]
N/A

If you use antibiotic prophylaxis: which mediaction do you use? (multiple answers and subdivision into age groups possible) [Nitrofurantoin]
N/A

If you use antibiotic prophylaxis: which mediaction do you use? (multiple answers and subdivision into age groups possible) [Amoxicillin]
N/A

If you use antibiotic prophylaxis: which mediaction do you use? (multiple answers and subdivision into age groups possible) [Cefaclor]
N/A

If you use antibiotic prophylaxis: which mediaction do you use? (multiple answers and subdivision into age groups possible) [Amoxicillin/clavulanic acid]
N/A

If you use antibiotic prophylaxis: which mediaction do you use? (multiple answers and subdivision into age groups possible) [Other]
N/A

Trimethoprim: which time schedule do you use? [Day(s) before VUS/VCUG, how many?]
N/A

Trimethoprim: which time schedule do you use? [Day of VUS/VCUG]
N/A

Trimethoprim: which time schedule do you use? [Day(s) after VUS/VCUG, how many?]
N/A

Nitrofurantoin: which time schedule do you use? [Day(s) before VUS/VCUG, how many?]
N/A

Nitrofurantoin: which time schedule do you use? [Day of VUS/VCUG]
N/A

Nitrofurantoin: which time schedule do you use? [Day(s) after VUS/VCUG, how many?]
N/A

Amoxicillin: which time schedule do you use? [Day(s) before VUS/VCUG, how many?]
N/A

Amoxicillin: which time schedule do you use? [Day of VUS/VCUG]
N/A

Amoxicillin: which time schedule do you use? [Day(s) after VUS/VCUG, how many?]
N/A

Cefaclor: which time schedule do you use? [Days(s) before VUS/VCUG, how many?]
N/A

Cefaclor: which time schedule do you use? [Day of VUS/VCUG]
N/A

Cefaclor: which time schedule do you use? [Day(s) after VUS/VCUG, how many?]
N/A

Amoxicillin/clavulanic acid: in what dosage? (Amoxicillin portion) [prophylaktic (10-20 mg/Kg bodyweight/d)]
N/A

Amoxicillin/clavulanic acid: in what dosage? (Amoxicillin portion) [double prophylactic (20-40 mg/Kg bodyweight/d)]
N/A

Amoxicillin/clavulanic acid: in what dosage? (Amoxicillin portion) [therapeutic (80-100 mg/Kg bodyweight/d)]
N/A

Amoxicillin/clavulanic acid: which time schedule do you use? [Day(s) before VUS/VCUG]
N/A

Amoxicillin/clavulanic acid: which time schedule do you use? [Day of VUS/VCUG]
N/A

Amoxicillin/clavulanic acid: which time schedule do you use? [Day(s) after VUS/VCUG]
N/A

Other: which time schedule do you use? [Day(s) before VUS/VCUG, how many?]
N/A

Other: which time schedule do you use? [Day of VUS/VCUG]
N/A

Other: which time schedule do you use? [Day(s) after VUS/VCUG, how many?]
N/A

Indication for prophylaxis

Which patients receive periinterventional antibiotic prophylaxis? (multiple answers possible) [all]
N/A

Which patients receive periinterventional antibiotic prophylaxis? (multiple answers possible) [patients post urinary tract infection/pyelonephritis/urosepsis]
N/A

Which patients receive periinterventional antibiotic prophylaxis? (multiple answers possible) [patients with urinary tract dilation in ultrasound]
N/A

Which patients receive periinterventional antibiotic prophylaxis? (multiple answers possible) [patients with prenatal urinary tract dilation]
N/A

Which patients receive periinterventional antibiotic prophylaxis? (multiple answers possible) [patients post VUR surgery]
N/A

Which patients receive periinterventional antibiotic prophylaxis? (multiple answers possible) [none]
N/A

Are there situations or clinical findings in the patient where you deviate from your prophylaxis standard (medication, dose, duration, age of the patient)? (multiple answers possible, please specify in answer box) [in case of already known vesicoureteral reflux (VUR)]
N/A

Are there situations or clinical findings in the patient where you deviate from your prophylaxis standard (medication, dose, duration, age of the patient)? (multiple answers possible, please specify in answer box) [in patients with recurrent urinary tract infections (UTI)]
N/A

Are there situations or clinical findings in the patient where you deviate from your prophylaxis standard (medication, dose, duration, age of the patient)? (multiple answers possible, please specify in answer box) [if multidrug-resistant organisms (MDRO) are detected in urine cultures]
N/A

Are there situations or clinical findings in the patient where you deviate from your prophylaxis standard (medication, dose, duration, age of the patient)? (multiple answers possible, please specify in answer box) [in newborns]
N/A

Are there situations in which you use intravenous prophylaxis?
N/A

Voiding analysis

Are there differences in antibiotic prophylaxis between VUS and VCUG in your department?
N/A

Is a non-pathological urine strip test required in preparation for VUS/VCUG in your department?
N/A

Is a non-pathological urine culture required in preparation for VUS/VCUG in your department?
N/A

Do patients only receive post-procedure prophylaxis if vesicoureteral reflux (VUR) is detected in VUS/VCUG?
N/A

Do other MUS/MCU findings (e.g. urethral valves) have an influence on post-procedure prophylaxis?
N/A

End of survey

May we contact you personally if we have further questions?
N/A

Survey response 100

Response ID
102

Date submitted
2025-01-28 14:40:47

Last page
5

Start language
de

Seed
1193125860

Date started
2025-01-28 14:36:33

Date last action
2025-01-28 14:40:47

Standard Operation Procedure

Who fills out this survey?
Other

Who fills out this survey? [Other]
Kindernephrologe

In which type of hospital are you employed?
Community hospital (>50 beds for pediatric patients)

In which type of hospital are you employed? [Other]

In which country do you work?
Schweiz

Does your department have a defined protocol for infection prophylaxis in the context of VUS/VCUG?
Yes

Has this protocol been transformed into a written standard operating procedure (SOP)?
No

Antibiotic Prophylaxis

Are there situations/constellations in which you do not use antibiotic prophylaxis for VUS/VCUG?
No

Please specify these situations/constellations: [girl with first uncomplicated UTI without urinary tract dilation in ultrasound]
N/A

Please specify these situations/constellations: [boy with first uncomplicated UTI without urinary tract dilation in ultrasound]
N/A

Please specify these situations/constellations: [in patients with low grade urinary tract dilation (up to 2nd grade) and no UTI in medical history]
N/A

If you use antibiotic prophylaxis: which mediaction do you use? (multiple answers and subdivision into age groups possible) [Trimethoprim]
Yes

If you use antibiotic prophylaxis: which mediaction do you use? (multiple answers and subdivision into age groups possible) [Nitrofurantoin]
No

If you use antibiotic prophylaxis: which mediaction do you use? (multiple answers and subdivision into age groups possible) [Amoxicillin]
No

If you use antibiotic prophylaxis: which mediaction do you use? (multiple answers and subdivision into age groups possible) [Cefaclor]
No

If you use antibiotic prophylaxis: which mediaction do you use? (multiple answers and subdivision into age groups possible) [Amoxicillin/clavulanic acid]
No

If you use antibiotic prophylaxis: which mediaction do you use? (multiple answers and subdivision into age groups possible) [Other]
No

Trimethoprim: in what dosage?
therapeutic (6 mg/kg bodyweight/d)

Trimethoprim: in what dosage? [Other]

Trimethoprim: for which age group do you use trimethoprim?
alle

Trimethoprim: which time schedule do you use? [Day(s) before VUS/VCUG, how many?]
No

Trimethoprim: which time schedule do you use? [Day(s) before VUS/VCUG, how many?][Comment]

Trimethoprim: which time schedule do you use? [Day of VUS/VCUG]
Yes

Trimethoprim: which time schedule do you use? [Day of VUS/VCUG][Comment]

Trimethoprim: which time schedule do you use? [Day(s) after VUS/VCUG, how many?]
No

Trimethoprim: which time schedule do you use? [Day(s) after VUS/VCUG, how many?][Comment]

Nitrofurantoin: which time schedule do you use? [Day(s) before VUS/VCUG, how many?]
N/A

Nitrofurantoin: which time schedule do you use? [Day of VUS/VCUG]
N/A

Nitrofurantoin: which time schedule do you use? [Day(s) after VUS/VCUG, how many?]
N/A

Amoxicillin: which time schedule do you use? [Day(s) before VUS/VCUG, how many?]
N/A

Amoxicillin: which time schedule do you use? [Day of VUS/VCUG]
N/A

Amoxicillin: which time schedule do you use? [Day(s) after VUS/VCUG, how many?]
N/A

Cefaclor: which time schedule do you use? [Days(s) before VUS/VCUG, how many?]
N/A

Cefaclor: which time schedule do you use? [Day of VUS/VCUG]
N/A

Cefaclor: which time schedule do you use? [Day(s) after VUS/VCUG, how many?]
N/A

Amoxicillin/clavulanic acid: in what dosage? (Amoxicillin portion) [prophylaktic (10-20 mg/Kg bodyweight/d)]
N/A

Amoxicillin/clavulanic acid: in what dosage? (Amoxicillin portion) [double prophylactic (20-40 mg/Kg bodyweight/d)]
N/A

Amoxicillin/clavulanic acid: in what dosage? (Amoxicillin portion) [therapeutic (80-100 mg/Kg bodyweight/d)]
N/A

Amoxicillin/clavulanic acid: which time schedule do you use? [Day(s) before VUS/VCUG]
N/A

Amoxicillin/clavulanic acid: which time schedule do you use? [Day of VUS/VCUG]
N/A

Amoxicillin/clavulanic acid: which time schedule do you use? [Day(s) after VUS/VCUG]
N/A

Other: which time schedule do you use? [Day(s) before VUS/VCUG, how many?]
N/A

Other: which time schedule do you use? [Day of VUS/VCUG]
N/A

Other: which time schedule do you use? [Day(s) after VUS/VCUG, how many?]
N/A

Indication for prophylaxis

Which patients receive periinterventional antibiotic prophylaxis? (multiple answers possible) [all]
Yes

Which patients receive periinterventional antibiotic prophylaxis? (multiple answers possible) [patients post urinary tract infection/pyelonephritis/urosepsis]
N/A

Which patients receive periinterventional antibiotic prophylaxis? (multiple answers possible) [patients with urinary tract dilation in ultrasound]
N/A

Which patients receive periinterventional antibiotic prophylaxis? (multiple answers possible) [patients with prenatal urinary tract dilation]
N/A

Which patients receive periinterventional antibiotic prophylaxis? (multiple answers possible) [patients post VUR surgery]
N/A

Which patients receive periinterventional antibiotic prophylaxis? (multiple answers possible) [none]
N/A

Are there situations or clinical findings in the patient where you deviate from your prophylaxis standard (medication, dose, duration, age of the patient)? (multiple answers possible, please specify in answer box) [in case of already known vesicoureteral reflux (VUR)]
No

Are there situations or clinical findings in the patient where you deviate from your prophylaxis standard (medication, dose, duration, age of the patient)? (multiple answers possible, please specify in answer box) [in case of already known vesicoureteral reflux (VUR)][Comment]

Are there situations or clinical findings in the patient where you deviate from your prophylaxis standard (medication, dose, duration, age of the patient)? (multiple answers possible, please specify in answer box) [in patients with recurrent urinary tract infections (UTI)]
No

Are there situations or clinical findings in the patient where you deviate from your prophylaxis standard (medication, dose, duration, age of the patient)? (multiple answers possible, please specify in answer box) [in patients with recurrent urinary tract infections (UTI)][Comment]

Are there situations or clinical findings in the patient where you deviate from your prophylaxis standard (medication, dose, duration, age of the patient)? (multiple answers possible, please specify in answer box) [if multidrug-resistant organisms (MDRO) are detected in urine cultures]
No

Are there situations or clinical findings in the patient where you deviate from your prophylaxis standard (medication, dose, duration, age of the patient)? (multiple answers possible, please specify in answer box) [if multidrug-resistant organisms (MDRO) are detected in urine cultures][Comment]

Are there situations or clinical findings in the patient where you deviate from your prophylaxis standard (medication, dose, duration, age of the patient)? (multiple answers possible, please specify in answer box) [in newborns]
No

Are there situations or clinical findings in the patient where you deviate from your prophylaxis standard (medication, dose, duration, age of the patient)? (multiple answers possible, please specify in answer box) [in newborns][Comment]

Are there situations or clinical findings in the patient where you deviate from your prophylaxis standard (medication, dose, duration, age of the patient)? (multiple answers possible, please specify in answer box) [Other]
nein

Are there situations or clinical findings in the patient where you deviate from your prophylaxis standard (medication, dose, duration, age of the patient)? (multiple answers possible, please specify in answer box) [Other comment]

Are there situations in which you use intravenous prophylaxis?
No

Voiding analysis

Are there differences in antibiotic prophylaxis between VUS and VCUG in your department?
No

Is a non-pathological urine strip test required in preparation for VUS/VCUG in your department?
Yes

What is the maximum amount of days accepted for urine strip test to be performed prior to the exam?
0

Is a non-pathological urine culture required in preparation for VUS/VCUG in your department?
No

Do patients only receive post-procedure prophylaxis if vesicoureteral reflux (VUR) is detected in VUS/VCUG?
No

Do other MUS/MCU findings (e.g. urethral valves) have an influence on post-procedure prophylaxis?
No

End of survey

Thank you for taking the time to answer this survey!

May we contact you personally if we have further questions?
No

Survey response 101

Response ID
103

Last page
0

Start language
de

Seed
1678939687

Date started
2025-01-28 14:42:01

Date last action
2025-01-28 14:42:01

Standard Operation Procedure

Does your department have a defined protocol for infection prophylaxis in the context of VUS/VCUG?
N/A

Has this protocol been transformed into a written standard operating procedure (SOP)?
N/A

Antibiotic Prophylaxis

Are there situations/constellations in which you do not use antibiotic prophylaxis for VUS/VCUG?
N/A

Please specify these situations/constellations: [girl with first uncomplicated UTI without urinary tract dilation in ultrasound]
N/A

Please specify these situations/constellations: [boy with first uncomplicated UTI without urinary tract dilation in ultrasound]
N/A

Please specify these situations/constellations: [in patients with low grade urinary tract dilation (up to 2nd grade) and no UTI in medical history]
N/A

If you use antibiotic prophylaxis: which mediaction do you use? (multiple answers and subdivision into age groups possible) [Trimethoprim]
N/A

If you use antibiotic prophylaxis: which mediaction do you use? (multiple answers and subdivision into age groups possible) [Nitrofurantoin]
N/A

If you use antibiotic prophylaxis: which mediaction do you use? (multiple answers and subdivision into age groups possible) [Amoxicillin]
N/A

If you use antibiotic prophylaxis: which mediaction do you use? (multiple answers and subdivision into age groups possible) [Cefaclor]
N/A

If you use antibiotic prophylaxis: which mediaction do you use? (multiple answers and subdivision into age groups possible) [Amoxicillin/clavulanic acid]
N/A

If you use antibiotic prophylaxis: which mediaction do you use? (multiple answers and subdivision into age groups possible) [Other]
N/A

Trimethoprim: which time schedule do you use? [Day(s) before VUS/VCUG, how many?]
N/A

Trimethoprim: which time schedule do you use? [Day of VUS/VCUG]
N/A

Trimethoprim: which time schedule do you use? [Day(s) after VUS/VCUG, how many?]
N/A

Nitrofurantoin: which time schedule do you use? [Day(s) before VUS/VCUG, how many?]
N/A

Nitrofurantoin: which time schedule do you use? [Day of VUS/VCUG]
N/A

Nitrofurantoin: which time schedule do you use? [Day(s) after VUS/VCUG, how many?]
N/A

Amoxicillin: which time schedule do you use? [Day(s) before VUS/VCUG, how many?]
N/A

Amoxicillin: which time schedule do you use? [Day of VUS/VCUG]
N/A

Amoxicillin: which time schedule do you use? [Day(s) after VUS/VCUG, how many?]
N/A

Cefaclor: which time schedule do you use? [Days(s) before VUS/VCUG, how many?]
N/A

Cefaclor: which time schedule do you use? [Day of VUS/VCUG]
N/A

Cefaclor: which time schedule do you use? [Day(s) after VUS/VCUG, how many?]
N/A

Amoxicillin/clavulanic acid: in what dosage? (Amoxicillin portion) [prophylaktic (10-20 mg/Kg bodyweight/d)]
N/A

Amoxicillin/clavulanic acid: in what dosage? (Amoxicillin portion) [double prophylactic (20-40 mg/Kg bodyweight/d)]
N/A

Amoxicillin/clavulanic acid: in what dosage? (Amoxicillin portion) [therapeutic (80-100 mg/Kg bodyweight/d)]
N/A

Amoxicillin/clavulanic acid: which time schedule do you use? [Day(s) before VUS/VCUG]
N/A

Amoxicillin/clavulanic acid: which time schedule do you use? [Day of VUS/VCUG]
N/A

Amoxicillin/clavulanic acid: which time schedule do you use? [Day(s) after VUS/VCUG]
N/A

Other: which time schedule do you use? [Day(s) before VUS/VCUG, how many?]
N/A

Other: which time schedule do you use? [Day of VUS/VCUG]
N/A

Other: which time schedule do you use? [Day(s) after VUS/VCUG, how many?]
N/A

Indication for prophylaxis

Which patients receive periinterventional antibiotic prophylaxis? (multiple answers possible) [all]
N/A

Which patients receive periinterventional antibiotic prophylaxis? (multiple answers possible) [patients post urinary tract infection/pyelonephritis/urosepsis]
N/A

Which patients receive periinterventional antibiotic prophylaxis? (multiple answers possible) [patients with urinary tract dilation in ultrasound]
N/A

Which patients receive periinterventional antibiotic prophylaxis? (multiple answers possible) [patients with prenatal urinary tract dilation]
N/A

Which patients receive periinterventional antibiotic prophylaxis? (multiple answers possible) [patients post VUR surgery]
N/A

Which patients receive periinterventional antibiotic prophylaxis? (multiple answers possible) [none]
N/A

Are there situations or clinical findings in the patient where you deviate from your prophylaxis standard (medication, dose, duration, age of the patient)? (multiple answers possible, please specify in answer box) [in case of already known vesicoureteral reflux (VUR)]
N/A

Are there situations or clinical findings in the patient where you deviate from your prophylaxis standard (medication, dose, duration, age of the patient)? (multiple answers possible, please specify in answer box) [in patients with recurrent urinary tract infections (UTI)]
N/A

Are there situations or clinical findings in the patient where you deviate from your prophylaxis standard (medication, dose, duration, age of the patient)? (multiple answers possible, please specify in answer box) [if multidrug-resistant organisms (MDRO) are detected in urine cultures]
N/A

Are there situations or clinical findings in the patient where you deviate from your prophylaxis standard (medication, dose, duration, age of the patient)? (multiple answers possible, please specify in answer box) [in newborns]
N/A

Are there situations in which you use intravenous prophylaxis?
N/A

Voiding analysis

Are there differences in antibiotic prophylaxis between VUS and VCUG in your department?
N/A

Is a non-pathological urine strip test required in preparation for VUS/VCUG in your department?
N/A

Is a non-pathological urine culture required in preparation for VUS/VCUG in your department?
N/A

Do patients only receive post-procedure prophylaxis if vesicoureteral reflux (VUR) is detected in VUS/VCUG?
N/A

Do other MUS/MCU findings (e.g. urethral valves) have an influence on post-procedure prophylaxis?
N/A

End of survey

May we contact you personally if we have further questions?
N/A

Survey response 102

Response ID
104

Date submitted
2025-01-28 15:46:19

Last page
5

Start language
de

Seed
231313689

Date started
2025-01-28 15:37:27

Date last action
2025-01-28 15:46:19

Standard Operation Procedure

Who fills out this survey?
Pediatrician

Who fills out this survey? [Other]

In which type of hospital are you employed?
University Hospital

In which type of hospital are you employed? [Other]

In which country do you work?
Deutschland

Does your department have a defined protocol for infection prophylaxis in the context of VUS/VCUG?
No

Has this protocol been transformed into a written standard operating procedure (SOP)?
N/A

Antibiotic Prophylaxis

Are there situations/constellations in which you do not use antibiotic prophylaxis for VUS/VCUG?
Yes

Please specify these situations/constellations: [girl with first uncomplicated UTI without urinary tract dilation in ultrasound]
Yes

Please specify these situations/constellations: [boy with first uncomplicated UTI without urinary tract dilation in ultrasound]
Yes

Please specify these situations/constellations: [in patients with low grade urinary tract dilation (up to 2nd grade) and no UTI in medical history]
Yes

Please specify these situations/constellations: [Other]

If you use antibiotic prophylaxis: which mediaction do you use? (multiple answers and subdivision into age groups possible) [Trimethoprim]
Yes

If you use antibiotic prophylaxis: which mediaction do you use? (multiple answers and subdivision into age groups possible) [Nitrofurantoin]
No

If you use antibiotic prophylaxis: which mediaction do you use? (multiple answers and subdivision into age groups possible) [Amoxicillin]
No

If you use antibiotic prophylaxis: which mediaction do you use? (multiple answers and subdivision into age groups possible) [Cefaclor]
Yes

If you use antibiotic prophylaxis: which mediaction do you use? (multiple answers and subdivision into age groups possible) [Amoxicillin/clavulanic acid]
No

If you use antibiotic prophylaxis: which mediaction do you use? (multiple answers and subdivision into age groups possible) [Other]
No

Trimethoprim: in what dosage?
therapeutic (6 mg/kg bodyweight/d)

Trimethoprim: in what dosage? [Other]

Trimethoprim: for which age group do you use trimethoprim?
ab 6 Wochen

Trimethoprim: which time schedule do you use? [Day(s) before VUS/VCUG, how many?]
No

Trimethoprim: which time schedule do you use? [Day(s) before VUS/VCUG, how many?][Comment]

Trimethoprim: which time schedule do you use? [Day of VUS/VCUG]
Yes

Trimethoprim: which time schedule do you use? [Day of VUS/VCUG][Comment]

Trimethoprim: which time schedule do you use? [Day(s) after VUS/VCUG, how many?]
Yes

Trimethoprim: which time schedule do you use? [Day(s) after VUS/VCUG, how many?][Comment]
2

Nitrofurantoin: which time schedule do you use? [Day(s) before VUS/VCUG, how many?]
N/A

Nitrofurantoin: which time schedule do you use? [Day of VUS/VCUG]
N/A

Nitrofurantoin: which time schedule do you use? [Day(s) after VUS/VCUG, how many?]
N/A

Amoxicillin: which time schedule do you use? [Day(s) before VUS/VCUG, how many?]
N/A

Amoxicillin: which time schedule do you use? [Day of VUS/VCUG]
N/A

Amoxicillin: which time schedule do you use? [Day(s) after VUS/VCUG, how many?]
N/A

Cefaclor: in what dosage?
therapeutic (40 mg/kg bodyweight/d)

Cefaclor: in what dosage? [Other]

Cefaclor: for which age group do you use cefaclor?
bis 6 Wochen

Cefaclor: which time schedule do you use? [Days(s) before VUS/VCUG, how many?]
No

Cefaclor: which time schedule do you use? [Days(s) before VUS/VCUG, how many?][Comment]

Cefaclor: which time schedule do you use? [Day of VUS/VCUG]
Yes

Cefaclor: which time schedule do you use? [Day of VUS/VCUG][Comment]

Cefaclor: which time schedule do you use? [Day(s) after VUS/VCUG, how many?]
Yes

Cefaclor: which time schedule do you use? [Day(s) after VUS/VCUG, how many?][Comment]
2

Amoxicillin/clavulanic acid: in what dosage? (Amoxicillin portion) [prophylaktic (10-20 mg/Kg bodyweight/d)]
N/A

Amoxicillin/clavulanic acid: in what dosage? (Amoxicillin portion) [double prophylactic (20-40 mg/Kg bodyweight/d)]
N/A

Amoxicillin/clavulanic acid: in what dosage? (Amoxicillin portion) [therapeutic (80-100 mg/Kg bodyweight/d)]
N/A

Amoxicillin/clavulanic acid: which time schedule do you use? [Day(s) before VUS/VCUG]
N/A

Amoxicillin/clavulanic acid: which time schedule do you use? [Day of VUS/VCUG]
N/A

Amoxicillin/clavulanic acid: which time schedule do you use? [Day(s) after VUS/VCUG]
N/A

Other: which time schedule do you use? [Day(s) before VUS/VCUG, how many?]
N/A

Other: which time schedule do you use? [Day of VUS/VCUG]
N/A

Other: which time schedule do you use? [Day(s) after VUS/VCUG, how many?]
N/A

Indication for prophylaxis

Which patients receive periinterventional antibiotic prophylaxis? (multiple answers possible) [all]
No

Which patients receive periinterventional antibiotic prophylaxis? (multiple answers possible) [patients post urinary tract infection/pyelonephritis/urosepsis]
Yes

Which patients receive periinterventional antibiotic prophylaxis? (multiple answers possible) [patients with urinary tract dilation in ultrasound]
Yes

Which patients receive periinterventional antibiotic prophylaxis? (multiple answers possible) [patients with prenatal urinary tract dilation]
Yes

Which patients receive periinterventional antibiotic prophylaxis? (multiple answers possible) [patients post VUR surgery]
Yes

Which patients receive periinterventional antibiotic prophylaxis? (multiple answers possible) [none]
No

Which patients receive periinterventional antibiotic prophylaxis? (multiple answers possible) [Other]

patients status post urinary tract infection/pyelonephritis/urosepsis: what is the minimum time between infection and VUS/VCUG? (in days)
14

In patients with urinary tract dilation in ultrasound: from what degree on? Image from Beetz R, Bokenkamp A, Brandis M et al (2001) Diagnosis of congenital dilatation of the urinary tract. Consensus Group of the Pediatric Nephrology Working Society in cooperation with the Pediatric Urology Working Group of the German Society of Urology and with the Pediatric Urology Working Society in the Germany Society of Pediatric Surgery. Urologe A 40:495–507 (quiz 8–9)
grade 2

In patients with prenatal urinary tract dilation: from what degree on? Image from Beetz R, Bokenkamp A, Brandis M et al (2001) Diagnosis of congenital dilatation of the urinary tract. Consensus Group of the Pediatric Nephrology Working Society in cooperation with the Pediatric Urology Working Group of the German Society of Urology and with the Pediatric Urology Working Society in the Germany Society of Pediatric Surgery. Urologe A 40:495–507 (quiz 8–9)
grade 2

Are there situations or clinical findings in the patient where you deviate from your prophylaxis standard (medication, dose, duration, age of the patient)? (multiple answers possible, please specify in answer box) [in case of already known vesicoureteral reflux (VUR)]
Yes

Are there situations or clinical findings in the patient where you deviate from your prophylaxis standard (medication, dose, duration, age of the patient)? (multiple answers possible, please specify in answer box) [in case of already known vesicoureteral reflux (VUR)][Comment]
bei bestehender Prophylaxe erfolgt die periinterventionelle antibiotische Prophylaxe mit dem selben Medikament in therapeut. Dosis

Are there situations or clinical findings in the patient where you deviate from your prophylaxis standard (medication, dose, duration, age of the patient)? (multiple answers possible, please specify in answer box) [in patients with recurrent urinary tract infections (UTI)]
No

Are there situations or clinical findings in the patient where you deviate from your prophylaxis standard (medication, dose, duration, age of the patient)? (multiple answers possible, please specify in answer box) [in patients with recurrent urinary tract infections (UTI)][Comment]

Are there situations or clinical findings in the patient where you deviate from your prophylaxis standard (medication, dose, duration, age of the patient)? (multiple answers possible, please specify in answer box) [if multidrug-resistant organisms (MDRO) are detected in urine cultures]
Yes

Are there situations or clinical findings in the patient where you deviate from your prophylaxis standard (medication, dose, duration, age of the patient)? (multiple answers possible, please specify in answer box) [if multidrug-resistant organisms (MDRO) are detected in urine cultures][Comment]
Medikament angepasst an Resistogramm

Are there situations or clinical findings in the patient where you deviate from your prophylaxis standard (medication, dose, duration, age of the patient)? (multiple answers possible, please specify in answer box) [in newborns]
No

Are there situations or clinical findings in the patient where you deviate from your prophylaxis standard (medication, dose, duration, age of the patient)? (multiple answers possible, please specify in answer box) [in newborns][Comment]

Are there situations or clinical findings in the patient where you deviate from your prophylaxis standard (medication, dose, duration, age of the patient)? (multiple answers possible, please specify in answer box) [Other]

Are there situations or clinical findings in the patient where you deviate from your prophylaxis standard (medication, dose, duration, age of the patient)? (multiple answers possible, please specify in answer box) [Other comment]

Are there situations in which you use intravenous prophylaxis?
No

Voiding analysis

Are there differences in antibiotic prophylaxis between VUS and VCUG in your department?
No

Is a non-pathological urine strip test required in preparation for VUS/VCUG in your department?
No

Is a non-pathological urine culture required in preparation for VUS/VCUG in your department?
No

Do patients only receive post-procedure prophylaxis if vesicoureteral reflux (VUR) is detected in VUS/VCUG?
No

Do other MUS/MCU findings (e.g. urethral valves) have an influence on post-procedure prophylaxis?
No

End of survey

Thank you for taking the time to answer this survey!

May we contact you personally if we have further questions?
No

Survey response 103

Response ID
105

Date submitted
2025-01-28 16:03:37

Last page
5

Start language
de

Seed
1079895112

Date started
2025-01-28 15:57:33

Date last action
2025-01-28 16:03:37

Standard Operation Procedure

Who fills out this survey?
Pediatrician

Who fills out this survey? [Other]

In which type of hospital are you employed?
University Hospital

In which type of hospital are you employed? [Other]

In which country do you work?
Deutschland

Does your department have a defined protocol for infection prophylaxis in the context of VUS/VCUG?
Yes

Has this protocol been transformed into a written standard operating procedure (SOP)?
No

Antibiotic Prophylaxis

Are there situations/constellations in which you do not use antibiotic prophylaxis for VUS/VCUG?
Yes

Please specify these situations/constellations: [girl with first uncomplicated UTI without urinary tract dilation in ultrasound]
No

Please specify these situations/constellations: [boy with first uncomplicated UTI without urinary tract dilation in ultrasound]
No

Please specify these situations/constellations: [in patients with low grade urinary tract dilation (up to 2nd grade) and no UTI in medical history]
No

Please specify these situations/constellations: [Other]
Eltern wünschen keine Prophylaxe

If you use antibiotic prophylaxis: which mediaction do you use? (multiple answers and subdivision into age groups possible) [Trimethoprim]
Yes

If you use antibiotic prophylaxis: which mediaction do you use? (multiple answers and subdivision into age groups possible) [Nitrofurantoin]
Yes

If you use antibiotic prophylaxis: which mediaction do you use? (multiple answers and subdivision into age groups possible) [Amoxicillin]
No

If you use antibiotic prophylaxis: which mediaction do you use? (multiple answers and subdivision into age groups possible) [Cefaclor]
Yes

If you use antibiotic prophylaxis: which mediaction do you use? (multiple answers and subdivision into age groups possible) [Amoxicillin/clavulanic acid]
No

If you use antibiotic prophylaxis: which mediaction do you use? (multiple answers and subdivision into age groups possible) [Other]
No

Trimethoprim: in what dosage?
therapeutic (6 mg/kg bodyweight/d)

Trimethoprim: in what dosage? [Other]

Trimethoprim: for which age group do you use trimethoprim?
ab der 6. Lebenswoche

Trimethoprim: which time schedule do you use? [Day(s) before VUS/VCUG, how many?]
No

Trimethoprim: which time schedule do you use? [Day(s) before VUS/VCUG, how many?][Comment]

Trimethoprim: which time schedule do you use? [Day of VUS/VCUG]
Yes

Trimethoprim: which time schedule do you use? [Day of VUS/VCUG][Comment]

Trimethoprim: which time schedule do you use? [Day(s) after VUS/VCUG, how many?]
Yes

Trimethoprim: which time schedule do you use? [Day(s) after VUS/VCUG, how many?][Comment]
1-2

Nitrofurantoin: in what dosage?
therapeutic (5 mg/kg bodyweight/d)

Nitrofurantoin: in what dosage? [Other]

Nitrofurantoin: for which age group do you use nitrofurantoin?
ab 3. Lebensmonat

Nitrofurantoin: which time schedule do you use? [Day(s) before VUS/VCUG, how many?]
No

Nitrofurantoin: which time schedule do you use? [Day(s) before VUS/VCUG, how many?][Comment]

Nitrofurantoin: which time schedule do you use? [Day of VUS/VCUG]
Yes

Nitrofurantoin: which time schedule do you use? [Day of VUS/VCUG][Comment]

Nitrofurantoin: which time schedule do you use? [Day(s) after VUS/VCUG, how many?]
Yes

Nitrofurantoin: which time schedule do you use? [Day(s) after VUS/VCUG, how many?][Comment]
1 - 2

Amoxicillin: which time schedule do you use? [Day(s) before VUS/VCUG, how many?]
N/A

Amoxicillin: which time schedule do you use? [Day of VUS/VCUG]
N/A

Amoxicillin: which time schedule do you use? [Day(s) after VUS/VCUG, how many?]
N/A

Cefaclor: in what dosage?
therapeutic (40 mg/kg bodyweight/d)

Cefaclor: in what dosage? [Other]

Cefaclor: for which age group do you use cefaclor?
vor der 6. Lebenswoche

Cefaclor: which time schedule do you use? [Days(s) before VUS/VCUG, how many?]
No

Cefaclor: which time schedule do you use? [Days(s) before VUS/VCUG, how many?][Comment]

Cefaclor: which time schedule do you use? [Day of VUS/VCUG]
Yes

Cefaclor: which time schedule do you use? [Day of VUS/VCUG][Comment]

Cefaclor: which time schedule do you use? [Day(s) after VUS/VCUG, how many?]
Yes

Cefaclor: which time schedule do you use? [Day(s) after VUS/VCUG, how many?][Comment]
1-2

Amoxicillin/clavulanic acid: in what dosage? (Amoxicillin portion) [prophylaktic (10-20 mg/Kg bodyweight/d)]
N/A

Amoxicillin/clavulanic acid: in what dosage? (Amoxicillin portion) [double prophylactic (20-40 mg/Kg bodyweight/d)]
N/A

Amoxicillin/clavulanic acid: in what dosage? (Amoxicillin portion) [therapeutic (80-100 mg/Kg bodyweight/d)]
N/A

Amoxicillin/clavulanic acid: which time schedule do you use? [Day(s) before VUS/VCUG]
N/A

Amoxicillin/clavulanic acid: which time schedule do you use? [Day of VUS/VCUG]
N/A

Amoxicillin/clavulanic acid: which time schedule do you use? [Day(s) after VUS/VCUG]
N/A

Other: which time schedule do you use? [Day(s) before VUS/VCUG, how many?]
N/A

Other: which time schedule do you use? [Day of VUS/VCUG]
N/A

Other: which time schedule do you use? [Day(s) after VUS/VCUG, how many?]
N/A

Indication for prophylaxis

Which patients receive periinterventional antibiotic prophylaxis? (multiple answers possible) [all]
Yes

Which patients receive periinterventional antibiotic prophylaxis? (multiple answers possible) [patients post urinary tract infection/pyelonephritis/urosepsis]
N/A

Which patients receive periinterventional antibiotic prophylaxis? (multiple answers possible) [patients with urinary tract dilation in ultrasound]
N/A

Which patients receive periinterventional antibiotic prophylaxis? (multiple answers possible) [patients with prenatal urinary tract dilation]
N/A

Which patients receive periinterventional antibiotic prophylaxis? (multiple answers possible) [patients post VUR surgery]
N/A

Which patients receive periinterventional antibiotic prophylaxis? (multiple answers possible) [none]
N/A

Are there situations or clinical findings in the patient where you deviate from your prophylaxis standard (medication, dose, duration, age of the patient)? (multiple answers possible, please specify in answer box) [in case of already known vesicoureteral reflux (VUR)]
No

Are there situations or clinical findings in the patient where you deviate from your prophylaxis standard (medication, dose, duration, age of the patient)? (multiple answers possible, please specify in answer box) [in case of already known vesicoureteral reflux (VUR)][Comment]

Are there situations or clinical findings in the patient where you deviate from your prophylaxis standard (medication, dose, duration, age of the patient)? (multiple answers possible, please specify in answer box) [in patients with recurrent urinary tract infections (UTI)]
No

Are there situations or clinical findings in the patient where you deviate from your prophylaxis standard (medication, dose, duration, age of the patient)? (multiple answers possible, please specify in answer box) [in patients with recurrent urinary tract infections (UTI)][Comment]

Are there situations or clinical findings in the patient where you deviate from your prophylaxis standard (medication, dose, duration, age of the patient)? (multiple answers possible, please specify in answer box) [if multidrug-resistant organisms (MDRO) are detected in urine cultures]
Yes

Are there situations or clinical findings in the patient where you deviate from your prophylaxis standard (medication, dose, duration, age of the patient)? (multiple answers possible, please specify in answer box) [if multidrug-resistant organisms (MDRO) are detected in urine cultures][Comment]

Are there situations or clinical findings in the patient where you deviate from your prophylaxis standard (medication, dose, duration, age of the patient)? (multiple answers possible, please specify in answer box) [in newborns]
No

Are there situations or clinical findings in the patient where you deviate from your prophylaxis standard (medication, dose, duration, age of the patient)? (multiple answers possible, please specify in answer box) [in newborns][Comment]

Are there situations or clinical findings in the patient where you deviate from your prophylaxis standard (medication, dose, duration, age of the patient)? (multiple answers possible, please specify in answer box) [Other]

Are there situations or clinical findings in the patient where you deviate from your prophylaxis standard (medication, dose, duration, age of the patient)? (multiple answers possible, please specify in answer box) [Other comment]

Are there situations in which you use intravenous prophylaxis?
No

Voiding analysis

Are there differences in antibiotic prophylaxis between VUS and VCUG in your department?
No

Is a non-pathological urine strip test required in preparation for VUS/VCUG in your department?
No

Is a non-pathological urine culture required in preparation for VUS/VCUG in your department?
No

Do patients only receive post-procedure prophylaxis if vesicoureteral reflux (VUR) is detected in VUS/VCUG?
No

Do other MUS/MCU findings (e.g. urethral valves) have an influence on post-procedure prophylaxis?
No

End of survey

Thank you for taking the time to answer this survey!

May we contact you personally if we have further questions?
Yes

E-mail address:
swantje.hagemann@uksh.de

Survey response 104

Response ID
106

Last page
0

Start language
de

Seed
1290172503

Date started
2025-01-28 16:31:50

Date last action
2025-01-28 16:31:50

Standard Operation Procedure

Does your department have a defined protocol for infection prophylaxis in the context of VUS/VCUG?
N/A

Has this protocol been transformed into a written standard operating procedure (SOP)?
N/A

Antibiotic Prophylaxis

Are there situations/constellations in which you do not use antibiotic prophylaxis for VUS/VCUG?
N/A

Please specify these situations/constellations: [girl with first uncomplicated UTI without urinary tract dilation in ultrasound]
N/A

Please specify these situations/constellations: [boy with first uncomplicated UTI without urinary tract dilation in ultrasound]
N/A

Please specify these situations/constellations: [in patients with low grade urinary tract dilation (up to 2nd grade) and no UTI in medical history]
N/A

If you use antibiotic prophylaxis: which mediaction do you use? (multiple answers and subdivision into age groups possible) [Trimethoprim]
N/A

If you use antibiotic prophylaxis: which mediaction do you use? (multiple answers and subdivision into age groups possible) [Nitrofurantoin]
N/A

If you use antibiotic prophylaxis: which mediaction do you use? (multiple answers and subdivision into age groups possible) [Amoxicillin]
N/A

If you use antibiotic prophylaxis: which mediaction do you use? (multiple answers and subdivision into age groups possible) [Cefaclor]
N/A

If you use antibiotic prophylaxis: which mediaction do you use? (multiple answers and subdivision into age groups possible) [Amoxicillin/clavulanic acid]
N/A

If you use antibiotic prophylaxis: which mediaction do you use? (multiple answers and subdivision into age groups possible) [Other]
N/A

Trimethoprim: which time schedule do you use? [Day(s) before VUS/VCUG, how many?]
N/A

Trimethoprim: which time schedule do you use? [Day of VUS/VCUG]
N/A

Trimethoprim: which time schedule do you use? [Day(s) after VUS/VCUG, how many?]
N/A

Nitrofurantoin: which time schedule do you use? [Day(s) before VUS/VCUG, how many?]
N/A

Nitrofurantoin: which time schedule do you use? [Day of VUS/VCUG]
N/A

Nitrofurantoin: which time schedule do you use? [Day(s) after VUS/VCUG, how many?]
N/A

Amoxicillin: which time schedule do you use? [Day(s) before VUS/VCUG, how many?]
N/A

Amoxicillin: which time schedule do you use? [Day of VUS/VCUG]
N/A

Amoxicillin: which time schedule do you use? [Day(s) after VUS/VCUG, how many?]
N/A

Cefaclor: which time schedule do you use? [Days(s) before VUS/VCUG, how many?]
N/A

Cefaclor: which time schedule do you use? [Day of VUS/VCUG]
N/A

Cefaclor: which time schedule do you use? [Day(s) after VUS/VCUG, how many?]
N/A

Amoxicillin/clavulanic acid: in what dosage? (Amoxicillin portion) [prophylaktic (10-20 mg/Kg bodyweight/d)]
N/A

Amoxicillin/clavulanic acid: in what dosage? (Amoxicillin portion) [double prophylactic (20-40 mg/Kg bodyweight/d)]
N/A

Amoxicillin/clavulanic acid: in what dosage? (Amoxicillin portion) [therapeutic (80-100 mg/Kg bodyweight/d)]
N/A

Amoxicillin/clavulanic acid: which time schedule do you use? [Day(s) before VUS/VCUG]
N/A

Amoxicillin/clavulanic acid: which time schedule do you use? [Day of VUS/VCUG]
N/A

Amoxicillin/clavulanic acid: which time schedule do you use? [Day(s) after VUS/VCUG]
N/A

Other: which time schedule do you use? [Day(s) before VUS/VCUG, how many?]
N/A

Other: which time schedule do you use? [Day of VUS/VCUG]
N/A

Other: which time schedule do you use? [Day(s) after VUS/VCUG, how many?]
N/A

Indication for prophylaxis

Which patients receive periinterventional antibiotic prophylaxis? (multiple answers possible) [all]
N/A

Which patients receive periinterventional antibiotic prophylaxis? (multiple answers possible) [patients post urinary tract infection/pyelonephritis/urosepsis]
N/A

Which patients receive periinterventional antibiotic prophylaxis? (multiple answers possible) [patients with urinary tract dilation in ultrasound]
N/A

Which patients receive periinterventional antibiotic prophylaxis? (multiple answers possible) [patients with prenatal urinary tract dilation]
N/A

Which patients receive periinterventional antibiotic prophylaxis? (multiple answers possible) [patients post VUR surgery]
N/A

Which patients receive periinterventional antibiotic prophylaxis? (multiple answers possible) [none]
N/A

Are there situations or clinical findings in the patient where you deviate from your prophylaxis standard (medication, dose, duration, age of the patient)? (multiple answers possible, please specify in answer box) [in case of already known vesicoureteral reflux (VUR)]
N/A

Are there situations or clinical findings in the patient where you deviate from your prophylaxis standard (medication, dose, duration, age of the patient)? (multiple answers possible, please specify in answer box) [in patients with recurrent urinary tract infections (UTI)]
N/A

Are there situations or clinical findings in the patient where you deviate from your prophylaxis standard (medication, dose, duration, age of the patient)? (multiple answers possible, please specify in answer box) [if multidrug-resistant organisms (MDRO) are detected in urine cultures]
N/A

Are there situations or clinical findings in the patient where you deviate from your prophylaxis standard (medication, dose, duration, age of the patient)? (multiple answers possible, please specify in answer box) [in newborns]
N/A

Are there situations in which you use intravenous prophylaxis?
N/A

Voiding analysis

Are there differences in antibiotic prophylaxis between VUS and VCUG in your department?
N/A

Is a non-pathological urine strip test required in preparation for VUS/VCUG in your department?
N/A

Is a non-pathological urine culture required in preparation for VUS/VCUG in your department?
N/A

Do patients only receive post-procedure prophylaxis if vesicoureteral reflux (VUR) is detected in VUS/VCUG?
N/A

Do other MUS/MCU findings (e.g. urethral valves) have an influence on post-procedure prophylaxis?
N/A

End of survey

May we contact you personally if we have further questions?
N/A

Survey response 105

Response ID
107

Date submitted
2025-01-28 17:04:38

Last page
5

Start language
de

Seed
154525132

Date started
2025-01-28 16:45:08

Date last action
2025-01-28 17:04:38

Standard Operation Procedure

Who fills out this survey?
Pediatrician

Who fills out this survey? [Other]

In which type of hospital are you employed?
Community hospital (>50 beds for pediatric patients)

In which type of hospital are you employed? [Other]

In which country do you work?
Deutschland

Does your department have a defined protocol for infection prophylaxis in the context of VUS/VCUG?
No

Has this protocol been transformed into a written standard operating procedure (SOP)?
N/A

Antibiotic Prophylaxis

Are there situations/constellations in which you do not use antibiotic prophylaxis for VUS/VCUG?
Yes

Please specify these situations/constellations: [girl with first uncomplicated UTI without urinary tract dilation in ultrasound]
No

Please specify these situations/constellations: [boy with first uncomplicated UTI without urinary tract dilation in ultrasound]
No

Please specify these situations/constellations: [in patients with low grade urinary tract dilation (up to 2nd grade) and no UTI in medical history]
No

Please specify these situations/constellations: [Other]
Immer wenn Kind ohen Prophylaxe zur Refluxprüfung vorgestellt wird. Danach abhängig vom Ergebnis der Refluxprüfung

If you use antibiotic prophylaxis: which mediaction do you use? (multiple answers and subdivision into age groups possible) [Trimethoprim]
Yes

If you use antibiotic prophylaxis: which mediaction do you use? (multiple answers and subdivision into age groups possible) [Nitrofurantoin]
Yes

If you use antibiotic prophylaxis: which mediaction do you use? (multiple answers and subdivision into age groups possible) [Amoxicillin]
No

If you use antibiotic prophylaxis: which mediaction do you use? (multiple answers and subdivision into age groups possible) [Cefaclor]
Yes

If you use antibiotic prophylaxis: which mediaction do you use? (multiple answers and subdivision into age groups possible) [Amoxicillin/clavulanic acid]
No

If you use antibiotic prophylaxis: which mediaction do you use? (multiple answers and subdivision into age groups possible) [Other]
Yes

Trimethoprim: in what dosage?
prophylactic (2 mg/kg bodyweight/d)

Trimethoprim: in what dosage? [Other]

Trimethoprim: for which age group do you use trimethoprim?
> 12 Wochen

Trimethoprim: which time schedule do you use? [Day(s) before VUS/VCUG, how many?]
Yes

Trimethoprim: which time schedule do you use? [Day(s) before VUS/VCUG, how many?][Comment]
Prophylaxe besteht bereits mit Indikationsstellung zur Refluxprüfung

Trimethoprim: which time schedule do you use? [Day of VUS/VCUG]
No

Trimethoprim: which time schedule do you use? [Day of VUS/VCUG][Comment]

Trimethoprim: which time schedule do you use? [Day(s) after VUS/VCUG, how many?]
Yes

Trimethoprim: which time schedule do you use? [Day(s) after VUS/VCUG, how many?][Comment]
3

Nitrofurantoin: in what dosage?
prophylactic (1-2 mg/kg bodyweight/d)

Nitrofurantoin: in what dosage? [Other]

Nitrofurantoin: for which age group do you use nitrofurantoin?
>12 Wochen

Nitrofurantoin: which time schedule do you use? [Day(s) before VUS/VCUG, how many?]
Yes

Nitrofurantoin: which time schedule do you use? [Day(s) before VUS/VCUG, how many?][Comment]
Prophylaxe besteht bereits mit Indikationsstellung zur Refluxprüfung

Nitrofurantoin: which time schedule do you use? [Day of VUS/VCUG]
No

Nitrofurantoin: which time schedule do you use? [Day of VUS/VCUG][Comment]

Nitrofurantoin: which time schedule do you use? [Day(s) after VUS/VCUG, how many?]
Yes

Nitrofurantoin: which time schedule do you use? [Day(s) after VUS/VCUG, how many?][Comment]
3

Amoxicillin: which time schedule do you use? [Day(s) before VUS/VCUG, how many?]
N/A

Amoxicillin: which time schedule do you use? [Day of VUS/VCUG]
N/A

Amoxicillin: which time schedule do you use? [Day(s) after VUS/VCUG, how many?]
N/A

Cefaclor: in what dosage?
prophylactic (10 mg/kg bodyweight/d)

Cefaclor: in what dosage? [Other]

Cefaclor: for which age group do you use cefaclor?
0 - 12 Wochen

Cefaclor: which time schedule do you use? [Days(s) before VUS/VCUG, how many?]
Yes

Cefaclor: which time schedule do you use? [Days(s) before VUS/VCUG, how many?][Comment]
Prophylaxe besteht bereits mit Indikationsstellung zur Refluxprüfung

Cefaclor: which time schedule do you use? [Day of VUS/VCUG]
No

Cefaclor: which time schedule do you use? [Day of VUS/VCUG][Comment]

Cefaclor: which time schedule do you use? [Day(s) after VUS/VCUG, how many?]
Yes

Cefaclor: which time schedule do you use? [Day(s) after VUS/VCUG, how many?][Comment]
3

Amoxicillin/clavulanic acid: in what dosage? (Amoxicillin portion) [prophylaktic (10-20 mg/Kg bodyweight/d)]
N/A

Amoxicillin/clavulanic acid: in what dosage? (Amoxicillin portion) [double prophylactic (20-40 mg/Kg bodyweight/d)]
N/A

Amoxicillin/clavulanic acid: in what dosage? (Amoxicillin portion) [therapeutic (80-100 mg/Kg bodyweight/d)]
N/A

Amoxicillin/clavulanic acid: which time schedule do you use? [Day(s) before VUS/VCUG]
N/A

Amoxicillin/clavulanic acid: which time schedule do you use? [Day of VUS/VCUG]
N/A

Amoxicillin/clavulanic acid: which time schedule do you use? [Day(s) after VUS/VCUG]
N/A

Other: which medication and in what dosage?
Cotrim 1-2 mg/kg

Other: for which age group do you use this medication?
>12 Wochen

Other: which time schedule do you use? [Day(s) before VUS/VCUG, how many?]
Yes

Other: which time schedule do you use? [Day(s) before VUS/VCUG, how many?][Comment]
Prophylaxe besteht bereits mit Indikationsstellung zur Refluxprüfung

Other: which time schedule do you use? [Day of VUS/VCUG]
No

Other: which time schedule do you use? [Day of VUS/VCUG][Comment]

Other: which time schedule do you use? [Day(s) after VUS/VCUG, how many?]
Yes

Other: which time schedule do you use? [Day(s) after VUS/VCUG, how many?][Comment]
3

Indication for prophylaxis

Which patients receive periinterventional antibiotic prophylaxis? (multiple answers possible) [all]
No

Which patients receive periinterventional antibiotic prophylaxis? (multiple answers possible) [patients post urinary tract infection/pyelonephritis/urosepsis]
Yes

Which patients receive periinterventional antibiotic prophylaxis? (multiple answers possible) [patients with urinary tract dilation in ultrasound]
Yes

Which patients receive periinterventional antibiotic prophylaxis? (multiple answers possible) [patients with prenatal urinary tract dilation]
No

Which patients receive periinterventional antibiotic prophylaxis? (multiple answers possible) [patients post VUR surgery]
Yes

Which patients receive periinterventional antibiotic prophylaxis? (multiple answers possible) [none]
No

Which patients receive periinterventional antibiotic prophylaxis? (multiple answers possible) [Other]

patients status post urinary tract infection/pyelonephritis/urosepsis: what is the minimum time between infection and VUS/VCUG? (in days)
Am Ende der Therapie

In patients with urinary tract dilation in ultrasound: from what degree on? Image from Beetz R, Bokenkamp A, Brandis M et al (2001) Diagnosis of congenital dilatation of the urinary tract. Consensus Group of the Pediatric Nephrology Working Society in cooperation with the Pediatric Urology Working Group of the German Society of Urology and with the Pediatric Urology Working Society in the Germany Society of Pediatric Surgery. Urologe A 40:495–507 (quiz 8–9)
grade 2

Are there situations or clinical findings in the patient where you deviate from your prophylaxis standard (medication, dose, duration, age of the patient)? (multiple answers possible, please specify in answer box) [in case of already known vesicoureteral reflux (VUR)]
No

Are there situations or clinical findings in the patient where you deviate from your prophylaxis standard (medication, dose, duration, age of the patient)? (multiple answers possible, please specify in answer box) [in case of already known vesicoureteral reflux (VUR)][Comment]

Are there situations or clinical findings in the patient where you deviate from your prophylaxis standard (medication, dose, duration, age of the patient)? (multiple answers possible, please specify in answer box) [in patients with recurrent urinary tract infections (UTI)]
No

Are there situations or clinical findings in the patient where you deviate from your prophylaxis standard (medication, dose, duration, age of the patient)? (multiple answers possible, please specify in answer box) [in patients with recurrent urinary tract infections (UTI)][Comment]

Are there situations or clinical findings in the patient where you deviate from your prophylaxis standard (medication, dose, duration, age of the patient)? (multiple answers possible, please specify in answer box) [if multidrug-resistant organisms (MDRO) are detected in urine cultures]
Yes

Are there situations or clinical findings in the patient where you deviate from your prophylaxis standard (medication, dose, duration, age of the patient)? (multiple answers possible, please specify in answer box) [if multidrug-resistant organisms (MDRO) are detected in urine cultures][Comment]
Resistenzgerecht

Are there situations or clinical findings in the patient where you deviate from your prophylaxis standard (medication, dose, duration, age of the patient)? (multiple answers possible, please specify in answer box) [in newborns]
No

Are there situations or clinical findings in the patient where you deviate from your prophylaxis standard (medication, dose, duration, age of the patient)? (multiple answers possible, please specify in answer box) [in newborns][Comment]

Are there situations or clinical findings in the patient where you deviate from your prophylaxis standard (medication, dose, duration, age of the patient)? (multiple answers possible, please specify in answer box) [Other]

Are there situations or clinical findings in the patient where you deviate from your prophylaxis standard (medication, dose, duration, age of the patient)? (multiple answers possible, please specify in answer box) [Other comment]

Are there situations in which you use intravenous prophylaxis?
No

Voiding analysis

Are there differences in antibiotic prophylaxis between VUS and VCUG in your department?
No

Is a non-pathological urine strip test required in preparation for VUS/VCUG in your department?
Yes

What is the maximum amount of days accepted for urine strip test to be performed prior to the exam?
3

Is a non-pathological urine culture required in preparation for VUS/VCUG in your department?
No

Do patients only receive post-procedure prophylaxis if vesicoureteral reflux (VUR) is detected in VUS/VCUG?
No

Do other MUS/MCU findings (e.g. urethral valves) have an influence on post-procedure prophylaxis?
No

End of survey

Thank you for taking the time to answer this survey!

May we contact you personally if we have further questions?
Yes

E-mail address:
henry.fehrenbach@klinikum-memmingen.de

Survey response 106

Response ID
108

Date submitted
2025-01-28 19:02:48

Last page
5

Start language
de

Seed
1209815091

Date started
2025-01-28 18:42:11

Date last action
2025-01-28 19:02:48

Standard Operation Procedure

Who fills out this survey?
Other

Who fills out this survey? [Other]
Kinder nephrologe

In which type of hospital are you employed?
Other

In which type of hospital are you employed? [Other]
Praxis

In which country do you work?
Bayern

Does your department have a defined protocol for infection prophylaxis in the context of VUS/VCUG?
Yes

Has this protocol been transformed into a written standard operating procedure (SOP)?
Yes

Which department is the author of the SOP for infection prophylaxis in VUS/VCUG?
Pediatric nephrology

Which department is the author of the SOP for infection prophylaxis in VUS/VCUG? [Other]

Antibiotic Prophylaxis

Are there situations/constellations in which you do not use antibiotic prophylaxis for VUS/VCUG?
No

Please specify these situations/constellations: [girl with first uncomplicated UTI without urinary tract dilation in ultrasound]
N/A

Please specify these situations/constellations: [boy with first uncomplicated UTI without urinary tract dilation in ultrasound]
N/A

Please specify these situations/constellations: [in patients with low grade urinary tract dilation (up to 2nd grade) and no UTI in medical history]
N/A

If you use antibiotic prophylaxis: which mediaction do you use? (multiple answers and subdivision into age groups possible) [Trimethoprim]
No

If you use antibiotic prophylaxis: which mediaction do you use? (multiple answers and subdivision into age groups possible) [Nitrofurantoin]
No

If you use antibiotic prophylaxis: which mediaction do you use? (multiple answers and subdivision into age groups possible) [Amoxicillin]
No

If you use antibiotic prophylaxis: which mediaction do you use? (multiple answers and subdivision into age groups possible) [Cefaclor]
Yes

If you use antibiotic prophylaxis: which mediaction do you use? (multiple answers and subdivision into age groups possible) [Amoxicillin/clavulanic acid]
No

If you use antibiotic prophylaxis: which mediaction do you use? (multiple answers and subdivision into age groups possible) [Other]
No

Trimethoprim: which time schedule do you use? [Day(s) before VUS/VCUG, how many?]
N/A

Trimethoprim: which time schedule do you use? [Day of VUS/VCUG]
N/A

Trimethoprim: which time schedule do you use? [Day(s) after VUS/VCUG, how many?]
N/A

Nitrofurantoin: which time schedule do you use? [Day(s) before VUS/VCUG, how many?]
N/A

Nitrofurantoin: which time schedule do you use? [Day of VUS/VCUG]
N/A

Nitrofurantoin: which time schedule do you use? [Day(s) after VUS/VCUG, how many?]
N/A

Amoxicillin: which time schedule do you use? [Day(s) before VUS/VCUG, how many?]
N/A

Amoxicillin: which time schedule do you use? [Day of VUS/VCUG]
N/A

Amoxicillin: which time schedule do you use? [Day(s) after VUS/VCUG, how many?]
N/A

Cefaclor: in what dosage?
therapeutic (40 mg/kg bodyweight/d)

Cefaclor: in what dosage? [Other]

Cefaclor: for which age group do you use cefaclor?
Alle

Cefaclor: which time schedule do you use? [Days(s) before VUS/VCUG, how many?]
Yes

Cefaclor: which time schedule do you use? [Days(s) before VUS/VCUG, how many?][Comment]
1

Cefaclor: which time schedule do you use? [Day of VUS/VCUG]
Yes

Cefaclor: which time schedule do you use? [Day of VUS/VCUG][Comment]

Cefaclor: which time schedule do you use? [Day(s) after VUS/VCUG, how many?]
Yes

Cefaclor: which time schedule do you use? [Day(s) after VUS/VCUG, how many?][Comment]
2

Amoxicillin/clavulanic acid: in what dosage? (Amoxicillin portion) [prophylaktic (10-20 mg/Kg bodyweight/d)]
N/A

Amoxicillin/clavulanic acid: in what dosage? (Amoxicillin portion) [double prophylactic (20-40 mg/Kg bodyweight/d)]
N/A

Amoxicillin/clavulanic acid: in what dosage? (Amoxicillin portion) [therapeutic (80-100 mg/Kg bodyweight/d)]
N/A

Amoxicillin/clavulanic acid: which time schedule do you use? [Day(s) before VUS/VCUG]
N/A

Amoxicillin/clavulanic acid: which time schedule do you use? [Day of VUS/VCUG]
N/A

Amoxicillin/clavulanic acid: which time schedule do you use? [Day(s) after VUS/VCUG]
N/A

Other: which time schedule do you use? [Day(s) before VUS/VCUG, how many?]
N/A

Other: which time schedule do you use? [Day of VUS/VCUG]
N/A

Other: which time schedule do you use? [Day(s) after VUS/VCUG, how many?]
N/A

Indication for prophylaxis

Which patients receive periinterventional antibiotic prophylaxis? (multiple answers possible) [all]
Yes

Which patients receive periinterventional antibiotic prophylaxis? (multiple answers possible) [patients post urinary tract infection/pyelonephritis/urosepsis]
N/A

Which patients receive periinterventional antibiotic prophylaxis? (multiple answers possible) [patients with urinary tract dilation in ultrasound]
N/A

Which patients receive periinterventional antibiotic prophylaxis? (multiple answers possible) [patients with prenatal urinary tract dilation]
N/A

Which patients receive periinterventional antibiotic prophylaxis? (multiple answers possible) [patients post VUR surgery]
N/A

Which patients receive periinterventional antibiotic prophylaxis? (multiple answers possible) [none]
N/A

Are there situations or clinical findings in the patient where you deviate from your prophylaxis standard (medication, dose, duration, age of the patient)? (multiple answers possible, please specify in answer box) [in case of already known vesicoureteral reflux (VUR)]
No

Are there situations or clinical findings in the patient where you deviate from your prophylaxis standard (medication, dose, duration, age of the patient)? (multiple answers possible, please specify in answer box) [in case of already known vesicoureteral reflux (VUR)][Comment]

Are there situations or clinical findings in the patient where you deviate from your prophylaxis standard (medication, dose, duration, age of the patient)? (multiple answers possible, please specify in answer box) [in patients with recurrent urinary tract infections (UTI)]
No

Are there situations or clinical findings in the patient where you deviate from your prophylaxis standard (medication, dose, duration, age of the patient)? (multiple answers possible, please specify in answer box) [in patients with recurrent urinary tract infections (UTI)][Comment]

Are there situations or clinical findings in the patient where you deviate from your prophylaxis standard (medication, dose, duration, age of the patient)? (multiple answers possible, please specify in answer box) [if multidrug-resistant organisms (MDRO) are detected in urine cultures]
Yes

Are there situations or clinical findings in the patient where you deviate from your prophylaxis standard (medication, dose, duration, age of the patient)? (multiple answers possible, please specify in answer box) [if multidrug-resistant organisms (MDRO) are detected in urine cultures][Comment]
Nach resistogramn

Are there situations or clinical findings in the patient where you deviate from your prophylaxis standard (medication, dose, duration, age of the patient)? (multiple answers possible, please specify in answer box) [in newborns]
No

Are there situations or clinical findings in the patient where you deviate from your prophylaxis standard (medication, dose, duration, age of the patient)? (multiple answers possible, please specify in answer box) [in newborns][Comment]

Are there situations or clinical findings in the patient where you deviate from your prophylaxis standard (medication, dose, duration, age of the patient)? (multiple answers possible, please specify in answer box) [Other]

Are there situations or clinical findings in the patient where you deviate from your prophylaxis standard (medication, dose, duration, age of the patient)? (multiple answers possible, please specify in answer box) [Other comment]

Are there situations in which you use intravenous prophylaxis?
No

Voiding analysis

Are there differences in antibiotic prophylaxis between VUS and VCUG in your department?
No

Is a non-pathological urine strip test required in preparation for VUS/VCUG in your department?
No

Is a non-pathological urine culture required in preparation for VUS/VCUG in your department?
No

Do patients only receive post-procedure prophylaxis if vesicoureteral reflux (VUR) is detected in VUS/VCUG?
No

Do other MUS/MCU findings (e.g. urethral valves) have an influence on post-procedure prophylaxis?
No

End of survey

Thank you for taking the time to answer this survey!

May we contact you personally if we have further questions?
No

Survey response 107

Response ID
109

Last page
4

Start language
de

Seed
112369753

Date started
2025-01-28 18:52:59

Date last action
2025-01-28 19:01:58

Standard Operation Procedure

Who fills out this survey?
Pediatrician

Who fills out this survey? [Other]

In which type of hospital are you employed?
Community hospital (

In which type of hospital are you employed? [Other]

In which country do you work?
Deutschland, Baden-Württemberg

Does your department have a defined protocol for infection prophylaxis in the context of VUS/VCUG?
No

Has this protocol been transformed into a written standard operating procedure (SOP)?
N/A

Antibiotic Prophylaxis

Are there situations/constellations in which you do not use antibiotic prophylaxis for VUS/VCUG?
Yes

Please specify these situations/constellations: [girl with first uncomplicated UTI without urinary tract dilation in ultrasound]
Yes

Please specify these situations/constellations: [boy with first uncomplicated UTI without urinary tract dilation in ultrasound]
Yes

Please specify these situations/constellations: [in patients with low grade urinary tract dilation (up to 2nd grade) and no UTI in medical history]
Yes

Please specify these situations/constellations: [Other]

If you use antibiotic prophylaxis: which mediaction do you use? (multiple answers and subdivision into age groups possible) [Trimethoprim]
No

If you use antibiotic prophylaxis: which mediaction do you use? (multiple answers and subdivision into age groups possible) [Nitrofurantoin]
No

If you use antibiotic prophylaxis: which mediaction do you use? (multiple answers and subdivision into age groups possible) [Amoxicillin]
No

If you use antibiotic prophylaxis: which mediaction do you use? (multiple answers and subdivision into age groups possible) [Cefaclor]
Yes

If you use antibiotic prophylaxis: which mediaction do you use? (multiple answers and subdivision into age groups possible) [Amoxicillin/clavulanic acid]
No

If you use antibiotic prophylaxis: which mediaction do you use? (multiple answers and subdivision into age groups possible) [Other]
No

Trimethoprim: which time schedule do you use? [Day(s) before VUS/VCUG, how many?]
N/A

Trimethoprim: which time schedule do you use? [Day of VUS/VCUG]
N/A

Trimethoprim: which time schedule do you use? [Day(s) after VUS/VCUG, how many?]
N/A

Nitrofurantoin: which time schedule do you use? [Day(s) before VUS/VCUG, how many?]
N/A

Nitrofurantoin: which time schedule do you use? [Day of VUS/VCUG]
N/A

Nitrofurantoin: which time schedule do you use? [Day(s) after VUS/VCUG, how many?]
N/A

Amoxicillin: which time schedule do you use? [Day(s) before VUS/VCUG, how many?]
N/A

Amoxicillin: which time schedule do you use? [Day of VUS/VCUG]
N/A

Amoxicillin: which time schedule do you use? [Day(s) after VUS/VCUG, how many?]
N/A

Cefaclor: in what dosage?
therapeutic (40 mg/kg bodyweight/d)

Cefaclor: in what dosage? [Other]

Cefaclor: for which age group do you use cefaclor?
< 3 Monate

Cefaclor: which time schedule do you use? [Days(s) before VUS/VCUG, how many?]
No

Cefaclor: which time schedule do you use? [Days(s) before VUS/VCUG, how many?][Comment]

Cefaclor: which time schedule do you use? [Day of VUS/VCUG]
No

Cefaclor: which time schedule do you use? [Day of VUS/VCUG][Comment]

Cefaclor: which time schedule do you use? [Day(s) after VUS/VCUG, how many?]
Yes

Cefaclor: which time schedule do you use? [Day(s) after VUS/VCUG, how many?][Comment]
2

Amoxicillin/clavulanic acid: in what dosage? (Amoxicillin portion) [prophylaktic (10-20 mg/Kg bodyweight/d)]
N/A

Amoxicillin/clavulanic acid: in what dosage? (Amoxicillin portion) [double prophylactic (20-40 mg/Kg bodyweight/d)]
N/A

Amoxicillin/clavulanic acid: in what dosage? (Amoxicillin portion) [therapeutic (80-100 mg/Kg bodyweight/d)]
N/A

Amoxicillin/clavulanic acid: which time schedule do you use? [Day(s) before VUS/VCUG]
N/A

Amoxicillin/clavulanic acid: which time schedule do you use? [Day of VUS/VCUG]
N/A

Amoxicillin/clavulanic acid: which time schedule do you use? [Day(s) after VUS/VCUG]
N/A

Other: which time schedule do you use? [Day(s) before VUS/VCUG, how many?]
N/A

Other: which time schedule do you use? [Day of VUS/VCUG]
N/A

Other: which time schedule do you use? [Day(s) after VUS/VCUG, how many?]
N/A

Indication for prophylaxis

Which patients receive periinterventional antibiotic prophylaxis? (multiple answers possible) [all]
No

Which patients receive periinterventional antibiotic prophylaxis? (multiple answers possible) [patients post urinary tract infection/pyelonephritis/urosepsis]
No

Which patients receive periinterventional antibiotic prophylaxis? (multiple answers possible) [patients with urinary tract dilation in ultrasound]
No

Which patients receive periinterventional antibiotic prophylaxis? (multiple answers possible) [patients with prenatal urinary tract dilation]
No

Which patients receive periinterventional antibiotic prophylaxis? (multiple answers possible) [patients post VUR surgery]
No

Which patients receive periinterventional antibiotic prophylaxis? (multiple answers possible) [none]
No

Which patients receive periinterventional antibiotic prophylaxis? (multiple answers possible) [Other]
Urinstatus nicht sauber und Nachweis von Reflux

Are there situations or clinical findings in the patient where you deviate from your prophylaxis standard (medication, dose, duration, age of the patient)? (multiple answers possible, please specify in answer box) [in case of already known vesicoureteral reflux (VUR)]
No

Are there situations or clinical findings in the patient where you deviate from your prophylaxis standard (medication, dose, duration, age of the patient)? (multiple answers possible, please specify in answer box) [in case of already known vesicoureteral reflux (VUR)][Comment]

Are there situations or clinical findings in the patient where you deviate from your prophylaxis standard (medication, dose, duration, age of the patient)? (multiple answers possible, please specify in answer box) [in patients with recurrent urinary tract infections (UTI)]
No

Are there situations or clinical findings in the patient where you deviate from your prophylaxis standard (medication, dose, duration, age of the patient)? (multiple answers possible, please specify in answer box) [in patients with recurrent urinary tract infections (UTI)][Comment]

Are there situations or clinical findings in the patient where you deviate from your prophylaxis standard (medication, dose, duration, age of the patient)? (multiple answers possible, please specify in answer box) [if multidrug-resistant organisms (MDRO) are detected in urine cultures]
No

Are there situations or clinical findings in the patient where you deviate from your prophylaxis standard (medication, dose, duration, age of the patient)? (multiple answers possible, please specify in answer box) [if multidrug-resistant organisms (MDRO) are detected in urine cultures][Comment]

Are there situations or clinical findings in the patient where you deviate from your prophylaxis standard (medication, dose, duration, age of the patient)? (multiple answers possible, please specify in answer box) [in newborns]
No

Are there situations or clinical findings in the patient where you deviate from your prophylaxis standard (medication, dose, duration, age of the patient)? (multiple answers possible, please specify in answer box) [in newborns][Comment]

Are there situations or clinical findings in the patient where you deviate from your prophylaxis standard (medication, dose, duration, age of the patient)? (multiple answers possible, please specify in answer box) [Other]
asdfd

Are there situations or clinical findings in the patient where you deviate from your prophylaxis standard (medication, dose, duration, age of the patient)? (multiple answers possible, please specify in answer box) [Other comment]

Are there situations in which you use intravenous prophylaxis?
Yes

In which situations?
Fieber kurz nach Untersuchung

Voiding analysis

Are there differences in antibiotic prophylaxis between VUS and VCUG in your department?
No

Is a non-pathological urine strip test required in preparation for VUS/VCUG in your department?
Yes

What is the maximum amount of days accepted for urine strip test to be performed prior to the exam?
0

Is a non-pathological urine culture required in preparation for VUS/VCUG in your department?
No

Do patients only receive post-procedure prophylaxis if vesicoureteral reflux (VUR) is detected in VUS/VCUG?
No

Do other MUS/MCU findings (e.g. urethral valves) have an influence on post-procedure prophylaxis?
No

End of survey

Thank you for taking the time to answer this survey!

May we contact you personally if we have further questions?
N/A

Survey response 108

Response ID
110

Date submitted
2025-01-29 05:15:55

Last page
5

Start language
de

Seed
1887109350

Date started
2025-01-29 05:10:24

Date last action
2025-01-29 05:15:55

Standard Operation Procedure

Who fills out this survey?
Pediatrician

Who fills out this survey? [Other]

In which type of hospital are you employed?
Other

In which type of hospital are you employed? [Other]
kindernephrologische Praxis

In which country do you work?
Deutschland

Does your department have a defined protocol for infection prophylaxis in the context of VUS/VCUG?
Yes

Has this protocol been transformed into a written standard operating procedure (SOP)?
No

Antibiotic Prophylaxis

Are there situations/constellations in which you do not use antibiotic prophylaxis for VUS/VCUG?
No

Please specify these situations/constellations: [girl with first uncomplicated UTI without urinary tract dilation in ultrasound]
N/A

Please specify these situations/constellations: [boy with first uncomplicated UTI without urinary tract dilation in ultrasound]
N/A

Please specify these situations/constellations: [in patients with low grade urinary tract dilation (up to 2nd grade) and no UTI in medical history]
N/A

If you use antibiotic prophylaxis: which mediaction do you use? (multiple answers and subdivision into age groups possible) [Trimethoprim]
Yes

If you use antibiotic prophylaxis: which mediaction do you use? (multiple answers and subdivision into age groups possible) [Nitrofurantoin]
Yes

If you use antibiotic prophylaxis: which mediaction do you use? (multiple answers and subdivision into age groups possible) [Amoxicillin]
No

If you use antibiotic prophylaxis: which mediaction do you use? (multiple answers and subdivision into age groups possible) [Cefaclor]
No

If you use antibiotic prophylaxis: which mediaction do you use? (multiple answers and subdivision into age groups possible) [Amoxicillin/clavulanic acid]
No

If you use antibiotic prophylaxis: which mediaction do you use? (multiple answers and subdivision into age groups possible) [Other]
No

Trimethoprim: in what dosage?
double prophylakctic (4 mg/kg bodyweight/d)

Trimethoprim: in what dosage? [Other]

Trimethoprim: for which age group do you use trimethoprim?
eher bei den Kleineren, ab 6Wochen wie zugelassen

Trimethoprim: which time schedule do you use? [Day(s) before VUS/VCUG, how many?]
No

Trimethoprim: which time schedule do you use? [Day(s) before VUS/VCUG, how many?][Comment]

Trimethoprim: which time schedule do you use? [Day of VUS/VCUG]
Yes

Trimethoprim: which time schedule do you use? [Day of VUS/VCUG][Comment]

Trimethoprim: which time schedule do you use? [Day(s) after VUS/VCUG, how many?]
Yes

Trimethoprim: which time schedule do you use? [Day(s) after VUS/VCUG, how many?][Comment]
2

Nitrofurantoin: in what dosage?
therapeutic (5 mg/kg bodyweight/d)

Nitrofurantoin: in what dosage? [Other]

Nitrofurantoin: for which age group do you use nitrofurantoin?
eher bei den größeren, nach Antibiogramm

Nitrofurantoin: which time schedule do you use? [Day(s) before VUS/VCUG, how many?]
No

Nitrofurantoin: which time schedule do you use? [Day(s) before VUS/VCUG, how many?][Comment]

Nitrofurantoin: which time schedule do you use? [Day of VUS/VCUG]
Yes

Nitrofurantoin: which time schedule do you use? [Day of VUS/VCUG][Comment]

Nitrofurantoin: which time schedule do you use? [Day(s) after VUS/VCUG, how many?]
Yes

Nitrofurantoin: which time schedule do you use? [Day(s) after VUS/VCUG, how many?][Comment]
2

Amoxicillin: which time schedule do you use? [Day(s) before VUS/VCUG, how many?]
N/A

Amoxicillin: which time schedule do you use? [Day of VUS/VCUG]
N/A

Amoxicillin: which time schedule do you use? [Day(s) after VUS/VCUG, how many?]
N/A

Cefaclor: which time schedule do you use? [Days(s) before VUS/VCUG, how many?]
N/A

Cefaclor: which time schedule do you use? [Day of VUS/VCUG]
N/A

Cefaclor: which time schedule do you use? [Day(s) after VUS/VCUG, how many?]
N/A

Amoxicillin/clavulanic acid: in what dosage? (Amoxicillin portion) [prophylaktic (10-20 mg/Kg bodyweight/d)]
N/A

Amoxicillin/clavulanic acid: in what dosage? (Amoxicillin portion) [double prophylactic (20-40 mg/Kg bodyweight/d)]
N/A

Amoxicillin/clavulanic acid: in what dosage? (Amoxicillin portion) [therapeutic (80-100 mg/Kg bodyweight/d)]
N/A

Amoxicillin/clavulanic acid: which time schedule do you use? [Day(s) before VUS/VCUG]
N/A

Amoxicillin/clavulanic acid: which time schedule do you use? [Day of VUS/VCUG]
N/A

Amoxicillin/clavulanic acid: which time schedule do you use? [Day(s) after VUS/VCUG]
N/A

Other: which time schedule do you use? [Day(s) before VUS/VCUG, how many?]
N/A

Other: which time schedule do you use? [Day of VUS/VCUG]
N/A

Other: which time schedule do you use? [Day(s) after VUS/VCUG, how many?]
N/A

Indication for prophylaxis

Which patients receive periinterventional antibiotic prophylaxis? (multiple answers possible) [all]
Yes

Which patients receive periinterventional antibiotic prophylaxis? (multiple answers possible) [patients post urinary tract infection/pyelonephritis/urosepsis]
N/A

Which patients receive periinterventional antibiotic prophylaxis? (multiple answers possible) [patients with urinary tract dilation in ultrasound]
N/A

Which patients receive periinterventional antibiotic prophylaxis? (multiple answers possible) [patients with prenatal urinary tract dilation]
N/A

Which patients receive periinterventional antibiotic prophylaxis? (multiple answers possible) [patients post VUR surgery]
N/A

Which patients receive periinterventional antibiotic prophylaxis? (multiple answers possible) [none]
N/A

Are there situations or clinical findings in the patient where you deviate from your prophylaxis standard (medication, dose, duration, age of the patient)? (multiple answers possible, please specify in answer box) [in case of already known vesicoureteral reflux (VUR)]
No

Are there situations or clinical findings in the patient where you deviate from your prophylaxis standard (medication, dose, duration, age of the patient)? (multiple answers possible, please specify in answer box) [in case of already known vesicoureteral reflux (VUR)][Comment]

Are there situations or clinical findings in the patient where you deviate from your prophylaxis standard (medication, dose, duration, age of the patient)? (multiple answers possible, please specify in answer box) [in patients with recurrent urinary tract infections (UTI)]
No

Are there situations or clinical findings in the patient where you deviate from your prophylaxis standard (medication, dose, duration, age of the patient)? (multiple answers possible, please specify in answer box) [in patients with recurrent urinary tract infections (UTI)][Comment]

Are there situations or clinical findings in the patient where you deviate from your prophylaxis standard (medication, dose, duration, age of the patient)? (multiple answers possible, please specify in answer box) [if multidrug-resistant organisms (MDRO) are detected in urine cultures]
Yes

Are there situations or clinical findings in the patient where you deviate from your prophylaxis standard (medication, dose, duration, age of the patient)? (multiple answers possible, please specify in answer box) [if multidrug-resistant organisms (MDRO) are detected in urine cultures][Comment]

Are there situations or clinical findings in the patient where you deviate from your prophylaxis standard (medication, dose, duration, age of the patient)? (multiple answers possible, please specify in answer box) [in newborns]
Yes

Are there situations or clinical findings in the patient where you deviate from your prophylaxis standard (medication, dose, duration, age of the patient)? (multiple answers possible, please specify in answer box) [in newborns][Comment]

Are there situations or clinical findings in the patient where you deviate from your prophylaxis standard (medication, dose, duration, age of the patient)? (multiple answers possible, please specify in answer box) [Other]

Are there situations or clinical findings in the patient where you deviate from your prophylaxis standard (medication, dose, duration, age of the patient)? (multiple answers possible, please specify in answer box) [Other comment]

Are there situations in which you use intravenous prophylaxis?
No

Voiding analysis

Are there differences in antibiotic prophylaxis between VUS and VCUG in your department?
No

Is a non-pathological urine strip test required in preparation for VUS/VCUG in your department?
Yes

What is the maximum amount of days accepted for urine strip test to be performed prior to the exam?
nicht bekannt

Is a non-pathological urine culture required in preparation for VUS/VCUG in your department?
No

Do patients only receive post-procedure prophylaxis if vesicoureteral reflux (VUR) is detected in VUS/VCUG?
No

Do other MUS/MCU findings (e.g. urethral valves) have an influence on post-procedure prophylaxis?
No

End of survey

Thank you for taking the time to answer this survey!

May we contact you personally if we have further questions?
Yes

E-mail address:
jasmin.hambsch@knz-bonn.de

Survey response 109

Response ID
111

Date submitted
2025-01-29 06:15:21

Last page
5

Start language
de

Seed
888626400

Date started
2025-01-29 06:07:35

Date last action
2025-01-29 06:15:21

Standard Operation Procedure

Who fills out this survey?
Pediatric nephrologist

Who fills out this survey? [Other]

In which type of hospital are you employed?
Community hospital (>50 beds for pediatric patients)

In which type of hospital are you employed? [Other]

In which country do you work?
Schweiz

Does your department have a defined protocol for infection prophylaxis in the context of VUS/VCUG?
Yes

Has this protocol been transformed into a written standard operating procedure (SOP)?
Yes

Which department is the author of the SOP for infection prophylaxis in VUS/VCUG?
Pediatric nephrology

Which department is the author of the SOP for infection prophylaxis in VUS/VCUG? [Other]

Antibiotic Prophylaxis

Are there situations/constellations in which you do not use antibiotic prophylaxis for VUS/VCUG?
No

Please specify these situations/constellations: [girl with first uncomplicated UTI without urinary tract dilation in ultrasound]
N/A

Please specify these situations/constellations: [boy with first uncomplicated UTI without urinary tract dilation in ultrasound]
N/A

Please specify these situations/constellations: [in patients with low grade urinary tract dilation (up to 2nd grade) and no UTI in medical history]
N/A

If you use antibiotic prophylaxis: which mediaction do you use? (multiple answers and subdivision into age groups possible) [Trimethoprim]
Yes

If you use antibiotic prophylaxis: which mediaction do you use? (multiple answers and subdivision into age groups possible) [Nitrofurantoin]
No

If you use antibiotic prophylaxis: which mediaction do you use? (multiple answers and subdivision into age groups possible) [Amoxicillin]
No

If you use antibiotic prophylaxis: which mediaction do you use? (multiple answers and subdivision into age groups possible) [Cefaclor]
No

If you use antibiotic prophylaxis: which mediaction do you use? (multiple answers and subdivision into age groups possible) [Amoxicillin/clavulanic acid]
No

If you use antibiotic prophylaxis: which mediaction do you use? (multiple answers and subdivision into age groups possible) [Other]
No

Trimethoprim: in what dosage?
double prophylakctic (4 mg/kg bodyweight/d)

Trimethoprim: in what dosage? [Other]

Trimethoprim: for which age group do you use trimethoprim?
ab 1 Lebensmonat

Trimethoprim: which time schedule do you use? [Day(s) before VUS/VCUG, how many?]
No

Trimethoprim: which time schedule do you use? [Day(s) before VUS/VCUG, how many?][Comment]

Trimethoprim: which time schedule do you use? [Day of VUS/VCUG]
Yes

Trimethoprim: which time schedule do you use? [Day of VUS/VCUG][Comment]

Trimethoprim: which time schedule do you use? [Day(s) after VUS/VCUG, how many?]
Yes

Trimethoprim: which time schedule do you use? [Day(s) after VUS/VCUG, how many?][Comment]
1

Nitrofurantoin: which time schedule do you use? [Day(s) before VUS/VCUG, how many?]
N/A

Nitrofurantoin: which time schedule do you use? [Day of VUS/VCUG]
N/A

Nitrofurantoin: which time schedule do you use? [Day(s) after VUS/VCUG, how many?]
N/A

Amoxicillin: which time schedule do you use? [Day(s) before VUS/VCUG, how many?]
N/A

Amoxicillin: which time schedule do you use? [Day of VUS/VCUG]
N/A

Amoxicillin: which time schedule do you use? [Day(s) after VUS/VCUG, how many?]
N/A

Cefaclor: which time schedule do you use? [Days(s) before VUS/VCUG, how many?]
N/A

Cefaclor: which time schedule do you use? [Day of VUS/VCUG]
N/A

Cefaclor: which time schedule do you use? [Day(s) after VUS/VCUG, how many?]
N/A

Amoxicillin/clavulanic acid: in what dosage? (Amoxicillin portion) [prophylaktic (10-20 mg/Kg bodyweight/d)]
N/A

Amoxicillin/clavulanic acid: in what dosage? (Amoxicillin portion) [double prophylactic (20-40 mg/Kg bodyweight/d)]
N/A

Amoxicillin/clavulanic acid: in what dosage? (Amoxicillin portion) [therapeutic (80-100 mg/Kg bodyweight/d)]
N/A

Amoxicillin/clavulanic acid: which time schedule do you use? [Day(s) before VUS/VCUG]
N/A

Amoxicillin/clavulanic acid: which time schedule do you use? [Day of VUS/VCUG]
N/A

Amoxicillin/clavulanic acid: which time schedule do you use? [Day(s) after VUS/VCUG]
N/A

Other: which time schedule do you use? [Day(s) before VUS/VCUG, how many?]
N/A

Other: which time schedule do you use? [Day of VUS/VCUG]
N/A

Other: which time schedule do you use? [Day(s) after VUS/VCUG, how many?]
N/A

Indication for prophylaxis

Which patients receive periinterventional antibiotic prophylaxis? (multiple answers possible) [all]
No

Which patients receive periinterventional antibiotic prophylaxis? (multiple answers possible) [patients post urinary tract infection/pyelonephritis/urosepsis]
No

Which patients receive periinterventional antibiotic prophylaxis? (multiple answers possible) [patients with urinary tract dilation in ultrasound]
Yes

Which patients receive periinterventional antibiotic prophylaxis? (multiple answers possible) [patients with prenatal urinary tract dilation]
No

Which patients receive periinterventional antibiotic prophylaxis? (multiple answers possible) [patients post VUR surgery]
Yes

Which patients receive periinterventional antibiotic prophylaxis? (multiple answers possible) [none]
No

Which patients receive periinterventional antibiotic prophylaxis? (multiple answers possible) [Other]
Nach 2 PN oder non-E-coli

In patients with urinary tract dilation in ultrasound: from what degree on? Image from Beetz R, Bokenkamp A, Brandis M et al (2001) Diagnosis of congenital dilatation of the urinary tract. Consensus Group of the Pediatric Nephrology Working Society in cooperation with the Pediatric Urology Working Group of the German Society of Urology and with the Pediatric Urology Working Society in the Germany Society of Pediatric Surgery. Urologe A 40:495–507 (quiz 8–9)
grade 2

Are there situations or clinical findings in the patient where you deviate from your prophylaxis standard (medication, dose, duration, age of the patient)? (multiple answers possible, please specify in answer box) [in case of already known vesicoureteral reflux (VUR)]
No

Are there situations or clinical findings in the patient where you deviate from your prophylaxis standard (medication, dose, duration, age of the patient)? (multiple answers possible, please specify in answer box) [in case of already known vesicoureteral reflux (VUR)][Comment]

Are there situations or clinical findings in the patient where you deviate from your prophylaxis standard (medication, dose, duration, age of the patient)? (multiple answers possible, please specify in answer box) [in patients with recurrent urinary tract infections (UTI)]
No

Are there situations or clinical findings in the patient where you deviate from your prophylaxis standard (medication, dose, duration, age of the patient)? (multiple answers possible, please specify in answer box) [in patients with recurrent urinary tract infections (UTI)][Comment]

Are there situations or clinical findings in the patient where you deviate from your prophylaxis standard (medication, dose, duration, age of the patient)? (multiple answers possible, please specify in answer box) [if multidrug-resistant organisms (MDRO) are detected in urine cultures]
No

Are there situations or clinical findings in the patient where you deviate from your prophylaxis standard (medication, dose, duration, age of the patient)? (multiple answers possible, please specify in answer box) [if multidrug-resistant organisms (MDRO) are detected in urine cultures][Comment]

Are there situations or clinical findings in the patient where you deviate from your prophylaxis standard (medication, dose, duration, age of the patient)? (multiple answers possible, please specify in answer box) [in newborns]
No

Are there situations or clinical findings in the patient where you deviate from your prophylaxis standard (medication, dose, duration, age of the patient)? (multiple answers possible, please specify in answer box) [in newborns][Comment]

Are there situations or clinical findings in the patient where you deviate from your prophylaxis standard (medication, dose, duration, age of the patient)? (multiple answers possible, please specify in answer box) [Other]
Individuell entschieden

Are there situations or clinical findings in the patient where you deviate from your prophylaxis standard (medication, dose, duration, age of the patient)? (multiple answers possible, please specify in answer box) [Other comment]

Are there situations in which you use intravenous prophylaxis?
No

Voiding analysis

Are there differences in antibiotic prophylaxis between VUS and VCUG in your department?
No

Is a non-pathological urine strip test required in preparation for VUS/VCUG in your department?
Yes

What is the maximum amount of days accepted for urine strip test to be performed prior to the exam?
1 Tag

Is a non-pathological urine culture required in preparation for VUS/VCUG in your department?
Yes

What is the maximum amount of days accepted for urine culture to be performed prior to the exam?
2 Tage

Do patients only receive post-procedure prophylaxis if vesicoureteral reflux (VUR) is detected in VUS/VCUG?
No

Do other MUS/MCU findings (e.g. urethral valves) have an influence on post-procedure prophylaxis?
No

End of survey

Thank you for taking the time to answer this survey!

May we contact you personally if we have further questions?
Yes

E-mail address:
guido.laube@ksb.ch

Survey response 110

Response ID
112

Date submitted
2025-01-29 06:19:06

Last page
5

Start language
de

Seed
1227417396

Date started
2025-01-29 06:13:44

Date last action
2025-01-29 06:19:06

Standard Operation Procedure

Who fills out this survey?
Pediatric nephrologist

Who fills out this survey? [Other]

In which type of hospital are you employed?
University Hospital

In which type of hospital are you employed? [Other]

In which country do you work?
Deutschland

Does your department have a defined protocol for infection prophylaxis in the context of VUS/VCUG?
Yes

Has this protocol been transformed into a written standard operating procedure (SOP)?
No

Antibiotic Prophylaxis

Are there situations/constellations in which you do not use antibiotic prophylaxis for VUS/VCUG?
No

Please specify these situations/constellations: [girl with first uncomplicated UTI without urinary tract dilation in ultrasound]
N/A

Please specify these situations/constellations: [boy with first uncomplicated UTI without urinary tract dilation in ultrasound]
N/A

Please specify these situations/constellations: [in patients with low grade urinary tract dilation (up to 2nd grade) and no UTI in medical history]
N/A

If you use antibiotic prophylaxis: which mediaction do you use? (multiple answers and subdivision into age groups possible) [Trimethoprim]
Yes

If you use antibiotic prophylaxis: which mediaction do you use? (multiple answers and subdivision into age groups possible) [Nitrofurantoin]
No

If you use antibiotic prophylaxis: which mediaction do you use? (multiple answers and subdivision into age groups possible) [Amoxicillin]
No

If you use antibiotic prophylaxis: which mediaction do you use? (multiple answers and subdivision into age groups possible) [Cefaclor]
Yes

If you use antibiotic prophylaxis: which mediaction do you use? (multiple answers and subdivision into age groups possible) [Amoxicillin/clavulanic acid]
No

If you use antibiotic prophylaxis: which mediaction do you use? (multiple answers and subdivision into age groups possible) [Other]
No

Trimethoprim: in what dosage?
therapeutic (6 mg/kg bodyweight/d)

Trimethoprim: in what dosage? [Other]

Trimethoprim: for which age group do you use trimethoprim?
>3 Monate

Trimethoprim: which time schedule do you use? [Day(s) before VUS/VCUG, how many?]
No

Trimethoprim: which time schedule do you use? [Day(s) before VUS/VCUG, how many?][Comment]

Trimethoprim: which time schedule do you use? [Day of VUS/VCUG]
Yes

Trimethoprim: which time schedule do you use? [Day of VUS/VCUG][Comment]

Trimethoprim: which time schedule do you use? [Day(s) after VUS/VCUG, how many?]
Yes

Trimethoprim: which time schedule do you use? [Day(s) after VUS/VCUG, how many?][Comment]
3

Nitrofurantoin: which time schedule do you use? [Day(s) before VUS/VCUG, how many?]
N/A

Nitrofurantoin: which time schedule do you use? [Day of VUS/VCUG]
N/A

Nitrofurantoin: which time schedule do you use? [Day(s) after VUS/VCUG, how many?]
N/A

Amoxicillin: which time schedule do you use? [Day(s) before VUS/VCUG, how many?]
N/A

Amoxicillin: which time schedule do you use? [Day of VUS/VCUG]
N/A

Amoxicillin: which time schedule do you use? [Day(s) after VUS/VCUG, how many?]
N/A

Cefaclor: in what dosage?
therapeutic (40 mg/kg bodyweight/d)

Cefaclor: in what dosage? [Other]

Cefaclor: for which age group do you use cefaclor?
< 3 Monate

Cefaclor: which time schedule do you use? [Days(s) before VUS/VCUG, how many?]
No

Cefaclor: which time schedule do you use? [Days(s) before VUS/VCUG, how many?][Comment]

Cefaclor: which time schedule do you use? [Day of VUS/VCUG]
Yes

Cefaclor: which time schedule do you use? [Day of VUS/VCUG][Comment]

Cefaclor: which time schedule do you use? [Day(s) after VUS/VCUG, how many?]
Yes

Cefaclor: which time schedule do you use? [Day(s) after VUS/VCUG, how many?][Comment]
3

Amoxicillin/clavulanic acid: in what dosage? (Amoxicillin portion) [prophylaktic (10-20 mg/Kg bodyweight/d)]
N/A

Amoxicillin/clavulanic acid: in what dosage? (Amoxicillin portion) [double prophylactic (20-40 mg/Kg bodyweight/d)]
N/A

Amoxicillin/clavulanic acid: in what dosage? (Amoxicillin portion) [therapeutic (80-100 mg/Kg bodyweight/d)]
N/A

Amoxicillin/clavulanic acid: which time schedule do you use? [Day(s) before VUS/VCUG]
N/A

Amoxicillin/clavulanic acid: which time schedule do you use? [Day of VUS/VCUG]
N/A

Amoxicillin/clavulanic acid: which time schedule do you use? [Day(s) after VUS/VCUG]
N/A

Other: which time schedule do you use? [Day(s) before VUS/VCUG, how many?]
N/A

Other: which time schedule do you use? [Day of VUS/VCUG]
N/A

Other: which time schedule do you use? [Day(s) after VUS/VCUG, how many?]
N/A

Indication for prophylaxis

Which patients receive periinterventional antibiotic prophylaxis? (multiple answers possible) [all]
Yes

Which patients receive periinterventional antibiotic prophylaxis? (multiple answers possible) [patients post urinary tract infection/pyelonephritis/urosepsis]
N/A

Which patients receive periinterventional antibiotic prophylaxis? (multiple answers possible) [patients with urinary tract dilation in ultrasound]
N/A

Which patients receive periinterventional antibiotic prophylaxis? (multiple answers possible) [patients with prenatal urinary tract dilation]
N/A

Which patients receive periinterventional antibiotic prophylaxis? (multiple answers possible) [patients post VUR surgery]
N/A

Which patients receive periinterventional antibiotic prophylaxis? (multiple answers possible) [none]
N/A

Are there situations or clinical findings in the patient where you deviate from your prophylaxis standard (medication, dose, duration, age of the patient)? (multiple answers possible, please specify in answer box) [in case of already known vesicoureteral reflux (VUR)]
No

Are there situations or clinical findings in the patient where you deviate from your prophylaxis standard (medication, dose, duration, age of the patient)? (multiple answers possible, please specify in answer box) [in case of already known vesicoureteral reflux (VUR)][Comment]

Are there situations or clinical findings in the patient where you deviate from your prophylaxis standard (medication, dose, duration, age of the patient)? (multiple answers possible, please specify in answer box) [in patients with recurrent urinary tract infections (UTI)]
No

Are there situations or clinical findings in the patient where you deviate from your prophylaxis standard (medication, dose, duration, age of the patient)? (multiple answers possible, please specify in answer box) [in patients with recurrent urinary tract infections (UTI)][Comment]

Are there situations or clinical findings in the patient where you deviate from your prophylaxis standard (medication, dose, duration, age of the patient)? (multiple answers possible, please specify in answer box) [if multidrug-resistant organisms (MDRO) are detected in urine cultures]
Yes

Are there situations or clinical findings in the patient where you deviate from your prophylaxis standard (medication, dose, duration, age of the patient)? (multiple answers possible, please specify in answer box) [if multidrug-resistant organisms (MDRO) are detected in urine cultures][Comment]

Are there situations or clinical findings in the patient where you deviate from your prophylaxis standard (medication, dose, duration, age of the patient)? (multiple answers possible, please specify in answer box) [in newborns]
No

Are there situations or clinical findings in the patient where you deviate from your prophylaxis standard (medication, dose, duration, age of the patient)? (multiple answers possible, please specify in answer box) [in newborns][Comment]

Are there situations or clinical findings in the patient where you deviate from your prophylaxis standard (medication, dose, duration, age of the patient)? (multiple answers possible, please specify in answer box) [Other]

Are there situations or clinical findings in the patient where you deviate from your prophylaxis standard (medication, dose, duration, age of the patient)? (multiple answers possible, please specify in answer box) [Other comment]

Are there situations in which you use intravenous prophylaxis?
No

Voiding analysis

Are there differences in antibiotic prophylaxis between VUS and VCUG in your department?
No

Is a non-pathological urine strip test required in preparation for VUS/VCUG in your department?
Yes

What is the maximum amount of days accepted for urine strip test to be performed prior to the exam?
1

Is a non-pathological urine culture required in preparation for VUS/VCUG in your department?
No

Do patients only receive post-procedure prophylaxis if vesicoureteral reflux (VUR) is detected in VUS/VCUG?
No

Do other MUS/MCU findings (e.g. urethral valves) have an influence on post-procedure prophylaxis?
No

End of survey

Thank you for taking the time to answer this survey!

May we contact you personally if we have further questions?
Yes

E-mail address:
Jens.koenig@ukmuenster.de

Survey response 111

Response ID
113

Last page
1

Start language
de

Seed
1619734591

Date started
2025-01-29 07:11:55

Date last action
2025-01-29 07:12:25

Standard Operation Procedure

Who fills out this survey?
Pediatric nephrologist

Who fills out this survey? [Other]

In which type of hospital are you employed?
University Hospital

In which type of hospital are you employed? [Other]

In which country do you work?
Deutschland

Does your department have a defined protocol for infection prophylaxis in the context of VUS/VCUG?
No

Has this protocol been transformed into a written standard operating procedure (SOP)?
N/A

Antibiotic Prophylaxis

Are there situations/constellations in which you do not use antibiotic prophylaxis for VUS/VCUG?
N/A

Please specify these situations/constellations: [girl with first uncomplicated UTI without urinary tract dilation in ultrasound]
N/A

Please specify these situations/constellations: [boy with first uncomplicated UTI without urinary tract dilation in ultrasound]
N/A

Please specify these situations/constellations: [in patients with low grade urinary tract dilation (up to 2nd grade) and no UTI in medical history]
N/A

If you use antibiotic prophylaxis: which mediaction do you use? (multiple answers and subdivision into age groups possible) [Trimethoprim]
N/A

If you use antibiotic prophylaxis: which mediaction do you use? (multiple answers and subdivision into age groups possible) [Nitrofurantoin]
N/A

If you use antibiotic prophylaxis: which mediaction do you use? (multiple answers and subdivision into age groups possible) [Amoxicillin]
N/A

If you use antibiotic prophylaxis: which mediaction do you use? (multiple answers and subdivision into age groups possible) [Cefaclor]
N/A

If you use antibiotic prophylaxis: which mediaction do you use? (multiple answers and subdivision into age groups possible) [Amoxicillin/clavulanic acid]
N/A

If you use antibiotic prophylaxis: which mediaction do you use? (multiple answers and subdivision into age groups possible) [Other]
N/A

Trimethoprim: which time schedule do you use? [Day(s) before VUS/VCUG, how many?]
N/A

Trimethoprim: which time schedule do you use? [Day of VUS/VCUG]
N/A

Trimethoprim: which time schedule do you use? [Day(s) after VUS/VCUG, how many?]
N/A

Nitrofurantoin: which time schedule do you use? [Day(s) before VUS/VCUG, how many?]
N/A

Nitrofurantoin: which time schedule do you use? [Day of VUS/VCUG]
N/A

Nitrofurantoin: which time schedule do you use? [Day(s) after VUS/VCUG, how many?]
N/A

Amoxicillin: which time schedule do you use? [Day(s) before VUS/VCUG, how many?]
N/A

Amoxicillin: which time schedule do you use? [Day of VUS/VCUG]
N/A

Amoxicillin: which time schedule do you use? [Day(s) after VUS/VCUG, how many?]
N/A

Cefaclor: which time schedule do you use? [Days(s) before VUS/VCUG, how many?]
N/A

Cefaclor: which time schedule do you use? [Day of VUS/VCUG]
N/A

Cefaclor: which time schedule do you use? [Day(s) after VUS/VCUG, how many?]
N/A

Amoxicillin/clavulanic acid: in what dosage? (Amoxicillin portion) [prophylaktic (10-20 mg/Kg bodyweight/d)]
N/A

Amoxicillin/clavulanic acid: in what dosage? (Amoxicillin portion) [double prophylactic (20-40 mg/Kg bodyweight/d)]
N/A

Amoxicillin/clavulanic acid: in what dosage? (Amoxicillin portion) [therapeutic (80-100 mg/Kg bodyweight/d)]
N/A

Amoxicillin/clavulanic acid: which time schedule do you use? [Day(s) before VUS/VCUG]
N/A

Amoxicillin/clavulanic acid: which time schedule do you use? [Day of VUS/VCUG]
N/A

Amoxicillin/clavulanic acid: which time schedule do you use? [Day(s) after VUS/VCUG]
N/A

Other: which time schedule do you use? [Day(s) before VUS/VCUG, how many?]
N/A

Other: which time schedule do you use? [Day of VUS/VCUG]
N/A

Other: which time schedule do you use? [Day(s) after VUS/VCUG, how many?]
N/A

Indication for prophylaxis

Which patients receive periinterventional antibiotic prophylaxis? (multiple answers possible) [all]
N/A

Which patients receive periinterventional antibiotic prophylaxis? (multiple answers possible) [patients post urinary tract infection/pyelonephritis/urosepsis]
N/A

Which patients receive periinterventional antibiotic prophylaxis? (multiple answers possible) [patients with urinary tract dilation in ultrasound]
N/A

Which patients receive periinterventional antibiotic prophylaxis? (multiple answers possible) [patients with prenatal urinary tract dilation]
N/A

Which patients receive periinterventional antibiotic prophylaxis? (multiple answers possible) [patients post VUR surgery]
N/A

Which patients receive periinterventional antibiotic prophylaxis? (multiple answers possible) [none]
N/A

Are there situations or clinical findings in the patient where you deviate from your prophylaxis standard (medication, dose, duration, age of the patient)? (multiple answers possible, please specify in answer box) [in case of already known vesicoureteral reflux (VUR)]
N/A

Are there situations or clinical findings in the patient where you deviate from your prophylaxis standard (medication, dose, duration, age of the patient)? (multiple answers possible, please specify in answer box) [in patients with recurrent urinary tract infections (UTI)]
N/A

Are there situations or clinical findings in the patient where you deviate from your prophylaxis standard (medication, dose, duration, age of the patient)? (multiple answers possible, please specify in answer box) [if multidrug-resistant organisms (MDRO) are detected in urine cultures]
N/A

Are there situations or clinical findings in the patient where you deviate from your prophylaxis standard (medication, dose, duration, age of the patient)? (multiple answers possible, please specify in answer box) [in newborns]
N/A

Are there situations in which you use intravenous prophylaxis?
N/A

Voiding analysis

Are there differences in antibiotic prophylaxis between VUS and VCUG in your department?
N/A

Is a non-pathological urine strip test required in preparation for VUS/VCUG in your department?
N/A

Is a non-pathological urine culture required in preparation for VUS/VCUG in your department?
N/A

Do patients only receive post-procedure prophylaxis if vesicoureteral reflux (VUR) is detected in VUS/VCUG?
N/A

Do other MUS/MCU findings (e.g. urethral valves) have an influence on post-procedure prophylaxis?
N/A

End of survey

May we contact you personally if we have further questions?
N/A

Survey response 112

Response ID
114

Date submitted
2025-01-29 07:22:37

Last page
5

Start language
de

Seed
1184752594

Date started
2025-01-29 07:13:56

Date last action
2025-01-29 07:22:37

Standard Operation Procedure

Who fills out this survey?
Pediatric nephrologist

Who fills out this survey? [Other]

In which type of hospital are you employed?
University Hospital

In which type of hospital are you employed? [Other]

In which country do you work?
Deutschland

Does your department have a defined protocol for infection prophylaxis in the context of VUS/VCUG?
Yes

Has this protocol been transformed into a written standard operating procedure (SOP)?
Yes

Which department is the author of the SOP for infection prophylaxis in VUS/VCUG?
Pediatrics

Which department is the author of the SOP for infection prophylaxis in VUS/VCUG? [Other]

Antibiotic Prophylaxis

Are there situations/constellations in which you do not use antibiotic prophylaxis for VUS/VCUG?
Yes

Please specify these situations/constellations: [girl with first uncomplicated UTI without urinary tract dilation in ultrasound]
Yes

Please specify these situations/constellations: [boy with first uncomplicated UTI without urinary tract dilation in ultrasound]
Yes

Please specify these situations/constellations: [in patients with low grade urinary tract dilation (up to 2nd grade) and no UTI in medical history]
Yes

Please specify these situations/constellations: [Other]

If you use antibiotic prophylaxis: which mediaction do you use? (multiple answers and subdivision into age groups possible) [Trimethoprim]
No

If you use antibiotic prophylaxis: which mediaction do you use? (multiple answers and subdivision into age groups possible) [Nitrofurantoin]
No

If you use antibiotic prophylaxis: which mediaction do you use? (multiple answers and subdivision into age groups possible) [Amoxicillin]
No

If you use antibiotic prophylaxis: which mediaction do you use? (multiple answers and subdivision into age groups possible) [Cefaclor]
Yes

If you use antibiotic prophylaxis: which mediaction do you use? (multiple answers and subdivision into age groups possible) [Amoxicillin/clavulanic acid]
No

If you use antibiotic prophylaxis: which mediaction do you use? (multiple answers and subdivision into age groups possible) [Other]
No

Trimethoprim: which time schedule do you use? [Day(s) before VUS/VCUG, how many?]
N/A

Trimethoprim: which time schedule do you use? [Day of VUS/VCUG]
N/A

Trimethoprim: which time schedule do you use? [Day(s) after VUS/VCUG, how many?]
N/A

Nitrofurantoin: which time schedule do you use? [Day(s) before VUS/VCUG, how many?]
N/A

Nitrofurantoin: which time schedule do you use? [Day of VUS/VCUG]
N/A

Nitrofurantoin: which time schedule do you use? [Day(s) after VUS/VCUG, how many?]
N/A

Amoxicillin: which time schedule do you use? [Day(s) before VUS/VCUG, how many?]
N/A

Amoxicillin: which time schedule do you use? [Day of VUS/VCUG]
N/A

Amoxicillin: which time schedule do you use? [Day(s) after VUS/VCUG, how many?]
N/A

Cefaclor: in what dosage?
prophylactic (10 mg/kg bodyweight/d)

Cefaclor: in what dosage? [Other]

Cefaclor: for which age group do you use cefaclor?
Alle

Cefaclor: which time schedule do you use? [Days(s) before VUS/VCUG, how many?]
No

Cefaclor: which time schedule do you use? [Days(s) before VUS/VCUG, how many?][Comment]

Cefaclor: which time schedule do you use? [Day of VUS/VCUG]
Yes

Cefaclor: which time schedule do you use? [Day of VUS/VCUG][Comment]

Cefaclor: which time schedule do you use? [Day(s) after VUS/VCUG, how many?]
Yes

Cefaclor: which time schedule do you use? [Day(s) after VUS/VCUG, how many?][Comment]
bei nachgewiesenem höhergradigem (3. plus) Reflux ggfs. 2 Tage länger

Amoxicillin/clavulanic acid: in what dosage? (Amoxicillin portion) [prophylaktic (10-20 mg/Kg bodyweight/d)]
N/A

Amoxicillin/clavulanic acid: in what dosage? (Amoxicillin portion) [double prophylactic (20-40 mg/Kg bodyweight/d)]
N/A

Amoxicillin/clavulanic acid: in what dosage? (Amoxicillin portion) [therapeutic (80-100 mg/Kg bodyweight/d)]
N/A

Amoxicillin/clavulanic acid: which time schedule do you use? [Day(s) before VUS/VCUG]
N/A

Amoxicillin/clavulanic acid: which time schedule do you use? [Day of VUS/VCUG]
N/A

Amoxicillin/clavulanic acid: which time schedule do you use? [Day(s) after VUS/VCUG]
N/A

Other: which time schedule do you use? [Day(s) before VUS/VCUG, how many?]
N/A

Other: which time schedule do you use? [Day of VUS/VCUG]
N/A

Other: which time schedule do you use? [Day(s) after VUS/VCUG, how many?]
N/A

Indication for prophylaxis

Which patients receive periinterventional antibiotic prophylaxis? (multiple answers possible) [all]
No

Which patients receive periinterventional antibiotic prophylaxis? (multiple answers possible) [patients post urinary tract infection/pyelonephritis/urosepsis]
No

Which patients receive periinterventional antibiotic prophylaxis? (multiple answers possible) [patients with urinary tract dilation in ultrasound]
No

Which patients receive periinterventional antibiotic prophylaxis? (multiple answers possible) [patients with prenatal urinary tract dilation]
No

Which patients receive periinterventional antibiotic prophylaxis? (multiple answers possible) [patients post VUR surgery]
No

Which patients receive periinterventional antibiotic prophylaxis? (multiple answers possible) [none]
No

Which patients receive periinterventional antibiotic prophylaxis? (multiple answers possible) [Other]
ggfs., wenn höhergradiger Reflux

Are there situations or clinical findings in the patient where you deviate from your prophylaxis standard (medication, dose, duration, age of the patient)? (multiple answers possible, please specify in answer box) [in case of already known vesicoureteral reflux (VUR)]
Yes

Are there situations or clinical findings in the patient where you deviate from your prophylaxis standard (medication, dose, duration, age of the patient)? (multiple answers possible, please specify in answer box) [in case of already known vesicoureteral reflux (VUR)][Comment]
ggfs., wenn höhergradiger Reflux

Are there situations or clinical findings in the patient where you deviate from your prophylaxis standard (medication, dose, duration, age of the patient)? (multiple answers possible, please specify in answer box) [in patients with recurrent urinary tract infections (UTI)]
Yes

Are there situations or clinical findings in the patient where you deviate from your prophylaxis standard (medication, dose, duration, age of the patient)? (multiple answers possible, please specify in answer box) [in patients with recurrent urinary tract infections (UTI)][Comment]
ggfs., wenn höhergradiger Reflux

Are there situations or clinical findings in the patient where you deviate from your prophylaxis standard (medication, dose, duration, age of the patient)? (multiple answers possible, please specify in answer box) [if multidrug-resistant organisms (MDRO) are detected in urine cultures]
No

Are there situations or clinical findings in the patient where you deviate from your prophylaxis standard (medication, dose, duration, age of the patient)? (multiple answers possible, please specify in answer box) [if multidrug-resistant organisms (MDRO) are detected in urine cultures][Comment]

Are there situations or clinical findings in the patient where you deviate from your prophylaxis standard (medication, dose, duration, age of the patient)? (multiple answers possible, please specify in answer box) [in newborns]
No

Are there situations or clinical findings in the patient where you deviate from your prophylaxis standard (medication, dose, duration, age of the patient)? (multiple answers possible, please specify in answer box) [in newborns][Comment]

Are there situations or clinical findings in the patient where you deviate from your prophylaxis standard (medication, dose, duration, age of the patient)? (multiple answers possible, please specify in answer box) [Other]

Are there situations or clinical findings in the patient where you deviate from your prophylaxis standard (medication, dose, duration, age of the patient)? (multiple answers possible, please specify in answer box) [Other comment]

Are there situations in which you use intravenous prophylaxis?
Yes

In which situations?
ggfs., wenn MCU "erzwungen" werden muss oder MCU(s) vorher aufgrund von akzidentiell entdeckten HWI nicht gemacht werden konnte.

Voiding analysis

Are there differences in antibiotic prophylaxis between VUS and VCUG in your department?
No

Is a non-pathological urine strip test required in preparation for VUS/VCUG in your department?
Yes

What is the maximum amount of days accepted for urine strip test to be performed prior to the exam?
Am Tag der MCU wird im Rahmen der Katherisierung ein Urinstatus durchgeführt, selten auch bei "komplizierten" Verhältnissen (schwierige Anlage/ev. in Sedierung) am Vortag. Auffälliger Urinstatus ist aber kein Ausschlusskriterium bei gutem AZ und wenn MCU am Untersuchungstag durchgeführt werden "muss"

Is a non-pathological urine culture required in preparation for VUS/VCUG in your department?
No

Do patients only receive post-procedure prophylaxis if vesicoureteral reflux (VUR) is detected in VUS/VCUG?
No

Do other MUS/MCU findings (e.g. urethral valves) have an influence on post-procedure prophylaxis?
No

End of survey

Thank you for taking the time to answer this survey!

May we contact you personally if we have further questions?
Yes

E-mail address:
boris.utsch@paediat.med.uni-giessen.de

Survey response 113

Response ID
115

Date submitted
2025-01-29 07:36:20

Last page
5

Start language
de

Seed
205504777

Date started
2025-01-29 07:32:21

Date last action
2025-01-29 07:36:20

Standard Operation Procedure

Who fills out this survey?
Pediatric nephrologist

Who fills out this survey? [Other]

In which type of hospital are you employed?
University Hospital

In which type of hospital are you employed? [Other]

In which country do you work?
Österreich

Does your department have a defined protocol for infection prophylaxis in the context of VUS/VCUG?
Yes

Has this protocol been transformed into a written standard operating procedure (SOP)?
Yes

Which department is the author of the SOP for infection prophylaxis in VUS/VCUG?
Pediatric nephrology

Which department is the author of the SOP for infection prophylaxis in VUS/VCUG? [Other]

Antibiotic Prophylaxis

Are there situations/constellations in which you do not use antibiotic prophylaxis for VUS/VCUG?
No

Please specify these situations/constellations: [girl with first uncomplicated UTI without urinary tract dilation in ultrasound]
N/A

Please specify these situations/constellations: [boy with first uncomplicated UTI without urinary tract dilation in ultrasound]
N/A

Please specify these situations/constellations: [in patients with low grade urinary tract dilation (up to 2nd grade) and no UTI in medical history]
N/A

If you use antibiotic prophylaxis: which mediaction do you use? (multiple answers and subdivision into age groups possible) [Trimethoprim]
No

If you use antibiotic prophylaxis: which mediaction do you use? (multiple answers and subdivision into age groups possible) [Nitrofurantoin]
Yes

If you use antibiotic prophylaxis: which mediaction do you use? (multiple answers and subdivision into age groups possible) [Amoxicillin]
No

If you use antibiotic prophylaxis: which mediaction do you use? (multiple answers and subdivision into age groups possible) [Cefaclor]
Yes

If you use antibiotic prophylaxis: which mediaction do you use? (multiple answers and subdivision into age groups possible) [Amoxicillin/clavulanic acid]
No

If you use antibiotic prophylaxis: which mediaction do you use? (multiple answers and subdivision into age groups possible) [Other]
No

Trimethoprim: which time schedule do you use? [Day(s) before VUS/VCUG, how many?]
N/A

Trimethoprim: which time schedule do you use? [Day of VUS/VCUG]
N/A

Trimethoprim: which time schedule do you use? [Day(s) after VUS/VCUG, how many?]
N/A

Nitrofurantoin: in what dosage?
prophylactic (1-2 mg/kg bodyweight/d)

Nitrofurantoin: in what dosage? [Other]

Nitrofurantoin: for which age group do you use nitrofurantoin?
ab 9. LM

Nitrofurantoin: which time schedule do you use? [Day(s) before VUS/VCUG, how many?]
No

Nitrofurantoin: which time schedule do you use? [Day(s) before VUS/VCUG, how many?][Comment]

Nitrofurantoin: which time schedule do you use? [Day of VUS/VCUG]
Yes

Nitrofurantoin: which time schedule do you use? [Day of VUS/VCUG][Comment]

Nitrofurantoin: which time schedule do you use? [Day(s) after VUS/VCUG, how many?]
Yes

Nitrofurantoin: which time schedule do you use? [Day(s) after VUS/VCUG, how many?][Comment]
über insgesamt 48h :1x1 mg/kg/abends, 2 Gaben

Amoxicillin: which time schedule do you use? [Day(s) before VUS/VCUG, how many?]
N/A

Amoxicillin: which time schedule do you use? [Day of VUS/VCUG]
N/A

Amoxicillin: which time schedule do you use? [Day(s) after VUS/VCUG, how many?]
N/A

Cefaclor: in what dosage?
prophylactic (10 mg/kg bodyweight/d)

Cefaclor: in what dosage? [Other]

Cefaclor: for which age group do you use cefaclor?
unter 9. LM

Cefaclor: which time schedule do you use? [Days(s) before VUS/VCUG, how many?]
No

Cefaclor: which time schedule do you use? [Days(s) before VUS/VCUG, how many?][Comment]

Cefaclor: which time schedule do you use? [Day of VUS/VCUG]
Yes

Cefaclor: which time schedule do you use? [Day of VUS/VCUG][Comment]

Cefaclor: which time schedule do you use? [Day(s) after VUS/VCUG, how many?]
Yes

Cefaclor: which time schedule do you use? [Day(s) after VUS/VCUG, how many?][Comment]
über ins. 48h 1x 10 mg/kg abends, 2 Gaben

Amoxicillin/clavulanic acid: in what dosage? (Amoxicillin portion) [prophylaktic (10-20 mg/Kg bodyweight/d)]
N/A

Amoxicillin/clavulanic acid: in what dosage? (Amoxicillin portion) [double prophylactic (20-40 mg/Kg bodyweight/d)]
N/A

Amoxicillin/clavulanic acid: in what dosage? (Amoxicillin portion) [therapeutic (80-100 mg/Kg bodyweight/d)]
N/A

Amoxicillin/clavulanic acid: which time schedule do you use? [Day(s) before VUS/VCUG]
N/A

Amoxicillin/clavulanic acid: which time schedule do you use? [Day of VUS/VCUG]
N/A

Amoxicillin/clavulanic acid: which time schedule do you use? [Day(s) after VUS/VCUG]
N/A

Other: which time schedule do you use? [Day(s) before VUS/VCUG, how many?]
N/A

Other: which time schedule do you use? [Day of VUS/VCUG]
N/A

Other: which time schedule do you use? [Day(s) after VUS/VCUG, how many?]
N/A

Indication for prophylaxis

Which patients receive periinterventional antibiotic prophylaxis? (multiple answers possible) [all]
Yes

Which patients receive periinterventional antibiotic prophylaxis? (multiple answers possible) [patients post urinary tract infection/pyelonephritis/urosepsis]
N/A

Which patients receive periinterventional antibiotic prophylaxis? (multiple answers possible) [patients with urinary tract dilation in ultrasound]
N/A

Which patients receive periinterventional antibiotic prophylaxis? (multiple answers possible) [patients with prenatal urinary tract dilation]
N/A

Which patients receive periinterventional antibiotic prophylaxis? (multiple answers possible) [patients post VUR surgery]
N/A

Which patients receive periinterventional antibiotic prophylaxis? (multiple answers possible) [none]
N/A

Are there situations or clinical findings in the patient where you deviate from your prophylaxis standard (medication, dose, duration, age of the patient)? (multiple answers possible, please specify in answer box) [in case of already known vesicoureteral reflux (VUR)]
No

Are there situations or clinical findings in the patient where you deviate from your prophylaxis standard (medication, dose, duration, age of the patient)? (multiple answers possible, please specify in answer box) [in case of already known vesicoureteral reflux (VUR)][Comment]

Are there situations or clinical findings in the patient where you deviate from your prophylaxis standard (medication, dose, duration, age of the patient)? (multiple answers possible, please specify in answer box) [in patients with recurrent urinary tract infections (UTI)]
No

Are there situations or clinical findings in the patient where you deviate from your prophylaxis standard (medication, dose, duration, age of the patient)? (multiple answers possible, please specify in answer box) [in patients with recurrent urinary tract infections (UTI)][Comment]

Are there situations or clinical findings in the patient where you deviate from your prophylaxis standard (medication, dose, duration, age of the patient)? (multiple answers possible, please specify in answer box) [if multidrug-resistant organisms (MDRO) are detected in urine cultures]
No

Are there situations or clinical findings in the patient where you deviate from your prophylaxis standard (medication, dose, duration, age of the patient)? (multiple answers possible, please specify in answer box) [if multidrug-resistant organisms (MDRO) are detected in urine cultures][Comment]

Are there situations or clinical findings in the patient where you deviate from your prophylaxis standard (medication, dose, duration, age of the patient)? (multiple answers possible, please specify in answer box) [in newborns]
No

Are there situations or clinical findings in the patient where you deviate from your prophylaxis standard (medication, dose, duration, age of the patient)? (multiple answers possible, please specify in answer box) [in newborns][Comment]

Are there situations or clinical findings in the patient where you deviate from your prophylaxis standard (medication, dose, duration, age of the patient)? (multiple answers possible, please specify in answer box) [Other]
keine

Are there situations or clinical findings in the patient where you deviate from your prophylaxis standard (medication, dose, duration, age of the patient)? (multiple answers possible, please specify in answer box) [Other comment]

Are there situations in which you use intravenous prophylaxis?
No

Voiding analysis

Are there differences in antibiotic prophylaxis between VUS and VCUG in your department?
No

Is a non-pathological urine strip test required in preparation for VUS/VCUG in your department?
No

Is a non-pathological urine culture required in preparation for VUS/VCUG in your department?
No

Do patients only receive post-procedure prophylaxis if vesicoureteral reflux (VUR) is detected in VUS/VCUG?
No

Do other MUS/MCU findings (e.g. urethral valves) have an influence on post-procedure prophylaxis?
No

End of survey

Thank you for taking the time to answer this survey!

May we contact you personally if we have further questions?
Yes

E-mail address:
cecile.dau@tulln.lknoe.at

Survey response 114

Response ID
116

Date submitted
2025-01-29 08:29:36

Last page
5

Start language
en

Seed
1363151624

Date started
2025-01-29 08:13:49

Date last action
2025-01-29 08:29:36

Standard Operation Procedure

Who fills out this survey?
Pediatrician

Who fills out this survey? [Other]

In which type of hospital are you employed?
Community hospital (

In which type of hospital are you employed? [Other]

In which country do you work?
Germany

Does your department have a defined protocol for infection prophylaxis in the context of VUS/VCUG?
No

Has this protocol been transformed into a written standard operating procedure (SOP)?
N/A

Antibiotic Prophylaxis

Are there situations/constellations in which you do not use antibiotic prophylaxis for VUS/VCUG?
Yes

Please specify these situations/constellations: [girl with first uncomplicated UTI without urinary tract dilation in ultrasound]
No

Please specify these situations/constellations: [boy with first uncomplicated UTI without urinary tract dilation in ultrasound]
No

Please specify these situations/constellations: [in patients with low grade urinary tract dilation (up to 2nd grade) and no UTI in medical history]
No

Please specify these situations/constellations: [Other]
we avoid prophylaxis whenever bedside urine sample is unremarkable

If you use antibiotic prophylaxis: which mediaction do you use? (multiple answers and subdivision into age groups possible) [Trimethoprim]
Yes

If you use antibiotic prophylaxis: which mediaction do you use? (multiple answers and subdivision into age groups possible) [Nitrofurantoin]
No

If you use antibiotic prophylaxis: which mediaction do you use? (multiple answers and subdivision into age groups possible) [Amoxicillin]
No

If you use antibiotic prophylaxis: which mediaction do you use? (multiple answers and subdivision into age groups possible) [Cefaclor]
No

If you use antibiotic prophylaxis: which mediaction do you use? (multiple answers and subdivision into age groups possible) [Amoxicillin/clavulanic acid]
No

If you use antibiotic prophylaxis: which mediaction do you use? (multiple answers and subdivision into age groups possible) [Other]
No

Trimethoprim: in what dosage?
prophylactic (2 mg/kg bodyweight/d)

Trimethoprim: in what dosage? [Other]

Trimethoprim: for which age group do you use trimethoprim?
any >4 wks

Trimethoprim: which time schedule do you use? [Day(s) before VUS/VCUG, how many?]
No

Trimethoprim: which time schedule do you use? [Day(s) before VUS/VCUG, how many?][Comment]

Trimethoprim: which time schedule do you use? [Day of VUS/VCUG]
Yes

Trimethoprim: which time schedule do you use? [Day of VUS/VCUG][Comment]

Trimethoprim: which time schedule do you use? [Day(s) after VUS/VCUG, how many?]
No

Trimethoprim: which time schedule do you use? [Day(s) after VUS/VCUG, how many?][Comment]

Nitrofurantoin: which time schedule do you use? [Day(s) before VUS/VCUG, how many?]
N/A

Nitrofurantoin: which time schedule do you use? [Day of VUS/VCUG]
N/A

Nitrofurantoin: which time schedule do you use? [Day(s) after VUS/VCUG, how many?]
N/A

Amoxicillin: which time schedule do you use? [Day(s) before VUS/VCUG, how many?]
N/A

Amoxicillin: which time schedule do you use? [Day of VUS/VCUG]
N/A

Amoxicillin: which time schedule do you use? [Day(s) after VUS/VCUG, how many?]
N/A

Cefaclor: which time schedule do you use? [Days(s) before VUS/VCUG, how many?]
N/A

Cefaclor: which time schedule do you use? [Day of VUS/VCUG]
N/A

Cefaclor: which time schedule do you use? [Day(s) after VUS/VCUG, how many?]
N/A

Amoxicillin/clavulanic acid: in what dosage? (Amoxicillin portion) [prophylaktic (10-20 mg/Kg bodyweight/d)]
N/A

Amoxicillin/clavulanic acid: in what dosage? (Amoxicillin portion) [double prophylactic (20-40 mg/Kg bodyweight/d)]
N/A

Amoxicillin/clavulanic acid: in what dosage? (Amoxicillin portion) [therapeutic (80-100 mg/Kg bodyweight/d)]
N/A

Amoxicillin/clavulanic acid: which time schedule do you use? [Day(s) before VUS/VCUG]
N/A

Amoxicillin/clavulanic acid: which time schedule do you use? [Day of VUS/VCUG]
N/A

Amoxicillin/clavulanic acid: which time schedule do you use? [Day(s) after VUS/VCUG]
N/A

Other: which time schedule do you use? [Day(s) before VUS/VCUG, how many?]
N/A

Other: which time schedule do you use? [Day of VUS/VCUG]
N/A

Other: which time schedule do you use? [Day(s) after VUS/VCUG, how many?]
N/A

Indication for prophylaxis

Which patients receive periinterventional antibiotic prophylaxis? (multiple answers possible) [all]
No

Which patients receive periinterventional antibiotic prophylaxis? (multiple answers possible) [patients post urinary tract infection/pyelonephritis/urosepsis]
No

Which patients receive periinterventional antibiotic prophylaxis? (multiple answers possible) [patients with urinary tract dilation in ultrasound]
No

Which patients receive periinterventional antibiotic prophylaxis? (multiple answers possible) [patients with prenatal urinary tract dilation]
No

Which patients receive periinterventional antibiotic prophylaxis? (multiple answers possible) [patients post VUR surgery]
No

Which patients receive periinterventional antibiotic prophylaxis? (multiple answers possible) [none]
Yes

Which patients receive periinterventional antibiotic prophylaxis? (multiple answers possible) [Other]

Are there situations or clinical findings in the patient where you deviate from your prophylaxis standard (medication, dose, duration, age of the patient)? (multiple answers possible, please specify in answer box) [in case of already known vesicoureteral reflux (VUR)]
No

Are there situations or clinical findings in the patient where you deviate from your prophylaxis standard (medication, dose, duration, age of the patient)? (multiple answers possible, please specify in answer box) [in case of already known vesicoureteral reflux (VUR)][Comment]

Are there situations or clinical findings in the patient where you deviate from your prophylaxis standard (medication, dose, duration, age of the patient)? (multiple answers possible, please specify in answer box) [in patients with recurrent urinary tract infections (UTI)]
No

Are there situations or clinical findings in the patient where you deviate from your prophylaxis standard (medication, dose, duration, age of the patient)? (multiple answers possible, please specify in answer box) [in patients with recurrent urinary tract infections (UTI)][Comment]

Are there situations or clinical findings in the patient where you deviate from your prophylaxis standard (medication, dose, duration, age of the patient)? (multiple answers possible, please specify in answer box) [if multidrug-resistant organisms (MDRO) are detected in urine cultures]
No

Are there situations or clinical findings in the patient where you deviate from your prophylaxis standard (medication, dose, duration, age of the patient)? (multiple answers possible, please specify in answer box) [if multidrug-resistant organisms (MDRO) are detected in urine cultures][Comment]

Are there situations or clinical findings in the patient where you deviate from your prophylaxis standard (medication, dose, duration, age of the patient)? (multiple answers possible, please specify in answer box) [in newborns]
No

Are there situations or clinical findings in the patient where you deviate from your prophylaxis standard (medication, dose, duration, age of the patient)? (multiple answers possible, please specify in answer box) [in newborns][Comment]

Are there situations or clinical findings in the patient where you deviate from your prophylaxis standard (medication, dose, duration, age of the patient)? (multiple answers possible, please specify in answer box) [Other]
consider prophylaxis

Are there situations or clinical findings in the patient where you deviate from your prophylaxis standard (medication, dose, duration, age of the patient)? (multiple answers possible, please specify in answer box) [Other comment]
if access to bladder complicated

Are there situations in which you use intravenous prophylaxis?
No

Voiding analysis

Are there differences in antibiotic prophylaxis between VUS and VCUG in your department?
Yes

To what extent?
Radiologists tend to apply prophylaxis

Is a non-pathological urine strip test required in preparation for VUS/VCUG in your department?
Yes

What is the maximum amount of days accepted for urine strip test to be performed prior to the exam?
sample of the same day

Is a non-pathological urine culture required in preparation for VUS/VCUG in your department?
No

Do patients only receive post-procedure prophylaxis if vesicoureteral reflux (VUR) is detected in VUS/VCUG?
No

Do other MUS/MCU findings (e.g. urethral valves) have an influence on post-procedure prophylaxis?
Yes

Please specify:
see above

End of survey

Thank you for taking the time to answer this survey!

May we contact you personally if we have further questions?
No

Survey response 115

Response ID
117

Date submitted
2025-01-29 17:31:15

Last page
5

Start language
de

Seed
866444618

Date started
2025-01-29 17:26:19

Date last action
2025-01-29 17:31:15

Standard Operation Procedure

Who fills out this survey?
Pediatric nephrologist

Who fills out this survey? [Other]

In which type of hospital are you employed?
University Hospital

In which type of hospital are you employed? [Other]

In which country do you work?
Tschechien

Does your department have a defined protocol for infection prophylaxis in the context of VUS/VCUG?
No

Has this protocol been transformed into a written standard operating procedure (SOP)?
N/A

Antibiotic Prophylaxis

Are there situations/constellations in which you do not use antibiotic prophylaxis for VUS/VCUG?
No

Please specify these situations/constellations: [girl with first uncomplicated UTI without urinary tract dilation in ultrasound]
N/A

Please specify these situations/constellations: [boy with first uncomplicated UTI without urinary tract dilation in ultrasound]
N/A

Please specify these situations/constellations: [in patients with low grade urinary tract dilation (up to 2nd grade) and no UTI in medical history]
N/A

If you use antibiotic prophylaxis: which mediaction do you use? (multiple answers and subdivision into age groups possible) [Trimethoprim]
Yes

If you use antibiotic prophylaxis: which mediaction do you use? (multiple answers and subdivision into age groups possible) [Nitrofurantoin]
No

If you use antibiotic prophylaxis: which mediaction do you use? (multiple answers and subdivision into age groups possible) [Amoxicillin]
No

If you use antibiotic prophylaxis: which mediaction do you use? (multiple answers and subdivision into age groups possible) [Cefaclor]
No

If you use antibiotic prophylaxis: which mediaction do you use? (multiple answers and subdivision into age groups possible) [Amoxicillin/clavulanic acid]
No

If you use antibiotic prophylaxis: which mediaction do you use? (multiple answers and subdivision into age groups possible) [Other]
No

Trimethoprim: in what dosage?
prophylactic (2 mg/kg bodyweight/d)

Trimethoprim: in what dosage? [Other]

Trimethoprim: for which age group do you use trimethoprim?
ab 1 Monat

Trimethoprim: which time schedule do you use? [Day(s) before VUS/VCUG, how many?]
No

Trimethoprim: which time schedule do you use? [Day(s) before VUS/VCUG, how many?][Comment]

Trimethoprim: which time schedule do you use? [Day of VUS/VCUG]
Yes

Trimethoprim: which time schedule do you use? [Day of VUS/VCUG][Comment]

Trimethoprim: which time schedule do you use? [Day(s) after VUS/VCUG, how many?]
Yes

Trimethoprim: which time schedule do you use? [Day(s) after VUS/VCUG, how many?][Comment]
1

Nitrofurantoin: which time schedule do you use? [Day(s) before VUS/VCUG, how many?]
N/A

Nitrofurantoin: which time schedule do you use? [Day of VUS/VCUG]
N/A

Nitrofurantoin: which time schedule do you use? [Day(s) after VUS/VCUG, how many?]
N/A

Amoxicillin: which time schedule do you use? [Day(s) before VUS/VCUG, how many?]
N/A

Amoxicillin: which time schedule do you use? [Day of VUS/VCUG]
N/A

Amoxicillin: which time schedule do you use? [Day(s) after VUS/VCUG, how many?]
N/A

Cefaclor: which time schedule do you use? [Days(s) before VUS/VCUG, how many?]
N/A

Cefaclor: which time schedule do you use? [Day of VUS/VCUG]
N/A

Cefaclor: which time schedule do you use? [Day(s) after VUS/VCUG, how many?]
N/A

Amoxicillin/clavulanic acid: in what dosage? (Amoxicillin portion) [prophylaktic (10-20 mg/Kg bodyweight/d)]
N/A

Amoxicillin/clavulanic acid: in what dosage? (Amoxicillin portion) [double prophylactic (20-40 mg/Kg bodyweight/d)]
N/A

Amoxicillin/clavulanic acid: in what dosage? (Amoxicillin portion) [therapeutic (80-100 mg/Kg bodyweight/d)]
N/A

Amoxicillin/clavulanic acid: which time schedule do you use? [Day(s) before VUS/VCUG]
N/A

Amoxicillin/clavulanic acid: which time schedule do you use? [Day of VUS/VCUG]
N/A

Amoxicillin/clavulanic acid: which time schedule do you use? [Day(s) after VUS/VCUG]
N/A

Other: which time schedule do you use? [Day(s) before VUS/VCUG, how many?]
N/A

Other: which time schedule do you use? [Day of VUS/VCUG]
N/A

Other: which time schedule do you use? [Day(s) after VUS/VCUG, how many?]
N/A

Indication for prophylaxis

Which patients receive periinterventional antibiotic prophylaxis? (multiple answers possible) [all]
Yes

Which patients receive periinterventional antibiotic prophylaxis? (multiple answers possible) [patients post urinary tract infection/pyelonephritis/urosepsis]
N/A

Which patients receive periinterventional antibiotic prophylaxis? (multiple answers possible) [patients with urinary tract dilation in ultrasound]
N/A

Which patients receive periinterventional antibiotic prophylaxis? (multiple answers possible) [patients with prenatal urinary tract dilation]
N/A

Which patients receive periinterventional antibiotic prophylaxis? (multiple answers possible) [patients post VUR surgery]
N/A

Which patients receive periinterventional antibiotic prophylaxis? (multiple answers possible) [none]
N/A

Are there situations or clinical findings in the patient where you deviate from your prophylaxis standard (medication, dose, duration, age of the patient)? (multiple answers possible, please specify in answer box) [in case of already known vesicoureteral reflux (VUR)]
No

Are there situations or clinical findings in the patient where you deviate from your prophylaxis standard (medication, dose, duration, age of the patient)? (multiple answers possible, please specify in answer box) [in case of already known vesicoureteral reflux (VUR)][Comment]

Are there situations or clinical findings in the patient where you deviate from your prophylaxis standard (medication, dose, duration, age of the patient)? (multiple answers possible, please specify in answer box) [in patients with recurrent urinary tract infections (UTI)]
No

Are there situations or clinical findings in the patient where you deviate from your prophylaxis standard (medication, dose, duration, age of the patient)? (multiple answers possible, please specify in answer box) [in patients with recurrent urinary tract infections (UTI)][Comment]

Are there situations or clinical findings in the patient where you deviate from your prophylaxis standard (medication, dose, duration, age of the patient)? (multiple answers possible, please specify in answer box) [if multidrug-resistant organisms (MDRO) are detected in urine cultures]
Yes

Are there situations or clinical findings in the patient where you deviate from your prophylaxis standard (medication, dose, duration, age of the patient)? (multiple answers possible, please specify in answer box) [if multidrug-resistant organisms (MDRO) are detected in urine cultures][Comment]
je nach Empfindlichkeit

Are there situations or clinical findings in the patient where you deviate from your prophylaxis standard (medication, dose, duration, age of the patient)? (multiple answers possible, please specify in answer box) [in newborns]
Yes

Are there situations or clinical findings in the patient where you deviate from your prophylaxis standard (medication, dose, duration, age of the patient)? (multiple answers possible, please specify in answer box) [in newborns][Comment]
Amox

Are there situations or clinical findings in the patient where you deviate from your prophylaxis standard (medication, dose, duration, age of the patient)? (multiple answers possible, please specify in answer box) [Other]

Are there situations or clinical findings in the patient where you deviate from your prophylaxis standard (medication, dose, duration, age of the patient)? (multiple answers possible, please specify in answer box) [Other comment]

Are there situations in which you use intravenous prophylaxis?
No

Voiding analysis

Are there differences in antibiotic prophylaxis between VUS and VCUG in your department?
No

Is a non-pathological urine strip test required in preparation for VUS/VCUG in your department?
Yes

What is the maximum amount of days accepted for urine strip test to be performed prior to the exam?
1-7

Is a non-pathological urine culture required in preparation for VUS/VCUG in your department?
Yes

What is the maximum amount of days accepted for urine culture to be performed prior to the exam?
1-7

Do patients only receive post-procedure prophylaxis if vesicoureteral reflux (VUR) is detected in VUS/VCUG?
No

Do other MUS/MCU findings (e.g. urethral valves) have an influence on post-procedure prophylaxis?
No

End of survey

Thank you for taking the time to answer this survey!

May we contact you personally if we have further questions?
No

Survey response 116

Response ID
118

Last page
1

Start language
de

Seed
1076217270

Date started
2025-01-30 13:40:22

Date last action
2025-01-30 13:41:15

Standard Operation Procedure

Who fills out this survey?
Pediatrician

Who fills out this survey? [Other]

In which type of hospital are you employed?
Community hospital (

In which type of hospital are you employed? [Other]

In which country do you work?
Deutschland

Does your department have a defined protocol for infection prophylaxis in the context of VUS/VCUG?
No

Has this protocol been transformed into a written standard operating procedure (SOP)?
N/A

Antibiotic Prophylaxis

Are there situations/constellations in which you do not use antibiotic prophylaxis for VUS/VCUG?
N/A

Please specify these situations/constellations: [girl with first uncomplicated UTI without urinary tract dilation in ultrasound]
N/A

Please specify these situations/constellations: [boy with first uncomplicated UTI without urinary tract dilation in ultrasound]
N/A

Please specify these situations/constellations: [in patients with low grade urinary tract dilation (up to 2nd grade) and no UTI in medical history]
N/A

If you use antibiotic prophylaxis: which mediaction do you use? (multiple answers and subdivision into age groups possible) [Trimethoprim]
N/A

If you use antibiotic prophylaxis: which mediaction do you use? (multiple answers and subdivision into age groups possible) [Nitrofurantoin]
N/A

If you use antibiotic prophylaxis: which mediaction do you use? (multiple answers and subdivision into age groups possible) [Amoxicillin]
N/A

If you use antibiotic prophylaxis: which mediaction do you use? (multiple answers and subdivision into age groups possible) [Cefaclor]
N/A

If you use antibiotic prophylaxis: which mediaction do you use? (multiple answers and subdivision into age groups possible) [Amoxicillin/clavulanic acid]
N/A

If you use antibiotic prophylaxis: which mediaction do you use? (multiple answers and subdivision into age groups possible) [Other]
N/A

Trimethoprim: which time schedule do you use? [Day(s) before VUS/VCUG, how many?]
N/A

Trimethoprim: which time schedule do you use? [Day of VUS/VCUG]
N/A

Trimethoprim: which time schedule do you use? [Day(s) after VUS/VCUG, how many?]
N/A

Nitrofurantoin: which time schedule do you use? [Day(s) before VUS/VCUG, how many?]
N/A

Nitrofurantoin: which time schedule do you use? [Day of VUS/VCUG]
N/A

Nitrofurantoin: which time schedule do you use? [Day(s) after VUS/VCUG, how many?]
N/A

Amoxicillin: which time schedule do you use? [Day(s) before VUS/VCUG, how many?]
N/A

Amoxicillin: which time schedule do you use? [Day of VUS/VCUG]
N/A

Amoxicillin: which time schedule do you use? [Day(s) after VUS/VCUG, how many?]
N/A

Cefaclor: which time schedule do you use? [Days(s) before VUS/VCUG, how many?]
N/A

Cefaclor: which time schedule do you use? [Day of VUS/VCUG]
N/A

Cefaclor: which time schedule do you use? [Day(s) after VUS/VCUG, how many?]
N/A

Amoxicillin/clavulanic acid: in what dosage? (Amoxicillin portion) [prophylaktic (10-20 mg/Kg bodyweight/d)]
N/A

Amoxicillin/clavulanic acid: in what dosage? (Amoxicillin portion) [double prophylactic (20-40 mg/Kg bodyweight/d)]
N/A

Amoxicillin/clavulanic acid: in what dosage? (Amoxicillin portion) [therapeutic (80-100 mg/Kg bodyweight/d)]
N/A

Amoxicillin/clavulanic acid: which time schedule do you use? [Day(s) before VUS/VCUG]
N/A

Amoxicillin/clavulanic acid: which time schedule do you use? [Day of VUS/VCUG]
N/A

Amoxicillin/clavulanic acid: which time schedule do you use? [Day(s) after VUS/VCUG]
N/A

Other: which time schedule do you use? [Day(s) before VUS/VCUG, how many?]
N/A

Other: which time schedule do you use? [Day of VUS/VCUG]
N/A

Other: which time schedule do you use? [Day(s) after VUS/VCUG, how many?]
N/A

Indication for prophylaxis

Which patients receive periinterventional antibiotic prophylaxis? (multiple answers possible) [all]
N/A

Which patients receive periinterventional antibiotic prophylaxis? (multiple answers possible) [patients post urinary tract infection/pyelonephritis/urosepsis]
N/A

Which patients receive periinterventional antibiotic prophylaxis? (multiple answers possible) [patients with urinary tract dilation in ultrasound]
N/A

Which patients receive periinterventional antibiotic prophylaxis? (multiple answers possible) [patients with prenatal urinary tract dilation]
N/A

Which patients receive periinterventional antibiotic prophylaxis? (multiple answers possible) [patients post VUR surgery]
N/A

Which patients receive periinterventional antibiotic prophylaxis? (multiple answers possible) [none]
N/A

Are there situations or clinical findings in the patient where you deviate from your prophylaxis standard (medication, dose, duration, age of the patient)? (multiple answers possible, please specify in answer box) [in case of already known vesicoureteral reflux (VUR)]
N/A

Are there situations or clinical findings in the patient where you deviate from your prophylaxis standard (medication, dose, duration, age of the patient)? (multiple answers possible, please specify in answer box) [in patients with recurrent urinary tract infections (UTI)]
N/A

Are there situations or clinical findings in the patient where you deviate from your prophylaxis standard (medication, dose, duration, age of the patient)? (multiple answers possible, please specify in answer box) [if multidrug-resistant organisms (MDRO) are detected in urine cultures]
N/A

Are there situations or clinical findings in the patient where you deviate from your prophylaxis standard (medication, dose, duration, age of the patient)? (multiple answers possible, please specify in answer box) [in newborns]
N/A

Are there situations in which you use intravenous prophylaxis?
N/A

Voiding analysis

Are there differences in antibiotic prophylaxis between VUS and VCUG in your department?
N/A

Is a non-pathological urine strip test required in preparation for VUS/VCUG in your department?
N/A

Is a non-pathological urine culture required in preparation for VUS/VCUG in your department?
N/A

Do patients only receive post-procedure prophylaxis if vesicoureteral reflux (VUR) is detected in VUS/VCUG?
N/A

Do other MUS/MCU findings (e.g. urethral valves) have an influence on post-procedure prophylaxis?
N/A

End of survey

May we contact you personally if we have further questions?
N/A

Survey response 117

Response ID
119

Date submitted
2025-01-30 14:29:14

Last page
5

Start language
de

Seed
1856893580

Date started
2025-01-30 14:19:23

Date last action
2025-01-30 14:29:14

Standard Operation Procedure

Who fills out this survey?
Pediatrician

Who fills out this survey? [Other]

In which type of hospital are you employed?
Community hospital (

In which type of hospital are you employed? [Other]

In which country do you work?
Deutschland

Does your department have a defined protocol for infection prophylaxis in the context of VUS/VCUG?
No

Has this protocol been transformed into a written standard operating procedure (SOP)?
N/A

Antibiotic Prophylaxis

Are there situations/constellations in which you do not use antibiotic prophylaxis for VUS/VCUG?
Yes

Please specify these situations/constellations: [girl with first uncomplicated UTI without urinary tract dilation in ultrasound]
No

Please specify these situations/constellations: [boy with first uncomplicated UTI without urinary tract dilation in ultrasound]
No

Please specify these situations/constellations: [in patients with low grade urinary tract dilation (up to 2nd grade) and no UTI in medical history]
No

Please specify these situations/constellations: [Other]
in aller Regel keine

If you use antibiotic prophylaxis: which mediaction do you use? (multiple answers and subdivision into age groups possible) [Trimethoprim]
No

If you use antibiotic prophylaxis: which mediaction do you use? (multiple answers and subdivision into age groups possible) [Nitrofurantoin]
No

If you use antibiotic prophylaxis: which mediaction do you use? (multiple answers and subdivision into age groups possible) [Amoxicillin]
No

If you use antibiotic prophylaxis: which mediaction do you use? (multiple answers and subdivision into age groups possible) [Cefaclor]
No

If you use antibiotic prophylaxis: which mediaction do you use? (multiple answers and subdivision into age groups possible) [Amoxicillin/clavulanic acid]
No

If you use antibiotic prophylaxis: which mediaction do you use? (multiple answers and subdivision into age groups possible) [Other]
Yes

Trimethoprim: which time schedule do you use? [Day(s) before VUS/VCUG, how many?]
N/A

Trimethoprim: which time schedule do you use? [Day of VUS/VCUG]
N/A

Trimethoprim: which time schedule do you use? [Day(s) after VUS/VCUG, how many?]
N/A

Nitrofurantoin: which time schedule do you use? [Day(s) before VUS/VCUG, how many?]
N/A

Nitrofurantoin: which time schedule do you use? [Day of VUS/VCUG]
N/A

Nitrofurantoin: which time schedule do you use? [Day(s) after VUS/VCUG, how many?]
N/A

Amoxicillin: which time schedule do you use? [Day(s) before VUS/VCUG, how many?]
N/A

Amoxicillin: which time schedule do you use? [Day of VUS/VCUG]
N/A

Amoxicillin: which time schedule do you use? [Day(s) after VUS/VCUG, how many?]
N/A

Cefaclor: which time schedule do you use? [Days(s) before VUS/VCUG, how many?]
N/A

Cefaclor: which time schedule do you use? [Day of VUS/VCUG]
N/A

Cefaclor: which time schedule do you use? [Day(s) after VUS/VCUG, how many?]
N/A

Amoxicillin/clavulanic acid: in what dosage? (Amoxicillin portion) [prophylaktic (10-20 mg/Kg bodyweight/d)]
N/A

Amoxicillin/clavulanic acid: in what dosage? (Amoxicillin portion) [double prophylactic (20-40 mg/Kg bodyweight/d)]
N/A

Amoxicillin/clavulanic acid: in what dosage? (Amoxicillin portion) [therapeutic (80-100 mg/Kg bodyweight/d)]
N/A

Amoxicillin/clavulanic acid: which time schedule do you use? [Day(s) before VUS/VCUG]
N/A

Amoxicillin/clavulanic acid: which time schedule do you use? [Day of VUS/VCUG]
N/A

Amoxicillin/clavulanic acid: which time schedule do you use? [Day(s) after VUS/VCUG]
N/A

Other: which medication and in what dosage?
Nur in begründeten Ausnahmefällen. Letztes Jahr einziger Fall: 3J Mädchen mit häufig rez. PN durch Pseudomonas aeroginosa. Dieses erhielt 1 Dosis Pip/Taz in therapeutischer Dosierung

Other: for which age group do you use this medication?
s.o.

Other: which time schedule do you use? [Day(s) before VUS/VCUG, how many?]
No

Other: which time schedule do you use? [Day(s) before VUS/VCUG, how many?][Comment]

Other: which time schedule do you use? [Day of VUS/VCUG]
Yes

Other: which time schedule do you use? [Day of VUS/VCUG][Comment]

Other: which time schedule do you use? [Day(s) after VUS/VCUG, how many?]
No

Other: which time schedule do you use? [Day(s) after VUS/VCUG, how many?][Comment]

Indication for prophylaxis

Which patients receive periinterventional antibiotic prophylaxis? (multiple answers possible) [all]
No

Which patients receive periinterventional antibiotic prophylaxis? (multiple answers possible) [patients post urinary tract infection/pyelonephritis/urosepsis]
No

Which patients receive periinterventional antibiotic prophylaxis? (multiple answers possible) [patients with urinary tract dilation in ultrasound]
No

Which patients receive periinterventional antibiotic prophylaxis? (multiple answers possible) [patients with prenatal urinary tract dilation]
No

Which patients receive periinterventional antibiotic prophylaxis? (multiple answers possible) [patients post VUR surgery]
No

Which patients receive periinterventional antibiotic prophylaxis? (multiple answers possible) [none]
No

Which patients receive periinterventional antibiotic prophylaxis? (multiple answers possible) [Other]
s.o. nur in Einzelfällen

Are there situations or clinical findings in the patient where you deviate from your prophylaxis standard (medication, dose, duration, age of the patient)? (multiple answers possible, please specify in answer box) [in case of already known vesicoureteral reflux (VUR)]
No

Are there situations or clinical findings in the patient where you deviate from your prophylaxis standard (medication, dose, duration, age of the patient)? (multiple answers possible, please specify in answer box) [in case of already known vesicoureteral reflux (VUR)][Comment]

Are there situations or clinical findings in the patient where you deviate from your prophylaxis standard (medication, dose, duration, age of the patient)? (multiple answers possible, please specify in answer box) [in patients with recurrent urinary tract infections (UTI)]
No

Are there situations or clinical findings in the patient where you deviate from your prophylaxis standard (medication, dose, duration, age of the patient)? (multiple answers possible, please specify in answer box) [in patients with recurrent urinary tract infections (UTI)][Comment]

Are there situations or clinical findings in the patient where you deviate from your prophylaxis standard (medication, dose, duration, age of the patient)? (multiple answers possible, please specify in answer box) [if multidrug-resistant organisms (MDRO) are detected in urine cultures]
No

Are there situations or clinical findings in the patient where you deviate from your prophylaxis standard (medication, dose, duration, age of the patient)? (multiple answers possible, please specify in answer box) [if multidrug-resistant organisms (MDRO) are detected in urine cultures][Comment]

Are there situations or clinical findings in the patient where you deviate from your prophylaxis standard (medication, dose, duration, age of the patient)? (multiple answers possible, please specify in answer box) [in newborns]
No

Are there situations or clinical findings in the patient where you deviate from your prophylaxis standard (medication, dose, duration, age of the patient)? (multiple answers possible, please specify in answer box) [in newborns][Comment]

Are there situations or clinical findings in the patient where you deviate from your prophylaxis standard (medication, dose, duration, age of the patient)? (multiple answers possible, please specify in answer box) [Other]
s.o.

Are there situations or clinical findings in the patient where you deviate from your prophylaxis standard (medication, dose, duration, age of the patient)? (multiple answers possible, please specify in answer box) [Other comment]
AB nur im Einzelfall

Are there situations in which you use intravenous prophylaxis?
Yes

In which situations?
s.o.

Voiding analysis

Are there differences in antibiotic prophylaxis between VUS and VCUG in your department?
No

Is a non-pathological urine strip test required in preparation for VUS/VCUG in your department?
No

Is a non-pathological urine culture required in preparation for VUS/VCUG in your department?
No

Do patients only receive post-procedure prophylaxis if vesicoureteral reflux (VUR) is detected in VUS/VCUG?
No

Do other MUS/MCU findings (e.g. urethral valves) have an influence on post-procedure prophylaxis?
No

End of survey

Thank you for taking the time to answer this survey!

May we contact you personally if we have further questions?
Yes

E-mail address:
reinhard1.kaiser@gmail.com

Survey response 118

Response ID
120

Date submitted
2025-02-03 10:56:24

Last page
5

Start language
de

Seed
834468212

Date started
2025-02-03 10:50:15

Date last action
2025-02-03 10:56:24

Standard Operation Procedure

Who fills out this survey?
Pediatric nephrologist

Who fills out this survey? [Other]

In which type of hospital are you employed?
Community hospital (>50 beds for pediatric patients)

In which type of hospital are you employed? [Other]

In which country do you work?
Deutschland

Does your department have a defined protocol for infection prophylaxis in the context of VUS/VCUG?
Yes

Has this protocol been transformed into a written standard operating procedure (SOP)?
Yes

Which department is the author of the SOP for infection prophylaxis in VUS/VCUG?
Pediatric nephrology

Which department is the author of the SOP for infection prophylaxis in VUS/VCUG? [Other]

Antibiotic Prophylaxis

Are there situations/constellations in which you do not use antibiotic prophylaxis for VUS/VCUG?
No

Please specify these situations/constellations: [girl with first uncomplicated UTI without urinary tract dilation in ultrasound]
N/A

Please specify these situations/constellations: [boy with first uncomplicated UTI without urinary tract dilation in ultrasound]
N/A

Please specify these situations/constellations: [in patients with low grade urinary tract dilation (up to 2nd grade) and no UTI in medical history]
N/A

If you use antibiotic prophylaxis: which mediaction do you use? (multiple answers and subdivision into age groups possible) [Trimethoprim]
No

If you use antibiotic prophylaxis: which mediaction do you use? (multiple answers and subdivision into age groups possible) [Nitrofurantoin]
No

If you use antibiotic prophylaxis: which mediaction do you use? (multiple answers and subdivision into age groups possible) [Amoxicillin]
No

If you use antibiotic prophylaxis: which mediaction do you use? (multiple answers and subdivision into age groups possible) [Cefaclor]
Yes

If you use antibiotic prophylaxis: which mediaction do you use? (multiple answers and subdivision into age groups possible) [Amoxicillin/clavulanic acid]
No

If you use antibiotic prophylaxis: which mediaction do you use? (multiple answers and subdivision into age groups possible) [Other]
No

Trimethoprim: which time schedule do you use? [Day(s) before VUS/VCUG, how many?]
N/A

Trimethoprim: which time schedule do you use? [Day of VUS/VCUG]
N/A

Trimethoprim: which time schedule do you use? [Day(s) after VUS/VCUG, how many?]
N/A

Nitrofurantoin: which time schedule do you use? [Day(s) before VUS/VCUG, how many?]
N/A

Nitrofurantoin: which time schedule do you use? [Day of VUS/VCUG]
N/A

Nitrofurantoin: which time schedule do you use? [Day(s) after VUS/VCUG, how many?]
N/A

Amoxicillin: which time schedule do you use? [Day(s) before VUS/VCUG, how many?]
N/A

Amoxicillin: which time schedule do you use? [Day of VUS/VCUG]
N/A

Amoxicillin: which time schedule do you use? [Day(s) after VUS/VCUG, how many?]
N/A

Cefaclor: in what dosage?
Other

Cefaclor: in what dosage? [Other]
3 x 10 mg/kg/d

Cefaclor: for which age group do you use cefaclor?
Ab Geburt

Cefaclor: which time schedule do you use? [Days(s) before VUS/VCUG, how many?]
No

Cefaclor: which time schedule do you use? [Days(s) before VUS/VCUG, how many?][Comment]

Cefaclor: which time schedule do you use? [Day of VUS/VCUG]
Yes

Cefaclor: which time schedule do you use? [Day of VUS/VCUG][Comment]

Cefaclor: which time schedule do you use? [Day(s) after VUS/VCUG, how many?]
Yes

Cefaclor: which time schedule do you use? [Day(s) after VUS/VCUG, how many?][Comment]
2

Amoxicillin/clavulanic acid: in what dosage? (Amoxicillin portion) [prophylaktic (10-20 mg/Kg bodyweight/d)]
N/A

Amoxicillin/clavulanic acid: in what dosage? (Amoxicillin portion) [double prophylactic (20-40 mg/Kg bodyweight/d)]
N/A

Amoxicillin/clavulanic acid: in what dosage? (Amoxicillin portion) [therapeutic (80-100 mg/Kg bodyweight/d)]
N/A

Amoxicillin/clavulanic acid: which time schedule do you use? [Day(s) before VUS/VCUG]
N/A

Amoxicillin/clavulanic acid: which time schedule do you use? [Day of VUS/VCUG]
N/A

Amoxicillin/clavulanic acid: which time schedule do you use? [Day(s) after VUS/VCUG]
N/A

Other: which time schedule do you use? [Day(s) before VUS/VCUG, how many?]
N/A

Other: which time schedule do you use? [Day of VUS/VCUG]
N/A

Other: which time schedule do you use? [Day(s) after VUS/VCUG, how many?]
N/A

Indication for prophylaxis

Which patients receive periinterventional antibiotic prophylaxis? (multiple answers possible) [all]
Yes

Which patients receive periinterventional antibiotic prophylaxis? (multiple answers possible) [patients post urinary tract infection/pyelonephritis/urosepsis]
N/A

Which patients receive periinterventional antibiotic prophylaxis? (multiple answers possible) [patients with urinary tract dilation in ultrasound]
N/A

Which patients receive periinterventional antibiotic prophylaxis? (multiple answers possible) [patients with prenatal urinary tract dilation]
N/A

Which patients receive periinterventional antibiotic prophylaxis? (multiple answers possible) [patients post VUR surgery]
N/A

Which patients receive periinterventional antibiotic prophylaxis? (multiple answers possible) [none]
N/A

Are there situations or clinical findings in the patient where you deviate from your prophylaxis standard (medication, dose, duration, age of the patient)? (multiple answers possible, please specify in answer box) [in case of already known vesicoureteral reflux (VUR)]
Yes

Are there situations or clinical findings in the patient where you deviate from your prophylaxis standard (medication, dose, duration, age of the patient)? (multiple answers possible, please specify in answer box) [in case of already known vesicoureteral reflux (VUR)][Comment]
Anheben der Reinfektionsprohylaxe (Nifurantin) auf therapeutische Dosis für 3 Tage

Are there situations or clinical findings in the patient where you deviate from your prophylaxis standard (medication, dose, duration, age of the patient)? (multiple answers possible, please specify in answer box) [in patients with recurrent urinary tract infections (UTI)]
No

Are there situations or clinical findings in the patient where you deviate from your prophylaxis standard (medication, dose, duration, age of the patient)? (multiple answers possible, please specify in answer box) [in patients with recurrent urinary tract infections (UTI)][Comment]

Are there situations or clinical findings in the patient where you deviate from your prophylaxis standard (medication, dose, duration, age of the patient)? (multiple answers possible, please specify in answer box) [if multidrug-resistant organisms (MDRO) are detected in urine cultures]
No

Are there situations or clinical findings in the patient where you deviate from your prophylaxis standard (medication, dose, duration, age of the patient)? (multiple answers possible, please specify in answer box) [if multidrug-resistant organisms (MDRO) are detected in urine cultures][Comment]

Are there situations or clinical findings in the patient where you deviate from your prophylaxis standard (medication, dose, duration, age of the patient)? (multiple answers possible, please specify in answer box) [in newborns]
No

Are there situations or clinical findings in the patient where you deviate from your prophylaxis standard (medication, dose, duration, age of the patient)? (multiple answers possible, please specify in answer box) [in newborns][Comment]

Are there situations or clinical findings in the patient where you deviate from your prophylaxis standard (medication, dose, duration, age of the patient)? (multiple answers possible, please specify in answer box) [Other]

Are there situations or clinical findings in the patient where you deviate from your prophylaxis standard (medication, dose, duration, age of the patient)? (multiple answers possible, please specify in answer box) [Other comment]

Are there situations in which you use intravenous prophylaxis?
No

Voiding analysis

Are there differences in antibiotic prophylaxis between VUS and VCUG in your department?
No

Is a non-pathological urine strip test required in preparation for VUS/VCUG in your department?
No

Is a non-pathological urine culture required in preparation for VUS/VCUG in your department?
No

Do patients only receive post-procedure prophylaxis if vesicoureteral reflux (VUR) is detected in VUS/VCUG?
No

Do other MUS/MCU findings (e.g. urethral valves) have an influence on post-procedure prophylaxis?
No

End of survey

Thank you for taking the time to answer this survey!

May we contact you personally if we have further questions?
Yes

E-mail address:
m.hagenberg@krankenhaus-halle-saale.de

Survey response 119

Response ID
121

Date submitted
2025-02-03 17:44:44

Last page
5

Start language
de

Seed
463554790

Date started
2025-02-03 17:40:28

Date last action
2025-02-03 17:44:44

Standard Operation Procedure

Who fills out this survey?
Pediatric nephrologist

Who fills out this survey? [Other]

In which type of hospital are you employed?
Community hospital (

In which type of hospital are you employed? [Other]

In which country do you work?
Italien

Does your department have a defined protocol for infection prophylaxis in the context of VUS/VCUG?
Yes

Has this protocol been transformed into a written standard operating procedure (SOP)?
No

Antibiotic Prophylaxis

Are there situations/constellations in which you do not use antibiotic prophylaxis for VUS/VCUG?
Yes

Please specify these situations/constellations: [girl with first uncomplicated UTI without urinary tract dilation in ultrasound]
Yes

Please specify these situations/constellations: [boy with first uncomplicated UTI without urinary tract dilation in ultrasound]
Yes

Please specify these situations/constellations: [in patients with low grade urinary tract dilation (up to 2nd grade) and no UTI in medical history]
Yes

Please specify these situations/constellations: [Other]

If you use antibiotic prophylaxis: which mediaction do you use? (multiple answers and subdivision into age groups possible) [Trimethoprim]
No

If you use antibiotic prophylaxis: which mediaction do you use? (multiple answers and subdivision into age groups possible) [Nitrofurantoin]
No

If you use antibiotic prophylaxis: which mediaction do you use? (multiple answers and subdivision into age groups possible) [Amoxicillin]
No

If you use antibiotic prophylaxis: which mediaction do you use? (multiple answers and subdivision into age groups possible) [Cefaclor]
No

If you use antibiotic prophylaxis: which mediaction do you use? (multiple answers and subdivision into age groups possible) [Amoxicillin/clavulanic acid]
No

If you use antibiotic prophylaxis: which mediaction do you use? (multiple answers and subdivision into age groups possible) [Other]
No

Trimethoprim: which time schedule do you use? [Day(s) before VUS/VCUG, how many?]
N/A

Trimethoprim: which time schedule do you use? [Day of VUS/VCUG]
N/A

Trimethoprim: which time schedule do you use? [Day(s) after VUS/VCUG, how many?]
N/A

Nitrofurantoin: which time schedule do you use? [Day(s) before VUS/VCUG, how many?]
N/A

Nitrofurantoin: which time schedule do you use? [Day of VUS/VCUG]
N/A

Nitrofurantoin: which time schedule do you use? [Day(s) after VUS/VCUG, how many?]
N/A

Amoxicillin: which time schedule do you use? [Day(s) before VUS/VCUG, how many?]
N/A

Amoxicillin: which time schedule do you use? [Day of VUS/VCUG]
N/A

Amoxicillin: which time schedule do you use? [Day(s) after VUS/VCUG, how many?]
N/A

Cefaclor: which time schedule do you use? [Days(s) before VUS/VCUG, how many?]
N/A

Cefaclor: which time schedule do you use? [Day of VUS/VCUG]
N/A

Cefaclor: which time schedule do you use? [Day(s) after VUS/VCUG, how many?]
N/A

Amoxicillin/clavulanic acid: in what dosage? (Amoxicillin portion) [prophylaktic (10-20 mg/Kg bodyweight/d)]
N/A

Amoxicillin/clavulanic acid: in what dosage? (Amoxicillin portion) [double prophylactic (20-40 mg/Kg bodyweight/d)]
N/A

Amoxicillin/clavulanic acid: in what dosage? (Amoxicillin portion) [therapeutic (80-100 mg/Kg bodyweight/d)]
N/A

Amoxicillin/clavulanic acid: which time schedule do you use? [Day(s) before VUS/VCUG]
N/A

Amoxicillin/clavulanic acid: which time schedule do you use? [Day of VUS/VCUG]
N/A

Amoxicillin/clavulanic acid: which time schedule do you use? [Day(s) after VUS/VCUG]
N/A

Other: which time schedule do you use? [Day(s) before VUS/VCUG, how many?]
N/A

Other: which time schedule do you use? [Day of VUS/VCUG]
N/A

Other: which time schedule do you use? [Day(s) after VUS/VCUG, how many?]
N/A

Indication for prophylaxis

Which patients receive periinterventional antibiotic prophylaxis? (multiple answers possible) [all]
No

Which patients receive periinterventional antibiotic prophylaxis? (multiple answers possible) [patients post urinary tract infection/pyelonephritis/urosepsis]
Yes

Which patients receive periinterventional antibiotic prophylaxis? (multiple answers possible) [patients with urinary tract dilation in ultrasound]
Yes

Which patients receive periinterventional antibiotic prophylaxis? (multiple answers possible) [patients with prenatal urinary tract dilation]
No

Which patients receive periinterventional antibiotic prophylaxis? (multiple answers possible) [patients post VUR surgery]
No

Which patients receive periinterventional antibiotic prophylaxis? (multiple answers possible) [none]
No

Which patients receive periinterventional antibiotic prophylaxis? (multiple answers possible) [Other]

patients status post urinary tract infection/pyelonephritis/urosepsis: what is the minimum time between infection and VUS/VCUG? (in days)
3-4 Wochen

In patients with urinary tract dilation in ultrasound: from what degree on? Image from Beetz R, Bokenkamp A, Brandis M et al (2001) Diagnosis of congenital dilatation of the urinary tract. Consensus Group of the Pediatric Nephrology Working Society in cooperation with the Pediatric Urology Working Group of the German Society of Urology and with the Pediatric Urology Working Society in the Germany Society of Pediatric Surgery. Urologe A 40:495–507 (quiz 8–9)
grade 3

Are there situations or clinical findings in the patient where you deviate from your prophylaxis standard (medication, dose, duration, age of the patient)? (multiple answers possible, please specify in answer box) [in case of already known vesicoureteral reflux (VUR)]
No

Are there situations or clinical findings in the patient where you deviate from your prophylaxis standard (medication, dose, duration, age of the patient)? (multiple answers possible, please specify in answer box) [in case of already known vesicoureteral reflux (VUR)][Comment]

Are there situations or clinical findings in the patient where you deviate from your prophylaxis standard (medication, dose, duration, age of the patient)? (multiple answers possible, please specify in answer box) [in patients with recurrent urinary tract infections (UTI)]
No

Are there situations or clinical findings in the patient where you deviate from your prophylaxis standard (medication, dose, duration, age of the patient)? (multiple answers possible, please specify in answer box) [in patients with recurrent urinary tract infections (UTI)][Comment]

Are there situations or clinical findings in the patient where you deviate from your prophylaxis standard (medication, dose, duration, age of the patient)? (multiple answers possible, please specify in answer box) [if multidrug-resistant organisms (MDRO) are detected in urine cultures]
Yes

Are there situations or clinical findings in the patient where you deviate from your prophylaxis standard (medication, dose, duration, age of the patient)? (multiple answers possible, please specify in answer box) [if multidrug-resistant organisms (MDRO) are detected in urine cultures][Comment]

Are there situations or clinical findings in the patient where you deviate from your prophylaxis standard (medication, dose, duration, age of the patient)? (multiple answers possible, please specify in answer box) [in newborns]
No

Are there situations or clinical findings in the patient where you deviate from your prophylaxis standard (medication, dose, duration, age of the patient)? (multiple answers possible, please specify in answer box) [in newborns][Comment]

Are there situations or clinical findings in the patient where you deviate from your prophylaxis standard (medication, dose, duration, age of the patient)? (multiple answers possible, please specify in answer box) [Other]

Are there situations or clinical findings in the patient where you deviate from your prophylaxis standard (medication, dose, duration, age of the patient)? (multiple answers possible, please specify in answer box) [Other comment]

Are there situations in which you use intravenous prophylaxis?
No

Voiding analysis

Are there differences in antibiotic prophylaxis between VUS and VCUG in your department?
No

Is a non-pathological urine strip test required in preparation for VUS/VCUG in your department?
Yes

What is the maximum amount of days accepted for urine strip test to be performed prior to the exam?
1 Woche

Is a non-pathological urine culture required in preparation for VUS/VCUG in your department?
Yes

What is the maximum amount of days accepted for urine culture to be performed prior to the exam?
1 Woche

Do patients only receive post-procedure prophylaxis if vesicoureteral reflux (VUR) is detected in VUS/VCUG?
No

Do other MUS/MCU findings (e.g. urethral valves) have an influence on post-procedure prophylaxis?
No

End of survey

Thank you for taking the time to answer this survey!

May we contact you personally if we have further questions?
Yes

E-mail address:
walburga.cassar@sabes.it

Survey response 120

Response ID
122

Date submitted
2025-02-04 14:06:44

Last page
5

Start language
de

Seed
1639148918

Date started
2025-02-04 13:59:48

Date last action
2025-02-04 14:06:44

Standard Operation Procedure

Who fills out this survey?
Pediatric nephrologist

Who fills out this survey? [Other]

In which type of hospital are you employed?
University Hospital

In which type of hospital are you employed? [Other]

In which country do you work?
Deutschland

Does your department have a defined protocol for infection prophylaxis in the context of VUS/VCUG?
No

Has this protocol been transformed into a written standard operating procedure (SOP)?
N/A

Antibiotic Prophylaxis

Are there situations/constellations in which you do not use antibiotic prophylaxis for VUS/VCUG?
No

Please specify these situations/constellations: [girl with first uncomplicated UTI without urinary tract dilation in ultrasound]
N/A

Please specify these situations/constellations: [boy with first uncomplicated UTI without urinary tract dilation in ultrasound]
N/A

Please specify these situations/constellations: [in patients with low grade urinary tract dilation (up to 2nd grade) and no UTI in medical history]
N/A

If you use antibiotic prophylaxis: which mediaction do you use? (multiple answers and subdivision into age groups possible) [Trimethoprim]
Yes

If you use antibiotic prophylaxis: which mediaction do you use? (multiple answers and subdivision into age groups possible) [Nitrofurantoin]
No

If you use antibiotic prophylaxis: which mediaction do you use? (multiple answers and subdivision into age groups possible) [Amoxicillin]
No

If you use antibiotic prophylaxis: which mediaction do you use? (multiple answers and subdivision into age groups possible) [Cefaclor]
Yes

If you use antibiotic prophylaxis: which mediaction do you use? (multiple answers and subdivision into age groups possible) [Amoxicillin/clavulanic acid]
No

If you use antibiotic prophylaxis: which mediaction do you use? (multiple answers and subdivision into age groups possible) [Other]
No

Trimethoprim: in what dosage?
prophylactic (2 mg/kg bodyweight/d)

Trimethoprim: in what dosage? [Other]

Trimethoprim: for which age group do you use trimethoprim?
alle

Trimethoprim: which time schedule do you use? [Day(s) before VUS/VCUG, how many?]
Yes

Trimethoprim: which time schedule do you use? [Day(s) before VUS/VCUG, how many?][Comment]
nach Ende der PN-Behandlung

Trimethoprim: which time schedule do you use? [Day of VUS/VCUG]
Yes

Trimethoprim: which time schedule do you use? [Day of VUS/VCUG][Comment]

Trimethoprim: which time schedule do you use? [Day(s) after VUS/VCUG, how many?]
Yes

Trimethoprim: which time schedule do you use? [Day(s) after VUS/VCUG, how many?][Comment]
abh. von Befund

Nitrofurantoin: which time schedule do you use? [Day(s) before VUS/VCUG, how many?]
N/A

Nitrofurantoin: which time schedule do you use? [Day of VUS/VCUG]
N/A

Nitrofurantoin: which time schedule do you use? [Day(s) after VUS/VCUG, how many?]
N/A

Amoxicillin: which time schedule do you use? [Day(s) before VUS/VCUG, how many?]
N/A

Amoxicillin: which time schedule do you use? [Day of VUS/VCUG]
N/A

Amoxicillin: which time schedule do you use? [Day(s) after VUS/VCUG, how many?]
N/A

Cefaclor: in what dosage?
double prophylactic (20 mg/kg bodyweight/d)

Cefaclor: in what dosage? [Other]

Cefaclor: for which age group do you use cefaclor?
alle

Cefaclor: which time schedule do you use? [Days(s) before VUS/VCUG, how many?]
Yes

Cefaclor: which time schedule do you use? [Days(s) before VUS/VCUG, how many?][Comment]
1

Cefaclor: which time schedule do you use? [Day of VUS/VCUG]
Yes

Cefaclor: which time schedule do you use? [Day of VUS/VCUG][Comment]

Cefaclor: which time schedule do you use? [Day(s) after VUS/VCUG, how many?]
Yes

Cefaclor: which time schedule do you use? [Day(s) after VUS/VCUG, how many?][Comment]
1

Amoxicillin/clavulanic acid: in what dosage? (Amoxicillin portion) [prophylaktic (10-20 mg/Kg bodyweight/d)]
N/A

Amoxicillin/clavulanic acid: in what dosage? (Amoxicillin portion) [double prophylactic (20-40 mg/Kg bodyweight/d)]
N/A

Amoxicillin/clavulanic acid: in what dosage? (Amoxicillin portion) [therapeutic (80-100 mg/Kg bodyweight/d)]
N/A

Amoxicillin/clavulanic acid: which time schedule do you use? [Day(s) before VUS/VCUG]
N/A

Amoxicillin/clavulanic acid: which time schedule do you use? [Day of VUS/VCUG]
N/A

Amoxicillin/clavulanic acid: which time schedule do you use? [Day(s) after VUS/VCUG]
N/A

Other: which time schedule do you use? [Day(s) before VUS/VCUG, how many?]
N/A

Other: which time schedule do you use? [Day of VUS/VCUG]
N/A

Other: which time schedule do you use? [Day(s) after VUS/VCUG, how many?]
N/A

Indication for prophylaxis

Which patients receive periinterventional antibiotic prophylaxis? (multiple answers possible) [all]
Yes

Which patients receive periinterventional antibiotic prophylaxis? (multiple answers possible) [patients post urinary tract infection/pyelonephritis/urosepsis]
N/A

Which patients receive periinterventional antibiotic prophylaxis? (multiple answers possible) [patients with urinary tract dilation in ultrasound]
N/A

Which patients receive periinterventional antibiotic prophylaxis? (multiple answers possible) [patients with prenatal urinary tract dilation]
N/A

Which patients receive periinterventional antibiotic prophylaxis? (multiple answers possible) [patients post VUR surgery]
N/A

Which patients receive periinterventional antibiotic prophylaxis? (multiple answers possible) [none]
N/A

Are there situations or clinical findings in the patient where you deviate from your prophylaxis standard (medication, dose, duration, age of the patient)? (multiple answers possible, please specify in answer box) [in case of already known vesicoureteral reflux (VUR)]
No

Are there situations or clinical findings in the patient where you deviate from your prophylaxis standard (medication, dose, duration, age of the patient)? (multiple answers possible, please specify in answer box) [in case of already known vesicoureteral reflux (VUR)][Comment]

Are there situations or clinical findings in the patient where you deviate from your prophylaxis standard (medication, dose, duration, age of the patient)? (multiple answers possible, please specify in answer box) [in patients with recurrent urinary tract infections (UTI)]
No

Are there situations or clinical findings in the patient where you deviate from your prophylaxis standard (medication, dose, duration, age of the patient)? (multiple answers possible, please specify in answer box) [in patients with recurrent urinary tract infections (UTI)][Comment]

Are there situations or clinical findings in the patient where you deviate from your prophylaxis standard (medication, dose, duration, age of the patient)? (multiple answers possible, please specify in answer box) [if multidrug-resistant organisms (MDRO) are detected in urine cultures]
Yes

Are there situations or clinical findings in the patient where you deviate from your prophylaxis standard (medication, dose, duration, age of the patient)? (multiple answers possible, please specify in answer box) [if multidrug-resistant organisms (MDRO) are detected in urine cultures][Comment]
Anpassung

Are there situations or clinical findings in the patient where you deviate from your prophylaxis standard (medication, dose, duration, age of the patient)? (multiple answers possible, please specify in answer box) [in newborns]
No

Are there situations or clinical findings in the patient where you deviate from your prophylaxis standard (medication, dose, duration, age of the patient)? (multiple answers possible, please specify in answer box) [in newborns][Comment]

Are there situations or clinical findings in the patient where you deviate from your prophylaxis standard (medication, dose, duration, age of the patient)? (multiple answers possible, please specify in answer box) [Other]

Are there situations or clinical findings in the patient where you deviate from your prophylaxis standard (medication, dose, duration, age of the patient)? (multiple answers possible, please specify in answer box) [Other comment]

Are there situations in which you use intravenous prophylaxis?
No

Voiding analysis

Are there differences in antibiotic prophylaxis between VUS and VCUG in your department?
No

Is a non-pathological urine strip test required in preparation for VUS/VCUG in your department?
No

Is a non-pathological urine culture required in preparation for VUS/VCUG in your department?
No

Do patients only receive post-procedure prophylaxis if vesicoureteral reflux (VUR) is detected in VUS/VCUG?
No

Do other MUS/MCU findings (e.g. urethral valves) have an influence on post-procedure prophylaxis?
No

End of survey

Thank you for taking the time to answer this survey!

May we contact you personally if we have further questions?
No

Survey response 121

Response ID
123

Date submitted
2025-02-05 19:06:20

Last page
5

Start language
de

Seed
576362656

Date started
2025-02-05 18:54:36

Date last action
2025-02-05 19:06:20

Standard Operation Procedure

Who fills out this survey?
Pediatrician

Who fills out this survey? [Other]

In which type of hospital are you employed?
University Hospital

In which type of hospital are you employed? [Other]

In which country do you work?
Deutschland

Does your department have a defined protocol for infection prophylaxis in the context of VUS/VCUG?
No

Has this protocol been transformed into a written standard operating procedure (SOP)?
N/A

Antibiotic Prophylaxis

Are there situations/constellations in which you do not use antibiotic prophylaxis for VUS/VCUG?
Yes

Please specify these situations/constellations: [girl with first uncomplicated UTI without urinary tract dilation in ultrasound]
Yes

Please specify these situations/constellations: [boy with first uncomplicated UTI without urinary tract dilation in ultrasound]
No

Please specify these situations/constellations: [in patients with low grade urinary tract dilation (up to 2nd grade) and no UTI in medical history]
No

Please specify these situations/constellations: [Other]

If you use antibiotic prophylaxis: which mediaction do you use? (multiple answers and subdivision into age groups possible) [Trimethoprim]
Yes

If you use antibiotic prophylaxis: which mediaction do you use? (multiple answers and subdivision into age groups possible) [Nitrofurantoin]
No

If you use antibiotic prophylaxis: which mediaction do you use? (multiple answers and subdivision into age groups possible) [Amoxicillin]
No

If you use antibiotic prophylaxis: which mediaction do you use? (multiple answers and subdivision into age groups possible) [Cefaclor]
Yes

If you use antibiotic prophylaxis: which mediaction do you use? (multiple answers and subdivision into age groups possible) [Amoxicillin/clavulanic acid]
No

If you use antibiotic prophylaxis: which mediaction do you use? (multiple answers and subdivision into age groups possible) [Other]
No

Trimethoprim: in what dosage?
prophylactic (2 mg/kg bodyweight/d)

Trimethoprim: in what dosage? [Other]

Trimethoprim: for which age group do you use trimethoprim?
>8 Wochen, männliches Geschlecht

Trimethoprim: which time schedule do you use? [Day(s) before VUS/VCUG, how many?]
No

Trimethoprim: which time schedule do you use? [Day(s) before VUS/VCUG, how many?][Comment]

Trimethoprim: which time schedule do you use? [Day of VUS/VCUG]
Yes

Trimethoprim: which time schedule do you use? [Day of VUS/VCUG][Comment]

Trimethoprim: which time schedule do you use? [Day(s) after VUS/VCUG, how many?]
No

Trimethoprim: which time schedule do you use? [Day(s) after VUS/VCUG, how many?][Comment]

Nitrofurantoin: which time schedule do you use? [Day(s) before VUS/VCUG, how many?]
N/A

Nitrofurantoin: which time schedule do you use? [Day of VUS/VCUG]
N/A

Nitrofurantoin: which time schedule do you use? [Day(s) after VUS/VCUG, how many?]
N/A

Amoxicillin: which time schedule do you use? [Day(s) before VUS/VCUG, how many?]
N/A

Amoxicillin: which time schedule do you use? [Day of VUS/VCUG]
N/A

Amoxicillin: which time schedule do you use? [Day(s) after VUS/VCUG, how many?]
N/A

Cefaclor: in what dosage?
double prophylactic (20 mg/kg bodyweight/d)

Cefaclor: in what dosage? [Other]

Cefaclor: for which age group do you use cefaclor?
bis 3 Monate (ab 3 Mon. Nitrofurantoin.....

Cefaclor: which time schedule do you use? [Days(s) before VUS/VCUG, how many?]
No

Cefaclor: which time schedule do you use? [Days(s) before VUS/VCUG, how many?][Comment]

Cefaclor: which time schedule do you use? [Day of VUS/VCUG]
Yes

Cefaclor: which time schedule do you use? [Day of VUS/VCUG][Comment]

Cefaclor: which time schedule do you use? [Day(s) after VUS/VCUG, how many?]
No

Cefaclor: which time schedule do you use? [Day(s) after VUS/VCUG, how many?][Comment]

Amoxicillin/clavulanic acid: in what dosage? (Amoxicillin portion) [prophylaktic (10-20 mg/Kg bodyweight/d)]
N/A

Amoxicillin/clavulanic acid: in what dosage? (Amoxicillin portion) [double prophylactic (20-40 mg/Kg bodyweight/d)]
N/A

Amoxicillin/clavulanic acid: in what dosage? (Amoxicillin portion) [therapeutic (80-100 mg/Kg bodyweight/d)]
N/A

Amoxicillin/clavulanic acid: which time schedule do you use? [Day(s) before VUS/VCUG]
N/A

Amoxicillin/clavulanic acid: which time schedule do you use? [Day of VUS/VCUG]
N/A

Amoxicillin/clavulanic acid: which time schedule do you use? [Day(s) after VUS/VCUG]
N/A

Other: which time schedule do you use? [Day(s) before VUS/VCUG, how many?]
N/A

Other: which time schedule do you use? [Day of VUS/VCUG]
N/A

Other: which time schedule do you use? [Day(s) after VUS/VCUG, how many?]
N/A

Indication for prophylaxis

Which patients receive periinterventional antibiotic prophylaxis? (multiple answers possible) [all]
No

Which patients receive periinterventional antibiotic prophylaxis? (multiple answers possible) [patients post urinary tract infection/pyelonephritis/urosepsis]
No

Which patients receive periinterventional antibiotic prophylaxis? (multiple answers possible) [patients with urinary tract dilation in ultrasound]
No

Which patients receive periinterventional antibiotic prophylaxis? (multiple answers possible) [patients with prenatal urinary tract dilation]
No

Which patients receive periinterventional antibiotic prophylaxis? (multiple answers possible) [patients post VUR surgery]
No

Which patients receive periinterventional antibiotic prophylaxis? (multiple answers possible) [none]
No

Which patients receive periinterventional antibiotic prophylaxis? (multiple answers possible) [Other]
bei nachgewiesenen VUR

Are there situations or clinical findings in the patient where you deviate from your prophylaxis standard (medication, dose, duration, age of the patient)? (multiple answers possible, please specify in answer box) [in case of already known vesicoureteral reflux (VUR)]
No

Are there situations or clinical findings in the patient where you deviate from your prophylaxis standard (medication, dose, duration, age of the patient)? (multiple answers possible, please specify in answer box) [in case of already known vesicoureteral reflux (VUR)][Comment]

Are there situations or clinical findings in the patient where you deviate from your prophylaxis standard (medication, dose, duration, age of the patient)? (multiple answers possible, please specify in answer box) [in patients with recurrent urinary tract infections (UTI)]
Yes

Are there situations or clinical findings in the patient where you deviate from your prophylaxis standard (medication, dose, duration, age of the patient)? (multiple answers possible, please specify in answer box) [in patients with recurrent urinary tract infections (UTI)][Comment]
MCU Ind?, bis dahin Prophylaxe, mit Dg. VUR am Tag der Untersuchung 2x proph. Dosis

Are there situations or clinical findings in the patient where you deviate from your prophylaxis standard (medication, dose, duration, age of the patient)? (multiple answers possible, please specify in answer box) [if multidrug-resistant organisms (MDRO) are detected in urine cultures]
Yes

Are there situations or clinical findings in the patient where you deviate from your prophylaxis standard (medication, dose, duration, age of the patient)? (multiple answers possible, please specify in answer box) [if multidrug-resistant organisms (MDRO) are detected in urine cultures][Comment]
prophylaxe wird Resistogramm angepasst

Are there situations or clinical findings in the patient where you deviate from your prophylaxis standard (medication, dose, duration, age of the patient)? (multiple answers possible, please specify in answer box) [in newborns]
Yes

Are there situations or clinical findings in the patient where you deviate from your prophylaxis standard (medication, dose, duration, age of the patient)? (multiple answers possible, please specify in answer box) [in newborns][Comment]
cefaclor zugelassen

Are there situations or clinical findings in the patient where you deviate from your prophylaxis standard (medication, dose, duration, age of the patient)? (multiple answers possible, please specify in answer box) [Other]

Are there situations or clinical findings in the patient where you deviate from your prophylaxis standard (medication, dose, duration, age of the patient)? (multiple answers possible, please specify in answer box) [Other comment]

Are there situations in which you use intravenous prophylaxis?
Yes

In which situations?
Wenn zusätzlich ein kritischer Herzfehler (z.B. Ebstein- Anomalie) vorliegt

Voiding analysis

Are there differences in antibiotic prophylaxis between VUS and VCUG in your department?
No

Is a non-pathological urine strip test required in preparation for VUS/VCUG in your department?
Yes

What is the maximum amount of days accepted for urine strip test to be performed prior to the exam?
Am Untersuchungstag mit Anlage Katheter

Is a non-pathological urine culture required in preparation for VUS/VCUG in your department?
No

Do patients only receive post-procedure prophylaxis if vesicoureteral reflux (VUR) is detected in VUS/VCUG?
No

Do other MUS/MCU findings (e.g. urethral valves) have an influence on post-procedure prophylaxis?
No

End of survey

Thank you for taking the time to answer this survey!

May we contact you personally if we have further questions?
Yes

E-mail address:
ute.derichs@unimedizin-mainz.de

Survey response 122

Response ID
124

Last page
0

Start language
de

Seed
1666688150

Date started
2025-02-05 19:49:33

Date last action
2025-02-05 19:49:33

Standard Operation Procedure

Does your department have a defined protocol for infection prophylaxis in the context of VUS/VCUG?
N/A

Has this protocol been transformed into a written standard operating procedure (SOP)?
N/A

Antibiotic Prophylaxis

Are there situations/constellations in which you do not use antibiotic prophylaxis for VUS/VCUG?
N/A

Please specify these situations/constellations: [girl with first uncomplicated UTI without urinary tract dilation in ultrasound]
N/A

Please specify these situations/constellations: [boy with first uncomplicated UTI without urinary tract dilation in ultrasound]
N/A

Please specify these situations/constellations: [in patients with low grade urinary tract dilation (up to 2nd grade) and no UTI in medical history]
N/A

If you use antibiotic prophylaxis: which mediaction do you use? (multiple answers and subdivision into age groups possible) [Trimethoprim]
N/A

If you use antibiotic prophylaxis: which mediaction do you use? (multiple answers and subdivision into age groups possible) [Nitrofurantoin]
N/A

If you use antibiotic prophylaxis: which mediaction do you use? (multiple answers and subdivision into age groups possible) [Amoxicillin]
N/A

If you use antibiotic prophylaxis: which mediaction do you use? (multiple answers and subdivision into age groups possible) [Cefaclor]
N/A

If you use antibiotic prophylaxis: which mediaction do you use? (multiple answers and subdivision into age groups possible) [Amoxicillin/clavulanic acid]
N/A

If you use antibiotic prophylaxis: which mediaction do you use? (multiple answers and subdivision into age groups possible) [Other]
N/A

Trimethoprim: which time schedule do you use? [Day(s) before VUS/VCUG, how many?]
N/A

Trimethoprim: which time schedule do you use? [Day of VUS/VCUG]
N/A

Trimethoprim: which time schedule do you use? [Day(s) after VUS/VCUG, how many?]
N/A

Nitrofurantoin: which time schedule do you use? [Day(s) before VUS/VCUG, how many?]
N/A

Nitrofurantoin: which time schedule do you use? [Day of VUS/VCUG]
N/A

Nitrofurantoin: which time schedule do you use? [Day(s) after VUS/VCUG, how many?]
N/A

Amoxicillin: which time schedule do you use? [Day(s) before VUS/VCUG, how many?]
N/A

Amoxicillin: which time schedule do you use? [Day of VUS/VCUG]
N/A

Amoxicillin: which time schedule do you use? [Day(s) after VUS/VCUG, how many?]
N/A

Cefaclor: which time schedule do you use? [Days(s) before VUS/VCUG, how many?]
N/A

Cefaclor: which time schedule do you use? [Day of VUS/VCUG]
N/A

Cefaclor: which time schedule do you use? [Day(s) after VUS/VCUG, how many?]
N/A

Amoxicillin/clavulanic acid: in what dosage? (Amoxicillin portion) [prophylaktic (10-20 mg/Kg bodyweight/d)]
N/A

Amoxicillin/clavulanic acid: in what dosage? (Amoxicillin portion) [double prophylactic (20-40 mg/Kg bodyweight/d)]
N/A

Amoxicillin/clavulanic acid: in what dosage? (Amoxicillin portion) [therapeutic (80-100 mg/Kg bodyweight/d)]
N/A

Amoxicillin/clavulanic acid: which time schedule do you use? [Day(s) before VUS/VCUG]
N/A

Amoxicillin/clavulanic acid: which time schedule do you use? [Day of VUS/VCUG]
N/A

Amoxicillin/clavulanic acid: which time schedule do you use? [Day(s) after VUS/VCUG]
N/A

Other: which time schedule do you use? [Day(s) before VUS/VCUG, how many?]
N/A

Other: which time schedule do you use? [Day of VUS/VCUG]
N/A

Other: which time schedule do you use? [Day(s) after VUS/VCUG, how many?]
N/A

Indication for prophylaxis

Which patients receive periinterventional antibiotic prophylaxis? (multiple answers possible) [all]
N/A

Which patients receive periinterventional antibiotic prophylaxis? (multiple answers possible) [patients post urinary tract infection/pyelonephritis/urosepsis]
N/A

Which patients receive periinterventional antibiotic prophylaxis? (multiple answers possible) [patients with urinary tract dilation in ultrasound]
N/A

Which patients receive periinterventional antibiotic prophylaxis? (multiple answers possible) [patients with prenatal urinary tract dilation]
N/A

Which patients receive periinterventional antibiotic prophylaxis? (multiple answers possible) [patients post VUR surgery]
N/A

Which patients receive periinterventional antibiotic prophylaxis? (multiple answers possible) [none]
N/A

Are there situations or clinical findings in the patient where you deviate from your prophylaxis standard (medication, dose, duration, age of the patient)? (multiple answers possible, please specify in answer box) [in case of already known vesicoureteral reflux (VUR)]
N/A

Are there situations or clinical findings in the patient where you deviate from your prophylaxis standard (medication, dose, duration, age of the patient)? (multiple answers possible, please specify in answer box) [in patients with recurrent urinary tract infections (UTI)]
N/A

Are there situations or clinical findings in the patient where you deviate from your prophylaxis standard (medication, dose, duration, age of the patient)? (multiple answers possible, please specify in answer box) [if multidrug-resistant organisms (MDRO) are detected in urine cultures]
N/A

Are there situations or clinical findings in the patient where you deviate from your prophylaxis standard (medication, dose, duration, age of the patient)? (multiple answers possible, please specify in answer box) [in newborns]
N/A

Are there situations in which you use intravenous prophylaxis?
N/A

Voiding analysis

Are there differences in antibiotic prophylaxis between VUS and VCUG in your department?
N/A

Is a non-pathological urine strip test required in preparation for VUS/VCUG in your department?
N/A

Is a non-pathological urine culture required in preparation for VUS/VCUG in your department?
N/A

Do patients only receive post-procedure prophylaxis if vesicoureteral reflux (VUR) is detected in VUS/VCUG?
N/A

Do other MUS/MCU findings (e.g. urethral valves) have an influence on post-procedure prophylaxis?
N/A

End of survey

May we contact you personally if we have further questions?
N/A

Survey response 123

Response ID
125

Date submitted
2025-02-07 08:40:30

Last page
5

Start language
de

Seed
1896473352

Date started
2025-02-07 08:15:07

Date last action
2025-02-07 08:40:30

Standard Operation Procedure

Who fills out this survey?
Pediatric Radiologist

Who fills out this survey? [Other]

In which type of hospital are you employed?
Community hospital (

In which type of hospital are you employed? [Other]

In which country do you work?
Deutschland

Does your department have a defined protocol for infection prophylaxis in the context of VUS/VCUG?
Yes

Has this protocol been transformed into a written standard operating procedure (SOP)?
Yes

Which department is the author of the SOP for infection prophylaxis in VUS/VCUG?
Pediatric radiology

Which department is the author of the SOP for infection prophylaxis in VUS/VCUG? [Other]

Antibiotic Prophylaxis

Are there situations/constellations in which you do not use antibiotic prophylaxis for VUS/VCUG?
Yes

Please specify these situations/constellations: [girl with first uncomplicated UTI without urinary tract dilation in ultrasound]
No

Please specify these situations/constellations: [boy with first uncomplicated UTI without urinary tract dilation in ultrasound]
No

Please specify these situations/constellations: [in patients with low grade urinary tract dilation (up to 2nd grade) and no UTI in medical history]
No

Please specify these situations/constellations: [Other]
wenn Eltern ablehen

If you use antibiotic prophylaxis: which mediaction do you use? (multiple answers and subdivision into age groups possible) [Trimethoprim]
No

If you use antibiotic prophylaxis: which mediaction do you use? (multiple answers and subdivision into age groups possible) [Nitrofurantoin]
No

If you use antibiotic prophylaxis: which mediaction do you use? (multiple answers and subdivision into age groups possible) [Amoxicillin]
No

If you use antibiotic prophylaxis: which mediaction do you use? (multiple answers and subdivision into age groups possible) [Cefaclor]
No

If you use antibiotic prophylaxis: which mediaction do you use? (multiple answers and subdivision into age groups possible) [Amoxicillin/clavulanic acid]
No

If you use antibiotic prophylaxis: which mediaction do you use? (multiple answers and subdivision into age groups possible) [Other]
Yes

Trimethoprim: which time schedule do you use? [Day(s) before VUS/VCUG, how many?]
N/A

Trimethoprim: which time schedule do you use? [Day of VUS/VCUG]
N/A

Trimethoprim: which time schedule do you use? [Day(s) after VUS/VCUG, how many?]
N/A

Nitrofurantoin: which time schedule do you use? [Day(s) before VUS/VCUG, how many?]
N/A

Nitrofurantoin: which time schedule do you use? [Day of VUS/VCUG]
N/A

Nitrofurantoin: which time schedule do you use? [Day(s) after VUS/VCUG, how many?]
N/A

Amoxicillin: which time schedule do you use? [Day(s) before VUS/VCUG, how many?]
N/A

Amoxicillin: which time schedule do you use? [Day of VUS/VCUG]
N/A

Amoxicillin: which time schedule do you use? [Day(s) after VUS/VCUG, how many?]
N/A

Cefaclor: which time schedule do you use? [Days(s) before VUS/VCUG, how many?]
N/A

Cefaclor: which time schedule do you use? [Day of VUS/VCUG]
N/A

Cefaclor: which time schedule do you use? [Day(s) after VUS/VCUG, how many?]
N/A

Amoxicillin/clavulanic acid: in what dosage? (Amoxicillin portion) [prophylaktic (10-20 mg/Kg bodyweight/d)]
N/A

Amoxicillin/clavulanic acid: in what dosage? (Amoxicillin portion) [double prophylactic (20-40 mg/Kg bodyweight/d)]
N/A

Amoxicillin/clavulanic acid: in what dosage? (Amoxicillin portion) [therapeutic (80-100 mg/Kg bodyweight/d)]
N/A

Amoxicillin/clavulanic acid: which time schedule do you use? [Day(s) before VUS/VCUG]
N/A

Amoxicillin/clavulanic acid: which time schedule do you use? [Day of VUS/VCUG]
N/A

Amoxicillin/clavulanic acid: which time schedule do you use? [Day(s) after VUS/VCUG]
N/A

Other: which medication and in what dosage?
Cefpodoxim 3 mal 4 mg/KG

Other: for which age group do you use this medication?
alle

Other: which time schedule do you use? [Day(s) before VUS/VCUG, how many?]
No

Other: which time schedule do you use? [Day(s) before VUS/VCUG, how many?][Comment]

Other: which time schedule do you use? [Day of VUS/VCUG]
Yes

Other: which time schedule do you use? [Day of VUS/VCUG][Comment]

Other: which time schedule do you use? [Day(s) after VUS/VCUG, how many?]
No

Other: which time schedule do you use? [Day(s) after VUS/VCUG, how many?][Comment]

Indication for prophylaxis

Which patients receive periinterventional antibiotic prophylaxis? (multiple answers possible) [all]
Yes

Which patients receive periinterventional antibiotic prophylaxis? (multiple answers possible) [patients post urinary tract infection/pyelonephritis/urosepsis]
N/A

Which patients receive periinterventional antibiotic prophylaxis? (multiple answers possible) [patients with urinary tract dilation in ultrasound]
N/A

Which patients receive periinterventional antibiotic prophylaxis? (multiple answers possible) [patients with prenatal urinary tract dilation]
N/A

Which patients receive periinterventional antibiotic prophylaxis? (multiple answers possible) [patients post VUR surgery]
N/A

Which patients receive periinterventional antibiotic prophylaxis? (multiple answers possible) [none]
N/A

Are there situations or clinical findings in the patient where you deviate from your prophylaxis standard (medication, dose, duration, age of the patient)? (multiple answers possible, please specify in answer box) [in case of already known vesicoureteral reflux (VUR)]
No

Are there situations or clinical findings in the patient where you deviate from your prophylaxis standard (medication, dose, duration, age of the patient)? (multiple answers possible, please specify in answer box) [in case of already known vesicoureteral reflux (VUR)][Comment]

Are there situations or clinical findings in the patient where you deviate from your prophylaxis standard (medication, dose, duration, age of the patient)? (multiple answers possible, please specify in answer box) [in patients with recurrent urinary tract infections (UTI)]
No

Are there situations or clinical findings in the patient where you deviate from your prophylaxis standard (medication, dose, duration, age of the patient)? (multiple answers possible, please specify in answer box) [in patients with recurrent urinary tract infections (UTI)][Comment]

Are there situations or clinical findings in the patient where you deviate from your prophylaxis standard (medication, dose, duration, age of the patient)? (multiple answers possible, please specify in answer box) [if multidrug-resistant organisms (MDRO) are detected in urine cultures]
Yes

Are there situations or clinical findings in the patient where you deviate from your prophylaxis standard (medication, dose, duration, age of the patient)? (multiple answers possible, please specify in answer box) [if multidrug-resistant organisms (MDRO) are detected in urine cultures][Comment]
Anpassung nach Resistogramm

Are there situations or clinical findings in the patient where you deviate from your prophylaxis standard (medication, dose, duration, age of the patient)? (multiple answers possible, please specify in answer box) [in newborns]
No

Are there situations or clinical findings in the patient where you deviate from your prophylaxis standard (medication, dose, duration, age of the patient)? (multiple answers possible, please specify in answer box) [in newborns][Comment]

Are there situations or clinical findings in the patient where you deviate from your prophylaxis standard (medication, dose, duration, age of the patient)? (multiple answers possible, please specify in answer box) [Other]

Are there situations or clinical findings in the patient where you deviate from your prophylaxis standard (medication, dose, duration, age of the patient)? (multiple answers possible, please specify in answer box) [Other comment]

Are there situations in which you use intravenous prophylaxis?
No

Voiding analysis

Are there differences in antibiotic prophylaxis between VUS and VCUG in your department?
No

Is a non-pathological urine strip test required in preparation for VUS/VCUG in your department?
No

Is a non-pathological urine culture required in preparation for VUS/VCUG in your department?
No

Do patients only receive post-procedure prophylaxis if vesicoureteral reflux (VUR) is detected in VUS/VCUG?
No

Do other MUS/MCU findings (e.g. urethral valves) have an influence on post-procedure prophylaxis?
No

End of survey

Thank you for taking the time to answer this survey!

May we contact you personally if we have further questions?
Yes

E-mail address:
thhaufe@t-online.de

Survey response 124

Response ID
126

Date submitted
2025-02-13 10:00:24

Last page
5

Start language
de

Seed
585376511

Date started
2025-02-13 09:53:28

Date last action
2025-02-13 10:00:24

Standard Operation Procedure

Who fills out this survey?
Other

Who fills out this survey? [Other]
Kinderurologe

In which type of hospital are you employed?
University Hospital

In which type of hospital are you employed? [Other]

In which country do you work?
Deutschland

Does your department have a defined protocol for infection prophylaxis in the context of VUS/VCUG?
No

Has this protocol been transformed into a written standard operating procedure (SOP)?
N/A

Antibiotic Prophylaxis

Are there situations/constellations in which you do not use antibiotic prophylaxis for VUS/VCUG?
No

Please specify these situations/constellations: [girl with first uncomplicated UTI without urinary tract dilation in ultrasound]
N/A

Please specify these situations/constellations: [boy with first uncomplicated UTI without urinary tract dilation in ultrasound]
N/A

Please specify these situations/constellations: [in patients with low grade urinary tract dilation (up to 2nd grade) and no UTI in medical history]
N/A

If you use antibiotic prophylaxis: which mediaction do you use? (multiple answers and subdivision into age groups possible) [Trimethoprim]
Yes

If you use antibiotic prophylaxis: which mediaction do you use? (multiple answers and subdivision into age groups possible) [Nitrofurantoin]
No

If you use antibiotic prophylaxis: which mediaction do you use? (multiple answers and subdivision into age groups possible) [Amoxicillin]
No

If you use antibiotic prophylaxis: which mediaction do you use? (multiple answers and subdivision into age groups possible) [Cefaclor]
No

If you use antibiotic prophylaxis: which mediaction do you use? (multiple answers and subdivision into age groups possible) [Amoxicillin/clavulanic acid]
No

If you use antibiotic prophylaxis: which mediaction do you use? (multiple answers and subdivision into age groups possible) [Other]
No

Trimethoprim: in what dosage?
prophylactic (2 mg/kg bodyweight/d)

Trimethoprim: in what dosage? [Other]

Trimethoprim: for which age group do you use trimethoprim?
ab 6 Wochen

Trimethoprim: which time schedule do you use? [Day(s) before VUS/VCUG, how many?]
Yes

Trimethoprim: which time schedule do you use? [Day(s) before VUS/VCUG, how many?][Comment]
Dauerprophylaxe

Trimethoprim: which time schedule do you use? [Day of VUS/VCUG]
No

Trimethoprim: which time schedule do you use? [Day of VUS/VCUG][Comment]

Trimethoprim: which time schedule do you use? [Day(s) after VUS/VCUG, how many?]
Yes

Trimethoprim: which time schedule do you use? [Day(s) after VUS/VCUG, how many?][Comment]
Dauerprophylaxe

Nitrofurantoin: which time schedule do you use? [Day(s) before VUS/VCUG, how many?]
N/A

Nitrofurantoin: which time schedule do you use? [Day of VUS/VCUG]
N/A

Nitrofurantoin: which time schedule do you use? [Day(s) after VUS/VCUG, how many?]
N/A

Amoxicillin: which time schedule do you use? [Day(s) before VUS/VCUG, how many?]
N/A

Amoxicillin: which time schedule do you use? [Day of VUS/VCUG]
N/A

Amoxicillin: which time schedule do you use? [Day(s) after VUS/VCUG, how many?]
N/A

Cefaclor: which time schedule do you use? [Days(s) before VUS/VCUG, how many?]
N/A

Cefaclor: which time schedule do you use? [Day of VUS/VCUG]
N/A

Cefaclor: which time schedule do you use? [Day(s) after VUS/VCUG, how many?]
N/A

Amoxicillin/clavulanic acid: in what dosage? (Amoxicillin portion) [prophylaktic (10-20 mg/Kg bodyweight/d)]
N/A

Amoxicillin/clavulanic acid: in what dosage? (Amoxicillin portion) [double prophylactic (20-40 mg/Kg bodyweight/d)]
N/A

Amoxicillin/clavulanic acid: in what dosage? (Amoxicillin portion) [therapeutic (80-100 mg/Kg bodyweight/d)]
N/A

Amoxicillin/clavulanic acid: which time schedule do you use? [Day(s) before VUS/VCUG]
N/A

Amoxicillin/clavulanic acid: which time schedule do you use? [Day of VUS/VCUG]
N/A

Amoxicillin/clavulanic acid: which time schedule do you use? [Day(s) after VUS/VCUG]
N/A

Other: which time schedule do you use? [Day(s) before VUS/VCUG, how many?]
N/A

Other: which time schedule do you use? [Day of VUS/VCUG]
N/A

Other: which time schedule do you use? [Day(s) after VUS/VCUG, how many?]
N/A

Indication for prophylaxis

Which patients receive periinterventional antibiotic prophylaxis? (multiple answers possible) [all]
No

Which patients receive periinterventional antibiotic prophylaxis? (multiple answers possible) [patients post urinary tract infection/pyelonephritis/urosepsis]
Yes

Which patients receive periinterventional antibiotic prophylaxis? (multiple answers possible) [patients with urinary tract dilation in ultrasound]
No

Which patients receive periinterventional antibiotic prophylaxis? (multiple answers possible) [patients with prenatal urinary tract dilation]
No

Which patients receive periinterventional antibiotic prophylaxis? (multiple answers possible) [patients post VUR surgery]
No

Which patients receive periinterventional antibiotic prophylaxis? (multiple answers possible) [none]
No

Which patients receive periinterventional antibiotic prophylaxis? (multiple answers possible) [Other]
bei klinischem Verdacht

patients status post urinary tract infection/pyelonephritis/urosepsis: what is the minimum time between infection and VUS/VCUG? (in days)
14 tage

Are there situations or clinical findings in the patient where you deviate from your prophylaxis standard (medication, dose, duration, age of the patient)? (multiple answers possible, please specify in answer box) [in case of already known vesicoureteral reflux (VUR)]
No

Are there situations or clinical findings in the patient where you deviate from your prophylaxis standard (medication, dose, duration, age of the patient)? (multiple answers possible, please specify in answer box) [in case of already known vesicoureteral reflux (VUR)][Comment]

Are there situations or clinical findings in the patient where you deviate from your prophylaxis standard (medication, dose, duration, age of the patient)? (multiple answers possible, please specify in answer box) [in patients with recurrent urinary tract infections (UTI)]
No

Are there situations or clinical findings in the patient where you deviate from your prophylaxis standard (medication, dose, duration, age of the patient)? (multiple answers possible, please specify in answer box) [in patients with recurrent urinary tract infections (UTI)][Comment]

Are there situations or clinical findings in the patient where you deviate from your prophylaxis standard (medication, dose, duration, age of the patient)? (multiple answers possible, please specify in answer box) [if multidrug-resistant organisms (MDRO) are detected in urine cultures]
Yes

Are there situations or clinical findings in the patient where you deviate from your prophylaxis standard (medication, dose, duration, age of the patient)? (multiple answers possible, please specify in answer box) [if multidrug-resistant organisms (MDRO) are detected in urine cultures][Comment]

Are there situations or clinical findings in the patient where you deviate from your prophylaxis standard (medication, dose, duration, age of the patient)? (multiple answers possible, please specify in answer box) [in newborns]
Yes

Are there situations or clinical findings in the patient where you deviate from your prophylaxis standard (medication, dose, duration, age of the patient)? (multiple answers possible, please specify in answer box) [in newborns][Comment]

Are there situations or clinical findings in the patient where you deviate from your prophylaxis standard (medication, dose, duration, age of the patient)? (multiple answers possible, please specify in answer box) [Other]

Are there situations or clinical findings in the patient where you deviate from your prophylaxis standard (medication, dose, duration, age of the patient)? (multiple answers possible, please specify in answer box) [Other comment]

Are there situations in which you use intravenous prophylaxis?
No

Voiding analysis

Are there differences in antibiotic prophylaxis between VUS and VCUG in your department?
No

Is a non-pathological urine strip test required in preparation for VUS/VCUG in your department?
No

Is a non-pathological urine culture required in preparation for VUS/VCUG in your department?
No

Do patients only receive post-procedure prophylaxis if vesicoureteral reflux (VUR) is detected in VUS/VCUG?
No

Do other MUS/MCU findings (e.g. urethral valves) have an influence on post-procedure prophylaxis?
Yes

Please specify:
Meist ohnehin Dauerprophylaxe

End of survey

Thank you for taking the time to answer this survey!

May we contact you personally if we have further questions?
Yes

E-mail address:
Guido.Fechner@ukbonn.de

Survey response 125

Response ID
127

Last page
0

Start language
en

Seed
859999592

Date started
2025-02-13 12:50:57

Date last action
2025-02-13 12:50:57

Standard Operation Procedure

Does your department have a defined protocol for infection prophylaxis in the context of VUS/VCUG?
N/A

Has this protocol been transformed into a written standard operating procedure (SOP)?
N/A

Antibiotic Prophylaxis

Are there situations/constellations in which you do not use antibiotic prophylaxis for VUS/VCUG?
N/A

Please specify these situations/constellations: [girl with first uncomplicated UTI without urinary tract dilation in ultrasound]
N/A

Please specify these situations/constellations: [boy with first uncomplicated UTI without urinary tract dilation in ultrasound]
N/A

Please specify these situations/constellations: [in patients with low grade urinary tract dilation (up to 2nd grade) and no UTI in medical history]
N/A

If you use antibiotic prophylaxis: which mediaction do you use? (multiple answers and subdivision into age groups possible) [Trimethoprim]
N/A

If you use antibiotic prophylaxis: which mediaction do you use? (multiple answers and subdivision into age groups possible) [Nitrofurantoin]
N/A

If you use antibiotic prophylaxis: which mediaction do you use? (multiple answers and subdivision into age groups possible) [Amoxicillin]
N/A

If you use antibiotic prophylaxis: which mediaction do you use? (multiple answers and subdivision into age groups possible) [Cefaclor]
N/A

If you use antibiotic prophylaxis: which mediaction do you use? (multiple answers and subdivision into age groups possible) [Amoxicillin/clavulanic acid]
N/A

If you use antibiotic prophylaxis: which mediaction do you use? (multiple answers and subdivision into age groups possible) [Other]
N/A

Trimethoprim: which time schedule do you use? [Day(s) before VUS/VCUG, how many?]
N/A

Trimethoprim: which time schedule do you use? [Day of VUS/VCUG]
N/A

Trimethoprim: which time schedule do you use? [Day(s) after VUS/VCUG, how many?]
N/A

Nitrofurantoin: which time schedule do you use? [Day(s) before VUS/VCUG, how many?]
N/A

Nitrofurantoin: which time schedule do you use? [Day of VUS/VCUG]
N/A

Nitrofurantoin: which time schedule do you use? [Day(s) after VUS/VCUG, how many?]
N/A

Amoxicillin: which time schedule do you use? [Day(s) before VUS/VCUG, how many?]
N/A

Amoxicillin: which time schedule do you use? [Day of VUS/VCUG]
N/A

Amoxicillin: which time schedule do you use? [Day(s) after VUS/VCUG, how many?]
N/A

Cefaclor: which time schedule do you use? [Days(s) before VUS/VCUG, how many?]
N/A

Cefaclor: which time schedule do you use? [Day of VUS/VCUG]
N/A

Cefaclor: which time schedule do you use? [Day(s) after VUS/VCUG, how many?]
N/A

Amoxicillin/clavulanic acid: in what dosage? (Amoxicillin portion) [prophylaktic (10-20 mg/Kg bodyweight/d)]
N/A

Amoxicillin/clavulanic acid: in what dosage? (Amoxicillin portion) [double prophylactic (20-40 mg/Kg bodyweight/d)]
N/A

Amoxicillin/clavulanic acid: in what dosage? (Amoxicillin portion) [therapeutic (80-100 mg/Kg bodyweight/d)]
N/A

Amoxicillin/clavulanic acid: which time schedule do you use? [Day(s) before VUS/VCUG]
N/A

Amoxicillin/clavulanic acid: which time schedule do you use? [Day of VUS/VCUG]
N/A

Amoxicillin/clavulanic acid: which time schedule do you use? [Day(s) after VUS/VCUG]
N/A

Other: which time schedule do you use? [Day(s) before VUS/VCUG, how many?]
N/A

Other: which time schedule do you use? [Day of VUS/VCUG]
N/A

Other: which time schedule do you use? [Day(s) after VUS/VCUG, how many?]
N/A

Indication for prophylaxis

Which patients receive periinterventional antibiotic prophylaxis? (multiple answers possible) [all]
N/A

Which patients receive periinterventional antibiotic prophylaxis? (multiple answers possible) [patients post urinary tract infection/pyelonephritis/urosepsis]
N/A

Which patients receive periinterventional antibiotic prophylaxis? (multiple answers possible) [patients with urinary tract dilation in ultrasound]
N/A

Which patients receive periinterventional antibiotic prophylaxis? (multiple answers possible) [patients with prenatal urinary tract dilation]
N/A

Which patients receive periinterventional antibiotic prophylaxis? (multiple answers possible) [patients post VUR surgery]
N/A

Which patients receive periinterventional antibiotic prophylaxis? (multiple answers possible) [none]
N/A

Are there situations or clinical findings in the patient where you deviate from your prophylaxis standard (medication, dose, duration, age of the patient)? (multiple answers possible, please specify in answer box) [in case of already known vesicoureteral reflux (VUR)]
N/A

Are there situations or clinical findings in the patient where you deviate from your prophylaxis standard (medication, dose, duration, age of the patient)? (multiple answers possible, please specify in answer box) [in patients with recurrent urinary tract infections (UTI)]
N/A

Are there situations or clinical findings in the patient where you deviate from your prophylaxis standard (medication, dose, duration, age of the patient)? (multiple answers possible, please specify in answer box) [if multidrug-resistant organisms (MDRO) are detected in urine cultures]
N/A

Are there situations or clinical findings in the patient where you deviate from your prophylaxis standard (medication, dose, duration, age of the patient)? (multiple answers possible, please specify in answer box) [in newborns]
N/A

Are there situations in which you use intravenous prophylaxis?
N/A

Voiding analysis

Are there differences in antibiotic prophylaxis between VUS and VCUG in your department?
N/A

Is a non-pathological urine strip test required in preparation for VUS/VCUG in your department?
N/A

Is a non-pathological urine culture required in preparation for VUS/VCUG in your department?
N/A

Do patients only receive post-procedure prophylaxis if vesicoureteral reflux (VUR) is detected in VUS/VCUG?
N/A

Do other MUS/MCU findings (e.g. urethral valves) have an influence on post-procedure prophylaxis?
N/A

End of survey

May we contact you personally if we have further questions?
N/A

Survey response 126

Response ID
128

Last page
1

Start language
en

Seed
145682032

Date started
2025-02-13 13:04:48

Date last action
2025-02-13 13:05:21

Standard Operation Procedure

Who fills out this survey?
Pediatric Radiologist

Who fills out this survey? [Other]

In which type of hospital are you employed?
University Hospital

In which type of hospital are you employed? [Other]

In which country do you work?
italy

Does your department have a defined protocol for infection prophylaxis in the context of VUS/VCUG?
Yes

Has this protocol been transformed into a written standard operating procedure (SOP)?
Yes

Which department is the author of the SOP for infection prophylaxis in VUS/VCUG?
Pediatric radiology

Which department is the author of the SOP for infection prophylaxis in VUS/VCUG? [Other]

Antibiotic Prophylaxis

Are there situations/constellations in which you do not use antibiotic prophylaxis for VUS/VCUG?
N/A

Please specify these situations/constellations: [girl with first uncomplicated UTI without urinary tract dilation in ultrasound]
N/A

Please specify these situations/constellations: [boy with first uncomplicated UTI without urinary tract dilation in ultrasound]
N/A

Please specify these situations/constellations: [in patients with low grade urinary tract dilation (up to 2nd grade) and no UTI in medical history]
N/A

If you use antibiotic prophylaxis: which mediaction do you use? (multiple answers and subdivision into age groups possible) [Trimethoprim]
N/A

If you use antibiotic prophylaxis: which mediaction do you use? (multiple answers and subdivision into age groups possible) [Nitrofurantoin]
N/A

If you use antibiotic prophylaxis: which mediaction do you use? (multiple answers and subdivision into age groups possible) [Amoxicillin]
N/A

If you use antibiotic prophylaxis: which mediaction do you use? (multiple answers and subdivision into age groups possible) [Cefaclor]
N/A

If you use antibiotic prophylaxis: which mediaction do you use? (multiple answers and subdivision into age groups possible) [Amoxicillin/clavulanic acid]
N/A

If you use antibiotic prophylaxis: which mediaction do you use? (multiple answers and subdivision into age groups possible) [Other]
N/A

Trimethoprim: which time schedule do you use? [Day(s) before VUS/VCUG, how many?]
N/A

Trimethoprim: which time schedule do you use? [Day of VUS/VCUG]
N/A

Trimethoprim: which time schedule do you use? [Day(s) after VUS/VCUG, how many?]
N/A

Nitrofurantoin: which time schedule do you use? [Day(s) before VUS/VCUG, how many?]
N/A

Nitrofurantoin: which time schedule do you use? [Day of VUS/VCUG]
N/A

Nitrofurantoin: which time schedule do you use? [Day(s) after VUS/VCUG, how many?]
N/A

Amoxicillin: which time schedule do you use? [Day(s) before VUS/VCUG, how many?]
N/A

Amoxicillin: which time schedule do you use? [Day of VUS/VCUG]
N/A

Amoxicillin: which time schedule do you use? [Day(s) after VUS/VCUG, how many?]
N/A

Cefaclor: which time schedule do you use? [Days(s) before VUS/VCUG, how many?]
N/A

Cefaclor: which time schedule do you use? [Day of VUS/VCUG]
N/A

Cefaclor: which time schedule do you use? [Day(s) after VUS/VCUG, how many?]
N/A

Amoxicillin/clavulanic acid: in what dosage? (Amoxicillin portion) [prophylaktic (10-20 mg/Kg bodyweight/d)]
N/A

Amoxicillin/clavulanic acid: in what dosage? (Amoxicillin portion) [double prophylactic (20-40 mg/Kg bodyweight/d)]
N/A

Amoxicillin/clavulanic acid: in what dosage? (Amoxicillin portion) [therapeutic (80-100 mg/Kg bodyweight/d)]
N/A

Amoxicillin/clavulanic acid: which time schedule do you use? [Day(s) before VUS/VCUG]
N/A

Amoxicillin/clavulanic acid: which time schedule do you use? [Day of VUS/VCUG]
N/A

Amoxicillin/clavulanic acid: which time schedule do you use? [Day(s) after VUS/VCUG]
N/A

Other: which time schedule do you use? [Day(s) before VUS/VCUG, how many?]
N/A

Other: which time schedule do you use? [Day of VUS/VCUG]
N/A

Other: which time schedule do you use? [Day(s) after VUS/VCUG, how many?]
N/A

Indication for prophylaxis

Which patients receive periinterventional antibiotic prophylaxis? (multiple answers possible) [all]
N/A

Which patients receive periinterventional antibiotic prophylaxis? (multiple answers possible) [patients post urinary tract infection/pyelonephritis/urosepsis]
N/A

Which patients receive periinterventional antibiotic prophylaxis? (multiple answers possible) [patients with urinary tract dilation in ultrasound]
N/A

Which patients receive periinterventional antibiotic prophylaxis? (multiple answers possible) [patients with prenatal urinary tract dilation]
N/A

Which patients receive periinterventional antibiotic prophylaxis? (multiple answers possible) [patients post VUR surgery]
N/A

Which patients receive periinterventional antibiotic prophylaxis? (multiple answers possible) [none]
N/A

Are there situations or clinical findings in the patient where you deviate from your prophylaxis standard (medication, dose, duration, age of the patient)? (multiple answers possible, please specify in answer box) [in case of already known vesicoureteral reflux (VUR)]
N/A

Are there situations or clinical findings in the patient where you deviate from your prophylaxis standard (medication, dose, duration, age of the patient)? (multiple answers possible, please specify in answer box) [in patients with recurrent urinary tract infections (UTI)]
N/A

Are there situations or clinical findings in the patient where you deviate from your prophylaxis standard (medication, dose, duration, age of the patient)? (multiple answers possible, please specify in answer box) [if multidrug-resistant organisms (MDRO) are detected in urine cultures]
N/A

Are there situations or clinical findings in the patient where you deviate from your prophylaxis standard (medication, dose, duration, age of the patient)? (multiple answers possible, please specify in answer box) [in newborns]
N/A

Are there situations in which you use intravenous prophylaxis?
N/A

Voiding analysis

Are there differences in antibiotic prophylaxis between VUS and VCUG in your department?
N/A

Is a non-pathological urine strip test required in preparation for VUS/VCUG in your department?
N/A

Is a non-pathological urine culture required in preparation for VUS/VCUG in your department?
N/A

Do patients only receive post-procedure prophylaxis if vesicoureteral reflux (VUR) is detected in VUS/VCUG?
N/A

Do other MUS/MCU findings (e.g. urethral valves) have an influence on post-procedure prophylaxis?
N/A

End of survey

May we contact you personally if we have further questions?
N/A

Survey response 127

Response ID
129

Date submitted
2025-02-13 13:42:23

Last page
5

Start language
en

Seed
1851959227

Date started
2025-02-13 13:38:44

Date last action
2025-02-13 13:42:23

Standard Operation Procedure

Who fills out this survey?
Pediatric Radiologist

Who fills out this survey? [Other]

In which type of hospital are you employed?
University Hospital

In which type of hospital are you employed? [Other]

In which country do you work?
United States

Does your department have a defined protocol for infection prophylaxis in the context of VUS/VCUG?
No

Has this protocol been transformed into a written standard operating procedure (SOP)?
N/A

Antibiotic Prophylaxis

Are there situations/constellations in which you do not use antibiotic prophylaxis for VUS/VCUG?
Yes

Please specify these situations/constellations: [girl with first uncomplicated UTI without urinary tract dilation in ultrasound]
Yes

Please specify these situations/constellations: [boy with first uncomplicated UTI without urinary tract dilation in ultrasound]
Yes

Please specify these situations/constellations: [in patients with low grade urinary tract dilation (up to 2nd grade) and no UTI in medical history]
Yes

Please specify these situations/constellations: [Other]

If you use antibiotic prophylaxis: which mediaction do you use? (multiple answers and subdivision into age groups possible) [Trimethoprim]
No

If you use antibiotic prophylaxis: which mediaction do you use? (multiple answers and subdivision into age groups possible) [Nitrofurantoin]
No

If you use antibiotic prophylaxis: which mediaction do you use? (multiple answers and subdivision into age groups possible) [Amoxicillin]
No

If you use antibiotic prophylaxis: which mediaction do you use? (multiple answers and subdivision into age groups possible) [Cefaclor]
No

If you use antibiotic prophylaxis: which mediaction do you use? (multiple answers and subdivision into age groups possible) [Amoxicillin/clavulanic acid]
No

If you use antibiotic prophylaxis: which mediaction do you use? (multiple answers and subdivision into age groups possible) [Other]
No

Trimethoprim: which time schedule do you use? [Day(s) before VUS/VCUG, how many?]
N/A

Trimethoprim: which time schedule do you use? [Day of VUS/VCUG]
N/A

Trimethoprim: which time schedule do you use? [Day(s) after VUS/VCUG, how many?]
N/A

Nitrofurantoin: which time schedule do you use? [Day(s) before VUS/VCUG, how many?]
N/A

Nitrofurantoin: which time schedule do you use? [Day of VUS/VCUG]
N/A

Nitrofurantoin: which time schedule do you use? [Day(s) after VUS/VCUG, how many?]
N/A

Amoxicillin: which time schedule do you use? [Day(s) before VUS/VCUG, how many?]
N/A

Amoxicillin: which time schedule do you use? [Day of VUS/VCUG]
N/A

Amoxicillin: which time schedule do you use? [Day(s) after VUS/VCUG, how many?]
N/A

Cefaclor: which time schedule do you use? [Days(s) before VUS/VCUG, how many?]
N/A

Cefaclor: which time schedule do you use? [Day of VUS/VCUG]
N/A

Cefaclor: which time schedule do you use? [Day(s) after VUS/VCUG, how many?]
N/A

Amoxicillin/clavulanic acid: in what dosage? (Amoxicillin portion) [prophylaktic (10-20 mg/Kg bodyweight/d)]
N/A

Amoxicillin/clavulanic acid: in what dosage? (Amoxicillin portion) [double prophylactic (20-40 mg/Kg bodyweight/d)]
N/A

Amoxicillin/clavulanic acid: in what dosage? (Amoxicillin portion) [therapeutic (80-100 mg/Kg bodyweight/d)]
N/A

Amoxicillin/clavulanic acid: which time schedule do you use? [Day(s) before VUS/VCUG]
N/A

Amoxicillin/clavulanic acid: which time schedule do you use? [Day of VUS/VCUG]
N/A

Amoxicillin/clavulanic acid: which time schedule do you use? [Day(s) after VUS/VCUG]
N/A

Other: which time schedule do you use? [Day(s) before VUS/VCUG, how many?]
N/A

Other: which time schedule do you use? [Day of VUS/VCUG]
N/A

Other: which time schedule do you use? [Day(s) after VUS/VCUG, how many?]
N/A

Indication for prophylaxis

Which patients receive periinterventional antibiotic prophylaxis? (multiple answers possible) [all]
No

Which patients receive periinterventional antibiotic prophylaxis? (multiple answers possible) [patients post urinary tract infection/pyelonephritis/urosepsis]
Yes

Which patients receive periinterventional antibiotic prophylaxis? (multiple answers possible) [patients with urinary tract dilation in ultrasound]
No

Which patients receive periinterventional antibiotic prophylaxis? (multiple answers possible) [patients with prenatal urinary tract dilation]
No

Which patients receive periinterventional antibiotic prophylaxis? (multiple answers possible) [patients post VUR surgery]
No

Which patients receive periinterventional antibiotic prophylaxis? (multiple answers possible) [none]
No

Which patients receive periinterventional antibiotic prophylaxis? (multiple answers possible) [Other]

patients status post urinary tract infection/pyelonephritis/urosepsis: what is the minimum time between infection and VUS/VCUG? (in days)
Variable

Are there situations or clinical findings in the patient where you deviate from your prophylaxis standard (medication, dose, duration, age of the patient)? (multiple answers possible, please specify in answer box) [in case of already known vesicoureteral reflux (VUR)]
No

Are there situations or clinical findings in the patient where you deviate from your prophylaxis standard (medication, dose, duration, age of the patient)? (multiple answers possible, please specify in answer box) [in case of already known vesicoureteral reflux (VUR)][Comment]

Are there situations or clinical findings in the patient where you deviate from your prophylaxis standard (medication, dose, duration, age of the patient)? (multiple answers possible, please specify in answer box) [in patients with recurrent urinary tract infections (UTI)]
Yes

Are there situations or clinical findings in the patient where you deviate from your prophylaxis standard (medication, dose, duration, age of the patient)? (multiple answers possible, please specify in answer box) [in patients with recurrent urinary tract infections (UTI)][Comment]

Are there situations or clinical findings in the patient where you deviate from your prophylaxis standard (medication, dose, duration, age of the patient)? (multiple answers possible, please specify in answer box) [if multidrug-resistant organisms (MDRO) are detected in urine cultures]
No

Are there situations or clinical findings in the patient where you deviate from your prophylaxis standard (medication, dose, duration, age of the patient)? (multiple answers possible, please specify in answer box) [if multidrug-resistant organisms (MDRO) are detected in urine cultures][Comment]

Are there situations or clinical findings in the patient where you deviate from your prophylaxis standard (medication, dose, duration, age of the patient)? (multiple answers possible, please specify in answer box) [in newborns]
No

Are there situations or clinical findings in the patient where you deviate from your prophylaxis standard (medication, dose, duration, age of the patient)? (multiple answers possible, please specify in answer box) [in newborns][Comment]

Are there situations or clinical findings in the patient where you deviate from your prophylaxis standard (medication, dose, duration, age of the patient)? (multiple answers possible, please specify in answer box) [Other]

Are there situations or clinical findings in the patient where you deviate from your prophylaxis standard (medication, dose, duration, age of the patient)? (multiple answers possible, please specify in answer box) [Other comment]

Are there situations in which you use intravenous prophylaxis?
No

Voiding analysis

Are there differences in antibiotic prophylaxis between VUS and VCUG in your department?
No

Is a non-pathological urine strip test required in preparation for VUS/VCUG in your department?
No

Is a non-pathological urine culture required in preparation for VUS/VCUG in your department?
No

Do patients only receive post-procedure prophylaxis if vesicoureteral reflux (VUR) is detected in VUS/VCUG?
Yes

Please explain your approach:
yes

Do other MUS/MCU findings (e.g. urethral valves) have an influence on post-procedure prophylaxis?
No

End of survey

Thank you for taking the time to answer this survey!

May we contact you personally if we have further questions?
No

Survey response 128

Response ID
130

Date submitted
2025-02-13 14:37:03

Last page
5

Start language
en

Seed
150571293

Date started
2025-02-13 14:25:04

Date last action
2025-02-13 14:37:03

Standard Operation Procedure

Who fills out this survey?
Pediatric Radiologist

Who fills out this survey? [Other]

In which type of hospital are you employed?
University Hospital

In which type of hospital are you employed? [Other]

In which country do you work?
Netherlands

Does your department have a defined protocol for infection prophylaxis in the context of VUS/VCUG?
Yes

Has this protocol been transformed into a written standard operating procedure (SOP)?
No

Antibiotic Prophylaxis

Are there situations/constellations in which you do not use antibiotic prophylaxis for VUS/VCUG?
No

Please specify these situations/constellations: [girl with first uncomplicated UTI without urinary tract dilation in ultrasound]
N/A

Please specify these situations/constellations: [boy with first uncomplicated UTI without urinary tract dilation in ultrasound]
N/A

Please specify these situations/constellations: [in patients with low grade urinary tract dilation (up to 2nd grade) and no UTI in medical history]
N/A

If you use antibiotic prophylaxis: which mediaction do you use? (multiple answers and subdivision into age groups possible) [Trimethoprim]
No

If you use antibiotic prophylaxis: which mediaction do you use? (multiple answers and subdivision into age groups possible) [Nitrofurantoin]
No

If you use antibiotic prophylaxis: which mediaction do you use? (multiple answers and subdivision into age groups possible) [Amoxicillin]
No

If you use antibiotic prophylaxis: which mediaction do you use? (multiple answers and subdivision into age groups possible) [Cefaclor]
No

If you use antibiotic prophylaxis: which mediaction do you use? (multiple answers and subdivision into age groups possible) [Amoxicillin/clavulanic acid]
Yes

If you use antibiotic prophylaxis: which mediaction do you use? (multiple answers and subdivision into age groups possible) [Other]
No

Trimethoprim: which time schedule do you use? [Day(s) before VUS/VCUG, how many?]
N/A

Trimethoprim: which time schedule do you use? [Day of VUS/VCUG]
N/A

Trimethoprim: which time schedule do you use? [Day(s) after VUS/VCUG, how many?]
N/A

Nitrofurantoin: which time schedule do you use? [Day(s) before VUS/VCUG, how many?]
N/A

Nitrofurantoin: which time schedule do you use? [Day of VUS/VCUG]
N/A

Nitrofurantoin: which time schedule do you use? [Day(s) after VUS/VCUG, how many?]
N/A

Amoxicillin: which time schedule do you use? [Day(s) before VUS/VCUG, how many?]
N/A

Amoxicillin: which time schedule do you use? [Day of VUS/VCUG]
N/A

Amoxicillin: which time schedule do you use? [Day(s) after VUS/VCUG, how many?]
N/A

Cefaclor: which time schedule do you use? [Days(s) before VUS/VCUG, how many?]
N/A

Cefaclor: which time schedule do you use? [Day of VUS/VCUG]
N/A

Cefaclor: which time schedule do you use? [Day(s) after VUS/VCUG, how many?]
N/A

Amoxicillin/clavulanic acid: in what dosage? (Amoxicillin portion) [prophylaktic (10-20 mg/Kg bodyweight/d)]
No

Amoxicillin/clavulanic acid: in what dosage? (Amoxicillin portion) [double prophylactic (20-40 mg/Kg bodyweight/d)]
Yes

Amoxicillin/clavulanic acid: in what dosage? (Amoxicillin portion) [therapeutic (80-100 mg/Kg bodyweight/d)]
No

Amoxicillin/clavulanic acid: in what dosage? (Amoxicillin portion) [Other]

Amoxicillin/clavulanic acid: for which age group do you use amoxicillin/clavulanic acid?
all ages

Amoxicillin/clavulanic acid: which time schedule do you use? [Day(s) before VUS/VCUG]
No

Amoxicillin/clavulanic acid: which time schedule do you use? [Day(s) before VUS/VCUG][Comment]

Amoxicillin/clavulanic acid: which time schedule do you use? [Day of VUS/VCUG]
Yes

Amoxicillin/clavulanic acid: which time schedule do you use? [Day of VUS/VCUG][Comment]

Amoxicillin/clavulanic acid: which time schedule do you use? [Day(s) after VUS/VCUG]
No

Amoxicillin/clavulanic acid: which time schedule do you use? [Day(s) after VUS/VCUG][Comment]

Other: which time schedule do you use? [Day(s) before VUS/VCUG, how many?]
N/A

Other: which time schedule do you use? [Day of VUS/VCUG]
N/A

Other: which time schedule do you use? [Day(s) after VUS/VCUG, how many?]
N/A

Indication for prophylaxis

Which patients receive periinterventional antibiotic prophylaxis? (multiple answers possible) [all]
Yes

Which patients receive periinterventional antibiotic prophylaxis? (multiple answers possible) [patients post urinary tract infection/pyelonephritis/urosepsis]
N/A

Which patients receive periinterventional antibiotic prophylaxis? (multiple answers possible) [patients with urinary tract dilation in ultrasound]
N/A

Which patients receive periinterventional antibiotic prophylaxis? (multiple answers possible) [patients with prenatal urinary tract dilation]
N/A

Which patients receive periinterventional antibiotic prophylaxis? (multiple answers possible) [patients post VUR surgery]
N/A

Which patients receive periinterventional antibiotic prophylaxis? (multiple answers possible) [none]
N/A

Are there situations or clinical findings in the patient where you deviate from your prophylaxis standard (medication, dose, duration, age of the patient)? (multiple answers possible, please specify in answer box) [in case of already known vesicoureteral reflux (VUR)]
No

Are there situations or clinical findings in the patient where you deviate from your prophylaxis standard (medication, dose, duration, age of the patient)? (multiple answers possible, please specify in answer box) [in case of already known vesicoureteral reflux (VUR)][Comment]

Are there situations or clinical findings in the patient where you deviate from your prophylaxis standard (medication, dose, duration, age of the patient)? (multiple answers possible, please specify in answer box) [in patients with recurrent urinary tract infections (UTI)]
No

Are there situations or clinical findings in the patient where you deviate from your prophylaxis standard (medication, dose, duration, age of the patient)? (multiple answers possible, please specify in answer box) [in patients with recurrent urinary tract infections (UTI)][Comment]

Are there situations or clinical findings in the patient where you deviate from your prophylaxis standard (medication, dose, duration, age of the patient)? (multiple answers possible, please specify in answer box) [if multidrug-resistant organisms (MDRO) are detected in urine cultures]
Yes

Are there situations or clinical findings in the patient where you deviate from your prophylaxis standard (medication, dose, duration, age of the patient)? (multiple answers possible, please specify in answer box) [if multidrug-resistant organisms (MDRO) are detected in urine cultures][Comment]

Are there situations or clinical findings in the patient where you deviate from your prophylaxis standard (medication, dose, duration, age of the patient)? (multiple answers possible, please specify in answer box) [in newborns]
No

Are there situations or clinical findings in the patient where you deviate from your prophylaxis standard (medication, dose, duration, age of the patient)? (multiple answers possible, please specify in answer box) [in newborns][Comment]

Are there situations or clinical findings in the patient where you deviate from your prophylaxis standard (medication, dose, duration, age of the patient)? (multiple answers possible, please specify in answer box) [Other]

Are there situations or clinical findings in the patient where you deviate from your prophylaxis standard (medication, dose, duration, age of the patient)? (multiple answers possible, please specify in answer box) [Other comment]

Are there situations in which you use intravenous prophylaxis?
No

Voiding analysis

Are there differences in antibiotic prophylaxis between VUS and VCUG in your department?
No

Is a non-pathological urine strip test required in preparation for VUS/VCUG in your department?
No

Is a non-pathological urine culture required in preparation for VUS/VCUG in your department?
No

Do patients only receive post-procedure prophylaxis if vesicoureteral reflux (VUR) is detected in VUS/VCUG?
No

Do other MUS/MCU findings (e.g. urethral valves) have an influence on post-procedure prophylaxis?
No

End of survey

Thank you for taking the time to answer this survey!

May we contact you personally if we have further questions?
Yes

E-mail address:
a.m.smets@amsterdamumc.nl

Survey response 129

Response ID
131

Date submitted
2025-02-13 16:50:25

Last page
5

Start language
de

Seed
721798540

Date started
2025-02-13 16:19:14

Date last action
2025-02-13 16:50:25

Standard Operation Procedure

Who fills out this survey?
Pediatric nephrologist

Who fills out this survey? [Other]

In which type of hospital are you employed?
University Hospital

In which type of hospital are you employed? [Other]

In which country do you work?
Deutschland

Does your department have a defined protocol for infection prophylaxis in the context of VUS/VCUG?
Yes

Has this protocol been transformed into a written standard operating procedure (SOP)?
No

Antibiotic Prophylaxis

Are there situations/constellations in which you do not use antibiotic prophylaxis for VUS/VCUG?
No

Please specify these situations/constellations: [girl with first uncomplicated UTI without urinary tract dilation in ultrasound]
N/A

Please specify these situations/constellations: [boy with first uncomplicated UTI without urinary tract dilation in ultrasound]
N/A

Please specify these situations/constellations: [in patients with low grade urinary tract dilation (up to 2nd grade) and no UTI in medical history]
N/A

If you use antibiotic prophylaxis: which mediaction do you use? (multiple answers and subdivision into age groups possible) [Trimethoprim]
Yes

If you use antibiotic prophylaxis: which mediaction do you use? (multiple answers and subdivision into age groups possible) [Nitrofurantoin]
Yes

If you use antibiotic prophylaxis: which mediaction do you use? (multiple answers and subdivision into age groups possible) [Amoxicillin]
No

If you use antibiotic prophylaxis: which mediaction do you use? (multiple answers and subdivision into age groups possible) [Cefaclor]
Yes

If you use antibiotic prophylaxis: which mediaction do you use? (multiple answers and subdivision into age groups possible) [Amoxicillin/clavulanic acid]
No

If you use antibiotic prophylaxis: which mediaction do you use? (multiple answers and subdivision into age groups possible) [Other]
No

Trimethoprim: in what dosage?
Other

Trimethoprim: in what dosage? [Other]
1-2mg/kgKG als Einmaldosis nach der Untersuchung

Trimethoprim: for which age group do you use trimethoprim?
Säuglinge ab 6 Wochen und bis ca. 15 kg bzw. wenn Nifuretten noch nicht sicher geschluckt werden können

Trimethoprim: which time schedule do you use? [Day(s) before VUS/VCUG, how many?]
Yes

Trimethoprim: which time schedule do you use? [Day(s) before VUS/VCUG, how many?][Comment]
ggf. laufende Dauerreinfektionsprophylaxe (diese wird entsprechend des Befundes nach der Untersuchung fortgesetzt oder beendet)

Trimethoprim: which time schedule do you use? [Day of VUS/VCUG]
Yes

Trimethoprim: which time schedule do you use? [Day of VUS/VCUG][Comment]

Trimethoprim: which time schedule do you use? [Day(s) after VUS/VCUG, how many?]
Yes

Trimethoprim: which time schedule do you use? [Day(s) after VUS/VCUG, how many?][Comment]
nur bei auffälligem Befund in RS mit weiterbetreuenden Nephrologen/Kinderchirurgen

Nitrofurantoin: in what dosage?
prophylactic (1-2 mg/kg bodyweight/d)

Nitrofurantoin: in what dosage? [Other]

Nitrofurantoin: for which age group do you use nitrofurantoin?
ab ca. 15 kg wenn Nifuretten geschuckt werden können

Nitrofurantoin: which time schedule do you use? [Day(s) before VUS/VCUG, how many?]
Yes

Nitrofurantoin: which time schedule do you use? [Day(s) before VUS/VCUG, how many?][Comment]
ggf. laufende Dauerreinfektionsprophylaxe (diese wird entsprechend des Befundes nach der Untersuchung fortgesetzt oder beendet)

Nitrofurantoin: which time schedule do you use? [Day of VUS/VCUG]
Yes

Nitrofurantoin: which time schedule do you use? [Day of VUS/VCUG][Comment]

Nitrofurantoin: which time schedule do you use? [Day(s) after VUS/VCUG, how many?]
Yes

Nitrofurantoin: which time schedule do you use? [Day(s) after VUS/VCUG, how many?][Comment]
nur bei auffälligem Befund in RS mit weiterbetreuenden Nephrologen/Kinderchirurgen

Amoxicillin: which time schedule do you use? [Day(s) before VUS/VCUG, how many?]
N/A

Amoxicillin: which time schedule do you use? [Day of VUS/VCUG]
N/A

Amoxicillin: which time schedule do you use? [Day(s) after VUS/VCUG, how many?]
N/A

Cefaclor: in what dosage?
prophylactic (10 mg/kg bodyweight/d)

Cefaclor: in what dosage? [Other]

Cefaclor: for which age group do you use cefaclor?
Säuglinge < 6 Wochen

Cefaclor: which time schedule do you use? [Days(s) before VUS/VCUG, how many?]
Yes

Cefaclor: which time schedule do you use? [Days(s) before VUS/VCUG, how many?][Comment]
ggf. laufende Dauerreinfektionsprophylaxe (diese wird entsprechend des Befundes nach der Untersuchung fortgesetzt oder beendet)

Cefaclor: which time schedule do you use? [Day of VUS/VCUG]
Yes

Cefaclor: which time schedule do you use? [Day of VUS/VCUG][Comment]

Cefaclor: which time schedule do you use? [Day(s) after VUS/VCUG, how many?]
Yes

Cefaclor: which time schedule do you use? [Day(s) after VUS/VCUG, how many?][Comment]
nur bei auffälligem Befund in RS mit weiterbetreuenden Nephrologen/Kinderchirurgen

Amoxicillin/clavulanic acid: in what dosage? (Amoxicillin portion) [prophylaktic (10-20 mg/Kg bodyweight/d)]
N/A

Amoxicillin/clavulanic acid: in what dosage? (Amoxicillin portion) [double prophylactic (20-40 mg/Kg bodyweight/d)]
N/A

Amoxicillin/clavulanic acid: in what dosage? (Amoxicillin portion) [therapeutic (80-100 mg/Kg bodyweight/d)]
N/A

Amoxicillin/clavulanic acid: which time schedule do you use? [Day(s) before VUS/VCUG]
N/A

Amoxicillin/clavulanic acid: which time schedule do you use? [Day of VUS/VCUG]
N/A

Amoxicillin/clavulanic acid: which time schedule do you use? [Day(s) after VUS/VCUG]
N/A

Other: which time schedule do you use? [Day(s) before VUS/VCUG, how many?]
N/A

Other: which time schedule do you use? [Day of VUS/VCUG]
N/A

Other: which time schedule do you use? [Day(s) after VUS/VCUG, how many?]
N/A

Indication for prophylaxis

Which patients receive periinterventional antibiotic prophylaxis? (multiple answers possible) [all]
Yes

Which patients receive periinterventional antibiotic prophylaxis? (multiple answers possible) [patients post urinary tract infection/pyelonephritis/urosepsis]
N/A

Which patients receive periinterventional antibiotic prophylaxis? (multiple answers possible) [patients with urinary tract dilation in ultrasound]
N/A

Which patients receive periinterventional antibiotic prophylaxis? (multiple answers possible) [patients with prenatal urinary tract dilation]
N/A

Which patients receive periinterventional antibiotic prophylaxis? (multiple answers possible) [patients post VUR surgery]
N/A

Which patients receive periinterventional antibiotic prophylaxis? (multiple answers possible) [none]
N/A

Are there situations or clinical findings in the patient where you deviate from your prophylaxis standard (medication, dose, duration, age of the patient)? (multiple answers possible, please specify in answer box) [in case of already known vesicoureteral reflux (VUR)]
Yes

Are there situations or clinical findings in the patient where you deviate from your prophylaxis standard (medication, dose, duration, age of the patient)? (multiple answers possible, please specify in answer box) [in case of already known vesicoureteral reflux (VUR)][Comment]
Entscheid in Abhängigkeit des Refluxgrades; teilweise auch Konsensfindung mit der Familie; Auswahl des Medikamentes in Abhängigkeit der bereits laufenden Prophylaxe/vorbekanntem Resistenzspektrum der Keime

Are there situations or clinical findings in the patient where you deviate from your prophylaxis standard (medication, dose, duration, age of the patient)? (multiple answers possible, please specify in answer box) [in patients with recurrent urinary tract infections (UTI)]
Yes

Are there situations or clinical findings in the patient where you deviate from your prophylaxis standard (medication, dose, duration, age of the patient)? (multiple answers possible, please specify in answer box) [in patients with recurrent urinary tract infections (UTI)][Comment]
Entscheid in Abhängigkeit des Refluxgrades; teilweise auch Konsensfindung mit der Familie; Versuch alternativer Prophylaxemaßnahmen und Optimierung Miktions- und Defäkationsgewohnheiten

Are there situations or clinical findings in the patient where you deviate from your prophylaxis standard (medication, dose, duration, age of the patient)? (multiple answers possible, please specify in answer box) [if multidrug-resistant organisms (MDRO) are detected in urine cultures]
Yes

Are there situations or clinical findings in the patient where you deviate from your prophylaxis standard (medication, dose, duration, age of the patient)? (multiple answers possible, please specify in answer box) [if multidrug-resistant organisms (MDRO) are detected in urine cultures][Comment]
Versuch Anpassung nach Resistenzspektrum

Are there situations or clinical findings in the patient where you deviate from your prophylaxis standard (medication, dose, duration, age of the patient)? (multiple answers possible, please specify in answer box) [in newborns]
Yes

Are there situations or clinical findings in the patient where you deviate from your prophylaxis standard (medication, dose, duration, age of the patient)? (multiple answers possible, please specify in answer box) [in newborns][Comment]
idealerweise Cefaclor; b.B. z.B. Resistenzspektrum auch alternative Prophylaxe

Are there situations or clinical findings in the patient where you deviate from your prophylaxis standard (medication, dose, duration, age of the patient)? (multiple answers possible, please specify in answer box) [Other]

Are there situations or clinical findings in the patient where you deviate from your prophylaxis standard (medication, dose, duration, age of the patient)? (multiple answers possible, please specify in answer box) [Other comment]

Are there situations in which you use intravenous prophylaxis?
No

Voiding analysis

Are there differences in antibiotic prophylaxis between VUS and VCUG in your department?
No

Is a non-pathological urine strip test required in preparation for VUS/VCUG in your department?
Yes

What is the maximum amount of days accepted for urine strip test to be performed prior to the exam?
3-(7) Tage

Is a non-pathological urine culture required in preparation for VUS/VCUG in your department?
No

Do patients only receive post-procedure prophylaxis if vesicoureteral reflux (VUR) is detected in VUS/VCUG?
No

Do other MUS/MCU findings (e.g. urethral valves) have an influence on post-procedure prophylaxis?
No

End of survey

Thank you for taking the time to answer this survey!

May we contact you personally if we have further questions?
Yes

E-mail address:
brigitte.mayer@ukdd.de

Survey response 130

Response ID
132

Last page
0

Start language
en

Seed
960772445

Date started
2025-02-17 15:49:30

Date last action
2025-02-17 15:49:30

Standard Operation Procedure

Does your department have a defined protocol for infection prophylaxis in the context of VUS/VCUG?
N/A

Has this protocol been transformed into a written standard operating procedure (SOP)?
N/A

Antibiotic Prophylaxis

Are there situations/constellations in which you do not use antibiotic prophylaxis for VUS/VCUG?
N/A

Please specify these situations/constellations: [girl with first uncomplicated UTI without urinary tract dilation in ultrasound]
N/A

Please specify these situations/constellations: [boy with first uncomplicated UTI without urinary tract dilation in ultrasound]
N/A

Please specify these situations/constellations: [in patients with low grade urinary tract dilation (up to 2nd grade) and no UTI in medical history]
N/A

If you use antibiotic prophylaxis: which mediaction do you use? (multiple answers and subdivision into age groups possible) [Trimethoprim]
N/A

If you use antibiotic prophylaxis: which mediaction do you use? (multiple answers and subdivision into age groups possible) [Nitrofurantoin]
N/A

If you use antibiotic prophylaxis: which mediaction do you use? (multiple answers and subdivision into age groups possible) [Amoxicillin]
N/A

If you use antibiotic prophylaxis: which mediaction do you use? (multiple answers and subdivision into age groups possible) [Cefaclor]
N/A

If you use antibiotic prophylaxis: which mediaction do you use? (multiple answers and subdivision into age groups possible) [Amoxicillin/clavulanic acid]
N/A

If you use antibiotic prophylaxis: which mediaction do you use? (multiple answers and subdivision into age groups possible) [Other]
N/A

Trimethoprim: which time schedule do you use? [Day(s) before VUS/VCUG, how many?]
N/A

Trimethoprim: which time schedule do you use? [Day of VUS/VCUG]
N/A

Trimethoprim: which time schedule do you use? [Day(s) after VUS/VCUG, how many?]
N/A

Nitrofurantoin: which time schedule do you use? [Day(s) before VUS/VCUG, how many?]
N/A

Nitrofurantoin: which time schedule do you use? [Day of VUS/VCUG]
N/A

Nitrofurantoin: which time schedule do you use? [Day(s) after VUS/VCUG, how many?]
N/A

Amoxicillin: which time schedule do you use? [Day(s) before VUS/VCUG, how many?]
N/A

Amoxicillin: which time schedule do you use? [Day of VUS/VCUG]
N/A

Amoxicillin: which time schedule do you use? [Day(s) after VUS/VCUG, how many?]
N/A

Cefaclor: which time schedule do you use? [Days(s) before VUS/VCUG, how many?]
N/A

Cefaclor: which time schedule do you use? [Day of VUS/VCUG]
N/A

Cefaclor: which time schedule do you use? [Day(s) after VUS/VCUG, how many?]
N/A

Amoxicillin/clavulanic acid: in what dosage? (Amoxicillin portion) [prophylaktic (10-20 mg/Kg bodyweight/d)]
N/A

Amoxicillin/clavulanic acid: in what dosage? (Amoxicillin portion) [double prophylactic (20-40 mg/Kg bodyweight/d)]
N/A

Amoxicillin/clavulanic acid: in what dosage? (Amoxicillin portion) [therapeutic (80-100 mg/Kg bodyweight/d)]
N/A

Amoxicillin/clavulanic acid: which time schedule do you use? [Day(s) before VUS/VCUG]
N/A

Amoxicillin/clavulanic acid: which time schedule do you use? [Day of VUS/VCUG]
N/A

Amoxicillin/clavulanic acid: which time schedule do you use? [Day(s) after VUS/VCUG]
N/A

Other: which time schedule do you use? [Day(s) before VUS/VCUG, how many?]
N/A

Other: which time schedule do you use? [Day of VUS/VCUG]
N/A

Other: which time schedule do you use? [Day(s) after VUS/VCUG, how many?]
N/A

Indication for prophylaxis

Which patients receive periinterventional antibiotic prophylaxis? (multiple answers possible) [all]
N/A

Which patients receive periinterventional antibiotic prophylaxis? (multiple answers possible) [patients post urinary tract infection/pyelonephritis/urosepsis]
N/A

Which patients receive periinterventional antibiotic prophylaxis? (multiple answers possible) [patients with urinary tract dilation in ultrasound]
N/A

Which patients receive periinterventional antibiotic prophylaxis? (multiple answers possible) [patients with prenatal urinary tract dilation]
N/A

Which patients receive periinterventional antibiotic prophylaxis? (multiple answers possible) [patients post VUR surgery]
N/A

Which patients receive periinterventional antibiotic prophylaxis? (multiple answers possible) [none]
N/A

Are there situations or clinical findings in the patient where you deviate from your prophylaxis standard (medication, dose, duration, age of the patient)? (multiple answers possible, please specify in answer box) [in case of already known vesicoureteral reflux (VUR)]
N/A

Are there situations or clinical findings in the patient where you deviate from your prophylaxis standard (medication, dose, duration, age of the patient)? (multiple answers possible, please specify in answer box) [in patients with recurrent urinary tract infections (UTI)]
N/A

Are there situations or clinical findings in the patient where you deviate from your prophylaxis standard (medication, dose, duration, age of the patient)? (multiple answers possible, please specify in answer box) [if multidrug-resistant organisms (MDRO) are detected in urine cultures]
N/A

Are there situations or clinical findings in the patient where you deviate from your prophylaxis standard (medication, dose, duration, age of the patient)? (multiple answers possible, please specify in answer box) [in newborns]
N/A

Are there situations in which you use intravenous prophylaxis?
N/A

Voiding analysis

Are there differences in antibiotic prophylaxis between VUS and VCUG in your department?
N/A

Is a non-pathological urine strip test required in preparation for VUS/VCUG in your department?
N/A

Is a non-pathological urine culture required in preparation for VUS/VCUG in your department?
N/A

Do patients only receive post-procedure prophylaxis if vesicoureteral reflux (VUR) is detected in VUS/VCUG?
N/A

Do other MUS/MCU findings (e.g. urethral valves) have an influence on post-procedure prophylaxis?
N/A

End of survey

May we contact you personally if we have further questions?
N/A

Survey response 131

Response ID
133

Last page
0

Start language
en

Seed
249340496

Date started
2025-02-17 15:50:12

Date last action
2025-02-17 15:50:31

Standard Operation Procedure

Who fills out this survey?

Who fills out this survey? [Other]

In which type of hospital are you employed?

In which type of hospital are you employed? [Other]

In which country do you work?

Does your department have a defined protocol for infection prophylaxis in the context of VUS/VCUG?
N/A

Has this protocol been transformed into a written standard operating procedure (SOP)?
N/A

Antibiotic Prophylaxis

Are there situations/constellations in which you do not use antibiotic prophylaxis for VUS/VCUG?
N/A

Please specify these situations/constellations: [girl with first uncomplicated UTI without urinary tract dilation in ultrasound]
N/A

Please specify these situations/constellations: [boy with first uncomplicated UTI without urinary tract dilation in ultrasound]
N/A

Please specify these situations/constellations: [in patients with low grade urinary tract dilation (up to 2nd grade) and no UTI in medical history]
N/A

If you use antibiotic prophylaxis: which mediaction do you use? (multiple answers and subdivision into age groups possible) [Trimethoprim]
N/A

If you use antibiotic prophylaxis: which mediaction do you use? (multiple answers and subdivision into age groups possible) [Nitrofurantoin]
N/A

If you use antibiotic prophylaxis: which mediaction do you use? (multiple answers and subdivision into age groups possible) [Amoxicillin]
N/A

If you use antibiotic prophylaxis: which mediaction do you use? (multiple answers and subdivision into age groups possible) [Cefaclor]
N/A

If you use antibiotic prophylaxis: which mediaction do you use? (multiple answers and subdivision into age groups possible) [Amoxicillin/clavulanic acid]
N/A

If you use antibiotic prophylaxis: which mediaction do you use? (multiple answers and subdivision into age groups possible) [Other]
N/A

Trimethoprim: which time schedule do you use? [Day(s) before VUS/VCUG, how many?]
N/A

Trimethoprim: which time schedule do you use? [Day of VUS/VCUG]
N/A

Trimethoprim: which time schedule do you use? [Day(s) after VUS/VCUG, how many?]
N/A

Nitrofurantoin: which time schedule do you use? [Day(s) before VUS/VCUG, how many?]
N/A

Nitrofurantoin: which time schedule do you use? [Day of VUS/VCUG]
N/A

Nitrofurantoin: which time schedule do you use? [Day(s) after VUS/VCUG, how many?]
N/A

Amoxicillin: which time schedule do you use? [Day(s) before VUS/VCUG, how many?]
N/A

Amoxicillin: which time schedule do you use? [Day of VUS/VCUG]
N/A

Amoxicillin: which time schedule do you use? [Day(s) after VUS/VCUG, how many?]
N/A

Cefaclor: which time schedule do you use? [Days(s) before VUS/VCUG, how many?]
N/A

Cefaclor: which time schedule do you use? [Day of VUS/VCUG]
N/A

Cefaclor: which time schedule do you use? [Day(s) after VUS/VCUG, how many?]
N/A

Amoxicillin/clavulanic acid: in what dosage? (Amoxicillin portion) [prophylaktic (10-20 mg/Kg bodyweight/d)]
N/A

Amoxicillin/clavulanic acid: in what dosage? (Amoxicillin portion) [double prophylactic (20-40 mg/Kg bodyweight/d)]
N/A

Amoxicillin/clavulanic acid: in what dosage? (Amoxicillin portion) [therapeutic (80-100 mg/Kg bodyweight/d)]
N/A

Amoxicillin/clavulanic acid: which time schedule do you use? [Day(s) before VUS/VCUG]
N/A

Amoxicillin/clavulanic acid: which time schedule do you use? [Day of VUS/VCUG]
N/A

Amoxicillin/clavulanic acid: which time schedule do you use? [Day(s) after VUS/VCUG]
N/A

Other: which time schedule do you use? [Day(s) before VUS/VCUG, how many?]
N/A

Other: which time schedule do you use? [Day of VUS/VCUG]
N/A

Other: which time schedule do you use? [Day(s) after VUS/VCUG, how many?]
N/A

Indication for prophylaxis

Which patients receive periinterventional antibiotic prophylaxis? (multiple answers possible) [all]
N/A

Which patients receive periinterventional antibiotic prophylaxis? (multiple answers possible) [patients post urinary tract infection/pyelonephritis/urosepsis]
N/A

Which patients receive periinterventional antibiotic prophylaxis? (multiple answers possible) [patients with urinary tract dilation in ultrasound]
N/A

Which patients receive periinterventional antibiotic prophylaxis? (multiple answers possible) [patients with prenatal urinary tract dilation]
N/A

Which patients receive periinterventional antibiotic prophylaxis? (multiple answers possible) [patients post VUR surgery]
N/A

Which patients receive periinterventional antibiotic prophylaxis? (multiple answers possible) [none]
N/A

Are there situations or clinical findings in the patient where you deviate from your prophylaxis standard (medication, dose, duration, age of the patient)? (multiple answers possible, please specify in answer box) [in case of already known vesicoureteral reflux (VUR)]
N/A

Are there situations or clinical findings in the patient where you deviate from your prophylaxis standard (medication, dose, duration, age of the patient)? (multiple answers possible, please specify in answer box) [in patients with recurrent urinary tract infections (UTI)]
N/A

Are there situations or clinical findings in the patient where you deviate from your prophylaxis standard (medication, dose, duration, age of the patient)? (multiple answers possible, please specify in answer box) [if multidrug-resistant organisms (MDRO) are detected in urine cultures]
N/A

Are there situations or clinical findings in the patient where you deviate from your prophylaxis standard (medication, dose, duration, age of the patient)? (multiple answers possible, please specify in answer box) [in newborns]
N/A

Are there situations in which you use intravenous prophylaxis?
N/A

Voiding analysis

Are there differences in antibiotic prophylaxis between VUS and VCUG in your department?
N/A

Is a non-pathological urine strip test required in preparation for VUS/VCUG in your department?
N/A

Is a non-pathological urine culture required in preparation for VUS/VCUG in your department?
N/A

Do patients only receive post-procedure prophylaxis if vesicoureteral reflux (VUR) is detected in VUS/VCUG?
N/A

Do other MUS/MCU findings (e.g. urethral valves) have an influence on post-procedure prophylaxis?
N/A

End of survey

May we contact you personally if we have further questions?
N/A

Survey response 132

Response ID
134

Date submitted
2025-02-18 07:39:15

Last page
5

Start language
en

Seed
516372154

Date started
2025-02-18 07:36:58

Date last action
2025-02-18 07:39:15

Standard Operation Procedure

Who fills out this survey?
Pediatric Radiologist

Who fills out this survey? [Other]

In which type of hospital are you employed?
University Hospital

In which type of hospital are you employed? [Other]

In which country do you work?
Netherlands

Does your department have a defined protocol for infection prophylaxis in the context of VUS/VCUG?
Yes

Has this protocol been transformed into a written standard operating procedure (SOP)?
No

Antibiotic Prophylaxis

Are there situations/constellations in which you do not use antibiotic prophylaxis for VUS/VCUG?
No

Please specify these situations/constellations: [girl with first uncomplicated UTI without urinary tract dilation in ultrasound]
N/A

Please specify these situations/constellations: [boy with first uncomplicated UTI without urinary tract dilation in ultrasound]
N/A

Please specify these situations/constellations: [in patients with low grade urinary tract dilation (up to 2nd grade) and no UTI in medical history]
N/A

If you use antibiotic prophylaxis: which mediaction do you use? (multiple answers and subdivision into age groups possible) [Trimethoprim]
Yes

If you use antibiotic prophylaxis: which mediaction do you use? (multiple answers and subdivision into age groups possible) [Nitrofurantoin]
No

If you use antibiotic prophylaxis: which mediaction do you use? (multiple answers and subdivision into age groups possible) [Amoxicillin]
No

If you use antibiotic prophylaxis: which mediaction do you use? (multiple answers and subdivision into age groups possible) [Cefaclor]
No

If you use antibiotic prophylaxis: which mediaction do you use? (multiple answers and subdivision into age groups possible) [Amoxicillin/clavulanic acid]
No

If you use antibiotic prophylaxis: which mediaction do you use? (multiple answers and subdivision into age groups possible) [Other]
No

Trimethoprim: in what dosage?
double prophylakctic (4 mg/kg bodyweight/d)

Trimethoprim: in what dosage? [Other]

Trimethoprim: for which age group do you use trimethoprim?
all children

Trimethoprim: which time schedule do you use? [Day(s) before VUS/VCUG, how many?]
Yes

Trimethoprim: which time schedule do you use? [Day(s) before VUS/VCUG, how many?][Comment]
1

Trimethoprim: which time schedule do you use? [Day of VUS/VCUG]
Yes

Trimethoprim: which time schedule do you use? [Day of VUS/VCUG][Comment]

Trimethoprim: which time schedule do you use? [Day(s) after VUS/VCUG, how many?]
Yes

Trimethoprim: which time schedule do you use? [Day(s) after VUS/VCUG, how many?][Comment]
1

Nitrofurantoin: which time schedule do you use? [Day(s) before VUS/VCUG, how many?]
N/A

Nitrofurantoin: which time schedule do you use? [Day of VUS/VCUG]
N/A

Nitrofurantoin: which time schedule do you use? [Day(s) after VUS/VCUG, how many?]
N/A

Amoxicillin: which time schedule do you use? [Day(s) before VUS/VCUG, how many?]
N/A

Amoxicillin: which time schedule do you use? [Day of VUS/VCUG]
N/A

Amoxicillin: which time schedule do you use? [Day(s) after VUS/VCUG, how many?]
N/A

Cefaclor: which time schedule do you use? [Days(s) before VUS/VCUG, how many?]
N/A

Cefaclor: which time schedule do you use? [Day of VUS/VCUG]
N/A

Cefaclor: which time schedule do you use? [Day(s) after VUS/VCUG, how many?]
N/A

Amoxicillin/clavulanic acid: in what dosage? (Amoxicillin portion) [prophylaktic (10-20 mg/Kg bodyweight/d)]
N/A

Amoxicillin/clavulanic acid: in what dosage? (Amoxicillin portion) [double prophylactic (20-40 mg/Kg bodyweight/d)]
N/A

Amoxicillin/clavulanic acid: in what dosage? (Amoxicillin portion) [therapeutic (80-100 mg/Kg bodyweight/d)]
N/A

Amoxicillin/clavulanic acid: which time schedule do you use? [Day(s) before VUS/VCUG]
N/A

Amoxicillin/clavulanic acid: which time schedule do you use? [Day of VUS/VCUG]
N/A

Amoxicillin/clavulanic acid: which time schedule do you use? [Day(s) after VUS/VCUG]
N/A

Other: which time schedule do you use? [Day(s) before VUS/VCUG, how many?]
N/A

Other: which time schedule do you use? [Day of VUS/VCUG]
N/A

Other: which time schedule do you use? [Day(s) after VUS/VCUG, how many?]
N/A

Indication for prophylaxis

Which patients receive periinterventional antibiotic prophylaxis? (multiple answers possible) [all]
Yes

Which patients receive periinterventional antibiotic prophylaxis? (multiple answers possible) [patients post urinary tract infection/pyelonephritis/urosepsis]
N/A

Which patients receive periinterventional antibiotic prophylaxis? (multiple answers possible) [patients with urinary tract dilation in ultrasound]
N/A

Which patients receive periinterventional antibiotic prophylaxis? (multiple answers possible) [patients with prenatal urinary tract dilation]
N/A

Which patients receive periinterventional antibiotic prophylaxis? (multiple answers possible) [patients post VUR surgery]
N/A

Which patients receive periinterventional antibiotic prophylaxis? (multiple answers possible) [none]
N/A

Are there situations or clinical findings in the patient where you deviate from your prophylaxis standard (medication, dose, duration, age of the patient)? (multiple answers possible, please specify in answer box) [in case of already known vesicoureteral reflux (VUR)]
No

Are there situations or clinical findings in the patient where you deviate from your prophylaxis standard (medication, dose, duration, age of the patient)? (multiple answers possible, please specify in answer box) [in case of already known vesicoureteral reflux (VUR)][Comment]

Are there situations or clinical findings in the patient where you deviate from your prophylaxis standard (medication, dose, duration, age of the patient)? (multiple answers possible, please specify in answer box) [in patients with recurrent urinary tract infections (UTI)]
No

Are there situations or clinical findings in the patient where you deviate from your prophylaxis standard (medication, dose, duration, age of the patient)? (multiple answers possible, please specify in answer box) [in patients with recurrent urinary tract infections (UTI)][Comment]

Are there situations or clinical findings in the patient where you deviate from your prophylaxis standard (medication, dose, duration, age of the patient)? (multiple answers possible, please specify in answer box) [if multidrug-resistant organisms (MDRO) are detected in urine cultures]
No

Are there situations or clinical findings in the patient where you deviate from your prophylaxis standard (medication, dose, duration, age of the patient)? (multiple answers possible, please specify in answer box) [if multidrug-resistant organisms (MDRO) are detected in urine cultures][Comment]

Are there situations or clinical findings in the patient where you deviate from your prophylaxis standard (medication, dose, duration, age of the patient)? (multiple answers possible, please specify in answer box) [in newborns]
No

Are there situations or clinical findings in the patient where you deviate from your prophylaxis standard (medication, dose, duration, age of the patient)? (multiple answers possible, please specify in answer box) [in newborns][Comment]

Are there situations or clinical findings in the patient where you deviate from your prophylaxis standard (medication, dose, duration, age of the patient)? (multiple answers possible, please specify in answer box) [Other]
no

Are there situations or clinical findings in the patient where you deviate from your prophylaxis standard (medication, dose, duration, age of the patient)? (multiple answers possible, please specify in answer box) [Other comment]

Are there situations in which you use intravenous prophylaxis?
No

Voiding analysis

Are there differences in antibiotic prophylaxis between VUS and VCUG in your department?
No

Is a non-pathological urine strip test required in preparation for VUS/VCUG in your department?
No

Is a non-pathological urine culture required in preparation for VUS/VCUG in your department?
No

Do patients only receive post-procedure prophylaxis if vesicoureteral reflux (VUR) is detected in VUS/VCUG?
No

Do other MUS/MCU findings (e.g. urethral valves) have an influence on post-procedure prophylaxis?
No

End of survey

Thank you for taking the time to answer this survey!

May we contact you personally if we have further questions?
Yes

E-mail address:
m.verhagen@umcg.nl

Survey response 133

Response ID
135

Date submitted
2025-03-07 14:28:31

Last page
5

Start language
de

Seed
742153633

Date started
2025-03-07 14:18:11

Date last action
2025-03-07 14:28:31

Standard Operation Procedure

Who fills out this survey?
Pediatric nephrologist

Who fills out this survey? [Other]

In which type of hospital are you employed?
University Hospital

In which type of hospital are you employed? [Other]

In which country do you work?
Deutschland

Does your department have a defined protocol for infection prophylaxis in the context of VUS/VCUG?
Yes

Has this protocol been transformed into a written standard operating procedure (SOP)?
No

Antibiotic Prophylaxis

Are there situations/constellations in which you do not use antibiotic prophylaxis for VUS/VCUG?
Yes

Please specify these situations/constellations: [girl with first uncomplicated UTI without urinary tract dilation in ultrasound]
No

Please specify these situations/constellations: [boy with first uncomplicated UTI without urinary tract dilation in ultrasound]
No

Please specify these situations/constellations: [in patients with low grade urinary tract dilation (up to 2nd grade) and no UTI in medical history]
Yes

Please specify these situations/constellations: [Other]

If you use antibiotic prophylaxis: which mediaction do you use? (multiple answers and subdivision into age groups possible) [Trimethoprim]
Yes

If you use antibiotic prophylaxis: which mediaction do you use? (multiple answers and subdivision into age groups possible) [Nitrofurantoin]
Yes

If you use antibiotic prophylaxis: which mediaction do you use? (multiple answers and subdivision into age groups possible) [Amoxicillin]
No

If you use antibiotic prophylaxis: which mediaction do you use? (multiple answers and subdivision into age groups possible) [Cefaclor]
Yes

If you use antibiotic prophylaxis: which mediaction do you use? (multiple answers and subdivision into age groups possible) [Amoxicillin/clavulanic acid]
No

If you use antibiotic prophylaxis: which mediaction do you use? (multiple answers and subdivision into age groups possible) [Other]
No

Trimethoprim: in what dosage?
prophylactic (2 mg/kg bodyweight/d)

Trimethoprim: in what dosage? [Other]

Trimethoprim: for which age group do you use trimethoprim?
Ab 6 Wochen

Trimethoprim: which time schedule do you use? [Day(s) before VUS/VCUG, how many?]
Yes

Trimethoprim: which time schedule do you use? [Day(s) before VUS/VCUG, how many?][Comment]
Ab Indikationsstellung MCU/MUS

Trimethoprim: which time schedule do you use? [Day of VUS/VCUG]
No

Trimethoprim: which time schedule do you use? [Day of VUS/VCUG][Comment]

Trimethoprim: which time schedule do you use? [Day(s) after VUS/VCUG, how many?]
Yes

Trimethoprim: which time schedule do you use? [Day(s) after VUS/VCUG, how many?][Comment]
Therapeutische Dosis für 3 Tage post MCU/MUS

Nitrofurantoin: in what dosage?
prophylactic (1-2 mg/kg bodyweight/d)

Nitrofurantoin: in what dosage? [Other]

Nitrofurantoin: for which age group do you use nitrofurantoin?
Ab 3 Monaten

Nitrofurantoin: which time schedule do you use? [Day(s) before VUS/VCUG, how many?]
Yes

Nitrofurantoin: which time schedule do you use? [Day(s) before VUS/VCUG, how many?][Comment]
Ab Indikationsstellung MCU/MUS

Nitrofurantoin: which time schedule do you use? [Day of VUS/VCUG]
No

Nitrofurantoin: which time schedule do you use? [Day of VUS/VCUG][Comment]

Nitrofurantoin: which time schedule do you use? [Day(s) after VUS/VCUG, how many?]
Yes

Nitrofurantoin: which time schedule do you use? [Day(s) after VUS/VCUG, how many?][Comment]
Therapeutische Dosis für 3 Tage post MCU/MUS

Amoxicillin: which time schedule do you use? [Day(s) before VUS/VCUG, how many?]
N/A

Amoxicillin: which time schedule do you use? [Day of VUS/VCUG]
N/A

Amoxicillin: which time schedule do you use? [Day(s) after VUS/VCUG, how many?]
N/A

Cefaclor: in what dosage?
prophylactic (10 mg/kg bodyweight/d)

Cefaclor: in what dosage? [Other]

Cefaclor: for which age group do you use cefaclor?
0-6 Wochen

Cefaclor: which time schedule do you use? [Days(s) before VUS/VCUG, how many?]
Yes

Cefaclor: which time schedule do you use? [Days(s) before VUS/VCUG, how many?][Comment]
Ab Indikationsstellung MCU/MUS

Cefaclor: which time schedule do you use? [Day of VUS/VCUG]
No

Cefaclor: which time schedule do you use? [Day of VUS/VCUG][Comment]

Cefaclor: which time schedule do you use? [Day(s) after VUS/VCUG, how many?]
Yes

Cefaclor: which time schedule do you use? [Day(s) after VUS/VCUG, how many?][Comment]
Therapeutische Dosis für 3 Tage post MCU/MUS

Amoxicillin/clavulanic acid: in what dosage? (Amoxicillin portion) [prophylaktic (10-20 mg/Kg bodyweight/d)]
N/A

Amoxicillin/clavulanic acid: in what dosage? (Amoxicillin portion) [double prophylactic (20-40 mg/Kg bodyweight/d)]
N/A

Amoxicillin/clavulanic acid: in what dosage? (Amoxicillin portion) [therapeutic (80-100 mg/Kg bodyweight/d)]
N/A

Amoxicillin/clavulanic acid: which time schedule do you use? [Day(s) before VUS/VCUG]
N/A

Amoxicillin/clavulanic acid: which time schedule do you use? [Day of VUS/VCUG]
N/A

Amoxicillin/clavulanic acid: which time schedule do you use? [Day(s) after VUS/VCUG]
N/A

Other: which time schedule do you use? [Day(s) before VUS/VCUG, how many?]
N/A

Other: which time schedule do you use? [Day of VUS/VCUG]
N/A

Other: which time schedule do you use? [Day(s) after VUS/VCUG, how many?]
N/A

Indication for prophylaxis

Which patients receive periinterventional antibiotic prophylaxis? (multiple answers possible) [all]
No

Which patients receive periinterventional antibiotic prophylaxis? (multiple answers possible) [patients post urinary tract infection/pyelonephritis/urosepsis]
Yes

Which patients receive periinterventional antibiotic prophylaxis? (multiple answers possible) [patients with urinary tract dilation in ultrasound]
No

Which patients receive periinterventional antibiotic prophylaxis? (multiple answers possible) [patients with prenatal urinary tract dilation]
No

Which patients receive periinterventional antibiotic prophylaxis? (multiple answers possible) [patients post VUR surgery]
Yes

Which patients receive periinterventional antibiotic prophylaxis? (multiple answers possible) [none]
No

Which patients receive periinterventional antibiotic prophylaxis? (multiple answers possible) [Other]

patients status post urinary tract infection/pyelonephritis/urosepsis: what is the minimum time between infection and VUS/VCUG? (in days)
Urin saniert

Are there situations or clinical findings in the patient where you deviate from your prophylaxis standard (medication, dose, duration, age of the patient)? (multiple answers possible, please specify in answer box) [in case of already known vesicoureteral reflux (VUR)]
No

Are there situations or clinical findings in the patient where you deviate from your prophylaxis standard (medication, dose, duration, age of the patient)? (multiple answers possible, please specify in answer box) [in case of already known vesicoureteral reflux (VUR)][Comment]

Are there situations or clinical findings in the patient where you deviate from your prophylaxis standard (medication, dose, duration, age of the patient)? (multiple answers possible, please specify in answer box) [in patients with recurrent urinary tract infections (UTI)]
No

Are there situations or clinical findings in the patient where you deviate from your prophylaxis standard (medication, dose, duration, age of the patient)? (multiple answers possible, please specify in answer box) [in patients with recurrent urinary tract infections (UTI)][Comment]

Are there situations or clinical findings in the patient where you deviate from your prophylaxis standard (medication, dose, duration, age of the patient)? (multiple answers possible, please specify in answer box) [if multidrug-resistant organisms (MDRO) are detected in urine cultures]
Yes

Are there situations or clinical findings in the patient where you deviate from your prophylaxis standard (medication, dose, duration, age of the patient)? (multiple answers possible, please specify in answer box) [if multidrug-resistant organisms (MDRO) are detected in urine cultures][Comment]
ggf. Verwendung eines nicht typischen Prophylaxemedikamentes

Are there situations or clinical findings in the patient where you deviate from your prophylaxis standard (medication, dose, duration, age of the patient)? (multiple answers possible, please specify in answer box) [in newborns]
No

Are there situations or clinical findings in the patient where you deviate from your prophylaxis standard (medication, dose, duration, age of the patient)? (multiple answers possible, please specify in answer box) [in newborns][Comment]

Are there situations or clinical findings in the patient where you deviate from your prophylaxis standard (medication, dose, duration, age of the patient)? (multiple answers possible, please specify in answer box) [Other]

Are there situations or clinical findings in the patient where you deviate from your prophylaxis standard (medication, dose, duration, age of the patient)? (multiple answers possible, please specify in answer box) [Other comment]

Are there situations in which you use intravenous prophylaxis?
No

Voiding analysis

Are there differences in antibiotic prophylaxis between VUS and VCUG in your department?
No

Is a non-pathological urine strip test required in preparation for VUS/VCUG in your department?
Yes

What is the maximum amount of days accepted for urine strip test to be performed prior to the exam?
Am Untersuchungstag

Is a non-pathological urine culture required in preparation for VUS/VCUG in your department?
No

Do patients only receive post-procedure prophylaxis if vesicoureteral reflux (VUR) is detected in VUS/VCUG?
No

Do other MUS/MCU findings (e.g. urethral valves) have an influence on post-procedure prophylaxis?
No

End of survey

Thank you for taking the time to answer this survey!

May we contact you personally if we have further questions?
Yes

E-mail address:
marie-therese.hansen@med.uni-goettingen.de

Survey response 134

Response ID
136

Date submitted
2025-03-07 21:02:12

Last page
5

Start language
de

Seed
540597141

Date started
2025-03-07 20:57:14

Date last action
2025-03-07 21:02:12

Standard Operation Procedure

Who fills out this survey?
Pediatric urologist

Who fills out this survey? [Other]

In which type of hospital are you employed?
Community hospital (>50 beds for pediatric patients)

In which type of hospital are you employed? [Other]

In which country do you work?
Deutschland

Does your department have a defined protocol for infection prophylaxis in the context of VUS/VCUG?
No

Has this protocol been transformed into a written standard operating procedure (SOP)?
N/A

Antibiotic Prophylaxis

Are there situations/constellations in which you do not use antibiotic prophylaxis for VUS/VCUG?
Yes

Please specify these situations/constellations: [girl with first uncomplicated UTI without urinary tract dilation in ultrasound]
No

Please specify these situations/constellations: [boy with first uncomplicated UTI without urinary tract dilation in ultrasound]
No

Please specify these situations/constellations: [in patients with low grade urinary tract dilation (up to 2nd grade) and no UTI in medical history]
No

Please specify these situations/constellations: [Other]
Kinder >1J, im Rahmen neurourologischer Diagnostik (ohne Infekte)

If you use antibiotic prophylaxis: which mediaction do you use? (multiple answers and subdivision into age groups possible) [Trimethoprim]
Yes

If you use antibiotic prophylaxis: which mediaction do you use? (multiple answers and subdivision into age groups possible) [Nitrofurantoin]
No

If you use antibiotic prophylaxis: which mediaction do you use? (multiple answers and subdivision into age groups possible) [Amoxicillin]
No

If you use antibiotic prophylaxis: which mediaction do you use? (multiple answers and subdivision into age groups possible) [Cefaclor]
Yes

If you use antibiotic prophylaxis: which mediaction do you use? (multiple answers and subdivision into age groups possible) [Amoxicillin/clavulanic acid]
No

If you use antibiotic prophylaxis: which mediaction do you use? (multiple answers and subdivision into age groups possible) [Other]
No

Trimethoprim: in what dosage?
prophylactic (2 mg/kg bodyweight/d)

Trimethoprim: in what dosage? [Other]

Trimethoprim: for which age group do you use trimethoprim?
>6 Wochen

Trimethoprim: which time schedule do you use? [Day(s) before VUS/VCUG, how many?]
No

Trimethoprim: which time schedule do you use? [Day(s) before VUS/VCUG, how many?][Comment]

Trimethoprim: which time schedule do you use? [Day of VUS/VCUG]
Yes

Trimethoprim: which time schedule do you use? [Day of VUS/VCUG][Comment]

Trimethoprim: which time schedule do you use? [Day(s) after VUS/VCUG, how many?]
Yes

Trimethoprim: which time schedule do you use? [Day(s) after VUS/VCUG, how many?][Comment]
2

Nitrofurantoin: which time schedule do you use? [Day(s) before VUS/VCUG, how many?]
N/A

Nitrofurantoin: which time schedule do you use? [Day of VUS/VCUG]
N/A

Nitrofurantoin: which time schedule do you use? [Day(s) after VUS/VCUG, how many?]
N/A

Amoxicillin: which time schedule do you use? [Day(s) before VUS/VCUG, how many?]
N/A

Amoxicillin: which time schedule do you use? [Day of VUS/VCUG]
N/A

Amoxicillin: which time schedule do you use? [Day(s) after VUS/VCUG, how many?]
N/A

Cefaclor: in what dosage?
prophylactic (10 mg/kg bodyweight/d)

Cefaclor: in what dosage? [Other]

Cefaclor: for which age group do you use cefaclor?
< 6Wochen

Cefaclor: which time schedule do you use? [Days(s) before VUS/VCUG, how many?]
No

Cefaclor: which time schedule do you use? [Days(s) before VUS/VCUG, how many?][Comment]

Cefaclor: which time schedule do you use? [Day of VUS/VCUG]
Yes

Cefaclor: which time schedule do you use? [Day of VUS/VCUG][Comment]

Cefaclor: which time schedule do you use? [Day(s) after VUS/VCUG, how many?]
Yes

Cefaclor: which time schedule do you use? [Day(s) after VUS/VCUG, how many?][Comment]
2

Amoxicillin/clavulanic acid: in what dosage? (Amoxicillin portion) [prophylaktic (10-20 mg/Kg bodyweight/d)]
N/A

Amoxicillin/clavulanic acid: in what dosage? (Amoxicillin portion) [double prophylactic (20-40 mg/Kg bodyweight/d)]
N/A

Amoxicillin/clavulanic acid: in what dosage? (Amoxicillin portion) [therapeutic (80-100 mg/Kg bodyweight/d)]
N/A

Amoxicillin/clavulanic acid: which time schedule do you use? [Day(s) before VUS/VCUG]
N/A

Amoxicillin/clavulanic acid: which time schedule do you use? [Day of VUS/VCUG]
N/A

Amoxicillin/clavulanic acid: which time schedule do you use? [Day(s) after VUS/VCUG]
N/A

Other: which time schedule do you use? [Day(s) before VUS/VCUG, how many?]
N/A

Other: which time schedule do you use? [Day of VUS/VCUG]
N/A

Other: which time schedule do you use? [Day(s) after VUS/VCUG, how many?]
N/A

Indication for prophylaxis

Which patients receive periinterventional antibiotic prophylaxis? (multiple answers possible) [all]
No

Which patients receive periinterventional antibiotic prophylaxis? (multiple answers possible) [patients post urinary tract infection/pyelonephritis/urosepsis]
Yes

Which patients receive periinterventional antibiotic prophylaxis? (multiple answers possible) [patients with urinary tract dilation in ultrasound]
No

Which patients receive periinterventional antibiotic prophylaxis? (multiple answers possible) [patients with prenatal urinary tract dilation]
No

Which patients receive periinterventional antibiotic prophylaxis? (multiple answers possible) [patients post VUR surgery]
No

Which patients receive periinterventional antibiotic prophylaxis? (multiple answers possible) [none]
No

Which patients receive periinterventional antibiotic prophylaxis? (multiple answers possible) [Other]
< 1J

patients status post urinary tract infection/pyelonephritis/urosepsis: what is the minimum time between infection and VUS/VCUG? (in days)
mind. 48h Fieberfrei und unter resistogrammgerechter Antibiose

Are there situations or clinical findings in the patient where you deviate from your prophylaxis standard (medication, dose, duration, age of the patient)? (multiple answers possible, please specify in answer box) [in case of already known vesicoureteral reflux (VUR)]
No

Are there situations or clinical findings in the patient where you deviate from your prophylaxis standard (medication, dose, duration, age of the patient)? (multiple answers possible, please specify in answer box) [in case of already known vesicoureteral reflux (VUR)][Comment]

Are there situations or clinical findings in the patient where you deviate from your prophylaxis standard (medication, dose, duration, age of the patient)? (multiple answers possible, please specify in answer box) [in patients with recurrent urinary tract infections (UTI)]
No

Are there situations or clinical findings in the patient where you deviate from your prophylaxis standard (medication, dose, duration, age of the patient)? (multiple answers possible, please specify in answer box) [in patients with recurrent urinary tract infections (UTI)][Comment]

Are there situations or clinical findings in the patient where you deviate from your prophylaxis standard (medication, dose, duration, age of the patient)? (multiple answers possible, please specify in answer box) [if multidrug-resistant organisms (MDRO) are detected in urine cultures]
Yes

Are there situations or clinical findings in the patient where you deviate from your prophylaxis standard (medication, dose, duration, age of the patient)? (multiple answers possible, please specify in answer box) [if multidrug-resistant organisms (MDRO) are detected in urine cultures][Comment]

Are there situations or clinical findings in the patient where you deviate from your prophylaxis standard (medication, dose, duration, age of the patient)? (multiple answers possible, please specify in answer box) [in newborns]
No

Are there situations or clinical findings in the patient where you deviate from your prophylaxis standard (medication, dose, duration, age of the patient)? (multiple answers possible, please specify in answer box) [in newborns][Comment]

Are there situations or clinical findings in the patient where you deviate from your prophylaxis standard (medication, dose, duration, age of the patient)? (multiple answers possible, please specify in answer box) [Other]

Are there situations or clinical findings in the patient where you deviate from your prophylaxis standard (medication, dose, duration, age of the patient)? (multiple answers possible, please specify in answer box) [Other comment]

Are there situations in which you use intravenous prophylaxis?
Yes

In which situations?
Unter i.v.-infekttherapie

Voiding analysis

Are there differences in antibiotic prophylaxis between VUS and VCUG in your department?
No

Is a non-pathological urine strip test required in preparation for VUS/VCUG in your department?
No

Is a non-pathological urine culture required in preparation for VUS/VCUG in your department?
No

Do patients only receive post-procedure prophylaxis if vesicoureteral reflux (VUR) is detected in VUS/VCUG?
No

Do other MUS/MCU findings (e.g. urethral valves) have an influence on post-procedure prophylaxis?
No

End of survey

Thank you for taking the time to answer this survey!

May we contact you personally if we have further questions?
No

Survey response 135

Response ID
137

Date submitted
2025-03-10 16:55:15

Last page
5

Start language
de

Seed
1635930022

Date started
2025-03-10 16:50:16

Date last action
2025-03-10 16:55:15

Standard Operation Procedure

Who fills out this survey?
Pediatric Surgeon

Who fills out this survey? [Other]

In which type of hospital are you employed?
Community hospital (>50 beds for pediatric patients)

In which type of hospital are you employed? [Other]

In which country do you work?
Deutschland

Does your department have a defined protocol for infection prophylaxis in the context of VUS/VCUG?
No

Has this protocol been transformed into a written standard operating procedure (SOP)?
N/A

Antibiotic Prophylaxis

Are there situations/constellations in which you do not use antibiotic prophylaxis for VUS/VCUG?
No

Please specify these situations/constellations: [girl with first uncomplicated UTI without urinary tract dilation in ultrasound]
N/A

Please specify these situations/constellations: [boy with first uncomplicated UTI without urinary tract dilation in ultrasound]
N/A

Please specify these situations/constellations: [in patients with low grade urinary tract dilation (up to 2nd grade) and no UTI in medical history]
N/A

If you use antibiotic prophylaxis: which mediaction do you use? (multiple answers and subdivision into age groups possible) [Trimethoprim]
Yes

If you use antibiotic prophylaxis: which mediaction do you use? (multiple answers and subdivision into age groups possible) [Nitrofurantoin]
Yes

If you use antibiotic prophylaxis: which mediaction do you use? (multiple answers and subdivision into age groups possible) [Amoxicillin]
No

If you use antibiotic prophylaxis: which mediaction do you use? (multiple answers and subdivision into age groups possible) [Cefaclor]
Yes

If you use antibiotic prophylaxis: which mediaction do you use? (multiple answers and subdivision into age groups possible) [Amoxicillin/clavulanic acid]
No

If you use antibiotic prophylaxis: which mediaction do you use? (multiple answers and subdivision into age groups possible) [Other]
No

Trimethoprim: in what dosage?
double prophylakctic (4 mg/kg bodyweight/d)

Trimethoprim: in what dosage? [Other]

Trimethoprim: for which age group do you use trimethoprim?
ab drittem Lebensmonat

Trimethoprim: which time schedule do you use? [Day(s) before VUS/VCUG, how many?]
No

Trimethoprim: which time schedule do you use? [Day(s) before VUS/VCUG, how many?][Comment]

Trimethoprim: which time schedule do you use? [Day of VUS/VCUG]
Yes

Trimethoprim: which time schedule do you use? [Day of VUS/VCUG][Comment]

Trimethoprim: which time schedule do you use? [Day(s) after VUS/VCUG, how many?]
Yes

Trimethoprim: which time schedule do you use? [Day(s) after VUS/VCUG, how many?][Comment]
2

Nitrofurantoin: in what dosage?
double prophylactic (2-4 mg/kg bodyweight/d)

Nitrofurantoin: in what dosage? [Other]

Nitrofurantoin: for which age group do you use nitrofurantoin?
ab drittem Lebensmonat

Nitrofurantoin: which time schedule do you use? [Day(s) before VUS/VCUG, how many?]
No

Nitrofurantoin: which time schedule do you use? [Day(s) before VUS/VCUG, how many?][Comment]

Nitrofurantoin: which time schedule do you use? [Day of VUS/VCUG]
Yes

Nitrofurantoin: which time schedule do you use? [Day of VUS/VCUG][Comment]

Nitrofurantoin: which time schedule do you use? [Day(s) after VUS/VCUG, how many?]
Yes

Nitrofurantoin: which time schedule do you use? [Day(s) after VUS/VCUG, how many?][Comment]
2

Amoxicillin: which time schedule do you use? [Day(s) before VUS/VCUG, how many?]
N/A

Amoxicillin: which time schedule do you use? [Day of VUS/VCUG]
N/A

Amoxicillin: which time schedule do you use? [Day(s) after VUS/VCUG, how many?]
N/A

Cefaclor: in what dosage?
double prophylactic (20 mg/kg bodyweight/d)

Cefaclor: in what dosage? [Other]

Cefaclor: for which age group do you use cefaclor?
ab Neugeboren

Cefaclor: which time schedule do you use? [Days(s) before VUS/VCUG, how many?]
No

Cefaclor: which time schedule do you use? [Days(s) before VUS/VCUG, how many?][Comment]

Cefaclor: which time schedule do you use? [Day of VUS/VCUG]
Yes

Cefaclor: which time schedule do you use? [Day of VUS/VCUG][Comment]

Cefaclor: which time schedule do you use? [Day(s) after VUS/VCUG, how many?]
Yes

Cefaclor: which time schedule do you use? [Day(s) after VUS/VCUG, how many?][Comment]
2

Amoxicillin/clavulanic acid: in what dosage? (Amoxicillin portion) [prophylaktic (10-20 mg/Kg bodyweight/d)]
N/A

Amoxicillin/clavulanic acid: in what dosage? (Amoxicillin portion) [double prophylactic (20-40 mg/Kg bodyweight/d)]
N/A

Amoxicillin/clavulanic acid: in what dosage? (Amoxicillin portion) [therapeutic (80-100 mg/Kg bodyweight/d)]
N/A

Amoxicillin/clavulanic acid: which time schedule do you use? [Day(s) before VUS/VCUG]
N/A

Amoxicillin/clavulanic acid: which time schedule do you use? [Day of VUS/VCUG]
N/A

Amoxicillin/clavulanic acid: which time schedule do you use? [Day(s) after VUS/VCUG]
N/A

Other: which time schedule do you use? [Day(s) before VUS/VCUG, how many?]
N/A

Other: which time schedule do you use? [Day of VUS/VCUG]
N/A

Other: which time schedule do you use? [Day(s) after VUS/VCUG, how many?]
N/A

Indication for prophylaxis

Which patients receive periinterventional antibiotic prophylaxis? (multiple answers possible) [all]
Yes

Which patients receive periinterventional antibiotic prophylaxis? (multiple answers possible) [patients post urinary tract infection/pyelonephritis/urosepsis]
N/A

Which patients receive periinterventional antibiotic prophylaxis? (multiple answers possible) [patients with urinary tract dilation in ultrasound]
N/A

Which patients receive periinterventional antibiotic prophylaxis? (multiple answers possible) [patients with prenatal urinary tract dilation]
N/A

Which patients receive periinterventional antibiotic prophylaxis? (multiple answers possible) [patients post VUR surgery]
N/A

Which patients receive periinterventional antibiotic prophylaxis? (multiple answers possible) [none]
N/A

Are there situations or clinical findings in the patient where you deviate from your prophylaxis standard (medication, dose, duration, age of the patient)? (multiple answers possible, please specify in answer box) [in case of already known vesicoureteral reflux (VUR)]
No

Are there situations or clinical findings in the patient where you deviate from your prophylaxis standard (medication, dose, duration, age of the patient)? (multiple answers possible, please specify in answer box) [in case of already known vesicoureteral reflux (VUR)][Comment]

Are there situations or clinical findings in the patient where you deviate from your prophylaxis standard (medication, dose, duration, age of the patient)? (multiple answers possible, please specify in answer box) [in patients with recurrent urinary tract infections (UTI)]
No

Are there situations or clinical findings in the patient where you deviate from your prophylaxis standard (medication, dose, duration, age of the patient)? (multiple answers possible, please specify in answer box) [in patients with recurrent urinary tract infections (UTI)][Comment]

Are there situations or clinical findings in the patient where you deviate from your prophylaxis standard (medication, dose, duration, age of the patient)? (multiple answers possible, please specify in answer box) [if multidrug-resistant organisms (MDRO) are detected in urine cultures]
Yes

Are there situations or clinical findings in the patient where you deviate from your prophylaxis standard (medication, dose, duration, age of the patient)? (multiple answers possible, please specify in answer box) [if multidrug-resistant organisms (MDRO) are detected in urine cultures][Comment]
Anpassung an Antibiogramm

Are there situations or clinical findings in the patient where you deviate from your prophylaxis standard (medication, dose, duration, age of the patient)? (multiple answers possible, please specify in answer box) [in newborns]
No

Are there situations or clinical findings in the patient where you deviate from your prophylaxis standard (medication, dose, duration, age of the patient)? (multiple answers possible, please specify in answer box) [in newborns][Comment]

Are there situations or clinical findings in the patient where you deviate from your prophylaxis standard (medication, dose, duration, age of the patient)? (multiple answers possible, please specify in answer box) [Other]

Are there situations or clinical findings in the patient where you deviate from your prophylaxis standard (medication, dose, duration, age of the patient)? (multiple answers possible, please specify in answer box) [Other comment]

Are there situations in which you use intravenous prophylaxis?
No

Voiding analysis

Are there differences in antibiotic prophylaxis between VUS and VCUG in your department?
No

Is a non-pathological urine strip test required in preparation for VUS/VCUG in your department?
No

Is a non-pathological urine culture required in preparation for VUS/VCUG in your department?
No

Do patients only receive post-procedure prophylaxis if vesicoureteral reflux (VUR) is detected in VUS/VCUG?
No

Do other MUS/MCU findings (e.g. urethral valves) have an influence on post-procedure prophylaxis?
No

End of survey

Thank you for taking the time to answer this survey!

May we contact you personally if we have further questions?
No

Survey response 136

Response ID
138

Last page
1

Start language
en

Seed
1120750491

Date started
2025-03-12 07:28:52

Date last action
2025-03-12 07:39:19

Standard Operation Procedure

Who fills out this survey?
Pediatric Radiologist

Who fills out this survey? [Other]

In which type of hospital are you employed?
University Hospital

In which type of hospital are you employed? [Other]

In which country do you work?
Belgium

Does your department have a defined protocol for infection prophylaxis in the context of VUS/VCUG?
Yes

Has this protocol been transformed into a written standard operating procedure (SOP)?
Yes

Which department is the author of the SOP for infection prophylaxis in VUS/VCUG?
Pediatric nephrology

Which department is the author of the SOP for infection prophylaxis in VUS/VCUG? [Other]

Antibiotic Prophylaxis

Are there situations/constellations in which you do not use antibiotic prophylaxis for VUS/VCUG?
N/A

Please specify these situations/constellations: [girl with first uncomplicated UTI without urinary tract dilation in ultrasound]
N/A

Please specify these situations/constellations: [boy with first uncomplicated UTI without urinary tract dilation in ultrasound]
N/A

Please specify these situations/constellations: [in patients with low grade urinary tract dilation (up to 2nd grade) and no UTI in medical history]
N/A

If you use antibiotic prophylaxis: which mediaction do you use? (multiple answers and subdivision into age groups possible) [Trimethoprim]
N/A

If you use antibiotic prophylaxis: which mediaction do you use? (multiple answers and subdivision into age groups possible) [Nitrofurantoin]
N/A

If you use antibiotic prophylaxis: which mediaction do you use? (multiple answers and subdivision into age groups possible) [Amoxicillin]
N/A

If you use antibiotic prophylaxis: which mediaction do you use? (multiple answers and subdivision into age groups possible) [Cefaclor]
N/A

If you use antibiotic prophylaxis: which mediaction do you use? (multiple answers and subdivision into age groups possible) [Amoxicillin/clavulanic acid]
N/A

If you use antibiotic prophylaxis: which mediaction do you use? (multiple answers and subdivision into age groups possible) [Other]
N/A

Trimethoprim: which time schedule do you use? [Day(s) before VUS/VCUG, how many?]
N/A

Trimethoprim: which time schedule do you use? [Day of VUS/VCUG]
N/A

Trimethoprim: which time schedule do you use? [Day(s) after VUS/VCUG, how many?]
N/A

Nitrofurantoin: which time schedule do you use? [Day(s) before VUS/VCUG, how many?]
N/A

Nitrofurantoin: which time schedule do you use? [Day of VUS/VCUG]
N/A

Nitrofurantoin: which time schedule do you use? [Day(s) after VUS/VCUG, how many?]
N/A

Amoxicillin: which time schedule do you use? [Day(s) before VUS/VCUG, how many?]
N/A

Amoxicillin: which time schedule do you use? [Day of VUS/VCUG]
N/A

Amoxicillin: which time schedule do you use? [Day(s) after VUS/VCUG, how many?]
N/A

Cefaclor: which time schedule do you use? [Days(s) before VUS/VCUG, how many?]
N/A

Cefaclor: which time schedule do you use? [Day of VUS/VCUG]
N/A

Cefaclor: which time schedule do you use? [Day(s) after VUS/VCUG, how many?]
N/A

Amoxicillin/clavulanic acid: in what dosage? (Amoxicillin portion) [prophylaktic (10-20 mg/Kg bodyweight/d)]
N/A

Amoxicillin/clavulanic acid: in what dosage? (Amoxicillin portion) [double prophylactic (20-40 mg/Kg bodyweight/d)]
N/A

Amoxicillin/clavulanic acid: in what dosage? (Amoxicillin portion) [therapeutic (80-100 mg/Kg bodyweight/d)]
N/A

Amoxicillin/clavulanic acid: which time schedule do you use? [Day(s) before VUS/VCUG]
N/A

Amoxicillin/clavulanic acid: which time schedule do you use? [Day of VUS/VCUG]
N/A

Amoxicillin/clavulanic acid: which time schedule do you use? [Day(s) after VUS/VCUG]
N/A

Other: which time schedule do you use? [Day(s) before VUS/VCUG, how many?]
N/A

Other: which time schedule do you use? [Day of VUS/VCUG]
N/A

Other: which time schedule do you use? [Day(s) after VUS/VCUG, how many?]
N/A

Indication for prophylaxis

Which patients receive periinterventional antibiotic prophylaxis? (multiple answers possible) [all]
N/A

Which patients receive periinterventional antibiotic prophylaxis? (multiple answers possible) [patients post urinary tract infection/pyelonephritis/urosepsis]
N/A

Which patients receive periinterventional antibiotic prophylaxis? (multiple answers possible) [patients with urinary tract dilation in ultrasound]
N/A

Which patients receive periinterventional antibiotic prophylaxis? (multiple answers possible) [patients with prenatal urinary tract dilation]
N/A

Which patients receive periinterventional antibiotic prophylaxis? (multiple answers possible) [patients post VUR surgery]
N/A

Which patients receive periinterventional antibiotic prophylaxis? (multiple answers possible) [none]
N/A

Are there situations or clinical findings in the patient where you deviate from your prophylaxis standard (medication, dose, duration, age of the patient)? (multiple answers possible, please specify in answer box) [in case of already known vesicoureteral reflux (VUR)]
N/A

Are there situations or clinical findings in the patient where you deviate from your prophylaxis standard (medication, dose, duration, age of the patient)? (multiple answers possible, please specify in answer box) [in patients with recurrent urinary tract infections (UTI)]
N/A

Are there situations or clinical findings in the patient where you deviate from your prophylaxis standard (medication, dose, duration, age of the patient)? (multiple answers possible, please specify in answer box) [if multidrug-resistant organisms (MDRO) are detected in urine cultures]
N/A

Are there situations or clinical findings in the patient where you deviate from your prophylaxis standard (medication, dose, duration, age of the patient)? (multiple answers possible, please specify in answer box) [in newborns]
N/A

Are there situations in which you use intravenous prophylaxis?
N/A

Voiding analysis

Are there differences in antibiotic prophylaxis between VUS and VCUG in your department?
N/A

Is a non-pathological urine strip test required in preparation for VUS/VCUG in your department?
N/A

Is a non-pathological urine culture required in preparation for VUS/VCUG in your department?
N/A

Do patients only receive post-procedure prophylaxis if vesicoureteral reflux (VUR) is detected in VUS/VCUG?
N/A

Do other MUS/MCU findings (e.g. urethral valves) have an influence on post-procedure prophylaxis?
N/A

End of survey

May we contact you personally if we have further questions?
N/A

Survey response 137

Response ID
139

Last page
1

Start language
en

Seed
1891908094

Date started
2025-03-12 15:43:04

Date last action
2025-03-12 15:43:33

Standard Operation Procedure

Who fills out this survey?
Pediatric Radiologist

Who fills out this survey? [Other]

In which type of hospital are you employed?
University Hospital

In which type of hospital are you employed? [Other]

In which country do you work?
Belgium

Does your department have a defined protocol for infection prophylaxis in the context of VUS/VCUG?
Yes

Has this protocol been transformed into a written standard operating procedure (SOP)?
No

Antibiotic Prophylaxis

Are there situations/constellations in which you do not use antibiotic prophylaxis for VUS/VCUG?
No

Please specify these situations/constellations: [girl with first uncomplicated UTI without urinary tract dilation in ultrasound]
N/A

Please specify these situations/constellations: [boy with first uncomplicated UTI without urinary tract dilation in ultrasound]
N/A

Please specify these situations/constellations: [in patients with low grade urinary tract dilation (up to 2nd grade) and no UTI in medical history]
N/A

If you use antibiotic prophylaxis: which mediaction do you use? (multiple answers and subdivision into age groups possible) [Trimethoprim]
No

If you use antibiotic prophylaxis: which mediaction do you use? (multiple answers and subdivision into age groups possible) [Nitrofurantoin]
No

If you use antibiotic prophylaxis: which mediaction do you use? (multiple answers and subdivision into age groups possible) [Amoxicillin]
No

If you use antibiotic prophylaxis: which mediaction do you use? (multiple answers and subdivision into age groups possible) [Cefaclor]
Yes

If you use antibiotic prophylaxis: which mediaction do you use? (multiple answers and subdivision into age groups possible) [Amoxicillin/clavulanic acid]
No

If you use antibiotic prophylaxis: which mediaction do you use? (multiple answers and subdivision into age groups possible) [Other]
No

Trimethoprim: which time schedule do you use? [Day(s) before VUS/VCUG, how many?]
N/A

Trimethoprim: which time schedule do you use? [Day of VUS/VCUG]
N/A

Trimethoprim: which time schedule do you use? [Day(s) after VUS/VCUG, how many?]
N/A

Nitrofurantoin: which time schedule do you use? [Day(s) before VUS/VCUG, how many?]
N/A

Nitrofurantoin: which time schedule do you use? [Day of VUS/VCUG]
N/A

Nitrofurantoin: which time schedule do you use? [Day(s) after VUS/VCUG, how many?]
N/A

Amoxicillin: which time schedule do you use? [Day(s) before VUS/VCUG, how many?]
N/A

Amoxicillin: which time schedule do you use? [Day of VUS/VCUG]
N/A

Amoxicillin: which time schedule do you use? [Day(s) after VUS/VCUG, how many?]
N/A

Cefaclor: in what dosage?
prophylactic (10 mg/kg bodyweight/d)

Cefaclor: in what dosage? [Other]

Cefaclor: for which age group do you use cefaclor?

Cefaclor: which time schedule do you use? [Days(s) before VUS/VCUG, how many?]
Yes

Cefaclor: which time schedule do you use? [Days(s) before VUS/VCUG, how many?][Comment]
1

Cefaclor: which time schedule do you use? [Day of VUS/VCUG]
Yes

Cefaclor: which time schedule do you use? [Day of VUS/VCUG][Comment]

Cefaclor: which time schedule do you use? [Day(s) after VUS/VCUG, how many?]
Yes

Cefaclor: which time schedule do you use? [Day(s) after VUS/VCUG, how many?][Comment]
1

Amoxicillin/clavulanic acid: in what dosage? (Amoxicillin portion) [prophylaktic (10-20 mg/Kg bodyweight/d)]
N/A

Amoxicillin/clavulanic acid: in what dosage? (Amoxicillin portion) [double prophylactic (20-40 mg/Kg bodyweight/d)]
N/A

Amoxicillin/clavulanic acid: in what dosage? (Amoxicillin portion) [therapeutic (80-100 mg/Kg bodyweight/d)]
N/A

Amoxicillin/clavulanic acid: which time schedule do you use? [Day(s) before VUS/VCUG]
N/A

Amoxicillin/clavulanic acid: which time schedule do you use? [Day of VUS/VCUG]
N/A

Amoxicillin/clavulanic acid: which time schedule do you use? [Day(s) after VUS/VCUG]
N/A

Other: which time schedule do you use? [Day(s) before VUS/VCUG, how many?]
N/A

Other: which time schedule do you use? [Day of VUS/VCUG]
N/A

Other: which time schedule do you use? [Day(s) after VUS/VCUG, how many?]
N/A

Indication for prophylaxis

Which patients receive periinterventional antibiotic prophylaxis? (multiple answers possible) [all]
N/A

Which patients receive periinterventional antibiotic prophylaxis? (multiple answers possible) [patients post urinary tract infection/pyelonephritis/urosepsis]
N/A

Which patients receive periinterventional antibiotic prophylaxis? (multiple answers possible) [patients with urinary tract dilation in ultrasound]
N/A

Which patients receive periinterventional antibiotic prophylaxis? (multiple answers possible) [patients with prenatal urinary tract dilation]
N/A

Which patients receive periinterventional antibiotic prophylaxis? (multiple answers possible) [patients post VUR surgery]
N/A

Which patients receive periinterventional antibiotic prophylaxis? (multiple answers possible) [none]
N/A

Are there situations or clinical findings in the patient where you deviate from your prophylaxis standard (medication, dose, duration, age of the patient)? (multiple answers possible, please specify in answer box) [in case of already known vesicoureteral reflux (VUR)]
N/A

Are there situations or clinical findings in the patient where you deviate from your prophylaxis standard (medication, dose, duration, age of the patient)? (multiple answers possible, please specify in answer box) [in patients with recurrent urinary tract infections (UTI)]
N/A

Are there situations or clinical findings in the patient where you deviate from your prophylaxis standard (medication, dose, duration, age of the patient)? (multiple answers possible, please specify in answer box) [if multidrug-resistant organisms (MDRO) are detected in urine cultures]
N/A

Are there situations or clinical findings in the patient where you deviate from your prophylaxis standard (medication, dose, duration, age of the patient)? (multiple answers possible, please specify in answer box) [in newborns]
N/A

Are there situations in which you use intravenous prophylaxis?
N/A

Voiding analysis

Are there differences in antibiotic prophylaxis between VUS and VCUG in your department?
N/A

Is a non-pathological urine strip test required in preparation for VUS/VCUG in your department?
N/A

Is a non-pathological urine culture required in preparation for VUS/VCUG in your department?
N/A

Do patients only receive post-procedure prophylaxis if vesicoureteral reflux (VUR) is detected in VUS/VCUG?
N/A

Do other MUS/MCU findings (e.g. urethral valves) have an influence on post-procedure prophylaxis?
N/A

End of survey

May we contact you personally if we have further questions?
N/A

Survey response 138

Response ID
140

Date submitted
2025-03-13 09:44:12

Last page
5

Start language
de

Seed
524158816

Date started
2025-03-13 09:33:06

Date last action
2025-03-13 09:44:12

Standard Operation Procedure

Who fills out this survey?
Pediatric Radiologist

Who fills out this survey? [Other]

In which type of hospital are you employed?
Community hospital (>50 beds for pediatric patients)

In which type of hospital are you employed? [Other]

In which country do you work?
Deutschland

Does your department have a defined protocol for infection prophylaxis in the context of VUS/VCUG?
Yes

Has this protocol been transformed into a written standard operating procedure (SOP)?
Yes

Which department is the author of the SOP for infection prophylaxis in VUS/VCUG?
Pediatric surgery

Which department is the author of the SOP for infection prophylaxis in VUS/VCUG? [Other]

Antibiotic Prophylaxis

Are there situations/constellations in which you do not use antibiotic prophylaxis for VUS/VCUG?
No

Please specify these situations/constellations: [girl with first uncomplicated UTI without urinary tract dilation in ultrasound]
N/A

Please specify these situations/constellations: [boy with first uncomplicated UTI without urinary tract dilation in ultrasound]
N/A

Please specify these situations/constellations: [in patients with low grade urinary tract dilation (up to 2nd grade) and no UTI in medical history]
N/A

If you use antibiotic prophylaxis: which mediaction do you use? (multiple answers and subdivision into age groups possible) [Trimethoprim]
No

If you use antibiotic prophylaxis: which mediaction do you use? (multiple answers and subdivision into age groups possible) [Nitrofurantoin]
No

If you use antibiotic prophylaxis: which mediaction do you use? (multiple answers and subdivision into age groups possible) [Amoxicillin]
No

If you use antibiotic prophylaxis: which mediaction do you use? (multiple answers and subdivision into age groups possible) [Cefaclor]
Yes

If you use antibiotic prophylaxis: which mediaction do you use? (multiple answers and subdivision into age groups possible) [Amoxicillin/clavulanic acid]
No

If you use antibiotic prophylaxis: which mediaction do you use? (multiple answers and subdivision into age groups possible) [Other]
No

Trimethoprim: which time schedule do you use? [Day(s) before VUS/VCUG, how many?]
N/A

Trimethoprim: which time schedule do you use? [Day of VUS/VCUG]
N/A

Trimethoprim: which time schedule do you use? [Day(s) after VUS/VCUG, how many?]
N/A

Nitrofurantoin: which time schedule do you use? [Day(s) before VUS/VCUG, how many?]
N/A

Nitrofurantoin: which time schedule do you use? [Day of VUS/VCUG]
N/A

Nitrofurantoin: which time schedule do you use? [Day(s) after VUS/VCUG, how many?]
N/A

Amoxicillin: which time schedule do you use? [Day(s) before VUS/VCUG, how many?]
N/A

Amoxicillin: which time schedule do you use? [Day of VUS/VCUG]
N/A

Amoxicillin: which time schedule do you use? [Day(s) after VUS/VCUG, how many?]
N/A

Cefaclor: in what dosage?
double prophylactic (20 mg/kg bodyweight/d)

Cefaclor: in what dosage? [Other]

Cefaclor: for which age group do you use cefaclor?
1-18

Cefaclor: which time schedule do you use? [Days(s) before VUS/VCUG, how many?]
Yes

Cefaclor: which time schedule do you use? [Days(s) before VUS/VCUG, how many?][Comment]
1

Cefaclor: which time schedule do you use? [Day of VUS/VCUG]
Yes

Cefaclor: which time schedule do you use? [Day of VUS/VCUG][Comment]

Cefaclor: which time schedule do you use? [Day(s) after VUS/VCUG, how many?]
Yes

Cefaclor: which time schedule do you use? [Day(s) after VUS/VCUG, how many?][Comment]
1

Amoxicillin/clavulanic acid: in what dosage? (Amoxicillin portion) [prophylaktic (10-20 mg/Kg bodyweight/d)]
N/A

Amoxicillin/clavulanic acid: in what dosage? (Amoxicillin portion) [double prophylactic (20-40 mg/Kg bodyweight/d)]
N/A

Amoxicillin/clavulanic acid: in what dosage? (Amoxicillin portion) [therapeutic (80-100 mg/Kg bodyweight/d)]
N/A

Amoxicillin/clavulanic acid: which time schedule do you use? [Day(s) before VUS/VCUG]
N/A

Amoxicillin/clavulanic acid: which time schedule do you use? [Day of VUS/VCUG]
N/A

Amoxicillin/clavulanic acid: which time schedule do you use? [Day(s) after VUS/VCUG]
N/A

Other: which time schedule do you use? [Day(s) before VUS/VCUG, how many?]
N/A

Other: which time schedule do you use? [Day of VUS/VCUG]
N/A

Other: which time schedule do you use? [Day(s) after VUS/VCUG, how many?]
N/A

Indication for prophylaxis

Which patients receive periinterventional antibiotic prophylaxis? (multiple answers possible) [all]
Yes

Which patients receive periinterventional antibiotic prophylaxis? (multiple answers possible) [patients post urinary tract infection/pyelonephritis/urosepsis]
N/A

Which patients receive periinterventional antibiotic prophylaxis? (multiple answers possible) [patients with urinary tract dilation in ultrasound]
N/A

Which patients receive periinterventional antibiotic prophylaxis? (multiple answers possible) [patients with prenatal urinary tract dilation]
N/A

Which patients receive periinterventional antibiotic prophylaxis? (multiple answers possible) [patients post VUR surgery]
N/A

Which patients receive periinterventional antibiotic prophylaxis? (multiple answers possible) [none]
N/A

Are there situations or clinical findings in the patient where you deviate from your prophylaxis standard (medication, dose, duration, age of the patient)? (multiple answers possible, please specify in answer box) [in case of already known vesicoureteral reflux (VUR)]
Yes

Are there situations or clinical findings in the patient where you deviate from your prophylaxis standard (medication, dose, duration, age of the patient)? (multiple answers possible, please specify in answer box) [in case of already known vesicoureteral reflux (VUR)][Comment]

Are there situations or clinical findings in the patient where you deviate from your prophylaxis standard (medication, dose, duration, age of the patient)? (multiple answers possible, please specify in answer box) [in patients with recurrent urinary tract infections (UTI)]
No

Are there situations or clinical findings in the patient where you deviate from your prophylaxis standard (medication, dose, duration, age of the patient)? (multiple answers possible, please specify in answer box) [in patients with recurrent urinary tract infections (UTI)][Comment]

Are there situations or clinical findings in the patient where you deviate from your prophylaxis standard (medication, dose, duration, age of the patient)? (multiple answers possible, please specify in answer box) [if multidrug-resistant organisms (MDRO) are detected in urine cultures]
Yes

Are there situations or clinical findings in the patient where you deviate from your prophylaxis standard (medication, dose, duration, age of the patient)? (multiple answers possible, please specify in answer box) [if multidrug-resistant organisms (MDRO) are detected in urine cultures][Comment]

Are there situations or clinical findings in the patient where you deviate from your prophylaxis standard (medication, dose, duration, age of the patient)? (multiple answers possible, please specify in answer box) [in newborns]
No

Are there situations or clinical findings in the patient where you deviate from your prophylaxis standard (medication, dose, duration, age of the patient)? (multiple answers possible, please specify in answer box) [in newborns][Comment]

Are there situations or clinical findings in the patient where you deviate from your prophylaxis standard (medication, dose, duration, age of the patient)? (multiple answers possible, please specify in answer box) [Other]

Are there situations or clinical findings in the patient where you deviate from your prophylaxis standard (medication, dose, duration, age of the patient)? (multiple answers possible, please specify in answer box) [Other comment]

Are there situations in which you use intravenous prophylaxis?
No

Voiding analysis

Are there differences in antibiotic prophylaxis between VUS and VCUG in your department?
No

Is a non-pathological urine strip test required in preparation for VUS/VCUG in your department?
No

Is a non-pathological urine culture required in preparation for VUS/VCUG in your department?
No

Do patients only receive post-procedure prophylaxis if vesicoureteral reflux (VUR) is detected in VUS/VCUG?
No

Do other MUS/MCU findings (e.g. urethral valves) have an influence on post-procedure prophylaxis?
No

End of survey

Thank you for taking the time to answer this survey!

May we contact you personally if we have further questions?
No

Survey response 139

Response ID
141

Date submitted
2025-03-13 20:29:15

Last page
5

Start language
en

Seed
1155715708

Date started
2025-03-13 20:17:18

Date last action
2025-03-13 20:29:15

Standard Operation Procedure

Who fills out this survey?
Pediatric Radiologist

Who fills out this survey? [Other]

In which type of hospital are you employed?
University Hospital

In which type of hospital are you employed? [Other]

In which country do you work?
Portugal

Does your department have a defined protocol for infection prophylaxis in the context of VUS/VCUG?
Yes

Has this protocol been transformed into a written standard operating procedure (SOP)?
No

Antibiotic Prophylaxis

Are there situations/constellations in which you do not use antibiotic prophylaxis for VUS/VCUG?
Yes

Please specify these situations/constellations: [girl with first uncomplicated UTI without urinary tract dilation in ultrasound]
No

Please specify these situations/constellations: [boy with first uncomplicated UTI without urinary tract dilation in ultrasound]
No

Please specify these situations/constellations: [in patients with low grade urinary tract dilation (up to 2nd grade) and no UTI in medical history]
No

Please specify these situations/constellations: [Other]
older children without previous prophiylaxis for dilatation or infection

If you use antibiotic prophylaxis: which mediaction do you use? (multiple answers and subdivision into age groups possible) [Trimethoprim]
No

If you use antibiotic prophylaxis: which mediaction do you use? (multiple answers and subdivision into age groups possible) [Nitrofurantoin]
No

If you use antibiotic prophylaxis: which mediaction do you use? (multiple answers and subdivision into age groups possible) [Amoxicillin]
No

If you use antibiotic prophylaxis: which mediaction do you use? (multiple answers and subdivision into age groups possible) [Cefaclor]
No

If you use antibiotic prophylaxis: which mediaction do you use? (multiple answers and subdivision into age groups possible) [Amoxicillin/clavulanic acid]
No

If you use antibiotic prophylaxis: which mediaction do you use? (multiple answers and subdivision into age groups possible) [Other]
No

Trimethoprim: which time schedule do you use? [Day(s) before VUS/VCUG, how many?]
N/A

Trimethoprim: which time schedule do you use? [Day of VUS/VCUG]
N/A

Trimethoprim: which time schedule do you use? [Day(s) after VUS/VCUG, how many?]
N/A

Nitrofurantoin: which time schedule do you use? [Day(s) before VUS/VCUG, how many?]
N/A

Nitrofurantoin: which time schedule do you use? [Day of VUS/VCUG]
N/A

Nitrofurantoin: which time schedule do you use? [Day(s) after VUS/VCUG, how many?]
N/A

Amoxicillin: which time schedule do you use? [Day(s) before VUS/VCUG, how many?]
N/A

Amoxicillin: which time schedule do you use? [Day of VUS/VCUG]
N/A

Amoxicillin: which time schedule do you use? [Day(s) after VUS/VCUG, how many?]
N/A

Cefaclor: which time schedule do you use? [Days(s) before VUS/VCUG, how many?]
N/A

Cefaclor: which time schedule do you use? [Day of VUS/VCUG]
N/A

Cefaclor: which time schedule do you use? [Day(s) after VUS/VCUG, how many?]
N/A

Amoxicillin/clavulanic acid: in what dosage? (Amoxicillin portion) [prophylaktic (10-20 mg/Kg bodyweight/d)]
N/A

Amoxicillin/clavulanic acid: in what dosage? (Amoxicillin portion) [double prophylactic (20-40 mg/Kg bodyweight/d)]
N/A

Amoxicillin/clavulanic acid: in what dosage? (Amoxicillin portion) [therapeutic (80-100 mg/Kg bodyweight/d)]
N/A

Amoxicillin/clavulanic acid: which time schedule do you use? [Day(s) before VUS/VCUG]
N/A

Amoxicillin/clavulanic acid: which time schedule do you use? [Day of VUS/VCUG]
N/A

Amoxicillin/clavulanic acid: which time schedule do you use? [Day(s) after VUS/VCUG]
N/A

Other: which time schedule do you use? [Day(s) before VUS/VCUG, how many?]
N/A

Other: which time schedule do you use? [Day of VUS/VCUG]
N/A

Other: which time schedule do you use? [Day(s) after VUS/VCUG, how many?]
N/A

Indication for prophylaxis

Which patients receive periinterventional antibiotic prophylaxis? (multiple answers possible) [all]
No

Which patients receive periinterventional antibiotic prophylaxis? (multiple answers possible) [patients post urinary tract infection/pyelonephritis/urosepsis]
Yes

Which patients receive periinterventional antibiotic prophylaxis? (multiple answers possible) [patients with urinary tract dilation in ultrasound]
Yes

Which patients receive periinterventional antibiotic prophylaxis? (multiple answers possible) [patients with prenatal urinary tract dilation]
No

Which patients receive periinterventional antibiotic prophylaxis? (multiple answers possible) [patients post VUR surgery]
Yes

Which patients receive periinterventional antibiotic prophylaxis? (multiple answers possible) [none]
No

Which patients receive periinterventional antibiotic prophylaxis? (multiple answers possible) [Other]
according to the pediatric urologist or neprologist

patients status post urinary tract infection/pyelonephritis/urosepsis: what is the minimum time between infection and VUS/VCUG? (in days)
15 days

In patients with urinary tract dilation in ultrasound: from what degree on? Image from Beetz R, Bokenkamp A, Brandis M et al (2001) Diagnosis of congenital dilatation of the urinary tract. Consensus Group of the Pediatric Nephrology Working Society in cooperation with the Pediatric Urology Working Group of the German Society of Urology and with the Pediatric Urology Working Society in the Germany Society of Pediatric Surgery. Urologe A 40:495–507 (quiz 8–9)
grade 3

Are there situations or clinical findings in the patient where you deviate from your prophylaxis standard (medication, dose, duration, age of the patient)? (multiple answers possible, please specify in answer box) [in case of already known vesicoureteral reflux (VUR)]
No

Are there situations or clinical findings in the patient where you deviate from your prophylaxis standard (medication, dose, duration, age of the patient)? (multiple answers possible, please specify in answer box) [in case of already known vesicoureteral reflux (VUR)][Comment]

Are there situations or clinical findings in the patient where you deviate from your prophylaxis standard (medication, dose, duration, age of the patient)? (multiple answers possible, please specify in answer box) [in patients with recurrent urinary tract infections (UTI)]
No

Are there situations or clinical findings in the patient where you deviate from your prophylaxis standard (medication, dose, duration, age of the patient)? (multiple answers possible, please specify in answer box) [in patients with recurrent urinary tract infections (UTI)][Comment]

Are there situations or clinical findings in the patient where you deviate from your prophylaxis standard (medication, dose, duration, age of the patient)? (multiple answers possible, please specify in answer box) [if multidrug-resistant organisms (MDRO) are detected in urine cultures]
No

Are there situations or clinical findings in the patient where you deviate from your prophylaxis standard (medication, dose, duration, age of the patient)? (multiple answers possible, please specify in answer box) [if multidrug-resistant organisms (MDRO) are detected in urine cultures][Comment]

Are there situations or clinical findings in the patient where you deviate from your prophylaxis standard (medication, dose, duration, age of the patient)? (multiple answers possible, please specify in answer box) [in newborns]
No

Are there situations or clinical findings in the patient where you deviate from your prophylaxis standard (medication, dose, duration, age of the patient)? (multiple answers possible, please specify in answer box) [in newborns][Comment]

Are there situations or clinical findings in the patient where you deviate from your prophylaxis standard (medication, dose, duration, age of the patient)? (multiple answers possible, please specify in answer box) [Other]
all but

Are there situations or clinical findings in the patient where you deviate from your prophylaxis standard (medication, dose, duration, age of the patient)? (multiple answers possible, please specify in answer box) [Other comment]
according to pediatrc urologist / nephrologist

Are there situations in which you use intravenous prophylaxis?
No

Voiding analysis

Are there differences in antibiotic prophylaxis between VUS and VCUG in your department?
No

Is a non-pathological urine strip test required in preparation for VUS/VCUG in your department?
Yes

What is the maximum amount of days accepted for urine strip test to be performed prior to the exam?
7 days

Is a non-pathological urine culture required in preparation for VUS/VCUG in your department?
Yes

What is the maximum amount of days accepted for urine culture to be performed prior to the exam?
7 days

Do patients only receive post-procedure prophylaxis if vesicoureteral reflux (VUR) is detected in VUS/VCUG?
No

Do other MUS/MCU findings (e.g. urethral valves) have an influence on post-procedure prophylaxis?
No

End of survey

Thank you for taking the time to answer this survey!

May we contact you personally if we have further questions?
Yes

E-mail address:
mluisalobo@gmail.com

Survey response 140

Response ID
142

Last page
0

Start language
en

Seed
203446167

Date started
2025-03-18 10:14:51

Date last action
2025-03-18 10:14:51

Standard Operation Procedure

Does your department have a defined protocol for infection prophylaxis in the context of VUS/VCUG?
N/A

Has this protocol been transformed into a written standard operating procedure (SOP)?
N/A

Antibiotic Prophylaxis

Are there situations/constellations in which you do not use antibiotic prophylaxis for VUS/VCUG?
N/A

Please specify these situations/constellations: [girl with first uncomplicated UTI without urinary tract dilation in ultrasound]
N/A

Please specify these situations/constellations: [boy with first uncomplicated UTI without urinary tract dilation in ultrasound]
N/A

Please specify these situations/constellations: [in patients with low grade urinary tract dilation (up to 2nd grade) and no UTI in medical history]
N/A

If you use antibiotic prophylaxis: which mediaction do you use? (multiple answers and subdivision into age groups possible) [Trimethoprim]
N/A

If you use antibiotic prophylaxis: which mediaction do you use? (multiple answers and subdivision into age groups possible) [Nitrofurantoin]
N/A

If you use antibiotic prophylaxis: which mediaction do you use? (multiple answers and subdivision into age groups possible) [Amoxicillin]
N/A

If you use antibiotic prophylaxis: which mediaction do you use? (multiple answers and subdivision into age groups possible) [Cefaclor]
N/A

If you use antibiotic prophylaxis: which mediaction do you use? (multiple answers and subdivision into age groups possible) [Amoxicillin/clavulanic acid]
N/A

If you use antibiotic prophylaxis: which mediaction do you use? (multiple answers and subdivision into age groups possible) [Other]
N/A

Trimethoprim: which time schedule do you use? [Day(s) before VUS/VCUG, how many?]
N/A

Trimethoprim: which time schedule do you use? [Day of VUS/VCUG]
N/A

Trimethoprim: which time schedule do you use? [Day(s) after VUS/VCUG, how many?]
N/A

Nitrofurantoin: which time schedule do you use? [Day(s) before VUS/VCUG, how many?]
N/A

Nitrofurantoin: which time schedule do you use? [Day of VUS/VCUG]
N/A

Nitrofurantoin: which time schedule do you use? [Day(s) after VUS/VCUG, how many?]
N/A

Amoxicillin: which time schedule do you use? [Day(s) before VUS/VCUG, how many?]
N/A

Amoxicillin: which time schedule do you use? [Day of VUS/VCUG]
N/A

Amoxicillin: which time schedule do you use? [Day(s) after VUS/VCUG, how many?]
N/A

Cefaclor: which time schedule do you use? [Days(s) before VUS/VCUG, how many?]
N/A

Cefaclor: which time schedule do you use? [Day of VUS/VCUG]
N/A

Cefaclor: which time schedule do you use? [Day(s) after VUS/VCUG, how many?]
N/A

Amoxicillin/clavulanic acid: in what dosage? (Amoxicillin portion) [prophylaktic (10-20 mg/Kg bodyweight/d)]
N/A

Amoxicillin/clavulanic acid: in what dosage? (Amoxicillin portion) [double prophylactic (20-40 mg/Kg bodyweight/d)]
N/A

Amoxicillin/clavulanic acid: in what dosage? (Amoxicillin portion) [therapeutic (80-100 mg/Kg bodyweight/d)]
N/A

Amoxicillin/clavulanic acid: which time schedule do you use? [Day(s) before VUS/VCUG]
N/A

Amoxicillin/clavulanic acid: which time schedule do you use? [Day of VUS/VCUG]
N/A

Amoxicillin/clavulanic acid: which time schedule do you use? [Day(s) after VUS/VCUG]
N/A

Other: which time schedule do you use? [Day(s) before VUS/VCUG, how many?]
N/A

Other: which time schedule do you use? [Day of VUS/VCUG]
N/A

Other: which time schedule do you use? [Day(s) after VUS/VCUG, how many?]
N/A

Indication for prophylaxis

Which patients receive periinterventional antibiotic prophylaxis? (multiple answers possible) [all]
N/A

Which patients receive periinterventional antibiotic prophylaxis? (multiple answers possible) [patients post urinary tract infection/pyelonephritis/urosepsis]
N/A

Which patients receive periinterventional antibiotic prophylaxis? (multiple answers possible) [patients with urinary tract dilation in ultrasound]
N/A

Which patients receive periinterventional antibiotic prophylaxis? (multiple answers possible) [patients with prenatal urinary tract dilation]
N/A

Which patients receive periinterventional antibiotic prophylaxis? (multiple answers possible) [patients post VUR surgery]
N/A

Which patients receive periinterventional antibiotic prophylaxis? (multiple answers possible) [none]
N/A

Are there situations or clinical findings in the patient where you deviate from your prophylaxis standard (medication, dose, duration, age of the patient)? (multiple answers possible, please specify in answer box) [in case of already known vesicoureteral reflux (VUR)]
N/A

Are there situations or clinical findings in the patient where you deviate from your prophylaxis standard (medication, dose, duration, age of the patient)? (multiple answers possible, please specify in answer box) [in patients with recurrent urinary tract infections (UTI)]
N/A

Are there situations or clinical findings in the patient where you deviate from your prophylaxis standard (medication, dose, duration, age of the patient)? (multiple answers possible, please specify in answer box) [if multidrug-resistant organisms (MDRO) are detected in urine cultures]
N/A

Are there situations or clinical findings in the patient where you deviate from your prophylaxis standard (medication, dose, duration, age of the patient)? (multiple answers possible, please specify in answer box) [in newborns]
N/A

Are there situations in which you use intravenous prophylaxis?
N/A

Voiding analysis

Are there differences in antibiotic prophylaxis between VUS and VCUG in your department?
N/A

Is a non-pathological urine strip test required in preparation for VUS/VCUG in your department?
N/A

Is a non-pathological urine culture required in preparation for VUS/VCUG in your department?
N/A

Do patients only receive post-procedure prophylaxis if vesicoureteral reflux (VUR) is detected in VUS/VCUG?
N/A

Do other MUS/MCU findings (e.g. urethral valves) have an influence on post-procedure prophylaxis?
N/A

End of survey

May we contact you personally if we have further questions?
N/A

Survey response 141

Response ID
143

Date submitted
2025-03-21 18:36:08

Last page
5

Start language
de

Seed
1221992235

Date started
2025-03-21 18:32:20

Date last action
2025-03-21 18:36:08

Standard Operation Procedure

Who fills out this survey?
Pediatric Surgeon

Who fills out this survey? [Other]

In which type of hospital are you employed?
Community hospital (>50 beds for pediatric patients)

In which type of hospital are you employed? [Other]

In which country do you work?
Deutschland

Does your department have a defined protocol for infection prophylaxis in the context of VUS/VCUG?
Yes

Has this protocol been transformed into a written standard operating procedure (SOP)?
Yes

Which department is the author of the SOP for infection prophylaxis in VUS/VCUG?
Pediatric surgery

Which department is the author of the SOP for infection prophylaxis in VUS/VCUG? [Other]

Antibiotic Prophylaxis

Are there situations/constellations in which you do not use antibiotic prophylaxis for VUS/VCUG?
No

Please specify these situations/constellations: [girl with first uncomplicated UTI without urinary tract dilation in ultrasound]
N/A

Please specify these situations/constellations: [boy with first uncomplicated UTI without urinary tract dilation in ultrasound]
N/A

Please specify these situations/constellations: [in patients with low grade urinary tract dilation (up to 2nd grade) and no UTI in medical history]
N/A

If you use antibiotic prophylaxis: which mediaction do you use? (multiple answers and subdivision into age groups possible) [Trimethoprim]
Yes

If you use antibiotic prophylaxis: which mediaction do you use? (multiple answers and subdivision into age groups possible) [Nitrofurantoin]
No

If you use antibiotic prophylaxis: which mediaction do you use? (multiple answers and subdivision into age groups possible) [Amoxicillin]
No

If you use antibiotic prophylaxis: which mediaction do you use? (multiple answers and subdivision into age groups possible) [Cefaclor]
No

If you use antibiotic prophylaxis: which mediaction do you use? (multiple answers and subdivision into age groups possible) [Amoxicillin/clavulanic acid]
No

If you use antibiotic prophylaxis: which mediaction do you use? (multiple answers and subdivision into age groups possible) [Other]
No

Trimethoprim: in what dosage?
double prophylakctic (4 mg/kg bodyweight/d)

Trimethoprim: in what dosage? [Other]

Trimethoprim: for which age group do you use trimethoprim?
alle

Trimethoprim: which time schedule do you use? [Day(s) before VUS/VCUG, how many?]
Yes

Trimethoprim: which time schedule do you use? [Day(s) before VUS/VCUG, how many?][Comment]
1

Trimethoprim: which time schedule do you use? [Day of VUS/VCUG]
Yes

Trimethoprim: which time schedule do you use? [Day of VUS/VCUG][Comment]

Trimethoprim: which time schedule do you use? [Day(s) after VUS/VCUG, how many?]
Yes

Trimethoprim: which time schedule do you use? [Day(s) after VUS/VCUG, how many?][Comment]
1

Nitrofurantoin: which time schedule do you use? [Day(s) before VUS/VCUG, how many?]
N/A

Nitrofurantoin: which time schedule do you use? [Day of VUS/VCUG]
N/A

Nitrofurantoin: which time schedule do you use? [Day(s) after VUS/VCUG, how many?]
N/A

Amoxicillin: which time schedule do you use? [Day(s) before VUS/VCUG, how many?]
N/A

Amoxicillin: which time schedule do you use? [Day of VUS/VCUG]
N/A

Amoxicillin: which time schedule do you use? [Day(s) after VUS/VCUG, how many?]
N/A

Cefaclor: which time schedule do you use? [Days(s) before VUS/VCUG, how many?]
N/A

Cefaclor: which time schedule do you use? [Day of VUS/VCUG]
N/A

Cefaclor: which time schedule do you use? [Day(s) after VUS/VCUG, how many?]
N/A

Amoxicillin/clavulanic acid: in what dosage? (Amoxicillin portion) [prophylaktic (10-20 mg/Kg bodyweight/d)]
N/A

Amoxicillin/clavulanic acid: in what dosage? (Amoxicillin portion) [double prophylactic (20-40 mg/Kg bodyweight/d)]
N/A

Amoxicillin/clavulanic acid: in what dosage? (Amoxicillin portion) [therapeutic (80-100 mg/Kg bodyweight/d)]
N/A

Amoxicillin/clavulanic acid: which time schedule do you use? [Day(s) before VUS/VCUG]
N/A

Amoxicillin/clavulanic acid: which time schedule do you use? [Day of VUS/VCUG]
N/A

Amoxicillin/clavulanic acid: which time schedule do you use? [Day(s) after VUS/VCUG]
N/A

Other: which time schedule do you use? [Day(s) before VUS/VCUG, how many?]
N/A

Other: which time schedule do you use? [Day of VUS/VCUG]
N/A

Other: which time schedule do you use? [Day(s) after VUS/VCUG, how many?]
N/A

Indication for prophylaxis

Which patients receive periinterventional antibiotic prophylaxis? (multiple answers possible) [all]
Yes

Which patients receive periinterventional antibiotic prophylaxis? (multiple answers possible) [patients post urinary tract infection/pyelonephritis/urosepsis]
N/A

Which patients receive periinterventional antibiotic prophylaxis? (multiple answers possible) [patients with urinary tract dilation in ultrasound]
N/A

Which patients receive periinterventional antibiotic prophylaxis? (multiple answers possible) [patients with prenatal urinary tract dilation]
N/A

Which patients receive periinterventional antibiotic prophylaxis? (multiple answers possible) [patients post VUR surgery]
N/A

Which patients receive periinterventional antibiotic prophylaxis? (multiple answers possible) [none]
N/A

Are there situations or clinical findings in the patient where you deviate from your prophylaxis standard (medication, dose, duration, age of the patient)? (multiple answers possible, please specify in answer box) [in case of already known vesicoureteral reflux (VUR)]
No

Are there situations or clinical findings in the patient where you deviate from your prophylaxis standard (medication, dose, duration, age of the patient)? (multiple answers possible, please specify in answer box) [in case of already known vesicoureteral reflux (VUR)][Comment]

Are there situations or clinical findings in the patient where you deviate from your prophylaxis standard (medication, dose, duration, age of the patient)? (multiple answers possible, please specify in answer box) [in patients with recurrent urinary tract infections (UTI)]
No

Are there situations or clinical findings in the patient where you deviate from your prophylaxis standard (medication, dose, duration, age of the patient)? (multiple answers possible, please specify in answer box) [in patients with recurrent urinary tract infections (UTI)][Comment]

Are there situations or clinical findings in the patient where you deviate from your prophylaxis standard (medication, dose, duration, age of the patient)? (multiple answers possible, please specify in answer box) [if multidrug-resistant organisms (MDRO) are detected in urine cultures]
Yes

Are there situations or clinical findings in the patient where you deviate from your prophylaxis standard (medication, dose, duration, age of the patient)? (multiple answers possible, please specify in answer box) [if multidrug-resistant organisms (MDRO) are detected in urine cultures][Comment]
nach Resistogramm

Are there situations or clinical findings in the patient where you deviate from your prophylaxis standard (medication, dose, duration, age of the patient)? (multiple answers possible, please specify in answer box) [in newborns]
No

Are there situations or clinical findings in the patient where you deviate from your prophylaxis standard (medication, dose, duration, age of the patient)? (multiple answers possible, please specify in answer box) [in newborns][Comment]

Are there situations or clinical findings in the patient where you deviate from your prophylaxis standard (medication, dose, duration, age of the patient)? (multiple answers possible, please specify in answer box) [Other]

Are there situations or clinical findings in the patient where you deviate from your prophylaxis standard (medication, dose, duration, age of the patient)? (multiple answers possible, please specify in answer box) [Other comment]

Are there situations in which you use intravenous prophylaxis?
Yes

In which situations?
wenn Resistogramm keine orale AB enthält

Voiding analysis

Are there differences in antibiotic prophylaxis between VUS and VCUG in your department?
No

Is a non-pathological urine strip test required in preparation for VUS/VCUG in your department?
Yes

What is the maximum amount of days accepted for urine strip test to be performed prior to the exam?
14

Is a non-pathological urine culture required in preparation for VUS/VCUG in your department?
No

Do patients only receive post-procedure prophylaxis if vesicoureteral reflux (VUR) is detected in VUS/VCUG?
No

Do other MUS/MCU findings (e.g. urethral valves) have an influence on post-procedure prophylaxis?
No

End of survey

Thank you for taking the time to answer this survey!

May we contact you personally if we have further questions?
Yes

E-mail address:
julian.busch@klinikum-nuernberg.de

Survey response 142

Response ID
144

Last page
1

Start language
de

Seed
939169754

Date started
2025-03-21 19:40:06

Date last action
2025-03-21 19:42:19

Standard Operation Procedure

Who fills out this survey?
Pediatric Surgeon

Who fills out this survey? [Other]

In which type of hospital are you employed?
Community hospital (>50 beds for pediatric patients)

In which type of hospital are you employed? [Other]

In which country do you work?
Hamburg

Does your department have a defined protocol for infection prophylaxis in the context of VUS/VCUG?
No

Has this protocol been transformed into a written standard operating procedure (SOP)?
N/A

Antibiotic Prophylaxis

Are there situations/constellations in which you do not use antibiotic prophylaxis for VUS/VCUG?
N/A

Please specify these situations/constellations: [girl with first uncomplicated UTI without urinary tract dilation in ultrasound]
N/A

Please specify these situations/constellations: [boy with first uncomplicated UTI without urinary tract dilation in ultrasound]
N/A

Please specify these situations/constellations: [in patients with low grade urinary tract dilation (up to 2nd grade) and no UTI in medical history]
N/A

If you use antibiotic prophylaxis: which mediaction do you use? (multiple answers and subdivision into age groups possible) [Trimethoprim]
No

If you use antibiotic prophylaxis: which mediaction do you use? (multiple answers and subdivision into age groups possible) [Nitrofurantoin]
No

If you use antibiotic prophylaxis: which mediaction do you use? (multiple answers and subdivision into age groups possible) [Amoxicillin]
No

If you use antibiotic prophylaxis: which mediaction do you use? (multiple answers and subdivision into age groups possible) [Cefaclor]
No

If you use antibiotic prophylaxis: which mediaction do you use? (multiple answers and subdivision into age groups possible) [Amoxicillin/clavulanic acid]
No

If you use antibiotic prophylaxis: which mediaction do you use? (multiple answers and subdivision into age groups possible) [Other]
No

Trimethoprim: which time schedule do you use? [Day(s) before VUS/VCUG, how many?]
N/A

Trimethoprim: which time schedule do you use? [Day of VUS/VCUG]
N/A

Trimethoprim: which time schedule do you use? [Day(s) after VUS/VCUG, how many?]
N/A

Nitrofurantoin: which time schedule do you use? [Day(s) before VUS/VCUG, how many?]
N/A

Nitrofurantoin: which time schedule do you use? [Day of VUS/VCUG]
N/A

Nitrofurantoin: which time schedule do you use? [Day(s) after VUS/VCUG, how many?]
N/A

Amoxicillin: which time schedule do you use? [Day(s) before VUS/VCUG, how many?]
N/A

Amoxicillin: which time schedule do you use? [Day of VUS/VCUG]
N/A

Amoxicillin: which time schedule do you use? [Day(s) after VUS/VCUG, how many?]
N/A

Cefaclor: which time schedule do you use? [Days(s) before VUS/VCUG, how many?]
N/A

Cefaclor: which time schedule do you use? [Day of VUS/VCUG]
N/A

Cefaclor: which time schedule do you use? [Day(s) after VUS/VCUG, how many?]
N/A

Amoxicillin/clavulanic acid: in what dosage? (Amoxicillin portion) [prophylaktic (10-20 mg/Kg bodyweight/d)]
N/A

Amoxicillin/clavulanic acid: in what dosage? (Amoxicillin portion) [double prophylactic (20-40 mg/Kg bodyweight/d)]
N/A

Amoxicillin/clavulanic acid: in what dosage? (Amoxicillin portion) [therapeutic (80-100 mg/Kg bodyweight/d)]
N/A

Amoxicillin/clavulanic acid: which time schedule do you use? [Day(s) before VUS/VCUG]
N/A

Amoxicillin/clavulanic acid: which time schedule do you use? [Day of VUS/VCUG]
N/A

Amoxicillin/clavulanic acid: which time schedule do you use? [Day(s) after VUS/VCUG]
N/A

Other: which time schedule do you use? [Day(s) before VUS/VCUG, how many?]
N/A

Other: which time schedule do you use? [Day of VUS/VCUG]
N/A

Other: which time schedule do you use? [Day(s) after VUS/VCUG, how many?]
N/A

Indication for prophylaxis

Which patients receive periinterventional antibiotic prophylaxis? (multiple answers possible) [all]
N/A

Which patients receive periinterventional antibiotic prophylaxis? (multiple answers possible) [patients post urinary tract infection/pyelonephritis/urosepsis]
N/A

Which patients receive periinterventional antibiotic prophylaxis? (multiple answers possible) [patients with urinary tract dilation in ultrasound]
N/A

Which patients receive periinterventional antibiotic prophylaxis? (multiple answers possible) [patients with prenatal urinary tract dilation]
N/A

Which patients receive periinterventional antibiotic prophylaxis? (multiple answers possible) [patients post VUR surgery]
N/A

Which patients receive periinterventional antibiotic prophylaxis? (multiple answers possible) [none]
N/A

Are there situations or clinical findings in the patient where you deviate from your prophylaxis standard (medication, dose, duration, age of the patient)? (multiple answers possible, please specify in answer box) [in case of already known vesicoureteral reflux (VUR)]
N/A

Are there situations or clinical findings in the patient where you deviate from your prophylaxis standard (medication, dose, duration, age of the patient)? (multiple answers possible, please specify in answer box) [in patients with recurrent urinary tract infections (UTI)]
N/A

Are there situations or clinical findings in the patient where you deviate from your prophylaxis standard (medication, dose, duration, age of the patient)? (multiple answers possible, please specify in answer box) [if multidrug-resistant organisms (MDRO) are detected in urine cultures]
N/A

Are there situations or clinical findings in the patient where you deviate from your prophylaxis standard (medication, dose, duration, age of the patient)? (multiple answers possible, please specify in answer box) [in newborns]
N/A

Are there situations in which you use intravenous prophylaxis?
N/A

Voiding analysis

Are there differences in antibiotic prophylaxis between VUS and VCUG in your department?
N/A

Is a non-pathological urine strip test required in preparation for VUS/VCUG in your department?
N/A

Is a non-pathological urine culture required in preparation for VUS/VCUG in your department?
N/A

Do patients only receive post-procedure prophylaxis if vesicoureteral reflux (VUR) is detected in VUS/VCUG?
N/A

Do other MUS/MCU findings (e.g. urethral valves) have an influence on post-procedure prophylaxis?
N/A

End of survey

May we contact you personally if we have further questions?
N/A

Survey response 143

Response ID
145

Date submitted
2025-03-21 22:36:11

Last page
5

Start language
de

Seed
1709005732

Date started
2025-03-21 22:32:23

Date last action
2025-03-21 22:36:11

Standard Operation Procedure

Who fills out this survey?
Pediatric Surgeon

Who fills out this survey? [Other]

In which type of hospital are you employed?
University Hospital

In which type of hospital are you employed? [Other]

In which country do you work?
Deutschland

Does your department have a defined protocol for infection prophylaxis in the context of VUS/VCUG?
Yes

Has this protocol been transformed into a written standard operating procedure (SOP)?
No

Antibiotic Prophylaxis

Are there situations/constellations in which you do not use antibiotic prophylaxis for VUS/VCUG?
No

Please specify these situations/constellations: [girl with first uncomplicated UTI without urinary tract dilation in ultrasound]
N/A

Please specify these situations/constellations: [boy with first uncomplicated UTI without urinary tract dilation in ultrasound]
N/A

Please specify these situations/constellations: [in patients with low grade urinary tract dilation (up to 2nd grade) and no UTI in medical history]
N/A

If you use antibiotic prophylaxis: which mediaction do you use? (multiple answers and subdivision into age groups possible) [Trimethoprim]
Yes

If you use antibiotic prophylaxis: which mediaction do you use? (multiple answers and subdivision into age groups possible) [Nitrofurantoin]
No

If you use antibiotic prophylaxis: which mediaction do you use? (multiple answers and subdivision into age groups possible) [Amoxicillin]
No

If you use antibiotic prophylaxis: which mediaction do you use? (multiple answers and subdivision into age groups possible) [Cefaclor]
No

If you use antibiotic prophylaxis: which mediaction do you use? (multiple answers and subdivision into age groups possible) [Amoxicillin/clavulanic acid]
No

If you use antibiotic prophylaxis: which mediaction do you use? (multiple answers and subdivision into age groups possible) [Other]
No

Trimethoprim: in what dosage?
double prophylakctic (4 mg/kg bodyweight/d)

Trimethoprim: in what dosage? [Other]

Trimethoprim: for which age group do you use trimethoprim?
Ab 1 monat

Trimethoprim: which time schedule do you use? [Day(s) before VUS/VCUG, how many?]
Yes

Trimethoprim: which time schedule do you use? [Day(s) before VUS/VCUG, how many?][Comment]
1

Trimethoprim: which time schedule do you use? [Day of VUS/VCUG]
Yes

Trimethoprim: which time schedule do you use? [Day of VUS/VCUG][Comment]

Trimethoprim: which time schedule do you use? [Day(s) after VUS/VCUG, how many?]
Yes

Trimethoprim: which time schedule do you use? [Day(s) after VUS/VCUG, how many?][Comment]
1

Nitrofurantoin: which time schedule do you use? [Day(s) before VUS/VCUG, how many?]
N/A

Nitrofurantoin: which time schedule do you use? [Day of VUS/VCUG]
N/A

Nitrofurantoin: which time schedule do you use? [Day(s) after VUS/VCUG, how many?]
N/A

Amoxicillin: which time schedule do you use? [Day(s) before VUS/VCUG, how many?]
N/A

Amoxicillin: which time schedule do you use? [Day of VUS/VCUG]
N/A

Amoxicillin: which time schedule do you use? [Day(s) after VUS/VCUG, how many?]
N/A

Cefaclor: which time schedule do you use? [Days(s) before VUS/VCUG, how many?]
N/A

Cefaclor: which time schedule do you use? [Day of VUS/VCUG]
N/A

Cefaclor: which time schedule do you use? [Day(s) after VUS/VCUG, how many?]
N/A

Amoxicillin/clavulanic acid: in what dosage? (Amoxicillin portion) [prophylaktic (10-20 mg/Kg bodyweight/d)]
N/A

Amoxicillin/clavulanic acid: in what dosage? (Amoxicillin portion) [double prophylactic (20-40 mg/Kg bodyweight/d)]
N/A

Amoxicillin/clavulanic acid: in what dosage? (Amoxicillin portion) [therapeutic (80-100 mg/Kg bodyweight/d)]
N/A

Amoxicillin/clavulanic acid: which time schedule do you use? [Day(s) before VUS/VCUG]
N/A

Amoxicillin/clavulanic acid: which time schedule do you use? [Day of VUS/VCUG]
N/A

Amoxicillin/clavulanic acid: which time schedule do you use? [Day(s) after VUS/VCUG]
N/A

Other: which time schedule do you use? [Day(s) before VUS/VCUG, how many?]
N/A

Other: which time schedule do you use? [Day of VUS/VCUG]
N/A

Other: which time schedule do you use? [Day(s) after VUS/VCUG, how many?]
N/A

Indication for prophylaxis

Which patients receive periinterventional antibiotic prophylaxis? (multiple answers possible) [all]
Yes

Which patients receive periinterventional antibiotic prophylaxis? (multiple answers possible) [patients post urinary tract infection/pyelonephritis/urosepsis]
N/A

Which patients receive periinterventional antibiotic prophylaxis? (multiple answers possible) [patients with urinary tract dilation in ultrasound]
N/A

Which patients receive periinterventional antibiotic prophylaxis? (multiple answers possible) [patients with prenatal urinary tract dilation]
N/A

Which patients receive periinterventional antibiotic prophylaxis? (multiple answers possible) [patients post VUR surgery]
N/A

Which patients receive periinterventional antibiotic prophylaxis? (multiple answers possible) [none]
N/A

Are there situations or clinical findings in the patient where you deviate from your prophylaxis standard (medication, dose, duration, age of the patient)? (multiple answers possible, please specify in answer box) [in case of already known vesicoureteral reflux (VUR)]
No

Are there situations or clinical findings in the patient where you deviate from your prophylaxis standard (medication, dose, duration, age of the patient)? (multiple answers possible, please specify in answer box) [in case of already known vesicoureteral reflux (VUR)][Comment]

Are there situations or clinical findings in the patient where you deviate from your prophylaxis standard (medication, dose, duration, age of the patient)? (multiple answers possible, please specify in answer box) [in patients with recurrent urinary tract infections (UTI)]
No

Are there situations or clinical findings in the patient where you deviate from your prophylaxis standard (medication, dose, duration, age of the patient)? (multiple answers possible, please specify in answer box) [in patients with recurrent urinary tract infections (UTI)][Comment]

Are there situations or clinical findings in the patient where you deviate from your prophylaxis standard (medication, dose, duration, age of the patient)? (multiple answers possible, please specify in answer box) [if multidrug-resistant organisms (MDRO) are detected in urine cultures]
Yes

Are there situations or clinical findings in the patient where you deviate from your prophylaxis standard (medication, dose, duration, age of the patient)? (multiple answers possible, please specify in answer box) [if multidrug-resistant organisms (MDRO) are detected in urine cultures][Comment]

Are there situations or clinical findings in the patient where you deviate from your prophylaxis standard (medication, dose, duration, age of the patient)? (multiple answers possible, please specify in answer box) [in newborns]
No

Are there situations or clinical findings in the patient where you deviate from your prophylaxis standard (medication, dose, duration, age of the patient)? (multiple answers possible, please specify in answer box) [in newborns][Comment]

Are there situations or clinical findings in the patient where you deviate from your prophylaxis standard (medication, dose, duration, age of the patient)? (multiple answers possible, please specify in answer box) [Other]

Are there situations or clinical findings in the patient where you deviate from your prophylaxis standard (medication, dose, duration, age of the patient)? (multiple answers possible, please specify in answer box) [Other comment]

Are there situations in which you use intravenous prophylaxis?
Yes

In which situations?
Zb am Ende einer pyeolnephritis Behandlung und Durchführung eines MCU/MUS

Voiding analysis

Are there differences in antibiotic prophylaxis between VUS and VCUG in your department?
No

Is a non-pathological urine strip test required in preparation for VUS/VCUG in your department?
Yes

What is the maximum amount of days accepted for urine strip test to be performed prior to the exam?
Keine SOP

Is a non-pathological urine culture required in preparation for VUS/VCUG in your department?
No

Do patients only receive post-procedure prophylaxis if vesicoureteral reflux (VUR) is detected in VUS/VCUG?
No

Do other MUS/MCU findings (e.g. urethral valves) have an influence on post-procedure prophylaxis?
No

End of survey

Thank you for taking the time to answer this survey!

May we contact you personally if we have further questions?
Yes

E-mail address:
Katja.dressen@ukbonn.de

Survey response 144

Response ID
146

Date submitted
2025-03-22 12:38:03

Last page
5

Start language
de

Seed
1635291647

Date started
2025-03-22 12:33:01

Date last action
2025-03-22 12:38:03

Standard Operation Procedure

Who fills out this survey?
Pediatric Surgeon

Who fills out this survey? [Other]

In which type of hospital are you employed?
Community hospital (>50 beds for pediatric patients)

In which type of hospital are you employed? [Other]

In which country do you work?
Deutschland

Does your department have a defined protocol for infection prophylaxis in the context of VUS/VCUG?
Yes

Has this protocol been transformed into a written standard operating procedure (SOP)?
Yes

Which department is the author of the SOP for infection prophylaxis in VUS/VCUG?
Pediatric surgery

Which department is the author of the SOP for infection prophylaxis in VUS/VCUG? [Other]

Antibiotic Prophylaxis

Are there situations/constellations in which you do not use antibiotic prophylaxis for VUS/VCUG?
Yes

Please specify these situations/constellations: [girl with first uncomplicated UTI without urinary tract dilation in ultrasound]
No

Please specify these situations/constellations: [boy with first uncomplicated UTI without urinary tract dilation in ultrasound]
No

Please specify these situations/constellations: [in patients with low grade urinary tract dilation (up to 2nd grade) and no UTI in medical history]
Yes

Please specify these situations/constellations: [Other]

If you use antibiotic prophylaxis: which mediaction do you use? (multiple answers and subdivision into age groups possible) [Trimethoprim]
Yes

If you use antibiotic prophylaxis: which mediaction do you use? (multiple answers and subdivision into age groups possible) [Nitrofurantoin]
No

If you use antibiotic prophylaxis: which mediaction do you use? (multiple answers and subdivision into age groups possible) [Amoxicillin]
No

If you use antibiotic prophylaxis: which mediaction do you use? (multiple answers and subdivision into age groups possible) [Cefaclor]
No

If you use antibiotic prophylaxis: which mediaction do you use? (multiple answers and subdivision into age groups possible) [Amoxicillin/clavulanic acid]
No

If you use antibiotic prophylaxis: which mediaction do you use? (multiple answers and subdivision into age groups possible) [Other]
No

Trimethoprim: in what dosage?
therapeutic (6 mg/kg bodyweight/d)

Trimethoprim: in what dosage? [Other]

Trimethoprim: for which age group do you use trimethoprim?
0-16

Trimethoprim: which time schedule do you use? [Day(s) before VUS/VCUG, how many?]
No

Trimethoprim: which time schedule do you use? [Day(s) before VUS/VCUG, how many?][Comment]

Trimethoprim: which time schedule do you use? [Day of VUS/VCUG]
Yes

Trimethoprim: which time schedule do you use? [Day of VUS/VCUG][Comment]

Trimethoprim: which time schedule do you use? [Day(s) after VUS/VCUG, how many?]
No

Trimethoprim: which time schedule do you use? [Day(s) after VUS/VCUG, how many?][Comment]

Nitrofurantoin: which time schedule do you use? [Day(s) before VUS/VCUG, how many?]
N/A

Nitrofurantoin: which time schedule do you use? [Day of VUS/VCUG]
N/A

Nitrofurantoin: which time schedule do you use? [Day(s) after VUS/VCUG, how many?]
N/A

Amoxicillin: which time schedule do you use? [Day(s) before VUS/VCUG, how many?]
N/A

Amoxicillin: which time schedule do you use? [Day of VUS/VCUG]
N/A

Amoxicillin: which time schedule do you use? [Day(s) after VUS/VCUG, how many?]
N/A

Cefaclor: which time schedule do you use? [Days(s) before VUS/VCUG, how many?]
N/A

Cefaclor: which time schedule do you use? [Day of VUS/VCUG]
N/A

Cefaclor: which time schedule do you use? [Day(s) after VUS/VCUG, how many?]
N/A

Amoxicillin/clavulanic acid: in what dosage? (Amoxicillin portion) [prophylaktic (10-20 mg/Kg bodyweight/d)]
N/A

Amoxicillin/clavulanic acid: in what dosage? (Amoxicillin portion) [double prophylactic (20-40 mg/Kg bodyweight/d)]
N/A

Amoxicillin/clavulanic acid: in what dosage? (Amoxicillin portion) [therapeutic (80-100 mg/Kg bodyweight/d)]
N/A

Amoxicillin/clavulanic acid: which time schedule do you use? [Day(s) before VUS/VCUG]
N/A

Amoxicillin/clavulanic acid: which time schedule do you use? [Day of VUS/VCUG]
N/A

Amoxicillin/clavulanic acid: which time schedule do you use? [Day(s) after VUS/VCUG]
N/A

Other: which time schedule do you use? [Day(s) before VUS/VCUG, how many?]
N/A

Other: which time schedule do you use? [Day of VUS/VCUG]
N/A

Other: which time schedule do you use? [Day(s) after VUS/VCUG, how many?]
N/A

Indication for prophylaxis

Which patients receive periinterventional antibiotic prophylaxis? (multiple answers possible) [all]
No

Which patients receive periinterventional antibiotic prophylaxis? (multiple answers possible) [patients post urinary tract infection/pyelonephritis/urosepsis]
Yes

Which patients receive periinterventional antibiotic prophylaxis? (multiple answers possible) [patients with urinary tract dilation in ultrasound]
No

Which patients receive periinterventional antibiotic prophylaxis? (multiple answers possible) [patients with prenatal urinary tract dilation]
No

Which patients receive periinterventional antibiotic prophylaxis? (multiple answers possible) [patients post VUR surgery]
No

Which patients receive periinterventional antibiotic prophylaxis? (multiple answers possible) [none]
No

Which patients receive periinterventional antibiotic prophylaxis? (multiple answers possible) [Other]

patients status post urinary tract infection/pyelonephritis/urosepsis: what is the minimum time between infection and VUS/VCUG? (in days)
3-4 Wochen

Are there situations or clinical findings in the patient where you deviate from your prophylaxis standard (medication, dose, duration, age of the patient)? (multiple answers possible, please specify in answer box) [in case of already known vesicoureteral reflux (VUR)]
Yes

Are there situations or clinical findings in the patient where you deviate from your prophylaxis standard (medication, dose, duration, age of the patient)? (multiple answers possible, please specify in answer box) [in case of already known vesicoureteral reflux (VUR)][Comment]
Fortführen der vorbestehenden Dauerprophylaxe

Are there situations or clinical findings in the patient where you deviate from your prophylaxis standard (medication, dose, duration, age of the patient)? (multiple answers possible, please specify in answer box) [in patients with recurrent urinary tract infections (UTI)]
No

Are there situations or clinical findings in the patient where you deviate from your prophylaxis standard (medication, dose, duration, age of the patient)? (multiple answers possible, please specify in answer box) [in patients with recurrent urinary tract infections (UTI)][Comment]

Are there situations or clinical findings in the patient where you deviate from your prophylaxis standard (medication, dose, duration, age of the patient)? (multiple answers possible, please specify in answer box) [if multidrug-resistant organisms (MDRO) are detected in urine cultures]
Yes

Are there situations or clinical findings in the patient where you deviate from your prophylaxis standard (medication, dose, duration, age of the patient)? (multiple answers possible, please specify in answer box) [if multidrug-resistant organisms (MDRO) are detected in urine cultures][Comment]
adaptierte Prophylaxe

Are there situations or clinical findings in the patient where you deviate from your prophylaxis standard (medication, dose, duration, age of the patient)? (multiple answers possible, please specify in answer box) [in newborns]
Yes

Are there situations or clinical findings in the patient where you deviate from your prophylaxis standard (medication, dose, duration, age of the patient)? (multiple answers possible, please specify in answer box) [in newborns][Comment]
eher Cefuroxim

Are there situations or clinical findings in the patient where you deviate from your prophylaxis standard (medication, dose, duration, age of the patient)? (multiple answers possible, please specify in answer box) [Other]

Are there situations or clinical findings in the patient where you deviate from your prophylaxis standard (medication, dose, duration, age of the patient)? (multiple answers possible, please specify in answer box) [Other comment]

Are there situations in which you use intravenous prophylaxis?
No

Voiding analysis

Are there differences in antibiotic prophylaxis between VUS and VCUG in your department?
No

Is a non-pathological urine strip test required in preparation for VUS/VCUG in your department?
No

Is a non-pathological urine culture required in preparation for VUS/VCUG in your department?
No

Do patients only receive post-procedure prophylaxis if vesicoureteral reflux (VUR) is detected in VUS/VCUG?
No

Do other MUS/MCU findings (e.g. urethral valves) have an influence on post-procedure prophylaxis?
No

End of survey

Thank you for taking the time to answer this survey!

May we contact you personally if we have further questions?
Yes

E-mail address:
hartwig.sauter@rkh-gesundheit.de

Survey response 145

Response ID
147

Date submitted
2025-03-22 16:53:24

Last page
5

Start language
de

Seed
1401888234

Date started
2025-03-22 16:45:51

Date last action
2025-03-22 16:53:24

Standard Operation Procedure

Who fills out this survey?
Pediatric Surgeon

Who fills out this survey? [Other]

In which type of hospital are you employed?
Community hospital (>50 beds for pediatric patients)

In which type of hospital are you employed? [Other]

In which country do you work?
Deutschland

Does your department have a defined protocol for infection prophylaxis in the context of VUS/VCUG?
No

Has this protocol been transformed into a written standard operating procedure (SOP)?
N/A

Antibiotic Prophylaxis

Are there situations/constellations in which you do not use antibiotic prophylaxis for VUS/VCUG?
Yes

Please specify these situations/constellations: [girl with first uncomplicated UTI without urinary tract dilation in ultrasound]
No

Please specify these situations/constellations: [boy with first uncomplicated UTI without urinary tract dilation in ultrasound]
No

Please specify these situations/constellations: [in patients with low grade urinary tract dilation (up to 2nd grade) and no UTI in medical history]
No

Please specify these situations/constellations: [Other]
Ablehnung durch Eltern

If you use antibiotic prophylaxis: which mediaction do you use? (multiple answers and subdivision into age groups possible) [Trimethoprim]
Yes

If you use antibiotic prophylaxis: which mediaction do you use? (multiple answers and subdivision into age groups possible) [Nitrofurantoin]
Yes

If you use antibiotic prophylaxis: which mediaction do you use? (multiple answers and subdivision into age groups possible) [Amoxicillin]
No

If you use antibiotic prophylaxis: which mediaction do you use? (multiple answers and subdivision into age groups possible) [Cefaclor]
Yes

If you use antibiotic prophylaxis: which mediaction do you use? (multiple answers and subdivision into age groups possible) [Amoxicillin/clavulanic acid]
No

If you use antibiotic prophylaxis: which mediaction do you use? (multiple answers and subdivision into age groups possible) [Other]
No

Trimethoprim: in what dosage?
prophylactic (2 mg/kg bodyweight/d)

Trimethoprim: in what dosage? [Other]

Trimethoprim: for which age group do you use trimethoprim?
Ab 6 Monaten

Trimethoprim: which time schedule do you use? [Day(s) before VUS/VCUG, how many?]
No

Trimethoprim: which time schedule do you use? [Day(s) before VUS/VCUG, how many?][Comment]

Trimethoprim: which time schedule do you use? [Day of VUS/VCUG]
Yes

Trimethoprim: which time schedule do you use? [Day of VUS/VCUG][Comment]

Trimethoprim: which time schedule do you use? [Day(s) after VUS/VCUG, how many?]
No

Trimethoprim: which time schedule do you use? [Day(s) after VUS/VCUG, how many?][Comment]

Nitrofurantoin: in what dosage?
prophylactic (1-2 mg/kg bodyweight/d)

Nitrofurantoin: in what dosage? [Other]

Nitrofurantoin: for which age group do you use nitrofurantoin?
Ab 6 Monaten

Nitrofurantoin: which time schedule do you use? [Day(s) before VUS/VCUG, how many?]
No

Nitrofurantoin: which time schedule do you use? [Day(s) before VUS/VCUG, how many?][Comment]

Nitrofurantoin: which time schedule do you use? [Day of VUS/VCUG]
Yes

Nitrofurantoin: which time schedule do you use? [Day of VUS/VCUG][Comment]

Nitrofurantoin: which time schedule do you use? [Day(s) after VUS/VCUG, how many?]
No

Nitrofurantoin: which time schedule do you use? [Day(s) after VUS/VCUG, how many?][Comment]

Amoxicillin: which time schedule do you use? [Day(s) before VUS/VCUG, how many?]
N/A

Amoxicillin: which time schedule do you use? [Day of VUS/VCUG]
N/A

Amoxicillin: which time schedule do you use? [Day(s) after VUS/VCUG, how many?]
N/A

Cefaclor: in what dosage?
prophylactic (10 mg/kg bodyweight/d)

Cefaclor: in what dosage? [Other]

Cefaclor: for which age group do you use cefaclor?
Bis 6 Monate

Cefaclor: which time schedule do you use? [Days(s) before VUS/VCUG, how many?]
No

Cefaclor: which time schedule do you use? [Days(s) before VUS/VCUG, how many?][Comment]

Cefaclor: which time schedule do you use? [Day of VUS/VCUG]
Yes

Cefaclor: which time schedule do you use? [Day of VUS/VCUG][Comment]

Cefaclor: which time schedule do you use? [Day(s) after VUS/VCUG, how many?]
No

Cefaclor: which time schedule do you use? [Day(s) after VUS/VCUG, how many?][Comment]

Amoxicillin/clavulanic acid: in what dosage? (Amoxicillin portion) [prophylaktic (10-20 mg/Kg bodyweight/d)]
N/A

Amoxicillin/clavulanic acid: in what dosage? (Amoxicillin portion) [double prophylactic (20-40 mg/Kg bodyweight/d)]
N/A

Amoxicillin/clavulanic acid: in what dosage? (Amoxicillin portion) [therapeutic (80-100 mg/Kg bodyweight/d)]
N/A

Amoxicillin/clavulanic acid: which time schedule do you use? [Day(s) before VUS/VCUG]
N/A

Amoxicillin/clavulanic acid: which time schedule do you use? [Day of VUS/VCUG]
N/A

Amoxicillin/clavulanic acid: which time schedule do you use? [Day(s) after VUS/VCUG]
N/A

Other: which time schedule do you use? [Day(s) before VUS/VCUG, how many?]
N/A

Other: which time schedule do you use? [Day of VUS/VCUG]
N/A

Other: which time schedule do you use? [Day(s) after VUS/VCUG, how many?]
N/A

Indication for prophylaxis

Which patients receive periinterventional antibiotic prophylaxis? (multiple answers possible) [all]
No

Which patients receive periinterventional antibiotic prophylaxis? (multiple answers possible) [patients post urinary tract infection/pyelonephritis/urosepsis]
Yes

Which patients receive periinterventional antibiotic prophylaxis? (multiple answers possible) [patients with urinary tract dilation in ultrasound]
Yes

Which patients receive periinterventional antibiotic prophylaxis? (multiple answers possible) [patients with prenatal urinary tract dilation]
No

Which patients receive periinterventional antibiotic prophylaxis? (multiple answers possible) [patients post VUR surgery]
No

Which patients receive periinterventional antibiotic prophylaxis? (multiple answers possible) [none]
No

Which patients receive periinterventional antibiotic prophylaxis? (multiple answers possible) [Other]

patients status post urinary tract infection/pyelonephritis/urosepsis: what is the minimum time between infection and VUS/VCUG? (in days)
Ende der Therapie

In patients with urinary tract dilation in ultrasound: from what degree on? Image from Beetz R, Bokenkamp A, Brandis M et al (2001) Diagnosis of congenital dilatation of the urinary tract. Consensus Group of the Pediatric Nephrology Working Society in cooperation with the Pediatric Urology Working Group of the German Society of Urology and with the Pediatric Urology Working Society in the Germany Society of Pediatric Surgery. Urologe A 40:495–507 (quiz 8–9)
grade 1

Are there situations or clinical findings in the patient where you deviate from your prophylaxis standard (medication, dose, duration, age of the patient)? (multiple answers possible, please specify in answer box) [in case of already known vesicoureteral reflux (VUR)]
Yes

Are there situations or clinical findings in the patient where you deviate from your prophylaxis standard (medication, dose, duration, age of the patient)? (multiple answers possible, please specify in answer box) [in case of already known vesicoureteral reflux (VUR)][Comment]

Are there situations or clinical findings in the patient where you deviate from your prophylaxis standard (medication, dose, duration, age of the patient)? (multiple answers possible, please specify in answer box) [in patients with recurrent urinary tract infections (UTI)]
No

Are there situations or clinical findings in the patient where you deviate from your prophylaxis standard (medication, dose, duration, age of the patient)? (multiple answers possible, please specify in answer box) [in patients with recurrent urinary tract infections (UTI)][Comment]

Are there situations or clinical findings in the patient where you deviate from your prophylaxis standard (medication, dose, duration, age of the patient)? (multiple answers possible, please specify in answer box) [if multidrug-resistant organisms (MDRO) are detected in urine cultures]
Yes

Are there situations or clinical findings in the patient where you deviate from your prophylaxis standard (medication, dose, duration, age of the patient)? (multiple answers possible, please specify in answer box) [if multidrug-resistant organisms (MDRO) are detected in urine cultures][Comment]

Are there situations or clinical findings in the patient where you deviate from your prophylaxis standard (medication, dose, duration, age of the patient)? (multiple answers possible, please specify in answer box) [in newborns]
No

Are there situations or clinical findings in the patient where you deviate from your prophylaxis standard (medication, dose, duration, age of the patient)? (multiple answers possible, please specify in answer box) [in newborns][Comment]

Are there situations or clinical findings in the patient where you deviate from your prophylaxis standard (medication, dose, duration, age of the patient)? (multiple answers possible, please specify in answer box) [Other]

Are there situations or clinical findings in the patient where you deviate from your prophylaxis standard (medication, dose, duration, age of the patient)? (multiple answers possible, please specify in answer box) [Other comment]

Are there situations in which you use intravenous prophylaxis?
No

Voiding analysis

Are there differences in antibiotic prophylaxis between VUS and VCUG in your department?
No

Is a non-pathological urine strip test required in preparation for VUS/VCUG in your department?
Yes

What is the maximum amount of days accepted for urine strip test to be performed prior to the exam?
Direkt vor der Untersuchung

Is a non-pathological urine culture required in preparation for VUS/VCUG in your department?
No

Do patients only receive post-procedure prophylaxis if vesicoureteral reflux (VUR) is detected in VUS/VCUG?
Yes

Please explain your approach:
Antibiose weitet bis zur Therapie

Do other MUS/MCU findings (e.g. urethral valves) have an influence on post-procedure prophylaxis?
No

End of survey

Thank you for taking the time to answer this survey!

May we contact you personally if we have further questions?
Yes

E-mail address:
andrea.etzler@valeo-kliniken.de

Survey response 146

Response ID
148

Date submitted
2025-03-23 01:53:18

Last page
5

Start language
de

Seed
1990903333

Date started
2025-03-23 01:48:59

Date last action
2025-03-23 01:53:18

Standard Operation Procedure

Who fills out this survey?
Pediatric Surgeon

Who fills out this survey? [Other]

In which type of hospital are you employed?
Community hospital (>50 beds for pediatric patients)

In which type of hospital are you employed? [Other]

In which country do you work?
D

Does your department have a defined protocol for infection prophylaxis in the context of VUS/VCUG?
Yes

Has this protocol been transformed into a written standard operating procedure (SOP)?
Yes

Which department is the author of the SOP for infection prophylaxis in VUS/VCUG?
Pediatric surgery

Which department is the author of the SOP for infection prophylaxis in VUS/VCUG? [Other]

Antibiotic Prophylaxis

Are there situations/constellations in which you do not use antibiotic prophylaxis for VUS/VCUG?
No

Please specify these situations/constellations: [girl with first uncomplicated UTI without urinary tract dilation in ultrasound]
N/A

Please specify these situations/constellations: [boy with first uncomplicated UTI without urinary tract dilation in ultrasound]
N/A

Please specify these situations/constellations: [in patients with low grade urinary tract dilation (up to 2nd grade) and no UTI in medical history]
N/A

If you use antibiotic prophylaxis: which mediaction do you use? (multiple answers and subdivision into age groups possible) [Trimethoprim]
Yes

If you use antibiotic prophylaxis: which mediaction do you use? (multiple answers and subdivision into age groups possible) [Nitrofurantoin]
No

If you use antibiotic prophylaxis: which mediaction do you use? (multiple answers and subdivision into age groups possible) [Amoxicillin]
No

If you use antibiotic prophylaxis: which mediaction do you use? (multiple answers and subdivision into age groups possible) [Cefaclor]
Yes

If you use antibiotic prophylaxis: which mediaction do you use? (multiple answers and subdivision into age groups possible) [Amoxicillin/clavulanic acid]
No

If you use antibiotic prophylaxis: which mediaction do you use? (multiple answers and subdivision into age groups possible) [Other]
No

Trimethoprim: in what dosage?
therapeutic (6 mg/kg bodyweight/d)

Trimethoprim: in what dosage? [Other]

Trimethoprim: for which age group do you use trimethoprim?
Ab 3. Monat

Trimethoprim: which time schedule do you use? [Day(s) before VUS/VCUG, how many?]
No

Trimethoprim: which time schedule do you use? [Day(s) before VUS/VCUG, how many?][Comment]

Trimethoprim: which time schedule do you use? [Day of VUS/VCUG]
Yes

Trimethoprim: which time schedule do you use? [Day of VUS/VCUG][Comment]

Trimethoprim: which time schedule do you use? [Day(s) after VUS/VCUG, how many?]
No

Trimethoprim: which time schedule do you use? [Day(s) after VUS/VCUG, how many?][Comment]

Nitrofurantoin: which time schedule do you use? [Day(s) before VUS/VCUG, how many?]
N/A

Nitrofurantoin: which time schedule do you use? [Day of VUS/VCUG]
N/A

Nitrofurantoin: which time schedule do you use? [Day(s) after VUS/VCUG, how many?]
N/A

Amoxicillin: which time schedule do you use? [Day(s) before VUS/VCUG, how many?]
N/A

Amoxicillin: which time schedule do you use? [Day of VUS/VCUG]
N/A

Amoxicillin: which time schedule do you use? [Day(s) after VUS/VCUG, how many?]
N/A

Cefaclor: in what dosage?
therapeutic (40 mg/kg bodyweight/d)

Cefaclor: in what dosage? [Other]

Cefaclor: for which age group do you use cefaclor?
Bis 3 Monate

Cefaclor: which time schedule do you use? [Days(s) before VUS/VCUG, how many?]
No

Cefaclor: which time schedule do you use? [Days(s) before VUS/VCUG, how many?][Comment]

Cefaclor: which time schedule do you use? [Day of VUS/VCUG]
Yes

Cefaclor: which time schedule do you use? [Day of VUS/VCUG][Comment]

Cefaclor: which time schedule do you use? [Day(s) after VUS/VCUG, how many?]
No

Cefaclor: which time schedule do you use? [Day(s) after VUS/VCUG, how many?][Comment]

Amoxicillin/clavulanic acid: in what dosage? (Amoxicillin portion) [prophylaktic (10-20 mg/Kg bodyweight/d)]
N/A

Amoxicillin/clavulanic acid: in what dosage? (Amoxicillin portion) [double prophylactic (20-40 mg/Kg bodyweight/d)]
N/A

Amoxicillin/clavulanic acid: in what dosage? (Amoxicillin portion) [therapeutic (80-100 mg/Kg bodyweight/d)]
N/A

Amoxicillin/clavulanic acid: which time schedule do you use? [Day(s) before VUS/VCUG]
N/A

Amoxicillin/clavulanic acid: which time schedule do you use? [Day of VUS/VCUG]
N/A

Amoxicillin/clavulanic acid: which time schedule do you use? [Day(s) after VUS/VCUG]
N/A

Other: which time schedule do you use? [Day(s) before VUS/VCUG, how many?]
N/A

Other: which time schedule do you use? [Day of VUS/VCUG]
N/A

Other: which time schedule do you use? [Day(s) after VUS/VCUG, how many?]
N/A

Indication for prophylaxis

Which patients receive periinterventional antibiotic prophylaxis? (multiple answers possible) [all]
Yes

Which patients receive periinterventional antibiotic prophylaxis? (multiple answers possible) [patients post urinary tract infection/pyelonephritis/urosepsis]
N/A

Which patients receive periinterventional antibiotic prophylaxis? (multiple answers possible) [patients with urinary tract dilation in ultrasound]
N/A

Which patients receive periinterventional antibiotic prophylaxis? (multiple answers possible) [patients with prenatal urinary tract dilation]
N/A

Which patients receive periinterventional antibiotic prophylaxis? (multiple answers possible) [patients post VUR surgery]
N/A

Which patients receive periinterventional antibiotic prophylaxis? (multiple answers possible) [none]
N/A

Are there situations or clinical findings in the patient where you deviate from your prophylaxis standard (medication, dose, duration, age of the patient)? (multiple answers possible, please specify in answer box) [in case of already known vesicoureteral reflux (VUR)]
No

Are there situations or clinical findings in the patient where you deviate from your prophylaxis standard (medication, dose, duration, age of the patient)? (multiple answers possible, please specify in answer box) [in case of already known vesicoureteral reflux (VUR)][Comment]

Are there situations or clinical findings in the patient where you deviate from your prophylaxis standard (medication, dose, duration, age of the patient)? (multiple answers possible, please specify in answer box) [in patients with recurrent urinary tract infections (UTI)]
No

Are there situations or clinical findings in the patient where you deviate from your prophylaxis standard (medication, dose, duration, age of the patient)? (multiple answers possible, please specify in answer box) [in patients with recurrent urinary tract infections (UTI)][Comment]

Are there situations or clinical findings in the patient where you deviate from your prophylaxis standard (medication, dose, duration, age of the patient)? (multiple answers possible, please specify in answer box) [if multidrug-resistant organisms (MDRO) are detected in urine cultures]
Yes

Are there situations or clinical findings in the patient where you deviate from your prophylaxis standard (medication, dose, duration, age of the patient)? (multiple answers possible, please specify in answer box) [if multidrug-resistant organisms (MDRO) are detected in urine cultures][Comment]

Are there situations or clinical findings in the patient where you deviate from your prophylaxis standard (medication, dose, duration, age of the patient)? (multiple answers possible, please specify in answer box) [in newborns]
No

Are there situations or clinical findings in the patient where you deviate from your prophylaxis standard (medication, dose, duration, age of the patient)? (multiple answers possible, please specify in answer box) [in newborns][Comment]

Are there situations or clinical findings in the patient where you deviate from your prophylaxis standard (medication, dose, duration, age of the patient)? (multiple answers possible, please specify in answer box) [Other]

Are there situations or clinical findings in the patient where you deviate from your prophylaxis standard (medication, dose, duration, age of the patient)? (multiple answers possible, please specify in answer box) [Other comment]

Are there situations in which you use intravenous prophylaxis?
No

Voiding analysis

Are there differences in antibiotic prophylaxis between VUS and VCUG in your department?
No

Is a non-pathological urine strip test required in preparation for VUS/VCUG in your department?
No

Is a non-pathological urine culture required in preparation for VUS/VCUG in your department?
No

Do patients only receive post-procedure prophylaxis if vesicoureteral reflux (VUR) is detected in VUS/VCUG?
No

Do other MUS/MCU findings (e.g. urethral valves) have an influence on post-procedure prophylaxis?
No

End of survey

Thank you for taking the time to answer this survey!

May we contact you personally if we have further questions?
No

Survey response 147

Response ID
149

Date submitted
2025-03-23 14:54:53

Last page
5

Start language
de

Seed
1773317944

Date started
2025-03-23 14:46:23

Date last action
2025-03-23 14:54:53

Standard Operation Procedure

Who fills out this survey?
Pediatric Surgeon

Who fills out this survey? [Other]

In which type of hospital are you employed?
Community hospital (

In which type of hospital are you employed? [Other]

In which country do you work?
Hamburg

Does your department have a defined protocol for infection prophylaxis in the context of VUS/VCUG?
Yes

Has this protocol been transformed into a written standard operating procedure (SOP)?
No

Antibiotic Prophylaxis

Are there situations/constellations in which you do not use antibiotic prophylaxis for VUS/VCUG?
No

Please specify these situations/constellations: [girl with first uncomplicated UTI without urinary tract dilation in ultrasound]
N/A

Please specify these situations/constellations: [boy with first uncomplicated UTI without urinary tract dilation in ultrasound]
N/A

Please specify these situations/constellations: [in patients with low grade urinary tract dilation (up to 2nd grade) and no UTI in medical history]
N/A

If you use antibiotic prophylaxis: which mediaction do you use? (multiple answers and subdivision into age groups possible) [Trimethoprim]
Yes

If you use antibiotic prophylaxis: which mediaction do you use? (multiple answers and subdivision into age groups possible) [Nitrofurantoin]
Yes

If you use antibiotic prophylaxis: which mediaction do you use? (multiple answers and subdivision into age groups possible) [Amoxicillin]
No

If you use antibiotic prophylaxis: which mediaction do you use? (multiple answers and subdivision into age groups possible) [Cefaclor]
Yes

If you use antibiotic prophylaxis: which mediaction do you use? (multiple answers and subdivision into age groups possible) [Amoxicillin/clavulanic acid]
No

If you use antibiotic prophylaxis: which mediaction do you use? (multiple answers and subdivision into age groups possible) [Other]
No

Trimethoprim: in what dosage?
double prophylakctic (4 mg/kg bodyweight/d)

Trimethoprim: in what dosage? [Other]

Trimethoprim: for which age group do you use trimethoprim?
Säuglinge ab 2. Lebenshalbjahr und Kleinkinder

Trimethoprim: which time schedule do you use? [Day(s) before VUS/VCUG, how many?]
Yes

Trimethoprim: which time schedule do you use? [Day(s) before VUS/VCUG, how many?][Comment]
1

Trimethoprim: which time schedule do you use? [Day of VUS/VCUG]
Yes

Trimethoprim: which time schedule do you use? [Day of VUS/VCUG][Comment]

Trimethoprim: which time schedule do you use? [Day(s) after VUS/VCUG, how many?]
Yes

Trimethoprim: which time schedule do you use? [Day(s) after VUS/VCUG, how many?][Comment]
2

Nitrofurantoin: in what dosage?
double prophylactic (2-4 mg/kg bodyweight/d)

Nitrofurantoin: in what dosage? [Other]

Nitrofurantoin: for which age group do you use nitrofurantoin?
Schulkind

Nitrofurantoin: which time schedule do you use? [Day(s) before VUS/VCUG, how many?]
Yes

Nitrofurantoin: which time schedule do you use? [Day(s) before VUS/VCUG, how many?][Comment]
1

Nitrofurantoin: which time schedule do you use? [Day of VUS/VCUG]
Yes

Nitrofurantoin: which time schedule do you use? [Day of VUS/VCUG][Comment]

Nitrofurantoin: which time schedule do you use? [Day(s) after VUS/VCUG, how many?]
Yes

Nitrofurantoin: which time schedule do you use? [Day(s) after VUS/VCUG, how many?][Comment]
2

Amoxicillin: which time schedule do you use? [Day(s) before VUS/VCUG, how many?]
N/A

Amoxicillin: which time schedule do you use? [Day of VUS/VCUG]
N/A

Amoxicillin: which time schedule do you use? [Day(s) after VUS/VCUG, how many?]
N/A

Cefaclor: in what dosage?
double prophylactic (20 mg/kg bodyweight/d)

Cefaclor: in what dosage? [Other]

Cefaclor: for which age group do you use cefaclor?
0-6Monate

Cefaclor: which time schedule do you use? [Days(s) before VUS/VCUG, how many?]
Yes

Cefaclor: which time schedule do you use? [Days(s) before VUS/VCUG, how many?][Comment]
1

Cefaclor: which time schedule do you use? [Day of VUS/VCUG]
Yes

Cefaclor: which time schedule do you use? [Day of VUS/VCUG][Comment]

Cefaclor: which time schedule do you use? [Day(s) after VUS/VCUG, how many?]
Yes

Cefaclor: which time schedule do you use? [Day(s) after VUS/VCUG, how many?][Comment]
2

Amoxicillin/clavulanic acid: in what dosage? (Amoxicillin portion) [prophylaktic (10-20 mg/Kg bodyweight/d)]
N/A

Amoxicillin/clavulanic acid: in what dosage? (Amoxicillin portion) [double prophylactic (20-40 mg/Kg bodyweight/d)]
N/A

Amoxicillin/clavulanic acid: in what dosage? (Amoxicillin portion) [therapeutic (80-100 mg/Kg bodyweight/d)]
N/A

Amoxicillin/clavulanic acid: which time schedule do you use? [Day(s) before VUS/VCUG]
N/A

Amoxicillin/clavulanic acid: which time schedule do you use? [Day of VUS/VCUG]
N/A

Amoxicillin/clavulanic acid: which time schedule do you use? [Day(s) after VUS/VCUG]
N/A

Other: which time schedule do you use? [Day(s) before VUS/VCUG, how many?]
N/A

Other: which time schedule do you use? [Day of VUS/VCUG]
N/A

Other: which time schedule do you use? [Day(s) after VUS/VCUG, how many?]
N/A

Indication for prophylaxis

Which patients receive periinterventional antibiotic prophylaxis? (multiple answers possible) [all]
Yes

Which patients receive periinterventional antibiotic prophylaxis? (multiple answers possible) [patients post urinary tract infection/pyelonephritis/urosepsis]
N/A

Which patients receive periinterventional antibiotic prophylaxis? (multiple answers possible) [patients with urinary tract dilation in ultrasound]
N/A

Which patients receive periinterventional antibiotic prophylaxis? (multiple answers possible) [patients with prenatal urinary tract dilation]
N/A

Which patients receive periinterventional antibiotic prophylaxis? (multiple answers possible) [patients post VUR surgery]
N/A

Which patients receive periinterventional antibiotic prophylaxis? (multiple answers possible) [none]
N/A

Are there situations or clinical findings in the patient where you deviate from your prophylaxis standard (medication, dose, duration, age of the patient)? (multiple answers possible, please specify in answer box) [in case of already known vesicoureteral reflux (VUR)]
No

Are there situations or clinical findings in the patient where you deviate from your prophylaxis standard (medication, dose, duration, age of the patient)? (multiple answers possible, please specify in answer box) [in case of already known vesicoureteral reflux (VUR)][Comment]

Are there situations or clinical findings in the patient where you deviate from your prophylaxis standard (medication, dose, duration, age of the patient)? (multiple answers possible, please specify in answer box) [in patients with recurrent urinary tract infections (UTI)]
No

Are there situations or clinical findings in the patient where you deviate from your prophylaxis standard (medication, dose, duration, age of the patient)? (multiple answers possible, please specify in answer box) [in patients with recurrent urinary tract infections (UTI)][Comment]

Are there situations or clinical findings in the patient where you deviate from your prophylaxis standard (medication, dose, duration, age of the patient)? (multiple answers possible, please specify in answer box) [if multidrug-resistant organisms (MDRO) are detected in urine cultures]
Yes

Are there situations or clinical findings in the patient where you deviate from your prophylaxis standard (medication, dose, duration, age of the patient)? (multiple answers possible, please specify in answer box) [if multidrug-resistant organisms (MDRO) are detected in urine cultures][Comment]
ggf nach Resistogramm auch i.v.AB

Are there situations or clinical findings in the patient where you deviate from your prophylaxis standard (medication, dose, duration, age of the patient)? (multiple answers possible, please specify in answer box) [in newborns]
No

Are there situations or clinical findings in the patient where you deviate from your prophylaxis standard (medication, dose, duration, age of the patient)? (multiple answers possible, please specify in answer box) [in newborns][Comment]

Are there situations or clinical findings in the patient where you deviate from your prophylaxis standard (medication, dose, duration, age of the patient)? (multiple answers possible, please specify in answer box) [Other]

Are there situations or clinical findings in the patient where you deviate from your prophylaxis standard (medication, dose, duration, age of the patient)? (multiple answers possible, please specify in answer box) [Other comment]

Are there situations in which you use intravenous prophylaxis?
Yes

In which situations?
für orale AB resistente Keime im Reistogramm und nachgewiesenem VUR

Voiding analysis

Are there differences in antibiotic prophylaxis between VUS and VCUG in your department?
No

Is a non-pathological urine strip test required in preparation for VUS/VCUG in your department?
Yes

What is the maximum amount of days accepted for urine strip test to be performed prior to the exam?
1

Is a non-pathological urine culture required in preparation for VUS/VCUG in your department?
No

Do patients only receive post-procedure prophylaxis if vesicoureteral reflux (VUR) is detected in VUS/VCUG?
No

Do other MUS/MCU findings (e.g. urethral valves) have an influence on post-procedure prophylaxis?
No

End of survey

Thank you for taking the time to answer this survey!

May we contact you personally if we have further questions?
No

Survey response 148

Response ID
150

Date submitted
2025-03-24 13:29:33

Last page
5

Start language
de

Seed
182364528

Date started
2025-03-24 13:26:33

Date last action
2025-03-24 13:29:33

Standard Operation Procedure

Who fills out this survey?
Pediatric Surgeon

Who fills out this survey? [Other]

In which type of hospital are you employed?
Community hospital (>50 beds for pediatric patients)

In which type of hospital are you employed? [Other]

In which country do you work?
Berlin

Does your department have a defined protocol for infection prophylaxis in the context of VUS/VCUG?
No

Has this protocol been transformed into a written standard operating procedure (SOP)?
N/A

Antibiotic Prophylaxis

Are there situations/constellations in which you do not use antibiotic prophylaxis for VUS/VCUG?
Yes

Please specify these situations/constellations: [girl with first uncomplicated UTI without urinary tract dilation in ultrasound]
No

Please specify these situations/constellations: [boy with first uncomplicated UTI without urinary tract dilation in ultrasound]
No

Please specify these situations/constellations: [in patients with low grade urinary tract dilation (up to 2nd grade) and no UTI in medical history]
No

Please specify these situations/constellations: [Other]
immer

If you use antibiotic prophylaxis: which mediaction do you use? (multiple answers and subdivision into age groups possible) [Trimethoprim]
No

If you use antibiotic prophylaxis: which mediaction do you use? (multiple answers and subdivision into age groups possible) [Nitrofurantoin]
No

If you use antibiotic prophylaxis: which mediaction do you use? (multiple answers and subdivision into age groups possible) [Amoxicillin]
No

If you use antibiotic prophylaxis: which mediaction do you use? (multiple answers and subdivision into age groups possible) [Cefaclor]
No

If you use antibiotic prophylaxis: which mediaction do you use? (multiple answers and subdivision into age groups possible) [Amoxicillin/clavulanic acid]
No

If you use antibiotic prophylaxis: which mediaction do you use? (multiple answers and subdivision into age groups possible) [Other]
No

Trimethoprim: which time schedule do you use? [Day(s) before VUS/VCUG, how many?]
N/A

Trimethoprim: which time schedule do you use? [Day of VUS/VCUG]
N/A

Trimethoprim: which time schedule do you use? [Day(s) after VUS/VCUG, how many?]
N/A

Nitrofurantoin: which time schedule do you use? [Day(s) before VUS/VCUG, how many?]
N/A

Nitrofurantoin: which time schedule do you use? [Day of VUS/VCUG]
N/A

Nitrofurantoin: which time schedule do you use? [Day(s) after VUS/VCUG, how many?]
N/A

Amoxicillin: which time schedule do you use? [Day(s) before VUS/VCUG, how many?]
N/A

Amoxicillin: which time schedule do you use? [Day of VUS/VCUG]
N/A

Amoxicillin: which time schedule do you use? [Day(s) after VUS/VCUG, how many?]
N/A

Cefaclor: which time schedule do you use? [Days(s) before VUS/VCUG, how many?]
N/A

Cefaclor: which time schedule do you use? [Day of VUS/VCUG]
N/A

Cefaclor: which time schedule do you use? [Day(s) after VUS/VCUG, how many?]
N/A

Amoxicillin/clavulanic acid: in what dosage? (Amoxicillin portion) [prophylaktic (10-20 mg/Kg bodyweight/d)]
N/A

Amoxicillin/clavulanic acid: in what dosage? (Amoxicillin portion) [double prophylactic (20-40 mg/Kg bodyweight/d)]
N/A

Amoxicillin/clavulanic acid: in what dosage? (Amoxicillin portion) [therapeutic (80-100 mg/Kg bodyweight/d)]
N/A

Amoxicillin/clavulanic acid: which time schedule do you use? [Day(s) before VUS/VCUG]
N/A

Amoxicillin/clavulanic acid: which time schedule do you use? [Day of VUS/VCUG]
N/A

Amoxicillin/clavulanic acid: which time schedule do you use? [Day(s) after VUS/VCUG]
N/A

Other: which time schedule do you use? [Day(s) before VUS/VCUG, how many?]
N/A

Other: which time schedule do you use? [Day of VUS/VCUG]
N/A

Other: which time schedule do you use? [Day(s) after VUS/VCUG, how many?]
N/A

Indication for prophylaxis

Which patients receive periinterventional antibiotic prophylaxis? (multiple answers possible) [all]
No

Which patients receive periinterventional antibiotic prophylaxis? (multiple answers possible) [patients post urinary tract infection/pyelonephritis/urosepsis]
No

Which patients receive periinterventional antibiotic prophylaxis? (multiple answers possible) [patients with urinary tract dilation in ultrasound]
No

Which patients receive periinterventional antibiotic prophylaxis? (multiple answers possible) [patients with prenatal urinary tract dilation]
No

Which patients receive periinterventional antibiotic prophylaxis? (multiple answers possible) [patients post VUR surgery]
No

Which patients receive periinterventional antibiotic prophylaxis? (multiple answers possible) [none]
Yes

Which patients receive periinterventional antibiotic prophylaxis? (multiple answers possible) [Other]

Are there situations or clinical findings in the patient where you deviate from your prophylaxis standard (medication, dose, duration, age of the patient)? (multiple answers possible, please specify in answer box) [in case of already known vesicoureteral reflux (VUR)]
No

Are there situations or clinical findings in the patient where you deviate from your prophylaxis standard (medication, dose, duration, age of the patient)? (multiple answers possible, please specify in answer box) [in case of already known vesicoureteral reflux (VUR)][Comment]

Are there situations or clinical findings in the patient where you deviate from your prophylaxis standard (medication, dose, duration, age of the patient)? (multiple answers possible, please specify in answer box) [in patients with recurrent urinary tract infections (UTI)]
No

Are there situations or clinical findings in the patient where you deviate from your prophylaxis standard (medication, dose, duration, age of the patient)? (multiple answers possible, please specify in answer box) [in patients with recurrent urinary tract infections (UTI)][Comment]

Are there situations or clinical findings in the patient where you deviate from your prophylaxis standard (medication, dose, duration, age of the patient)? (multiple answers possible, please specify in answer box) [if multidrug-resistant organisms (MDRO) are detected in urine cultures]
No

Are there situations or clinical findings in the patient where you deviate from your prophylaxis standard (medication, dose, duration, age of the patient)? (multiple answers possible, please specify in answer box) [if multidrug-resistant organisms (MDRO) are detected in urine cultures][Comment]

Are there situations or clinical findings in the patient where you deviate from your prophylaxis standard (medication, dose, duration, age of the patient)? (multiple answers possible, please specify in answer box) [in newborns]
No

Are there situations or clinical findings in the patient where you deviate from your prophylaxis standard (medication, dose, duration, age of the patient)? (multiple answers possible, please specify in answer box) [in newborns][Comment]

Are there situations or clinical findings in the patient where you deviate from your prophylaxis standard (medication, dose, duration, age of the patient)? (multiple answers possible, please specify in answer box) [Other]
keine

Are there situations or clinical findings in the patient where you deviate from your prophylaxis standard (medication, dose, duration, age of the patient)? (multiple answers possible, please specify in answer box) [Other comment]

Are there situations in which you use intravenous prophylaxis?
No

Voiding analysis

Are there differences in antibiotic prophylaxis between VUS and VCUG in your department?
No

Is a non-pathological urine strip test required in preparation for VUS/VCUG in your department?
No

Is a non-pathological urine culture required in preparation for VUS/VCUG in your department?
No

Do patients only receive post-procedure prophylaxis if vesicoureteral reflux (VUR) is detected in VUS/VCUG?
No

Do other MUS/MCU findings (e.g. urethral valves) have an influence on post-procedure prophylaxis?
No

End of survey

Thank you for taking the time to answer this survey!

May we contact you personally if we have further questions?
No

Survey response 149

Response ID
151

Last page
1

Start language
en

Seed
1580939475

Date started
2025-03-25 08:42:15

Date last action
2025-03-25 08:42:58

Standard Operation Procedure

Who fills out this survey?
Pediatric Radiologist

Who fills out this survey? [Other]

In which type of hospital are you employed?
University Hospital

In which type of hospital are you employed? [Other]

In which country do you work?
Ireland

Does your department have a defined protocol for infection prophylaxis in the context of VUS/VCUG?
Yes

Has this protocol been transformed into a written standard operating procedure (SOP)?
Yes

Which department is the author of the SOP for infection prophylaxis in VUS/VCUG?
Pediatric surgery

Which department is the author of the SOP for infection prophylaxis in VUS/VCUG? [Other]

Antibiotic Prophylaxis

Are there situations/constellations in which you do not use antibiotic prophylaxis for VUS/VCUG?
N/A

Please specify these situations/constellations: [girl with first uncomplicated UTI without urinary tract dilation in ultrasound]
N/A

Please specify these situations/constellations: [boy with first uncomplicated UTI without urinary tract dilation in ultrasound]
N/A

Please specify these situations/constellations: [in patients with low grade urinary tract dilation (up to 2nd grade) and no UTI in medical history]
N/A

If you use antibiotic prophylaxis: which mediaction do you use? (multiple answers and subdivision into age groups possible) [Trimethoprim]
N/A

If you use antibiotic prophylaxis: which mediaction do you use? (multiple answers and subdivision into age groups possible) [Nitrofurantoin]
N/A

If you use antibiotic prophylaxis: which mediaction do you use? (multiple answers and subdivision into age groups possible) [Amoxicillin]
N/A

If you use antibiotic prophylaxis: which mediaction do you use? (multiple answers and subdivision into age groups possible) [Cefaclor]
N/A

If you use antibiotic prophylaxis: which mediaction do you use? (multiple answers and subdivision into age groups possible) [Amoxicillin/clavulanic acid]
N/A

If you use antibiotic prophylaxis: which mediaction do you use? (multiple answers and subdivision into age groups possible) [Other]
N/A

Trimethoprim: which time schedule do you use? [Day(s) before VUS/VCUG, how many?]
N/A

Trimethoprim: which time schedule do you use? [Day of VUS/VCUG]
N/A

Trimethoprim: which time schedule do you use? [Day(s) after VUS/VCUG, how many?]
N/A

Nitrofurantoin: which time schedule do you use? [Day(s) before VUS/VCUG, how many?]
N/A

Nitrofurantoin: which time schedule do you use? [Day of VUS/VCUG]
N/A

Nitrofurantoin: which time schedule do you use? [Day(s) after VUS/VCUG, how many?]
N/A

Amoxicillin: which time schedule do you use? [Day(s) before VUS/VCUG, how many?]
N/A

Amoxicillin: which time schedule do you use? [Day of VUS/VCUG]
N/A

Amoxicillin: which time schedule do you use? [Day(s) after VUS/VCUG, how many?]
N/A

Cefaclor: which time schedule do you use? [Days(s) before VUS/VCUG, how many?]
N/A

Cefaclor: which time schedule do you use? [Day of VUS/VCUG]
N/A

Cefaclor: which time schedule do you use? [Day(s) after VUS/VCUG, how many?]
N/A

Amoxicillin/clavulanic acid: in what dosage? (Amoxicillin portion) [prophylaktic (10-20 mg/Kg bodyweight/d)]
N/A

Amoxicillin/clavulanic acid: in what dosage? (Amoxicillin portion) [double prophylactic (20-40 mg/Kg bodyweight/d)]
N/A

Amoxicillin/clavulanic acid: in what dosage? (Amoxicillin portion) [therapeutic (80-100 mg/Kg bodyweight/d)]
N/A

Amoxicillin/clavulanic acid: which time schedule do you use? [Day(s) before VUS/VCUG]
N/A

Amoxicillin/clavulanic acid: which time schedule do you use? [Day of VUS/VCUG]
N/A

Amoxicillin/clavulanic acid: which time schedule do you use? [Day(s) after VUS/VCUG]
N/A

Other: which time schedule do you use? [Day(s) before VUS/VCUG, how many?]
N/A

Other: which time schedule do you use? [Day of VUS/VCUG]
N/A

Other: which time schedule do you use? [Day(s) after VUS/VCUG, how many?]
N/A

Indication for prophylaxis

Which patients receive periinterventional antibiotic prophylaxis? (multiple answers possible) [all]
N/A

Which patients receive periinterventional antibiotic prophylaxis? (multiple answers possible) [patients post urinary tract infection/pyelonephritis/urosepsis]
N/A

Which patients receive periinterventional antibiotic prophylaxis? (multiple answers possible) [patients with urinary tract dilation in ultrasound]
N/A

Which patients receive periinterventional antibiotic prophylaxis? (multiple answers possible) [patients with prenatal urinary tract dilation]
N/A

Which patients receive periinterventional antibiotic prophylaxis? (multiple answers possible) [patients post VUR surgery]
N/A

Which patients receive periinterventional antibiotic prophylaxis? (multiple answers possible) [none]
N/A

Are there situations or clinical findings in the patient where you deviate from your prophylaxis standard (medication, dose, duration, age of the patient)? (multiple answers possible, please specify in answer box) [in case of already known vesicoureteral reflux (VUR)]
N/A

Are there situations or clinical findings in the patient where you deviate from your prophylaxis standard (medication, dose, duration, age of the patient)? (multiple answers possible, please specify in answer box) [in patients with recurrent urinary tract infections (UTI)]
N/A

Are there situations or clinical findings in the patient where you deviate from your prophylaxis standard (medication, dose, duration, age of the patient)? (multiple answers possible, please specify in answer box) [if multidrug-resistant organisms (MDRO) are detected in urine cultures]
N/A

Are there situations or clinical findings in the patient where you deviate from your prophylaxis standard (medication, dose, duration, age of the patient)? (multiple answers possible, please specify in answer box) [in newborns]
N/A

Are there situations in which you use intravenous prophylaxis?
N/A

Voiding analysis

Are there differences in antibiotic prophylaxis between VUS and VCUG in your department?
N/A

Is a non-pathological urine strip test required in preparation for VUS/VCUG in your department?
N/A

Is a non-pathological urine culture required in preparation for VUS/VCUG in your department?
N/A

Do patients only receive post-procedure prophylaxis if vesicoureteral reflux (VUR) is detected in VUS/VCUG?
N/A

Do other MUS/MCU findings (e.g. urethral valves) have an influence on post-procedure prophylaxis?
N/A

End of survey

May we contact you personally if we have further questions?
N/A

Survey response 150

Response ID
152

Date submitted
2025-03-25 08:47:32

Last page
5

Start language
en

Seed
813316695

Date started
2025-03-25 08:43:29

Date last action
2025-03-25 08:47:32

Standard Operation Procedure

Who fills out this survey?
Radiologist

Who fills out this survey? [Other]

In which type of hospital are you employed?
Community hospital (>50 beds for pediatric patients)

In which type of hospital are you employed? [Other]

In which country do you work?
Norway

Does your department have a defined protocol for infection prophylaxis in the context of VUS/VCUG?
Yes

Has this protocol been transformed into a written standard operating procedure (SOP)?
No

Antibiotic Prophylaxis

Are there situations/constellations in which you do not use antibiotic prophylaxis for VUS/VCUG?
No

Please specify these situations/constellations: [girl with first uncomplicated UTI without urinary tract dilation in ultrasound]
N/A

Please specify these situations/constellations: [boy with first uncomplicated UTI without urinary tract dilation in ultrasound]
N/A

Please specify these situations/constellations: [in patients with low grade urinary tract dilation (up to 2nd grade) and no UTI in medical history]
N/A

If you use antibiotic prophylaxis: which mediaction do you use? (multiple answers and subdivision into age groups possible) [Trimethoprim]
No

If you use antibiotic prophylaxis: which mediaction do you use? (multiple answers and subdivision into age groups possible) [Nitrofurantoin]
No

If you use antibiotic prophylaxis: which mediaction do you use? (multiple answers and subdivision into age groups possible) [Amoxicillin]
No

If you use antibiotic prophylaxis: which mediaction do you use? (multiple answers and subdivision into age groups possible) [Cefaclor]
No

If you use antibiotic prophylaxis: which mediaction do you use? (multiple answers and subdivision into age groups possible) [Amoxicillin/clavulanic acid]
No

If you use antibiotic prophylaxis: which mediaction do you use? (multiple answers and subdivision into age groups possible) [Other]
No

Trimethoprim: which time schedule do you use? [Day(s) before VUS/VCUG, how many?]
N/A

Trimethoprim: which time schedule do you use? [Day of VUS/VCUG]
N/A

Trimethoprim: which time schedule do you use? [Day(s) after VUS/VCUG, how many?]
N/A

Nitrofurantoin: which time schedule do you use? [Day(s) before VUS/VCUG, how many?]
N/A

Nitrofurantoin: which time schedule do you use? [Day of VUS/VCUG]
N/A

Nitrofurantoin: which time schedule do you use? [Day(s) after VUS/VCUG, how many?]
N/A

Amoxicillin: which time schedule do you use? [Day(s) before VUS/VCUG, how many?]
N/A

Amoxicillin: which time schedule do you use? [Day of VUS/VCUG]
N/A

Amoxicillin: which time schedule do you use? [Day(s) after VUS/VCUG, how many?]
N/A

Cefaclor: which time schedule do you use? [Days(s) before VUS/VCUG, how many?]
N/A

Cefaclor: which time schedule do you use? [Day of VUS/VCUG]
N/A

Cefaclor: which time schedule do you use? [Day(s) after VUS/VCUG, how many?]
N/A

Amoxicillin/clavulanic acid: in what dosage? (Amoxicillin portion) [prophylaktic (10-20 mg/Kg bodyweight/d)]
N/A

Amoxicillin/clavulanic acid: in what dosage? (Amoxicillin portion) [double prophylactic (20-40 mg/Kg bodyweight/d)]
N/A

Amoxicillin/clavulanic acid: in what dosage? (Amoxicillin portion) [therapeutic (80-100 mg/Kg bodyweight/d)]
N/A

Amoxicillin/clavulanic acid: which time schedule do you use? [Day(s) before VUS/VCUG]
N/A

Amoxicillin/clavulanic acid: which time schedule do you use? [Day of VUS/VCUG]
N/A

Amoxicillin/clavulanic acid: which time schedule do you use? [Day(s) after VUS/VCUG]
N/A

Other: which time schedule do you use? [Day(s) before VUS/VCUG, how many?]
N/A

Other: which time schedule do you use? [Day of VUS/VCUG]
N/A

Other: which time schedule do you use? [Day(s) after VUS/VCUG, how many?]
N/A

Indication for prophylaxis

Which patients receive periinterventional antibiotic prophylaxis? (multiple answers possible) [all]
Yes

Which patients receive periinterventional antibiotic prophylaxis? (multiple answers possible) [patients post urinary tract infection/pyelonephritis/urosepsis]
N/A

Which patients receive periinterventional antibiotic prophylaxis? (multiple answers possible) [patients with urinary tract dilation in ultrasound]
N/A

Which patients receive periinterventional antibiotic prophylaxis? (multiple answers possible) [patients with prenatal urinary tract dilation]
N/A

Which patients receive periinterventional antibiotic prophylaxis? (multiple answers possible) [patients post VUR surgery]
N/A

Which patients receive periinterventional antibiotic prophylaxis? (multiple answers possible) [none]
N/A

Are there situations or clinical findings in the patient where you deviate from your prophylaxis standard (medication, dose, duration, age of the patient)? (multiple answers possible, please specify in answer box) [in case of already known vesicoureteral reflux (VUR)]
Yes

Are there situations or clinical findings in the patient where you deviate from your prophylaxis standard (medication, dose, duration, age of the patient)? (multiple answers possible, please specify in answer box) [in case of already known vesicoureteral reflux (VUR)][Comment]
If they are taking prophylactic medication routinely

Are there situations or clinical findings in the patient where you deviate from your prophylaxis standard (medication, dose, duration, age of the patient)? (multiple answers possible, please specify in answer box) [in patients with recurrent urinary tract infections (UTI)]
No

Are there situations or clinical findings in the patient where you deviate from your prophylaxis standard (medication, dose, duration, age of the patient)? (multiple answers possible, please specify in answer box) [in patients with recurrent urinary tract infections (UTI)][Comment]

Are there situations or clinical findings in the patient where you deviate from your prophylaxis standard (medication, dose, duration, age of the patient)? (multiple answers possible, please specify in answer box) [if multidrug-resistant organisms (MDRO) are detected in urine cultures]
No

Are there situations or clinical findings in the patient where you deviate from your prophylaxis standard (medication, dose, duration, age of the patient)? (multiple answers possible, please specify in answer box) [if multidrug-resistant organisms (MDRO) are detected in urine cultures][Comment]

Are there situations or clinical findings in the patient where you deviate from your prophylaxis standard (medication, dose, duration, age of the patient)? (multiple answers possible, please specify in answer box) [in newborns]
No

Are there situations or clinical findings in the patient where you deviate from your prophylaxis standard (medication, dose, duration, age of the patient)? (multiple answers possible, please specify in answer box) [in newborns][Comment]

Are there situations or clinical findings in the patient where you deviate from your prophylaxis standard (medication, dose, duration, age of the patient)? (multiple answers possible, please specify in answer box) [Other]

Are there situations or clinical findings in the patient where you deviate from your prophylaxis standard (medication, dose, duration, age of the patient)? (multiple answers possible, please specify in answer box) [Other comment]

Are there situations in which you use intravenous prophylaxis?
No

Voiding analysis

Are there differences in antibiotic prophylaxis between VUS and VCUG in your department?
No

Is a non-pathological urine strip test required in preparation for VUS/VCUG in your department?
No

Is a non-pathological urine culture required in preparation for VUS/VCUG in your department?
No

Do patients only receive post-procedure prophylaxis if vesicoureteral reflux (VUR) is detected in VUS/VCUG?
No

Do other MUS/MCU findings (e.g. urethral valves) have an influence on post-procedure prophylaxis?
No

End of survey

Thank you for taking the time to answer this survey!

May we contact you personally if we have further questions?
Yes

E-mail address:
Maria.myhre.bjorndal@sshf.no

Survey response 151

Response ID
153

Date submitted
2025-03-25 08:50:40

Last page
5

Start language
en

Seed
186350341

Date started
2025-03-25 08:43:56

Date last action
2025-03-25 08:50:40

Standard Operation Procedure

Who fills out this survey?
Radiologist

Who fills out this survey? [Other]

In which type of hospital are you employed?
Community hospital (

In which type of hospital are you employed? [Other]

In which country do you work?
Greece

Does your department have a defined protocol for infection prophylaxis in the context of VUS/VCUG?
Yes

Has this protocol been transformed into a written standard operating procedure (SOP)?
Yes

Which department is the author of the SOP for infection prophylaxis in VUS/VCUG?
Pediatrics

Which department is the author of the SOP for infection prophylaxis in VUS/VCUG? [Other]

Antibiotic Prophylaxis

Are there situations/constellations in which you do not use antibiotic prophylaxis for VUS/VCUG?
No

Please specify these situations/constellations: [girl with first uncomplicated UTI without urinary tract dilation in ultrasound]
N/A

Please specify these situations/constellations: [boy with first uncomplicated UTI without urinary tract dilation in ultrasound]
N/A

Please specify these situations/constellations: [in patients with low grade urinary tract dilation (up to 2nd grade) and no UTI in medical history]
N/A

If you use antibiotic prophylaxis: which mediaction do you use? (multiple answers and subdivision into age groups possible) [Trimethoprim]
No

If you use antibiotic prophylaxis: which mediaction do you use? (multiple answers and subdivision into age groups possible) [Nitrofurantoin]
Yes

If you use antibiotic prophylaxis: which mediaction do you use? (multiple answers and subdivision into age groups possible) [Amoxicillin]
No

If you use antibiotic prophylaxis: which mediaction do you use? (multiple answers and subdivision into age groups possible) [Cefaclor]
Yes

If you use antibiotic prophylaxis: which mediaction do you use? (multiple answers and subdivision into age groups possible) [Amoxicillin/clavulanic acid]
No

If you use antibiotic prophylaxis: which mediaction do you use? (multiple answers and subdivision into age groups possible) [Other]
No

Trimethoprim: which time schedule do you use? [Day(s) before VUS/VCUG, how many?]
N/A

Trimethoprim: which time schedule do you use? [Day of VUS/VCUG]
N/A

Trimethoprim: which time schedule do you use? [Day(s) after VUS/VCUG, how many?]
N/A

Nitrofurantoin: in what dosage?
therapeutic (5 mg/kg bodyweight/d)

Nitrofurantoin: in what dosage? [Other]

Nitrofurantoin: for which age group do you use nitrofurantoin?
2-12m

Nitrofurantoin: which time schedule do you use? [Day(s) before VUS/VCUG, how many?]
Yes

Nitrofurantoin: which time schedule do you use? [Day(s) before VUS/VCUG, how many?][Comment]
2

Nitrofurantoin: which time schedule do you use? [Day of VUS/VCUG]
No

Nitrofurantoin: which time schedule do you use? [Day of VUS/VCUG][Comment]

Nitrofurantoin: which time schedule do you use? [Day(s) after VUS/VCUG, how many?]
Yes

Nitrofurantoin: which time schedule do you use? [Day(s) after VUS/VCUG, how many?][Comment]
2

Amoxicillin: which time schedule do you use? [Day(s) before VUS/VCUG, how many?]
N/A

Amoxicillin: which time schedule do you use? [Day of VUS/VCUG]
N/A

Amoxicillin: which time schedule do you use? [Day(s) after VUS/VCUG, how many?]
N/A

Cefaclor: in what dosage?
therapeutic (40 mg/kg bodyweight/d)

Cefaclor: in what dosage? [Other]

Cefaclor: for which age group do you use cefaclor?
6-36m

Cefaclor: which time schedule do you use? [Days(s) before VUS/VCUG, how many?]
Yes

Cefaclor: which time schedule do you use? [Days(s) before VUS/VCUG, how many?][Comment]
2

Cefaclor: which time schedule do you use? [Day of VUS/VCUG]
No

Cefaclor: which time schedule do you use? [Day of VUS/VCUG][Comment]

Cefaclor: which time schedule do you use? [Day(s) after VUS/VCUG, how many?]
Yes

Cefaclor: which time schedule do you use? [Day(s) after VUS/VCUG, how many?][Comment]
2

Amoxicillin/clavulanic acid: in what dosage? (Amoxicillin portion) [prophylaktic (10-20 mg/Kg bodyweight/d)]
N/A

Amoxicillin/clavulanic acid: in what dosage? (Amoxicillin portion) [double prophylactic (20-40 mg/Kg bodyweight/d)]
N/A

Amoxicillin/clavulanic acid: in what dosage? (Amoxicillin portion) [therapeutic (80-100 mg/Kg bodyweight/d)]
N/A

Amoxicillin/clavulanic acid: which time schedule do you use? [Day(s) before VUS/VCUG]
N/A

Amoxicillin/clavulanic acid: which time schedule do you use? [Day of VUS/VCUG]
N/A

Amoxicillin/clavulanic acid: which time schedule do you use? [Day(s) after VUS/VCUG]
N/A

Other: which time schedule do you use? [Day(s) before VUS/VCUG, how many?]
N/A

Other: which time schedule do you use? [Day of VUS/VCUG]
N/A

Other: which time schedule do you use? [Day(s) after VUS/VCUG, how many?]
N/A

Indication for prophylaxis

Which patients receive periinterventional antibiotic prophylaxis? (multiple answers possible) [all]
No

Which patients receive periinterventional antibiotic prophylaxis? (multiple answers possible) [patients post urinary tract infection/pyelonephritis/urosepsis]
Yes

Which patients receive periinterventional antibiotic prophylaxis? (multiple answers possible) [patients with urinary tract dilation in ultrasound]
Yes

Which patients receive periinterventional antibiotic prophylaxis? (multiple answers possible) [patients with prenatal urinary tract dilation]
Yes

Which patients receive periinterventional antibiotic prophylaxis? (multiple answers possible) [patients post VUR surgery]
Yes

Which patients receive periinterventional antibiotic prophylaxis? (multiple answers possible) [none]
No

Which patients receive periinterventional antibiotic prophylaxis? (multiple answers possible) [Other]

patients status post urinary tract infection/pyelonephritis/urosepsis: what is the minimum time between infection and VUS/VCUG? (in days)
10d

In patients with urinary tract dilation in ultrasound: from what degree on? Image from Beetz R, Bokenkamp A, Brandis M et al (2001) Diagnosis of congenital dilatation of the urinary tract. Consensus Group of the Pediatric Nephrology Working Society in cooperation with the Pediatric Urology Working Group of the German Society of Urology and with the Pediatric Urology Working Society in the Germany Society of Pediatric Surgery. Urologe A 40:495–507 (quiz 8–9)
grade 2

In patients with prenatal urinary tract dilation: from what degree on? Image from Beetz R, Bokenkamp A, Brandis M et al (2001) Diagnosis of congenital dilatation of the urinary tract. Consensus Group of the Pediatric Nephrology Working Society in cooperation with the Pediatric Urology Working Group of the German Society of Urology and with the Pediatric Urology Working Society in the Germany Society of Pediatric Surgery. Urologe A 40:495–507 (quiz 8–9)
grade 2

Are there situations or clinical findings in the patient where you deviate from your prophylaxis standard (medication, dose, duration, age of the patient)? (multiple answers possible, please specify in answer box) [in case of already known vesicoureteral reflux (VUR)]
Yes

Are there situations or clinical findings in the patient where you deviate from your prophylaxis standard (medication, dose, duration, age of the patient)? (multiple answers possible, please specify in answer box) [in case of already known vesicoureteral reflux (VUR)][Comment]

Are there situations or clinical findings in the patient where you deviate from your prophylaxis standard (medication, dose, duration, age of the patient)? (multiple answers possible, please specify in answer box) [in patients with recurrent urinary tract infections (UTI)]
No

Are there situations or clinical findings in the patient where you deviate from your prophylaxis standard (medication, dose, duration, age of the patient)? (multiple answers possible, please specify in answer box) [in patients with recurrent urinary tract infections (UTI)][Comment]

Are there situations or clinical findings in the patient where you deviate from your prophylaxis standard (medication, dose, duration, age of the patient)? (multiple answers possible, please specify in answer box) [if multidrug-resistant organisms (MDRO) are detected in urine cultures]
Yes

Are there situations or clinical findings in the patient where you deviate from your prophylaxis standard (medication, dose, duration, age of the patient)? (multiple answers possible, please specify in answer box) [if multidrug-resistant organisms (MDRO) are detected in urine cultures][Comment]

Are there situations or clinical findings in the patient where you deviate from your prophylaxis standard (medication, dose, duration, age of the patient)? (multiple answers possible, please specify in answer box) [in newborns]
No

Are there situations or clinical findings in the patient where you deviate from your prophylaxis standard (medication, dose, duration, age of the patient)? (multiple answers possible, please specify in answer box) [in newborns][Comment]

Are there situations or clinical findings in the patient where you deviate from your prophylaxis standard (medication, dose, duration, age of the patient)? (multiple answers possible, please specify in answer box) [Other]

Are there situations or clinical findings in the patient where you deviate from your prophylaxis standard (medication, dose, duration, age of the patient)? (multiple answers possible, please specify in answer box) [Other comment]

Are there situations in which you use intravenous prophylaxis?
No

Voiding analysis

Are there differences in antibiotic prophylaxis between VUS and VCUG in your department?
No

Is a non-pathological urine strip test required in preparation for VUS/VCUG in your department?
Yes

What is the maximum amount of days accepted for urine strip test to be performed prior to the exam?
7d

Is a non-pathological urine culture required in preparation for VUS/VCUG in your department?
Yes

What is the maximum amount of days accepted for urine culture to be performed prior to the exam?
10d

Do patients only receive post-procedure prophylaxis if vesicoureteral reflux (VUR) is detected in VUS/VCUG?
No

Do other MUS/MCU findings (e.g. urethral valves) have an influence on post-procedure prophylaxis?
No

End of survey

Thank you for taking the time to answer this survey!

May we contact you personally if we have further questions?
Yes

E-mail address:
papadaki1@yahoo.com

Survey response 152

Response ID
154

Date submitted
2025-03-25 09:29:37

Last page
5

Start language
en

Seed
919784384

Date started
2025-03-25 08:44:01

Date last action
2025-03-25 09:29:37

Standard Operation Procedure

Who fills out this survey?
Radiologist

Who fills out this survey? [Other]

In which type of hospital are you employed?
University Hospital

In which type of hospital are you employed? [Other]

In which country do you work?
France

Does your department have a defined protocol for infection prophylaxis in the context of VUS/VCUG?
Yes

Has this protocol been transformed into a written standard operating procedure (SOP)?
Yes

Which department is the author of the SOP for infection prophylaxis in VUS/VCUG?
Other

Which department is the author of the SOP for infection prophylaxis in VUS/VCUG? [Other]
multidisciplinary

Antibiotic Prophylaxis

Are there situations/constellations in which you do not use antibiotic prophylaxis for VUS/VCUG?
Yes

Please specify these situations/constellations: [girl with first uncomplicated UTI without urinary tract dilation in ultrasound]
No

Please specify these situations/constellations: [boy with first uncomplicated UTI without urinary tract dilation in ultrasound]
No

Please specify these situations/constellations: [in patients with low grade urinary tract dilation (up to 2nd grade) and no UTI in medical history]
No

Please specify these situations/constellations: [Other]
in all situation

If you use antibiotic prophylaxis: which mediaction do you use? (multiple answers and subdivision into age groups possible) [Trimethoprim]
No

If you use antibiotic prophylaxis: which mediaction do you use? (multiple answers and subdivision into age groups possible) [Nitrofurantoin]
No

If you use antibiotic prophylaxis: which mediaction do you use? (multiple answers and subdivision into age groups possible) [Amoxicillin]
No

If you use antibiotic prophylaxis: which mediaction do you use? (multiple answers and subdivision into age groups possible) [Cefaclor]
No

If you use antibiotic prophylaxis: which mediaction do you use? (multiple answers and subdivision into age groups possible) [Amoxicillin/clavulanic acid]
No

If you use antibiotic prophylaxis: which mediaction do you use? (multiple answers and subdivision into age groups possible) [Other]
No

Trimethoprim: which time schedule do you use? [Day(s) before VUS/VCUG, how many?]
N/A

Trimethoprim: which time schedule do you use? [Day of VUS/VCUG]
N/A

Trimethoprim: which time schedule do you use? [Day(s) after VUS/VCUG, how many?]
N/A

Nitrofurantoin: which time schedule do you use? [Day(s) before VUS/VCUG, how many?]
N/A

Nitrofurantoin: which time schedule do you use? [Day of VUS/VCUG]
N/A

Nitrofurantoin: which time schedule do you use? [Day(s) after VUS/VCUG, how many?]
N/A

Amoxicillin: which time schedule do you use? [Day(s) before VUS/VCUG, how many?]
N/A

Amoxicillin: which time schedule do you use? [Day of VUS/VCUG]
N/A

Amoxicillin: which time schedule do you use? [Day(s) after VUS/VCUG, how many?]
N/A

Cefaclor: which time schedule do you use? [Days(s) before VUS/VCUG, how many?]
N/A

Cefaclor: which time schedule do you use? [Day of VUS/VCUG]
N/A

Cefaclor: which time schedule do you use? [Day(s) after VUS/VCUG, how many?]
N/A

Amoxicillin/clavulanic acid: in what dosage? (Amoxicillin portion) [prophylaktic (10-20 mg/Kg bodyweight/d)]
N/A

Amoxicillin/clavulanic acid: in what dosage? (Amoxicillin portion) [double prophylactic (20-40 mg/Kg bodyweight/d)]
N/A

Amoxicillin/clavulanic acid: in what dosage? (Amoxicillin portion) [therapeutic (80-100 mg/Kg bodyweight/d)]
N/A

Amoxicillin/clavulanic acid: which time schedule do you use? [Day(s) before VUS/VCUG]
N/A

Amoxicillin/clavulanic acid: which time schedule do you use? [Day of VUS/VCUG]
N/A

Amoxicillin/clavulanic acid: which time schedule do you use? [Day(s) after VUS/VCUG]
N/A

Other: which time schedule do you use? [Day(s) before VUS/VCUG, how many?]
N/A

Other: which time schedule do you use? [Day of VUS/VCUG]
N/A

Other: which time schedule do you use? [Day(s) after VUS/VCUG, how many?]
N/A

Indication for prophylaxis

Which patients receive periinterventional antibiotic prophylaxis? (multiple answers possible) [all]
No

Which patients receive periinterventional antibiotic prophylaxis? (multiple answers possible) [patients post urinary tract infection/pyelonephritis/urosepsis]
No

Which patients receive periinterventional antibiotic prophylaxis? (multiple answers possible) [patients with urinary tract dilation in ultrasound]
No

Which patients receive periinterventional antibiotic prophylaxis? (multiple answers possible) [patients with prenatal urinary tract dilation]
No

Which patients receive periinterventional antibiotic prophylaxis? (multiple answers possible) [patients post VUR surgery]
No

Which patients receive periinterventional antibiotic prophylaxis? (multiple answers possible) [none]
Yes

Which patients receive periinterventional antibiotic prophylaxis? (multiple answers possible) [Other]
patients already under antibiotics are eligible for VCUGare eligible

Are there situations or clinical findings in the patient where you deviate from your prophylaxis standard (medication, dose, duration, age of the patient)? (multiple answers possible, please specify in answer box) [in case of already known vesicoureteral reflux (VUR)]
Yes

Are there situations or clinical findings in the patient where you deviate from your prophylaxis standard (medication, dose, duration, age of the patient)? (multiple answers possible, please specify in answer box) [in case of already known vesicoureteral reflux (VUR)][Comment]
No prophylaxis

Are there situations or clinical findings in the patient where you deviate from your prophylaxis standard (medication, dose, duration, age of the patient)? (multiple answers possible, please specify in answer box) [in patients with recurrent urinary tract infections (UTI)]
Yes

Are there situations or clinical findings in the patient where you deviate from your prophylaxis standard (medication, dose, duration, age of the patient)? (multiple answers possible, please specify in answer box) [in patients with recurrent urinary tract infections (UTI)][Comment]
No prophylaxis

Are there situations or clinical findings in the patient where you deviate from your prophylaxis standard (medication, dose, duration, age of the patient)? (multiple answers possible, please specify in answer box) [if multidrug-resistant organisms (MDRO) are detected in urine cultures]
Yes

Are there situations or clinical findings in the patient where you deviate from your prophylaxis standard (medication, dose, duration, age of the patient)? (multiple answers possible, please specify in answer box) [if multidrug-resistant organisms (MDRO) are detected in urine cultures][Comment]
Wait for resolution of actual infection if not controled

Are there situations or clinical findings in the patient where you deviate from your prophylaxis standard (medication, dose, duration, age of the patient)? (multiple answers possible, please specify in answer box) [in newborns]
Yes

Are there situations or clinical findings in the patient where you deviate from your prophylaxis standard (medication, dose, duration, age of the patient)? (multiple answers possible, please specify in answer box) [in newborns][Comment]
No prophylaxis

Are there situations or clinical findings in the patient where you deviate from your prophylaxis standard (medication, dose, duration, age of the patient)? (multiple answers possible, please specify in answer box) [Other]

Are there situations or clinical findings in the patient where you deviate from your prophylaxis standard (medication, dose, duration, age of the patient)? (multiple answers possible, please specify in answer box) [Other comment]

Are there situations in which you use intravenous prophylaxis?
No

Voiding analysis

Are there differences in antibiotic prophylaxis between VUS and VCUG in your department?
No

Is a non-pathological urine strip test required in preparation for VUS/VCUG in your department?
Yes

What is the maximum amount of days accepted for urine strip test to be performed prior to the exam?
0 - done the day of the examination

Is a non-pathological urine culture required in preparation for VUS/VCUG in your department?
No

Do patients only receive post-procedure prophylaxis if vesicoureteral reflux (VUR) is detected in VUS/VCUG?
Yes

Please explain your approach:
VUR increases the risk of infection °=°)

Do other MUS/MCU findings (e.g. urethral valves) have an influence on post-procedure prophylaxis?
No

End of survey

Thank you for taking the time to answer this survey!

May we contact you personally if we have further questions?
No

Survey response 153

Response ID
155

Date submitted
2025-03-25 08:49:22

Last page
5

Start language
en

Seed
1816861052

Date started
2025-03-25 08:46:05

Date last action
2025-03-25 08:49:22

Standard Operation Procedure

Who fills out this survey?
Pediatric Radiologist

Who fills out this survey? [Other]

In which type of hospital are you employed?
University Hospital

In which type of hospital are you employed? [Other]

In which country do you work?
Latvia

Does your department have a defined protocol for infection prophylaxis in the context of VUS/VCUG?
No

Has this protocol been transformed into a written standard operating procedure (SOP)?
N/A

Antibiotic Prophylaxis

Are there situations/constellations in which you do not use antibiotic prophylaxis for VUS/VCUG?
Yes

Please specify these situations/constellations: [girl with first uncomplicated UTI without urinary tract dilation in ultrasound]
No

Please specify these situations/constellations: [boy with first uncomplicated UTI without urinary tract dilation in ultrasound]
No

Please specify these situations/constellations: [in patients with low grade urinary tract dilation (up to 2nd grade) and no UTI in medical history]
No

Please specify these situations/constellations: [Other]
We never use

If you use antibiotic prophylaxis: which mediaction do you use? (multiple answers and subdivision into age groups possible) [Trimethoprim]
No

If you use antibiotic prophylaxis: which mediaction do you use? (multiple answers and subdivision into age groups possible) [Nitrofurantoin]
No

If you use antibiotic prophylaxis: which mediaction do you use? (multiple answers and subdivision into age groups possible) [Amoxicillin]
No

If you use antibiotic prophylaxis: which mediaction do you use? (multiple answers and subdivision into age groups possible) [Cefaclor]
No

If you use antibiotic prophylaxis: which mediaction do you use? (multiple answers and subdivision into age groups possible) [Amoxicillin/clavulanic acid]
No

If you use antibiotic prophylaxis: which mediaction do you use? (multiple answers and subdivision into age groups possible) [Other]
No

Trimethoprim: which time schedule do you use? [Day(s) before VUS/VCUG, how many?]
N/A

Trimethoprim: which time schedule do you use? [Day of VUS/VCUG]
N/A

Trimethoprim: which time schedule do you use? [Day(s) after VUS/VCUG, how many?]
N/A

Nitrofurantoin: which time schedule do you use? [Day(s) before VUS/VCUG, how many?]
N/A

Nitrofurantoin: which time schedule do you use? [Day of VUS/VCUG]
N/A

Nitrofurantoin: which time schedule do you use? [Day(s) after VUS/VCUG, how many?]
N/A

Amoxicillin: which time schedule do you use? [Day(s) before VUS/VCUG, how many?]
N/A

Amoxicillin: which time schedule do you use? [Day of VUS/VCUG]
N/A

Amoxicillin: which time schedule do you use? [Day(s) after VUS/VCUG, how many?]
N/A

Cefaclor: which time schedule do you use? [Days(s) before VUS/VCUG, how many?]
N/A

Cefaclor: which time schedule do you use? [Day of VUS/VCUG]
N/A

Cefaclor: which time schedule do you use? [Day(s) after VUS/VCUG, how many?]
N/A

Amoxicillin/clavulanic acid: in what dosage? (Amoxicillin portion) [prophylaktic (10-20 mg/Kg bodyweight/d)]
N/A

Amoxicillin/clavulanic acid: in what dosage? (Amoxicillin portion) [double prophylactic (20-40 mg/Kg bodyweight/d)]
N/A

Amoxicillin/clavulanic acid: in what dosage? (Amoxicillin portion) [therapeutic (80-100 mg/Kg bodyweight/d)]
N/A

Amoxicillin/clavulanic acid: which time schedule do you use? [Day(s) before VUS/VCUG]
N/A

Amoxicillin/clavulanic acid: which time schedule do you use? [Day of VUS/VCUG]
N/A

Amoxicillin/clavulanic acid: which time schedule do you use? [Day(s) after VUS/VCUG]
N/A

Other: which time schedule do you use? [Day(s) before VUS/VCUG, how many?]
N/A

Other: which time schedule do you use? [Day of VUS/VCUG]
N/A

Other: which time schedule do you use? [Day(s) after VUS/VCUG, how many?]
N/A

Indication for prophylaxis

Which patients receive periinterventional antibiotic prophylaxis? (multiple answers possible) [all]
No

Which patients receive periinterventional antibiotic prophylaxis? (multiple answers possible) [patients post urinary tract infection/pyelonephritis/urosepsis]
No

Which patients receive periinterventional antibiotic prophylaxis? (multiple answers possible) [patients with urinary tract dilation in ultrasound]
No

Which patients receive periinterventional antibiotic prophylaxis? (multiple answers possible) [patients with prenatal urinary tract dilation]
No

Which patients receive periinterventional antibiotic prophylaxis? (multiple answers possible) [patients post VUR surgery]
No

Which patients receive periinterventional antibiotic prophylaxis? (multiple answers possible) [none]
Yes

Which patients receive periinterventional antibiotic prophylaxis? (multiple answers possible) [Other]

Are there situations or clinical findings in the patient where you deviate from your prophylaxis standard (medication, dose, duration, age of the patient)? (multiple answers possible, please specify in answer box) [in case of already known vesicoureteral reflux (VUR)]
Yes

Are there situations or clinical findings in the patient where you deviate from your prophylaxis standard (medication, dose, duration, age of the patient)? (multiple answers possible, please specify in answer box) [in case of already known vesicoureteral reflux (VUR)][Comment]
never

Are there situations or clinical findings in the patient where you deviate from your prophylaxis standard (medication, dose, duration, age of the patient)? (multiple answers possible, please specify in answer box) [in patients with recurrent urinary tract infections (UTI)]
Yes

Are there situations or clinical findings in the patient where you deviate from your prophylaxis standard (medication, dose, duration, age of the patient)? (multiple answers possible, please specify in answer box) [in patients with recurrent urinary tract infections (UTI)][Comment]
never

Are there situations or clinical findings in the patient where you deviate from your prophylaxis standard (medication, dose, duration, age of the patient)? (multiple answers possible, please specify in answer box) [if multidrug-resistant organisms (MDRO) are detected in urine cultures]
Yes

Are there situations or clinical findings in the patient where you deviate from your prophylaxis standard (medication, dose, duration, age of the patient)? (multiple answers possible, please specify in answer box) [if multidrug-resistant organisms (MDRO) are detected in urine cultures][Comment]
never

Are there situations or clinical findings in the patient where you deviate from your prophylaxis standard (medication, dose, duration, age of the patient)? (multiple answers possible, please specify in answer box) [in newborns]
Yes

Are there situations or clinical findings in the patient where you deviate from your prophylaxis standard (medication, dose, duration, age of the patient)? (multiple answers possible, please specify in answer box) [in newborns][Comment]
never

Are there situations or clinical findings in the patient where you deviate from your prophylaxis standard (medication, dose, duration, age of the patient)? (multiple answers possible, please specify in answer box) [Other]

Are there situations or clinical findings in the patient where you deviate from your prophylaxis standard (medication, dose, duration, age of the patient)? (multiple answers possible, please specify in answer box) [Other comment]

Are there situations in which you use intravenous prophylaxis?
No

Voiding analysis

Are there differences in antibiotic prophylaxis between VUS and VCUG in your department?
No

Is a non-pathological urine strip test required in preparation for VUS/VCUG in your department?
No

Is a non-pathological urine culture required in preparation for VUS/VCUG in your department?
No

Do patients only receive post-procedure prophylaxis if vesicoureteral reflux (VUR) is detected in VUS/VCUG?
No

Do other MUS/MCU findings (e.g. urethral valves) have an influence on post-procedure prophylaxis?
No

End of survey

Thank you for taking the time to answer this survey!

May we contact you personally if we have further questions?
No

Survey response 154

Response ID
156

Last page
0

Start language
de

Seed
1038000076

Date started
2025-03-25 08:47:42

Date last action
2025-03-25 08:47:42

Standard Operation Procedure

Does your department have a defined protocol for infection prophylaxis in the context of VUS/VCUG?
N/A

Has this protocol been transformed into a written standard operating procedure (SOP)?
N/A

Antibiotic Prophylaxis

Are there situations/constellations in which you do not use antibiotic prophylaxis for VUS/VCUG?
N/A

Please specify these situations/constellations: [girl with first uncomplicated UTI without urinary tract dilation in ultrasound]
N/A

Please specify these situations/constellations: [boy with first uncomplicated UTI without urinary tract dilation in ultrasound]
N/A

Please specify these situations/constellations: [in patients with low grade urinary tract dilation (up to 2nd grade) and no UTI in medical history]
N/A

If you use antibiotic prophylaxis: which mediaction do you use? (multiple answers and subdivision into age groups possible) [Trimethoprim]
N/A

If you use antibiotic prophylaxis: which mediaction do you use? (multiple answers and subdivision into age groups possible) [Nitrofurantoin]
N/A

If you use antibiotic prophylaxis: which mediaction do you use? (multiple answers and subdivision into age groups possible) [Amoxicillin]
N/A

If you use antibiotic prophylaxis: which mediaction do you use? (multiple answers and subdivision into age groups possible) [Cefaclor]
N/A

If you use antibiotic prophylaxis: which mediaction do you use? (multiple answers and subdivision into age groups possible) [Amoxicillin/clavulanic acid]
N/A

If you use antibiotic prophylaxis: which mediaction do you use? (multiple answers and subdivision into age groups possible) [Other]
N/A

Trimethoprim: which time schedule do you use? [Day(s) before VUS/VCUG, how many?]
N/A

Trimethoprim: which time schedule do you use? [Day of VUS/VCUG]
N/A

Trimethoprim: which time schedule do you use? [Day(s) after VUS/VCUG, how many?]
N/A

Nitrofurantoin: which time schedule do you use? [Day(s) before VUS/VCUG, how many?]
N/A

Nitrofurantoin: which time schedule do you use? [Day of VUS/VCUG]
N/A

Nitrofurantoin: which time schedule do you use? [Day(s) after VUS/VCUG, how many?]
N/A

Amoxicillin: which time schedule do you use? [Day(s) before VUS/VCUG, how many?]
N/A

Amoxicillin: which time schedule do you use? [Day of VUS/VCUG]
N/A

Amoxicillin: which time schedule do you use? [Day(s) after VUS/VCUG, how many?]
N/A

Cefaclor: which time schedule do you use? [Days(s) before VUS/VCUG, how many?]
N/A

Cefaclor: which time schedule do you use? [Day of VUS/VCUG]
N/A

Cefaclor: which time schedule do you use? [Day(s) after VUS/VCUG, how many?]
N/A

Amoxicillin/clavulanic acid: in what dosage? (Amoxicillin portion) [prophylaktic (10-20 mg/Kg bodyweight/d)]
N/A

Amoxicillin/clavulanic acid: in what dosage? (Amoxicillin portion) [double prophylactic (20-40 mg/Kg bodyweight/d)]
N/A

Amoxicillin/clavulanic acid: in what dosage? (Amoxicillin portion) [therapeutic (80-100 mg/Kg bodyweight/d)]
N/A

Amoxicillin/clavulanic acid: which time schedule do you use? [Day(s) before VUS/VCUG]
N/A

Amoxicillin/clavulanic acid: which time schedule do you use? [Day of VUS/VCUG]
N/A

Amoxicillin/clavulanic acid: which time schedule do you use? [Day(s) after VUS/VCUG]
N/A

Other: which time schedule do you use? [Day(s) before VUS/VCUG, how many?]
N/A

Other: which time schedule do you use? [Day of VUS/VCUG]
N/A

Other: which time schedule do you use? [Day(s) after VUS/VCUG, how many?]
N/A

Indication for prophylaxis

Which patients receive periinterventional antibiotic prophylaxis? (multiple answers possible) [all]
N/A

Which patients receive periinterventional antibiotic prophylaxis? (multiple answers possible) [patients post urinary tract infection/pyelonephritis/urosepsis]
N/A

Which patients receive periinterventional antibiotic prophylaxis? (multiple answers possible) [patients with urinary tract dilation in ultrasound]
N/A

Which patients receive periinterventional antibiotic prophylaxis? (multiple answers possible) [patients with prenatal urinary tract dilation]
N/A

Which patients receive periinterventional antibiotic prophylaxis? (multiple answers possible) [patients post VUR surgery]
N/A

Which patients receive periinterventional antibiotic prophylaxis? (multiple answers possible) [none]
N/A

Are there situations or clinical findings in the patient where you deviate from your prophylaxis standard (medication, dose, duration, age of the patient)? (multiple answers possible, please specify in answer box) [in case of already known vesicoureteral reflux (VUR)]
N/A

Are there situations or clinical findings in the patient where you deviate from your prophylaxis standard (medication, dose, duration, age of the patient)? (multiple answers possible, please specify in answer box) [in patients with recurrent urinary tract infections (UTI)]
N/A

Are there situations or clinical findings in the patient where you deviate from your prophylaxis standard (medication, dose, duration, age of the patient)? (multiple answers possible, please specify in answer box) [if multidrug-resistant organisms (MDRO) are detected in urine cultures]
N/A

Are there situations or clinical findings in the patient where you deviate from your prophylaxis standard (medication, dose, duration, age of the patient)? (multiple answers possible, please specify in answer box) [in newborns]
N/A

Are there situations in which you use intravenous prophylaxis?
N/A

Voiding analysis

Are there differences in antibiotic prophylaxis between VUS and VCUG in your department?
N/A

Is a non-pathological urine strip test required in preparation for VUS/VCUG in your department?
N/A

Is a non-pathological urine culture required in preparation for VUS/VCUG in your department?
N/A

Do patients only receive post-procedure prophylaxis if vesicoureteral reflux (VUR) is detected in VUS/VCUG?
N/A

Do other MUS/MCU findings (e.g. urethral valves) have an influence on post-procedure prophylaxis?
N/A

End of survey

May we contact you personally if we have further questions?
N/A

Survey response 155

Response ID
157

Date submitted
2025-03-25 09:11:31

Last page
5

Start language
en

Seed
804319933

Date started
2025-03-25 08:55:40

Date last action
2025-03-25 09:11:31

Standard Operation Procedure

Who fills out this survey?
Pediatric Radiologist

Who fills out this survey? [Other]

In which type of hospital are you employed?
University Hospital

In which type of hospital are you employed? [Other]

In which country do you work?
Norway

Does your department have a defined protocol for infection prophylaxis in the context of VUS/VCUG?
Yes

Has this protocol been transformed into a written standard operating procedure (SOP)?
Yes

Which department is the author of the SOP for infection prophylaxis in VUS/VCUG?
Pediatric radiology

Which department is the author of the SOP for infection prophylaxis in VUS/VCUG? [Other]

Antibiotic Prophylaxis

Are there situations/constellations in which you do not use antibiotic prophylaxis for VUS/VCUG?
No

Please specify these situations/constellations: [girl with first uncomplicated UTI without urinary tract dilation in ultrasound]
N/A

Please specify these situations/constellations: [boy with first uncomplicated UTI without urinary tract dilation in ultrasound]
N/A

Please specify these situations/constellations: [in patients with low grade urinary tract dilation (up to 2nd grade) and no UTI in medical history]
N/A

If you use antibiotic prophylaxis: which mediaction do you use? (multiple answers and subdivision into age groups possible) [Trimethoprim]
No

If you use antibiotic prophylaxis: which mediaction do you use? (multiple answers and subdivision into age groups possible) [Nitrofurantoin]
Yes

If you use antibiotic prophylaxis: which mediaction do you use? (multiple answers and subdivision into age groups possible) [Amoxicillin]
No

If you use antibiotic prophylaxis: which mediaction do you use? (multiple answers and subdivision into age groups possible) [Cefaclor]
No

If you use antibiotic prophylaxis: which mediaction do you use? (multiple answers and subdivision into age groups possible) [Amoxicillin/clavulanic acid]
No

If you use antibiotic prophylaxis: which mediaction do you use? (multiple answers and subdivision into age groups possible) [Other]
No

Trimethoprim: which time schedule do you use? [Day(s) before VUS/VCUG, how many?]
N/A

Trimethoprim: which time schedule do you use? [Day of VUS/VCUG]
N/A

Trimethoprim: which time schedule do you use? [Day(s) after VUS/VCUG, how many?]
N/A

Nitrofurantoin: in what dosage?
prophylactic (1-2 mg/kg bodyweight/d)

Nitrofurantoin: in what dosage? [Other]

Nitrofurantoin: for which age group do you use nitrofurantoin?
0-18

Nitrofurantoin: which time schedule do you use? [Day(s) before VUS/VCUG, how many?]
No

Nitrofurantoin: which time schedule do you use? [Day(s) before VUS/VCUG, how many?][Comment]

Nitrofurantoin: which time schedule do you use? [Day of VUS/VCUG]
Yes

Nitrofurantoin: which time schedule do you use? [Day of VUS/VCUG][Comment]

Nitrofurantoin: which time schedule do you use? [Day(s) after VUS/VCUG, how many?]
Yes

Nitrofurantoin: which time schedule do you use? [Day(s) after VUS/VCUG, how many?][Comment]
2

Amoxicillin: which time schedule do you use? [Day(s) before VUS/VCUG, how many?]
N/A

Amoxicillin: which time schedule do you use? [Day of VUS/VCUG]
N/A

Amoxicillin: which time schedule do you use? [Day(s) after VUS/VCUG, how many?]
N/A

Cefaclor: which time schedule do you use? [Days(s) before VUS/VCUG, how many?]
N/A

Cefaclor: which time schedule do you use? [Day of VUS/VCUG]
N/A

Cefaclor: which time schedule do you use? [Day(s) after VUS/VCUG, how many?]
N/A

Amoxicillin/clavulanic acid: in what dosage? (Amoxicillin portion) [prophylaktic (10-20 mg/Kg bodyweight/d)]
N/A

Amoxicillin/clavulanic acid: in what dosage? (Amoxicillin portion) [double prophylactic (20-40 mg/Kg bodyweight/d)]
N/A

Amoxicillin/clavulanic acid: in what dosage? (Amoxicillin portion) [therapeutic (80-100 mg/Kg bodyweight/d)]
N/A

Amoxicillin/clavulanic acid: which time schedule do you use? [Day(s) before VUS/VCUG]
N/A

Amoxicillin/clavulanic acid: which time schedule do you use? [Day of VUS/VCUG]
N/A

Amoxicillin/clavulanic acid: which time schedule do you use? [Day(s) after VUS/VCUG]
N/A

Other: which time schedule do you use? [Day(s) before VUS/VCUG, how many?]
N/A

Other: which time schedule do you use? [Day of VUS/VCUG]
N/A

Other: which time schedule do you use? [Day(s) after VUS/VCUG, how many?]
N/A

Indication for prophylaxis

Which patients receive periinterventional antibiotic prophylaxis? (multiple answers possible) [all]
Yes

Which patients receive periinterventional antibiotic prophylaxis? (multiple answers possible) [patients post urinary tract infection/pyelonephritis/urosepsis]
N/A

Which patients receive periinterventional antibiotic prophylaxis? (multiple answers possible) [patients with urinary tract dilation in ultrasound]
N/A

Which patients receive periinterventional antibiotic prophylaxis? (multiple answers possible) [patients with prenatal urinary tract dilation]
N/A

Which patients receive periinterventional antibiotic prophylaxis? (multiple answers possible) [patients post VUR surgery]
N/A

Which patients receive periinterventional antibiotic prophylaxis? (multiple answers possible) [none]
N/A

Are there situations or clinical findings in the patient where you deviate from your prophylaxis standard (medication, dose, duration, age of the patient)? (multiple answers possible, please specify in answer box) [in case of already known vesicoureteral reflux (VUR)]
Yes

Are there situations or clinical findings in the patient where you deviate from your prophylaxis standard (medication, dose, duration, age of the patient)? (multiple answers possible, please specify in answer box) [in case of already known vesicoureteral reflux (VUR)][Comment]
They allready stand on medication for urinary tract infections

Are there situations or clinical findings in the patient where you deviate from your prophylaxis standard (medication, dose, duration, age of the patient)? (multiple answers possible, please specify in answer box) [in patients with recurrent urinary tract infections (UTI)]
Yes

Are there situations or clinical findings in the patient where you deviate from your prophylaxis standard (medication, dose, duration, age of the patient)? (multiple answers possible, please specify in answer box) [in patients with recurrent urinary tract infections (UTI)][Comment]
They allready stand on medication for urinary tract infections

Are there situations or clinical findings in the patient where you deviate from your prophylaxis standard (medication, dose, duration, age of the patient)? (multiple answers possible, please specify in answer box) [if multidrug-resistant organisms (MDRO) are detected in urine cultures]
No

Are there situations or clinical findings in the patient where you deviate from your prophylaxis standard (medication, dose, duration, age of the patient)? (multiple answers possible, please specify in answer box) [if multidrug-resistant organisms (MDRO) are detected in urine cultures][Comment]

Are there situations or clinical findings in the patient where you deviate from your prophylaxis standard (medication, dose, duration, age of the patient)? (multiple answers possible, please specify in answer box) [in newborns]
No

Are there situations or clinical findings in the patient where you deviate from your prophylaxis standard (medication, dose, duration, age of the patient)? (multiple answers possible, please specify in answer box) [in newborns][Comment]

Are there situations or clinical findings in the patient where you deviate from your prophylaxis standard (medication, dose, duration, age of the patient)? (multiple answers possible, please specify in answer box) [Other]

Are there situations or clinical findings in the patient where you deviate from your prophylaxis standard (medication, dose, duration, age of the patient)? (multiple answers possible, please specify in answer box) [Other comment]

Are there situations in which you use intravenous prophylaxis?
No

Voiding analysis

Are there differences in antibiotic prophylaxis between VUS and VCUG in your department?
No

Is a non-pathological urine strip test required in preparation for VUS/VCUG in your department?
No

Is a non-pathological urine culture required in preparation for VUS/VCUG in your department?
Yes

What is the maximum amount of days accepted for urine culture to be performed prior to the exam?
7

Do patients only receive post-procedure prophylaxis if vesicoureteral reflux (VUR) is detected in VUS/VCUG?
No

Do other MUS/MCU findings (e.g. urethral valves) have an influence on post-procedure prophylaxis?
No

End of survey

Thank you for taking the time to answer this survey!

May we contact you personally if we have further questions?
No

Survey response 156

Response ID
158

Last page
0

Start language
en

Seed
719351338

Date started
2025-03-25 08:58:36

Date last action
2025-03-25 08:58:36

Standard Operation Procedure

Does your department have a defined protocol for infection prophylaxis in the context of VUS/VCUG?
N/A

Has this protocol been transformed into a written standard operating procedure (SOP)?
N/A

Antibiotic Prophylaxis

Are there situations/constellations in which you do not use antibiotic prophylaxis for VUS/VCUG?
N/A

Please specify these situations/constellations: [girl with first uncomplicated UTI without urinary tract dilation in ultrasound]
N/A

Please specify these situations/constellations: [boy with first uncomplicated UTI without urinary tract dilation in ultrasound]
N/A

Please specify these situations/constellations: [in patients with low grade urinary tract dilation (up to 2nd grade) and no UTI in medical history]
N/A

If you use antibiotic prophylaxis: which mediaction do you use? (multiple answers and subdivision into age groups possible) [Trimethoprim]
N/A

If you use antibiotic prophylaxis: which mediaction do you use? (multiple answers and subdivision into age groups possible) [Nitrofurantoin]
N/A

If you use antibiotic prophylaxis: which mediaction do you use? (multiple answers and subdivision into age groups possible) [Amoxicillin]
N/A

If you use antibiotic prophylaxis: which mediaction do you use? (multiple answers and subdivision into age groups possible) [Cefaclor]
N/A

If you use antibiotic prophylaxis: which mediaction do you use? (multiple answers and subdivision into age groups possible) [Amoxicillin/clavulanic acid]
N/A

If you use antibiotic prophylaxis: which mediaction do you use? (multiple answers and subdivision into age groups possible) [Other]
N/A

Trimethoprim: which time schedule do you use? [Day(s) before VUS/VCUG, how many?]
N/A

Trimethoprim: which time schedule do you use? [Day of VUS/VCUG]
N/A

Trimethoprim: which time schedule do you use? [Day(s) after VUS/VCUG, how many?]
N/A

Nitrofurantoin: which time schedule do you use? [Day(s) before VUS/VCUG, how many?]
N/A

Nitrofurantoin: which time schedule do you use? [Day of VUS/VCUG]
N/A

Nitrofurantoin: which time schedule do you use? [Day(s) after VUS/VCUG, how many?]
N/A

Amoxicillin: which time schedule do you use? [Day(s) before VUS/VCUG, how many?]
N/A

Amoxicillin: which time schedule do you use? [Day of VUS/VCUG]
N/A

Amoxicillin: which time schedule do you use? [Day(s) after VUS/VCUG, how many?]
N/A

Cefaclor: which time schedule do you use? [Days(s) before VUS/VCUG, how many?]
N/A

Cefaclor: which time schedule do you use? [Day of VUS/VCUG]
N/A

Cefaclor: which time schedule do you use? [Day(s) after VUS/VCUG, how many?]
N/A

Amoxicillin/clavulanic acid: in what dosage? (Amoxicillin portion) [prophylaktic (10-20 mg/Kg bodyweight/d)]
N/A

Amoxicillin/clavulanic acid: in what dosage? (Amoxicillin portion) [double prophylactic (20-40 mg/Kg bodyweight/d)]
N/A

Amoxicillin/clavulanic acid: in what dosage? (Amoxicillin portion) [therapeutic (80-100 mg/Kg bodyweight/d)]
N/A

Amoxicillin/clavulanic acid: which time schedule do you use? [Day(s) before VUS/VCUG]
N/A

Amoxicillin/clavulanic acid: which time schedule do you use? [Day of VUS/VCUG]
N/A

Amoxicillin/clavulanic acid: which time schedule do you use? [Day(s) after VUS/VCUG]
N/A

Other: which time schedule do you use? [Day(s) before VUS/VCUG, how many?]
N/A

Other: which time schedule do you use? [Day of VUS/VCUG]
N/A

Other: which time schedule do you use? [Day(s) after VUS/VCUG, how many?]
N/A

Indication for prophylaxis

Which patients receive periinterventional antibiotic prophylaxis? (multiple answers possible) [all]
N/A

Which patients receive periinterventional antibiotic prophylaxis? (multiple answers possible) [patients post urinary tract infection/pyelonephritis/urosepsis]
N/A

Which patients receive periinterventional antibiotic prophylaxis? (multiple answers possible) [patients with urinary tract dilation in ultrasound]
N/A

Which patients receive periinterventional antibiotic prophylaxis? (multiple answers possible) [patients with prenatal urinary tract dilation]
N/A

Which patients receive periinterventional antibiotic prophylaxis? (multiple answers possible) [patients post VUR surgery]
N/A

Which patients receive periinterventional antibiotic prophylaxis? (multiple answers possible) [none]
N/A

Are there situations or clinical findings in the patient where you deviate from your prophylaxis standard (medication, dose, duration, age of the patient)? (multiple answers possible, please specify in answer box) [in case of already known vesicoureteral reflux (VUR)]
N/A

Are there situations or clinical findings in the patient where you deviate from your prophylaxis standard (medication, dose, duration, age of the patient)? (multiple answers possible, please specify in answer box) [in patients with recurrent urinary tract infections (UTI)]
N/A

Are there situations or clinical findings in the patient where you deviate from your prophylaxis standard (medication, dose, duration, age of the patient)? (multiple answers possible, please specify in answer box) [if multidrug-resistant organisms (MDRO) are detected in urine cultures]
N/A

Are there situations or clinical findings in the patient where you deviate from your prophylaxis standard (medication, dose, duration, age of the patient)? (multiple answers possible, please specify in answer box) [in newborns]
N/A

Are there situations in which you use intravenous prophylaxis?
N/A

Voiding analysis

Are there differences in antibiotic prophylaxis between VUS and VCUG in your department?
N/A

Is a non-pathological urine strip test required in preparation for VUS/VCUG in your department?
N/A

Is a non-pathological urine culture required in preparation for VUS/VCUG in your department?
N/A

Do patients only receive post-procedure prophylaxis if vesicoureteral reflux (VUR) is detected in VUS/VCUG?
N/A

Do other MUS/MCU findings (e.g. urethral valves) have an influence on post-procedure prophylaxis?
N/A

End of survey

May we contact you personally if we have further questions?
N/A

Survey response 157

Response ID
159

Last page
1

Start language
en

Seed
770010382

Date started
2025-03-25 09:05:03

Date last action
2025-03-25 09:05:37

Standard Operation Procedure

Who fills out this survey?
Pediatric Radiologist

Who fills out this survey? [Other]

In which type of hospital are you employed?
University Hospital

In which type of hospital are you employed? [Other]

In which country do you work?
Australia

Does your department have a defined protocol for infection prophylaxis in the context of VUS/VCUG?
Yes

Has this protocol been transformed into a written standard operating procedure (SOP)?
No

Antibiotic Prophylaxis

Are there situations/constellations in which you do not use antibiotic prophylaxis for VUS/VCUG?
N/A

Please specify these situations/constellations: [girl with first uncomplicated UTI without urinary tract dilation in ultrasound]
N/A

Please specify these situations/constellations: [boy with first uncomplicated UTI without urinary tract dilation in ultrasound]
N/A

Please specify these situations/constellations: [in patients with low grade urinary tract dilation (up to 2nd grade) and no UTI in medical history]
N/A

If you use antibiotic prophylaxis: which mediaction do you use? (multiple answers and subdivision into age groups possible) [Trimethoprim]
N/A

If you use antibiotic prophylaxis: which mediaction do you use? (multiple answers and subdivision into age groups possible) [Nitrofurantoin]
N/A

If you use antibiotic prophylaxis: which mediaction do you use? (multiple answers and subdivision into age groups possible) [Amoxicillin]
N/A

If you use antibiotic prophylaxis: which mediaction do you use? (multiple answers and subdivision into age groups possible) [Cefaclor]
N/A

If you use antibiotic prophylaxis: which mediaction do you use? (multiple answers and subdivision into age groups possible) [Amoxicillin/clavulanic acid]
N/A

If you use antibiotic prophylaxis: which mediaction do you use? (multiple answers and subdivision into age groups possible) [Other]
N/A

Trimethoprim: which time schedule do you use? [Day(s) before VUS/VCUG, how many?]
N/A

Trimethoprim: which time schedule do you use? [Day of VUS/VCUG]
N/A

Trimethoprim: which time schedule do you use? [Day(s) after VUS/VCUG, how many?]
N/A

Nitrofurantoin: which time schedule do you use? [Day(s) before VUS/VCUG, how many?]
N/A

Nitrofurantoin: which time schedule do you use? [Day of VUS/VCUG]
N/A

Nitrofurantoin: which time schedule do you use? [Day(s) after VUS/VCUG, how many?]
N/A

Amoxicillin: which time schedule do you use? [Day(s) before VUS/VCUG, how many?]
N/A

Amoxicillin: which time schedule do you use? [Day of VUS/VCUG]
N/A

Amoxicillin: which time schedule do you use? [Day(s) after VUS/VCUG, how many?]
N/A

Cefaclor: which time schedule do you use? [Days(s) before VUS/VCUG, how many?]
N/A

Cefaclor: which time schedule do you use? [Day of VUS/VCUG]
N/A

Cefaclor: which time schedule do you use? [Day(s) after VUS/VCUG, how many?]
N/A

Amoxicillin/clavulanic acid: in what dosage? (Amoxicillin portion) [prophylaktic (10-20 mg/Kg bodyweight/d)]
N/A

Amoxicillin/clavulanic acid: in what dosage? (Amoxicillin portion) [double prophylactic (20-40 mg/Kg bodyweight/d)]
N/A

Amoxicillin/clavulanic acid: in what dosage? (Amoxicillin portion) [therapeutic (80-100 mg/Kg bodyweight/d)]
N/A

Amoxicillin/clavulanic acid: which time schedule do you use? [Day(s) before VUS/VCUG]
N/A

Amoxicillin/clavulanic acid: which time schedule do you use? [Day of VUS/VCUG]
N/A

Amoxicillin/clavulanic acid: which time schedule do you use? [Day(s) after VUS/VCUG]
N/A

Other: which time schedule do you use? [Day(s) before VUS/VCUG, how many?]
N/A

Other: which time schedule do you use? [Day of VUS/VCUG]
N/A

Other: which time schedule do you use? [Day(s) after VUS/VCUG, how many?]
N/A

Indication for prophylaxis

Which patients receive periinterventional antibiotic prophylaxis? (multiple answers possible) [all]
N/A

Which patients receive periinterventional antibiotic prophylaxis? (multiple answers possible) [patients post urinary tract infection/pyelonephritis/urosepsis]
N/A

Which patients receive periinterventional antibiotic prophylaxis? (multiple answers possible) [patients with urinary tract dilation in ultrasound]
N/A

Which patients receive periinterventional antibiotic prophylaxis? (multiple answers possible) [patients with prenatal urinary tract dilation]
N/A

Which patients receive periinterventional antibiotic prophylaxis? (multiple answers possible) [patients post VUR surgery]
N/A

Which patients receive periinterventional antibiotic prophylaxis? (multiple answers possible) [none]
N/A

Are there situations or clinical findings in the patient where you deviate from your prophylaxis standard (medication, dose, duration, age of the patient)? (multiple answers possible, please specify in answer box) [in case of already known vesicoureteral reflux (VUR)]
N/A

Are there situations or clinical findings in the patient where you deviate from your prophylaxis standard (medication, dose, duration, age of the patient)? (multiple answers possible, please specify in answer box) [in patients with recurrent urinary tract infections (UTI)]
N/A

Are there situations or clinical findings in the patient where you deviate from your prophylaxis standard (medication, dose, duration, age of the patient)? (multiple answers possible, please specify in answer box) [if multidrug-resistant organisms (MDRO) are detected in urine cultures]
N/A

Are there situations or clinical findings in the patient where you deviate from your prophylaxis standard (medication, dose, duration, age of the patient)? (multiple answers possible, please specify in answer box) [in newborns]
N/A

Are there situations in which you use intravenous prophylaxis?
N/A

Voiding analysis

Are there differences in antibiotic prophylaxis between VUS and VCUG in your department?
N/A

Is a non-pathological urine strip test required in preparation for VUS/VCUG in your department?
N/A

Is a non-pathological urine culture required in preparation for VUS/VCUG in your department?
N/A

Do patients only receive post-procedure prophylaxis if vesicoureteral reflux (VUR) is detected in VUS/VCUG?
N/A

Do other MUS/MCU findings (e.g. urethral valves) have an influence on post-procedure prophylaxis?
N/A

End of survey

May we contact you personally if we have further questions?
N/A

Survey response 158

Response ID
160

Last page
1

Start language
en

Seed
120189174

Date started
2025-03-25 09:15:08

Date last action
2025-03-25 09:15:56

Standard Operation Procedure

Who fills out this survey?
Pediatric Radiologist

Who fills out this survey? [Other]

In which type of hospital are you employed?
University Hospital

In which type of hospital are you employed? [Other]

In which country do you work?
Malaysia

Does your department have a defined protocol for infection prophylaxis in the context of VUS/VCUG?
Yes

Has this protocol been transformed into a written standard operating procedure (SOP)?
Yes

Which department is the author of the SOP for infection prophylaxis in VUS/VCUG?
Pediatric surgery

Which department is the author of the SOP for infection prophylaxis in VUS/VCUG? [Other]

Antibiotic Prophylaxis

Are there situations/constellations in which you do not use antibiotic prophylaxis for VUS/VCUG?
N/A

Please specify these situations/constellations: [girl with first uncomplicated UTI without urinary tract dilation in ultrasound]
N/A

Please specify these situations/constellations: [boy with first uncomplicated UTI without urinary tract dilation in ultrasound]
N/A

Please specify these situations/constellations: [in patients with low grade urinary tract dilation (up to 2nd grade) and no UTI in medical history]
N/A

If you use antibiotic prophylaxis: which mediaction do you use? (multiple answers and subdivision into age groups possible) [Trimethoprim]
N/A

If you use antibiotic prophylaxis: which mediaction do you use? (multiple answers and subdivision into age groups possible) [Nitrofurantoin]
N/A

If you use antibiotic prophylaxis: which mediaction do you use? (multiple answers and subdivision into age groups possible) [Amoxicillin]
N/A

If you use antibiotic prophylaxis: which mediaction do you use? (multiple answers and subdivision into age groups possible) [Cefaclor]
N/A

If you use antibiotic prophylaxis: which mediaction do you use? (multiple answers and subdivision into age groups possible) [Amoxicillin/clavulanic acid]
N/A

If you use antibiotic prophylaxis: which mediaction do you use? (multiple answers and subdivision into age groups possible) [Other]
N/A

Trimethoprim: which time schedule do you use? [Day(s) before VUS/VCUG, how many?]
N/A

Trimethoprim: which time schedule do you use? [Day of VUS/VCUG]
N/A

Trimethoprim: which time schedule do you use? [Day(s) after VUS/VCUG, how many?]
N/A

Nitrofurantoin: which time schedule do you use? [Day(s) before VUS/VCUG, how many?]
N/A

Nitrofurantoin: which time schedule do you use? [Day of VUS/VCUG]
N/A

Nitrofurantoin: which time schedule do you use? [Day(s) after VUS/VCUG, how many?]
N/A

Amoxicillin: which time schedule do you use? [Day(s) before VUS/VCUG, how many?]
N/A

Amoxicillin: which time schedule do you use? [Day of VUS/VCUG]
N/A

Amoxicillin: which time schedule do you use? [Day(s) after VUS/VCUG, how many?]
N/A

Cefaclor: which time schedule do you use? [Days(s) before VUS/VCUG, how many?]
N/A

Cefaclor: which time schedule do you use? [Day of VUS/VCUG]
N/A

Cefaclor: which time schedule do you use? [Day(s) after VUS/VCUG, how many?]
N/A

Amoxicillin/clavulanic acid: in what dosage? (Amoxicillin portion) [prophylaktic (10-20 mg/Kg bodyweight/d)]
N/A

Amoxicillin/clavulanic acid: in what dosage? (Amoxicillin portion) [double prophylactic (20-40 mg/Kg bodyweight/d)]
N/A

Amoxicillin/clavulanic acid: in what dosage? (Amoxicillin portion) [therapeutic (80-100 mg/Kg bodyweight/d)]
N/A

Amoxicillin/clavulanic acid: which time schedule do you use? [Day(s) before VUS/VCUG]
N/A

Amoxicillin/clavulanic acid: which time schedule do you use? [Day of VUS/VCUG]
N/A

Amoxicillin/clavulanic acid: which time schedule do you use? [Day(s) after VUS/VCUG]
N/A

Other: which time schedule do you use? [Day(s) before VUS/VCUG, how many?]
N/A

Other: which time schedule do you use? [Day of VUS/VCUG]
N/A

Other: which time schedule do you use? [Day(s) after VUS/VCUG, how many?]
N/A

Indication for prophylaxis

Which patients receive periinterventional antibiotic prophylaxis? (multiple answers possible) [all]
N/A

Which patients receive periinterventional antibiotic prophylaxis? (multiple answers possible) [patients post urinary tract infection/pyelonephritis/urosepsis]
N/A

Which patients receive periinterventional antibiotic prophylaxis? (multiple answers possible) [patients with urinary tract dilation in ultrasound]
N/A

Which patients receive periinterventional antibiotic prophylaxis? (multiple answers possible) [patients with prenatal urinary tract dilation]
N/A

Which patients receive periinterventional antibiotic prophylaxis? (multiple answers possible) [patients post VUR surgery]
N/A

Which patients receive periinterventional antibiotic prophylaxis? (multiple answers possible) [none]
N/A

Are there situations or clinical findings in the patient where you deviate from your prophylaxis standard (medication, dose, duration, age of the patient)? (multiple answers possible, please specify in answer box) [in case of already known vesicoureteral reflux (VUR)]
N/A

Are there situations or clinical findings in the patient where you deviate from your prophylaxis standard (medication, dose, duration, age of the patient)? (multiple answers possible, please specify in answer box) [in patients with recurrent urinary tract infections (UTI)]
N/A

Are there situations or clinical findings in the patient where you deviate from your prophylaxis standard (medication, dose, duration, age of the patient)? (multiple answers possible, please specify in answer box) [if multidrug-resistant organisms (MDRO) are detected in urine cultures]
N/A

Are there situations or clinical findings in the patient where you deviate from your prophylaxis standard (medication, dose, duration, age of the patient)? (multiple answers possible, please specify in answer box) [in newborns]
N/A

Are there situations in which you use intravenous prophylaxis?
N/A

Voiding analysis

Are there differences in antibiotic prophylaxis between VUS and VCUG in your department?
N/A

Is a non-pathological urine strip test required in preparation for VUS/VCUG in your department?
N/A

Is a non-pathological urine culture required in preparation for VUS/VCUG in your department?
N/A

Do patients only receive post-procedure prophylaxis if vesicoureteral reflux (VUR) is detected in VUS/VCUG?
N/A

Do other MUS/MCU findings (e.g. urethral valves) have an influence on post-procedure prophylaxis?
N/A

End of survey

May we contact you personally if we have further questions?
N/A

Survey response 159

Response ID
161

Last page
1

Start language
de

Seed
84191415

Date started
2025-03-25 09:19:00

Date last action
2025-03-25 09:19:36

Standard Operation Procedure

Who fills out this survey?
Radiologist

Who fills out this survey? [Other]

In which type of hospital are you employed?
University Hospital

In which type of hospital are you employed? [Other]

In which country do you work?
Deutschland

Does your department have a defined protocol for infection prophylaxis in the context of VUS/VCUG?
No

Has this protocol been transformed into a written standard operating procedure (SOP)?
N/A

Antibiotic Prophylaxis

Are there situations/constellations in which you do not use antibiotic prophylaxis for VUS/VCUG?
N/A

Please specify these situations/constellations: [girl with first uncomplicated UTI without urinary tract dilation in ultrasound]
N/A

Please specify these situations/constellations: [boy with first uncomplicated UTI without urinary tract dilation in ultrasound]
N/A

Please specify these situations/constellations: [in patients with low grade urinary tract dilation (up to 2nd grade) and no UTI in medical history]
N/A

If you use antibiotic prophylaxis: which mediaction do you use? (multiple answers and subdivision into age groups possible) [Trimethoprim]
N/A

If you use antibiotic prophylaxis: which mediaction do you use? (multiple answers and subdivision into age groups possible) [Nitrofurantoin]
N/A

If you use antibiotic prophylaxis: which mediaction do you use? (multiple answers and subdivision into age groups possible) [Amoxicillin]
N/A

If you use antibiotic prophylaxis: which mediaction do you use? (multiple answers and subdivision into age groups possible) [Cefaclor]
N/A

If you use antibiotic prophylaxis: which mediaction do you use? (multiple answers and subdivision into age groups possible) [Amoxicillin/clavulanic acid]
N/A

If you use antibiotic prophylaxis: which mediaction do you use? (multiple answers and subdivision into age groups possible) [Other]
N/A

Trimethoprim: which time schedule do you use? [Day(s) before VUS/VCUG, how many?]
N/A

Trimethoprim: which time schedule do you use? [Day of VUS/VCUG]
N/A

Trimethoprim: which time schedule do you use? [Day(s) after VUS/VCUG, how many?]
N/A

Nitrofurantoin: which time schedule do you use? [Day(s) before VUS/VCUG, how many?]
N/A

Nitrofurantoin: which time schedule do you use? [Day of VUS/VCUG]
N/A

Nitrofurantoin: which time schedule do you use? [Day(s) after VUS/VCUG, how many?]
N/A

Amoxicillin: which time schedule do you use? [Day(s) before VUS/VCUG, how many?]
N/A

Amoxicillin: which time schedule do you use? [Day of VUS/VCUG]
N/A

Amoxicillin: which time schedule do you use? [Day(s) after VUS/VCUG, how many?]
N/A

Cefaclor: which time schedule do you use? [Days(s) before VUS/VCUG, how many?]
N/A

Cefaclor: which time schedule do you use? [Day of VUS/VCUG]
N/A

Cefaclor: which time schedule do you use? [Day(s) after VUS/VCUG, how many?]
N/A

Amoxicillin/clavulanic acid: in what dosage? (Amoxicillin portion) [prophylaktic (10-20 mg/Kg bodyweight/d)]
N/A

Amoxicillin/clavulanic acid: in what dosage? (Amoxicillin portion) [double prophylactic (20-40 mg/Kg bodyweight/d)]
N/A

Amoxicillin/clavulanic acid: in what dosage? (Amoxicillin portion) [therapeutic (80-100 mg/Kg bodyweight/d)]
N/A

Amoxicillin/clavulanic acid: which time schedule do you use? [Day(s) before VUS/VCUG]
N/A

Amoxicillin/clavulanic acid: which time schedule do you use? [Day of VUS/VCUG]
N/A

Amoxicillin/clavulanic acid: which time schedule do you use? [Day(s) after VUS/VCUG]
N/A

Other: which time schedule do you use? [Day(s) before VUS/VCUG, how many?]
N/A

Other: which time schedule do you use? [Day of VUS/VCUG]
N/A

Other: which time schedule do you use? [Day(s) after VUS/VCUG, how many?]
N/A

Indication for prophylaxis

Which patients receive periinterventional antibiotic prophylaxis? (multiple answers possible) [all]
N/A

Which patients receive periinterventional antibiotic prophylaxis? (multiple answers possible) [patients post urinary tract infection/pyelonephritis/urosepsis]
N/A

Which patients receive periinterventional antibiotic prophylaxis? (multiple answers possible) [patients with urinary tract dilation in ultrasound]
N/A

Which patients receive periinterventional antibiotic prophylaxis? (multiple answers possible) [patients with prenatal urinary tract dilation]
N/A

Which patients receive periinterventional antibiotic prophylaxis? (multiple answers possible) [patients post VUR surgery]
N/A

Which patients receive periinterventional antibiotic prophylaxis? (multiple answers possible) [none]
N/A

Are there situations or clinical findings in the patient where you deviate from your prophylaxis standard (medication, dose, duration, age of the patient)? (multiple answers possible, please specify in answer box) [in case of already known vesicoureteral reflux (VUR)]
N/A

Are there situations or clinical findings in the patient where you deviate from your prophylaxis standard (medication, dose, duration, age of the patient)? (multiple answers possible, please specify in answer box) [in patients with recurrent urinary tract infections (UTI)]
N/A

Are there situations or clinical findings in the patient where you deviate from your prophylaxis standard (medication, dose, duration, age of the patient)? (multiple answers possible, please specify in answer box) [if multidrug-resistant organisms (MDRO) are detected in urine cultures]
N/A

Are there situations or clinical findings in the patient where you deviate from your prophylaxis standard (medication, dose, duration, age of the patient)? (multiple answers possible, please specify in answer box) [in newborns]
N/A

Are there situations in which you use intravenous prophylaxis?
N/A

Voiding analysis

Are there differences in antibiotic prophylaxis between VUS and VCUG in your department?
N/A

Is a non-pathological urine strip test required in preparation for VUS/VCUG in your department?
N/A

Is a non-pathological urine culture required in preparation for VUS/VCUG in your department?
N/A

Do patients only receive post-procedure prophylaxis if vesicoureteral reflux (VUR) is detected in VUS/VCUG?
N/A

Do other MUS/MCU findings (e.g. urethral valves) have an influence on post-procedure prophylaxis?
N/A

End of survey

May we contact you personally if we have further questions?
N/A

Survey response 160

Response ID
162

Date submitted
2025-03-25 09:21:59

Last page
5

Start language
en

Seed
251146402

Date started
2025-03-25 09:19:14

Date last action
2025-03-25 09:21:59

Standard Operation Procedure

Who fills out this survey?
Radiologist

Who fills out this survey? [Other]

In which type of hospital are you employed?
University Hospital

In which type of hospital are you employed? [Other]

In which country do you work?
Austria

Does your department have a defined protocol for infection prophylaxis in the context of VUS/VCUG?
No

Has this protocol been transformed into a written standard operating procedure (SOP)?
N/A

Antibiotic Prophylaxis

Are there situations/constellations in which you do not use antibiotic prophylaxis for VUS/VCUG?
Yes

Please specify these situations/constellations: [girl with first uncomplicated UTI without urinary tract dilation in ultrasound]
No

Please specify these situations/constellations: [boy with first uncomplicated UTI without urinary tract dilation in ultrasound]
No

Please specify these situations/constellations: [in patients with low grade urinary tract dilation (up to 2nd grade) and no UTI in medical history]
No

Please specify these situations/constellations: [Other]
Never use antibiotic prophylaxis

If you use antibiotic prophylaxis: which mediaction do you use? (multiple answers and subdivision into age groups possible) [Trimethoprim]
No

If you use antibiotic prophylaxis: which mediaction do you use? (multiple answers and subdivision into age groups possible) [Nitrofurantoin]
No

If you use antibiotic prophylaxis: which mediaction do you use? (multiple answers and subdivision into age groups possible) [Amoxicillin]
No

If you use antibiotic prophylaxis: which mediaction do you use? (multiple answers and subdivision into age groups possible) [Cefaclor]
No

If you use antibiotic prophylaxis: which mediaction do you use? (multiple answers and subdivision into age groups possible) [Amoxicillin/clavulanic acid]
No

If you use antibiotic prophylaxis: which mediaction do you use? (multiple answers and subdivision into age groups possible) [Other]
No

Trimethoprim: which time schedule do you use? [Day(s) before VUS/VCUG, how many?]
N/A

Trimethoprim: which time schedule do you use? [Day of VUS/VCUG]
N/A

Trimethoprim: which time schedule do you use? [Day(s) after VUS/VCUG, how many?]
N/A

Nitrofurantoin: which time schedule do you use? [Day(s) before VUS/VCUG, how many?]
N/A

Nitrofurantoin: which time schedule do you use? [Day of VUS/VCUG]
N/A

Nitrofurantoin: which time schedule do you use? [Day(s) after VUS/VCUG, how many?]
N/A

Amoxicillin: which time schedule do you use? [Day(s) before VUS/VCUG, how many?]
N/A

Amoxicillin: which time schedule do you use? [Day of VUS/VCUG]
N/A

Amoxicillin: which time schedule do you use? [Day(s) after VUS/VCUG, how many?]
N/A

Cefaclor: which time schedule do you use? [Days(s) before VUS/VCUG, how many?]
N/A

Cefaclor: which time schedule do you use? [Day of VUS/VCUG]
N/A

Cefaclor: which time schedule do you use? [Day(s) after VUS/VCUG, how many?]
N/A

Amoxicillin/clavulanic acid: in what dosage? (Amoxicillin portion) [prophylaktic (10-20 mg/Kg bodyweight/d)]
N/A

Amoxicillin/clavulanic acid: in what dosage? (Amoxicillin portion) [double prophylactic (20-40 mg/Kg bodyweight/d)]
N/A

Amoxicillin/clavulanic acid: in what dosage? (Amoxicillin portion) [therapeutic (80-100 mg/Kg bodyweight/d)]
N/A

Amoxicillin/clavulanic acid: which time schedule do you use? [Day(s) before VUS/VCUG]
N/A

Amoxicillin/clavulanic acid: which time schedule do you use? [Day of VUS/VCUG]
N/A

Amoxicillin/clavulanic acid: which time schedule do you use? [Day(s) after VUS/VCUG]
N/A

Other: which time schedule do you use? [Day(s) before VUS/VCUG, how many?]
N/A

Other: which time schedule do you use? [Day of VUS/VCUG]
N/A

Other: which time schedule do you use? [Day(s) after VUS/VCUG, how many?]
N/A

Indication for prophylaxis

Which patients receive periinterventional antibiotic prophylaxis? (multiple answers possible) [all]
No

Which patients receive periinterventional antibiotic prophylaxis? (multiple answers possible) [patients post urinary tract infection/pyelonephritis/urosepsis]
No

Which patients receive periinterventional antibiotic prophylaxis? (multiple answers possible) [patients with urinary tract dilation in ultrasound]
No

Which patients receive periinterventional antibiotic prophylaxis? (multiple answers possible) [patients with prenatal urinary tract dilation]
No

Which patients receive periinterventional antibiotic prophylaxis? (multiple answers possible) [patients post VUR surgery]
No

Which patients receive periinterventional antibiotic prophylaxis? (multiple answers possible) [none]
Yes

Which patients receive periinterventional antibiotic prophylaxis? (multiple answers possible) [Other]

Are there situations or clinical findings in the patient where you deviate from your prophylaxis standard (medication, dose, duration, age of the patient)? (multiple answers possible, please specify in answer box) [in case of already known vesicoureteral reflux (VUR)]
Yes

Are there situations or clinical findings in the patient where you deviate from your prophylaxis standard (medication, dose, duration, age of the patient)? (multiple answers possible, please specify in answer box) [in case of already known vesicoureteral reflux (VUR)][Comment]

Are there situations or clinical findings in the patient where you deviate from your prophylaxis standard (medication, dose, duration, age of the patient)? (multiple answers possible, please specify in answer box) [in patients with recurrent urinary tract infections (UTI)]
No

Are there situations or clinical findings in the patient where you deviate from your prophylaxis standard (medication, dose, duration, age of the patient)? (multiple answers possible, please specify in answer box) [in patients with recurrent urinary tract infections (UTI)][Comment]

Are there situations or clinical findings in the patient where you deviate from your prophylaxis standard (medication, dose, duration, age of the patient)? (multiple answers possible, please specify in answer box) [if multidrug-resistant organisms (MDRO) are detected in urine cultures]
Yes

Are there situations or clinical findings in the patient where you deviate from your prophylaxis standard (medication, dose, duration, age of the patient)? (multiple answers possible, please specify in answer box) [if multidrug-resistant organisms (MDRO) are detected in urine cultures][Comment]

Are there situations or clinical findings in the patient where you deviate from your prophylaxis standard (medication, dose, duration, age of the patient)? (multiple answers possible, please specify in answer box) [in newborns]
No

Are there situations or clinical findings in the patient where you deviate from your prophylaxis standard (medication, dose, duration, age of the patient)? (multiple answers possible, please specify in answer box) [in newborns][Comment]

Are there situations or clinical findings in the patient where you deviate from your prophylaxis standard (medication, dose, duration, age of the patient)? (multiple answers possible, please specify in answer box) [Other]

Are there situations or clinical findings in the patient where you deviate from your prophylaxis standard (medication, dose, duration, age of the patient)? (multiple answers possible, please specify in answer box) [Other comment]

Are there situations in which you use intravenous prophylaxis?
No

Voiding analysis

Are there differences in antibiotic prophylaxis between VUS and VCUG in your department?
No

Is a non-pathological urine strip test required in preparation for VUS/VCUG in your department?
Yes

What is the maximum amount of days accepted for urine strip test to be performed prior to the exam?
1

Is a non-pathological urine culture required in preparation for VUS/VCUG in your department?
No

Do patients only receive post-procedure prophylaxis if vesicoureteral reflux (VUR) is detected in VUS/VCUG?
No

Do other MUS/MCU findings (e.g. urethral valves) have an influence on post-procedure prophylaxis?
No

End of survey

Thank you for taking the time to answer this survey!

May we contact you personally if we have further questions?
No

Survey response 161

Response ID
163

Last page
1

Start language
en

Seed
1672290784

Date started
2025-03-25 09:19:15

Date last action
2025-03-25 09:19:50

Standard Operation Procedure

Who fills out this survey?
Pediatric Radiologist

Who fills out this survey? [Other]

In which type of hospital are you employed?
University Hospital

In which type of hospital are you employed? [Other]

In which country do you work?
Italy

Does your department have a defined protocol for infection prophylaxis in the context of VUS/VCUG?
Yes

Has this protocol been transformed into a written standard operating procedure (SOP)?
No

Antibiotic Prophylaxis

Are there situations/constellations in which you do not use antibiotic prophylaxis for VUS/VCUG?
N/A

Please specify these situations/constellations: [girl with first uncomplicated UTI without urinary tract dilation in ultrasound]
N/A

Please specify these situations/constellations: [boy with first uncomplicated UTI without urinary tract dilation in ultrasound]
N/A

Please specify these situations/constellations: [in patients with low grade urinary tract dilation (up to 2nd grade) and no UTI in medical history]
N/A

If you use antibiotic prophylaxis: which mediaction do you use? (multiple answers and subdivision into age groups possible) [Trimethoprim]
N/A

If you use antibiotic prophylaxis: which mediaction do you use? (multiple answers and subdivision into age groups possible) [Nitrofurantoin]
N/A

If you use antibiotic prophylaxis: which mediaction do you use? (multiple answers and subdivision into age groups possible) [Amoxicillin]
N/A

If you use antibiotic prophylaxis: which mediaction do you use? (multiple answers and subdivision into age groups possible) [Cefaclor]
N/A

If you use antibiotic prophylaxis: which mediaction do you use? (multiple answers and subdivision into age groups possible) [Amoxicillin/clavulanic acid]
N/A

If you use antibiotic prophylaxis: which mediaction do you use? (multiple answers and subdivision into age groups possible) [Other]
N/A

Trimethoprim: which time schedule do you use? [Day(s) before VUS/VCUG, how many?]
N/A

Trimethoprim: which time schedule do you use? [Day of VUS/VCUG]
N/A

Trimethoprim: which time schedule do you use? [Day(s) after VUS/VCUG, how many?]
N/A

Nitrofurantoin: which time schedule do you use? [Day(s) before VUS/VCUG, how many?]
N/A

Nitrofurantoin: which time schedule do you use? [Day of VUS/VCUG]
N/A

Nitrofurantoin: which time schedule do you use? [Day(s) after VUS/VCUG, how many?]
N/A

Amoxicillin: which time schedule do you use? [Day(s) before VUS/VCUG, how many?]
N/A

Amoxicillin: which time schedule do you use? [Day of VUS/VCUG]
N/A

Amoxicillin: which time schedule do you use? [Day(s) after VUS/VCUG, how many?]
N/A

Cefaclor: which time schedule do you use? [Days(s) before VUS/VCUG, how many?]
N/A

Cefaclor: which time schedule do you use? [Day of VUS/VCUG]
N/A

Cefaclor: which time schedule do you use? [Day(s) after VUS/VCUG, how many?]
N/A

Amoxicillin/clavulanic acid: in what dosage? (Amoxicillin portion) [prophylaktic (10-20 mg/Kg bodyweight/d)]
N/A

Amoxicillin/clavulanic acid: in what dosage? (Amoxicillin portion) [double prophylactic (20-40 mg/Kg bodyweight/d)]
N/A

Amoxicillin/clavulanic acid: in what dosage? (Amoxicillin portion) [therapeutic (80-100 mg/Kg bodyweight/d)]
N/A

Amoxicillin/clavulanic acid: which time schedule do you use? [Day(s) before VUS/VCUG]
N/A

Amoxicillin/clavulanic acid: which time schedule do you use? [Day of VUS/VCUG]
N/A

Amoxicillin/clavulanic acid: which time schedule do you use? [Day(s) after VUS/VCUG]
N/A

Other: which time schedule do you use? [Day(s) before VUS/VCUG, how many?]
N/A

Other: which time schedule do you use? [Day of VUS/VCUG]
N/A

Other: which time schedule do you use? [Day(s) after VUS/VCUG, how many?]
N/A

Indication for prophylaxis

Which patients receive periinterventional antibiotic prophylaxis? (multiple answers possible) [all]
N/A

Which patients receive periinterventional antibiotic prophylaxis? (multiple answers possible) [patients post urinary tract infection/pyelonephritis/urosepsis]
N/A

Which patients receive periinterventional antibiotic prophylaxis? (multiple answers possible) [patients with urinary tract dilation in ultrasound]
N/A

Which patients receive periinterventional antibiotic prophylaxis? (multiple answers possible) [patients with prenatal urinary tract dilation]
N/A

Which patients receive periinterventional antibiotic prophylaxis? (multiple answers possible) [patients post VUR surgery]
N/A

Which patients receive periinterventional antibiotic prophylaxis? (multiple answers possible) [none]
N/A

Are there situations or clinical findings in the patient where you deviate from your prophylaxis standard (medication, dose, duration, age of the patient)? (multiple answers possible, please specify in answer box) [in case of already known vesicoureteral reflux (VUR)]
N/A

Are there situations or clinical findings in the patient where you deviate from your prophylaxis standard (medication, dose, duration, age of the patient)? (multiple answers possible, please specify in answer box) [in patients with recurrent urinary tract infections (UTI)]
N/A

Are there situations or clinical findings in the patient where you deviate from your prophylaxis standard (medication, dose, duration, age of the patient)? (multiple answers possible, please specify in answer box) [if multidrug-resistant organisms (MDRO) are detected in urine cultures]
N/A

Are there situations or clinical findings in the patient where you deviate from your prophylaxis standard (medication, dose, duration, age of the patient)? (multiple answers possible, please specify in answer box) [in newborns]
N/A

Are there situations in which you use intravenous prophylaxis?
N/A

Voiding analysis

Are there differences in antibiotic prophylaxis between VUS and VCUG in your department?
N/A

Is a non-pathological urine strip test required in preparation for VUS/VCUG in your department?
N/A

Is a non-pathological urine culture required in preparation for VUS/VCUG in your department?
N/A

Do patients only receive post-procedure prophylaxis if vesicoureteral reflux (VUR) is detected in VUS/VCUG?
N/A

Do other MUS/MCU findings (e.g. urethral valves) have an influence on post-procedure prophylaxis?
N/A

End of survey

May we contact you personally if we have further questions?
N/A

Survey response 162

Response ID
164

Last page
1

Start language
en

Seed
1667202962

Date started
2025-03-25 09:31:07

Date last action
2025-03-25 09:31:30

Standard Operation Procedure

Who fills out this survey?
Pediatric Radiologist

Who fills out this survey? [Other]

In which type of hospital are you employed?
University Hospital

In which type of hospital are you employed? [Other]

In which country do you work?
Finland

Does your department have a defined protocol for infection prophylaxis in the context of VUS/VCUG?
No

Has this protocol been transformed into a written standard operating procedure (SOP)?
N/A

Antibiotic Prophylaxis

Are there situations/constellations in which you do not use antibiotic prophylaxis for VUS/VCUG?
N/A

Please specify these situations/constellations: [girl with first uncomplicated UTI without urinary tract dilation in ultrasound]
N/A

Please specify these situations/constellations: [boy with first uncomplicated UTI without urinary tract dilation in ultrasound]
N/A

Please specify these situations/constellations: [in patients with low grade urinary tract dilation (up to 2nd grade) and no UTI in medical history]
N/A

If you use antibiotic prophylaxis: which mediaction do you use? (multiple answers and subdivision into age groups possible) [Trimethoprim]
N/A

If you use antibiotic prophylaxis: which mediaction do you use? (multiple answers and subdivision into age groups possible) [Nitrofurantoin]
N/A

If you use antibiotic prophylaxis: which mediaction do you use? (multiple answers and subdivision into age groups possible) [Amoxicillin]
N/A

If you use antibiotic prophylaxis: which mediaction do you use? (multiple answers and subdivision into age groups possible) [Cefaclor]
N/A

If you use antibiotic prophylaxis: which mediaction do you use? (multiple answers and subdivision into age groups possible) [Amoxicillin/clavulanic acid]
N/A

If you use antibiotic prophylaxis: which mediaction do you use? (multiple answers and subdivision into age groups possible) [Other]
N/A

Trimethoprim: which time schedule do you use? [Day(s) before VUS/VCUG, how many?]
N/A

Trimethoprim: which time schedule do you use? [Day of VUS/VCUG]
N/A

Trimethoprim: which time schedule do you use? [Day(s) after VUS/VCUG, how many?]
N/A

Nitrofurantoin: which time schedule do you use? [Day(s) before VUS/VCUG, how many?]
N/A

Nitrofurantoin: which time schedule do you use? [Day of VUS/VCUG]
N/A

Nitrofurantoin: which time schedule do you use? [Day(s) after VUS/VCUG, how many?]
N/A

Amoxicillin: which time schedule do you use? [Day(s) before VUS/VCUG, how many?]
N/A

Amoxicillin: which time schedule do you use? [Day of VUS/VCUG]
N/A

Amoxicillin: which time schedule do you use? [Day(s) after VUS/VCUG, how many?]
N/A

Cefaclor: which time schedule do you use? [Days(s) before VUS/VCUG, how many?]
N/A

Cefaclor: which time schedule do you use? [Day of VUS/VCUG]
N/A

Cefaclor: which time schedule do you use? [Day(s) after VUS/VCUG, how many?]
N/A

Amoxicillin/clavulanic acid: in what dosage? (Amoxicillin portion) [prophylaktic (10-20 mg/Kg bodyweight/d)]
N/A

Amoxicillin/clavulanic acid: in what dosage? (Amoxicillin portion) [double prophylactic (20-40 mg/Kg bodyweight/d)]
N/A

Amoxicillin/clavulanic acid: in what dosage? (Amoxicillin portion) [therapeutic (80-100 mg/Kg bodyweight/d)]
N/A

Amoxicillin/clavulanic acid: which time schedule do you use? [Day(s) before VUS/VCUG]
N/A

Amoxicillin/clavulanic acid: which time schedule do you use? [Day of VUS/VCUG]
N/A

Amoxicillin/clavulanic acid: which time schedule do you use? [Day(s) after VUS/VCUG]
N/A

Other: which time schedule do you use? [Day(s) before VUS/VCUG, how many?]
N/A

Other: which time schedule do you use? [Day of VUS/VCUG]
N/A

Other: which time schedule do you use? [Day(s) after VUS/VCUG, how many?]
N/A

Indication for prophylaxis

Which patients receive periinterventional antibiotic prophylaxis? (multiple answers possible) [all]
N/A

Which patients receive periinterventional antibiotic prophylaxis? (multiple answers possible) [patients post urinary tract infection/pyelonephritis/urosepsis]
N/A

Which patients receive periinterventional antibiotic prophylaxis? (multiple answers possible) [patients with urinary tract dilation in ultrasound]
N/A

Which patients receive periinterventional antibiotic prophylaxis? (multiple answers possible) [patients with prenatal urinary tract dilation]
N/A

Which patients receive periinterventional antibiotic prophylaxis? (multiple answers possible) [patients post VUR surgery]
N/A

Which patients receive periinterventional antibiotic prophylaxis? (multiple answers possible) [none]
N/A

Are there situations or clinical findings in the patient where you deviate from your prophylaxis standard (medication, dose, duration, age of the patient)? (multiple answers possible, please specify in answer box) [in case of already known vesicoureteral reflux (VUR)]
N/A

Are there situations or clinical findings in the patient where you deviate from your prophylaxis standard (medication, dose, duration, age of the patient)? (multiple answers possible, please specify in answer box) [in patients with recurrent urinary tract infections (UTI)]
N/A

Are there situations or clinical findings in the patient where you deviate from your prophylaxis standard (medication, dose, duration, age of the patient)? (multiple answers possible, please specify in answer box) [if multidrug-resistant organisms (MDRO) are detected in urine cultures]
N/A

Are there situations or clinical findings in the patient where you deviate from your prophylaxis standard (medication, dose, duration, age of the patient)? (multiple answers possible, please specify in answer box) [in newborns]
N/A

Are there situations in which you use intravenous prophylaxis?
N/A

Voiding analysis

Are there differences in antibiotic prophylaxis between VUS and VCUG in your department?
N/A

Is a non-pathological urine strip test required in preparation for VUS/VCUG in your department?
N/A

Is a non-pathological urine culture required in preparation for VUS/VCUG in your department?
N/A

Do patients only receive post-procedure prophylaxis if vesicoureteral reflux (VUR) is detected in VUS/VCUG?
N/A

Do other MUS/MCU findings (e.g. urethral valves) have an influence on post-procedure prophylaxis?
N/A

End of survey

May we contact you personally if we have further questions?
N/A

Survey response 163

Response ID
165

Last page
1

Start language
en

Seed
1084681016

Date started
2025-03-25 09:42:23

Date last action
2025-03-25 09:42:41

Standard Operation Procedure

Who fills out this survey?
Pediatric Radiologist

Who fills out this survey? [Other]

In which type of hospital are you employed?
University Hospital

In which type of hospital are you employed? [Other]

In which country do you work?
United Kingdom

Does your department have a defined protocol for infection prophylaxis in the context of VUS/VCUG?
Yes

Has this protocol been transformed into a written standard operating procedure (SOP)?
Yes

Which department is the author of the SOP for infection prophylaxis in VUS/VCUG?
Pediatric nephrology

Which department is the author of the SOP for infection prophylaxis in VUS/VCUG? [Other]

Antibiotic Prophylaxis

Are there situations/constellations in which you do not use antibiotic prophylaxis for VUS/VCUG?
N/A

Please specify these situations/constellations: [girl with first uncomplicated UTI without urinary tract dilation in ultrasound]
N/A

Please specify these situations/constellations: [boy with first uncomplicated UTI without urinary tract dilation in ultrasound]
N/A

Please specify these situations/constellations: [in patients with low grade urinary tract dilation (up to 2nd grade) and no UTI in medical history]
N/A

If you use antibiotic prophylaxis: which mediaction do you use? (multiple answers and subdivision into age groups possible) [Trimethoprim]
N/A

If you use antibiotic prophylaxis: which mediaction do you use? (multiple answers and subdivision into age groups possible) [Nitrofurantoin]
N/A

If you use antibiotic prophylaxis: which mediaction do you use? (multiple answers and subdivision into age groups possible) [Amoxicillin]
N/A

If you use antibiotic prophylaxis: which mediaction do you use? (multiple answers and subdivision into age groups possible) [Cefaclor]
N/A

If you use antibiotic prophylaxis: which mediaction do you use? (multiple answers and subdivision into age groups possible) [Amoxicillin/clavulanic acid]
N/A

If you use antibiotic prophylaxis: which mediaction do you use? (multiple answers and subdivision into age groups possible) [Other]
N/A

Trimethoprim: which time schedule do you use? [Day(s) before VUS/VCUG, how many?]
N/A

Trimethoprim: which time schedule do you use? [Day of VUS/VCUG]
N/A

Trimethoprim: which time schedule do you use? [Day(s) after VUS/VCUG, how many?]
N/A

Nitrofurantoin: which time schedule do you use? [Day(s) before VUS/VCUG, how many?]
N/A

Nitrofurantoin: which time schedule do you use? [Day of VUS/VCUG]
N/A

Nitrofurantoin: which time schedule do you use? [Day(s) after VUS/VCUG, how many?]
N/A

Amoxicillin: which time schedule do you use? [Day(s) before VUS/VCUG, how many?]
N/A

Amoxicillin: which time schedule do you use? [Day of VUS/VCUG]
N/A

Amoxicillin: which time schedule do you use? [Day(s) after VUS/VCUG, how many?]
N/A

Cefaclor: which time schedule do you use? [Days(s) before VUS/VCUG, how many?]
N/A

Cefaclor: which time schedule do you use? [Day of VUS/VCUG]
N/A

Cefaclor: which time schedule do you use? [Day(s) after VUS/VCUG, how many?]
N/A

Amoxicillin/clavulanic acid: in what dosage? (Amoxicillin portion) [prophylaktic (10-20 mg/Kg bodyweight/d)]
N/A

Amoxicillin/clavulanic acid: in what dosage? (Amoxicillin portion) [double prophylactic (20-40 mg/Kg bodyweight/d)]
N/A

Amoxicillin/clavulanic acid: in what dosage? (Amoxicillin portion) [therapeutic (80-100 mg/Kg bodyweight/d)]
N/A

Amoxicillin/clavulanic acid: which time schedule do you use? [Day(s) before VUS/VCUG]
N/A

Amoxicillin/clavulanic acid: which time schedule do you use? [Day of VUS/VCUG]
N/A

Amoxicillin/clavulanic acid: which time schedule do you use? [Day(s) after VUS/VCUG]
N/A

Other: which time schedule do you use? [Day(s) before VUS/VCUG, how many?]
N/A

Other: which time schedule do you use? [Day of VUS/VCUG]
N/A

Other: which time schedule do you use? [Day(s) after VUS/VCUG, how many?]
N/A

Indication for prophylaxis

Which patients receive periinterventional antibiotic prophylaxis? (multiple answers possible) [all]
N/A

Which patients receive periinterventional antibiotic prophylaxis? (multiple answers possible) [patients post urinary tract infection/pyelonephritis/urosepsis]
N/A

Which patients receive periinterventional antibiotic prophylaxis? (multiple answers possible) [patients with urinary tract dilation in ultrasound]
N/A

Which patients receive periinterventional antibiotic prophylaxis? (multiple answers possible) [patients with prenatal urinary tract dilation]
N/A

Which patients receive periinterventional antibiotic prophylaxis? (multiple answers possible) [patients post VUR surgery]
N/A

Which patients receive periinterventional antibiotic prophylaxis? (multiple answers possible) [none]
N/A

Are there situations or clinical findings in the patient where you deviate from your prophylaxis standard (medication, dose, duration, age of the patient)? (multiple answers possible, please specify in answer box) [in case of already known vesicoureteral reflux (VUR)]
N/A

Are there situations or clinical findings in the patient where you deviate from your prophylaxis standard (medication, dose, duration, age of the patient)? (multiple answers possible, please specify in answer box) [in patients with recurrent urinary tract infections (UTI)]
N/A

Are there situations or clinical findings in the patient where you deviate from your prophylaxis standard (medication, dose, duration, age of the patient)? (multiple answers possible, please specify in answer box) [if multidrug-resistant organisms (MDRO) are detected in urine cultures]
N/A

Are there situations or clinical findings in the patient where you deviate from your prophylaxis standard (medication, dose, duration, age of the patient)? (multiple answers possible, please specify in answer box) [in newborns]
N/A

Are there situations in which you use intravenous prophylaxis?
N/A

Voiding analysis

Are there differences in antibiotic prophylaxis between VUS and VCUG in your department?
N/A

Is a non-pathological urine strip test required in preparation for VUS/VCUG in your department?
N/A

Is a non-pathological urine culture required in preparation for VUS/VCUG in your department?
N/A

Do patients only receive post-procedure prophylaxis if vesicoureteral reflux (VUR) is detected in VUS/VCUG?
N/A

Do other MUS/MCU findings (e.g. urethral valves) have an influence on post-procedure prophylaxis?
N/A

End of survey

May we contact you personally if we have further questions?
N/A

Survey response 164

Response ID
166

Last page
1

Start language
en

Seed
1027064923

Date started
2025-03-25 09:46:43

Date last action
2025-03-25 09:47:19

Standard Operation Procedure

Who fills out this survey?
Pediatric Radiologist

Who fills out this survey? [Other]

In which type of hospital are you employed?
Other

In which type of hospital are you employed? [Other]

In which country do you work?
Jordan

Does your department have a defined protocol for infection prophylaxis in the context of VUS/VCUG?
No

Has this protocol been transformed into a written standard operating procedure (SOP)?
N/A

Antibiotic Prophylaxis

Are there situations/constellations in which you do not use antibiotic prophylaxis for VUS/VCUG?
N/A

Please specify these situations/constellations: [girl with first uncomplicated UTI without urinary tract dilation in ultrasound]
N/A

Please specify these situations/constellations: [boy with first uncomplicated UTI without urinary tract dilation in ultrasound]
N/A

Please specify these situations/constellations: [in patients with low grade urinary tract dilation (up to 2nd grade) and no UTI in medical history]
N/A

If you use antibiotic prophylaxis: which mediaction do you use? (multiple answers and subdivision into age groups possible) [Trimethoprim]
N/A

If you use antibiotic prophylaxis: which mediaction do you use? (multiple answers and subdivision into age groups possible) [Nitrofurantoin]
N/A

If you use antibiotic prophylaxis: which mediaction do you use? (multiple answers and subdivision into age groups possible) [Amoxicillin]
N/A

If you use antibiotic prophylaxis: which mediaction do you use? (multiple answers and subdivision into age groups possible) [Cefaclor]
N/A

If you use antibiotic prophylaxis: which mediaction do you use? (multiple answers and subdivision into age groups possible) [Amoxicillin/clavulanic acid]
N/A

If you use antibiotic prophylaxis: which mediaction do you use? (multiple answers and subdivision into age groups possible) [Other]
N/A

Trimethoprim: which time schedule do you use? [Day(s) before VUS/VCUG, how many?]
N/A

Trimethoprim: which time schedule do you use? [Day of VUS/VCUG]
N/A

Trimethoprim: which time schedule do you use? [Day(s) after VUS/VCUG, how many?]
N/A

Nitrofurantoin: which time schedule do you use? [Day(s) before VUS/VCUG, how many?]
N/A

Nitrofurantoin: which time schedule do you use? [Day of VUS/VCUG]
N/A

Nitrofurantoin: which time schedule do you use? [Day(s) after VUS/VCUG, how many?]
N/A

Amoxicillin: which time schedule do you use? [Day(s) before VUS/VCUG, how many?]
N/A

Amoxicillin: which time schedule do you use? [Day of VUS/VCUG]
N/A

Amoxicillin: which time schedule do you use? [Day(s) after VUS/VCUG, how many?]
N/A

Cefaclor: which time schedule do you use? [Days(s) before VUS/VCUG, how many?]
N/A

Cefaclor: which time schedule do you use? [Day of VUS/VCUG]
N/A

Cefaclor: which time schedule do you use? [Day(s) after VUS/VCUG, how many?]
N/A

Amoxicillin/clavulanic acid: in what dosage? (Amoxicillin portion) [prophylaktic (10-20 mg/Kg bodyweight/d)]
N/A

Amoxicillin/clavulanic acid: in what dosage? (Amoxicillin portion) [double prophylactic (20-40 mg/Kg bodyweight/d)]
N/A

Amoxicillin/clavulanic acid: in what dosage? (Amoxicillin portion) [therapeutic (80-100 mg/Kg bodyweight/d)]
N/A

Amoxicillin/clavulanic acid: which time schedule do you use? [Day(s) before VUS/VCUG]
N/A

Amoxicillin/clavulanic acid: which time schedule do you use? [Day of VUS/VCUG]
N/A

Amoxicillin/clavulanic acid: which time schedule do you use? [Day(s) after VUS/VCUG]
N/A

Other: which time schedule do you use? [Day(s) before VUS/VCUG, how many?]
N/A

Other: which time schedule do you use? [Day of VUS/VCUG]
N/A

Other: which time schedule do you use? [Day(s) after VUS/VCUG, how many?]
N/A

Indication for prophylaxis

Which patients receive periinterventional antibiotic prophylaxis? (multiple answers possible) [all]
N/A

Which patients receive periinterventional antibiotic prophylaxis? (multiple answers possible) [patients post urinary tract infection/pyelonephritis/urosepsis]
N/A

Which patients receive periinterventional antibiotic prophylaxis? (multiple answers possible) [patients with urinary tract dilation in ultrasound]
N/A

Which patients receive periinterventional antibiotic prophylaxis? (multiple answers possible) [patients with prenatal urinary tract dilation]
N/A

Which patients receive periinterventional antibiotic prophylaxis? (multiple answers possible) [patients post VUR surgery]
N/A

Which patients receive periinterventional antibiotic prophylaxis? (multiple answers possible) [none]
N/A

Are there situations or clinical findings in the patient where you deviate from your prophylaxis standard (medication, dose, duration, age of the patient)? (multiple answers possible, please specify in answer box) [in case of already known vesicoureteral reflux (VUR)]
N/A

Are there situations or clinical findings in the patient where you deviate from your prophylaxis standard (medication, dose, duration, age of the patient)? (multiple answers possible, please specify in answer box) [in patients with recurrent urinary tract infections (UTI)]
N/A

Are there situations or clinical findings in the patient where you deviate from your prophylaxis standard (medication, dose, duration, age of the patient)? (multiple answers possible, please specify in answer box) [if multidrug-resistant organisms (MDRO) are detected in urine cultures]
N/A

Are there situations or clinical findings in the patient where you deviate from your prophylaxis standard (medication, dose, duration, age of the patient)? (multiple answers possible, please specify in answer box) [in newborns]
N/A

Are there situations in which you use intravenous prophylaxis?
N/A

Voiding analysis

Are there differences in antibiotic prophylaxis between VUS and VCUG in your department?
N/A

Is a non-pathological urine strip test required in preparation for VUS/VCUG in your department?
N/A

Is a non-pathological urine culture required in preparation for VUS/VCUG in your department?
N/A

Do patients only receive post-procedure prophylaxis if vesicoureteral reflux (VUR) is detected in VUS/VCUG?
N/A

Do other MUS/MCU findings (e.g. urethral valves) have an influence on post-procedure prophylaxis?
N/A

End of survey

May we contact you personally if we have further questions?
N/A

Survey response 165

Response ID
167

Last page
3

Start language
en

Seed
1717762120

Date started
2025-03-25 09:58:36

Date last action
2025-03-25 10:03:12

Standard Operation Procedure

Who fills out this survey?
Pediatric Radiologist

Who fills out this survey? [Other]

In which type of hospital are you employed?
University Hospital

In which type of hospital are you employed? [Other]

In which country do you work?
New Zealand

Does your department have a defined protocol for infection prophylaxis in the context of VUS/VCUG?
Yes

Has this protocol been transformed into a written standard operating procedure (SOP)?
No

Antibiotic Prophylaxis

Are there situations/constellations in which you do not use antibiotic prophylaxis for VUS/VCUG?
Yes

Please specify these situations/constellations: [girl with first uncomplicated UTI without urinary tract dilation in ultrasound]
No

Please specify these situations/constellations: [boy with first uncomplicated UTI without urinary tract dilation in ultrasound]
No

Please specify these situations/constellations: [in patients with low grade urinary tract dilation (up to 2nd grade) and no UTI in medical history]
No

Please specify these situations/constellations: [Other]
Under 6 months of age

If you use antibiotic prophylaxis: which mediaction do you use? (multiple answers and subdivision into age groups possible) [Trimethoprim]
No

If you use antibiotic prophylaxis: which mediaction do you use? (multiple answers and subdivision into age groups possible) [Nitrofurantoin]
No

If you use antibiotic prophylaxis: which mediaction do you use? (multiple answers and subdivision into age groups possible) [Amoxicillin]
No

If you use antibiotic prophylaxis: which mediaction do you use? (multiple answers and subdivision into age groups possible) [Cefaclor]
Yes

If you use antibiotic prophylaxis: which mediaction do you use? (multiple answers and subdivision into age groups possible) [Amoxicillin/clavulanic acid]
Yes

If you use antibiotic prophylaxis: which mediaction do you use? (multiple answers and subdivision into age groups possible) [Other]
No

Trimethoprim: which time schedule do you use? [Day(s) before VUS/VCUG, how many?]
N/A

Trimethoprim: which time schedule do you use? [Day of VUS/VCUG]
N/A

Trimethoprim: which time schedule do you use? [Day(s) after VUS/VCUG, how many?]
N/A

Nitrofurantoin: which time schedule do you use? [Day(s) before VUS/VCUG, how many?]
N/A

Nitrofurantoin: which time schedule do you use? [Day of VUS/VCUG]
N/A

Nitrofurantoin: which time schedule do you use? [Day(s) after VUS/VCUG, how many?]
N/A

Amoxicillin: which time schedule do you use? [Day(s) before VUS/VCUG, how many?]
N/A

Amoxicillin: which time schedule do you use? [Day of VUS/VCUG]
N/A

Amoxicillin: which time schedule do you use? [Day(s) after VUS/VCUG, how many?]
N/A

Cefaclor: in what dosage?
double prophylactic (20 mg/kg bodyweight/d)

Cefaclor: in what dosage? [Other]

Cefaclor: for which age group do you use cefaclor?
Discuss with paediatriciand

Cefaclor: which time schedule do you use? [Days(s) before VUS/VCUG, how many?]
No

Cefaclor: which time schedule do you use? [Days(s) before VUS/VCUG, how many?][Comment]

Cefaclor: which time schedule do you use? [Day of VUS/VCUG]
Yes

Cefaclor: which time schedule do you use? [Day of VUS/VCUG][Comment]

Cefaclor: which time schedule do you use? [Day(s) after VUS/VCUG, how many?]
No

Cefaclor: which time schedule do you use? [Day(s) after VUS/VCUG, how many?][Comment]

Amoxicillin/clavulanic acid: in what dosage? (Amoxicillin portion) [prophylaktic (10-20 mg/Kg bodyweight/d)]
No

Amoxicillin/clavulanic acid: in what dosage? (Amoxicillin portion) [double prophylactic (20-40 mg/Kg bodyweight/d)]
Yes

Amoxicillin/clavulanic acid: in what dosage? (Amoxicillin portion) [therapeutic (80-100 mg/Kg bodyweight/d)]
No

Amoxicillin/clavulanic acid: in what dosage? (Amoxicillin portion) [Other]

Amoxicillin/clavulanic acid: for which age group do you use amoxicillin/clavulanic acid?
Discuss with oseduatricians

Amoxicillin/clavulanic acid: which time schedule do you use? [Day(s) before VUS/VCUG]
No

Amoxicillin/clavulanic acid: which time schedule do you use? [Day(s) before VUS/VCUG][Comment]

Amoxicillin/clavulanic acid: which time schedule do you use? [Day of VUS/VCUG]
Yes

Amoxicillin/clavulanic acid: which time schedule do you use? [Day of VUS/VCUG][Comment]

Amoxicillin/clavulanic acid: which time schedule do you use? [Day(s) after VUS/VCUG]
No

Amoxicillin/clavulanic acid: which time schedule do you use? [Day(s) after VUS/VCUG][Comment]

Other: which time schedule do you use? [Day(s) before VUS/VCUG, how many?]
N/A

Other: which time schedule do you use? [Day of VUS/VCUG]
N/A

Other: which time schedule do you use? [Day(s) after VUS/VCUG, how many?]
N/A

Indication for prophylaxis

Which patients receive periinterventional antibiotic prophylaxis? (multiple answers possible) [all]
No

Which patients receive periinterventional antibiotic prophylaxis? (multiple answers possible) [patients post urinary tract infection/pyelonephritis/urosepsis]
No

Which patients receive periinterventional antibiotic prophylaxis? (multiple answers possible) [patients with urinary tract dilation in ultrasound]
No

Which patients receive periinterventional antibiotic prophylaxis? (multiple answers possible) [patients with prenatal urinary tract dilation]
No

Which patients receive periinterventional antibiotic prophylaxis? (multiple answers possible) [patients post VUR surgery]
No

Which patients receive periinterventional antibiotic prophylaxis? (multiple answers possible) [none]
No

Which patients receive periinterventional antibiotic prophylaxis? (multiple answers possible) [Other]
All under 6 months of age

Are there situations or clinical findings in the patient where you deviate from your prophylaxis standard (medication, dose, duration, age of the patient)? (multiple answers possible, please specify in answer box) [in case of already known vesicoureteral reflux (VUR)]
No

Are there situations or clinical findings in the patient where you deviate from your prophylaxis standard (medication, dose, duration, age of the patient)? (multiple answers possible, please specify in answer box) [in case of already known vesicoureteral reflux (VUR)][Comment]

Are there situations or clinical findings in the patient where you deviate from your prophylaxis standard (medication, dose, duration, age of the patient)? (multiple answers possible, please specify in answer box) [in patients with recurrent urinary tract infections (UTI)]
No

Are there situations or clinical findings in the patient where you deviate from your prophylaxis standard (medication, dose, duration, age of the patient)? (multiple answers possible, please specify in answer box) [in patients with recurrent urinary tract infections (UTI)][Comment]

Are there situations or clinical findings in the patient where you deviate from your prophylaxis standard (medication, dose, duration, age of the patient)? (multiple answers possible, please specify in answer box) [if multidrug-resistant organisms (MDRO) are detected in urine cultures]
No

Are there situations or clinical findings in the patient where you deviate from your prophylaxis standard (medication, dose, duration, age of the patient)? (multiple answers possible, please specify in answer box) [if multidrug-resistant organisms (MDRO) are detected in urine cultures][Comment]

Are there situations or clinical findings in the patient where you deviate from your prophylaxis standard (medication, dose, duration, age of the patient)? (multiple answers possible, please specify in answer box) [in newborns]
Yes

Are there situations or clinical findings in the patient where you deviate from your prophylaxis standard (medication, dose, duration, age of the patient)? (multiple answers possible, please specify in answer box) [in newborns][Comment]

Are there situations or clinical findings in the patient where you deviate from your prophylaxis standard (medication, dose, duration, age of the patient)? (multiple answers possible, please specify in answer box) [Other]

Are there situations or clinical findings in the patient where you deviate from your prophylaxis standard (medication, dose, duration, age of the patient)? (multiple answers possible, please specify in answer box) [Other comment]

Are there situations in which you use intravenous prophylaxis?
No

Voiding analysis

Are there differences in antibiotic prophylaxis between VUS and VCUG in your department?
N/A

Is a non-pathological urine strip test required in preparation for VUS/VCUG in your department?
N/A

Is a non-pathological urine culture required in preparation for VUS/VCUG in your department?
N/A

Do patients only receive post-procedure prophylaxis if vesicoureteral reflux (VUR) is detected in VUS/VCUG?
N/A

Do other MUS/MCU findings (e.g. urethral valves) have an influence on post-procedure prophylaxis?
N/A

End of survey

May we contact you personally if we have further questions?
N/A

Survey response 166

Response ID
168

Last page
0

Start language
en

Seed
2057948614

Date started
2025-03-25 10:05:58

Date last action
2025-03-25 10:05:58

Standard Operation Procedure

Does your department have a defined protocol for infection prophylaxis in the context of VUS/VCUG?
N/A

Has this protocol been transformed into a written standard operating procedure (SOP)?
N/A

Antibiotic Prophylaxis

Are there situations/constellations in which you do not use antibiotic prophylaxis for VUS/VCUG?
N/A

Please specify these situations/constellations: [girl with first uncomplicated UTI without urinary tract dilation in ultrasound]
N/A

Please specify these situations/constellations: [boy with first uncomplicated UTI without urinary tract dilation in ultrasound]
N/A

Please specify these situations/constellations: [in patients with low grade urinary tract dilation (up to 2nd grade) and no UTI in medical history]
N/A

If you use antibiotic prophylaxis: which mediaction do you use? (multiple answers and subdivision into age groups possible) [Trimethoprim]
N/A

If you use antibiotic prophylaxis: which mediaction do you use? (multiple answers and subdivision into age groups possible) [Nitrofurantoin]
N/A

If you use antibiotic prophylaxis: which mediaction do you use? (multiple answers and subdivision into age groups possible) [Amoxicillin]
N/A

If you use antibiotic prophylaxis: which mediaction do you use? (multiple answers and subdivision into age groups possible) [Cefaclor]
N/A

If you use antibiotic prophylaxis: which mediaction do you use? (multiple answers and subdivision into age groups possible) [Amoxicillin/clavulanic acid]
N/A

If you use antibiotic prophylaxis: which mediaction do you use? (multiple answers and subdivision into age groups possible) [Other]
N/A

Trimethoprim: which time schedule do you use? [Day(s) before VUS/VCUG, how many?]
N/A

Trimethoprim: which time schedule do you use? [Day of VUS/VCUG]
N/A

Trimethoprim: which time schedule do you use? [Day(s) after VUS/VCUG, how many?]
N/A

Nitrofurantoin: which time schedule do you use? [Day(s) before VUS/VCUG, how many?]
N/A

Nitrofurantoin: which time schedule do you use? [Day of VUS/VCUG]
N/A

Nitrofurantoin: which time schedule do you use? [Day(s) after VUS/VCUG, how many?]
N/A

Amoxicillin: which time schedule do you use? [Day(s) before VUS/VCUG, how many?]
N/A

Amoxicillin: which time schedule do you use? [Day of VUS/VCUG]
N/A

Amoxicillin: which time schedule do you use? [Day(s) after VUS/VCUG, how many?]
N/A

Cefaclor: which time schedule do you use? [Days(s) before VUS/VCUG, how many?]
N/A

Cefaclor: which time schedule do you use? [Day of VUS/VCUG]
N/A

Cefaclor: which time schedule do you use? [Day(s) after VUS/VCUG, how many?]
N/A

Amoxicillin/clavulanic acid: in what dosage? (Amoxicillin portion) [prophylaktic (10-20 mg/Kg bodyweight/d)]
N/A

Amoxicillin/clavulanic acid: in what dosage? (Amoxicillin portion) [double prophylactic (20-40 mg/Kg bodyweight/d)]
N/A

Amoxicillin/clavulanic acid: in what dosage? (Amoxicillin portion) [therapeutic (80-100 mg/Kg bodyweight/d)]
N/A

Amoxicillin/clavulanic acid: which time schedule do you use? [Day(s) before VUS/VCUG]
N/A

Amoxicillin/clavulanic acid: which time schedule do you use? [Day of VUS/VCUG]
N/A

Amoxicillin/clavulanic acid: which time schedule do you use? [Day(s) after VUS/VCUG]
N/A

Other: which time schedule do you use? [Day(s) before VUS/VCUG, how many?]
N/A

Other: which time schedule do you use? [Day of VUS/VCUG]
N/A

Other: which time schedule do you use? [Day(s) after VUS/VCUG, how many?]
N/A

Indication for prophylaxis

Which patients receive periinterventional antibiotic prophylaxis? (multiple answers possible) [all]
N/A

Which patients receive periinterventional antibiotic prophylaxis? (multiple answers possible) [patients post urinary tract infection/pyelonephritis/urosepsis]
N/A

Which patients receive periinterventional antibiotic prophylaxis? (multiple answers possible) [patients with urinary tract dilation in ultrasound]
N/A

Which patients receive periinterventional antibiotic prophylaxis? (multiple answers possible) [patients with prenatal urinary tract dilation]
N/A

Which patients receive periinterventional antibiotic prophylaxis? (multiple answers possible) [patients post VUR surgery]
N/A

Which patients receive periinterventional antibiotic prophylaxis? (multiple answers possible) [none]
N/A

Are there situations or clinical findings in the patient where you deviate from your prophylaxis standard (medication, dose, duration, age of the patient)? (multiple answers possible, please specify in answer box) [in case of already known vesicoureteral reflux (VUR)]
N/A

Are there situations or clinical findings in the patient where you deviate from your prophylaxis standard (medication, dose, duration, age of the patient)? (multiple answers possible, please specify in answer box) [in patients with recurrent urinary tract infections (UTI)]
N/A

Are there situations or clinical findings in the patient where you deviate from your prophylaxis standard (medication, dose, duration, age of the patient)? (multiple answers possible, please specify in answer box) [if multidrug-resistant organisms (MDRO) are detected in urine cultures]
N/A

Are there situations or clinical findings in the patient where you deviate from your prophylaxis standard (medication, dose, duration, age of the patient)? (multiple answers possible, please specify in answer box) [in newborns]
N/A

Are there situations in which you use intravenous prophylaxis?
N/A

Voiding analysis

Are there differences in antibiotic prophylaxis between VUS and VCUG in your department?
N/A

Is a non-pathological urine strip test required in preparation for VUS/VCUG in your department?
N/A

Is a non-pathological urine culture required in preparation for VUS/VCUG in your department?
N/A

Do patients only receive post-procedure prophylaxis if vesicoureteral reflux (VUR) is detected in VUS/VCUG?
N/A

Do other MUS/MCU findings (e.g. urethral valves) have an influence on post-procedure prophylaxis?
N/A

End of survey

May we contact you personally if we have further questions?
N/A

Survey response 167

Response ID
169

Last page
1

Start language
en

Seed
2123497964

Date started
2025-03-25 10:11:47

Date last action
2025-03-25 10:12:27

Standard Operation Procedure

Who fills out this survey?
Pediatric Radiologist

Who fills out this survey? [Other]

In which type of hospital are you employed?
University Hospital

In which type of hospital are you employed? [Other]

In which country do you work?
The Netherlands

Does your department have a defined protocol for infection prophylaxis in the context of VUS/VCUG?
No

Has this protocol been transformed into a written standard operating procedure (SOP)?
N/A

Antibiotic Prophylaxis

Are there situations/constellations in which you do not use antibiotic prophylaxis for VUS/VCUG?
N/A

Please specify these situations/constellations: [girl with first uncomplicated UTI without urinary tract dilation in ultrasound]
N/A

Please specify these situations/constellations: [boy with first uncomplicated UTI without urinary tract dilation in ultrasound]
N/A

Please specify these situations/constellations: [in patients with low grade urinary tract dilation (up to 2nd grade) and no UTI in medical history]
N/A

If you use antibiotic prophylaxis: which mediaction do you use? (multiple answers and subdivision into age groups possible) [Trimethoprim]
N/A

If you use antibiotic prophylaxis: which mediaction do you use? (multiple answers and subdivision into age groups possible) [Nitrofurantoin]
N/A

If you use antibiotic prophylaxis: which mediaction do you use? (multiple answers and subdivision into age groups possible) [Amoxicillin]
N/A

If you use antibiotic prophylaxis: which mediaction do you use? (multiple answers and subdivision into age groups possible) [Cefaclor]
N/A

If you use antibiotic prophylaxis: which mediaction do you use? (multiple answers and subdivision into age groups possible) [Amoxicillin/clavulanic acid]
N/A

If you use antibiotic prophylaxis: which mediaction do you use? (multiple answers and subdivision into age groups possible) [Other]
N/A

Trimethoprim: which time schedule do you use? [Day(s) before VUS/VCUG, how many?]
N/A

Trimethoprim: which time schedule do you use? [Day of VUS/VCUG]
N/A

Trimethoprim: which time schedule do you use? [Day(s) after VUS/VCUG, how many?]
N/A

Nitrofurantoin: which time schedule do you use? [Day(s) before VUS/VCUG, how many?]
N/A

Nitrofurantoin: which time schedule do you use? [Day of VUS/VCUG]
N/A

Nitrofurantoin: which time schedule do you use? [Day(s) after VUS/VCUG, how many?]
N/A

Amoxicillin: which time schedule do you use? [Day(s) before VUS/VCUG, how many?]
N/A

Amoxicillin: which time schedule do you use? [Day of VUS/VCUG]
N/A

Amoxicillin: which time schedule do you use? [Day(s) after VUS/VCUG, how many?]
N/A

Cefaclor: which time schedule do you use? [Days(s) before VUS/VCUG, how many?]
N/A

Cefaclor: which time schedule do you use? [Day of VUS/VCUG]
N/A

Cefaclor: which time schedule do you use? [Day(s) after VUS/VCUG, how many?]
N/A

Amoxicillin/clavulanic acid: in what dosage? (Amoxicillin portion) [prophylaktic (10-20 mg/Kg bodyweight/d)]
N/A

Amoxicillin/clavulanic acid: in what dosage? (Amoxicillin portion) [double prophylactic (20-40 mg/Kg bodyweight/d)]
N/A

Amoxicillin/clavulanic acid: in what dosage? (Amoxicillin portion) [therapeutic (80-100 mg/Kg bodyweight/d)]
N/A

Amoxicillin/clavulanic acid: which time schedule do you use? [Day(s) before VUS/VCUG]
N/A

Amoxicillin/clavulanic acid: which time schedule do you use? [Day of VUS/VCUG]
N/A

Amoxicillin/clavulanic acid: which time schedule do you use? [Day(s) after VUS/VCUG]
N/A

Other: which time schedule do you use? [Day(s) before VUS/VCUG, how many?]
N/A

Other: which time schedule do you use? [Day of VUS/VCUG]
N/A

Other: which time schedule do you use? [Day(s) after VUS/VCUG, how many?]
N/A

Indication for prophylaxis

Which patients receive periinterventional antibiotic prophylaxis? (multiple answers possible) [all]
N/A

Which patients receive periinterventional antibiotic prophylaxis? (multiple answers possible) [patients post urinary tract infection/pyelonephritis/urosepsis]
N/A

Which patients receive periinterventional antibiotic prophylaxis? (multiple answers possible) [patients with urinary tract dilation in ultrasound]
N/A

Which patients receive periinterventional antibiotic prophylaxis? (multiple answers possible) [patients with prenatal urinary tract dilation]
N/A

Which patients receive periinterventional antibiotic prophylaxis? (multiple answers possible) [patients post VUR surgery]
N/A

Which patients receive periinterventional antibiotic prophylaxis? (multiple answers possible) [none]
N/A

Are there situations or clinical findings in the patient where you deviate from your prophylaxis standard (medication, dose, duration, age of the patient)? (multiple answers possible, please specify in answer box) [in case of already known vesicoureteral reflux (VUR)]
N/A

Are there situations or clinical findings in the patient where you deviate from your prophylaxis standard (medication, dose, duration, age of the patient)? (multiple answers possible, please specify in answer box) [in patients with recurrent urinary tract infections (UTI)]
N/A

Are there situations or clinical findings in the patient where you deviate from your prophylaxis standard (medication, dose, duration, age of the patient)? (multiple answers possible, please specify in answer box) [if multidrug-resistant organisms (MDRO) are detected in urine cultures]
N/A

Are there situations or clinical findings in the patient where you deviate from your prophylaxis standard (medication, dose, duration, age of the patient)? (multiple answers possible, please specify in answer box) [in newborns]
N/A

Are there situations in which you use intravenous prophylaxis?
N/A

Voiding analysis

Are there differences in antibiotic prophylaxis between VUS and VCUG in your department?
N/A

Is a non-pathological urine strip test required in preparation for VUS/VCUG in your department?
N/A

Is a non-pathological urine culture required in preparation for VUS/VCUG in your department?
N/A

Do patients only receive post-procedure prophylaxis if vesicoureteral reflux (VUR) is detected in VUS/VCUG?
N/A

Do other MUS/MCU findings (e.g. urethral valves) have an influence on post-procedure prophylaxis?
N/A

End of survey

May we contact you personally if we have further questions?
N/A

Survey response 168

Response ID
170

Last page
1

Start language
en

Seed
128436659

Date started
2025-03-25 10:20:27

Date last action
2025-03-25 10:21:11

Standard Operation Procedure

Who fills out this survey?
Pediatric Radiologist

Who fills out this survey? [Other]

In which type of hospital are you employed?
Community hospital (>50 beds for pediatric patients)

In which type of hospital are you employed? [Other]

In which country do you work?
Germany

Does your department have a defined protocol for infection prophylaxis in the context of VUS/VCUG?
Yes

Has this protocol been transformed into a written standard operating procedure (SOP)?
No

Antibiotic Prophylaxis

Are there situations/constellations in which you do not use antibiotic prophylaxis for VUS/VCUG?
N/A

Please specify these situations/constellations: [girl with first uncomplicated UTI without urinary tract dilation in ultrasound]
N/A

Please specify these situations/constellations: [boy with first uncomplicated UTI without urinary tract dilation in ultrasound]
N/A

Please specify these situations/constellations: [in patients with low grade urinary tract dilation (up to 2nd grade) and no UTI in medical history]
N/A

If you use antibiotic prophylaxis: which mediaction do you use? (multiple answers and subdivision into age groups possible) [Trimethoprim]
N/A

If you use antibiotic prophylaxis: which mediaction do you use? (multiple answers and subdivision into age groups possible) [Nitrofurantoin]
N/A

If you use antibiotic prophylaxis: which mediaction do you use? (multiple answers and subdivision into age groups possible) [Amoxicillin]
N/A

If you use antibiotic prophylaxis: which mediaction do you use? (multiple answers and subdivision into age groups possible) [Cefaclor]
N/A

If you use antibiotic prophylaxis: which mediaction do you use? (multiple answers and subdivision into age groups possible) [Amoxicillin/clavulanic acid]
N/A

If you use antibiotic prophylaxis: which mediaction do you use? (multiple answers and subdivision into age groups possible) [Other]
N/A

Trimethoprim: which time schedule do you use? [Day(s) before VUS/VCUG, how many?]
N/A

Trimethoprim: which time schedule do you use? [Day of VUS/VCUG]
N/A

Trimethoprim: which time schedule do you use? [Day(s) after VUS/VCUG, how many?]
N/A

Nitrofurantoin: which time schedule do you use? [Day(s) before VUS/VCUG, how many?]
N/A

Nitrofurantoin: which time schedule do you use? [Day of VUS/VCUG]
N/A

Nitrofurantoin: which time schedule do you use? [Day(s) after VUS/VCUG, how many?]
N/A

Amoxicillin: which time schedule do you use? [Day(s) before VUS/VCUG, how many?]
N/A

Amoxicillin: which time schedule do you use? [Day of VUS/VCUG]
N/A

Amoxicillin: which time schedule do you use? [Day(s) after VUS/VCUG, how many?]
N/A

Cefaclor: which time schedule do you use? [Days(s) before VUS/VCUG, how many?]
N/A

Cefaclor: which time schedule do you use? [Day of VUS/VCUG]
N/A

Cefaclor: which time schedule do you use? [Day(s) after VUS/VCUG, how many?]
N/A

Amoxicillin/clavulanic acid: in what dosage? (Amoxicillin portion) [prophylaktic (10-20 mg/Kg bodyweight/d)]
N/A

Amoxicillin/clavulanic acid: in what dosage? (Amoxicillin portion) [double prophylactic (20-40 mg/Kg bodyweight/d)]
N/A

Amoxicillin/clavulanic acid: in what dosage? (Amoxicillin portion) [therapeutic (80-100 mg/Kg bodyweight/d)]
N/A

Amoxicillin/clavulanic acid: which time schedule do you use? [Day(s) before VUS/VCUG]
N/A

Amoxicillin/clavulanic acid: which time schedule do you use? [Day of VUS/VCUG]
N/A

Amoxicillin/clavulanic acid: which time schedule do you use? [Day(s) after VUS/VCUG]
N/A

Other: which time schedule do you use? [Day(s) before VUS/VCUG, how many?]
N/A

Other: which time schedule do you use? [Day of VUS/VCUG]
N/A

Other: which time schedule do you use? [Day(s) after VUS/VCUG, how many?]
N/A

Indication for prophylaxis

Which patients receive periinterventional antibiotic prophylaxis? (multiple answers possible) [all]
N/A

Which patients receive periinterventional antibiotic prophylaxis? (multiple answers possible) [patients post urinary tract infection/pyelonephritis/urosepsis]
N/A

Which patients receive periinterventional antibiotic prophylaxis? (multiple answers possible) [patients with urinary tract dilation in ultrasound]
N/A

Which patients receive periinterventional antibiotic prophylaxis? (multiple answers possible) [patients with prenatal urinary tract dilation]
N/A

Which patients receive periinterventional antibiotic prophylaxis? (multiple answers possible) [patients post VUR surgery]
N/A

Which patients receive periinterventional antibiotic prophylaxis? (multiple answers possible) [none]
N/A

Are there situations or clinical findings in the patient where you deviate from your prophylaxis standard (medication, dose, duration, age of the patient)? (multiple answers possible, please specify in answer box) [in case of already known vesicoureteral reflux (VUR)]
N/A

Are there situations or clinical findings in the patient where you deviate from your prophylaxis standard (medication, dose, duration, age of the patient)? (multiple answers possible, please specify in answer box) [in patients with recurrent urinary tract infections (UTI)]
N/A

Are there situations or clinical findings in the patient where you deviate from your prophylaxis standard (medication, dose, duration, age of the patient)? (multiple answers possible, please specify in answer box) [if multidrug-resistant organisms (MDRO) are detected in urine cultures]
N/A

Are there situations or clinical findings in the patient where you deviate from your prophylaxis standard (medication, dose, duration, age of the patient)? (multiple answers possible, please specify in answer box) [in newborns]
N/A

Are there situations in which you use intravenous prophylaxis?
N/A

Voiding analysis

Are there differences in antibiotic prophylaxis between VUS and VCUG in your department?
N/A

Is a non-pathological urine strip test required in preparation for VUS/VCUG in your department?
N/A

Is a non-pathological urine culture required in preparation for VUS/VCUG in your department?
N/A

Do patients only receive post-procedure prophylaxis if vesicoureteral reflux (VUR) is detected in VUS/VCUG?
N/A

Do other MUS/MCU findings (e.g. urethral valves) have an influence on post-procedure prophylaxis?
N/A

End of survey

May we contact you personally if we have further questions?
N/A

Survey response 169

Response ID
171

Last page
1

Start language
en

Seed
470318772

Date started
2025-03-25 10:47:31

Date last action
2025-03-25 10:48:15

Standard Operation Procedure

Who fills out this survey?
Pediatric Radiologist

Who fills out this survey? [Other]

In which type of hospital are you employed?
University Hospital

In which type of hospital are you employed? [Other]

In which country do you work?
Germany

Does your department have a defined protocol for infection prophylaxis in the context of VUS/VCUG?
No

Has this protocol been transformed into a written standard operating procedure (SOP)?
N/A

Antibiotic Prophylaxis

Are there situations/constellations in which you do not use antibiotic prophylaxis for VUS/VCUG?
N/A

Please specify these situations/constellations: [girl with first uncomplicated UTI without urinary tract dilation in ultrasound]
N/A

Please specify these situations/constellations: [boy with first uncomplicated UTI without urinary tract dilation in ultrasound]
N/A

Please specify these situations/constellations: [in patients with low grade urinary tract dilation (up to 2nd grade) and no UTI in medical history]
N/A

If you use antibiotic prophylaxis: which mediaction do you use? (multiple answers and subdivision into age groups possible) [Trimethoprim]
N/A

If you use antibiotic prophylaxis: which mediaction do you use? (multiple answers and subdivision into age groups possible) [Nitrofurantoin]
N/A

If you use antibiotic prophylaxis: which mediaction do you use? (multiple answers and subdivision into age groups possible) [Amoxicillin]
N/A

If you use antibiotic prophylaxis: which mediaction do you use? (multiple answers and subdivision into age groups possible) [Cefaclor]
N/A

If you use antibiotic prophylaxis: which mediaction do you use? (multiple answers and subdivision into age groups possible) [Amoxicillin/clavulanic acid]
N/A

If you use antibiotic prophylaxis: which mediaction do you use? (multiple answers and subdivision into age groups possible) [Other]
N/A

Trimethoprim: which time schedule do you use? [Day(s) before VUS/VCUG, how many?]
N/A

Trimethoprim: which time schedule do you use? [Day of VUS/VCUG]
N/A

Trimethoprim: which time schedule do you use? [Day(s) after VUS/VCUG, how many?]
N/A

Nitrofurantoin: which time schedule do you use? [Day(s) before VUS/VCUG, how many?]
N/A

Nitrofurantoin: which time schedule do you use? [Day of VUS/VCUG]
N/A

Nitrofurantoin: which time schedule do you use? [Day(s) after VUS/VCUG, how many?]
N/A

Amoxicillin: which time schedule do you use? [Day(s) before VUS/VCUG, how many?]
N/A

Amoxicillin: which time schedule do you use? [Day of VUS/VCUG]
N/A

Amoxicillin: which time schedule do you use? [Day(s) after VUS/VCUG, how many?]
N/A

Cefaclor: which time schedule do you use? [Days(s) before VUS/VCUG, how many?]
N/A

Cefaclor: which time schedule do you use? [Day of VUS/VCUG]
N/A

Cefaclor: which time schedule do you use? [Day(s) after VUS/VCUG, how many?]
N/A

Amoxicillin/clavulanic acid: in what dosage? (Amoxicillin portion) [prophylaktic (10-20 mg/Kg bodyweight/d)]
N/A

Amoxicillin/clavulanic acid: in what dosage? (Amoxicillin portion) [double prophylactic (20-40 mg/Kg bodyweight/d)]
N/A

Amoxicillin/clavulanic acid: in what dosage? (Amoxicillin portion) [therapeutic (80-100 mg/Kg bodyweight/d)]
N/A

Amoxicillin/clavulanic acid: which time schedule do you use? [Day(s) before VUS/VCUG]
N/A

Amoxicillin/clavulanic acid: which time schedule do you use? [Day of VUS/VCUG]
N/A

Amoxicillin/clavulanic acid: which time schedule do you use? [Day(s) after VUS/VCUG]
N/A

Other: which time schedule do you use? [Day(s) before VUS/VCUG, how many?]
N/A

Other: which time schedule do you use? [Day of VUS/VCUG]
N/A

Other: which time schedule do you use? [Day(s) after VUS/VCUG, how many?]
N/A

Indication for prophylaxis

Which patients receive periinterventional antibiotic prophylaxis? (multiple answers possible) [all]
N/A

Which patients receive periinterventional antibiotic prophylaxis? (multiple answers possible) [patients post urinary tract infection/pyelonephritis/urosepsis]
N/A

Which patients receive periinterventional antibiotic prophylaxis? (multiple answers possible) [patients with urinary tract dilation in ultrasound]
N/A

Which patients receive periinterventional antibiotic prophylaxis? (multiple answers possible) [patients with prenatal urinary tract dilation]
N/A

Which patients receive periinterventional antibiotic prophylaxis? (multiple answers possible) [patients post VUR surgery]
N/A

Which patients receive periinterventional antibiotic prophylaxis? (multiple answers possible) [none]
N/A

Are there situations or clinical findings in the patient where you deviate from your prophylaxis standard (medication, dose, duration, age of the patient)? (multiple answers possible, please specify in answer box) [in case of already known vesicoureteral reflux (VUR)]
N/A

Are there situations or clinical findings in the patient where you deviate from your prophylaxis standard (medication, dose, duration, age of the patient)? (multiple answers possible, please specify in answer box) [in patients with recurrent urinary tract infections (UTI)]
N/A

Are there situations or clinical findings in the patient where you deviate from your prophylaxis standard (medication, dose, duration, age of the patient)? (multiple answers possible, please specify in answer box) [if multidrug-resistant organisms (MDRO) are detected in urine cultures]
N/A

Are there situations or clinical findings in the patient where you deviate from your prophylaxis standard (medication, dose, duration, age of the patient)? (multiple answers possible, please specify in answer box) [in newborns]
N/A

Are there situations in which you use intravenous prophylaxis?
N/A

Voiding analysis

Are there differences in antibiotic prophylaxis between VUS and VCUG in your department?
N/A

Is a non-pathological urine strip test required in preparation for VUS/VCUG in your department?
N/A

Is a non-pathological urine culture required in preparation for VUS/VCUG in your department?
N/A

Do patients only receive post-procedure prophylaxis if vesicoureteral reflux (VUR) is detected in VUS/VCUG?
N/A

Do other MUS/MCU findings (e.g. urethral valves) have an influence on post-procedure prophylaxis?
N/A

End of survey

May we contact you personally if we have further questions?
N/A

Survey response 170

Response ID
172

Date submitted
2025-03-25 10:59:28

Last page
5

Start language
en

Seed
975383214

Date started
2025-03-25 10:54:57

Date last action
2025-03-25 10:59:28

Standard Operation Procedure

Who fills out this survey?
Pediatric Radiologist

Who fills out this survey? [Other]

In which type of hospital are you employed?
University Hospital

In which type of hospital are you employed? [Other]

In which country do you work?
Germany

Does your department have a defined protocol for infection prophylaxis in the context of VUS/VCUG?
No

Has this protocol been transformed into a written standard operating procedure (SOP)?
N/A

Antibiotic Prophylaxis

Are there situations/constellations in which you do not use antibiotic prophylaxis for VUS/VCUG?
Yes

Please specify these situations/constellations: [girl with first uncomplicated UTI without urinary tract dilation in ultrasound]
No

Please specify these situations/constellations: [boy with first uncomplicated UTI without urinary tract dilation in ultrasound]
Yes

Please specify these situations/constellations: [in patients with low grade urinary tract dilation (up to 2nd grade) and no UTI in medical history]
No

Please specify these situations/constellations: [Other]

If you use antibiotic prophylaxis: which mediaction do you use? (multiple answers and subdivision into age groups possible) [Trimethoprim]
No

If you use antibiotic prophylaxis: which mediaction do you use? (multiple answers and subdivision into age groups possible) [Nitrofurantoin]
Yes

If you use antibiotic prophylaxis: which mediaction do you use? (multiple answers and subdivision into age groups possible) [Amoxicillin]
No

If you use antibiotic prophylaxis: which mediaction do you use? (multiple answers and subdivision into age groups possible) [Cefaclor]
No

If you use antibiotic prophylaxis: which mediaction do you use? (multiple answers and subdivision into age groups possible) [Amoxicillin/clavulanic acid]
No

If you use antibiotic prophylaxis: which mediaction do you use? (multiple answers and subdivision into age groups possible) [Other]
No

Trimethoprim: which time schedule do you use? [Day(s) before VUS/VCUG, how many?]
N/A

Trimethoprim: which time schedule do you use? [Day of VUS/VCUG]
N/A

Trimethoprim: which time schedule do you use? [Day(s) after VUS/VCUG, how many?]
N/A

Nitrofurantoin: in what dosage?
Other

Nitrofurantoin: in what dosage? [Other]
AP is instructed by the reffering colleague

Nitrofurantoin: for which age group do you use nitrofurantoin?
see above

Nitrofurantoin: which time schedule do you use? [Day(s) before VUS/VCUG, how many?]
No

Nitrofurantoin: which time schedule do you use? [Day(s) before VUS/VCUG, how many?][Comment]

Nitrofurantoin: which time schedule do you use? [Day of VUS/VCUG]
Yes

Nitrofurantoin: which time schedule do you use? [Day of VUS/VCUG][Comment]

Nitrofurantoin: which time schedule do you use? [Day(s) after VUS/VCUG, how many?]
Yes

Nitrofurantoin: which time schedule do you use? [Day(s) after VUS/VCUG, how many?][Comment]
2

Amoxicillin: which time schedule do you use? [Day(s) before VUS/VCUG, how many?]
N/A

Amoxicillin: which time schedule do you use? [Day of VUS/VCUG]
N/A

Amoxicillin: which time schedule do you use? [Day(s) after VUS/VCUG, how many?]
N/A

Cefaclor: which time schedule do you use? [Days(s) before VUS/VCUG, how many?]
N/A

Cefaclor: which time schedule do you use? [Day of VUS/VCUG]
N/A

Cefaclor: which time schedule do you use? [Day(s) after VUS/VCUG, how many?]
N/A

Amoxicillin/clavulanic acid: in what dosage? (Amoxicillin portion) [prophylaktic (10-20 mg/Kg bodyweight/d)]
N/A

Amoxicillin/clavulanic acid: in what dosage? (Amoxicillin portion) [double prophylactic (20-40 mg/Kg bodyweight/d)]
N/A

Amoxicillin/clavulanic acid: in what dosage? (Amoxicillin portion) [therapeutic (80-100 mg/Kg bodyweight/d)]
N/A

Amoxicillin/clavulanic acid: which time schedule do you use? [Day(s) before VUS/VCUG]
N/A

Amoxicillin/clavulanic acid: which time schedule do you use? [Day of VUS/VCUG]
N/A

Amoxicillin/clavulanic acid: which time schedule do you use? [Day(s) after VUS/VCUG]
N/A

Other: which time schedule do you use? [Day(s) before VUS/VCUG, how many?]
N/A

Other: which time schedule do you use? [Day of VUS/VCUG]
N/A

Other: which time schedule do you use? [Day(s) after VUS/VCUG, how many?]
N/A

Indication for prophylaxis

Which patients receive periinterventional antibiotic prophylaxis? (multiple answers possible) [all]
No

Which patients receive periinterventional antibiotic prophylaxis? (multiple answers possible) [patients post urinary tract infection/pyelonephritis/urosepsis]
Yes

Which patients receive periinterventional antibiotic prophylaxis? (multiple answers possible) [patients with urinary tract dilation in ultrasound]
No

Which patients receive periinterventional antibiotic prophylaxis? (multiple answers possible) [patients with prenatal urinary tract dilation]
No

Which patients receive periinterventional antibiotic prophylaxis? (multiple answers possible) [patients post VUR surgery]
No

Which patients receive periinterventional antibiotic prophylaxis? (multiple answers possible) [none]
No

Which patients receive periinterventional antibiotic prophylaxis? (multiple answers possible) [Other]

patients status post urinary tract infection/pyelonephritis/urosepsis: what is the minimum time between infection and VUS/VCUG? (in days)
10

Are there situations or clinical findings in the patient where you deviate from your prophylaxis standard (medication, dose, duration, age of the patient)? (multiple answers possible, please specify in answer box) [in case of already known vesicoureteral reflux (VUR)]
No

Are there situations or clinical findings in the patient where you deviate from your prophylaxis standard (medication, dose, duration, age of the patient)? (multiple answers possible, please specify in answer box) [in case of already known vesicoureteral reflux (VUR)][Comment]

Are there situations or clinical findings in the patient where you deviate from your prophylaxis standard (medication, dose, duration, age of the patient)? (multiple answers possible, please specify in answer box) [in patients with recurrent urinary tract infections (UTI)]
Yes

Are there situations or clinical findings in the patient where you deviate from your prophylaxis standard (medication, dose, duration, age of the patient)? (multiple answers possible, please specify in answer box) [in patients with recurrent urinary tract infections (UTI)][Comment]

Are there situations or clinical findings in the patient where you deviate from your prophylaxis standard (medication, dose, duration, age of the patient)? (multiple answers possible, please specify in answer box) [if multidrug-resistant organisms (MDRO) are detected in urine cultures]
Yes

Are there situations or clinical findings in the patient where you deviate from your prophylaxis standard (medication, dose, duration, age of the patient)? (multiple answers possible, please specify in answer box) [if multidrug-resistant organisms (MDRO) are detected in urine cultures][Comment]

Are there situations or clinical findings in the patient where you deviate from your prophylaxis standard (medication, dose, duration, age of the patient)? (multiple answers possible, please specify in answer box) [in newborns]
No

Are there situations or clinical findings in the patient where you deviate from your prophylaxis standard (medication, dose, duration, age of the patient)? (multiple answers possible, please specify in answer box) [in newborns][Comment]

Are there situations or clinical findings in the patient where you deviate from your prophylaxis standard (medication, dose, duration, age of the patient)? (multiple answers possible, please specify in answer box) [Other]

Are there situations or clinical findings in the patient where you deviate from your prophylaxis standard (medication, dose, duration, age of the patient)? (multiple answers possible, please specify in answer box) [Other comment]

Are there situations in which you use intravenous prophylaxis?
No

Voiding analysis

Are there differences in antibiotic prophylaxis between VUS and VCUG in your department?
No

Is a non-pathological urine strip test required in preparation for VUS/VCUG in your department?
Yes

What is the maximum amount of days accepted for urine strip test to be performed prior to the exam?
same day

Is a non-pathological urine culture required in preparation for VUS/VCUG in your department?
No

Do patients only receive post-procedure prophylaxis if vesicoureteral reflux (VUR) is detected in VUS/VCUG?
No

Do other MUS/MCU findings (e.g. urethral valves) have an influence on post-procedure prophylaxis?
No

End of survey

Thank you for taking the time to answer this survey!

May we contact you personally if we have further questions?
No

Survey response 171

Response ID
173

Last page
3

Start language
en

Seed
944519618

Date started
2025-03-25 10:58:58

Date last action
2025-03-25 11:00:33

Standard Operation Procedure

Who fills out this survey?
Pediatric Radiologist

Who fills out this survey? [Other]

In which type of hospital are you employed?
University Hospital

In which type of hospital are you employed? [Other]

In which country do you work?
Canada

Does your department have a defined protocol for infection prophylaxis in the context of VUS/VCUG?
No

Has this protocol been transformed into a written standard operating procedure (SOP)?
N/A

Antibiotic Prophylaxis

Are there situations/constellations in which you do not use antibiotic prophylaxis for VUS/VCUG?
Yes

Please specify these situations/constellations: [girl with first uncomplicated UTI without urinary tract dilation in ultrasound]
No

Please specify these situations/constellations: [boy with first uncomplicated UTI without urinary tract dilation in ultrasound]
No

Please specify these situations/constellations: [in patients with low grade urinary tract dilation (up to 2nd grade) and no UTI in medical history]
No

Please specify these situations/constellations: [Other]
We do not provide antibiotic prophylaxis

If you use antibiotic prophylaxis: which mediaction do you use? (multiple answers and subdivision into age groups possible) [Trimethoprim]
No

If you use antibiotic prophylaxis: which mediaction do you use? (multiple answers and subdivision into age groups possible) [Nitrofurantoin]
No

If you use antibiotic prophylaxis: which mediaction do you use? (multiple answers and subdivision into age groups possible) [Amoxicillin]
No

If you use antibiotic prophylaxis: which mediaction do you use? (multiple answers and subdivision into age groups possible) [Cefaclor]
No

If you use antibiotic prophylaxis: which mediaction do you use? (multiple answers and subdivision into age groups possible) [Amoxicillin/clavulanic acid]
No

If you use antibiotic prophylaxis: which mediaction do you use? (multiple answers and subdivision into age groups possible) [Other]
Yes

Trimethoprim: which time schedule do you use? [Day(s) before VUS/VCUG, how many?]
N/A

Trimethoprim: which time schedule do you use? [Day of VUS/VCUG]
N/A

Trimethoprim: which time schedule do you use? [Day(s) after VUS/VCUG, how many?]
N/A

Nitrofurantoin: which time schedule do you use? [Day(s) before VUS/VCUG, how many?]
N/A

Nitrofurantoin: which time schedule do you use? [Day of VUS/VCUG]
N/A

Nitrofurantoin: which time schedule do you use? [Day(s) after VUS/VCUG, how many?]
N/A

Amoxicillin: which time schedule do you use? [Day(s) before VUS/VCUG, how many?]
N/A

Amoxicillin: which time schedule do you use? [Day of VUS/VCUG]
N/A

Amoxicillin: which time schedule do you use? [Day(s) after VUS/VCUG, how many?]
N/A

Cefaclor: which time schedule do you use? [Days(s) before VUS/VCUG, how many?]
N/A

Cefaclor: which time schedule do you use? [Day of VUS/VCUG]
N/A

Cefaclor: which time schedule do you use? [Day(s) after VUS/VCUG, how many?]
N/A

Amoxicillin/clavulanic acid: in what dosage? (Amoxicillin portion) [prophylaktic (10-20 mg/Kg bodyweight/d)]
N/A

Amoxicillin/clavulanic acid: in what dosage? (Amoxicillin portion) [double prophylactic (20-40 mg/Kg bodyweight/d)]
N/A

Amoxicillin/clavulanic acid: in what dosage? (Amoxicillin portion) [therapeutic (80-100 mg/Kg bodyweight/d)]
N/A

Amoxicillin/clavulanic acid: which time schedule do you use? [Day(s) before VUS/VCUG]
N/A

Amoxicillin/clavulanic acid: which time schedule do you use? [Day of VUS/VCUG]
N/A

Amoxicillin/clavulanic acid: which time schedule do you use? [Day(s) after VUS/VCUG]
N/A

Other: which medication and in what dosage?
n/a

Other: for which age group do you use this medication?
n/a

Other: which time schedule do you use? [Day(s) before VUS/VCUG, how many?]
Yes

Other: which time schedule do you use? [Day(s) before VUS/VCUG, how many?][Comment]
n/a

Other: which time schedule do you use? [Day of VUS/VCUG]
No

Other: which time schedule do you use? [Day of VUS/VCUG][Comment]

Other: which time schedule do you use? [Day(s) after VUS/VCUG, how many?]
Yes

Other: which time schedule do you use? [Day(s) after VUS/VCUG, how many?][Comment]
n/a

Indication for prophylaxis

Which patients receive periinterventional antibiotic prophylaxis? (multiple answers possible) [all]
No

Which patients receive periinterventional antibiotic prophylaxis? (multiple answers possible) [patients post urinary tract infection/pyelonephritis/urosepsis]
No

Which patients receive periinterventional antibiotic prophylaxis? (multiple answers possible) [patients with urinary tract dilation in ultrasound]
No

Which patients receive periinterventional antibiotic prophylaxis? (multiple answers possible) [patients with prenatal urinary tract dilation]
No

Which patients receive periinterventional antibiotic prophylaxis? (multiple answers possible) [patients post VUR surgery]
No

Which patients receive periinterventional antibiotic prophylaxis? (multiple answers possible) [none]
Yes

Which patients receive periinterventional antibiotic prophylaxis? (multiple answers possible) [Other]

Are there situations or clinical findings in the patient where you deviate from your prophylaxis standard (medication, dose, duration, age of the patient)? (multiple answers possible, please specify in answer box) [in case of already known vesicoureteral reflux (VUR)]
No

Are there situations or clinical findings in the patient where you deviate from your prophylaxis standard (medication, dose, duration, age of the patient)? (multiple answers possible, please specify in answer box) [in case of already known vesicoureteral reflux (VUR)][Comment]

Are there situations or clinical findings in the patient where you deviate from your prophylaxis standard (medication, dose, duration, age of the patient)? (multiple answers possible, please specify in answer box) [in patients with recurrent urinary tract infections (UTI)]
No

Are there situations or clinical findings in the patient where you deviate from your prophylaxis standard (medication, dose, duration, age of the patient)? (multiple answers possible, please specify in answer box) [in patients with recurrent urinary tract infections (UTI)][Comment]

Are there situations or clinical findings in the patient where you deviate from your prophylaxis standard (medication, dose, duration, age of the patient)? (multiple answers possible, please specify in answer box) [if multidrug-resistant organisms (MDRO) are detected in urine cultures]
No

Are there situations or clinical findings in the patient where you deviate from your prophylaxis standard (medication, dose, duration, age of the patient)? (multiple answers possible, please specify in answer box) [if multidrug-resistant organisms (MDRO) are detected in urine cultures][Comment]

Are there situations or clinical findings in the patient where you deviate from your prophylaxis standard (medication, dose, duration, age of the patient)? (multiple answers possible, please specify in answer box) [in newborns]
No

Are there situations or clinical findings in the patient where you deviate from your prophylaxis standard (medication, dose, duration, age of the patient)? (multiple answers possible, please specify in answer box) [in newborns][Comment]

Are there situations or clinical findings in the patient where you deviate from your prophylaxis standard (medication, dose, duration, age of the patient)? (multiple answers possible, please specify in answer box) [Other]
n/a

Are there situations or clinical findings in the patient where you deviate from your prophylaxis standard (medication, dose, duration, age of the patient)? (multiple answers possible, please specify in answer box) [Other comment]
n/a

Are there situations in which you use intravenous prophylaxis?
No

Voiding analysis

Are there differences in antibiotic prophylaxis between VUS and VCUG in your department?
N/A

Is a non-pathological urine strip test required in preparation for VUS/VCUG in your department?
N/A

Is a non-pathological urine culture required in preparation for VUS/VCUG in your department?
N/A

Do patients only receive post-procedure prophylaxis if vesicoureteral reflux (VUR) is detected in VUS/VCUG?
N/A

Do other MUS/MCU findings (e.g. urethral valves) have an influence on post-procedure prophylaxis?
N/A

End of survey

May we contact you personally if we have further questions?
N/A

Survey response 172

Response ID
174

Date submitted
2025-03-25 11:05:56

Last page
5

Start language
en

Seed
1238417277

Date started
2025-03-25 11:01:33

Date last action
2025-03-25 11:05:56

Standard Operation Procedure

Who fills out this survey?
Pediatric Radiologist

Who fills out this survey? [Other]

In which type of hospital are you employed?
University Hospital

In which type of hospital are you employed? [Other]

In which country do you work?
Österreich

Does your department have a defined protocol for infection prophylaxis in the context of VUS/VCUG?
Yes

Has this protocol been transformed into a written standard operating procedure (SOP)?
No

Antibiotic Prophylaxis

Are there situations/constellations in which you do not use antibiotic prophylaxis for VUS/VCUG?
No

Please specify these situations/constellations: [girl with first uncomplicated UTI without urinary tract dilation in ultrasound]
N/A

Please specify these situations/constellations: [boy with first uncomplicated UTI without urinary tract dilation in ultrasound]
N/A

Please specify these situations/constellations: [in patients with low grade urinary tract dilation (up to 2nd grade) and no UTI in medical history]
N/A

If you use antibiotic prophylaxis: which mediaction do you use? (multiple answers and subdivision into age groups possible) [Trimethoprim]
No

If you use antibiotic prophylaxis: which mediaction do you use? (multiple answers and subdivision into age groups possible) [Nitrofurantoin]
No

If you use antibiotic prophylaxis: which mediaction do you use? (multiple answers and subdivision into age groups possible) [Amoxicillin]
No

If you use antibiotic prophylaxis: which mediaction do you use? (multiple answers and subdivision into age groups possible) [Cefaclor]
No

If you use antibiotic prophylaxis: which mediaction do you use? (multiple answers and subdivision into age groups possible) [Amoxicillin/clavulanic acid]
No

If you use antibiotic prophylaxis: which mediaction do you use? (multiple answers and subdivision into age groups possible) [Other]
Yes

Trimethoprim: which time schedule do you use? [Day(s) before VUS/VCUG, how many?]
N/A

Trimethoprim: which time schedule do you use? [Day of VUS/VCUG]
N/A

Trimethoprim: which time schedule do you use? [Day(s) after VUS/VCUG, how many?]
N/A

Nitrofurantoin: which time schedule do you use? [Day(s) before VUS/VCUG, how many?]
N/A

Nitrofurantoin: which time schedule do you use? [Day of VUS/VCUG]
N/A

Nitrofurantoin: which time schedule do you use? [Day(s) after VUS/VCUG, how many?]
N/A

Amoxicillin: which time schedule do you use? [Day(s) before VUS/VCUG, how many?]
N/A

Amoxicillin: which time schedule do you use? [Day of VUS/VCUG]
N/A

Amoxicillin: which time schedule do you use? [Day(s) after VUS/VCUG, how many?]
N/A

Cefaclor: which time schedule do you use? [Days(s) before VUS/VCUG, how many?]
N/A

Cefaclor: which time schedule do you use? [Day of VUS/VCUG]
N/A

Cefaclor: which time schedule do you use? [Day(s) after VUS/VCUG, how many?]
N/A

Amoxicillin/clavulanic acid: in what dosage? (Amoxicillin portion) [prophylaktic (10-20 mg/Kg bodyweight/d)]
N/A

Amoxicillin/clavulanic acid: in what dosage? (Amoxicillin portion) [double prophylactic (20-40 mg/Kg bodyweight/d)]
N/A

Amoxicillin/clavulanic acid: in what dosage? (Amoxicillin portion) [therapeutic (80-100 mg/Kg bodyweight/d)]
N/A

Amoxicillin/clavulanic acid: which time schedule do you use? [Day(s) before VUS/VCUG]
N/A

Amoxicillin/clavulanic acid: which time schedule do you use? [Day of VUS/VCUG]
N/A

Amoxicillin/clavulanic acid: which time schedule do you use? [Day(s) after VUS/VCUG]
N/A

Other: which medication and in what dosage?
AB / antiseptic mixed to contrast infusion

Other: for which age group do you use this medication?
all

Other: which time schedule do you use? [Day(s) before VUS/VCUG, how many?]
No

Other: which time schedule do you use? [Day(s) before VUS/VCUG, how many?][Comment]

Other: which time schedule do you use? [Day of VUS/VCUG]
Yes

Other: which time schedule do you use? [Day of VUS/VCUG][Comment]

Other: which time schedule do you use? [Day(s) after VUS/VCUG, how many?]
Yes

Other: which time schedule do you use? [Day(s) after VUS/VCUG, how many?][Comment]
only in risk population for 3 days

Indication for prophylaxis

Which patients receive periinterventional antibiotic prophylaxis? (multiple answers possible) [all]
Yes

Which patients receive periinterventional antibiotic prophylaxis? (multiple answers possible) [patients post urinary tract infection/pyelonephritis/urosepsis]
N/A

Which patients receive periinterventional antibiotic prophylaxis? (multiple answers possible) [patients with urinary tract dilation in ultrasound]
N/A

Which patients receive periinterventional antibiotic prophylaxis? (multiple answers possible) [patients with prenatal urinary tract dilation]
N/A

Which patients receive periinterventional antibiotic prophylaxis? (multiple answers possible) [patients post VUR surgery]
N/A

Which patients receive periinterventional antibiotic prophylaxis? (multiple answers possible) [none]
N/A

Are there situations or clinical findings in the patient where you deviate from your prophylaxis standard (medication, dose, duration, age of the patient)? (multiple answers possible, please specify in answer box) [in case of already known vesicoureteral reflux (VUR)]
No

Are there situations or clinical findings in the patient where you deviate from your prophylaxis standard (medication, dose, duration, age of the patient)? (multiple answers possible, please specify in answer box) [in case of already known vesicoureteral reflux (VUR)][Comment]

Are there situations or clinical findings in the patient where you deviate from your prophylaxis standard (medication, dose, duration, age of the patient)? (multiple answers possible, please specify in answer box) [in patients with recurrent urinary tract infections (UTI)]
Yes

Are there situations or clinical findings in the patient where you deviate from your prophylaxis standard (medication, dose, duration, age of the patient)? (multiple answers possible, please specify in answer box) [in patients with recurrent urinary tract infections (UTI)][Comment]

Are there situations or clinical findings in the patient where you deviate from your prophylaxis standard (medication, dose, duration, age of the patient)? (multiple answers possible, please specify in answer box) [if multidrug-resistant organisms (MDRO) are detected in urine cultures]
Yes

Are there situations or clinical findings in the patient where you deviate from your prophylaxis standard (medication, dose, duration, age of the patient)? (multiple answers possible, please specify in answer box) [if multidrug-resistant organisms (MDRO) are detected in urine cultures][Comment]

Are there situations or clinical findings in the patient where you deviate from your prophylaxis standard (medication, dose, duration, age of the patient)? (multiple answers possible, please specify in answer box) [in newborns]
No

Are there situations or clinical findings in the patient where you deviate from your prophylaxis standard (medication, dose, duration, age of the patient)? (multiple answers possible, please specify in answer box) [in newborns][Comment]

Are there situations or clinical findings in the patient where you deviate from your prophylaxis standard (medication, dose, duration, age of the patient)? (multiple answers possible, please specify in answer box) [Other]

Are there situations or clinical findings in the patient where you deviate from your prophylaxis standard (medication, dose, duration, age of the patient)? (multiple answers possible, please specify in answer box) [Other comment]

Are there situations in which you use intravenous prophylaxis?
No

Voiding analysis

Are there differences in antibiotic prophylaxis between VUS and VCUG in your department?
No

Is a non-pathological urine strip test required in preparation for VUS/VCUG in your department?
Yes

What is the maximum amount of days accepted for urine strip test to be performed prior to the exam?
3-7 days

Is a non-pathological urine culture required in preparation for VUS/VCUG in your department?
No

Do patients only receive post-procedure prophylaxis if vesicoureteral reflux (VUR) is detected in VUS/VCUG?
No

Do other MUS/MCU findings (e.g. urethral valves) have an influence on post-procedure prophylaxis?
Yes

Please specify:
regular urine checks, possibly prohylactic AB

End of survey

Thank you for taking the time to answer this survey!

May we contact you personally if we have further questions?
No

Survey response 173

Response ID
175

Last page
0

Start language
en

Seed
829280728

Date started
2025-03-25 11:23:25

Date last action
2025-03-25 11:23:25

Standard Operation Procedure

Does your department have a defined protocol for infection prophylaxis in the context of VUS/VCUG?
N/A

Has this protocol been transformed into a written standard operating procedure (SOP)?
N/A

Antibiotic Prophylaxis

Are there situations/constellations in which you do not use antibiotic prophylaxis for VUS/VCUG?
N/A

Please specify these situations/constellations: [girl with first uncomplicated UTI without urinary tract dilation in ultrasound]
N/A

Please specify these situations/constellations: [boy with first uncomplicated UTI without urinary tract dilation in ultrasound]
N/A

Please specify these situations/constellations: [in patients with low grade urinary tract dilation (up to 2nd grade) and no UTI in medical history]
N/A

If you use antibiotic prophylaxis: which mediaction do you use? (multiple answers and subdivision into age groups possible) [Trimethoprim]
N/A

If you use antibiotic prophylaxis: which mediaction do you use? (multiple answers and subdivision into age groups possible) [Nitrofurantoin]
N/A

If you use antibiotic prophylaxis: which mediaction do you use? (multiple answers and subdivision into age groups possible) [Amoxicillin]
N/A

If you use antibiotic prophylaxis: which mediaction do you use? (multiple answers and subdivision into age groups possible) [Cefaclor]
N/A

If you use antibiotic prophylaxis: which mediaction do you use? (multiple answers and subdivision into age groups possible) [Amoxicillin/clavulanic acid]
N/A

If you use antibiotic prophylaxis: which mediaction do you use? (multiple answers and subdivision into age groups possible) [Other]
N/A

Trimethoprim: which time schedule do you use? [Day(s) before VUS/VCUG, how many?]
N/A

Trimethoprim: which time schedule do you use? [Day of VUS/VCUG]
N/A

Trimethoprim: which time schedule do you use? [Day(s) after VUS/VCUG, how many?]
N/A

Nitrofurantoin: which time schedule do you use? [Day(s) before VUS/VCUG, how many?]
N/A

Nitrofurantoin: which time schedule do you use? [Day of VUS/VCUG]
N/A

Nitrofurantoin: which time schedule do you use? [Day(s) after VUS/VCUG, how many?]
N/A

Amoxicillin: which time schedule do you use? [Day(s) before VUS/VCUG, how many?]
N/A

Amoxicillin: which time schedule do you use? [Day of VUS/VCUG]
N/A

Amoxicillin: which time schedule do you use? [Day(s) after VUS/VCUG, how many?]
N/A

Cefaclor: which time schedule do you use? [Days(s) before VUS/VCUG, how many?]
N/A

Cefaclor: which time schedule do you use? [Day of VUS/VCUG]
N/A

Cefaclor: which time schedule do you use? [Day(s) after VUS/VCUG, how many?]
N/A

Amoxicillin/clavulanic acid: in what dosage? (Amoxicillin portion) [prophylaktic (10-20 mg/Kg bodyweight/d)]
N/A

Amoxicillin/clavulanic acid: in what dosage? (Amoxicillin portion) [double prophylactic (20-40 mg/Kg bodyweight/d)]
N/A

Amoxicillin/clavulanic acid: in what dosage? (Amoxicillin portion) [therapeutic (80-100 mg/Kg bodyweight/d)]
N/A

Amoxicillin/clavulanic acid: which time schedule do you use? [Day(s) before VUS/VCUG]
N/A

Amoxicillin/clavulanic acid: which time schedule do you use? [Day of VUS/VCUG]
N/A

Amoxicillin/clavulanic acid: which time schedule do you use? [Day(s) after VUS/VCUG]
N/A

Other: which time schedule do you use? [Day(s) before VUS/VCUG, how many?]
N/A

Other: which time schedule do you use? [Day of VUS/VCUG]
N/A

Other: which time schedule do you use? [Day(s) after VUS/VCUG, how many?]
N/A

Indication for prophylaxis

Which patients receive periinterventional antibiotic prophylaxis? (multiple answers possible) [all]
N/A

Which patients receive periinterventional antibiotic prophylaxis? (multiple answers possible) [patients post urinary tract infection/pyelonephritis/urosepsis]
N/A

Which patients receive periinterventional antibiotic prophylaxis? (multiple answers possible) [patients with urinary tract dilation in ultrasound]
N/A

Which patients receive periinterventional antibiotic prophylaxis? (multiple answers possible) [patients with prenatal urinary tract dilation]
N/A

Which patients receive periinterventional antibiotic prophylaxis? (multiple answers possible) [patients post VUR surgery]
N/A

Which patients receive periinterventional antibiotic prophylaxis? (multiple answers possible) [none]
N/A

Are there situations or clinical findings in the patient where you deviate from your prophylaxis standard (medication, dose, duration, age of the patient)? (multiple answers possible, please specify in answer box) [in case of already known vesicoureteral reflux (VUR)]
N/A

Are there situations or clinical findings in the patient where you deviate from your prophylaxis standard (medication, dose, duration, age of the patient)? (multiple answers possible, please specify in answer box) [in patients with recurrent urinary tract infections (UTI)]
N/A

Are there situations or clinical findings in the patient where you deviate from your prophylaxis standard (medication, dose, duration, age of the patient)? (multiple answers possible, please specify in answer box) [if multidrug-resistant organisms (MDRO) are detected in urine cultures]
N/A

Are there situations or clinical findings in the patient where you deviate from your prophylaxis standard (medication, dose, duration, age of the patient)? (multiple answers possible, please specify in answer box) [in newborns]
N/A

Are there situations in which you use intravenous prophylaxis?
N/A

Voiding analysis

Are there differences in antibiotic prophylaxis between VUS and VCUG in your department?
N/A

Is a non-pathological urine strip test required in preparation for VUS/VCUG in your department?
N/A

Is a non-pathological urine culture required in preparation for VUS/VCUG in your department?
N/A

Do patients only receive post-procedure prophylaxis if vesicoureteral reflux (VUR) is detected in VUS/VCUG?
N/A

Do other MUS/MCU findings (e.g. urethral valves) have an influence on post-procedure prophylaxis?
N/A

End of survey

May we contact you personally if we have further questions?
N/A

Survey response 174

Response ID
176

Last page
1

Start language
en

Seed
204373064

Date started
2025-03-25 11:31:01

Date last action
2025-03-25 11:31:16

Standard Operation Procedure

Who fills out this survey?
Radiologist

Who fills out this survey? [Other]

In which type of hospital are you employed?
University Hospital

In which type of hospital are you employed? [Other]

In which country do you work?
Greece

Does your department have a defined protocol for infection prophylaxis in the context of VUS/VCUG?
No

Has this protocol been transformed into a written standard operating procedure (SOP)?
N/A

Antibiotic Prophylaxis

Are there situations/constellations in which you do not use antibiotic prophylaxis for VUS/VCUG?
N/A

Please specify these situations/constellations: [girl with first uncomplicated UTI without urinary tract dilation in ultrasound]
N/A

Please specify these situations/constellations: [boy with first uncomplicated UTI without urinary tract dilation in ultrasound]
N/A

Please specify these situations/constellations: [in patients with low grade urinary tract dilation (up to 2nd grade) and no UTI in medical history]
N/A

If you use antibiotic prophylaxis: which mediaction do you use? (multiple answers and subdivision into age groups possible) [Trimethoprim]
N/A

If you use antibiotic prophylaxis: which mediaction do you use? (multiple answers and subdivision into age groups possible) [Nitrofurantoin]
N/A

If you use antibiotic prophylaxis: which mediaction do you use? (multiple answers and subdivision into age groups possible) [Amoxicillin]
N/A

If you use antibiotic prophylaxis: which mediaction do you use? (multiple answers and subdivision into age groups possible) [Cefaclor]
N/A

If you use antibiotic prophylaxis: which mediaction do you use? (multiple answers and subdivision into age groups possible) [Amoxicillin/clavulanic acid]
N/A

If you use antibiotic prophylaxis: which mediaction do you use? (multiple answers and subdivision into age groups possible) [Other]
N/A

Trimethoprim: which time schedule do you use? [Day(s) before VUS/VCUG, how many?]
N/A

Trimethoprim: which time schedule do you use? [Day of VUS/VCUG]
N/A

Trimethoprim: which time schedule do you use? [Day(s) after VUS/VCUG, how many?]
N/A

Nitrofurantoin: which time schedule do you use? [Day(s) before VUS/VCUG, how many?]
N/A

Nitrofurantoin: which time schedule do you use? [Day of VUS/VCUG]
N/A

Nitrofurantoin: which time schedule do you use? [Day(s) after VUS/VCUG, how many?]
N/A

Amoxicillin: which time schedule do you use? [Day(s) before VUS/VCUG, how many?]
N/A

Amoxicillin: which time schedule do you use? [Day of VUS/VCUG]
N/A

Amoxicillin: which time schedule do you use? [Day(s) after VUS/VCUG, how many?]
N/A

Cefaclor: which time schedule do you use? [Days(s) before VUS/VCUG, how many?]
N/A

Cefaclor: which time schedule do you use? [Day of VUS/VCUG]
N/A

Cefaclor: which time schedule do you use? [Day(s) after VUS/VCUG, how many?]
N/A

Amoxicillin/clavulanic acid: in what dosage? (Amoxicillin portion) [prophylaktic (10-20 mg/Kg bodyweight/d)]
N/A

Amoxicillin/clavulanic acid: in what dosage? (Amoxicillin portion) [double prophylactic (20-40 mg/Kg bodyweight/d)]
N/A

Amoxicillin/clavulanic acid: in what dosage? (Amoxicillin portion) [therapeutic (80-100 mg/Kg bodyweight/d)]
N/A

Amoxicillin/clavulanic acid: which time schedule do you use? [Day(s) before VUS/VCUG]
N/A

Amoxicillin/clavulanic acid: which time schedule do you use? [Day of VUS/VCUG]
N/A

Amoxicillin/clavulanic acid: which time schedule do you use? [Day(s) after VUS/VCUG]
N/A

Other: which time schedule do you use? [Day(s) before VUS/VCUG, how many?]
N/A

Other: which time schedule do you use? [Day of VUS/VCUG]
N/A

Other: which time schedule do you use? [Day(s) after VUS/VCUG, how many?]
N/A

Indication for prophylaxis

Which patients receive periinterventional antibiotic prophylaxis? (multiple answers possible) [all]
N/A

Which patients receive periinterventional antibiotic prophylaxis? (multiple answers possible) [patients post urinary tract infection/pyelonephritis/urosepsis]
N/A

Which patients receive periinterventional antibiotic prophylaxis? (multiple answers possible) [patients with urinary tract dilation in ultrasound]
N/A

Which patients receive periinterventional antibiotic prophylaxis? (multiple answers possible) [patients with prenatal urinary tract dilation]
N/A

Which patients receive periinterventional antibiotic prophylaxis? (multiple answers possible) [patients post VUR surgery]
N/A

Which patients receive periinterventional antibiotic prophylaxis? (multiple answers possible) [none]
N/A

Are there situations or clinical findings in the patient where you deviate from your prophylaxis standard (medication, dose, duration, age of the patient)? (multiple answers possible, please specify in answer box) [in case of already known vesicoureteral reflux (VUR)]
N/A

Are there situations or clinical findings in the patient where you deviate from your prophylaxis standard (medication, dose, duration, age of the patient)? (multiple answers possible, please specify in answer box) [in patients with recurrent urinary tract infections (UTI)]
N/A

Are there situations or clinical findings in the patient where you deviate from your prophylaxis standard (medication, dose, duration, age of the patient)? (multiple answers possible, please specify in answer box) [if multidrug-resistant organisms (MDRO) are detected in urine cultures]
N/A

Are there situations or clinical findings in the patient where you deviate from your prophylaxis standard (medication, dose, duration, age of the patient)? (multiple answers possible, please specify in answer box) [in newborns]
N/A

Are there situations in which you use intravenous prophylaxis?
N/A

Voiding analysis

Are there differences in antibiotic prophylaxis between VUS and VCUG in your department?
N/A

Is a non-pathological urine strip test required in preparation for VUS/VCUG in your department?
N/A

Is a non-pathological urine culture required in preparation for VUS/VCUG in your department?
N/A

Do patients only receive post-procedure prophylaxis if vesicoureteral reflux (VUR) is detected in VUS/VCUG?
N/A

Do other MUS/MCU findings (e.g. urethral valves) have an influence on post-procedure prophylaxis?
N/A

End of survey

May we contact you personally if we have further questions?
N/A

Survey response 175

Response ID
177

Last page
1

Start language
en

Seed
2030541323

Date started
2025-03-25 11:34:55

Date last action
2025-03-25 11:35:29

Standard Operation Procedure

Who fills out this survey?
Pediatric Radiologist

Who fills out this survey? [Other]

In which type of hospital are you employed?
University Hospital

In which type of hospital are you employed? [Other]

In which country do you work?
Switzerland

Does your department have a defined protocol for infection prophylaxis in the context of VUS/VCUG?
Yes

Has this protocol been transformed into a written standard operating procedure (SOP)?
Yes

Which department is the author of the SOP for infection prophylaxis in VUS/VCUG?
Pediatric radiology

Which department is the author of the SOP for infection prophylaxis in VUS/VCUG? [Other]

Antibiotic Prophylaxis

Are there situations/constellations in which you do not use antibiotic prophylaxis for VUS/VCUG?
N/A

Please specify these situations/constellations: [girl with first uncomplicated UTI without urinary tract dilation in ultrasound]
N/A

Please specify these situations/constellations: [boy with first uncomplicated UTI without urinary tract dilation in ultrasound]
N/A

Please specify these situations/constellations: [in patients with low grade urinary tract dilation (up to 2nd grade) and no UTI in medical history]
N/A

If you use antibiotic prophylaxis: which mediaction do you use? (multiple answers and subdivision into age groups possible) [Trimethoprim]
N/A

If you use antibiotic prophylaxis: which mediaction do you use? (multiple answers and subdivision into age groups possible) [Nitrofurantoin]
N/A

If you use antibiotic prophylaxis: which mediaction do you use? (multiple answers and subdivision into age groups possible) [Amoxicillin]
N/A

If you use antibiotic prophylaxis: which mediaction do you use? (multiple answers and subdivision into age groups possible) [Cefaclor]
N/A

If you use antibiotic prophylaxis: which mediaction do you use? (multiple answers and subdivision into age groups possible) [Amoxicillin/clavulanic acid]
N/A

If you use antibiotic prophylaxis: which mediaction do you use? (multiple answers and subdivision into age groups possible) [Other]
N/A

Trimethoprim: which time schedule do you use? [Day(s) before VUS/VCUG, how many?]
N/A

Trimethoprim: which time schedule do you use? [Day of VUS/VCUG]
N/A

Trimethoprim: which time schedule do you use? [Day(s) after VUS/VCUG, how many?]
N/A

Nitrofurantoin: which time schedule do you use? [Day(s) before VUS/VCUG, how many?]
N/A

Nitrofurantoin: which time schedule do you use? [Day of VUS/VCUG]
N/A

Nitrofurantoin: which time schedule do you use? [Day(s) after VUS/VCUG, how many?]
N/A

Amoxicillin: which time schedule do you use? [Day(s) before VUS/VCUG, how many?]
N/A

Amoxicillin: which time schedule do you use? [Day of VUS/VCUG]
N/A

Amoxicillin: which time schedule do you use? [Day(s) after VUS/VCUG, how many?]
N/A

Cefaclor: which time schedule do you use? [Days(s) before VUS/VCUG, how many?]
N/A

Cefaclor: which time schedule do you use? [Day of VUS/VCUG]
N/A

Cefaclor: which time schedule do you use? [Day(s) after VUS/VCUG, how many?]
N/A

Amoxicillin/clavulanic acid: in what dosage? (Amoxicillin portion) [prophylaktic (10-20 mg/Kg bodyweight/d)]
N/A

Amoxicillin/clavulanic acid: in what dosage? (Amoxicillin portion) [double prophylactic (20-40 mg/Kg bodyweight/d)]
N/A

Amoxicillin/clavulanic acid: in what dosage? (Amoxicillin portion) [therapeutic (80-100 mg/Kg bodyweight/d)]
N/A

Amoxicillin/clavulanic acid: which time schedule do you use? [Day(s) before VUS/VCUG]
N/A

Amoxicillin/clavulanic acid: which time schedule do you use? [Day of VUS/VCUG]
N/A

Amoxicillin/clavulanic acid: which time schedule do you use? [Day(s) after VUS/VCUG]
N/A

Other: which time schedule do you use? [Day(s) before VUS/VCUG, how many?]
N/A

Other: which time schedule do you use? [Day of VUS/VCUG]
N/A

Other: which time schedule do you use? [Day(s) after VUS/VCUG, how many?]
N/A

Indication for prophylaxis

Which patients receive periinterventional antibiotic prophylaxis? (multiple answers possible) [all]
N/A

Which patients receive periinterventional antibiotic prophylaxis? (multiple answers possible) [patients post urinary tract infection/pyelonephritis/urosepsis]
N/A

Which patients receive periinterventional antibiotic prophylaxis? (multiple answers possible) [patients with urinary tract dilation in ultrasound]
N/A

Which patients receive periinterventional antibiotic prophylaxis? (multiple answers possible) [patients with prenatal urinary tract dilation]
N/A

Which patients receive periinterventional antibiotic prophylaxis? (multiple answers possible) [patients post VUR surgery]
N/A

Which patients receive periinterventional antibiotic prophylaxis? (multiple answers possible) [none]
N/A

Are there situations or clinical findings in the patient where you deviate from your prophylaxis standard (medication, dose, duration, age of the patient)? (multiple answers possible, please specify in answer box) [in case of already known vesicoureteral reflux (VUR)]
N/A

Are there situations or clinical findings in the patient where you deviate from your prophylaxis standard (medication, dose, duration, age of the patient)? (multiple answers possible, please specify in answer box) [in patients with recurrent urinary tract infections (UTI)]
N/A

Are there situations or clinical findings in the patient where you deviate from your prophylaxis standard (medication, dose, duration, age of the patient)? (multiple answers possible, please specify in answer box) [if multidrug-resistant organisms (MDRO) are detected in urine cultures]
N/A

Are there situations or clinical findings in the patient where you deviate from your prophylaxis standard (medication, dose, duration, age of the patient)? (multiple answers possible, please specify in answer box) [in newborns]
N/A

Are there situations in which you use intravenous prophylaxis?
N/A

Voiding analysis

Are there differences in antibiotic prophylaxis between VUS and VCUG in your department?
N/A

Is a non-pathological urine strip test required in preparation for VUS/VCUG in your department?
N/A

Is a non-pathological urine culture required in preparation for VUS/VCUG in your department?
N/A

Do patients only receive post-procedure prophylaxis if vesicoureteral reflux (VUR) is detected in VUS/VCUG?
N/A

Do other MUS/MCU findings (e.g. urethral valves) have an influence on post-procedure prophylaxis?
N/A

End of survey

May we contact you personally if we have further questions?
N/A

Survey response 176

Response ID
178

Date submitted
2025-03-25 11:55:35

Last page
5

Start language
en

Seed
893420680

Date started
2025-03-25 11:53:02

Date last action
2025-03-25 11:55:35

Standard Operation Procedure

Who fills out this survey?
Pediatric Radiologist

Who fills out this survey? [Other]

In which type of hospital are you employed?
Community hospital (

In which type of hospital are you employed? [Other]

In which country do you work?
United Kingdom

Does your department have a defined protocol for infection prophylaxis in the context of VUS/VCUG?
Yes

Has this protocol been transformed into a written standard operating procedure (SOP)?
Yes

Which department is the author of the SOP for infection prophylaxis in VUS/VCUG?
Pediatric radiology

Which department is the author of the SOP for infection prophylaxis in VUS/VCUG? [Other]

Antibiotic Prophylaxis

Are there situations/constellations in which you do not use antibiotic prophylaxis for VUS/VCUG?
No

Please specify these situations/constellations: [girl with first uncomplicated UTI without urinary tract dilation in ultrasound]
N/A

Please specify these situations/constellations: [boy with first uncomplicated UTI without urinary tract dilation in ultrasound]
N/A

Please specify these situations/constellations: [in patients with low grade urinary tract dilation (up to 2nd grade) and no UTI in medical history]
N/A

If you use antibiotic prophylaxis: which mediaction do you use? (multiple answers and subdivision into age groups possible) [Trimethoprim]
Yes

If you use antibiotic prophylaxis: which mediaction do you use? (multiple answers and subdivision into age groups possible) [Nitrofurantoin]
No

If you use antibiotic prophylaxis: which mediaction do you use? (multiple answers and subdivision into age groups possible) [Amoxicillin]
No

If you use antibiotic prophylaxis: which mediaction do you use? (multiple answers and subdivision into age groups possible) [Cefaclor]
No

If you use antibiotic prophylaxis: which mediaction do you use? (multiple answers and subdivision into age groups possible) [Amoxicillin/clavulanic acid]
No

If you use antibiotic prophylaxis: which mediaction do you use? (multiple answers and subdivision into age groups possible) [Other]
No

Trimethoprim: in what dosage?
double prophylakctic (4 mg/kg bodyweight/d)

Trimethoprim: in what dosage? [Other]

Trimethoprim: for which age group do you use trimethoprim?
Under 1yo.

Trimethoprim: which time schedule do you use? [Day(s) before VUS/VCUG, how many?]
Yes

Trimethoprim: which time schedule do you use? [Day(s) before VUS/VCUG, how many?][Comment]
1

Trimethoprim: which time schedule do you use? [Day of VUS/VCUG]
Yes

Trimethoprim: which time schedule do you use? [Day of VUS/VCUG][Comment]

Trimethoprim: which time schedule do you use? [Day(s) after VUS/VCUG, how many?]
Yes

Trimethoprim: which time schedule do you use? [Day(s) after VUS/VCUG, how many?][Comment]
1

Nitrofurantoin: which time schedule do you use? [Day(s) before VUS/VCUG, how many?]
N/A

Nitrofurantoin: which time schedule do you use? [Day of VUS/VCUG]
N/A

Nitrofurantoin: which time schedule do you use? [Day(s) after VUS/VCUG, how many?]
N/A

Amoxicillin: which time schedule do you use? [Day(s) before VUS/VCUG, how many?]
N/A

Amoxicillin: which time schedule do you use? [Day of VUS/VCUG]
N/A

Amoxicillin: which time schedule do you use? [Day(s) after VUS/VCUG, how many?]
N/A

Cefaclor: which time schedule do you use? [Days(s) before VUS/VCUG, how many?]
N/A

Cefaclor: which time schedule do you use? [Day of VUS/VCUG]
N/A

Cefaclor: which time schedule do you use? [Day(s) after VUS/VCUG, how many?]
N/A

Amoxicillin/clavulanic acid: in what dosage? (Amoxicillin portion) [prophylaktic (10-20 mg/Kg bodyweight/d)]
N/A

Amoxicillin/clavulanic acid: in what dosage? (Amoxicillin portion) [double prophylactic (20-40 mg/Kg bodyweight/d)]
N/A

Amoxicillin/clavulanic acid: in what dosage? (Amoxicillin portion) [therapeutic (80-100 mg/Kg bodyweight/d)]
N/A

Amoxicillin/clavulanic acid: which time schedule do you use? [Day(s) before VUS/VCUG]
N/A

Amoxicillin/clavulanic acid: which time schedule do you use? [Day of VUS/VCUG]
N/A

Amoxicillin/clavulanic acid: which time schedule do you use? [Day(s) after VUS/VCUG]
N/A

Other: which time schedule do you use? [Day(s) before VUS/VCUG, how many?]
N/A

Other: which time schedule do you use? [Day of VUS/VCUG]
N/A

Other: which time schedule do you use? [Day(s) after VUS/VCUG, how many?]
N/A

Indication for prophylaxis

Which patients receive periinterventional antibiotic prophylaxis? (multiple answers possible) [all]
Yes

Which patients receive periinterventional antibiotic prophylaxis? (multiple answers possible) [patients post urinary tract infection/pyelonephritis/urosepsis]
N/A

Which patients receive periinterventional antibiotic prophylaxis? (multiple answers possible) [patients with urinary tract dilation in ultrasound]
N/A

Which patients receive periinterventional antibiotic prophylaxis? (multiple answers possible) [patients with prenatal urinary tract dilation]
N/A

Which patients receive periinterventional antibiotic prophylaxis? (multiple answers possible) [patients post VUR surgery]
N/A

Which patients receive periinterventional antibiotic prophylaxis? (multiple answers possible) [none]
N/A

Are there situations or clinical findings in the patient where you deviate from your prophylaxis standard (medication, dose, duration, age of the patient)? (multiple answers possible, please specify in answer box) [in case of already known vesicoureteral reflux (VUR)]
No

Are there situations or clinical findings in the patient where you deviate from your prophylaxis standard (medication, dose, duration, age of the patient)? (multiple answers possible, please specify in answer box) [in case of already known vesicoureteral reflux (VUR)][Comment]

Are there situations or clinical findings in the patient where you deviate from your prophylaxis standard (medication, dose, duration, age of the patient)? (multiple answers possible, please specify in answer box) [in patients with recurrent urinary tract infections (UTI)]
No

Are there situations or clinical findings in the patient where you deviate from your prophylaxis standard (medication, dose, duration, age of the patient)? (multiple answers possible, please specify in answer box) [in patients with recurrent urinary tract infections (UTI)][Comment]

Are there situations or clinical findings in the patient where you deviate from your prophylaxis standard (medication, dose, duration, age of the patient)? (multiple answers possible, please specify in answer box) [if multidrug-resistant organisms (MDRO) are detected in urine cultures]
Yes

Are there situations or clinical findings in the patient where you deviate from your prophylaxis standard (medication, dose, duration, age of the patient)? (multiple answers possible, please specify in answer box) [if multidrug-resistant organisms (MDRO) are detected in urine cultures][Comment]

Are there situations or clinical findings in the patient where you deviate from your prophylaxis standard (medication, dose, duration, age of the patient)? (multiple answers possible, please specify in answer box) [in newborns]
No

Are there situations or clinical findings in the patient where you deviate from your prophylaxis standard (medication, dose, duration, age of the patient)? (multiple answers possible, please specify in answer box) [in newborns][Comment]

Are there situations or clinical findings in the patient where you deviate from your prophylaxis standard (medication, dose, duration, age of the patient)? (multiple answers possible, please specify in answer box) [Other]

Are there situations or clinical findings in the patient where you deviate from your prophylaxis standard (medication, dose, duration, age of the patient)? (multiple answers possible, please specify in answer box) [Other comment]

Are there situations in which you use intravenous prophylaxis?
No

Voiding analysis

Are there differences in antibiotic prophylaxis between VUS and VCUG in your department?
No

Is a non-pathological urine strip test required in preparation for VUS/VCUG in your department?
No

Is a non-pathological urine culture required in preparation for VUS/VCUG in your department?
No

Do patients only receive post-procedure prophylaxis if vesicoureteral reflux (VUR) is detected in VUS/VCUG?
No

Do other MUS/MCU findings (e.g. urethral valves) have an influence on post-procedure prophylaxis?
No

End of survey

Thank you for taking the time to answer this survey!

May we contact you personally if we have further questions?
Yes

E-mail address:
n.davies18@nhs.net

Survey response 177

Response ID
179

Date submitted
2025-03-25 12:32:23

Last page
5

Start language
de

Seed
200048149

Date started
2025-03-25 12:27:25

Date last action
2025-03-25 12:32:23

Standard Operation Procedure

Who fills out this survey?
Pediatric Radiologist

Who fills out this survey? [Other]

In which type of hospital are you employed?
Other

In which type of hospital are you employed? [Other]
Kinderklinik nicht universitär

In which country do you work?
Deutschland

Does your department have a defined protocol for infection prophylaxis in the context of VUS/VCUG?
No

Has this protocol been transformed into a written standard operating procedure (SOP)?
N/A

Antibiotic Prophylaxis

Are there situations/constellations in which you do not use antibiotic prophylaxis for VUS/VCUG?
Yes

Please specify these situations/constellations: [girl with first uncomplicated UTI without urinary tract dilation in ultrasound]
No

Please specify these situations/constellations: [boy with first uncomplicated UTI without urinary tract dilation in ultrasound]
No

Please specify these situations/constellations: [in patients with low grade urinary tract dilation (up to 2nd grade) and no UTI in medical history]
No

Please specify these situations/constellations: [Other]
Wenn ein HWI ausgeheiilt ist und zunächst keine Prophylaxe angesetzt wurde (Meinungen dazu unterschiedlich zwischen Pädiatern und Radiologen)

If you use antibiotic prophylaxis: which mediaction do you use? (multiple answers and subdivision into age groups possible) [Trimethoprim]
Yes

If you use antibiotic prophylaxis: which mediaction do you use? (multiple answers and subdivision into age groups possible) [Nitrofurantoin]
No

If you use antibiotic prophylaxis: which mediaction do you use? (multiple answers and subdivision into age groups possible) [Amoxicillin]
No

If you use antibiotic prophylaxis: which mediaction do you use? (multiple answers and subdivision into age groups possible) [Cefaclor]
Yes

If you use antibiotic prophylaxis: which mediaction do you use? (multiple answers and subdivision into age groups possible) [Amoxicillin/clavulanic acid]
No

If you use antibiotic prophylaxis: which mediaction do you use? (multiple answers and subdivision into age groups possible) [Other]
No

Trimethoprim: in what dosage?
prophylactic (2 mg/kg bodyweight/d)

Trimethoprim: in what dosage? [Other]

Trimethoprim: for which age group do you use trimethoprim?
nicht mehr kleine Säuglinge

Trimethoprim: which time schedule do you use? [Day(s) before VUS/VCUG, how many?]
Yes

Trimethoprim: which time schedule do you use? [Day(s) before VUS/VCUG, how many?][Comment]
1

Trimethoprim: which time schedule do you use? [Day of VUS/VCUG]
Yes

Trimethoprim: which time schedule do you use? [Day of VUS/VCUG][Comment]

Trimethoprim: which time schedule do you use? [Day(s) after VUS/VCUG, how many?]
Yes

Trimethoprim: which time schedule do you use? [Day(s) after VUS/VCUG, how many?][Comment]
2

Nitrofurantoin: which time schedule do you use? [Day(s) before VUS/VCUG, how many?]
N/A

Nitrofurantoin: which time schedule do you use? [Day of VUS/VCUG]
N/A

Nitrofurantoin: which time schedule do you use? [Day(s) after VUS/VCUG, how many?]
N/A

Amoxicillin: which time schedule do you use? [Day(s) before VUS/VCUG, how many?]
N/A

Amoxicillin: which time schedule do you use? [Day of VUS/VCUG]
N/A

Amoxicillin: which time schedule do you use? [Day(s) after VUS/VCUG, how many?]
N/A

Cefaclor: in what dosage?
prophylactic (10 mg/kg bodyweight/d)

Cefaclor: in what dosage? [Other]

Cefaclor: for which age group do you use cefaclor?
alle

Cefaclor: which time schedule do you use? [Days(s) before VUS/VCUG, how many?]
Yes

Cefaclor: which time schedule do you use? [Days(s) before VUS/VCUG, how many?][Comment]
1

Cefaclor: which time schedule do you use? [Day of VUS/VCUG]
Yes

Cefaclor: which time schedule do you use? [Day of VUS/VCUG][Comment]

Cefaclor: which time schedule do you use? [Day(s) after VUS/VCUG, how many?]
Yes

Cefaclor: which time schedule do you use? [Day(s) after VUS/VCUG, how many?][Comment]
2

Amoxicillin/clavulanic acid: in what dosage? (Amoxicillin portion) [prophylaktic (10-20 mg/Kg bodyweight/d)]
N/A

Amoxicillin/clavulanic acid: in what dosage? (Amoxicillin portion) [double prophylactic (20-40 mg/Kg bodyweight/d)]
N/A

Amoxicillin/clavulanic acid: in what dosage? (Amoxicillin portion) [therapeutic (80-100 mg/Kg bodyweight/d)]
N/A

Amoxicillin/clavulanic acid: which time schedule do you use? [Day(s) before VUS/VCUG]
N/A

Amoxicillin/clavulanic acid: which time schedule do you use? [Day of VUS/VCUG]
N/A

Amoxicillin/clavulanic acid: which time schedule do you use? [Day(s) after VUS/VCUG]
N/A

Other: which time schedule do you use? [Day(s) before VUS/VCUG, how many?]
N/A

Other: which time schedule do you use? [Day of VUS/VCUG]
N/A

Other: which time schedule do you use? [Day(s) after VUS/VCUG, how many?]
N/A

Indication for prophylaxis

Which patients receive periinterventional antibiotic prophylaxis? (multiple answers possible) [all]
No

Which patients receive periinterventional antibiotic prophylaxis? (multiple answers possible) [patients post urinary tract infection/pyelonephritis/urosepsis]
Yes

Which patients receive periinterventional antibiotic prophylaxis? (multiple answers possible) [patients with urinary tract dilation in ultrasound]
No

Which patients receive periinterventional antibiotic prophylaxis? (multiple answers possible) [patients with prenatal urinary tract dilation]
No

Which patients receive periinterventional antibiotic prophylaxis? (multiple answers possible) [patients post VUR surgery]
No

Which patients receive periinterventional antibiotic prophylaxis? (multiple answers possible) [none]
No

Which patients receive periinterventional antibiotic prophylaxis? (multiple answers possible) [Other]
die genannten stellen oft keine Indikation zum MCU/MUS dar

patients status post urinary tract infection/pyelonephritis/urosepsis: what is the minimum time between infection and VUS/VCUG? (in days)
10

Are there situations or clinical findings in the patient where you deviate from your prophylaxis standard (medication, dose, duration, age of the patient)? (multiple answers possible, please specify in answer box) [in case of already known vesicoureteral reflux (VUR)]
Yes

Are there situations or clinical findings in the patient where you deviate from your prophylaxis standard (medication, dose, duration, age of the patient)? (multiple answers possible, please specify in answer box) [in case of already known vesicoureteral reflux (VUR)][Comment]
wozu dann MCU/MUS

Are there situations or clinical findings in the patient where you deviate from your prophylaxis standard (medication, dose, duration, age of the patient)? (multiple answers possible, please specify in answer box) [in patients with recurrent urinary tract infections (UTI)]
No

Are there situations or clinical findings in the patient where you deviate from your prophylaxis standard (medication, dose, duration, age of the patient)? (multiple answers possible, please specify in answer box) [in patients with recurrent urinary tract infections (UTI)][Comment]

Are there situations or clinical findings in the patient where you deviate from your prophylaxis standard (medication, dose, duration, age of the patient)? (multiple answers possible, please specify in answer box) [if multidrug-resistant organisms (MDRO) are detected in urine cultures]
Yes

Are there situations or clinical findings in the patient where you deviate from your prophylaxis standard (medication, dose, duration, age of the patient)? (multiple answers possible, please specify in answer box) [if multidrug-resistant organisms (MDRO) are detected in urine cultures][Comment]
evtl. anderes Medikament

Are there situations or clinical findings in the patient where you deviate from your prophylaxis standard (medication, dose, duration, age of the patient)? (multiple answers possible, please specify in answer box) [in newborns]
No

Are there situations or clinical findings in the patient where you deviate from your prophylaxis standard (medication, dose, duration, age of the patient)? (multiple answers possible, please specify in answer box) [in newborns][Comment]

Are there situations or clinical findings in the patient where you deviate from your prophylaxis standard (medication, dose, duration, age of the patient)? (multiple answers possible, please specify in answer box) [Other]

Are there situations or clinical findings in the patient where you deviate from your prophylaxis standard (medication, dose, duration, age of the patient)? (multiple answers possible, please specify in answer box) [Other comment]

Are there situations in which you use intravenous prophylaxis?
No

Voiding analysis

Are there differences in antibiotic prophylaxis between VUS and VCUG in your department?
No

Is a non-pathological urine strip test required in preparation for VUS/VCUG in your department?
No

Is a non-pathological urine culture required in preparation for VUS/VCUG in your department?
No

Do patients only receive post-procedure prophylaxis if vesicoureteral reflux (VUR) is detected in VUS/VCUG?
No

Do other MUS/MCU findings (e.g. urethral valves) have an influence on post-procedure prophylaxis?
No

End of survey

Thank you for taking the time to answer this survey!

May we contact you personally if we have further questions?
Yes

E-mail address:
j.stegmann@kkh-wilhelmstift.de

Survey response 178

Response ID
180

Date submitted
2025-03-25 12:39:30

Last page
5

Start language
en

Seed
1288572720

Date started
2025-03-25 12:32:18

Date last action
2025-03-25 12:39:30

Standard Operation Procedure

Who fills out this survey?
Radiologist

Who fills out this survey? [Other]

In which type of hospital are you employed?
Other

In which type of hospital are you employed? [Other]
Ιδιώτης Ακτινολόγος

In which country do you work?
Ελλάδα

Does your department have a defined protocol for infection prophylaxis in the context of VUS/VCUG?
Yes

Has this protocol been transformed into a written standard operating procedure (SOP)?
Yes

Which department is the author of the SOP for infection prophylaxis in VUS/VCUG?
Other

Which department is the author of the SOP for infection prophylaxis in VUS/VCUG? [Other]
Παιδιατρος

Antibiotic Prophylaxis

Are there situations/constellations in which you do not use antibiotic prophylaxis for VUS/VCUG?
No

Please specify these situations/constellations: [girl with first uncomplicated UTI without urinary tract dilation in ultrasound]
N/A

Please specify these situations/constellations: [boy with first uncomplicated UTI without urinary tract dilation in ultrasound]
N/A

Please specify these situations/constellations: [in patients with low grade urinary tract dilation (up to 2nd grade) and no UTI in medical history]
N/A

If you use antibiotic prophylaxis: which mediaction do you use? (multiple answers and subdivision into age groups possible) [Trimethoprim]
No

If you use antibiotic prophylaxis: which mediaction do you use? (multiple answers and subdivision into age groups possible) [Nitrofurantoin]
No

If you use antibiotic prophylaxis: which mediaction do you use? (multiple answers and subdivision into age groups possible) [Amoxicillin]
Yes

If you use antibiotic prophylaxis: which mediaction do you use? (multiple answers and subdivision into age groups possible) [Cefaclor]
No

If you use antibiotic prophylaxis: which mediaction do you use? (multiple answers and subdivision into age groups possible) [Amoxicillin/clavulanic acid]
No

If you use antibiotic prophylaxis: which mediaction do you use? (multiple answers and subdivision into age groups possible) [Other]
No

Trimethoprim: which time schedule do you use? [Day(s) before VUS/VCUG, how many?]
N/A

Trimethoprim: which time schedule do you use? [Day of VUS/VCUG]
N/A

Trimethoprim: which time schedule do you use? [Day(s) after VUS/VCUG, how many?]
N/A

Nitrofurantoin: which time schedule do you use? [Day(s) before VUS/VCUG, how many?]
N/A

Nitrofurantoin: which time schedule do you use? [Day of VUS/VCUG]
N/A

Nitrofurantoin: which time schedule do you use? [Day(s) after VUS/VCUG, how many?]
N/A

Amoxicillin: in what dosage?
prophylactic (10-20 mg/kg bodyweight/d)

Amoxicillin: in what dosage? [Other]

Amoxicillin: for which age group do you use amoxicillin?
2 μηνών έως 3 ετων

Amoxicillin: which time schedule do you use? [Day(s) before VUS/VCUG, how many?]
Yes

Amoxicillin: which time schedule do you use? [Day(s) before VUS/VCUG, how many?][Comment]
1

Amoxicillin: which time schedule do you use? [Day of VUS/VCUG]
Yes

Amoxicillin: which time schedule do you use? [Day of VUS/VCUG][Comment]

Amoxicillin: which time schedule do you use? [Day(s) after VUS/VCUG, how many?]
Yes

Amoxicillin: which time schedule do you use? [Day(s) after VUS/VCUG, how many?][Comment]
1

Cefaclor: which time schedule do you use? [Days(s) before VUS/VCUG, how many?]
N/A

Cefaclor: which time schedule do you use? [Day of VUS/VCUG]
N/A

Cefaclor: which time schedule do you use? [Day(s) after VUS/VCUG, how many?]
N/A

Amoxicillin/clavulanic acid: in what dosage? (Amoxicillin portion) [prophylaktic (10-20 mg/Kg bodyweight/d)]
N/A

Amoxicillin/clavulanic acid: in what dosage? (Amoxicillin portion) [double prophylactic (20-40 mg/Kg bodyweight/d)]
N/A

Amoxicillin/clavulanic acid: in what dosage? (Amoxicillin portion) [therapeutic (80-100 mg/Kg bodyweight/d)]
N/A

Amoxicillin/clavulanic acid: which time schedule do you use? [Day(s) before VUS/VCUG]
N/A

Amoxicillin/clavulanic acid: which time schedule do you use? [Day of VUS/VCUG]
N/A

Amoxicillin/clavulanic acid: which time schedule do you use? [Day(s) after VUS/VCUG]
N/A

Other: which time schedule do you use? [Day(s) before VUS/VCUG, how many?]
N/A

Other: which time schedule do you use? [Day of VUS/VCUG]
N/A

Other: which time schedule do you use? [Day(s) after VUS/VCUG, how many?]
N/A

Indication for prophylaxis

Which patients receive periinterventional antibiotic prophylaxis? (multiple answers possible) [all]
Yes

Which patients receive periinterventional antibiotic prophylaxis? (multiple answers possible) [patients post urinary tract infection/pyelonephritis/urosepsis]
N/A

Which patients receive periinterventional antibiotic prophylaxis? (multiple answers possible) [patients with urinary tract dilation in ultrasound]
N/A

Which patients receive periinterventional antibiotic prophylaxis? (multiple answers possible) [patients with prenatal urinary tract dilation]
N/A

Which patients receive periinterventional antibiotic prophylaxis? (multiple answers possible) [patients post VUR surgery]
N/A

Which patients receive periinterventional antibiotic prophylaxis? (multiple answers possible) [none]
N/A

Are there situations or clinical findings in the patient where you deviate from your prophylaxis standard (medication, dose, duration, age of the patient)? (multiple answers possible, please specify in answer box) [in case of already known vesicoureteral reflux (VUR)]
No

Are there situations or clinical findings in the patient where you deviate from your prophylaxis standard (medication, dose, duration, age of the patient)? (multiple answers possible, please specify in answer box) [in case of already known vesicoureteral reflux (VUR)][Comment]

Are there situations or clinical findings in the patient where you deviate from your prophylaxis standard (medication, dose, duration, age of the patient)? (multiple answers possible, please specify in answer box) [in patients with recurrent urinary tract infections (UTI)]
No

Are there situations or clinical findings in the patient where you deviate from your prophylaxis standard (medication, dose, duration, age of the patient)? (multiple answers possible, please specify in answer box) [in patients with recurrent urinary tract infections (UTI)][Comment]

Are there situations or clinical findings in the patient where you deviate from your prophylaxis standard (medication, dose, duration, age of the patient)? (multiple answers possible, please specify in answer box) [if multidrug-resistant organisms (MDRO) are detected in urine cultures]
No

Are there situations or clinical findings in the patient where you deviate from your prophylaxis standard (medication, dose, duration, age of the patient)? (multiple answers possible, please specify in answer box) [if multidrug-resistant organisms (MDRO) are detected in urine cultures][Comment]

Are there situations or clinical findings in the patient where you deviate from your prophylaxis standard (medication, dose, duration, age of the patient)? (multiple answers possible, please specify in answer box) [in newborns]
Yes

Are there situations or clinical findings in the patient where you deviate from your prophylaxis standard (medication, dose, duration, age of the patient)? (multiple answers possible, please specify in answer box) [in newborns][Comment]

Are there situations or clinical findings in the patient where you deviate from your prophylaxis standard (medication, dose, duration, age of the patient)? (multiple answers possible, please specify in answer box) [Other]

Are there situations or clinical findings in the patient where you deviate from your prophylaxis standard (medication, dose, duration, age of the patient)? (multiple answers possible, please specify in answer box) [Other comment]

Are there situations in which you use intravenous prophylaxis?
No

Voiding analysis

Are there differences in antibiotic prophylaxis between VUS and VCUG in your department?
No

Is a non-pathological urine strip test required in preparation for VUS/VCUG in your department?
Yes

What is the maximum amount of days accepted for urine strip test to be performed prior to the exam?
Δυο

Is a non-pathological urine culture required in preparation for VUS/VCUG in your department?
Yes

What is the maximum amount of days accepted for urine culture to be performed prior to the exam?
Δυο

Do patients only receive post-procedure prophylaxis if vesicoureteral reflux (VUR) is detected in VUS/VCUG?
No

Do other MUS/MCU findings (e.g. urethral valves) have an influence on post-procedure prophylaxis?
No

End of survey

Thank you for taking the time to answer this survey!

May we contact you personally if we have further questions?
Yes

E-mail address:
andrianakik@gmail.com

Survey response 179

Response ID
181

Date submitted
2025-03-25 12:46:39

Last page
5

Start language
en

Seed
1056232430

Date started
2025-03-25 12:38:14

Date last action
2025-03-25 12:46:39

Standard Operation Procedure

Who fills out this survey?
Pediatric Radiologist

Who fills out this survey? [Other]

In which type of hospital are you employed?
University Hospital

In which type of hospital are you employed? [Other]

In which country do you work?
Norway

Does your department have a defined protocol for infection prophylaxis in the context of VUS/VCUG?
Yes

Has this protocol been transformed into a written standard operating procedure (SOP)?
Yes

Which department is the author of the SOP for infection prophylaxis in VUS/VCUG?
Pediatrics

Which department is the author of the SOP for infection prophylaxis in VUS/VCUG? [Other]

Antibiotic Prophylaxis

Are there situations/constellations in which you do not use antibiotic prophylaxis for VUS/VCUG?
No

Please specify these situations/constellations: [girl with first uncomplicated UTI without urinary tract dilation in ultrasound]
N/A

Please specify these situations/constellations: [boy with first uncomplicated UTI without urinary tract dilation in ultrasound]
N/A

Please specify these situations/constellations: [in patients with low grade urinary tract dilation (up to 2nd grade) and no UTI in medical history]
N/A

If you use antibiotic prophylaxis: which mediaction do you use? (multiple answers and subdivision into age groups possible) [Trimethoprim]
Yes

If you use antibiotic prophylaxis: which mediaction do you use? (multiple answers and subdivision into age groups possible) [Nitrofurantoin]
No

If you use antibiotic prophylaxis: which mediaction do you use? (multiple answers and subdivision into age groups possible) [Amoxicillin]
No

If you use antibiotic prophylaxis: which mediaction do you use? (multiple answers and subdivision into age groups possible) [Cefaclor]
No

If you use antibiotic prophylaxis: which mediaction do you use? (multiple answers and subdivision into age groups possible) [Amoxicillin/clavulanic acid]
No

If you use antibiotic prophylaxis: which mediaction do you use? (multiple answers and subdivision into age groups possible) [Other]
No

Trimethoprim: in what dosage?
double prophylakctic (4 mg/kg bodyweight/d)

Trimethoprim: in what dosage? [Other]

Trimethoprim: for which age group do you use trimethoprim?
All ages

Trimethoprim: which time schedule do you use? [Day(s) before VUS/VCUG, how many?]
No

Trimethoprim: which time schedule do you use? [Day(s) before VUS/VCUG, how many?][Comment]

Trimethoprim: which time schedule do you use? [Day of VUS/VCUG]
No

Trimethoprim: which time schedule do you use? [Day of VUS/VCUG][Comment]

Trimethoprim: which time schedule do you use? [Day(s) after VUS/VCUG, how many?]
Yes

Trimethoprim: which time schedule do you use? [Day(s) after VUS/VCUG, how many?][Comment]
3 days

Nitrofurantoin: which time schedule do you use? [Day(s) before VUS/VCUG, how many?]
N/A

Nitrofurantoin: which time schedule do you use? [Day of VUS/VCUG]
N/A

Nitrofurantoin: which time schedule do you use? [Day(s) after VUS/VCUG, how many?]
N/A

Amoxicillin: which time schedule do you use? [Day(s) before VUS/VCUG, how many?]
N/A

Amoxicillin: which time schedule do you use? [Day of VUS/VCUG]
N/A

Amoxicillin: which time schedule do you use? [Day(s) after VUS/VCUG, how many?]
N/A

Cefaclor: which time schedule do you use? [Days(s) before VUS/VCUG, how many?]
N/A

Cefaclor: which time schedule do you use? [Day of VUS/VCUG]
N/A

Cefaclor: which time schedule do you use? [Day(s) after VUS/VCUG, how many?]
N/A

Amoxicillin/clavulanic acid: in what dosage? (Amoxicillin portion) [prophylaktic (10-20 mg/Kg bodyweight/d)]
N/A

Amoxicillin/clavulanic acid: in what dosage? (Amoxicillin portion) [double prophylactic (20-40 mg/Kg bodyweight/d)]
N/A

Amoxicillin/clavulanic acid: in what dosage? (Amoxicillin portion) [therapeutic (80-100 mg/Kg bodyweight/d)]
N/A

Amoxicillin/clavulanic acid: which time schedule do you use? [Day(s) before VUS/VCUG]
N/A

Amoxicillin/clavulanic acid: which time schedule do you use? [Day of VUS/VCUG]
N/A

Amoxicillin/clavulanic acid: which time schedule do you use? [Day(s) after VUS/VCUG]
N/A

Other: which time schedule do you use? [Day(s) before VUS/VCUG, how many?]
N/A

Other: which time schedule do you use? [Day of VUS/VCUG]
N/A

Other: which time schedule do you use? [Day(s) after VUS/VCUG, how many?]
N/A

Indication for prophylaxis

Which patients receive periinterventional antibiotic prophylaxis? (multiple answers possible) [all]
Yes

Which patients receive periinterventional antibiotic prophylaxis? (multiple answers possible) [patients post urinary tract infection/pyelonephritis/urosepsis]
N/A

Which patients receive periinterventional antibiotic prophylaxis? (multiple answers possible) [patients with urinary tract dilation in ultrasound]
N/A

Which patients receive periinterventional antibiotic prophylaxis? (multiple answers possible) [patients with prenatal urinary tract dilation]
N/A

Which patients receive periinterventional antibiotic prophylaxis? (multiple answers possible) [patients post VUR surgery]
N/A

Which patients receive periinterventional antibiotic prophylaxis? (multiple answers possible) [none]
N/A

Are there situations or clinical findings in the patient where you deviate from your prophylaxis standard (medication, dose, duration, age of the patient)? (multiple answers possible, please specify in answer box) [in case of already known vesicoureteral reflux (VUR)]
Yes

Are there situations or clinical findings in the patient where you deviate from your prophylaxis standard (medication, dose, duration, age of the patient)? (multiple answers possible, please specify in answer box) [in case of already known vesicoureteral reflux (VUR)][Comment]

Are there situations or clinical findings in the patient where you deviate from your prophylaxis standard (medication, dose, duration, age of the patient)? (multiple answers possible, please specify in answer box) [in patients with recurrent urinary tract infections (UTI)]
No

Are there situations or clinical findings in the patient where you deviate from your prophylaxis standard (medication, dose, duration, age of the patient)? (multiple answers possible, please specify in answer box) [in patients with recurrent urinary tract infections (UTI)][Comment]

Are there situations or clinical findings in the patient where you deviate from your prophylaxis standard (medication, dose, duration, age of the patient)? (multiple answers possible, please specify in answer box) [if multidrug-resistant organisms (MDRO) are detected in urine cultures]
No

Are there situations or clinical findings in the patient where you deviate from your prophylaxis standard (medication, dose, duration, age of the patient)? (multiple answers possible, please specify in answer box) [if multidrug-resistant organisms (MDRO) are detected in urine cultures][Comment]

Are there situations or clinical findings in the patient where you deviate from your prophylaxis standard (medication, dose, duration, age of the patient)? (multiple answers possible, please specify in answer box) [in newborns]
No

Are there situations or clinical findings in the patient where you deviate from your prophylaxis standard (medication, dose, duration, age of the patient)? (multiple answers possible, please specify in answer box) [in newborns][Comment]

Are there situations or clinical findings in the patient where you deviate from your prophylaxis standard (medication, dose, duration, age of the patient)? (multiple answers possible, please specify in answer box) [Other]
If already on prophylaxis: double dose for three days

Are there situations or clinical findings in the patient where you deviate from your prophylaxis standard (medication, dose, duration, age of the patient)? (multiple answers possible, please specify in answer box) [Other comment]
If not yet on prophylaxis: only prophylactic dose for three days

Are there situations in which you use intravenous prophylaxis?
No

Voiding analysis

Are there differences in antibiotic prophylaxis between VUS and VCUG in your department?
No

Is a non-pathological urine strip test required in preparation for VUS/VCUG in your department?
Yes

What is the maximum amount of days accepted for urine strip test to be performed prior to the exam?
Same day

Is a non-pathological urine culture required in preparation for VUS/VCUG in your department?
No

Do patients only receive post-procedure prophylaxis if vesicoureteral reflux (VUR) is detected in VUS/VCUG?
No

Do other MUS/MCU findings (e.g. urethral valves) have an influence on post-procedure prophylaxis?
No

End of survey

Thank you for taking the time to answer this survey!

May we contact you personally if we have further questions?
Yes

E-mail address:
joosjebomer@gmail.com

Survey response 180

Response ID
182

Last page
1

Start language
en

Seed
739998959

Date started
2025-03-25 13:07:04

Date last action
2025-03-25 13:07:59

Standard Operation Procedure

Who fills out this survey?
Pediatric Radiologist

Who fills out this survey? [Other]

In which type of hospital are you employed?
Community hospital (>50 beds for pediatric patients)

In which type of hospital are you employed? [Other]

In which country do you work?
Portugal

Does your department have a defined protocol for infection prophylaxis in the context of VUS/VCUG?
Yes

Has this protocol been transformed into a written standard operating procedure (SOP)?
Yes

Which department is the author of the SOP for infection prophylaxis in VUS/VCUG?
Pediatrics

Which department is the author of the SOP for infection prophylaxis in VUS/VCUG? [Other]

Antibiotic Prophylaxis

Are there situations/constellations in which you do not use antibiotic prophylaxis for VUS/VCUG?
N/A

Please specify these situations/constellations: [girl with first uncomplicated UTI without urinary tract dilation in ultrasound]
N/A

Please specify these situations/constellations: [boy with first uncomplicated UTI without urinary tract dilation in ultrasound]
N/A

Please specify these situations/constellations: [in patients with low grade urinary tract dilation (up to 2nd grade) and no UTI in medical history]
N/A

If you use antibiotic prophylaxis: which mediaction do you use? (multiple answers and subdivision into age groups possible) [Trimethoprim]
N/A

If you use antibiotic prophylaxis: which mediaction do you use? (multiple answers and subdivision into age groups possible) [Nitrofurantoin]
N/A

If you use antibiotic prophylaxis: which mediaction do you use? (multiple answers and subdivision into age groups possible) [Amoxicillin]
N/A

If you use antibiotic prophylaxis: which mediaction do you use? (multiple answers and subdivision into age groups possible) [Cefaclor]
N/A

If you use antibiotic prophylaxis: which mediaction do you use? (multiple answers and subdivision into age groups possible) [Amoxicillin/clavulanic acid]
N/A

If you use antibiotic prophylaxis: which mediaction do you use? (multiple answers and subdivision into age groups possible) [Other]
N/A

Trimethoprim: which time schedule do you use? [Day(s) before VUS/VCUG, how many?]
N/A

Trimethoprim: which time schedule do you use? [Day of VUS/VCUG]
N/A

Trimethoprim: which time schedule do you use? [Day(s) after VUS/VCUG, how many?]
N/A

Nitrofurantoin: which time schedule do you use? [Day(s) before VUS/VCUG, how many?]
N/A

Nitrofurantoin: which time schedule do you use? [Day of VUS/VCUG]
N/A

Nitrofurantoin: which time schedule do you use? [Day(s) after VUS/VCUG, how many?]
N/A

Amoxicillin: which time schedule do you use? [Day(s) before VUS/VCUG, how many?]
N/A

Amoxicillin: which time schedule do you use? [Day of VUS/VCUG]
N/A

Amoxicillin: which time schedule do you use? [Day(s) after VUS/VCUG, how many?]
N/A

Cefaclor: which time schedule do you use? [Days(s) before VUS/VCUG, how many?]
N/A

Cefaclor: which time schedule do you use? [Day of VUS/VCUG]
N/A

Cefaclor: which time schedule do you use? [Day(s) after VUS/VCUG, how many?]
N/A

Amoxicillin/clavulanic acid: in what dosage? (Amoxicillin portion) [prophylaktic (10-20 mg/Kg bodyweight/d)]
N/A

Amoxicillin/clavulanic acid: in what dosage? (Amoxicillin portion) [double prophylactic (20-40 mg/Kg bodyweight/d)]
N/A

Amoxicillin/clavulanic acid: in what dosage? (Amoxicillin portion) [therapeutic (80-100 mg/Kg bodyweight/d)]
N/A

Amoxicillin/clavulanic acid: which time schedule do you use? [Day(s) before VUS/VCUG]
N/A

Amoxicillin/clavulanic acid: which time schedule do you use? [Day of VUS/VCUG]
N/A

Amoxicillin/clavulanic acid: which time schedule do you use? [Day(s) after VUS/VCUG]
N/A

Other: which time schedule do you use? [Day(s) before VUS/VCUG, how many?]
N/A

Other: which time schedule do you use? [Day of VUS/VCUG]
N/A

Other: which time schedule do you use? [Day(s) after VUS/VCUG, how many?]
N/A

Indication for prophylaxis

Which patients receive periinterventional antibiotic prophylaxis? (multiple answers possible) [all]
N/A

Which patients receive periinterventional antibiotic prophylaxis? (multiple answers possible) [patients post urinary tract infection/pyelonephritis/urosepsis]
N/A

Which patients receive periinterventional antibiotic prophylaxis? (multiple answers possible) [patients with urinary tract dilation in ultrasound]
N/A

Which patients receive periinterventional antibiotic prophylaxis? (multiple answers possible) [patients with prenatal urinary tract dilation]
N/A

Which patients receive periinterventional antibiotic prophylaxis? (multiple answers possible) [patients post VUR surgery]
N/A

Which patients receive periinterventional antibiotic prophylaxis? (multiple answers possible) [none]
N/A

Are there situations or clinical findings in the patient where you deviate from your prophylaxis standard (medication, dose, duration, age of the patient)? (multiple answers possible, please specify in answer box) [in case of already known vesicoureteral reflux (VUR)]
N/A

Are there situations or clinical findings in the patient where you deviate from your prophylaxis standard (medication, dose, duration, age of the patient)? (multiple answers possible, please specify in answer box) [in patients with recurrent urinary tract infections (UTI)]
N/A

Are there situations or clinical findings in the patient where you deviate from your prophylaxis standard (medication, dose, duration, age of the patient)? (multiple answers possible, please specify in answer box) [if multidrug-resistant organisms (MDRO) are detected in urine cultures]
N/A

Are there situations or clinical findings in the patient where you deviate from your prophylaxis standard (medication, dose, duration, age of the patient)? (multiple answers possible, please specify in answer box) [in newborns]
N/A

Are there situations in which you use intravenous prophylaxis?
N/A

Voiding analysis

Are there differences in antibiotic prophylaxis between VUS and VCUG in your department?
N/A

Is a non-pathological urine strip test required in preparation for VUS/VCUG in your department?
N/A

Is a non-pathological urine culture required in preparation for VUS/VCUG in your department?
N/A

Do patients only receive post-procedure prophylaxis if vesicoureteral reflux (VUR) is detected in VUS/VCUG?
N/A

Do other MUS/MCU findings (e.g. urethral valves) have an influence on post-procedure prophylaxis?
N/A

End of survey

May we contact you personally if we have further questions?
N/A

Survey response 181

Response ID
183

Last page
1

Start language
en

Seed
814889506

Date started
2025-03-25 13:57:46

Date last action
2025-03-25 13:59:46

Standard Operation Procedure

Who fills out this survey?
Pediatric Radiologist

Who fills out this survey? [Other]

In which type of hospital are you employed?
Community hospital (>50 beds for pediatric patients)

In which type of hospital are you employed? [Other]

In which country do you work?
Türkiye

Does your department have a defined protocol for infection prophylaxis in the context of VUS/VCUG?
Yes

Has this protocol been transformed into a written standard operating procedure (SOP)?
Yes

Which department is the author of the SOP for infection prophylaxis in VUS/VCUG?
Pediatric radiology

Which department is the author of the SOP for infection prophylaxis in VUS/VCUG? [Other]

Antibiotic Prophylaxis

Are there situations/constellations in which you do not use antibiotic prophylaxis for VUS/VCUG?
Yes

Please specify these situations/constellations: [girl with first uncomplicated UTI without urinary tract dilation in ultrasound]
Yes

Please specify these situations/constellations: [boy with first uncomplicated UTI without urinary tract dilation in ultrasound]
Yes

Please specify these situations/constellations: [in patients with low grade urinary tract dilation (up to 2nd grade) and no UTI in medical history]
Yes

Please specify these situations/constellations: [Other]

If you use antibiotic prophylaxis: which mediaction do you use? (multiple answers and subdivision into age groups possible) [Trimethoprim]
No

If you use antibiotic prophylaxis: which mediaction do you use? (multiple answers and subdivision into age groups possible) [Nitrofurantoin]
Yes

If you use antibiotic prophylaxis: which mediaction do you use? (multiple answers and subdivision into age groups possible) [Amoxicillin]
No

If you use antibiotic prophylaxis: which mediaction do you use? (multiple answers and subdivision into age groups possible) [Cefaclor]
No

If you use antibiotic prophylaxis: which mediaction do you use? (multiple answers and subdivision into age groups possible) [Amoxicillin/clavulanic acid]
No

If you use antibiotic prophylaxis: which mediaction do you use? (multiple answers and subdivision into age groups possible) [Other]
No

Trimethoprim: which time schedule do you use? [Day(s) before VUS/VCUG, how many?]
N/A

Trimethoprim: which time schedule do you use? [Day of VUS/VCUG]
N/A

Trimethoprim: which time schedule do you use? [Day(s) after VUS/VCUG, how many?]
N/A

Nitrofurantoin: in what dosage?
prophylactic (1-2 mg/kg bodyweight/d)

Nitrofurantoin: in what dosage? [Other]

Nitrofurantoin: for which age group do you use nitrofurantoin?

Nitrofurantoin: which time schedule do you use? [Day(s) before VUS/VCUG, how many?]
Yes

Nitrofurantoin: which time schedule do you use? [Day(s) before VUS/VCUG, how many?][Comment]
3

Nitrofurantoin: which time schedule do you use? [Day of VUS/VCUG]
No

Nitrofurantoin: which time schedule do you use? [Day of VUS/VCUG][Comment]

Nitrofurantoin: which time schedule do you use? [Day(s) after VUS/VCUG, how many?]
No

Nitrofurantoin: which time schedule do you use? [Day(s) after VUS/VCUG, how many?][Comment]

Amoxicillin: which time schedule do you use? [Day(s) before VUS/VCUG, how many?]
N/A

Amoxicillin: which time schedule do you use? [Day of VUS/VCUG]
N/A

Amoxicillin: which time schedule do you use? [Day(s) after VUS/VCUG, how many?]
N/A

Cefaclor: which time schedule do you use? [Days(s) before VUS/VCUG, how many?]
N/A

Cefaclor: which time schedule do you use? [Day of VUS/VCUG]
N/A

Cefaclor: which time schedule do you use? [Day(s) after VUS/VCUG, how many?]
N/A

Amoxicillin/clavulanic acid: in what dosage? (Amoxicillin portion) [prophylaktic (10-20 mg/Kg bodyweight/d)]
N/A

Amoxicillin/clavulanic acid: in what dosage? (Amoxicillin portion) [double prophylactic (20-40 mg/Kg bodyweight/d)]
N/A

Amoxicillin/clavulanic acid: in what dosage? (Amoxicillin portion) [therapeutic (80-100 mg/Kg bodyweight/d)]
N/A

Amoxicillin/clavulanic acid: which time schedule do you use? [Day(s) before VUS/VCUG]
N/A

Amoxicillin/clavulanic acid: which time schedule do you use? [Day of VUS/VCUG]
N/A

Amoxicillin/clavulanic acid: which time schedule do you use? [Day(s) after VUS/VCUG]
N/A

Other: which time schedule do you use? [Day(s) before VUS/VCUG, how many?]
N/A

Other: which time schedule do you use? [Day of VUS/VCUG]
N/A

Other: which time schedule do you use? [Day(s) after VUS/VCUG, how many?]
N/A

Indication for prophylaxis

Which patients receive periinterventional antibiotic prophylaxis? (multiple answers possible) [all]
N/A

Which patients receive periinterventional antibiotic prophylaxis? (multiple answers possible) [patients post urinary tract infection/pyelonephritis/urosepsis]
N/A

Which patients receive periinterventional antibiotic prophylaxis? (multiple answers possible) [patients with urinary tract dilation in ultrasound]
N/A

Which patients receive periinterventional antibiotic prophylaxis? (multiple answers possible) [patients with prenatal urinary tract dilation]
N/A

Which patients receive periinterventional antibiotic prophylaxis? (multiple answers possible) [patients post VUR surgery]
N/A

Which patients receive periinterventional antibiotic prophylaxis? (multiple answers possible) [none]
N/A

Are there situations or clinical findings in the patient where you deviate from your prophylaxis standard (medication, dose, duration, age of the patient)? (multiple answers possible, please specify in answer box) [in case of already known vesicoureteral reflux (VUR)]
N/A

Are there situations or clinical findings in the patient where you deviate from your prophylaxis standard (medication, dose, duration, age of the patient)? (multiple answers possible, please specify in answer box) [in patients with recurrent urinary tract infections (UTI)]
N/A

Are there situations or clinical findings in the patient where you deviate from your prophylaxis standard (medication, dose, duration, age of the patient)? (multiple answers possible, please specify in answer box) [if multidrug-resistant organisms (MDRO) are detected in urine cultures]
N/A

Are there situations or clinical findings in the patient where you deviate from your prophylaxis standard (medication, dose, duration, age of the patient)? (multiple answers possible, please specify in answer box) [in newborns]
N/A

Are there situations in which you use intravenous prophylaxis?
N/A

Voiding analysis

Are there differences in antibiotic prophylaxis between VUS and VCUG in your department?
N/A

Is a non-pathological urine strip test required in preparation for VUS/VCUG in your department?
N/A

Is a non-pathological urine culture required in preparation for VUS/VCUG in your department?
N/A

Do patients only receive post-procedure prophylaxis if vesicoureteral reflux (VUR) is detected in VUS/VCUG?
N/A

Do other MUS/MCU findings (e.g. urethral valves) have an influence on post-procedure prophylaxis?
N/A

End of survey

May we contact you personally if we have further questions?
N/A

Survey response 182

Response ID
184

Date submitted
2025-03-25 15:26:07

Last page
5

Start language
en

Seed
72088789

Date started
2025-03-25 15:21:22

Date last action
2025-03-25 15:26:07

Standard Operation Procedure

Who fills out this survey?
Pediatric Radiologist

Who fills out this survey? [Other]

In which type of hospital are you employed?
University Hospital

In which type of hospital are you employed? [Other]

In which country do you work?
United Kingdom

Does your department have a defined protocol for infection prophylaxis in the context of VUS/VCUG?
Yes

Has this protocol been transformed into a written standard operating procedure (SOP)?
Yes

Which department is the author of the SOP for infection prophylaxis in VUS/VCUG?
Pediatrics

Which department is the author of the SOP for infection prophylaxis in VUS/VCUG? [Other]

Antibiotic Prophylaxis

Are there situations/constellations in which you do not use antibiotic prophylaxis for VUS/VCUG?
No

Please specify these situations/constellations: [girl with first uncomplicated UTI without urinary tract dilation in ultrasound]
N/A

Please specify these situations/constellations: [boy with first uncomplicated UTI without urinary tract dilation in ultrasound]
N/A

Please specify these situations/constellations: [in patients with low grade urinary tract dilation (up to 2nd grade) and no UTI in medical history]
N/A

If you use antibiotic prophylaxis: which mediaction do you use? (multiple answers and subdivision into age groups possible) [Trimethoprim]
Yes

If you use antibiotic prophylaxis: which mediaction do you use? (multiple answers and subdivision into age groups possible) [Nitrofurantoin]
No

If you use antibiotic prophylaxis: which mediaction do you use? (multiple answers and subdivision into age groups possible) [Amoxicillin]
No

If you use antibiotic prophylaxis: which mediaction do you use? (multiple answers and subdivision into age groups possible) [Cefaclor]
No

If you use antibiotic prophylaxis: which mediaction do you use? (multiple answers and subdivision into age groups possible) [Amoxicillin/clavulanic acid]
No

If you use antibiotic prophylaxis: which mediaction do you use? (multiple answers and subdivision into age groups possible) [Other]
No

Trimethoprim: in what dosage?
Other

Trimethoprim: in what dosage? [Other]
Quadruple dose - Double prophylactic AND double frequency

Trimethoprim: for which age group do you use trimethoprim?
All

Trimethoprim: which time schedule do you use? [Day(s) before VUS/VCUG, how many?]
Yes

Trimethoprim: which time schedule do you use? [Day(s) before VUS/VCUG, how many?][Comment]
1

Trimethoprim: which time schedule do you use? [Day of VUS/VCUG]
Yes

Trimethoprim: which time schedule do you use? [Day of VUS/VCUG][Comment]

Trimethoprim: which time schedule do you use? [Day(s) after VUS/VCUG, how many?]
Yes

Trimethoprim: which time schedule do you use? [Day(s) after VUS/VCUG, how many?][Comment]
1

Nitrofurantoin: which time schedule do you use? [Day(s) before VUS/VCUG, how many?]
N/A

Nitrofurantoin: which time schedule do you use? [Day of VUS/VCUG]
N/A

Nitrofurantoin: which time schedule do you use? [Day(s) after VUS/VCUG, how many?]
N/A

Amoxicillin: which time schedule do you use? [Day(s) before VUS/VCUG, how many?]
N/A

Amoxicillin: which time schedule do you use? [Day of VUS/VCUG]
N/A

Amoxicillin: which time schedule do you use? [Day(s) after VUS/VCUG, how many?]
N/A

Cefaclor: which time schedule do you use? [Days(s) before VUS/VCUG, how many?]
N/A

Cefaclor: which time schedule do you use? [Day of VUS/VCUG]
N/A

Cefaclor: which time schedule do you use? [Day(s) after VUS/VCUG, how many?]
N/A

Amoxicillin/clavulanic acid: in what dosage? (Amoxicillin portion) [prophylaktic (10-20 mg/Kg bodyweight/d)]
N/A

Amoxicillin/clavulanic acid: in what dosage? (Amoxicillin portion) [double prophylactic (20-40 mg/Kg bodyweight/d)]
N/A

Amoxicillin/clavulanic acid: in what dosage? (Amoxicillin portion) [therapeutic (80-100 mg/Kg bodyweight/d)]
N/A

Amoxicillin/clavulanic acid: which time schedule do you use? [Day(s) before VUS/VCUG]
N/A

Amoxicillin/clavulanic acid: which time schedule do you use? [Day of VUS/VCUG]
N/A

Amoxicillin/clavulanic acid: which time schedule do you use? [Day(s) after VUS/VCUG]
N/A

Other: which time schedule do you use? [Day(s) before VUS/VCUG, how many?]
N/A

Other: which time schedule do you use? [Day of VUS/VCUG]
N/A

Other: which time schedule do you use? [Day(s) after VUS/VCUG, how many?]
N/A

Indication for prophylaxis

Which patients receive periinterventional antibiotic prophylaxis? (multiple answers possible) [all]
Yes

Which patients receive periinterventional antibiotic prophylaxis? (multiple answers possible) [patients post urinary tract infection/pyelonephritis/urosepsis]
N/A

Which patients receive periinterventional antibiotic prophylaxis? (multiple answers possible) [patients with urinary tract dilation in ultrasound]
N/A

Which patients receive periinterventional antibiotic prophylaxis? (multiple answers possible) [patients with prenatal urinary tract dilation]
N/A

Which patients receive periinterventional antibiotic prophylaxis? (multiple answers possible) [patients post VUR surgery]
N/A

Which patients receive periinterventional antibiotic prophylaxis? (multiple answers possible) [none]
N/A

Are there situations or clinical findings in the patient where you deviate from your prophylaxis standard (medication, dose, duration, age of the patient)? (multiple answers possible, please specify in answer box) [in case of already known vesicoureteral reflux (VUR)]
No

Are there situations or clinical findings in the patient where you deviate from your prophylaxis standard (medication, dose, duration, age of the patient)? (multiple answers possible, please specify in answer box) [in case of already known vesicoureteral reflux (VUR)][Comment]

Are there situations or clinical findings in the patient where you deviate from your prophylaxis standard (medication, dose, duration, age of the patient)? (multiple answers possible, please specify in answer box) [in patients with recurrent urinary tract infections (UTI)]
No

Are there situations or clinical findings in the patient where you deviate from your prophylaxis standard (medication, dose, duration, age of the patient)? (multiple answers possible, please specify in answer box) [in patients with recurrent urinary tract infections (UTI)][Comment]

Are there situations or clinical findings in the patient where you deviate from your prophylaxis standard (medication, dose, duration, age of the patient)? (multiple answers possible, please specify in answer box) [if multidrug-resistant organisms (MDRO) are detected in urine cultures]
Yes

Are there situations or clinical findings in the patient where you deviate from your prophylaxis standard (medication, dose, duration, age of the patient)? (multiple answers possible, please specify in answer box) [if multidrug-resistant organisms (MDRO) are detected in urine cultures][Comment]

Are there situations or clinical findings in the patient where you deviate from your prophylaxis standard (medication, dose, duration, age of the patient)? (multiple answers possible, please specify in answer box) [in newborns]
No

Are there situations or clinical findings in the patient where you deviate from your prophylaxis standard (medication, dose, duration, age of the patient)? (multiple answers possible, please specify in answer box) [in newborns][Comment]

Are there situations or clinical findings in the patient where you deviate from your prophylaxis standard (medication, dose, duration, age of the patient)? (multiple answers possible, please specify in answer box) [Other]
Allergy - eg nitrofurantoin

Are there situations or clinical findings in the patient where you deviate from your prophylaxis standard (medication, dose, duration, age of the patient)? (multiple answers possible, please specify in answer box) [Other comment]
Allergy - eg nitrofurantoin

Are there situations in which you use intravenous prophylaxis?
Yes

In which situations?
Rare - incorrectly taken oral prophylaxis, in a study needing to be done urgently

Voiding analysis

Are there differences in antibiotic prophylaxis between VUS and VCUG in your department?
No

Is a non-pathological urine strip test required in preparation for VUS/VCUG in your department?
No

Is a non-pathological urine culture required in preparation for VUS/VCUG in your department?
No

Do patients only receive post-procedure prophylaxis if vesicoureteral reflux (VUR) is detected in VUS/VCUG?
No

Do other MUS/MCU findings (e.g. urethral valves) have an influence on post-procedure prophylaxis?
No

End of survey

Thank you for taking the time to answer this survey!

May we contact you personally if we have further questions?
No

Survey response 183

Response ID
185

Last page
0

Start language
en

Seed
1991403965

Date started
2025-03-25 16:31:39

Date last action
2025-03-25 16:31:39

Standard Operation Procedure

Does your department have a defined protocol for infection prophylaxis in the context of VUS/VCUG?
N/A

Has this protocol been transformed into a written standard operating procedure (SOP)?
N/A

Antibiotic Prophylaxis

Are there situations/constellations in which you do not use antibiotic prophylaxis for VUS/VCUG?
N/A

Please specify these situations/constellations: [girl with first uncomplicated UTI without urinary tract dilation in ultrasound]
N/A

Please specify these situations/constellations: [boy with first uncomplicated UTI without urinary tract dilation in ultrasound]
N/A

Please specify these situations/constellations: [in patients with low grade urinary tract dilation (up to 2nd grade) and no UTI in medical history]
N/A

If you use antibiotic prophylaxis: which mediaction do you use? (multiple answers and subdivision into age groups possible) [Trimethoprim]
N/A

If you use antibiotic prophylaxis: which mediaction do you use? (multiple answers and subdivision into age groups possible) [Nitrofurantoin]
N/A

If you use antibiotic prophylaxis: which mediaction do you use? (multiple answers and subdivision into age groups possible) [Amoxicillin]
N/A

If you use antibiotic prophylaxis: which mediaction do you use? (multiple answers and subdivision into age groups possible) [Cefaclor]
N/A

If you use antibiotic prophylaxis: which mediaction do you use? (multiple answers and subdivision into age groups possible) [Amoxicillin/clavulanic acid]
N/A

If you use antibiotic prophylaxis: which mediaction do you use? (multiple answers and subdivision into age groups possible) [Other]
N/A

Trimethoprim: which time schedule do you use? [Day(s) before VUS/VCUG, how many?]
N/A

Trimethoprim: which time schedule do you use? [Day of VUS/VCUG]
N/A

Trimethoprim: which time schedule do you use? [Day(s) after VUS/VCUG, how many?]
N/A

Nitrofurantoin: which time schedule do you use? [Day(s) before VUS/VCUG, how many?]
N/A

Nitrofurantoin: which time schedule do you use? [Day of VUS/VCUG]
N/A

Nitrofurantoin: which time schedule do you use? [Day(s) after VUS/VCUG, how many?]
N/A

Amoxicillin: which time schedule do you use? [Day(s) before VUS/VCUG, how many?]
N/A

Amoxicillin: which time schedule do you use? [Day of VUS/VCUG]
N/A

Amoxicillin: which time schedule do you use? [Day(s) after VUS/VCUG, how many?]
N/A

Cefaclor: which time schedule do you use? [Days(s) before VUS/VCUG, how many?]
N/A

Cefaclor: which time schedule do you use? [Day of VUS/VCUG]
N/A

Cefaclor: which time schedule do you use? [Day(s) after VUS/VCUG, how many?]
N/A

Amoxicillin/clavulanic acid: in what dosage? (Amoxicillin portion) [prophylaktic (10-20 mg/Kg bodyweight/d)]
N/A

Amoxicillin/clavulanic acid: in what dosage? (Amoxicillin portion) [double prophylactic (20-40 mg/Kg bodyweight/d)]
N/A

Amoxicillin/clavulanic acid: in what dosage? (Amoxicillin portion) [therapeutic (80-100 mg/Kg bodyweight/d)]
N/A

Amoxicillin/clavulanic acid: which time schedule do you use? [Day(s) before VUS/VCUG]
N/A

Amoxicillin/clavulanic acid: which time schedule do you use? [Day of VUS/VCUG]
N/A

Amoxicillin/clavulanic acid: which time schedule do you use? [Day(s) after VUS/VCUG]
N/A

Other: which time schedule do you use? [Day(s) before VUS/VCUG, how many?]
N/A

Other: which time schedule do you use? [Day of VUS/VCUG]
N/A

Other: which time schedule do you use? [Day(s) after VUS/VCUG, how many?]
N/A

Indication for prophylaxis

Which patients receive periinterventional antibiotic prophylaxis? (multiple answers possible) [all]
N/A

Which patients receive periinterventional antibiotic prophylaxis? (multiple answers possible) [patients post urinary tract infection/pyelonephritis/urosepsis]
N/A

Which patients receive periinterventional antibiotic prophylaxis? (multiple answers possible) [patients with urinary tract dilation in ultrasound]
N/A

Which patients receive periinterventional antibiotic prophylaxis? (multiple answers possible) [patients with prenatal urinary tract dilation]
N/A

Which patients receive periinterventional antibiotic prophylaxis? (multiple answers possible) [patients post VUR surgery]
N/A

Which patients receive periinterventional antibiotic prophylaxis? (multiple answers possible) [none]
N/A

Are there situations or clinical findings in the patient where you deviate from your prophylaxis standard (medication, dose, duration, age of the patient)? (multiple answers possible, please specify in answer box) [in case of already known vesicoureteral reflux (VUR)]
N/A

Are there situations or clinical findings in the patient where you deviate from your prophylaxis standard (medication, dose, duration, age of the patient)? (multiple answers possible, please specify in answer box) [in patients with recurrent urinary tract infections (UTI)]
N/A

Are there situations or clinical findings in the patient where you deviate from your prophylaxis standard (medication, dose, duration, age of the patient)? (multiple answers possible, please specify in answer box) [if multidrug-resistant organisms (MDRO) are detected in urine cultures]
N/A

Are there situations or clinical findings in the patient where you deviate from your prophylaxis standard (medication, dose, duration, age of the patient)? (multiple answers possible, please specify in answer box) [in newborns]
N/A

Are there situations in which you use intravenous prophylaxis?
N/A

Voiding analysis

Are there differences in antibiotic prophylaxis between VUS and VCUG in your department?
N/A

Is a non-pathological urine strip test required in preparation for VUS/VCUG in your department?
N/A

Is a non-pathological urine culture required in preparation for VUS/VCUG in your department?
N/A

Do patients only receive post-procedure prophylaxis if vesicoureteral reflux (VUR) is detected in VUS/VCUG?
N/A

Do other MUS/MCU findings (e.g. urethral valves) have an influence on post-procedure prophylaxis?
N/A

End of survey

May we contact you personally if we have further questions?
N/A

Survey response 184

Response ID
186

Last page
3

Start language
en

Seed
1687626822

Date started
2025-03-25 16:34:01

Date last action
2025-03-25 16:35:55

Standard Operation Procedure

Who fills out this survey?
Other

Who fills out this survey? [Other]
asdasdasdasd

In which type of hospital are you employed?
University Hospital

In which type of hospital are you employed? [Other]

In which country do you work?
asd

Does your department have a defined protocol for infection prophylaxis in the context of VUS/VCUG?
Yes

Has this protocol been transformed into a written standard operating procedure (SOP)?
No

Antibiotic Prophylaxis

Are there situations/constellations in which you do not use antibiotic prophylaxis for VUS/VCUG?
Yes

Please specify these situations/constellations: [girl with first uncomplicated UTI without urinary tract dilation in ultrasound]
Yes

Please specify these situations/constellations: [boy with first uncomplicated UTI without urinary tract dilation in ultrasound]
Yes

Please specify these situations/constellations: [in patients with low grade urinary tract dilation (up to 2nd grade) and no UTI in medical history]
Yes

Please specify these situations/constellations: [Other]

If you use antibiotic prophylaxis: which mediaction do you use? (multiple answers and subdivision into age groups possible) [Trimethoprim]
No

If you use antibiotic prophylaxis: which mediaction do you use? (multiple answers and subdivision into age groups possible) [Nitrofurantoin]
No

If you use antibiotic prophylaxis: which mediaction do you use? (multiple answers and subdivision into age groups possible) [Amoxicillin]
No

If you use antibiotic prophylaxis: which mediaction do you use? (multiple answers and subdivision into age groups possible) [Cefaclor]
No

If you use antibiotic prophylaxis: which mediaction do you use? (multiple answers and subdivision into age groups possible) [Amoxicillin/clavulanic acid]
No

If you use antibiotic prophylaxis: which mediaction do you use? (multiple answers and subdivision into age groups possible) [Other]
No

Trimethoprim: which time schedule do you use? [Day(s) before VUS/VCUG, how many?]
N/A

Trimethoprim: which time schedule do you use? [Day of VUS/VCUG]
N/A

Trimethoprim: which time schedule do you use? [Day(s) after VUS/VCUG, how many?]
N/A

Nitrofurantoin: which time schedule do you use? [Day(s) before VUS/VCUG, how many?]
N/A

Nitrofurantoin: which time schedule do you use? [Day of VUS/VCUG]
N/A

Nitrofurantoin: which time schedule do you use? [Day(s) after VUS/VCUG, how many?]
N/A

Amoxicillin: which time schedule do you use? [Day(s) before VUS/VCUG, how many?]
N/A

Amoxicillin: which time schedule do you use? [Day of VUS/VCUG]
N/A

Amoxicillin: which time schedule do you use? [Day(s) after VUS/VCUG, how many?]
N/A

Cefaclor: which time schedule do you use? [Days(s) before VUS/VCUG, how many?]
N/A

Cefaclor: which time schedule do you use? [Day of VUS/VCUG]
N/A

Cefaclor: which time schedule do you use? [Day(s) after VUS/VCUG, how many?]
N/A

Amoxicillin/clavulanic acid: in what dosage? (Amoxicillin portion) [prophylaktic (10-20 mg/Kg bodyweight/d)]
N/A

Amoxicillin/clavulanic acid: in what dosage? (Amoxicillin portion) [double prophylactic (20-40 mg/Kg bodyweight/d)]
N/A

Amoxicillin/clavulanic acid: in what dosage? (Amoxicillin portion) [therapeutic (80-100 mg/Kg bodyweight/d)]
N/A

Amoxicillin/clavulanic acid: which time schedule do you use? [Day(s) before VUS/VCUG]
N/A

Amoxicillin/clavulanic acid: which time schedule do you use? [Day of VUS/VCUG]
N/A

Amoxicillin/clavulanic acid: which time schedule do you use? [Day(s) after VUS/VCUG]
N/A

Other: which time schedule do you use? [Day(s) before VUS/VCUG, how many?]
N/A

Other: which time schedule do you use? [Day of VUS/VCUG]
N/A

Other: which time schedule do you use? [Day(s) after VUS/VCUG, how many?]
N/A

Indication for prophylaxis

Which patients receive periinterventional antibiotic prophylaxis? (multiple answers possible) [all]
Yes

Which patients receive periinterventional antibiotic prophylaxis? (multiple answers possible) [patients post urinary tract infection/pyelonephritis/urosepsis]
N/A

Which patients receive periinterventional antibiotic prophylaxis? (multiple answers possible) [patients with urinary tract dilation in ultrasound]
N/A

Which patients receive periinterventional antibiotic prophylaxis? (multiple answers possible) [patients with prenatal urinary tract dilation]
N/A

Which patients receive periinterventional antibiotic prophylaxis? (multiple answers possible) [patients post VUR surgery]
N/A

Which patients receive periinterventional antibiotic prophylaxis? (multiple answers possible) [none]
N/A

Are there situations or clinical findings in the patient where you deviate from your prophylaxis standard (medication, dose, duration, age of the patient)? (multiple answers possible, please specify in answer box) [in case of already known vesicoureteral reflux (VUR)]
Yes

Are there situations or clinical findings in the patient where you deviate from your prophylaxis standard (medication, dose, duration, age of the patient)? (multiple answers possible, please specify in answer box) [in case of already known vesicoureteral reflux (VUR)][Comment]

Are there situations or clinical findings in the patient where you deviate from your prophylaxis standard (medication, dose, duration, age of the patient)? (multiple answers possible, please specify in answer box) [in patients with recurrent urinary tract infections (UTI)]
No

Are there situations or clinical findings in the patient where you deviate from your prophylaxis standard (medication, dose, duration, age of the patient)? (multiple answers possible, please specify in answer box) [in patients with recurrent urinary tract infections (UTI)][Comment]

Are there situations or clinical findings in the patient where you deviate from your prophylaxis standard (medication, dose, duration, age of the patient)? (multiple answers possible, please specify in answer box) [if multidrug-resistant organisms (MDRO) are detected in urine cultures]
No

Are there situations or clinical findings in the patient where you deviate from your prophylaxis standard (medication, dose, duration, age of the patient)? (multiple answers possible, please specify in answer box) [if multidrug-resistant organisms (MDRO) are detected in urine cultures][Comment]

Are there situations or clinical findings in the patient where you deviate from your prophylaxis standard (medication, dose, duration, age of the patient)? (multiple answers possible, please specify in answer box) [in newborns]
No

Are there situations or clinical findings in the patient where you deviate from your prophylaxis standard (medication, dose, duration, age of the patient)? (multiple answers possible, please specify in answer box) [in newborns][Comment]

Are there situations or clinical findings in the patient where you deviate from your prophylaxis standard (medication, dose, duration, age of the patient)? (multiple answers possible, please specify in answer box) [Other]

Are there situations or clinical findings in the patient where you deviate from your prophylaxis standard (medication, dose, duration, age of the patient)? (multiple answers possible, please specify in answer box) [Other comment]

Are there situations in which you use intravenous prophylaxis?
No

Voiding analysis

Are there differences in antibiotic prophylaxis between VUS and VCUG in your department?
N/A

Is a non-pathological urine strip test required in preparation for VUS/VCUG in your department?
N/A

Is a non-pathological urine culture required in preparation for VUS/VCUG in your department?
N/A

Do patients only receive post-procedure prophylaxis if vesicoureteral reflux (VUR) is detected in VUS/VCUG?
N/A

Do other MUS/MCU findings (e.g. urethral valves) have an influence on post-procedure prophylaxis?
N/A

End of survey

May we contact you personally if we have further questions?
N/A

Survey response 185

Response ID
187

Last page
1

Start language
en

Seed
1346996368

Date started
2025-03-25 16:34:02

Date last action
2025-03-25 16:34:48

Standard Operation Procedure

Who fills out this survey?
Pediatric Radiologist

Who fills out this survey? [Other]

In which type of hospital are you employed?
Other

In which type of hospital are you employed? [Other]
Pediatric private hospital

In which country do you work?
Greece

Does your department have a defined protocol for infection prophylaxis in the context of VUS/VCUG?
Yes

Has this protocol been transformed into a written standard operating procedure (SOP)?
No

Antibiotic Prophylaxis

Are there situations/constellations in which you do not use antibiotic prophylaxis for VUS/VCUG?
N/A

Please specify these situations/constellations: [girl with first uncomplicated UTI without urinary tract dilation in ultrasound]
N/A

Please specify these situations/constellations: [boy with first uncomplicated UTI without urinary tract dilation in ultrasound]
N/A

Please specify these situations/constellations: [in patients with low grade urinary tract dilation (up to 2nd grade) and no UTI in medical history]
N/A

If you use antibiotic prophylaxis: which mediaction do you use? (multiple answers and subdivision into age groups possible) [Trimethoprim]
N/A

If you use antibiotic prophylaxis: which mediaction do you use? (multiple answers and subdivision into age groups possible) [Nitrofurantoin]
N/A

If you use antibiotic prophylaxis: which mediaction do you use? (multiple answers and subdivision into age groups possible) [Amoxicillin]
N/A

If you use antibiotic prophylaxis: which mediaction do you use? (multiple answers and subdivision into age groups possible) [Cefaclor]
N/A

If you use antibiotic prophylaxis: which mediaction do you use? (multiple answers and subdivision into age groups possible) [Amoxicillin/clavulanic acid]
N/A

If you use antibiotic prophylaxis: which mediaction do you use? (multiple answers and subdivision into age groups possible) [Other]
N/A

Trimethoprim: which time schedule do you use? [Day(s) before VUS/VCUG, how many?]
N/A

Trimethoprim: which time schedule do you use? [Day of VUS/VCUG]
N/A

Trimethoprim: which time schedule do you use? [Day(s) after VUS/VCUG, how many?]
N/A

Nitrofurantoin: which time schedule do you use? [Day(s) before VUS/VCUG, how many?]
N/A

Nitrofurantoin: which time schedule do you use? [Day of VUS/VCUG]
N/A

Nitrofurantoin: which time schedule do you use? [Day(s) after VUS/VCUG, how many?]
N/A

Amoxicillin: which time schedule do you use? [Day(s) before VUS/VCUG, how many?]
N/A

Amoxicillin: which time schedule do you use? [Day of VUS/VCUG]
N/A

Amoxicillin: which time schedule do you use? [Day(s) after VUS/VCUG, how many?]
N/A

Cefaclor: which time schedule do you use? [Days(s) before VUS/VCUG, how many?]
N/A

Cefaclor: which time schedule do you use? [Day of VUS/VCUG]
N/A

Cefaclor: which time schedule do you use? [Day(s) after VUS/VCUG, how many?]
N/A

Amoxicillin/clavulanic acid: in what dosage? (Amoxicillin portion) [prophylaktic (10-20 mg/Kg bodyweight/d)]
N/A

Amoxicillin/clavulanic acid: in what dosage? (Amoxicillin portion) [double prophylactic (20-40 mg/Kg bodyweight/d)]
N/A

Amoxicillin/clavulanic acid: in what dosage? (Amoxicillin portion) [therapeutic (80-100 mg/Kg bodyweight/d)]
N/A

Amoxicillin/clavulanic acid: which time schedule do you use? [Day(s) before VUS/VCUG]
N/A

Amoxicillin/clavulanic acid: which time schedule do you use? [Day of VUS/VCUG]
N/A

Amoxicillin/clavulanic acid: which time schedule do you use? [Day(s) after VUS/VCUG]
N/A

Other: which time schedule do you use? [Day(s) before VUS/VCUG, how many?]
N/A

Other: which time schedule do you use? [Day of VUS/VCUG]
N/A

Other: which time schedule do you use? [Day(s) after VUS/VCUG, how many?]
N/A

Indication for prophylaxis

Which patients receive periinterventional antibiotic prophylaxis? (multiple answers possible) [all]
N/A

Which patients receive periinterventional antibiotic prophylaxis? (multiple answers possible) [patients post urinary tract infection/pyelonephritis/urosepsis]
N/A

Which patients receive periinterventional antibiotic prophylaxis? (multiple answers possible) [patients with urinary tract dilation in ultrasound]
N/A

Which patients receive periinterventional antibiotic prophylaxis? (multiple answers possible) [patients with prenatal urinary tract dilation]
N/A

Which patients receive periinterventional antibiotic prophylaxis? (multiple answers possible) [patients post VUR surgery]
N/A

Which patients receive periinterventional antibiotic prophylaxis? (multiple answers possible) [none]
N/A

Are there situations or clinical findings in the patient where you deviate from your prophylaxis standard (medication, dose, duration, age of the patient)? (multiple answers possible, please specify in answer box) [in case of already known vesicoureteral reflux (VUR)]
N/A

Are there situations or clinical findings in the patient where you deviate from your prophylaxis standard (medication, dose, duration, age of the patient)? (multiple answers possible, please specify in answer box) [in patients with recurrent urinary tract infections (UTI)]
N/A

Are there situations or clinical findings in the patient where you deviate from your prophylaxis standard (medication, dose, duration, age of the patient)? (multiple answers possible, please specify in answer box) [if multidrug-resistant organisms (MDRO) are detected in urine cultures]
N/A

Are there situations or clinical findings in the patient where you deviate from your prophylaxis standard (medication, dose, duration, age of the patient)? (multiple answers possible, please specify in answer box) [in newborns]
N/A

Are there situations in which you use intravenous prophylaxis?
N/A

Voiding analysis

Are there differences in antibiotic prophylaxis between VUS and VCUG in your department?
N/A

Is a non-pathological urine strip test required in preparation for VUS/VCUG in your department?
N/A

Is a non-pathological urine culture required in preparation for VUS/VCUG in your department?
N/A

Do patients only receive post-procedure prophylaxis if vesicoureteral reflux (VUR) is detected in VUS/VCUG?
N/A

Do other MUS/MCU findings (e.g. urethral valves) have an influence on post-procedure prophylaxis?
N/A

End of survey

May we contact you personally if we have further questions?
N/A

Survey response 186

Response ID
188

Date submitted
2025-03-25 16:52:06

Last page
5

Start language
de

Seed
738146875

Date started
2025-03-25 16:49:30

Date last action
2025-03-25 16:52:06

Standard Operation Procedure

Who fills out this survey?
Pediatric Radiologist

Who fills out this survey? [Other]

In which type of hospital are you employed?
Community hospital (>50 beds for pediatric patients)

In which type of hospital are you employed? [Other]

In which country do you work?
Deutschland

Does your department have a defined protocol for infection prophylaxis in the context of VUS/VCUG?
No

Has this protocol been transformed into a written standard operating procedure (SOP)?
N/A

Antibiotic Prophylaxis

Are there situations/constellations in which you do not use antibiotic prophylaxis for VUS/VCUG?
No

Please specify these situations/constellations: [girl with first uncomplicated UTI without urinary tract dilation in ultrasound]
N/A

Please specify these situations/constellations: [boy with first uncomplicated UTI without urinary tract dilation in ultrasound]
N/A

Please specify these situations/constellations: [in patients with low grade urinary tract dilation (up to 2nd grade) and no UTI in medical history]
N/A

If you use antibiotic prophylaxis: which mediaction do you use? (multiple answers and subdivision into age groups possible) [Trimethoprim]
No

If you use antibiotic prophylaxis: which mediaction do you use? (multiple answers and subdivision into age groups possible) [Nitrofurantoin]
No

If you use antibiotic prophylaxis: which mediaction do you use? (multiple answers and subdivision into age groups possible) [Amoxicillin]
No

If you use antibiotic prophylaxis: which mediaction do you use? (multiple answers and subdivision into age groups possible) [Cefaclor]
No

If you use antibiotic prophylaxis: which mediaction do you use? (multiple answers and subdivision into age groups possible) [Amoxicillin/clavulanic acid]
No

If you use antibiotic prophylaxis: which mediaction do you use? (multiple answers and subdivision into age groups possible) [Other]
No

Trimethoprim: which time schedule do you use? [Day(s) before VUS/VCUG, how many?]
N/A

Trimethoprim: which time schedule do you use? [Day of VUS/VCUG]
N/A

Trimethoprim: which time schedule do you use? [Day(s) after VUS/VCUG, how many?]
N/A

Nitrofurantoin: which time schedule do you use? [Day(s) before VUS/VCUG, how many?]
N/A

Nitrofurantoin: which time schedule do you use? [Day of VUS/VCUG]
N/A

Nitrofurantoin: which time schedule do you use? [Day(s) after VUS/VCUG, how many?]
N/A

Amoxicillin: which time schedule do you use? [Day(s) before VUS/VCUG, how many?]
N/A

Amoxicillin: which time schedule do you use? [Day of VUS/VCUG]
N/A

Amoxicillin: which time schedule do you use? [Day(s) after VUS/VCUG, how many?]
N/A

Cefaclor: which time schedule do you use? [Days(s) before VUS/VCUG, how many?]
N/A

Cefaclor: which time schedule do you use? [Day of VUS/VCUG]
N/A

Cefaclor: which time schedule do you use? [Day(s) after VUS/VCUG, how many?]
N/A

Amoxicillin/clavulanic acid: in what dosage? (Amoxicillin portion) [prophylaktic (10-20 mg/Kg bodyweight/d)]
N/A

Amoxicillin/clavulanic acid: in what dosage? (Amoxicillin portion) [double prophylactic (20-40 mg/Kg bodyweight/d)]
N/A

Amoxicillin/clavulanic acid: in what dosage? (Amoxicillin portion) [therapeutic (80-100 mg/Kg bodyweight/d)]
N/A

Amoxicillin/clavulanic acid: which time schedule do you use? [Day(s) before VUS/VCUG]
N/A

Amoxicillin/clavulanic acid: which time schedule do you use? [Day of VUS/VCUG]
N/A

Amoxicillin/clavulanic acid: which time schedule do you use? [Day(s) after VUS/VCUG]
N/A

Other: which time schedule do you use? [Day(s) before VUS/VCUG, how many?]
N/A

Other: which time schedule do you use? [Day of VUS/VCUG]
N/A

Other: which time schedule do you use? [Day(s) after VUS/VCUG, how many?]
N/A

Indication for prophylaxis

Which patients receive periinterventional antibiotic prophylaxis? (multiple answers possible) [all]
Yes

Which patients receive periinterventional antibiotic prophylaxis? (multiple answers possible) [patients post urinary tract infection/pyelonephritis/urosepsis]
N/A

Which patients receive periinterventional antibiotic prophylaxis? (multiple answers possible) [patients with urinary tract dilation in ultrasound]
N/A

Which patients receive periinterventional antibiotic prophylaxis? (multiple answers possible) [patients with prenatal urinary tract dilation]
N/A

Which patients receive periinterventional antibiotic prophylaxis? (multiple answers possible) [patients post VUR surgery]
N/A

Which patients receive periinterventional antibiotic prophylaxis? (multiple answers possible) [none]
N/A

Are there situations or clinical findings in the patient where you deviate from your prophylaxis standard (medication, dose, duration, age of the patient)? (multiple answers possible, please specify in answer box) [in case of already known vesicoureteral reflux (VUR)]
No

Are there situations or clinical findings in the patient where you deviate from your prophylaxis standard (medication, dose, duration, age of the patient)? (multiple answers possible, please specify in answer box) [in case of already known vesicoureteral reflux (VUR)][Comment]

Are there situations or clinical findings in the patient where you deviate from your prophylaxis standard (medication, dose, duration, age of the patient)? (multiple answers possible, please specify in answer box) [in patients with recurrent urinary tract infections (UTI)]
No

Are there situations or clinical findings in the patient where you deviate from your prophylaxis standard (medication, dose, duration, age of the patient)? (multiple answers possible, please specify in answer box) [in patients with recurrent urinary tract infections (UTI)][Comment]

Are there situations or clinical findings in the patient where you deviate from your prophylaxis standard (medication, dose, duration, age of the patient)? (multiple answers possible, please specify in answer box) [if multidrug-resistant organisms (MDRO) are detected in urine cultures]
No

Are there situations or clinical findings in the patient where you deviate from your prophylaxis standard (medication, dose, duration, age of the patient)? (multiple answers possible, please specify in answer box) [if multidrug-resistant organisms (MDRO) are detected in urine cultures][Comment]

Are there situations or clinical findings in the patient where you deviate from your prophylaxis standard (medication, dose, duration, age of the patient)? (multiple answers possible, please specify in answer box) [in newborns]
No

Are there situations or clinical findings in the patient where you deviate from your prophylaxis standard (medication, dose, duration, age of the patient)? (multiple answers possible, please specify in answer box) [in newborns][Comment]

Are there situations or clinical findings in the patient where you deviate from your prophylaxis standard (medication, dose, duration, age of the patient)? (multiple answers possible, please specify in answer box) [Other]
Keine

Are there situations or clinical findings in the patient where you deviate from your prophylaxis standard (medication, dose, duration, age of the patient)? (multiple answers possible, please specify in answer box) [Other comment]

Are there situations in which you use intravenous prophylaxis?
No

Voiding analysis

Are there differences in antibiotic prophylaxis between VUS and VCUG in your department?
No

Is a non-pathological urine strip test required in preparation for VUS/VCUG in your department?
No

Is a non-pathological urine culture required in preparation for VUS/VCUG in your department?
No

Do patients only receive post-procedure prophylaxis if vesicoureteral reflux (VUR) is detected in VUS/VCUG?
No

Do other MUS/MCU findings (e.g. urethral valves) have an influence on post-procedure prophylaxis?
No

End of survey

Thank you for taking the time to answer this survey!

May we contact you personally if we have further questions?
No

Survey response 187

Response ID
189

Date submitted
2025-03-25 17:04:12

Last page
5

Start language
en

Seed
367377206

Date started
2025-03-25 16:57:53

Date last action
2025-03-25 17:04:12

Standard Operation Procedure

Who fills out this survey?
Pediatric Radiologist

Who fills out this survey? [Other]

In which type of hospital are you employed?
University Hospital

In which type of hospital are you employed? [Other]

In which country do you work?
United Arab Emirates

Does your department have a defined protocol for infection prophylaxis in the context of VUS/VCUG?
Yes

Has this protocol been transformed into a written standard operating procedure (SOP)?
No

Antibiotic Prophylaxis

Are there situations/constellations in which you do not use antibiotic prophylaxis for VUS/VCUG?
No

Please specify these situations/constellations: [girl with first uncomplicated UTI without urinary tract dilation in ultrasound]
N/A

Please specify these situations/constellations: [boy with first uncomplicated UTI without urinary tract dilation in ultrasound]
N/A

Please specify these situations/constellations: [in patients with low grade urinary tract dilation (up to 2nd grade) and no UTI in medical history]
N/A

If you use antibiotic prophylaxis: which mediaction do you use? (multiple answers and subdivision into age groups possible) [Trimethoprim]
Yes

If you use antibiotic prophylaxis: which mediaction do you use? (multiple answers and subdivision into age groups possible) [Nitrofurantoin]
No

If you use antibiotic prophylaxis: which mediaction do you use? (multiple answers and subdivision into age groups possible) [Amoxicillin]
No

If you use antibiotic prophylaxis: which mediaction do you use? (multiple answers and subdivision into age groups possible) [Cefaclor]
No

If you use antibiotic prophylaxis: which mediaction do you use? (multiple answers and subdivision into age groups possible) [Amoxicillin/clavulanic acid]
No

If you use antibiotic prophylaxis: which mediaction do you use? (multiple answers and subdivision into age groups possible) [Other]
No

Trimethoprim: in what dosage?
double prophylakctic (4 mg/kg bodyweight/d)

Trimethoprim: in what dosage? [Other]

Trimethoprim: for which age group do you use trimethoprim?
No specific age group

Trimethoprim: which time schedule do you use? [Day(s) before VUS/VCUG, how many?]
Yes

Trimethoprim: which time schedule do you use? [Day(s) before VUS/VCUG, how many?][Comment]
One

Trimethoprim: which time schedule do you use? [Day of VUS/VCUG]
Yes

Trimethoprim: which time schedule do you use? [Day of VUS/VCUG][Comment]

Trimethoprim: which time schedule do you use? [Day(s) after VUS/VCUG, how many?]
Yes

Trimethoprim: which time schedule do you use? [Day(s) after VUS/VCUG, how many?][Comment]
One

Nitrofurantoin: which time schedule do you use? [Day(s) before VUS/VCUG, how many?]
N/A

Nitrofurantoin: which time schedule do you use? [Day of VUS/VCUG]
N/A

Nitrofurantoin: which time schedule do you use? [Day(s) after VUS/VCUG, how many?]
N/A

Amoxicillin: which time schedule do you use? [Day(s) before VUS/VCUG, how many?]
N/A

Amoxicillin: which time schedule do you use? [Day of VUS/VCUG]
N/A

Amoxicillin: which time schedule do you use? [Day(s) after VUS/VCUG, how many?]
N/A

Cefaclor: which time schedule do you use? [Days(s) before VUS/VCUG, how many?]
N/A

Cefaclor: which time schedule do you use? [Day of VUS/VCUG]
N/A

Cefaclor: which time schedule do you use? [Day(s) after VUS/VCUG, how many?]
N/A

Amoxicillin/clavulanic acid: in what dosage? (Amoxicillin portion) [prophylaktic (10-20 mg/Kg bodyweight/d)]
N/A

Amoxicillin/clavulanic acid: in what dosage? (Amoxicillin portion) [double prophylactic (20-40 mg/Kg bodyweight/d)]
N/A

Amoxicillin/clavulanic acid: in what dosage? (Amoxicillin portion) [therapeutic (80-100 mg/Kg bodyweight/d)]
N/A

Amoxicillin/clavulanic acid: which time schedule do you use? [Day(s) before VUS/VCUG]
N/A

Amoxicillin/clavulanic acid: which time schedule do you use? [Day of VUS/VCUG]
N/A

Amoxicillin/clavulanic acid: which time schedule do you use? [Day(s) after VUS/VCUG]
N/A

Other: which time schedule do you use? [Day(s) before VUS/VCUG, how many?]
N/A

Other: which time schedule do you use? [Day of VUS/VCUG]
N/A

Other: which time schedule do you use? [Day(s) after VUS/VCUG, how many?]
N/A

Indication for prophylaxis

Which patients receive periinterventional antibiotic prophylaxis? (multiple answers possible) [all]
Yes

Which patients receive periinterventional antibiotic prophylaxis? (multiple answers possible) [patients post urinary tract infection/pyelonephritis/urosepsis]
N/A

Which patients receive periinterventional antibiotic prophylaxis? (multiple answers possible) [patients with urinary tract dilation in ultrasound]
N/A

Which patients receive periinterventional antibiotic prophylaxis? (multiple answers possible) [patients with prenatal urinary tract dilation]
N/A

Which patients receive periinterventional antibiotic prophylaxis? (multiple answers possible) [patients post VUR surgery]
N/A

Which patients receive periinterventional antibiotic prophylaxis? (multiple answers possible) [none]
N/A

Are there situations or clinical findings in the patient where you deviate from your prophylaxis standard (medication, dose, duration, age of the patient)? (multiple answers possible, please specify in answer box) [in case of already known vesicoureteral reflux (VUR)]
No

Are there situations or clinical findings in the patient where you deviate from your prophylaxis standard (medication, dose, duration, age of the patient)? (multiple answers possible, please specify in answer box) [in case of already known vesicoureteral reflux (VUR)][Comment]

Are there situations or clinical findings in the patient where you deviate from your prophylaxis standard (medication, dose, duration, age of the patient)? (multiple answers possible, please specify in answer box) [in patients with recurrent urinary tract infections (UTI)]
No

Are there situations or clinical findings in the patient where you deviate from your prophylaxis standard (medication, dose, duration, age of the patient)? (multiple answers possible, please specify in answer box) [in patients with recurrent urinary tract infections (UTI)][Comment]

Are there situations or clinical findings in the patient where you deviate from your prophylaxis standard (medication, dose, duration, age of the patient)? (multiple answers possible, please specify in answer box) [if multidrug-resistant organisms (MDRO) are detected in urine cultures]
No

Are there situations or clinical findings in the patient where you deviate from your prophylaxis standard (medication, dose, duration, age of the patient)? (multiple answers possible, please specify in answer box) [if multidrug-resistant organisms (MDRO) are detected in urine cultures][Comment]

Are there situations or clinical findings in the patient where you deviate from your prophylaxis standard (medication, dose, duration, age of the patient)? (multiple answers possible, please specify in answer box) [in newborns]
Yes

Are there situations or clinical findings in the patient where you deviate from your prophylaxis standard (medication, dose, duration, age of the patient)? (multiple answers possible, please specify in answer box) [in newborns][Comment]
A negative urine examination

Are there situations or clinical findings in the patient where you deviate from your prophylaxis standard (medication, dose, duration, age of the patient)? (multiple answers possible, please specify in answer box) [Other]

Are there situations or clinical findings in the patient where you deviate from your prophylaxis standard (medication, dose, duration, age of the patient)? (multiple answers possible, please specify in answer box) [Other comment]

Are there situations in which you use intravenous prophylaxis?
No

Voiding analysis

Are there differences in antibiotic prophylaxis between VUS and VCUG in your department?
No

Is a non-pathological urine strip test required in preparation for VUS/VCUG in your department?
No

Is a non-pathological urine culture required in preparation for VUS/VCUG in your department?
Yes

What is the maximum amount of days accepted for urine culture to be performed prior to the exam?
Seven

Do patients only receive post-procedure prophylaxis if vesicoureteral reflux (VUR) is detected in VUS/VCUG?
No

Do other MUS/MCU findings (e.g. urethral valves) have an influence on post-procedure prophylaxis?
No

End of survey

Thank you for taking the time to answer this survey!

May we contact you personally if we have further questions?
Yes

E-mail address:
APDsouza@dubaihealth.ae

Survey response 188

Response ID
190

Last page
0

Start language
de

Seed
1940188754

Date started
2025-03-25 17:52:33

Date last action
2025-03-25 17:52:33

Standard Operation Procedure

Does your department have a defined protocol for infection prophylaxis in the context of VUS/VCUG?
N/A

Has this protocol been transformed into a written standard operating procedure (SOP)?
N/A

Antibiotic Prophylaxis

Are there situations/constellations in which you do not use antibiotic prophylaxis for VUS/VCUG?
N/A

Please specify these situations/constellations: [girl with first uncomplicated UTI without urinary tract dilation in ultrasound]
N/A

Please specify these situations/constellations: [boy with first uncomplicated UTI without urinary tract dilation in ultrasound]
N/A

Please specify these situations/constellations: [in patients with low grade urinary tract dilation (up to 2nd grade) and no UTI in medical history]
N/A

If you use antibiotic prophylaxis: which mediaction do you use? (multiple answers and subdivision into age groups possible) [Trimethoprim]
N/A

If you use antibiotic prophylaxis: which mediaction do you use? (multiple answers and subdivision into age groups possible) [Nitrofurantoin]
N/A

If you use antibiotic prophylaxis: which mediaction do you use? (multiple answers and subdivision into age groups possible) [Amoxicillin]
N/A

If you use antibiotic prophylaxis: which mediaction do you use? (multiple answers and subdivision into age groups possible) [Cefaclor]
N/A

If you use antibiotic prophylaxis: which mediaction do you use? (multiple answers and subdivision into age groups possible) [Amoxicillin/clavulanic acid]
N/A

If you use antibiotic prophylaxis: which mediaction do you use? (multiple answers and subdivision into age groups possible) [Other]
N/A

Trimethoprim: which time schedule do you use? [Day(s) before VUS/VCUG, how many?]
N/A

Trimethoprim: which time schedule do you use? [Day of VUS/VCUG]
N/A

Trimethoprim: which time schedule do you use? [Day(s) after VUS/VCUG, how many?]
N/A

Nitrofurantoin: which time schedule do you use? [Day(s) before VUS/VCUG, how many?]
N/A

Nitrofurantoin: which time schedule do you use? [Day of VUS/VCUG]
N/A

Nitrofurantoin: which time schedule do you use? [Day(s) after VUS/VCUG, how many?]
N/A

Amoxicillin: which time schedule do you use? [Day(s) before VUS/VCUG, how many?]
N/A

Amoxicillin: which time schedule do you use? [Day of VUS/VCUG]
N/A

Amoxicillin: which time schedule do you use? [Day(s) after VUS/VCUG, how many?]
N/A

Cefaclor: which time schedule do you use? [Days(s) before VUS/VCUG, how many?]
N/A

Cefaclor: which time schedule do you use? [Day of VUS/VCUG]
N/A

Cefaclor: which time schedule do you use? [Day(s) after VUS/VCUG, how many?]
N/A

Amoxicillin/clavulanic acid: in what dosage? (Amoxicillin portion) [prophylaktic (10-20 mg/Kg bodyweight/d)]
N/A

Amoxicillin/clavulanic acid: in what dosage? (Amoxicillin portion) [double prophylactic (20-40 mg/Kg bodyweight/d)]
N/A

Amoxicillin/clavulanic acid: in what dosage? (Amoxicillin portion) [therapeutic (80-100 mg/Kg bodyweight/d)]
N/A

Amoxicillin/clavulanic acid: which time schedule do you use? [Day(s) before VUS/VCUG]
N/A

Amoxicillin/clavulanic acid: which time schedule do you use? [Day of VUS/VCUG]
N/A

Amoxicillin/clavulanic acid: which time schedule do you use? [Day(s) after VUS/VCUG]
N/A

Other: which time schedule do you use? [Day(s) before VUS/VCUG, how many?]
N/A

Other: which time schedule do you use? [Day of VUS/VCUG]
N/A

Other: which time schedule do you use? [Day(s) after VUS/VCUG, how many?]
N/A

Indication for prophylaxis

Which patients receive periinterventional antibiotic prophylaxis? (multiple answers possible) [all]
N/A

Which patients receive periinterventional antibiotic prophylaxis? (multiple answers possible) [patients post urinary tract infection/pyelonephritis/urosepsis]
N/A

Which patients receive periinterventional antibiotic prophylaxis? (multiple answers possible) [patients with urinary tract dilation in ultrasound]
N/A

Which patients receive periinterventional antibiotic prophylaxis? (multiple answers possible) [patients with prenatal urinary tract dilation]
N/A

Which patients receive periinterventional antibiotic prophylaxis? (multiple answers possible) [patients post VUR surgery]
N/A

Which patients receive periinterventional antibiotic prophylaxis? (multiple answers possible) [none]
N/A

Are there situations or clinical findings in the patient where you deviate from your prophylaxis standard (medication, dose, duration, age of the patient)? (multiple answers possible, please specify in answer box) [in case of already known vesicoureteral reflux (VUR)]
N/A

Are there situations or clinical findings in the patient where you deviate from your prophylaxis standard (medication, dose, duration, age of the patient)? (multiple answers possible, please specify in answer box) [in patients with recurrent urinary tract infections (UTI)]
N/A

Are there situations or clinical findings in the patient where you deviate from your prophylaxis standard (medication, dose, duration, age of the patient)? (multiple answers possible, please specify in answer box) [if multidrug-resistant organisms (MDRO) are detected in urine cultures]
N/A

Are there situations or clinical findings in the patient where you deviate from your prophylaxis standard (medication, dose, duration, age of the patient)? (multiple answers possible, please specify in answer box) [in newborns]
N/A

Are there situations in which you use intravenous prophylaxis?
N/A

Voiding analysis

Are there differences in antibiotic prophylaxis between VUS and VCUG in your department?
N/A

Is a non-pathological urine strip test required in preparation for VUS/VCUG in your department?
N/A

Is a non-pathological urine culture required in preparation for VUS/VCUG in your department?
N/A

Do patients only receive post-procedure prophylaxis if vesicoureteral reflux (VUR) is detected in VUS/VCUG?
N/A

Do other MUS/MCU findings (e.g. urethral valves) have an influence on post-procedure prophylaxis?
N/A

End of survey

May we contact you personally if we have further questions?
N/A

Survey response 189

Response ID
191

Date submitted
2025-03-25 18:40:06

Last page
5

Start language
en

Seed
488572820

Date started
2025-03-25 18:28:43

Date last action
2025-03-25 18:40:06

Standard Operation Procedure

Who fills out this survey?
Pediatric Radiologist

Who fills out this survey? [Other]

In which type of hospital are you employed?
Community hospital (>50 beds for pediatric patients)

In which type of hospital are you employed? [Other]

In which country do you work?
GREECE

Does your department have a defined protocol for infection prophylaxis in the context of VUS/VCUG?
Yes

Has this protocol been transformed into a written standard operating procedure (SOP)?
No

Antibiotic Prophylaxis

Are there situations/constellations in which you do not use antibiotic prophylaxis for VUS/VCUG?
No

Please specify these situations/constellations: [girl with first uncomplicated UTI without urinary tract dilation in ultrasound]
N/A

Please specify these situations/constellations: [boy with first uncomplicated UTI without urinary tract dilation in ultrasound]
N/A

Please specify these situations/constellations: [in patients with low grade urinary tract dilation (up to 2nd grade) and no UTI in medical history]
N/A

If you use antibiotic prophylaxis: which mediaction do you use? (multiple answers and subdivision into age groups possible) [Trimethoprim]
No

If you use antibiotic prophylaxis: which mediaction do you use? (multiple answers and subdivision into age groups possible) [Nitrofurantoin]
No

If you use antibiotic prophylaxis: which mediaction do you use? (multiple answers and subdivision into age groups possible) [Amoxicillin]
No

If you use antibiotic prophylaxis: which mediaction do you use? (multiple answers and subdivision into age groups possible) [Cefaclor]
No

If you use antibiotic prophylaxis: which mediaction do you use? (multiple answers and subdivision into age groups possible) [Amoxicillin/clavulanic acid]
No

If you use antibiotic prophylaxis: which mediaction do you use? (multiple answers and subdivision into age groups possible) [Other]
Yes

Trimethoprim: which time schedule do you use? [Day(s) before VUS/VCUG, how many?]
N/A

Trimethoprim: which time schedule do you use? [Day of VUS/VCUG]
N/A

Trimethoprim: which time schedule do you use? [Day(s) after VUS/VCUG, how many?]
N/A

Nitrofurantoin: which time schedule do you use? [Day(s) before VUS/VCUG, how many?]
N/A

Nitrofurantoin: which time schedule do you use? [Day of VUS/VCUG]
N/A

Nitrofurantoin: which time schedule do you use? [Day(s) after VUS/VCUG, how many?]
N/A

Amoxicillin: which time schedule do you use? [Day(s) before VUS/VCUG, how many?]
N/A

Amoxicillin: which time schedule do you use? [Day of VUS/VCUG]
N/A

Amoxicillin: which time schedule do you use? [Day(s) after VUS/VCUG, how many?]
N/A

Cefaclor: which time schedule do you use? [Days(s) before VUS/VCUG, how many?]
N/A

Cefaclor: which time schedule do you use? [Day of VUS/VCUG]
N/A

Cefaclor: which time schedule do you use? [Day(s) after VUS/VCUG, how many?]
N/A

Amoxicillin/clavulanic acid: in what dosage? (Amoxicillin portion) [prophylaktic (10-20 mg/Kg bodyweight/d)]
N/A

Amoxicillin/clavulanic acid: in what dosage? (Amoxicillin portion) [double prophylactic (20-40 mg/Kg bodyweight/d)]
N/A

Amoxicillin/clavulanic acid: in what dosage? (Amoxicillin portion) [therapeutic (80-100 mg/Kg bodyweight/d)]
N/A

Amoxicillin/clavulanic acid: which time schedule do you use? [Day(s) before VUS/VCUG]
N/A

Amoxicillin/clavulanic acid: which time schedule do you use? [Day of VUS/VCUG]
N/A

Amoxicillin/clavulanic acid: which time schedule do you use? [Day(s) after VUS/VCUG]
N/A

Other: which medication and in what dosage?
I ask parents/caregivers to communicate with their pediatrician who is able to prescribe the medication.

Other: for which age group do you use this medication?
All

Other: which time schedule do you use? [Day(s) before VUS/VCUG, how many?]
No

Other: which time schedule do you use? [Day(s) before VUS/VCUG, how many?][Comment]

Other: which time schedule do you use? [Day of VUS/VCUG]
Yes

Other: which time schedule do you use? [Day of VUS/VCUG][Comment]

Other: which time schedule do you use? [Day(s) after VUS/VCUG, how many?]
Yes

Other: which time schedule do you use? [Day(s) after VUS/VCUG, how many?][Comment]
2

Indication for prophylaxis

Which patients receive periinterventional antibiotic prophylaxis? (multiple answers possible) [all]
Yes

Which patients receive periinterventional antibiotic prophylaxis? (multiple answers possible) [patients post urinary tract infection/pyelonephritis/urosepsis]
N/A

Which patients receive periinterventional antibiotic prophylaxis? (multiple answers possible) [patients with urinary tract dilation in ultrasound]
N/A

Which patients receive periinterventional antibiotic prophylaxis? (multiple answers possible) [patients with prenatal urinary tract dilation]
N/A

Which patients receive periinterventional antibiotic prophylaxis? (multiple answers possible) [patients post VUR surgery]
N/A

Which patients receive periinterventional antibiotic prophylaxis? (multiple answers possible) [none]
N/A

Are there situations or clinical findings in the patient where you deviate from your prophylaxis standard (medication, dose, duration, age of the patient)? (multiple answers possible, please specify in answer box) [in case of already known vesicoureteral reflux (VUR)]
No

Are there situations or clinical findings in the patient where you deviate from your prophylaxis standard (medication, dose, duration, age of the patient)? (multiple answers possible, please specify in answer box) [in case of already known vesicoureteral reflux (VUR)][Comment]

Are there situations or clinical findings in the patient where you deviate from your prophylaxis standard (medication, dose, duration, age of the patient)? (multiple answers possible, please specify in answer box) [in patients with recurrent urinary tract infections (UTI)]
No

Are there situations or clinical findings in the patient where you deviate from your prophylaxis standard (medication, dose, duration, age of the patient)? (multiple answers possible, please specify in answer box) [in patients with recurrent urinary tract infections (UTI)][Comment]

Are there situations or clinical findings in the patient where you deviate from your prophylaxis standard (medication, dose, duration, age of the patient)? (multiple answers possible, please specify in answer box) [if multidrug-resistant organisms (MDRO) are detected in urine cultures]
No

Are there situations or clinical findings in the patient where you deviate from your prophylaxis standard (medication, dose, duration, age of the patient)? (multiple answers possible, please specify in answer box) [if multidrug-resistant organisms (MDRO) are detected in urine cultures][Comment]

Are there situations or clinical findings in the patient where you deviate from your prophylaxis standard (medication, dose, duration, age of the patient)? (multiple answers possible, please specify in answer box) [in newborns]
No

Are there situations or clinical findings in the patient where you deviate from your prophylaxis standard (medication, dose, duration, age of the patient)? (multiple answers possible, please specify in answer box) [in newborns][Comment]

Are there situations or clinical findings in the patient where you deviate from your prophylaxis standard (medication, dose, duration, age of the patient)? (multiple answers possible, please specify in answer box) [Other]
Laborious catheterization

Are there situations or clinical findings in the patient where you deviate from your prophylaxis standard (medication, dose, duration, age of the patient)? (multiple answers possible, please specify in answer box) [Other comment]
Higher dose

Are there situations in which you use intravenous prophylaxis?
No

Voiding analysis

Are there differences in antibiotic prophylaxis between VUS and VCUG in your department?
No

Is a non-pathological urine strip test required in preparation for VUS/VCUG in your department?
No

Is a non-pathological urine culture required in preparation for VUS/VCUG in your department?
Yes

What is the maximum amount of days accepted for urine culture to be performed prior to the exam?
5 days from the test, 3 days from the results

Do patients only receive post-procedure prophylaxis if vesicoureteral reflux (VUR) is detected in VUS/VCUG?
No

Do other MUS/MCU findings (e.g. urethral valves) have an influence on post-procedure prophylaxis?
No

End of survey

Thank you for taking the time to answer this survey!

May we contact you personally if we have further questions?
Yes

E-mail address:
lenamanoli@yahoo.gr

Survey response 190

Response ID
192

Date submitted
2025-03-25 19:00:50

Last page
5

Start language
en

Seed
1281584148

Date started
2025-03-25 18:57:48

Date last action
2025-03-25 19:00:50

Standard Operation Procedure

Who fills out this survey?
Radiologist

Who fills out this survey? [Other]

In which type of hospital are you employed?
University Hospital

In which type of hospital are you employed? [Other]

In which country do you work?
netherlands

Does your department have a defined protocol for infection prophylaxis in the context of VUS/VCUG?
Yes

Has this protocol been transformed into a written standard operating procedure (SOP)?
Yes

Which department is the author of the SOP for infection prophylaxis in VUS/VCUG?
Pediatric radiology

Which department is the author of the SOP for infection prophylaxis in VUS/VCUG? [Other]

Antibiotic Prophylaxis

Are there situations/constellations in which you do not use antibiotic prophylaxis for VUS/VCUG?
No

Please specify these situations/constellations: [girl with first uncomplicated UTI without urinary tract dilation in ultrasound]
N/A

Please specify these situations/constellations: [boy with first uncomplicated UTI without urinary tract dilation in ultrasound]
N/A

Please specify these situations/constellations: [in patients with low grade urinary tract dilation (up to 2nd grade) and no UTI in medical history]
N/A

If you use antibiotic prophylaxis: which mediaction do you use? (multiple answers and subdivision into age groups possible) [Trimethoprim]
No

If you use antibiotic prophylaxis: which mediaction do you use? (multiple answers and subdivision into age groups possible) [Nitrofurantoin]
No

If you use antibiotic prophylaxis: which mediaction do you use? (multiple answers and subdivision into age groups possible) [Amoxicillin]
Yes

If you use antibiotic prophylaxis: which mediaction do you use? (multiple answers and subdivision into age groups possible) [Cefaclor]
No

If you use antibiotic prophylaxis: which mediaction do you use? (multiple answers and subdivision into age groups possible) [Amoxicillin/clavulanic acid]
No

If you use antibiotic prophylaxis: which mediaction do you use? (multiple answers and subdivision into age groups possible) [Other]
No

Trimethoprim: which time schedule do you use? [Day(s) before VUS/VCUG, how many?]
N/A

Trimethoprim: which time schedule do you use? [Day of VUS/VCUG]
N/A

Trimethoprim: which time schedule do you use? [Day(s) after VUS/VCUG, how many?]
N/A

Nitrofurantoin: which time schedule do you use? [Day(s) before VUS/VCUG, how many?]
N/A

Nitrofurantoin: which time schedule do you use? [Day of VUS/VCUG]
N/A

Nitrofurantoin: which time schedule do you use? [Day(s) after VUS/VCUG, how many?]
N/A

Amoxicillin: in what dosage?
prophylactic (10-20 mg/kg bodyweight/d)

Amoxicillin: in what dosage? [Other]

Amoxicillin: for which age group do you use amoxicillin?
all

Amoxicillin: which time schedule do you use? [Day(s) before VUS/VCUG, how many?]
Yes

Amoxicillin: which time schedule do you use? [Day(s) before VUS/VCUG, how many?][Comment]
1

Amoxicillin: which time schedule do you use? [Day of VUS/VCUG]
Yes

Amoxicillin: which time schedule do you use? [Day of VUS/VCUG][Comment]

Amoxicillin: which time schedule do you use? [Day(s) after VUS/VCUG, how many?]
Yes

Amoxicillin: which time schedule do you use? [Day(s) after VUS/VCUG, how many?][Comment]
2

Cefaclor: which time schedule do you use? [Days(s) before VUS/VCUG, how many?]
N/A

Cefaclor: which time schedule do you use? [Day of VUS/VCUG]
N/A

Cefaclor: which time schedule do you use? [Day(s) after VUS/VCUG, how many?]
N/A

Amoxicillin/clavulanic acid: in what dosage? (Amoxicillin portion) [prophylaktic (10-20 mg/Kg bodyweight/d)]
N/A

Amoxicillin/clavulanic acid: in what dosage? (Amoxicillin portion) [double prophylactic (20-40 mg/Kg bodyweight/d)]
N/A

Amoxicillin/clavulanic acid: in what dosage? (Amoxicillin portion) [therapeutic (80-100 mg/Kg bodyweight/d)]
N/A

Amoxicillin/clavulanic acid: which time schedule do you use? [Day(s) before VUS/VCUG]
N/A

Amoxicillin/clavulanic acid: which time schedule do you use? [Day of VUS/VCUG]
N/A

Amoxicillin/clavulanic acid: which time schedule do you use? [Day(s) after VUS/VCUG]
N/A

Other: which time schedule do you use? [Day(s) before VUS/VCUG, how many?]
N/A

Other: which time schedule do you use? [Day of VUS/VCUG]
N/A

Other: which time schedule do you use? [Day(s) after VUS/VCUG, how many?]
N/A

Indication for prophylaxis

Which patients receive periinterventional antibiotic prophylaxis? (multiple answers possible) [all]
Yes

Which patients receive periinterventional antibiotic prophylaxis? (multiple answers possible) [patients post urinary tract infection/pyelonephritis/urosepsis]
N/A

Which patients receive periinterventional antibiotic prophylaxis? (multiple answers possible) [patients with urinary tract dilation in ultrasound]
N/A

Which patients receive periinterventional antibiotic prophylaxis? (multiple answers possible) [patients with prenatal urinary tract dilation]
N/A

Which patients receive periinterventional antibiotic prophylaxis? (multiple answers possible) [patients post VUR surgery]
N/A

Which patients receive periinterventional antibiotic prophylaxis? (multiple answers possible) [none]
N/A

Are there situations or clinical findings in the patient where you deviate from your prophylaxis standard (medication, dose, duration, age of the patient)? (multiple answers possible, please specify in answer box) [in case of already known vesicoureteral reflux (VUR)]
No

Are there situations or clinical findings in the patient where you deviate from your prophylaxis standard (medication, dose, duration, age of the patient)? (multiple answers possible, please specify in answer box) [in case of already known vesicoureteral reflux (VUR)][Comment]

Are there situations or clinical findings in the patient where you deviate from your prophylaxis standard (medication, dose, duration, age of the patient)? (multiple answers possible, please specify in answer box) [in patients with recurrent urinary tract infections (UTI)]
No

Are there situations or clinical findings in the patient where you deviate from your prophylaxis standard (medication, dose, duration, age of the patient)? (multiple answers possible, please specify in answer box) [in patients with recurrent urinary tract infections (UTI)][Comment]

Are there situations or clinical findings in the patient where you deviate from your prophylaxis standard (medication, dose, duration, age of the patient)? (multiple answers possible, please specify in answer box) [if multidrug-resistant organisms (MDRO) are detected in urine cultures]
No

Are there situations or clinical findings in the patient where you deviate from your prophylaxis standard (medication, dose, duration, age of the patient)? (multiple answers possible, please specify in answer box) [if multidrug-resistant organisms (MDRO) are detected in urine cultures][Comment]

Are there situations or clinical findings in the patient where you deviate from your prophylaxis standard (medication, dose, duration, age of the patient)? (multiple answers possible, please specify in answer box) [in newborns]
No

Are there situations or clinical findings in the patient where you deviate from your prophylaxis standard (medication, dose, duration, age of the patient)? (multiple answers possible, please specify in answer box) [in newborns][Comment]

Are there situations or clinical findings in the patient where you deviate from your prophylaxis standard (medication, dose, duration, age of the patient)? (multiple answers possible, please specify in answer box) [Other]
non

Are there situations or clinical findings in the patient where you deviate from your prophylaxis standard (medication, dose, duration, age of the patient)? (multiple answers possible, please specify in answer box) [Other comment]
non

Are there situations in which you use intravenous prophylaxis?
No

Voiding analysis

Are there differences in antibiotic prophylaxis between VUS and VCUG in your department?
Yes

To what extent?
idk

Is a non-pathological urine strip test required in preparation for VUS/VCUG in your department?
No

Is a non-pathological urine culture required in preparation for VUS/VCUG in your department?
No

Do patients only receive post-procedure prophylaxis if vesicoureteral reflux (VUR) is detected in VUS/VCUG?
No

Do other MUS/MCU findings (e.g. urethral valves) have an influence on post-procedure prophylaxis?
No

End of survey

Thank you for taking the time to answer this survey!

May we contact you personally if we have further questions?
No

Survey response 191

Response ID
193

Date submitted
2025-03-25 21:25:11

Last page
5

Start language
en

Seed
1259666921

Date started
2025-03-25 21:13:56

Date last action
2025-03-25 21:25:11

Standard Operation Procedure

Who fills out this survey?
Pediatric Radiologist

Who fills out this survey? [Other]

In which type of hospital are you employed?
University Hospital

In which type of hospital are you employed? [Other]

In which country do you work?
Romania

Does your department have a defined protocol for infection prophylaxis in the context of VUS/VCUG?
Yes

Has this protocol been transformed into a written standard operating procedure (SOP)?
No

Antibiotic Prophylaxis

Are there situations/constellations in which you do not use antibiotic prophylaxis for VUS/VCUG?
Yes

Please specify these situations/constellations: [girl with first uncomplicated UTI without urinary tract dilation in ultrasound]
No

Please specify these situations/constellations: [boy with first uncomplicated UTI without urinary tract dilation in ultrasound]
No

Please specify these situations/constellations: [in patients with low grade urinary tract dilation (up to 2nd grade) and no UTI in medical history]
No

Please specify these situations/constellations: [Other]
We perform VCUG (no prophylaxis) if no UTI is diagnosed. If there is infection, it shoud be treated according to antibiogram at least 5 days if not cured.

If you use antibiotic prophylaxis: which mediaction do you use? (multiple answers and subdivision into age groups possible) [Trimethoprim]
No

If you use antibiotic prophylaxis: which mediaction do you use? (multiple answers and subdivision into age groups possible) [Nitrofurantoin]
No

If you use antibiotic prophylaxis: which mediaction do you use? (multiple answers and subdivision into age groups possible) [Amoxicillin]
No

If you use antibiotic prophylaxis: which mediaction do you use? (multiple answers and subdivision into age groups possible) [Cefaclor]
No

If you use antibiotic prophylaxis: which mediaction do you use? (multiple answers and subdivision into age groups possible) [Amoxicillin/clavulanic acid]
No

If you use antibiotic prophylaxis: which mediaction do you use? (multiple answers and subdivision into age groups possible) [Other]
No

Trimethoprim: which time schedule do you use? [Day(s) before VUS/VCUG, how many?]
N/A

Trimethoprim: which time schedule do you use? [Day of VUS/VCUG]
N/A

Trimethoprim: which time schedule do you use? [Day(s) after VUS/VCUG, how many?]
N/A

Nitrofurantoin: which time schedule do you use? [Day(s) before VUS/VCUG, how many?]
N/A

Nitrofurantoin: which time schedule do you use? [Day of VUS/VCUG]
N/A

Nitrofurantoin: which time schedule do you use? [Day(s) after VUS/VCUG, how many?]
N/A

Amoxicillin: which time schedule do you use? [Day(s) before VUS/VCUG, how many?]
N/A

Amoxicillin: which time schedule do you use? [Day of VUS/VCUG]
N/A

Amoxicillin: which time schedule do you use? [Day(s) after VUS/VCUG, how many?]
N/A

Cefaclor: which time schedule do you use? [Days(s) before VUS/VCUG, how many?]
N/A

Cefaclor: which time schedule do you use? [Day of VUS/VCUG]
N/A

Cefaclor: which time schedule do you use? [Day(s) after VUS/VCUG, how many?]
N/A

Amoxicillin/clavulanic acid: in what dosage? (Amoxicillin portion) [prophylaktic (10-20 mg/Kg bodyweight/d)]
N/A

Amoxicillin/clavulanic acid: in what dosage? (Amoxicillin portion) [double prophylactic (20-40 mg/Kg bodyweight/d)]
N/A

Amoxicillin/clavulanic acid: in what dosage? (Amoxicillin portion) [therapeutic (80-100 mg/Kg bodyweight/d)]
N/A

Amoxicillin/clavulanic acid: which time schedule do you use? [Day(s) before VUS/VCUG]
N/A

Amoxicillin/clavulanic acid: which time schedule do you use? [Day of VUS/VCUG]
N/A

Amoxicillin/clavulanic acid: which time schedule do you use? [Day(s) after VUS/VCUG]
N/A

Other: which time schedule do you use? [Day(s) before VUS/VCUG, how many?]
N/A

Other: which time schedule do you use? [Day of VUS/VCUG]
N/A

Other: which time schedule do you use? [Day(s) after VUS/VCUG, how many?]
N/A

Indication for prophylaxis

Which patients receive periinterventional antibiotic prophylaxis? (multiple answers possible) [all]
No

Which patients receive periinterventional antibiotic prophylaxis? (multiple answers possible) [patients post urinary tract infection/pyelonephritis/urosepsis]
No

Which patients receive periinterventional antibiotic prophylaxis? (multiple answers possible) [patients with urinary tract dilation in ultrasound]
No

Which patients receive periinterventional antibiotic prophylaxis? (multiple answers possible) [patients with prenatal urinary tract dilation]
No

Which patients receive periinterventional antibiotic prophylaxis? (multiple answers possible) [patients post VUR surgery]
No

Which patients receive periinterventional antibiotic prophylaxis? (multiple answers possible) [none]
Yes

Which patients receive periinterventional antibiotic prophylaxis? (multiple answers possible) [Other]

Are there situations or clinical findings in the patient where you deviate from your prophylaxis standard (medication, dose, duration, age of the patient)? (multiple answers possible, please specify in answer box) [in case of already known vesicoureteral reflux (VUR)]
No

Are there situations or clinical findings in the patient where you deviate from your prophylaxis standard (medication, dose, duration, age of the patient)? (multiple answers possible, please specify in answer box) [in case of already known vesicoureteral reflux (VUR)][Comment]

Are there situations or clinical findings in the patient where you deviate from your prophylaxis standard (medication, dose, duration, age of the patient)? (multiple answers possible, please specify in answer box) [in patients with recurrent urinary tract infections (UTI)]
No

Are there situations or clinical findings in the patient where you deviate from your prophylaxis standard (medication, dose, duration, age of the patient)? (multiple answers possible, please specify in answer box) [in patients with recurrent urinary tract infections (UTI)][Comment]

Are there situations or clinical findings in the patient where you deviate from your prophylaxis standard (medication, dose, duration, age of the patient)? (multiple answers possible, please specify in answer box) [if multidrug-resistant organisms (MDRO) are detected in urine cultures]
No

Are there situations or clinical findings in the patient where you deviate from your prophylaxis standard (medication, dose, duration, age of the patient)? (multiple answers possible, please specify in answer box) [if multidrug-resistant organisms (MDRO) are detected in urine cultures][Comment]

Are there situations or clinical findings in the patient where you deviate from your prophylaxis standard (medication, dose, duration, age of the patient)? (multiple answers possible, please specify in answer box) [in newborns]
No

Are there situations or clinical findings in the patient where you deviate from your prophylaxis standard (medication, dose, duration, age of the patient)? (multiple answers possible, please specify in answer box) [in newborns][Comment]

Are there situations or clinical findings in the patient where you deviate from your prophylaxis standard (medication, dose, duration, age of the patient)? (multiple answers possible, please specify in answer box) [Other]
all cases

Are there situations or clinical findings in the patient where you deviate from your prophylaxis standard (medication, dose, duration, age of the patient)? (multiple answers possible, please specify in answer box) [Other comment]
no prophylaxis if no oinfection; regular treatment of UTI. At least 5 days of treatment before VCUG in case of infection.

Are there situations in which you use intravenous prophylaxis?
No

Voiding analysis

Are there differences in antibiotic prophylaxis between VUS and VCUG in your department?
No

Is a non-pathological urine strip test required in preparation for VUS/VCUG in your department?
Yes

What is the maximum amount of days accepted for urine strip test to be performed prior to the exam?
few days and no symptoms

Is a non-pathological urine culture required in preparation for VUS/VCUG in your department?
No

Do patients only receive post-procedure prophylaxis if vesicoureteral reflux (VUR) is detected in VUS/VCUG?
No

Do other MUS/MCU findings (e.g. urethral valves) have an influence on post-procedure prophylaxis?
No

End of survey

Thank you for taking the time to answer this survey!

May we contact you personally if we have further questions?
Yes

E-mail address:
bogdan_olteanu!@yahoo.com

Survey response 192

Response ID
194

Date submitted
2025-03-25 22:16:57

Last page
5

Start language
en

Seed
1858704375

Date started
2025-03-25 22:11:44

Date last action
2025-03-25 22:16:57

Standard Operation Procedure

Who fills out this survey?
Pediatric Radiologist

Who fills out this survey? [Other]

In which type of hospital are you employed?
University Hospital

In which type of hospital are you employed? [Other]

In which country do you work?
USA

Does your department have a defined protocol for infection prophylaxis in the context of VUS/VCUG?
Yes

Has this protocol been transformed into a written standard operating procedure (SOP)?
Yes

Which department is the author of the SOP for infection prophylaxis in VUS/VCUG?
Pediatric radiology

Which department is the author of the SOP for infection prophylaxis in VUS/VCUG? [Other]

Antibiotic Prophylaxis

Are there situations/constellations in which you do not use antibiotic prophylaxis for VUS/VCUG?
Yes

Please specify these situations/constellations: [girl with first uncomplicated UTI without urinary tract dilation in ultrasound]
No

Please specify these situations/constellations: [boy with first uncomplicated UTI without urinary tract dilation in ultrasound]
No

Please specify these situations/constellations: [in patients with low grade urinary tract dilation (up to 2nd grade) and no UTI in medical history]
No

Please specify these situations/constellations: [Other]
we do not recommend antibiotic prophylaxis

If you use antibiotic prophylaxis: which mediaction do you use? (multiple answers and subdivision into age groups possible) [Trimethoprim]
No

If you use antibiotic prophylaxis: which mediaction do you use? (multiple answers and subdivision into age groups possible) [Nitrofurantoin]
No

If you use antibiotic prophylaxis: which mediaction do you use? (multiple answers and subdivision into age groups possible) [Amoxicillin]
No

If you use antibiotic prophylaxis: which mediaction do you use? (multiple answers and subdivision into age groups possible) [Cefaclor]
No

If you use antibiotic prophylaxis: which mediaction do you use? (multiple answers and subdivision into age groups possible) [Amoxicillin/clavulanic acid]
No

If you use antibiotic prophylaxis: which mediaction do you use? (multiple answers and subdivision into age groups possible) [Other]
No

Trimethoprim: which time schedule do you use? [Day(s) before VUS/VCUG, how many?]
N/A

Trimethoprim: which time schedule do you use? [Day of VUS/VCUG]
N/A

Trimethoprim: which time schedule do you use? [Day(s) after VUS/VCUG, how many?]
N/A

Nitrofurantoin: which time schedule do you use? [Day(s) before VUS/VCUG, how many?]
N/A

Nitrofurantoin: which time schedule do you use? [Day of VUS/VCUG]
N/A

Nitrofurantoin: which time schedule do you use? [Day(s) after VUS/VCUG, how many?]
N/A

Amoxicillin: which time schedule do you use? [Day(s) before VUS/VCUG, how many?]
N/A

Amoxicillin: which time schedule do you use? [Day of VUS/VCUG]
N/A

Amoxicillin: which time schedule do you use? [Day(s) after VUS/VCUG, how many?]
N/A

Cefaclor: which time schedule do you use? [Days(s) before VUS/VCUG, how many?]
N/A

Cefaclor: which time schedule do you use? [Day of VUS/VCUG]
N/A

Cefaclor: which time schedule do you use? [Day(s) after VUS/VCUG, how many?]
N/A

Amoxicillin/clavulanic acid: in what dosage? (Amoxicillin portion) [prophylaktic (10-20 mg/Kg bodyweight/d)]
N/A

Amoxicillin/clavulanic acid: in what dosage? (Amoxicillin portion) [double prophylactic (20-40 mg/Kg bodyweight/d)]
N/A

Amoxicillin/clavulanic acid: in what dosage? (Amoxicillin portion) [therapeutic (80-100 mg/Kg bodyweight/d)]
N/A

Amoxicillin/clavulanic acid: which time schedule do you use? [Day(s) before VUS/VCUG]
N/A

Amoxicillin/clavulanic acid: which time schedule do you use? [Day of VUS/VCUG]
N/A

Amoxicillin/clavulanic acid: which time schedule do you use? [Day(s) after VUS/VCUG]
N/A

Other: which time schedule do you use? [Day(s) before VUS/VCUG, how many?]
N/A

Other: which time schedule do you use? [Day of VUS/VCUG]
N/A

Other: which time schedule do you use? [Day(s) after VUS/VCUG, how many?]
N/A

Indication for prophylaxis

Which patients receive periinterventional antibiotic prophylaxis? (multiple answers possible) [all]
No

Which patients receive periinterventional antibiotic prophylaxis? (multiple answers possible) [patients post urinary tract infection/pyelonephritis/urosepsis]
No

Which patients receive periinterventional antibiotic prophylaxis? (multiple answers possible) [patients with urinary tract dilation in ultrasound]
No

Which patients receive periinterventional antibiotic prophylaxis? (multiple answers possible) [patients with prenatal urinary tract dilation]
No

Which patients receive periinterventional antibiotic prophylaxis? (multiple answers possible) [patients post VUR surgery]
No

Which patients receive periinterventional antibiotic prophylaxis? (multiple answers possible) [none]
Yes

Which patients receive periinterventional antibiotic prophylaxis? (multiple answers possible) [Other]

Are there situations or clinical findings in the patient where you deviate from your prophylaxis standard (medication, dose, duration, age of the patient)? (multiple answers possible, please specify in answer box) [in case of already known vesicoureteral reflux (VUR)]
No

Are there situations or clinical findings in the patient where you deviate from your prophylaxis standard (medication, dose, duration, age of the patient)? (multiple answers possible, please specify in answer box) [in case of already known vesicoureteral reflux (VUR)][Comment]

Are there situations or clinical findings in the patient where you deviate from your prophylaxis standard (medication, dose, duration, age of the patient)? (multiple answers possible, please specify in answer box) [in patients with recurrent urinary tract infections (UTI)]
No

Are there situations or clinical findings in the patient where you deviate from your prophylaxis standard (medication, dose, duration, age of the patient)? (multiple answers possible, please specify in answer box) [in patients with recurrent urinary tract infections (UTI)][Comment]

Are there situations or clinical findings in the patient where you deviate from your prophylaxis standard (medication, dose, duration, age of the patient)? (multiple answers possible, please specify in answer box) [if multidrug-resistant organisms (MDRO) are detected in urine cultures]
No

Are there situations or clinical findings in the patient where you deviate from your prophylaxis standard (medication, dose, duration, age of the patient)? (multiple answers possible, please specify in answer box) [if multidrug-resistant organisms (MDRO) are detected in urine cultures][Comment]

Are there situations or clinical findings in the patient where you deviate from your prophylaxis standard (medication, dose, duration, age of the patient)? (multiple answers possible, please specify in answer box) [in newborns]
No

Are there situations or clinical findings in the patient where you deviate from your prophylaxis standard (medication, dose, duration, age of the patient)? (multiple answers possible, please specify in answer box) [in newborns][Comment]

Are there situations or clinical findings in the patient where you deviate from your prophylaxis standard (medication, dose, duration, age of the patient)? (multiple answers possible, please specify in answer box) [Other]
we don't recommend prophylaxis

Are there situations or clinical findings in the patient where you deviate from your prophylaxis standard (medication, dose, duration, age of the patient)? (multiple answers possible, please specify in answer box) [Other comment]

Are there situations in which you use intravenous prophylaxis?
No

Voiding analysis

Are there differences in antibiotic prophylaxis between VUS and VCUG in your department?
No

Is a non-pathological urine strip test required in preparation for VUS/VCUG in your department?
No

Is a non-pathological urine culture required in preparation for VUS/VCUG in your department?
No

Do patients only receive post-procedure prophylaxis if vesicoureteral reflux (VUR) is detected in VUS/VCUG?
Yes

Please explain your approach:
per pediatric urology recommendation

Do other MUS/MCU findings (e.g. urethral valves) have an influence on post-procedure prophylaxis?
No

End of survey

Thank you for taking the time to answer this survey!

May we contact you personally if we have further questions?
Yes

E-mail address:
mazurud@health.missouri.edu

Survey response 193

Response ID
195

Last page
1

Start language
en

Seed
1625700542

Date started
2025-03-25 22:12:13

Date last action
2025-03-25 22:12:47

Standard Operation Procedure

Who fills out this survey?
Pediatric Radiologist

Who fills out this survey? [Other]

In which type of hospital are you employed?
Community hospital (

In which type of hospital are you employed? [Other]

In which country do you work?
United Kingdom

Does your department have a defined protocol for infection prophylaxis in the context of VUS/VCUG?
Yes

Has this protocol been transformed into a written standard operating procedure (SOP)?
Yes

Which department is the author of the SOP for infection prophylaxis in VUS/VCUG?
Pediatrics

Which department is the author of the SOP for infection prophylaxis in VUS/VCUG? [Other]

Antibiotic Prophylaxis

Are there situations/constellations in which you do not use antibiotic prophylaxis for VUS/VCUG?
N/A

Please specify these situations/constellations: [girl with first uncomplicated UTI without urinary tract dilation in ultrasound]
N/A

Please specify these situations/constellations: [boy with first uncomplicated UTI without urinary tract dilation in ultrasound]
N/A

Please specify these situations/constellations: [in patients with low grade urinary tract dilation (up to 2nd grade) and no UTI in medical history]
N/A

If you use antibiotic prophylaxis: which mediaction do you use? (multiple answers and subdivision into age groups possible) [Trimethoprim]
N/A

If you use antibiotic prophylaxis: which mediaction do you use? (multiple answers and subdivision into age groups possible) [Nitrofurantoin]
N/A

If you use antibiotic prophylaxis: which mediaction do you use? (multiple answers and subdivision into age groups possible) [Amoxicillin]
N/A

If you use antibiotic prophylaxis: which mediaction do you use? (multiple answers and subdivision into age groups possible) [Cefaclor]
N/A

If you use antibiotic prophylaxis: which mediaction do you use? (multiple answers and subdivision into age groups possible) [Amoxicillin/clavulanic acid]
N/A

If you use antibiotic prophylaxis: which mediaction do you use? (multiple answers and subdivision into age groups possible) [Other]
N/A

Trimethoprim: which time schedule do you use? [Day(s) before VUS/VCUG, how many?]
N/A

Trimethoprim: which time schedule do you use? [Day of VUS/VCUG]
N/A

Trimethoprim: which time schedule do you use? [Day(s) after VUS/VCUG, how many?]
N/A

Nitrofurantoin: which time schedule do you use? [Day(s) before VUS/VCUG, how many?]
N/A

Nitrofurantoin: which time schedule do you use? [Day of VUS/VCUG]
N/A

Nitrofurantoin: which time schedule do you use? [Day(s) after VUS/VCUG, how many?]
N/A

Amoxicillin: which time schedule do you use? [Day(s) before VUS/VCUG, how many?]
N/A

Amoxicillin: which time schedule do you use? [Day of VUS/VCUG]
N/A

Amoxicillin: which time schedule do you use? [Day(s) after VUS/VCUG, how many?]
N/A

Cefaclor: which time schedule do you use? [Days(s) before VUS/VCUG, how many?]
N/A

Cefaclor: which time schedule do you use? [Day of VUS/VCUG]
N/A

Cefaclor: which time schedule do you use? [Day(s) after VUS/VCUG, how many?]
N/A

Amoxicillin/clavulanic acid: in what dosage? (Amoxicillin portion) [prophylaktic (10-20 mg/Kg bodyweight/d)]
N/A

Amoxicillin/clavulanic acid: in what dosage? (Amoxicillin portion) [double prophylactic (20-40 mg/Kg bodyweight/d)]
N/A

Amoxicillin/clavulanic acid: in what dosage? (Amoxicillin portion) [therapeutic (80-100 mg/Kg bodyweight/d)]
N/A

Amoxicillin/clavulanic acid: which time schedule do you use? [Day(s) before VUS/VCUG]
N/A

Amoxicillin/clavulanic acid: which time schedule do you use? [Day of VUS/VCUG]
N/A

Amoxicillin/clavulanic acid: which time schedule do you use? [Day(s) after VUS/VCUG]
N/A

Other: which time schedule do you use? [Day(s) before VUS/VCUG, how many?]
N/A

Other: which time schedule do you use? [Day of VUS/VCUG]
N/A

Other: which time schedule do you use? [Day(s) after VUS/VCUG, how many?]
N/A

Indication for prophylaxis

Which patients receive periinterventional antibiotic prophylaxis? (multiple answers possible) [all]
N/A

Which patients receive periinterventional antibiotic prophylaxis? (multiple answers possible) [patients post urinary tract infection/pyelonephritis/urosepsis]
N/A

Which patients receive periinterventional antibiotic prophylaxis? (multiple answers possible) [patients with urinary tract dilation in ultrasound]
N/A

Which patients receive periinterventional antibiotic prophylaxis? (multiple answers possible) [patients with prenatal urinary tract dilation]
N/A

Which patients receive periinterventional antibiotic prophylaxis? (multiple answers possible) [patients post VUR surgery]
N/A

Which patients receive periinterventional antibiotic prophylaxis? (multiple answers possible) [none]
N/A

Are there situations or clinical findings in the patient where you deviate from your prophylaxis standard (medication, dose, duration, age of the patient)? (multiple answers possible, please specify in answer box) [in case of already known vesicoureteral reflux (VUR)]
N/A

Are there situations or clinical findings in the patient where you deviate from your prophylaxis standard (medication, dose, duration, age of the patient)? (multiple answers possible, please specify in answer box) [in patients with recurrent urinary tract infections (UTI)]
N/A

Are there situations or clinical findings in the patient where you deviate from your prophylaxis standard (medication, dose, duration, age of the patient)? (multiple answers possible, please specify in answer box) [if multidrug-resistant organisms (MDRO) are detected in urine cultures]
N/A

Are there situations or clinical findings in the patient where you deviate from your prophylaxis standard (medication, dose, duration, age of the patient)? (multiple answers possible, please specify in answer box) [in newborns]
N/A

Are there situations in which you use intravenous prophylaxis?
N/A

Voiding analysis

Are there differences in antibiotic prophylaxis between VUS and VCUG in your department?
N/A

Is a non-pathological urine strip test required in preparation for VUS/VCUG in your department?
N/A

Is a non-pathological urine culture required in preparation for VUS/VCUG in your department?
N/A

Do patients only receive post-procedure prophylaxis if vesicoureteral reflux (VUR) is detected in VUS/VCUG?
N/A

Do other MUS/MCU findings (e.g. urethral valves) have an influence on post-procedure prophylaxis?
N/A

End of survey

May we contact you personally if we have further questions?
N/A

Survey response 194

Response ID
196

Date submitted
2025-03-26 07:33:30

Last page
5

Start language
en

Seed
1779467707

Date started
2025-03-26 07:31:22

Date last action
2025-03-26 07:33:30

Standard Operation Procedure

Who fills out this survey?
Pediatric Radiologist

Who fills out this survey? [Other]

In which type of hospital are you employed?
University Hospital

In which type of hospital are you employed? [Other]

In which country do you work?
netherlands

Does your department have a defined protocol for infection prophylaxis in the context of VUS/VCUG?
Yes

Has this protocol been transformed into a written standard operating procedure (SOP)?
Yes

Which department is the author of the SOP for infection prophylaxis in VUS/VCUG?
Pediatric radiology

Which department is the author of the SOP for infection prophylaxis in VUS/VCUG? [Other]

Antibiotic Prophylaxis

Are there situations/constellations in which you do not use antibiotic prophylaxis for VUS/VCUG?
No

Please specify these situations/constellations: [girl with first uncomplicated UTI without urinary tract dilation in ultrasound]
N/A

Please specify these situations/constellations: [boy with first uncomplicated UTI without urinary tract dilation in ultrasound]
N/A

Please specify these situations/constellations: [in patients with low grade urinary tract dilation (up to 2nd grade) and no UTI in medical history]
N/A

If you use antibiotic prophylaxis: which mediaction do you use? (multiple answers and subdivision into age groups possible) [Trimethoprim]
Yes

If you use antibiotic prophylaxis: which mediaction do you use? (multiple answers and subdivision into age groups possible) [Nitrofurantoin]
No

If you use antibiotic prophylaxis: which mediaction do you use? (multiple answers and subdivision into age groups possible) [Amoxicillin]
No

If you use antibiotic prophylaxis: which mediaction do you use? (multiple answers and subdivision into age groups possible) [Cefaclor]
No

If you use antibiotic prophylaxis: which mediaction do you use? (multiple answers and subdivision into age groups possible) [Amoxicillin/clavulanic acid]
No

If you use antibiotic prophylaxis: which mediaction do you use? (multiple answers and subdivision into age groups possible) [Other]
No

Trimethoprim: in what dosage?
double prophylakctic (4 mg/kg bodyweight/d)

Trimethoprim: in what dosage? [Other]

Trimethoprim: for which age group do you use trimethoprim?
all

Trimethoprim: which time schedule do you use? [Day(s) before VUS/VCUG, how many?]
Yes

Trimethoprim: which time schedule do you use? [Day(s) before VUS/VCUG, how many?][Comment]
1

Trimethoprim: which time schedule do you use? [Day of VUS/VCUG]
Yes

Trimethoprim: which time schedule do you use? [Day of VUS/VCUG][Comment]

Trimethoprim: which time schedule do you use? [Day(s) after VUS/VCUG, how many?]
Yes

Trimethoprim: which time schedule do you use? [Day(s) after VUS/VCUG, how many?][Comment]
1

Nitrofurantoin: which time schedule do you use? [Day(s) before VUS/VCUG, how many?]
N/A

Nitrofurantoin: which time schedule do you use? [Day of VUS/VCUG]
N/A

Nitrofurantoin: which time schedule do you use? [Day(s) after VUS/VCUG, how many?]
N/A

Amoxicillin: which time schedule do you use? [Day(s) before VUS/VCUG, how many?]
N/A

Amoxicillin: which time schedule do you use? [Day of VUS/VCUG]
N/A

Amoxicillin: which time schedule do you use? [Day(s) after VUS/VCUG, how many?]
N/A

Cefaclor: which time schedule do you use? [Days(s) before VUS/VCUG, how many?]
N/A

Cefaclor: which time schedule do you use? [Day of VUS/VCUG]
N/A

Cefaclor: which time schedule do you use? [Day(s) after VUS/VCUG, how many?]
N/A

Amoxicillin/clavulanic acid: in what dosage? (Amoxicillin portion) [prophylaktic (10-20 mg/Kg bodyweight/d)]
N/A

Amoxicillin/clavulanic acid: in what dosage? (Amoxicillin portion) [double prophylactic (20-40 mg/Kg bodyweight/d)]
N/A

Amoxicillin/clavulanic acid: in what dosage? (Amoxicillin portion) [therapeutic (80-100 mg/Kg bodyweight/d)]
N/A

Amoxicillin/clavulanic acid: which time schedule do you use? [Day(s) before VUS/VCUG]
N/A

Amoxicillin/clavulanic acid: which time schedule do you use? [Day of VUS/VCUG]
N/A

Amoxicillin/clavulanic acid: which time schedule do you use? [Day(s) after VUS/VCUG]
N/A

Other: which time schedule do you use? [Day(s) before VUS/VCUG, how many?]
N/A

Other: which time schedule do you use? [Day of VUS/VCUG]
N/A

Other: which time schedule do you use? [Day(s) after VUS/VCUG, how many?]
N/A

Indication for prophylaxis

Which patients receive periinterventional antibiotic prophylaxis? (multiple answers possible) [all]
Yes

Which patients receive periinterventional antibiotic prophylaxis? (multiple answers possible) [patients post urinary tract infection/pyelonephritis/urosepsis]
N/A

Which patients receive periinterventional antibiotic prophylaxis? (multiple answers possible) [patients with urinary tract dilation in ultrasound]
N/A

Which patients receive periinterventional antibiotic prophylaxis? (multiple answers possible) [patients with prenatal urinary tract dilation]
N/A

Which patients receive periinterventional antibiotic prophylaxis? (multiple answers possible) [patients post VUR surgery]
N/A

Which patients receive periinterventional antibiotic prophylaxis? (multiple answers possible) [none]
N/A

Are there situations or clinical findings in the patient where you deviate from your prophylaxis standard (medication, dose, duration, age of the patient)? (multiple answers possible, please specify in answer box) [in case of already known vesicoureteral reflux (VUR)]
No

Are there situations or clinical findings in the patient where you deviate from your prophylaxis standard (medication, dose, duration, age of the patient)? (multiple answers possible, please specify in answer box) [in case of already known vesicoureteral reflux (VUR)][Comment]

Are there situations or clinical findings in the patient where you deviate from your prophylaxis standard (medication, dose, duration, age of the patient)? (multiple answers possible, please specify in answer box) [in patients with recurrent urinary tract infections (UTI)]
No

Are there situations or clinical findings in the patient where you deviate from your prophylaxis standard (medication, dose, duration, age of the patient)? (multiple answers possible, please specify in answer box) [in patients with recurrent urinary tract infections (UTI)][Comment]

Are there situations or clinical findings in the patient where you deviate from your prophylaxis standard (medication, dose, duration, age of the patient)? (multiple answers possible, please specify in answer box) [if multidrug-resistant organisms (MDRO) are detected in urine cultures]
Yes

Are there situations or clinical findings in the patient where you deviate from your prophylaxis standard (medication, dose, duration, age of the patient)? (multiple answers possible, please specify in answer box) [if multidrug-resistant organisms (MDRO) are detected in urine cultures][Comment]
yes

Are there situations or clinical findings in the patient where you deviate from your prophylaxis standard (medication, dose, duration, age of the patient)? (multiple answers possible, please specify in answer box) [in newborns]
No

Are there situations or clinical findings in the patient where you deviate from your prophylaxis standard (medication, dose, duration, age of the patient)? (multiple answers possible, please specify in answer box) [in newborns][Comment]

Are there situations or clinical findings in the patient where you deviate from your prophylaxis standard (medication, dose, duration, age of the patient)? (multiple answers possible, please specify in answer box) [Other]

Are there situations or clinical findings in the patient where you deviate from your prophylaxis standard (medication, dose, duration, age of the patient)? (multiple answers possible, please specify in answer box) [Other comment]

Are there situations in which you use intravenous prophylaxis?
No

Voiding analysis

Are there differences in antibiotic prophylaxis between VUS and VCUG in your department?
No

Is a non-pathological urine strip test required in preparation for VUS/VCUG in your department?
No

Is a non-pathological urine culture required in preparation for VUS/VCUG in your department?
No

Do patients only receive post-procedure prophylaxis if vesicoureteral reflux (VUR) is detected in VUS/VCUG?
Yes

Please explain your approach:
nefrology takes care of this

Do other MUS/MCU findings (e.g. urethral valves) have an influence on post-procedure prophylaxis?
No

End of survey

Thank you for taking the time to answer this survey!

May we contact you personally if we have further questions?
Yes

E-mail address:
m.verhagen@umcg.nl

Survey response 195

Response ID
197

Date submitted
2025-03-26 11:03:24

Last page
5

Start language
de

Seed
1668077647

Date started
2025-03-26 10:59:27

Date last action
2025-03-26 11:03:24

Standard Operation Procedure

Who fills out this survey?
Pediatric urologist

Who fills out this survey? [Other]

In which type of hospital are you employed?
University Hospital

In which type of hospital are you employed? [Other]

In which country do you work?
Deutschland

Does your department have a defined protocol for infection prophylaxis in the context of VUS/VCUG?
Yes

Has this protocol been transformed into a written standard operating procedure (SOP)?
Yes

Which department is the author of the SOP for infection prophylaxis in VUS/VCUG?
Pediatric surgery

Which department is the author of the SOP for infection prophylaxis in VUS/VCUG? [Other]

Antibiotic Prophylaxis

Are there situations/constellations in which you do not use antibiotic prophylaxis for VUS/VCUG?
No

Please specify these situations/constellations: [girl with first uncomplicated UTI without urinary tract dilation in ultrasound]
N/A

Please specify these situations/constellations: [boy with first uncomplicated UTI without urinary tract dilation in ultrasound]
N/A

Please specify these situations/constellations: [in patients with low grade urinary tract dilation (up to 2nd grade) and no UTI in medical history]
N/A

If you use antibiotic prophylaxis: which mediaction do you use? (multiple answers and subdivision into age groups possible) [Trimethoprim]
No

If you use antibiotic prophylaxis: which mediaction do you use? (multiple answers and subdivision into age groups possible) [Nitrofurantoin]
No

If you use antibiotic prophylaxis: which mediaction do you use? (multiple answers and subdivision into age groups possible) [Amoxicillin]
No

If you use antibiotic prophylaxis: which mediaction do you use? (multiple answers and subdivision into age groups possible) [Cefaclor]
Yes

If you use antibiotic prophylaxis: which mediaction do you use? (multiple answers and subdivision into age groups possible) [Amoxicillin/clavulanic acid]
No

If you use antibiotic prophylaxis: which mediaction do you use? (multiple answers and subdivision into age groups possible) [Other]
No

Trimethoprim: which time schedule do you use? [Day(s) before VUS/VCUG, how many?]
N/A

Trimethoprim: which time schedule do you use? [Day of VUS/VCUG]
N/A

Trimethoprim: which time schedule do you use? [Day(s) after VUS/VCUG, how many?]
N/A

Nitrofurantoin: which time schedule do you use? [Day(s) before VUS/VCUG, how many?]
N/A

Nitrofurantoin: which time schedule do you use? [Day of VUS/VCUG]
N/A

Nitrofurantoin: which time schedule do you use? [Day(s) after VUS/VCUG, how many?]
N/A

Amoxicillin: which time schedule do you use? [Day(s) before VUS/VCUG, how many?]
N/A

Amoxicillin: which time schedule do you use? [Day of VUS/VCUG]
N/A

Amoxicillin: which time schedule do you use? [Day(s) after VUS/VCUG, how many?]
N/A

Cefaclor: in what dosage?
double prophylactic (20 mg/kg bodyweight/d)

Cefaclor: in what dosage? [Other]

Cefaclor: for which age group do you use cefaclor?
alle

Cefaclor: which time schedule do you use? [Days(s) before VUS/VCUG, how many?]
Yes

Cefaclor: which time schedule do you use? [Days(s) before VUS/VCUG, how many?][Comment]
1

Cefaclor: which time schedule do you use? [Day of VUS/VCUG]
No

Cefaclor: which time schedule do you use? [Day of VUS/VCUG][Comment]

Cefaclor: which time schedule do you use? [Day(s) after VUS/VCUG, how many?]
Yes

Cefaclor: which time schedule do you use? [Day(s) after VUS/VCUG, how many?][Comment]
1

Amoxicillin/clavulanic acid: in what dosage? (Amoxicillin portion) [prophylaktic (10-20 mg/Kg bodyweight/d)]
N/A

Amoxicillin/clavulanic acid: in what dosage? (Amoxicillin portion) [double prophylactic (20-40 mg/Kg bodyweight/d)]
N/A

Amoxicillin/clavulanic acid: in what dosage? (Amoxicillin portion) [therapeutic (80-100 mg/Kg bodyweight/d)]
N/A

Amoxicillin/clavulanic acid: which time schedule do you use? [Day(s) before VUS/VCUG]
N/A

Amoxicillin/clavulanic acid: which time schedule do you use? [Day of VUS/VCUG]
N/A

Amoxicillin/clavulanic acid: which time schedule do you use? [Day(s) after VUS/VCUG]
N/A

Other: which time schedule do you use? [Day(s) before VUS/VCUG, how many?]
N/A

Other: which time schedule do you use? [Day of VUS/VCUG]
N/A

Other: which time schedule do you use? [Day(s) after VUS/VCUG, how many?]
N/A

Indication for prophylaxis

Which patients receive periinterventional antibiotic prophylaxis? (multiple answers possible) [all]
Yes

Which patients receive periinterventional antibiotic prophylaxis? (multiple answers possible) [patients post urinary tract infection/pyelonephritis/urosepsis]
N/A

Which patients receive periinterventional antibiotic prophylaxis? (multiple answers possible) [patients with urinary tract dilation in ultrasound]
N/A

Which patients receive periinterventional antibiotic prophylaxis? (multiple answers possible) [patients with prenatal urinary tract dilation]
N/A

Which patients receive periinterventional antibiotic prophylaxis? (multiple answers possible) [patients post VUR surgery]
N/A

Which patients receive periinterventional antibiotic prophylaxis? (multiple answers possible) [none]
N/A

Are there situations or clinical findings in the patient where you deviate from your prophylaxis standard (medication, dose, duration, age of the patient)? (multiple answers possible, please specify in answer box) [in case of already known vesicoureteral reflux (VUR)]
No

Are there situations or clinical findings in the patient where you deviate from your prophylaxis standard (medication, dose, duration, age of the patient)? (multiple answers possible, please specify in answer box) [in case of already known vesicoureteral reflux (VUR)][Comment]

Are there situations or clinical findings in the patient where you deviate from your prophylaxis standard (medication, dose, duration, age of the patient)? (multiple answers possible, please specify in answer box) [in patients with recurrent urinary tract infections (UTI)]
No

Are there situations or clinical findings in the patient where you deviate from your prophylaxis standard (medication, dose, duration, age of the patient)? (multiple answers possible, please specify in answer box) [in patients with recurrent urinary tract infections (UTI)][Comment]

Are there situations or clinical findings in the patient where you deviate from your prophylaxis standard (medication, dose, duration, age of the patient)? (multiple answers possible, please specify in answer box) [if multidrug-resistant organisms (MDRO) are detected in urine cultures]
Yes

Are there situations or clinical findings in the patient where you deviate from your prophylaxis standard (medication, dose, duration, age of the patient)? (multiple answers possible, please specify in answer box) [if multidrug-resistant organisms (MDRO) are detected in urine cultures][Comment]

Are there situations or clinical findings in the patient where you deviate from your prophylaxis standard (medication, dose, duration, age of the patient)? (multiple answers possible, please specify in answer box) [in newborns]
No

Are there situations or clinical findings in the patient where you deviate from your prophylaxis standard (medication, dose, duration, age of the patient)? (multiple answers possible, please specify in answer box) [in newborns][Comment]

Are there situations or clinical findings in the patient where you deviate from your prophylaxis standard (medication, dose, duration, age of the patient)? (multiple answers possible, please specify in answer box) [Other]

Are there situations or clinical findings in the patient where you deviate from your prophylaxis standard (medication, dose, duration, age of the patient)? (multiple answers possible, please specify in answer box) [Other comment]

Are there situations in which you use intravenous prophylaxis?
No

Voiding analysis

Are there differences in antibiotic prophylaxis between VUS and VCUG in your department?
No

Is a non-pathological urine strip test required in preparation for VUS/VCUG in your department?
Yes

What is the maximum amount of days accepted for urine strip test to be performed prior to the exam?
3

Is a non-pathological urine culture required in preparation for VUS/VCUG in your department?
No

Do patients only receive post-procedure prophylaxis if vesicoureteral reflux (VUR) is detected in VUS/VCUG?
No

Do other MUS/MCU findings (e.g. urethral valves) have an influence on post-procedure prophylaxis?
No

End of survey

Thank you for taking the time to answer this survey!

May we contact you personally if we have further questions?
Yes

E-mail address:
hofmann.alejandro@mh-hannover.de

Survey response 196

Response ID
198

Date submitted
2025-03-26 11:25:39

Last page
5

Start language
en

Seed
1305131137

Date started
2025-03-26 11:21:15

Date last action
2025-03-26 11:25:39

Standard Operation Procedure

Who fills out this survey?
Pediatric Radiologist

Who fills out this survey? [Other]

In which type of hospital are you employed?
University Hospital

In which type of hospital are you employed? [Other]

In which country do you work?
Greece

Does your department have a defined protocol for infection prophylaxis in the context of VUS/VCUG?
Yes

Has this protocol been transformed into a written standard operating procedure (SOP)?
No

Antibiotic Prophylaxis

Are there situations/constellations in which you do not use antibiotic prophylaxis for VUS/VCUG?
No

Please specify these situations/constellations: [girl with first uncomplicated UTI without urinary tract dilation in ultrasound]
N/A

Please specify these situations/constellations: [boy with first uncomplicated UTI without urinary tract dilation in ultrasound]
N/A

Please specify these situations/constellations: [in patients with low grade urinary tract dilation (up to 2nd grade) and no UTI in medical history]
N/A

If you use antibiotic prophylaxis: which mediaction do you use? (multiple answers and subdivision into age groups possible) [Trimethoprim]
No

If you use antibiotic prophylaxis: which mediaction do you use? (multiple answers and subdivision into age groups possible) [Nitrofurantoin]
No

If you use antibiotic prophylaxis: which mediaction do you use? (multiple answers and subdivision into age groups possible) [Amoxicillin]
No

If you use antibiotic prophylaxis: which mediaction do you use? (multiple answers and subdivision into age groups possible) [Cefaclor]
Yes

If you use antibiotic prophylaxis: which mediaction do you use? (multiple answers and subdivision into age groups possible) [Amoxicillin/clavulanic acid]
Yes

If you use antibiotic prophylaxis: which mediaction do you use? (multiple answers and subdivision into age groups possible) [Other]
No

Trimethoprim: which time schedule do you use? [Day(s) before VUS/VCUG, how many?]
N/A

Trimethoprim: which time schedule do you use? [Day of VUS/VCUG]
N/A

Trimethoprim: which time schedule do you use? [Day(s) after VUS/VCUG, how many?]
N/A

Nitrofurantoin: which time schedule do you use? [Day(s) before VUS/VCUG, how many?]
N/A

Nitrofurantoin: which time schedule do you use? [Day of VUS/VCUG]
N/A

Nitrofurantoin: which time schedule do you use? [Day(s) after VUS/VCUG, how many?]
N/A

Amoxicillin: which time schedule do you use? [Day(s) before VUS/VCUG, how many?]
N/A

Amoxicillin: which time schedule do you use? [Day of VUS/VCUG]
N/A

Amoxicillin: which time schedule do you use? [Day(s) after VUS/VCUG, how many?]
N/A

Cefaclor: in what dosage?
prophylactic (10 mg/kg bodyweight/d)

Cefaclor: in what dosage? [Other]

Cefaclor: for which age group do you use cefaclor?
All

Cefaclor: which time schedule do you use? [Days(s) before VUS/VCUG, how many?]
Yes

Cefaclor: which time schedule do you use? [Days(s) before VUS/VCUG, how many?][Comment]
2

Cefaclor: which time schedule do you use? [Day of VUS/VCUG]
Yes

Cefaclor: which time schedule do you use? [Day of VUS/VCUG][Comment]

Cefaclor: which time schedule do you use? [Day(s) after VUS/VCUG, how many?]
Yes

Cefaclor: which time schedule do you use? [Day(s) after VUS/VCUG, how many?][Comment]
2

Amoxicillin/clavulanic acid: in what dosage? (Amoxicillin portion) [prophylaktic (10-20 mg/Kg bodyweight/d)]
Yes

Amoxicillin/clavulanic acid: in what dosage? (Amoxicillin portion) [double prophylactic (20-40 mg/Kg bodyweight/d)]
No

Amoxicillin/clavulanic acid: in what dosage? (Amoxicillin portion) [therapeutic (80-100 mg/Kg bodyweight/d)]
No

Amoxicillin/clavulanic acid: in what dosage? (Amoxicillin portion) [Other]

Amoxicillin/clavulanic acid: for which age group do you use amoxicillin/clavulanic acid?
All

Amoxicillin/clavulanic acid: which time schedule do you use? [Day(s) before VUS/VCUG]
Yes

Amoxicillin/clavulanic acid: which time schedule do you use? [Day(s) before VUS/VCUG][Comment]
2

Amoxicillin/clavulanic acid: which time schedule do you use? [Day of VUS/VCUG]
Yes

Amoxicillin/clavulanic acid: which time schedule do you use? [Day of VUS/VCUG][Comment]

Amoxicillin/clavulanic acid: which time schedule do you use? [Day(s) after VUS/VCUG]
Yes

Amoxicillin/clavulanic acid: which time schedule do you use? [Day(s) after VUS/VCUG][Comment]
2

Other: which time schedule do you use? [Day(s) before VUS/VCUG, how many?]
N/A

Other: which time schedule do you use? [Day of VUS/VCUG]
N/A

Other: which time schedule do you use? [Day(s) after VUS/VCUG, how many?]
N/A

Indication for prophylaxis

Which patients receive periinterventional antibiotic prophylaxis? (multiple answers possible) [all]
Yes

Which patients receive periinterventional antibiotic prophylaxis? (multiple answers possible) [patients post urinary tract infection/pyelonephritis/urosepsis]
N/A

Which patients receive periinterventional antibiotic prophylaxis? (multiple answers possible) [patients with urinary tract dilation in ultrasound]
N/A

Which patients receive periinterventional antibiotic prophylaxis? (multiple answers possible) [patients with prenatal urinary tract dilation]
N/A

Which patients receive periinterventional antibiotic prophylaxis? (multiple answers possible) [patients post VUR surgery]
N/A

Which patients receive periinterventional antibiotic prophylaxis? (multiple answers possible) [none]
N/A

Are there situations or clinical findings in the patient where you deviate from your prophylaxis standard (medication, dose, duration, age of the patient)? (multiple answers possible, please specify in answer box) [in case of already known vesicoureteral reflux (VUR)]
Yes

Are there situations or clinical findings in the patient where you deviate from your prophylaxis standard (medication, dose, duration, age of the patient)? (multiple answers possible, please specify in answer box) [in case of already known vesicoureteral reflux (VUR)][Comment]

Are there situations or clinical findings in the patient where you deviate from your prophylaxis standard (medication, dose, duration, age of the patient)? (multiple answers possible, please specify in answer box) [in patients with recurrent urinary tract infections (UTI)]
Yes

Are there situations or clinical findings in the patient where you deviate from your prophylaxis standard (medication, dose, duration, age of the patient)? (multiple answers possible, please specify in answer box) [in patients with recurrent urinary tract infections (UTI)][Comment]

Are there situations or clinical findings in the patient where you deviate from your prophylaxis standard (medication, dose, duration, age of the patient)? (multiple answers possible, please specify in answer box) [if multidrug-resistant organisms (MDRO) are detected in urine cultures]
No

Are there situations or clinical findings in the patient where you deviate from your prophylaxis standard (medication, dose, duration, age of the patient)? (multiple answers possible, please specify in answer box) [if multidrug-resistant organisms (MDRO) are detected in urine cultures][Comment]

Are there situations or clinical findings in the patient where you deviate from your prophylaxis standard (medication, dose, duration, age of the patient)? (multiple answers possible, please specify in answer box) [in newborns]
No

Are there situations or clinical findings in the patient where you deviate from your prophylaxis standard (medication, dose, duration, age of the patient)? (multiple answers possible, please specify in answer box) [in newborns][Comment]

Are there situations or clinical findings in the patient where you deviate from your prophylaxis standard (medication, dose, duration, age of the patient)? (multiple answers possible, please specify in answer box) [Other]

Are there situations or clinical findings in the patient where you deviate from your prophylaxis standard (medication, dose, duration, age of the patient)? (multiple answers possible, please specify in answer box) [Other comment]

Are there situations in which you use intravenous prophylaxis?
No

Voiding analysis

Are there differences in antibiotic prophylaxis between VUS and VCUG in your department?
No

Is a non-pathological urine strip test required in preparation for VUS/VCUG in your department?
Yes

What is the maximum amount of days accepted for urine strip test to be performed prior to the exam?
2-7

Is a non-pathological urine culture required in preparation for VUS/VCUG in your department?
Yes

What is the maximum amount of days accepted for urine culture to be performed prior to the exam?
7

Do patients only receive post-procedure prophylaxis if vesicoureteral reflux (VUR) is detected in VUS/VCUG?
No

Do other MUS/MCU findings (e.g. urethral valves) have an influence on post-procedure prophylaxis?
No

End of survey

Thank you for taking the time to answer this survey!

May we contact you personally if we have further questions?
No

Survey response 197

Response ID
199

Last page
0

Start language
en

Seed
29354261

Date started
2025-03-26 11:46:01

Date last action
2025-03-26 11:46:01

Standard Operation Procedure

Does your department have a defined protocol for infection prophylaxis in the context of VUS/VCUG?
N/A

Has this protocol been transformed into a written standard operating procedure (SOP)?
N/A

Antibiotic Prophylaxis

Are there situations/constellations in which you do not use antibiotic prophylaxis for VUS/VCUG?
N/A

Please specify these situations/constellations: [girl with first uncomplicated UTI without urinary tract dilation in ultrasound]
N/A

Please specify these situations/constellations: [boy with first uncomplicated UTI without urinary tract dilation in ultrasound]
N/A

Please specify these situations/constellations: [in patients with low grade urinary tract dilation (up to 2nd grade) and no UTI in medical history]
N/A

If you use antibiotic prophylaxis: which mediaction do you use? (multiple answers and subdivision into age groups possible) [Trimethoprim]
N/A

If you use antibiotic prophylaxis: which mediaction do you use? (multiple answers and subdivision into age groups possible) [Nitrofurantoin]
N/A

If you use antibiotic prophylaxis: which mediaction do you use? (multiple answers and subdivision into age groups possible) [Amoxicillin]
N/A

If you use antibiotic prophylaxis: which mediaction do you use? (multiple answers and subdivision into age groups possible) [Cefaclor]
N/A

If you use antibiotic prophylaxis: which mediaction do you use? (multiple answers and subdivision into age groups possible) [Amoxicillin/clavulanic acid]
N/A

If you use antibiotic prophylaxis: which mediaction do you use? (multiple answers and subdivision into age groups possible) [Other]
N/A

Trimethoprim: which time schedule do you use? [Day(s) before VUS/VCUG, how many?]
N/A

Trimethoprim: which time schedule do you use? [Day of VUS/VCUG]
N/A

Trimethoprim: which time schedule do you use? [Day(s) after VUS/VCUG, how many?]
N/A

Nitrofurantoin: which time schedule do you use? [Day(s) before VUS/VCUG, how many?]
N/A

Nitrofurantoin: which time schedule do you use? [Day of VUS/VCUG]
N/A

Nitrofurantoin: which time schedule do you use? [Day(s) after VUS/VCUG, how many?]
N/A

Amoxicillin: which time schedule do you use? [Day(s) before VUS/VCUG, how many?]
N/A

Amoxicillin: which time schedule do you use? [Day of VUS/VCUG]
N/A

Amoxicillin: which time schedule do you use? [Day(s) after VUS/VCUG, how many?]
N/A

Cefaclor: which time schedule do you use? [Days(s) before VUS/VCUG, how many?]
N/A

Cefaclor: which time schedule do you use? [Day of VUS/VCUG]
N/A

Cefaclor: which time schedule do you use? [Day(s) after VUS/VCUG, how many?]
N/A

Amoxicillin/clavulanic acid: in what dosage? (Amoxicillin portion) [prophylaktic (10-20 mg/Kg bodyweight/d)]
N/A

Amoxicillin/clavulanic acid: in what dosage? (Amoxicillin portion) [double prophylactic (20-40 mg/Kg bodyweight/d)]
N/A

Amoxicillin/clavulanic acid: in what dosage? (Amoxicillin portion) [therapeutic (80-100 mg/Kg bodyweight/d)]
N/A

Amoxicillin/clavulanic acid: which time schedule do you use? [Day(s) before VUS/VCUG]
N/A

Amoxicillin/clavulanic acid: which time schedule do you use? [Day of VUS/VCUG]
N/A

Amoxicillin/clavulanic acid: which time schedule do you use? [Day(s) after VUS/VCUG]
N/A

Other: which time schedule do you use? [Day(s) before VUS/VCUG, how many?]
N/A

Other: which time schedule do you use? [Day of VUS/VCUG]
N/A

Other: which time schedule do you use? [Day(s) after VUS/VCUG, how many?]
N/A

Indication for prophylaxis

Which patients receive periinterventional antibiotic prophylaxis? (multiple answers possible) [all]
N/A

Which patients receive periinterventional antibiotic prophylaxis? (multiple answers possible) [patients post urinary tract infection/pyelonephritis/urosepsis]
N/A

Which patients receive periinterventional antibiotic prophylaxis? (multiple answers possible) [patients with urinary tract dilation in ultrasound]
N/A

Which patients receive periinterventional antibiotic prophylaxis? (multiple answers possible) [patients with prenatal urinary tract dilation]
N/A

Which patients receive periinterventional antibiotic prophylaxis? (multiple answers possible) [patients post VUR surgery]
N/A

Which patients receive periinterventional antibiotic prophylaxis? (multiple answers possible) [none]
N/A

Are there situations or clinical findings in the patient where you deviate from your prophylaxis standard (medication, dose, duration, age of the patient)? (multiple answers possible, please specify in answer box) [in case of already known vesicoureteral reflux (VUR)]
N/A

Are there situations or clinical findings in the patient where you deviate from your prophylaxis standard (medication, dose, duration, age of the patient)? (multiple answers possible, please specify in answer box) [in patients with recurrent urinary tract infections (UTI)]
N/A

Are there situations or clinical findings in the patient where you deviate from your prophylaxis standard (medication, dose, duration, age of the patient)? (multiple answers possible, please specify in answer box) [if multidrug-resistant organisms (MDRO) are detected in urine cultures]
N/A

Are there situations or clinical findings in the patient where you deviate from your prophylaxis standard (medication, dose, duration, age of the patient)? (multiple answers possible, please specify in answer box) [in newborns]
N/A

Are there situations in which you use intravenous prophylaxis?
N/A

Voiding analysis

Are there differences in antibiotic prophylaxis between VUS and VCUG in your department?
N/A

Is a non-pathological urine strip test required in preparation for VUS/VCUG in your department?
N/A

Is a non-pathological urine culture required in preparation for VUS/VCUG in your department?
N/A

Do patients only receive post-procedure prophylaxis if vesicoureteral reflux (VUR) is detected in VUS/VCUG?
N/A

Do other MUS/MCU findings (e.g. urethral valves) have an influence on post-procedure prophylaxis?
N/A

End of survey

May we contact you personally if we have further questions?
N/A

Survey response 198

Response ID
200

Last page
1

Start language
en

Seed
600488331

Date started
2025-03-26 12:02:46

Date last action
2025-03-26 12:03:12

Standard Operation Procedure

Who fills out this survey?
Pediatric Radiologist

Who fills out this survey? [Other]

In which type of hospital are you employed?
University Hospital

In which type of hospital are you employed? [Other]

In which country do you work?
Serbia

Does your department have a defined protocol for infection prophylaxis in the context of VUS/VCUG?
Yes

Has this protocol been transformed into a written standard operating procedure (SOP)?
No

Antibiotic Prophylaxis

Are there situations/constellations in which you do not use antibiotic prophylaxis for VUS/VCUG?
N/A

Please specify these situations/constellations: [girl with first uncomplicated UTI without urinary tract dilation in ultrasound]
N/A

Please specify these situations/constellations: [boy with first uncomplicated UTI without urinary tract dilation in ultrasound]
N/A

Please specify these situations/constellations: [in patients with low grade urinary tract dilation (up to 2nd grade) and no UTI in medical history]
N/A

If you use antibiotic prophylaxis: which mediaction do you use? (multiple answers and subdivision into age groups possible) [Trimethoprim]
N/A

If you use antibiotic prophylaxis: which mediaction do you use? (multiple answers and subdivision into age groups possible) [Nitrofurantoin]
N/A

If you use antibiotic prophylaxis: which mediaction do you use? (multiple answers and subdivision into age groups possible) [Amoxicillin]
N/A

If you use antibiotic prophylaxis: which mediaction do you use? (multiple answers and subdivision into age groups possible) [Cefaclor]
N/A

If you use antibiotic prophylaxis: which mediaction do you use? (multiple answers and subdivision into age groups possible) [Amoxicillin/clavulanic acid]
N/A

If you use antibiotic prophylaxis: which mediaction do you use? (multiple answers and subdivision into age groups possible) [Other]
N/A

Trimethoprim: which time schedule do you use? [Day(s) before VUS/VCUG, how many?]
N/A

Trimethoprim: which time schedule do you use? [Day of VUS/VCUG]
N/A

Trimethoprim: which time schedule do you use? [Day(s) after VUS/VCUG, how many?]
N/A

Nitrofurantoin: which time schedule do you use? [Day(s) before VUS/VCUG, how many?]
N/A

Nitrofurantoin: which time schedule do you use? [Day of VUS/VCUG]
N/A

Nitrofurantoin: which time schedule do you use? [Day(s) after VUS/VCUG, how many?]
N/A

Amoxicillin: which time schedule do you use? [Day(s) before VUS/VCUG, how many?]
N/A

Amoxicillin: which time schedule do you use? [Day of VUS/VCUG]
N/A

Amoxicillin: which time schedule do you use? [Day(s) after VUS/VCUG, how many?]
N/A

Cefaclor: which time schedule do you use? [Days(s) before VUS/VCUG, how many?]
N/A

Cefaclor: which time schedule do you use? [Day of VUS/VCUG]
N/A

Cefaclor: which time schedule do you use? [Day(s) after VUS/VCUG, how many?]
N/A

Amoxicillin/clavulanic acid: in what dosage? (Amoxicillin portion) [prophylaktic (10-20 mg/Kg bodyweight/d)]
N/A

Amoxicillin/clavulanic acid: in what dosage? (Amoxicillin portion) [double prophylactic (20-40 mg/Kg bodyweight/d)]
N/A

Amoxicillin/clavulanic acid: in what dosage? (Amoxicillin portion) [therapeutic (80-100 mg/Kg bodyweight/d)]
N/A

Amoxicillin/clavulanic acid: which time schedule do you use? [Day(s) before VUS/VCUG]
N/A

Amoxicillin/clavulanic acid: which time schedule do you use? [Day of VUS/VCUG]
N/A

Amoxicillin/clavulanic acid: which time schedule do you use? [Day(s) after VUS/VCUG]
N/A

Other: which time schedule do you use? [Day(s) before VUS/VCUG, how many?]
N/A

Other: which time schedule do you use? [Day of VUS/VCUG]
N/A

Other: which time schedule do you use? [Day(s) after VUS/VCUG, how many?]
N/A

Indication for prophylaxis

Which patients receive periinterventional antibiotic prophylaxis? (multiple answers possible) [all]
N/A

Which patients receive periinterventional antibiotic prophylaxis? (multiple answers possible) [patients post urinary tract infection/pyelonephritis/urosepsis]
N/A

Which patients receive periinterventional antibiotic prophylaxis? (multiple answers possible) [patients with urinary tract dilation in ultrasound]
N/A

Which patients receive periinterventional antibiotic prophylaxis? (multiple answers possible) [patients with prenatal urinary tract dilation]
N/A

Which patients receive periinterventional antibiotic prophylaxis? (multiple answers possible) [patients post VUR surgery]
N/A

Which patients receive periinterventional antibiotic prophylaxis? (multiple answers possible) [none]
N/A

Are there situations or clinical findings in the patient where you deviate from your prophylaxis standard (medication, dose, duration, age of the patient)? (multiple answers possible, please specify in answer box) [in case of already known vesicoureteral reflux (VUR)]
N/A

Are there situations or clinical findings in the patient where you deviate from your prophylaxis standard (medication, dose, duration, age of the patient)? (multiple answers possible, please specify in answer box) [in patients with recurrent urinary tract infections (UTI)]
N/A

Are there situations or clinical findings in the patient where you deviate from your prophylaxis standard (medication, dose, duration, age of the patient)? (multiple answers possible, please specify in answer box) [if multidrug-resistant organisms (MDRO) are detected in urine cultures]
N/A

Are there situations or clinical findings in the patient where you deviate from your prophylaxis standard (medication, dose, duration, age of the patient)? (multiple answers possible, please specify in answer box) [in newborns]
N/A

Are there situations in which you use intravenous prophylaxis?
N/A

Voiding analysis

Are there differences in antibiotic prophylaxis between VUS and VCUG in your department?
N/A

Is a non-pathological urine strip test required in preparation for VUS/VCUG in your department?
N/A

Is a non-pathological urine culture required in preparation for VUS/VCUG in your department?
N/A

Do patients only receive post-procedure prophylaxis if vesicoureteral reflux (VUR) is detected in VUS/VCUG?
N/A

Do other MUS/MCU findings (e.g. urethral valves) have an influence on post-procedure prophylaxis?
N/A

End of survey

May we contact you personally if we have further questions?
N/A

Survey response 199

Response ID
201

Date submitted
2025-03-26 12:26:10

Last page
5

Start language
de

Seed
213800134

Date started
2025-03-26 12:09:12

Date last action
2025-03-26 12:26:10

Standard Operation Procedure

Who fills out this survey?
Pediatric Radiologist

Who fills out this survey? [Other]

In which type of hospital are you employed?
Community hospital (

In which type of hospital are you employed? [Other]

In which country do you work?
Schweiz

Does your department have a defined protocol for infection prophylaxis in the context of VUS/VCUG?
Yes

Has this protocol been transformed into a written standard operating procedure (SOP)?
Yes

Which department is the author of the SOP for infection prophylaxis in VUS/VCUG?
Pediatrics

Which department is the author of the SOP for infection prophylaxis in VUS/VCUG? [Other]

Antibiotic Prophylaxis

Are there situations/constellations in which you do not use antibiotic prophylaxis for VUS/VCUG?
Yes

Please specify these situations/constellations: [girl with first uncomplicated UTI without urinary tract dilation in ultrasound]
No

Please specify these situations/constellations: [boy with first uncomplicated UTI without urinary tract dilation in ultrasound]
No

Please specify these situations/constellations: [in patients with low grade urinary tract dilation (up to 2nd grade) and no UTI in medical history]
No

Please specify these situations/constellations: [Other]
Wunsch der Eltern

If you use antibiotic prophylaxis: which mediaction do you use? (multiple answers and subdivision into age groups possible) [Trimethoprim]
No

If you use antibiotic prophylaxis: which mediaction do you use? (multiple answers and subdivision into age groups possible) [Nitrofurantoin]
No

If you use antibiotic prophylaxis: which mediaction do you use? (multiple answers and subdivision into age groups possible) [Amoxicillin]
No

If you use antibiotic prophylaxis: which mediaction do you use? (multiple answers and subdivision into age groups possible) [Cefaclor]
No

If you use antibiotic prophylaxis: which mediaction do you use? (multiple answers and subdivision into age groups possible) [Amoxicillin/clavulanic acid]
No

If you use antibiotic prophylaxis: which mediaction do you use? (multiple answers and subdivision into age groups possible) [Other]
Yes

Trimethoprim: which time schedule do you use? [Day(s) before VUS/VCUG, how many?]
N/A

Trimethoprim: which time schedule do you use? [Day of VUS/VCUG]
N/A

Trimethoprim: which time schedule do you use? [Day(s) after VUS/VCUG, how many?]
N/A

Nitrofurantoin: which time schedule do you use? [Day(s) before VUS/VCUG, how many?]
N/A

Nitrofurantoin: which time schedule do you use? [Day of VUS/VCUG]
N/A

Nitrofurantoin: which time schedule do you use? [Day(s) after VUS/VCUG, how many?]
N/A

Amoxicillin: which time schedule do you use? [Day(s) before VUS/VCUG, how many?]
N/A

Amoxicillin: which time schedule do you use? [Day of VUS/VCUG]
N/A

Amoxicillin: which time schedule do you use? [Day(s) after VUS/VCUG, how many?]
N/A

Cefaclor: which time schedule do you use? [Days(s) before VUS/VCUG, how many?]
N/A

Cefaclor: which time schedule do you use? [Day of VUS/VCUG]
N/A

Cefaclor: which time schedule do you use? [Day(s) after VUS/VCUG, how many?]
N/A

Amoxicillin/clavulanic acid: in what dosage? (Amoxicillin portion) [prophylaktic (10-20 mg/Kg bodyweight/d)]
N/A

Amoxicillin/clavulanic acid: in what dosage? (Amoxicillin portion) [double prophylactic (20-40 mg/Kg bodyweight/d)]
N/A

Amoxicillin/clavulanic acid: in what dosage? (Amoxicillin portion) [therapeutic (80-100 mg/Kg bodyweight/d)]
N/A

Amoxicillin/clavulanic acid: which time schedule do you use? [Day(s) before VUS/VCUG]
N/A

Amoxicillin/clavulanic acid: which time schedule do you use? [Day of VUS/VCUG]
N/A

Amoxicillin/clavulanic acid: which time schedule do you use? [Day(s) after VUS/VCUG]
N/A

Other: which medication and in what dosage?
Nopil/ Cotrimoxazol

Other: for which age group do you use this medication?
Alle

Other: which time schedule do you use? [Day(s) before VUS/VCUG, how many?]
Yes

Other: which time schedule do you use? [Day(s) before VUS/VCUG, how many?][Comment]
ab Indikationsstellung zum MUS/MCU

Other: which time schedule do you use? [Day of VUS/VCUG]
No

Other: which time schedule do you use? [Day of VUS/VCUG][Comment]

Other: which time schedule do you use? [Day(s) after VUS/VCUG, how many?]
Yes

Other: which time schedule do you use? [Day(s) after VUS/VCUG, how many?][Comment]
1

Indication for prophylaxis

Which patients receive periinterventional antibiotic prophylaxis? (multiple answers possible) [all]
Yes

Which patients receive periinterventional antibiotic prophylaxis? (multiple answers possible) [patients post urinary tract infection/pyelonephritis/urosepsis]
N/A

Which patients receive periinterventional antibiotic prophylaxis? (multiple answers possible) [patients with urinary tract dilation in ultrasound]
N/A

Which patients receive periinterventional antibiotic prophylaxis? (multiple answers possible) [patients with prenatal urinary tract dilation]
N/A

Which patients receive periinterventional antibiotic prophylaxis? (multiple answers possible) [patients post VUR surgery]
N/A

Which patients receive periinterventional antibiotic prophylaxis? (multiple answers possible) [none]
N/A

Are there situations or clinical findings in the patient where you deviate from your prophylaxis standard (medication, dose, duration, age of the patient)? (multiple answers possible, please specify in answer box) [in case of already known vesicoureteral reflux (VUR)]
No

Are there situations or clinical findings in the patient where you deviate from your prophylaxis standard (medication, dose, duration, age of the patient)? (multiple answers possible, please specify in answer box) [in case of already known vesicoureteral reflux (VUR)][Comment]

Are there situations or clinical findings in the patient where you deviate from your prophylaxis standard (medication, dose, duration, age of the patient)? (multiple answers possible, please specify in answer box) [in patients with recurrent urinary tract infections (UTI)]
No

Are there situations or clinical findings in the patient where you deviate from your prophylaxis standard (medication, dose, duration, age of the patient)? (multiple answers possible, please specify in answer box) [in patients with recurrent urinary tract infections (UTI)][Comment]

Are there situations or clinical findings in the patient where you deviate from your prophylaxis standard (medication, dose, duration, age of the patient)? (multiple answers possible, please specify in answer box) [if multidrug-resistant organisms (MDRO) are detected in urine cultures]
Yes

Are there situations or clinical findings in the patient where you deviate from your prophylaxis standard (medication, dose, duration, age of the patient)? (multiple answers possible, please specify in answer box) [if multidrug-resistant organisms (MDRO) are detected in urine cultures][Comment]

Are there situations or clinical findings in the patient where you deviate from your prophylaxis standard (medication, dose, duration, age of the patient)? (multiple answers possible, please specify in answer box) [in newborns]
No

Are there situations or clinical findings in the patient where you deviate from your prophylaxis standard (medication, dose, duration, age of the patient)? (multiple answers possible, please specify in answer box) [in newborns][Comment]

Are there situations or clinical findings in the patient where you deviate from your prophylaxis standard (medication, dose, duration, age of the patient)? (multiple answers possible, please specify in answer box) [Other]

Are there situations or clinical findings in the patient where you deviate from your prophylaxis standard (medication, dose, duration, age of the patient)? (multiple answers possible, please specify in answer box) [Other comment]

Are there situations in which you use intravenous prophylaxis?
No

Voiding analysis

Are there differences in antibiotic prophylaxis between VUS and VCUG in your department?
No

Is a non-pathological urine strip test required in preparation for VUS/VCUG in your department?
No

Is a non-pathological urine culture required in preparation for VUS/VCUG in your department?
No

Do patients only receive post-procedure prophylaxis if vesicoureteral reflux (VUR) is detected in VUS/VCUG?
No

Do other MUS/MCU findings (e.g. urethral valves) have an influence on post-procedure prophylaxis?
No

End of survey

Thank you for taking the time to answer this survey!

May we contact you personally if we have further questions?
Yes

E-mail address:
andrea.sommer@ksw.ch

Survey response 200

Response ID
202

Last page
3

Start language
en

Seed
952544046

Date started
2025-03-26 14:40:52

Date last action
2025-03-26 15:15:37

Standard Operation Procedure

Who fills out this survey?
Pediatric Radiologist

Who fills out this survey? [Other]

In which type of hospital are you employed?
University Hospital

In which type of hospital are you employed? [Other]

In which country do you work?
Germany

Does your department have a defined protocol for infection prophylaxis in the context of VUS/VCUG?
No

Has this protocol been transformed into a written standard operating procedure (SOP)?
N/A

Antibiotic Prophylaxis

Are there situations/constellations in which you do not use antibiotic prophylaxis for VUS/VCUG?
Yes

Please specify these situations/constellations: [girl with first uncomplicated UTI without urinary tract dilation in ultrasound]
No

Please specify these situations/constellations: [boy with first uncomplicated UTI without urinary tract dilation in ultrasound]
No

Please specify these situations/constellations: [in patients with low grade urinary tract dilation (up to 2nd grade) and no UTI in medical history]
No

Please specify these situations/constellations: [Other]
the referring doctors take care of prophylaxis

If you use antibiotic prophylaxis: which mediaction do you use? (multiple answers and subdivision into age groups possible) [Trimethoprim]
No

If you use antibiotic prophylaxis: which mediaction do you use? (multiple answers and subdivision into age groups possible) [Nitrofurantoin]
No

If you use antibiotic prophylaxis: which mediaction do you use? (multiple answers and subdivision into age groups possible) [Amoxicillin]
No

If you use antibiotic prophylaxis: which mediaction do you use? (multiple answers and subdivision into age groups possible) [Cefaclor]
No

If you use antibiotic prophylaxis: which mediaction do you use? (multiple answers and subdivision into age groups possible) [Amoxicillin/clavulanic acid]
No

If you use antibiotic prophylaxis: which mediaction do you use? (multiple answers and subdivision into age groups possible) [Other]
No

Trimethoprim: which time schedule do you use? [Day(s) before VUS/VCUG, how many?]
N/A

Trimethoprim: which time schedule do you use? [Day of VUS/VCUG]
N/A

Trimethoprim: which time schedule do you use? [Day(s) after VUS/VCUG, how many?]
N/A

Nitrofurantoin: which time schedule do you use? [Day(s) before VUS/VCUG, how many?]
N/A

Nitrofurantoin: which time schedule do you use? [Day of VUS/VCUG]
N/A

Nitrofurantoin: which time schedule do you use? [Day(s) after VUS/VCUG, how many?]
N/A

Amoxicillin: which time schedule do you use? [Day(s) before VUS/VCUG, how many?]
N/A

Amoxicillin: which time schedule do you use? [Day of VUS/VCUG]
N/A

Amoxicillin: which time schedule do you use? [Day(s) after VUS/VCUG, how many?]
N/A

Cefaclor: which time schedule do you use? [Days(s) before VUS/VCUG, how many?]
N/A

Cefaclor: which time schedule do you use? [Day of VUS/VCUG]
N/A

Cefaclor: which time schedule do you use? [Day(s) after VUS/VCUG, how many?]
N/A

Amoxicillin/clavulanic acid: in what dosage? (Amoxicillin portion) [prophylaktic (10-20 mg/Kg bodyweight/d)]
N/A

Amoxicillin/clavulanic acid: in what dosage? (Amoxicillin portion) [double prophylactic (20-40 mg/Kg bodyweight/d)]
N/A

Amoxicillin/clavulanic acid: in what dosage? (Amoxicillin portion) [therapeutic (80-100 mg/Kg bodyweight/d)]
N/A

Amoxicillin/clavulanic acid: which time schedule do you use? [Day(s) before VUS/VCUG]
N/A

Amoxicillin/clavulanic acid: which time schedule do you use? [Day of VUS/VCUG]
N/A

Amoxicillin/clavulanic acid: which time schedule do you use? [Day(s) after VUS/VCUG]
N/A

Other: which time schedule do you use? [Day(s) before VUS/VCUG, how many?]
N/A

Other: which time schedule do you use? [Day of VUS/VCUG]
N/A

Other: which time schedule do you use? [Day(s) after VUS/VCUG, how many?]
N/A

Indication for prophylaxis

Which patients receive periinterventional antibiotic prophylaxis? (multiple answers possible) [all]
No

Which patients receive periinterventional antibiotic prophylaxis? (multiple answers possible) [patients post urinary tract infection/pyelonephritis/urosepsis]
No

Which patients receive periinterventional antibiotic prophylaxis? (multiple answers possible) [patients with urinary tract dilation in ultrasound]
No

Which patients receive periinterventional antibiotic prophylaxis? (multiple answers possible) [patients with prenatal urinary tract dilation]
No

Which patients receive periinterventional antibiotic prophylaxis? (multiple answers possible) [patients post VUR surgery]
No

Which patients receive periinterventional antibiotic prophylaxis? (multiple answers possible) [none]
No

Which patients receive periinterventional antibiotic prophylaxis? (multiple answers possible) [Other]
at the discretion of referring doctor

Are there situations or clinical findings in the patient where you deviate from your prophylaxis standard (medication, dose, duration, age of the patient)? (multiple answers possible, please specify in answer box) [in case of already known vesicoureteral reflux (VUR)]
No

Are there situations or clinical findings in the patient where you deviate from your prophylaxis standard (medication, dose, duration, age of the patient)? (multiple answers possible, please specify in answer box) [in case of already known vesicoureteral reflux (VUR)][Comment]

Are there situations or clinical findings in the patient where you deviate from your prophylaxis standard (medication, dose, duration, age of the patient)? (multiple answers possible, please specify in answer box) [in patients with recurrent urinary tract infections (UTI)]
No

Are there situations or clinical findings in the patient where you deviate from your prophylaxis standard (medication, dose, duration, age of the patient)? (multiple answers possible, please specify in answer box) [in patients with recurrent urinary tract infections (UTI)][Comment]

Are there situations or clinical findings in the patient where you deviate from your prophylaxis standard (medication, dose, duration, age of the patient)? (multiple answers possible, please specify in answer box) [if multidrug-resistant organisms (MDRO) are detected in urine cultures]
No

Are there situations or clinical findings in the patient where you deviate from your prophylaxis standard (medication, dose, duration, age of the patient)? (multiple answers possible, please specify in answer box) [if multidrug-resistant organisms (MDRO) are detected in urine cultures][Comment]

Are there situations or clinical findings in the patient where you deviate from your prophylaxis standard (medication, dose, duration, age of the patient)? (multiple answers possible, please specify in answer box) [in newborns]
No

Are there situations or clinical findings in the patient where you deviate from your prophylaxis standard (medication, dose, duration, age of the patient)? (multiple answers possible, please specify in answer box) [in newborns][Comment]

Are there situations or clinical findings in the patient where you deviate from your prophylaxis standard (medication, dose, duration, age of the patient)? (multiple answers possible, please specify in answer box) [Other]
referring doctors

Are there situations or clinical findings in the patient where you deviate from your prophylaxis standard (medication, dose, duration, age of the patient)? (multiple answers possible, please specify in answer box) [Other comment]
our referring doctors take care of this

Are there situations in which you use intravenous prophylaxis?
No

Voiding analysis

Are there differences in antibiotic prophylaxis between VUS and VCUG in your department?
N/A

Is a non-pathological urine strip test required in preparation for VUS/VCUG in your department?
N/A

Is a non-pathological urine culture required in preparation for VUS/VCUG in your department?
N/A

Do patients only receive post-procedure prophylaxis if vesicoureteral reflux (VUR) is detected in VUS/VCUG?
N/A

Do other MUS/MCU findings (e.g. urethral valves) have an influence on post-procedure prophylaxis?
N/A

End of survey

May we contact you personally if we have further questions?
N/A

Survey response 201

Response ID
203

Date submitted
2025-03-26 16:33:28

Last page
5

Start language
en

Seed
809731853

Date started
2025-03-26 16:28:43

Date last action
2025-03-26 16:33:28

Standard Operation Procedure

Who fills out this survey?
Pediatric Radiologist

Who fills out this survey? [Other]

In which type of hospital are you employed?
University Hospital

In which type of hospital are you employed? [Other]

In which country do you work?
United Kingdom

Does your department have a defined protocol for infection prophylaxis in the context of VUS/VCUG?
Yes

Has this protocol been transformed into a written standard operating procedure (SOP)?
Yes

Which department is the author of the SOP for infection prophylaxis in VUS/VCUG?
Pediatrics

Which department is the author of the SOP for infection prophylaxis in VUS/VCUG? [Other]

Antibiotic Prophylaxis

Are there situations/constellations in which you do not use antibiotic prophylaxis for VUS/VCUG?
No

Please specify these situations/constellations: [girl with first uncomplicated UTI without urinary tract dilation in ultrasound]
N/A

Please specify these situations/constellations: [boy with first uncomplicated UTI without urinary tract dilation in ultrasound]
N/A

Please specify these situations/constellations: [in patients with low grade urinary tract dilation (up to 2nd grade) and no UTI in medical history]
N/A

If you use antibiotic prophylaxis: which mediaction do you use? (multiple answers and subdivision into age groups possible) [Trimethoprim]
Yes

If you use antibiotic prophylaxis: which mediaction do you use? (multiple answers and subdivision into age groups possible) [Nitrofurantoin]
No

If you use antibiotic prophylaxis: which mediaction do you use? (multiple answers and subdivision into age groups possible) [Amoxicillin]
No

If you use antibiotic prophylaxis: which mediaction do you use? (multiple answers and subdivision into age groups possible) [Cefaclor]
No

If you use antibiotic prophylaxis: which mediaction do you use? (multiple answers and subdivision into age groups possible) [Amoxicillin/clavulanic acid]
No

If you use antibiotic prophylaxis: which mediaction do you use? (multiple answers and subdivision into age groups possible) [Other]
No

Trimethoprim: in what dosage?
double prophylakctic (4 mg/kg bodyweight/d)

Trimethoprim: in what dosage? [Other]

Trimethoprim: for which age group do you use trimethoprim?

Trimethoprim: which time schedule do you use? [Day(s) before VUS/VCUG, how many?]
Yes

Trimethoprim: which time schedule do you use? [Day(s) before VUS/VCUG, how many?][Comment]
1

Trimethoprim: which time schedule do you use? [Day of VUS/VCUG]
Yes

Trimethoprim: which time schedule do you use? [Day of VUS/VCUG][Comment]

Trimethoprim: which time schedule do you use? [Day(s) after VUS/VCUG, how many?]
Yes

Trimethoprim: which time schedule do you use? [Day(s) after VUS/VCUG, how many?][Comment]
1

Nitrofurantoin: which time schedule do you use? [Day(s) before VUS/VCUG, how many?]
N/A

Nitrofurantoin: which time schedule do you use? [Day of VUS/VCUG]
N/A

Nitrofurantoin: which time schedule do you use? [Day(s) after VUS/VCUG, how many?]
N/A

Amoxicillin: which time schedule do you use? [Day(s) before VUS/VCUG, how many?]
N/A

Amoxicillin: which time schedule do you use? [Day of VUS/VCUG]
N/A

Amoxicillin: which time schedule do you use? [Day(s) after VUS/VCUG, how many?]
N/A

Cefaclor: which time schedule do you use? [Days(s) before VUS/VCUG, how many?]
N/A

Cefaclor: which time schedule do you use? [Day of VUS/VCUG]
N/A

Cefaclor: which time schedule do you use? [Day(s) after VUS/VCUG, how many?]
N/A

Amoxicillin/clavulanic acid: in what dosage? (Amoxicillin portion) [prophylaktic (10-20 mg/Kg bodyweight/d)]
N/A

Amoxicillin/clavulanic acid: in what dosage? (Amoxicillin portion) [double prophylactic (20-40 mg/Kg bodyweight/d)]
N/A

Amoxicillin/clavulanic acid: in what dosage? (Amoxicillin portion) [therapeutic (80-100 mg/Kg bodyweight/d)]
N/A

Amoxicillin/clavulanic acid: which time schedule do you use? [Day(s) before VUS/VCUG]
N/A

Amoxicillin/clavulanic acid: which time schedule do you use? [Day of VUS/VCUG]
N/A

Amoxicillin/clavulanic acid: which time schedule do you use? [Day(s) after VUS/VCUG]
N/A

Other: which time schedule do you use? [Day(s) before VUS/VCUG, how many?]
N/A

Other: which time schedule do you use? [Day of VUS/VCUG]
N/A

Other: which time schedule do you use? [Day(s) after VUS/VCUG, how many?]
N/A

Indication for prophylaxis

Which patients receive periinterventional antibiotic prophylaxis? (multiple answers possible) [all]
No

Which patients receive periinterventional antibiotic prophylaxis? (multiple answers possible) [patients post urinary tract infection/pyelonephritis/urosepsis]
Yes

Which patients receive periinterventional antibiotic prophylaxis? (multiple answers possible) [patients with urinary tract dilation in ultrasound]
Yes

Which patients receive periinterventional antibiotic prophylaxis? (multiple answers possible) [patients with prenatal urinary tract dilation]
Yes

Which patients receive periinterventional antibiotic prophylaxis? (multiple answers possible) [patients post VUR surgery]
Yes

Which patients receive periinterventional antibiotic prophylaxis? (multiple answers possible) [none]
No

Which patients receive periinterventional antibiotic prophylaxis? (multiple answers possible) [Other]

patients status post urinary tract infection/pyelonephritis/urosepsis: what is the minimum time between infection and VUS/VCUG? (in days)
5

In patients with urinary tract dilation in ultrasound: from what degree on? Image from Beetz R, Bokenkamp A, Brandis M et al (2001) Diagnosis of congenital dilatation of the urinary tract. Consensus Group of the Pediatric Nephrology Working Society in cooperation with the Pediatric Urology Working Group of the German Society of Urology and with the Pediatric Urology Working Society in the Germany Society of Pediatric Surgery. Urologe A 40:495–507 (quiz 8–9)
grade 1

In patients with prenatal urinary tract dilation: from what degree on? Image from Beetz R, Bokenkamp A, Brandis M et al (2001) Diagnosis of congenital dilatation of the urinary tract. Consensus Group of the Pediatric Nephrology Working Society in cooperation with the Pediatric Urology Working Group of the German Society of Urology and with the Pediatric Urology Working Society in the Germany Society of Pediatric Surgery. Urologe A 40:495–507 (quiz 8–9)
grade 1

Are there situations or clinical findings in the patient where you deviate from your prophylaxis standard (medication, dose, duration, age of the patient)? (multiple answers possible, please specify in answer box) [in case of already known vesicoureteral reflux (VUR)]
No

Are there situations or clinical findings in the patient where you deviate from your prophylaxis standard (medication, dose, duration, age of the patient)? (multiple answers possible, please specify in answer box) [in case of already known vesicoureteral reflux (VUR)][Comment]

Are there situations or clinical findings in the patient where you deviate from your prophylaxis standard (medication, dose, duration, age of the patient)? (multiple answers possible, please specify in answer box) [in patients with recurrent urinary tract infections (UTI)]
No

Are there situations or clinical findings in the patient where you deviate from your prophylaxis standard (medication, dose, duration, age of the patient)? (multiple answers possible, please specify in answer box) [in patients with recurrent urinary tract infections (UTI)][Comment]

Are there situations or clinical findings in the patient where you deviate from your prophylaxis standard (medication, dose, duration, age of the patient)? (multiple answers possible, please specify in answer box) [if multidrug-resistant organisms (MDRO) are detected in urine cultures]
Yes

Are there situations or clinical findings in the patient where you deviate from your prophylaxis standard (medication, dose, duration, age of the patient)? (multiple answers possible, please specify in answer box) [if multidrug-resistant organisms (MDRO) are detected in urine cultures][Comment]
whatever sensitivity exists

Are there situations or clinical findings in the patient where you deviate from your prophylaxis standard (medication, dose, duration, age of the patient)? (multiple answers possible, please specify in answer box) [in newborns]
No

Are there situations or clinical findings in the patient where you deviate from your prophylaxis standard (medication, dose, duration, age of the patient)? (multiple answers possible, please specify in answer box) [in newborns][Comment]

Are there situations or clinical findings in the patient where you deviate from your prophylaxis standard (medication, dose, duration, age of the patient)? (multiple answers possible, please specify in answer box) [Other]

Are there situations or clinical findings in the patient where you deviate from your prophylaxis standard (medication, dose, duration, age of the patient)? (multiple answers possible, please specify in answer box) [Other comment]

Are there situations in which you use intravenous prophylaxis?
No

Voiding analysis

Are there differences in antibiotic prophylaxis between VUS and VCUG in your department?
No

Is a non-pathological urine strip test required in preparation for VUS/VCUG in your department?
No

Is a non-pathological urine culture required in preparation for VUS/VCUG in your department?
No

Do patients only receive post-procedure prophylaxis if vesicoureteral reflux (VUR) is detected in VUS/VCUG?
No

Do other MUS/MCU findings (e.g. urethral valves) have an influence on post-procedure prophylaxis?
Yes

Please specify:
if not on continuous ABx, treatment continued until D/W clinicians

End of survey

Thank you for taking the time to answer this survey!

May we contact you personally if we have further questions?
No

Survey response 202

Response ID
204

Date submitted
2025-03-26 17:24:48

Last page
5

Start language
en

Seed
604173538

Date started
2025-03-26 17:17:02

Date last action
2025-03-26 17:24:48

Standard Operation Procedure

Who fills out this survey?
Pediatric nephrologist

Who fills out this survey? [Other]

In which type of hospital are you employed?
Community hospital (

In which type of hospital are you employed? [Other]

In which country do you work?
România

Does your department have a defined protocol for infection prophylaxis in the context of VUS/VCUG?
Yes

Has this protocol been transformed into a written standard operating procedure (SOP)?
Yes

Which department is the author of the SOP for infection prophylaxis in VUS/VCUG?
Pediatrics

Which department is the author of the SOP for infection prophylaxis in VUS/VCUG? [Other]

Antibiotic Prophylaxis

Are there situations/constellations in which you do not use antibiotic prophylaxis for VUS/VCUG?
No

Please specify these situations/constellations: [girl with first uncomplicated UTI without urinary tract dilation in ultrasound]
N/A

Please specify these situations/constellations: [boy with first uncomplicated UTI without urinary tract dilation in ultrasound]
N/A

Please specify these situations/constellations: [in patients with low grade urinary tract dilation (up to 2nd grade) and no UTI in medical history]
N/A

If you use antibiotic prophylaxis: which mediaction do you use? (multiple answers and subdivision into age groups possible) [Trimethoprim]
Yes

If you use antibiotic prophylaxis: which mediaction do you use? (multiple answers and subdivision into age groups possible) [Nitrofurantoin]
Yes

If you use antibiotic prophylaxis: which mediaction do you use? (multiple answers and subdivision into age groups possible) [Amoxicillin]
No

If you use antibiotic prophylaxis: which mediaction do you use? (multiple answers and subdivision into age groups possible) [Cefaclor]
Yes

If you use antibiotic prophylaxis: which mediaction do you use? (multiple answers and subdivision into age groups possible) [Amoxicillin/clavulanic acid]
No

If you use antibiotic prophylaxis: which mediaction do you use? (multiple answers and subdivision into age groups possible) [Other]
No

Trimethoprim: in what dosage?
therapeutic (6 mg/kg bodyweight/d)

Trimethoprim: in what dosage? [Other]

Trimethoprim: for which age group do you use trimethoprim?
> 3 months

Trimethoprim: which time schedule do you use? [Day(s) before VUS/VCUG, how many?]
Yes

Trimethoprim: which time schedule do you use? [Day(s) before VUS/VCUG, how many?][Comment]
2

Trimethoprim: which time schedule do you use? [Day of VUS/VCUG]
Yes

Trimethoprim: which time schedule do you use? [Day of VUS/VCUG][Comment]

Trimethoprim: which time schedule do you use? [Day(s) after VUS/VCUG, how many?]
Yes

Trimethoprim: which time schedule do you use? [Day(s) after VUS/VCUG, how many?][Comment]
2

Nitrofurantoin: in what dosage?
therapeutic (5 mg/kg bodyweight/d)

Nitrofurantoin: in what dosage? [Other]

Nitrofurantoin: for which age group do you use nitrofurantoin?
> 3 months

Nitrofurantoin: which time schedule do you use? [Day(s) before VUS/VCUG, how many?]
Yes

Nitrofurantoin: which time schedule do you use? [Day(s) before VUS/VCUG, how many?][Comment]
2

Nitrofurantoin: which time schedule do you use? [Day of VUS/VCUG]
Yes

Nitrofurantoin: which time schedule do you use? [Day of VUS/VCUG][Comment]

Nitrofurantoin: which time schedule do you use? [Day(s) after VUS/VCUG, how many?]
Yes

Nitrofurantoin: which time schedule do you use? [Day(s) after VUS/VCUG, how many?][Comment]
2

Amoxicillin: which time schedule do you use? [Day(s) before VUS/VCUG, how many?]
N/A

Amoxicillin: which time schedule do you use? [Day of VUS/VCUG]
N/A

Amoxicillin: which time schedule do you use? [Day(s) after VUS/VCUG, how many?]
N/A

Cefaclor: in what dosage?
double prophylactic (20 mg/kg bodyweight/d)

Cefaclor: in what dosage? [Other]

Cefaclor: for which age group do you use cefaclor?
> 1 months

Cefaclor: which time schedule do you use? [Days(s) before VUS/VCUG, how many?]
Yes

Cefaclor: which time schedule do you use? [Days(s) before VUS/VCUG, how many?][Comment]
2

Cefaclor: which time schedule do you use? [Day of VUS/VCUG]
Yes

Cefaclor: which time schedule do you use? [Day of VUS/VCUG][Comment]

Cefaclor: which time schedule do you use? [Day(s) after VUS/VCUG, how many?]
Yes

Cefaclor: which time schedule do you use? [Day(s) after VUS/VCUG, how many?][Comment]
2

Amoxicillin/clavulanic acid: in what dosage? (Amoxicillin portion) [prophylaktic (10-20 mg/Kg bodyweight/d)]
N/A

Amoxicillin/clavulanic acid: in what dosage? (Amoxicillin portion) [double prophylactic (20-40 mg/Kg bodyweight/d)]
N/A

Amoxicillin/clavulanic acid: in what dosage? (Amoxicillin portion) [therapeutic (80-100 mg/Kg bodyweight/d)]
N/A

Amoxicillin/clavulanic acid: which time schedule do you use? [Day(s) before VUS/VCUG]
N/A

Amoxicillin/clavulanic acid: which time schedule do you use? [Day of VUS/VCUG]
N/A

Amoxicillin/clavulanic acid: which time schedule do you use? [Day(s) after VUS/VCUG]
N/A

Other: which time schedule do you use? [Day(s) before VUS/VCUG, how many?]
N/A

Other: which time schedule do you use? [Day of VUS/VCUG]
N/A

Other: which time schedule do you use? [Day(s) after VUS/VCUG, how many?]
N/A

Indication for prophylaxis

Which patients receive periinterventional antibiotic prophylaxis? (multiple answers possible) [all]
No

Which patients receive periinterventional antibiotic prophylaxis? (multiple answers possible) [patients post urinary tract infection/pyelonephritis/urosepsis]
No

Which patients receive periinterventional antibiotic prophylaxis? (multiple answers possible) [patients with urinary tract dilation in ultrasound]
Yes

Which patients receive periinterventional antibiotic prophylaxis? (multiple answers possible) [patients with prenatal urinary tract dilation]
Yes

Which patients receive periinterventional antibiotic prophylaxis? (multiple answers possible) [patients post VUR surgery]
No

Which patients receive periinterventional antibiotic prophylaxis? (multiple answers possible) [none]
No

Which patients receive periinterventional antibiotic prophylaxis? (multiple answers possible) [Other]

In patients with urinary tract dilation in ultrasound: from what degree on? Image from Beetz R, Bokenkamp A, Brandis M et al (2001) Diagnosis of congenital dilatation of the urinary tract. Consensus Group of the Pediatric Nephrology Working Society in cooperation with the Pediatric Urology Working Group of the German Society of Urology and with the Pediatric Urology Working Society in the Germany Society of Pediatric Surgery. Urologe A 40:495–507 (quiz 8–9)
grade 4

In patients with prenatal urinary tract dilation: from what degree on? Image from Beetz R, Bokenkamp A, Brandis M et al (2001) Diagnosis of congenital dilatation of the urinary tract. Consensus Group of the Pediatric Nephrology Working Society in cooperation with the Pediatric Urology Working Group of the German Society of Urology and with the Pediatric Urology Working Society in the Germany Society of Pediatric Surgery. Urologe A 40:495–507 (quiz 8–9)
grade 4

Are there situations or clinical findings in the patient where you deviate from your prophylaxis standard (medication, dose, duration, age of the patient)? (multiple answers possible, please specify in answer box) [in case of already known vesicoureteral reflux (VUR)]
No

Are there situations or clinical findings in the patient where you deviate from your prophylaxis standard (medication, dose, duration, age of the patient)? (multiple answers possible, please specify in answer box) [in case of already known vesicoureteral reflux (VUR)][Comment]

Are there situations or clinical findings in the patient where you deviate from your prophylaxis standard (medication, dose, duration, age of the patient)? (multiple answers possible, please specify in answer box) [in patients with recurrent urinary tract infections (UTI)]
No

Are there situations or clinical findings in the patient where you deviate from your prophylaxis standard (medication, dose, duration, age of the patient)? (multiple answers possible, please specify in answer box) [in patients with recurrent urinary tract infections (UTI)][Comment]

Are there situations or clinical findings in the patient where you deviate from your prophylaxis standard (medication, dose, duration, age of the patient)? (multiple answers possible, please specify in answer box) [if multidrug-resistant organisms (MDRO) are detected in urine cultures]
Yes

Are there situations or clinical findings in the patient where you deviate from your prophylaxis standard (medication, dose, duration, age of the patient)? (multiple answers possible, please specify in answer box) [if multidrug-resistant organisms (MDRO) are detected in urine cultures][Comment]

Are there situations or clinical findings in the patient where you deviate from your prophylaxis standard (medication, dose, duration, age of the patient)? (multiple answers possible, please specify in answer box) [in newborns]
No

Are there situations or clinical findings in the patient where you deviate from your prophylaxis standard (medication, dose, duration, age of the patient)? (multiple answers possible, please specify in answer box) [in newborns][Comment]

Are there situations or clinical findings in the patient where you deviate from your prophylaxis standard (medication, dose, duration, age of the patient)? (multiple answers possible, please specify in answer box) [Other]

Are there situations or clinical findings in the patient where you deviate from your prophylaxis standard (medication, dose, duration, age of the patient)? (multiple answers possible, please specify in answer box) [Other comment]

Are there situations in which you use intravenous prophylaxis?
No

Voiding analysis

Are there differences in antibiotic prophylaxis between VUS and VCUG in your department?
No

Is a non-pathological urine strip test required in preparation for VUS/VCUG in your department?
Yes

What is the maximum amount of days accepted for urine strip test to be performed prior to the exam?
48 h

Is a non-pathological urine culture required in preparation for VUS/VCUG in your department?
Yes

What is the maximum amount of days accepted for urine culture to be performed prior to the exam?
48 h

Do patients only receive post-procedure prophylaxis if vesicoureteral reflux (VUR) is detected in VUS/VCUG?
Yes

Please explain your approach:
VUR > 3 degere

Do other MUS/MCU findings (e.g. urethral valves) have an influence on post-procedure prophylaxis?
Yes

Please specify:
VUP

End of survey

Thank you for taking the time to answer this survey!

May we contact you personally if we have further questions?
Yes

E-mail address:
Codruța.iliescu@arcadiamedical.ro

Survey response 203

Response ID
205

Last page
0

Start language
de

Seed
289162550

Date started
2025-03-27 09:14:32

Date last action
2025-03-27 09:14:32

Standard Operation Procedure

Does your department have a defined protocol for infection prophylaxis in the context of VUS/VCUG?
N/A

Has this protocol been transformed into a written standard operating procedure (SOP)?
N/A

Antibiotic Prophylaxis

Are there situations/constellations in which you do not use antibiotic prophylaxis for VUS/VCUG?
N/A

Please specify these situations/constellations: [girl with first uncomplicated UTI without urinary tract dilation in ultrasound]
N/A

Please specify these situations/constellations: [boy with first uncomplicated UTI without urinary tract dilation in ultrasound]
N/A

Please specify these situations/constellations: [in patients with low grade urinary tract dilation (up to 2nd grade) and no UTI in medical history]
N/A

If you use antibiotic prophylaxis: which mediaction do you use? (multiple answers and subdivision into age groups possible) [Trimethoprim]
N/A

If you use antibiotic prophylaxis: which mediaction do you use? (multiple answers and subdivision into age groups possible) [Nitrofurantoin]
N/A

If you use antibiotic prophylaxis: which mediaction do you use? (multiple answers and subdivision into age groups possible) [Amoxicillin]
N/A

If you use antibiotic prophylaxis: which mediaction do you use? (multiple answers and subdivision into age groups possible) [Cefaclor]
N/A

If you use antibiotic prophylaxis: which mediaction do you use? (multiple answers and subdivision into age groups possible) [Amoxicillin/clavulanic acid]
N/A

If you use antibiotic prophylaxis: which mediaction do you use? (multiple answers and subdivision into age groups possible) [Other]
N/A

Trimethoprim: which time schedule do you use? [Day(s) before VUS/VCUG, how many?]
N/A

Trimethoprim: which time schedule do you use? [Day of VUS/VCUG]
N/A

Trimethoprim: which time schedule do you use? [Day(s) after VUS/VCUG, how many?]
N/A

Nitrofurantoin: which time schedule do you use? [Day(s) before VUS/VCUG, how many?]
N/A

Nitrofurantoin: which time schedule do you use? [Day of VUS/VCUG]
N/A

Nitrofurantoin: which time schedule do you use? [Day(s) after VUS/VCUG, how many?]
N/A

Amoxicillin: which time schedule do you use? [Day(s) before VUS/VCUG, how many?]
N/A

Amoxicillin: which time schedule do you use? [Day of VUS/VCUG]
N/A

Amoxicillin: which time schedule do you use? [Day(s) after VUS/VCUG, how many?]
N/A

Cefaclor: which time schedule do you use? [Days(s) before VUS/VCUG, how many?]
N/A

Cefaclor: which time schedule do you use? [Day of VUS/VCUG]
N/A

Cefaclor: which time schedule do you use? [Day(s) after VUS/VCUG, how many?]
N/A

Amoxicillin/clavulanic acid: in what dosage? (Amoxicillin portion) [prophylaktic (10-20 mg/Kg bodyweight/d)]
N/A

Amoxicillin/clavulanic acid: in what dosage? (Amoxicillin portion) [double prophylactic (20-40 mg/Kg bodyweight/d)]
N/A

Amoxicillin/clavulanic acid: in what dosage? (Amoxicillin portion) [therapeutic (80-100 mg/Kg bodyweight/d)]
N/A

Amoxicillin/clavulanic acid: which time schedule do you use? [Day(s) before VUS/VCUG]
N/A

Amoxicillin/clavulanic acid: which time schedule do you use? [Day of VUS/VCUG]
N/A

Amoxicillin/clavulanic acid: which time schedule do you use? [Day(s) after VUS/VCUG]
N/A

Other: which time schedule do you use? [Day(s) before VUS/VCUG, how many?]
N/A

Other: which time schedule do you use? [Day of VUS/VCUG]
N/A

Other: which time schedule do you use? [Day(s) after VUS/VCUG, how many?]
N/A

Indication for prophylaxis

Which patients receive periinterventional antibiotic prophylaxis? (multiple answers possible) [all]
N/A

Which patients receive periinterventional antibiotic prophylaxis? (multiple answers possible) [patients post urinary tract infection/pyelonephritis/urosepsis]
N/A

Which patients receive periinterventional antibiotic prophylaxis? (multiple answers possible) [patients with urinary tract dilation in ultrasound]
N/A

Which patients receive periinterventional antibiotic prophylaxis? (multiple answers possible) [patients with prenatal urinary tract dilation]
N/A

Which patients receive periinterventional antibiotic prophylaxis? (multiple answers possible) [patients post VUR surgery]
N/A

Which patients receive periinterventional antibiotic prophylaxis? (multiple answers possible) [none]
N/A

Are there situations or clinical findings in the patient where you deviate from your prophylaxis standard (medication, dose, duration, age of the patient)? (multiple answers possible, please specify in answer box) [in case of already known vesicoureteral reflux (VUR)]
N/A

Are there situations or clinical findings in the patient where you deviate from your prophylaxis standard (medication, dose, duration, age of the patient)? (multiple answers possible, please specify in answer box) [in patients with recurrent urinary tract infections (UTI)]
N/A

Are there situations or clinical findings in the patient where you deviate from your prophylaxis standard (medication, dose, duration, age of the patient)? (multiple answers possible, please specify in answer box) [if multidrug-resistant organisms (MDRO) are detected in urine cultures]
N/A

Are there situations or clinical findings in the patient where you deviate from your prophylaxis standard (medication, dose, duration, age of the patient)? (multiple answers possible, please specify in answer box) [in newborns]
N/A

Are there situations in which you use intravenous prophylaxis?
N/A

Voiding analysis

Are there differences in antibiotic prophylaxis between VUS and VCUG in your department?
N/A

Is a non-pathological urine strip test required in preparation for VUS/VCUG in your department?
N/A

Is a non-pathological urine culture required in preparation for VUS/VCUG in your department?
N/A

Do patients only receive post-procedure prophylaxis if vesicoureteral reflux (VUR) is detected in VUS/VCUG?
N/A

Do other MUS/MCU findings (e.g. urethral valves) have an influence on post-procedure prophylaxis?
N/A

End of survey

May we contact you personally if we have further questions?
N/A

Survey response 204

Response ID
206

Last page
1

Start language
de

Seed
926264621

Date started
2025-03-27 19:31:07

Date last action
2025-03-27 19:32:00

Standard Operation Procedure

Who fills out this survey?
Pediatric Surgeon

Who fills out this survey? [Other]

In which type of hospital are you employed?
Community hospital (>50 beds for pediatric patients)

In which type of hospital are you employed? [Other]

In which country do you work?
Deutschland

Does your department have a defined protocol for infection prophylaxis in the context of VUS/VCUG?
Yes

Has this protocol been transformed into a written standard operating procedure (SOP)?
No

Antibiotic Prophylaxis

Are there situations/constellations in which you do not use antibiotic prophylaxis for VUS/VCUG?
N/A

Please specify these situations/constellations: [girl with first uncomplicated UTI without urinary tract dilation in ultrasound]
N/A

Please specify these situations/constellations: [boy with first uncomplicated UTI without urinary tract dilation in ultrasound]
N/A

Please specify these situations/constellations: [in patients with low grade urinary tract dilation (up to 2nd grade) and no UTI in medical history]
N/A

If you use antibiotic prophylaxis: which mediaction do you use? (multiple answers and subdivision into age groups possible) [Trimethoprim]
N/A

If you use antibiotic prophylaxis: which mediaction do you use? (multiple answers and subdivision into age groups possible) [Nitrofurantoin]
N/A

If you use antibiotic prophylaxis: which mediaction do you use? (multiple answers and subdivision into age groups possible) [Amoxicillin]
N/A

If you use antibiotic prophylaxis: which mediaction do you use? (multiple answers and subdivision into age groups possible) [Cefaclor]
N/A

If you use antibiotic prophylaxis: which mediaction do you use? (multiple answers and subdivision into age groups possible) [Amoxicillin/clavulanic acid]
N/A

If you use antibiotic prophylaxis: which mediaction do you use? (multiple answers and subdivision into age groups possible) [Other]
N/A

Trimethoprim: which time schedule do you use? [Day(s) before VUS/VCUG, how many?]
N/A

Trimethoprim: which time schedule do you use? [Day of VUS/VCUG]
N/A

Trimethoprim: which time schedule do you use? [Day(s) after VUS/VCUG, how many?]
N/A

Nitrofurantoin: which time schedule do you use? [Day(s) before VUS/VCUG, how many?]
N/A

Nitrofurantoin: which time schedule do you use? [Day of VUS/VCUG]
N/A

Nitrofurantoin: which time schedule do you use? [Day(s) after VUS/VCUG, how many?]
N/A

Amoxicillin: which time schedule do you use? [Day(s) before VUS/VCUG, how many?]
N/A

Amoxicillin: which time schedule do you use? [Day of VUS/VCUG]
N/A

Amoxicillin: which time schedule do you use? [Day(s) after VUS/VCUG, how many?]
N/A

Cefaclor: which time schedule do you use? [Days(s) before VUS/VCUG, how many?]
N/A

Cefaclor: which time schedule do you use? [Day of VUS/VCUG]
N/A

Cefaclor: which time schedule do you use? [Day(s) after VUS/VCUG, how many?]
N/A

Amoxicillin/clavulanic acid: in what dosage? (Amoxicillin portion) [prophylaktic (10-20 mg/Kg bodyweight/d)]
N/A

Amoxicillin/clavulanic acid: in what dosage? (Amoxicillin portion) [double prophylactic (20-40 mg/Kg bodyweight/d)]
N/A

Amoxicillin/clavulanic acid: in what dosage? (Amoxicillin portion) [therapeutic (80-100 mg/Kg bodyweight/d)]
N/A

Amoxicillin/clavulanic acid: which time schedule do you use? [Day(s) before VUS/VCUG]
N/A

Amoxicillin/clavulanic acid: which time schedule do you use? [Day of VUS/VCUG]
N/A

Amoxicillin/clavulanic acid: which time schedule do you use? [Day(s) after VUS/VCUG]
N/A

Other: which time schedule do you use? [Day(s) before VUS/VCUG, how many?]
N/A

Other: which time schedule do you use? [Day of VUS/VCUG]
N/A

Other: which time schedule do you use? [Day(s) after VUS/VCUG, how many?]
N/A

Indication for prophylaxis

Which patients receive periinterventional antibiotic prophylaxis? (multiple answers possible) [all]
N/A

Which patients receive periinterventional antibiotic prophylaxis? (multiple answers possible) [patients post urinary tract infection/pyelonephritis/urosepsis]
N/A

Which patients receive periinterventional antibiotic prophylaxis? (multiple answers possible) [patients with urinary tract dilation in ultrasound]
N/A

Which patients receive periinterventional antibiotic prophylaxis? (multiple answers possible) [patients with prenatal urinary tract dilation]
N/A

Which patients receive periinterventional antibiotic prophylaxis? (multiple answers possible) [patients post VUR surgery]
N/A

Which patients receive periinterventional antibiotic prophylaxis? (multiple answers possible) [none]
N/A

Are there situations or clinical findings in the patient where you deviate from your prophylaxis standard (medication, dose, duration, age of the patient)? (multiple answers possible, please specify in answer box) [in case of already known vesicoureteral reflux (VUR)]
N/A

Are there situations or clinical findings in the patient where you deviate from your prophylaxis standard (medication, dose, duration, age of the patient)? (multiple answers possible, please specify in answer box) [in patients with recurrent urinary tract infections (UTI)]
N/A

Are there situations or clinical findings in the patient where you deviate from your prophylaxis standard (medication, dose, duration, age of the patient)? (multiple answers possible, please specify in answer box) [if multidrug-resistant organisms (MDRO) are detected in urine cultures]
N/A

Are there situations or clinical findings in the patient where you deviate from your prophylaxis standard (medication, dose, duration, age of the patient)? (multiple answers possible, please specify in answer box) [in newborns]
N/A

Are there situations in which you use intravenous prophylaxis?
N/A

Voiding analysis

Are there differences in antibiotic prophylaxis between VUS and VCUG in your department?
N/A

Is a non-pathological urine strip test required in preparation for VUS/VCUG in your department?
N/A

Is a non-pathological urine culture required in preparation for VUS/VCUG in your department?
N/A

Do patients only receive post-procedure prophylaxis if vesicoureteral reflux (VUR) is detected in VUS/VCUG?
N/A

Do other MUS/MCU findings (e.g. urethral valves) have an influence on post-procedure prophylaxis?
N/A

End of survey

May we contact you personally if we have further questions?
N/A

Survey response 205

Response ID
207

Date submitted
2025-03-27 20:08:51

Last page
5

Start language
en

Seed
1510643829

Date started
2025-03-27 20:06:11

Date last action
2025-03-27 20:08:51

Standard Operation Procedure

Who fills out this survey?
Pediatric Radiologist

Who fills out this survey? [Other]

In which type of hospital are you employed?
University Hospital

In which type of hospital are you employed? [Other]

In which country do you work?
Greece

Does your department have a defined protocol for infection prophylaxis in the context of VUS/VCUG?
Yes

Has this protocol been transformed into a written standard operating procedure (SOP)?
Yes

Which department is the author of the SOP for infection prophylaxis in VUS/VCUG?
Pediatric surgery

Which department is the author of the SOP for infection prophylaxis in VUS/VCUG? [Other]

Antibiotic Prophylaxis

Are there situations/constellations in which you do not use antibiotic prophylaxis for VUS/VCUG?
No

Please specify these situations/constellations: [girl with first uncomplicated UTI without urinary tract dilation in ultrasound]
N/A

Please specify these situations/constellations: [boy with first uncomplicated UTI without urinary tract dilation in ultrasound]
N/A

Please specify these situations/constellations: [in patients with low grade urinary tract dilation (up to 2nd grade) and no UTI in medical history]
N/A

If you use antibiotic prophylaxis: which mediaction do you use? (multiple answers and subdivision into age groups possible) [Trimethoprim]
No

If you use antibiotic prophylaxis: which mediaction do you use? (multiple answers and subdivision into age groups possible) [Nitrofurantoin]
No

If you use antibiotic prophylaxis: which mediaction do you use? (multiple answers and subdivision into age groups possible) [Amoxicillin]
No

If you use antibiotic prophylaxis: which mediaction do you use? (multiple answers and subdivision into age groups possible) [Cefaclor]
Yes

If you use antibiotic prophylaxis: which mediaction do you use? (multiple answers and subdivision into age groups possible) [Amoxicillin/clavulanic acid]
No

If you use antibiotic prophylaxis: which mediaction do you use? (multiple answers and subdivision into age groups possible) [Other]
No

Trimethoprim: which time schedule do you use? [Day(s) before VUS/VCUG, how many?]
N/A

Trimethoprim: which time schedule do you use? [Day of VUS/VCUG]
N/A

Trimethoprim: which time schedule do you use? [Day(s) after VUS/VCUG, how many?]
N/A

Nitrofurantoin: which time schedule do you use? [Day(s) before VUS/VCUG, how many?]
N/A

Nitrofurantoin: which time schedule do you use? [Day of VUS/VCUG]
N/A

Nitrofurantoin: which time schedule do you use? [Day(s) after VUS/VCUG, how many?]
N/A

Amoxicillin: which time schedule do you use? [Day(s) before VUS/VCUG, how many?]
N/A

Amoxicillin: which time schedule do you use? [Day of VUS/VCUG]
N/A

Amoxicillin: which time schedule do you use? [Day(s) after VUS/VCUG, how many?]
N/A

Cefaclor: in what dosage?
prophylactic (10 mg/kg bodyweight/d)

Cefaclor: in what dosage? [Other]

Cefaclor: for which age group do you use cefaclor?
0-3 yo

Cefaclor: which time schedule do you use? [Days(s) before VUS/VCUG, how many?]
Yes

Cefaclor: which time schedule do you use? [Days(s) before VUS/VCUG, how many?][Comment]
7

Cefaclor: which time schedule do you use? [Day of VUS/VCUG]
No

Cefaclor: which time schedule do you use? [Day of VUS/VCUG][Comment]

Cefaclor: which time schedule do you use? [Day(s) after VUS/VCUG, how many?]
No

Cefaclor: which time schedule do you use? [Day(s) after VUS/VCUG, how many?][Comment]

Amoxicillin/clavulanic acid: in what dosage? (Amoxicillin portion) [prophylaktic (10-20 mg/Kg bodyweight/d)]
N/A

Amoxicillin/clavulanic acid: in what dosage? (Amoxicillin portion) [double prophylactic (20-40 mg/Kg bodyweight/d)]
N/A

Amoxicillin/clavulanic acid: in what dosage? (Amoxicillin portion) [therapeutic (80-100 mg/Kg bodyweight/d)]
N/A

Amoxicillin/clavulanic acid: which time schedule do you use? [Day(s) before VUS/VCUG]
N/A

Amoxicillin/clavulanic acid: which time schedule do you use? [Day of VUS/VCUG]
N/A

Amoxicillin/clavulanic acid: which time schedule do you use? [Day(s) after VUS/VCUG]
N/A

Other: which time schedule do you use? [Day(s) before VUS/VCUG, how many?]
N/A

Other: which time schedule do you use? [Day of VUS/VCUG]
N/A

Other: which time schedule do you use? [Day(s) after VUS/VCUG, how many?]
N/A

Indication for prophylaxis

Which patients receive periinterventional antibiotic prophylaxis? (multiple answers possible) [all]
Yes

Which patients receive periinterventional antibiotic prophylaxis? (multiple answers possible) [patients post urinary tract infection/pyelonephritis/urosepsis]
N/A

Which patients receive periinterventional antibiotic prophylaxis? (multiple answers possible) [patients with urinary tract dilation in ultrasound]
N/A

Which patients receive periinterventional antibiotic prophylaxis? (multiple answers possible) [patients with prenatal urinary tract dilation]
N/A

Which patients receive periinterventional antibiotic prophylaxis? (multiple answers possible) [patients post VUR surgery]
N/A

Which patients receive periinterventional antibiotic prophylaxis? (multiple answers possible) [none]
N/A

Are there situations or clinical findings in the patient where you deviate from your prophylaxis standard (medication, dose, duration, age of the patient)? (multiple answers possible, please specify in answer box) [in case of already known vesicoureteral reflux (VUR)]
Yes

Are there situations or clinical findings in the patient where you deviate from your prophylaxis standard (medication, dose, duration, age of the patient)? (multiple answers possible, please specify in answer box) [in case of already known vesicoureteral reflux (VUR)][Comment]

Are there situations or clinical findings in the patient where you deviate from your prophylaxis standard (medication, dose, duration, age of the patient)? (multiple answers possible, please specify in answer box) [in patients with recurrent urinary tract infections (UTI)]
Yes

Are there situations or clinical findings in the patient where you deviate from your prophylaxis standard (medication, dose, duration, age of the patient)? (multiple answers possible, please specify in answer box) [in patients with recurrent urinary tract infections (UTI)][Comment]

Are there situations or clinical findings in the patient where you deviate from your prophylaxis standard (medication, dose, duration, age of the patient)? (multiple answers possible, please specify in answer box) [if multidrug-resistant organisms (MDRO) are detected in urine cultures]
Yes

Are there situations or clinical findings in the patient where you deviate from your prophylaxis standard (medication, dose, duration, age of the patient)? (multiple answers possible, please specify in answer box) [if multidrug-resistant organisms (MDRO) are detected in urine cultures][Comment]

Are there situations or clinical findings in the patient where you deviate from your prophylaxis standard (medication, dose, duration, age of the patient)? (multiple answers possible, please specify in answer box) [in newborns]
No

Are there situations or clinical findings in the patient where you deviate from your prophylaxis standard (medication, dose, duration, age of the patient)? (multiple answers possible, please specify in answer box) [in newborns][Comment]

Are there situations or clinical findings in the patient where you deviate from your prophylaxis standard (medication, dose, duration, age of the patient)? (multiple answers possible, please specify in answer box) [Other]

Are there situations or clinical findings in the patient where you deviate from your prophylaxis standard (medication, dose, duration, age of the patient)? (multiple answers possible, please specify in answer box) [Other comment]

Are there situations in which you use intravenous prophylaxis?
No

Voiding analysis

Are there differences in antibiotic prophylaxis between VUS and VCUG in your department?
No

Is a non-pathological urine strip test required in preparation for VUS/VCUG in your department?
Yes

What is the maximum amount of days accepted for urine strip test to be performed prior to the exam?
7

Is a non-pathological urine culture required in preparation for VUS/VCUG in your department?
Yes

What is the maximum amount of days accepted for urine culture to be performed prior to the exam?
7

Do patients only receive post-procedure prophylaxis if vesicoureteral reflux (VUR) is detected in VUS/VCUG?
No

Do other MUS/MCU findings (e.g. urethral valves) have an influence on post-procedure prophylaxis?
No

End of survey

Thank you for taking the time to answer this survey!

May we contact you personally if we have further questions?
No

Survey response 206

Response ID
208

Last page
2

Start language
en

Seed
86758797

Date started
2025-03-28 06:50:11

Date last action
2025-03-28 06:52:54

Standard Operation Procedure

Who fills out this survey?
Pediatric Radiologist

Who fills out this survey? [Other]

In which type of hospital are you employed?
University Hospital

In which type of hospital are you employed? [Other]

In which country do you work?
Slovenia

Does your department have a defined protocol for infection prophylaxis in the context of VUS/VCUG?
Yes

Has this protocol been transformed into a written standard operating procedure (SOP)?
Yes

Which department is the author of the SOP for infection prophylaxis in VUS/VCUG?
Pediatric nephrology

Which department is the author of the SOP for infection prophylaxis in VUS/VCUG? [Other]

Antibiotic Prophylaxis

Are there situations/constellations in which you do not use antibiotic prophylaxis for VUS/VCUG?
No

Please specify these situations/constellations: [girl with first uncomplicated UTI without urinary tract dilation in ultrasound]
N/A

Please specify these situations/constellations: [boy with first uncomplicated UTI without urinary tract dilation in ultrasound]
N/A

Please specify these situations/constellations: [in patients with low grade urinary tract dilation (up to 2nd grade) and no UTI in medical history]
N/A

If you use antibiotic prophylaxis: which mediaction do you use? (multiple answers and subdivision into age groups possible) [Trimethoprim]
No

If you use antibiotic prophylaxis: which mediaction do you use? (multiple answers and subdivision into age groups possible) [Nitrofurantoin]
No

If you use antibiotic prophylaxis: which mediaction do you use? (multiple answers and subdivision into age groups possible) [Amoxicillin]
No

If you use antibiotic prophylaxis: which mediaction do you use? (multiple answers and subdivision into age groups possible) [Cefaclor]
No

If you use antibiotic prophylaxis: which mediaction do you use? (multiple answers and subdivision into age groups possible) [Amoxicillin/clavulanic acid]
No

If you use antibiotic prophylaxis: which mediaction do you use? (multiple answers and subdivision into age groups possible) [Other]
No

Trimethoprim: which time schedule do you use? [Day(s) before VUS/VCUG, how many?]
N/A

Trimethoprim: which time schedule do you use? [Day of VUS/VCUG]
N/A

Trimethoprim: which time schedule do you use? [Day(s) after VUS/VCUG, how many?]
N/A

Nitrofurantoin: which time schedule do you use? [Day(s) before VUS/VCUG, how many?]
N/A

Nitrofurantoin: which time schedule do you use? [Day of VUS/VCUG]
N/A

Nitrofurantoin: which time schedule do you use? [Day(s) after VUS/VCUG, how many?]
N/A

Amoxicillin: which time schedule do you use? [Day(s) before VUS/VCUG, how many?]
N/A

Amoxicillin: which time schedule do you use? [Day of VUS/VCUG]
N/A

Amoxicillin: which time schedule do you use? [Day(s) after VUS/VCUG, how many?]
N/A

Cefaclor: which time schedule do you use? [Days(s) before VUS/VCUG, how many?]
N/A

Cefaclor: which time schedule do you use? [Day of VUS/VCUG]
N/A

Cefaclor: which time schedule do you use? [Day(s) after VUS/VCUG, how many?]
N/A

Amoxicillin/clavulanic acid: in what dosage? (Amoxicillin portion) [prophylaktic (10-20 mg/Kg bodyweight/d)]
N/A

Amoxicillin/clavulanic acid: in what dosage? (Amoxicillin portion) [double prophylactic (20-40 mg/Kg bodyweight/d)]
N/A

Amoxicillin/clavulanic acid: in what dosage? (Amoxicillin portion) [therapeutic (80-100 mg/Kg bodyweight/d)]
N/A

Amoxicillin/clavulanic acid: which time schedule do you use? [Day(s) before VUS/VCUG]
N/A

Amoxicillin/clavulanic acid: which time schedule do you use? [Day of VUS/VCUG]
N/A

Amoxicillin/clavulanic acid: which time schedule do you use? [Day(s) after VUS/VCUG]
N/A

Other: which time schedule do you use? [Day(s) before VUS/VCUG, how many?]
N/A

Other: which time schedule do you use? [Day of VUS/VCUG]
N/A

Other: which time schedule do you use? [Day(s) after VUS/VCUG, how many?]
N/A

Indication for prophylaxis

Which patients receive periinterventional antibiotic prophylaxis? (multiple answers possible) [all]
No

Which patients receive periinterventional antibiotic prophylaxis? (multiple answers possible) [patients post urinary tract infection/pyelonephritis/urosepsis]
No

Which patients receive periinterventional antibiotic prophylaxis? (multiple answers possible) [patients with urinary tract dilation in ultrasound]
No

Which patients receive periinterventional antibiotic prophylaxis? (multiple answers possible) [patients with prenatal urinary tract dilation]
No

Which patients receive periinterventional antibiotic prophylaxis? (multiple answers possible) [patients post VUR surgery]
No

Which patients receive periinterventional antibiotic prophylaxis? (multiple answers possible) [none]
No

Which patients receive periinterventional antibiotic prophylaxis? (multiple answers possible) [Other]

Are there situations or clinical findings in the patient where you deviate from your prophylaxis standard (medication, dose, duration, age of the patient)? (multiple answers possible, please specify in answer box) [in case of already known vesicoureteral reflux (VUR)]
No

Are there situations or clinical findings in the patient where you deviate from your prophylaxis standard (medication, dose, duration, age of the patient)? (multiple answers possible, please specify in answer box) [in case of already known vesicoureteral reflux (VUR)][Comment]

Are there situations or clinical findings in the patient where you deviate from your prophylaxis standard (medication, dose, duration, age of the patient)? (multiple answers possible, please specify in answer box) [in patients with recurrent urinary tract infections (UTI)]
No

Are there situations or clinical findings in the patient where you deviate from your prophylaxis standard (medication, dose, duration, age of the patient)? (multiple answers possible, please specify in answer box) [in patients with recurrent urinary tract infections (UTI)][Comment]

Are there situations or clinical findings in the patient where you deviate from your prophylaxis standard (medication, dose, duration, age of the patient)? (multiple answers possible, please specify in answer box) [if multidrug-resistant organisms (MDRO) are detected in urine cultures]
No

Are there situations or clinical findings in the patient where you deviate from your prophylaxis standard (medication, dose, duration, age of the patient)? (multiple answers possible, please specify in answer box) [if multidrug-resistant organisms (MDRO) are detected in urine cultures][Comment]

Are there situations or clinical findings in the patient where you deviate from your prophylaxis standard (medication, dose, duration, age of the patient)? (multiple answers possible, please specify in answer box) [in newborns]
No

Are there situations or clinical findings in the patient where you deviate from your prophylaxis standard (medication, dose, duration, age of the patient)? (multiple answers possible, please specify in answer box) [in newborns][Comment]

Are there situations or clinical findings in the patient where you deviate from your prophylaxis standard (medication, dose, duration, age of the patient)? (multiple answers possible, please specify in answer box) [Other]

Are there situations or clinical findings in the patient where you deviate from your prophylaxis standard (medication, dose, duration, age of the patient)? (multiple answers possible, please specify in answer box) [Other comment]

Are there situations in which you use intravenous prophylaxis?
Yes

In which situations?
Newborn that is in hospital

Voiding analysis

Are there differences in antibiotic prophylaxis between VUS and VCUG in your department?
N/A

Is a non-pathological urine strip test required in preparation for VUS/VCUG in your department?
N/A

Is a non-pathological urine culture required in preparation for VUS/VCUG in your department?
N/A

Do patients only receive post-procedure prophylaxis if vesicoureteral reflux (VUR) is detected in VUS/VCUG?
N/A

Do other MUS/MCU findings (e.g. urethral valves) have an influence on post-procedure prophylaxis?
N/A

End of survey

May we contact you personally if we have further questions?
N/A

Survey response 207

Response ID
209

Last page
1

Start language
en

Seed
1954439015

Date started
2025-03-28 08:54:01

Date last action
2025-03-28 08:54:31

Standard Operation Procedure

Who fills out this survey?
Pediatric Radiologist

Who fills out this survey? [Other]

In which type of hospital are you employed?
University Hospital

In which type of hospital are you employed? [Other]

In which country do you work?
France

Does your department have a defined protocol for infection prophylaxis in the context of VUS/VCUG?
No

Has this protocol been transformed into a written standard operating procedure (SOP)?
N/A

Antibiotic Prophylaxis

Are there situations/constellations in which you do not use antibiotic prophylaxis for VUS/VCUG?
N/A

Please specify these situations/constellations: [girl with first uncomplicated UTI without urinary tract dilation in ultrasound]
N/A

Please specify these situations/constellations: [boy with first uncomplicated UTI without urinary tract dilation in ultrasound]
N/A

Please specify these situations/constellations: [in patients with low grade urinary tract dilation (up to 2nd grade) and no UTI in medical history]
N/A

If you use antibiotic prophylaxis: which mediaction do you use? (multiple answers and subdivision into age groups possible) [Trimethoprim]
N/A

If you use antibiotic prophylaxis: which mediaction do you use? (multiple answers and subdivision into age groups possible) [Nitrofurantoin]
N/A

If you use antibiotic prophylaxis: which mediaction do you use? (multiple answers and subdivision into age groups possible) [Amoxicillin]
N/A

If you use antibiotic prophylaxis: which mediaction do you use? (multiple answers and subdivision into age groups possible) [Cefaclor]
N/A

If you use antibiotic prophylaxis: which mediaction do you use? (multiple answers and subdivision into age groups possible) [Amoxicillin/clavulanic acid]
N/A

If you use antibiotic prophylaxis: which mediaction do you use? (multiple answers and subdivision into age groups possible) [Other]
N/A

Trimethoprim: which time schedule do you use? [Day(s) before VUS/VCUG, how many?]
N/A

Trimethoprim: which time schedule do you use? [Day of VUS/VCUG]
N/A

Trimethoprim: which time schedule do you use? [Day(s) after VUS/VCUG, how many?]
N/A

Nitrofurantoin: which time schedule do you use? [Day(s) before VUS/VCUG, how many?]
N/A

Nitrofurantoin: which time schedule do you use? [Day of VUS/VCUG]
N/A

Nitrofurantoin: which time schedule do you use? [Day(s) after VUS/VCUG, how many?]
N/A

Amoxicillin: which time schedule do you use? [Day(s) before VUS/VCUG, how many?]
N/A

Amoxicillin: which time schedule do you use? [Day of VUS/VCUG]
N/A

Amoxicillin: which time schedule do you use? [Day(s) after VUS/VCUG, how many?]
N/A

Cefaclor: which time schedule do you use? [Days(s) before VUS/VCUG, how many?]
N/A

Cefaclor: which time schedule do you use? [Day of VUS/VCUG]
N/A

Cefaclor: which time schedule do you use? [Day(s) after VUS/VCUG, how many?]
N/A

Amoxicillin/clavulanic acid: in what dosage? (Amoxicillin portion) [prophylaktic (10-20 mg/Kg bodyweight/d)]
N/A

Amoxicillin/clavulanic acid: in what dosage? (Amoxicillin portion) [double prophylactic (20-40 mg/Kg bodyweight/d)]
N/A

Amoxicillin/clavulanic acid: in what dosage? (Amoxicillin portion) [therapeutic (80-100 mg/Kg bodyweight/d)]
N/A

Amoxicillin/clavulanic acid: which time schedule do you use? [Day(s) before VUS/VCUG]
N/A

Amoxicillin/clavulanic acid: which time schedule do you use? [Day of VUS/VCUG]
N/A

Amoxicillin/clavulanic acid: which time schedule do you use? [Day(s) after VUS/VCUG]
N/A

Other: which time schedule do you use? [Day(s) before VUS/VCUG, how many?]
N/A

Other: which time schedule do you use? [Day of VUS/VCUG]
N/A

Other: which time schedule do you use? [Day(s) after VUS/VCUG, how many?]
N/A

Indication for prophylaxis

Which patients receive periinterventional antibiotic prophylaxis? (multiple answers possible) [all]
N/A

Which patients receive periinterventional antibiotic prophylaxis? (multiple answers possible) [patients post urinary tract infection/pyelonephritis/urosepsis]
N/A

Which patients receive periinterventional antibiotic prophylaxis? (multiple answers possible) [patients with urinary tract dilation in ultrasound]
N/A

Which patients receive periinterventional antibiotic prophylaxis? (multiple answers possible) [patients with prenatal urinary tract dilation]
N/A

Which patients receive periinterventional antibiotic prophylaxis? (multiple answers possible) [patients post VUR surgery]
N/A

Which patients receive periinterventional antibiotic prophylaxis? (multiple answers possible) [none]
N/A

Are there situations or clinical findings in the patient where you deviate from your prophylaxis standard (medication, dose, duration, age of the patient)? (multiple answers possible, please specify in answer box) [in case of already known vesicoureteral reflux (VUR)]
N/A

Are there situations or clinical findings in the patient where you deviate from your prophylaxis standard (medication, dose, duration, age of the patient)? (multiple answers possible, please specify in answer box) [in patients with recurrent urinary tract infections (UTI)]
N/A

Are there situations or clinical findings in the patient where you deviate from your prophylaxis standard (medication, dose, duration, age of the patient)? (multiple answers possible, please specify in answer box) [if multidrug-resistant organisms (MDRO) are detected in urine cultures]
N/A

Are there situations or clinical findings in the patient where you deviate from your prophylaxis standard (medication, dose, duration, age of the patient)? (multiple answers possible, please specify in answer box) [in newborns]
N/A

Are there situations in which you use intravenous prophylaxis?
N/A

Voiding analysis

Are there differences in antibiotic prophylaxis between VUS and VCUG in your department?
N/A

Is a non-pathological urine strip test required in preparation for VUS/VCUG in your department?
N/A

Is a non-pathological urine culture required in preparation for VUS/VCUG in your department?
N/A

Do patients only receive post-procedure prophylaxis if vesicoureteral reflux (VUR) is detected in VUS/VCUG?
N/A

Do other MUS/MCU findings (e.g. urethral valves) have an influence on post-procedure prophylaxis?
N/A

End of survey

May we contact you personally if we have further questions?
N/A

Survey response 208

Response ID
210

Last page
1

Start language
en

Seed
130493307

Date started
2025-03-28 15:16:13

Date last action
2025-03-28 15:17:04

Standard Operation Procedure

Who fills out this survey?
Pediatric Radiologist

Who fills out this survey? [Other]

In which type of hospital are you employed?
University Hospital

In which type of hospital are you employed? [Other]

In which country do you work?
Slovenia

Does your department have a defined protocol for infection prophylaxis in the context of VUS/VCUG?
No

Has this protocol been transformed into a written standard operating procedure (SOP)?
N/A

Antibiotic Prophylaxis

Are there situations/constellations in which you do not use antibiotic prophylaxis for VUS/VCUG?
N/A

Please specify these situations/constellations: [girl with first uncomplicated UTI without urinary tract dilation in ultrasound]
N/A

Please specify these situations/constellations: [boy with first uncomplicated UTI without urinary tract dilation in ultrasound]
N/A

Please specify these situations/constellations: [in patients with low grade urinary tract dilation (up to 2nd grade) and no UTI in medical history]
N/A

If you use antibiotic prophylaxis: which mediaction do you use? (multiple answers and subdivision into age groups possible) [Trimethoprim]
N/A

If you use antibiotic prophylaxis: which mediaction do you use? (multiple answers and subdivision into age groups possible) [Nitrofurantoin]
N/A

If you use antibiotic prophylaxis: which mediaction do you use? (multiple answers and subdivision into age groups possible) [Amoxicillin]
N/A

If you use antibiotic prophylaxis: which mediaction do you use? (multiple answers and subdivision into age groups possible) [Cefaclor]
N/A

If you use antibiotic prophylaxis: which mediaction do you use? (multiple answers and subdivision into age groups possible) [Amoxicillin/clavulanic acid]
N/A

If you use antibiotic prophylaxis: which mediaction do you use? (multiple answers and subdivision into age groups possible) [Other]
N/A

Trimethoprim: which time schedule do you use? [Day(s) before VUS/VCUG, how many?]
N/A

Trimethoprim: which time schedule do you use? [Day of VUS/VCUG]
N/A

Trimethoprim: which time schedule do you use? [Day(s) after VUS/VCUG, how many?]
N/A

Nitrofurantoin: which time schedule do you use? [Day(s) before VUS/VCUG, how many?]
N/A

Nitrofurantoin: which time schedule do you use? [Day of VUS/VCUG]
N/A

Nitrofurantoin: which time schedule do you use? [Day(s) after VUS/VCUG, how many?]
N/A

Amoxicillin: which time schedule do you use? [Day(s) before VUS/VCUG, how many?]
N/A

Amoxicillin: which time schedule do you use? [Day of VUS/VCUG]
N/A

Amoxicillin: which time schedule do you use? [Day(s) after VUS/VCUG, how many?]
N/A

Cefaclor: which time schedule do you use? [Days(s) before VUS/VCUG, how many?]
N/A

Cefaclor: which time schedule do you use? [Day of VUS/VCUG]
N/A

Cefaclor: which time schedule do you use? [Day(s) after VUS/VCUG, how many?]
N/A

Amoxicillin/clavulanic acid: in what dosage? (Amoxicillin portion) [prophylaktic (10-20 mg/Kg bodyweight/d)]
N/A

Amoxicillin/clavulanic acid: in what dosage? (Amoxicillin portion) [double prophylactic (20-40 mg/Kg bodyweight/d)]
N/A

Amoxicillin/clavulanic acid: in what dosage? (Amoxicillin portion) [therapeutic (80-100 mg/Kg bodyweight/d)]
N/A

Amoxicillin/clavulanic acid: which time schedule do you use? [Day(s) before VUS/VCUG]
N/A

Amoxicillin/clavulanic acid: which time schedule do you use? [Day of VUS/VCUG]
N/A

Amoxicillin/clavulanic acid: which time schedule do you use? [Day(s) after VUS/VCUG]
N/A

Other: which time schedule do you use? [Day(s) before VUS/VCUG, how many?]
N/A

Other: which time schedule do you use? [Day of VUS/VCUG]
N/A

Other: which time schedule do you use? [Day(s) after VUS/VCUG, how many?]
N/A

Indication for prophylaxis

Which patients receive periinterventional antibiotic prophylaxis? (multiple answers possible) [all]
N/A

Which patients receive periinterventional antibiotic prophylaxis? (multiple answers possible) [patients post urinary tract infection/pyelonephritis/urosepsis]
N/A

Which patients receive periinterventional antibiotic prophylaxis? (multiple answers possible) [patients with urinary tract dilation in ultrasound]
N/A

Which patients receive periinterventional antibiotic prophylaxis? (multiple answers possible) [patients with prenatal urinary tract dilation]
N/A

Which patients receive periinterventional antibiotic prophylaxis? (multiple answers possible) [patients post VUR surgery]
N/A

Which patients receive periinterventional antibiotic prophylaxis? (multiple answers possible) [none]
N/A

Are there situations or clinical findings in the patient where you deviate from your prophylaxis standard (medication, dose, duration, age of the patient)? (multiple answers possible, please specify in answer box) [in case of already known vesicoureteral reflux (VUR)]
N/A

Are there situations or clinical findings in the patient where you deviate from your prophylaxis standard (medication, dose, duration, age of the patient)? (multiple answers possible, please specify in answer box) [in patients with recurrent urinary tract infections (UTI)]
N/A

Are there situations or clinical findings in the patient where you deviate from your prophylaxis standard (medication, dose, duration, age of the patient)? (multiple answers possible, please specify in answer box) [if multidrug-resistant organisms (MDRO) are detected in urine cultures]
N/A

Are there situations or clinical findings in the patient where you deviate from your prophylaxis standard (medication, dose, duration, age of the patient)? (multiple answers possible, please specify in answer box) [in newborns]
N/A

Are there situations in which you use intravenous prophylaxis?
N/A

Voiding analysis

Are there differences in antibiotic prophylaxis between VUS and VCUG in your department?
N/A

Is a non-pathological urine strip test required in preparation for VUS/VCUG in your department?
N/A

Is a non-pathological urine culture required in preparation for VUS/VCUG in your department?
N/A

Do patients only receive post-procedure prophylaxis if vesicoureteral reflux (VUR) is detected in VUS/VCUG?
N/A

Do other MUS/MCU findings (e.g. urethral valves) have an influence on post-procedure prophylaxis?
N/A

End of survey

May we contact you personally if we have further questions?
N/A

Survey response 209

Response ID
211

Last page
1

Start language
en

Seed
1996672878

Date started
2025-03-28 23:37:09

Date last action
2025-03-28 23:37:52

Standard Operation Procedure

Who fills out this survey?
Pediatric Radiologist

Who fills out this survey? [Other]

In which type of hospital are you employed?
Community hospital (>50 beds for pediatric patients)

In which type of hospital are you employed? [Other]

In which country do you work?
UK

Does your department have a defined protocol for infection prophylaxis in the context of VUS/VCUG?
Yes

Has this protocol been transformed into a written standard operating procedure (SOP)?
No

Antibiotic Prophylaxis

Are there situations/constellations in which you do not use antibiotic prophylaxis for VUS/VCUG?
N/A

Please specify these situations/constellations: [girl with first uncomplicated UTI without urinary tract dilation in ultrasound]
N/A

Please specify these situations/constellations: [boy with first uncomplicated UTI without urinary tract dilation in ultrasound]
N/A

Please specify these situations/constellations: [in patients with low grade urinary tract dilation (up to 2nd grade) and no UTI in medical history]
N/A

If you use antibiotic prophylaxis: which mediaction do you use? (multiple answers and subdivision into age groups possible) [Trimethoprim]
N/A

If you use antibiotic prophylaxis: which mediaction do you use? (multiple answers and subdivision into age groups possible) [Nitrofurantoin]
N/A

If you use antibiotic prophylaxis: which mediaction do you use? (multiple answers and subdivision into age groups possible) [Amoxicillin]
N/A

If you use antibiotic prophylaxis: which mediaction do you use? (multiple answers and subdivision into age groups possible) [Cefaclor]
N/A

If you use antibiotic prophylaxis: which mediaction do you use? (multiple answers and subdivision into age groups possible) [Amoxicillin/clavulanic acid]
N/A

If you use antibiotic prophylaxis: which mediaction do you use? (multiple answers and subdivision into age groups possible) [Other]
N/A

Trimethoprim: which time schedule do you use? [Day(s) before VUS/VCUG, how many?]
N/A

Trimethoprim: which time schedule do you use? [Day of VUS/VCUG]
N/A

Trimethoprim: which time schedule do you use? [Day(s) after VUS/VCUG, how many?]
N/A

Nitrofurantoin: which time schedule do you use? [Day(s) before VUS/VCUG, how many?]
N/A

Nitrofurantoin: which time schedule do you use? [Day of VUS/VCUG]
N/A

Nitrofurantoin: which time schedule do you use? [Day(s) after VUS/VCUG, how many?]
N/A

Amoxicillin: which time schedule do you use? [Day(s) before VUS/VCUG, how many?]
N/A

Amoxicillin: which time schedule do you use? [Day of VUS/VCUG]
N/A

Amoxicillin: which time schedule do you use? [Day(s) after VUS/VCUG, how many?]
N/A

Cefaclor: which time schedule do you use? [Days(s) before VUS/VCUG, how many?]
N/A

Cefaclor: which time schedule do you use? [Day of VUS/VCUG]
N/A

Cefaclor: which time schedule do you use? [Day(s) after VUS/VCUG, how many?]
N/A

Amoxicillin/clavulanic acid: in what dosage? (Amoxicillin portion) [prophylaktic (10-20 mg/Kg bodyweight/d)]
N/A

Amoxicillin/clavulanic acid: in what dosage? (Amoxicillin portion) [double prophylactic (20-40 mg/Kg bodyweight/d)]
N/A

Amoxicillin/clavulanic acid: in what dosage? (Amoxicillin portion) [therapeutic (80-100 mg/Kg bodyweight/d)]
N/A

Amoxicillin/clavulanic acid: which time schedule do you use? [Day(s) before VUS/VCUG]
N/A

Amoxicillin/clavulanic acid: which time schedule do you use? [Day of VUS/VCUG]
N/A

Amoxicillin/clavulanic acid: which time schedule do you use? [Day(s) after VUS/VCUG]
N/A

Other: which time schedule do you use? [Day(s) before VUS/VCUG, how many?]
N/A

Other: which time schedule do you use? [Day of VUS/VCUG]
N/A

Other: which time schedule do you use? [Day(s) after VUS/VCUG, how many?]
N/A

Indication for prophylaxis

Which patients receive periinterventional antibiotic prophylaxis? (multiple answers possible) [all]
N/A

Which patients receive periinterventional antibiotic prophylaxis? (multiple answers possible) [patients post urinary tract infection/pyelonephritis/urosepsis]
N/A

Which patients receive periinterventional antibiotic prophylaxis? (multiple answers possible) [patients with urinary tract dilation in ultrasound]
N/A

Which patients receive periinterventional antibiotic prophylaxis? (multiple answers possible) [patients with prenatal urinary tract dilation]
N/A

Which patients receive periinterventional antibiotic prophylaxis? (multiple answers possible) [patients post VUR surgery]
N/A

Which patients receive periinterventional antibiotic prophylaxis? (multiple answers possible) [none]
N/A

Are there situations or clinical findings in the patient where you deviate from your prophylaxis standard (medication, dose, duration, age of the patient)? (multiple answers possible, please specify in answer box) [in case of already known vesicoureteral reflux (VUR)]
N/A

Are there situations or clinical findings in the patient where you deviate from your prophylaxis standard (medication, dose, duration, age of the patient)? (multiple answers possible, please specify in answer box) [in patients with recurrent urinary tract infections (UTI)]
N/A

Are there situations or clinical findings in the patient where you deviate from your prophylaxis standard (medication, dose, duration, age of the patient)? (multiple answers possible, please specify in answer box) [if multidrug-resistant organisms (MDRO) are detected in urine cultures]
N/A

Are there situations or clinical findings in the patient where you deviate from your prophylaxis standard (medication, dose, duration, age of the patient)? (multiple answers possible, please specify in answer box) [in newborns]
N/A

Are there situations in which you use intravenous prophylaxis?
N/A

Voiding analysis

Are there differences in antibiotic prophylaxis between VUS and VCUG in your department?
N/A

Is a non-pathological urine strip test required in preparation for VUS/VCUG in your department?
N/A

Is a non-pathological urine culture required in preparation for VUS/VCUG in your department?
N/A

Do patients only receive post-procedure prophylaxis if vesicoureteral reflux (VUR) is detected in VUS/VCUG?
N/A

Do other MUS/MCU findings (e.g. urethral valves) have an influence on post-procedure prophylaxis?
N/A

End of survey

May we contact you personally if we have further questions?
N/A

Survey response 210

Response ID
212

Date submitted
2025-03-29 18:17:44

Last page
5

Start language
en

Seed
55562243

Date started
2025-03-29 18:00:35

Date last action
2025-03-29 18:17:44

Standard Operation Procedure

Who fills out this survey?
Pediatric Radiologist

Who fills out this survey? [Other]

In which type of hospital are you employed?
University Hospital

In which type of hospital are you employed? [Other]

In which country do you work?
Ireland

Does your department have a defined protocol for infection prophylaxis in the context of VUS/VCUG?
Yes

Has this protocol been transformed into a written standard operating procedure (SOP)?
Yes

Which department is the author of the SOP for infection prophylaxis in VUS/VCUG?
Pediatric radiology

Which department is the author of the SOP for infection prophylaxis in VUS/VCUG? [Other]

Antibiotic Prophylaxis

Are there situations/constellations in which you do not use antibiotic prophylaxis for VUS/VCUG?
No

Please specify these situations/constellations: [girl with first uncomplicated UTI without urinary tract dilation in ultrasound]
N/A

Please specify these situations/constellations: [boy with first uncomplicated UTI without urinary tract dilation in ultrasound]
N/A

Please specify these situations/constellations: [in patients with low grade urinary tract dilation (up to 2nd grade) and no UTI in medical history]
N/A

If you use antibiotic prophylaxis: which mediaction do you use? (multiple answers and subdivision into age groups possible) [Trimethoprim]
Yes

If you use antibiotic prophylaxis: which mediaction do you use? (multiple answers and subdivision into age groups possible) [Nitrofurantoin]
No

If you use antibiotic prophylaxis: which mediaction do you use? (multiple answers and subdivision into age groups possible) [Amoxicillin]
No

If you use antibiotic prophylaxis: which mediaction do you use? (multiple answers and subdivision into age groups possible) [Cefaclor]
Yes

If you use antibiotic prophylaxis: which mediaction do you use? (multiple answers and subdivision into age groups possible) [Amoxicillin/clavulanic acid]
Yes

If you use antibiotic prophylaxis: which mediaction do you use? (multiple answers and subdivision into age groups possible) [Other]
Yes

Trimethoprim: in what dosage?
double prophylakctic (4 mg/kg bodyweight/d)

Trimethoprim: in what dosage? [Other]

Trimethoprim: for which age group do you use trimethoprim?
birth to 2 years of age approximately

Trimethoprim: which time schedule do you use? [Day(s) before VUS/VCUG, how many?]
Yes

Trimethoprim: which time schedule do you use? [Day(s) before VUS/VCUG, how many?][Comment]
1 day before (12 hours before usually)

Trimethoprim: which time schedule do you use? [Day of VUS/VCUG]
Yes

Trimethoprim: which time schedule do you use? [Day of VUS/VCUG][Comment]

Trimethoprim: which time schedule do you use? [Day(s) after VUS/VCUG, how many?]
Yes

Trimethoprim: which time schedule do you use? [Day(s) after VUS/VCUG, how many?][Comment]
2 days after (i.e. 48 hours)

Nitrofurantoin: which time schedule do you use? [Day(s) before VUS/VCUG, how many?]
N/A

Nitrofurantoin: which time schedule do you use? [Day of VUS/VCUG]
N/A

Nitrofurantoin: which time schedule do you use? [Day(s) after VUS/VCUG, how many?]
N/A

Amoxicillin: which time schedule do you use? [Day(s) before VUS/VCUG, how many?]
N/A

Amoxicillin: which time schedule do you use? [Day of VUS/VCUG]
N/A

Amoxicillin: which time schedule do you use? [Day(s) after VUS/VCUG, how many?]
N/A

Cefaclor: in what dosage?
prophylactic (10 mg/kg bodyweight/d)

Cefaclor: in what dosage? [Other]

Cefaclor: for which age group do you use cefaclor?
birth to 2 years of age approximately if trimethoprim not suitable for use

Cefaclor: which time schedule do you use? [Days(s) before VUS/VCUG, how many?]
Yes

Cefaclor: which time schedule do you use? [Days(s) before VUS/VCUG, how many?][Comment]
1 day before (12 hours before usually)

Cefaclor: which time schedule do you use? [Day of VUS/VCUG]
Yes

Cefaclor: which time schedule do you use? [Day of VUS/VCUG][Comment]

Cefaclor: which time schedule do you use? [Day(s) after VUS/VCUG, how many?]
Yes

Cefaclor: which time schedule do you use? [Day(s) after VUS/VCUG, how many?][Comment]
2 days after test (48 hours)

Amoxicillin/clavulanic acid: in what dosage? (Amoxicillin portion) [prophylaktic (10-20 mg/Kg bodyweight/d)]
No

Amoxicillin/clavulanic acid: in what dosage? (Amoxicillin portion) [double prophylactic (20-40 mg/Kg bodyweight/d)]
Yes

Amoxicillin/clavulanic acid: in what dosage? (Amoxicillin portion) [therapeutic (80-100 mg/Kg bodyweight/d)]
No

Amoxicillin/clavulanic acid: in what dosage? (Amoxicillin portion) [Other]

Amoxicillin/clavulanic acid: for which age group do you use amoxicillin/clavulanic acid?
birth to two years of age approximately (if trimethoprim unsuitable for use)

Amoxicillin/clavulanic acid: which time schedule do you use? [Day(s) before VUS/VCUG]
Yes

Amoxicillin/clavulanic acid: which time schedule do you use? [Day(s) before VUS/VCUG][Comment]
1 day (12 hours before test usually)

Amoxicillin/clavulanic acid: which time schedule do you use? [Day of VUS/VCUG]
Yes

Amoxicillin/clavulanic acid: which time schedule do you use? [Day of VUS/VCUG][Comment]

Amoxicillin/clavulanic acid: which time schedule do you use? [Day(s) after VUS/VCUG]
Yes

Amoxicillin/clavulanic acid: which time schedule do you use? [Day(s) after VUS/VCUG][Comment]
2 days after test (48 hours)

Other: which medication and in what dosage?
IV gentamycin if doing an VCUG in a boy less than 3 months of age (for query posterior urethral valves)

Other: for which age group do you use this medication?
boys less than 3 months of age

Other: which time schedule do you use? [Day(s) before VUS/VCUG, how many?]
Yes

Other: which time schedule do you use? [Day(s) before VUS/VCUG, how many?][Comment]
the day of the examination only IV

Other: which time schedule do you use? [Day of VUS/VCUG]
Yes

Other: which time schedule do you use? [Day of VUS/VCUG][Comment]

Other: which time schedule do you use? [Day(s) after VUS/VCUG, how many?]
No

Other: which time schedule do you use? [Day(s) after VUS/VCUG, how many?][Comment]

Indication for prophylaxis

Which patients receive periinterventional antibiotic prophylaxis? (multiple answers possible) [all]
Yes

Which patients receive periinterventional antibiotic prophylaxis? (multiple answers possible) [patients post urinary tract infection/pyelonephritis/urosepsis]
N/A

Which patients receive periinterventional antibiotic prophylaxis? (multiple answers possible) [patients with urinary tract dilation in ultrasound]
N/A

Which patients receive periinterventional antibiotic prophylaxis? (multiple answers possible) [patients with prenatal urinary tract dilation]
N/A

Which patients receive periinterventional antibiotic prophylaxis? (multiple answers possible) [patients post VUR surgery]
N/A

Which patients receive periinterventional antibiotic prophylaxis? (multiple answers possible) [none]
N/A

Are there situations or clinical findings in the patient where you deviate from your prophylaxis standard (medication, dose, duration, age of the patient)? (multiple answers possible, please specify in answer box) [in case of already known vesicoureteral reflux (VUR)]
No

Are there situations or clinical findings in the patient where you deviate from your prophylaxis standard (medication, dose, duration, age of the patient)? (multiple answers possible, please specify in answer box) [in case of already known vesicoureteral reflux (VUR)][Comment]

Are there situations or clinical findings in the patient where you deviate from your prophylaxis standard (medication, dose, duration, age of the patient)? (multiple answers possible, please specify in answer box) [in patients with recurrent urinary tract infections (UTI)]
No

Are there situations or clinical findings in the patient where you deviate from your prophylaxis standard (medication, dose, duration, age of the patient)? (multiple answers possible, please specify in answer box) [in patients with recurrent urinary tract infections (UTI)][Comment]

Are there situations or clinical findings in the patient where you deviate from your prophylaxis standard (medication, dose, duration, age of the patient)? (multiple answers possible, please specify in answer box) [if multidrug-resistant organisms (MDRO) are detected in urine cultures]
Yes

Are there situations or clinical findings in the patient where you deviate from your prophylaxis standard (medication, dose, duration, age of the patient)? (multiple answers possible, please specify in answer box) [if multidrug-resistant organisms (MDRO) are detected in urine cultures][Comment]
the choice of antibiotic depends on the sensitivities of the organisms on growth

Are there situations or clinical findings in the patient where you deviate from your prophylaxis standard (medication, dose, duration, age of the patient)? (multiple answers possible, please specify in answer box) [in newborns]
Yes

Are there situations or clinical findings in the patient where you deviate from your prophylaxis standard (medication, dose, duration, age of the patient)? (multiple answers possible, please specify in answer box) [in newborns][Comment]
IV gentamycin given only in this circumstance

Are there situations or clinical findings in the patient where you deviate from your prophylaxis standard (medication, dose, duration, age of the patient)? (multiple answers possible, please specify in answer box) [Other]

Are there situations or clinical findings in the patient where you deviate from your prophylaxis standard (medication, dose, duration, age of the patient)? (multiple answers possible, please specify in answer box) [Other comment]

Are there situations in which you use intravenous prophylaxis?
Yes

In which situations?
We usually do not preform VCUG in children less than 3 months of age. The only exception to this is in boys where there is a clinical and imaging query of posterior urethral valves where we do the test in the newborn with antibiotic prophylaxsis of IV gentamycin

Voiding analysis

Are there differences in antibiotic prophylaxis between VUS and VCUG in your department?
No

Is a non-pathological urine strip test required in preparation for VUS/VCUG in your department?
No

Is a non-pathological urine culture required in preparation for VUS/VCUG in your department?
Yes

What is the maximum amount of days accepted for urine culture to be performed prior to the exam?
one week

Do patients only receive post-procedure prophylaxis if vesicoureteral reflux (VUR) is detected in VUS/VCUG?
No

Do other MUS/MCU findings (e.g. urethral valves) have an influence on post-procedure prophylaxis?
No

End of survey

Thank you for taking the time to answer this survey!

May we contact you personally if we have further questions?
Yes

E-mail address:
eilish.twomey@childrenshealthireland.ie

Survey response 211

Response ID
213

Last page
1

Start language
en

Seed
1013452224

Date started
2025-03-30 17:40:05

Date last action
2025-03-30 17:40:28

Standard Operation Procedure

Who fills out this survey?
Pediatric Radiologist

Who fills out this survey? [Other]

In which type of hospital are you employed?
University Hospital

In which type of hospital are you employed? [Other]

In which country do you work?
Slovenia

Does your department have a defined protocol for infection prophylaxis in the context of VUS/VCUG?
Yes

Has this protocol been transformed into a written standard operating procedure (SOP)?
Yes

Which department is the author of the SOP for infection prophylaxis in VUS/VCUG?
Pediatrics

Which department is the author of the SOP for infection prophylaxis in VUS/VCUG? [Other]

Antibiotic Prophylaxis

Are there situations/constellations in which you do not use antibiotic prophylaxis for VUS/VCUG?
N/A

Please specify these situations/constellations: [girl with first uncomplicated UTI without urinary tract dilation in ultrasound]
N/A

Please specify these situations/constellations: [boy with first uncomplicated UTI without urinary tract dilation in ultrasound]
N/A

Please specify these situations/constellations: [in patients with low grade urinary tract dilation (up to 2nd grade) and no UTI in medical history]
N/A

If you use antibiotic prophylaxis: which mediaction do you use? (multiple answers and subdivision into age groups possible) [Trimethoprim]
N/A

If you use antibiotic prophylaxis: which mediaction do you use? (multiple answers and subdivision into age groups possible) [Nitrofurantoin]
N/A

If you use antibiotic prophylaxis: which mediaction do you use? (multiple answers and subdivision into age groups possible) [Amoxicillin]
N/A

If you use antibiotic prophylaxis: which mediaction do you use? (multiple answers and subdivision into age groups possible) [Cefaclor]
N/A

If you use antibiotic prophylaxis: which mediaction do you use? (multiple answers and subdivision into age groups possible) [Amoxicillin/clavulanic acid]
N/A

If you use antibiotic prophylaxis: which mediaction do you use? (multiple answers and subdivision into age groups possible) [Other]
N/A

Trimethoprim: which time schedule do you use? [Day(s) before VUS/VCUG, how many?]
N/A

Trimethoprim: which time schedule do you use? [Day of VUS/VCUG]
N/A

Trimethoprim: which time schedule do you use? [Day(s) after VUS/VCUG, how many?]
N/A

Nitrofurantoin: which time schedule do you use? [Day(s) before VUS/VCUG, how many?]
N/A

Nitrofurantoin: which time schedule do you use? [Day of VUS/VCUG]
N/A

Nitrofurantoin: which time schedule do you use? [Day(s) after VUS/VCUG, how many?]
N/A

Amoxicillin: which time schedule do you use? [Day(s) before VUS/VCUG, how many?]
N/A

Amoxicillin: which time schedule do you use? [Day of VUS/VCUG]
N/A

Amoxicillin: which time schedule do you use? [Day(s) after VUS/VCUG, how many?]
N/A

Cefaclor: which time schedule do you use? [Days(s) before VUS/VCUG, how many?]
N/A

Cefaclor: which time schedule do you use? [Day of VUS/VCUG]
N/A

Cefaclor: which time schedule do you use? [Day(s) after VUS/VCUG, how many?]
N/A

Amoxicillin/clavulanic acid: in what dosage? (Amoxicillin portion) [prophylaktic (10-20 mg/Kg bodyweight/d)]
N/A

Amoxicillin/clavulanic acid: in what dosage? (Amoxicillin portion) [double prophylactic (20-40 mg/Kg bodyweight/d)]
N/A

Amoxicillin/clavulanic acid: in what dosage? (Amoxicillin portion) [therapeutic (80-100 mg/Kg bodyweight/d)]
N/A

Amoxicillin/clavulanic acid: which time schedule do you use? [Day(s) before VUS/VCUG]
N/A

Amoxicillin/clavulanic acid: which time schedule do you use? [Day of VUS/VCUG]
N/A

Amoxicillin/clavulanic acid: which time schedule do you use? [Day(s) after VUS/VCUG]
N/A

Other: which time schedule do you use? [Day(s) before VUS/VCUG, how many?]
N/A

Other: which time schedule do you use? [Day of VUS/VCUG]
N/A

Other: which time schedule do you use? [Day(s) after VUS/VCUG, how many?]
N/A

Indication for prophylaxis

Which patients receive periinterventional antibiotic prophylaxis? (multiple answers possible) [all]
N/A

Which patients receive periinterventional antibiotic prophylaxis? (multiple answers possible) [patients post urinary tract infection/pyelonephritis/urosepsis]
N/A

Which patients receive periinterventional antibiotic prophylaxis? (multiple answers possible) [patients with urinary tract dilation in ultrasound]
N/A

Which patients receive periinterventional antibiotic prophylaxis? (multiple answers possible) [patients with prenatal urinary tract dilation]
N/A

Which patients receive periinterventional antibiotic prophylaxis? (multiple answers possible) [patients post VUR surgery]
N/A

Which patients receive periinterventional antibiotic prophylaxis? (multiple answers possible) [none]
N/A

Are there situations or clinical findings in the patient where you deviate from your prophylaxis standard (medication, dose, duration, age of the patient)? (multiple answers possible, please specify in answer box) [in case of already known vesicoureteral reflux (VUR)]
N/A

Are there situations or clinical findings in the patient where you deviate from your prophylaxis standard (medication, dose, duration, age of the patient)? (multiple answers possible, please specify in answer box) [in patients with recurrent urinary tract infections (UTI)]
N/A

Are there situations or clinical findings in the patient where you deviate from your prophylaxis standard (medication, dose, duration, age of the patient)? (multiple answers possible, please specify in answer box) [if multidrug-resistant organisms (MDRO) are detected in urine cultures]
N/A

Are there situations or clinical findings in the patient where you deviate from your prophylaxis standard (medication, dose, duration, age of the patient)? (multiple answers possible, please specify in answer box) [in newborns]
N/A

Are there situations in which you use intravenous prophylaxis?
N/A

Voiding analysis

Are there differences in antibiotic prophylaxis between VUS and VCUG in your department?
N/A

Is a non-pathological urine strip test required in preparation for VUS/VCUG in your department?
N/A

Is a non-pathological urine culture required in preparation for VUS/VCUG in your department?
N/A

Do patients only receive post-procedure prophylaxis if vesicoureteral reflux (VUR) is detected in VUS/VCUG?
N/A

Do other MUS/MCU findings (e.g. urethral valves) have an influence on post-procedure prophylaxis?
N/A

End of survey

May we contact you personally if we have further questions?
N/A

Survey response 212

Response ID
214

Date submitted
2025-03-31 08:56:34

Last page
5

Start language
en

Seed
1995460348

Date started
2025-03-31 08:39:24

Date last action
2025-03-31 08:56:34

Standard Operation Procedure

Who fills out this survey?
Pediatric Radiologist

Who fills out this survey? [Other]

In which type of hospital are you employed?
University Hospital

In which type of hospital are you employed? [Other]

In which country do you work?
Norway

Does your department have a defined protocol for infection prophylaxis in the context of VUS/VCUG?
Yes

Has this protocol been transformed into a written standard operating procedure (SOP)?
Yes

Which department is the author of the SOP for infection prophylaxis in VUS/VCUG?
Pediatrics

Which department is the author of the SOP for infection prophylaxis in VUS/VCUG? [Other]

Antibiotic Prophylaxis

Are there situations/constellations in which you do not use antibiotic prophylaxis for VUS/VCUG?
No

Please specify these situations/constellations: [girl with first uncomplicated UTI without urinary tract dilation in ultrasound]
N/A

Please specify these situations/constellations: [boy with first uncomplicated UTI without urinary tract dilation in ultrasound]
N/A

Please specify these situations/constellations: [in patients with low grade urinary tract dilation (up to 2nd grade) and no UTI in medical history]
N/A

If you use antibiotic prophylaxis: which mediaction do you use? (multiple answers and subdivision into age groups possible) [Trimethoprim]
Yes

If you use antibiotic prophylaxis: which mediaction do you use? (multiple answers and subdivision into age groups possible) [Nitrofurantoin]
Yes

If you use antibiotic prophylaxis: which mediaction do you use? (multiple answers and subdivision into age groups possible) [Amoxicillin]
No

If you use antibiotic prophylaxis: which mediaction do you use? (multiple answers and subdivision into age groups possible) [Cefaclor]
No

If you use antibiotic prophylaxis: which mediaction do you use? (multiple answers and subdivision into age groups possible) [Amoxicillin/clavulanic acid]
No

If you use antibiotic prophylaxis: which mediaction do you use? (multiple answers and subdivision into age groups possible) [Other]
No

Trimethoprim: in what dosage?
therapeutic (6 mg/kg bodyweight/d)

Trimethoprim: in what dosage? [Other]

Trimethoprim: for which age group do you use trimethoprim?
We use Trimethoprim when the child is already on this mediacation as a prophylaxis

Trimethoprim: which time schedule do you use? [Day(s) before VUS/VCUG, how many?]
No

Trimethoprim: which time schedule do you use? [Day(s) before VUS/VCUG, how many?][Comment]

Trimethoprim: which time schedule do you use? [Day of VUS/VCUG]
Yes

Trimethoprim: which time schedule do you use? [Day of VUS/VCUG][Comment]

Trimethoprim: which time schedule do you use? [Day(s) after VUS/VCUG, how many?]
No

Trimethoprim: which time schedule do you use? [Day(s) after VUS/VCUG, how many?][Comment]

Nitrofurantoin: in what dosage?
double prophylactic (2-4 mg/kg bodyweight/d)

Nitrofurantoin: in what dosage? [Other]

Nitrofurantoin: for which age group do you use nitrofurantoin?
Children age over 1 mnd

Nitrofurantoin: which time schedule do you use? [Day(s) before VUS/VCUG, how many?]
No

Nitrofurantoin: which time schedule do you use? [Day(s) before VUS/VCUG, how many?][Comment]

Nitrofurantoin: which time schedule do you use? [Day of VUS/VCUG]
Yes

Nitrofurantoin: which time schedule do you use? [Day of VUS/VCUG][Comment]

Nitrofurantoin: which time schedule do you use? [Day(s) after VUS/VCUG, how many?]
No

Nitrofurantoin: which time schedule do you use? [Day(s) after VUS/VCUG, how many?][Comment]

Amoxicillin: which time schedule do you use? [Day(s) before VUS/VCUG, how many?]
N/A

Amoxicillin: which time schedule do you use? [Day of VUS/VCUG]
N/A

Amoxicillin: which time schedule do you use? [Day(s) after VUS/VCUG, how many?]
N/A

Cefaclor: which time schedule do you use? [Days(s) before VUS/VCUG, how many?]
N/A

Cefaclor: which time schedule do you use? [Day of VUS/VCUG]
N/A

Cefaclor: which time schedule do you use? [Day(s) after VUS/VCUG, how many?]
N/A

Amoxicillin/clavulanic acid: in what dosage? (Amoxicillin portion) [prophylaktic (10-20 mg/Kg bodyweight/d)]
N/A

Amoxicillin/clavulanic acid: in what dosage? (Amoxicillin portion) [double prophylactic (20-40 mg/Kg bodyweight/d)]
N/A

Amoxicillin/clavulanic acid: in what dosage? (Amoxicillin portion) [therapeutic (80-100 mg/Kg bodyweight/d)]
N/A

Amoxicillin/clavulanic acid: which time schedule do you use? [Day(s) before VUS/VCUG]
N/A

Amoxicillin/clavulanic acid: which time schedule do you use? [Day of VUS/VCUG]
N/A

Amoxicillin/clavulanic acid: which time schedule do you use? [Day(s) after VUS/VCUG]
N/A

Other: which time schedule do you use? [Day(s) before VUS/VCUG, how many?]
N/A

Other: which time schedule do you use? [Day of VUS/VCUG]
N/A

Other: which time schedule do you use? [Day(s) after VUS/VCUG, how many?]
N/A

Indication for prophylaxis

Which patients receive periinterventional antibiotic prophylaxis? (multiple answers possible) [all]
Yes

Which patients receive periinterventional antibiotic prophylaxis? (multiple answers possible) [patients post urinary tract infection/pyelonephritis/urosepsis]
N/A

Which patients receive periinterventional antibiotic prophylaxis? (multiple answers possible) [patients with urinary tract dilation in ultrasound]
N/A

Which patients receive periinterventional antibiotic prophylaxis? (multiple answers possible) [patients with prenatal urinary tract dilation]
N/A

Which patients receive periinterventional antibiotic prophylaxis? (multiple answers possible) [patients post VUR surgery]
N/A

Which patients receive periinterventional antibiotic prophylaxis? (multiple answers possible) [none]
N/A

Are there situations or clinical findings in the patient where you deviate from your prophylaxis standard (medication, dose, duration, age of the patient)? (multiple answers possible, please specify in answer box) [in case of already known vesicoureteral reflux (VUR)]
Yes

Are there situations or clinical findings in the patient where you deviate from your prophylaxis standard (medication, dose, duration, age of the patient)? (multiple answers possible, please specify in answer box) [in case of already known vesicoureteral reflux (VUR)][Comment]
Pediatrician order the post prosedyral medication in risk-patients

Are there situations or clinical findings in the patient where you deviate from your prophylaxis standard (medication, dose, duration, age of the patient)? (multiple answers possible, please specify in answer box) [in patients with recurrent urinary tract infections (UTI)]
Yes

Are there situations or clinical findings in the patient where you deviate from your prophylaxis standard (medication, dose, duration, age of the patient)? (multiple answers possible, please specify in answer box) [in patients with recurrent urinary tract infections (UTI)][Comment]
Pediatrician order the post prosedyral medication in risk-patients

Are there situations or clinical findings in the patient where you deviate from your prophylaxis standard (medication, dose, duration, age of the patient)? (multiple answers possible, please specify in answer box) [if multidrug-resistant organisms (MDRO) are detected in urine cultures]
Yes

Are there situations or clinical findings in the patient where you deviate from your prophylaxis standard (medication, dose, duration, age of the patient)? (multiple answers possible, please specify in answer box) [if multidrug-resistant organisms (MDRO) are detected in urine cultures][Comment]
Pediatrician order the post prosedyral medication in risk-patients

Are there situations or clinical findings in the patient where you deviate from your prophylaxis standard (medication, dose, duration, age of the patient)? (multiple answers possible, please specify in answer box) [in newborns]
Yes

Are there situations or clinical findings in the patient where you deviate from your prophylaxis standard (medication, dose, duration, age of the patient)? (multiple answers possible, please specify in answer box) [in newborns][Comment]
Pediatrician order the post prosedyral medication in newborns

Are there situations or clinical findings in the patient where you deviate from your prophylaxis standard (medication, dose, duration, age of the patient)? (multiple answers possible, please specify in answer box) [Other]

Are there situations or clinical findings in the patient where you deviate from your prophylaxis standard (medication, dose, duration, age of the patient)? (multiple answers possible, please specify in answer box) [Other comment]

Are there situations in which you use intravenous prophylaxis?
No

Voiding analysis

Are there differences in antibiotic prophylaxis between VUS and VCUG in your department?
No

Is a non-pathological urine strip test required in preparation for VUS/VCUG in your department?
No

Is a non-pathological urine culture required in preparation for VUS/VCUG in your department?
No

Do patients only receive post-procedure prophylaxis if vesicoureteral reflux (VUR) is detected in VUS/VCUG?
Yes

Please explain your approach:
Traditionally its enough

Do other MUS/MCU findings (e.g. urethral valves) have an influence on post-procedure prophylaxis?
Yes

Please specify:
With anatomical findings like a uretheral valve a pediatrician will order the post procedure prophylaxis

End of survey

Thank you for taking the time to answer this survey!

May we contact you personally if we have further questions?
No

Survey response 213

Response ID
215

Date submitted
2025-03-31 14:23:42

Last page
5

Start language
en

Seed
372126151

Date started
2025-03-31 14:17:39

Date last action
2025-03-31 14:23:42

Standard Operation Procedure

Who fills out this survey?
Pediatric nephrologist

Who fills out this survey? [Other]

In which type of hospital are you employed?
Community hospital (>50 beds for pediatric patients)

In which type of hospital are you employed? [Other]

In which country do you work?
Germany

Does your department have a defined protocol for infection prophylaxis in the context of VUS/VCUG?
No

Has this protocol been transformed into a written standard operating procedure (SOP)?
N/A

Antibiotic Prophylaxis

Are there situations/constellations in which you do not use antibiotic prophylaxis for VUS/VCUG?
No

Please specify these situations/constellations: [girl with first uncomplicated UTI without urinary tract dilation in ultrasound]
N/A

Please specify these situations/constellations: [boy with first uncomplicated UTI without urinary tract dilation in ultrasound]
N/A

Please specify these situations/constellations: [in patients with low grade urinary tract dilation (up to 2nd grade) and no UTI in medical history]
N/A

If you use antibiotic prophylaxis: which mediaction do you use? (multiple answers and subdivision into age groups possible) [Trimethoprim]
Yes

If you use antibiotic prophylaxis: which mediaction do you use? (multiple answers and subdivision into age groups possible) [Nitrofurantoin]
Yes

If you use antibiotic prophylaxis: which mediaction do you use? (multiple answers and subdivision into age groups possible) [Amoxicillin]
No

If you use antibiotic prophylaxis: which mediaction do you use? (multiple answers and subdivision into age groups possible) [Cefaclor]
Yes

If you use antibiotic prophylaxis: which mediaction do you use? (multiple answers and subdivision into age groups possible) [Amoxicillin/clavulanic acid]
No

If you use antibiotic prophylaxis: which mediaction do you use? (multiple answers and subdivision into age groups possible) [Other]
No

Trimethoprim: in what dosage?
Other

Trimethoprim: in what dosage? [Other]
1 mg/kf bw

Trimethoprim: for which age group do you use trimethoprim?
since birth, no restriction

Trimethoprim: which time schedule do you use? [Day(s) before VUS/VCUG, how many?]
No

Trimethoprim: which time schedule do you use? [Day(s) before VUS/VCUG, how many?][Comment]

Trimethoprim: which time schedule do you use? [Day of VUS/VCUG]
No

Trimethoprim: which time schedule do you use? [Day of VUS/VCUG][Comment]

Trimethoprim: which time schedule do you use? [Day(s) after VUS/VCUG, how many?]
Yes

Trimethoprim: which time schedule do you use? [Day(s) after VUS/VCUG, how many?][Comment]
1 day, but most patients on continous prophylaxis

Nitrofurantoin: in what dosage?
prophylactic (1-2 mg/kg bodyweight/d)

Nitrofurantoin: in what dosage? [Other]

Nitrofurantoin: for which age group do you use nitrofurantoin?
after 4 to 6 months

Nitrofurantoin: which time schedule do you use? [Day(s) before VUS/VCUG, how many?]
No

Nitrofurantoin: which time schedule do you use? [Day(s) before VUS/VCUG, how many?][Comment]

Nitrofurantoin: which time schedule do you use? [Day of VUS/VCUG]
No

Nitrofurantoin: which time schedule do you use? [Day of VUS/VCUG][Comment]

Nitrofurantoin: which time schedule do you use? [Day(s) after VUS/VCUG, how many?]
Yes

Nitrofurantoin: which time schedule do you use? [Day(s) after VUS/VCUG, how many?][Comment]
1 day, but most patients on continous prophylaxis

Amoxicillin: which time schedule do you use? [Day(s) before VUS/VCUG, how many?]
N/A

Amoxicillin: which time schedule do you use? [Day of VUS/VCUG]
N/A

Amoxicillin: which time schedule do you use? [Day(s) after VUS/VCUG, how many?]
N/A

Cefaclor: in what dosage?
prophylactic (10 mg/kg bodyweight/d)

Cefaclor: in what dosage? [Other]

Cefaclor: for which age group do you use cefaclor?
since births

Cefaclor: which time schedule do you use? [Days(s) before VUS/VCUG, how many?]
No

Cefaclor: which time schedule do you use? [Days(s) before VUS/VCUG, how many?][Comment]

Cefaclor: which time schedule do you use? [Day of VUS/VCUG]
No

Cefaclor: which time schedule do you use? [Day of VUS/VCUG][Comment]

Cefaclor: which time schedule do you use? [Day(s) after VUS/VCUG, how many?]
Yes

Cefaclor: which time schedule do you use? [Day(s) after VUS/VCUG, how many?][Comment]
1 day, but most patients on continous prophylaxis

Amoxicillin/clavulanic acid: in what dosage? (Amoxicillin portion) [prophylaktic (10-20 mg/Kg bodyweight/d)]
N/A

Amoxicillin/clavulanic acid: in what dosage? (Amoxicillin portion) [double prophylactic (20-40 mg/Kg bodyweight/d)]
N/A

Amoxicillin/clavulanic acid: in what dosage? (Amoxicillin portion) [therapeutic (80-100 mg/Kg bodyweight/d)]
N/A

Amoxicillin/clavulanic acid: which time schedule do you use? [Day(s) before VUS/VCUG]
N/A

Amoxicillin/clavulanic acid: which time schedule do you use? [Day of VUS/VCUG]
N/A

Amoxicillin/clavulanic acid: which time schedule do you use? [Day(s) after VUS/VCUG]
N/A

Other: which time schedule do you use? [Day(s) before VUS/VCUG, how many?]
N/A

Other: which time schedule do you use? [Day of VUS/VCUG]
N/A

Other: which time schedule do you use? [Day(s) after VUS/VCUG, how many?]
N/A

Indication for prophylaxis

Which patients receive periinterventional antibiotic prophylaxis? (multiple answers possible) [all]
Yes

Which patients receive periinterventional antibiotic prophylaxis? (multiple answers possible) [patients post urinary tract infection/pyelonephritis/urosepsis]
N/A

Which patients receive periinterventional antibiotic prophylaxis? (multiple answers possible) [patients with urinary tract dilation in ultrasound]
N/A

Which patients receive periinterventional antibiotic prophylaxis? (multiple answers possible) [patients with prenatal urinary tract dilation]
N/A

Which patients receive periinterventional antibiotic prophylaxis? (multiple answers possible) [patients post VUR surgery]
N/A

Which patients receive periinterventional antibiotic prophylaxis? (multiple answers possible) [none]
N/A

Are there situations or clinical findings in the patient where you deviate from your prophylaxis standard (medication, dose, duration, age of the patient)? (multiple answers possible, please specify in answer box) [in case of already known vesicoureteral reflux (VUR)]
Yes

Are there situations or clinical findings in the patient where you deviate from your prophylaxis standard (medication, dose, duration, age of the patient)? (multiple answers possible, please specify in answer box) [in case of already known vesicoureteral reflux (VUR)][Comment]
they are on continous prophylaxis

Are there situations or clinical findings in the patient where you deviate from your prophylaxis standard (medication, dose, duration, age of the patient)? (multiple answers possible, please specify in answer box) [in patients with recurrent urinary tract infections (UTI)]
No

Are there situations or clinical findings in the patient where you deviate from your prophylaxis standard (medication, dose, duration, age of the patient)? (multiple answers possible, please specify in answer box) [in patients with recurrent urinary tract infections (UTI)][Comment]

Are there situations or clinical findings in the patient where you deviate from your prophylaxis standard (medication, dose, duration, age of the patient)? (multiple answers possible, please specify in answer box) [if multidrug-resistant organisms (MDRO) are detected in urine cultures]
Yes

Are there situations or clinical findings in the patient where you deviate from your prophylaxis standard (medication, dose, duration, age of the patient)? (multiple answers possible, please specify in answer box) [if multidrug-resistant organisms (MDRO) are detected in urine cultures][Comment]
only after having done urin culture before

Are there situations or clinical findings in the patient where you deviate from your prophylaxis standard (medication, dose, duration, age of the patient)? (multiple answers possible, please specify in answer box) [in newborns]
No

Are there situations or clinical findings in the patient where you deviate from your prophylaxis standard (medication, dose, duration, age of the patient)? (multiple answers possible, please specify in answer box) [in newborns][Comment]

Are there situations or clinical findings in the patient where you deviate from your prophylaxis standard (medication, dose, duration, age of the patient)? (multiple answers possible, please specify in answer box) [Other]

Are there situations or clinical findings in the patient where you deviate from your prophylaxis standard (medication, dose, duration, age of the patient)? (multiple answers possible, please specify in answer box) [Other comment]

Are there situations in which you use intravenous prophylaxis?
No

Voiding analysis

Are there differences in antibiotic prophylaxis between VUS and VCUG in your department?
No

Is a non-pathological urine strip test required in preparation for VUS/VCUG in your department?
No

Is a non-pathological urine culture required in preparation for VUS/VCUG in your department?
No

Do patients only receive post-procedure prophylaxis if vesicoureteral reflux (VUR) is detected in VUS/VCUG?
No

Do other MUS/MCU findings (e.g. urethral valves) have an influence on post-procedure prophylaxis?
No

End of survey

Thank you for taking the time to answer this survey!

May we contact you personally if we have further questions?
Yes

E-mail address:
m.bald@klinikum-stuttgart.de

Survey response 214

Response ID
216

Date submitted
2025-04-01 10:24:51

Last page
5

Start language
en

Seed
1060095485

Date started
2025-04-01 10:05:43

Date last action
2025-04-01 10:24:51

Standard Operation Procedure

Who fills out this survey?
Radiologist

Who fills out this survey? [Other]

In which type of hospital are you employed?
Community hospital (>50 beds for pediatric patients)

In which type of hospital are you employed? [Other]

In which country do you work?
Austria

Does your department have a defined protocol for infection prophylaxis in the context of VUS/VCUG?
Yes

Has this protocol been transformed into a written standard operating procedure (SOP)?
Yes

Which department is the author of the SOP for infection prophylaxis in VUS/VCUG?
Other

Which department is the author of the SOP for infection prophylaxis in VUS/VCUG? [Other]
Radiology in interdisciplinary accordance with pediatrics

Antibiotic Prophylaxis

Are there situations/constellations in which you do not use antibiotic prophylaxis for VUS/VCUG?
No

Please specify these situations/constellations: [girl with first uncomplicated UTI without urinary tract dilation in ultrasound]
N/A

Please specify these situations/constellations: [boy with first uncomplicated UTI without urinary tract dilation in ultrasound]
N/A

Please specify these situations/constellations: [in patients with low grade urinary tract dilation (up to 2nd grade) and no UTI in medical history]
N/A

If you use antibiotic prophylaxis: which mediaction do you use? (multiple answers and subdivision into age groups possible) [Trimethoprim]
Yes

If you use antibiotic prophylaxis: which mediaction do you use? (multiple answers and subdivision into age groups possible) [Nitrofurantoin]
No

If you use antibiotic prophylaxis: which mediaction do you use? (multiple answers and subdivision into age groups possible) [Amoxicillin]
No

If you use antibiotic prophylaxis: which mediaction do you use? (multiple answers and subdivision into age groups possible) [Cefaclor]
No

If you use antibiotic prophylaxis: which mediaction do you use? (multiple answers and subdivision into age groups possible) [Amoxicillin/clavulanic acid]
Yes

If you use antibiotic prophylaxis: which mediaction do you use? (multiple answers and subdivision into age groups possible) [Other]
Yes

Trimethoprim: in what dosage?
prophylactic (2 mg/kg bodyweight/d)

Trimethoprim: in what dosage? [Other]

Trimethoprim: for which age group do you use trimethoprim?
0-18

Trimethoprim: which time schedule do you use? [Day(s) before VUS/VCUG, how many?]
Yes

Trimethoprim: which time schedule do you use? [Day(s) before VUS/VCUG, how many?][Comment]
1

Trimethoprim: which time schedule do you use? [Day of VUS/VCUG]
Yes

Trimethoprim: which time schedule do you use? [Day of VUS/VCUG][Comment]

Trimethoprim: which time schedule do you use? [Day(s) after VUS/VCUG, how many?]
Yes

Trimethoprim: which time schedule do you use? [Day(s) after VUS/VCUG, how many?][Comment]
2-3

Nitrofurantoin: which time schedule do you use? [Day(s) before VUS/VCUG, how many?]
N/A

Nitrofurantoin: which time schedule do you use? [Day of VUS/VCUG]
N/A

Nitrofurantoin: which time schedule do you use? [Day(s) after VUS/VCUG, how many?]
N/A

Amoxicillin: which time schedule do you use? [Day(s) before VUS/VCUG, how many?]
N/A

Amoxicillin: which time schedule do you use? [Day of VUS/VCUG]
N/A

Amoxicillin: which time schedule do you use? [Day(s) after VUS/VCUG, how many?]
N/A

Cefaclor: which time schedule do you use? [Days(s) before VUS/VCUG, how many?]
N/A

Cefaclor: which time schedule do you use? [Day of VUS/VCUG]
N/A

Cefaclor: which time schedule do you use? [Day(s) after VUS/VCUG, how many?]
N/A

Amoxicillin/clavulanic acid: in what dosage? (Amoxicillin portion) [prophylaktic (10-20 mg/Kg bodyweight/d)]
No

Amoxicillin/clavulanic acid: in what dosage? (Amoxicillin portion) [double prophylactic (20-40 mg/Kg bodyweight/d)]
Yes

Amoxicillin/clavulanic acid: in what dosage? (Amoxicillin portion) [therapeutic (80-100 mg/Kg bodyweight/d)]
No

Amoxicillin/clavulanic acid: in what dosage? (Amoxicillin portion) [Other]

Amoxicillin/clavulanic acid: for which age group do you use amoxicillin/clavulanic acid?
0-18

Amoxicillin/clavulanic acid: which time schedule do you use? [Day(s) before VUS/VCUG]
Yes

Amoxicillin/clavulanic acid: which time schedule do you use? [Day(s) before VUS/VCUG][Comment]
1

Amoxicillin/clavulanic acid: which time schedule do you use? [Day of VUS/VCUG]
Yes

Amoxicillin/clavulanic acid: which time schedule do you use? [Day of VUS/VCUG][Comment]

Amoxicillin/clavulanic acid: which time schedule do you use? [Day(s) after VUS/VCUG]
Yes

Amoxicillin/clavulanic acid: which time schedule do you use? [Day(s) after VUS/VCUG][Comment]
2-3

Other: which medication and in what dosage?
according to urine culture, about 30-40% to normal therapeutic dose

Other: for which age group do you use this medication?
0-18

Other: which time schedule do you use? [Day(s) before VUS/VCUG, how many?]
Yes

Other: which time schedule do you use? [Day(s) before VUS/VCUG, how many?][Comment]
1

Other: which time schedule do you use? [Day of VUS/VCUG]
Yes

Other: which time schedule do you use? [Day of VUS/VCUG][Comment]

Other: which time schedule do you use? [Day(s) after VUS/VCUG, how many?]
Yes

Other: which time schedule do you use? [Day(s) after VUS/VCUG, how many?][Comment]
2-3

Indication for prophylaxis

Which patients receive periinterventional antibiotic prophylaxis? (multiple answers possible) [all]
No

Which patients receive periinterventional antibiotic prophylaxis? (multiple answers possible) [patients post urinary tract infection/pyelonephritis/urosepsis]
No

Which patients receive periinterventional antibiotic prophylaxis? (multiple answers possible) [patients with urinary tract dilation in ultrasound]
No

Which patients receive periinterventional antibiotic prophylaxis? (multiple answers possible) [patients with prenatal urinary tract dilation]
No

Which patients receive periinterventional antibiotic prophylaxis? (multiple answers possible) [patients post VUR surgery]
No

Which patients receive periinterventional antibiotic prophylaxis? (multiple answers possible) [none]
No

Which patients receive periinterventional antibiotic prophylaxis? (multiple answers possible) [Other]
oral systemic AB prophylaxis only exceptionally, regular procedure: local antibiotics (Gentamycin) mixed into VCUG contrast media

Are there situations or clinical findings in the patient where you deviate from your prophylaxis standard (medication, dose, duration, age of the patient)? (multiple answers possible, please specify in answer box) [in case of already known vesicoureteral reflux (VUR)]
No

Are there situations or clinical findings in the patient where you deviate from your prophylaxis standard (medication, dose, duration, age of the patient)? (multiple answers possible, please specify in answer box) [in case of already known vesicoureteral reflux (VUR)][Comment]

Are there situations or clinical findings in the patient where you deviate from your prophylaxis standard (medication, dose, duration, age of the patient)? (multiple answers possible, please specify in answer box) [in patients with recurrent urinary tract infections (UTI)]
No

Are there situations or clinical findings in the patient where you deviate from your prophylaxis standard (medication, dose, duration, age of the patient)? (multiple answers possible, please specify in answer box) [in patients with recurrent urinary tract infections (UTI)][Comment]

Are there situations or clinical findings in the patient where you deviate from your prophylaxis standard (medication, dose, duration, age of the patient)? (multiple answers possible, please specify in answer box) [if multidrug-resistant organisms (MDRO) are detected in urine cultures]
Yes

Are there situations or clinical findings in the patient where you deviate from your prophylaxis standard (medication, dose, duration, age of the patient)? (multiple answers possible, please specify in answer box) [if multidrug-resistant organisms (MDRO) are detected in urine cultures][Comment]
according to urine culture

Are there situations or clinical findings in the patient where you deviate from your prophylaxis standard (medication, dose, duration, age of the patient)? (multiple answers possible, please specify in answer box) [in newborns]
No

Are there situations or clinical findings in the patient where you deviate from your prophylaxis standard (medication, dose, duration, age of the patient)? (multiple answers possible, please specify in answer box) [in newborns][Comment]

Are there situations or clinical findings in the patient where you deviate from your prophylaxis standard (medication, dose, duration, age of the patient)? (multiple answers possible, please specify in answer box) [Other]

Are there situations or clinical findings in the patient where you deviate from your prophylaxis standard (medication, dose, duration, age of the patient)? (multiple answers possible, please specify in answer box) [Other comment]

Are there situations in which you use intravenous prophylaxis?
No

Voiding analysis

Are there differences in antibiotic prophylaxis between VUS and VCUG in your department?
No

Is a non-pathological urine strip test required in preparation for VUS/VCUG in your department?
Yes

What is the maximum amount of days accepted for urine strip test to be performed prior to the exam?
urine strip test during catheterisation procedure

Is a non-pathological urine culture required in preparation for VUS/VCUG in your department?
No

Do patients only receive post-procedure prophylaxis if vesicoureteral reflux (VUR) is detected in VUS/VCUG?
No

Do other MUS/MCU findings (e.g. urethral valves) have an influence on post-procedure prophylaxis?
Yes

Please specify:
according to pediatric surgery

End of survey

Thank you for taking the time to answer this survey!

May we contact you personally if we have further questions?
Yes

E-mail address:
gerald.paertan@gesundheitsverbund.at

Survey response 215

Response ID
217

Date submitted
2025-04-01 10:24:35

Last page
5

Start language
en

Seed
940519597

Date started
2025-04-01 10:05:55

Date last action
2025-04-01 10:24:35

Standard Operation Procedure

Who fills out this survey?
Pediatrician

Who fills out this survey? [Other]

In which type of hospital are you employed?
Community hospital (>50 beds for pediatric patients)

In which type of hospital are you employed? [Other]

In which country do you work?
Austria, Vienna

Does your department have a defined protocol for infection prophylaxis in the context of VUS/VCUG?
Yes

Has this protocol been transformed into a written standard operating procedure (SOP)?
No

Antibiotic Prophylaxis

Are there situations/constellations in which you do not use antibiotic prophylaxis for VUS/VCUG?
No

Please specify these situations/constellations: [girl with first uncomplicated UTI without urinary tract dilation in ultrasound]
N/A

Please specify these situations/constellations: [boy with first uncomplicated UTI without urinary tract dilation in ultrasound]
N/A

Please specify these situations/constellations: [in patients with low grade urinary tract dilation (up to 2nd grade) and no UTI in medical history]
N/A

If you use antibiotic prophylaxis: which mediaction do you use? (multiple answers and subdivision into age groups possible) [Trimethoprim]
Yes

If you use antibiotic prophylaxis: which mediaction do you use? (multiple answers and subdivision into age groups possible) [Nitrofurantoin]
No

If you use antibiotic prophylaxis: which mediaction do you use? (multiple answers and subdivision into age groups possible) [Amoxicillin]
No

If you use antibiotic prophylaxis: which mediaction do you use? (multiple answers and subdivision into age groups possible) [Cefaclor]
No

If you use antibiotic prophylaxis: which mediaction do you use? (multiple answers and subdivision into age groups possible) [Amoxicillin/clavulanic acid]
Yes

If you use antibiotic prophylaxis: which mediaction do you use? (multiple answers and subdivision into age groups possible) [Other]
Yes

Trimethoprim: in what dosage?
prophylactic (2 mg/kg bodyweight/d)

Trimethoprim: in what dosage? [Other]

Trimethoprim: for which age group do you use trimethoprim?
0-18a

Trimethoprim: which time schedule do you use? [Day(s) before VUS/VCUG, how many?]
Yes

Trimethoprim: which time schedule do you use? [Day(s) before VUS/VCUG, how many?][Comment]
1

Trimethoprim: which time schedule do you use? [Day of VUS/VCUG]
Yes

Trimethoprim: which time schedule do you use? [Day of VUS/VCUG][Comment]

Trimethoprim: which time schedule do you use? [Day(s) after VUS/VCUG, how many?]
Yes

Trimethoprim: which time schedule do you use? [Day(s) after VUS/VCUG, how many?][Comment]
2-3

Nitrofurantoin: which time schedule do you use? [Day(s) before VUS/VCUG, how many?]
N/A

Nitrofurantoin: which time schedule do you use? [Day of VUS/VCUG]
N/A

Nitrofurantoin: which time schedule do you use? [Day(s) after VUS/VCUG, how many?]
N/A

Amoxicillin: which time schedule do you use? [Day(s) before VUS/VCUG, how many?]
N/A

Amoxicillin: which time schedule do you use? [Day of VUS/VCUG]
N/A

Amoxicillin: which time schedule do you use? [Day(s) after VUS/VCUG, how many?]
N/A

Cefaclor: which time schedule do you use? [Days(s) before VUS/VCUG, how many?]
N/A

Cefaclor: which time schedule do you use? [Day of VUS/VCUG]
N/A

Cefaclor: which time schedule do you use? [Day(s) after VUS/VCUG, how many?]
N/A

Amoxicillin/clavulanic acid: in what dosage? (Amoxicillin portion) [prophylaktic (10-20 mg/Kg bodyweight/d)]
No

Amoxicillin/clavulanic acid: in what dosage? (Amoxicillin portion) [double prophylactic (20-40 mg/Kg bodyweight/d)]
Yes

Amoxicillin/clavulanic acid: in what dosage? (Amoxicillin portion) [therapeutic (80-100 mg/Kg bodyweight/d)]
No

Amoxicillin/clavulanic acid: in what dosage? (Amoxicillin portion) [Other]

Amoxicillin/clavulanic acid: for which age group do you use amoxicillin/clavulanic acid?
0-18a

Amoxicillin/clavulanic acid: which time schedule do you use? [Day(s) before VUS/VCUG]
Yes

Amoxicillin/clavulanic acid: which time schedule do you use? [Day(s) before VUS/VCUG][Comment]
1

Amoxicillin/clavulanic acid: which time schedule do you use? [Day of VUS/VCUG]
Yes

Amoxicillin/clavulanic acid: which time schedule do you use? [Day of VUS/VCUG][Comment]

Amoxicillin/clavulanic acid: which time schedule do you use? [Day(s) after VUS/VCUG]
Yes

Amoxicillin/clavulanic acid: which time schedule do you use? [Day(s) after VUS/VCUG][Comment]
2-3

Other: which medication and in what dosage?
accoring to former urin culture - about 30-40% to normal tnerapeutic dosage

Other: for which age group do you use this medication?
0-18a

Other: which time schedule do you use? [Day(s) before VUS/VCUG, how many?]
Yes

Other: which time schedule do you use? [Day(s) before VUS/VCUG, how many?][Comment]
1

Other: which time schedule do you use? [Day of VUS/VCUG]
Yes

Other: which time schedule do you use? [Day of VUS/VCUG][Comment]

Other: which time schedule do you use? [Day(s) after VUS/VCUG, how many?]
Yes

Other: which time schedule do you use? [Day(s) after VUS/VCUG, how many?][Comment]
2-3

Indication for prophylaxis

Which patients receive periinterventional antibiotic prophylaxis? (multiple answers possible) [all]
No

Which patients receive periinterventional antibiotic prophylaxis? (multiple answers possible) [patients post urinary tract infection/pyelonephritis/urosepsis]
No

Which patients receive periinterventional antibiotic prophylaxis? (multiple answers possible) [patients with urinary tract dilation in ultrasound]
No

Which patients receive periinterventional antibiotic prophylaxis? (multiple answers possible) [patients with prenatal urinary tract dilation]
No

Which patients receive periinterventional antibiotic prophylaxis? (multiple answers possible) [patients post VUR surgery]
No

Which patients receive periinterventional antibiotic prophylaxis? (multiple answers possible) [none]
No

Which patients receive periinterventional antibiotic prophylaxis? (multiple answers possible) [Other]
oral (see former sheet) or local single-shot during examination (combined with the contrastmedia)

Are there situations or clinical findings in the patient where you deviate from your prophylaxis standard (medication, dose, duration, age of the patient)? (multiple answers possible, please specify in answer box) [in case of already known vesicoureteral reflux (VUR)]
No

Are there situations or clinical findings in the patient where you deviate from your prophylaxis standard (medication, dose, duration, age of the patient)? (multiple answers possible, please specify in answer box) [in case of already known vesicoureteral reflux (VUR)][Comment]

Are there situations or clinical findings in the patient where you deviate from your prophylaxis standard (medication, dose, duration, age of the patient)? (multiple answers possible, please specify in answer box) [in patients with recurrent urinary tract infections (UTI)]
No

Are there situations or clinical findings in the patient where you deviate from your prophylaxis standard (medication, dose, duration, age of the patient)? (multiple answers possible, please specify in answer box) [in patients with recurrent urinary tract infections (UTI)][Comment]

Are there situations or clinical findings in the patient where you deviate from your prophylaxis standard (medication, dose, duration, age of the patient)? (multiple answers possible, please specify in answer box) [if multidrug-resistant organisms (MDRO) are detected in urine cultures]
Yes

Are there situations or clinical findings in the patient where you deviate from your prophylaxis standard (medication, dose, duration, age of the patient)? (multiple answers possible, please specify in answer box) [if multidrug-resistant organisms (MDRO) are detected in urine cultures][Comment]
antibiotic prophylaxis according to former urine culture

Are there situations or clinical findings in the patient where you deviate from your prophylaxis standard (medication, dose, duration, age of the patient)? (multiple answers possible, please specify in answer box) [in newborns]
No

Are there situations or clinical findings in the patient where you deviate from your prophylaxis standard (medication, dose, duration, age of the patient)? (multiple answers possible, please specify in answer box) [in newborns][Comment]

Are there situations or clinical findings in the patient where you deviate from your prophylaxis standard (medication, dose, duration, age of the patient)? (multiple answers possible, please specify in answer box) [Other]
lokal antibiotics preferred to oral ones

Are there situations or clinical findings in the patient where you deviate from your prophylaxis standard (medication, dose, duration, age of the patient)? (multiple answers possible, please specify in answer box) [Other comment]
lokal antibiotics preferred to oral ones : ingle-shot versus 4-5 Days oral

Are there situations in which you use intravenous prophylaxis?
No

Voiding analysis

Are there differences in antibiotic prophylaxis between VUS and VCUG in your department?
No

Is a non-pathological urine strip test required in preparation for VUS/VCUG in your department?
Yes

What is the maximum amount of days accepted for urine strip test to be performed prior to the exam?
we test during catheterprocedure

Is a non-pathological urine culture required in preparation for VUS/VCUG in your department?
No

Do patients only receive post-procedure prophylaxis if vesicoureteral reflux (VUR) is detected in VUS/VCUG?
No

Do other MUS/MCU findings (e.g. urethral valves) have an influence on post-procedure prophylaxis?
Yes

Please specify:
according to pädiatric surgery

End of survey

Thank you for taking the time to answer this survey!

May we contact you personally if we have further questions?
No

Survey response 216

Response ID
218

Date submitted
2025-04-01 10:12:38

Last page
5

Start language
de

Seed
1219355894

Date started
2025-04-01 10:09:59

Date last action
2025-04-01 10:12:38

Standard Operation Procedure

Who fills out this survey?
Pediatric Surgeon

Who fills out this survey? [Other]

In which type of hospital are you employed?
Community hospital (>50 beds for pediatric patients)

In which type of hospital are you employed? [Other]

In which country do you work?
Deutschland

Does your department have a defined protocol for infection prophylaxis in the context of VUS/VCUG?
Yes

Has this protocol been transformed into a written standard operating procedure (SOP)?
Yes

Which department is the author of the SOP for infection prophylaxis in VUS/VCUG?
Pediatric radiology

Which department is the author of the SOP for infection prophylaxis in VUS/VCUG? [Other]

Antibiotic Prophylaxis

Are there situations/constellations in which you do not use antibiotic prophylaxis for VUS/VCUG?
No

Please specify these situations/constellations: [girl with first uncomplicated UTI without urinary tract dilation in ultrasound]
N/A

Please specify these situations/constellations: [boy with first uncomplicated UTI without urinary tract dilation in ultrasound]
N/A

Please specify these situations/constellations: [in patients with low grade urinary tract dilation (up to 2nd grade) and no UTI in medical history]
N/A

If you use antibiotic prophylaxis: which mediaction do you use? (multiple answers and subdivision into age groups possible) [Trimethoprim]
Yes

If you use antibiotic prophylaxis: which mediaction do you use? (multiple answers and subdivision into age groups possible) [Nitrofurantoin]
No

If you use antibiotic prophylaxis: which mediaction do you use? (multiple answers and subdivision into age groups possible) [Amoxicillin]
No

If you use antibiotic prophylaxis: which mediaction do you use? (multiple answers and subdivision into age groups possible) [Cefaclor]
No

If you use antibiotic prophylaxis: which mediaction do you use? (multiple answers and subdivision into age groups possible) [Amoxicillin/clavulanic acid]
No

If you use antibiotic prophylaxis: which mediaction do you use? (multiple answers and subdivision into age groups possible) [Other]
No

Trimethoprim: in what dosage?
double prophylakctic (4 mg/kg bodyweight/d)

Trimethoprim: in what dosage? [Other]

Trimethoprim: for which age group do you use trimethoprim?
0-6

Trimethoprim: which time schedule do you use? [Day(s) before VUS/VCUG, how many?]
No

Trimethoprim: which time schedule do you use? [Day(s) before VUS/VCUG, how many?][Comment]

Trimethoprim: which time schedule do you use? [Day of VUS/VCUG]
Yes

Trimethoprim: which time schedule do you use? [Day of VUS/VCUG][Comment]

Trimethoprim: which time schedule do you use? [Day(s) after VUS/VCUG, how many?]
Yes

Trimethoprim: which time schedule do you use? [Day(s) after VUS/VCUG, how many?][Comment]
2

Nitrofurantoin: which time schedule do you use? [Day(s) before VUS/VCUG, how many?]
N/A

Nitrofurantoin: which time schedule do you use? [Day of VUS/VCUG]
N/A

Nitrofurantoin: which time schedule do you use? [Day(s) after VUS/VCUG, how many?]
N/A

Amoxicillin: which time schedule do you use? [Day(s) before VUS/VCUG, how many?]
N/A

Amoxicillin: which time schedule do you use? [Day of VUS/VCUG]
N/A

Amoxicillin: which time schedule do you use? [Day(s) after VUS/VCUG, how many?]
N/A

Cefaclor: which time schedule do you use? [Days(s) before VUS/VCUG, how many?]
N/A

Cefaclor: which time schedule do you use? [Day of VUS/VCUG]
N/A

Cefaclor: which time schedule do you use? [Day(s) after VUS/VCUG, how many?]
N/A

Amoxicillin/clavulanic acid: in what dosage? (Amoxicillin portion) [prophylaktic (10-20 mg/Kg bodyweight/d)]
N/A

Amoxicillin/clavulanic acid: in what dosage? (Amoxicillin portion) [double prophylactic (20-40 mg/Kg bodyweight/d)]
N/A

Amoxicillin/clavulanic acid: in what dosage? (Amoxicillin portion) [therapeutic (80-100 mg/Kg bodyweight/d)]
N/A

Amoxicillin/clavulanic acid: which time schedule do you use? [Day(s) before VUS/VCUG]
N/A

Amoxicillin/clavulanic acid: which time schedule do you use? [Day of VUS/VCUG]
N/A

Amoxicillin/clavulanic acid: which time schedule do you use? [Day(s) after VUS/VCUG]
N/A

Other: which time schedule do you use? [Day(s) before VUS/VCUG, how many?]
N/A

Other: which time schedule do you use? [Day of VUS/VCUG]
N/A

Other: which time schedule do you use? [Day(s) after VUS/VCUG, how many?]
N/A

Indication for prophylaxis

Which patients receive periinterventional antibiotic prophylaxis? (multiple answers possible) [all]
Yes

Which patients receive periinterventional antibiotic prophylaxis? (multiple answers possible) [patients post urinary tract infection/pyelonephritis/urosepsis]
N/A

Which patients receive periinterventional antibiotic prophylaxis? (multiple answers possible) [patients with urinary tract dilation in ultrasound]
N/A

Which patients receive periinterventional antibiotic prophylaxis? (multiple answers possible) [patients with prenatal urinary tract dilation]
N/A

Which patients receive periinterventional antibiotic prophylaxis? (multiple answers possible) [patients post VUR surgery]
N/A

Which patients receive periinterventional antibiotic prophylaxis? (multiple answers possible) [none]
N/A

Are there situations or clinical findings in the patient where you deviate from your prophylaxis standard (medication, dose, duration, age of the patient)? (multiple answers possible, please specify in answer box) [in case of already known vesicoureteral reflux (VUR)]
No

Are there situations or clinical findings in the patient where you deviate from your prophylaxis standard (medication, dose, duration, age of the patient)? (multiple answers possible, please specify in answer box) [in case of already known vesicoureteral reflux (VUR)][Comment]

Are there situations or clinical findings in the patient where you deviate from your prophylaxis standard (medication, dose, duration, age of the patient)? (multiple answers possible, please specify in answer box) [in patients with recurrent urinary tract infections (UTI)]
Yes

Are there situations or clinical findings in the patient where you deviate from your prophylaxis standard (medication, dose, duration, age of the patient)? (multiple answers possible, please specify in answer box) [in patients with recurrent urinary tract infections (UTI)][Comment]

Are there situations or clinical findings in the patient where you deviate from your prophylaxis standard (medication, dose, duration, age of the patient)? (multiple answers possible, please specify in answer box) [if multidrug-resistant organisms (MDRO) are detected in urine cultures]
No

Are there situations or clinical findings in the patient where you deviate from your prophylaxis standard (medication, dose, duration, age of the patient)? (multiple answers possible, please specify in answer box) [if multidrug-resistant organisms (MDRO) are detected in urine cultures][Comment]

Are there situations or clinical findings in the patient where you deviate from your prophylaxis standard (medication, dose, duration, age of the patient)? (multiple answers possible, please specify in answer box) [in newborns]
No

Are there situations or clinical findings in the patient where you deviate from your prophylaxis standard (medication, dose, duration, age of the patient)? (multiple answers possible, please specify in answer box) [in newborns][Comment]

Are there situations or clinical findings in the patient where you deviate from your prophylaxis standard (medication, dose, duration, age of the patient)? (multiple answers possible, please specify in answer box) [Other]

Are there situations or clinical findings in the patient where you deviate from your prophylaxis standard (medication, dose, duration, age of the patient)? (multiple answers possible, please specify in answer box) [Other comment]

Are there situations in which you use intravenous prophylaxis?
No

Voiding analysis

Are there differences in antibiotic prophylaxis between VUS and VCUG in your department?
No

Is a non-pathological urine strip test required in preparation for VUS/VCUG in your department?
No

Is a non-pathological urine culture required in preparation for VUS/VCUG in your department?
No

Do patients only receive post-procedure prophylaxis if vesicoureteral reflux (VUR) is detected in VUS/VCUG?
No

Do other MUS/MCU findings (e.g. urethral valves) have an influence on post-procedure prophylaxis?
No

End of survey

Thank you for taking the time to answer this survey!

May we contact you personally if we have further questions?
No

Survey response 217

Response ID
219

Date submitted
2025-04-01 13:09:11

Last page
5

Start language
en

Seed
1015790047

Date started
2025-04-01 13:02:07

Date last action
2025-04-01 13:09:11

Standard Operation Procedure

Who fills out this survey?
Pediatric Radiologist

Who fills out this survey? [Other]

In which type of hospital are you employed?
University Hospital

In which type of hospital are you employed? [Other]

In which country do you work?
France

Does your department have a defined protocol for infection prophylaxis in the context of VUS/VCUG?
Yes

Has this protocol been transformed into a written standard operating procedure (SOP)?
Yes

Which department is the author of the SOP for infection prophylaxis in VUS/VCUG?
Pediatric surgery

Which department is the author of the SOP for infection prophylaxis in VUS/VCUG? [Other]

Antibiotic Prophylaxis

Are there situations/constellations in which you do not use antibiotic prophylaxis for VUS/VCUG?
Yes

Please specify these situations/constellations: [girl with first uncomplicated UTI without urinary tract dilation in ultrasound]
Yes

Please specify these situations/constellations: [boy with first uncomplicated UTI without urinary tract dilation in ultrasound]
Yes

Please specify these situations/constellations: [in patients with low grade urinary tract dilation (up to 2nd grade) and no UTI in medical history]
Yes

Please specify these situations/constellations: [Other]
we never use antibiotic prophylaxis for VUS/VCUG

If you use antibiotic prophylaxis: which mediaction do you use? (multiple answers and subdivision into age groups possible) [Trimethoprim]
No

If you use antibiotic prophylaxis: which mediaction do you use? (multiple answers and subdivision into age groups possible) [Nitrofurantoin]
No

If you use antibiotic prophylaxis: which mediaction do you use? (multiple answers and subdivision into age groups possible) [Amoxicillin]
No

If you use antibiotic prophylaxis: which mediaction do you use? (multiple answers and subdivision into age groups possible) [Cefaclor]
No

If you use antibiotic prophylaxis: which mediaction do you use? (multiple answers and subdivision into age groups possible) [Amoxicillin/clavulanic acid]
No

If you use antibiotic prophylaxis: which mediaction do you use? (multiple answers and subdivision into age groups possible) [Other]
No

Trimethoprim: which time schedule do you use? [Day(s) before VUS/VCUG, how many?]
N/A

Trimethoprim: which time schedule do you use? [Day of VUS/VCUG]
N/A

Trimethoprim: which time schedule do you use? [Day(s) after VUS/VCUG, how many?]
N/A

Nitrofurantoin: which time schedule do you use? [Day(s) before VUS/VCUG, how many?]
N/A

Nitrofurantoin: which time schedule do you use? [Day of VUS/VCUG]
N/A

Nitrofurantoin: which time schedule do you use? [Day(s) after VUS/VCUG, how many?]
N/A

Amoxicillin: which time schedule do you use? [Day(s) before VUS/VCUG, how many?]
N/A

Amoxicillin: which time schedule do you use? [Day of VUS/VCUG]
N/A

Amoxicillin: which time schedule do you use? [Day(s) after VUS/VCUG, how many?]
N/A

Cefaclor: which time schedule do you use? [Days(s) before VUS/VCUG, how many?]
N/A

Cefaclor: which time schedule do you use? [Day of VUS/VCUG]
N/A

Cefaclor: which time schedule do you use? [Day(s) after VUS/VCUG, how many?]
N/A

Amoxicillin/clavulanic acid: in what dosage? (Amoxicillin portion) [prophylaktic (10-20 mg/Kg bodyweight/d)]
N/A

Amoxicillin/clavulanic acid: in what dosage? (Amoxicillin portion) [double prophylactic (20-40 mg/Kg bodyweight/d)]
N/A

Amoxicillin/clavulanic acid: in what dosage? (Amoxicillin portion) [therapeutic (80-100 mg/Kg bodyweight/d)]
N/A

Amoxicillin/clavulanic acid: which time schedule do you use? [Day(s) before VUS/VCUG]
N/A

Amoxicillin/clavulanic acid: which time schedule do you use? [Day of VUS/VCUG]
N/A

Amoxicillin/clavulanic acid: which time schedule do you use? [Day(s) after VUS/VCUG]
N/A

Other: which time schedule do you use? [Day(s) before VUS/VCUG, how many?]
N/A

Other: which time schedule do you use? [Day of VUS/VCUG]
N/A

Other: which time schedule do you use? [Day(s) after VUS/VCUG, how many?]
N/A

Indication for prophylaxis

Which patients receive periinterventional antibiotic prophylaxis? (multiple answers possible) [all]
No

Which patients receive periinterventional antibiotic prophylaxis? (multiple answers possible) [patients post urinary tract infection/pyelonephritis/urosepsis]
No

Which patients receive periinterventional antibiotic prophylaxis? (multiple answers possible) [patients with urinary tract dilation in ultrasound]
No

Which patients receive periinterventional antibiotic prophylaxis? (multiple answers possible) [patients with prenatal urinary tract dilation]
No

Which patients receive periinterventional antibiotic prophylaxis? (multiple answers possible) [patients post VUR surgery]
No

Which patients receive periinterventional antibiotic prophylaxis? (multiple answers possible) [none]
Yes

Which patients receive periinterventional antibiotic prophylaxis? (multiple answers possible) [Other]

Are there situations or clinical findings in the patient where you deviate from your prophylaxis standard (medication, dose, duration, age of the patient)? (multiple answers possible, please specify in answer box) [in case of already known vesicoureteral reflux (VUR)]
No

Are there situations or clinical findings in the patient where you deviate from your prophylaxis standard (medication, dose, duration, age of the patient)? (multiple answers possible, please specify in answer box) [in case of already known vesicoureteral reflux (VUR)][Comment]

Are there situations or clinical findings in the patient where you deviate from your prophylaxis standard (medication, dose, duration, age of the patient)? (multiple answers possible, please specify in answer box) [in patients with recurrent urinary tract infections (UTI)]
No

Are there situations or clinical findings in the patient where you deviate from your prophylaxis standard (medication, dose, duration, age of the patient)? (multiple answers possible, please specify in answer box) [in patients with recurrent urinary tract infections (UTI)][Comment]

Are there situations or clinical findings in the patient where you deviate from your prophylaxis standard (medication, dose, duration, age of the patient)? (multiple answers possible, please specify in answer box) [if multidrug-resistant organisms (MDRO) are detected in urine cultures]
No

Are there situations or clinical findings in the patient where you deviate from your prophylaxis standard (medication, dose, duration, age of the patient)? (multiple answers possible, please specify in answer box) [if multidrug-resistant organisms (MDRO) are detected in urine cultures][Comment]

Are there situations or clinical findings in the patient where you deviate from your prophylaxis standard (medication, dose, duration, age of the patient)? (multiple answers possible, please specify in answer box) [in newborns]
No

Are there situations or clinical findings in the patient where you deviate from your prophylaxis standard (medication, dose, duration, age of the patient)? (multiple answers possible, please specify in answer box) [in newborns][Comment]

Are there situations or clinical findings in the patient where you deviate from your prophylaxis standard (medication, dose, duration, age of the patient)? (multiple answers possible, please specify in answer box) [Other]
none

Are there situations or clinical findings in the patient where you deviate from your prophylaxis standard (medication, dose, duration, age of the patient)? (multiple answers possible, please specify in answer box) [Other comment]

Are there situations in which you use intravenous prophylaxis?
No

Voiding analysis

Are there differences in antibiotic prophylaxis between VUS and VCUG in your department?
No

Is a non-pathological urine strip test required in preparation for VUS/VCUG in your department?
No

Is a non-pathological urine culture required in preparation for VUS/VCUG in your department?
No

Do patients only receive post-procedure prophylaxis if vesicoureteral reflux (VUR) is detected in VUS/VCUG?
No

Do other MUS/MCU findings (e.g. urethral valves) have an influence on post-procedure prophylaxis?
No

End of survey

Thank you for taking the time to answer this survey!

May we contact you personally if we have further questions?
Yes

E-mail address:
marianne.alison@aphp.fr

Survey response 218

Response ID
220

Last page
2

Start language
de

Seed
1608688730

Date started
2025-04-01 19:05:54

Date last action
2025-04-01 19:07:51

Standard Operation Procedure

Who fills out this survey?
Pediatric Surgeon

Who fills out this survey? [Other]

In which type of hospital are you employed?
Other

In which type of hospital are you employed? [Other]
Stiftung

In which country do you work?
Niedersachsen

Does your department have a defined protocol for infection prophylaxis in the context of VUS/VCUG?
No

Has this protocol been transformed into a written standard operating procedure (SOP)?
N/A

Antibiotic Prophylaxis

Are there situations/constellations in which you do not use antibiotic prophylaxis for VUS/VCUG?
Yes

Please specify these situations/constellations: [girl with first uncomplicated UTI without urinary tract dilation in ultrasound]
Yes

Please specify these situations/constellations: [boy with first uncomplicated UTI without urinary tract dilation in ultrasound]
Yes

Please specify these situations/constellations: [in patients with low grade urinary tract dilation (up to 2nd grade) and no UTI in medical history]
Yes

Please specify these situations/constellations: [Other]

If you use antibiotic prophylaxis: which mediaction do you use? (multiple answers and subdivision into age groups possible) [Trimethoprim]
Yes

If you use antibiotic prophylaxis: which mediaction do you use? (multiple answers and subdivision into age groups possible) [Nitrofurantoin]
No

If you use antibiotic prophylaxis: which mediaction do you use? (multiple answers and subdivision into age groups possible) [Amoxicillin]
No

If you use antibiotic prophylaxis: which mediaction do you use? (multiple answers and subdivision into age groups possible) [Cefaclor]
No

If you use antibiotic prophylaxis: which mediaction do you use? (multiple answers and subdivision into age groups possible) [Amoxicillin/clavulanic acid]
No

If you use antibiotic prophylaxis: which mediaction do you use? (multiple answers and subdivision into age groups possible) [Other]
No

Trimethoprim: in what dosage?
therapeutic (6 mg/kg bodyweight/d)

Trimethoprim: in what dosage? [Other]

Trimethoprim: for which age group do you use trimethoprim?
Bis 6

Trimethoprim: which time schedule do you use? [Day(s) before VUS/VCUG, how many?]
No

Trimethoprim: which time schedule do you use? [Day(s) before VUS/VCUG, how many?][Comment]

Trimethoprim: which time schedule do you use? [Day of VUS/VCUG]
Yes

Trimethoprim: which time schedule do you use? [Day of VUS/VCUG][Comment]

Trimethoprim: which time schedule do you use? [Day(s) after VUS/VCUG, how many?]
No

Trimethoprim: which time schedule do you use? [Day(s) after VUS/VCUG, how many?][Comment]

Nitrofurantoin: which time schedule do you use? [Day(s) before VUS/VCUG, how many?]
N/A

Nitrofurantoin: which time schedule do you use? [Day of VUS/VCUG]
N/A

Nitrofurantoin: which time schedule do you use? [Day(s) after VUS/VCUG, how many?]
N/A

Amoxicillin: which time schedule do you use? [Day(s) before VUS/VCUG, how many?]
N/A

Amoxicillin: which time schedule do you use? [Day of VUS/VCUG]
N/A

Amoxicillin: which time schedule do you use? [Day(s) after VUS/VCUG, how many?]
N/A

Cefaclor: which time schedule do you use? [Days(s) before VUS/VCUG, how many?]
N/A

Cefaclor: which time schedule do you use? [Day of VUS/VCUG]
N/A

Cefaclor: which time schedule do you use? [Day(s) after VUS/VCUG, how many?]
N/A

Amoxicillin/clavulanic acid: in what dosage? (Amoxicillin portion) [prophylaktic (10-20 mg/Kg bodyweight/d)]
N/A

Amoxicillin/clavulanic acid: in what dosage? (Amoxicillin portion) [double prophylactic (20-40 mg/Kg bodyweight/d)]
N/A

Amoxicillin/clavulanic acid: in what dosage? (Amoxicillin portion) [therapeutic (80-100 mg/Kg bodyweight/d)]
N/A

Amoxicillin/clavulanic acid: which time schedule do you use? [Day(s) before VUS/VCUG]
N/A

Amoxicillin/clavulanic acid: which time schedule do you use? [Day of VUS/VCUG]
N/A

Amoxicillin/clavulanic acid: which time schedule do you use? [Day(s) after VUS/VCUG]
N/A

Other: which time schedule do you use? [Day(s) before VUS/VCUG, how many?]
N/A

Other: which time schedule do you use? [Day of VUS/VCUG]
N/A

Other: which time schedule do you use? [Day(s) after VUS/VCUG, how many?]
N/A

Indication for prophylaxis

Which patients receive periinterventional antibiotic prophylaxis? (multiple answers possible) [all]
N/A

Which patients receive periinterventional antibiotic prophylaxis? (multiple answers possible) [patients post urinary tract infection/pyelonephritis/urosepsis]
N/A

Which patients receive periinterventional antibiotic prophylaxis? (multiple answers possible) [patients with urinary tract dilation in ultrasound]
N/A

Which patients receive periinterventional antibiotic prophylaxis? (multiple answers possible) [patients with prenatal urinary tract dilation]
N/A

Which patients receive periinterventional antibiotic prophylaxis? (multiple answers possible) [patients post VUR surgery]
N/A

Which patients receive periinterventional antibiotic prophylaxis? (multiple answers possible) [none]
N/A

Are there situations or clinical findings in the patient where you deviate from your prophylaxis standard (medication, dose, duration, age of the patient)? (multiple answers possible, please specify in answer box) [in case of already known vesicoureteral reflux (VUR)]
N/A

Are there situations or clinical findings in the patient where you deviate from your prophylaxis standard (medication, dose, duration, age of the patient)? (multiple answers possible, please specify in answer box) [in patients with recurrent urinary tract infections (UTI)]
N/A

Are there situations or clinical findings in the patient where you deviate from your prophylaxis standard (medication, dose, duration, age of the patient)? (multiple answers possible, please specify in answer box) [if multidrug-resistant organisms (MDRO) are detected in urine cultures]
N/A

Are there situations or clinical findings in the patient where you deviate from your prophylaxis standard (medication, dose, duration, age of the patient)? (multiple answers possible, please specify in answer box) [in newborns]
N/A

Are there situations in which you use intravenous prophylaxis?
N/A

Voiding analysis

Are there differences in antibiotic prophylaxis between VUS and VCUG in your department?
N/A

Is a non-pathological urine strip test required in preparation for VUS/VCUG in your department?
N/A

Is a non-pathological urine culture required in preparation for VUS/VCUG in your department?
N/A

Do patients only receive post-procedure prophylaxis if vesicoureteral reflux (VUR) is detected in VUS/VCUG?
N/A

Do other MUS/MCU findings (e.g. urethral valves) have an influence on post-procedure prophylaxis?
N/A

End of survey

May we contact you personally if we have further questions?
N/A

Survey response 219

Response ID
221

Last page
0

Start language
de

Seed
1078397474

Date started
2025-04-02 05:36:30

Date last action
2025-04-02 05:36:41

Standard Operation Procedure

Who fills out this survey?

Who fills out this survey? [Other]

In which type of hospital are you employed?

In which type of hospital are you employed? [Other]

In which country do you work?

Does your department have a defined protocol for infection prophylaxis in the context of VUS/VCUG?
N/A

Has this protocol been transformed into a written standard operating procedure (SOP)?
N/A

Antibiotic Prophylaxis

Are there situations/constellations in which you do not use antibiotic prophylaxis for VUS/VCUG?
N/A

Please specify these situations/constellations: [girl with first uncomplicated UTI without urinary tract dilation in ultrasound]
N/A

Please specify these situations/constellations: [boy with first uncomplicated UTI without urinary tract dilation in ultrasound]
N/A

Please specify these situations/constellations: [in patients with low grade urinary tract dilation (up to 2nd grade) and no UTI in medical history]
N/A

If you use antibiotic prophylaxis: which mediaction do you use? (multiple answers and subdivision into age groups possible) [Trimethoprim]
N/A

If you use antibiotic prophylaxis: which mediaction do you use? (multiple answers and subdivision into age groups possible) [Nitrofurantoin]
N/A

If you use antibiotic prophylaxis: which mediaction do you use? (multiple answers and subdivision into age groups possible) [Amoxicillin]
N/A

If you use antibiotic prophylaxis: which mediaction do you use? (multiple answers and subdivision into age groups possible) [Cefaclor]
N/A

If you use antibiotic prophylaxis: which mediaction do you use? (multiple answers and subdivision into age groups possible) [Amoxicillin/clavulanic acid]
N/A

If you use antibiotic prophylaxis: which mediaction do you use? (multiple answers and subdivision into age groups possible) [Other]
N/A

Trimethoprim: which time schedule do you use? [Day(s) before VUS/VCUG, how many?]
N/A

Trimethoprim: which time schedule do you use? [Day of VUS/VCUG]
N/A

Trimethoprim: which time schedule do you use? [Day(s) after VUS/VCUG, how many?]
N/A

Nitrofurantoin: which time schedule do you use? [Day(s) before VUS/VCUG, how many?]
N/A

Nitrofurantoin: which time schedule do you use? [Day of VUS/VCUG]
N/A

Nitrofurantoin: which time schedule do you use? [Day(s) after VUS/VCUG, how many?]
N/A

Amoxicillin: which time schedule do you use? [Day(s) before VUS/VCUG, how many?]
N/A

Amoxicillin: which time schedule do you use? [Day of VUS/VCUG]
N/A

Amoxicillin: which time schedule do you use? [Day(s) after VUS/VCUG, how many?]
N/A

Cefaclor: which time schedule do you use? [Days(s) before VUS/VCUG, how many?]
N/A

Cefaclor: which time schedule do you use? [Day of VUS/VCUG]
N/A

Cefaclor: which time schedule do you use? [Day(s) after VUS/VCUG, how many?]
N/A

Amoxicillin/clavulanic acid: in what dosage? (Amoxicillin portion) [prophylaktic (10-20 mg/Kg bodyweight/d)]
N/A

Amoxicillin/clavulanic acid: in what dosage? (Amoxicillin portion) [double prophylactic (20-40 mg/Kg bodyweight/d)]
N/A

Amoxicillin/clavulanic acid: in what dosage? (Amoxicillin portion) [therapeutic (80-100 mg/Kg bodyweight/d)]
N/A

Amoxicillin/clavulanic acid: which time schedule do you use? [Day(s) before VUS/VCUG]
N/A

Amoxicillin/clavulanic acid: which time schedule do you use? [Day of VUS/VCUG]
N/A

Amoxicillin/clavulanic acid: which time schedule do you use? [Day(s) after VUS/VCUG]
N/A

Other: which time schedule do you use? [Day(s) before VUS/VCUG, how many?]
N/A

Other: which time schedule do you use? [Day of VUS/VCUG]
N/A

Other: which time schedule do you use? [Day(s) after VUS/VCUG, how many?]
N/A

Indication for prophylaxis

Which patients receive periinterventional antibiotic prophylaxis? (multiple answers possible) [all]
N/A

Which patients receive periinterventional antibiotic prophylaxis? (multiple answers possible) [patients post urinary tract infection/pyelonephritis/urosepsis]
N/A

Which patients receive periinterventional antibiotic prophylaxis? (multiple answers possible) [patients with urinary tract dilation in ultrasound]
N/A

Which patients receive periinterventional antibiotic prophylaxis? (multiple answers possible) [patients with prenatal urinary tract dilation]
N/A

Which patients receive periinterventional antibiotic prophylaxis? (multiple answers possible) [patients post VUR surgery]
N/A

Which patients receive periinterventional antibiotic prophylaxis? (multiple answers possible) [none]
N/A

Are there situations or clinical findings in the patient where you deviate from your prophylaxis standard (medication, dose, duration, age of the patient)? (multiple answers possible, please specify in answer box) [in case of already known vesicoureteral reflux (VUR)]
N/A

Are there situations or clinical findings in the patient where you deviate from your prophylaxis standard (medication, dose, duration, age of the patient)? (multiple answers possible, please specify in answer box) [in patients with recurrent urinary tract infections (UTI)]
N/A

Are there situations or clinical findings in the patient where you deviate from your prophylaxis standard (medication, dose, duration, age of the patient)? (multiple answers possible, please specify in answer box) [if multidrug-resistant organisms (MDRO) are detected in urine cultures]
N/A

Are there situations or clinical findings in the patient where you deviate from your prophylaxis standard (medication, dose, duration, age of the patient)? (multiple answers possible, please specify in answer box) [in newborns]
N/A

Are there situations in which you use intravenous prophylaxis?
N/A

Voiding analysis

Are there differences in antibiotic prophylaxis between VUS and VCUG in your department?
N/A

Is a non-pathological urine strip test required in preparation for VUS/VCUG in your department?
N/A

Is a non-pathological urine culture required in preparation for VUS/VCUG in your department?
N/A

Do patients only receive post-procedure prophylaxis if vesicoureteral reflux (VUR) is detected in VUS/VCUG?
N/A

Do other MUS/MCU findings (e.g. urethral valves) have an influence on post-procedure prophylaxis?
N/A

End of survey

May we contact you personally if we have further questions?
N/A

Survey response 220

Response ID
222

Date submitted
2025-04-02 07:12:22

Last page
5

Start language
de

Seed
1701798344

Date started
2025-04-02 07:07:47

Date last action
2025-04-02 07:12:22

Standard Operation Procedure

Who fills out this survey?
Other

Who fills out this survey? [Other]
Kinderchirurgin

In which type of hospital are you employed?
University Hospital

In which type of hospital are you employed? [Other]

In which country do you work?
Deutschland

Does your department have a defined protocol for infection prophylaxis in the context of VUS/VCUG?
Yes

Has this protocol been transformed into a written standard operating procedure (SOP)?
Yes

Which department is the author of the SOP for infection prophylaxis in VUS/VCUG?
Pediatric surgery

Which department is the author of the SOP for infection prophylaxis in VUS/VCUG? [Other]

Antibiotic Prophylaxis

Are there situations/constellations in which you do not use antibiotic prophylaxis for VUS/VCUG?
No

Please specify these situations/constellations: [girl with first uncomplicated UTI without urinary tract dilation in ultrasound]
N/A

Please specify these situations/constellations: [boy with first uncomplicated UTI without urinary tract dilation in ultrasound]
N/A

Please specify these situations/constellations: [in patients with low grade urinary tract dilation (up to 2nd grade) and no UTI in medical history]
N/A

If you use antibiotic prophylaxis: which mediaction do you use? (multiple answers and subdivision into age groups possible) [Trimethoprim]
Yes

If you use antibiotic prophylaxis: which mediaction do you use? (multiple answers and subdivision into age groups possible) [Nitrofurantoin]
No

If you use antibiotic prophylaxis: which mediaction do you use? (multiple answers and subdivision into age groups possible) [Amoxicillin]
No

If you use antibiotic prophylaxis: which mediaction do you use? (multiple answers and subdivision into age groups possible) [Cefaclor]
No

If you use antibiotic prophylaxis: which mediaction do you use? (multiple answers and subdivision into age groups possible) [Amoxicillin/clavulanic acid]
No

If you use antibiotic prophylaxis: which mediaction do you use? (multiple answers and subdivision into age groups possible) [Other]
No

Trimethoprim: in what dosage?
therapeutic (6 mg/kg bodyweight/d)

Trimethoprim: in what dosage? [Other]

Trimethoprim: for which age group do you use trimethoprim?
alle, besonders Säuglinge

Trimethoprim: which time schedule do you use? [Day(s) before VUS/VCUG, how many?]
No

Trimethoprim: which time schedule do you use? [Day(s) before VUS/VCUG, how many?][Comment]

Trimethoprim: which time schedule do you use? [Day of VUS/VCUG]
Yes

Trimethoprim: which time schedule do you use? [Day of VUS/VCUG][Comment]

Trimethoprim: which time schedule do you use? [Day(s) after VUS/VCUG, how many?]
Yes

Trimethoprim: which time schedule do you use? [Day(s) after VUS/VCUG, how many?][Comment]
2

Nitrofurantoin: which time schedule do you use? [Day(s) before VUS/VCUG, how many?]
N/A

Nitrofurantoin: which time schedule do you use? [Day of VUS/VCUG]
N/A

Nitrofurantoin: which time schedule do you use? [Day(s) after VUS/VCUG, how many?]
N/A

Amoxicillin: which time schedule do you use? [Day(s) before VUS/VCUG, how many?]
N/A

Amoxicillin: which time schedule do you use? [Day of VUS/VCUG]
N/A

Amoxicillin: which time schedule do you use? [Day(s) after VUS/VCUG, how many?]
N/A

Cefaclor: which time schedule do you use? [Days(s) before VUS/VCUG, how many?]
N/A

Cefaclor: which time schedule do you use? [Day of VUS/VCUG]
N/A

Cefaclor: which time schedule do you use? [Day(s) after VUS/VCUG, how many?]
N/A

Amoxicillin/clavulanic acid: in what dosage? (Amoxicillin portion) [prophylaktic (10-20 mg/Kg bodyweight/d)]
N/A

Amoxicillin/clavulanic acid: in what dosage? (Amoxicillin portion) [double prophylactic (20-40 mg/Kg bodyweight/d)]
N/A

Amoxicillin/clavulanic acid: in what dosage? (Amoxicillin portion) [therapeutic (80-100 mg/Kg bodyweight/d)]
N/A

Amoxicillin/clavulanic acid: which time schedule do you use? [Day(s) before VUS/VCUG]
N/A

Amoxicillin/clavulanic acid: which time schedule do you use? [Day of VUS/VCUG]
N/A

Amoxicillin/clavulanic acid: which time schedule do you use? [Day(s) after VUS/VCUG]
N/A

Other: which time schedule do you use? [Day(s) before VUS/VCUG, how many?]
N/A

Other: which time schedule do you use? [Day of VUS/VCUG]
N/A

Other: which time schedule do you use? [Day(s) after VUS/VCUG, how many?]
N/A

Indication for prophylaxis

Which patients receive periinterventional antibiotic prophylaxis? (multiple answers possible) [all]
Yes

Which patients receive periinterventional antibiotic prophylaxis? (multiple answers possible) [patients post urinary tract infection/pyelonephritis/urosepsis]
N/A

Which patients receive periinterventional antibiotic prophylaxis? (multiple answers possible) [patients with urinary tract dilation in ultrasound]
N/A

Which patients receive periinterventional antibiotic prophylaxis? (multiple answers possible) [patients with prenatal urinary tract dilation]
N/A

Which patients receive periinterventional antibiotic prophylaxis? (multiple answers possible) [patients post VUR surgery]
N/A

Which patients receive periinterventional antibiotic prophylaxis? (multiple answers possible) [none]
N/A

Are there situations or clinical findings in the patient where you deviate from your prophylaxis standard (medication, dose, duration, age of the patient)? (multiple answers possible, please specify in answer box) [in case of already known vesicoureteral reflux (VUR)]
No

Are there situations or clinical findings in the patient where you deviate from your prophylaxis standard (medication, dose, duration, age of the patient)? (multiple answers possible, please specify in answer box) [in case of already known vesicoureteral reflux (VUR)][Comment]

Are there situations or clinical findings in the patient where you deviate from your prophylaxis standard (medication, dose, duration, age of the patient)? (multiple answers possible, please specify in answer box) [in patients with recurrent urinary tract infections (UTI)]
Yes

Are there situations or clinical findings in the patient where you deviate from your prophylaxis standard (medication, dose, duration, age of the patient)? (multiple answers possible, please specify in answer box) [in patients with recurrent urinary tract infections (UTI)][Comment]
nach voeliegendem Resistogramm / Keimnachweis

Are there situations or clinical findings in the patient where you deviate from your prophylaxis standard (medication, dose, duration, age of the patient)? (multiple answers possible, please specify in answer box) [if multidrug-resistant organisms (MDRO) are detected in urine cultures]
Yes

Are there situations or clinical findings in the patient where you deviate from your prophylaxis standard (medication, dose, duration, age of the patient)? (multiple answers possible, please specify in answer box) [if multidrug-resistant organisms (MDRO) are detected in urine cultures][Comment]
entsprechend Resistenzlage und Keim

Are there situations or clinical findings in the patient where you deviate from your prophylaxis standard (medication, dose, duration, age of the patient)? (multiple answers possible, please specify in answer box) [in newborns]
No

Are there situations or clinical findings in the patient where you deviate from your prophylaxis standard (medication, dose, duration, age of the patient)? (multiple answers possible, please specify in answer box) [in newborns][Comment]

Are there situations or clinical findings in the patient where you deviate from your prophylaxis standard (medication, dose, duration, age of the patient)? (multiple answers possible, please specify in answer box) [Other]
Neugeborene

Are there situations or clinical findings in the patient where you deviate from your prophylaxis standard (medication, dose, duration, age of the patient)? (multiple answers possible, please specify in answer box) [Other comment]
bekommen keine Refluxprüfung, nicht indiziert!

Are there situations in which you use intravenous prophylaxis?
No

Voiding analysis

Are there differences in antibiotic prophylaxis between VUS and VCUG in your department?
No

Is a non-pathological urine strip test required in preparation for VUS/VCUG in your department?
Yes

What is the maximum amount of days accepted for urine strip test to be performed prior to the exam?
3

Is a non-pathological urine culture required in preparation for VUS/VCUG in your department?
No

Do patients only receive post-procedure prophylaxis if vesicoureteral reflux (VUR) is detected in VUS/VCUG?
No

Do other MUS/MCU findings (e.g. urethral valves) have an influence on post-procedure prophylaxis?
No

End of survey

Thank you for taking the time to answer this survey!

May we contact you personally if we have further questions?
Yes

E-mail address:
felicitas.eckoldt@med.uni-jena.de

Survey response 221

Response ID
223

Date submitted
2025-04-02 07:41:08

Last page
5

Start language
de

Seed
651708396

Date started
2025-04-02 07:18:42

Date last action
2025-04-02 07:41:08

Standard Operation Procedure

Who fills out this survey?
Pediatric urologist

Who fills out this survey? [Other]

In which type of hospital are you employed?
Other

In which type of hospital are you employed? [Other]
kirchliches Haus: ca. 64

In which country do you work?
Deutschland

Does your department have a defined protocol for infection prophylaxis in the context of VUS/VCUG?
Yes

Has this protocol been transformed into a written standard operating procedure (SOP)?
Yes

Which department is the author of the SOP for infection prophylaxis in VUS/VCUG?
Pediatric surgery

Which department is the author of the SOP for infection prophylaxis in VUS/VCUG? [Other]

Antibiotic Prophylaxis

Are there situations/constellations in which you do not use antibiotic prophylaxis for VUS/VCUG?
Yes

Please specify these situations/constellations: [girl with first uncomplicated UTI without urinary tract dilation in ultrasound]
No

Please specify these situations/constellations: [boy with first uncomplicated UTI without urinary tract dilation in ultrasound]
No

Please specify these situations/constellations: [in patients with low grade urinary tract dilation (up to 2nd grade) and no UTI in medical history]
Yes

Please specify these situations/constellations: [Other]

If you use antibiotic prophylaxis: which mediaction do you use? (multiple answers and subdivision into age groups possible) [Trimethoprim]
Yes

If you use antibiotic prophylaxis: which mediaction do you use? (multiple answers and subdivision into age groups possible) [Nitrofurantoin]
No

If you use antibiotic prophylaxis: which mediaction do you use? (multiple answers and subdivision into age groups possible) [Amoxicillin]
No

If you use antibiotic prophylaxis: which mediaction do you use? (multiple answers and subdivision into age groups possible) [Cefaclor]
No

If you use antibiotic prophylaxis: which mediaction do you use? (multiple answers and subdivision into age groups possible) [Amoxicillin/clavulanic acid]
No

If you use antibiotic prophylaxis: which mediaction do you use? (multiple answers and subdivision into age groups possible) [Other]
No

Trimethoprim: in what dosage?
prophylactic (2 mg/kg bodyweight/d)

Trimethoprim: in what dosage? [Other]

Trimethoprim: for which age group do you use trimethoprim?
ab 6 Wochen

Trimethoprim: which time schedule do you use? [Day(s) before VUS/VCUG, how many?]
No

Trimethoprim: which time schedule do you use? [Day(s) before VUS/VCUG, how many?][Comment]

Trimethoprim: which time schedule do you use? [Day of VUS/VCUG]
Yes

Trimethoprim: which time schedule do you use? [Day of VUS/VCUG][Comment]

Trimethoprim: which time schedule do you use? [Day(s) after VUS/VCUG, how many?]
No

Trimethoprim: which time schedule do you use? [Day(s) after VUS/VCUG, how many?][Comment]

Nitrofurantoin: which time schedule do you use? [Day(s) before VUS/VCUG, how many?]
N/A

Nitrofurantoin: which time schedule do you use? [Day of VUS/VCUG]
N/A

Nitrofurantoin: which time schedule do you use? [Day(s) after VUS/VCUG, how many?]
N/A

Amoxicillin: which time schedule do you use? [Day(s) before VUS/VCUG, how many?]
N/A

Amoxicillin: which time schedule do you use? [Day of VUS/VCUG]
N/A

Amoxicillin: which time schedule do you use? [Day(s) after VUS/VCUG, how many?]
N/A

Cefaclor: which time schedule do you use? [Days(s) before VUS/VCUG, how many?]
N/A

Cefaclor: which time schedule do you use? [Day of VUS/VCUG]
N/A

Cefaclor: which time schedule do you use? [Day(s) after VUS/VCUG, how many?]
N/A

Amoxicillin/clavulanic acid: in what dosage? (Amoxicillin portion) [prophylaktic (10-20 mg/Kg bodyweight/d)]
N/A

Amoxicillin/clavulanic acid: in what dosage? (Amoxicillin portion) [double prophylactic (20-40 mg/Kg bodyweight/d)]
N/A

Amoxicillin/clavulanic acid: in what dosage? (Amoxicillin portion) [therapeutic (80-100 mg/Kg bodyweight/d)]
N/A

Amoxicillin/clavulanic acid: which time schedule do you use? [Day(s) before VUS/VCUG]
N/A

Amoxicillin/clavulanic acid: which time schedule do you use? [Day of VUS/VCUG]
N/A

Amoxicillin/clavulanic acid: which time schedule do you use? [Day(s) after VUS/VCUG]
N/A

Other: which time schedule do you use? [Day(s) before VUS/VCUG, how many?]
N/A

Other: which time schedule do you use? [Day of VUS/VCUG]
N/A

Other: which time schedule do you use? [Day(s) after VUS/VCUG, how many?]
N/A

Indication for prophylaxis

Which patients receive periinterventional antibiotic prophylaxis? (multiple answers possible) [all]
Yes

Which patients receive periinterventional antibiotic prophylaxis? (multiple answers possible) [patients post urinary tract infection/pyelonephritis/urosepsis]
N/A

Which patients receive periinterventional antibiotic prophylaxis? (multiple answers possible) [patients with urinary tract dilation in ultrasound]
N/A

Which patients receive periinterventional antibiotic prophylaxis? (multiple answers possible) [patients with prenatal urinary tract dilation]
N/A

Which patients receive periinterventional antibiotic prophylaxis? (multiple answers possible) [patients post VUR surgery]
N/A

Which patients receive periinterventional antibiotic prophylaxis? (multiple answers possible) [none]
N/A

Are there situations or clinical findings in the patient where you deviate from your prophylaxis standard (medication, dose, duration, age of the patient)? (multiple answers possible, please specify in answer box) [in case of already known vesicoureteral reflux (VUR)]
No

Are there situations or clinical findings in the patient where you deviate from your prophylaxis standard (medication, dose, duration, age of the patient)? (multiple answers possible, please specify in answer box) [in case of already known vesicoureteral reflux (VUR)][Comment]

Are there situations or clinical findings in the patient where you deviate from your prophylaxis standard (medication, dose, duration, age of the patient)? (multiple answers possible, please specify in answer box) [in patients with recurrent urinary tract infections (UTI)]
Yes

Are there situations or clinical findings in the patient where you deviate from your prophylaxis standard (medication, dose, duration, age of the patient)? (multiple answers possible, please specify in answer box) [in patients with recurrent urinary tract infections (UTI)][Comment]
nach Antibiogramm

Are there situations or clinical findings in the patient where you deviate from your prophylaxis standard (medication, dose, duration, age of the patient)? (multiple answers possible, please specify in answer box) [if multidrug-resistant organisms (MDRO) are detected in urine cultures]
Yes

Are there situations or clinical findings in the patient where you deviate from your prophylaxis standard (medication, dose, duration, age of the patient)? (multiple answers possible, please specify in answer box) [if multidrug-resistant organisms (MDRO) are detected in urine cultures][Comment]
nach Antibiogramm

Are there situations or clinical findings in the patient where you deviate from your prophylaxis standard (medication, dose, duration, age of the patient)? (multiple answers possible, please specify in answer box) [in newborns]
No

Are there situations or clinical findings in the patient where you deviate from your prophylaxis standard (medication, dose, duration, age of the patient)? (multiple answers possible, please specify in answer box) [in newborns][Comment]

Are there situations or clinical findings in the patient where you deviate from your prophylaxis standard (medication, dose, duration, age of the patient)? (multiple answers possible, please specify in answer box) [Other]

Are there situations or clinical findings in the patient where you deviate from your prophylaxis standard (medication, dose, duration, age of the patient)? (multiple answers possible, please specify in answer box) [Other comment]

Are there situations in which you use intravenous prophylaxis?
Yes

In which situations?
immer wenn ein i.v.-Zugang aus anderen Gründen benötigt wird

Voiding analysis

Are there differences in antibiotic prophylaxis between VUS and VCUG in your department?
No

Is a non-pathological urine strip test required in preparation for VUS/VCUG in your department?
Yes

What is the maximum amount of days accepted for urine strip test to be performed prior to the exam?
5

Is a non-pathological urine culture required in preparation for VUS/VCUG in your department?
No

Do patients only receive post-procedure prophylaxis if vesicoureteral reflux (VUR) is detected in VUS/VCUG?
No

Do other MUS/MCU findings (e.g. urethral valves) have an influence on post-procedure prophylaxis?
Yes

Please specify:
Dauer der Prophylaxe abhängig von Blasenentleerung ggf. verlängert

End of survey

Thank you for taking the time to answer this survey!

May we contact you personally if we have further questions?
Yes

E-mail address:
p.flacker@contilia.de

Survey response 222

Response ID
224

Last page
1

Start language
de

Seed
988487908

Date started
2025-04-02 07:21:55

Date last action
2025-04-02 07:23:22

Standard Operation Procedure

Who fills out this survey?
Pediatric Surgeon

Who fills out this survey? [Other]

In which type of hospital are you employed?
Community hospital (>50 beds for pediatric patients)

In which type of hospital are you employed? [Other]

In which country do you work?
Deutschland

Does your department have a defined protocol for infection prophylaxis in the context of VUS/VCUG?
Yes

Has this protocol been transformed into a written standard operating procedure (SOP)?
Yes

Which department is the author of the SOP for infection prophylaxis in VUS/VCUG?
Pediatrics

Which department is the author of the SOP for infection prophylaxis in VUS/VCUG? [Other]

Antibiotic Prophylaxis

Are there situations/constellations in which you do not use antibiotic prophylaxis for VUS/VCUG?
Yes

Please specify these situations/constellations: [girl with first uncomplicated UTI without urinary tract dilation in ultrasound]
No

Please specify these situations/constellations: [boy with first uncomplicated UTI without urinary tract dilation in ultrasound]
No

Please specify these situations/constellations: [in patients with low grade urinary tract dilation (up to 2nd grade) and no UTI in medical history]
No

Please specify these situations/constellations: [Other]

If you use antibiotic prophylaxis: which mediaction do you use? (multiple answers and subdivision into age groups possible) [Trimethoprim]
No

If you use antibiotic prophylaxis: which mediaction do you use? (multiple answers and subdivision into age groups possible) [Nitrofurantoin]
No

If you use antibiotic prophylaxis: which mediaction do you use? (multiple answers and subdivision into age groups possible) [Amoxicillin]
No

If you use antibiotic prophylaxis: which mediaction do you use? (multiple answers and subdivision into age groups possible) [Cefaclor]
No

If you use antibiotic prophylaxis: which mediaction do you use? (multiple answers and subdivision into age groups possible) [Amoxicillin/clavulanic acid]
No

If you use antibiotic prophylaxis: which mediaction do you use? (multiple answers and subdivision into age groups possible) [Other]
No

Trimethoprim: which time schedule do you use? [Day(s) before VUS/VCUG, how many?]
N/A

Trimethoprim: which time schedule do you use? [Day of VUS/VCUG]
N/A

Trimethoprim: which time schedule do you use? [Day(s) after VUS/VCUG, how many?]
N/A

Nitrofurantoin: which time schedule do you use? [Day(s) before VUS/VCUG, how many?]
N/A

Nitrofurantoin: which time schedule do you use? [Day of VUS/VCUG]
N/A

Nitrofurantoin: which time schedule do you use? [Day(s) after VUS/VCUG, how many?]
N/A

Amoxicillin: which time schedule do you use? [Day(s) before VUS/VCUG, how many?]
N/A

Amoxicillin: which time schedule do you use? [Day of VUS/VCUG]
N/A

Amoxicillin: which time schedule do you use? [Day(s) after VUS/VCUG, how many?]
N/A

Cefaclor: which time schedule do you use? [Days(s) before VUS/VCUG, how many?]
N/A

Cefaclor: which time schedule do you use? [Day of VUS/VCUG]
N/A

Cefaclor: which time schedule do you use? [Day(s) after VUS/VCUG, how many?]
N/A

Amoxicillin/clavulanic acid: in what dosage? (Amoxicillin portion) [prophylaktic (10-20 mg/Kg bodyweight/d)]
N/A

Amoxicillin/clavulanic acid: in what dosage? (Amoxicillin portion) [double prophylactic (20-40 mg/Kg bodyweight/d)]
N/A

Amoxicillin/clavulanic acid: in what dosage? (Amoxicillin portion) [therapeutic (80-100 mg/Kg bodyweight/d)]
N/A

Amoxicillin/clavulanic acid: which time schedule do you use? [Day(s) before VUS/VCUG]
N/A

Amoxicillin/clavulanic acid: which time schedule do you use? [Day of VUS/VCUG]
N/A

Amoxicillin/clavulanic acid: which time schedule do you use? [Day(s) after VUS/VCUG]
N/A

Other: which time schedule do you use? [Day(s) before VUS/VCUG, how many?]
N/A

Other: which time schedule do you use? [Day of VUS/VCUG]
N/A

Other: which time schedule do you use? [Day(s) after VUS/VCUG, how many?]
N/A

Indication for prophylaxis

Which patients receive periinterventional antibiotic prophylaxis? (multiple answers possible) [all]
N/A

Which patients receive periinterventional antibiotic prophylaxis? (multiple answers possible) [patients post urinary tract infection/pyelonephritis/urosepsis]
N/A

Which patients receive periinterventional antibiotic prophylaxis? (multiple answers possible) [patients with urinary tract dilation in ultrasound]
N/A

Which patients receive periinterventional antibiotic prophylaxis? (multiple answers possible) [patients with prenatal urinary tract dilation]
N/A

Which patients receive periinterventional antibiotic prophylaxis? (multiple answers possible) [patients post VUR surgery]
N/A

Which patients receive periinterventional antibiotic prophylaxis? (multiple answers possible) [none]
N/A

Are there situations or clinical findings in the patient where you deviate from your prophylaxis standard (medication, dose, duration, age of the patient)? (multiple answers possible, please specify in answer box) [in case of already known vesicoureteral reflux (VUR)]
N/A

Are there situations or clinical findings in the patient where you deviate from your prophylaxis standard (medication, dose, duration, age of the patient)? (multiple answers possible, please specify in answer box) [in patients with recurrent urinary tract infections (UTI)]
N/A

Are there situations or clinical findings in the patient where you deviate from your prophylaxis standard (medication, dose, duration, age of the patient)? (multiple answers possible, please specify in answer box) [if multidrug-resistant organisms (MDRO) are detected in urine cultures]
N/A

Are there situations or clinical findings in the patient where you deviate from your prophylaxis standard (medication, dose, duration, age of the patient)? (multiple answers possible, please specify in answer box) [in newborns]
N/A

Are there situations in which you use intravenous prophylaxis?
N/A

Voiding analysis

Are there differences in antibiotic prophylaxis between VUS and VCUG in your department?
N/A

Is a non-pathological urine strip test required in preparation for VUS/VCUG in your department?
N/A

Is a non-pathological urine culture required in preparation for VUS/VCUG in your department?
N/A

Do patients only receive post-procedure prophylaxis if vesicoureteral reflux (VUR) is detected in VUS/VCUG?
N/A

Do other MUS/MCU findings (e.g. urethral valves) have an influence on post-procedure prophylaxis?
N/A

End of survey

May we contact you personally if we have further questions?
N/A

Survey response 223

Response ID
225

Last page
0

Start language
de

Seed
1359906907

Date started
2025-04-02 07:50:41

Date last action
2025-04-02 07:50:41

Standard Operation Procedure

Does your department have a defined protocol for infection prophylaxis in the context of VUS/VCUG?
N/A

Has this protocol been transformed into a written standard operating procedure (SOP)?
N/A

Antibiotic Prophylaxis

Are there situations/constellations in which you do not use antibiotic prophylaxis for VUS/VCUG?
N/A

Please specify these situations/constellations: [girl with first uncomplicated UTI without urinary tract dilation in ultrasound]
N/A

Please specify these situations/constellations: [boy with first uncomplicated UTI without urinary tract dilation in ultrasound]
N/A

Please specify these situations/constellations: [in patients with low grade urinary tract dilation (up to 2nd grade) and no UTI in medical history]
N/A

If you use antibiotic prophylaxis: which mediaction do you use? (multiple answers and subdivision into age groups possible) [Trimethoprim]
N/A

If you use antibiotic prophylaxis: which mediaction do you use? (multiple answers and subdivision into age groups possible) [Nitrofurantoin]
N/A

If you use antibiotic prophylaxis: which mediaction do you use? (multiple answers and subdivision into age groups possible) [Amoxicillin]
N/A

If you use antibiotic prophylaxis: which mediaction do you use? (multiple answers and subdivision into age groups possible) [Cefaclor]
N/A

If you use antibiotic prophylaxis: which mediaction do you use? (multiple answers and subdivision into age groups possible) [Amoxicillin/clavulanic acid]
N/A

If you use antibiotic prophylaxis: which mediaction do you use? (multiple answers and subdivision into age groups possible) [Other]
N/A

Trimethoprim: which time schedule do you use? [Day(s) before VUS/VCUG, how many?]
N/A

Trimethoprim: which time schedule do you use? [Day of VUS/VCUG]
N/A

Trimethoprim: which time schedule do you use? [Day(s) after VUS/VCUG, how many?]
N/A

Nitrofurantoin: which time schedule do you use? [Day(s) before VUS/VCUG, how many?]
N/A

Nitrofurantoin: which time schedule do you use? [Day of VUS/VCUG]
N/A

Nitrofurantoin: which time schedule do you use? [Day(s) after VUS/VCUG, how many?]
N/A

Amoxicillin: which time schedule do you use? [Day(s) before VUS/VCUG, how many?]
N/A

Amoxicillin: which time schedule do you use? [Day of VUS/VCUG]
N/A

Amoxicillin: which time schedule do you use? [Day(s) after VUS/VCUG, how many?]
N/A

Cefaclor: which time schedule do you use? [Days(s) before VUS/VCUG, how many?]
N/A

Cefaclor: which time schedule do you use? [Day of VUS/VCUG]
N/A

Cefaclor: which time schedule do you use? [Day(s) after VUS/VCUG, how many?]
N/A

Amoxicillin/clavulanic acid: in what dosage? (Amoxicillin portion) [prophylaktic (10-20 mg/Kg bodyweight/d)]
N/A

Amoxicillin/clavulanic acid: in what dosage? (Amoxicillin portion) [double prophylactic (20-40 mg/Kg bodyweight/d)]
N/A

Amoxicillin/clavulanic acid: in what dosage? (Amoxicillin portion) [therapeutic (80-100 mg/Kg bodyweight/d)]
N/A

Amoxicillin/clavulanic acid: which time schedule do you use? [Day(s) before VUS/VCUG]
N/A

Amoxicillin/clavulanic acid: which time schedule do you use? [Day of VUS/VCUG]
N/A

Amoxicillin/clavulanic acid: which time schedule do you use? [Day(s) after VUS/VCUG]
N/A

Other: which time schedule do you use? [Day(s) before VUS/VCUG, how many?]
N/A

Other: which time schedule do you use? [Day of VUS/VCUG]
N/A

Other: which time schedule do you use? [Day(s) after VUS/VCUG, how many?]
N/A

Indication for prophylaxis

Which patients receive periinterventional antibiotic prophylaxis? (multiple answers possible) [all]
N/A

Which patients receive periinterventional antibiotic prophylaxis? (multiple answers possible) [patients post urinary tract infection/pyelonephritis/urosepsis]
N/A

Which patients receive periinterventional antibiotic prophylaxis? (multiple answers possible) [patients with urinary tract dilation in ultrasound]
N/A

Which patients receive periinterventional antibiotic prophylaxis? (multiple answers possible) [patients with prenatal urinary tract dilation]
N/A

Which patients receive periinterventional antibiotic prophylaxis? (multiple answers possible) [patients post VUR surgery]
N/A

Which patients receive periinterventional antibiotic prophylaxis? (multiple answers possible) [none]
N/A

Are there situations or clinical findings in the patient where you deviate from your prophylaxis standard (medication, dose, duration, age of the patient)? (multiple answers possible, please specify in answer box) [in case of already known vesicoureteral reflux (VUR)]
N/A

Are there situations or clinical findings in the patient where you deviate from your prophylaxis standard (medication, dose, duration, age of the patient)? (multiple answers possible, please specify in answer box) [in patients with recurrent urinary tract infections (UTI)]
N/A

Are there situations or clinical findings in the patient where you deviate from your prophylaxis standard (medication, dose, duration, age of the patient)? (multiple answers possible, please specify in answer box) [if multidrug-resistant organisms (MDRO) are detected in urine cultures]
N/A

Are there situations or clinical findings in the patient where you deviate from your prophylaxis standard (medication, dose, duration, age of the patient)? (multiple answers possible, please specify in answer box) [in newborns]
N/A

Are there situations in which you use intravenous prophylaxis?
N/A

Voiding analysis

Are there differences in antibiotic prophylaxis between VUS and VCUG in your department?
N/A

Is a non-pathological urine strip test required in preparation for VUS/VCUG in your department?
N/A

Is a non-pathological urine culture required in preparation for VUS/VCUG in your department?
N/A

Do patients only receive post-procedure prophylaxis if vesicoureteral reflux (VUR) is detected in VUS/VCUG?
N/A

Do other MUS/MCU findings (e.g. urethral valves) have an influence on post-procedure prophylaxis?
N/A

End of survey

May we contact you personally if we have further questions?
N/A

Survey response 224

Response ID
226

Date submitted
2025-04-02 08:54:24

Last page
5

Start language
de

Seed
680845329

Date started
2025-04-02 08:52:09

Date last action
2025-04-02 08:54:24

Standard Operation Procedure

Who fills out this survey?
Pediatric Surgeon

Who fills out this survey? [Other]

In which type of hospital are you employed?
University Hospital

In which type of hospital are you employed? [Other]

In which country do you work?
Deutschland

Does your department have a defined protocol for infection prophylaxis in the context of VUS/VCUG?
Yes

Has this protocol been transformed into a written standard operating procedure (SOP)?
Yes

Which department is the author of the SOP for infection prophylaxis in VUS/VCUG?
Pediatric surgery

Which department is the author of the SOP for infection prophylaxis in VUS/VCUG? [Other]

Antibiotic Prophylaxis

Are there situations/constellations in which you do not use antibiotic prophylaxis for VUS/VCUG?
No

Please specify these situations/constellations: [girl with first uncomplicated UTI without urinary tract dilation in ultrasound]
N/A

Please specify these situations/constellations: [boy with first uncomplicated UTI without urinary tract dilation in ultrasound]
N/A

Please specify these situations/constellations: [in patients with low grade urinary tract dilation (up to 2nd grade) and no UTI in medical history]
N/A

If you use antibiotic prophylaxis: which mediaction do you use? (multiple answers and subdivision into age groups possible) [Trimethoprim]
Yes

If you use antibiotic prophylaxis: which mediaction do you use? (multiple answers and subdivision into age groups possible) [Nitrofurantoin]
No

If you use antibiotic prophylaxis: which mediaction do you use? (multiple answers and subdivision into age groups possible) [Amoxicillin]
No

If you use antibiotic prophylaxis: which mediaction do you use? (multiple answers and subdivision into age groups possible) [Cefaclor]
No

If you use antibiotic prophylaxis: which mediaction do you use? (multiple answers and subdivision into age groups possible) [Amoxicillin/clavulanic acid]
No

If you use antibiotic prophylaxis: which mediaction do you use? (multiple answers and subdivision into age groups possible) [Other]
No

Trimethoprim: in what dosage?
double prophylakctic (4 mg/kg bodyweight/d)

Trimethoprim: in what dosage? [Other]

Trimethoprim: for which age group do you use trimethoprim?
alle

Trimethoprim: which time schedule do you use? [Day(s) before VUS/VCUG, how many?]
No

Trimethoprim: which time schedule do you use? [Day(s) before VUS/VCUG, how many?][Comment]

Trimethoprim: which time schedule do you use? [Day of VUS/VCUG]
Yes

Trimethoprim: which time schedule do you use? [Day of VUS/VCUG][Comment]

Trimethoprim: which time schedule do you use? [Day(s) after VUS/VCUG, how many?]
No

Trimethoprim: which time schedule do you use? [Day(s) after VUS/VCUG, how many?][Comment]

Nitrofurantoin: which time schedule do you use? [Day(s) before VUS/VCUG, how many?]
N/A

Nitrofurantoin: which time schedule do you use? [Day of VUS/VCUG]
N/A

Nitrofurantoin: which time schedule do you use? [Day(s) after VUS/VCUG, how many?]
N/A

Amoxicillin: which time schedule do you use? [Day(s) before VUS/VCUG, how many?]
N/A

Amoxicillin: which time schedule do you use? [Day of VUS/VCUG]
N/A

Amoxicillin: which time schedule do you use? [Day(s) after VUS/VCUG, how many?]
N/A

Cefaclor: which time schedule do you use? [Days(s) before VUS/VCUG, how many?]
N/A

Cefaclor: which time schedule do you use? [Day of VUS/VCUG]
N/A

Cefaclor: which time schedule do you use? [Day(s) after VUS/VCUG, how many?]
N/A

Amoxicillin/clavulanic acid: in what dosage? (Amoxicillin portion) [prophylaktic (10-20 mg/Kg bodyweight/d)]
N/A

Amoxicillin/clavulanic acid: in what dosage? (Amoxicillin portion) [double prophylactic (20-40 mg/Kg bodyweight/d)]
N/A

Amoxicillin/clavulanic acid: in what dosage? (Amoxicillin portion) [therapeutic (80-100 mg/Kg bodyweight/d)]
N/A

Amoxicillin/clavulanic acid: which time schedule do you use? [Day(s) before VUS/VCUG]
N/A

Amoxicillin/clavulanic acid: which time schedule do you use? [Day of VUS/VCUG]
N/A

Amoxicillin/clavulanic acid: which time schedule do you use? [Day(s) after VUS/VCUG]
N/A

Other: which time schedule do you use? [Day(s) before VUS/VCUG, how many?]
N/A

Other: which time schedule do you use? [Day of VUS/VCUG]
N/A

Other: which time schedule do you use? [Day(s) after VUS/VCUG, how many?]
N/A

Indication for prophylaxis

Which patients receive periinterventional antibiotic prophylaxis? (multiple answers possible) [all]
Yes

Which patients receive periinterventional antibiotic prophylaxis? (multiple answers possible) [patients post urinary tract infection/pyelonephritis/urosepsis]
N/A

Which patients receive periinterventional antibiotic prophylaxis? (multiple answers possible) [patients with urinary tract dilation in ultrasound]
N/A

Which patients receive periinterventional antibiotic prophylaxis? (multiple answers possible) [patients with prenatal urinary tract dilation]
N/A

Which patients receive periinterventional antibiotic prophylaxis? (multiple answers possible) [patients post VUR surgery]
N/A

Which patients receive periinterventional antibiotic prophylaxis? (multiple answers possible) [none]
N/A

Are there situations or clinical findings in the patient where you deviate from your prophylaxis standard (medication, dose, duration, age of the patient)? (multiple answers possible, please specify in answer box) [in case of already known vesicoureteral reflux (VUR)]
No

Are there situations or clinical findings in the patient where you deviate from your prophylaxis standard (medication, dose, duration, age of the patient)? (multiple answers possible, please specify in answer box) [in case of already known vesicoureteral reflux (VUR)][Comment]

Are there situations or clinical findings in the patient where you deviate from your prophylaxis standard (medication, dose, duration, age of the patient)? (multiple answers possible, please specify in answer box) [in patients with recurrent urinary tract infections (UTI)]
No

Are there situations or clinical findings in the patient where you deviate from your prophylaxis standard (medication, dose, duration, age of the patient)? (multiple answers possible, please specify in answer box) [in patients with recurrent urinary tract infections (UTI)][Comment]

Are there situations or clinical findings in the patient where you deviate from your prophylaxis standard (medication, dose, duration, age of the patient)? (multiple answers possible, please specify in answer box) [if multidrug-resistant organisms (MDRO) are detected in urine cultures]
Yes

Are there situations or clinical findings in the patient where you deviate from your prophylaxis standard (medication, dose, duration, age of the patient)? (multiple answers possible, please specify in answer box) [if multidrug-resistant organisms (MDRO) are detected in urine cultures][Comment]

Are there situations or clinical findings in the patient where you deviate from your prophylaxis standard (medication, dose, duration, age of the patient)? (multiple answers possible, please specify in answer box) [in newborns]
No

Are there situations or clinical findings in the patient where you deviate from your prophylaxis standard (medication, dose, duration, age of the patient)? (multiple answers possible, please specify in answer box) [in newborns][Comment]

Are there situations or clinical findings in the patient where you deviate from your prophylaxis standard (medication, dose, duration, age of the patient)? (multiple answers possible, please specify in answer box) [Other]

Are there situations or clinical findings in the patient where you deviate from your prophylaxis standard (medication, dose, duration, age of the patient)? (multiple answers possible, please specify in answer box) [Other comment]

Are there situations in which you use intravenous prophylaxis?
No

Voiding analysis

Are there differences in antibiotic prophylaxis between VUS and VCUG in your department?
No

Is a non-pathological urine strip test required in preparation for VUS/VCUG in your department?
Yes

What is the maximum amount of days accepted for urine strip test to be performed prior to the exam?
1

Is a non-pathological urine culture required in preparation for VUS/VCUG in your department?
No

Do patients only receive post-procedure prophylaxis if vesicoureteral reflux (VUR) is detected in VUS/VCUG?
No

Do other MUS/MCU findings (e.g. urethral valves) have an influence on post-procedure prophylaxis?
No

End of survey

Thank you for taking the time to answer this survey!

May we contact you personally if we have further questions?
No

Survey response 225

Response ID
227

Last page
1

Start language
de

Seed
2014407524

Date started
2025-04-02 10:08:03

Date last action
2025-04-02 10:08:21

Standard Operation Procedure

Who fills out this survey?
Pediatric Surgeon

Who fills out this survey? [Other]

In which type of hospital are you employed?
University Hospital

In which type of hospital are you employed? [Other]

In which country do you work?
Deutschland

Does your department have a defined protocol for infection prophylaxis in the context of VUS/VCUG?
Yes

Has this protocol been transformed into a written standard operating procedure (SOP)?
Yes

Which department is the author of the SOP for infection prophylaxis in VUS/VCUG?
Pediatric surgery

Which department is the author of the SOP for infection prophylaxis in VUS/VCUG? [Other]

Antibiotic Prophylaxis

Are there situations/constellations in which you do not use antibiotic prophylaxis for VUS/VCUG?
N/A

Please specify these situations/constellations: [girl with first uncomplicated UTI without urinary tract dilation in ultrasound]
N/A

Please specify these situations/constellations: [boy with first uncomplicated UTI without urinary tract dilation in ultrasound]
N/A

Please specify these situations/constellations: [in patients with low grade urinary tract dilation (up to 2nd grade) and no UTI in medical history]
N/A

If you use antibiotic prophylaxis: which mediaction do you use? (multiple answers and subdivision into age groups possible) [Trimethoprim]
N/A

If you use antibiotic prophylaxis: which mediaction do you use? (multiple answers and subdivision into age groups possible) [Nitrofurantoin]
N/A

If you use antibiotic prophylaxis: which mediaction do you use? (multiple answers and subdivision into age groups possible) [Amoxicillin]
N/A

If you use antibiotic prophylaxis: which mediaction do you use? (multiple answers and subdivision into age groups possible) [Cefaclor]
N/A

If you use antibiotic prophylaxis: which mediaction do you use? (multiple answers and subdivision into age groups possible) [Amoxicillin/clavulanic acid]
N/A

If you use antibiotic prophylaxis: which mediaction do you use? (multiple answers and subdivision into age groups possible) [Other]
N/A

Trimethoprim: which time schedule do you use? [Day(s) before VUS/VCUG, how many?]
N/A

Trimethoprim: which time schedule do you use? [Day of VUS/VCUG]
N/A

Trimethoprim: which time schedule do you use? [Day(s) after VUS/VCUG, how many?]
N/A

Nitrofurantoin: which time schedule do you use? [Day(s) before VUS/VCUG, how many?]
N/A

Nitrofurantoin: which time schedule do you use? [Day of VUS/VCUG]
N/A

Nitrofurantoin: which time schedule do you use? [Day(s) after VUS/VCUG, how many?]
N/A

Amoxicillin: which time schedule do you use? [Day(s) before VUS/VCUG, how many?]
N/A

Amoxicillin: which time schedule do you use? [Day of VUS/VCUG]
N/A

Amoxicillin: which time schedule do you use? [Day(s) after VUS/VCUG, how many?]
N/A

Cefaclor: which time schedule do you use? [Days(s) before VUS/VCUG, how many?]
N/A

Cefaclor: which time schedule do you use? [Day of VUS/VCUG]
N/A

Cefaclor: which time schedule do you use? [Day(s) after VUS/VCUG, how many?]
N/A

Amoxicillin/clavulanic acid: in what dosage? (Amoxicillin portion) [prophylaktic (10-20 mg/Kg bodyweight/d)]
N/A

Amoxicillin/clavulanic acid: in what dosage? (Amoxicillin portion) [double prophylactic (20-40 mg/Kg bodyweight/d)]
N/A

Amoxicillin/clavulanic acid: in what dosage? (Amoxicillin portion) [therapeutic (80-100 mg/Kg bodyweight/d)]
N/A

Amoxicillin/clavulanic acid: which time schedule do you use? [Day(s) before VUS/VCUG]
N/A

Amoxicillin/clavulanic acid: which time schedule do you use? [Day of VUS/VCUG]
N/A

Amoxicillin/clavulanic acid: which time schedule do you use? [Day(s) after VUS/VCUG]
N/A

Other: which time schedule do you use? [Day(s) before VUS/VCUG, how many?]
N/A

Other: which time schedule do you use? [Day of VUS/VCUG]
N/A

Other: which time schedule do you use? [Day(s) after VUS/VCUG, how many?]
N/A

Indication for prophylaxis

Which patients receive periinterventional antibiotic prophylaxis? (multiple answers possible) [all]
N/A

Which patients receive periinterventional antibiotic prophylaxis? (multiple answers possible) [patients post urinary tract infection/pyelonephritis/urosepsis]
N/A

Which patients receive periinterventional antibiotic prophylaxis? (multiple answers possible) [patients with urinary tract dilation in ultrasound]
N/A

Which patients receive periinterventional antibiotic prophylaxis? (multiple answers possible) [patients with prenatal urinary tract dilation]
N/A

Which patients receive periinterventional antibiotic prophylaxis? (multiple answers possible) [patients post VUR surgery]
N/A

Which patients receive periinterventional antibiotic prophylaxis? (multiple answers possible) [none]
N/A

Are there situations or clinical findings in the patient where you deviate from your prophylaxis standard (medication, dose, duration, age of the patient)? (multiple answers possible, please specify in answer box) [in case of already known vesicoureteral reflux (VUR)]
N/A

Are there situations or clinical findings in the patient where you deviate from your prophylaxis standard (medication, dose, duration, age of the patient)? (multiple answers possible, please specify in answer box) [in patients with recurrent urinary tract infections (UTI)]
N/A

Are there situations or clinical findings in the patient where you deviate from your prophylaxis standard (medication, dose, duration, age of the patient)? (multiple answers possible, please specify in answer box) [if multidrug-resistant organisms (MDRO) are detected in urine cultures]
N/A

Are there situations or clinical findings in the patient where you deviate from your prophylaxis standard (medication, dose, duration, age of the patient)? (multiple answers possible, please specify in answer box) [in newborns]
N/A

Are there situations in which you use intravenous prophylaxis?
N/A

Voiding analysis

Are there differences in antibiotic prophylaxis between VUS and VCUG in your department?
N/A

Is a non-pathological urine strip test required in preparation for VUS/VCUG in your department?
N/A

Is a non-pathological urine culture required in preparation for VUS/VCUG in your department?
N/A

Do patients only receive post-procedure prophylaxis if vesicoureteral reflux (VUR) is detected in VUS/VCUG?
N/A

Do other MUS/MCU findings (e.g. urethral valves) have an influence on post-procedure prophylaxis?
N/A

End of survey

May we contact you personally if we have further questions?
N/A

Survey response 226

Response ID
228

Last page
1

Start language
de

Seed
1718240530

Date started
2025-04-02 13:48:44

Date last action
2025-04-02 13:49:25

Standard Operation Procedure

Who fills out this survey?
Pediatric urologist

Who fills out this survey? [Other]

In which type of hospital are you employed?
University Hospital

In which type of hospital are you employed? [Other]

In which country do you work?
Deutschland

Does your department have a defined protocol for infection prophylaxis in the context of VUS/VCUG?
Yes

Has this protocol been transformed into a written standard operating procedure (SOP)?
Yes

Which department is the author of the SOP for infection prophylaxis in VUS/VCUG?
Pediatric surgery

Which department is the author of the SOP for infection prophylaxis in VUS/VCUG? [Other]

Antibiotic Prophylaxis

Are there situations/constellations in which you do not use antibiotic prophylaxis for VUS/VCUG?
N/A

Please specify these situations/constellations: [girl with first uncomplicated UTI without urinary tract dilation in ultrasound]
N/A

Please specify these situations/constellations: [boy with first uncomplicated UTI without urinary tract dilation in ultrasound]
N/A

Please specify these situations/constellations: [in patients with low grade urinary tract dilation (up to 2nd grade) and no UTI in medical history]
N/A

If you use antibiotic prophylaxis: which mediaction do you use? (multiple answers and subdivision into age groups possible) [Trimethoprim]
N/A

If you use antibiotic prophylaxis: which mediaction do you use? (multiple answers and subdivision into age groups possible) [Nitrofurantoin]
N/A

If you use antibiotic prophylaxis: which mediaction do you use? (multiple answers and subdivision into age groups possible) [Amoxicillin]
N/A

If you use antibiotic prophylaxis: which mediaction do you use? (multiple answers and subdivision into age groups possible) [Cefaclor]
N/A

If you use antibiotic prophylaxis: which mediaction do you use? (multiple answers and subdivision into age groups possible) [Amoxicillin/clavulanic acid]
N/A

If you use antibiotic prophylaxis: which mediaction do you use? (multiple answers and subdivision into age groups possible) [Other]
N/A

Trimethoprim: which time schedule do you use? [Day(s) before VUS/VCUG, how many?]
N/A

Trimethoprim: which time schedule do you use? [Day of VUS/VCUG]
N/A

Trimethoprim: which time schedule do you use? [Day(s) after VUS/VCUG, how many?]
N/A

Nitrofurantoin: which time schedule do you use? [Day(s) before VUS/VCUG, how many?]
N/A

Nitrofurantoin: which time schedule do you use? [Day of VUS/VCUG]
N/A

Nitrofurantoin: which time schedule do you use? [Day(s) after VUS/VCUG, how many?]
N/A

Amoxicillin: which time schedule do you use? [Day(s) before VUS/VCUG, how many?]
N/A

Amoxicillin: which time schedule do you use? [Day of VUS/VCUG]
N/A

Amoxicillin: which time schedule do you use? [Day(s) after VUS/VCUG, how many?]
N/A

Cefaclor: which time schedule do you use? [Days(s) before VUS/VCUG, how many?]
N/A

Cefaclor: which time schedule do you use? [Day of VUS/VCUG]
N/A

Cefaclor: which time schedule do you use? [Day(s) after VUS/VCUG, how many?]
N/A

Amoxicillin/clavulanic acid: in what dosage? (Amoxicillin portion) [prophylaktic (10-20 mg/Kg bodyweight/d)]
N/A

Amoxicillin/clavulanic acid: in what dosage? (Amoxicillin portion) [double prophylactic (20-40 mg/Kg bodyweight/d)]
N/A

Amoxicillin/clavulanic acid: in what dosage? (Amoxicillin portion) [therapeutic (80-100 mg/Kg bodyweight/d)]
N/A

Amoxicillin/clavulanic acid: which time schedule do you use? [Day(s) before VUS/VCUG]
N/A

Amoxicillin/clavulanic acid: which time schedule do you use? [Day of VUS/VCUG]
N/A

Amoxicillin/clavulanic acid: which time schedule do you use? [Day(s) after VUS/VCUG]
N/A

Other: which time schedule do you use? [Day(s) before VUS/VCUG, how many?]
N/A

Other: which time schedule do you use? [Day of VUS/VCUG]
N/A

Other: which time schedule do you use? [Day(s) after VUS/VCUG, how many?]
N/A

Indication for prophylaxis

Which patients receive periinterventional antibiotic prophylaxis? (multiple answers possible) [all]
N/A

Which patients receive periinterventional antibiotic prophylaxis? (multiple answers possible) [patients post urinary tract infection/pyelonephritis/urosepsis]
N/A

Which patients receive periinterventional antibiotic prophylaxis? (multiple answers possible) [patients with urinary tract dilation in ultrasound]
N/A

Which patients receive periinterventional antibiotic prophylaxis? (multiple answers possible) [patients with prenatal urinary tract dilation]
N/A

Which patients receive periinterventional antibiotic prophylaxis? (multiple answers possible) [patients post VUR surgery]
N/A

Which patients receive periinterventional antibiotic prophylaxis? (multiple answers possible) [none]
N/A

Are there situations or clinical findings in the patient where you deviate from your prophylaxis standard (medication, dose, duration, age of the patient)? (multiple answers possible, please specify in answer box) [in case of already known vesicoureteral reflux (VUR)]
N/A

Are there situations or clinical findings in the patient where you deviate from your prophylaxis standard (medication, dose, duration, age of the patient)? (multiple answers possible, please specify in answer box) [in patients with recurrent urinary tract infections (UTI)]
N/A

Are there situations or clinical findings in the patient where you deviate from your prophylaxis standard (medication, dose, duration, age of the patient)? (multiple answers possible, please specify in answer box) [if multidrug-resistant organisms (MDRO) are detected in urine cultures]
N/A

Are there situations or clinical findings in the patient where you deviate from your prophylaxis standard (medication, dose, duration, age of the patient)? (multiple answers possible, please specify in answer box) [in newborns]
N/A

Are there situations in which you use intravenous prophylaxis?
N/A

Voiding analysis

Are there differences in antibiotic prophylaxis between VUS and VCUG in your department?
N/A

Is a non-pathological urine strip test required in preparation for VUS/VCUG in your department?
N/A

Is a non-pathological urine culture required in preparation for VUS/VCUG in your department?
N/A

Do patients only receive post-procedure prophylaxis if vesicoureteral reflux (VUR) is detected in VUS/VCUG?
N/A

Do other MUS/MCU findings (e.g. urethral valves) have an influence on post-procedure prophylaxis?
N/A

End of survey

May we contact you personally if we have further questions?
N/A

Survey response 227

Response ID
229

Last page
1

Start language
de

Seed
906523836

Date started
2025-04-02 19:14:36

Date last action
2025-04-02 19:15:25

Standard Operation Procedure

Who fills out this survey?
Pediatric Surgeon

Who fills out this survey? [Other]

In which type of hospital are you employed?
University Hospital

In which type of hospital are you employed? [Other]

In which country do you work?
Deutschland

Does your department have a defined protocol for infection prophylaxis in the context of VUS/VCUG?
Yes

Has this protocol been transformed into a written standard operating procedure (SOP)?
No

Antibiotic Prophylaxis

Are there situations/constellations in which you do not use antibiotic prophylaxis for VUS/VCUG?
N/A

Please specify these situations/constellations: [girl with first uncomplicated UTI without urinary tract dilation in ultrasound]
N/A

Please specify these situations/constellations: [boy with first uncomplicated UTI without urinary tract dilation in ultrasound]
N/A

Please specify these situations/constellations: [in patients with low grade urinary tract dilation (up to 2nd grade) and no UTI in medical history]
N/A

If you use antibiotic prophylaxis: which mediaction do you use? (multiple answers and subdivision into age groups possible) [Trimethoprim]
N/A

If you use antibiotic prophylaxis: which mediaction do you use? (multiple answers and subdivision into age groups possible) [Nitrofurantoin]
N/A

If you use antibiotic prophylaxis: which mediaction do you use? (multiple answers and subdivision into age groups possible) [Amoxicillin]
N/A

If you use antibiotic prophylaxis: which mediaction do you use? (multiple answers and subdivision into age groups possible) [Cefaclor]
N/A

If you use antibiotic prophylaxis: which mediaction do you use? (multiple answers and subdivision into age groups possible) [Amoxicillin/clavulanic acid]
N/A

If you use antibiotic prophylaxis: which mediaction do you use? (multiple answers and subdivision into age groups possible) [Other]
N/A

Trimethoprim: which time schedule do you use? [Day(s) before VUS/VCUG, how many?]
N/A

Trimethoprim: which time schedule do you use? [Day of VUS/VCUG]
N/A

Trimethoprim: which time schedule do you use? [Day(s) after VUS/VCUG, how many?]
N/A

Nitrofurantoin: which time schedule do you use? [Day(s) before VUS/VCUG, how many?]
N/A

Nitrofurantoin: which time schedule do you use? [Day of VUS/VCUG]
N/A

Nitrofurantoin: which time schedule do you use? [Day(s) after VUS/VCUG, how many?]
N/A

Amoxicillin: which time schedule do you use? [Day(s) before VUS/VCUG, how many?]
N/A

Amoxicillin: which time schedule do you use? [Day of VUS/VCUG]
N/A

Amoxicillin: which time schedule do you use? [Day(s) after VUS/VCUG, how many?]
N/A

Cefaclor: which time schedule do you use? [Days(s) before VUS/VCUG, how many?]
N/A

Cefaclor: which time schedule do you use? [Day of VUS/VCUG]
N/A

Cefaclor: which time schedule do you use? [Day(s) after VUS/VCUG, how many?]
N/A

Amoxicillin/clavulanic acid: in what dosage? (Amoxicillin portion) [prophylaktic (10-20 mg/Kg bodyweight/d)]
N/A

Amoxicillin/clavulanic acid: in what dosage? (Amoxicillin portion) [double prophylactic (20-40 mg/Kg bodyweight/d)]
N/A

Amoxicillin/clavulanic acid: in what dosage? (Amoxicillin portion) [therapeutic (80-100 mg/Kg bodyweight/d)]
N/A

Amoxicillin/clavulanic acid: which time schedule do you use? [Day(s) before VUS/VCUG]
N/A

Amoxicillin/clavulanic acid: which time schedule do you use? [Day of VUS/VCUG]
N/A

Amoxicillin/clavulanic acid: which time schedule do you use? [Day(s) after VUS/VCUG]
N/A

Other: which time schedule do you use? [Day(s) before VUS/VCUG, how many?]
N/A

Other: which time schedule do you use? [Day of VUS/VCUG]
N/A

Other: which time schedule do you use? [Day(s) after VUS/VCUG, how many?]
N/A

Indication for prophylaxis

Which patients receive periinterventional antibiotic prophylaxis? (multiple answers possible) [all]
N/A

Which patients receive periinterventional antibiotic prophylaxis? (multiple answers possible) [patients post urinary tract infection/pyelonephritis/urosepsis]
N/A

Which patients receive periinterventional antibiotic prophylaxis? (multiple answers possible) [patients with urinary tract dilation in ultrasound]
N/A

Which patients receive periinterventional antibiotic prophylaxis? (multiple answers possible) [patients with prenatal urinary tract dilation]
N/A

Which patients receive periinterventional antibiotic prophylaxis? (multiple answers possible) [patients post VUR surgery]
N/A

Which patients receive periinterventional antibiotic prophylaxis? (multiple answers possible) [none]
N/A

Are there situations or clinical findings in the patient where you deviate from your prophylaxis standard (medication, dose, duration, age of the patient)? (multiple answers possible, please specify in answer box) [in case of already known vesicoureteral reflux (VUR)]
N/A

Are there situations or clinical findings in the patient where you deviate from your prophylaxis standard (medication, dose, duration, age of the patient)? (multiple answers possible, please specify in answer box) [in patients with recurrent urinary tract infections (UTI)]
N/A

Are there situations or clinical findings in the patient where you deviate from your prophylaxis standard (medication, dose, duration, age of the patient)? (multiple answers possible, please specify in answer box) [if multidrug-resistant organisms (MDRO) are detected in urine cultures]
N/A

Are there situations or clinical findings in the patient where you deviate from your prophylaxis standard (medication, dose, duration, age of the patient)? (multiple answers possible, please specify in answer box) [in newborns]
N/A

Are there situations in which you use intravenous prophylaxis?
N/A

Voiding analysis

Are there differences in antibiotic prophylaxis between VUS and VCUG in your department?
N/A

Is a non-pathological urine strip test required in preparation for VUS/VCUG in your department?
N/A

Is a non-pathological urine culture required in preparation for VUS/VCUG in your department?
N/A

Do patients only receive post-procedure prophylaxis if vesicoureteral reflux (VUR) is detected in VUS/VCUG?
N/A

Do other MUS/MCU findings (e.g. urethral valves) have an influence on post-procedure prophylaxis?
N/A

End of survey

May we contact you personally if we have further questions?
N/A

Survey response 228

Response ID
230

Date submitted
2025-04-03 10:19:16

Last page
5

Start language
de

Seed
815071020

Date started
2025-04-03 10:15:50

Date last action
2025-04-03 10:19:16

Standard Operation Procedure

Who fills out this survey?
Pediatric Surgeon

Who fills out this survey? [Other]

In which type of hospital are you employed?
University Hospital

In which type of hospital are you employed? [Other]

In which country do you work?
Deutschland

Does your department have a defined protocol for infection prophylaxis in the context of VUS/VCUG?
No

Has this protocol been transformed into a written standard operating procedure (SOP)?
N/A

Antibiotic Prophylaxis

Are there situations/constellations in which you do not use antibiotic prophylaxis for VUS/VCUG?
Yes

Please specify these situations/constellations: [girl with first uncomplicated UTI without urinary tract dilation in ultrasound]
No

Please specify these situations/constellations: [boy with first uncomplicated UTI without urinary tract dilation in ultrasound]
No

Please specify these situations/constellations: [in patients with low grade urinary tract dilation (up to 2nd grade) and no UTI in medical history]
No

Please specify these situations/constellations: [Other]
Kein VUR im MCU

If you use antibiotic prophylaxis: which mediaction do you use? (multiple answers and subdivision into age groups possible) [Trimethoprim]
No

If you use antibiotic prophylaxis: which mediaction do you use? (multiple answers and subdivision into age groups possible) [Nitrofurantoin]
No

If you use antibiotic prophylaxis: which mediaction do you use? (multiple answers and subdivision into age groups possible) [Amoxicillin]
No

If you use antibiotic prophylaxis: which mediaction do you use? (multiple answers and subdivision into age groups possible) [Cefaclor]
Yes

If you use antibiotic prophylaxis: which mediaction do you use? (multiple answers and subdivision into age groups possible) [Amoxicillin/clavulanic acid]
No

If you use antibiotic prophylaxis: which mediaction do you use? (multiple answers and subdivision into age groups possible) [Other]
No

Trimethoprim: which time schedule do you use? [Day(s) before VUS/VCUG, how many?]
N/A

Trimethoprim: which time schedule do you use? [Day of VUS/VCUG]
N/A

Trimethoprim: which time schedule do you use? [Day(s) after VUS/VCUG, how many?]
N/A

Nitrofurantoin: which time schedule do you use? [Day(s) before VUS/VCUG, how many?]
N/A

Nitrofurantoin: which time schedule do you use? [Day of VUS/VCUG]
N/A

Nitrofurantoin: which time schedule do you use? [Day(s) after VUS/VCUG, how many?]
N/A

Amoxicillin: which time schedule do you use? [Day(s) before VUS/VCUG, how many?]
N/A

Amoxicillin: which time schedule do you use? [Day of VUS/VCUG]
N/A

Amoxicillin: which time schedule do you use? [Day(s) after VUS/VCUG, how many?]
N/A

Cefaclor: in what dosage?
therapeutic (40 mg/kg bodyweight/d)

Cefaclor: in what dosage? [Other]

Cefaclor: for which age group do you use cefaclor?
Unter 6 Wochen

Cefaclor: which time schedule do you use? [Days(s) before VUS/VCUG, how many?]
No

Cefaclor: which time schedule do you use? [Days(s) before VUS/VCUG, how many?][Comment]

Cefaclor: which time schedule do you use? [Day of VUS/VCUG]
Yes

Cefaclor: which time schedule do you use? [Day of VUS/VCUG][Comment]

Cefaclor: which time schedule do you use? [Day(s) after VUS/VCUG, how many?]
Yes

Cefaclor: which time schedule do you use? [Day(s) after VUS/VCUG, how many?][Comment]
3 d

Amoxicillin/clavulanic acid: in what dosage? (Amoxicillin portion) [prophylaktic (10-20 mg/Kg bodyweight/d)]
N/A

Amoxicillin/clavulanic acid: in what dosage? (Amoxicillin portion) [double prophylactic (20-40 mg/Kg bodyweight/d)]
N/A

Amoxicillin/clavulanic acid: in what dosage? (Amoxicillin portion) [therapeutic (80-100 mg/Kg bodyweight/d)]
N/A

Amoxicillin/clavulanic acid: which time schedule do you use? [Day(s) before VUS/VCUG]
N/A

Amoxicillin/clavulanic acid: which time schedule do you use? [Day of VUS/VCUG]
N/A

Amoxicillin/clavulanic acid: which time schedule do you use? [Day(s) after VUS/VCUG]
N/A

Other: which time schedule do you use? [Day(s) before VUS/VCUG, how many?]
N/A

Other: which time schedule do you use? [Day of VUS/VCUG]
N/A

Other: which time schedule do you use? [Day(s) after VUS/VCUG, how many?]
N/A

Indication for prophylaxis

Which patients receive periinterventional antibiotic prophylaxis? (multiple answers possible) [all]
No

Which patients receive periinterventional antibiotic prophylaxis? (multiple answers possible) [patients post urinary tract infection/pyelonephritis/urosepsis]
No

Which patients receive periinterventional antibiotic prophylaxis? (multiple answers possible) [patients with urinary tract dilation in ultrasound]
No

Which patients receive periinterventional antibiotic prophylaxis? (multiple answers possible) [patients with prenatal urinary tract dilation]
No

Which patients receive periinterventional antibiotic prophylaxis? (multiple answers possible) [patients post VUR surgery]
No

Which patients receive periinterventional antibiotic prophylaxis? (multiple answers possible) [none]
Yes

Which patients receive periinterventional antibiotic prophylaxis? (multiple answers possible) [Other]

Are there situations or clinical findings in the patient where you deviate from your prophylaxis standard (medication, dose, duration, age of the patient)? (multiple answers possible, please specify in answer box) [in case of already known vesicoureteral reflux (VUR)]
No

Are there situations or clinical findings in the patient where you deviate from your prophylaxis standard (medication, dose, duration, age of the patient)? (multiple answers possible, please specify in answer box) [in case of already known vesicoureteral reflux (VUR)][Comment]

Are there situations or clinical findings in the patient where you deviate from your prophylaxis standard (medication, dose, duration, age of the patient)? (multiple answers possible, please specify in answer box) [in patients with recurrent urinary tract infections (UTI)]
No

Are there situations or clinical findings in the patient where you deviate from your prophylaxis standard (medication, dose, duration, age of the patient)? (multiple answers possible, please specify in answer box) [in patients with recurrent urinary tract infections (UTI)][Comment]

Are there situations or clinical findings in the patient where you deviate from your prophylaxis standard (medication, dose, duration, age of the patient)? (multiple answers possible, please specify in answer box) [if multidrug-resistant organisms (MDRO) are detected in urine cultures]
Yes

Are there situations or clinical findings in the patient where you deviate from your prophylaxis standard (medication, dose, duration, age of the patient)? (multiple answers possible, please specify in answer box) [if multidrug-resistant organisms (MDRO) are detected in urine cultures][Comment]

Are there situations or clinical findings in the patient where you deviate from your prophylaxis standard (medication, dose, duration, age of the patient)? (multiple answers possible, please specify in answer box) [in newborns]
No

Are there situations or clinical findings in the patient where you deviate from your prophylaxis standard (medication, dose, duration, age of the patient)? (multiple answers possible, please specify in answer box) [in newborns][Comment]

Are there situations or clinical findings in the patient where you deviate from your prophylaxis standard (medication, dose, duration, age of the patient)? (multiple answers possible, please specify in answer box) [Other]

Are there situations or clinical findings in the patient where you deviate from your prophylaxis standard (medication, dose, duration, age of the patient)? (multiple answers possible, please specify in answer box) [Other comment]

Are there situations in which you use intravenous prophylaxis?
No

Voiding analysis

Are there differences in antibiotic prophylaxis between VUS and VCUG in your department?
No

Is a non-pathological urine strip test required in preparation for VUS/VCUG in your department?
No

Is a non-pathological urine culture required in preparation for VUS/VCUG in your department?
No

Do patients only receive post-procedure prophylaxis if vesicoureteral reflux (VUR) is detected in VUS/VCUG?
Yes

Please explain your approach:
Therapeutische Gabe von AB für 3d nach Untersuchung

Do other MUS/MCU findings (e.g. urethral valves) have an influence on post-procedure prophylaxis?
No

End of survey

Thank you for taking the time to answer this survey!

May we contact you personally if we have further questions?
No

Survey response 229

Response ID
231

Date submitted
2025-04-03 11:17:46

Last page
5

Start language
de

Seed
111782990

Date started
2025-04-03 11:12:13

Date last action
2025-04-03 11:17:46

Standard Operation Procedure

Who fills out this survey?
Pediatric Surgeon

Who fills out this survey? [Other]

In which type of hospital are you employed?
Community hospital (>50 beds for pediatric patients)

In which type of hospital are you employed? [Other]

In which country do you work?
Deutschland

Does your department have a defined protocol for infection prophylaxis in the context of VUS/VCUG?
Yes

Has this protocol been transformed into a written standard operating procedure (SOP)?
No

Antibiotic Prophylaxis

Are there situations/constellations in which you do not use antibiotic prophylaxis for VUS/VCUG?
No

Please specify these situations/constellations: [girl with first uncomplicated UTI without urinary tract dilation in ultrasound]
N/A

Please specify these situations/constellations: [boy with first uncomplicated UTI without urinary tract dilation in ultrasound]
N/A

Please specify these situations/constellations: [in patients with low grade urinary tract dilation (up to 2nd grade) and no UTI in medical history]
N/A

If you use antibiotic prophylaxis: which mediaction do you use? (multiple answers and subdivision into age groups possible) [Trimethoprim]
Yes

If you use antibiotic prophylaxis: which mediaction do you use? (multiple answers and subdivision into age groups possible) [Nitrofurantoin]
No

If you use antibiotic prophylaxis: which mediaction do you use? (multiple answers and subdivision into age groups possible) [Amoxicillin]
Yes

If you use antibiotic prophylaxis: which mediaction do you use? (multiple answers and subdivision into age groups possible) [Cefaclor]
Yes

If you use antibiotic prophylaxis: which mediaction do you use? (multiple answers and subdivision into age groups possible) [Amoxicillin/clavulanic acid]
No

If you use antibiotic prophylaxis: which mediaction do you use? (multiple answers and subdivision into age groups possible) [Other]
No

Trimethoprim: in what dosage?
therapeutic (6 mg/kg bodyweight/d)

Trimethoprim: in what dosage? [Other]

Trimethoprim: for which age group do you use trimethoprim?
ab 3 Monaten

Trimethoprim: which time schedule do you use? [Day(s) before VUS/VCUG, how many?]
No

Trimethoprim: which time schedule do you use? [Day(s) before VUS/VCUG, how many?][Comment]

Trimethoprim: which time schedule do you use? [Day of VUS/VCUG]
Yes

Trimethoprim: which time schedule do you use? [Day of VUS/VCUG][Comment]

Trimethoprim: which time schedule do you use? [Day(s) after VUS/VCUG, how many?]
Yes

Trimethoprim: which time schedule do you use? [Day(s) after VUS/VCUG, how many?][Comment]
2

Nitrofurantoin: which time schedule do you use? [Day(s) before VUS/VCUG, how many?]
N/A

Nitrofurantoin: which time schedule do you use? [Day of VUS/VCUG]
N/A

Nitrofurantoin: which time schedule do you use? [Day(s) after VUS/VCUG, how many?]
N/A

Amoxicillin: in what dosage?
therapeutic (50-100 mg/kg bodyweight/d)

Amoxicillin: in what dosage? [Other]

Amoxicillin: for which age group do you use amoxicillin?
alle

Amoxicillin: which time schedule do you use? [Day(s) before VUS/VCUG, how many?]
No

Amoxicillin: which time schedule do you use? [Day(s) before VUS/VCUG, how many?][Comment]

Amoxicillin: which time schedule do you use? [Day of VUS/VCUG]
Yes

Amoxicillin: which time schedule do you use? [Day of VUS/VCUG][Comment]

Amoxicillin: which time schedule do you use? [Day(s) after VUS/VCUG, how many?]
Yes

Amoxicillin: which time schedule do you use? [Day(s) after VUS/VCUG, how many?][Comment]
2

Cefaclor: in what dosage?
therapeutic (40 mg/kg bodyweight/d)

Cefaclor: in what dosage? [Other]

Cefaclor: for which age group do you use cefaclor?
alle

Cefaclor: which time schedule do you use? [Days(s) before VUS/VCUG, how many?]
No

Cefaclor: which time schedule do you use? [Days(s) before VUS/VCUG, how many?][Comment]

Cefaclor: which time schedule do you use? [Day of VUS/VCUG]
Yes

Cefaclor: which time schedule do you use? [Day of VUS/VCUG][Comment]

Cefaclor: which time schedule do you use? [Day(s) after VUS/VCUG, how many?]
Yes

Cefaclor: which time schedule do you use? [Day(s) after VUS/VCUG, how many?][Comment]
2

Amoxicillin/clavulanic acid: in what dosage? (Amoxicillin portion) [prophylaktic (10-20 mg/Kg bodyweight/d)]
N/A

Amoxicillin/clavulanic acid: in what dosage? (Amoxicillin portion) [double prophylactic (20-40 mg/Kg bodyweight/d)]
N/A

Amoxicillin/clavulanic acid: in what dosage? (Amoxicillin portion) [therapeutic (80-100 mg/Kg bodyweight/d)]
N/A

Amoxicillin/clavulanic acid: which time schedule do you use? [Day(s) before VUS/VCUG]
N/A

Amoxicillin/clavulanic acid: which time schedule do you use? [Day of VUS/VCUG]
N/A

Amoxicillin/clavulanic acid: which time schedule do you use? [Day(s) after VUS/VCUG]
N/A

Other: which time schedule do you use? [Day(s) before VUS/VCUG, how many?]
N/A

Other: which time schedule do you use? [Day of VUS/VCUG]
N/A

Other: which time schedule do you use? [Day(s) after VUS/VCUG, how many?]
N/A

Indication for prophylaxis

Which patients receive periinterventional antibiotic prophylaxis? (multiple answers possible) [all]
No

Which patients receive periinterventional antibiotic prophylaxis? (multiple answers possible) [patients post urinary tract infection/pyelonephritis/urosepsis]
Yes

Which patients receive periinterventional antibiotic prophylaxis? (multiple answers possible) [patients with urinary tract dilation in ultrasound]
Yes

Which patients receive periinterventional antibiotic prophylaxis? (multiple answers possible) [patients with prenatal urinary tract dilation]
Yes

Which patients receive periinterventional antibiotic prophylaxis? (multiple answers possible) [patients post VUR surgery]
Yes

Which patients receive periinterventional antibiotic prophylaxis? (multiple answers possible) [none]
No

Which patients receive periinterventional antibiotic prophylaxis? (multiple answers possible) [Other]

patients status post urinary tract infection/pyelonephritis/urosepsis: what is the minimum time between infection and VUS/VCUG? (in days)
10

In patients with urinary tract dilation in ultrasound: from what degree on? Image from Beetz R, Bokenkamp A, Brandis M et al (2001) Diagnosis of congenital dilatation of the urinary tract. Consensus Group of the Pediatric Nephrology Working Society in cooperation with the Pediatric Urology Working Group of the German Society of Urology and with the Pediatric Urology Working Society in the Germany Society of Pediatric Surgery. Urologe A 40:495–507 (quiz 8–9)
grade 2

In patients with prenatal urinary tract dilation: from what degree on? Image from Beetz R, Bokenkamp A, Brandis M et al (2001) Diagnosis of congenital dilatation of the urinary tract. Consensus Group of the Pediatric Nephrology Working Society in cooperation with the Pediatric Urology Working Group of the German Society of Urology and with the Pediatric Urology Working Society in the Germany Society of Pediatric Surgery. Urologe A 40:495–507 (quiz 8–9)
grade 2

Are there situations or clinical findings in the patient where you deviate from your prophylaxis standard (medication, dose, duration, age of the patient)? (multiple answers possible, please specify in answer box) [in case of already known vesicoureteral reflux (VUR)]
No

Are there situations or clinical findings in the patient where you deviate from your prophylaxis standard (medication, dose, duration, age of the patient)? (multiple answers possible, please specify in answer box) [in case of already known vesicoureteral reflux (VUR)][Comment]

Are there situations or clinical findings in the patient where you deviate from your prophylaxis standard (medication, dose, duration, age of the patient)? (multiple answers possible, please specify in answer box) [in patients with recurrent urinary tract infections (UTI)]
Yes

Are there situations or clinical findings in the patient where you deviate from your prophylaxis standard (medication, dose, duration, age of the patient)? (multiple answers possible, please specify in answer box) [in patients with recurrent urinary tract infections (UTI)][Comment]

Are there situations or clinical findings in the patient where you deviate from your prophylaxis standard (medication, dose, duration, age of the patient)? (multiple answers possible, please specify in answer box) [if multidrug-resistant organisms (MDRO) are detected in urine cultures]
Yes

Are there situations or clinical findings in the patient where you deviate from your prophylaxis standard (medication, dose, duration, age of the patient)? (multiple answers possible, please specify in answer box) [if multidrug-resistant organisms (MDRO) are detected in urine cultures][Comment]

Are there situations or clinical findings in the patient where you deviate from your prophylaxis standard (medication, dose, duration, age of the patient)? (multiple answers possible, please specify in answer box) [in newborns]
No

Are there situations or clinical findings in the patient where you deviate from your prophylaxis standard (medication, dose, duration, age of the patient)? (multiple answers possible, please specify in answer box) [in newborns][Comment]

Are there situations or clinical findings in the patient where you deviate from your prophylaxis standard (medication, dose, duration, age of the patient)? (multiple answers possible, please specify in answer box) [Other]

Are there situations or clinical findings in the patient where you deviate from your prophylaxis standard (medication, dose, duration, age of the patient)? (multiple answers possible, please specify in answer box) [Other comment]

Are there situations in which you use intravenous prophylaxis?
Yes

In which situations?
komplexe Konstellationen

Voiding analysis

Are there differences in antibiotic prophylaxis between VUS and VCUG in your department?
No

Is a non-pathological urine strip test required in preparation for VUS/VCUG in your department?
No

Is a non-pathological urine culture required in preparation for VUS/VCUG in your department?
No

Do patients only receive post-procedure prophylaxis if vesicoureteral reflux (VUR) is detected in VUS/VCUG?
No

Do other MUS/MCU findings (e.g. urethral valves) have an influence on post-procedure prophylaxis?
No

End of survey

Thank you for taking the time to answer this survey!

May we contact you personally if we have further questions?
No

Survey response 230

Response ID
232

Last page
1

Start language
de

Seed
1944931629

Date started
2025-04-03 16:00:47

Date last action
2025-04-03 16:07:31

Standard Operation Procedure

Who fills out this survey?
Pediatric Surgeon

Who fills out this survey? [Other]

In which type of hospital are you employed?
Community hospital (>50 beds for pediatric patients)

In which type of hospital are you employed? [Other]

In which country do you work?
Deutschland

Does your department have a defined protocol for infection prophylaxis in the context of VUS/VCUG?
Yes

Has this protocol been transformed into a written standard operating procedure (SOP)?
Yes

Which department is the author of the SOP for infection prophylaxis in VUS/VCUG?
Pediatric radiology

Which department is the author of the SOP for infection prophylaxis in VUS/VCUG? [Other]

Antibiotic Prophylaxis

Are there situations/constellations in which you do not use antibiotic prophylaxis for VUS/VCUG?
Yes

Please specify these situations/constellations: [girl with first uncomplicated UTI without urinary tract dilation in ultrasound]
Yes

Please specify these situations/constellations: [boy with first uncomplicated UTI without urinary tract dilation in ultrasound]
Yes

Please specify these situations/constellations: [in patients with low grade urinary tract dilation (up to 2nd grade) and no UTI in medical history]
Yes

Please specify these situations/constellations: [Other]

If you use antibiotic prophylaxis: which mediaction do you use? (multiple answers and subdivision into age groups possible) [Trimethoprim]
No

If you use antibiotic prophylaxis: which mediaction do you use? (multiple answers and subdivision into age groups possible) [Nitrofurantoin]
No

If you use antibiotic prophylaxis: which mediaction do you use? (multiple answers and subdivision into age groups possible) [Amoxicillin]
No

If you use antibiotic prophylaxis: which mediaction do you use? (multiple answers and subdivision into age groups possible) [Cefaclor]
No

If you use antibiotic prophylaxis: which mediaction do you use? (multiple answers and subdivision into age groups possible) [Amoxicillin/clavulanic acid]
Yes

If you use antibiotic prophylaxis: which mediaction do you use? (multiple answers and subdivision into age groups possible) [Other]
No

Trimethoprim: which time schedule do you use? [Day(s) before VUS/VCUG, how many?]
N/A

Trimethoprim: which time schedule do you use? [Day of VUS/VCUG]
N/A

Trimethoprim: which time schedule do you use? [Day(s) after VUS/VCUG, how many?]
N/A

Nitrofurantoin: which time schedule do you use? [Day(s) before VUS/VCUG, how many?]
N/A

Nitrofurantoin: which time schedule do you use? [Day of VUS/VCUG]
N/A

Nitrofurantoin: which time schedule do you use? [Day(s) after VUS/VCUG, how many?]
N/A

Amoxicillin: which time schedule do you use? [Day(s) before VUS/VCUG, how many?]
N/A

Amoxicillin: which time schedule do you use? [Day of VUS/VCUG]
N/A

Amoxicillin: which time schedule do you use? [Day(s) after VUS/VCUG, how many?]
N/A

Cefaclor: which time schedule do you use? [Days(s) before VUS/VCUG, how many?]
N/A

Cefaclor: which time schedule do you use? [Day of VUS/VCUG]
N/A

Cefaclor: which time schedule do you use? [Day(s) after VUS/VCUG, how many?]
N/A

Amoxicillin/clavulanic acid: in what dosage? (Amoxicillin portion) [prophylaktic (10-20 mg/Kg bodyweight/d)]
Yes

Amoxicillin/clavulanic acid: in what dosage? (Amoxicillin portion) [double prophylactic (20-40 mg/Kg bodyweight/d)]
No

Amoxicillin/clavulanic acid: in what dosage? (Amoxicillin portion) [therapeutic (80-100 mg/Kg bodyweight/d)]
No

Amoxicillin/clavulanic acid: in what dosage? (Amoxicillin portion) [Other]

Amoxicillin/clavulanic acid: for which age group do you use amoxicillin/clavulanic acid?

Amoxicillin/clavulanic acid: which time schedule do you use? [Day(s) before VUS/VCUG]
No

Amoxicillin/clavulanic acid: which time schedule do you use? [Day(s) before VUS/VCUG][Comment]

Amoxicillin/clavulanic acid: which time schedule do you use? [Day of VUS/VCUG]
No

Amoxicillin/clavulanic acid: which time schedule do you use? [Day of VUS/VCUG][Comment]

Amoxicillin/clavulanic acid: which time schedule do you use? [Day(s) after VUS/VCUG]
No

Amoxicillin/clavulanic acid: which time schedule do you use? [Day(s) after VUS/VCUG][Comment]

Other: which time schedule do you use? [Day(s) before VUS/VCUG, how many?]
N/A

Other: which time schedule do you use? [Day of VUS/VCUG]
N/A

Other: which time schedule do you use? [Day(s) after VUS/VCUG, how many?]
N/A

Indication for prophylaxis

Which patients receive periinterventional antibiotic prophylaxis? (multiple answers possible) [all]
N/A

Which patients receive periinterventional antibiotic prophylaxis? (multiple answers possible) [patients post urinary tract infection/pyelonephritis/urosepsis]
N/A

Which patients receive periinterventional antibiotic prophylaxis? (multiple answers possible) [patients with urinary tract dilation in ultrasound]
N/A

Which patients receive periinterventional antibiotic prophylaxis? (multiple answers possible) [patients with prenatal urinary tract dilation]
N/A

Which patients receive periinterventional antibiotic prophylaxis? (multiple answers possible) [patients post VUR surgery]
N/A

Which patients receive periinterventional antibiotic prophylaxis? (multiple answers possible) [none]
N/A

Are there situations or clinical findings in the patient where you deviate from your prophylaxis standard (medication, dose, duration, age of the patient)? (multiple answers possible, please specify in answer box) [in case of already known vesicoureteral reflux (VUR)]
N/A

Are there situations or clinical findings in the patient where you deviate from your prophylaxis standard (medication, dose, duration, age of the patient)? (multiple answers possible, please specify in answer box) [in patients with recurrent urinary tract infections (UTI)]
N/A

Are there situations or clinical findings in the patient where you deviate from your prophylaxis standard (medication, dose, duration, age of the patient)? (multiple answers possible, please specify in answer box) [if multidrug-resistant organisms (MDRO) are detected in urine cultures]
N/A

Are there situations or clinical findings in the patient where you deviate from your prophylaxis standard (medication, dose, duration, age of the patient)? (multiple answers possible, please specify in answer box) [in newborns]
N/A

Are there situations in which you use intravenous prophylaxis?
N/A

Voiding analysis

Are there differences in antibiotic prophylaxis between VUS and VCUG in your department?
N/A

Is a non-pathological urine strip test required in preparation for VUS/VCUG in your department?
N/A

Is a non-pathological urine culture required in preparation for VUS/VCUG in your department?
N/A

Do patients only receive post-procedure prophylaxis if vesicoureteral reflux (VUR) is detected in VUS/VCUG?
N/A

Do other MUS/MCU findings (e.g. urethral valves) have an influence on post-procedure prophylaxis?
N/A

End of survey

May we contact you personally if we have further questions?
N/A

Survey response 231

Response ID
233

Date submitted
2025-04-04 12:56:18

Last page
5

Start language
de

Seed
406602467

Date started
2025-04-04 12:52:03

Date last action
2025-04-04 12:56:18

Standard Operation Procedure

Who fills out this survey?
Pediatric Surgeon

Who fills out this survey? [Other]

In which type of hospital are you employed?
University Hospital

In which type of hospital are you employed? [Other]

In which country do you work?
Deutschland

Does your department have a defined protocol for infection prophylaxis in the context of VUS/VCUG?
Yes

Has this protocol been transformed into a written standard operating procedure (SOP)?
No

Antibiotic Prophylaxis

Are there situations/constellations in which you do not use antibiotic prophylaxis for VUS/VCUG?
Yes

Please specify these situations/constellations: [girl with first uncomplicated UTI without urinary tract dilation in ultrasound]
No

Please specify these situations/constellations: [boy with first uncomplicated UTI without urinary tract dilation in ultrasound]
No

Please specify these situations/constellations: [in patients with low grade urinary tract dilation (up to 2nd grade) and no UTI in medical history]
No

Please specify these situations/constellations: [Other]
Bei Kindern > 1 Jahr

If you use antibiotic prophylaxis: which mediaction do you use? (multiple answers and subdivision into age groups possible) [Trimethoprim]
Yes

If you use antibiotic prophylaxis: which mediaction do you use? (multiple answers and subdivision into age groups possible) [Nitrofurantoin]
No

If you use antibiotic prophylaxis: which mediaction do you use? (multiple answers and subdivision into age groups possible) [Amoxicillin]
No

If you use antibiotic prophylaxis: which mediaction do you use? (multiple answers and subdivision into age groups possible) [Cefaclor]
No

If you use antibiotic prophylaxis: which mediaction do you use? (multiple answers and subdivision into age groups possible) [Amoxicillin/clavulanic acid]
No

If you use antibiotic prophylaxis: which mediaction do you use? (multiple answers and subdivision into age groups possible) [Other]
No

Trimethoprim: in what dosage?
therapeutic (6 mg/kg bodyweight/d)

Trimethoprim: in what dosage? [Other]

Trimethoprim: for which age group do you use trimethoprim?
> 6 Wochen

Trimethoprim: which time schedule do you use? [Day(s) before VUS/VCUG, how many?]
Yes

Trimethoprim: which time schedule do you use? [Day(s) before VUS/VCUG, how many?][Comment]
1

Trimethoprim: which time schedule do you use? [Day of VUS/VCUG]
Yes

Trimethoprim: which time schedule do you use? [Day of VUS/VCUG][Comment]

Trimethoprim: which time schedule do you use? [Day(s) after VUS/VCUG, how many?]
No

Trimethoprim: which time schedule do you use? [Day(s) after VUS/VCUG, how many?][Comment]

Nitrofurantoin: which time schedule do you use? [Day(s) before VUS/VCUG, how many?]
N/A

Nitrofurantoin: which time schedule do you use? [Day of VUS/VCUG]
N/A

Nitrofurantoin: which time schedule do you use? [Day(s) after VUS/VCUG, how many?]
N/A

Amoxicillin: which time schedule do you use? [Day(s) before VUS/VCUG, how many?]
N/A

Amoxicillin: which time schedule do you use? [Day of VUS/VCUG]
N/A

Amoxicillin: which time schedule do you use? [Day(s) after VUS/VCUG, how many?]
N/A

Cefaclor: which time schedule do you use? [Days(s) before VUS/VCUG, how many?]
N/A

Cefaclor: which time schedule do you use? [Day of VUS/VCUG]
N/A

Cefaclor: which time schedule do you use? [Day(s) after VUS/VCUG, how many?]
N/A

Amoxicillin/clavulanic acid: in what dosage? (Amoxicillin portion) [prophylaktic (10-20 mg/Kg bodyweight/d)]
N/A

Amoxicillin/clavulanic acid: in what dosage? (Amoxicillin portion) [double prophylactic (20-40 mg/Kg bodyweight/d)]
N/A

Amoxicillin/clavulanic acid: in what dosage? (Amoxicillin portion) [therapeutic (80-100 mg/Kg bodyweight/d)]
N/A

Amoxicillin/clavulanic acid: which time schedule do you use? [Day(s) before VUS/VCUG]
N/A

Amoxicillin/clavulanic acid: which time schedule do you use? [Day of VUS/VCUG]
N/A

Amoxicillin/clavulanic acid: which time schedule do you use? [Day(s) after VUS/VCUG]
N/A

Other: which time schedule do you use? [Day(s) before VUS/VCUG, how many?]
N/A

Other: which time schedule do you use? [Day of VUS/VCUG]
N/A

Other: which time schedule do you use? [Day(s) after VUS/VCUG, how many?]
N/A

Indication for prophylaxis

Which patients receive periinterventional antibiotic prophylaxis? (multiple answers possible) [all]
No

Which patients receive periinterventional antibiotic prophylaxis? (multiple answers possible) [patients post urinary tract infection/pyelonephritis/urosepsis]
No

Which patients receive periinterventional antibiotic prophylaxis? (multiple answers possible) [patients with urinary tract dilation in ultrasound]
No

Which patients receive periinterventional antibiotic prophylaxis? (multiple answers possible) [patients with prenatal urinary tract dilation]
No

Which patients receive periinterventional antibiotic prophylaxis? (multiple answers possible) [patients post VUR surgery]
No

Which patients receive periinterventional antibiotic prophylaxis? (multiple answers possible) [none]
No

Which patients receive periinterventional antibiotic prophylaxis? (multiple answers possible) [Other]
alle < 1 Jahr

Are there situations or clinical findings in the patient where you deviate from your prophylaxis standard (medication, dose, duration, age of the patient)? (multiple answers possible, please specify in answer box) [in case of already known vesicoureteral reflux (VUR)]
No

Are there situations or clinical findings in the patient where you deviate from your prophylaxis standard (medication, dose, duration, age of the patient)? (multiple answers possible, please specify in answer box) [in case of already known vesicoureteral reflux (VUR)][Comment]

Are there situations or clinical findings in the patient where you deviate from your prophylaxis standard (medication, dose, duration, age of the patient)? (multiple answers possible, please specify in answer box) [in patients with recurrent urinary tract infections (UTI)]
No

Are there situations or clinical findings in the patient where you deviate from your prophylaxis standard (medication, dose, duration, age of the patient)? (multiple answers possible, please specify in answer box) [in patients with recurrent urinary tract infections (UTI)][Comment]

Are there situations or clinical findings in the patient where you deviate from your prophylaxis standard (medication, dose, duration, age of the patient)? (multiple answers possible, please specify in answer box) [if multidrug-resistant organisms (MDRO) are detected in urine cultures]
Yes

Are there situations or clinical findings in the patient where you deviate from your prophylaxis standard (medication, dose, duration, age of the patient)? (multiple answers possible, please specify in answer box) [if multidrug-resistant organisms (MDRO) are detected in urine cultures][Comment]

Are there situations or clinical findings in the patient where you deviate from your prophylaxis standard (medication, dose, duration, age of the patient)? (multiple answers possible, please specify in answer box) [in newborns]
No

Are there situations or clinical findings in the patient where you deviate from your prophylaxis standard (medication, dose, duration, age of the patient)? (multiple answers possible, please specify in answer box) [in newborns][Comment]

Are there situations or clinical findings in the patient where you deviate from your prophylaxis standard (medication, dose, duration, age of the patient)? (multiple answers possible, please specify in answer box) [Other]

Are there situations or clinical findings in the patient where you deviate from your prophylaxis standard (medication, dose, duration, age of the patient)? (multiple answers possible, please specify in answer box) [Other comment]

Are there situations in which you use intravenous prophylaxis?
Yes

In which situations?
Wenn Kind eh schon PVK liegen hat

Voiding analysis

Are there differences in antibiotic prophylaxis between VUS and VCUG in your department?
No

Is a non-pathological urine strip test required in preparation for VUS/VCUG in your department?
Yes

What is the maximum amount of days accepted for urine strip test to be performed prior to the exam?
Bei Katheteranlage

Is a non-pathological urine culture required in preparation for VUS/VCUG in your department?
No

Do patients only receive post-procedure prophylaxis if vesicoureteral reflux (VUR) is detected in VUS/VCUG?
No

Do other MUS/MCU findings (e.g. urethral valves) have an influence on post-procedure prophylaxis?
No

End of survey

Thank you for taking the time to answer this survey!

May we contact you personally if we have further questions?
No

Survey response 232

Response ID
234

Date submitted
2025-04-06 10:14:40

Last page
5

Start language
en

Seed
940602217

Date started
2025-04-06 09:59:26

Date last action
2025-04-06 10:14:40

Standard Operation Procedure

Who fills out this survey?
Pediatric Radiologist

Who fills out this survey? [Other]

In which type of hospital are you employed?
University Hospital

In which type of hospital are you employed? [Other]

In which country do you work?
GREECE

Does your department have a defined protocol for infection prophylaxis in the context of VUS/VCUG?
Yes

Has this protocol been transformed into a written standard operating procedure (SOP)?
Yes

Which department is the author of the SOP for infection prophylaxis in VUS/VCUG?
Pediatric radiology

Which department is the author of the SOP for infection prophylaxis in VUS/VCUG? [Other]

Antibiotic Prophylaxis

Are there situations/constellations in which you do not use antibiotic prophylaxis for VUS/VCUG?
No

Please specify these situations/constellations: [girl with first uncomplicated UTI without urinary tract dilation in ultrasound]
N/A

Please specify these situations/constellations: [boy with first uncomplicated UTI without urinary tract dilation in ultrasound]
N/A

Please specify these situations/constellations: [in patients with low grade urinary tract dilation (up to 2nd grade) and no UTI in medical history]
N/A

If you use antibiotic prophylaxis: which mediaction do you use? (multiple answers and subdivision into age groups possible) [Trimethoprim]
No

If you use antibiotic prophylaxis: which mediaction do you use? (multiple answers and subdivision into age groups possible) [Nitrofurantoin]
No

If you use antibiotic prophylaxis: which mediaction do you use? (multiple answers and subdivision into age groups possible) [Amoxicillin]
No

If you use antibiotic prophylaxis: which mediaction do you use? (multiple answers and subdivision into age groups possible) [Cefaclor]
No

If you use antibiotic prophylaxis: which mediaction do you use? (multiple answers and subdivision into age groups possible) [Amoxicillin/clavulanic acid]
No

If you use antibiotic prophylaxis: which mediaction do you use? (multiple answers and subdivision into age groups possible) [Other]
No

Trimethoprim: which time schedule do you use? [Day(s) before VUS/VCUG, how many?]
N/A

Trimethoprim: which time schedule do you use? [Day of VUS/VCUG]
N/A

Trimethoprim: which time schedule do you use? [Day(s) after VUS/VCUG, how many?]
N/A

Nitrofurantoin: which time schedule do you use? [Day(s) before VUS/VCUG, how many?]
N/A

Nitrofurantoin: which time schedule do you use? [Day of VUS/VCUG]
N/A

Nitrofurantoin: which time schedule do you use? [Day(s) after VUS/VCUG, how many?]
N/A

Amoxicillin: which time schedule do you use? [Day(s) before VUS/VCUG, how many?]
N/A

Amoxicillin: which time schedule do you use? [Day of VUS/VCUG]
N/A

Amoxicillin: which time schedule do you use? [Day(s) after VUS/VCUG, how many?]
N/A

Cefaclor: which time schedule do you use? [Days(s) before VUS/VCUG, how many?]
N/A

Cefaclor: which time schedule do you use? [Day of VUS/VCUG]
N/A

Cefaclor: which time schedule do you use? [Day(s) after VUS/VCUG, how many?]
N/A

Amoxicillin/clavulanic acid: in what dosage? (Amoxicillin portion) [prophylaktic (10-20 mg/Kg bodyweight/d)]
N/A

Amoxicillin/clavulanic acid: in what dosage? (Amoxicillin portion) [double prophylactic (20-40 mg/Kg bodyweight/d)]
N/A

Amoxicillin/clavulanic acid: in what dosage? (Amoxicillin portion) [therapeutic (80-100 mg/Kg bodyweight/d)]
N/A

Amoxicillin/clavulanic acid: which time schedule do you use? [Day(s) before VUS/VCUG]
N/A

Amoxicillin/clavulanic acid: which time schedule do you use? [Day of VUS/VCUG]
N/A

Amoxicillin/clavulanic acid: which time schedule do you use? [Day(s) after VUS/VCUG]
N/A

Other: which time schedule do you use? [Day(s) before VUS/VCUG, how many?]
N/A

Other: which time schedule do you use? [Day of VUS/VCUG]
N/A

Other: which time schedule do you use? [Day(s) after VUS/VCUG, how many?]
N/A

Indication for prophylaxis

Which patients receive periinterventional antibiotic prophylaxis? (multiple answers possible) [all]
Yes

Which patients receive periinterventional antibiotic prophylaxis? (multiple answers possible) [patients post urinary tract infection/pyelonephritis/urosepsis]
N/A

Which patients receive periinterventional antibiotic prophylaxis? (multiple answers possible) [patients with urinary tract dilation in ultrasound]
N/A

Which patients receive periinterventional antibiotic prophylaxis? (multiple answers possible) [patients with prenatal urinary tract dilation]
N/A

Which patients receive periinterventional antibiotic prophylaxis? (multiple answers possible) [patients post VUR surgery]
N/A

Which patients receive periinterventional antibiotic prophylaxis? (multiple answers possible) [none]
N/A

Are there situations or clinical findings in the patient where you deviate from your prophylaxis standard (medication, dose, duration, age of the patient)? (multiple answers possible, please specify in answer box) [in case of already known vesicoureteral reflux (VUR)]
Yes

Are there situations or clinical findings in the patient where you deviate from your prophylaxis standard (medication, dose, duration, age of the patient)? (multiple answers possible, please specify in answer box) [in case of already known vesicoureteral reflux (VUR)][Comment]
If they already take antibiotic prophylaxis for their VUR, they continue with their medication but with theurapeutic dose according to their paediatrician

Are there situations or clinical findings in the patient where you deviate from your prophylaxis standard (medication, dose, duration, age of the patient)? (multiple answers possible, please specify in answer box) [in patients with recurrent urinary tract infections (UTI)]
No

Are there situations or clinical findings in the patient where you deviate from your prophylaxis standard (medication, dose, duration, age of the patient)? (multiple answers possible, please specify in answer box) [in patients with recurrent urinary tract infections (UTI)][Comment]

Are there situations or clinical findings in the patient where you deviate from your prophylaxis standard (medication, dose, duration, age of the patient)? (multiple answers possible, please specify in answer box) [if multidrug-resistant organisms (MDRO) are detected in urine cultures]
Yes

Are there situations or clinical findings in the patient where you deviate from your prophylaxis standard (medication, dose, duration, age of the patient)? (multiple answers possible, please specify in answer box) [if multidrug-resistant organisms (MDRO) are detected in urine cultures][Comment]
the paediatrician decides for the kind of antibiotic but in theurapeutic dose

Are there situations or clinical findings in the patient where you deviate from your prophylaxis standard (medication, dose, duration, age of the patient)? (multiple answers possible, please specify in answer box) [in newborns]
No

Are there situations or clinical findings in the patient where you deviate from your prophylaxis standard (medication, dose, duration, age of the patient)? (multiple answers possible, please specify in answer box) [in newborns][Comment]

Are there situations or clinical findings in the patient where you deviate from your prophylaxis standard (medication, dose, duration, age of the patient)? (multiple answers possible, please specify in answer box) [Other]

Are there situations or clinical findings in the patient where you deviate from your prophylaxis standard (medication, dose, duration, age of the patient)? (multiple answers possible, please specify in answer box) [Other comment]

Are there situations in which you use intravenous prophylaxis?
No

Voiding analysis

Are there differences in antibiotic prophylaxis between VUS and VCUG in your department?
No

Is a non-pathological urine strip test required in preparation for VUS/VCUG in your department?
No

Is a non-pathological urine culture required in preparation for VUS/VCUG in your department?
Yes

What is the maximum amount of days accepted for urine culture to be performed prior to the exam?
4-5 days, because 2 days needed for the results of the culture and then 2 days theurapeutic dose of antibiotic before the VUS/VCUG and then is the day of the VUS/VCUG

Do patients only receive post-procedure prophylaxis if vesicoureteral reflux (VUR) is detected in VUS/VCUG?
No

Do other MUS/MCU findings (e.g. urethral valves) have an influence on post-procedure prophylaxis?
No

End of survey

Thank you for taking the time to answer this survey!

May we contact you personally if we have further questions?
Yes

E-mail address:
marvakaki@yahoo.gr (I have not written that we continue antibiotic in theraupetic dose 2 days after VUS/VCUG)

Survey response 233

Response ID
235

Date submitted
2025-04-06 18:15:16

Last page
5

Start language
en

Seed
787912782

Date started
2025-04-06 18:12:17

Date last action
2025-04-06 18:15:16

Standard Operation Procedure

Who fills out this survey?
Pediatric Radiologist

Who fills out this survey? [Other]

In which type of hospital are you employed?
University Hospital

In which type of hospital are you employed? [Other]

In which country do you work?
England

Does your department have a defined protocol for infection prophylaxis in the context of VUS/VCUG?
Yes

Has this protocol been transformed into a written standard operating procedure (SOP)?
Yes

Which department is the author of the SOP for infection prophylaxis in VUS/VCUG?
Pediatric radiology

Which department is the author of the SOP for infection prophylaxis in VUS/VCUG? [Other]

Antibiotic Prophylaxis

Are there situations/constellations in which you do not use antibiotic prophylaxis for VUS/VCUG?
No

Please specify these situations/constellations: [girl with first uncomplicated UTI without urinary tract dilation in ultrasound]
N/A

Please specify these situations/constellations: [boy with first uncomplicated UTI without urinary tract dilation in ultrasound]
N/A

Please specify these situations/constellations: [in patients with low grade urinary tract dilation (up to 2nd grade) and no UTI in medical history]
N/A

If you use antibiotic prophylaxis: which mediaction do you use? (multiple answers and subdivision into age groups possible) [Trimethoprim]
Yes

If you use antibiotic prophylaxis: which mediaction do you use? (multiple answers and subdivision into age groups possible) [Nitrofurantoin]
No

If you use antibiotic prophylaxis: which mediaction do you use? (multiple answers and subdivision into age groups possible) [Amoxicillin]
No

If you use antibiotic prophylaxis: which mediaction do you use? (multiple answers and subdivision into age groups possible) [Cefaclor]
No

If you use antibiotic prophylaxis: which mediaction do you use? (multiple answers and subdivision into age groups possible) [Amoxicillin/clavulanic acid]
No

If you use antibiotic prophylaxis: which mediaction do you use? (multiple answers and subdivision into age groups possible) [Other]
No

Trimethoprim: in what dosage?
double prophylakctic (4 mg/kg bodyweight/d)

Trimethoprim: in what dosage? [Other]

Trimethoprim: for which age group do you use trimethoprim?
0-12months

Trimethoprim: which time schedule do you use? [Day(s) before VUS/VCUG, how many?]
Yes

Trimethoprim: which time schedule do you use? [Day(s) before VUS/VCUG, how many?][Comment]
1

Trimethoprim: which time schedule do you use? [Day of VUS/VCUG]
Yes

Trimethoprim: which time schedule do you use? [Day of VUS/VCUG][Comment]

Trimethoprim: which time schedule do you use? [Day(s) after VUS/VCUG, how many?]
Yes

Trimethoprim: which time schedule do you use? [Day(s) after VUS/VCUG, how many?][Comment]
1

Nitrofurantoin: which time schedule do you use? [Day(s) before VUS/VCUG, how many?]
N/A

Nitrofurantoin: which time schedule do you use? [Day of VUS/VCUG]
N/A

Nitrofurantoin: which time schedule do you use? [Day(s) after VUS/VCUG, how many?]
N/A

Amoxicillin: which time schedule do you use? [Day(s) before VUS/VCUG, how many?]
N/A

Amoxicillin: which time schedule do you use? [Day of VUS/VCUG]
N/A

Amoxicillin: which time schedule do you use? [Day(s) after VUS/VCUG, how many?]
N/A

Cefaclor: which time schedule do you use? [Days(s) before VUS/VCUG, how many?]
N/A

Cefaclor: which time schedule do you use? [Day of VUS/VCUG]
N/A

Cefaclor: which time schedule do you use? [Day(s) after VUS/VCUG, how many?]
N/A

Amoxicillin/clavulanic acid: in what dosage? (Amoxicillin portion) [prophylaktic (10-20 mg/Kg bodyweight/d)]
N/A

Amoxicillin/clavulanic acid: in what dosage? (Amoxicillin portion) [double prophylactic (20-40 mg/Kg bodyweight/d)]
N/A

Amoxicillin/clavulanic acid: in what dosage? (Amoxicillin portion) [therapeutic (80-100 mg/Kg bodyweight/d)]
N/A

Amoxicillin/clavulanic acid: which time schedule do you use? [Day(s) before VUS/VCUG]
N/A

Amoxicillin/clavulanic acid: which time schedule do you use? [Day of VUS/VCUG]
N/A

Amoxicillin/clavulanic acid: which time schedule do you use? [Day(s) after VUS/VCUG]
N/A

Other: which time schedule do you use? [Day(s) before VUS/VCUG, how many?]
N/A

Other: which time schedule do you use? [Day of VUS/VCUG]
N/A

Other: which time schedule do you use? [Day(s) after VUS/VCUG, how many?]
N/A

Indication for prophylaxis

Which patients receive periinterventional antibiotic prophylaxis? (multiple answers possible) [all]
Yes

Which patients receive periinterventional antibiotic prophylaxis? (multiple answers possible) [patients post urinary tract infection/pyelonephritis/urosepsis]
N/A

Which patients receive periinterventional antibiotic prophylaxis? (multiple answers possible) [patients with urinary tract dilation in ultrasound]
N/A

Which patients receive periinterventional antibiotic prophylaxis? (multiple answers possible) [patients with prenatal urinary tract dilation]
N/A

Which patients receive periinterventional antibiotic prophylaxis? (multiple answers possible) [patients post VUR surgery]
N/A

Which patients receive periinterventional antibiotic prophylaxis? (multiple answers possible) [none]
N/A

Are there situations or clinical findings in the patient where you deviate from your prophylaxis standard (medication, dose, duration, age of the patient)? (multiple answers possible, please specify in answer box) [in case of already known vesicoureteral reflux (VUR)]
No

Are there situations or clinical findings in the patient where you deviate from your prophylaxis standard (medication, dose, duration, age of the patient)? (multiple answers possible, please specify in answer box) [in case of already known vesicoureteral reflux (VUR)][Comment]

Are there situations or clinical findings in the patient where you deviate from your prophylaxis standard (medication, dose, duration, age of the patient)? (multiple answers possible, please specify in answer box) [in patients with recurrent urinary tract infections (UTI)]
No

Are there situations or clinical findings in the patient where you deviate from your prophylaxis standard (medication, dose, duration, age of the patient)? (multiple answers possible, please specify in answer box) [in patients with recurrent urinary tract infections (UTI)][Comment]

Are there situations or clinical findings in the patient where you deviate from your prophylaxis standard (medication, dose, duration, age of the patient)? (multiple answers possible, please specify in answer box) [if multidrug-resistant organisms (MDRO) are detected in urine cultures]
No

Are there situations or clinical findings in the patient where you deviate from your prophylaxis standard (medication, dose, duration, age of the patient)? (multiple answers possible, please specify in answer box) [if multidrug-resistant organisms (MDRO) are detected in urine cultures][Comment]

Are there situations or clinical findings in the patient where you deviate from your prophylaxis standard (medication, dose, duration, age of the patient)? (multiple answers possible, please specify in answer box) [in newborns]
No

Are there situations or clinical findings in the patient where you deviate from your prophylaxis standard (medication, dose, duration, age of the patient)? (multiple answers possible, please specify in answer box) [in newborns][Comment]

Are there situations or clinical findings in the patient where you deviate from your prophylaxis standard (medication, dose, duration, age of the patient)? (multiple answers possible, please specify in answer box) [Other]
no

Are there situations or clinical findings in the patient where you deviate from your prophylaxis standard (medication, dose, duration, age of the patient)? (multiple answers possible, please specify in answer box) [Other comment]

Are there situations in which you use intravenous prophylaxis?
No

Voiding analysis

Are there differences in antibiotic prophylaxis between VUS and VCUG in your department?
No

Is a non-pathological urine strip test required in preparation for VUS/VCUG in your department?
No

Is a non-pathological urine culture required in preparation for VUS/VCUG in your department?
No

Do patients only receive post-procedure prophylaxis if vesicoureteral reflux (VUR) is detected in VUS/VCUG?
No

Do other MUS/MCU findings (e.g. urethral valves) have an influence on post-procedure prophylaxis?
No

End of survey

Thank you for taking the time to answer this survey!

May we contact you personally if we have further questions?
No

Survey response 234

Response ID
236

Last page
1

Start language
de

Seed
1158135605

Date started
2025-04-07 06:43:55

Date last action
2025-04-07 06:45:46

Standard Operation Procedure

Who fills out this survey?
Pediatric Surgeon

Who fills out this survey? [Other]

In which type of hospital are you employed?
University Hospital

In which type of hospital are you employed? [Other]

In which country do you work?
Deutschland

Does your department have a defined protocol for infection prophylaxis in the context of VUS/VCUG?
No

Has this protocol been transformed into a written standard operating procedure (SOP)?
N/A

Antibiotic Prophylaxis

Are there situations/constellations in which you do not use antibiotic prophylaxis for VUS/VCUG?
Yes

Please specify these situations/constellations: [girl with first uncomplicated UTI without urinary tract dilation in ultrasound]
Yes

Please specify these situations/constellations: [boy with first uncomplicated UTI without urinary tract dilation in ultrasound]
Yes

Please specify these situations/constellations: [in patients with low grade urinary tract dilation (up to 2nd grade) and no UTI in medical history]
Yes

Please specify these situations/constellations: [Other]

If you use antibiotic prophylaxis: which mediaction do you use? (multiple answers and subdivision into age groups possible) [Trimethoprim]
Yes

If you use antibiotic prophylaxis: which mediaction do you use? (multiple answers and subdivision into age groups possible) [Nitrofurantoin]
No

If you use antibiotic prophylaxis: which mediaction do you use? (multiple answers and subdivision into age groups possible) [Amoxicillin]
No

If you use antibiotic prophylaxis: which mediaction do you use? (multiple answers and subdivision into age groups possible) [Cefaclor]
No

If you use antibiotic prophylaxis: which mediaction do you use? (multiple answers and subdivision into age groups possible) [Amoxicillin/clavulanic acid]
No

If you use antibiotic prophylaxis: which mediaction do you use? (multiple answers and subdivision into age groups possible) [Other]
No

Trimethoprim: in what dosage?
prophylactic (2 mg/kg bodyweight/d)

Trimethoprim: in what dosage? [Other]

Trimethoprim: for which age group do you use trimethoprim?

Trimethoprim: which time schedule do you use? [Day(s) before VUS/VCUG, how many?]
Yes

Trimethoprim: which time schedule do you use? [Day(s) before VUS/VCUG, how many?][Comment]

Trimethoprim: which time schedule do you use? [Day of VUS/VCUG]
No

Trimethoprim: which time schedule do you use? [Day of VUS/VCUG][Comment]

Trimethoprim: which time schedule do you use? [Day(s) after VUS/VCUG, how many?]
No

Trimethoprim: which time schedule do you use? [Day(s) after VUS/VCUG, how many?][Comment]

Nitrofurantoin: which time schedule do you use? [Day(s) before VUS/VCUG, how many?]
N/A

Nitrofurantoin: which time schedule do you use? [Day of VUS/VCUG]
N/A

Nitrofurantoin: which time schedule do you use? [Day(s) after VUS/VCUG, how many?]
N/A

Amoxicillin: which time schedule do you use? [Day(s) before VUS/VCUG, how many?]
N/A

Amoxicillin: which time schedule do you use? [Day of VUS/VCUG]
N/A

Amoxicillin: which time schedule do you use? [Day(s) after VUS/VCUG, how many?]
N/A

Cefaclor: which time schedule do you use? [Days(s) before VUS/VCUG, how many?]
N/A

Cefaclor: which time schedule do you use? [Day of VUS/VCUG]
N/A

Cefaclor: which time schedule do you use? [Day(s) after VUS/VCUG, how many?]
N/A

Amoxicillin/clavulanic acid: in what dosage? (Amoxicillin portion) [prophylaktic (10-20 mg/Kg bodyweight/d)]
N/A

Amoxicillin/clavulanic acid: in what dosage? (Amoxicillin portion) [double prophylactic (20-40 mg/Kg bodyweight/d)]
N/A

Amoxicillin/clavulanic acid: in what dosage? (Amoxicillin portion) [therapeutic (80-100 mg/Kg bodyweight/d)]
N/A

Amoxicillin/clavulanic acid: which time schedule do you use? [Day(s) before VUS/VCUG]
N/A

Amoxicillin/clavulanic acid: which time schedule do you use? [Day of VUS/VCUG]
N/A

Amoxicillin/clavulanic acid: which time schedule do you use? [Day(s) after VUS/VCUG]
N/A

Other: which time schedule do you use? [Day(s) before VUS/VCUG, how many?]
N/A

Other: which time schedule do you use? [Day of VUS/VCUG]
N/A

Other: which time schedule do you use? [Day(s) after VUS/VCUG, how many?]
N/A

Indication for prophylaxis

Which patients receive periinterventional antibiotic prophylaxis? (multiple answers possible) [all]
N/A

Which patients receive periinterventional antibiotic prophylaxis? (multiple answers possible) [patients post urinary tract infection/pyelonephritis/urosepsis]
N/A

Which patients receive periinterventional antibiotic prophylaxis? (multiple answers possible) [patients with urinary tract dilation in ultrasound]
N/A

Which patients receive periinterventional antibiotic prophylaxis? (multiple answers possible) [patients with prenatal urinary tract dilation]
N/A

Which patients receive periinterventional antibiotic prophylaxis? (multiple answers possible) [patients post VUR surgery]
N/A

Which patients receive periinterventional antibiotic prophylaxis? (multiple answers possible) [none]
N/A

Are there situations or clinical findings in the patient where you deviate from your prophylaxis standard (medication, dose, duration, age of the patient)? (multiple answers possible, please specify in answer box) [in case of already known vesicoureteral reflux (VUR)]
N/A

Are there situations or clinical findings in the patient where you deviate from your prophylaxis standard (medication, dose, duration, age of the patient)? (multiple answers possible, please specify in answer box) [in patients with recurrent urinary tract infections (UTI)]
N/A

Are there situations or clinical findings in the patient where you deviate from your prophylaxis standard (medication, dose, duration, age of the patient)? (multiple answers possible, please specify in answer box) [if multidrug-resistant organisms (MDRO) are detected in urine cultures]
N/A

Are there situations or clinical findings in the patient where you deviate from your prophylaxis standard (medication, dose, duration, age of the patient)? (multiple answers possible, please specify in answer box) [in newborns]
N/A

Are there situations in which you use intravenous prophylaxis?
N/A

Voiding analysis

Are there differences in antibiotic prophylaxis between VUS and VCUG in your department?
N/A

Is a non-pathological urine strip test required in preparation for VUS/VCUG in your department?
N/A

Is a non-pathological urine culture required in preparation for VUS/VCUG in your department?
N/A

Do patients only receive post-procedure prophylaxis if vesicoureteral reflux (VUR) is detected in VUS/VCUG?
N/A

Do other MUS/MCU findings (e.g. urethral valves) have an influence on post-procedure prophylaxis?
N/A

End of survey

May we contact you personally if we have further questions?
N/A

Survey response 235

Response ID
237

Last page
0

Start language
en

Seed
1499580516

Date started
2025-04-07 17:18:20

Date last action
2025-04-07 17:18:20

Standard Operation Procedure

Does your department have a defined protocol for infection prophylaxis in the context of VUS/VCUG?
N/A

Has this protocol been transformed into a written standard operating procedure (SOP)?
N/A

Antibiotic Prophylaxis

Are there situations/constellations in which you do not use antibiotic prophylaxis for VUS/VCUG?
N/A

Please specify these situations/constellations: [girl with first uncomplicated UTI without urinary tract dilation in ultrasound]
N/A

Please specify these situations/constellations: [boy with first uncomplicated UTI without urinary tract dilation in ultrasound]
N/A

Please specify these situations/constellations: [in patients with low grade urinary tract dilation (up to 2nd grade) and no UTI in medical history]
N/A

If you use antibiotic prophylaxis: which mediaction do you use? (multiple answers and subdivision into age groups possible) [Trimethoprim]
N/A

If you use antibiotic prophylaxis: which mediaction do you use? (multiple answers and subdivision into age groups possible) [Nitrofurantoin]
N/A

If you use antibiotic prophylaxis: which mediaction do you use? (multiple answers and subdivision into age groups possible) [Amoxicillin]
N/A

If you use antibiotic prophylaxis: which mediaction do you use? (multiple answers and subdivision into age groups possible) [Cefaclor]
N/A

If you use antibiotic prophylaxis: which mediaction do you use? (multiple answers and subdivision into age groups possible) [Amoxicillin/clavulanic acid]
N/A

If you use antibiotic prophylaxis: which mediaction do you use? (multiple answers and subdivision into age groups possible) [Other]
N/A

Trimethoprim: which time schedule do you use? [Day(s) before VUS/VCUG, how many?]
N/A

Trimethoprim: which time schedule do you use? [Day of VUS/VCUG]
N/A

Trimethoprim: which time schedule do you use? [Day(s) after VUS/VCUG, how many?]
N/A

Nitrofurantoin: which time schedule do you use? [Day(s) before VUS/VCUG, how many?]
N/A

Nitrofurantoin: which time schedule do you use? [Day of VUS/VCUG]
N/A

Nitrofurantoin: which time schedule do you use? [Day(s) after VUS/VCUG, how many?]
N/A

Amoxicillin: which time schedule do you use? [Day(s) before VUS/VCUG, how many?]
N/A

Amoxicillin: which time schedule do you use? [Day of VUS/VCUG]
N/A

Amoxicillin: which time schedule do you use? [Day(s) after VUS/VCUG, how many?]
N/A

Cefaclor: which time schedule do you use? [Days(s) before VUS/VCUG, how many?]
N/A

Cefaclor: which time schedule do you use? [Day of VUS/VCUG]
N/A

Cefaclor: which time schedule do you use? [Day(s) after VUS/VCUG, how many?]
N/A

Amoxicillin/clavulanic acid: in what dosage? (Amoxicillin portion) [prophylaktic (10-20 mg/Kg bodyweight/d)]
N/A

Amoxicillin/clavulanic acid: in what dosage? (Amoxicillin portion) [double prophylactic (20-40 mg/Kg bodyweight/d)]
N/A

Amoxicillin/clavulanic acid: in what dosage? (Amoxicillin portion) [therapeutic (80-100 mg/Kg bodyweight/d)]
N/A

Amoxicillin/clavulanic acid: which time schedule do you use? [Day(s) before VUS/VCUG]
N/A

Amoxicillin/clavulanic acid: which time schedule do you use? [Day of VUS/VCUG]
N/A

Amoxicillin/clavulanic acid: which time schedule do you use? [Day(s) after VUS/VCUG]
N/A

Other: which time schedule do you use? [Day(s) before VUS/VCUG, how many?]
N/A

Other: which time schedule do you use? [Day of VUS/VCUG]
N/A

Other: which time schedule do you use? [Day(s) after VUS/VCUG, how many?]
N/A

Indication for prophylaxis

Which patients receive periinterventional antibiotic prophylaxis? (multiple answers possible) [all]
N/A

Which patients receive periinterventional antibiotic prophylaxis? (multiple answers possible) [patients post urinary tract infection/pyelonephritis/urosepsis]
N/A

Which patients receive periinterventional antibiotic prophylaxis? (multiple answers possible) [patients with urinary tract dilation in ultrasound]
N/A

Which patients receive periinterventional antibiotic prophylaxis? (multiple answers possible) [patients with prenatal urinary tract dilation]
N/A

Which patients receive periinterventional antibiotic prophylaxis? (multiple answers possible) [patients post VUR surgery]
N/A

Which patients receive periinterventional antibiotic prophylaxis? (multiple answers possible) [none]
N/A

Are there situations or clinical findings in the patient where you deviate from your prophylaxis standard (medication, dose, duration, age of the patient)? (multiple answers possible, please specify in answer box) [in case of already known vesicoureteral reflux (VUR)]
N/A

Are there situations or clinical findings in the patient where you deviate from your prophylaxis standard (medication, dose, duration, age of the patient)? (multiple answers possible, please specify in answer box) [in patients with recurrent urinary tract infections (UTI)]
N/A

Are there situations or clinical findings in the patient where you deviate from your prophylaxis standard (medication, dose, duration, age of the patient)? (multiple answers possible, please specify in answer box) [if multidrug-resistant organisms (MDRO) are detected in urine cultures]
N/A

Are there situations or clinical findings in the patient where you deviate from your prophylaxis standard (medication, dose, duration, age of the patient)? (multiple answers possible, please specify in answer box) [in newborns]
N/A

Are there situations in which you use intravenous prophylaxis?
N/A

Voiding analysis

Are there differences in antibiotic prophylaxis between VUS and VCUG in your department?
N/A

Is a non-pathological urine strip test required in preparation for VUS/VCUG in your department?
N/A

Is a non-pathological urine culture required in preparation for VUS/VCUG in your department?
N/A

Do patients only receive post-procedure prophylaxis if vesicoureteral reflux (VUR) is detected in VUS/VCUG?
N/A

Do other MUS/MCU findings (e.g. urethral valves) have an influence on post-procedure prophylaxis?
N/A

End of survey

May we contact you personally if we have further questions?
N/A

Survey response 236

Response ID
238

Last page
0

Start language
de

Seed
1751433302

Date started
2025-04-08 07:13:33

Date last action
2025-04-08 07:13:33

Standard Operation Procedure

Does your department have a defined protocol for infection prophylaxis in the context of VUS/VCUG?
N/A

Has this protocol been transformed into a written standard operating procedure (SOP)?
N/A

Antibiotic Prophylaxis

Are there situations/constellations in which you do not use antibiotic prophylaxis for VUS/VCUG?
N/A

Please specify these situations/constellations: [girl with first uncomplicated UTI without urinary tract dilation in ultrasound]
N/A

Please specify these situations/constellations: [boy with first uncomplicated UTI without urinary tract dilation in ultrasound]
N/A

Please specify these situations/constellations: [in patients with low grade urinary tract dilation (up to 2nd grade) and no UTI in medical history]
N/A

If you use antibiotic prophylaxis: which mediaction do you use? (multiple answers and subdivision into age groups possible) [Trimethoprim]
N/A

If you use antibiotic prophylaxis: which mediaction do you use? (multiple answers and subdivision into age groups possible) [Nitrofurantoin]
N/A

If you use antibiotic prophylaxis: which mediaction do you use? (multiple answers and subdivision into age groups possible) [Amoxicillin]
N/A

If you use antibiotic prophylaxis: which mediaction do you use? (multiple answers and subdivision into age groups possible) [Cefaclor]
N/A

If you use antibiotic prophylaxis: which mediaction do you use? (multiple answers and subdivision into age groups possible) [Amoxicillin/clavulanic acid]
N/A

If you use antibiotic prophylaxis: which mediaction do you use? (multiple answers and subdivision into age groups possible) [Other]
N/A

Trimethoprim: which time schedule do you use? [Day(s) before VUS/VCUG, how many?]
N/A

Trimethoprim: which time schedule do you use? [Day of VUS/VCUG]
N/A

Trimethoprim: which time schedule do you use? [Day(s) after VUS/VCUG, how many?]
N/A

Nitrofurantoin: which time schedule do you use? [Day(s) before VUS/VCUG, how many?]
N/A

Nitrofurantoin: which time schedule do you use? [Day of VUS/VCUG]
N/A

Nitrofurantoin: which time schedule do you use? [Day(s) after VUS/VCUG, how many?]
N/A

Amoxicillin: which time schedule do you use? [Day(s) before VUS/VCUG, how many?]
N/A

Amoxicillin: which time schedule do you use? [Day of VUS/VCUG]
N/A

Amoxicillin: which time schedule do you use? [Day(s) after VUS/VCUG, how many?]
N/A

Cefaclor: which time schedule do you use? [Days(s) before VUS/VCUG, how many?]
N/A

Cefaclor: which time schedule do you use? [Day of VUS/VCUG]
N/A

Cefaclor: which time schedule do you use? [Day(s) after VUS/VCUG, how many?]
N/A

Amoxicillin/clavulanic acid: in what dosage? (Amoxicillin portion) [prophylaktic (10-20 mg/Kg bodyweight/d)]
N/A

Amoxicillin/clavulanic acid: in what dosage? (Amoxicillin portion) [double prophylactic (20-40 mg/Kg bodyweight/d)]
N/A

Amoxicillin/clavulanic acid: in what dosage? (Amoxicillin portion) [therapeutic (80-100 mg/Kg bodyweight/d)]
N/A

Amoxicillin/clavulanic acid: which time schedule do you use? [Day(s) before VUS/VCUG]
N/A

Amoxicillin/clavulanic acid: which time schedule do you use? [Day of VUS/VCUG]
N/A

Amoxicillin/clavulanic acid: which time schedule do you use? [Day(s) after VUS/VCUG]
N/A

Other: which time schedule do you use? [Day(s) before VUS/VCUG, how many?]
N/A

Other: which time schedule do you use? [Day of VUS/VCUG]
N/A

Other: which time schedule do you use? [Day(s) after VUS/VCUG, how many?]
N/A

Indication for prophylaxis

Which patients receive periinterventional antibiotic prophylaxis? (multiple answers possible) [all]
N/A

Which patients receive periinterventional antibiotic prophylaxis? (multiple answers possible) [patients post urinary tract infection/pyelonephritis/urosepsis]
N/A

Which patients receive periinterventional antibiotic prophylaxis? (multiple answers possible) [patients with urinary tract dilation in ultrasound]
N/A

Which patients receive periinterventional antibiotic prophylaxis? (multiple answers possible) [patients with prenatal urinary tract dilation]
N/A

Which patients receive periinterventional antibiotic prophylaxis? (multiple answers possible) [patients post VUR surgery]
N/A

Which patients receive periinterventional antibiotic prophylaxis? (multiple answers possible) [none]
N/A

Are there situations or clinical findings in the patient where you deviate from your prophylaxis standard (medication, dose, duration, age of the patient)? (multiple answers possible, please specify in answer box) [in case of already known vesicoureteral reflux (VUR)]
N/A

Are there situations or clinical findings in the patient where you deviate from your prophylaxis standard (medication, dose, duration, age of the patient)? (multiple answers possible, please specify in answer box) [in patients with recurrent urinary tract infections (UTI)]
N/A

Are there situations or clinical findings in the patient where you deviate from your prophylaxis standard (medication, dose, duration, age of the patient)? (multiple answers possible, please specify in answer box) [if multidrug-resistant organisms (MDRO) are detected in urine cultures]
N/A

Are there situations or clinical findings in the patient where you deviate from your prophylaxis standard (medication, dose, duration, age of the patient)? (multiple answers possible, please specify in answer box) [in newborns]
N/A

Are there situations in which you use intravenous prophylaxis?
N/A

Voiding analysis

Are there differences in antibiotic prophylaxis between VUS and VCUG in your department?
N/A

Is a non-pathological urine strip test required in preparation for VUS/VCUG in your department?
N/A

Is a non-pathological urine culture required in preparation for VUS/VCUG in your department?
N/A

Do patients only receive post-procedure prophylaxis if vesicoureteral reflux (VUR) is detected in VUS/VCUG?
N/A

Do other MUS/MCU findings (e.g. urethral valves) have an influence on post-procedure prophylaxis?
N/A

End of survey

May we contact you personally if we have further questions?
N/A

Survey response 237

Response ID
239

Last page
0

Start language
en

Seed
731397496

Date started
2025-04-08 15:48:20

Date last action
2025-04-08 15:48:20

Standard Operation Procedure

Does your department have a defined protocol for infection prophylaxis in the context of VUS/VCUG?
N/A

Has this protocol been transformed into a written standard operating procedure (SOP)?
N/A

Antibiotic Prophylaxis

Are there situations/constellations in which you do not use antibiotic prophylaxis for VUS/VCUG?
N/A

Please specify these situations/constellations: [girl with first uncomplicated UTI without urinary tract dilation in ultrasound]
N/A

Please specify these situations/constellations: [boy with first uncomplicated UTI without urinary tract dilation in ultrasound]
N/A

Please specify these situations/constellations: [in patients with low grade urinary tract dilation (up to 2nd grade) and no UTI in medical history]
N/A

If you use antibiotic prophylaxis: which mediaction do you use? (multiple answers and subdivision into age groups possible) [Trimethoprim]
N/A

If you use antibiotic prophylaxis: which mediaction do you use? (multiple answers and subdivision into age groups possible) [Nitrofurantoin]
N/A

If you use antibiotic prophylaxis: which mediaction do you use? (multiple answers and subdivision into age groups possible) [Amoxicillin]
N/A

If you use antibiotic prophylaxis: which mediaction do you use? (multiple answers and subdivision into age groups possible) [Cefaclor]
N/A

If you use antibiotic prophylaxis: which mediaction do you use? (multiple answers and subdivision into age groups possible) [Amoxicillin/clavulanic acid]
N/A

If you use antibiotic prophylaxis: which mediaction do you use? (multiple answers and subdivision into age groups possible) [Other]
N/A

Trimethoprim: which time schedule do you use? [Day(s) before VUS/VCUG, how many?]
N/A

Trimethoprim: which time schedule do you use? [Day of VUS/VCUG]
N/A

Trimethoprim: which time schedule do you use? [Day(s) after VUS/VCUG, how many?]
N/A

Nitrofurantoin: which time schedule do you use? [Day(s) before VUS/VCUG, how many?]
N/A

Nitrofurantoin: which time schedule do you use? [Day of VUS/VCUG]
N/A

Nitrofurantoin: which time schedule do you use? [Day(s) after VUS/VCUG, how many?]
N/A

Amoxicillin: which time schedule do you use? [Day(s) before VUS/VCUG, how many?]
N/A

Amoxicillin: which time schedule do you use? [Day of VUS/VCUG]
N/A

Amoxicillin: which time schedule do you use? [Day(s) after VUS/VCUG, how many?]
N/A

Cefaclor: which time schedule do you use? [Days(s) before VUS/VCUG, how many?]
N/A

Cefaclor: which time schedule do you use? [Day of VUS/VCUG]
N/A

Cefaclor: which time schedule do you use? [Day(s) after VUS/VCUG, how many?]
N/A

Amoxicillin/clavulanic acid: in what dosage? (Amoxicillin portion) [prophylaktic (10-20 mg/Kg bodyweight/d)]
N/A

Amoxicillin/clavulanic acid: in what dosage? (Amoxicillin portion) [double prophylactic (20-40 mg/Kg bodyweight/d)]
N/A

Amoxicillin/clavulanic acid: in what dosage? (Amoxicillin portion) [therapeutic (80-100 mg/Kg bodyweight/d)]
N/A

Amoxicillin/clavulanic acid: which time schedule do you use? [Day(s) before VUS/VCUG]
N/A

Amoxicillin/clavulanic acid: which time schedule do you use? [Day of VUS/VCUG]
N/A

Amoxicillin/clavulanic acid: which time schedule do you use? [Day(s) after VUS/VCUG]
N/A

Other: which time schedule do you use? [Day(s) before VUS/VCUG, how many?]
N/A

Other: which time schedule do you use? [Day of VUS/VCUG]
N/A

Other: which time schedule do you use? [Day(s) after VUS/VCUG, how many?]
N/A

Indication for prophylaxis

Which patients receive periinterventional antibiotic prophylaxis? (multiple answers possible) [all]
N/A

Which patients receive periinterventional antibiotic prophylaxis? (multiple answers possible) [patients post urinary tract infection/pyelonephritis/urosepsis]
N/A

Which patients receive periinterventional antibiotic prophylaxis? (multiple answers possible) [patients with urinary tract dilation in ultrasound]
N/A

Which patients receive periinterventional antibiotic prophylaxis? (multiple answers possible) [patients with prenatal urinary tract dilation]
N/A

Which patients receive periinterventional antibiotic prophylaxis? (multiple answers possible) [patients post VUR surgery]
N/A

Which patients receive periinterventional antibiotic prophylaxis? (multiple answers possible) [none]
N/A

Are there situations or clinical findings in the patient where you deviate from your prophylaxis standard (medication, dose, duration, age of the patient)? (multiple answers possible, please specify in answer box) [in case of already known vesicoureteral reflux (VUR)]
N/A

Are there situations or clinical findings in the patient where you deviate from your prophylaxis standard (medication, dose, duration, age of the patient)? (multiple answers possible, please specify in answer box) [in patients with recurrent urinary tract infections (UTI)]
N/A

Are there situations or clinical findings in the patient where you deviate from your prophylaxis standard (medication, dose, duration, age of the patient)? (multiple answers possible, please specify in answer box) [if multidrug-resistant organisms (MDRO) are detected in urine cultures]
N/A

Are there situations or clinical findings in the patient where you deviate from your prophylaxis standard (medication, dose, duration, age of the patient)? (multiple answers possible, please specify in answer box) [in newborns]
N/A

Are there situations in which you use intravenous prophylaxis?
N/A

Voiding analysis

Are there differences in antibiotic prophylaxis between VUS and VCUG in your department?
N/A

Is a non-pathological urine strip test required in preparation for VUS/VCUG in your department?
N/A

Is a non-pathological urine culture required in preparation for VUS/VCUG in your department?
N/A

Do patients only receive post-procedure prophylaxis if vesicoureteral reflux (VUR) is detected in VUS/VCUG?
N/A

Do other MUS/MCU findings (e.g. urethral valves) have an influence on post-procedure prophylaxis?
N/A

End of survey

May we contact you personally if we have further questions?
N/A

Survey response 238

Response ID
240

Date submitted
2025-04-09 09:36:42

Last page
5

Start language
de

Seed
1091684198

Date started
2025-04-09 09:31:28

Date last action
2025-04-09 09:36:42

Standard Operation Procedure

Who fills out this survey?
Pediatric Surgeon

Who fills out this survey? [Other]

In which type of hospital are you employed?
Community hospital (>50 beds for pediatric patients)

In which type of hospital are you employed? [Other]

In which country do you work?
Mecklenburg-Vorpommern

Does your department have a defined protocol for infection prophylaxis in the context of VUS/VCUG?
Yes

Has this protocol been transformed into a written standard operating procedure (SOP)?
Yes

Which department is the author of the SOP for infection prophylaxis in VUS/VCUG?
Pediatric surgery

Which department is the author of the SOP for infection prophylaxis in VUS/VCUG? [Other]

Antibiotic Prophylaxis

Are there situations/constellations in which you do not use antibiotic prophylaxis for VUS/VCUG?
No

Please specify these situations/constellations: [girl with first uncomplicated UTI without urinary tract dilation in ultrasound]
N/A

Please specify these situations/constellations: [boy with first uncomplicated UTI without urinary tract dilation in ultrasound]
N/A

Please specify these situations/constellations: [in patients with low grade urinary tract dilation (up to 2nd grade) and no UTI in medical history]
N/A

If you use antibiotic prophylaxis: which mediaction do you use? (multiple answers and subdivision into age groups possible) [Trimethoprim]
No

If you use antibiotic prophylaxis: which mediaction do you use? (multiple answers and subdivision into age groups possible) [Nitrofurantoin]
Yes

If you use antibiotic prophylaxis: which mediaction do you use? (multiple answers and subdivision into age groups possible) [Amoxicillin]
No

If you use antibiotic prophylaxis: which mediaction do you use? (multiple answers and subdivision into age groups possible) [Cefaclor]
Yes

If you use antibiotic prophylaxis: which mediaction do you use? (multiple answers and subdivision into age groups possible) [Amoxicillin/clavulanic acid]
No

If you use antibiotic prophylaxis: which mediaction do you use? (multiple answers and subdivision into age groups possible) [Other]
No

Trimethoprim: which time schedule do you use? [Day(s) before VUS/VCUG, how many?]
N/A

Trimethoprim: which time schedule do you use? [Day of VUS/VCUG]
N/A

Trimethoprim: which time schedule do you use? [Day(s) after VUS/VCUG, how many?]
N/A

Nitrofurantoin: in what dosage?
prophylactic (1-2 mg/kg bodyweight/d)

Nitrofurantoin: in what dosage? [Other]

Nitrofurantoin: for which age group do you use nitrofurantoin?
>1.LJ

Nitrofurantoin: which time schedule do you use? [Day(s) before VUS/VCUG, how many?]
No

Nitrofurantoin: which time schedule do you use? [Day(s) before VUS/VCUG, how many?][Comment]

Nitrofurantoin: which time schedule do you use? [Day of VUS/VCUG]
Yes

Nitrofurantoin: which time schedule do you use? [Day of VUS/VCUG][Comment]

Nitrofurantoin: which time schedule do you use? [Day(s) after VUS/VCUG, how many?]
Yes

Nitrofurantoin: which time schedule do you use? [Day(s) after VUS/VCUG, how many?][Comment]
2

Amoxicillin: which time schedule do you use? [Day(s) before VUS/VCUG, how many?]
N/A

Amoxicillin: which time schedule do you use? [Day of VUS/VCUG]
N/A

Amoxicillin: which time schedule do you use? [Day(s) after VUS/VCUG, how many?]
N/A

Cefaclor: in what dosage?
prophylactic (10 mg/kg bodyweight/d)

Cefaclor: in what dosage? [Other]

Cefaclor: for which age group do you use cefaclor?
bis 1.LJ. und Unverträglichkeiten von Nifurantoin

Cefaclor: which time schedule do you use? [Days(s) before VUS/VCUG, how many?]
No

Cefaclor: which time schedule do you use? [Days(s) before VUS/VCUG, how many?][Comment]

Cefaclor: which time schedule do you use? [Day of VUS/VCUG]
Yes

Cefaclor: which time schedule do you use? [Day of VUS/VCUG][Comment]

Cefaclor: which time schedule do you use? [Day(s) after VUS/VCUG, how many?]
Yes

Cefaclor: which time schedule do you use? [Day(s) after VUS/VCUG, how many?][Comment]
2

Amoxicillin/clavulanic acid: in what dosage? (Amoxicillin portion) [prophylaktic (10-20 mg/Kg bodyweight/d)]
N/A

Amoxicillin/clavulanic acid: in what dosage? (Amoxicillin portion) [double prophylactic (20-40 mg/Kg bodyweight/d)]
N/A

Amoxicillin/clavulanic acid: in what dosage? (Amoxicillin portion) [therapeutic (80-100 mg/Kg bodyweight/d)]
N/A

Amoxicillin/clavulanic acid: which time schedule do you use? [Day(s) before VUS/VCUG]
N/A

Amoxicillin/clavulanic acid: which time schedule do you use? [Day of VUS/VCUG]
N/A

Amoxicillin/clavulanic acid: which time schedule do you use? [Day(s) after VUS/VCUG]
N/A

Other: which time schedule do you use? [Day(s) before VUS/VCUG, how many?]
N/A

Other: which time schedule do you use? [Day of VUS/VCUG]
N/A

Other: which time schedule do you use? [Day(s) after VUS/VCUG, how many?]
N/A

Indication for prophylaxis

Which patients receive periinterventional antibiotic prophylaxis? (multiple answers possible) [all]
Yes

Which patients receive periinterventional antibiotic prophylaxis? (multiple answers possible) [patients post urinary tract infection/pyelonephritis/urosepsis]
N/A

Which patients receive periinterventional antibiotic prophylaxis? (multiple answers possible) [patients with urinary tract dilation in ultrasound]
N/A

Which patients receive periinterventional antibiotic prophylaxis? (multiple answers possible) [patients with prenatal urinary tract dilation]
N/A

Which patients receive periinterventional antibiotic prophylaxis? (multiple answers possible) [patients post VUR surgery]
N/A

Which patients receive periinterventional antibiotic prophylaxis? (multiple answers possible) [none]
N/A

Are there situations or clinical findings in the patient where you deviate from your prophylaxis standard (medication, dose, duration, age of the patient)? (multiple answers possible, please specify in answer box) [in case of already known vesicoureteral reflux (VUR)]
No

Are there situations or clinical findings in the patient where you deviate from your prophylaxis standard (medication, dose, duration, age of the patient)? (multiple answers possible, please specify in answer box) [in case of already known vesicoureteral reflux (VUR)][Comment]

Are there situations or clinical findings in the patient where you deviate from your prophylaxis standard (medication, dose, duration, age of the patient)? (multiple answers possible, please specify in answer box) [in patients with recurrent urinary tract infections (UTI)]
Yes

Are there situations or clinical findings in the patient where you deviate from your prophylaxis standard (medication, dose, duration, age of the patient)? (multiple answers possible, please specify in answer box) [in patients with recurrent urinary tract infections (UTI)][Comment]
dann Prophylaxe entsprechend Keimnachweis

Are there situations or clinical findings in the patient where you deviate from your prophylaxis standard (medication, dose, duration, age of the patient)? (multiple answers possible, please specify in answer box) [if multidrug-resistant organisms (MDRO) are detected in urine cultures]
Yes

Are there situations or clinical findings in the patient where you deviate from your prophylaxis standard (medication, dose, duration, age of the patient)? (multiple answers possible, please specify in answer box) [if multidrug-resistant organisms (MDRO) are detected in urine cultures][Comment]
dann ebenfalls Abweischen auf sensible AB

Are there situations or clinical findings in the patient where you deviate from your prophylaxis standard (medication, dose, duration, age of the patient)? (multiple answers possible, please specify in answer box) [in newborns]
No

Are there situations or clinical findings in the patient where you deviate from your prophylaxis standard (medication, dose, duration, age of the patient)? (multiple answers possible, please specify in answer box) [in newborns][Comment]

Are there situations or clinical findings in the patient where you deviate from your prophylaxis standard (medication, dose, duration, age of the patient)? (multiple answers possible, please specify in answer box) [Other]

Are there situations or clinical findings in the patient where you deviate from your prophylaxis standard (medication, dose, duration, age of the patient)? (multiple answers possible, please specify in answer box) [Other comment]

Are there situations in which you use intravenous prophylaxis?
No

Voiding analysis

Are there differences in antibiotic prophylaxis between VUS and VCUG in your department?
No

Is a non-pathological urine strip test required in preparation for VUS/VCUG in your department?
No

Is a non-pathological urine culture required in preparation for VUS/VCUG in your department?
No

Do patients only receive post-procedure prophylaxis if vesicoureteral reflux (VUR) is detected in VUS/VCUG?
No

Do other MUS/MCU findings (e.g. urethral valves) have an influence on post-procedure prophylaxis?
No

End of survey

Thank you for taking the time to answer this survey!

May we contact you personally if we have further questions?
Yes

E-mail address:
TiedtkeA@dbknb.de

Survey response 239

Response ID
241

Last page
1

Start language
de

Seed
45534986

Date started
2025-04-14 11:21:02

Date last action
2025-04-14 11:21:18

Standard Operation Procedure

Who fills out this survey?
Pediatric Surgeon

Who fills out this survey? [Other]

In which type of hospital are you employed?
Community hospital (>50 beds for pediatric patients)

In which type of hospital are you employed? [Other]

In which country do you work?
Deutschland

Does your department have a defined protocol for infection prophylaxis in the context of VUS/VCUG?
Yes

Has this protocol been transformed into a written standard operating procedure (SOP)?
No

Antibiotic Prophylaxis

Are there situations/constellations in which you do not use antibiotic prophylaxis for VUS/VCUG?
N/A

Please specify these situations/constellations: [girl with first uncomplicated UTI without urinary tract dilation in ultrasound]
N/A

Please specify these situations/constellations: [boy with first uncomplicated UTI without urinary tract dilation in ultrasound]
N/A

Please specify these situations/constellations: [in patients with low grade urinary tract dilation (up to 2nd grade) and no UTI in medical history]
N/A

If you use antibiotic prophylaxis: which mediaction do you use? (multiple answers and subdivision into age groups possible) [Trimethoprim]
N/A

If you use antibiotic prophylaxis: which mediaction do you use? (multiple answers and subdivision into age groups possible) [Nitrofurantoin]
N/A

If you use antibiotic prophylaxis: which mediaction do you use? (multiple answers and subdivision into age groups possible) [Amoxicillin]
N/A

If you use antibiotic prophylaxis: which mediaction do you use? (multiple answers and subdivision into age groups possible) [Cefaclor]
N/A

If you use antibiotic prophylaxis: which mediaction do you use? (multiple answers and subdivision into age groups possible) [Amoxicillin/clavulanic acid]
N/A

If you use antibiotic prophylaxis: which mediaction do you use? (multiple answers and subdivision into age groups possible) [Other]
N/A

Trimethoprim: which time schedule do you use? [Day(s) before VUS/VCUG, how many?]
N/A

Trimethoprim: which time schedule do you use? [Day of VUS/VCUG]
N/A

Trimethoprim: which time schedule do you use? [Day(s) after VUS/VCUG, how many?]
N/A

Nitrofurantoin: which time schedule do you use? [Day(s) before VUS/VCUG, how many?]
N/A

Nitrofurantoin: which time schedule do you use? [Day of VUS/VCUG]
N/A

Nitrofurantoin: which time schedule do you use? [Day(s) after VUS/VCUG, how many?]
N/A

Amoxicillin: which time schedule do you use? [Day(s) before VUS/VCUG, how many?]
N/A

Amoxicillin: which time schedule do you use? [Day of VUS/VCUG]
N/A

Amoxicillin: which time schedule do you use? [Day(s) after VUS/VCUG, how many?]
N/A

Cefaclor: which time schedule do you use? [Days(s) before VUS/VCUG, how many?]
N/A

Cefaclor: which time schedule do you use? [Day of VUS/VCUG]
N/A

Cefaclor: which time schedule do you use? [Day(s) after VUS/VCUG, how many?]
N/A

Amoxicillin/clavulanic acid: in what dosage? (Amoxicillin portion) [prophylaktic (10-20 mg/Kg bodyweight/d)]
N/A

Amoxicillin/clavulanic acid: in what dosage? (Amoxicillin portion) [double prophylactic (20-40 mg/Kg bodyweight/d)]
N/A

Amoxicillin/clavulanic acid: in what dosage? (Amoxicillin portion) [therapeutic (80-100 mg/Kg bodyweight/d)]
N/A

Amoxicillin/clavulanic acid: which time schedule do you use? [Day(s) before VUS/VCUG]
N/A

Amoxicillin/clavulanic acid: which time schedule do you use? [Day of VUS/VCUG]
N/A

Amoxicillin/clavulanic acid: which time schedule do you use? [Day(s) after VUS/VCUG]
N/A

Other: which time schedule do you use? [Day(s) before VUS/VCUG, how many?]
N/A

Other: which time schedule do you use? [Day of VUS/VCUG]
N/A

Other: which time schedule do you use? [Day(s) after VUS/VCUG, how many?]
N/A

Indication for prophylaxis

Which patients receive periinterventional antibiotic prophylaxis? (multiple answers possible) [all]
N/A

Which patients receive periinterventional antibiotic prophylaxis? (multiple answers possible) [patients post urinary tract infection/pyelonephritis/urosepsis]
N/A

Which patients receive periinterventional antibiotic prophylaxis? (multiple answers possible) [patients with urinary tract dilation in ultrasound]
N/A

Which patients receive periinterventional antibiotic prophylaxis? (multiple answers possible) [patients with prenatal urinary tract dilation]
N/A

Which patients receive periinterventional antibiotic prophylaxis? (multiple answers possible) [patients post VUR surgery]
N/A

Which patients receive periinterventional antibiotic prophylaxis? (multiple answers possible) [none]
N/A

Are there situations or clinical findings in the patient where you deviate from your prophylaxis standard (medication, dose, duration, age of the patient)? (multiple answers possible, please specify in answer box) [in case of already known vesicoureteral reflux (VUR)]
N/A

Are there situations or clinical findings in the patient where you deviate from your prophylaxis standard (medication, dose, duration, age of the patient)? (multiple answers possible, please specify in answer box) [in patients with recurrent urinary tract infections (UTI)]
N/A

Are there situations or clinical findings in the patient where you deviate from your prophylaxis standard (medication, dose, duration, age of the patient)? (multiple answers possible, please specify in answer box) [if multidrug-resistant organisms (MDRO) are detected in urine cultures]
N/A

Are there situations or clinical findings in the patient where you deviate from your prophylaxis standard (medication, dose, duration, age of the patient)? (multiple answers possible, please specify in answer box) [in newborns]
N/A

Are there situations in which you use intravenous prophylaxis?
N/A

Voiding analysis

Are there differences in antibiotic prophylaxis between VUS and VCUG in your department?
N/A

Is a non-pathological urine strip test required in preparation for VUS/VCUG in your department?
N/A

Is a non-pathological urine culture required in preparation for VUS/VCUG in your department?
N/A

Do patients only receive post-procedure prophylaxis if vesicoureteral reflux (VUR) is detected in VUS/VCUG?
N/A

Do other MUS/MCU findings (e.g. urethral valves) have an influence on post-procedure prophylaxis?
N/A

End of survey

May we contact you personally if we have further questions?
N/A

Survey response 240

Response ID
242

Last page
1

Start language
de

Seed
1129248103

Date started
2025-04-14 11:29:42

Date last action
2025-04-14 11:30:14

Standard Operation Procedure

Who fills out this survey?
Pediatric urologist

Who fills out this survey? [Other]

In which type of hospital are you employed?
University Hospital

In which type of hospital are you employed? [Other]

In which country do you work?
Deutschland

Does your department have a defined protocol for infection prophylaxis in the context of VUS/VCUG?
No

Has this protocol been transformed into a written standard operating procedure (SOP)?
N/A

Antibiotic Prophylaxis

Are there situations/constellations in which you do not use antibiotic prophylaxis for VUS/VCUG?
N/A

Please specify these situations/constellations: [girl with first uncomplicated UTI without urinary tract dilation in ultrasound]
N/A

Please specify these situations/constellations: [boy with first uncomplicated UTI without urinary tract dilation in ultrasound]
N/A

Please specify these situations/constellations: [in patients with low grade urinary tract dilation (up to 2nd grade) and no UTI in medical history]
N/A

If you use antibiotic prophylaxis: which mediaction do you use? (multiple answers and subdivision into age groups possible) [Trimethoprim]
N/A

If you use antibiotic prophylaxis: which mediaction do you use? (multiple answers and subdivision into age groups possible) [Nitrofurantoin]
N/A

If you use antibiotic prophylaxis: which mediaction do you use? (multiple answers and subdivision into age groups possible) [Amoxicillin]
N/A

If you use antibiotic prophylaxis: which mediaction do you use? (multiple answers and subdivision into age groups possible) [Cefaclor]
N/A

If you use antibiotic prophylaxis: which mediaction do you use? (multiple answers and subdivision into age groups possible) [Amoxicillin/clavulanic acid]
N/A

If you use antibiotic prophylaxis: which mediaction do you use? (multiple answers and subdivision into age groups possible) [Other]
N/A

Trimethoprim: which time schedule do you use? [Day(s) before VUS/VCUG, how many?]
N/A

Trimethoprim: which time schedule do you use? [Day of VUS/VCUG]
N/A

Trimethoprim: which time schedule do you use? [Day(s) after VUS/VCUG, how many?]
N/A

Nitrofurantoin: which time schedule do you use? [Day(s) before VUS/VCUG, how many?]
N/A

Nitrofurantoin: which time schedule do you use? [Day of VUS/VCUG]
N/A

Nitrofurantoin: which time schedule do you use? [Day(s) after VUS/VCUG, how many?]
N/A

Amoxicillin: which time schedule do you use? [Day(s) before VUS/VCUG, how many?]
N/A

Amoxicillin: which time schedule do you use? [Day of VUS/VCUG]
N/A

Amoxicillin: which time schedule do you use? [Day(s) after VUS/VCUG, how many?]
N/A

Cefaclor: which time schedule do you use? [Days(s) before VUS/VCUG, how many?]
N/A

Cefaclor: which time schedule do you use? [Day of VUS/VCUG]
N/A

Cefaclor: which time schedule do you use? [Day(s) after VUS/VCUG, how many?]
N/A

Amoxicillin/clavulanic acid: in what dosage? (Amoxicillin portion) [prophylaktic (10-20 mg/Kg bodyweight/d)]
N/A

Amoxicillin/clavulanic acid: in what dosage? (Amoxicillin portion) [double prophylactic (20-40 mg/Kg bodyweight/d)]
N/A

Amoxicillin/clavulanic acid: in what dosage? (Amoxicillin portion) [therapeutic (80-100 mg/Kg bodyweight/d)]
N/A

Amoxicillin/clavulanic acid: which time schedule do you use? [Day(s) before VUS/VCUG]
N/A

Amoxicillin/clavulanic acid: which time schedule do you use? [Day of VUS/VCUG]
N/A

Amoxicillin/clavulanic acid: which time schedule do you use? [Day(s) after VUS/VCUG]
N/A

Other: which time schedule do you use? [Day(s) before VUS/VCUG, how many?]
N/A

Other: which time schedule do you use? [Day of VUS/VCUG]
N/A

Other: which time schedule do you use? [Day(s) after VUS/VCUG, how many?]
N/A

Indication for prophylaxis

Which patients receive periinterventional antibiotic prophylaxis? (multiple answers possible) [all]
N/A

Which patients receive periinterventional antibiotic prophylaxis? (multiple answers possible) [patients post urinary tract infection/pyelonephritis/urosepsis]
N/A

Which patients receive periinterventional antibiotic prophylaxis? (multiple answers possible) [patients with urinary tract dilation in ultrasound]
N/A

Which patients receive periinterventional antibiotic prophylaxis? (multiple answers possible) [patients with prenatal urinary tract dilation]
N/A

Which patients receive periinterventional antibiotic prophylaxis? (multiple answers possible) [patients post VUR surgery]
N/A

Which patients receive periinterventional antibiotic prophylaxis? (multiple answers possible) [none]
N/A

Are there situations or clinical findings in the patient where you deviate from your prophylaxis standard (medication, dose, duration, age of the patient)? (multiple answers possible, please specify in answer box) [in case of already known vesicoureteral reflux (VUR)]
N/A

Are there situations or clinical findings in the patient where you deviate from your prophylaxis standard (medication, dose, duration, age of the patient)? (multiple answers possible, please specify in answer box) [in patients with recurrent urinary tract infections (UTI)]
N/A

Are there situations or clinical findings in the patient where you deviate from your prophylaxis standard (medication, dose, duration, age of the patient)? (multiple answers possible, please specify in answer box) [if multidrug-resistant organisms (MDRO) are detected in urine cultures]
N/A

Are there situations or clinical findings in the patient where you deviate from your prophylaxis standard (medication, dose, duration, age of the patient)? (multiple answers possible, please specify in answer box) [in newborns]
N/A

Are there situations in which you use intravenous prophylaxis?
N/A

Voiding analysis

Are there differences in antibiotic prophylaxis between VUS and VCUG in your department?
N/A

Is a non-pathological urine strip test required in preparation for VUS/VCUG in your department?
N/A

Is a non-pathological urine culture required in preparation for VUS/VCUG in your department?
N/A

Do patients only receive post-procedure prophylaxis if vesicoureteral reflux (VUR) is detected in VUS/VCUG?
N/A

Do other MUS/MCU findings (e.g. urethral valves) have an influence on post-procedure prophylaxis?
N/A

End of survey

May we contact you personally if we have further questions?
N/A

Survey response 241

Response ID
243

Last page
0

Start language
de

Seed
1530462089

Date started
2025-04-14 11:56:05

Date last action
2025-04-14 11:56:05

Standard Operation Procedure

Does your department have a defined protocol for infection prophylaxis in the context of VUS/VCUG?
N/A

Has this protocol been transformed into a written standard operating procedure (SOP)?
N/A

Antibiotic Prophylaxis

Are there situations/constellations in which you do not use antibiotic prophylaxis for VUS/VCUG?
N/A

Please specify these situations/constellations: [girl with first uncomplicated UTI without urinary tract dilation in ultrasound]
N/A

Please specify these situations/constellations: [boy with first uncomplicated UTI without urinary tract dilation in ultrasound]
N/A

Please specify these situations/constellations: [in patients with low grade urinary tract dilation (up to 2nd grade) and no UTI in medical history]
N/A

If you use antibiotic prophylaxis: which mediaction do you use? (multiple answers and subdivision into age groups possible) [Trimethoprim]
N/A

If you use antibiotic prophylaxis: which mediaction do you use? (multiple answers and subdivision into age groups possible) [Nitrofurantoin]
N/A

If you use antibiotic prophylaxis: which mediaction do you use? (multiple answers and subdivision into age groups possible) [Amoxicillin]
N/A

If you use antibiotic prophylaxis: which mediaction do you use? (multiple answers and subdivision into age groups possible) [Cefaclor]
N/A

If you use antibiotic prophylaxis: which mediaction do you use? (multiple answers and subdivision into age groups possible) [Amoxicillin/clavulanic acid]
N/A

If you use antibiotic prophylaxis: which mediaction do you use? (multiple answers and subdivision into age groups possible) [Other]
N/A

Trimethoprim: which time schedule do you use? [Day(s) before VUS/VCUG, how many?]
N/A

Trimethoprim: which time schedule do you use? [Day of VUS/VCUG]
N/A

Trimethoprim: which time schedule do you use? [Day(s) after VUS/VCUG, how many?]
N/A

Nitrofurantoin: which time schedule do you use? [Day(s) before VUS/VCUG, how many?]
N/A

Nitrofurantoin: which time schedule do you use? [Day of VUS/VCUG]
N/A

Nitrofurantoin: which time schedule do you use? [Day(s) after VUS/VCUG, how many?]
N/A

Amoxicillin: which time schedule do you use? [Day(s) before VUS/VCUG, how many?]
N/A

Amoxicillin: which time schedule do you use? [Day of VUS/VCUG]
N/A

Amoxicillin: which time schedule do you use? [Day(s) after VUS/VCUG, how many?]
N/A

Cefaclor: which time schedule do you use? [Days(s) before VUS/VCUG, how many?]
N/A

Cefaclor: which time schedule do you use? [Day of VUS/VCUG]
N/A

Cefaclor: which time schedule do you use? [Day(s) after VUS/VCUG, how many?]
N/A

Amoxicillin/clavulanic acid: in what dosage? (Amoxicillin portion) [prophylaktic (10-20 mg/Kg bodyweight/d)]
N/A

Amoxicillin/clavulanic acid: in what dosage? (Amoxicillin portion) [double prophylactic (20-40 mg/Kg bodyweight/d)]
N/A

Amoxicillin/clavulanic acid: in what dosage? (Amoxicillin portion) [therapeutic (80-100 mg/Kg bodyweight/d)]
N/A

Amoxicillin/clavulanic acid: which time schedule do you use? [Day(s) before VUS/VCUG]
N/A

Amoxicillin/clavulanic acid: which time schedule do you use? [Day of VUS/VCUG]
N/A

Amoxicillin/clavulanic acid: which time schedule do you use? [Day(s) after VUS/VCUG]
N/A

Other: which time schedule do you use? [Day(s) before VUS/VCUG, how many?]
N/A

Other: which time schedule do you use? [Day of VUS/VCUG]
N/A

Other: which time schedule do you use? [Day(s) after VUS/VCUG, how many?]
N/A

Indication for prophylaxis

Which patients receive periinterventional antibiotic prophylaxis? (multiple answers possible) [all]
N/A

Which patients receive periinterventional antibiotic prophylaxis? (multiple answers possible) [patients post urinary tract infection/pyelonephritis/urosepsis]
N/A

Which patients receive periinterventional antibiotic prophylaxis? (multiple answers possible) [patients with urinary tract dilation in ultrasound]
N/A

Which patients receive periinterventional antibiotic prophylaxis? (multiple answers possible) [patients with prenatal urinary tract dilation]
N/A

Which patients receive periinterventional antibiotic prophylaxis? (multiple answers possible) [patients post VUR surgery]
N/A

Which patients receive periinterventional antibiotic prophylaxis? (multiple answers possible) [none]
N/A

Are there situations or clinical findings in the patient where you deviate from your prophylaxis standard (medication, dose, duration, age of the patient)? (multiple answers possible, please specify in answer box) [in case of already known vesicoureteral reflux (VUR)]
N/A

Are there situations or clinical findings in the patient where you deviate from your prophylaxis standard (medication, dose, duration, age of the patient)? (multiple answers possible, please specify in answer box) [in patients with recurrent urinary tract infections (UTI)]
N/A

Are there situations or clinical findings in the patient where you deviate from your prophylaxis standard (medication, dose, duration, age of the patient)? (multiple answers possible, please specify in answer box) [if multidrug-resistant organisms (MDRO) are detected in urine cultures]
N/A

Are there situations or clinical findings in the patient where you deviate from your prophylaxis standard (medication, dose, duration, age of the patient)? (multiple answers possible, please specify in answer box) [in newborns]
N/A

Are there situations in which you use intravenous prophylaxis?
N/A

Voiding analysis

Are there differences in antibiotic prophylaxis between VUS and VCUG in your department?
N/A

Is a non-pathological urine strip test required in preparation for VUS/VCUG in your department?
N/A

Is a non-pathological urine culture required in preparation for VUS/VCUG in your department?
N/A

Do patients only receive post-procedure prophylaxis if vesicoureteral reflux (VUR) is detected in VUS/VCUG?
N/A

Do other MUS/MCU findings (e.g. urethral valves) have an influence on post-procedure prophylaxis?
N/A

End of survey

May we contact you personally if we have further questions?
N/A

Survey response 242

Response ID
244

Last page
0

Start language
de

Seed
1636884020

Date started
2025-04-14 12:48:32

Date last action
2025-04-14 12:48:32

Standard Operation Procedure

Does your department have a defined protocol for infection prophylaxis in the context of VUS/VCUG?
N/A

Has this protocol been transformed into a written standard operating procedure (SOP)?
N/A

Antibiotic Prophylaxis

Are there situations/constellations in which you do not use antibiotic prophylaxis for VUS/VCUG?
N/A

Please specify these situations/constellations: [girl with first uncomplicated UTI without urinary tract dilation in ultrasound]
N/A

Please specify these situations/constellations: [boy with first uncomplicated UTI without urinary tract dilation in ultrasound]
N/A

Please specify these situations/constellations: [in patients with low grade urinary tract dilation (up to 2nd grade) and no UTI in medical history]
N/A

If you use antibiotic prophylaxis: which mediaction do you use? (multiple answers and subdivision into age groups possible) [Trimethoprim]
N/A

If you use antibiotic prophylaxis: which mediaction do you use? (multiple answers and subdivision into age groups possible) [Nitrofurantoin]
N/A

If you use antibiotic prophylaxis: which mediaction do you use? (multiple answers and subdivision into age groups possible) [Amoxicillin]
N/A

If you use antibiotic prophylaxis: which mediaction do you use? (multiple answers and subdivision into age groups possible) [Cefaclor]
N/A

If you use antibiotic prophylaxis: which mediaction do you use? (multiple answers and subdivision into age groups possible) [Amoxicillin/clavulanic acid]
N/A

If you use antibiotic prophylaxis: which mediaction do you use? (multiple answers and subdivision into age groups possible) [Other]
N/A

Trimethoprim: which time schedule do you use? [Day(s) before VUS/VCUG, how many?]
N/A

Trimethoprim: which time schedule do you use? [Day of VUS/VCUG]
N/A

Trimethoprim: which time schedule do you use? [Day(s) after VUS/VCUG, how many?]
N/A

Nitrofurantoin: which time schedule do you use? [Day(s) before VUS/VCUG, how many?]
N/A

Nitrofurantoin: which time schedule do you use? [Day of VUS/VCUG]
N/A

Nitrofurantoin: which time schedule do you use? [Day(s) after VUS/VCUG, how many?]
N/A

Amoxicillin: which time schedule do you use? [Day(s) before VUS/VCUG, how many?]
N/A

Amoxicillin: which time schedule do you use? [Day of VUS/VCUG]
N/A

Amoxicillin: which time schedule do you use? [Day(s) after VUS/VCUG, how many?]
N/A

Cefaclor: which time schedule do you use? [Days(s) before VUS/VCUG, how many?]
N/A

Cefaclor: which time schedule do you use? [Day of VUS/VCUG]
N/A

Cefaclor: which time schedule do you use? [Day(s) after VUS/VCUG, how many?]
N/A

Amoxicillin/clavulanic acid: in what dosage? (Amoxicillin portion) [prophylaktic (10-20 mg/Kg bodyweight/d)]
N/A

Amoxicillin/clavulanic acid: in what dosage? (Amoxicillin portion) [double prophylactic (20-40 mg/Kg bodyweight/d)]
N/A

Amoxicillin/clavulanic acid: in what dosage? (Amoxicillin portion) [therapeutic (80-100 mg/Kg bodyweight/d)]
N/A

Amoxicillin/clavulanic acid: which time schedule do you use? [Day(s) before VUS/VCUG]
N/A

Amoxicillin/clavulanic acid: which time schedule do you use? [Day of VUS/VCUG]
N/A

Amoxicillin/clavulanic acid: which time schedule do you use? [Day(s) after VUS/VCUG]
N/A

Other: which time schedule do you use? [Day(s) before VUS/VCUG, how many?]
N/A

Other: which time schedule do you use? [Day of VUS/VCUG]
N/A

Other: which time schedule do you use? [Day(s) after VUS/VCUG, how many?]
N/A

Indication for prophylaxis

Which patients receive periinterventional antibiotic prophylaxis? (multiple answers possible) [all]
N/A

Which patients receive periinterventional antibiotic prophylaxis? (multiple answers possible) [patients post urinary tract infection/pyelonephritis/urosepsis]
N/A

Which patients receive periinterventional antibiotic prophylaxis? (multiple answers possible) [patients with urinary tract dilation in ultrasound]
N/A

Which patients receive periinterventional antibiotic prophylaxis? (multiple answers possible) [patients with prenatal urinary tract dilation]
N/A

Which patients receive periinterventional antibiotic prophylaxis? (multiple answers possible) [patients post VUR surgery]
N/A

Which patients receive periinterventional antibiotic prophylaxis? (multiple answers possible) [none]
N/A

Are there situations or clinical findings in the patient where you deviate from your prophylaxis standard (medication, dose, duration, age of the patient)? (multiple answers possible, please specify in answer box) [in case of already known vesicoureteral reflux (VUR)]
N/A

Are there situations or clinical findings in the patient where you deviate from your prophylaxis standard (medication, dose, duration, age of the patient)? (multiple answers possible, please specify in answer box) [in patients with recurrent urinary tract infections (UTI)]
N/A

Are there situations or clinical findings in the patient where you deviate from your prophylaxis standard (medication, dose, duration, age of the patient)? (multiple answers possible, please specify in answer box) [if multidrug-resistant organisms (MDRO) are detected in urine cultures]
N/A

Are there situations or clinical findings in the patient where you deviate from your prophylaxis standard (medication, dose, duration, age of the patient)? (multiple answers possible, please specify in answer box) [in newborns]
N/A

Are there situations in which you use intravenous prophylaxis?
N/A

Voiding analysis

Are there differences in antibiotic prophylaxis between VUS and VCUG in your department?
N/A

Is a non-pathological urine strip test required in preparation for VUS/VCUG in your department?
N/A

Is a non-pathological urine culture required in preparation for VUS/VCUG in your department?
N/A

Do patients only receive post-procedure prophylaxis if vesicoureteral reflux (VUR) is detected in VUS/VCUG?
N/A

Do other MUS/MCU findings (e.g. urethral valves) have an influence on post-procedure prophylaxis?
N/A

End of survey

May we contact you personally if we have further questions?
N/A

Survey response 243

Response ID
245

Date submitted
2025-04-14 20:42:09

Last page
5

Start language
de

Seed
1953800039

Date started
2025-04-14 20:34:42

Date last action
2025-04-14 20:42:09

Standard Operation Procedure

Who fills out this survey?
Pediatric Surgeon

Who fills out this survey? [Other]

In which type of hospital are you employed?
University Hospital

In which type of hospital are you employed? [Other]

In which country do you work?
Deutschland

Does your department have a defined protocol for infection prophylaxis in the context of VUS/VCUG?
No

Has this protocol been transformed into a written standard operating procedure (SOP)?
N/A

Antibiotic Prophylaxis

Are there situations/constellations in which you do not use antibiotic prophylaxis for VUS/VCUG?
Yes

Please specify these situations/constellations: [girl with first uncomplicated UTI without urinary tract dilation in ultrasound]
No

Please specify these situations/constellations: [boy with first uncomplicated UTI without urinary tract dilation in ultrasound]
No

Please specify these situations/constellations: [in patients with low grade urinary tract dilation (up to 2nd grade) and no UTI in medical history]
No

Please specify these situations/constellations: [Other]
Sauberer Urin

If you use antibiotic prophylaxis: which mediaction do you use? (multiple answers and subdivision into age groups possible) [Trimethoprim]
No

If you use antibiotic prophylaxis: which mediaction do you use? (multiple answers and subdivision into age groups possible) [Nitrofurantoin]
No

If you use antibiotic prophylaxis: which mediaction do you use? (multiple answers and subdivision into age groups possible) [Amoxicillin]
No

If you use antibiotic prophylaxis: which mediaction do you use? (multiple answers and subdivision into age groups possible) [Cefaclor]
No

If you use antibiotic prophylaxis: which mediaction do you use? (multiple answers and subdivision into age groups possible) [Amoxicillin/clavulanic acid]
No

If you use antibiotic prophylaxis: which mediaction do you use? (multiple answers and subdivision into age groups possible) [Other]
Yes

Trimethoprim: which time schedule do you use? [Day(s) before VUS/VCUG, how many?]
N/A

Trimethoprim: which time schedule do you use? [Day of VUS/VCUG]
N/A

Trimethoprim: which time schedule do you use? [Day(s) after VUS/VCUG, how many?]
N/A

Nitrofurantoin: which time schedule do you use? [Day(s) before VUS/VCUG, how many?]
N/A

Nitrofurantoin: which time schedule do you use? [Day of VUS/VCUG]
N/A

Nitrofurantoin: which time schedule do you use? [Day(s) after VUS/VCUG, how many?]
N/A

Amoxicillin: which time schedule do you use? [Day(s) before VUS/VCUG, how many?]
N/A

Amoxicillin: which time schedule do you use? [Day of VUS/VCUG]
N/A

Amoxicillin: which time schedule do you use? [Day(s) after VUS/VCUG, how many?]
N/A

Cefaclor: which time schedule do you use? [Days(s) before VUS/VCUG, how many?]
N/A

Cefaclor: which time schedule do you use? [Day of VUS/VCUG]
N/A

Cefaclor: which time schedule do you use? [Day(s) after VUS/VCUG, how many?]
N/A

Amoxicillin/clavulanic acid: in what dosage? (Amoxicillin portion) [prophylaktic (10-20 mg/Kg bodyweight/d)]
N/A

Amoxicillin/clavulanic acid: in what dosage? (Amoxicillin portion) [double prophylactic (20-40 mg/Kg bodyweight/d)]
N/A

Amoxicillin/clavulanic acid: in what dosage? (Amoxicillin portion) [therapeutic (80-100 mg/Kg bodyweight/d)]
N/A

Amoxicillin/clavulanic acid: which time schedule do you use? [Day(s) before VUS/VCUG]
N/A

Amoxicillin/clavulanic acid: which time schedule do you use? [Day of VUS/VCUG]
N/A

Amoxicillin/clavulanic acid: which time schedule do you use? [Day(s) after VUS/VCUG]
N/A

Other: which medication and in what dosage?
Je nach Keim zuvor kontrolliert

Other: for which age group do you use this medication?
Alle

Other: which time schedule do you use? [Day(s) before VUS/VCUG, how many?]
Yes

Other: which time schedule do you use? [Day(s) before VUS/VCUG, how many?][Comment]
2-5 Tage vorher

Other: which time schedule do you use? [Day of VUS/VCUG]
No

Other: which time schedule do you use? [Day of VUS/VCUG][Comment]

Other: which time schedule do you use? [Day(s) after VUS/VCUG, how many?]
No

Other: which time schedule do you use? [Day(s) after VUS/VCUG, how many?][Comment]

Indication for prophylaxis

Which patients receive periinterventional antibiotic prophylaxis? (multiple answers possible) [all]
No

Which patients receive periinterventional antibiotic prophylaxis? (multiple answers possible) [patients post urinary tract infection/pyelonephritis/urosepsis]
Yes

Which patients receive periinterventional antibiotic prophylaxis? (multiple answers possible) [patients with urinary tract dilation in ultrasound]
No

Which patients receive periinterventional antibiotic prophylaxis? (multiple answers possible) [patients with prenatal urinary tract dilation]
No

Which patients receive periinterventional antibiotic prophylaxis? (multiple answers possible) [patients post VUR surgery]
No

Which patients receive periinterventional antibiotic prophylaxis? (multiple answers possible) [none]
Yes

Which patients receive periinterventional antibiotic prophylaxis? (multiple answers possible) [Other]

patients status post urinary tract infection/pyelonephritis/urosepsis: what is the minimum time between infection and VUS/VCUG? (in days)
5 Tage

Are there situations or clinical findings in the patient where you deviate from your prophylaxis standard (medication, dose, duration, age of the patient)? (multiple answers possible, please specify in answer box) [in case of already known vesicoureteral reflux (VUR)]
No

Are there situations or clinical findings in the patient where you deviate from your prophylaxis standard (medication, dose, duration, age of the patient)? (multiple answers possible, please specify in answer box) [in case of already known vesicoureteral reflux (VUR)][Comment]

Are there situations or clinical findings in the patient where you deviate from your prophylaxis standard (medication, dose, duration, age of the patient)? (multiple answers possible, please specify in answer box) [in patients with recurrent urinary tract infections (UTI)]
Yes

Are there situations or clinical findings in the patient where you deviate from your prophylaxis standard (medication, dose, duration, age of the patient)? (multiple answers possible, please specify in answer box) [in patients with recurrent urinary tract infections (UTI)][Comment]

Are there situations or clinical findings in the patient where you deviate from your prophylaxis standard (medication, dose, duration, age of the patient)? (multiple answers possible, please specify in answer box) [if multidrug-resistant organisms (MDRO) are detected in urine cultures]
Yes

Are there situations or clinical findings in the patient where you deviate from your prophylaxis standard (medication, dose, duration, age of the patient)? (multiple answers possible, please specify in answer box) [if multidrug-resistant organisms (MDRO) are detected in urine cultures][Comment]

Are there situations or clinical findings in the patient where you deviate from your prophylaxis standard (medication, dose, duration, age of the patient)? (multiple answers possible, please specify in answer box) [in newborns]
No

Are there situations or clinical findings in the patient where you deviate from your prophylaxis standard (medication, dose, duration, age of the patient)? (multiple answers possible, please specify in answer box) [in newborns][Comment]

Are there situations or clinical findings in the patient where you deviate from your prophylaxis standard (medication, dose, duration, age of the patient)? (multiple answers possible, please specify in answer box) [Other]

Are there situations or clinical findings in the patient where you deviate from your prophylaxis standard (medication, dose, duration, age of the patient)? (multiple answers possible, please specify in answer box) [Other comment]

Are there situations in which you use intravenous prophylaxis?
Yes

In which situations?
Abhängig von Keim, keine oralen Medis verfügbar. Pat kann nicht Medis schlucken.

Voiding analysis

Are there differences in antibiotic prophylaxis between VUS and VCUG in your department?
No

Is a non-pathological urine strip test required in preparation for VUS/VCUG in your department?
Yes

What is the maximum amount of days accepted for urine strip test to be performed prior to the exam?
7

Is a non-pathological urine culture required in preparation for VUS/VCUG in your department?
Yes

What is the maximum amount of days accepted for urine culture to be performed prior to the exam?
7 d

Do patients only receive post-procedure prophylaxis if vesicoureteral reflux (VUR) is detected in VUS/VCUG?
No

Do other MUS/MCU findings (e.g. urethral valves) have an influence on post-procedure prophylaxis?
No

End of survey

Thank you for taking the time to answer this survey!

May we contact you personally if we have further questions?
Yes

E-mail address:
ouga98@hotmail.com

Survey response 244

Response ID
246

Last page
2

Start language
de

Seed
1610538404

Date started
2025-04-15 08:02:04

Date last action
2025-04-15 08:03:42

Standard Operation Procedure

Who fills out this survey?
Pediatric urologist

Who fills out this survey? [Other]

In which type of hospital are you employed?
University Hospital

In which type of hospital are you employed? [Other]

In which country do you work?
Germany

Does your department have a defined protocol for infection prophylaxis in the context of VUS/VCUG?
No

Has this protocol been transformed into a written standard operating procedure (SOP)?
N/A

Antibiotic Prophylaxis

Are there situations/constellations in which you do not use antibiotic prophylaxis for VUS/VCUG?
Yes

Please specify these situations/constellations: [girl with first uncomplicated UTI without urinary tract dilation in ultrasound]
No

Please specify these situations/constellations: [boy with first uncomplicated UTI without urinary tract dilation in ultrasound]
No

Please specify these situations/constellations: [in patients with low grade urinary tract dilation (up to 2nd grade) and no UTI in medical history]
No

Please specify these situations/constellations: [Other]
Mcu v.a. zur Harnröhrendarstellung

If you use antibiotic prophylaxis: which mediaction do you use? (multiple answers and subdivision into age groups possible) [Trimethoprim]
No

If you use antibiotic prophylaxis: which mediaction do you use? (multiple answers and subdivision into age groups possible) [Nitrofurantoin]
No

If you use antibiotic prophylaxis: which mediaction do you use? (multiple answers and subdivision into age groups possible) [Amoxicillin]
No

If you use antibiotic prophylaxis: which mediaction do you use? (multiple answers and subdivision into age groups possible) [Cefaclor]
Yes

If you use antibiotic prophylaxis: which mediaction do you use? (multiple answers and subdivision into age groups possible) [Amoxicillin/clavulanic acid]
No

If you use antibiotic prophylaxis: which mediaction do you use? (multiple answers and subdivision into age groups possible) [Other]
No

Trimethoprim: which time schedule do you use? [Day(s) before VUS/VCUG, how many?]
N/A

Trimethoprim: which time schedule do you use? [Day of VUS/VCUG]
N/A

Trimethoprim: which time schedule do you use? [Day(s) after VUS/VCUG, how many?]
N/A

Nitrofurantoin: which time schedule do you use? [Day(s) before VUS/VCUG, how many?]
N/A

Nitrofurantoin: which time schedule do you use? [Day of VUS/VCUG]
N/A

Nitrofurantoin: which time schedule do you use? [Day(s) after VUS/VCUG, how many?]
N/A

Amoxicillin: which time schedule do you use? [Day(s) before VUS/VCUG, how many?]
N/A

Amoxicillin: which time schedule do you use? [Day of VUS/VCUG]
N/A

Amoxicillin: which time schedule do you use? [Day(s) after VUS/VCUG, how many?]
N/A

Cefaclor: in what dosage?
prophylactic (10 mg/kg bodyweight/d)

Cefaclor: in what dosage? [Other]

Cefaclor: for which age group do you use cefaclor?
0-18

Cefaclor: which time schedule do you use? [Days(s) before VUS/VCUG, how many?]
No

Cefaclor: which time schedule do you use? [Days(s) before VUS/VCUG, how many?][Comment]

Cefaclor: which time schedule do you use? [Day of VUS/VCUG]
Yes

Cefaclor: which time schedule do you use? [Day of VUS/VCUG][Comment]

Cefaclor: which time schedule do you use? [Day(s) after VUS/VCUG, how many?]
No

Cefaclor: which time schedule do you use? [Day(s) after VUS/VCUG, how many?][Comment]

Amoxicillin/clavulanic acid: in what dosage? (Amoxicillin portion) [prophylaktic (10-20 mg/Kg bodyweight/d)]
N/A

Amoxicillin/clavulanic acid: in what dosage? (Amoxicillin portion) [double prophylactic (20-40 mg/Kg bodyweight/d)]
N/A

Amoxicillin/clavulanic acid: in what dosage? (Amoxicillin portion) [therapeutic (80-100 mg/Kg bodyweight/d)]
N/A

Amoxicillin/clavulanic acid: which time schedule do you use? [Day(s) before VUS/VCUG]
N/A

Amoxicillin/clavulanic acid: which time schedule do you use? [Day of VUS/VCUG]
N/A

Amoxicillin/clavulanic acid: which time schedule do you use? [Day(s) after VUS/VCUG]
N/A

Other: which time schedule do you use? [Day(s) before VUS/VCUG, how many?]
N/A

Other: which time schedule do you use? [Day of VUS/VCUG]
N/A

Other: which time schedule do you use? [Day(s) after VUS/VCUG, how many?]
N/A

Indication for prophylaxis

Which patients receive periinterventional antibiotic prophylaxis? (multiple answers possible) [all]
N/A

Which patients receive periinterventional antibiotic prophylaxis? (multiple answers possible) [patients post urinary tract infection/pyelonephritis/urosepsis]
N/A

Which patients receive periinterventional antibiotic prophylaxis? (multiple answers possible) [patients with urinary tract dilation in ultrasound]
N/A

Which patients receive periinterventional antibiotic prophylaxis? (multiple answers possible) [patients with prenatal urinary tract dilation]
N/A

Which patients receive periinterventional antibiotic prophylaxis? (multiple answers possible) [patients post VUR surgery]
N/A

Which patients receive periinterventional antibiotic prophylaxis? (multiple answers possible) [none]
N/A

Are there situations or clinical findings in the patient where you deviate from your prophylaxis standard (medication, dose, duration, age of the patient)? (multiple answers possible, please specify in answer box) [in case of already known vesicoureteral reflux (VUR)]
N/A

Are there situations or clinical findings in the patient where you deviate from your prophylaxis standard (medication, dose, duration, age of the patient)? (multiple answers possible, please specify in answer box) [in patients with recurrent urinary tract infections (UTI)]
N/A

Are there situations or clinical findings in the patient where you deviate from your prophylaxis standard (medication, dose, duration, age of the patient)? (multiple answers possible, please specify in answer box) [if multidrug-resistant organisms (MDRO) are detected in urine cultures]
N/A

Are there situations or clinical findings in the patient where you deviate from your prophylaxis standard (medication, dose, duration, age of the patient)? (multiple answers possible, please specify in answer box) [in newborns]
N/A

Are there situations in which you use intravenous prophylaxis?
N/A

Voiding analysis

Are there differences in antibiotic prophylaxis between VUS and VCUG in your department?
N/A

Is a non-pathological urine strip test required in preparation for VUS/VCUG in your department?
N/A

Is a non-pathological urine culture required in preparation for VUS/VCUG in your department?
N/A

Do patients only receive post-procedure prophylaxis if vesicoureteral reflux (VUR) is detected in VUS/VCUG?
N/A

Do other MUS/MCU findings (e.g. urethral valves) have an influence on post-procedure prophylaxis?
N/A

End of survey

May we contact you personally if we have further questions?
N/A

Survey response 245

Response ID
247

Last page
2

Start language
de

Seed
1907191199

Date started
2025-04-15 08:26:25

Date last action
2025-04-15 08:29:50

Standard Operation Procedure

Who fills out this survey?
Pediatric Surgeon

Who fills out this survey? [Other]

In which type of hospital are you employed?
University Hospital

In which type of hospital are you employed? [Other]

In which country do you work?
Deutschland

Does your department have a defined protocol for infection prophylaxis in the context of VUS/VCUG?
Yes

Has this protocol been transformed into a written standard operating procedure (SOP)?
No

Antibiotic Prophylaxis

Are there situations/constellations in which you do not use antibiotic prophylaxis for VUS/VCUG?
Yes

Please specify these situations/constellations: [girl with first uncomplicated UTI without urinary tract dilation in ultrasound]
No

Please specify these situations/constellations: [boy with first uncomplicated UTI without urinary tract dilation in ultrasound]
No

Please specify these situations/constellations: [in patients with low grade urinary tract dilation (up to 2nd grade) and no UTI in medical history]
Yes

Please specify these situations/constellations: [Other]

If you use antibiotic prophylaxis: which mediaction do you use? (multiple answers and subdivision into age groups possible) [Trimethoprim]
No

If you use antibiotic prophylaxis: which mediaction do you use? (multiple answers and subdivision into age groups possible) [Nitrofurantoin]
No

If you use antibiotic prophylaxis: which mediaction do you use? (multiple answers and subdivision into age groups possible) [Amoxicillin]
Yes

If you use antibiotic prophylaxis: which mediaction do you use? (multiple answers and subdivision into age groups possible) [Cefaclor]
Yes

If you use antibiotic prophylaxis: which mediaction do you use? (multiple answers and subdivision into age groups possible) [Amoxicillin/clavulanic acid]
No

If you use antibiotic prophylaxis: which mediaction do you use? (multiple answers and subdivision into age groups possible) [Other]
No

Trimethoprim: which time schedule do you use? [Day(s) before VUS/VCUG, how many?]
N/A

Trimethoprim: which time schedule do you use? [Day of VUS/VCUG]
N/A

Trimethoprim: which time schedule do you use? [Day(s) after VUS/VCUG, how many?]
N/A

Nitrofurantoin: which time schedule do you use? [Day(s) before VUS/VCUG, how many?]
N/A

Nitrofurantoin: which time schedule do you use? [Day of VUS/VCUG]
N/A

Nitrofurantoin: which time schedule do you use? [Day(s) after VUS/VCUG, how many?]
N/A

Amoxicillin: in what dosage?
therapeutic (50-100 mg/kg bodyweight/d)

Amoxicillin: in what dosage? [Other]

Amoxicillin: for which age group do you use amoxicillin?
Säuglinge unter 2 Wochen

Amoxicillin: which time schedule do you use? [Day(s) before VUS/VCUG, how many?]
No

Amoxicillin: which time schedule do you use? [Day(s) before VUS/VCUG, how many?][Comment]

Amoxicillin: which time schedule do you use? [Day of VUS/VCUG]
Yes

Amoxicillin: which time schedule do you use? [Day of VUS/VCUG][Comment]

Amoxicillin: which time schedule do you use? [Day(s) after VUS/VCUG, how many?]
Yes

Amoxicillin: which time schedule do you use? [Day(s) after VUS/VCUG, how many?][Comment]
2

Cefaclor: in what dosage?
double prophylactic (20 mg/kg bodyweight/d)

Cefaclor: in what dosage? [Other]

Cefaclor: for which age group do you use cefaclor?
Säuglinge - 6Wochen

Cefaclor: which time schedule do you use? [Days(s) before VUS/VCUG, how many?]
No

Cefaclor: which time schedule do you use? [Days(s) before VUS/VCUG, how many?][Comment]

Cefaclor: which time schedule do you use? [Day of VUS/VCUG]
Yes

Cefaclor: which time schedule do you use? [Day of VUS/VCUG][Comment]

Cefaclor: which time schedule do you use? [Day(s) after VUS/VCUG, how many?]
Yes

Cefaclor: which time schedule do you use? [Day(s) after VUS/VCUG, how many?][Comment]
2

Amoxicillin/clavulanic acid: in what dosage? (Amoxicillin portion) [prophylaktic (10-20 mg/Kg bodyweight/d)]
N/A

Amoxicillin/clavulanic acid: in what dosage? (Amoxicillin portion) [double prophylactic (20-40 mg/Kg bodyweight/d)]
N/A

Amoxicillin/clavulanic acid: in what dosage? (Amoxicillin portion) [therapeutic (80-100 mg/Kg bodyweight/d)]
N/A

Amoxicillin/clavulanic acid: which time schedule do you use? [Day(s) before VUS/VCUG]
N/A

Amoxicillin/clavulanic acid: which time schedule do you use? [Day of VUS/VCUG]
N/A

Amoxicillin/clavulanic acid: which time schedule do you use? [Day(s) after VUS/VCUG]
N/A

Other: which time schedule do you use? [Day(s) before VUS/VCUG, how many?]
N/A

Other: which time schedule do you use? [Day of VUS/VCUG]
N/A

Other: which time schedule do you use? [Day(s) after VUS/VCUG, how many?]
N/A

Indication for prophylaxis

Which patients receive periinterventional antibiotic prophylaxis? (multiple answers possible) [all]
N/A

Which patients receive periinterventional antibiotic prophylaxis? (multiple answers possible) [patients post urinary tract infection/pyelonephritis/urosepsis]
N/A

Which patients receive periinterventional antibiotic prophylaxis? (multiple answers possible) [patients with urinary tract dilation in ultrasound]
N/A

Which patients receive periinterventional antibiotic prophylaxis? (multiple answers possible) [patients with prenatal urinary tract dilation]
N/A

Which patients receive periinterventional antibiotic prophylaxis? (multiple answers possible) [patients post VUR surgery]
N/A

Which patients receive periinterventional antibiotic prophylaxis? (multiple answers possible) [none]
N/A

Are there situations or clinical findings in the patient where you deviate from your prophylaxis standard (medication, dose, duration, age of the patient)? (multiple answers possible, please specify in answer box) [in case of already known vesicoureteral reflux (VUR)]
N/A

Are there situations or clinical findings in the patient where you deviate from your prophylaxis standard (medication, dose, duration, age of the patient)? (multiple answers possible, please specify in answer box) [in patients with recurrent urinary tract infections (UTI)]
N/A

Are there situations or clinical findings in the patient where you deviate from your prophylaxis standard (medication, dose, duration, age of the patient)? (multiple answers possible, please specify in answer box) [if multidrug-resistant organisms (MDRO) are detected in urine cultures]
N/A

Are there situations or clinical findings in the patient where you deviate from your prophylaxis standard (medication, dose, duration, age of the patient)? (multiple answers possible, please specify in answer box) [in newborns]
N/A

Are there situations in which you use intravenous prophylaxis?
N/A

Voiding analysis

Are there differences in antibiotic prophylaxis between VUS and VCUG in your department?
N/A

Is a non-pathological urine strip test required in preparation for VUS/VCUG in your department?
N/A

Is a non-pathological urine culture required in preparation for VUS/VCUG in your department?
N/A

Do patients only receive post-procedure prophylaxis if vesicoureteral reflux (VUR) is detected in VUS/VCUG?
N/A

Do other MUS/MCU findings (e.g. urethral valves) have an influence on post-procedure prophylaxis?
N/A

End of survey

May we contact you personally if we have further questions?
N/A

Survey response 246

Response ID
248

Date submitted
2025-04-15 08:39:55

Last page
5

Start language
de

Seed
2080412860

Date started
2025-04-15 08:30:23

Date last action
2025-04-15 08:39:55

Standard Operation Procedure

Who fills out this survey?
Pediatric Surgeon

Who fills out this survey? [Other]

In which type of hospital are you employed?
University Hospital

In which type of hospital are you employed? [Other]

In which country do you work?
Deutschland

Does your department have a defined protocol for infection prophylaxis in the context of VUS/VCUG?
Yes

Has this protocol been transformed into a written standard operating procedure (SOP)?
No

Antibiotic Prophylaxis

Are there situations/constellations in which you do not use antibiotic prophylaxis for VUS/VCUG?
Yes

Please specify these situations/constellations: [girl with first uncomplicated UTI without urinary tract dilation in ultrasound]
No

Please specify these situations/constellations: [boy with first uncomplicated UTI without urinary tract dilation in ultrasound]
No

Please specify these situations/constellations: [in patients with low grade urinary tract dilation (up to 2nd grade) and no UTI in medical history]
Yes

Please specify these situations/constellations: [Other]

If you use antibiotic prophylaxis: which mediaction do you use? (multiple answers and subdivision into age groups possible) [Trimethoprim]
Yes

If you use antibiotic prophylaxis: which mediaction do you use? (multiple answers and subdivision into age groups possible) [Nitrofurantoin]
Yes

If you use antibiotic prophylaxis: which mediaction do you use? (multiple answers and subdivision into age groups possible) [Amoxicillin]
Yes

If you use antibiotic prophylaxis: which mediaction do you use? (multiple answers and subdivision into age groups possible) [Cefaclor]
Yes

If you use antibiotic prophylaxis: which mediaction do you use? (multiple answers and subdivision into age groups possible) [Amoxicillin/clavulanic acid]
No

If you use antibiotic prophylaxis: which mediaction do you use? (multiple answers and subdivision into age groups possible) [Other]
No

Trimethoprim: in what dosage?
prophylactic (2 mg/kg bodyweight/d)

Trimethoprim: in what dosage? [Other]

Trimethoprim: for which age group do you use trimethoprim?
Kinder älter 6 Wochen, jünger als 4 Monate, Unverträglichkeit Nitrofurantoin

Trimethoprim: which time schedule do you use? [Day(s) before VUS/VCUG, how many?]
No

Trimethoprim: which time schedule do you use? [Day(s) before VUS/VCUG, how many?][Comment]

Trimethoprim: which time schedule do you use? [Day of VUS/VCUG]
Yes

Trimethoprim: which time schedule do you use? [Day of VUS/VCUG][Comment]

Trimethoprim: which time schedule do you use? [Day(s) after VUS/VCUG, how many?]
Yes

Trimethoprim: which time schedule do you use? [Day(s) after VUS/VCUG, how many?][Comment]
2

Nitrofurantoin: in what dosage?
prophylactic (1-2 mg/kg bodyweight/d)

Nitrofurantoin: in what dosage? [Other]

Nitrofurantoin: for which age group do you use nitrofurantoin?
Kinder >4 Monate

Nitrofurantoin: which time schedule do you use? [Day(s) before VUS/VCUG, how many?]
Yes

Nitrofurantoin: which time schedule do you use? [Day(s) before VUS/VCUG, how many?][Comment]
1 Vorabend der Untersuchung

Nitrofurantoin: which time schedule do you use? [Day of VUS/VCUG]
Yes

Nitrofurantoin: which time schedule do you use? [Day of VUS/VCUG][Comment]

Nitrofurantoin: which time schedule do you use? [Day(s) after VUS/VCUG, how many?]
Yes

Nitrofurantoin: which time schedule do you use? [Day(s) after VUS/VCUG, how many?][Comment]
2

Amoxicillin: in what dosage?
prophylactic (10-20 mg/kg bodyweight/d)

Amoxicillin: in what dosage? [Other]

Amoxicillin: for which age group do you use amoxicillin?
Säuglinge unter 2 Wochen

Amoxicillin: which time schedule do you use? [Day(s) before VUS/VCUG, how many?]
No

Amoxicillin: which time schedule do you use? [Day(s) before VUS/VCUG, how many?][Comment]

Amoxicillin: which time schedule do you use? [Day of VUS/VCUG]
Yes

Amoxicillin: which time schedule do you use? [Day of VUS/VCUG][Comment]

Amoxicillin: which time schedule do you use? [Day(s) after VUS/VCUG, how many?]
Yes

Amoxicillin: which time schedule do you use? [Day(s) after VUS/VCUG, how many?][Comment]
2

Cefaclor: in what dosage?
prophylactic (10 mg/kg bodyweight/d)

Cefaclor: in what dosage? [Other]

Cefaclor: for which age group do you use cefaclor?
Säuglinge 2-6 Wochen

Cefaclor: which time schedule do you use? [Days(s) before VUS/VCUG, how many?]
No

Cefaclor: which time schedule do you use? [Days(s) before VUS/VCUG, how many?][Comment]

Cefaclor: which time schedule do you use? [Day of VUS/VCUG]
Yes

Cefaclor: which time schedule do you use? [Day of VUS/VCUG][Comment]

Cefaclor: which time schedule do you use? [Day(s) after VUS/VCUG, how many?]
Yes

Cefaclor: which time schedule do you use? [Day(s) after VUS/VCUG, how many?][Comment]
2

Amoxicillin/clavulanic acid: in what dosage? (Amoxicillin portion) [prophylaktic (10-20 mg/Kg bodyweight/d)]
N/A

Amoxicillin/clavulanic acid: in what dosage? (Amoxicillin portion) [double prophylactic (20-40 mg/Kg bodyweight/d)]
N/A

Amoxicillin/clavulanic acid: in what dosage? (Amoxicillin portion) [therapeutic (80-100 mg/Kg bodyweight/d)]
N/A

Amoxicillin/clavulanic acid: which time schedule do you use? [Day(s) before VUS/VCUG]
N/A

Amoxicillin/clavulanic acid: which time schedule do you use? [Day of VUS/VCUG]
N/A

Amoxicillin/clavulanic acid: which time schedule do you use? [Day(s) after VUS/VCUG]
N/A

Other: which time schedule do you use? [Day(s) before VUS/VCUG, how many?]
N/A

Other: which time schedule do you use? [Day of VUS/VCUG]
N/A

Other: which time schedule do you use? [Day(s) after VUS/VCUG, how many?]
N/A

Indication for prophylaxis

Which patients receive periinterventional antibiotic prophylaxis? (multiple answers possible) [all]
No

Which patients receive periinterventional antibiotic prophylaxis? (multiple answers possible) [patients post urinary tract infection/pyelonephritis/urosepsis]
Yes

Which patients receive periinterventional antibiotic prophylaxis? (multiple answers possible) [patients with urinary tract dilation in ultrasound]
Yes

Which patients receive periinterventional antibiotic prophylaxis? (multiple answers possible) [patients with prenatal urinary tract dilation]
Yes

Which patients receive periinterventional antibiotic prophylaxis? (multiple answers possible) [patients post VUR surgery]
Yes

Which patients receive periinterventional antibiotic prophylaxis? (multiple answers possible) [none]
No

Which patients receive periinterventional antibiotic prophylaxis? (multiple answers possible) [Other]

patients status post urinary tract infection/pyelonephritis/urosepsis: what is the minimum time between infection and VUS/VCUG? (in days)
14

In patients with urinary tract dilation in ultrasound: from what degree on? Image from Beetz R, Bokenkamp A, Brandis M et al (2001) Diagnosis of congenital dilatation of the urinary tract. Consensus Group of the Pediatric Nephrology Working Society in cooperation with the Pediatric Urology Working Group of the German Society of Urology and with the Pediatric Urology Working Society in the Germany Society of Pediatric Surgery. Urologe A 40:495–507 (quiz 8–9)
grade 2

In patients with prenatal urinary tract dilation: from what degree on? Image from Beetz R, Bokenkamp A, Brandis M et al (2001) Diagnosis of congenital dilatation of the urinary tract. Consensus Group of the Pediatric Nephrology Working Society in cooperation with the Pediatric Urology Working Group of the German Society of Urology and with the Pediatric Urology Working Society in the Germany Society of Pediatric Surgery. Urologe A 40:495–507 (quiz 8–9)
grade 2

Are there situations or clinical findings in the patient where you deviate from your prophylaxis standard (medication, dose, duration, age of the patient)? (multiple answers possible, please specify in answer box) [in case of already known vesicoureteral reflux (VUR)]
No

Are there situations or clinical findings in the patient where you deviate from your prophylaxis standard (medication, dose, duration, age of the patient)? (multiple answers possible, please specify in answer box) [in case of already known vesicoureteral reflux (VUR)][Comment]

Are there situations or clinical findings in the patient where you deviate from your prophylaxis standard (medication, dose, duration, age of the patient)? (multiple answers possible, please specify in answer box) [in patients with recurrent urinary tract infections (UTI)]
No

Are there situations or clinical findings in the patient where you deviate from your prophylaxis standard (medication, dose, duration, age of the patient)? (multiple answers possible, please specify in answer box) [in patients with recurrent urinary tract infections (UTI)][Comment]

Are there situations or clinical findings in the patient where you deviate from your prophylaxis standard (medication, dose, duration, age of the patient)? (multiple answers possible, please specify in answer box) [if multidrug-resistant organisms (MDRO) are detected in urine cultures]
Yes

Are there situations or clinical findings in the patient where you deviate from your prophylaxis standard (medication, dose, duration, age of the patient)? (multiple answers possible, please specify in answer box) [if multidrug-resistant organisms (MDRO) are detected in urine cultures][Comment]
resistogrammgerechte Prophylaxe

Are there situations or clinical findings in the patient where you deviate from your prophylaxis standard (medication, dose, duration, age of the patient)? (multiple answers possible, please specify in answer box) [in newborns]
No

Are there situations or clinical findings in the patient where you deviate from your prophylaxis standard (medication, dose, duration, age of the patient)? (multiple answers possible, please specify in answer box) [in newborns][Comment]

Are there situations or clinical findings in the patient where you deviate from your prophylaxis standard (medication, dose, duration, age of the patient)? (multiple answers possible, please specify in answer box) [Other]

Are there situations or clinical findings in the patient where you deviate from your prophylaxis standard (medication, dose, duration, age of the patient)? (multiple answers possible, please specify in answer box) [Other comment]

Are there situations in which you use intravenous prophylaxis?
Yes

In which situations?
Säuglinge

Voiding analysis

Are there differences in antibiotic prophylaxis between VUS and VCUG in your department?
No

Is a non-pathological urine strip test required in preparation for VUS/VCUG in your department?
No

Is a non-pathological urine culture required in preparation for VUS/VCUG in your department?
No

Do patients only receive post-procedure prophylaxis if vesicoureteral reflux (VUR) is detected in VUS/VCUG?
No

Do other MUS/MCU findings (e.g. urethral valves) have an influence on post-procedure prophylaxis?
No

End of survey

Thank you for taking the time to answer this survey!

May we contact you personally if we have further questions?
Yes

E-mail address:
karima-muna.amasheh@elisabethgruppe.de

Survey response 247

Response ID
249

Date submitted
2025-04-15 10:28:58

Last page
5

Start language
de

Seed
380364949

Date started
2025-04-15 10:25:41

Date last action
2025-04-15 10:28:58

Standard Operation Procedure

Who fills out this survey?
Pediatric urologist

Who fills out this survey? [Other]

In which type of hospital are you employed?
Community hospital (

In which type of hospital are you employed? [Other]

In which country do you work?
Z

Does your department have a defined protocol for infection prophylaxis in the context of VUS/VCUG?
Yes

Has this protocol been transformed into a written standard operating procedure (SOP)?
Yes

Which department is the author of the SOP for infection prophylaxis in VUS/VCUG?
Other

Which department is the author of the SOP for infection prophylaxis in VUS/VCUG? [Other]
Urologie

Antibiotic Prophylaxis

Are there situations/constellations in which you do not use antibiotic prophylaxis for VUS/VCUG?
Yes

Please specify these situations/constellations: [girl with first uncomplicated UTI without urinary tract dilation in ultrasound]
No

Please specify these situations/constellations: [boy with first uncomplicated UTI without urinary tract dilation in ultrasound]
No

Please specify these situations/constellations: [in patients with low grade urinary tract dilation (up to 2nd grade) and no UTI in medical history]
No

Please specify these situations/constellations: [Other]
Bestehende therapeutische Dosierung

If you use antibiotic prophylaxis: which mediaction do you use? (multiple answers and subdivision into age groups possible) [Trimethoprim]
Yes

If you use antibiotic prophylaxis: which mediaction do you use? (multiple answers and subdivision into age groups possible) [Nitrofurantoin]
No

If you use antibiotic prophylaxis: which mediaction do you use? (multiple answers and subdivision into age groups possible) [Amoxicillin]
No

If you use antibiotic prophylaxis: which mediaction do you use? (multiple answers and subdivision into age groups possible) [Cefaclor]
No

If you use antibiotic prophylaxis: which mediaction do you use? (multiple answers and subdivision into age groups possible) [Amoxicillin/clavulanic acid]
No

If you use antibiotic prophylaxis: which mediaction do you use? (multiple answers and subdivision into age groups possible) [Other]
No

Trimethoprim: in what dosage?
prophylactic (2 mg/kg bodyweight/d)

Trimethoprim: in what dosage? [Other]

Trimethoprim: for which age group do you use trimethoprim?
Ab 6 wochen

Trimethoprim: which time schedule do you use? [Day(s) before VUS/VCUG, how many?]
No

Trimethoprim: which time schedule do you use? [Day(s) before VUS/VCUG, how many?][Comment]

Trimethoprim: which time schedule do you use? [Day of VUS/VCUG]
Yes

Trimethoprim: which time schedule do you use? [Day of VUS/VCUG][Comment]

Trimethoprim: which time schedule do you use? [Day(s) after VUS/VCUG, how many?]
No

Trimethoprim: which time schedule do you use? [Day(s) after VUS/VCUG, how many?][Comment]

Nitrofurantoin: which time schedule do you use? [Day(s) before VUS/VCUG, how many?]
N/A

Nitrofurantoin: which time schedule do you use? [Day of VUS/VCUG]
N/A

Nitrofurantoin: which time schedule do you use? [Day(s) after VUS/VCUG, how many?]
N/A

Amoxicillin: which time schedule do you use? [Day(s) before VUS/VCUG, how many?]
N/A

Amoxicillin: which time schedule do you use? [Day of VUS/VCUG]
N/A

Amoxicillin: which time schedule do you use? [Day(s) after VUS/VCUG, how many?]
N/A

Cefaclor: which time schedule do you use? [Days(s) before VUS/VCUG, how many?]
N/A

Cefaclor: which time schedule do you use? [Day of VUS/VCUG]
N/A

Cefaclor: which time schedule do you use? [Day(s) after VUS/VCUG, how many?]
N/A

Amoxicillin/clavulanic acid: in what dosage? (Amoxicillin portion) [prophylaktic (10-20 mg/Kg bodyweight/d)]
N/A

Amoxicillin/clavulanic acid: in what dosage? (Amoxicillin portion) [double prophylactic (20-40 mg/Kg bodyweight/d)]
N/A

Amoxicillin/clavulanic acid: in what dosage? (Amoxicillin portion) [therapeutic (80-100 mg/Kg bodyweight/d)]
N/A

Amoxicillin/clavulanic acid: which time schedule do you use? [Day(s) before VUS/VCUG]
N/A

Amoxicillin/clavulanic acid: which time schedule do you use? [Day of VUS/VCUG]
N/A

Amoxicillin/clavulanic acid: which time schedule do you use? [Day(s) after VUS/VCUG]
N/A

Other: which time schedule do you use? [Day(s) before VUS/VCUG, how many?]
N/A

Other: which time schedule do you use? [Day of VUS/VCUG]
N/A

Other: which time schedule do you use? [Day(s) after VUS/VCUG, how many?]
N/A

Indication for prophylaxis

Which patients receive periinterventional antibiotic prophylaxis? (multiple answers possible) [all]
Yes

Which patients receive periinterventional antibiotic prophylaxis? (multiple answers possible) [patients post urinary tract infection/pyelonephritis/urosepsis]
N/A

Which patients receive periinterventional antibiotic prophylaxis? (multiple answers possible) [patients with urinary tract dilation in ultrasound]
N/A

Which patients receive periinterventional antibiotic prophylaxis? (multiple answers possible) [patients with prenatal urinary tract dilation]
N/A

Which patients receive periinterventional antibiotic prophylaxis? (multiple answers possible) [patients post VUR surgery]
N/A

Which patients receive periinterventional antibiotic prophylaxis? (multiple answers possible) [none]
N/A

Are there situations or clinical findings in the patient where you deviate from your prophylaxis standard (medication, dose, duration, age of the patient)? (multiple answers possible, please specify in answer box) [in case of already known vesicoureteral reflux (VUR)]
No

Are there situations or clinical findings in the patient where you deviate from your prophylaxis standard (medication, dose, duration, age of the patient)? (multiple answers possible, please specify in answer box) [in case of already known vesicoureteral reflux (VUR)][Comment]

Are there situations or clinical findings in the patient where you deviate from your prophylaxis standard (medication, dose, duration, age of the patient)? (multiple answers possible, please specify in answer box) [in patients with recurrent urinary tract infections (UTI)]
No

Are there situations or clinical findings in the patient where you deviate from your prophylaxis standard (medication, dose, duration, age of the patient)? (multiple answers possible, please specify in answer box) [in patients with recurrent urinary tract infections (UTI)][Comment]

Are there situations or clinical findings in the patient where you deviate from your prophylaxis standard (medication, dose, duration, age of the patient)? (multiple answers possible, please specify in answer box) [if multidrug-resistant organisms (MDRO) are detected in urine cultures]
No

Are there situations or clinical findings in the patient where you deviate from your prophylaxis standard (medication, dose, duration, age of the patient)? (multiple answers possible, please specify in answer box) [if multidrug-resistant organisms (MDRO) are detected in urine cultures][Comment]

Are there situations or clinical findings in the patient where you deviate from your prophylaxis standard (medication, dose, duration, age of the patient)? (multiple answers possible, please specify in answer box) [in newborns]
Yes

Are there situations or clinical findings in the patient where you deviate from your prophylaxis standard (medication, dose, duration, age of the patient)? (multiple answers possible, please specify in answer box) [in newborns][Comment]
Cephalosporine

Are there situations or clinical findings in the patient where you deviate from your prophylaxis standard (medication, dose, duration, age of the patient)? (multiple answers possible, please specify in answer box) [Other]

Are there situations or clinical findings in the patient where you deviate from your prophylaxis standard (medication, dose, duration, age of the patient)? (multiple answers possible, please specify in answer box) [Other comment]

Are there situations in which you use intravenous prophylaxis?
No

Voiding analysis

Are there differences in antibiotic prophylaxis between VUS and VCUG in your department?
No

Is a non-pathological urine strip test required in preparation for VUS/VCUG in your department?
Yes

What is the maximum amount of days accepted for urine strip test to be performed prior to the exam?
1

Is a non-pathological urine culture required in preparation for VUS/VCUG in your department?
No

Do patients only receive post-procedure prophylaxis if vesicoureteral reflux (VUR) is detected in VUS/VCUG?
No

Do other MUS/MCU findings (e.g. urethral valves) have an influence on post-procedure prophylaxis?
No

End of survey

Thank you for taking the time to answer this survey!

May we contact you personally if we have further questions?
No

Survey response 248

Response ID
250

Last page
1

Start language
en

Seed
185808441

Date started
2025-04-15 18:20:44

Date last action
2025-04-15 18:21:18

Standard Operation Procedure

Who fills out this survey?
Pediatric Radiologist

Who fills out this survey? [Other]

In which type of hospital are you employed?
University Hospital

In which type of hospital are you employed? [Other]

In which country do you work?
Netherlands

Does your department have a defined protocol for infection prophylaxis in the context of VUS/VCUG?
Yes

Has this protocol been transformed into a written standard operating procedure (SOP)?
No

Antibiotic Prophylaxis

Are there situations/constellations in which you do not use antibiotic prophylaxis for VUS/VCUG?
N/A

Please specify these situations/constellations: [girl with first uncomplicated UTI without urinary tract dilation in ultrasound]
N/A

Please specify these situations/constellations: [boy with first uncomplicated UTI without urinary tract dilation in ultrasound]
N/A

Please specify these situations/constellations: [in patients with low grade urinary tract dilation (up to 2nd grade) and no UTI in medical history]
N/A

If you use antibiotic prophylaxis: which mediaction do you use? (multiple answers and subdivision into age groups possible) [Trimethoprim]
N/A

If you use antibiotic prophylaxis: which mediaction do you use? (multiple answers and subdivision into age groups possible) [Nitrofurantoin]
N/A

If you use antibiotic prophylaxis: which mediaction do you use? (multiple answers and subdivision into age groups possible) [Amoxicillin]
N/A

If you use antibiotic prophylaxis: which mediaction do you use? (multiple answers and subdivision into age groups possible) [Cefaclor]
N/A

If you use antibiotic prophylaxis: which mediaction do you use? (multiple answers and subdivision into age groups possible) [Amoxicillin/clavulanic acid]
N/A

If you use antibiotic prophylaxis: which mediaction do you use? (multiple answers and subdivision into age groups possible) [Other]
N/A

Trimethoprim: which time schedule do you use? [Day(s) before VUS/VCUG, how many?]
N/A

Trimethoprim: which time schedule do you use? [Day of VUS/VCUG]
N/A

Trimethoprim: which time schedule do you use? [Day(s) after VUS/VCUG, how many?]
N/A

Nitrofurantoin: which time schedule do you use? [Day(s) before VUS/VCUG, how many?]
N/A

Nitrofurantoin: which time schedule do you use? [Day of VUS/VCUG]
N/A

Nitrofurantoin: which time schedule do you use? [Day(s) after VUS/VCUG, how many?]
N/A

Amoxicillin: which time schedule do you use? [Day(s) before VUS/VCUG, how many?]
N/A

Amoxicillin: which time schedule do you use? [Day of VUS/VCUG]
N/A

Amoxicillin: which time schedule do you use? [Day(s) after VUS/VCUG, how many?]
N/A

Cefaclor: which time schedule do you use? [Days(s) before VUS/VCUG, how many?]
N/A

Cefaclor: which time schedule do you use? [Day of VUS/VCUG]
N/A

Cefaclor: which time schedule do you use? [Day(s) after VUS/VCUG, how many?]
N/A

Amoxicillin/clavulanic acid: in what dosage? (Amoxicillin portion) [prophylaktic (10-20 mg/Kg bodyweight/d)]
N/A

Amoxicillin/clavulanic acid: in what dosage? (Amoxicillin portion) [double prophylactic (20-40 mg/Kg bodyweight/d)]
N/A

Amoxicillin/clavulanic acid: in what dosage? (Amoxicillin portion) [therapeutic (80-100 mg/Kg bodyweight/d)]
N/A

Amoxicillin/clavulanic acid: which time schedule do you use? [Day(s) before VUS/VCUG]
N/A

Amoxicillin/clavulanic acid: which time schedule do you use? [Day of VUS/VCUG]
N/A

Amoxicillin/clavulanic acid: which time schedule do you use? [Day(s) after VUS/VCUG]
N/A

Other: which time schedule do you use? [Day(s) before VUS/VCUG, how many?]
N/A

Other: which time schedule do you use? [Day of VUS/VCUG]
N/A

Other: which time schedule do you use? [Day(s) after VUS/VCUG, how many?]
N/A

Indication for prophylaxis

Which patients receive periinterventional antibiotic prophylaxis? (multiple answers possible) [all]
N/A

Which patients receive periinterventional antibiotic prophylaxis? (multiple answers possible) [patients post urinary tract infection/pyelonephritis/urosepsis]
N/A

Which patients receive periinterventional antibiotic prophylaxis? (multiple answers possible) [patients with urinary tract dilation in ultrasound]
N/A

Which patients receive periinterventional antibiotic prophylaxis? (multiple answers possible) [patients with prenatal urinary tract dilation]
N/A

Which patients receive periinterventional antibiotic prophylaxis? (multiple answers possible) [patients post VUR surgery]
N/A

Which patients receive periinterventional antibiotic prophylaxis? (multiple answers possible) [none]
N/A

Are there situations or clinical findings in the patient where you deviate from your prophylaxis standard (medication, dose, duration, age of the patient)? (multiple answers possible, please specify in answer box) [in case of already known vesicoureteral reflux (VUR)]
N/A

Are there situations or clinical findings in the patient where you deviate from your prophylaxis standard (medication, dose, duration, age of the patient)? (multiple answers possible, please specify in answer box) [in patients with recurrent urinary tract infections (UTI)]
N/A

Are there situations or clinical findings in the patient where you deviate from your prophylaxis standard (medication, dose, duration, age of the patient)? (multiple answers possible, please specify in answer box) [if multidrug-resistant organisms (MDRO) are detected in urine cultures]
N/A

Are there situations or clinical findings in the patient where you deviate from your prophylaxis standard (medication, dose, duration, age of the patient)? (multiple answers possible, please specify in answer box) [in newborns]
N/A

Are there situations in which you use intravenous prophylaxis?
N/A

Voiding analysis

Are there differences in antibiotic prophylaxis between VUS and VCUG in your department?
N/A

Is a non-pathological urine strip test required in preparation for VUS/VCUG in your department?
N/A

Is a non-pathological urine culture required in preparation for VUS/VCUG in your department?
N/A

Do patients only receive post-procedure prophylaxis if vesicoureteral reflux (VUR) is detected in VUS/VCUG?
N/A

Do other MUS/MCU findings (e.g. urethral valves) have an influence on post-procedure prophylaxis?
N/A

End of survey

May we contact you personally if we have further questions?
N/A

Survey response 249

Response ID
251

Last page
0

Start language
en

Seed
814983977

Date started
2025-04-17 09:20:34

Date last action
2025-04-17 09:20:34

Standard Operation Procedure

Does your department have a defined protocol for infection prophylaxis in the context of VUS/VCUG?
N/A

Has this protocol been transformed into a written standard operating procedure (SOP)?
N/A

Antibiotic Prophylaxis

Are there situations/constellations in which you do not use antibiotic prophylaxis for VUS/VCUG?
N/A

Please specify these situations/constellations: [girl with first uncomplicated UTI without urinary tract dilation in ultrasound]
N/A

Please specify these situations/constellations: [boy with first uncomplicated UTI without urinary tract dilation in ultrasound]
N/A

Please specify these situations/constellations: [in patients with low grade urinary tract dilation (up to 2nd grade) and no UTI in medical history]
N/A

If you use antibiotic prophylaxis: which mediaction do you use? (multiple answers and subdivision into age groups possible) [Trimethoprim]
N/A

If you use antibiotic prophylaxis: which mediaction do you use? (multiple answers and subdivision into age groups possible) [Nitrofurantoin]
N/A

If you use antibiotic prophylaxis: which mediaction do you use? (multiple answers and subdivision into age groups possible) [Amoxicillin]
N/A

If you use antibiotic prophylaxis: which mediaction do you use? (multiple answers and subdivision into age groups possible) [Cefaclor]
N/A

If you use antibiotic prophylaxis: which mediaction do you use? (multiple answers and subdivision into age groups possible) [Amoxicillin/clavulanic acid]
N/A

If you use antibiotic prophylaxis: which mediaction do you use? (multiple answers and subdivision into age groups possible) [Other]
N/A

Trimethoprim: which time schedule do you use? [Day(s) before VUS/VCUG, how many?]
N/A

Trimethoprim: which time schedule do you use? [Day of VUS/VCUG]
N/A

Trimethoprim: which time schedule do you use? [Day(s) after VUS/VCUG, how many?]
N/A

Nitrofurantoin: which time schedule do you use? [Day(s) before VUS/VCUG, how many?]
N/A

Nitrofurantoin: which time schedule do you use? [Day of VUS/VCUG]
N/A

Nitrofurantoin: which time schedule do you use? [Day(s) after VUS/VCUG, how many?]
N/A

Amoxicillin: which time schedule do you use? [Day(s) before VUS/VCUG, how many?]
N/A

Amoxicillin: which time schedule do you use? [Day of VUS/VCUG]
N/A

Amoxicillin: which time schedule do you use? [Day(s) after VUS/VCUG, how many?]
N/A

Cefaclor: which time schedule do you use? [Days(s) before VUS/VCUG, how many?]
N/A

Cefaclor: which time schedule do you use? [Day of VUS/VCUG]
N/A

Cefaclor: which time schedule do you use? [Day(s) after VUS/VCUG, how many?]
N/A

Amoxicillin/clavulanic acid: in what dosage? (Amoxicillin portion) [prophylaktic (10-20 mg/Kg bodyweight/d)]
N/A

Amoxicillin/clavulanic acid: in what dosage? (Amoxicillin portion) [double prophylactic (20-40 mg/Kg bodyweight/d)]
N/A

Amoxicillin/clavulanic acid: in what dosage? (Amoxicillin portion) [therapeutic (80-100 mg/Kg bodyweight/d)]
N/A

Amoxicillin/clavulanic acid: which time schedule do you use? [Day(s) before VUS/VCUG]
N/A

Amoxicillin/clavulanic acid: which time schedule do you use? [Day of VUS/VCUG]
N/A

Amoxicillin/clavulanic acid: which time schedule do you use? [Day(s) after VUS/VCUG]
N/A

Other: which time schedule do you use? [Day(s) before VUS/VCUG, how many?]
N/A

Other: which time schedule do you use? [Day of VUS/VCUG]
N/A

Other: which time schedule do you use? [Day(s) after VUS/VCUG, how many?]
N/A

Indication for prophylaxis

Which patients receive periinterventional antibiotic prophylaxis? (multiple answers possible) [all]
N/A

Which patients receive periinterventional antibiotic prophylaxis? (multiple answers possible) [patients post urinary tract infection/pyelonephritis/urosepsis]
N/A

Which patients receive periinterventional antibiotic prophylaxis? (multiple answers possible) [patients with urinary tract dilation in ultrasound]
N/A

Which patients receive periinterventional antibiotic prophylaxis? (multiple answers possible) [patients with prenatal urinary tract dilation]
N/A

Which patients receive periinterventional antibiotic prophylaxis? (multiple answers possible) [patients post VUR surgery]
N/A

Which patients receive periinterventional antibiotic prophylaxis? (multiple answers possible) [none]
N/A

Are there situations or clinical findings in the patient where you deviate from your prophylaxis standard (medication, dose, duration, age of the patient)? (multiple answers possible, please specify in answer box) [in case of already known vesicoureteral reflux (VUR)]
N/A

Are there situations or clinical findings in the patient where you deviate from your prophylaxis standard (medication, dose, duration, age of the patient)? (multiple answers possible, please specify in answer box) [in patients with recurrent urinary tract infections (UTI)]
N/A

Are there situations or clinical findings in the patient where you deviate from your prophylaxis standard (medication, dose, duration, age of the patient)? (multiple answers possible, please specify in answer box) [if multidrug-resistant organisms (MDRO) are detected in urine cultures]
N/A

Are there situations or clinical findings in the patient where you deviate from your prophylaxis standard (medication, dose, duration, age of the patient)? (multiple answers possible, please specify in answer box) [in newborns]
N/A

Are there situations in which you use intravenous prophylaxis?
N/A

Voiding analysis

Are there differences in antibiotic prophylaxis between VUS and VCUG in your department?
N/A

Is a non-pathological urine strip test required in preparation for VUS/VCUG in your department?
N/A

Is a non-pathological urine culture required in preparation for VUS/VCUG in your department?
N/A

Do patients only receive post-procedure prophylaxis if vesicoureteral reflux (VUR) is detected in VUS/VCUG?
N/A

Do other MUS/MCU findings (e.g. urethral valves) have an influence on post-procedure prophylaxis?
N/A

End of survey

May we contact you personally if we have further questions?
N/A

Survey response 250

Response ID
252

Last page
1

Start language
en

Seed
660371535

Date started
2025-05-01 07:51:43

Date last action
2025-05-01 07:52:18

Standard Operation Procedure

Who fills out this survey?
Radiologist

Who fills out this survey? [Other]

In which type of hospital are you employed?
University Hospital

In which type of hospital are you employed? [Other]

In which country do you work?
GREECE

Does your department have a defined protocol for infection prophylaxis in the context of VUS/VCUG?
Yes

Has this protocol been transformed into a written standard operating procedure (SOP)?
No

Antibiotic Prophylaxis

Are there situations/constellations in which you do not use antibiotic prophylaxis for VUS/VCUG?
N/A

Please specify these situations/constellations: [girl with first uncomplicated UTI without urinary tract dilation in ultrasound]
N/A

Please specify these situations/constellations: [boy with first uncomplicated UTI without urinary tract dilation in ultrasound]
N/A

Please specify these situations/constellations: [in patients with low grade urinary tract dilation (up to 2nd grade) and no UTI in medical history]
N/A

If you use antibiotic prophylaxis: which mediaction do you use? (multiple answers and subdivision into age groups possible) [Trimethoprim]
N/A

If you use antibiotic prophylaxis: which mediaction do you use? (multiple answers and subdivision into age groups possible) [Nitrofurantoin]
N/A

If you use antibiotic prophylaxis: which mediaction do you use? (multiple answers and subdivision into age groups possible) [Amoxicillin]
N/A

If you use antibiotic prophylaxis: which mediaction do you use? (multiple answers and subdivision into age groups possible) [Cefaclor]
N/A

If you use antibiotic prophylaxis: which mediaction do you use? (multiple answers and subdivision into age groups possible) [Amoxicillin/clavulanic acid]
N/A

If you use antibiotic prophylaxis: which mediaction do you use? (multiple answers and subdivision into age groups possible) [Other]
N/A

Trimethoprim: which time schedule do you use? [Day(s) before VUS/VCUG, how many?]
N/A

Trimethoprim: which time schedule do you use? [Day of VUS/VCUG]
N/A

Trimethoprim: which time schedule do you use? [Day(s) after VUS/VCUG, how many?]
N/A

Nitrofurantoin: which time schedule do you use? [Day(s) before VUS/VCUG, how many?]
N/A

Nitrofurantoin: which time schedule do you use? [Day of VUS/VCUG]
N/A

Nitrofurantoin: which time schedule do you use? [Day(s) after VUS/VCUG, how many?]
N/A

Amoxicillin: which time schedule do you use? [Day(s) before VUS/VCUG, how many?]
N/A

Amoxicillin: which time schedule do you use? [Day of VUS/VCUG]
N/A

Amoxicillin: which time schedule do you use? [Day(s) after VUS/VCUG, how many?]
N/A

Cefaclor: which time schedule do you use? [Days(s) before VUS/VCUG, how many?]
N/A

Cefaclor: which time schedule do you use? [Day of VUS/VCUG]
N/A

Cefaclor: which time schedule do you use? [Day(s) after VUS/VCUG, how many?]
N/A

Amoxicillin/clavulanic acid: in what dosage? (Amoxicillin portion) [prophylaktic (10-20 mg/Kg bodyweight/d)]
N/A

Amoxicillin/clavulanic acid: in what dosage? (Amoxicillin portion) [double prophylactic (20-40 mg/Kg bodyweight/d)]
N/A

Amoxicillin/clavulanic acid: in what dosage? (Amoxicillin portion) [therapeutic (80-100 mg/Kg bodyweight/d)]
N/A

Amoxicillin/clavulanic acid: which time schedule do you use? [Day(s) before VUS/VCUG]
N/A

Amoxicillin/clavulanic acid: which time schedule do you use? [Day of VUS/VCUG]
N/A

Amoxicillin/clavulanic acid: which time schedule do you use? [Day(s) after VUS/VCUG]
N/A

Other: which time schedule do you use? [Day(s) before VUS/VCUG, how many?]
N/A

Other: which time schedule do you use? [Day of VUS/VCUG]
N/A

Other: which time schedule do you use? [Day(s) after VUS/VCUG, how many?]
N/A

Indication for prophylaxis

Which patients receive periinterventional antibiotic prophylaxis? (multiple answers possible) [all]
N/A

Which patients receive periinterventional antibiotic prophylaxis? (multiple answers possible) [patients post urinary tract infection/pyelonephritis/urosepsis]
N/A

Which patients receive periinterventional antibiotic prophylaxis? (multiple answers possible) [patients with urinary tract dilation in ultrasound]
N/A

Which patients receive periinterventional antibiotic prophylaxis? (multiple answers possible) [patients with prenatal urinary tract dilation]
N/A

Which patients receive periinterventional antibiotic prophylaxis? (multiple answers possible) [patients post VUR surgery]
N/A

Which patients receive periinterventional antibiotic prophylaxis? (multiple answers possible) [none]
N/A

Are there situations or clinical findings in the patient where you deviate from your prophylaxis standard (medication, dose, duration, age of the patient)? (multiple answers possible, please specify in answer box) [in case of already known vesicoureteral reflux (VUR)]
N/A

Are there situations or clinical findings in the patient where you deviate from your prophylaxis standard (medication, dose, duration, age of the patient)? (multiple answers possible, please specify in answer box) [in patients with recurrent urinary tract infections (UTI)]
N/A

Are there situations or clinical findings in the patient where you deviate from your prophylaxis standard (medication, dose, duration, age of the patient)? (multiple answers possible, please specify in answer box) [if multidrug-resistant organisms (MDRO) are detected in urine cultures]
N/A

Are there situations or clinical findings in the patient where you deviate from your prophylaxis standard (medication, dose, duration, age of the patient)? (multiple answers possible, please specify in answer box) [in newborns]
N/A

Are there situations in which you use intravenous prophylaxis?
N/A

Voiding analysis

Are there differences in antibiotic prophylaxis between VUS and VCUG in your department?
N/A

Is a non-pathological urine strip test required in preparation for VUS/VCUG in your department?
N/A

Is a non-pathological urine culture required in preparation for VUS/VCUG in your department?
N/A

Do patients only receive post-procedure prophylaxis if vesicoureteral reflux (VUR) is detected in VUS/VCUG?
N/A

Do other MUS/MCU findings (e.g. urethral valves) have an influence on post-procedure prophylaxis?
N/A

End of survey

May we contact you personally if we have further questions?
N/A

Survey response 251

Response ID
253

Date submitted
2025-05-20 16:36:20

Last page
5

Start language
de

Seed
62689919

Date started
2025-05-20 16:27:25

Date last action
2025-05-20 16:36:20

Standard Operation Procedure

Who fills out this survey?
Pediatric Surgeon

Who fills out this survey? [Other]

In which type of hospital are you employed?
Community hospital (

In which type of hospital are you employed? [Other]

In which country do you work?
Rheinland/Pfalz

Does your department have a defined protocol for infection prophylaxis in the context of VUS/VCUG?
Yes

Has this protocol been transformed into a written standard operating procedure (SOP)?
Yes

Which department is the author of the SOP for infection prophylaxis in VUS/VCUG?
Pediatric surgery

Which department is the author of the SOP for infection prophylaxis in VUS/VCUG? [Other]

Antibiotic Prophylaxis

Are there situations/constellations in which you do not use antibiotic prophylaxis for VUS/VCUG?
No

Please specify these situations/constellations: [girl with first uncomplicated UTI without urinary tract dilation in ultrasound]
N/A

Please specify these situations/constellations: [boy with first uncomplicated UTI without urinary tract dilation in ultrasound]
N/A

Please specify these situations/constellations: [in patients with low grade urinary tract dilation (up to 2nd grade) and no UTI in medical history]
N/A

If you use antibiotic prophylaxis: which mediaction do you use? (multiple answers and subdivision into age groups possible) [Trimethoprim]
Yes

If you use antibiotic prophylaxis: which mediaction do you use? (multiple answers and subdivision into age groups possible) [Nitrofurantoin]
Yes

If you use antibiotic prophylaxis: which mediaction do you use? (multiple answers and subdivision into age groups possible) [Amoxicillin]
No

If you use antibiotic prophylaxis: which mediaction do you use? (multiple answers and subdivision into age groups possible) [Cefaclor]
Yes

If you use antibiotic prophylaxis: which mediaction do you use? (multiple answers and subdivision into age groups possible) [Amoxicillin/clavulanic acid]
No

If you use antibiotic prophylaxis: which mediaction do you use? (multiple answers and subdivision into age groups possible) [Other]
No

Trimethoprim: in what dosage?
double prophylakctic (4 mg/kg bodyweight/d)

Trimethoprim: in what dosage? [Other]

Trimethoprim: for which age group do you use trimethoprim?
über 6 Monaten

Trimethoprim: which time schedule do you use? [Day(s) before VUS/VCUG, how many?]
Yes

Trimethoprim: which time schedule do you use? [Day(s) before VUS/VCUG, how many?][Comment]
1

Trimethoprim: which time schedule do you use? [Day of VUS/VCUG]
Yes

Trimethoprim: which time schedule do you use? [Day of VUS/VCUG][Comment]

Trimethoprim: which time schedule do you use? [Day(s) after VUS/VCUG, how many?]
Yes

Trimethoprim: which time schedule do you use? [Day(s) after VUS/VCUG, how many?][Comment]
1

Nitrofurantoin: in what dosage?
double prophylactic (2-4 mg/kg bodyweight/d)

Nitrofurantoin: in what dosage? [Other]

Nitrofurantoin: for which age group do you use nitrofurantoin?
wenn Antibiogramm erforderlich

Nitrofurantoin: which time schedule do you use? [Day(s) before VUS/VCUG, how many?]
Yes

Nitrofurantoin: which time schedule do you use? [Day(s) before VUS/VCUG, how many?][Comment]
1

Nitrofurantoin: which time schedule do you use? [Day of VUS/VCUG]
Yes

Nitrofurantoin: which time schedule do you use? [Day of VUS/VCUG][Comment]

Nitrofurantoin: which time schedule do you use? [Day(s) after VUS/VCUG, how many?]
Yes

Nitrofurantoin: which time schedule do you use? [Day(s) after VUS/VCUG, how many?][Comment]
1

Amoxicillin: which time schedule do you use? [Day(s) before VUS/VCUG, how many?]
N/A

Amoxicillin: which time schedule do you use? [Day of VUS/VCUG]
N/A

Amoxicillin: which time schedule do you use? [Day(s) after VUS/VCUG, how many?]
N/A

Cefaclor: in what dosage?
therapeutic (40 mg/kg bodyweight/d)

Cefaclor: in what dosage? [Other]

Cefaclor: for which age group do you use cefaclor?
unter 6 Mo

Cefaclor: which time schedule do you use? [Days(s) before VUS/VCUG, how many?]
Yes

Cefaclor: which time schedule do you use? [Days(s) before VUS/VCUG, how many?][Comment]
1

Cefaclor: which time schedule do you use? [Day of VUS/VCUG]
Yes

Cefaclor: which time schedule do you use? [Day of VUS/VCUG][Comment]

Cefaclor: which time schedule do you use? [Day(s) after VUS/VCUG, how many?]
Yes

Cefaclor: which time schedule do you use? [Day(s) after VUS/VCUG, how many?][Comment]
1

Amoxicillin/clavulanic acid: in what dosage? (Amoxicillin portion) [prophylaktic (10-20 mg/Kg bodyweight/d)]
N/A

Amoxicillin/clavulanic acid: in what dosage? (Amoxicillin portion) [double prophylactic (20-40 mg/Kg bodyweight/d)]
N/A

Amoxicillin/clavulanic acid: in what dosage? (Amoxicillin portion) [therapeutic (80-100 mg/Kg bodyweight/d)]
N/A

Amoxicillin/clavulanic acid: which time schedule do you use? [Day(s) before VUS/VCUG]
N/A

Amoxicillin/clavulanic acid: which time schedule do you use? [Day of VUS/VCUG]
N/A

Amoxicillin/clavulanic acid: which time schedule do you use? [Day(s) after VUS/VCUG]
N/A

Other: which time schedule do you use? [Day(s) before VUS/VCUG, how many?]
N/A

Other: which time schedule do you use? [Day of VUS/VCUG]
N/A

Other: which time schedule do you use? [Day(s) after VUS/VCUG, how many?]
N/A

Indication for prophylaxis

Which patients receive periinterventional antibiotic prophylaxis? (multiple answers possible) [all]
No

Which patients receive periinterventional antibiotic prophylaxis? (multiple answers possible) [patients post urinary tract infection/pyelonephritis/urosepsis]
Yes

Which patients receive periinterventional antibiotic prophylaxis? (multiple answers possible) [patients with urinary tract dilation in ultrasound]
No

Which patients receive periinterventional antibiotic prophylaxis? (multiple answers possible) [patients with prenatal urinary tract dilation]
No

Which patients receive periinterventional antibiotic prophylaxis? (multiple answers possible) [patients post VUR surgery]
No

Which patients receive periinterventional antibiotic prophylaxis? (multiple answers possible) [none]
No

Which patients receive periinterventional antibiotic prophylaxis? (multiple answers possible) [Other]

patients status post urinary tract infection/pyelonephritis/urosepsis: what is the minimum time between infection and VUS/VCUG? (in days)
je nach Diagnostik; blander Urin und mindesten 5 Tage nach iv therapierter Pyelonephritis bzw Urosepsis

Are there situations or clinical findings in the patient where you deviate from your prophylaxis standard (medication, dose, duration, age of the patient)? (multiple answers possible, please specify in answer box) [in case of already known vesicoureteral reflux (VUR)]
No

Are there situations or clinical findings in the patient where you deviate from your prophylaxis standard (medication, dose, duration, age of the patient)? (multiple answers possible, please specify in answer box) [in case of already known vesicoureteral reflux (VUR)][Comment]

Are there situations or clinical findings in the patient where you deviate from your prophylaxis standard (medication, dose, duration, age of the patient)? (multiple answers possible, please specify in answer box) [in patients with recurrent urinary tract infections (UTI)]
Yes

Are there situations or clinical findings in the patient where you deviate from your prophylaxis standard (medication, dose, duration, age of the patient)? (multiple answers possible, please specify in answer box) [in patients with recurrent urinary tract infections (UTI)][Comment]
und auffälligem Urin, wenn die Untersuchung doch durchgeführt wurde ggf i.v.

Are there situations or clinical findings in the patient where you deviate from your prophylaxis standard (medication, dose, duration, age of the patient)? (multiple answers possible, please specify in answer box) [if multidrug-resistant organisms (MDRO) are detected in urine cultures]
Yes

Are there situations or clinical findings in the patient where you deviate from your prophylaxis standard (medication, dose, duration, age of the patient)? (multiple answers possible, please specify in answer box) [if multidrug-resistant organisms (MDRO) are detected in urine cultures][Comment]
antibiogrammgerecht

Are there situations or clinical findings in the patient where you deviate from your prophylaxis standard (medication, dose, duration, age of the patient)? (multiple answers possible, please specify in answer box) [in newborns]
No

Are there situations or clinical findings in the patient where you deviate from your prophylaxis standard (medication, dose, duration, age of the patient)? (multiple answers possible, please specify in answer box) [in newborns][Comment]

Are there situations or clinical findings in the patient where you deviate from your prophylaxis standard (medication, dose, duration, age of the patient)? (multiple answers possible, please specify in answer box) [Other]

Are there situations or clinical findings in the patient where you deviate from your prophylaxis standard (medication, dose, duration, age of the patient)? (multiple answers possible, please specify in answer box) [Other comment]

Are there situations in which you use intravenous prophylaxis?
Yes

In which situations?
s.o.

Voiding analysis

Are there differences in antibiotic prophylaxis between VUS and VCUG in your department?
No

Is a non-pathological urine strip test required in preparation for VUS/VCUG in your department?
Yes

What is the maximum amount of days accepted for urine strip test to be performed prior to the exam?
3-5

Is a non-pathological urine culture required in preparation for VUS/VCUG in your department?
No

Do patients only receive post-procedure prophylaxis if vesicoureteral reflux (VUR) is detected in VUS/VCUG?
No

Do other MUS/MCU findings (e.g. urethral valves) have an influence on post-procedure prophylaxis?
No

End of survey

Thank you for taking the time to answer this survey!

May we contact you personally if we have further questions?
Yes

E-mail address:
annamaria.ziegler@mutterhaus.de

Survey response 252

Response ID
254

Date submitted
2025-05-28 08:56:30

Last page
5

Start language
en

Seed
1844810570

Date started
2025-05-28 08:45:29

Date last action
2025-05-28 08:56:30

Standard Operation Procedure

Who fills out this survey?
Pediatric Radiologist

Who fills out this survey? [Other]

In which type of hospital are you employed?
University Hospital

In which type of hospital are you employed? [Other]

In which country do you work?
United Kingdom

Does your department have a defined protocol for infection prophylaxis in the context of VUS/VCUG?
Yes

Has this protocol been transformed into a written standard operating procedure (SOP)?
Yes

Which department is the author of the SOP for infection prophylaxis in VUS/VCUG?
Pediatric nephrology

Which department is the author of the SOP for infection prophylaxis in VUS/VCUG? [Other]

Antibiotic Prophylaxis

Are there situations/constellations in which you do not use antibiotic prophylaxis for VUS/VCUG?
No

Please specify these situations/constellations: [girl with first uncomplicated UTI without urinary tract dilation in ultrasound]
N/A

Please specify these situations/constellations: [boy with first uncomplicated UTI without urinary tract dilation in ultrasound]
N/A

Please specify these situations/constellations: [in patients with low grade urinary tract dilation (up to 2nd grade) and no UTI in medical history]
N/A

If you use antibiotic prophylaxis: which mediaction do you use? (multiple answers and subdivision into age groups possible) [Trimethoprim]
Yes

If you use antibiotic prophylaxis: which mediaction do you use? (multiple answers and subdivision into age groups possible) [Nitrofurantoin]
No

If you use antibiotic prophylaxis: which mediaction do you use? (multiple answers and subdivision into age groups possible) [Amoxicillin]
No

If you use antibiotic prophylaxis: which mediaction do you use? (multiple answers and subdivision into age groups possible) [Cefaclor]
No

If you use antibiotic prophylaxis: which mediaction do you use? (multiple answers and subdivision into age groups possible) [Amoxicillin/clavulanic acid]
No

If you use antibiotic prophylaxis: which mediaction do you use? (multiple answers and subdivision into age groups possible) [Other]
No

Trimethoprim: in what dosage?
prophylactic (2 mg/kg bodyweight/d)

Trimethoprim: in what dosage? [Other]

Trimethoprim: for which age group do you use trimethoprim?

Trimethoprim: which time schedule do you use? [Day(s) before VUS/VCUG, how many?]
Yes

Trimethoprim: which time schedule do you use? [Day(s) before VUS/VCUG, how many?][Comment]
1

Trimethoprim: which time schedule do you use? [Day of VUS/VCUG]
Yes

Trimethoprim: which time schedule do you use? [Day of VUS/VCUG][Comment]

Trimethoprim: which time schedule do you use? [Day(s) after VUS/VCUG, how many?]
Yes

Trimethoprim: which time schedule do you use? [Day(s) after VUS/VCUG, how many?][Comment]
1

Nitrofurantoin: which time schedule do you use? [Day(s) before VUS/VCUG, how many?]
N/A

Nitrofurantoin: which time schedule do you use? [Day of VUS/VCUG]
N/A

Nitrofurantoin: which time schedule do you use? [Day(s) after VUS/VCUG, how many?]
N/A

Amoxicillin: which time schedule do you use? [Day(s) before VUS/VCUG, how many?]
N/A

Amoxicillin: which time schedule do you use? [Day of VUS/VCUG]
N/A

Amoxicillin: which time schedule do you use? [Day(s) after VUS/VCUG, how many?]
N/A

Cefaclor: which time schedule do you use? [Days(s) before VUS/VCUG, how many?]
N/A

Cefaclor: which time schedule do you use? [Day of VUS/VCUG]
N/A

Cefaclor: which time schedule do you use? [Day(s) after VUS/VCUG, how many?]
N/A

Amoxicillin/clavulanic acid: in what dosage? (Amoxicillin portion) [prophylaktic (10-20 mg/Kg bodyweight/d)]
N/A

Amoxicillin/clavulanic acid: in what dosage? (Amoxicillin portion) [double prophylactic (20-40 mg/Kg bodyweight/d)]
N/A

Amoxicillin/clavulanic acid: in what dosage? (Amoxicillin portion) [therapeutic (80-100 mg/Kg bodyweight/d)]
N/A

Amoxicillin/clavulanic acid: which time schedule do you use? [Day(s) before VUS/VCUG]
N/A

Amoxicillin/clavulanic acid: which time schedule do you use? [Day of VUS/VCUG]
N/A

Amoxicillin/clavulanic acid: which time schedule do you use? [Day(s) after VUS/VCUG]
N/A

Other: which time schedule do you use? [Day(s) before VUS/VCUG, how many?]
N/A

Other: which time schedule do you use? [Day of VUS/VCUG]
N/A

Other: which time schedule do you use? [Day(s) after VUS/VCUG, how many?]
N/A

Indication for prophylaxis

Which patients receive periinterventional antibiotic prophylaxis? (multiple answers possible) [all]
Yes

Which patients receive periinterventional antibiotic prophylaxis? (multiple answers possible) [patients post urinary tract infection/pyelonephritis/urosepsis]
N/A

Which patients receive periinterventional antibiotic prophylaxis? (multiple answers possible) [patients with urinary tract dilation in ultrasound]
N/A

Which patients receive periinterventional antibiotic prophylaxis? (multiple answers possible) [patients with prenatal urinary tract dilation]
N/A

Which patients receive periinterventional antibiotic prophylaxis? (multiple answers possible) [patients post VUR surgery]
N/A

Which patients receive periinterventional antibiotic prophylaxis? (multiple answers possible) [none]
N/A

Are there situations or clinical findings in the patient where you deviate from your prophylaxis standard (medication, dose, duration, age of the patient)? (multiple answers possible, please specify in answer box) [in case of already known vesicoureteral reflux (VUR)]
No

Are there situations or clinical findings in the patient where you deviate from your prophylaxis standard (medication, dose, duration, age of the patient)? (multiple answers possible, please specify in answer box) [in case of already known vesicoureteral reflux (VUR)][Comment]

Are there situations or clinical findings in the patient where you deviate from your prophylaxis standard (medication, dose, duration, age of the patient)? (multiple answers possible, please specify in answer box) [in patients with recurrent urinary tract infections (UTI)]
No

Are there situations or clinical findings in the patient where you deviate from your prophylaxis standard (medication, dose, duration, age of the patient)? (multiple answers possible, please specify in answer box) [in patients with recurrent urinary tract infections (UTI)][Comment]

Are there situations or clinical findings in the patient where you deviate from your prophylaxis standard (medication, dose, duration, age of the patient)? (multiple answers possible, please specify in answer box) [if multidrug-resistant organisms (MDRO) are detected in urine cultures]
Yes

Are there situations or clinical findings in the patient where you deviate from your prophylaxis standard (medication, dose, duration, age of the patient)? (multiple answers possible, please specify in answer box) [if multidrug-resistant organisms (MDRO) are detected in urine cultures][Comment]

Are there situations or clinical findings in the patient where you deviate from your prophylaxis standard (medication, dose, duration, age of the patient)? (multiple answers possible, please specify in answer box) [in newborns]
No

Are there situations or clinical findings in the patient where you deviate from your prophylaxis standard (medication, dose, duration, age of the patient)? (multiple answers possible, please specify in answer box) [in newborns][Comment]

Are there situations or clinical findings in the patient where you deviate from your prophylaxis standard (medication, dose, duration, age of the patient)? (multiple answers possible, please specify in answer box) [Other]

Are there situations or clinical findings in the patient where you deviate from your prophylaxis standard (medication, dose, duration, age of the patient)? (multiple answers possible, please specify in answer box) [Other comment]

Are there situations in which you use intravenous prophylaxis?
No

Voiding analysis

Are there differences in antibiotic prophylaxis between VUS and VCUG in your department?
No

Is a non-pathological urine strip test required in preparation for VUS/VCUG in your department?
No

Is a non-pathological urine culture required in preparation for VUS/VCUG in your department?
No

Do patients only receive post-procedure prophylaxis if vesicoureteral reflux (VUR) is detected in VUS/VCUG?
No

Do other MUS/MCU findings (e.g. urethral valves) have an influence on post-procedure prophylaxis?
Yes

Please specify:
double the dose

End of survey

Thank you for taking the time to answer this survey!

May we contact you personally if we have further questions?
Yes

E-mail address:
bozena.starzyk@nhs.net
